# Proteome of Human Scalp Hair Follicles

**DOI:** 10.7759/cureus.115101

**Published:** 2026-08-24

**Authors:** Ashok Hegde, Yuvaraj Srinivasan, Milind A Chavan, Gurpreet Kalsi

**Affiliations:** 1 Department of Biosciences, ITC Life Sciences and Technology Center, Bangalore, IND; 2 School of Medical Sciences and Technology, Malla Reddy Vishwavidyapeeth, Hyderabad, IND

**Keywords:** gene network, gene ontology, interactome analysis, orbitrap, pathways, plucked hair follicles, protein identification, protein purification, proteomics, scalp

## Abstract

Proteomic analysis of hair follicles aids in the basic understanding of human scalp hair biology and can serve as a foundation for biomarker identification and targeted therapy in precision medicine. Proteomics of scalp hair follicles in the Indian population has not been reported previously. We performed Orbitrap mass spectrometry analysis of proteins extracted from plucked scalp hair follicles of five healthy, nonbalding donors. Proteins from hair follicles were resolved by SDS-PAGE, extracted, trypsin-digested, cleaned up using ZipTips, and purified on C18 reversed-phase Easy-columns for Orbitrap analysis. The Orbitrap mass spectrometry data were processed using Proteome Discoverer (Thermo Fisher Scientific, Waltham, MA, USA). The mass spectrometry proteomics data have been deposited in the ProteomeXchange Consortium via PRIDE, with the dataset identifier PXD034166. In this study, we identified 2,961 unique (nonredundant) proteins across all five samples and 190 proteins common to these samples. Ontology and pathway analyses were performed on the identified proteins. This study provides a well-annotated hair follicle proteomics repository of Indian scalp hair follicles.

## Introduction

Hair follicles are external appendages of the skin comprising epidermal (epithelial) and dermal (mesenchymal) components. Hair follicles undergo distinct growth cycles regulated by signaling pathways, cytokines, and growth factors [1]. One complete hair cycle includes anagen (growing), catagen (regression), telogen (resting), and exogen (shedding) phases [2,3]. Through the coordinated action of these growth cycles and pathways, hair cells support hair growth, and stem cells differentiate into hair lineage cell types to repopulate hair cells.

Although much is known about the hair growth cycle and genomic aspects, relatively less is known about hair proteomics. Differentiated corneocytes in the hair shaft provide hair follicles with the strength to resist physical and chemical stress and protein extraction. Corneocytes are largely composed of keratin and keratin-associated proteins (KAPs), which are the most complex subgroup of intermediate filament (IF) proteins [4,5]. About 80-85% of total hair follicle proteins are extractable under denaturing conditions, while the remaining 15-20% are largely intractable, mainly due to transglutaminase-mediated isopeptide bonding. These ε-(γ-glutamyl)lysine cross-links of transglutaminase substrates, such as trichohyalin, are proposed to be responsible for the high insolubility of epidermal and hair shaft proteins [6,7]. However, a recent study using shotgun proteomics of mouse hair follicles, and our present study using denaturing extraction conditions and Orbitrap mass spectrometry, have identified transglutaminase substrates, such as trichohyalin, in hair follicles, demonstrating that trichohyalin is tractable [8].

Several proteomic techniques have been used for hair proteomic analysis. MudPIT analysis and hair proteomic analysis using ion-exchange chromatography fractionation followed by reverse-phase chromatography and mass spectrometry (LC-MS/MS) have been demonstrated [9,10]. Using the MALDI-TOF/TOF method, a study identified 44 proteins involved in the mouse hair follicle cycle transition between the three stages, such as anagen, catagen, and telogen, and identified changes in the expression of proteins involved in Ca2+-regulated biological processes, migration, regulation of signal transduction, and other processes [11]. Layered expression scanning of plucked hair follicles in another study showed that this is a useful method for profiling hair proteins when hair follicles are limited; however, it is not suitable for large-scale identification of proteins [12]. Human hair shaft proteomic profiling using an LTQ ion trap mass spectrometer has demonstrated different proteomic profiles among four different ethnic groups, interindividual differences within a given ethnicity, and differences in hair shafts from different parts of the body [13]. Using a shotgun proteomic approach, histones and deamidated keratins in the hair shaft have been identified [14]. Moll reported the evaluation of plucked hair follicles for the identification of segments with high growth potential in culture, as well as gene expression and protein analysis [15]. Another study on the proteomics of the hair shaft showed the proteomics of hair follicles (including the hair root bud) of anagen hair follicles (trimmed to ~200-400 µm in length) and identified 63 proteins, including 38 keratins and KAPs [16].

A recent review of the literature on hair proteomics and methods identified gaps in hair proteomics research and suggested the potential of hair proteomics in establishing a medical repository of normal and disease-specific proteomes [17]. Although plucked hair follicles have been used in biomedical research, Orbitrap proteomics analysis of plucked hair follicles has not been reported. The present study is an exploratory descriptive proteomic resource study to establish a reference proteomic repository wherein we evaluated the proteome of plucked scalp hair follicles (including the hair shaft and hair root) using LTQ-Orbitrap mass spectrometry and identified 2,961 proteins. This study enables clinical, noninvasive biomarker discovery for diagnosing genetic disorders, metabolic states, and systemic diseases. It also provides reliable individual identification and biochemical profiling when traditional DNA evidence is degraded or missing, thereby aiding forensic applications.

## Materials and methods

The protocol (MSCR/ITCSC/2012-2013) for hair follicle collection was developed in collaboration with MS Clinical Research (MSCR), Bangalore, India, and was approved by Clinicom, Bangalore, India (an independent ethics committee for the evaluation of protocols for clinical research), under approval number 00739/01.10.12. The protocol was conducted following the principles stated in the Declaration of Helsinki. Hair follicles from the scalps of randomly selected, healthy, nonbalding male donors aged 25-35 years (identified based on the Norwood classification) who met the inclusion and exclusion criteria (Table 1) were collected by physicians at MSCR. Written informed consent for the collection of hair follicle samples and their analysis for research was obtained from the donors. Hair follicles were collected according to the approved protocol. Briefly, the donors were seated in a comfortable position, and the dermatologist collected hair follicles from the scalp using sterile forceps. Selected hair follicles were grasped with the forceps approximately 2 cm from the scalp; if necessary, the forceps were rotated clockwise or counterclockwise to tighten the grip, and the hair follicles were pulled out.

**Table 1:** Criteria for selection of subjects for normal (nonbalding) scalp hair follicle collection.

Inclusion/exclusion	Criteria
Inclusion criteria	Male subjects in generally good health; subjects aged 25-40 years; subjects with no baldness (Norwood scale); subjects willing to provide written informed consent; subjects willing to abide by and comply with the study protocol; subjects willing to undergo the biopsy procedure and provide two biopsy specimens; subjects willing to attend regular follow-ups; subjects who had not participated in a similar investigation in the previous four weeks; subjects who had not undergone any treatment for baldness, such as topical lotion/gel/foam, in the previous year.
Exclusion criteria	Subjects with skin diseases such as folliculitis, seborrheic dermatitis, scalp psoriasis, discoid lupus erythematosus, or scarring alopecia of any cause that could interfere with the test readings; subjects taking oral medications (e.g., steroids, antioxidants, or immunosuppressants) that could compromise the study; subjects consuming green tea continuously for more than 10 days; subjects unwilling to discontinue other topical hair products on the day of the procedure; subjects with known hypersensitivity to lignocaine and adrenaline injections or oral antibiotics; subjects with chronic illness that could influence the cutaneous state; subjects participating in any other cosmetic or therapeutic trials; subjects with any underlying uncontrolled medical illness, including diabetes mellitus, hypertension, liver disease, or a history of alcoholism, HIV, hepatitis, epilepsy, or any other serious medical illness; subjects with any significant laboratory findings.

Collected hair follicles were examined for the hair bud. Only follicles with an intact hair bud were placed in RNAlater solution. Thirty hair follicles plucked from a donor's scalp (six follicles each from the front, back, top, left side, and right side of the scalp) were pooled to make one sample. This pooling of 30 follicles from the same donor was performed to obtain a sufficient quantity of protein for SDS-PAGE and Orbitrap analysis. All five samples (designated N1, N2, N3, N4, and N5) included 30 hair follicles each. After collection, the hair follicles were placed in RNAlater (Life Technologies, Carlsbad, CA, USA), transported to the laboratory at room temperature, placed at 4°C for one day, and later transferred to -80°C for storage.

Protein extraction was performed using a method similar to that described in an earlier study [9]. The plucked scalp hair follicles were thawed and transferred to sodium phosphate buffer with 20 mM dithioerythritol (DTE) (2% SDS, 50 mM sodium phosphate, pH 7.8) in 1.5-mL Eppendorf tubes. The samples were then sonicated (three pulses of 10 seconds each) and placed on a hot surface at 65°C, with a 10-second pulse every 10 minutes, overnight. The next day, the supernatant was collected, and new buffer was added to the insoluble fraction for the next round of extraction as described above. Three rounds of extraction were performed for each sample. All three fractions from each sample were combined and mixed with six volumes of 100% acetone and placed at -80°C for protein precipitation. The samples were centrifuged at 6,000 × g for 10 minutes at 4°C. The pellet was washed twice with 70% acetone, dried at room temperature, and dissolved in 50 µL of dissolution buffer (6 M urea, 3 M thiourea, and 250 mM tetraethylammonium bromide). Protein quantity was determined using a 2D Quant Kit (GE Healthcare, Piscataway, NJ, USA).

Fifty micrograms of protein from each sample were resolved on a 4-20% SDS-PAGE gradient gel. Each sample lane on the SDS-PAGE gel was sliced into 15 parts and chopped into approximately 1 mm × 1 mm pieces with a scalpel blade and placed in separate 1.5-mL tubes. Gel pieces were washed twice with wash solution (30% acetonitrile in 50 mM ammonium bicarbonate) for five minutes, followed by washing with 100% acetonitrile and vacuum drying. Further, 10 mM TCEP was added and incubated for 30 minutes, and an equal amount of 50 mM iodoacetamide was added for another 30 minutes. The supernatant was discarded, and the gel pieces were washed twice in wash solution, followed by one wash in 100% acetonitrile and vacuum drying. For in-gel trypsin digestion, 50 µL of trypsin (0.2 µg/mL) prepared in 50 mM ammonium bicarbonate was added to each tube, followed by the addition of 100 µL of 50 mM ammonium bicarbonate, and the samples were incubated at 37°C for 16 hours. The gel pieces were then treated twice with 100% acetonitrile as described above, and the supernatant was collected. Milli-Q water (150 µL) was added to each tube for five minutes, and the gel pieces were dried in 100% acetonitrile. The supernatant was collected in the respective tubes, vacuum-dried, and stored at -80°C. In-gel trypsin-digested peptides were purified using C18 ZipTips (Millipore, Billerica, MA, USA) for desalting and cleanup and collected in Protein LoBind Eppendorf microcentrifuge tubes for mass spectrometry after resuspending the stored samples in 8 µL of equilibration buffer (0.1% trifluoroacetic acid in MS-grade water).

Sample cleanup was performed with ZipTips. Each sample was aspirated 15 times into a ZipTip prewetted with 100% acetonitrile. The ZipTip was then washed three times with washing solution (0.1% TFA in MS-grade water) and once with amplification solution (2.5% acetonitrile in 0.5% formic acid in MS-grade water). Peptides from the ZipTips were eluted in 5 µL of elution buffer (70% acetonitrile in 0.5% formic acid) twice into a fresh 1.5-mL tube, vacuum-dried, and stored at -80°C. For nano-HPLC and Orbitrap mass spectrometry, the samples were dried and resuspended in 0.1% formic acid with 5% acetonitrile in MS-grade water.

The peptides were loaded onto an EASY-nLC system (Proxeon, Odense, Denmark). Peptide mixtures were separated on a C18 reverse-phase column (PicoFrit capillary column, 75 µm × 10 cm, New Objective, Woburn, MA, USA) using a linear gradient of solvent A (0.1% formic acid in 5% acetonitrile) and solvent B (0.1% formic acid in 95% acetonitrile) at a flow rate of 250 nL/min directly into an LTQ-Orbitrap Velos mass spectrometer (Thermo Scientific, San Jose, CA, USA) with a nano-ESI source and a spray voltage set at 1.6 kV. Stable spray conditions were maintained prior to sample loading. The instrument was operated in data-dependent mode with dynamic exclusion enabled. MS/MS spectra of the 20 most abundant peptide ions in the full MS scan were obtained. The normalized CID was set at 35% for MS/MS. The mass spectrometry data were processed using Proteome Discoverer, version 1.4 (Thermo Fisher Scientific, Waltham, MA, USA). The files were subsequently submitted to an in-house SEQUEST server for database searching through the Proteome Discoverer 1.4 program. The mass spectrometry proteomics data have been deposited in the ProteomeXchange Consortium via PRIDE, the partner repository, with the dataset identifier PXD034166.

The MS spectra data were searched against the human sequence database of UniProt, with trypsin selected as the specific enzyme. A maximum of one missed cleavage was allowed. Peptide (parent ion) tolerance of 2.5 Da and fragment ion tolerance of 1 Da were allowed. Oxidation (M) was used as a variable modification, and carbamidomethylation (C) was used as a fixed modification. For detailed data analysis, proteins with at least two peptide ions were considered correctly identified. Peptide-spectrum matches were controlled at <1% FDR using a target-decoy approach, followed by <% protein-level FDR filtering. Protein confidence was not determined solely by the two-peptide criterion; rather, it was based on the combined peptide-spectrum-match evidence, FDR-controlled peptide identifications, and protein inference or grouping criteria. Normalizations were performed sample-wise (median) and column-wise, followed by global normalization. Differentially expressed proteins were identified using the in-house-developed Python-based tool “ProtLyser” as described earlier [18]. The data output files were further analyzed using List Operator, an online software tool used to identify exclusive, intersection, and union proteins. Raw intensity values were used to derive the fold change. Interactome analysis was performed using GeneMANIA.

## Results

**Figure 1:**
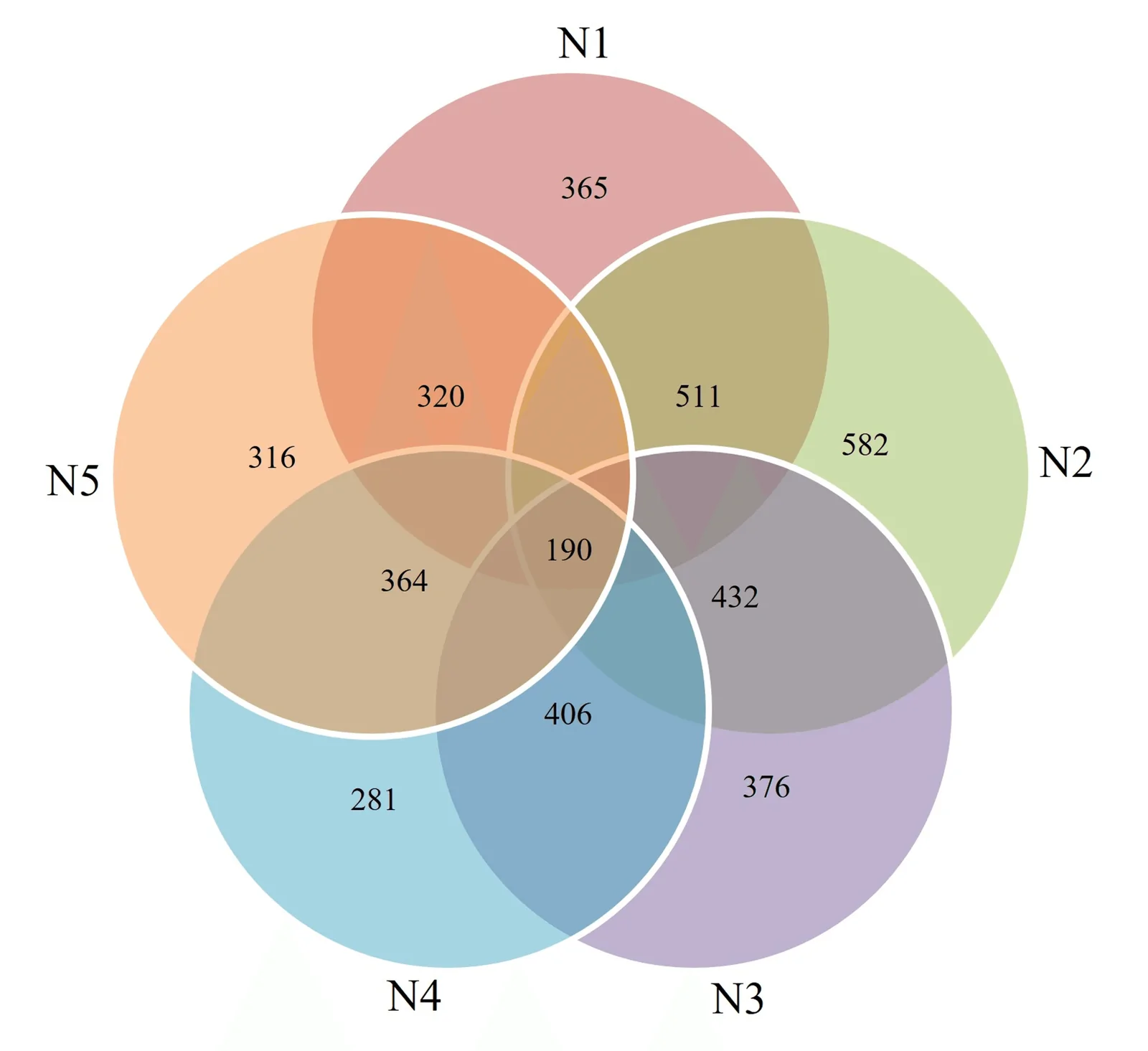
Proteins identified commonly across and exclusively in hair follicles from five human subjects designated as N1, N2, N3, N4, and N5.

In this study, we identified 1,046, 1,270, 1,035, 879, and 841 proteins in the five donor hair follicle samples N1 to N5, respectively. The percentages of proteins in these five samples represented by >5 unique peptides, >2 peptides, and 1 peptide were 10.58-16.3%, 28.66-38.19%, and 61.81-71.34%, respectively. Across all five samples, this study identified 2,961 proteins without any redundancies (Appendix A). These 2,961 proteins include 2,483 characterized proteins, 362 hypothetical proteins predicted based on cDNA clones, 80 putative proteins, and 36 uncharacterized proteins. Of these, 190 proteins were common to all five samples (Appendix B), and the proteins exclusively identified in each sample were 365, 582, 376, 281, and 316 in samples N1 to N5, respectively (Figure 1, Appendices C-G).

**Figure 2:**
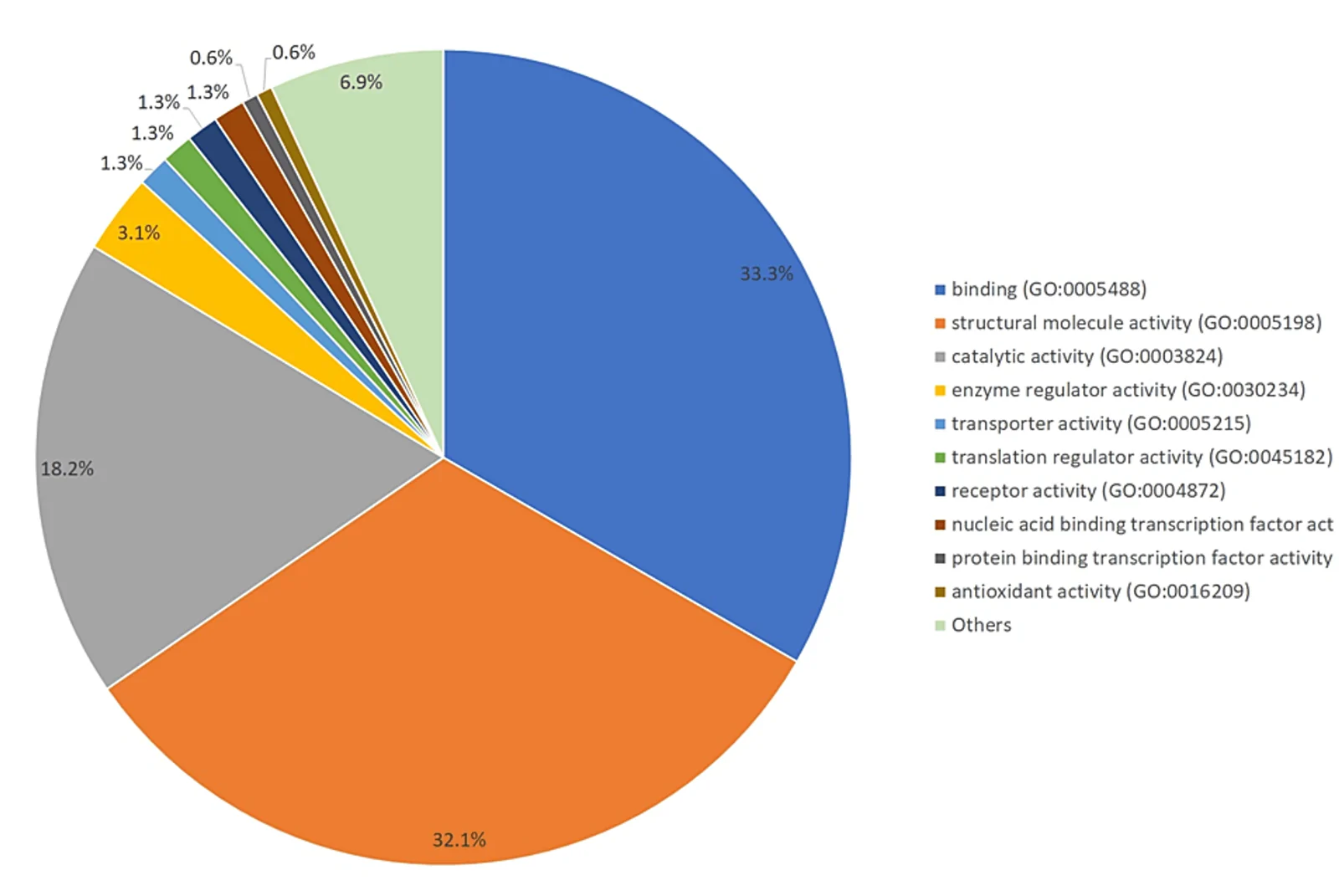
Molecular function of proteins.

**Figure 3:**
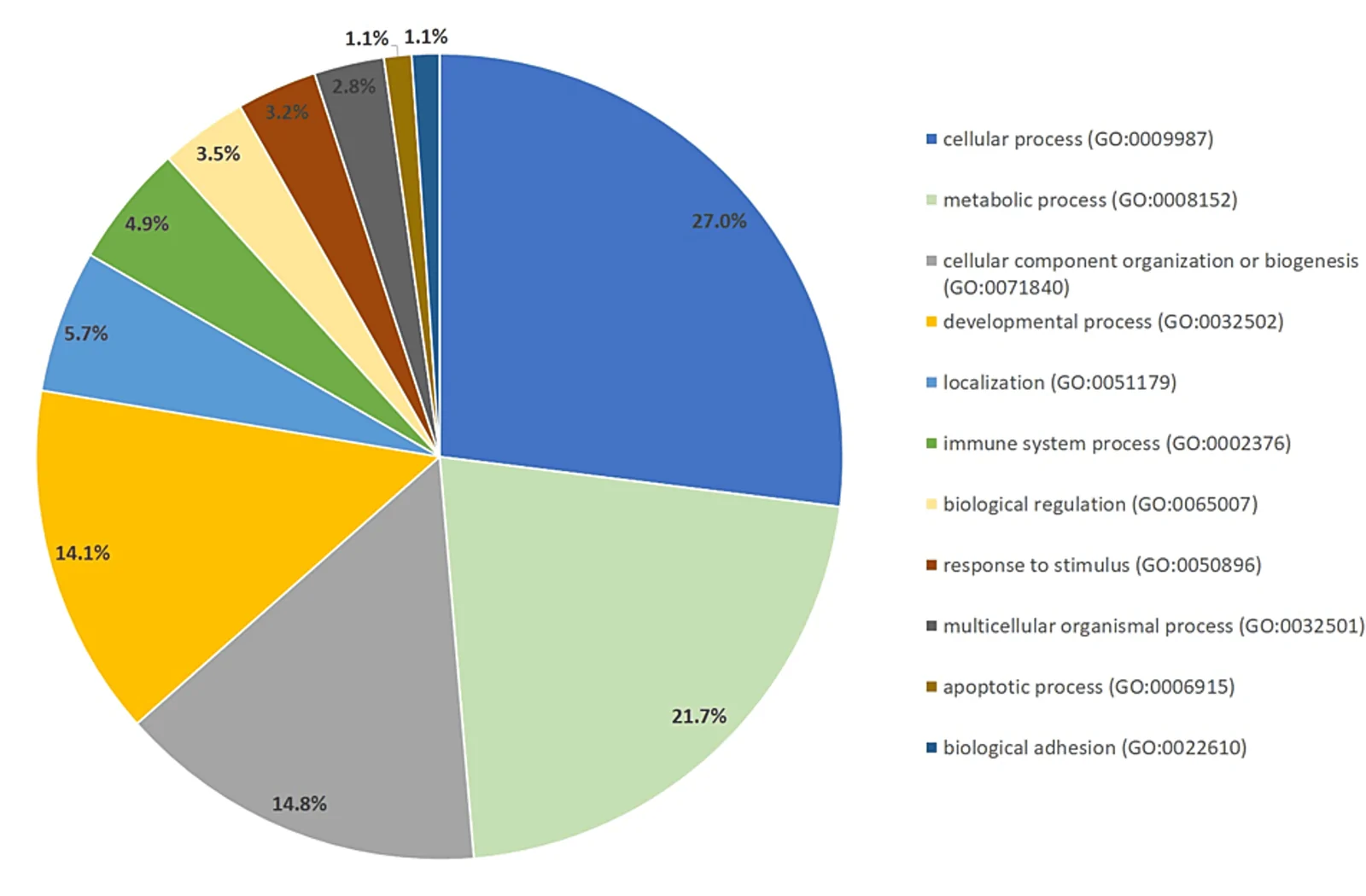
Biological processes related to the proteins.

**Figure 4:**
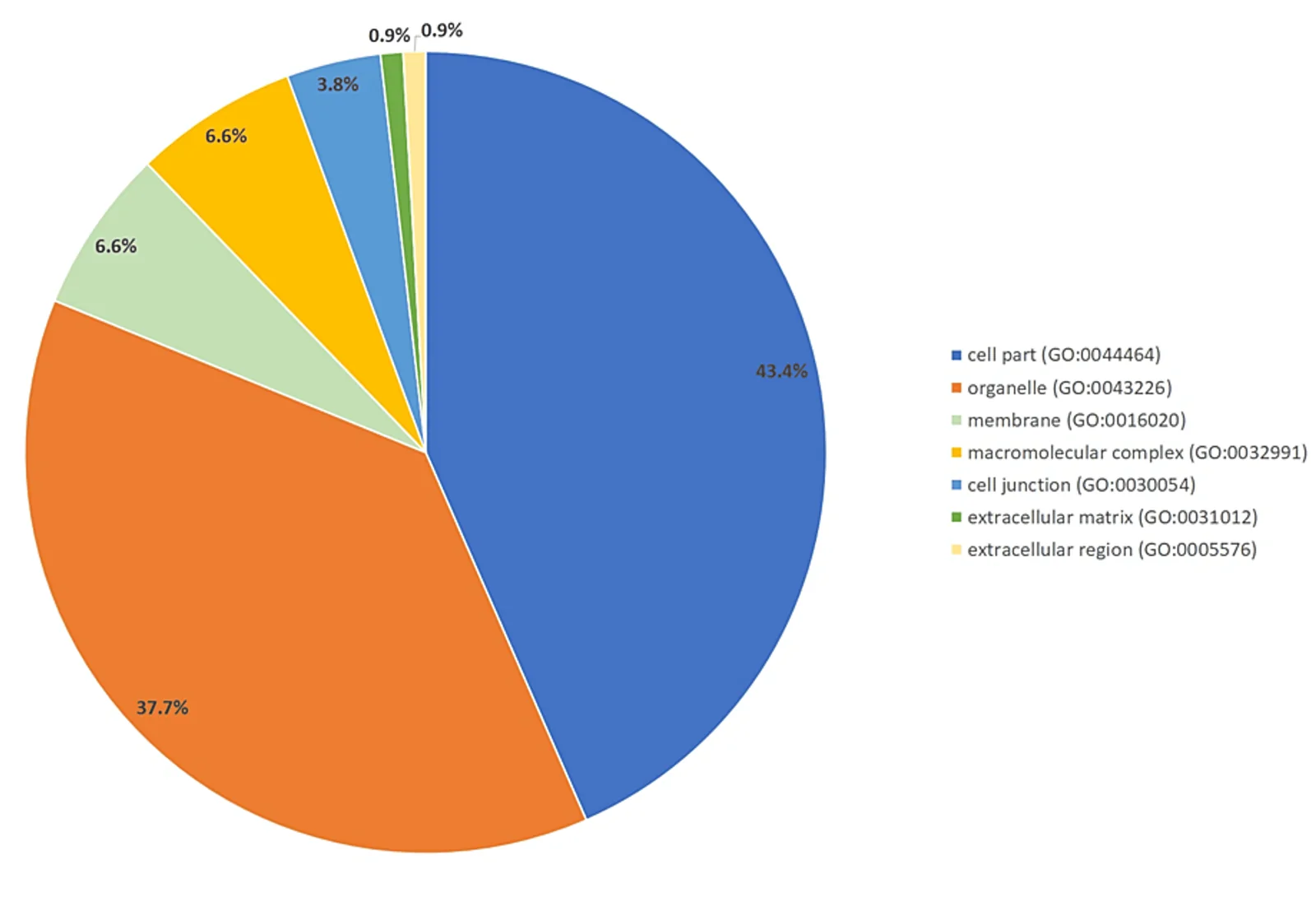
Cellular component distribution of proteins.

**Figure 5:**
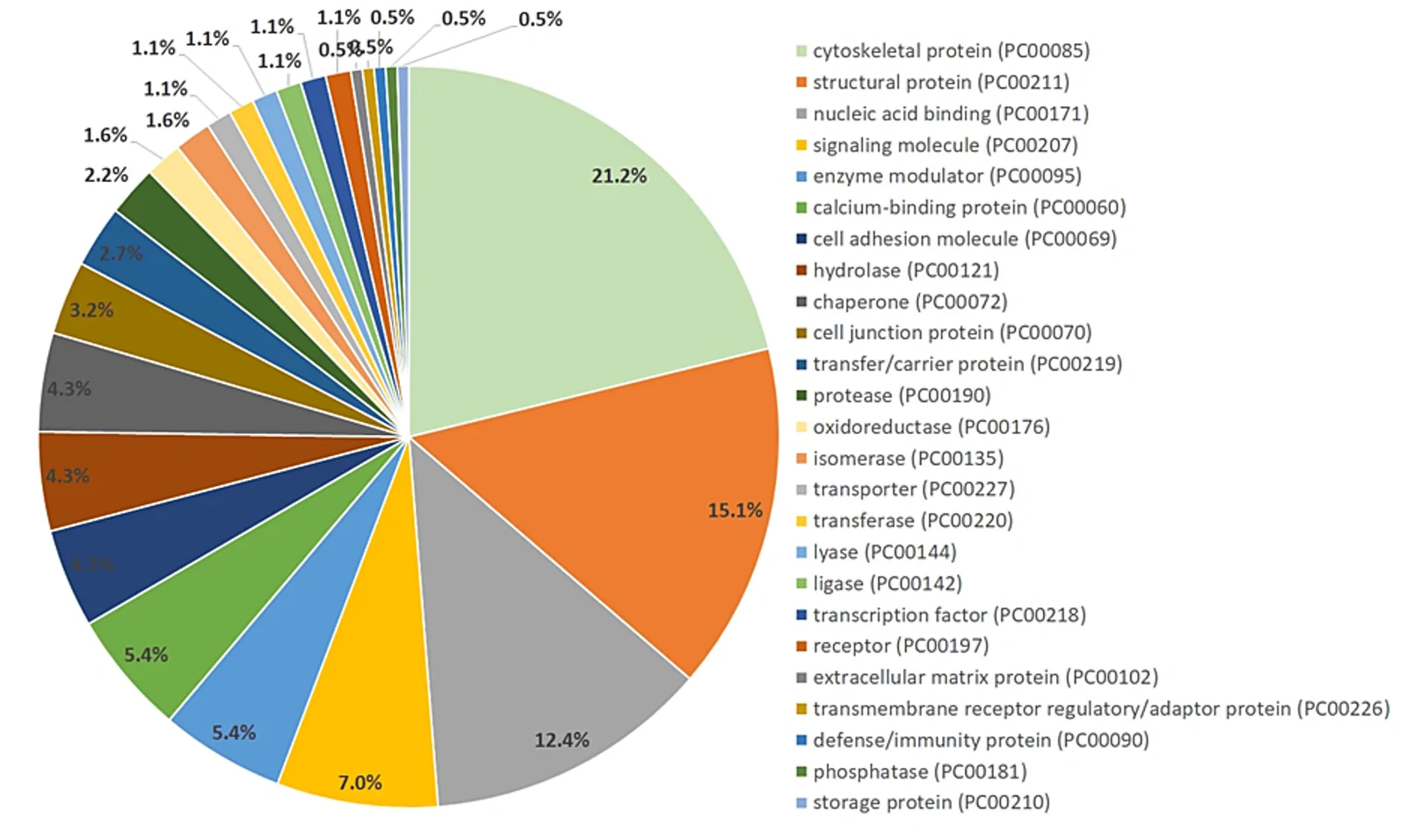
Protein class distribution.

**Figure 6:**
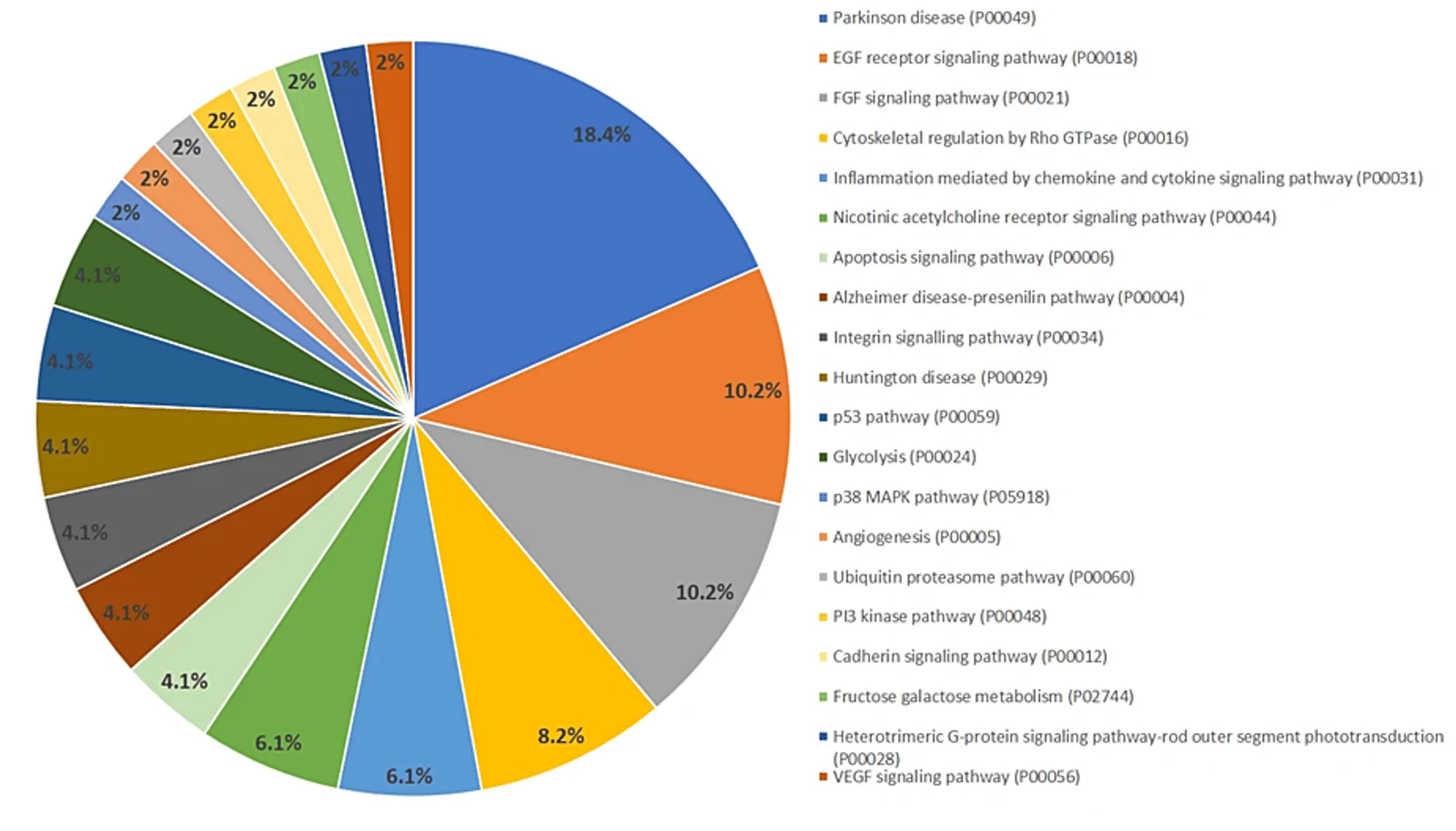
Pathway analysis of proteins.

We performed gene ontology analysis, including molecular function (Figure 2), biological process (Figure 3), and cellular component analysis (Figure 4), protein class distribution (Figure 5), and signaling pathway analysis (Figure 6) using PANTHER Bioinformatics on the common proteins identified across all samples (Appendix H). These proteins (Figures 2, 3) were mainly involved in binding functions (33.3%), followed by structural activity (32.1%), catalytic activity (18.2%), and enzyme activity (6.9%), and in cellular activities such as cellular processes (27.0%), metabolic processes (21.7%), cellular component organization and biogenesis (14.8%), developmental processes (14.1%), localization (5.7%), biological regulation (6.3%), and response to stimulus (5.7%). Most of these proteins were associated with cell parts (43.4%) and organelles (37.7%) (Figure 4), while the remaining proteins were associated with cellular components such as macromolecular complexes (6.6%), membranes (6.6%), cell junctions (3.8%), extracellular regions (0.9%), and matrices (0.9%). The major protein classes (Figure 5) were cytoskeletal proteins (21.2%), structural proteins (15.1%), nucleic acid-binding proteins (12.4%), signaling molecules (7.4%), enzyme modulators (5.4%), and calcium-binding proteins (5.4%). Functional pathway analysis (Figure 6) identified the 20 main pathways related to hair and skin biology, including fibroblast growth factor, epidermal growth factor, mitogen-activated protein kinase, vascular endothelial growth factor, and angiogenesis signaling.

**Figure 7:**
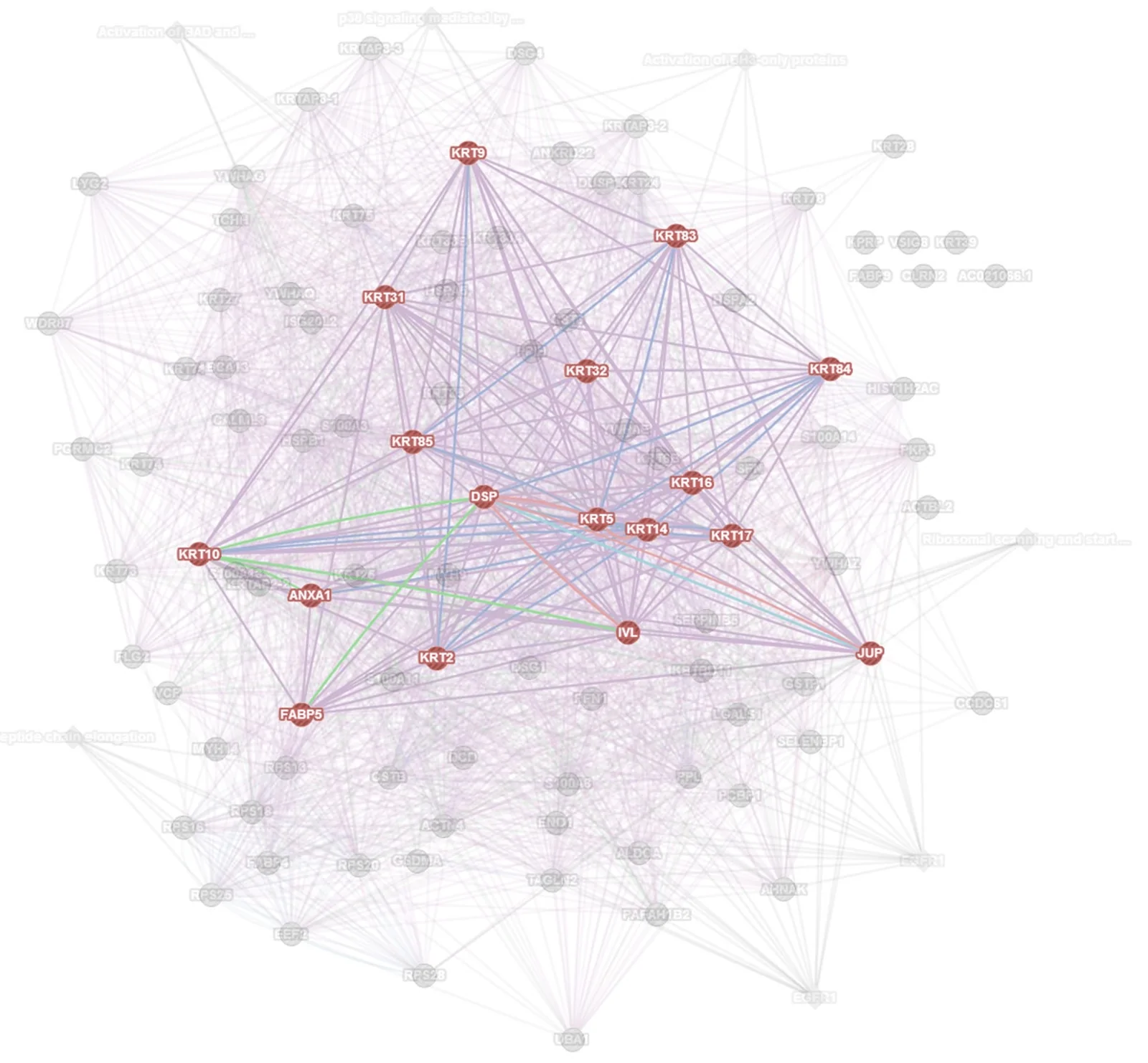
Interactome analysis of 100 proteins (identified in all five samples) showing a network of 17 proteins involved in epidermis development.

Furthermore, we performed interactome analysis using the GeneMANIA informatics resource (www.genemania.org) on this set of common proteins identified in all samples. GeneMANIA converts protein IDs into gene IDs and relates them to database-listed genes and functions. The interaction network and nodes provide details of the interactions and related genes. The parameters selected for interaction included coexpression, colocalization, physical interactions, pathways, and consolidated pathways, with molecular function-based weighting, while all predicted interactions were excluded. Interactome and network analysis of the top 100 proteins (a subset of the 190 proteins, based on the score value) showed that these proteins were functionally related (Table 2). The interactome analysis showed a main network of 17 proteins involved in epidermis development (Figure 7), followed by a network including 11 proteins involved in the structural constituent of the cytoskeleton and nine proteins belonging to the IF cytoskeleton network.

**Table 2:** 100 proteins identified in all five samples.

Accession	Protein description
O43790	Keratin, type II cuticular Hb6 (KRT86)
F8W6V8	Keratin, type II cuticular Hb1 (F8W6V8)
P78386	Keratin, type II cuticular Hb5 (KRT85)
P78385	Keratin, type II cuticular Hb3 (KRT83)
Q15323	Keratin, type I cuticular Ha1 (K1H1)
O76009	Keratin, type I cuticular Ha3-I (KT33A)
Q14525	Keratin, type I cuticular Ha3-II (KT33B)
H6VRF8	Keratin 1 (H6VRF8)
P13647	Keratin, type II cytoskeletal 5 (K2C5)
P35908	Keratin, type II cytoskeletal 2 epidermal (K22E)
P04259	Keratin, type II cytoskeletal 6B (K2C6B)
B4DRR0	cDNA FLJ53910, highly similar to Keratin, type II cytoskeletal 6A (B4DRR0)
P13645	Keratin, type I cytoskeletal 10 (K1C10)
B4DKV4	cDNA FLJ60647, highly similar to Keratin, type II cytoskeletal 6B (B4DKV4)
P02533	Keratin, type I cytoskeletal 14 (K1C14)
Q92764	Keratin, type I cuticular Ha5 (KRT35)
P35527	Keratin, type I cytoskeletal 9 (K1C9)
O95678	Keratin, type II cytoskeletal 75 (K2C75)
P08779	Keratin, type I cytoskeletal 16 (K1C16)
Q04695	Keratin, type I cytoskeletal 17 (K1C17)
Q14532	Keratin, type I cuticular Ha2 (K1H2)
Q9NSB2	Keratin, type II cuticular Hb4 (KRT84)
P15924	Desmoplakin (DESP)
F5GZD1	Keratin, type II cytoskeletal 7 (F5GZD1)
B3KVF5	cDNA FLJ16494 fis, clone CTONG3004576, highly similar to Keratin, type I cytoskeletal 15 (B3KVF5)
Q3SY84	Keratin, type II cytoskeletal 71 (K2C71)
Q86Y46	Keratin, type II cytoskeletal 73 (K2C73)
Q7RTS7	Keratin, type II cytoskeletal 74 (K2C74)
Q07283	Trichohyalin (TRHY)
Q7Z3Y7	Keratin, type I cytoskeletal 28 (K1C28)
B4DRW1	cDNA FLJ55805, highly similar to Keratin, type II cytoskeletal 4 (B4DRW1)
Q7Z3Y8	Keratin, type I cytoskeletal 27 (K1C27)
Q7Z3Z0	Keratin, type I cytoskeletal 25 (K1C25)
Q8WVW5	Putative uncharacterized protein (Fragment) (Q8WVW5)
P14923	Junction plakoglobin (PLAK)
P62805	Histone H4 (H4)
Q2M2I5	Keratin, type I cytoskeletal 24 (K1C24)
Q8N1N4	Keratin, type II cytoskeletal 78 (K2C78)
Q9BYR8	Keratin-associated protein 3-1 (KRA31)
Q93077	Histone H2A type 1-C (H2A1C)
Q8TC04	Keratin 23 (Histone deacetylase inducible) (Q8TC04)
P04792	Heat shock protein beta-1 (HSPB1)
P31947	14-3-3 protein sigma (1433S)
A8MUB1	Tubulin alpha-4A chain (A8MUB1)
P47929	Galectin-7 (LEG7)
O43707	Alpha-actinin-4 (ACTN4)
F8VW92	Tubulin beta chain (F8VW92)
A0N4V7	HCG2039797 (Fragment) (A0N4V7)
Q562R1	Beta-actin-like protein 2 (ACTBL)
Q6A163	Keratin, type I cytoskeletal 39 (K1C39)
A8MTN3	Putative keratin-associated protein ENSP00000381495 (KRA2X)
Q13835-2	Isoform 1 of Plakophilin-1 (PKP1)
Q86UQ4	ATP-binding cassette sub-family A member 13 (ABCAD)
P35579	Myosin-9 (MYH9)
Q6PK75	PRSS2 protein (Fragment) (Q6PK75)
Q9BYR6	Keratin-associated protein 3-3 (KRA33)
Q01469	Fatty acid-binding protein, epidermal (FABP5)
Q02413	Desmoglein-1 (DSG1)
Q2VIP3	PABP3 (Fragment) (Q2VIP3)
P27348	14-3-3 protein theta (1433T)
Q9BYR7	Keratin-associated protein 3-2 (KRA32)
B2RAW1	cDNA, FLJ95155, highly similar to Homo sapiens Bruton agammaglobulinemia tyrosine kinase (BTK), mRNA (B2RAW1)
F8W8Y7	Armadillo repeat-containing X-linked protein 4 (F8W8Y7)
P63104	14-3-3 protein zeta/delta (1433Z)
P81605	Dermcidin (DCD)
P61981	14-3-3 protein gamma (1433G)
Q86TY5	Full-length cDNA clone CS0DI041YE05 of Placenta of Homo sapiens (human) (Q86TY5)
P09382	Galectin-1 (LEG1)
Q09666	Neuroblast differentiation-associated protein AHNAK (AHNK)
Q8NGB0	Seven transmembrane helix receptor (Q8NGB0)
P54652	Heat shock-related 70 kDa protein 2 (HSP72)
B4DHC2	cDNA FLJ56434, highly similar to p130Cas-associated protein (B4DHC2)
A0PK11	Clarin-2 OS=Homo sapiens GN=CLRN2 PE=2 SV=1 - (CLRN2)
Q0Z7S8	Fatty acid-binding protein 9 (FABP9)
P33764	Protein S100-A3 (S10A3)
Q7Z406	Myosin-14 (MYH14)
O95147	Dual specificity protein phosphatase 14 (DUS14)
Q6ISF6	KRTAP9-2 protein (Fragment) (Q6ISF6)
P07737	Profilin-1 (PROF1)
P27482	Calmodulin-like protein 3 (CALL3)
B4DF70	cDNA FLJ60461, highly similar to Peroxiredoxin-2 (EC 1.11.1.15) (B4DF70)
Q86SJ6	Desmoglein-4 (DSG4)
B3KVD6	cDNA FLJ16433 fis, clone BRACE3013936, highly similar to cell death activator CIDE-B OS=Homo sapiens PE=2 SV=1 - (B3KVD6)
Q13228	Selenium-binding protein 1 (SBP1)
P62258	14-3-3 protein epsilon (1433E)
O94819	Kelch repeat and BTB domain-containing protein 11 (KBTBB)
B0AZQ4	cDNA, FLJ79494, highly similar to Structural maintenance of chromosome 3 (B0AZQ4)
Q15828	Cystatin-M (CYTM)
B4DVQ3	Calcium/calmodulin-dependent protein kinase type II subunit gamma (B4DVQ3)
O15173	Membrane-associated progesterone receptor component 2 (PGRC2)
P58107	Epiplakin (EPIPL)
P13639	Elongation factor 2 (EF2)
P37802	Transgelin-2 (TAGL2)
P31949	Protein S100-A11 (S10AB)
P55072	Transitional endoplasmic reticulum ATPase (TERA)
B7Z7A9	Phosphoglycerate kinase (B7Z7A9)
Q9Y6R9	Coiled-coil domain-containing protein 61 (CCD61)
P06733	Alpha-enolase (ENOA)
P36952	Serpin B5 (SPB5)
E5KNB2	DNA-apurinic or apyrimidinic site lyase 2 (E5KNB2)

## Discussion

We compared the 2,961 unique proteins identified in our study with those reported in previous studies on hair shaft proteomics [9,10] (Appendices A-H). The top 50 proteins (based on score value) among these most abundant proteins include 19 type I keratins, 18 type II keratins, seven uncharacterized proteins, keratin 81-like protein, desmoplakin (DESP), annexin (ANXA2), junction plakoglobin (PLAK), trichohyalin (TRHY), and tubulin beta chain (TUBB). Sixty-eight proteins identified in our study were also identified in proteomic profiling of the hair shaft, which detected 401 proteins [9,10]. Another proteomics study designed to develop a method for forensic examination of anagen hair buds with shafts identified 63 proteins [16]. Of these 63 proteins, 55 were also identified in our study, while the remaining eight proteins, including four keratins (KRT6C, KRT39, KRT72, and KRT82) and four KAPs (KRTAP2-3, KRTAP4-11, KRTAP9-7, and KRTAP13-1), were not detected. Thus, compared with the above three studies and other previously published hair follicle proteomics studies, a large proportion of the 2,961 proteins identified in the present study were exclusively identified in this study. This high number of proteins observed here could be mainly due to the higher sensitivity of Orbitrap and/or the inclusion of plucked hair follicles (including both the hair bud and hair shaft). Most earlier studies analyzed only hair shaft proteomics. However, the present study identified more proteins than the forensic method development study, in which small fragments of the hair shaft that included the hair bud were analyzed [16].

Lee et al. reported 343 proteins from the soluble fraction and 300 proteins from the insoluble fraction of hair follicles [9] and identified keratins, junctional proteins, and intracellular proteins, many of which were similar to the proteome of the nail plate [10]. In addition, 58 proteins were identified in the nonsoluble fraction [10]. Twenty-three of these proteins [9] were identical to proteins identified among the 190 common proteins across all samples, and 15 proteins from the other study [10] also overlapped with these proteins (Appendices A-H).

Among the 190 proteins identified in all five samples, there were 15 type I keratins, 14 type II keratins, and four KAPs. The proportion of KAPs was lower, as was also observed in an earlier study [9]. This low proportion of KAPs in the above study [9] and our present study could be due to the heating and denaturing protocol employed. In addition, KAPs may be underrepresented because they are small proteins (10-15 kDa) with limited enzyme cleavage sites and may be missed during identification by Orbitrap analysis. The denaturing extraction procedure used here yielded a low abundance of transglutaminase proteins, which are largely part of the insoluble fraction, as evidenced by the presence of only one transglutaminase-like protein (A8K5N5). This observation is similar to that of the previous study [9], which showed that the number of transglutaminases identified was far lower than that of other proteins. However, we identified trichohyalin in high abundance in all five samples, which shows that trichohyalin is tractable with denaturing extraction protocols, as observed in another recent study [8].

Gene network analysis of common proteins across the five samples showed that keratin 5 (KRT5), with 88 connections, was the most connected node protein, followed by keratin 14 (KRT14) and desmoplakin (DSP), with 78 and 71 connections, respectively (Appendix I). KRT5 and KRT14 are expressed in undifferentiated keratinocytes of the epidermis and are downregulated during differentiation of the basal layer [19], and these proteins have been identified in hair-derived proteins by LC/ESI-MS. The expression of KRT5 in the basal layer increases in the basal layer of the epidermis in radiation-induced alopecic mouse skin [20]. DSP protein and its mRNA expression have also been demonstrated in rat hair follicles in an earlier study [21]. KRT6B, with 70 connections in this network, is related to hair growth [22]. KRT75, an isoform of KRT6, is expressed in the companion layer and upper germinative matrix region of the hair follicle, the medulla of the hair shaft, and in epithelia and the nail bed [23]. It has also been shown to be involved in maintaining hair shaft and nail integrity [23]. Other network proteins, such as KRT17 (in newly regenerating hair follicles) [24], KRT10 (in an in vitro cultured keratinocyte cell line from the hair follicle) [25], and PPL (colocalized with hair follicles) [26], have also been reported in earlier studies. DSG3 is another connected protein in this network, indicating its important biological role in hair follicles. A recent report also demonstrated the role of DSG1 and DSG3 in the companion layer of hair follicles, more specifically in anchoring anagen hair to the follicle [27].

Antimicrobial proteins such as lysozyme C (LYSC), lysozyme g-like protein 2 (LYG2), and S100A8, as reported in earlier studies [14,28], were also observed here. Similarly, histones, including H1.3, H1.5, H2A, H2B, H3, and H4, were identified, as in a previous study [14]. In another proteomic study [29], the authors identified 28 human dermal papilla (DP) cell-specific proteins. Moreover, in the same study, 47 ECM proteins commonly expressed in both DP cells and dermal fibroblasts (DFs), including cytokines (C19ORF10, FAM3C, and AIMP1), growth factors (HDGF and TYMP), transporters (IGFBP3, AFP, and ALB), peptidases (PRSS3, MMP14, TRF, and ERAP1), and an enzyme (GPI), were also identified [29]. In our current study, we also identified TRY (trypsin-3) and TYMP (thymidine phosphorylase), suggesting that the plucked hair follicles possibly included some DP and DF cells or that these proteins are expressed in other parts of the hair follicles, other than in DP and DF cells.

Some variability in the protein profile of hair was observed among different ethnicities [13]. We compared the expression of six common, differentially expressed proteins (KRTAP2-4, KRTAP4-3, KRTAP13-1, KRT40, SBP1, and TGM3) observed among four different ethnicities (Caucasian, African American, Kenyan, and Korean) [13] with those in our study. Of these six proteins, SBP1 was the only protein commonly expressed in all five samples (N1-N5) here. TGM3 was expressed in four samples (N1, N3, N4, and N5), and KRTAP4-3 was expressed in only three samples (N3-N5). KRTAP2-4, KRTAP13-1, and KRT40 were not identified in any of the five samples. This provides some indication that there could be variation in protein expression in Indian scalp hair compared with that in other ethnicities. Another study, using 2D gel electrophoresis and MALDI-TOF/TOF analysis, also reported significantly altered protein abundance of K33B, K81, and K86 in hair shafts among three ethnicities (Malay, Chinese, and Indian) [30]. These proteomic variations could be similar to those observed in differential protein expression among inbred mouse strains [8] and may be akin to differential gene expression among individual humans and variations among specific human populations. However, one recent study has also suggested that there is no statistically significant difference in the proteome of racially and geometrically classified scalp hair samples from a South African cohort [17]. Further proteomic evaluations, including a large number of samples and statistical validation, are required to establish interindividual or interethnic proteomic differences.

Limitations and future studies

While this study presents one of the first comprehensive Orbitrap-based proteomic profiles of human scalp hair follicles from an Indian population, contributing a valuable reference dataset for future studies in hair biology and biomarker discovery, several limitations exist. This study is descriptive rather than hypothesis-driven, lacks quantitative comparative analyses, and focuses on healthy male hair without including balding or other hair conditions. The sample size of this study is small, comprising only five healthy male donors, which limits the generalizability of the findings and prevents meaningful assessment of interindividual biological variability. In addition, the functional pathways and network analyses are predictive bioinformatic interpretations without functional experimental validation. Further studies including larger sample sizes and validation of key identified proteins using Western blotting or immunohistochemistry are required to add statistical significance to the assessment of individual differences and quantitative identification.

## Conclusions

The study provides a proteomic repository useful for the fundamental understanding of hair proteins, and this knowledge can serve as a resource for personalized medicine and biomarker identification. The 2,961 proteins identified here include 362 hypothetical proteins predicted based on cDNA clones, 80 putative proteins, and 36 uncharacterized proteins that have not been reported previously in hair follicles. This study presents a resource for further exploration of their roles in hair biology.

## Appendices

Appendix A

**Table 3:** 2,961 unique proteins identified in this study.

Accession	Protein description
P13647	Keratin, type II cytoskeletal 5 OS=Homo sapiens GN=KRT5 PE=1 SV=3 - (K2C5_HUMAN)
P02533	Keratin, type I cytoskeletal 14 OS=Homo sapiens GN=KRT14 PE=1 SV=4 - (K1C14_HUMAN)
P04259	Keratin, type II cytoskeletal 6B OS=Homo sapiens GN=KRT6B PE=1 SV=5 - (K2C6B_HUMAN)
B4DRR0	cDNA FLJ53910, highly similar to Keratin, type II cytoskeletal 6A OS=Homo sapiens PE=2 SV=1 - (B4DRR0_HUMAN)
B4DKV4	cDNA FLJ60647, highly similar to Keratin, type II cytoskeletal 6B OS=Homo sapiens PE=2 SV=1 - (B4DKV4_HUMAN)
Q04695	Keratin, type I cytoskeletal 17 OS=Homo sapiens GN=KRT17 PE=1 SV=2 - (K1C17_HUMAN)
P08779	Keratin, type I cytoskeletal 16 OS=Homo sapiens GN=KRT16 PE=1 SV=4 - (K1C16_HUMAN)
O43790	Keratin, type II cuticular Hb6 OS=Homo sapiens GN=KRT86 PE=1 SV=1 - (KRT86_HUMAN)
H6VRF8	Keratin 1 OS=Homo sapiens GN=KRT1 PE=3 SV=1 - (H6VRF8_HUMAN)
P78386	Keratin, type II cuticular Hb5 OS=Homo sapiens GN=KRT85 PE=1 SV=1 - (KRT85_HUMAN)
P35908	Keratin, type II cytoskeletal 2 epidermal OS=Homo sapiens GN=KRT2 PE=1 SV=2 - (K22E_HUMAN)
Q15323	Keratin, type I cuticular Ha1 OS=Homo sapiens GN=KRT31 PE=2 SV=3 - (K1H1_HUMAN)
F8W6V8	Keratin, type II cuticular Hb1 OS=Homo sapiens GN=KRT81 PE=3 SV=1 - (F8W6V8_HUMAN)
P78385	Keratin, type II cuticular Hb3 OS=Homo sapiens GN=KRT83 PE=1 SV=2 - (KRT83_HUMAN)
P13645	Keratin, type I cytoskeletal 10 OS=Homo sapiens GN=KRT10 PE=1 SV=6 - (K1C10_HUMAN)
O95678	Keratin, type II cytoskeletal 75 OS=Homo sapiens GN=KRT75 PE=1 SV=2 - (K2C75_HUMAN)
H0YI76	Keratin, type II cytoskeletal 5 (Fragment) OS=Homo sapiens GN=KRT5 PE=3 SV=1 - (H0YI76_HUMAN)
Q14525	Keratin, type I cuticular Ha3-II OS=Homo sapiens GN=KRT33B PE=2 SV=3 - (KT33B_HUMAN)
O76009	Keratin, type I cuticular Ha3-I OS=Homo sapiens GN=KRT33A PE=2 SV=2 - (KT33A_HUMAN)
Q07283	Trichohyalin OS=Homo sapiens GN=TCHH PE=1 SV=2 - (TRHY_HUMAN)
A6NCN2	Keratin-81-like protein OS=Homo sapiens PE=2 SV=3 - (KT81L_HUMAN)
Q5XKE5	Keratin, type II cytoskeletal 79 OS=Homo sapiens GN=KRT79 PE=1 SV=2 - (K2C79_HUMAN)
P15924	Desmoplakin OS=Homo sapiens GN=DSP PE=1 SV=3 - (DESP_HUMAN)
Q3SY84	Keratin, type II cytoskeletal 71 OS=Homo sapiens GN=KRT71 PE=1 SV=3 - (K2C71_HUMAN)
Q01546	Keratin, type II cytoskeletal 2 oral OS=Homo sapiens GN=KRT76 PE=1 SV=2 - (K22O_HUMAN)
B3KVF5	cDNA FLJ16494 fis, clone CTONG3004576, highly similar to Keratin, type I cytoskeletal 15 OS=Homo sapiens PE=2 SV=1 - (B3KVF5_HUMAN)
P35527	Keratin, type I cytoskeletal 9 OS=Homo sapiens GN=KRT9 PE=1 SV=3 - (K1C9_HUMAN)
H9KV34	Uncharacterized protein OS=Homo sapiens PE=3 SV=1 - (H9KV34_HUMAN)
C4AMA3	Keratin, type I cuticular Ha4 OS=Homo sapiens GN=KRT34 PE=3 SV=1 - (C4AMA3_HUMAN)
P08727	Keratin, type I cytoskeletal 19 OS=Homo sapiens GN=KRT19 PE=1 SV=4 - (K1C19_HUMAN)
Q92764	KRTAP11–1
Q7Z3Y8	Keratin, type I cytoskeletal 27 OS=Homo sapiens GN=KRT27 PE=1 SV=2 - (K1C27_HUMAN)
Q7Z3Z0	Keratin, type I cytoskeletal 25 OS=Homo sapiens GN=KRT25 PE=1 SV=1 - (K1C25_HUMAN)
Q86Y46	Keratin, type II cytoskeletal 73 OS=Homo sapiens GN=KRT73 PE=1 SV=1 - (K2C73_HUMAN)
Q7RTS7	Keratin, type II cytoskeletal 74 OS=Homo sapiens GN=KRT74 PE=1 SV=2 - (K2C74_HUMAN)
Q14CN4-2	Isoform 2 of Keratin, type II cytoskeletal 72 OS=Homo sapiens GN=KRT72 - (K2C72_HUMAN)
P13646-3	Isoform 3 of Keratin, type I cytoskeletal 13 OS=Homo sapiens GN=KRT13 - (K1C13_HUMAN)
Q8WVW5	Putative uncharacterized protein (Fragment) OS=Homo sapiens PE=2 SV=1 - (Q8WVW5_HUMAN)
Q9NSB2	Keratin, type II cuticular Hb4 OS=Homo sapiens GN=KRT84 PE=1 SV=2 - (KRT84_HUMAN)
Q7Z3Y7	Keratin, type I cytoskeletal 28 OS=Homo sapiens GN=KRT28 PE=1 SV=2 - (K1C28_HUMAN)
Q9NSB4	Keratin, type II cuticular Hb2 OS=Homo sapiens GN=KRT82 PE=1 SV=3 - (KRT82_HUMAN)
Q7Z794	Keratin, type II cytoskeletal 1b OS=Homo sapiens GN=KRT77 PE=1 SV=3 - (K2C1B_HUMAN)
F5GZD1	Keratin, type II cytoskeletal 7 OS=Homo sapiens GN=KRT7 PE=4 SV=1 - (F5GZD1_HUMAN)
Q14532	Keratin, type I cuticular Ha2 OS=Homo sapiens GN=KRT32 PE=1 SV=3 - (K1H2_HUMAN)
B4DRW1	cDNA FLJ55805, highly similar to Keratin, type II cytoskeletal 4 OS=Homo sapiens PE=2 SV=1 - (B4DRW1_HUMAN)
P07355-2	Isoform 2 of Annexin A2 OS=Homo sapiens GN=ANXA2 - (ANXA2_HUMAN)
P14923	Junction plakoglobin OS=Homo sapiens GN=JUP PE=1 SV=3 - (PLAK_HUMAN)
Q99456	Keratin, type I cytoskeletal 12 OS=Homo sapiens GN=KRT12 PE=1 SV=1 - (K1C12_HUMAN)
B7Z6P1	cDNA FLJ53662, highly similar to Actin, alpha skeletal muscle OS=Homo sapiens PE=2 SV=1 - (B7Z6P1_HUMAN)
F8VW92	Tubulin beta chain OS=Homo sapiens GN=TUBB PE=3 SV=1 - (F8VW92_HUMAN)
P31947	14-3-3 protein sigma OS=Homo sapiens GN=SFN PE=1 SV=1 - (1433S_HUMAN)
P04792	Heat shock protein beta-1 OS=Homo sapiens GN=HSPB1 PE=1 SV=2 - (HSPB1_HUMAN)
B7Z565	cDNA FLJ54739, highly similar to Alpha-actinin-1 OS=Homo sapiens PE=2 SV=1 - (B7Z565_HUMAN)
O43707	Alpha-actinin-4 OS=Homo sapiens GN=ACTN4 PE=1 SV=2 - (ACTN4_HUMAN)
A8MUB1	Tubulin alpha-4A chain OS=Homo sapiens GN=TUBA4A PE=2 SV=1 - (A8MUB1_HUMAN)
G3V1U9	Tubulin alpha-1A chain OS=Homo sapiens GN=TUBA1A PE=3 SV=1 - (G3V1U9_HUMAN)
Q13885	Tubulin beta-2A chain OS=Homo sapiens GN=TUBB2A PE=1 SV=1 - (TBB2A_HUMAN)
B4DE77	cDNA FLJ55189, highly similar to Tubulin beta-4 chain OS=Homo sapiens PE=2 SV=1 - (B4DE77_HUMAN)
B7ZBC3	Spectrin repeat containing, nuclear envelope 1 OS=Homo sapiens GN=SYNE1 PE=4 SV=1 - (B7ZBC3_HUMAN)
Q5QNW6	Histone H2B type 2-F OS=Homo sapiens GN=HIST2H2BF PE=1 SV=3 - (H2B2F_HUMAN)
P04406	Glyceraldehyde-3-phosphate dehydrogenase OS=Homo sapiens GN=GAPDH PE=1 SV=3 - (G3P_HUMAN)
Q562R1	Beta-actin-like protein 2 OS=Homo sapiens GN=ACTBL2 PE=1 SV=2 - (ACTBL_HUMAN)
P62805	Histone H4 OS=Homo sapiens GN=HIST1H4A PE=1 SV=2 - (H4_HUMAN)
Q13835-2	Isoform 1 of Plakophilin-1 OS=Homo sapiens GN=PKP1 - (PKP1_HUMAN)
Q2M2I5	Keratin, type I cytoskeletal 24 OS=Homo sapiens GN=KRT24 PE=1 SV=1 - (K1C24_HUMAN)
Q8TC04	Keratin 23 (Histone deacetylase inducible) OS=Homo sapiens GN=KRT23 PE=2 SV=1 - (Q8TC04_HUMAN)
P47929	Galectin-7 OS=Homo sapiens GN=LGALS7 PE=1 SV=2 - (LEG7_HUMAN)
Q5VTE0	Putative elongation factor 1-alpha-like 3 OS=Homo sapiens GN=EEF1A1P5 PE=5 SV=1 - (EF1A3_HUMAN)
Q15149-3	Isoform 3 of Plectin OS=Homo sapiens GN=PLEC - (PLEC_HUMAN)
Q8N1N4	Keratin, type II cytoskeletal 78 OS=Homo sapiens GN=KRT78 PE=2 SV=2 - (K2C78_HUMAN)
Q9BTM1	Histone H2A.J OS=Homo sapiens GN=H2AFJ PE=1 SV=1 - (H2AJ_HUMAN)
Q93077	Histone H2A type 1-C OS=Homo sapiens GN=HIST1H2AC PE=1 SV=3 - (H2A1C_HUMAN)
Q13509	Tubulin beta-3 chain OS=Homo sapiens GN=TUBB3 PE=1 SV=2 - (TBB3_HUMAN)
P63104	14-3-3 protein zeta/delta OS=Homo sapiens GN=YWHAZ PE=1 SV=1 - (1433Z_HUMAN)
P27348	14-3-3 protein theta OS=Homo sapiens GN=YWHAQ PE=1 SV=1 - (1433T_HUMAN)
P35579	Myosin-9 OS=Homo sapiens GN=MYH9 PE=1 SV=4 - (MYH9_HUMAN)
B5BU24	14-3-3 protein beta/alpha OS=Homo sapiens GN=YWHAB PE=2 SV=1 - (B5BU24_HUMAN)
Q01469	Fatty acid-binding protein, epidermal OS=Homo sapiens GN=FABP5 PE=1 SV=3 - (FABP5_HUMAN)
P11142	Heat shock cognate 71 kDa protein OS=Homo sapiens GN=HSPA8 PE=1 SV=1 - (HSP7C_HUMAN)
P61981	14-3-3 protein gamma OS=Homo sapiens GN=YWHAG PE=1 SV=2 - (1433G_HUMAN)
D3DPG0	Titin, isoform CRA_a OS=Homo sapiens GN=TTN PE=4 SV=1 - (D3DPG0_HUMAN)
Q562V5	Actin-like protein (Fragment) OS=Homo sapiens GN=ACT PE=2 SV=1 - (Q562V5_HUMAN)
P08238	Heat shock protein HSP 90-beta OS=Homo sapiens GN=HSP90AB1 PE=1 SV=4 - (HS90B_HUMAN)
Q9HD64	G antigen family D member 2 OS=Homo sapiens GN=XAGE1A PE=2 SV=3 - (GAGD2_HUMAN)
F5H288	Vimentin OS=Homo sapiens GN=VIM PE=3 SV=1 - (F5H288_HUMAN)
P54652	Heat shock-related 70 kDa protein 2 OS=Homo sapiens GN=HSPA2 PE=1 SV=1 - (HSP72_HUMAN)
O75116	Rho-associated protein kinase 2 OS=Homo sapiens GN=ROCK2 PE=1 SV=4 - (ROCK2_HUMAN)
Q6KB66-2	Isoform 2 of Keratin, type II cytoskeletal 80 OS=Homo sapiens GN=KRT80 - (K2C80_HUMAN)
P06733	Alpha-enolase OS=Homo sapiens GN=ENO1 PE=1 SV=2 - (ENOA_HUMAN)
Q7Z406	Myosin-14 OS=Homo sapiens GN=MYH14 PE=1 SV=2 - (MYH14_HUMAN)
P62258	14-3-3 protein epsilon OS=Homo sapiens GN=YWHAE PE=1 SV=1 - (1433E_HUMAN)
P36952	Serpin B5 OS=Homo sapiens GN=SERPINB5 PE=1 SV=2 - (SPB5_HUMAN)
O75369-2	Isoform 2 of Filamin-B OS=Homo sapiens GN=FLNB - (FLNB_HUMAN)
P07900	Heat shock protein HSP 90-alpha OS=Homo sapiens GN=HSP90AA1 PE=1 SV=5 - (HS90A_HUMAN)
P68431	Histone H3.1 OS=Homo sapiens GN=HIST1H3A PE=1 SV=2 - (H31_HUMAN)
Q96QV6	Histone H2A type 1-A OS=Homo sapiens GN=HIST1H2AA PE=1 SV=3 - (H2A1A_HUMAN)
B4DPP6	cDNA FLJ54371, highly similar to Serum albumin OS=Homo sapiens PE=2 SV=1 - (B4DPP6_HUMAN)
P62979	Ubiquitin-40S ribosomal protein S27a OS=Homo sapiens GN=RPS27A PE=1 SV=2 - (RS27A_HUMAN)
A0N4V7	HCG2039797 (Fragment) OS=Homo sapiens GN=Tcr-alpha PE=4 SV=1 - (A0N4V7_HUMAN)
F8W8Y7	Armadillo repeat-containing X-linked protein 4 OS=Homo sapiens GN=ARMCX4 PE=4 SV=1 - (F8W8Y7_HUMAN)
P62987	Ubiquitin-60S ribosomal protein L40 OS=Homo sapiens GN=UBA52 PE=1 SV=2 - (RL40_HUMAN)
Q0Z7S8	Fatty acid-binding protein 9 OS=Homo sapiens GN=FABP9 PE=1 SV=1 - (FABP9_HUMAN)
Q86TY5	Full-length cDNA clone CS0DI041YE05 of Placenta of Homo sapiens (human) OS=Homo sapiens PE=2 SV=1 - (Q86TY5_HUMAN)
P13639	Elongation factor 2 OS=Homo sapiens GN=EEF2 PE=1 SV=4 - (EF2_HUMAN)
Q8WVJ2	NudC domain-containing protein 2 OS=Homo sapiens GN=NUDCD2 PE=1 SV=1 - (NUDC2_HUMAN)
Q6DN03	Putative histone H2B type 2-C OS=Homo sapiens GN=HIST2H2BC PE=5 SV=3 - (H2B2C_HUMAN)
P04083	Annexin A1 OS=Homo sapiens GN=ANXA1 PE=1 SV=2 - (ANXA1_HUMAN)
H0YG33	Heat shock 70 kDa protein 1A/1B OS=Homo sapiens GN=HSPA1B PE=3 SV=1 - (H0YG33_HUMAN)
Q15828	Cystatin-M OS=Homo sapiens GN=CST6 PE=1 SV=1 - (CYTM_HUMAN)
Q562Q2	Actin-like protein (Fragment) OS=Homo sapiens GN=ACT PE=3 SV=1 - (Q562Q2_HUMAN)
Q09666	Neuroblast differentiation-associated protein AHNAK OS=Homo sapiens GN=AHNAK PE=1 SV=2 - (AHNK_HUMAN)
Q6PK75	PRSS2 protein (Fragment) OS=Homo sapiens GN=PRSS2 PE=2 SV=1 - (Q6PK75_HUMAN)
Q5HY54	Filamin-A OS=Homo sapiens GN=FLNA PE=2 SV=1 - (Q5HY54_HUMAN)
P60174	Triosephosphate isomerase OS=Homo sapiens GN=TPI1 PE=1 SV=3 - (TPIS_HUMAN)
P02545-2	Isoform C of Prelamin-A/C OS=Homo sapiens GN=LMNA - (LMNA_HUMAN)
B3KWI4	cDNA FLJ43122 fis, clone CTONG3003737, highly similar to Leucine-rich repeat-containing protein 15 OS=Homo sapiens PE=2 SV=1 - (B3KWI4_HUMAN)
F8WA45	Gelsolin OS=Homo sapiens GN=GSN PE=4 SV=1 - (F8WA45_HUMAN)
O43175	D-3-phosphoglycerate dehydrogenase OS=Homo sapiens GN=PHGDH PE=1 SV=4 - (SERA_HUMAN)
Q02413	Desmoglein-1 OS=Homo sapiens GN=DSG1 PE=1 SV=2 - (DSG1_HUMAN)
P37802	Transgelin-2 OS=Homo sapiens GN=TAGLN2 PE=1 SV=3 - (TAGL2_HUMAN)
P00338-3	Isoform 3 of L-lactate dehydrogenase A chain OS=Homo sapiens GN=LDHA - (LDHA_HUMAN)
O15066	Kinesin-like protein KIF3B OS=Homo sapiens GN=KIF3B PE=1 SV=1 - (KIF3B_HUMAN)
O94819	Kelch repeat and BTB domain-containing protein 11 OS=Homo sapiens GN=KBTBD11 PE=1 SV=1 - (KBTBB_HUMAN)
P0C0S5	Histone H2A.Z OS=Homo sapiens GN=H2AFZ PE=1 SV=2 - (H2AZ_HUMAN)
Q86UQ4	ATP-binding cassette sub-family A member 13 OS=Homo sapiens GN=ABCA13 PE=2 SV=3 - (ABCAD_HUMAN)
P09211	Glutathione S-transferase P OS=Homo sapiens GN=GSTP1 PE=1 SV=2 - (GSTP1_HUMAN)
P25054-2	Isoform Short of Adenomatous polyposis coli protein OS=Homo sapiens GN=APC - (APC_HUMAN)
Q8IUX7	Adipocyte enhancer-binding protein 1 OS=Homo sapiens GN=AEBP1 PE=1 SV=1 - (AEBP1_HUMAN)
B4DF70	cDNA FLJ60461, highly similar to Peroxiredoxin-2 (EC 1.11.1.15) OS=Homo sapiens PE=2 SV=1 - (B4DF70_HUMAN)
Q3LI55	Keratin associated protein OS=Homo sapiens GN=KRTAP11-1 PE=2 SV=1 - (Q3LI55_HUMAN)
P15311	Ezrin OS=Homo sapiens GN=EZR PE=1 SV=4 - (EZRI_HUMAN)
P40926	Malate dehydrogenase, mitochondrial OS=Homo sapiens GN=MDH2 PE=1 SV=3 - (MDHM_HUMAN)
B4DWS9	cDNA FLJ57640, highly similar to Serpin B5 OS=Homo sapiens PE=2 SV=1 - (B4DWS9_HUMAN)
A8K486	Peptidyl-prolyl cis-trans isomerase OS=Homo sapiens PE=2 SV=1 - (A8K486_HUMAN)
C9J1U3	Protein unc-80 homolog OS=Homo sapiens GN=UNC80 PE=4 SV=1 - (C9J1U3_HUMAN)
P05109	Protein S100-A8 OS=Homo sapiens GN=S100A8 PE=1 SV=1 - (S10A8_HUMAN)
Q86YZ3	Hornerin OS=Homo sapiens GN=HRNR PE=1 SV=2 - (HORN_HUMAN)
Q15057	Arf-GAP with coiled-coil, ANK repeat and PH domain-containing protein 2 OS=Homo sapiens GN=ACAP2 PE=1 SV=3 - (ACAP2_HUMAN)
Q92817	Envoplakin OS=Homo sapiens GN=EVPL PE=1 SV=3 - (EVPL_HUMAN)
P27482	Calmodulin-like protein 3 OS=Homo sapiens GN=CALML3 PE=1 SV=2 - (CALL3_HUMAN)
P14618	Pyruvate kinase isozymes M1/M2 OS=Homo sapiens GN=PKM2 PE=1 SV=4 - (KPYM_HUMAN)
P55072	Transitional endoplasmic reticulum ATPase OS=Homo sapiens GN=VCP PE=1 SV=4 - (TERA_HUMAN)
P16402	Histone H1.3 OS=Homo sapiens GN=HIST1H1D PE=1 SV=2 - (H13_HUMAN)
P31949	Protein S100-A11 OS=Homo sapiens GN=S100A11 PE=1 SV=2 - (S10AB_HUMAN)
Q06830	Peroxiredoxin-1 OS=Homo sapiens GN=PRDX1 PE=1 SV=1 - (PRDX1_HUMAN)
Q14204	Cytoplasmic dynein 1 heavy chain 1 OS=Homo sapiens GN=DYNC1H1 PE=1 SV=5 - (DYHC1_HUMAN)
B0AZQ4	cDNA, FLJ79494, highly similar to Structural maintenance of chromosome 3 OS=Homo sapiens PE=2 SV=1 - (B0AZQ4_HUMAN)
H0YIG3	Keratin, type II cytoskeletal 72 (Fragment) OS=Homo sapiens GN=KRT72 PE=4 SV=1 - (H0YIG3_HUMAN)
P33764	Protein S100-A3 OS=Homo sapiens GN=S100A3 PE=1 SV=1 - (S10A3_HUMAN)
C9JH62	Zinc finger protein 277 (Fragment) OS=Homo sapiens GN=ZNF277 PE=4 SV=1 - (C9JH62_HUMAN)
P09382	Galectin-1 OS=Homo sapiens GN=LGALS1 PE=1 SV=2 - (LEG1_HUMAN)
Q8WUT4	Leucine-rich repeat neuronal protein 4 OS=Homo sapiens GN=LRRN4 PE=1 SV=3 - (LRRN4_HUMAN)
P28838-2	Isoform 2 of Cytosol aminopeptidase OS=Homo sapiens GN=LAP3 - (AMPL_HUMAN)
B2R4K7	60S ribosomal protein L6 OS=Homo sapiens PE=2 SV=1 - (B2R4K7_HUMAN)
Q14134	Tripartite motif-containing protein 29 OS=Homo sapiens GN=TRIM29 PE=1 SV=2 - (TRI29_HUMAN)
Q96IR1	RPS4X protein (Fragment) OS=Homo sapiens GN=RPS4X PE=2 SV=2 - (Q96IR1_HUMAN)
P07737	Profilin-1 OS=Homo sapiens GN=PFN1 PE=1 SV=2 - (PROF1_HUMAN)
F5H6K1	Dystrophin OS=Homo sapiens GN=DMD PE=4 SV=1 - (F5H6K1_HUMAN)
Q9UPN3	Microtubule-actin cross-linking factor 1, isoforms 1/2/3/5 OS=Homo sapiens GN=MACF1 PE=1 SV=4 - (MACF1_HUMAN)
P46782	40S ribosomal protein S5 OS=Homo sapiens GN=RPS5 PE=1 SV=4 - (RS5_HUMAN)
A3RLL6	Cellular apoptosis susceptibility protein variant 2 OS=Homo sapiens GN=CSE1L-2 PE=2 SV=1 - (A3RLL6_HUMAN)
P07195	L-lactate dehydrogenase B chain OS=Homo sapiens GN=LDHB PE=1 SV=2 - (LDHB_HUMAN)
F8VW21	60S acidic ribosomal protein P0 (Fragment) OS=Homo sapiens GN=RPLP0 PE=3 SV=1 - (F8VW21_HUMAN)
Q8NAM9	cDNA FLJ35099 fis, clone PLACE6006287 OS=Homo sapiens PE=2 SV=1 - (Q8NAM9_HUMAN)
A5D8V7	Coiled-coil domain-containing protein 151 OS=Homo sapiens GN=CCDC151 PE=2 SV=1 - (CC151_HUMAN)
O95613	Pericentrin OS=Homo sapiens GN=PCNT PE=1 SV=4 - (PCNT_HUMAN)
B7Z7A9	Phosphoglycerate kinase OS=Homo sapiens GN=PGK1 PE=2 SV=1 - (B7Z7A9_HUMAN)
A2RRR7	CCDC46 protein OS=Homo sapiens GN=CCDC46 PE=2 SV=1 - (A2RRR7_HUMAN)
Q96M86	Dynein heavy chain domain-containing protein 1 OS=Homo sapiens GN=DNHD1 PE=1 SV=2 - (DNHD1_HUMAN)
O15230	Laminin subunit alpha-5 OS=Homo sapiens GN=LAMA5 PE=1 SV=8 - (LAMA5_HUMAN)
A8K4Z4	cDNA FLJ75549, highly similar to Homo sapiens ribosomal protein, large, P0 (RPLP0), transcript variant 1, mRNA OS=Homo sapiens PE=2 SV=1 - (A8K4Z4_HUMAN)
O60437	Periplakin OS=Homo sapiens GN=PPL PE=1 SV=4 - (PEPL_HUMAN)
Q9UKE5-4	Isoform 4 of TRAF2 and NCK-interacting protein kinase OS=Homo sapiens GN=TNIK - (TNIK_HUMAN)
I0B0K8	Truncated profilaggrin OS=Homo sapiens GN=FLG PE=4 SV=1 - (I0B0K8_HUMAN)
P07476	Involucrin OS=Homo sapiens GN=IVL PE=1 SV=2 - (INVO_HUMAN)
A8K4W0	cDNA FLJ78591, highly similar to Homo sapiens ribosomal protein S3A (RPS3A), mRNA OS=Homo sapiens PE=2 SV=1 - (A8K4W0_HUMAN)
Q8IWY4	Signal peptide, CUB and EGF-like domain-containing protein 1 OS=Homo sapiens GN=SCUBE1 PE=1 SV=3 - (SCUB1_HUMAN)
B2RAW1	cDNA, FLJ95155, highly similar to Homo sapiens Bruton agammaglobulinemia tyrosine kinase (BTK), mRNA OS=Homo sapiens PE=2 SV=1 - (B2RAW1_HUMAN)
B4DSW9	cDNA FLJ59415, highly similar to Beta-catenin OS=Homo sapiens PE=2 SV=1 - (B4DSW9_HUMAN)
P07339	Cathepsin D OS=Homo sapiens GN=CTSD PE=1 SV=1 - (CATD_HUMAN)
B4DM97	cDNA FLJ55002, highly similar to Alpha-centractin OS=Homo sapiens PE=2 SV=1 - (B4DM97_HUMAN)
Q9P2M7	Cingulin OS=Homo sapiens GN=CGN PE=1 SV=2 - (CING_HUMAN)
Q96JS0	Tubby homologue (Fragment) OS=Homo sapiens GN=TUB PE=2 SV=1 - (Q96JS0_HUMAN)
E9PDB1	Non-erythrocytic beta-spectrin 4 OS=Homo sapiens GN=SPTBN4 PE=2 SV=2 - (E9PDB1_HUMAN)
G8JLA2	Myosin light polypeptide 6 OS=Homo sapiens GN=MYL6 PE=4 SV=1 - (G8JLA2_HUMAN)
P11021	78 kDa glucose-regulated protein OS=Homo sapiens GN=HSPA5 PE=1 SV=2 - (GRP78_HUMAN)
P49327	Fatty acid synthase OS=Homo sapiens GN=FASN PE=1 SV=3 - (FAS_HUMAN)
Q9Y446	Plakophilin-3 OS=Homo sapiens GN=PKP3 PE=1 SV=1 - (PKP3_HUMAN)
Q9NS87-2	Isoform 2 of Kinesin-like protein KIF15 OS=Homo sapiens GN=KIF15 - (KIF15_HUMAN)
O95447	Lebercilin-like protein OS=Homo sapiens GN=LCA5L PE=2 SV=1 - (LCA5L_HUMAN)
Q14152	Eukaryotic translation initiation factor 3 subunit A OS=Homo sapiens GN=EIF3A PE=1 SV=1 - (EIF3A_HUMAN)
P15090	Fatty acid-binding protein, adipocyte OS=Homo sapiens GN=FABP4 PE=1 SV=3 - (FABP4_HUMAN)
Q53TK3	Putative uncharacterized protein PRO2015 OS=Homo sapiens GN=PRO2015 PE=4 SV=1 - (Q53TK3_HUMAN)
Q5T6C5	Ataxin-7-like protein 2 OS=Homo sapiens GN=ATXN7L2 PE=2 SV=1 - (AT7L2_HUMAN)
E1NZA1	Peroxisome proliferator activated receptor interacting complex protein OS=Homo sapiens GN=PRIC295 PE=2 SV=1 - (E1NZA1_HUMAN)
Q99968	Tpr OS=Homo sapiens GN=tpr PE=2 SV=1 - (Q99968_HUMAN)
P04075	Fructose-bisphosphate aldolase A OS=Homo sapiens GN=ALDOA PE=1 SV=2 - (ALDOA_HUMAN)
P08758	Annexin A5 OS=Homo sapiens GN=ANXA5 PE=1 SV=2 - (ANXA5_HUMAN)
Q8WXH0	Nesprin-2 OS=Homo sapiens GN=SYNE2 PE=1 SV=3 - (SYNE2_HUMAN)
Q99996-5	Isoform 5 of A-kinase anchor protein 9 OS=Homo sapiens GN=AKAP9 - (AKAP9_HUMAN)
Q9NRC6	Spectrin beta chain, brain 4 OS=Homo sapiens GN=SPTBN5 PE=1 SV=1 - (SPTN5_HUMAN)
Q8IWJ2	GRIP and coiled-coil domain-containing protein 2 OS=Homo sapiens GN=GCC2 PE=1 SV=4 - (GCC2_HUMAN)
H3BLZ6	Sterol regulatory element-binding protein 1 (Fragment) OS=Homo sapiens GN=SREBF1 PE=4 SV=1 - (H3BLZ6_HUMAN)
P22626-2	Isoform A2 of Heterogeneous nuclear ribonucleoproteins A2/B1 OS=Homo sapiens GN=HNRNPA2B1 - (ROA2_HUMAN)
Q92556	Engulfment and cell motility protein 1 OS=Homo sapiens GN=ELMO1 PE=1 SV=2 - (ELMO1_HUMAN)
Q0QEN7	ATP synthase subunit beta (Fragment) OS=Homo sapiens GN=ATP5B PE=2 SV=1 - (Q0QEN7_HUMAN)
Q8NGB0	Seven transmembrane helix receptor OS=Homo sapiens GN=GPR142 PE=2 SV=1 - (Q8NGB0_HUMAN)
Q5T7N8	Protein FAM27D1 OS=Homo sapiens GN=FAM27D1 PE=3 SV=2 - (F27D1_HUMAN)
Q5CAQ5	Tumor rejection antigen (Gp96) 1 OS=Homo sapiens GN=TRA1 PE=2 SV=1 - (Q5CAQ5_HUMAN)
P22392-2	Isoform 3 of Nucleoside diphosphate kinase B OS=Homo sapiens GN=NME2 - (NDKB_HUMAN)
B2RDE1	cDNA, FLJ96568, highly similar to Homo sapiens tropomyosin 3 (TPM3), mRNA OS=Homo sapiens PE=2 SV=1 - (B2RDE1_HUMAN)
B4DHC2	cDNA FLJ56434, highly similar to p130Cas-associated protein OS=Homo sapiens PE=2 SV=1 - (B4DHC2_HUMAN)
O00362	Putative p150 OS=Homo sapiens PE=4 SV=1 - (O00362_HUMAN)
B2RTY4-4	Isoform 4 of Unconventional myosin-IXa OS=Homo sapiens GN=MYO9A - (MYO9A_HUMAN)
Q32MZ4	Leucine-rich repeat flightless-interacting protein 1 OS=Homo sapiens GN=LRRFIP1 PE=1 SV=2 - (LRRF1_HUMAN)
Q6ZQQ6	WD repeat-containing protein 87 OS=Homo sapiens GN=WDR87 PE=2 SV=3 - (WDR87_HUMAN)
C9J9K3	40S ribosomal protein SA (Fragment) OS=Homo sapiens GN=RPSA PE=3 SV=1 - (C9J9K3_HUMAN)
Q2VIP3	PABP3 (Fragment) OS=Homo sapiens PE=2 SV=1 - (Q2VIP3_HUMAN)
A6NCW0	Ubiquitin carboxyl-terminal hydrolase 17-like protein 3 OS=Homo sapiens GN=USP17L3 PE=3 SV=1 - (U17L3_HUMAN)
P58107	Epiplakin OS=Homo sapiens GN=EPPK1 PE=1 SV=2 - (EPIPL_HUMAN)
A8MTN3	Putative keratin-associated protein ENSP00000381495 OS=Homo sapiens PE=3 SV=2 - (KRA2X_HUMAN)
B7Z4T3	cDNA FLJ51903, highly similar to Stress-70 protein, mitochondrial OS=Homo sapiens PE=2 SV=1 - (B7Z4T3_HUMAN)
O60486	Plexin-C1 OS=Homo sapiens GN=PLXNC1 PE=1 SV=1 - (PLXC1_HUMAN)
Q6ZS99	cDNA FLJ45706 fis, clone FEBRA2028457, highly similar to Nucleolin OS=Homo sapiens PE=2 SV=1 - (Q6ZS99_HUMAN)
Q96Q15	Serine/threonine-protein kinase SMG1 OS=Homo sapiens GN=SMG1 PE=1 SV=3 - (SMG1_HUMAN)
P31151	Protein S100-A7 OS=Homo sapiens GN=S100A7 PE=1 SV=4 - (S10A7_HUMAN)
P81605	Dermcidin OS=Homo sapiens GN=DCD PE=1 SV=2 - (DCD_HUMAN)
A7MCY6	TANK-binding kinase 1-binding protein 1 OS=Homo sapiens GN=TBKBP1 PE=1 SV=1 - (TBKB1_HUMAN)
F5H7F0	Protein unc-13 homolog C OS=Homo sapiens GN=UNC13C PE=4 SV=1 - (F5H7F0_HUMAN)
P18669	Phosphoglycerate mutase 1 OS=Homo sapiens GN=PGAM1 PE=1 SV=2 - (PGAM1_HUMAN)
Q14574-2	Isoform 3B of Desmocollin-3 OS=Homo sapiens GN=DSC3 - (DSC3_HUMAN)
Q08ES8	Cell growth-inhibiting protein 34 OS=Homo sapiens PE=2 SV=1 - (Q08ES8_HUMAN)
Q96AG4	Leucine-rich repeat-containing protein 59 OS=Homo sapiens GN=LRRC59 PE=1 SV=1 - (LRC59_HUMAN)
B2R4M6	cDNA, FLJ92148, highly similar to Homo sapiens S100 calcium binding protein A9 (calgranulin B) (S100A9), mRNA OS=Homo sapiens PE=4 SV=1 - (B2R4M6_HUMAN)
Q4W4Y1	Dopamine receptor interacting protein 4 OS=Homo sapiens GN=DRIP4 PE=2 SV=1 - (Q4W4Y1_HUMAN)
O00221	NF-kappa-B inhibitor epsilon OS=Homo sapiens GN=NFKBIE PE=1 SV=3 - (IKBE_HUMAN)
Q99575	Ribonucleases P/MRP protein subunit POP1 OS=Homo sapiens GN=POP1 PE=1 SV=2 - (POP1_HUMAN)
F5H7T3	Vinculin OS=Homo sapiens GN=VCL PE=4 SV=1 - (F5H7T3_HUMAN)
A4QPB0	IQ motif containing GTPase activating protein 1 OS=Homo sapiens GN=IQGAP1 PE=2 SV=1 - (A4QPB0_HUMAN)
A6H8Y1-7	Isoform 7 of Transcription factor TFIIIB component B'' homolog OS=Homo sapiens GN=BDP1 - (BDP1_HUMAN)
P30050	60S ribosomal protein L12 OS=Homo sapiens GN=RPL12 PE=1 SV=1 - (RL12_HUMAN)
Q9P219	Protein Daple OS=Homo sapiens GN=CCDC88C PE=1 SV=3 - (DAPLE_HUMAN)
P62277	40S ribosomal protein S13 OS=Homo sapiens GN=RPS13 PE=1 SV=2 - (RS13_HUMAN)
B4DXS5	cDNA FLJ57802, highly similar to Zinc finger protein 225 OS=Homo sapiens PE=2 SV=1 - (B4DXS5_HUMAN)
P98164	Low-density lipoprotein receptor-related protein 2 OS=Homo sapiens GN=LRP2 PE=1 SV=3 - (LRP2_HUMAN)
E9PK25	Cofilin-1 OS=Homo sapiens GN=CFL1 PE=4 SV=1 - (E9PK25_HUMAN)
E1U1N1	Ubiquitously transcribed tetratricopeptide repeat protein Y-linked transcript variant 252 OS=Homo sapiens GN=UTY PE=2 SV=1 - (E1U1N1_HUMAN)
Q9H694	Protein bicaudal C homolog 1 OS=Homo sapiens GN=BICC1 PE=1 SV=2 - (BICC1_HUMAN)
F5GX82	Protein furry homolog-like OS=Homo sapiens GN=FRYL PE=4 SV=1 - (F5GX82_HUMAN)
P51587	Breast cancer type 2 susceptibility protein OS=Homo sapiens GN=BRCA2 PE=1 SV=2 - (BRCA2_HUMAN)
Q9P2D1	Chromodomain-helicase-DNA-binding protein 7 OS=Homo sapiens GN=CHD7 PE=1 SV=3 - (CHD7_HUMAN)
Q5U5K4	Homer homolog 1 (Drosophila) OS=Homo sapiens GN=HOMER1 PE=2 SV=1 - (Q5U5K4_HUMAN)
Q4L180-2	Isoform 2 of Filamin A-interacting protein 1-like OS=Homo sapiens GN=FILIP1L - (FIL1L_HUMAN)
Q5BJF6	Outer dense fiber protein 2 OS=Homo sapiens GN=ODF2 PE=1 SV=1 - (ODFP2_HUMAN)
P26641	Elongation factor 1-gamma OS=Homo sapiens GN=EEF1G PE=1 SV=3 - (EF1G_HUMAN)
Q86SG7	Lysozyme g-like protein 2 OS=Homo sapiens GN=LYG2 PE=1 SV=2 - (LYG2_HUMAN)
O75182-2	Isoform 2 of Paired amphipathic helix protein Sin3b OS=Homo sapiens GN=SIN3B - (SIN3B_HUMAN)
Q5HYW3	Retrotransposon gag domain-containing protein 4 OS=Homo sapiens GN=RGAG4 PE=2 SV=1 - (RGAG4_HUMAN)
P63244	Guanine nucleotide-binding protein subunit beta-2-like 1 OS=Homo sapiens GN=GNB2L1 PE=1 SV=3 - (GBLP_HUMAN)
Q92833	Protein Jumonji OS=Homo sapiens GN=JARID2 PE=1 SV=2 - (JARD2_HUMAN)
P16401	Histone H1.5 OS=Homo sapiens GN=HIST1H1B PE=1 SV=3 - (H15_HUMAN)
Q9HBI0-3	Isoform 3 of Gamma-parvin OS=Homo sapiens GN=PARVG - (PARVG_HUMAN)
P62249	40S ribosomal protein S16 OS=Homo sapiens GN=RPS16 PE=1 SV=2 - (RS16_HUMAN)
Q15365	Poly(rC)-binding protein 1 OS=Homo sapiens GN=PCBP1 PE=1 SV=2 - (PCBP1_HUMAN)
P07237	Protein disulfide-isomerase OS=Homo sapiens GN=P4HB PE=1 SV=3 - (PDIA1_HUMAN)
H0Y2N2	Ribonuclease 3 OS=Homo sapiens GN=DROSHA PE=4 SV=1 - (H0Y2N2_HUMAN)
E9PCF4	Kelch-like protein 5 OS=Homo sapiens GN=KLHL5 PE=4 SV=1 - (E9PCF4_HUMAN)
Q86SJ6	Desmoglein-4 OS=Homo sapiens GN=DSG4 PE=1 SV=1 - (DSG4_HUMAN)
P62269	40S ribosomal protein S18 OS=Homo sapiens GN=RPS18 PE=1 SV=3 - (RS18_HUMAN)
A0PK11	Clarin-2 OS=Homo sapiens GN=CLRN2 PE=2 SV=1 - (CLRN2_HUMAN)
Q04656-5	Isoform 5 of Copper-transporting ATPase 1 OS=Homo sapiens GN=ATP7A - (ATP7A_HUMAN)
Q68CX2	Putative uncharacterized protein DKFZp761A1822 OS=Homo sapiens GN=DKFZp761A1822 PE=2 SV=2 - (Q68CX2_HUMAN)
Q71RH4	FP1047 OS=Homo sapiens PE=2 SV=1 - (Q71RH4_HUMAN)
Q96QE3	ATPase family AAA domain-containing protein 5 OS=Homo sapiens GN=ATAD5 PE=1 SV=4 - (ATAD5_HUMAN)
Q9BTQ7	Similar to ribosomal protein L23 (Fragment) OS=Homo sapiens PE=1 SV=1 - (Q9BTQ7_HUMAN)
B2R717	cDNA, FLJ93235, highly similar to Homo sapiens cathepsin L2 (CTSL2), mRNA OS=Homo sapiens PE=2 SV=1 - (B2R717_HUMAN)
B2R5W2	cDNA, FLJ92657, highly similar to Homo sapiens heterogeneous nuclear ribonucleoprotein C (C1/C2) (HNRPC), transcript variant 2, mRNA OS=Homo sapiens PE=2 SV=1 - (B2R5W2_HUMAN)
Q13228	Selenium-binding protein 1 OS=Homo sapiens GN=SELENBP1 PE=1 SV=2 - (SBP1_HUMAN)
Q5JR95	40S ribosomal protein S8 OS=Homo sapiens GN=RPS8 PE=4 SV=1 - (Q5JR95_HUMAN)
Q8NI35	InaD-like protein OS=Homo sapiens GN=INADL PE=1 SV=3 - (INADL_HUMAN)
Q96IV0-3	Isoform 3 of Peptide-N(4)-(N-acetyl-beta-glucosaminyl)asparagine amidase OS=Homo sapiens GN=NGLY1 - (NGLY1_HUMAN)
P09651-3	Isoform 2 of Heterogeneous nuclear ribonucleoprotein A1 OS=Homo sapiens GN=HNRNPA1 - (ROA1_HUMAN)
Q5VYY1	Ankyrin repeat domain-containing protein 22 OS=Homo sapiens GN=ANKRD22 PE=2 SV=1 - (ANR22_HUMAN)
O75145-2	Isoform 2 of Liprin-alpha-3 OS=Homo sapiens GN=PPFIA3 - (LIPA3_HUMAN)
Q9UKN7	Unconventional myosin-XV OS=Homo sapiens GN=MYO15A PE=1 SV=2 - (MYO15_HUMAN)
P29034	Protein S100-A2 OS=Homo sapiens GN=S100A2 PE=1 SV=3 - (S10A2_HUMAN)
P50395	Rab GDP dissociation inhibitor beta OS=Homo sapiens GN=GDI2 PE=1 SV=2 - (GDIB_HUMAN)
Q96IU2	Zinc finger BED domain-containing protein 3 OS=Homo sapiens GN=ZBED3 PE=1 SV=1 - (ZBED3_HUMAN)
P62851	40S ribosomal protein S25 OS=Homo sapiens GN=RPS25 PE=1 SV=1 - (RS25_HUMAN)
C9JNW5	60S ribosomal protein L24 OS=Homo sapiens GN=RPL24 PE=4 SV=1 - (C9JNW5_HUMAN)
Q8NEZ4	Histone-lysine N-methyltransferase MLL3 OS=Homo sapiens GN=MLL3 PE=1 SV=3 - (MLL3_HUMAN)
Q9NZM3-3	Isoform 3 of Intersectin-2 OS=Homo sapiens GN=ITSN2 - (ITSN2_HUMAN)
Q6DT37	Serine/threonine-protein kinase MRCK gamma OS=Homo sapiens GN=CDC42BPG PE=1 SV=2 - (MRCKG_HUMAN)
P13928	Annexin A8 OS=Homo sapiens GN=ANXA8 PE=1 SV=3 - (ANXA8_HUMAN)
O95759	TBC1 domain family member 8 OS=Homo sapiens GN=TBC1D8 PE=1 SV=3 - (TBCD8_HUMAN)
P16152	Carbonyl reductase (NADPH) 1 OS=Homo sapiens GN=CBR1 PE=1 SV=3 - (CBR1_HUMAN)
E7ETZ4	Basic leucine zipper and W2 domain-containing protein 2 (Fragment) OS=Homo sapiens GN=BZW2 PE=4 SV=1 - (E7ETZ4_HUMAN)
Q5VU13	V-set and immunoglobulin domain-containing protein 8 OS=Homo sapiens GN=VSIG8 PE=1 SV=1 - (VSIG8_HUMAN)
Q96QA5	Gasdermin-A OS=Homo sapiens GN=GSDMA PE=2 SV=4 - (GSDMA_HUMAN)
F5H7C4	Methionine-R-sulfoxide reductase B3 (Fragment) OS=Homo sapiens GN=MSRB3 PE=4 SV=1 - (F5H7C4_HUMAN)
E9PC74	Translation initiation factor eIF-2B subunit epsilon OS=Homo sapiens GN=EIF2B5 PE=4 SV=1 - (E9PC74_HUMAN)
Q8NGN2	Olfactory receptor 10S1 OS=Homo sapiens GN=OR10S1 PE=2 SV=2 - (O10S1_HUMAN)
P04080	Cystatin-B OS=Homo sapiens GN=CSTB PE=1 SV=2 - (CYTB_HUMAN)
Q8WVX7	Ribosomal protein S19 (Fragment) OS=Homo sapiens PE=2 SV=1 - (Q8WVX7_HUMAN)
P62857	40S ribosomal protein S28 OS=Homo sapiens GN=RPS28 PE=1 SV=1 - (RS28_HUMAN)
Q9ULL8-2	Isoform 2 of Protein Shroom4 OS=Homo sapiens GN=SHROOM4 - (SHRM4_HUMAN)
B4DLZ5	N-acetylglucosamine kinase OS=Homo sapiens GN=NAGK PE=2 SV=1 - (B4DLZ5_HUMAN)
Q9UJ55	MAGE-like protein 2 OS=Homo sapiens GN=MAGEL2 PE=1 SV=1 - (MAGL2_HUMAN)
Q17RC7	Exocyst complex component 3-like protein 4 OS=Homo sapiens GN=EXOC3L4 PE=2 SV=2 - (EX3L4_HUMAN)
Q66LE6	Serine/threonine-protein phosphatase 2A 55 kDa regulatory subunit B delta isoform OS=Homo sapiens GN=PPP2R2D PE=1 SV=1 - (2ABD_HUMAN)
D6RAD4	Cyclin-dependent kinase 7 OS=Homo sapiens GN=CDK7 PE=4 SV=1 - (D6RAD4_HUMAN)
Q9BYR8	Keratin-associated protein 3-1 OS=Homo sapiens GN=KRTAP3-1 PE=1 SV=1 - (KRA31_HUMAN)
Q8N3C0	Activating signal cointegrator 1 complex subunit 3 OS=Homo sapiens GN=ASCC3 PE=1 SV=3 - (ASCC3_HUMAN)
O60282	Kinesin heavy chain isoform 5C OS=Homo sapiens GN=KIF5C PE=1 SV=1 - (KIF5C_HUMAN)
F8WD26	LIM domain only protein 7 OS=Homo sapiens GN=LMO7 PE=4 SV=1 - (F8WD26_HUMAN)
A2ABS5	Dimethylarginine dimethylaminohydrolase 2 (Fragment) OS=Homo sapiens GN=DDAH2 PE=4 SV=1 - (A2ABS5_HUMAN)
Q8N264	Rho GTPase-activating protein 24 OS=Homo sapiens GN=ARHGAP24 PE=1 SV=2 - (RHG24_HUMAN)
B2RCA2	cDNA, FLJ95932, Homo sapiens polyamine modulated factor 1 binding protein 1(PMFBP1), mRNA OS=Homo sapiens PE=2 SV=1 - (B2RCA2_HUMAN)
O43497-5	Isoform 4 of Voltage-dependent T-type calcium channel subunit alpha-1G OS=Homo sapiens GN=CACNA1G - (CAC1G_HUMAN)
H3BTF6	Uncharacterized protein C16orf59 (Fragment) OS=Homo sapiens GN=C16orf59 PE=4 SV=1 - (H3BTF6_HUMAN)
Q8IYB4-2	Isoform 2 of PEX5-related protein OS=Homo sapiens GN=PEX5L - (PEX5R_HUMAN)
O75629	Protein CREG1 OS=Homo sapiens GN=CREG1 PE=1 SV=1 - (CREG1_HUMAN)
B3W5Y6	Squamous cell carcinoma antigen-1 isoform SCCA-PD OS=Homo sapiens GN=SERPINB3 PE=2 SV=1 - (B3W5Y6_HUMAN)
Q149N8-4	Isoform 3 of E3 ubiquitin-protein ligase SHPRH OS=Homo sapiens GN=SHPRH - (SHPRH_HUMAN)
Q86VG2	Splicing factor proline/glutamine-rich (Polypyrimidine tract binding protein associated) OS=Homo sapiens GN=SFPQ PE=2 SV=1 - (Q86VG2_HUMAN)
A8K092	ATP synthase subunit alpha OS=Homo sapiens GN=ATP5A1 PE=2 SV=1 - (A8K092_HUMAN)
Q13515	Phakinin OS=Homo sapiens GN=BFSP2 PE=1 SV=1 - (BFSP2_HUMAN)
P01024	Complement C3 OS=Homo sapiens GN=C3 PE=1 SV=2 - (CO3_HUMAN)
Q5T7W0-2	Isoform 2 of Zinc finger protein 618 OS=Homo sapiens GN=ZNF618 - (ZN618_HUMAN)
Q9BTI9	NPM1 protein (Fragment) OS=Homo sapiens GN=NPM1 PE=2 SV=2 - (Q9BTI9_HUMAN)
B4DLR3	cDNA FLJ54020, highly similar to Heterogeneous nuclear ribonucleoprotein U OS=Homo sapiens PE=2 SV=1 - (B4DLR3_HUMAN)
B3KS79	cDNA FLJ35730 fis, clone TESTI2003131, highly similar to ALPHA-1-ANTICHYMOTRYPSIN OS=Homo sapiens PE=2 SV=1 - (B3KS79_HUMAN)
B3KVD6	cDNA FLJ16433 fis, clone BRACE3013936, highly similar to Cell death activator CIDE-B OS=Homo sapiens PE=2 SV=1 - (B3KVD6_HUMAN)
Q5K634	SCCA2/SCCA1 fusion protein isoform 1 OS=Homo sapiens PE=2 SV=1 - (Q5K634_HUMAN)
Q00610-2	Isoform 2 of Clathrin heavy chain 1 OS=Homo sapiens GN=CLTC - (CLH1_HUMAN)
O60930	Ribonuclease H1 OS=Homo sapiens GN=RNASEH1 PE=1 SV=2 - (RNH1_HUMAN)
P12236	ADP/ATP translocase 3 OS=Homo sapiens GN=SLC25A6 PE=1 SV=4 - (ADT3_HUMAN)
E9PBB0	PERQ amino acid-rich with GYF domain-containing protein 2 OS=Homo sapiens GN=GIGYF2 PE=4 SV=1 - (E9PBB0_HUMAN)
Q71UH4	DNA topoisomerase 2 (Fragment) OS=Homo sapiens GN=TOP2B PE=2 SV=1 - (Q71UH4_HUMAN)
P09467	Fructose-1,6-bisphosphatase 1 OS=Homo sapiens GN=FBP1 PE=1 SV=5 - (F16P1_HUMAN)
P45880-2	Isoform 2 of Voltage-dependent anion-selective channel protein 2 OS=Homo sapiens GN=VDAC2 - (VDAC2_HUMAN)
Q9NYJ1	Coiled-coil-helix-coiled-coil-helix domain-containing protein 8 OS=Homo sapiens GN=CHCHD8 PE=1 SV=2 - (CHCH8_HUMAN)
B3KMF7	cDNA FLJ10863 fis, clone NT2RP4001575, highly similar to Vacuolar protein sorting protein 52 OS=Homo sapiens PE=2 SV=1 - (B3KMF7_HUMAN)
E7EQG2	Eukaryotic initiation factor 4A-II OS=Homo sapiens GN=EIF4A2 PE=3 SV=1 - (E7EQG2_HUMAN)
P52272-2	Isoform 2 of Heterogeneous nuclear ribonucleoprotein M OS=Homo sapiens GN=HNRNPM - (HNRPM_HUMAN)
B4DSG0	Janus kinase and microtubule-interacting protein 2 OS=Homo sapiens GN=JAKMIP2 PE=2 SV=1 - (B4DSG0_HUMAN)
Q3LIE3	Putative uncharacterized protein Nbla04137 (Fragment) OS=Homo sapiens GN=Nbla04137 PE=2 SV=1 - (Q3LIE3_HUMAN)
B3KX11	cDNA FLJ44436 fis, clone UTERU2019706, highly similar to T-complex protein 1 subunit gamma OS=Homo sapiens PE=2 SV=1 - (B3KX11_HUMAN)
P05090	Apolipoprotein D OS=Homo sapiens GN=APOD PE=1 SV=1 - (APOD_HUMAN)
Q9HCY8	Protein S100-A14 OS=Homo sapiens GN=S100A14 PE=1 SV=1 - (S10AE_HUMAN)
H0YFF8	UPF0606 protein C11orf41 OS=Homo sapiens GN=C11orf41 PE=4 SV=1 - (H0YFF8_HUMAN)
Q9H7E2-3	Isoform 3 of Tudor domain-containing protein 3 OS=Homo sapiens GN=TDRD3 - (TDRD3_HUMAN)
P10599	Thioredoxin OS=Homo sapiens GN=TXN PE=1 SV=3 - (THIO_HUMAN)
P29373	Cellular retinoic acid-binding protein 2 OS=Homo sapiens GN=CRABP2 PE=1 SV=2 - (RABP2_HUMAN)
Q9Y6R9	Coiled-coil domain-containing protein 61 OS=Homo sapiens GN=CCDC61 PE=1 SV=2 - (CCD61_HUMAN)
P25787	Proteasome subunit alpha type-2 OS=Homo sapiens GN=PSMA2 PE=1 SV=2 - (PSA2_HUMAN)
P39023	60S ribosomal protein L3 OS=Homo sapiens GN=RPL3 PE=1 SV=2 - (RL3_HUMAN)
P31944	Caspase-14 OS=Homo sapiens GN=CASP14 PE=1 SV=2 - (CASPE_HUMAN)
A6NGS5	Laminin subunit alpha-2 OS=Homo sapiens GN=LAMA2 PE=4 SV=2 - (A6NGS5_HUMAN)
B4DL86	6-phosphogluconate dehydrogenase, decarboxylating OS=Homo sapiens PE=2 SV=1 - (B4DL86_HUMAN)
P0CG31	Putative zinc finger protein 286B OS=Homo sapiens GN=ZNF286B PE=5 SV=1 - (Z286B_HUMAN)
Q8TDW7-3	Isoform 3 of Protocadherin Fat 3 OS=Homo sapiens GN=FAT3 - (FAT3_HUMAN)
B4DSU0	cDNA FLJ61379, highly similar to Sterol regulatory element-binding protein 2 OS=Homo sapiens PE=2 SV=1 - (B4DSU0_HUMAN)
P00918	Carbonic anhydrase 2 OS=Homo sapiens GN=CA2 PE=1 SV=2 - (CAH2_HUMAN)
F8VXI9	ARF GTPase-activating protein GIT2 OS=Homo sapiens GN=GIT2 PE=4 SV=1 - (F8VXI9_HUMAN)
Q8WXX0	Dynein heavy chain 7, axonemal OS=Homo sapiens GN=DNAH7 PE=1 SV=2 - (DYH7_HUMAN)
A2A3R5	40S ribosomal protein S6 OS=Homo sapiens GN=RPS6 PE=3 SV=1 - (A2A3R5_HUMAN)
Q13867	Bleomycin hydrolase OS=Homo sapiens GN=BLMH PE=1 SV=1 - (BLMH_HUMAN)
Q9BVP2-2	Isoform 2 of Guanine nucleotide-binding protein-like 3 OS=Homo sapiens GN=GNL3 - (GNL3_HUMAN)
Q5JPC5	Putative uncharacterized protein DKFZp667E1714 OS=Homo sapiens GN=DKFZp667E1714 PE=2 SV=1 - (Q5JPC5_HUMAN)
Q5VTQ0-6	Isoform 6 of Tetratricopeptide repeat protein 39B OS=Homo sapiens GN=TTC39B - (TT39B_HUMAN)
Q8IY63-2	Isoform 2 of Angiomotin-like protein 1 OS=Homo sapiens GN=AMOTL1 - (AMOL1_HUMAN)
Q8N3D4	EH domain-binding protein 1-like protein 1 OS=Homo sapiens GN=EHBP1L1 PE=1 SV=2 - (EH1L1_HUMAN)
H9KVA1	Solute carrier family 22 member 17 OS=Homo sapiens GN=SLC22A17 PE=4 SV=1 - (H9KVA1_HUMAN)
O15173	Membrane-associated progesterone receptor component 2 OS=Homo sapiens GN=PGRMC2 PE=1 SV=1 - (PGRC2_HUMAN)
B4DI38	Adenylyl cyclase-associated protein OS=Homo sapiens PE=2 SV=1 - (B4DI38_HUMAN)
P32926	Desmoglein-3 OS=Homo sapiens GN=DSG3 PE=1 SV=2 - (DSG3_HUMAN)
Q08188	Protein-glutamine gamma-glutamyltransferase E OS=Homo sapiens GN=TGM3 PE=1 SV=4 - (TGM3_HUMAN)
P06744-2	Isoform 2 of Glucose-6-phosphate isomerase OS=Homo sapiens GN=GPI - (G6PI_HUMAN)
P46783	40S ribosomal protein S10 OS=Homo sapiens GN=RPS10 PE=1 SV=1 - (RS10_HUMAN)
P80188	Neutrophil gelatinase-associated lipocalin OS=Homo sapiens GN=LCN2 PE=1 SV=2 - (NGAL_HUMAN)
Q99714	3-hydroxyacyl-CoA dehydrogenase type-2 OS=Homo sapiens GN=HSD17B10 PE=1 SV=3 - (HCD2_HUMAN)
E7ENM8	Tyrosine-protein kinase Fes/Fps OS=Homo sapiens GN=FES PE=3 SV=1 - (E7ENM8_HUMAN)
E9PL10	Transcription factor BTF3 homolog 4 OS=Homo sapiens GN=BTF3L4 PE=4 SV=1 - (E9PL10_HUMAN)
Q08999	Retinoblastoma-like protein 2 OS=Homo sapiens GN=RBL2 PE=1 SV=3 - (RBL2_HUMAN)
Q96BY7	Autophagy-related protein 2 homolog B OS=Homo sapiens GN=ATG2B PE=1 SV=5 - (ATG2B_HUMAN)
C9J5W6	Transgelin-3 OS=Homo sapiens GN=TAGLN3 PE=4 SV=1 - (C9J5W6_HUMAN)
P02649	Apolipoprotein E OS=Homo sapiens GN=APOE PE=1 SV=1 - (APOE_HUMAN)
A8MXP9	Matrin-3 OS=Homo sapiens GN=MATR3 PE=4 SV=1 - (A8MXP9_HUMAN)
Q17RV3	Leucine-rich repeat kinase 2 OS=Homo sapiens GN=LRRK2 PE=2 SV=1 - (Q17RV3_HUMAN)
P12532	Creatine kinase U-type, mitochondrial OS=Homo sapiens GN=CKMT1A PE=1 SV=1 - (KCRU_HUMAN)
Q7Z3D4	LysM and putative peptidoglycan-binding domain-containing protein 3 OS=Homo sapiens GN=LYSMD3 PE=2 SV=2 - (LYSM3_HUMAN)
P23396	40S ribosomal protein S3 OS=Homo sapiens GN=RPS3 PE=1 SV=2 - (RS3_HUMAN)
E9PIY1	Protein kinase C and casein kinase substrate in neurons protein 3 OS=Homo sapiens GN=PACSIN3 PE=4 SV=1 - (E9PIY1_HUMAN)
O60502-2	Isoform 2 of Bifunctional protein NCOAT OS=Homo sapiens GN=MGEA5 - (NCOAT_HUMAN)
B0QY59	GTP binding protein 1 (Fragment) OS=Homo sapiens GN=GTPBP1 PE=4 SV=1 - (B0QY59_HUMAN)
E7EVA0	Microtubule-associated protein OS=Homo sapiens GN=MAP4 PE=4 SV=1 - (E7EVA0_HUMAN)
P61088	Ubiquitin-conjugating enzyme E2 N OS=Homo sapiens GN=UBE2N PE=1 SV=1 - (UBE2N_HUMAN)
P15880	40S ribosomal protein S2 OS=Homo sapiens GN=RPS2 PE=1 SV=2 - (RS2_HUMAN)
P35913	Rod cGMP-specific 3',5'-cyclic phosphodiesterase subunit beta OS=Homo sapiens GN=PDE6B PE=1 SV=2 - (PDE6B_HUMAN)
I3L0X1	Fatty aldehyde dehydrogenase OS=Homo sapiens GN=ALDH3A2 PE=4 SV=1 - (I3L0X1_HUMAN)
Q6P452	Annexin OS=Homo sapiens GN=ANXA4 PE=2 SV=1 - (Q6P452_HUMAN)
P60866	40S ribosomal protein S20 OS=Homo sapiens GN=RPS20 PE=1 SV=1 - (RS20_HUMAN)
D6RAF8	Heterogeneous nuclear ribonucleoprotein D0 (Fragment) OS=Homo sapiens GN=HNRNPD PE=4 SV=1 - (D6RAF8_HUMAN)
Q6A163	Keratin, type I cytoskeletal 39 OS=Homo sapiens GN=KRT39 PE=1 SV=2 - (K1C39_HUMAN)
Q9UPY3	Endoribonuclease Dicer OS=Homo sapiens GN=DICER1 PE=1 SV=3 - (DICER_HUMAN)
Q9Y2I6	Ninein-like protein OS=Homo sapiens GN=NINL PE=1 SV=2 - (NINL_HUMAN)
P56715	Oxygen-regulated protein 1 OS=Homo sapiens GN=RP1 PE=1 SV=1 - (RP1_HUMAN)
D3DUP2	WNK lysine deficient protein kinase 1, isoform CRA_d OS=Homo sapiens GN=WNK1 PE=4 SV=1 - (D3DUP2_HUMAN)
P02549-2	Isoform 2 of Spectrin alpha chain, erythrocyte OS=Homo sapiens GN=SPTA1 - (SPTA1_HUMAN)
A8K8P3-9	Isoform 9 of Protein SFI1 homolog OS=Homo sapiens GN=SFI1 - (SFI1_HUMAN)
Q9P2D3-3	Isoform 3 of HEAT repeat-containing protein 5B OS=Homo sapiens GN=HEATR5B - (HTR5B_HUMAN)
P35221	Catenin alpha-1 OS=Homo sapiens GN=CTNNA1 PE=1 SV=1 - (CTNA1_HUMAN)
F5GY37	Prohibitin-2 OS=Homo sapiens GN=PHB2 PE=4 SV=1 - (F5GY37_HUMAN)
F6RFD5	Destrin OS=Homo sapiens GN=DSTN PE=4 SV=1 - (F6RFD5_HUMAN)
Q96EY7	Pentatricopeptide repeat-containing protein 3, mitochondrial OS=Homo sapiens GN=PTCD3 PE=1 SV=3 - (PTCD3_HUMAN)
B4DL14	ATP synthase gamma chain OS=Homo sapiens GN=ATP5C1 PE=2 SV=1 - (B4DL14_HUMAN)
P25391	Laminin subunit alpha-1 OS=Homo sapiens GN=LAMA1 PE=1 SV=2 - (LAMA1_HUMAN)
B4DMP5	cDNA FLJ60050, highly similar to Vacuolar protein sorting protein 72 homolog OS=Homo sapiens PE=2 SV=1 - (B4DMP5_HUMAN)
E7EVE5	Rap guanine nucleotide exchange factor 4 OS=Homo sapiens GN=RAPGEF4 PE=4 SV=1 - (E7EVE5_HUMAN)
B4DG54	cDNA FLJ56635 OS=Homo sapiens PE=2 SV=1 - (B4DG54_HUMAN)
P63173	60S ribosomal protein L38 OS=Homo sapiens GN=RPL38 PE=1 SV=2 - (RL38_HUMAN)
P30041	Peroxiredoxin-6 OS=Homo sapiens GN=PRDX6 PE=1 SV=3 - (PRDX6_HUMAN)
P37837	Transaldolase OS=Homo sapiens GN=TALDO1 PE=1 SV=2 - (TALDO_HUMAN)
H0YKY3	Proline-serine-threonine phosphatase-interacting protein 1 OS=Homo sapiens GN=PSTPIP1 PE=4 SV=1 - (H0YKY3_HUMAN)
Q02487-2	Isoform 2B of Desmocollin-2 OS=Homo sapiens GN=DSC2 - (DSC2_HUMAN)
P54920	Alpha-soluble NSF attachment protein OS=Homo sapiens GN=NAPA PE=1 SV=3 - (SNAA_HUMAN)
Q9BV44	THUMP domain-containing protein 3 OS=Homo sapiens GN=THUMPD3 PE=1 SV=1 - (THUM3_HUMAN)
Q96FQ6	Protein S100-A16 OS=Homo sapiens GN=S100A16 PE=1 SV=1 - (S10AG_HUMAN)
Q6ZMS3	cDNA FLJ16726 fis, clone UTERU3014791 OS=Homo sapiens PE=2 SV=1 - (Q6ZMS3_HUMAN)
P28062-2	Isoform 2 of Proteasome subunit beta type-8 OS=Homo sapiens GN=PSMB8 - (PSB8_HUMAN)
B7Z478	Proteasome (Prosome, macropain) subunit, beta type, 2, isoform CRA_b OS=Homo sapiens GN=PSMB2 PE=2 SV=1 - (B7Z478_HUMAN)
Q9NVU3	cDNA FLJ10506 fis, clone NT2RP2000510 OS=Homo sapiens PE=2 SV=1 - (Q9NVU3_HUMAN)
Q7Z612	Acidic ribosomal phosphoprotein P1 OS=Homo sapiens PE=4 SV=1 - (Q7Z612_HUMAN)
B4DL49	cDNA FLJ58073, moderately similar to Cathepsin B (EC 3.4.22.1) OS=Homo sapiens PE=2 SV=1 - (B4DL49_HUMAN)
Q6YHK3	CD109 antigen OS=Homo sapiens GN=CD109 PE=1 SV=2 - (CD109_HUMAN)
B4DMN1	cDNA FLJ61136, highly similar to Ras-related protein Rab-11A OS=Homo sapiens PE=2 SV=1 - (B4DMN1_HUMAN)
Q9NVE4	Coiled-coil domain-containing protein 87 OS=Homo sapiens GN=CCDC87 PE=2 SV=2 - (CCD87_HUMAN)
C9JP48	Serine/threonine-protein phosphatase (Fragment) OS=Homo sapiens GN=PPP1CB PE=3 SV=1 - (C9JP48_HUMAN)
P14735	Insulin-degrading enzyme OS=Homo sapiens GN=IDE PE=1 SV=4 - (IDE_HUMAN)
Q0IJ56	ST13 protein (Fragment) OS=Homo sapiens GN=ST13 PE=2 SV=1 - (Q0IJ56_HUMAN)
Q5JWF2-2	Isoform XLas-2 of Guanine nucleotide-binding protein G(s) subunit alpha isoforms XLas OS=Homo sapiens GN=GNAS - (GNAS1_HUMAN)
Q9P2Z0	THAP domain-containing protein 10 OS=Homo sapiens GN=THAP10 PE=2 SV=1 - (THA10_HUMAN)
Q9UIS4	Small nuclear ribonucleoprotein-associated protein OS=Homo sapiens GN=SNRPB PE=2 SV=1 - (Q9UIS4_HUMAN)
E9PR44	Alpha-crystallin B chain (Fragment) OS=Homo sapiens GN=CRYAB PE=3 SV=1 - (E9PR44_HUMAN)
Q8NFA0	Ubiquitin carboxyl-terminal hydrolase 32 OS=Homo sapiens GN=USP32 PE=1 SV=1 - (UBP32_HUMAN)
Q86TB3	Alpha-protein kinase 2 OS=Homo sapiens GN=ALPK2 PE=1 SV=3 - (ALPK2_HUMAN)
P60900	Proteasome subunit alpha type-6 OS=Homo sapiens GN=PSMA6 PE=1 SV=1 - (PSA6_HUMAN)
Q86Z22	Putative uncharacterized protein OS=Homo sapiens PE=2 SV=1 - (Q86Z22_HUMAN)
P0CW22	40S ribosomal protein S17-like OS=Homo sapiens GN=RPS17L PE=3 SV=1 - (RS17L_HUMAN)
B4DRD7	Poly(rC)-binding protein 2 OS=Homo sapiens GN=PCBP2 PE=2 SV=1 - (B4DRD7_HUMAN)
Q13435	Splicing factor 3B subunit 2 OS=Homo sapiens GN=SF3B2 PE=1 SV=2 - (SF3B2_HUMAN)
F5H1Y4	Golgi-associated PDZ and coiled-coil motif-containing protein OS=Homo sapiens GN=GOPC PE=4 SV=1 - (F5H1Y4_HUMAN)
O00299	Chloride intracellular channel protein 1 OS=Homo sapiens GN=CLIC1 PE=1 SV=4 - (CLIC1_HUMAN)
P62280	40S ribosomal protein S11 OS=Homo sapiens GN=RPS11 PE=1 SV=3 - (RS11_HUMAN)
B4DRM3	cDNA FLJ54492, highly similar to Eukaryotic translation initiation factor 4B OS=Homo sapiens PE=2 SV=1 - (B4DRM3_HUMAN)
P11940-2	Isoform 2 of Polyadenylate-binding protein 1 OS=Homo sapiens GN=PABPC1 - (PABP1_HUMAN)
B8ZZK4	60S ribosomal protein L31 OS=Homo sapiens GN=RPL31 PE=4 SV=1 - (B8ZZK4_HUMAN)
P59998	Actin-related protein 2/3 complex subunit 4 OS=Homo sapiens GN=ARPC4 PE=1 SV=3 - (ARPC4_HUMAN)
P84077	ADP-ribosylation factor 1 OS=Homo sapiens GN=ARF1 PE=1 SV=2 - (ARF1_HUMAN)
Q8N300	Coiled-coil domain-containing protein 23 OS=Homo sapiens GN=CCDC23 PE=2 SV=1 - (CCD23_HUMAN)
P46781	40S ribosomal protein S9 OS=Homo sapiens GN=RPS9 PE=1 SV=3 - (RS9_HUMAN)
P30044	Peroxiredoxin-5, mitochondrial OS=Homo sapiens GN=PRDX5 PE=1 SV=4 - (PRDX5_HUMAN)
Q92788	Rad GTPase OS=Homo sapiens PE=2 SV=1 - (Q92788_HUMAN)
H3BQZ9	Adenine phosphoribosyltransferase OS=Homo sapiens GN=APRT PE=4 SV=1 - (H3BQZ9_HUMAN)
B7Z382	Cytosolic purine 5'-nucleotidase OS=Homo sapiens GN=NT5C2 PE=2 SV=1 - (B7Z382_HUMAN)
Q5Y190	Anchor protein OS=Homo sapiens GN=RESDA1 PE=2 SV=1 - (Q5Y190_HUMAN)
P18077	60S ribosomal protein L35a OS=Homo sapiens GN=RPL35A PE=1 SV=2 - (RL35A_HUMAN)
Q53RT3	Retroviral-like aspartic protease 1 OS=Homo sapiens GN=ASPRV1 PE=1 SV=1 - (APRV1_HUMAN)
Q8TCJ6	Putative uncharacterized protein DKFZp667C0210 (Fragment) OS=Homo sapiens GN=DKFZp667C0210 PE=2 SV=1 - (Q8TCJ6_HUMAN)
F2Z2C0	Exosome complex exonuclease RRP44 OS=Homo sapiens GN=DIS3 PE=4 SV=1 - (F2Z2C0_HUMAN)
O60506-2	Isoform 2 of Heterogeneous nuclear ribonucleoprotein Q OS=Homo sapiens GN=SYNCRIP - (HNRPQ_HUMAN)
Q9H2F9	Coiled-coil domain-containing protein 68 OS=Homo sapiens GN=CCDC68 PE=2 SV=1 - (CCD68_HUMAN)
Q9NVK5-2	Isoform 2 of FGFR1 oncogene partner 2 OS=Homo sapiens GN=FGFR1OP2 - (FGOP2_HUMAN)
Q9BUP0	EF-hand domain-containing protein D1 OS=Homo sapiens GN=EFHD1 PE=1 SV=1 - (EFHD1_HUMAN)
Q05877	Hepatocellular carcinoma protein HHCM OS=Homo sapiens PE=4 SV=1 - (HHCM_HUMAN)
Q9Y4F4	Protein FAM179B OS=Homo sapiens GN=FAM179B PE=1 SV=4 - (F179B_HUMAN)
A8MUD9	60S ribosomal protein L7 OS=Homo sapiens GN=RPL7 PE=3 SV=1 - (A8MUD9_HUMAN)
Q16176	Lsk protein OS=Homo sapiens GN=lsk PE=2 SV=1 - (Q16176_HUMAN)
P62263	40S ribosomal protein S14 OS=Homo sapiens GN=RPS14 PE=1 SV=3 - (RS14_HUMAN)
P62854	40S ribosomal protein S26 OS=Homo sapiens GN=RPS26 PE=1 SV=3 - (RS26_HUMAN)
F5GX11	Proteasome subunit alpha type-1 OS=Homo sapiens GN=PSMA1 PE=4 SV=1 - (F5GX11_HUMAN)
P36543	V-type proton ATPase subunit E 1 OS=Homo sapiens GN=ATP6V1E1 PE=1 SV=1 - (VATE1_HUMAN)
Q15126	Phosphomevalonate kinase OS=Homo sapiens GN=PMVK PE=1 SV=3 - (PMVK_HUMAN)
P23284	Peptidyl-prolyl cis-trans isomerase B OS=Homo sapiens GN=PPIB PE=1 SV=2 - (PPIB_HUMAN)
Q6ZME5	cDNA FLJ23974 fis, clone HEP18860 OS=Homo sapiens PE=2 SV=1 - (Q6ZME5_HUMAN)
Q8NDA2	Hemicentin-2 OS=Homo sapiens GN=HMCN2 PE=2 SV=2 - (HMCN2_HUMAN)
E7EPK6	40S ribosomal protein S24 OS=Homo sapiens GN=RPS24 PE=3 SV=1 - (E7EPK6_HUMAN)
P25398	40S ribosomal protein S12 OS=Homo sapiens GN=RPS12 PE=1 SV=3 - (RS12_HUMAN)
B4DJP7	Small nuclear ribonucleoprotein Sm D3 OS=Homo sapiens GN=SNRPD3 PE=2 SV=1 - (B4DJP7_HUMAN)
P63241-2	Isoform 2 of Eukaryotic translation initiation factor 5A-1 OS=Homo sapiens GN=EIF5A - (IF5A1_HUMAN)
B7Z399	cDNA FLJ50162, highly similar to Glycosyltransferase-like protein LARGE1 (EC 2.4.-.-) OS=Homo sapiens PE=2 SV=1 - (B7Z399_HUMAN)
H0YJA0	Putative E3 ubiquitin-protein ligase UBR7 (Fragment) OS=Homo sapiens GN=UBR7 PE=4 SV=1 - (H0YJA0_HUMAN)
Q5T321	Neurobeachin OS=Homo sapiens GN=NBEA PE=2 SV=1 - (Q5T321_HUMAN)
Q9ULG6-4	Isoform 4 of Cell cycle progression protein 1 OS=Homo sapiens GN=CCPG1 - (CCPG1_HUMAN)
P05089	Arginase-1 OS=Homo sapiens GN=ARG1 PE=1 SV=2 - (ARGI1_HUMAN)
B4DHK5	cDNA FLJ61590, moderately similar to Pleckstrin homology domain-containing family A member 5 (Fragment) OS=Homo sapiens PE=2 SV=1 - (B4DHK5_HUMAN)
P09493	Tropomyosin alpha-1 chain OS=Homo sapiens GN=TPM1 PE=1 SV=2 - (TPM1_HUMAN)
A4QN26	NBPF4 protein (Fragment) OS=Homo sapiens GN=NBPF4 PE=2 SV=1 - (A4QN26_HUMAN)
D3DV26	S100 calcium binding protein A10 (Annexin II ligand, calpactin I, light polypeptide (P11)), isoform CRA_b (Fragment) OS=Homo sapiens GN=S100A10 PE=4 SV=1 - (D3DV26_HUMAN)
P56730	Neurotrypsin OS=Homo sapiens GN=PRSS12 PE=2 SV=2 - (NETR_HUMAN)
E9PI98	Layilin (Fragment) OS=Homo sapiens GN=LAYN PE=4 SV=1 - (E9PI98_HUMAN)
P40925	Malate dehydrogenase, cytoplasmic OS=Homo sapiens GN=MDH1 PE=1 SV=4 - (MDHC_HUMAN)
O14662-2	Isoform A of Syntaxin-16 OS=Homo sapiens GN=STX16 - (STX16_HUMAN)
I3L3P7	40S ribosomal protein S15a OS=Homo sapiens GN=RPS15A PE=4 SV=1 - (I3L3P7_HUMAN)
Q5T6W2	Heterogeneous nuclear ribonucleoprotein K (Fragment) OS=Homo sapiens GN=HNRNPK PE=2 SV=1 - (Q5T6W2_HUMAN)
O14497	AT-rich interactive domain-containing protein 1A OS=Homo sapiens GN=ARID1A PE=1 SV=3 - (ARI1A_HUMAN)
Q8NEY1-7	Isoform 7 of Neuron navigator 1 OS=Homo sapiens GN=NAV1 - (NAV1_HUMAN)
Q9BWC7	COMT protein OS=Homo sapiens GN=COMT PE=2 SV=2 - (Q9BWC7_HUMAN)
P62750	60S ribosomal protein L23a OS=Homo sapiens GN=RPL23A PE=1 SV=1 - (RL23A_HUMAN)
E7EPB3	60S ribosomal protein L14 OS=Homo sapiens GN=RPL14 PE=4 SV=1 - (E7EPB3_HUMAN)
Q9NNZ3	DnaJ homolog subfamily C member 4 OS=Homo sapiens GN=DNAJC4 PE=2 SV=1 - (DNJC4_HUMAN)
B4E086	cDNA FLJ51584, highly similar to Homo sapiens spindle pole body component 24 homolog (SPBC24), mRNA OS=Homo sapiens PE=2 SV=1 - (B4E086_HUMAN)
Q7Z4W8	Heparin-binding protein HBp15 OS=Homo sapiens PE=2 SV=1 - (Q7Z4W8_HUMAN)
H7BXZ3	Unconventional myosin-VIIa OS=Homo sapiens GN=MYO7A PE=4 SV=1 - (H7BXZ3_HUMAN)
Q5D862	Filaggrin-2 OS=Homo sapiens GN=FLG2 PE=1 SV=1 - (FILA2_HUMAN)
P49207	60S ribosomal protein L34 OS=Homo sapiens GN=RPL34 PE=1 SV=3 - (RL34_HUMAN)
P20618	Proteasome subunit beta type-1 OS=Homo sapiens GN=PSMB1 PE=1 SV=2 - (PSB1_HUMAN)
Q96HZ4-2	Isoform 2 of Transcription cofactor HES-6 OS=Homo sapiens GN=HES6 - (HES6_HUMAN)
B7ZAV1	cDNA, FLJ79315, highly similar to Proto-oncogene DBL OS=Homo sapiens PE=2 SV=1 - (B7ZAV1_HUMAN)
Q8NEY8-3	Isoform 3 of Periphilin-1 OS=Homo sapiens GN=PPHLN1 - (PPHLN_HUMAN)
P61077	Ubiquitin-conjugating enzyme E2 D3 OS=Homo sapiens GN=UBE2D3 PE=1 SV=1 - (UB2D3_HUMAN)
P07108-4	Isoform 4 of Acyl-CoA-binding protein OS=Homo sapiens GN=DBI - (ACBP_HUMAN)
Q9UBT7-3	Isoform 3 of Alpha-catulin OS=Homo sapiens GN=CTNNAL1 - (CTNL1_HUMAN)
Q6ZPB4	cDNA FLJ26129 fis, clone TMS02971 OS=Homo sapiens PE=2 SV=1 - (Q6ZPB4_HUMAN)
B3KTS5	cDNA FLJ38670 fis, clone HSYRA2000190, highly similar to Voltage-dependent anion-selective channel protein 1 OS=Homo sapiens PE=2 SV=1 - (B3KTS5_HUMAN)
P26373	60S ribosomal protein L13 OS=Homo sapiens GN=RPL13 PE=1 SV=4 - (RL13_HUMAN)
O75962	Triple functional domain protein OS=Homo sapiens GN=TRIO PE=1 SV=2 - (TRIO_HUMAN)
E5RI99	60S ribosomal protein L30 (Fragment) OS=Homo sapiens GN=RPL30 PE=3 SV=1 - (E5RI99_HUMAN)
P62424	60S ribosomal protein L7a OS=Homo sapiens GN=RPL7A PE=1 SV=2 - (RL7A_HUMAN)
G3V193	Activating molecule in BECN1-regulated autophagy protein 1 OS=Homo sapiens GN=AMBRA1 PE=4 SV=1 - (G3V193_HUMAN)
P28074	Proteasome subunit beta type-5 OS=Homo sapiens GN=PSMB5 PE=1 SV=3 - (PSB5_HUMAN)
Q8WX93-2	Isoform 2 of Palladin OS=Homo sapiens GN=PALLD - (PALLD_HUMAN)
B3KUZ8	Aspartate aminotransferase OS=Homo sapiens PE=2 SV=1 - (B3KUZ8_HUMAN)
P18621	60S ribosomal protein L17 OS=Homo sapiens GN=RPL17 PE=1 SV=3 - (RL17_HUMAN)
P61626	Lysozyme C OS=Homo sapiens GN=LYZ PE=1 SV=1 - (LYSC_HUMAN)
P62826	GTP-binding nuclear protein Ran OS=Homo sapiens GN=RAN PE=1 SV=3 - (RAN_HUMAN)
Q2M1Z3	Rho GTPase-activating protein 31 OS=Homo sapiens GN=ARHGAP31 PE=1 SV=2 - (RHG31_HUMAN)
Q9UNM1	Chaperonin 10-related protein (Fragment) OS=Homo sapiens GN=EPFP1 PE=2 SV=1 - (Q9UNM1_HUMAN)
Q8ND71	GTPase IMAP family member 8 OS=Homo sapiens GN=GIMAP8 PE=1 SV=2 - (GIMA8_HUMAN)
B7Z539	cDNA FLJ56954, highly similar to Inter-alpha-trypsin inhibitor heavy chain H1 OS=Homo sapiens PE=2 SV=1 - (B7Z539_HUMAN)
P46776	60S ribosomal protein L27a OS=Homo sapiens GN=RPL27A PE=1 SV=2 - (RL27A_HUMAN)
O14818	Proteasome subunit alpha type-7 OS=Homo sapiens GN=PSMA7 PE=1 SV=1 - (PSA7_HUMAN)
A8MXN1	Receptor expression-enhancing protein 6 OS=Homo sapiens GN=REEP6 PE=4 SV=1 - (A8MXN1_HUMAN)
F5GY69	Serpin B11 OS=Homo sapiens GN=SERPINB11 PE=3 SV=1 - (F5GY69_HUMAN)
Q9NTK5	Obg-like ATPase 1 OS=Homo sapiens GN=OLA1 PE=1 SV=2 - (OLA1_HUMAN)
B9A6J0	Ecotropic viral integration site 5 OS=Homo sapiens GN=EVI5 PE=2 SV=1 - (B9A6J0_HUMAN)
O60284	Suppression of tumorigenicity 18 protein OS=Homo sapiens GN=ST18 PE=1 SV=1 - (ST18_HUMAN)
B4DUB7	Phosphorylase OS=Homo sapiens PE=2 SV=1 - (B4DUB7_HUMAN)
P00491	Purine nucleoside phosphorylase OS=Homo sapiens GN=PNP PE=1 SV=2 - (PNPH_HUMAN)
A8K0T9	cDNA FLJ75422, highly similar to Homo sapiens capping protein (actin filament) muscle Z-line, alpha 1, mRNA OS=Homo sapiens PE=2 SV=1 - (A8K0T9_HUMAN)
Q96EA4	Protein Spindly OS=Homo sapiens GN=CCDC99 PE=1 SV=2 - (SPDLY_HUMAN)
Q9H9L3	Interferon-stimulated 20 kDa exonuclease-like 2 OS=Homo sapiens GN=ISG20L2 PE=1 SV=1 - (I20L2_HUMAN)
F8VUA6	60S ribosomal protein L18 (Fragment) OS=Homo sapiens GN=RPL18 PE=4 SV=1 - (F8VUA6_HUMAN)
B4DJQ8	cDNA FLJ55694, highly similar to Dipeptidyl-peptidase 1 (EC 3.4.14.1) OS=Homo sapiens PE=2 SV=1 - (B4DJQ8_HUMAN)
B2RBG3	cDNA, FLJ95493, highly similar to Homo sapiens fucosidase, alpha-L- 1, tissue (FUCA1), mRNA OS=Homo sapiens PE=2 SV=1 - (B2RBG3_HUMAN)
B7Z233	Acetyl-CoA acetyltransferase, cytosolic OS=Homo sapiens GN=ACAT2 PE=2 SV=1 - (B7Z233_HUMAN)
P62266	40S ribosomal protein S23 OS=Homo sapiens GN=RPS23 PE=1 SV=3 - (RS23_HUMAN)
E9PIZ3	60S ribosomal protein L8 OS=Homo sapiens GN=RPL8 PE=4 SV=1 - (E9PIZ3_HUMAN)
F5H4R1	Mitogen-activated protein kinase kinase kinase 4 OS=Homo sapiens GN=MAP3K4 PE=4 SV=1 - (F5H4R1_HUMAN)
Q5T749	Keratinocyte proline-rich protein OS=Homo sapiens GN=KPRP PE=1 SV=1 - (KPRP_HUMAN)
Q2TNB3	Cell migration-inducing protein 22 OS=Homo sapiens PE=2 SV=1 - (Q2TNB3_HUMAN)
B3KUT2	cDNA FLJ40562 fis, clone THYMU2003981, moderately similar to Rattus norvegicus macrophage expressed gene 1 (Mpeg1), mRNA OS=Homo sapiens PE=2 SV=1 - (B3KUT2_HUMAN)
Q8IYY4	Zinc finger protein DZIP1L OS=Homo sapiens GN=DZIP1L PE=2 SV=2 - (DZI1L_HUMAN)
Q0D2H7	PCF11 protein (Fragment) OS=Homo sapiens GN=PCF11 PE=2 SV=1 - (Q0D2H7_HUMAN)
Q5VX52	Spermatogenesis-associated protein 1 OS=Homo sapiens GN=SPATA1 PE=2 SV=3 - (SPAT1_HUMAN)
Q86UY0	TXNDC5 protein OS=Homo sapiens GN=TXNDC5 PE=2 SV=1 - (Q86UY0_HUMAN)
B4E1U0	cDNA FLJ59148, highly similar to RNA-binding protein 4 OS=Homo sapiens PE=2 SV=1 - (B4E1U0_HUMAN)
Q9UMZ2-6	Isoform 5 of Synergin gamma OS=Homo sapiens GN=SYNRG - (SYNRG_HUMAN)
D6RA82	Annexin OS=Homo sapiens GN=ANXA3 PE=3 SV=1 - (D6RA82_HUMAN)
B4DYC8	cDNA FLJ52361, highly similar to T-complex protein 1 subunit epsilon OS=Homo sapiens PE=2 SV=1 - (B4DYC8_HUMAN)
Q9HD67	Unconventionnal myosin-X OS=Homo sapiens GN=MYO10 PE=1 SV=3 - (MYO10_HUMAN)
Q5SSJ5-3	Isoform 3 of Heterochromatin protein 1-binding protein 3 OS=Homo sapiens GN=HP1BP3 - (HP1B3_HUMAN)
B3KSL2	cDNA FLJ36533 fis, clone TRACH2004428, highly similar to Lactotransferrin (EC 3.4.21.-) (Fragment) OS=Homo sapiens PE=2 SV=1 - (B3KSL2_HUMAN)
B4DTN4	N6-adenosine-methyltransferase 70 kDa subunit OS=Homo sapiens GN=METTL3 PE=2 SV=1 - (B4DTN4_HUMAN)
Q63HR1	Putative uncharacterized protein DKFZp686P17171 OS=Homo sapiens GN=DKFZp686P17171 PE=2 SV=1 - (Q63HR1_HUMAN)
O60667-2	Isoform 2 of Fas apoptotic inhibitory molecule 3 OS=Homo sapiens GN=FAIM3 - (FAIM3_HUMAN)
A6NCL7	Ankyrin repeat domain-containing protein 33B OS=Homo sapiens GN=ANKRD33B PE=4 SV=1 - (AN33B_HUMAN)
F4ZW63	NF45 OS=Homo sapiens PE=2 SV=1 - (F4ZW63_HUMAN)
B3KR97	cDNA FLJ33900 fis, clone CTONG2008262, highly similar to Low density lipoprotein receptor adapter protein 1 OS=Homo sapiens PE=2 SV=1 - (B3KR97_HUMAN)
H7C4J1	Actin-related protein 3C (Fragment) OS=Homo sapiens GN=ACTR3C PE=4 SV=1 - (H7C4J1_HUMAN)
E9PBV3	Suprabasin OS=Homo sapiens GN=SBSN PE=4 SV=1 - (E9PBV3_HUMAN)
Q8N7B9	EF-hand calcium-binding domain-containing protein 3 OS=Homo sapiens GN=EFCAB3 PE=1 SV=1 - (EFCB3_HUMAN)
C9JA38	DALR anticodon-binding domain-containing protein 3 (Fragment) OS=Homo sapiens GN=DALRD3 PE=4 SV=2 - (C9JA38_HUMAN)
P13010	X-ray repair cross-complementing protein 5 OS=Homo sapiens GN=XRCC5 PE=1 SV=3 - (XRCC5_HUMAN)
P21108	Ribose-phosphate pyrophosphokinase 3 OS=Homo sapiens GN=PRPS1L1 PE=1 SV=2 - (PRPS3_HUMAN)
B4DLJ7	cDNA FLJ59334, highly similar to Transmembrane glycoprotein NMB OS=Homo sapiens PE=2 SV=1 - (B4DLJ7_HUMAN)
A8MUQ0	Putative paraneoplastic antigen-like protein 6B-like protein LOC649238 OS=Homo sapiens PE=3 SV=1 - (PN6BL_HUMAN)
Q9H9B7	Coatomer subunit gamma OS=Homo sapiens PE=2 SV=1 - (Q9H9B7_HUMAN)
Q08257	Quinone oxidoreductase OS=Homo sapiens GN=CRYZ PE=1 SV=1 - (QOR_HUMAN)
Q6ZU59	cDNA FLJ43980 fis, clone TESTI4018806 OS=Homo sapiens PE=2 SV=1 - (Q6ZU59_HUMAN)
O14647-2	Isoform 2 of Chromodomain-helicase-DNA-binding protein 2 OS=Homo sapiens GN=CHD2 - (CHD2_HUMAN)
H0YKD8	60S ribosomal protein L28 OS=Homo sapiens GN=RPL28 PE=4 SV=1 - (H0YKD8_HUMAN)
P68402	Platelet-activating factor acetylhydrolase IB subunit beta OS=Homo sapiens GN=PAFAH1B2 PE=1 SV=1 - (PA1B2_HUMAN)
Q52LG2	Keratin-associated protein 13-2 OS=Homo sapiens GN=KRTAP13-2 PE=1 SV=1 - (KR132_HUMAN)
Q9UKS7	Zinc finger protein Helios OS=Homo sapiens GN=IKZF2 PE=1 SV=2 - (IKZF2_HUMAN)
Q13239	Src-like-adapter OS=Homo sapiens GN=SLA PE=1 SV=3 - (SLAP1_HUMAN)
Q59GJ9	Acetyl-CoA carboxylase 2 variant (Fragment) OS=Homo sapiens PE=1 SV=1 - (Q59GJ9_HUMAN)
A6QKW0	SHINC3 OS=Homo sapiens GN=SHINC3 PE=2 SV=1 - (A6QKW0_HUMAN)
E7EWF1	60S ribosomal protein L4 OS=Homo sapiens GN=RPL4 PE=4 SV=1 - (E7EWF1_HUMAN)
B3KPZ8	cDNA FLJ32530 fis, clone SMINT2000185, highly similar to TRANSKETOLASE (EC 2.2.1.1) OS=Homo sapiens PE=2 SV=1 - (B3KPZ8_HUMAN)
B3KTT6	cDNA FLJ38699 fis, clone KIDNE2002168, highly similar to Short chain 3-hydroxyacyl-CoA dehydrogenase, mitochondrial (EC 1.1.1.35) OS=Homo sapiens PE=2 SV=1 - (B3KTT6_HUMAN)
Q7Z3U5	Putative uncharacterized protein DKFZp686L0869 OS=Homo sapiens GN=DKFZp686L0869 PE=2 SV=1 - (Q7Z3U5_HUMAN)
D6RA63	Protein GAPT (Fragment) OS=Homo sapiens GN=GAPT PE=4 SV=1 - (D6RA63_HUMAN)
C9JSL4	Prolyl 3-hydroxylase 2 (Fragment) OS=Homo sapiens GN=LEPREL1 PE=4 SV=1 - (C9JSL4_HUMAN)
Q15276-2	Isoform 2 of Rab GTPase-binding effector protein 1 OS=Homo sapiens GN=RABEP1 - (RABE1_HUMAN)
P30084	Enoyl-CoA hydratase, mitochondrial OS=Homo sapiens GN=ECHS1 PE=1 SV=4 - (ECHM_HUMAN)
H3BPC7	Inositol 1,4,5-trisphosphate receptor-interacting protein-like 2 (Fragment) OS=Homo sapiens GN=ITPRIPL2 PE=4 SV=2 - (H3BPC7_HUMAN)
Q5BKY2	EIF3H protein OS=Homo sapiens GN=EIF3H PE=2 SV=1 - (Q5BKY2_HUMAN)
Q99439-2	Isoform 2 of Calponin-2 OS=Homo sapiens GN=CNN2 - (CNN2_HUMAN)
Q92835-2	Isoform 2 of Phosphatidylinositol 3,4,5-trisphosphate 5-phosphatase 1 OS=Homo sapiens GN=INPP5D - (SHIP1_HUMAN)
A2VDJ1	GOLGA6 protein (Fragment) OS=Homo sapiens GN=GOLGA6 PE=2 SV=1 - (A2VDJ1_HUMAN)
P48047	ATP synthase subunit O, mitochondrial OS=Homo sapiens GN=ATP5O PE=1 SV=1 - (ATPO_HUMAN)
E9PN51	NADH dehydrogenase (ubiquinone) iron-sulfur protein 8, mitochondrial (Fragment) OS=Homo sapiens GN=NDUFS8 PE=4 SV=1 - (E9PN51_HUMAN)
D6RF73	Aryl hydrocarbon receptor repressor OS=Homo sapiens GN=AHRR PE=4 SV=1 - (D6RF73_HUMAN)
Q14692	Ribosome biogenesis protein BMS1 homolog OS=Homo sapiens GN=BMS1 PE=1 SV=1 - (BMS1_HUMAN)
Q9NXR1-2	Isoform 2 of Nuclear distribution protein nudE homolog 1 OS=Homo sapiens GN=NDE1 - (NDE1_HUMAN)
O00571-2	Isoform 2 of ATP-dependent RNA helicase DDX3X OS=Homo sapiens GN=DDX3X - (DDX3X_HUMAN)
I3L518	Cytosolic Fe-S cluster assembly factor NUBP1 (Fragment) OS=Homo sapiens GN=NUBP1 PE=4 SV=1 - (I3L518_HUMAN)
O60303	Uncharacterized protein KIAA0556 OS=Homo sapiens GN=KIAA0556 PE=1 SV=4 - (K0556_HUMAN)
Q9NPP6	Immunoglobulin heavy chain variant (Fragment) OS=Homo sapiens PE=2 SV=1 - (Q9NPP6_HUMAN)
A7BKA4	ATP-binding cassette protein OS=Homo sapiens GN=ABCB5 PE=2 SV=1 - (A7BKA4_HUMAN)
A8K865	cDNA FLJ75789, highly similar to Homo sapiens synaptophysin-like 1 (SYPL1), transcript variant 1, mRNA OS=Homo sapiens PE=2 SV=1 - (A8K865_HUMAN)
Q5HYM2	Putative uncharacterized protein DKFZp686O2462 (Fragment) OS=Homo sapiens GN=DKFZp686O2462 PE=2 SV=1 - (Q5HYM2_HUMAN)
D7PBN3	ESRP1/RAF1 fusion protein OS=Homo sapiens PE=2 SV=1 - (D7PBN3_HUMAN)
P53621	Coatomer subunit alpha OS=Homo sapiens GN=COPA PE=1 SV=2 - (COPA_HUMAN)
P63167	Dynein light chain 1, cytoplasmic OS=Homo sapiens GN=DYNLL1 PE=1 SV=1 - (DYL1_HUMAN)
A8K920	cDNA FLJ76953, highly similar to Homo sapiens plexin B3 (PLXNB3), mRNA OS=Homo sapiens PE=2 SV=1 - (A8K920_HUMAN)
A8KA04	cDNA FLJ77539 OS=Homo sapiens PE=2 SV=1 - (A8KA04_HUMAN)
Q5VY36	Novel protein similar to ribosomal protein L13a RPL13A OS=Homo sapiens GN=RP11-365K22.1-001 PE=4 SV=1 - (Q5VY36_HUMAN)
P07814	Bifunctional glutamate/proline--tRNA ligase OS=Homo sapiens GN=EPRS PE=1 SV=5 - (SYEP_HUMAN)
B7Z1I2	Aspartate aminotransferase OS=Homo sapiens GN=GOT1 PE=2 SV=1 - (B7Z1I2_HUMAN)
A6NKB8	Aminopeptidase B OS=Homo sapiens GN=RNPEP PE=4 SV=1 - (A6NKB8_HUMAN)
E9PRB8	26S protease regulatory subunit 6A OS=Homo sapiens GN=PSMC3 PE=4 SV=1 - (E9PRB8_HUMAN)
P05091	Aldehyde dehydrogenase, mitochondrial OS=Homo sapiens GN=ALDH2 PE=1 SV=2 - (ALDH2_HUMAN)
A0AVK6	Transcription factor E2F8 OS=Homo sapiens GN=E2F8 PE=1 SV=1 - (E2F8_HUMAN)
E7ESV6	Latrophilin-3 OS=Homo sapiens GN=LPHN3 PE=3 SV=1 - (E7ESV6_HUMAN)
Q86XL3	Ankyrin repeat and LEM domain-containing protein 2 OS=Homo sapiens GN=ANKLE2 PE=1 SV=4 - (ANKL2_HUMAN)
B7Z7I4	cDNA FLJ54568, highly similar to T-complex protein 1 subunit eta OS=Homo sapiens PE=2 SV=1 - (B7Z7I4_HUMAN)
H0YDT6	Eukaryotic translation initiation factor 3 subunit F (Fragment) OS=Homo sapiens GN=EIF3F PE=4 SV=1 - (H0YDT6_HUMAN)
Q4ZG57	Putative uncharacterized protein MCM6 (Fragment) OS=Homo sapiens GN=MCM6 PE=2 SV=1 - (Q4ZG57_HUMAN)
B4DQZ2	cDNA FLJ50621, highly similar to DNA (cytosine-5)-methyltransferase-like protein 2 OS=Homo sapiens PE=2 SV=1 - (B4DQZ2_HUMAN)
O00562-2	Isoform 2 of Membrane-associated phosphatidylinositol transfer protein 1 OS=Homo sapiens GN=PITPNM1 - (PITM1_HUMAN)
B7Z5N7	Sec1 family domain-containing protein 1 OS=Homo sapiens GN=SCFD1 PE=2 SV=1 - (B7Z5N7_HUMAN)
Q6ISF6	KRTAP9-2 protein (Fragment) OS=Homo sapiens GN=KRTAP9-2 PE=2 SV=1 - (Q6ISF6_HUMAN)
P49458	Signal recognition particle 9 kDa protein OS=Homo sapiens GN=SRP9 PE=1 SV=2 - (SRP09_HUMAN)
B4E054	cDNA FLJ58444, highly similar to Vacuolar ATP synthase subunit H (EC 3.6.3.14) OS=Homo sapiens PE=2 SV=1 - (B4E054_HUMAN)
A8K321	cDNA FLJ78524, highly similar to Homo sapiens SMILE protein (SMILE), mRNA OS=Homo sapiens PE=2 SV=1 - (A8K321_HUMAN)
Q6ZWJ8	Kielin/chordin-like protein OS=Homo sapiens GN=KCP PE=2 SV=2 - (KCP_HUMAN)
B3KVK3	cDNA FLJ16666 fis, clone THYMU2032732, highly similar to Forkhead box protein D4-like 1 OS=Homo sapiens PE=2 SV=1 - (B3KVK3_HUMAN)
Q9NYV4	Cyclin-dependent kinase 12 OS=Homo sapiens GN=CDK12 PE=1 SV=2 - (CDK12_HUMAN)
B1AK88	Capping protein (Actin filament) muscle Z-line, beta OS=Homo sapiens GN=CAPZB PE=4 SV=1 - (B1AK88_HUMAN)
C5H6M8	Cytochrome c oxidase subunit 2 OS=Homo sapiens GN=COII PE=3 SV=1 - (C5H6M8_HUMAN)
Q9NS56-2	Isoform 2 of E3 ubiquitin-protein ligase Topors OS=Homo sapiens GN=TOPORS - (TOPRS_HUMAN)
Q8IZH2-2	Isoform 2 of 5'-3' exoribonuclease 1 OS=Homo sapiens GN=XRN1 - (XRN1_HUMAN)
B5B2P0	Nuclear factor of activated T-cells c2 isoform IA-IIS-Xa OS=Homo sapiens GN=NFATC2 PE=2 SV=1 - (B5B2P0_HUMAN)
B2R7C2	cDNA, FLJ93375, highly similar to Homo sapiens ZW10, kinetochore associated, homolog (Drosophila) (ZW10), mRNA OS=Homo sapiens PE=2 SV=1 - (B2R7C2_HUMAN)
P28066	Proteasome subunit alpha type-5 OS=Homo sapiens GN=PSMA5 PE=1 SV=3 - (PSA5_HUMAN)
Q7Z3C0	Putative uncharacterized protein DKFZp686A04192 (Fragment) OS=Homo sapiens GN=DKFZp686A04192 PE=2 SV=1 - (Q7Z3C0_HUMAN)
A6NDW3	Lipolysis-stimulated lipoprotein receptor OS=Homo sapiens GN=LSR PE=4 SV=1 - (A6NDW3_HUMAN)
I3L0T4	Peroxisomal acyl-coenzyme A oxidase 1 OS=Homo sapiens GN=ACOX1 PE=4 SV=1 - (I3L0T4_HUMAN)
B4DGP8	cDNA FLJ55574, highly similar to Calnexin OS=Homo sapiens PE=2 SV=1 - (B4DGP8_HUMAN)
E9PE28	X-linked retinitis pigmentosa GTPase regulator OS=Homo sapiens GN=RPGR PE=4 SV=1 - (E9PE28_HUMAN)
B2R8E1	Ran-binding protein 9 OS=Homo sapiens GN=RANBP9 PE=2 SV=1 - (B2R8E1_HUMAN)
E5KNB2	DNA-apurinic or apyrimidinic site lyase 2 OS=Homo sapiens PE=4 SV=1 - (E5KNB2_HUMAN)
E9PPP7	Putative uncharacterized protein C8orf73 (Fragment) OS=Homo sapiens GN=C8orf73 PE=4 SV=1 - (E9PPP7_HUMAN)
A6NCD2	T-complex protein 1 subunit zeta OS=Homo sapiens GN=CCT6A PE=3 SV=1 - (A6NCD2_HUMAN)
Q9BYR7	Keratin-associated protein 3-2 OS=Homo sapiens GN=KRTAP3-2 PE=1 SV=1 - (KRA32_HUMAN)
B4DND6	cDNA FLJ55804, highly similar to COP9 signalosome complex subunit 1 OS=Homo sapiens PE=2 SV=1 - (B4DND6_HUMAN)
Q5T0N5-2	Isoform 2 of Formin-binding protein 1-like OS=Homo sapiens GN=FNBP1L - (FBP1L_HUMAN)
A4D160	Serine/threonine kinase 31 OS=Homo sapiens GN=STK31 PE=4 SV=1 - (A4D160_HUMAN)
P30086	Phosphatidylethanolamine-binding protein 1 OS=Homo sapiens GN=PEBP1 PE=1 SV=3 - (PEBP1_HUMAN)
B2RBR9	cDNA, FLJ95650, highly similar to Homo sapiens karyopherin (importin) beta 1 (KPNB1), mRNA OS=Homo sapiens PE=2 SV=1 - (B2RBR9_HUMAN)
Q53T70	Putative uncharacterized protein RAB10 (Fragment) OS=Homo sapiens GN=RAB10 PE=2 SV=1 - (Q53T70_HUMAN)
Q15102	Platelet-activating factor acetylhydrolase IB subunit gamma OS=Homo sapiens GN=PAFAH1B3 PE=1 SV=1 - (PA1B3_HUMAN)
Q5T5C0-3	Isoform 3 of Syntaxin-binding protein 5 OS=Homo sapiens GN=STXBP5 - (STXB5_HUMAN)
H3BNA1	Ubiquitin carboxyl-terminal hydrolase 10 (Fragment) OS=Homo sapiens GN=USP10 PE=4 SV=1 - (H3BNA1_HUMAN)
Q9BYR6	Keratin-associated protein 3-3 OS=Homo sapiens GN=KRTAP3-3 PE=1 SV=1 - (KRA33_HUMAN)
Q96HX3	Similar to ribophorin I (Fragment) OS=Homo sapiens PE=2 SV=1 - (Q96HX3_HUMAN)
B0VJZ1	Leukocyte receptor cluster (LRC) member 9 OS=Homo sapiens GN=LENG9 PE=4 SV=1 - (B0VJZ1_HUMAN)
Q9UNX4	WD repeat-containing protein 3 OS=Homo sapiens GN=WDR3 PE=1 SV=1 - (WDR3_HUMAN)
C9J1Z8	ADP-ribosylation factor 5 (Fragment) OS=Homo sapiens GN=ARF5 PE=3 SV=1 - (C9J1Z8_HUMAN)
B4DII5	Importin subunit alpha OS=Homo sapiens PE=2 SV=1 - (B4DII5_HUMAN)
E5KSX8	Mitochondrial transcription factor A OS=Homo sapiens PE=4 SV=1 - (E5KSX8_HUMAN)
Q9NVM6	DnaJ homolog subfamily C member 17 OS=Homo sapiens GN=DNAJC17 PE=1 SV=1 - (DJC17_HUMAN)
P40939	Trifunctional enzyme subunit alpha, mitochondrial OS=Homo sapiens GN=HADHA PE=1 SV=2 - (ECHA_HUMAN)
Q9HDC9	Adipocyte plasma membrane-associated protein OS=Homo sapiens GN=APMAP PE=1 SV=2 - (APMAP_HUMAN)
P62273	40S ribosomal protein S29 OS=Homo sapiens GN=RPS29 PE=1 SV=2 - (RS29_HUMAN)
B7Z6B8	2,4-dienoyl-CoA reductase, mitochondrial OS=Homo sapiens GN=DECR1 PE=2 SV=1 - (B7Z6B8_HUMAN)
F5H5L3	Ankyrin repeat domain-containing protein 36A OS=Homo sapiens GN=ANKRD36 PE=4 SV=1 - (F5H5L3_HUMAN)
B3KMX0	cDNA FLJ12837 fis, clone NT2RP2003228, highly similar to DNA replication licensing factor MCM4 OS=Homo sapiens PE=2 SV=1 - (B3KMX0_HUMAN)
Q08AD1-2	Isoform 2 of Calmodulin-regulated spectrin-associated protein 2 OS=Homo sapiens GN=CAMSAP2 - (CAMP2_HUMAN)
P06703	Protein S100-A6 OS=Homo sapiens GN=S100A6 PE=1 SV=1 - (S10A6_HUMAN)
Q9ULW8	Protein-arginine deiminase type-3 OS=Homo sapiens GN=PADI3 PE=1 SV=2 - (PADI3_HUMAN)
P14174	Macrophage migration inhibitory factor OS=Homo sapiens GN=MIF PE=1 SV=4 - (MIF_HUMAN)
P42766	60S ribosomal protein L35 OS=Homo sapiens GN=RPL35 PE=1 SV=2 - (RL35_HUMAN)
Q9P1F3	Costars family protein ABRACL OS=Homo sapiens GN=ABRACL PE=1 SV=1 - (ABRAL_HUMAN)
Q9NTN9	Semaphorin-4G OS=Homo sapiens GN=SEMA4G PE=2 SV=1 - (SEM4G_HUMAN)
Q8IZI4	B-lymphocyte stimulator (Fragment) OS=Homo sapiens GN=TNFSF13B PE=2 SV=1 - (Q8IZI4_HUMAN)
E5RI90	Uncharacterized protein C1orf198 (Fragment) OS=Homo sapiens GN=C1orf198 PE=4 SV=1 - (E5RI90_HUMAN)
Q9UBI6	Guanine nucleotide-binding protein G(I)/G(S)/G(O) subunit gamma-12 OS=Homo sapiens GN=GNG12 PE=1 SV=3 - (GBG12_HUMAN)
Q16832	Discoidin domain-containing receptor 2 OS=Homo sapiens GN=DDR2 PE=1 SV=2 - (DDR2_HUMAN)
C9J4N6	Isocitrate dehydrogenase (NADP) cytoplasmic (Fragment) OS=Homo sapiens GN=IDH1 PE=3 SV=1 - (C9J4N6_HUMAN)
B7Z589	Transporter OS=Homo sapiens GN=SLC6A9 PE=2 SV=1 - (B7Z589_HUMAN)
Q495G0	V-myb myeloblastosis viral oncogene homolog (Avian)-like 1 OS=Homo sapiens GN=MYBL1 PE=2 SV=1 - (Q495G0_HUMAN)
B2RCY5	cDNA, FLJ96373, highly similar to Homo sapiens X-ray repair complementing defective repair in Chinese hamster cells 1 (XRCC1), mRNA OS=Homo sapiens PE=2 SV=1 - (B2RCY5_HUMAN)
O95976	Immunoglobulin superfamily member 6 OS=Homo sapiens GN=IGSF6 PE=2 SV=2 - (IGSF6_HUMAN)
P48729	Casein kinase I isoform alpha OS=Homo sapiens GN=CSNK1A1 PE=1 SV=2 - (KC1A_HUMAN)
B7Z254	Protein disulfide-isomerase A6 OS=Homo sapiens GN=PDIA6 PE=2 SV=1 - (B7Z254_HUMAN)
Q86VP6-2	Isoform 2 of Cullin-associated NEDD8-dissociated protein 1 OS=Homo sapiens GN=CAND1 - (CAND1_HUMAN)
Q59H46	Integrin beta (Fragment) OS=Homo sapiens PE=2 SV=1 - (Q59H46_HUMAN)
H7BZW3	Voltage-dependent calcium channel subunit alpha-2-3 OS=Homo sapiens GN=CACNA2D3 PE=4 SV=1 - (H7BZW3_HUMAN)
O60716-24	Isoform 3 of Catenin delta-1 OS=Homo sapiens GN=CTNND1 - (CTND1_HUMAN)
O75820-3	Isoform 3 of Zinc finger protein 189 OS=Homo sapiens GN=ZNF189 - (ZN189_HUMAN)
Q13283	Ras GTPase-activating protein-binding protein 1 OS=Homo sapiens GN=G3BP1 PE=1 SV=1 - (G3BP1_HUMAN)
B3KP61	RAC-beta serine/threonine-protein kinase OS=Homo sapiens GN=AKT2 PE=2 SV=1 - (B3KP61_HUMAN)
Q32XH3	Ribosomal protein L18a-like protein OS=Homo sapiens PE=2 SV=1 - (Q32XH3_HUMAN)
B4DFL2	Isocitrate dehydrogenase (NADP) OS=Homo sapiens GN=IDH2 PE=2 SV=1 - (B4DFL2_HUMAN)
E9PAV3	Nascent polypeptide-associated complex subunit alpha OS=Homo sapiens GN=NACA PE=4 SV=1 - (E9PAV3_HUMAN)
Q8WVV4	Protein POF1B OS=Homo sapiens GN=POF1B PE=1 SV=3 - (POF1B_HUMAN)
P42677	40S ribosomal protein S27 OS=Homo sapiens GN=RPS27 PE=1 SV=3 - (RS27_HUMAN)
F5GYN4	Ubiquitin thioesterase OTUB1 OS=Homo sapiens GN=OTUB1 PE=4 SV=1 - (F5GYN4_HUMAN)
D3YTB1	60S ribosomal protein L32 (Fragment) OS=Homo sapiens GN=RPL32 PE=4 SV=1 - (D3YTB1_HUMAN)
P49411	Elongation factor Tu, mitochondrial OS=Homo sapiens GN=TUFM PE=1 SV=2 - (EFTU_HUMAN)
A8K646	cDNA FLJ75699, highly similar to Homo sapiens osteoclast stimulating factor 1 (OSTF1), mRNA OS=Homo sapiens PE=2 SV=1 - (A8K646_HUMAN)
Q8IV16	Glycosylphosphatidylinositol-anchored high density lipoprotein-binding protein 1 OS=Homo sapiens GN=GPIHBP1 PE=1 SV=2 - (HDBP1_HUMAN)
B7Z6Q1	cDNA FLJ51102, highly similar to DEP domain-containing protein 5 OS=Homo sapiens PE=2 SV=1 - (B7Z6Q1_HUMAN)
B7ZKX2	Putative uncharacterized protein OS=Homo sapiens PE=2 SV=1 - (B7ZKX2_HUMAN)
Q15406-3	Isoform 3 of Nuclear receptor subfamily 6 group A member 1 OS=Homo sapiens GN=NR6A1 - (NR6A1_HUMAN)
P22079	Lactoperoxidase OS=Homo sapiens GN=LPO PE=1 SV=2 - (PERL_HUMAN)
O95147	Dual specificity protein phosphatase 14 OS=Homo sapiens GN=DUSP14 PE=1 SV=1 - (DUS14_HUMAN)
F8W6V3	Protein TSPEAR OS=Homo sapiens GN=TSPEAR PE=4 SV=1 - (F8W6V3_HUMAN)
B2R9T9	cDNA, FLJ94551 OS=Homo sapiens PE=2 SV=1 - (B2R9T9_HUMAN)
Q2NKY6	Putative uncharacterized protein OS=Homo sapiens PE=2 SV=1 - (Q2NKY6_HUMAN)
Q9BX68	Histidine triad nucleotide-binding protein 2, mitochondrial OS=Homo sapiens GN=HINT2 PE=1 SV=1 - (HINT2_HUMAN)
Q5SQ08	Complement component 8, gamma polypeptide (Fragment) OS=Homo sapiens GN=C8G PE=2 SV=1 - (Q5SQ08_HUMAN)
E9PN17	ATP synthase subunit g, mitochondrial OS=Homo sapiens GN=ATP5L PE=4 SV=1 - (E9PN17_HUMAN)
A8K8S6	cDNA FLJ77231, highly similar to Homo sapiens hemogen (HEMGN), transcript variant 1, mRNA OS=Homo sapiens PE=2 SV=1 - (A8K8S6_HUMAN)
P62314	Small nuclear ribonucleoprotein Sm D1 OS=Homo sapiens GN=SNRPD1 PE=1 SV=1 - (SMD1_HUMAN)
P60985-2	Isoform 2 of Keratinocyte differentiation-associated protein OS=Homo sapiens GN=KRTDAP - (KTDAP_HUMAN)
Q86YF9-2	Isoform 2 of Zinc finger protein DZIP1 OS=Homo sapiens GN=DZIP1 - (DZIP1_HUMAN)
P13489	Ribonuclease inhibitor OS=Homo sapiens GN=RNH1 PE=1 SV=2 - (RINI_HUMAN)
P60369	Keratin-associated protein 10-3 OS=Homo sapiens GN=KRTAP10-3 PE=1 SV=2 - (KR103_HUMAN)
P27635	60S ribosomal protein L10 OS=Homo sapiens GN=RPL10 PE=1 SV=4 - (RL10_HUMAN)
Q0VDC6	Peptidyl-prolyl cis-trans isomerase OS=Homo sapiens GN=FKBP1A PE=2 SV=1 - (Q0VDC6_HUMAN)
H3BLU7	Aflatoxin B1 aldehyde reductase member 2 (Fragment) OS=Homo sapiens GN=AKR7A2 PE=4 SV=1 - (H3BLU7_HUMAN)
A8K5N5	cDNA FLJ78142, highly similar to Homo sapiens transglutaminase 1 (K polypeptide epidermal type I, protein-glutamine-gamma-glutamyltransferase), mRNA OS=Homo sapiens PE=2 SV=1 - (A8K5N5_HUMAN)
E2QRG3	Kinesin-like protein KIF6 OS=Homo sapiens GN=KIF6 PE=3 SV=2 - (E2QRG3_HUMAN)
Q92688-2	Isoform 2 of Acidic leucine-rich nuclear phosphoprotein 32 family member B OS=Homo sapiens GN=ANP32B - (AN32B_HUMAN)
F8VR50	Actin-related protein 2/3 complex subunit 3 (Fragment) OS=Homo sapiens GN=ARPC3 PE=4 SV=1 - (F8VR50_HUMAN)
Q1JQ76	Ribosomal protein (Fragment) OS=Homo sapiens GN=RPL10A PE=2 SV=1 - (Q1JQ76_HUMAN)
Q5T949	Protein phosphatase 2A activator, regulatory subunit 4 (Fragment) OS=Homo sapiens GN=PPP2R4 PE=2 SV=1 - (Q5T949_HUMAN)
G5E9R3	60S ribosomal protein L37a OS=Homo sapiens GN=RPL37A PE=4 SV=1 - (G5E9R3_HUMAN)
Q13946-2	Isoform PDE7A2 of High affinity cAMP-specific 3',5'-cyclic phosphodiesterase 7A OS=Homo sapiens GN=PDE7A - (PDE7A_HUMAN)
P49748-2	Isoform 2 of Very long-chain specific acyl-CoA dehydrogenase, mitochondrial OS=Homo sapiens GN=ACADVL - (ACADV_HUMAN)
Q9UIV8-2	Isoform 2 of Serpin B13 OS=Homo sapiens GN=SERPINB13 - (SPB13_HUMAN)
P18827	Syndecan-1 OS=Homo sapiens GN=SDC1 PE=1 SV=3 - (SDC1_HUMAN)
Q5H8C1	FRAS1-related extracellular matrix protein 1 OS=Homo sapiens GN=FREM1 PE=1 SV=3 - (FREM1_HUMAN)
H7BYH4	Superoxide dismutase (Cu-Zn) OS=Homo sapiens GN=SOD1 PE=3 SV=1 - (H7BYH4_HUMAN)
D6RBW1	Eukaryotic translation initiation factor 4E OS=Homo sapiens GN=EIF4E PE=3 SV=1 - (D6RBW1_HUMAN)
Q14651	Plastin-1 OS=Homo sapiens GN=PLS1 PE=1 SV=2 - (PLSI_HUMAN)
B4DU58	Macrophage-capping protein OS=Homo sapiens GN=CAPG PE=2 SV=1 - (B4DU58_HUMAN)
Q3LI83	Keratin-associated protein 24-1 OS=Homo sapiens GN=KRTAP24-1 PE=1 SV=1 - (KR241_HUMAN)
C5IX07	Tumor-associated calcium signal transducer 2 OS=Homo sapiens GN=TACSTD2 PE=4 SV=1 - (C5IX07_HUMAN)
Q59FM5	Guanine nucleotide-binding protein G, alpha subunit variant (Fragment) OS=Homo sapiens PE=2 SV=1 - (Q59FM5_HUMAN)
Q06323	Proteasome activator complex subunit 1 OS=Homo sapiens GN=PSME1 PE=1 SV=1 - (PSME1_HUMAN)
Q6UWL6-3	Isoform 3 of Kin of IRRE-like protein 2 OS=Homo sapiens GN=KIRREL2 - (KIRR2_HUMAN)
B4DIX5	Transient receptor potential cation channel subfamily M member 4 OS=Homo sapiens GN=TRPM4 PE=2 SV=1 - (B4DIX5_HUMAN)
B4DXN1	cDNA FLJ53236, highly similar to Homo sapiens IQ motif containing E (IQCE), mRNA OS=Homo sapiens PE=2 SV=1 - (B4DXN1_HUMAN)
P30046	D-dopachrome decarboxylase OS=Homo sapiens GN=DDT PE=1 SV=3 - (DOPD_HUMAN)
E9PPC4	E3 ubiquitin-protein ligase RAG1 OS=Homo sapiens GN=RAG1 PE=4 SV=1 - (E9PPC4_HUMAN)
A4D2P2	Ras-related C3 botulinum toxin substrate 1 (Rho family, small GTP binding protein Rac1) OS=Homo sapiens GN=RAC1 PE=3 SV=1 - (A4D2P2_HUMAN)
Q6NUJ1	Proactivator polypeptide-like 1 OS=Homo sapiens GN=PSAPL1 PE=2 SV=2 - (SAPL1_HUMAN)
P47870	Gamma-aminobutyric acid receptor subunit beta-2 OS=Homo sapiens GN=GABRB2 PE=1 SV=2 - (GBRB2_HUMAN)
O75582-2	Isoform 2 of Ribosomal protein S6 kinase alpha-5 OS=Homo sapiens GN=RPS6KA5 - (KS6A5_HUMAN)
H0YKT8	Proteasome subunit beta type (Fragment) OS=Homo sapiens GN=PSMA4 PE=3 SV=1 - (H0YKT8_HUMAN)
A8K8F9	cDNA FLJ75064, highly similar to Homo sapiens phospholipase C, delta 1 (PLCD1), mRNA OS=Homo sapiens PE=2 SV=1 - (A8K8F9_HUMAN)
F8WC88	Cyclin-dependent kinase-like 4 OS=Homo sapiens GN=CDKL4 PE=4 SV=1 - (F8WC88_HUMAN)
O75828	Carbonyl reductase (NADPH) 3 OS=Homo sapiens GN=CBR3 PE=1 SV=3 - (CBR3_HUMAN)
B0V2L2	HLA-B associated transcript 1 (Fragment) OS=Homo sapiens GN=BAT1 PE=4 SV=1 - (B0V2L2_HUMAN)
P49961-2	Isoform Placental I of Ectonucleoside triphosphate diphosphohydrolase 1 OS=Homo sapiens GN=ENTPD1 - (ENTP1_HUMAN)
D6RBK0	Prohibitin (Fragment) OS=Homo sapiens GN=PHB PE=4 SV=1 - (D6RBK0_HUMAN)
A8K6Q5	cDNA FLJ77327, highly similar to Homo sapiens cyclin H (CCNH), mRNA OS=Homo sapiens PE=2 SV=1 - (A8K6Q5_HUMAN)
Q9BYD5	Cornifelin OS=Homo sapiens GN=CNFN PE=1 SV=2 - (CNFN_HUMAN)
H0Y9D8	Inorganic pyrophosphatase 2, mitochondrial (Fragment) OS=Homo sapiens GN=PPA2 PE=4 SV=1 - (H0Y9D8_HUMAN)
Q6P6C2-2	Isoform 2 of Probable alpha-ketoglutarate-dependent dioxygenase ABH5 OS=Homo sapiens GN=ALKBH5 - (ALKB5_HUMAN)
B2RDB6	cDNA, FLJ96540, highly similar to Homo sapiens zinc finger protein 3 (A8-51) (ZNF3), transcript variant 2, mRNA OS=Homo sapiens PE=2 SV=1 - (B2RDB6_HUMAN)
P22314	Ubiquitin-like modifier-activating enzyme 1 OS=Homo sapiens GN=UBA1 PE=1 SV=3 - (UBA1_HUMAN)
Q96AU7	Thymic stromal lymphopoietin OS=Homo sapiens GN=TSLP PE=2 SV=1 - (Q96AU7_HUMAN)
D6RGH1	Uncharacterized protein OS=Homo sapiens PE=4 SV=1 - (D6RGH1_HUMAN)
I3L4A2	Leucine carboxyl methyltransferase 1 (Fragment) OS=Homo sapiens GN=LCMT1 PE=4 SV=1 - (I3L4A2_HUMAN)
Q53TD0	Putative uncharacterized protein SP100 (Fragment) OS=Homo sapiens GN=SP100 PE=2 SV=1 - (Q53TD0_HUMAN)
B2RAZ4	cDNA, FLJ95208, highly similar to Homo sapiens PYD and CARD domain containing (PYCARD), transcript variant 1, mRNA OS=Homo sapiens PE=2 SV=1 - (B2RAZ4_HUMAN)
H0YN94	Protein FAM81A (Fragment) OS=Homo sapiens GN=FAM81A PE=4 SV=1 - (H0YN94_HUMAN)
P84103	Serine/arginine-rich splicing factor 3 OS=Homo sapiens GN=SRSF3 PE=1 SV=1 - (SRSF3_HUMAN)
Q5T946	Glyoxylate reductase/hydroxypyruvate reductase OS=Homo sapiens GN=GRHPR PE=2 SV=1 - (Q5T946_HUMAN)
B7WNW7	HEAT repeat-containing protein 3 OS=Homo sapiens GN=HEATR3 PE=4 SV=1 - (B7WNW7_HUMAN)
C9JGI3	Thymidine phosphorylase (Fragment) OS=Homo sapiens GN=TYMP PE=4 SV=1 - (C9JGI3_HUMAN)
Q53F37	SAR1a gene homolog 2 variant (Fragment) OS=Homo sapiens PE=2 SV=1 - (Q53F37_HUMAN)
F8VVM2	Phosphate carrier protein, mitochondrial OS=Homo sapiens GN=SLC25A3 PE=3 SV=1 - (F8VVM2_HUMAN)
F5H022	Histone H1.0 OS=Homo sapiens GN=H1F0 PE=3 SV=1 - (F5H022_HUMAN)
P62308	Small nuclear ribonucleoprotein G OS=Homo sapiens GN=SNRPG PE=1 SV=1 - (RUXG_HUMAN)
Q6TGM5	Mutant NADH-cytochrome b5 reductase (Fragment) OS=Homo sapiens PE=2 SV=1 - (Q6TGM5_HUMAN)
O60493	Sorting nexin-3 OS=Homo sapiens GN=SNX3 PE=1 SV=3 - (SNX3_HUMAN)
H7C0M9	Uncharacterized protein (Fragment) OS=Homo sapiens PE=4 SV=1 - (H7C0M9_HUMAN)
Q6WWJ7	DELTA3PRO-IL-18 OS=Homo sapiens GN=IL18 PE=2 SV=1 - (Q6WWJ7_HUMAN)
Q96MT8	Centrosomal protein of 63 kDa OS=Homo sapiens GN=CEP63 PE=1 SV=1 - (CEP63_HUMAN)
H0YBX3	Ribonuclease UK114 (Fragment) OS=Homo sapiens GN=HRSP12 PE=4 SV=1 - (H0YBX3_HUMAN)
Q5J7W2	Migration-inducing gene 9 protein OS=Homo sapiens PE=2 SV=1 - (Q5J7W2_HUMAN)
Q9Y3U8	60S ribosomal protein L36 OS=Homo sapiens GN=RPL36 PE=1 SV=3 - (RL36_HUMAN)
Q08554-2	Isoform 1B of Desmocollin-1 OS=Homo sapiens GN=DSC1 - (DSC1_HUMAN)
H3BPU8	Deoxyribonuclease-1-like 2 (Fragment) OS=Homo sapiens GN=DNASE1L2 PE=4 SV=1 - (H3BPU8_HUMAN)
B2R4V2	cDNA, FLJ92227, highly similar to Homo sapiens ribosomal protein L36a-like (RPL36AL), mRNA OS=Homo sapiens PE=3 SV=1 - (B2R4V2_HUMAN)
O94933	SLIT and NTRK-like protein 3 OS=Homo sapiens GN=SLITRK3 PE=2 SV=2 - (SLIK3_HUMAN)
P05387	60S acidic ribosomal protein P2 OS=Homo sapiens GN=RPLP2 PE=1 SV=1 - (RLA2_HUMAN)
A8K2Q6	Peptidyl-prolyl cis-trans isomerase OS=Homo sapiens PE=2 SV=1 - (A8K2Q6_HUMAN)
F8W950	Aminoacyl tRNA synthase complex-interacting multifunctional protein 2 OS=Homo sapiens GN=AIMP2 PE=4 SV=1 - (F8W950_HUMAN)
A6NM69	Non-specific lipid-transfer protein OS=Homo sapiens GN=SCP2 PE=4 SV=2 - (A6NM69_HUMAN)
B5MCP9	40S ribosomal protein S7 OS=Homo sapiens GN=RPS7 PE=4 SV=1 - (B5MCP9_HUMAN)
B7Z6B3	cDNA FLJ53094, highly similar to Receptor expression-enhancing protein 5 OS=Homo sapiens PE=2 SV=1 - (B7Z6B3_HUMAN)
E9PQH6	Rho-related GTP-binding protein RhoC (Fragment) OS=Homo sapiens GN=RHOC PE=3 SV=1 - (E9PQH6_HUMAN)
E4W6B6	RPL27/NME2 fusion protein (Fragment) OS=Homo sapiens GN=RPL27 PE=2 SV=1 - (E4W6B6_HUMAN)
Q99497	Protein DJ-1 OS=Homo sapiens GN=PARK7 PE=1 SV=2 - (PARK7_HUMAN)
C9JFR7	Cytochrome c (Fragment) OS=Homo sapiens GN=CYCS PE=3 SV=1 - (C9JFR7_HUMAN)
P0C7V6	Putative protein FAM48B2 OS=Homo sapiens GN=FAM48B2 PE=5 SV=1 - (F48B2_HUMAN)
Q8N1F8-2	Isoform 2 of Serine/threonine-protein kinase 11-interacting protein OS=Homo sapiens GN=STK11IP - (S11IP_HUMAN)
P28070	Proteasome subunit beta type-4 OS=Homo sapiens GN=PSMB4 PE=1 SV=4 - (PSB4_HUMAN)
Q9Y3D6	Mitochondrial fission 1 protein OS=Homo sapiens GN=FIS1 PE=1 SV=2 - (FIS1_HUMAN)
P61254	60S ribosomal protein L26 OS=Homo sapiens GN=RPL26 PE=1 SV=1 - (RL26_HUMAN)
P28072	Proteasome subunit beta type-6 OS=Homo sapiens GN=PSMB6 PE=1 SV=4 - (PSB6_HUMAN)
A6H8Y5	Nibrin OS=Homo sapiens GN=NBN PE=2 SV=1 - (A6H8Y5_HUMAN)
E9PCY7	Heterogeneous nuclear ribonucleoprotein H, N-terminally processed OS=Homo sapiens GN=HNRNPH1 PE=4 SV=1 - (E9PCY7_HUMAN)
E7EUY0	DNA-dependent protein kinase catalytic subunit OS=Homo sapiens GN=PRKDC PE=4 SV=1 - (E7EUY0_HUMAN)
Q9BXG0	P231 OS=Homo sapiens PE=2 SV=1 - (Q9BXG0_HUMAN)
H0YFA4	Cysteine-rich protein 2 (Fragment) OS=Homo sapiens GN=CRIP2 PE=4 SV=1 - (H0YFA4_HUMAN)
Q0VD83-3	Isoform 3 of Apolipoprotein B receptor OS=Homo sapiens GN=APOBR - (APOBR_HUMAN)
B7ZW58	C13orf38 protein OS=Homo sapiens GN=C13orf38 PE=4 SV=1 - (B7ZW58_HUMAN)
Q04941-2	Isoform 2 of Proteolipid protein 2 OS=Homo sapiens GN=PLP2 - (PLP2_HUMAN)
F8W7F1	p53 apoptosis effector-related to PMP-22 OS=Homo sapiens GN=PERP PE=4 SV=1 - (F8W7F1_HUMAN)
Q9Y3R5-2	Isoform 2 of Protein dopey-2 OS=Homo sapiens GN=DOPEY2 - (DOP2_HUMAN)
Q6KC79-2	Isoform 2 of Nipped-B-like protein OS=Homo sapiens GN=NIPBL - (NIPBL_HUMAN)
Q9H116	GDNF-inducible zinc finger protein 1 OS=Homo sapiens GN=GZF1 PE=1 SV=1 - (GZF1_HUMAN)
Q69YN4-3	Isoform 3 of Protein virilizer homolog OS=Homo sapiens GN=KIAA1429 - (VIR_HUMAN)
B1ALC0	Actin-related protein 2/3 complex subunit 5 OS=Homo sapiens GN=ARPC5 PE=3 SV=1 - (B1ALC0_HUMAN)
P84090	Enhancer of rudimentary homolog OS=Homo sapiens GN=ERH PE=1 SV=1 - (ERH_HUMAN)
B3KY56	Extended synaptotagmin-1 OS=Homo sapiens GN=ESYT1 PE=2 SV=1 - (B3KY56_HUMAN)
G3V5R8	Alpha-1-antitrypsin (Fragment) OS=Homo sapiens GN=SERPINA1 PE=4 SV=1 - (G3V5R8_HUMAN)
A2RRP1	Neuroblastoma-amplified sequence OS=Homo sapiens GN=NBAS PE=1 SV=2 - (NBAS_HUMAN)
A8MUX0	Putative keratin-associated protein 10-like ENSP00000375147 OS=Homo sapiens PE=3 SV=1 - (KR10D_HUMAN)
F8WEV1	Protein C3orf15 OS=Homo sapiens GN=C3orf15 PE=4 SV=1 - (F8WEV1_HUMAN)
B4E2Y4	cDNA FLJ52462, moderately similar to Mus musculus proteasome (prosome, macropain) 26S subunit, non-ATPase, 8 (Psmd8), mRNA OS=Homo sapiens PE=2 SV=1 - (B4E2Y4_HUMAN)
E9PF49	NADH dehydrogenase (ubiquinone) 1 beta subcomplex subunit 9 OS=Homo sapiens GN=NDUFB9 PE=4 SV=1 - (E9PF49_HUMAN)
B4DV06	cDNA FLJ55904, highly similar to Homo sapiens leucine rich repeat and coiled-coil domain containing 1 (LRRCC1), mRNA (Fragment) OS=Homo sapiens PE=2 SV=1 - (B4DV06_HUMAN)
Q9UBS4	DnaJ homolog subfamily B member 11 OS=Homo sapiens GN=DNAJB11 PE=1 SV=1 - (DJB11_HUMAN)
Q59H49	Polypyrimidine tract-binding protein 1 isoform c variant (Fragment) OS=Homo sapiens PE=2 SV=1 - (Q59H49_HUMAN)
Q5JU30	SH2 domain containing 3C (Fragment) OS=Homo sapiens GN=SH2D3C PE=2 SV=1 - (Q5JU30_HUMAN)
Q16186	Proteasomal ubiquitin receptor ADRM1 OS=Homo sapiens GN=ADRM1 PE=1 SV=2 - (ADRM1_HUMAN)
P22528	Cornifin-B OS=Homo sapiens GN=SPRR1B PE=1 SV=2 - (SPR1B_HUMAN)
P52565	Rho GDP-dissociation inhibitor 1 OS=Homo sapiens GN=ARHGDIA PE=1 SV=3 - (GDIR1_HUMAN)
Q6ZUB1	Protein FAM75E1 OS=Homo sapiens GN=FAM75E1 PE=2 SV=2 - (F75E1_HUMAN)
O60216	Double-strand-break repair protein rad21 homolog OS=Homo sapiens GN=RAD21 PE=1 SV=2 - (RAD21_HUMAN)
O94907	Dickkopf-related protein 1 OS=Homo sapiens GN=DKK1 PE=1 SV=1 - (DKK1_HUMAN)
O60336-3	Isoform 3 of Mitogen-activated protein kinase-binding protein 1 OS=Homo sapiens GN=MAPKBP1 - (MABP1_HUMAN)
A6NKE1	Trafficking protein particle complex subunit 3 OS=Homo sapiens GN=TRAPPC3 PE=4 SV=1 - (A6NKE1_HUMAN)
Q96MR7	Putative uncharacterized protein C1orf145 OS=Homo sapiens GN=C1orf145 PE=5 SV=1 - (CA145_HUMAN)
A8K4I8	cDNA FLJ78131, highly similar to Homo sapiens nipsnap homolog 1 (C. elegans) (NIPSNAP1), mRNA OS=Homo sapiens PE=2 SV=1 - (A8K4I8_HUMAN)
E7EX53	Ribosomal protein L15 (Fragment) OS=Homo sapiens GN=RPL15 PE=3 SV=1 - (E7EX53_HUMAN)
Q07955	Serine/arginine-rich splicing factor 1 OS=Homo sapiens GN=SRSF1 PE=1 SV=2 - (SRSF1_HUMAN)
Q9H0R4	Haloacid dehalogenase-like hydrolase domain-containing protein 2 OS=Homo sapiens GN=HDHD2 PE=1 SV=1 - (HDHD2_HUMAN)
B3KRM2	Serine/threonine-protein phosphatase OS=Homo sapiens PE=2 SV=1 - (B3KRM2_HUMAN)
Q5T3W0	Absent in melanoma 2 (Fragment) OS=Homo sapiens GN=AIM2 PE=2 SV=1 - (Q5T3W0_HUMAN)
Q8IYS2-2	Isoform 2 of Uncharacterized protein KIAA2013 OS=Homo sapiens GN=KIAA2013 - (K2013_HUMAN)
C9JJ54	WD repeat and FYVE domain-containing protein 1 (Fragment) OS=Homo sapiens GN=WDFY1 PE=4 SV=1 - (C9JJ54_HUMAN)
P51572	B-cell receptor-associated protein 31 OS=Homo sapiens GN=BCAP31 PE=1 SV=3 - (BAP31_HUMAN)
A8K3Z3	26S protease regulatory subunit 8 OS=Homo sapiens GN=PSMC5 PE=2 SV=1 - (A8K3Z3_HUMAN)
O75368	SH3 domain-binding glutamic acid-rich-like protein OS=Homo sapiens GN=SH3BGRL PE=1 SV=1 - (SH3L1_HUMAN)
A8K0H3	cDNA FLJ78093, highly similar to Homo sapiens ribosomal protein L29 (RPL29), mRNA OS=Homo sapiens PE=2 SV=1 - (A8K0H3_HUMAN)
P98088	Mucin-5AC (Fragments) OS=Homo sapiens GN=MUC5AC PE=1 SV=3 - (MUC5A_HUMAN)
Q6GMX0	Putative uncharacterized protein OS=Homo sapiens PE=2 SV=1 - (Q6GMX0_HUMAN)
E5RIV2	Centrosomal protein CEP57L1 OS=Homo sapiens GN=CEP57L1 PE=4 SV=1 - (E5RIV2_HUMAN)
B7Z7X0	cDNA FLJ51151, highly similar to Receptor-interacting serine/threonine-protein kinase 4 (EC 2.7.11.1) OS=Homo sapiens PE=2 SV=1 - (B7Z7X0_HUMAN)
Q8NE71	ATP-binding cassette sub-family F member 1 OS=Homo sapiens GN=ABCF1 PE=1 SV=2 - (ABCF1_HUMAN)
P62304	Small nuclear ribonucleoprotein E OS=Homo sapiens GN=SNRPE PE=1 SV=1 - (RUXE_HUMAN)
Q96MH7	Uncharacterized protein C5orf34 OS=Homo sapiens GN=C5orf34 PE=2 SV=2 - (CE034_HUMAN)
Q9H293-2	Isoform 2 of Interleukin-25 OS=Homo sapiens GN=IL25 - (IL25_HUMAN)
B3KPC7	Actin-related protein 2/3 complex subunit 5 OS=Homo sapiens PE=2 SV=1 - (B3KPC7_HUMAN)
H0YJB9	UPF0568 protein C14orf166 (Fragment) OS=Homo sapiens GN=C14orf166 PE=4 SV=1 - (H0YJB9_HUMAN)
Q53H37	Calmodulin-like skin protein variant (Fragment) OS=Homo sapiens PE=2 SV=1 - (Q53H37_HUMAN)
C9IZG4	Protein CutA OS=Homo sapiens GN=CUTA PE=4 SV=1 - (C9IZG4_HUMAN)
O00231	26S proteasome non-ATPase regulatory subunit 11 OS=Homo sapiens GN=PSMD11 PE=1 SV=3 - (PSD11_HUMAN)
B4DVQ3	Calcium/calmodulin-dependent protein kinase type II subunit gamma OS=Homo sapiens GN=CAMK2G PE=2 SV=1 - (B4DVQ3_HUMAN)
E9PR17	CD59 glycoprotein OS=Homo sapiens GN=CD59 PE=4 SV=1 - (E9PR17_HUMAN)
P61960	Ubiquitin-fold modifier 1 OS=Homo sapiens GN=UFM1 PE=1 SV=1 - (UFM1_HUMAN)
B4DEL6	Phospholipase D3 OS=Homo sapiens GN=PLD3 PE=2 SV=1 - (B4DEL6_HUMAN)
Q3LI72	Keratin-associated protein 19-5 OS=Homo sapiens GN=KRTAP19-5 PE=1 SV=1 - (KR195_HUMAN)
B2R4N3	cDNA, FLJ92155, highly similar to Homo sapiens ubiquitin-like 5 (UBL5), mRNA OS=Homo sapiens PE=4 SV=1 - (B2R4N3_HUMAN)
H7BYG3	Leucine-rich repeats and immunoglobulin-like domains protein 1 OS=Homo sapiens GN=LRIG1 PE=4 SV=1 - (H7BYG3_HUMAN)
Q5TA02	Glutathione S-transferase omega 1 (Fragment) OS=Homo sapiens GN=GSTO1 PE=2 SV=1 - (Q5TA02_HUMAN)
E7ET82	Ly6/PLAUR domain-containing protein 3 OS=Homo sapiens GN=LYPD3 PE=4 SV=1 - (E7ET82_HUMAN)
Q4J6C6-4	Isoform 4 of Prolyl endopeptidase-like OS=Homo sapiens GN=PREPL - (PPCEL_HUMAN)
G3V2K7	Transmembrane emp24 domain-containing protein 10 OS=Homo sapiens GN=TMED10 PE=3 SV=1 - (G3V2K7_HUMAN)
O15144	Actin-related protein 2/3 complex subunit 2 OS=Homo sapiens GN=ARPC2 PE=1 SV=1 - (ARPC2_HUMAN)
Q8IV48	3'-5' exoribonuclease 1 OS=Homo sapiens GN=ERI1 PE=1 SV=3 - (ERI1_HUMAN)
Q4ZG77	Putative uncharacterized protein PSMD14 (Fragment) OS=Homo sapiens GN=PSMD14 PE=2 SV=1 - (Q4ZG77_HUMAN)
B3KU40	cDNA FLJ39163 fis, clone OCBBF2002615, highly similar to Low-density lipoprotein receptor-related protein 8 OS=Homo sapiens PE=2 SV=1 - (B3KU40_HUMAN)
P62861	40S ribosomal protein S30 OS=Homo sapiens GN=FAU PE=1 SV=1 - (RS30_HUMAN)
C9J7D1	Ras-related protein Rab-7a OS=Homo sapiens GN=RAB7A PE=4 SV=1 - (C9J7D1_HUMAN)
Q9BRL5	CALM3 protein OS=Homo sapiens PE=2 SV=1 - (Q9BRL5_HUMAN)
Q05CP6	ZNF140 protein OS=Homo sapiens GN=ZNF140 PE=2 SV=1 - (Q05CP6_HUMAN)
A8K4W8	cDNA FLJ77917, highly similar to Homo sapiens ubiquitin-conjugating enzyme E2L 3 (UBE2L3), transcript variant 1, mRNA OS=Homo sapiens PE=2 SV=1 - (A8K4W8_HUMAN)
Q9BYR3	Keratin-associated protein 4-4 OS=Homo sapiens GN=KRTAP4-4 PE=2 SV=1 - (KRA44_HUMAN)
Q8IX95	Putative cutaneous T-cell lymphoma-associated antigen 3 OS=Homo sapiens GN=CTAGE3P PE=5 SV=1 - (CTGE3_HUMAN)
F8VRZ3	Proliferation-associated protein 2G4 OS=Homo sapiens GN=PA2G4 PE=4 SV=1 - (F8VRZ3_HUMAN)
P49773	Histidine triad nucleotide-binding protein 1 OS=Homo sapiens GN=HINT1 PE=1 SV=2 - (HINT1_HUMAN)
G5EA35	Caspase recruitment domain-containing protein 18 OS=Homo sapiens GN=CARD18 PE=4 SV=1 - (G5EA35_HUMAN)
B4DHP2	Prostaglandin E synthase 3 OS=Homo sapiens GN=PTGES3 PE=2 SV=1 - (B4DHP2_HUMAN)
P04632	Calpain small subunit 1 OS=Homo sapiens GN=CAPNS1 PE=1 SV=1 - (CPNS1_HUMAN)
Q8NA70	Protein FAM47B OS=Homo sapiens GN=FAM47B PE=2 SV=2 - (FA47B_HUMAN)
G3V1C1	1,5-anhydro-D-fructose reductase OS=Homo sapiens GN=AKR1E2 PE=4 SV=1 - (G3V1C1_HUMAN)
B4DF76	cDNA FLJ59191, highly similar to NADH dehydrogenase (ubiquinone) 1 alpha subcomplex subunit 13 (EC 1.6.5.3) OS=Homo sapiens PE=2 SV=1 - (B4DF76_HUMAN)
P19105	Myosin regulatory light chain 12A OS=Homo sapiens GN=MYL12A PE=1 SV=2 - (ML12A_HUMAN)
B4DZF3	cDNA FLJ60995 OS=Homo sapiens PE=2 SV=1 - (B4DZF3_HUMAN)
Q9Y5G4-2	Isoform 2 of Protocadherin gamma-A9 OS=Homo sapiens GN=PCDHGA9 - (PCDG9_HUMAN)
Q04760-2	Isoform 2 of Lactoylglutathione lyase OS=Homo sapiens GN=GLO1 - (LGUL_HUMAN)
Q86YI3	PSD protein (Fragment) OS=Homo sapiens GN=PSD PE=2 SV=2 - (Q86YI3_HUMAN)
O75223	Gamma-glutamylcyclotransferase OS=Homo sapiens GN=GGCT PE=1 SV=1 - (GGCT_HUMAN)
H3BPG0	Cytochrome c oxidase subunit 4 isoform 1, mitochondrial (Fragment) OS=Homo sapiens GN=COX4I1 PE=4 SV=1 - (H3BPG0_HUMAN)
C1PHC4	CD44 molecule (Fragment) OS=Homo sapiens GN=CD44 PE=4 SV=1 - (C1PHC4_HUMAN)
I3L0K2	Thioredoxin domain-containing protein 17 OS=Homo sapiens GN=TXNDC17 PE=4 SV=1 - (I3L0K2_HUMAN)
Q8WZ58	Achaete-scute associated protein OS=Homo sapiens GN=hCG_1648122 PE=4 SV=1 - (Q8WZ58_HUMAN)
C9JR72	Kelch repeat and BTB domain-containing protein 13 OS=Homo sapiens GN=KBTBD13 PE=1 SV=1 - (KBTBD_HUMAN)
F2VNQ2	MHC class I antigen (Fragment) OS=Homo sapiens GN=HLA-C PE=3 SV=1 - (F2VNQ2_HUMAN)
Q59GT9	Gap junction protein (Fragment) OS=Homo sapiens PE=2 SV=1 - (Q59GT9_HUMAN)
I3L1H9	Zymogen granule protein 16 homolog B (Fragment) OS=Homo sapiens GN=ZG16B PE=4 SV=1 - (I3L1H9_HUMAN)
Q4QRI4	RGS9 protein OS=Homo sapiens GN=RGS9 PE=2 SV=1 - (Q4QRI4_HUMAN)
P62306	Small nuclear ribonucleoprotein F OS=Homo sapiens GN=SNRPF PE=1 SV=1 - (RUXF_HUMAN)
B3KTA3	cDNA FLJ37935 fis, clone CTONG2005290, highly similar to FASCIN OS=Homo sapiens PE=2 SV=1 - (B3KTA3_HUMAN)
P26998	Beta-crystallin B3 OS=Homo sapiens GN=CRYBB3 PE=1 SV=4 - (CRBB3_HUMAN)
P56556	NADH dehydrogenase (ubiquinone) 1 alpha subcomplex subunit 6 OS=Homo sapiens GN=NDUFA6 PE=1 SV=3 - (NDUA6_HUMAN)
B1N7B6	Cryocrystalglobulin CC1 heavy chain variable region (Fragment) OS=Homo sapiens PE=2 SV=1 - (B1N7B6_HUMAN)
Q5VTU8	ATP synthase subunit epsilon-like protein, mitochondrial OS=Homo sapiens GN=ATP5EP2 PE=1 SV=1 - (AT5EL_HUMAN)
B7Z3I9	Delta-aminolevulinic acid dehydratase OS=Homo sapiens PE=2 SV=1 - (B7Z3I9_HUMAN)
H7BY78	Hypoxanthine-guanine phosphoribosyltransferase OS=Homo sapiens GN=HPRT1 PE=4 SV=1 - (H7BY78_HUMAN)
B4DX37	cDNA FLJ58296, highly similar to Kelch-like protein 10 OS=Homo sapiens PE=2 SV=1 - (B4DX37_HUMAN)
Q9UI42-2	Isoform 2 of Carboxypeptidase A4 OS=Homo sapiens GN=CPA4 - (CBPA4_HUMAN)
A8K6K8	cDNA FLJ77325 OS=Homo sapiens PE=2 SV=1 - (A8K6K8_HUMAN)
Q8WV83-2	Isoform 2 of Solute carrier family 35 member F5 OS=Homo sapiens GN=SLC35F5 - (S35F5_HUMAN)
Q9Y6X9	MORC family CW-type zinc finger protein 2 OS=Homo sapiens GN=MORC2 PE=1 SV=2 - (MORC2_HUMAN)
D6R9P3	Heterogeneous nuclear ribonucleoprotein A/B OS=Homo sapiens GN=HNRNPAB PE=4 SV=1 - (D6R9P3_HUMAN)
Q14393-2	Isoform 2 of Growth arrest-specific protein 6 OS=Homo sapiens GN=GAS6 - (GAS6_HUMAN)
B3KT45	cDNA FLJ37607 fis, clone BRCOC2010980, highly similar to Thioredoxin-like protein 1 OS=Homo sapiens PE=2 SV=1 - (B3KT45_HUMAN)
A8K9G4	cDNA FLJ77745, highly similar to Homo sapiens neutral sphingomyelinase (N-SMase) activation associated factor (NSMAF), mRNA OS=Homo sapiens PE=2 SV=1 - (A8K9G4_HUMAN)
H3BPK3	Hydroxyacylglutathione hydrolase, mitochondrial (Fragment) OS=Homo sapiens GN=HAGH PE=3 SV=1 - (H3BPK3_HUMAN)
Q53FH6	Mitogen-activated protein kinase kinase 1 interacting protein 1 variant (Fragment) OS=Homo sapiens PE=2 SV=1 - (Q53FH6_HUMAN)
F6S6P2	Large proline-rich protein BAG6 (Fragment) OS=Homo sapiens GN=BAG6 PE=4 SV=1 - (F6S6P2_HUMAN)
Q8IWR8	Ribosomal protein L19 (Fragment) OS=Homo sapiens PE=2 SV=1 - (Q8IWR8_HUMAN)
B7Z2R9	Lysosome-associated membrane glycoprotein 2 OS=Homo sapiens GN=LAMP2 PE=2 SV=1 - (B7Z2R9_HUMAN)
Q96QR8	Transcriptional activator protein Pur-beta OS=Homo sapiens GN=PURB PE=1 SV=3 - (PURB_HUMAN)
B2REB8	Protein SET OS=Homo sapiens GN=SET PE=3 SV=1 - (B2REB8_HUMAN)
H0Y3U0	Parvalbumin alpha (Fragment) OS=Homo sapiens GN=PVALB PE=4 SV=1 - (H0Y3U0_HUMAN)
Q6IFR1	Olfactory receptor OR11-87 OS=Homo sapiens PE=2 SV=1 - (Q6IFR1_HUMAN)
P62841	40S ribosomal protein S15 OS=Homo sapiens GN=RPS15 PE=1 SV=2 - (RS15_HUMAN)
Q96K62	Zinc finger and BTB domain-containing protein 45 OS=Homo sapiens GN=ZBTB45 PE=2 SV=1 - (ZBT45_HUMAN)
C9K0U8	Single-stranded DNA-binding protein, mitochondrial (Fragment) OS=Homo sapiens GN=SSBP1 PE=4 SV=1 - (C9K0U8_HUMAN)
O95833	Chloride intracellular channel protein 3 OS=Homo sapiens GN=CLIC3 PE=1 SV=2 - (CLIC3_HUMAN)
F8VU40	Proline-rich protein 13 OS=Homo sapiens GN=PRR13 PE=4 SV=1 - (F8VU40_HUMAN)
F8VS20	Transmembrane protein 19 (Fragment) OS=Homo sapiens GN=TMEM19 PE=4 SV=1 - (F8VS20_HUMAN)
F5H5Y2	Translocon-associated protein subunit alpha OS=Homo sapiens GN=SSR1 PE=4 SV=1 - (F5H5Y2_HUMAN)
Q14DE1	GLDN protein OS=Homo sapiens GN=GLDN PE=2 SV=1 - (Q14DE1_HUMAN)
F8W9J5	Homeobox even-skipped homolog protein 1 OS=Homo sapiens GN=EVX1 PE=4 SV=1 - (F8W9J5_HUMAN)
P15121	Aldose reductase OS=Homo sapiens GN=AKR1B1 PE=1 SV=3 - (ALDR_HUMAN)
Q8NEJ1	Putative uncharacterized protein OS=Homo sapiens PE=2 SV=1 - (Q8NEJ1_HUMAN)
Q96NG3	Tetratricopeptide repeat protein 25 OS=Homo sapiens GN=TTC25 PE=1 SV=2 - (TTC25_HUMAN)
Q0EFA5	S protein OS=Homo sapiens GN=S PE=2 SV=1 - (Q0EFA5_HUMAN)
F8VQ14	T-complex protein 1 subunit beta OS=Homo sapiens GN=CCT2 PE=3 SV=1 - (F8VQ14_HUMAN)
B4E1K5	Uncharacterized protein OS=Homo sapiens GN=DHCR7 PE=2 SV=1 - (B4E1K5_HUMAN)
A6NKB5-2	Isoform 2 of Pecanex-like protein 2 OS=Homo sapiens GN=PCNXL2 - (PCX2_HUMAN)
B4E2W0	3-ketoacyl-CoA thiolase OS=Homo sapiens GN=HADHB PE=2 SV=1 - (B4E2W0_HUMAN)
I3L0X8	Coronin-7 (Fragment) OS=Homo sapiens GN=CORO7 PE=4 SV=1 - (I3L0X8_HUMAN)
C9J5N9	Apolipoprotein O-like OS=Homo sapiens GN=APOOL PE=4 SV=2 - (C9J5N9_HUMAN)
G3V3Q9	Paired box protein Pax-6 (Fragment) OS=Homo sapiens GN=PAX6 PE=3 SV=1 - (G3V3Q9_HUMAN)
A8K5Q6	cDNA FLJ75694, highly similar to Homo sapiens G protein-coupled receptor 44 (GPR44), mRNA OS=Homo sapiens PE=2 SV=1 - (A8K5Q6_HUMAN)
Q68CR9	Aspartate--tRNA ligase, cytoplasmic OS=Homo sapiens GN=DKFZp781B11202 PE=2 SV=1 - (Q68CR9_HUMAN)
O95336	6-phosphogluconolactonase OS=Homo sapiens GN=PGLS PE=1 SV=2 - (6PGL_HUMAN)
E7EUN2	Arf-GAP with GTPase, ANK repeat and PH domain-containing protein 1 OS=Homo sapiens GN=AGAP1 PE=4 SV=1 - (E7EUN2_HUMAN)
Q96SM3	Probable carboxypeptidase X1 OS=Homo sapiens GN=CPXM1 PE=2 SV=2 - (CPXM1_HUMAN)
Q53SX6	Putative uncharacterized protein KYNU (Fragment) OS=Homo sapiens GN=KYNU PE=2 SV=1 - (Q53SX6_HUMAN)
B2RAH1	cDNA, FLJ94913, highly similar to Homo sapiens holocarboxylase synthetase(biotin-(proprionyl-Coenzyme A-carboxylase (ATP-hydrolysing))ligase) (HLCS), mRNA OS=Homo sapiens PE=2 SV=1 - (B2RAH1_HUMAN)
Q6WG76	Antigen MLAA-20 (Fragment) OS=Homo sapiens PE=2 SV=1 - (Q6WG76_HUMAN)
B4DT68	Myotilin OS=Homo sapiens GN=MYOT PE=2 SV=1 - (B4DT68_HUMAN)
B4DET6	cDNA FLJ59314, highly similar to UV radiation resistance-associated gene protein OS=Homo sapiens PE=2 SV=1 - (B4DET6_HUMAN)
B3KY44	Solute carrier family 11 (Proton-coupled divalent metal ion transporters), member 2, isoform CRA_c OS=Homo sapiens GN=SLC11A2 PE=2 SV=1 - (B3KY44_HUMAN)
A8K8F6	cDNA FLJ78417, highly similar to Homo sapiens low density lipoprotein receptor-related protein associated protein 1 (LRPAP1), mRNA OS=Homo sapiens PE=2 SV=1 - (A8K8F6_HUMAN)
Q6AHY1	Putative uncharacterized protein DKFZp686O2142 OS=Homo sapiens GN=DKFZp686O2142 PE=2 SV=1 - (Q6AHY1_HUMAN)
Q9Y2S2-2	Isoform 2 of Lambda-crystallin homolog OS=Homo sapiens GN=CRYL1 - (CRYL1_HUMAN)
P30414	NK-tumor recognition protein OS=Homo sapiens GN=NKTR PE=1 SV=2 - (NKTR_HUMAN)
G3V1A8	Lymphocyte antigen 6 complex locus protein G6c OS=Homo sapiens GN=LY6G6C PE=4 SV=1 - (G3V1A8_HUMAN)
Q8WYK1	Contactin-associated protein-like 5 OS=Homo sapiens GN=CNTNAP5 PE=2 SV=1 - (CNTP5_HUMAN)
Q6T4R5	Nance-Horan syndrome protein OS=Homo sapiens GN=NHS PE=1 SV=1 - (NHS_HUMAN)
Q5EBM0	UMP-CMP kinase 2, mitochondrial OS=Homo sapiens GN=CMPK2 PE=1 SV=3 - (CMPK2_HUMAN)
P0C6C1	Ankyrin repeat domain-containing protein 34C OS=Homo sapiens GN=ANKRD34C PE=2 SV=1 - (AN34C_HUMAN)
Q96PC3-2	Isoform 2 of AP-1 complex subunit sigma-3 OS=Homo sapiens GN=AP1S3 - (AP1S3_HUMAN)
Q5T0J7-4	Isoform 4 of Uncharacterized protein C1orf49 OS=Homo sapiens GN=C1orf49 - (CA049_HUMAN)
Q8N998	Coiled-coil domain-containing protein 89 OS=Homo sapiens GN=CCDC89 PE=2 SV=1 - (CCD89_HUMAN)
Q0VDD5	Putative uncharacterized protein encoded by MIR22HG OS=Homo sapiens GN=MIR22HG PE=5 SV=1 - (CQ091_HUMAN)
O14602	Eukaryotic translation initiation factor 1A, Y-chromosomal OS=Homo sapiens GN=EIF1AY PE=1 SV=4 - (IF1AY_HUMAN)
Q3MIP1	Inositol 1,4,5-trisphosphate receptor-interacting protein-like 2 OS=Homo sapiens GN=ITPRIPL2 PE=2 SV=1 - (IPIL2_HUMAN)
Q8IXI1	Mitochondrial Rho GTPase 2 OS=Homo sapiens GN=RHOT2 PE=1 SV=2 - (MIRO2_HUMAN)
Q99542-3	Isoform 2 of Matrix metalloproteinase-19 OS=Homo sapiens GN=MMP19 - (MMP19_HUMAN)
Q9BV57-2	Isoform 2 of 1,2-dihydroxy-3-keto-5-methylthiopentene dioxygenase OS=Homo sapiens GN=ADI1 - (MTND_HUMAN)
P98179	Putative RNA-binding protein 3 OS=Homo sapiens GN=RBM3 PE=1 SV=1 - (RBM3_HUMAN)
Q9P1V8-2	Isoform 2 of Sterile alpha motif domain-containing protein 15 OS=Homo sapiens GN=SAMD15 - (SAM15_HUMAN)
Q9P0U3-2	Isoform 2 of Sentrin-specific protease 1 OS=Homo sapiens GN=SENP1 - (SENP1_HUMAN)
Q969X2-2	Isoform 2 of Alpha-N-acetylgalactosaminide alpha-2,6-sialyltransferase 6 OS=Homo sapiens GN=ST6GALNAC6 - (SIA7F_HUMAN)
Q8IWV8-3	Isoform 3 of E3 ubiquitin-protein ligase UBR2 OS=Homo sapiens GN=UBR2 - (UBR2_HUMAN)
Q9Y2H8	Zinc finger protein 510 OS=Homo sapiens GN=ZNF510 PE=2 SV=1 - (ZN510_HUMAN)
Q8NEK5	Zinc finger protein 548 OS=Homo sapiens GN=ZNF548 PE=2 SV=2 - (ZN548_HUMAN)
E9PEN1	Ankyrin repeat and SOCS box protein 11 OS=Homo sapiens GN=ASB11 PE=4 SV=1 - (E9PEN1_HUMAN)
B5BU41	Calcium/calmodulin-dependent protein kinase I OS=Homo sapiens GN=CAMK1 PE=2 SV=1 - (B5BU41_HUMAN)
B7ZAM9	Eukaryotic translation initiation factor 3 subunit K OS=Homo sapiens GN=EIF3K PE=2 SV=1 - (B7ZAM9_HUMAN)
B3KNR6	cDNA FLJ30255 fis, clone BRACE2002444, highly similar to LAS1-like protein OS=Homo sapiens PE=2 SV=1 - (B3KNR6_HUMAN)
A8KA83	cDNA FLJ78586, highly similar to Homo sapiens VAMP (vesicle-associated membrane protein)-associated protein A, 33kDa (VAPA), mRNA OS=Homo sapiens PE=2 SV=1 - (A8KA83_HUMAN)
B9EIM4	Olfactory receptor, family 4, subfamily A, member 47 OS=Homo sapiens GN=OR4A47 PE=2 SV=1 - (B9EIM4_HUMAN)
I3L3L9	A-kinase anchor protein 1, mitochondrial (Fragment) OS=Homo sapiens GN=AKAP1 PE=4 SV=1 - (I3L3L9_HUMAN)
B4DZZ0	cDNA FLJ52128, highly similar to PRA1 family protein 3 OS=Homo sapiens PE=2 SV=1 - (B4DZZ0_HUMAN)
F5H2Q4	Tyrosine-protein phosphatase non-receptor type 9 OS=Homo sapiens GN=PTPN9 PE=4 SV=1 - (F5H2Q4_HUMAN)
A8K3G7	cDNA FLJ75262, highly similar to Homo sapiens cAMP specific phosphodiesterase 7B (PDE7B gene) OS=Homo sapiens PE=2 SV=1 - (A8K3G7_HUMAN)
Q53H03	Nuclear autoantigenic sperm protein isoform 2 variant (Fragment) OS=Homo sapiens PE=2 SV=1 - (Q53H03_HUMAN)
E5RI82	Inositol monophosphatase 1 (Fragment) OS=Homo sapiens GN=IMPA1 PE=4 SV=1 - (E5RI82_HUMAN)
Q6PJ68	ZNF134 protein (Fragment) OS=Homo sapiens GN=ZNF134 PE=2 SV=1 - (Q6PJ68_HUMAN)
A4D142	Similar to ribosomal protein L23 OS=Homo sapiens GN=LOC222901 PE=3 SV=1 - (A4D142_HUMAN)
Q8WYX5	Putative uncharacterized protein pp10472 OS=Homo sapiens GN=pp10472 PE=2 SV=1 - (Q8WYX5_HUMAN)
A8K874	cDNA FLJ77588, highly similar to Homo sapiens MAK10 homolog, amino-acid N-acetyltransferase subunit, (S. cerevisiae) (MAK10), mRNA OS=Homo sapiens PE=2 SV=1 - (A8K874_HUMAN)
B4DP56	cDNA FLJ52237, highly similar to Creatine kinase B-type (EC 2.7.3.2) OS=Homo sapiens PE=2 SV=1 - (B4DP56_HUMAN)
B4DVS4	cDNA FLJ57619, highly similar to Delta 3,5-delta 2,4-dienoyl-CoA isomerase, mitochondrial (EC 5.3.3.-) OS=Homo sapiens PE=2 SV=1 - (B4DVS4_HUMAN)
E5RK52	Replication initiator 1 OS=Homo sapiens GN=REPIN1 PE=4 SV=1 - (E5RK52_HUMAN)
F5H686	Ras-related and estrogen-regulated growth inhibitor-like protein OS=Homo sapiens GN=RERGL PE=4 SV=1 - (F5H686_HUMAN)
F5H110	Zinc finger CCCH domain-containing protein 6 OS=Homo sapiens GN=ZC3H6 PE=4 SV=1 - (F5H110_HUMAN)
B7Z850	cDNA FLJ54514 OS=Homo sapiens PE=2 SV=1 - (B7Z850_HUMAN)
B4DWB4	cDNA FLJ58332, highly similar to MBD2-interacting zinc finger protein OS=Homo sapiens PE=2 SV=1 - (B4DWB4_HUMAN)
Q8NAV9	cDNA FLJ34690 fis, clone MESAN2000894 OS=Homo sapiens PE=2 SV=1 - (Q8NAV9_HUMAN)
A8K8R2	cDNA FLJ77381, highly similar to Homo sapiens cation channel, sperm associated 3 (CATSPER3), mRNA OS=Homo sapiens PE=2 SV=1 - (A8K8R2_HUMAN)
Q9Y2S1	Galanin-related peptide OS=Homo sapiens PE=4 SV=1 - (Q9Y2S1_HUMAN)
A8K0P8	cDNA FLJ78223 OS=Homo sapiens PE=2 SV=1 - (A8K0P8_HUMAN)
B3KMR4	cDNA FLJ12419 fis, clone MAMMA1003047, highly similar to Protein inhibitor of activated STAT protein 4 OS=Homo sapiens PE=2 SV=1 - (B3KMR4_HUMAN)
G3V4X5	Proteasome subunit alpha type-3 OS=Homo sapiens GN=PSMA3 PE=4 SV=1 - (G3V4X5_HUMAN)
B2R853	cDNA, FLJ93744, highly similar to Homo sapiens keratin 6E (KRT6E), mRNA OS=Homo sapiens PE=2 SV=1 - (B2R853_HUMAN)
Q13092	Epidermal type I keratin (Fragment) OS=Homo sapiens GN=K14 PE=1 SV=1 - (Q13092_HUMAN)
P12035	Keratin, type II cytoskeletal 3 OS=Homo sapiens GN=KRT3 PE=1 SV=3 - (K2C3_HUMAN)
I3L1M0	Uncharacterized protein OS=Homo sapiens PE=4 SV=1 - (I3L1M0_HUMAN)
H9KV51	Keratin, type II cytoskeletal 72 OS=Homo sapiens GN=KRT72 PE=3 SV=1 - (H9KV51_HUMAN)
P05787	Keratin, type II cytoskeletal 8 OS=Homo sapiens GN=KRT8 PE=1 SV=7 - (K2C8_HUMAN)
P07355	Annexin A2 OS=Homo sapiens GN=ANXA2 PE=1 SV=2 - (ANXA2_HUMAN)
P68371	Tubulin beta-4B chain OS=Homo sapiens GN=TUBB4B PE=1 SV=1 - (TBB4B_HUMAN)
P68032	Actin, alpha cardiac muscle 1 OS=Homo sapiens GN=ACTC1 PE=1 SV=1 - (ACTC_HUMAN)
P12814-2	Isoform 2 of Alpha-actinin-1 OS=Homo sapiens GN=ACTN1 - (ACTN1_HUMAN)
P08729	Keratin, type II cytoskeletal 7 OS=Homo sapiens GN=KRT7 PE=1 SV=5 - (K2C7_HUMAN)
O60814	Histone H2B type 1-K OS=Homo sapiens GN=HIST1H2BK PE=1 SV=3 - (H2B1K_HUMAN)
Q15149	Plectin OS=Homo sapiens GN=PLEC PE=1 SV=3 - (PLEC_HUMAN)
B2RBD5	cDNA, FLJ95457, highly similar to Homo sapiens tubulin, beta, 4 (TUBB4), mRNA OS=Homo sapiens PE=2 SV=1 - (B2RBD5_HUMAN)
P21333-2	Isoform 2 of Filamin-A OS=Homo sapiens GN=FLNA - (FLNA_HUMAN)
Q3ZCM7	Tubulin beta-8 chain OS=Homo sapiens GN=TUBB8 PE=1 SV=2 - (TBB8_HUMAN)
P31946-2	Isoform Short of 14-3-3 protein beta/alpha OS=Homo sapiens GN=YWHAB - (1433B_HUMAN)
P68104	Elongation factor 1-alpha 1 OS=Homo sapiens GN=EEF1A1 PE=1 SV=1 - (EF1A1_HUMAN)
P02545-3	Isoform ADelta10 of Prelamin-A/C OS=Homo sapiens GN=LMNA - (LMNA_HUMAN)
Q9NSB5	Type II hair keratin 1 (Fragment) OS=Homo sapiens GN=KRTHB1 PE=2 SV=1 - (Q9NSB5_HUMAN)
Q9H853	Putative tubulin-like protein alpha-4B OS=Homo sapiens GN=TUBA4B PE=5 SV=2 - (TBA4B_HUMAN)
D3DPU2	Adenylyl cyclase-associated protein OS=Homo sapiens GN=CAP1 PE=3 SV=1 - (D3DPU2_HUMAN)
P35609	Alpha-actinin-2 OS=Homo sapiens GN=ACTN2 PE=1 SV=1 - (ACTN2_HUMAN)
Q9P225-2	Isoform 2 of Dynein heavy chain 2, axonemal OS=Homo sapiens GN=DNAH2 - (DYH2_HUMAN)
P26038	Moesin OS=Homo sapiens GN=MSN PE=1 SV=3 - (MOES_HUMAN)
P84243	Histone H3.3 OS=Homo sapiens GN=H3F3A PE=1 SV=2 - (H33_HUMAN)
Q5DT20	Hornerin OS=Homo sapiens GN=HRNR PE=2 SV=1 - (Q5DT20_HUMAN)
B2R8Y4	cDNA, FLJ94117, highly similar to Homo sapiens actinin, alpha 3 (ACTN3), mRNA OS=Homo sapiens PE=2 SV=1 - (B2R8Y4_HUMAN)
A8K6T3	cDNA FLJ78674, highly similar to Homo sapiens desmocollin type 4 OS=Homo sapiens PE=2 SV=1 - (A8K6T3_HUMAN)
P06576	ATP synthase subunit beta, mitochondrial OS=Homo sapiens GN=ATP5B PE=1 SV=3 - (ATPB_HUMAN)
Q53HU8	Vimentin variant (Fragment) OS=Homo sapiens PE=2 SV=1 - (Q53HU8_HUMAN)
Q71DI3	Histone H3.2 OS=Homo sapiens GN=HIST2H3A PE=1 SV=3 - (H32_HUMAN)
Q9HD64-4	Isoform D of G antigen family D member 2 OS=Homo sapiens GN=XAGE1A - (GAGD2_HUMAN)
F6KPG5	Albumin (Fragment) OS=Homo sapiens PE=2 SV=1 - (F6KPG5_HUMAN)
B7ZLH8	EVPL protein OS=Homo sapiens GN=EVPL PE=2 SV=1 - (B7ZLH8_HUMAN)
P00338	L-lactate dehydrogenase A chain OS=Homo sapiens GN=LDHA PE=1 SV=2 - (LDHA_HUMAN)
P23528	Cofilin-1 OS=Homo sapiens GN=CFL1 PE=1 SV=3 - (COF1_HUMAN)
P07900-2	Isoform 2 of Heat shock protein HSP 90-alpha OS=Homo sapiens GN=HSP90AA1 - (HS90A_HUMAN)
D3DVC4	Nestin, isoform CRA_c OS=Homo sapiens GN=NES PE=3 SV=1 - (D3DVC4_HUMAN)
P50607-2	Isoform 2 of Tubby protein homolog OS=Homo sapiens GN=TUB - (TUB_HUMAN)
P18206-2	Isoform 1 of Vinculin OS=Homo sapiens GN=VCL - (VINC_HUMAN)
B4DPS6	cDNA FLJ59371, highly similar to Exportin-2 OS=Homo sapiens PE=2 SV=1 - (B4DPS6_HUMAN)
O15078	Centrosomal protein of 290 kDa OS=Homo sapiens GN=CEP290 PE=1 SV=2 - (CE290_HUMAN)
Q02878	60S ribosomal protein L6 OS=Homo sapiens GN=RPL6 PE=1 SV=3 - (RL6_HUMAN)
Q6KB66	Keratin, type II cytoskeletal 80 OS=Homo sapiens GN=KRT80 PE=1 SV=2 - (K2C80_HUMAN)
Q8N2C7-4	Isoform 4 of Protein unc-80 homolog OS=Homo sapiens GN=UNC80 - (UNC80_HUMAN)
C7DJS2	Glutathione S-transferase pi (Fragment) OS=Homo sapiens GN=GSTP1 PE=2 SV=1 - (C7DJS2_HUMAN)
G9JV18	Coiled-coil domain containing 154 OS=Homo sapiens GN=CCDC154 PE=2 SV=1 - (G9JV18_HUMAN)
Q8NEH6	Meiosis-specific nuclear structural protein 1 OS=Homo sapiens GN=MNS1 PE=2 SV=2 - (MNS1_HUMAN)
Q8IUC1	Keratin-associated protein 11-1 OS=Homo sapiens GN=KRTAP11-1 PE=1 SV=1 - (KR111_HUMAN)
Q6TXQ4	H3L-like histone (Fragment) OS=Homo sapiens PE=2 SV=1 - (Q6TXQ4_HUMAN)
Q5VU43-4	Isoform 4 of Myomegalin OS=Homo sapiens GN=PDE4DIP - (MYOME_HUMAN)
B7Z6Z4	Myosin light polypeptide 6 OS=Homo sapiens GN=MYL6 PE=2 SV=1 - (B7Z6Z4_HUMAN)
Q01518-2	Isoform 2 of Adenylyl cyclase-associated protein 1 OS=Homo sapiens GN=CAP1 - (CAP1_HUMAN)
Q6P9G8	Family with sequence similarity 184, member A OS=Homo sapiens GN=FAM184A PE=2 SV=2 - (Q6P9G8_HUMAN)
Q5TZA2	Rootletin OS=Homo sapiens GN=CROCC PE=1 SV=1 - (CROCC_HUMAN)
Q9Y490	Talin-1 OS=Homo sapiens GN=TLN1 PE=1 SV=3 - (TLN1_HUMAN)
Q75MZ1	Putative uncharacterized protein ZNF277 (Fragment) OS=Homo sapiens GN=ZNF277 PE=2 SV=1 - (Q75MZ1_HUMAN)
B4DUQ1	cDNA FLJ54552, highly similar to Heterogeneous nuclear ribonucleoprotein K OS=Homo sapiens PE=2 SV=1 - (B4DUQ1_HUMAN)
B9EK46	Cingulin OS=Homo sapiens GN=CGN PE=2 SV=1 - (B9EK46_HUMAN)
Q2Q9B7	Glucose-6-phosphate 1-dehydrogenase (Fragment) OS=Homo sapiens GN=G6PD PE=2 SV=1 - (Q2Q9B7_HUMAN)
Q6ZNE1	cDNA FLJ16186 fis, clone BRTHA2007060, moderately similar to EUKARYOTIC TRANSLATION INITIATION FACTOR 3 SUBUNIT 10 OS=Homo sapiens PE=2 SV=1 - (Q6ZNE1_HUMAN)
A6NHL2-2	Isoform 2 of Tubulin alpha chain-like 3 OS=Homo sapiens GN=TUBAL3 - (TBAL3_HUMAN)
P06748	Nucleophosmin OS=Homo sapiens GN=NPM1 PE=1 SV=2 - (NPM_HUMAN)
E9PKF4	Four and a half LIM domains protein 2 OS=Homo sapiens GN=FHL2 PE=4 SV=1 - (E9PKF4_HUMAN)
P62081	40S ribosomal protein S7 OS=Homo sapiens GN=RPS7 PE=1 SV=1 - (RS7_HUMAN)
F8W7K3	Spectrin alpha chain, brain OS=Homo sapiens GN=SPTAN1 PE=4 SV=1 - (F8W7K3_HUMAN)
C9JKA9	Kinesin-like protein KIF15 OS=Homo sapiens GN=KIF15 PE=4 SV=1 - (C9JKA9_HUMAN)
D3DX70	GRIP and coiled-coil domain containing 2, isoform CRA_a OS=Homo sapiens GN=GCC2 PE=4 SV=1 - (D3DX70_HUMAN)
P06753-3	Isoform 3 of Tropomyosin alpha-3 chain OS=Homo sapiens GN=TPM3 - (TPM3_HUMAN)
Q15075	Early endosome antigen 1 OS=Homo sapiens GN=EEA1 PE=1 SV=2 - (EEA1_HUMAN)
Q86UP2-3	Isoform 3 of Kinectin OS=Homo sapiens GN=KTN1 - (KTN1_HUMAN)
E9PRY8	Elongation factor 1-delta OS=Homo sapiens GN=EEF1D PE=3 SV=1 - (E9PRY8_HUMAN)
I3L3T4	TOM1-like protein 1 OS=Homo sapiens GN=TOM1L1 PE=4 SV=1 - (I3L3T4_HUMAN)
D3DTT5	TBK1 binding protein 1, isoform CRA_a OS=Homo sapiens GN=TBKBP1 PE=4 SV=1 - (D3DTT5_HUMAN)
P22626	Heterogeneous nuclear ribonucleoproteins A2/B1 OS=Homo sapiens GN=HNRNPA2B1 PE=1 SV=2 - (ROA2_HUMAN)
P67936-2	Isoform 2 of Tropomyosin alpha-4 chain OS=Homo sapiens GN=TPM4 - (TPM4_HUMAN)
Q13439-3	Isoform 3 of Golgin subfamily A member 4 OS=Homo sapiens GN=GOLGA4 - (GOGA4_HUMAN)
F5H3I4	Alpha-centractin OS=Homo sapiens GN=ACTR1A PE=3 SV=1 - (F5H3I4_HUMAN)
E7EV56	Pericentriolar material 1 protein OS=Homo sapiens GN=PCM1 PE=4 SV=1 - (E7EV56_HUMAN)
Q6ZRS2-3	Isoform 3 of Helicase SRCAP OS=Homo sapiens GN=SRCAP - (SRCAP_HUMAN)
F8VXY0	Heterogeneous nuclear ribonucleoprotein A1 OS=Homo sapiens GN=HNRNPA1 PE=4 SV=1 - (F8VXY0_HUMAN)
B2R9K8	cDNA, FLJ94440, highly similar to Homo sapiens chaperonin containing TCP1, subunit 6A (zeta 1)(CCT6A), mRNA OS=Homo sapiens PE=2 SV=1 - (B2R9K8_HUMAN)
F8WAQ9	Microtubule-associated serine/threonine-protein kinase 4 OS=Homo sapiens GN=MAST4 PE=4 SV=1 - (F8WAQ9_HUMAN)
P02461	Collagen alpha-1(III) chain OS=Homo sapiens GN=COL3A1 PE=1 SV=4 - (CO3A1_HUMAN)
P11532-4	Isoform 3 of Dystrophin OS=Homo sapiens GN=DMD - (DMD_HUMAN)
B3KTP9	cDNA FLJ38578 fis, clone HCHON2007674, highly similar to NUCLEOLIN OS=Homo sapiens PE=2 SV=1 - (B3KTP9_HUMAN)
A4D1E4	A kinase (PRKA) anchor protein (Yotiao) 9 OS=Homo sapiens GN=AKAP9 PE=4 SV=1 - (A4D1E4_HUMAN)
Q9P2P6	StAR-related lipid transfer protein 9 OS=Homo sapiens GN=STARD9 PE=1 SV=3 - (STAR9_HUMAN)
Q8NGR6	Olfactory receptor 1B1 OS=Homo sapiens GN=OR1B1 PE=2 SV=2 - (OR1B1_HUMAN)
B2RUT2	Thyroid hormone receptor interactor 11 OS=Homo sapiens GN=TRIP11 PE=2 SV=1 - (B2RUT2_HUMAN)
Q9BV73-2	Isoform 2 of Centrosome-associated protein CEP250 OS=Homo sapiens GN=CEP250 - (CP250_HUMAN)
Q8IVF2-3	Isoform 3 of Protein AHNAK2 OS=Homo sapiens GN=AHNAK2 - (AHNK2_HUMAN)
Q8N9W4	Golgin subfamily A member 6-like protein 2 OS=Homo sapiens GN=GOLGA6L2 PE=2 SV=2 - (GG6L2_HUMAN)
F4MH43	Ubiquitously transcribed tetratricopeptide repeat protein Y-linked transcript variant 4 OS=Homo sapiens GN=UTY PE=2 SV=1 - (F4MH43_HUMAN)
P61204	ADP-ribosylation factor 3 OS=Homo sapiens GN=ARF3 PE=1 SV=2 - (ARF3_HUMAN)
P01011	Alpha-1-antichymotrypsin OS=Homo sapiens GN=SERPINA3 PE=1 SV=2 - (AACT_HUMAN)
Q4L180-3	Isoform 3 of Filamin A-interacting protein 1-like OS=Homo sapiens GN=FILIP1L - (FIL1L_HUMAN)
B2R643	cDNA, FLJ92777, highly similar to Homo sapiens myosin IA (MYO1A), mRNA OS=Homo sapiens PE=2 SV=1 - (B2R643_HUMAN)
H0YC11	Alpha-1,3-mannosyl-glycoprotein 4-beta-N-acetylglucosaminyltransferase B (Fragment) OS=Homo sapiens GN=MGAT4B PE=4 SV=1 - (H0YC11_HUMAN)
Q9UIF8-4	Isoform 4 of Bromodomain adjacent to zinc finger domain protein 2B OS=Homo sapiens GN=BAZ2B - (BAZ2B_HUMAN)
H7C181	Sialic acid-binding Ig-like lectin 11 (Fragment) OS=Homo sapiens GN=SIGLEC11 PE=4 SV=1 - (H7C181_HUMAN)
B4DQY1	cDNA FLJ56133, highly similar to Serine/threonine-protein phosphatase 2A 65 kDa regulatory subunit A alpha isoform OS=Homo sapiens PE=2 SV=1 - (B4DQY1_HUMAN)
Q9UFD9	RIMS-binding protein 3A OS=Homo sapiens GN=RIMBP3 PE=1 SV=4 - (RIM3A_HUMAN)
E9PL22	Hypoxia up-regulated protein 1 OS=Homo sapiens GN=HYOU1 PE=3 SV=1 - (E9PL22_HUMAN)
Q8NHW5	60S acidic ribosomal protein P0-like OS=Homo sapiens GN=RPLP0P6 PE=5 SV=1 - (RLA0L_HUMAN)
P83731	60S ribosomal protein L24 OS=Homo sapiens GN=RPL24 PE=1 SV=1 - (RL24_HUMAN)
Q96L96	Alpha-protein kinase 3 OS=Homo sapiens GN=ALPK3 PE=2 SV=2 - (ALPK3_HUMAN)
P30622-1	Isoform 2 of CAP-Gly domain-containing linker protein 1 OS=Homo sapiens GN=CLIP1 - (CLIP1_HUMAN)
Q96F88	Processing of 1, ribonuclease P/MRP subunit (S. cerevisiae) OS=Homo sapiens GN=POP1 PE=2 SV=1 - (Q96F88_HUMAN)
Q96GM5-2	Isoform 2 of SWI/SNF-related matrix-associated actin-dependent regulator of chromatin subfamily D member 1 OS=Homo sapiens GN=SMARCD1 - (SMRD1_HUMAN)
Q5BJF6-3	Isoform 3 of Outer dense fiber protein 2 OS=Homo sapiens GN=ODF2 - (ODFP2_HUMAN)
P11055	Myosin-3 OS=Homo sapiens GN=MYH3 PE=1 SV=3 - (MYH3_HUMAN)
O15231-3	Isoform 3 of Zinc finger protein 185 OS=Homo sapiens GN=ZNF185 - (ZN185_HUMAN)
Q05682	Caldesmon OS=Homo sapiens GN=CALD1 PE=1 SV=3 - (CALD1_HUMAN)
Q9BYK8	Peroxisomal proliferator-activated receptor A-interacting complex 285 kDa protein OS=Homo sapiens GN=PRIC285 PE=1 SV=6 - (PR285_HUMAN)
Q5VZ66	Janus kinase and microtubule-interacting protein 3 OS=Homo sapiens GN=JAKMIP3 PE=2 SV=2 - (JKIP3_HUMAN)
Q2M329	Coiled-coil domain-containing protein 96 OS=Homo sapiens GN=CCDC96 PE=2 SV=2 - (CCD96_HUMAN)
D3DSS6	Dedicator of cytokinesis 5, isoform CRA_a OS=Homo sapiens GN=DOCK5 PE=4 SV=1 - (D3DSS6_HUMAN)
A8MY67	Serine/threonine-protein phosphatase 2A 65 kDa regulatory subunit A beta isoform OS=Homo sapiens GN=PPP2R1B PE=4 SV=2 - (A8MY67_HUMAN)
P78347-2	Isoform 2 of General transcription factor II-I OS=Homo sapiens GN=GTF2I - (GTF2I_HUMAN)
P12270	Nucleoprotein TPR OS=Homo sapiens GN=TPR PE=1 SV=3 - (TPR_HUMAN)
B3KPM8	cDNA FLJ31974 fis, clone NT2RP7008167, weakly similar to 85.1 kDa PROTEIN IN GREB-FEOA INTERGENIC REGION OS=Homo sapiens PE=2 SV=1 - (B3KPM8_HUMAN)
P07741	Adenine phosphoribosyltransferase OS=Homo sapiens GN=APRT PE=1 SV=2 - (APT_HUMAN)
Q504W7	Oculocerebrorenal syndrome of Lowe OS=Homo sapiens GN=OCRL PE=2 SV=1 - (Q504W7_HUMAN)
G3V203	60S ribosomal protein L18 OS=Homo sapiens GN=RPL18 PE=3 SV=1 - (G3V203_HUMAN)
Q3KNW1	Zinc finger protein SNAI3 OS=Homo sapiens GN=SNAI3 PE=2 SV=1 - (SNAI3_HUMAN)
Q05BA3	CCDC19 protein OS=Homo sapiens GN=CCDC19 PE=2 SV=1 - (Q05BA3_HUMAN)
P38646	Stress-70 protein, mitochondrial OS=Homo sapiens GN=HSPA9 PE=1 SV=2 - (GRP75_HUMAN)
P60953	Cell division control protein 42 homolog OS=Homo sapiens GN=CDC42 PE=1 SV=2 - (CDC42_HUMAN)
A7E2Y1	Myosin-7B OS=Homo sapiens GN=MYH7B PE=2 SV=3 - (MYH7B_HUMAN)
B3KSS3	cDNA FLJ36854 fis, clone ASTRO2014561, highly similar to Peroxisomal membrane protein 11B OS=Homo sapiens PE=2 SV=1 - (B3KSS3_HUMAN)
Q9P2K8-2	Isoform 2 of Eukaryotic translation initiation factor 2-alpha kinase 4 OS=Homo sapiens GN=EIF2AK4 - (E2AK4_HUMAN)
B7Z400	cDNA FLJ50558, highly similar to Homo sapiens microtubule-associated protein 7 (MAP7), mRNA OS=Homo sapiens PE=2 SV=1 - (B7Z400_HUMAN)
Q6NS36	Ferritin (Fragment) OS=Homo sapiens GN=FTH1 PE=2 SV=1 - (Q6NS36_HUMAN)
B4E060	Coiled-coil domain-containing protein 150 OS=Homo sapiens GN=CCDC150 PE=2 SV=1 - (B4E060_HUMAN)
F8WBW2	Coiled-coil domain-containing protein 39 OS=Homo sapiens GN=CCDC39 PE=4 SV=1 - (F8WBW2_HUMAN)
O43566-5	Isoform 3 of Regulator of G-protein signaling 14 OS=Homo sapiens GN=RGS14 - (RGS14_HUMAN)
P78371	T-complex protein 1 subunit beta OS=Homo sapiens GN=CCT2 PE=1 SV=4 - (TCPB_HUMAN)
H3BUB1	Leucine-rich repeat-containing protein 9 (Fragment) OS=Homo sapiens GN=LRRC9 PE=4 SV=1 - (H3BUB1_HUMAN)
Q5CZC0	Fibrous sheath-interacting protein 2 OS=Homo sapiens GN=FSIP2 PE=1 SV=4 - (FSIP2_HUMAN)
E7EUE6	Sodium channel protein type 3 subunit alpha (Fragment) OS=Homo sapiens GN=SCN3A PE=4 SV=1 - (E7EUE6_HUMAN)
Q8N6N2-2	Isoform 2 of Tetratricopeptide repeat protein 9B OS=Homo sapiens GN=TTC9B - (TTC9B_HUMAN)
Q8IY18	Structural maintenance of chromosomes protein 5 OS=Homo sapiens GN=SMC5 PE=1 SV=2 - (SMC5_HUMAN)
Q53FR4	Vacuolar protein sorting 35 variant (Fragment) OS=Homo sapiens PE=2 SV=1 - (Q53FR4_HUMAN)
P39019	40S ribosomal protein S19 OS=Homo sapiens GN=RPS19 PE=1 SV=2 - (RS19_HUMAN)
Q8IZX4	Transcription initiation factor TFIID subunit 1-like OS=Homo sapiens GN=TAF1L PE=1 SV=1 - (TAF1L_HUMAN)
B4DX08	cDNA FLJ58784, highly similar to T-complex protein 1 subunit epsilon OS=Homo sapiens PE=2 SV=1 - (B4DX08_HUMAN)
E9PGH8	TBC1 domain family member 1 OS=Homo sapiens GN=TBC1D1 PE=4 SV=1 - (E9PGH8_HUMAN)
Q86WN1	FCH and double SH3 domains protein 1 OS=Homo sapiens GN=FCHSD1 PE=1 SV=1 - (FCSD1_HUMAN)
Q12914	G2 (Fragment) OS=Homo sapiens PE=2 SV=1 - (Q12914_HUMAN)
B4DZQ5	cDNA FLJ51417, highly similar to Serine/threonine-protein kinase PRP4 homolog (EC 2.7.11.1) OS=Homo sapiens PE=2 SV=1 - (B4DZQ5_HUMAN)
Q07157-2	Isoform Short of Tight junction protein ZO-1 OS=Homo sapiens GN=TJP1 - (ZO1_HUMAN)
P20930	Filaggrin OS=Homo sapiens GN=FLG PE=1 SV=3 - (FILA_HUMAN)
B4DFZ6	cDNA FLJ51823, highly similar to Homo sapiens BRF2, subunit of RNA polymerase III transcription initiation factor, BRF1-like (BRF2), mRNA OS=Homo sapiens PE=2 SV=1 - (B4DFZ6_HUMAN)
Q9H1R3	Myosin light chain kinase 2, skeletal/cardiac muscle OS=Homo sapiens GN=MYLK2 PE=1 SV=3 - (MYLK2_HUMAN)
Q14004	Cyclin-dependent kinase 13 OS=Homo sapiens GN=CDK13 PE=1 SV=2 - (CDK13_HUMAN)
P32456	Interferon-induced guanylate-binding protein 2 OS=Homo sapiens GN=GBP2 PE=2 SV=3 - (GBP2_HUMAN)
A6H8W8	Intersectin 2 OS=Homo sapiens GN=ITSN2 PE=2 SV=1 - (A6H8W8_HUMAN)
B4DJ75	Serine/threonine-protein phosphatase OS=Homo sapiens PE=2 SV=1 - (B4DJ75_HUMAN)
P51648	Fatty aldehyde dehydrogenase OS=Homo sapiens GN=ALDH3A2 PE=1 SV=1 - (AL3A2_HUMAN)
B4DJ30	cDNA FLJ61290, highly similar to Neutral alpha-glucosidase AB OS=Homo sapiens PE=2 SV=1 - (B4DJ30_HUMAN)
O15085-2	Isoform 2 of Rho guanine nucleotide exchange factor 11 OS=Homo sapiens GN=ARHGEF11 - (ARHGB_HUMAN)
H0Y8G5	Heterogeneous nuclear ribonucleoprotein D0 (Fragment) OS=Homo sapiens GN=HNRNPD PE=4 SV=1 - (H0Y8G5_HUMAN)
Q96L19	L-lactate dehydrogenase OS=Homo sapiens GN=LDHAL6A PE=2 SV=1 - (Q96L19_HUMAN)
B4DTQ2	cDNA FLJ56889, moderately similar to Vigilin OS=Homo sapiens PE=2 SV=1 - (B4DTQ2_HUMAN)
C9JYT9	Vacuolar protein sorting-associated protein 37D OS=Homo sapiens GN=VPS37D PE=4 SV=1 - (C9JYT9_HUMAN)
Q8IVI3	Macrophage migration inhibitory factor homolog/MIF homolog (Fragment) OS=Homo sapiens PE=2 SV=1 - (Q8IVI3_HUMAN)
Q9HCD5	Nuclear receptor coactivator 5 OS=Homo sapiens GN=NCOA5 PE=1 SV=2 - (NCOA5_HUMAN)
Q5JPF3	Ankyrin repeat domain-containing protein 36C OS=Homo sapiens GN=ANKRD36C PE=2 SV=3 - (AN36C_HUMAN)
B7ZAX3	cDNA, FLJ79337, weakly similar to Homo sapiens phosphodiesterase 4D interacting protein, transcript variant 1, mRNA OS=Homo sapiens PE=2 SV=1 - (B7ZAX3_HUMAN)
D3DPK5	SH3 domain binding glutamic acid-rich protein like 3, isoform CRA_a (Fragment) OS=Homo sapiens GN=SH3BGRL3 PE=4 SV=1 - (D3DPK5_HUMAN)
Q15911	Zinc finger homeobox protein 3 OS=Homo sapiens GN=ZFHX3 PE=1 SV=2 - (ZFHX3_HUMAN)
Q4LE33	TNC variant protein (Fragment) OS=Homo sapiens GN=TNC variant protein PE=2 SV=1 - (Q4LE33_HUMAN)
A8K8P3-2	Isoform 2 of Protein SFI1 homolog OS=Homo sapiens GN=SFI1 - (SFI1_HUMAN)
B7Z3K3	cDNA FLJ57998, highly similar to Homo sapiens myo-inositol 1-phosphate synthase A1 (ISYNA1), mRNA OS=Homo sapiens PE=2 SV=1 - (B7Z3K3_HUMAN)
P21964-2	Isoform Soluble of Catechol O-methyltransferase OS=Homo sapiens GN=COMT - (COMT_HUMAN)
Q6URC4	Diaphanous 1 OS=Homo sapiens PE=2 SV=1 - (Q6URC4_HUMAN)
D3DTX7	Collagen, type I, alpha 1, isoform CRA_a OS=Homo sapiens GN=COL1A1 PE=4 SV=1 - (D3DTX7_HUMAN)
A8K8U1	cDNA FLJ77762, highly similar to Homo sapiens cullin-associated and neddylation-dissociated 1 (CAND1), mRNA OS=Homo sapiens PE=2 SV=1 - (A8K8U1_HUMAN)
Q8N879	cDNA FLJ39859 fis, clone SPLEN2015094, moderately similar to Mus musculus histone deacetylase mHDA1 mRNA OS=Homo sapiens PE=2 SV=1 - (Q8N879_HUMAN)
E9PK47	Phosphorylase OS=Homo sapiens GN=PYGL PE=3 SV=1 - (E9PK47_HUMAN)
Q9BRZ2	E3 ubiquitin-protein ligase TRIM56 OS=Homo sapiens GN=TRIM56 PE=1 SV=3 - (TRI56_HUMAN)
P11137-3	Isoform 3 of Microtubule-associated protein 2 OS=Homo sapiens GN=MAP2 - (MAP2_HUMAN)
B3KVR7	Serine-arginine repressor protein (35 kDa), isoform CRA_b OS=Homo sapiens GN=SRrp35 PE=2 SV=1 - (B3KVR7_HUMAN)
B0I1P5	Kidney ankyrin repeat-containing protein 3 OS=Homo sapiens GN=Kank3 PE=2 SV=1 - (B0I1P5_HUMAN)
B5MEH6	Zinc finger RNA-binding protein OS=Homo sapiens GN=ZFR PE=4 SV=1 - (B5MEH6_HUMAN)
Q9NT22	EMILIN-3 OS=Homo sapiens GN=EMILIN3 PE=2 SV=2 - (EMIL3_HUMAN)
Q14517	Protocadherin Fat 1 OS=Homo sapiens GN=FAT1 PE=1 SV=2 - (FAT1_HUMAN)
Q9H9Q2-2	Isoform 2 of COP9 signalosome complex subunit 7b OS=Homo sapiens GN=COPS7B - (CSN7B_HUMAN)
Q12955	Ankyrin-3 OS=Homo sapiens GN=ANK3 PE=1 SV=3 - (ANK3_HUMAN)
Q9UI15	Transgelin-3 OS=Homo sapiens GN=TAGLN3 PE=1 SV=2 - (TAGL3_HUMAN)
Q6P1N0-2	Isoform 2 of Coiled-coil and C2 domain-containing protein 1A OS=Homo sapiens GN=CC2D1A - (C2D1A_HUMAN)
Q9ULZ3-3	Isoform 3 of Apoptosis-associated speck-like protein containing a CARD OS=Homo sapiens GN=PYCARD - (ASC_HUMAN)
B4E2U0	6-phosphogluconate dehydrogenase, decarboxylating OS=Homo sapiens PE=2 SV=1 - (B4E2U0_HUMAN)
Q6P0Q8	Microtubule-associated serine/threonine-protein kinase 2 OS=Homo sapiens GN=MAST2 PE=1 SV=2 - (MAST2_HUMAN)
Q96MK8	cDNA FLJ32212 fis, clone PLACE6003399, weakly similar to SPIDROIN 1 OS=Homo sapiens PE=1 SV=1 - (Q96MK8_HUMAN)
C3UMV2	EWSR1/NFATC2 fusion protein OS=Homo sapiens PE=2 SV=1 - (C3UMV2_HUMAN)
F5H3P5	Actin-related protein 3 OS=Homo sapiens GN=ACTR3 PE=3 SV=1 - (F5H3P5_HUMAN)
O75367-3	Isoform 3 of Core histone macro-H2A.1 OS=Homo sapiens GN=H2AFY - (H2AY_HUMAN)
Q5VUA4	Zinc finger protein 318 OS=Homo sapiens GN=ZNF318 PE=1 SV=2 - (ZN318_HUMAN)
B4E363	Phenylalanine--tRNA ligase alpha subunit OS=Homo sapiens GN=FARSA PE=2 SV=1 - (B4E363_HUMAN)
P51149	Ras-related protein Rab-7a OS=Homo sapiens GN=RAB7A PE=1 SV=1 - (RAB7A_HUMAN)
P49748	Very long-chain specific acyl-CoA dehydrogenase, mitochondrial OS=Homo sapiens GN=ACADVL PE=1 SV=1 - (ACADV_HUMAN)
E7EU78	NF-kappa-B essential modulator OS=Homo sapiens GN=IKBKG PE=4 SV=1 - (E7EU78_HUMAN)
Q9Y333	U6 snRNA-associated Sm-like protein LSm2 OS=Homo sapiens GN=LSM2 PE=1 SV=1 - (LSM2_HUMAN)
Q92878-3	Isoform 3 of DNA repair protein RAD50 OS=Homo sapiens GN=RAD50 - (RAD50_HUMAN)
Q5VTQ0-5	Isoform 5 of Tetratricopeptide repeat protein 39B OS=Homo sapiens GN=TTC39B - (TT39B_HUMAN)
H3BQH0	Calponin-2 (Fragment) OS=Homo sapiens GN=CNN2 PE=4 SV=1 - (H3BQH0_HUMAN)
Q92786	Prospero homeobox protein 1 OS=Homo sapiens GN=PROX1 PE=1 SV=2 - (PROX1_HUMAN)
Q13459	Unconventional myosin-IXb OS=Homo sapiens GN=MYO9B PE=1 SV=3 - (MYO9B_HUMAN)
D6R922	WD repeat-containing protein 36 OS=Homo sapiens GN=WDR36 PE=4 SV=1 - (D6R922_HUMAN)
B4DU23	cDNA FLJ53488, highly similar to Homo sapiens leucine-rich repeats and calponin homology (CH) domain containing 3 (LRCH3), mRNA OS=Homo sapiens PE=2 SV=1 - (B4DU23_HUMAN)
B3GQS7	Mitochondrial heat shock 60kD protein 1 variant 1 OS=Homo sapiens GN=HSPD1 PE=2 SV=1 - (B3GQS7_HUMAN)
Q4ZFX3	Putative uncharacterized protein VSNL1 (Fragment) OS=Homo sapiens GN=VSNL1 PE=2 SV=1 - (Q4ZFX3_HUMAN)
B7Z6M1	Plastin-3 OS=Homo sapiens GN=PLS3 PE=2 SV=1 - (B7Z6M1_HUMAN)
Q2TAC6	Kinesin-like protein KIF19 OS=Homo sapiens GN=KIF19 PE=2 SV=2 - (KIF19_HUMAN)
Q8NDH2	Coiled-coil domain-containing protein 168 OS=Homo sapiens GN=CCDC168 PE=2 SV=2 - (CC168_HUMAN)
A5YM50	RAB11B protein OS=Homo sapiens GN=RAB11B PE=2 SV=1 - (A5YM50_HUMAN)
P62318	Small nuclear ribonucleoprotein Sm D3 OS=Homo sapiens GN=SNRPD3 PE=1 SV=1 - (SMD3_HUMAN)
D3DPC4	Putative uncharacterized protein FLJ21901 OS=Homo sapiens GN=FLJ21901 PE=4 SV=1 - (D3DPC4_HUMAN)
C9JX84	Inter-alpha-trypsin inhibitor heavy chain H3 OS=Homo sapiens GN=ITIH3 PE=4 SV=2 - (C9JX84_HUMAN)
E9PGD1	Homocysteine-responsive endoplasmic reticulum-resident ubiquitin-like domain member 1 protein OS=Homo sapiens GN=HERPUD1 PE=4 SV=1 - (E9PGD1_HUMAN)
B4DTU7	cDNA FLJ52076, highly similar to Homo sapiens aldehyde dehydrogenase 1 family, member L2 (ALDH1L2), mRNA OS=Homo sapiens PE=2 SV=1 - (B4DTU7_HUMAN)
Q6FIG4	RAB1B protein OS=Homo sapiens GN=RAB1B PE=2 SV=1 - (Q6FIG4_HUMAN)
Q53H10	Postreplication repair protein hRAD18p variant (Fragment) OS=Homo sapiens PE=2 SV=1 - (Q53H10_HUMAN)
O14981	TATA-binding protein-associated factor 172 OS=Homo sapiens GN=BTAF1 PE=1 SV=2 - (BTAF1_HUMAN)
H0Y7A7	Calmodulin (Fragment) OS=Homo sapiens GN=CALM2 PE=4 SV=1 - (H0Y7A7_HUMAN)
B3KUD4	cDNA FLJ39594 fis, clone SKNSH2001875, highly similar to Ribonuclease H1 (EC 3.1.26.4) OS=Homo sapiens PE=2 SV=1 - (B3KUD4_HUMAN)
O60716-8	Isoform 1 of Catenin delta-1 OS=Homo sapiens GN=CTNND1 - (CTND1_HUMAN)
Q92547	DNA topoisomerase 2-binding protein 1 OS=Homo sapiens GN=TOPBP1 PE=1 SV=3 - (TOPB1_HUMAN)
Q86US8	Telomerase-binding protein EST1A OS=Homo sapiens GN=SMG6 PE=1 SV=2 - (EST1A_HUMAN)
B4DRH6	cDNA FLJ54509, highly similar to Trifunctional enzyme subunit alpha, mitochondrial OS=Homo sapiens PE=2 SV=1 - (B4DRH6_HUMAN)
Q14240-2	Isoform 2 of Eukaryotic initiation factor 4A-II OS=Homo sapiens GN=EIF4A2 - (IF4A2_HUMAN)
P54136	Arginine--tRNA ligase, cytoplasmic OS=Homo sapiens GN=RARS PE=1 SV=2 - (SYRC_HUMAN)
E7EQE3	Telomerase protein component 1 OS=Homo sapiens GN=TEP1 PE=4 SV=1 - (E7EQE3_HUMAN)
C9JBW3	Protein sidekick-2 OS=Homo sapiens GN=SDK2 PE=4 SV=1 - (C9JBW3_HUMAN)
C1PHA2	Tyrosine-protein kinase receptor OS=Homo sapiens GN=KIF5B-ALK PE=2 SV=1 - (C1PHA2_HUMAN)
Q9NY30	Protein BTG4 OS=Homo sapiens GN=BTG4 PE=2 SV=1 - (BTG4_HUMAN)
B7Z8H0	cDNA FLJ61742, highly similar to Origin recognition complex subunit 1 OS=Homo sapiens PE=2 SV=1 - (B7Z8H0_HUMAN)
Q5TCK5	Progestin and adipoQ receptor family member 6 (Fragment) OS=Homo sapiens GN=PAQR6 PE=2 SV=1 - (Q5TCK5_HUMAN)
Q5VWI1	Transcription elongation regulator 1-like protein OS=Homo sapiens GN=TCERG1L PE=2 SV=2 - (TCRGL_HUMAN)
P14406	Cytochrome c oxidase subunit 7A2, mitochondrial OS=Homo sapiens GN=COX7A2 PE=1 SV=1 - (CX7A2_HUMAN)
B2R6V2	cDNA, FLJ93132, highly similar to Homo sapiens CDC7 cell division cycle 7 (S. cerevisiae) (CDC7), mRNA OS=Homo sapiens PE=2 SV=1 - (B2R6V2_HUMAN)
Q9BZJ0	Crooked neck-like protein 1 OS=Homo sapiens GN=CRNKL1 PE=1 SV=4 - (CRNL1_HUMAN)
Q9BQS8	FYVE and coiled-coil domain-containing protein 1 OS=Homo sapiens GN=FYCO1 PE=1 SV=3 - (FYCO1_HUMAN)
Q5JS13-2	Isoform 2 of Ras-specific guanine nucleotide-releasing factor RalGPS1 OS=Homo sapiens GN=RALGPS1 - (RGPS1_HUMAN)
Q9P2E9-2	Isoform 1 of Ribosome-binding protein 1 OS=Homo sapiens GN=RRBP1 - (RRBP1_HUMAN)
Q04637-5	Isoform D of Eukaryotic translation initiation factor 4 gamma 1 OS=Homo sapiens GN=EIF4G1 - (IF4G1_HUMAN)
D6RAN4	60S ribosomal protein L9 (Fragment) OS=Homo sapiens GN=RPL9 PE=4 SV=1 - (D6RAN4_HUMAN)
Q96L93-6	Isoform 5 of Kinesin-like protein KIF16B OS=Homo sapiens GN=KIF16B - (KI16B_HUMAN)
Q8TB01	Similar to cytoskeleton-associated protein 4 (Fragment) OS=Homo sapiens PE=2 SV=1 - (Q8TB01_HUMAN)
Q02543	60S ribosomal protein L18a OS=Homo sapiens GN=RPL18A PE=1 SV=2 - (RL18A_HUMAN)
B4E1G6	Galactokinase 1 OS=Homo sapiens GN=GALK1 PE=2 SV=1 - (B4E1G6_HUMAN)
A8K7B1	cDNA FLJ78384, highly similar to Homo sapiens RUN and FYVE domain containing 1 (RUFY1), mRNA OS=Homo sapiens PE=2 SV=1 - (A8K7B1_HUMAN)
D3DNE5	DAZ interacting protein 1-like, isoform CRA_c OS=Homo sapiens GN=DZIP1L PE=4 SV=1 - (D3DNE5_HUMAN)
Q12915	Ibd1 protein (Fragment) OS=Homo sapiens GN=Ibd1 PE=2 SV=1 - (Q12915_HUMAN)
P49770	Translation initiation factor eIF-2B subunit beta OS=Homo sapiens GN=EIF2B2 PE=1 SV=3 - (EI2BB_HUMAN)
P09429	High mobility group protein B1 OS=Homo sapiens GN=HMGB1 PE=1 SV=3 - (HMGB1_HUMAN)
B3KTC3	cDNA FLJ38059 fis, clone CTONG2014946, highly similar to Gigaxonin OS=Homo sapiens PE=2 SV=1 - (B3KTC3_HUMAN)
Q5T2S8	Armadillo repeat-containing protein 4 OS=Homo sapiens GN=ARMC4 PE=2 SV=1 - (ARMC4_HUMAN)
B4DE02	Annexin OS=Homo sapiens PE=2 SV=1 - (B4DE02_HUMAN)
P62244	40S ribosomal protein S15a OS=Homo sapiens GN=RPS15A PE=1 SV=2 - (RS15A_HUMAN)
B4DUI2	cDNA FLJ61415, highly similar to Protein kinase C and casein kinase substratein neurons protein 3 OS=Homo sapiens PE=2 SV=1 - (B4DUI2_HUMAN)
H0YJD6	Dynein light chain 1, axonemal (Fragment) OS=Homo sapiens GN=DNAL1 PE=4 SV=1 - (H0YJD6_HUMAN)
Q8WUF5	RelA-associated inhibitor OS=Homo sapiens GN=PPP1R13L PE=1 SV=4 - (IASPP_HUMAN)
Q8IVG5	Sterile alpha motif domain-containing protein 9-like OS=Homo sapiens GN=SAMD9L PE=1 SV=2 - (SAM9L_HUMAN)
Q53SY7	Putative uncharacterized protein CAD (Fragment) OS=Homo sapiens GN=CAD PE=2 SV=1 - (Q53SY7_HUMAN)
Q6NZ52	Ribosomal protein L27a OS=Homo sapiens GN=RPL27A PE=2 SV=1 - (Q6NZ52_HUMAN)
P16144-3	Isoform Beta-4B of Integrin beta-4 OS=Homo sapiens GN=ITGB4 - (ITB4_HUMAN)
O60447	Ecotropic viral integration site 5 protein homolog OS=Homo sapiens GN=EVI5 PE=1 SV=3 - (EVI5_HUMAN)
Q2TU64	PIG48 OS=Homo sapiens PE=2 SV=1 - (Q2TU64_HUMAN)
Q14126	Desmoglein-2 OS=Homo sapiens GN=DSG2 PE=1 SV=2 - (DSG2_HUMAN)
B7ZLX0	ITGAV protein OS=Homo sapiens GN=ITGAV PE=2 SV=1 - (B7ZLX0_HUMAN)
F5H2P0	Tetraspanin-11 OS=Homo sapiens GN=TSPAN11 PE=4 SV=1 - (F5H2P0_HUMAN)
Q0VGD6	HNRPR protein (Fragment) OS=Homo sapiens GN=HNRPR PE=2 SV=1 - (Q0VGD6_HUMAN)
Q9P241-2	Isoform 2 of Probable phospholipid-transporting ATPase VD OS=Homo sapiens GN=ATP10D - (AT10D_HUMAN)
B4DH02	cDNA FLJ50510, highly similar to Heat shock 70 kDa protein 4 OS=Homo sapiens PE=2 SV=1 - (B4DH02_HUMAN)
Q92887	Canalicular multispecific organic anion transporter 1 OS=Homo sapiens GN=ABCC2 PE=1 SV=3 - (MRP2_HUMAN)
B2R5M8	Isocitrate dehydrogenase (NADP) OS=Homo sapiens PE=2 SV=1 - (B2R5M8_HUMAN)
F8W6N8	Tripartite motif-containing protein 5 OS=Homo sapiens GN=TRIM5 PE=4 SV=1 - (F8W6N8_HUMAN)
O95036	Similar to 60S ribosomal protein L7; similar to P18124 (PID:d133021) OS=Homo sapiens GN=WUGSC:H_RG054D04.1 PE=2 SV=1 - (O95036_HUMAN)
C9J9U2	Protein PRRC2A OS=Homo sapiens GN=PRRC2A PE=4 SV=1 - (C9J9U2_HUMAN)
B3KM62	cDNA FLJ10371 fis, clone NT2RM2001903, highly similar to Homo sapiens ubiquitin protein ligase E3 component n-recognin 4 (UBR4), mRNA OS=Homo sapiens PE=2 SV=1 - (B3KM62_HUMAN)
P11216	Glycogen phosphorylase, brain form OS=Homo sapiens GN=PYGB PE=1 SV=5 - (PYGB_HUMAN)
B4DL79	Kinesin-like protein KIF20A OS=Homo sapiens GN=KIF20A PE=2 SV=1 - (B4DL79_HUMAN)
Q96AW5	QARS protein (Fragment) OS=Homo sapiens GN=QARS PE=2 SV=2 - (Q96AW5_HUMAN)
E9PKU4	60S ribosomal protein L8 (Fragment) OS=Homo sapiens GN=RPL8 PE=4 SV=1 - (E9PKU4_HUMAN)
P49720	Proteasome subunit beta type-3 OS=Homo sapiens GN=PSMB3 PE=1 SV=2 - (PSB3_HUMAN)
Q2TU89	Aging-associated protein 1 OS=Homo sapiens GN=AAG1 PE=2 SV=1 - (Q2TU89_HUMAN)
Q6EKJ0	General transcription factor II-I repeat domain-containing protein 2B OS=Homo sapiens GN=GTF2IRD2B PE=1 SV=1 - (GTD2B_HUMAN)
A8K9K1	cDNA FLJ77199, highly similar to Homo sapiens ATPase, Ca++ transporting, ubiquitous (ATP2A3), transcript variant 6, mRNA OS=Homo sapiens PE=2 SV=1 - (A8K9K1_HUMAN)
H0UI49	Laminin, alpha 4, isoform CRA_b OS=Homo sapiens GN=LAMA4 PE=4 SV=1 - (H0UI49_HUMAN)
B9EGR7	Rho guanine nucleotide exchange factor (GEF) 5 OS=Homo sapiens GN=ARHGEF5 PE=2 SV=1 - (B9EGR7_HUMAN)
Q2L696	Nucb2 splice variant OS=Homo sapiens GN=Nucb2 PE=2 SV=1 - (Q2L696_HUMAN)
Q15614	Olfactory receptor 8G2 OS=Homo sapiens GN=OR8G2 PE=2 SV=2 - (OR8G2_HUMAN)
Q9Y543-2	Isoform 2 of Transcription factor HES-2 OS=Homo sapiens GN=HES2 - (HES2_HUMAN)
P62333	26S protease regulatory subunit 10B OS=Homo sapiens GN=PSMC6 PE=1 SV=1 - (PRS10_HUMAN)
F5H7J5	Regulatory-associated protein of mTOR OS=Homo sapiens GN=RPTOR PE=4 SV=1 - (F5H7J5_HUMAN)
E9PP21	Cysteine and glycine-rich protein 1 OS=Homo sapiens GN=CSRP1 PE=4 SV=1 - (E9PP21_HUMAN)
Q59EK6	TNF receptor-associated protein 1 variant (Fragment) OS=Homo sapiens PE=2 SV=1 - (Q59EK6_HUMAN)
O95461-2	Isoform 2 of Glycosyltransferase-like protein LARGE1 OS=Homo sapiens GN=LARGE - (LARGE_HUMAN)
Q6P4A8	Phospholipase B-like 1 OS=Homo sapiens GN=PLBD1 PE=1 SV=2 - (PLBL1_HUMAN)
Q53H42	Syndecan 1 variant (Fragment) OS=Homo sapiens PE=2 SV=1 - (Q53H42_HUMAN)
Q2TU34	Fructose-1,6-bisphosphatase 1 OS=Homo sapiens GN=FBP1 PE=2 SV=1 - (Q2TU34_HUMAN)
B4E241	Serine/arginine-rich-splicing factor 3 OS=Homo sapiens GN=SRSF3 PE=2 SV=1 - (B4E241_HUMAN)
B4DNH0	cDNA FLJ60313, highly similar to Epithelial-cadherin OS=Homo sapiens PE=2 SV=1 - (B4DNH0_HUMAN)
B2RDN9	cDNA, FLJ96699, highly similar to Homo sapiens thyroid autoantigen 70kDa (Ku antigen) (G22P1), mRNA OS=Homo sapiens PE=2 SV=1 - (B2RDN9_HUMAN)
Q2VIN3	RBM1 (Fragment) OS=Homo sapiens PE=4 SV=1 - (Q2VIN3_HUMAN)
P07384	Calpain-1 catalytic subunit OS=Homo sapiens GN=CAPN1 PE=1 SV=1 - (CAN1_HUMAN)
B4DDL4	cDNA FLJ53966, moderately similar to Homo sapiens cytidylate kinase (CMPK), mRNA OS=Homo sapiens PE=2 SV=1 - (B4DDL4_HUMAN)
Q8TEK3	Histone-lysine N-methyltransferase, H3 lysine-79 specific OS=Homo sapiens GN=DOT1L PE=1 SV=2 - (DOT1L_HUMAN)
P54253	Ataxin-1 OS=Homo sapiens GN=ATXN1 PE=1 SV=2 - (ATX1_HUMAN)
H7C3X0	Protein O-GlcNAcase (Fragment) OS=Homo sapiens GN=MGEA5 PE=4 SV=1 - (H7C3X0_HUMAN)
E7EQT4	Apoptotic chromatin condensation inducer in the nucleus OS=Homo sapiens GN=ACIN1 PE=4 SV=2 - (E7EQT4_HUMAN)
B7Z1H4	cDNA FLJ56052, highly similar to Puromycin-sensitive aminopeptidase (EC 3.4.11.-) OS=Homo sapiens PE=2 SV=1 - (B7Z1H4_HUMAN)
Q5VVM3	Phosphopantothenate--cysteine ligase OS=Homo sapiens GN=PPCS PE=2 SV=1 - (Q5VVM3_HUMAN)
O95714	E3 ubiquitin-protein ligase HERC2 OS=Homo sapiens GN=HERC2 PE=1 SV=2 - (HERC2_HUMAN)
Q5R207	Carbamoylphosphate synthetase I OS=Homo sapiens GN=CPS1 PE=2 SV=1 - (Q5R207_HUMAN)
Q6P084	MAP3K2 protein (Fragment) OS=Homo sapiens GN=MAP3K2 PE=2 SV=1 - (Q6P084_HUMAN)
P20742-2	Isoform 2 of Pregnancy zone protein OS=Homo sapiens GN=PZP - (PZP_HUMAN)
B5MCX3	Septin-2 OS=Homo sapiens GN=SEPT2 PE=3 SV=1 - (B5MCX3_HUMAN)
H7BY74	Metal transporter CNNM1 OS=Homo sapiens GN=CNNM1 PE=4 SV=1 - (H7BY74_HUMAN)
Q8WVC2	40S ribosomal protein S21 OS=Homo sapiens GN=RPS21 PE=2 SV=1 - (Q8WVC2_HUMAN)
B4DKN9	cDNA FLJ57740, highly similar to Transforming protein RhoA OS=Homo sapiens PE=2 SV=1 - (B4DKN9_HUMAN)
B3KND4	Coatomer subunit gamma OS=Homo sapiens PE=2 SV=1 - (B3KND4_HUMAN)
O95425	Supervillin OS=Homo sapiens GN=SVIL PE=1 SV=2 - (SVIL_HUMAN)
Q9Y5H1-2	Isoform 2 of Protocadherin gamma-A2 OS=Homo sapiens GN=PCDHGA2 - (PCDG2_HUMAN)
E5RFF9	DNA replication complex GINS protein SLD5 OS=Homo sapiens GN=GINS4 PE=4 SV=1 - (E5RFF9_HUMAN)
P43686-2	Isoform 2 of 26S protease regulatory subunit 6B OS=Homo sapiens GN=PSMC4 - (PRS6B_HUMAN)
P49662-2	Isoform 2 of Caspase-4 OS=Homo sapiens GN=CASP4 - (CASP4_HUMAN)
B4E2Z3	4F2 cell-surface antigen heavy chain OS=Homo sapiens GN=SLC3A2 PE=2 SV=1 - (B4E2Z3_HUMAN)
B3KQT2	cDNA PSEC0148 fis, clone PLACE1007202, highly similar to Protein disulfide-isomerase A3 (EC 5.3.4.1) OS=Homo sapiens PE=2 SV=1 - (B3KQT2_HUMAN)
E7ES33	Septin-7 OS=Homo sapiens GN=SEPT7 PE=3 SV=2 - (E7ES33_HUMAN)
O75419-2	Isoform 2 of Cell division control protein 45 homolog OS=Homo sapiens GN=CDC45 - (CDC45_HUMAN)
P00568	Adenylate kinase isoenzyme 1 OS=Homo sapiens GN=AK1 PE=1 SV=3 - (KAD1_HUMAN)
Q86V20-2	Isoform 2 of Protein FAM35A OS=Homo sapiens GN=FAM35A - (FA35A_HUMAN)
Q58FF6	Putative heat shock protein HSP 90-beta 4 OS=Homo sapiens GN=HSP90AB4P PE=5 SV=1 - (H90B4_HUMAN)
H0YN26	Acidic leucine-rich nuclear phosphoprotein 32 family member A OS=Homo sapiens GN=ANP32A PE=4 SV=1 - (H0YN26_HUMAN)
Q92621	Nuclear pore complex protein Nup205 OS=Homo sapiens GN=NUP205 PE=1 SV=3 - (NU205_HUMAN)
H7C5G6	Protein FLJ39061 (Fragment) OS=Homo sapiens GN=FLJ39061 PE=4 SV=1 - (H7C5G6_HUMAN)
Q53G71	Calreticulin variant (Fragment) OS=Homo sapiens PE=2 SV=1 - (Q53G71_HUMAN)
E7EWS7	Ubiquitin-conjugating enzyme E2 L3 OS=Homo sapiens GN=UBE2L3 PE=3 SV=1 - (E7EWS7_HUMAN)
Q9BTA9	WW domain-containing adapter protein with coiled-coil OS=Homo sapiens GN=WAC PE=1 SV=3 - (WAC_HUMAN)
P84098	60S ribosomal protein L19 OS=Homo sapiens GN=RPL19 PE=1 SV=1 - (RL19_HUMAN)
F5GXF0	Nuclear receptor subfamily 4 group A member 1 OS=Homo sapiens GN=NR4A1 PE=3 SV=1 - (F5GXF0_HUMAN)
F8W118	Nucleosome assembly protein 1-like 1 (Fragment) OS=Homo sapiens GN=NAP1L1 PE=3 SV=1 - (F8W118_HUMAN)
H0YCV9	CD44 antigen (Fragment) OS=Homo sapiens GN=CD44 PE=4 SV=1 - (H0YCV9_HUMAN)
B3KRA9	cDNA FLJ33948 fis, clone CTONG2018379, highly similar to Hexokinase-1 (EC 2.7.1.1) OS=Homo sapiens PE=2 SV=1 - (B3KRA9_HUMAN)
Q9Y4G6	Talin-2 OS=Homo sapiens GN=TLN2 PE=1 SV=4 - (TLN2_HUMAN)
D3DNV8	Leprecan-like 1, isoform CRA_b OS=Homo sapiens GN=LEPREL1 PE=4 SV=1 - (D3DNV8_HUMAN)
Q53EM5	Transketolase variant (Fragment) OS=Homo sapiens PE=2 SV=1 - (Q53EM5_HUMAN)
Q53H34	Ribosomal protein L13a variant (Fragment) OS=Homo sapiens PE=2 SV=1 - (Q53H34_HUMAN)
Q8WVM7	Cohesin subunit SA-1 OS=Homo sapiens GN=STAG1 PE=1 SV=3 - (STAG1_HUMAN)
Q0D2K2	Kelch-like protein 30 OS=Homo sapiens GN=KLHL30 PE=2 SV=3 - (KLH30_HUMAN)
B3KTJ9	cDNA FLJ38393 fis, clone FEBRA2007212 OS=Homo sapiens PE=2 SV=1 - (B3KTJ9_HUMAN)
Q5TIA1	Meiosis inhibitor protein 1 OS=Homo sapiens GN=MEI1 PE=2 SV=2 - (MEI1_HUMAN)
P42345	Serine/threonine-protein kinase mTOR OS=Homo sapiens GN=MTOR PE=1 SV=1 - (MTOR_HUMAN)
C0IMJ3	Periostin isoform thy6 OS=Homo sapiens PE=2 SV=1 - (C0IMJ3_HUMAN)
Q07954	Prolow-density lipoprotein receptor-related protein 1 OS=Homo sapiens GN=LRP1 PE=1 SV=2 - (LRP1_HUMAN)
B2R8K9	cDNA, FLJ93950, highly similar to Homo sapiens glutamyl aminopeptidase (aminopeptidase A) (ENPEP), mRNA OS=Homo sapiens PE=2 SV=1 - (B2R8K9_HUMAN)
B4E0B7	Condensin complex subunit 1 OS=Homo sapiens PE=2 SV=1 - (B4E0B7_HUMAN)
G3V129	Putative uncharacterized protein LOC138046 OS=Homo sapiens GN=RALYL PE=4 SV=1 - (G3V129_HUMAN)
E9PQV5	Coiled-coil domain-containing protein 37 OS=Homo sapiens GN=CCDC37 PE=4 SV=1 - (E9PQV5_HUMAN)
Q96CA6	ACAA1 protein OS=Homo sapiens GN=ACAA1 PE=2 SV=1 - (Q96CA6_HUMAN)
C9JAB2	Serine/arginine-rich-splicing factor 7 OS=Homo sapiens GN=SRSF7 PE=4 SV=1 - (C9JAB2_HUMAN)
Q96LX7-5	Isoform 2 of Coiled-coil domain-containing protein 17 OS=Homo sapiens GN=CCDC17 - (CCD17_HUMAN)
E9PGK7	Transient receptor potential cation channel subfamily M member 2 OS=Homo sapiens GN=TRPM2 PE=4 SV=1 - (E9PGK7_HUMAN)
B5ME19	Eukaryotic translation initiation factor 3 subunit C OS=Homo sapiens GN=EIF3CL PE=4 SV=1 - (B5ME19_HUMAN)
Q7Z4W1	L-xylulose reductase OS=Homo sapiens GN=DCXR PE=1 SV=2 - (DCXR_HUMAN)
O00533	Neural cell adhesion molecule L1-like protein OS=Homo sapiens GN=CHL1 PE=1 SV=4 - (CHL1_HUMAN)
H9E7F7	Cytochrome c oxidase subunit 2 (Fragment) OS=Homo sapiens GN=COX2 PE=3 SV=1 - (H9E7F7_HUMAN)
O76090	Bestrophin-1 OS=Homo sapiens GN=BEST1 PE=1 SV=1 - (BEST1_HUMAN)
Q96CE4	Stathmin OS=Homo sapiens GN=STMN1 PE=2 SV=1 - (Q96CE4_HUMAN)
B7ZLS9	Synaptonemal complex protein 1 OS=Homo sapiens GN=SYCP1 PE=2 SV=1 - (B7ZLS9_HUMAN)
Q9H5Y7	SLIT and NTRK-like protein 6 OS=Homo sapiens GN=SLITRK6 PE=1 SV=3 - (SLIK6_HUMAN)
Q59GS3	Proteasome 26S ATPase subunit 5 variant (Fragment) OS=Homo sapiens PE=2 SV=1 - (Q59GS3_HUMAN)
B7ZKX5	KIF6 protein OS=Homo sapiens GN=KIF6 PE=2 SV=1 - (B7ZKX5_HUMAN)
B7Z8B1	T-complex protein 1 subunit delta OS=Homo sapiens PE=2 SV=1 - (B7Z8B1_HUMAN)
Q8TE49	OTU domain-containing protein 7A OS=Homo sapiens GN=OTUD7A PE=1 SV=1 - (OTU7A_HUMAN)
Q96N16-2	Isoform 2 of Janus kinase and microtubule-interacting protein 1 OS=Homo sapiens GN=JAKMIP1 - (JKIP1_HUMAN)
P35268	60S ribosomal protein L22 OS=Homo sapiens GN=RPL22 PE=1 SV=2 - (RL22_HUMAN)
B4DP66	cDNA FLJ60755, highly similar to Leucine zipper putative tumor suppressor 2 OS=Homo sapiens PE=2 SV=1 - (B4DP66_HUMAN)
Q12789-3	Isoform 2 of General transcription factor 3C polypeptide 1 OS=Homo sapiens GN=GTF3C1 - (TF3C1_HUMAN)
P13667	Protein disulfide-isomerase A4 OS=Homo sapiens GN=PDIA4 PE=1 SV=2 - (PDIA4_HUMAN)
Q5VZB9	Doublesex- and mab-3-related transcription factor A1 OS=Homo sapiens GN=DMRTA1 PE=2 SV=1 - (DMRTA_HUMAN)
A0MZ66-2	Isoform 2 of Shootin-1 OS=Homo sapiens GN=KIAA1598 - (SHOT1_HUMAN)
B3KMT7	cDNA FLJ12561 fis, clone NT2RM4000798, highly similar to Brefeldin A-inhibited guanine nucleotide-exchange protein 2 OS=Homo sapiens PE=2 SV=1 - (B3KMT7_HUMAN)
B3KMV8	cDNA FLJ12766 fis, clone NT2RP2001520, highly similar to Calcium-binding mitochondrial carrier protein Aralar1 OS=Homo sapiens PE=2 SV=1 - (B3KMV8_HUMAN)
F5H608	ATP synthase subunit d, mitochondrial OS=Homo sapiens GN=ATP5H PE=4 SV=1 - (F5H608_HUMAN)
E7EWW0	UPF0505 protein C16orf62 OS=Homo sapiens GN=C16orf62 PE=4 SV=2 - (E7EWW0_HUMAN)
P62906	60S ribosomal protein L10a OS=Homo sapiens GN=RPL10A PE=1 SV=2 - (RL10A_HUMAN)
O75146	Huntingtin-interacting protein 1-related protein OS=Homo sapiens GN=HIP1R PE=1 SV=2 - (HIP1R_HUMAN)
B3KXC0	cDNA FLJ45113 fis, clone BRAWH3034743, weakly similar to Mus musculus microtubule-associated protein 7 (Mtap7), mRNA OS=Homo sapiens PE=2 SV=1 - (B3KXC0_HUMAN)
Q59G46	Thioredoxin-like 1 variant (Fragment) OS=Homo sapiens PE=2 SV=1 - (Q59G46_HUMAN)
H7C5W9	Sarcoplasmic/endoplasmic reticulum calcium ATPase 2 (Fragment) OS=Homo sapiens GN=ATP2A2 PE=3 SV=1 - (H7C5W9_HUMAN)
Q6UW46	VGPW2523 OS=Homo sapiens GN=UNQ2523 PE=2 SV=1 - (Q6UW46_HUMAN)
Q15572-4	Isoform 4 of TATA box-binding protein-associated factor RNA polymerase I subunit C OS=Homo sapiens GN=TAF1C - (TAF1C_HUMAN)
Q9H1H9-3	Isoform 3 of Kinesin-like protein KIF13A OS=Homo sapiens GN=KIF13A - (KI13A_HUMAN)
Q6NUK1-2	Isoform 2 of Calcium-binding mitochondrial carrier protein SCaMC-1 OS=Homo sapiens GN=SLC25A24 - (SCMC1_HUMAN)
Q96BN8	Protein FAM105B OS=Homo sapiens GN=FAM105B PE=1 SV=3 - (F105B_HUMAN)
P41250	Glycine--tRNA ligase OS=Homo sapiens GN=GARS PE=1 SV=3 - (SYG_HUMAN)
E7EWJ1	Rab11 family-interacting protein 3 OS=Homo sapiens GN=RAB11FIP3 PE=4 SV=1 - (E7EWJ1_HUMAN)
P19086	Guanine nucleotide-binding protein G(z) subunit alpha OS=Homo sapiens GN=GNAZ PE=2 SV=3 - (GNAZ_HUMAN)
H0YCI4	Nucleosome assembly protein 1-like 4 (Fragment) OS=Homo sapiens GN=NAP1L4 PE=3 SV=1 - (H0YCI4_HUMAN)
Q99598	Translin-associated protein X OS=Homo sapiens GN=TSNAX PE=1 SV=1 - (TSNAX_HUMAN)
B4DKS8	Heterogeneous nuclear ribonucleoprotein F OS=Homo sapiens GN=HNRNPF PE=2 SV=1 - (B4DKS8_HUMAN)
Q9H4L5-5	Isoform 2a of Oxysterol-binding protein-related protein 3 OS=Homo sapiens GN=OSBPL3 - (OSBL3_HUMAN)
Q0EFC6	HSR1 protein OS=Homo sapiens GN=HSR1 PE=4 SV=1 - (Q0EFC6_HUMAN)
Q9Y3B8	Oligoribonuclease, mitochondrial OS=Homo sapiens GN=REXO2 PE=1 SV=3 - (ORN_HUMAN)
Q9UHF5	Interleukin-17B OS=Homo sapiens GN=IL17B PE=2 SV=1 - (IL17B_HUMAN)
F5H018	GTP-binding nuclear protein Ran (Fragment) OS=Homo sapiens GN=RAN PE=4 SV=1 - (F5H018_HUMAN)
P61970	Nuclear transport factor 2 OS=Homo sapiens GN=NUTF2 PE=1 SV=1 - (NTF2_HUMAN)
B2R5V0	cDNA, FLJ92637, highly similar to Homo sapiens nuclear receptor coactivator 4 (NCOA4), mRNA OS=Homo sapiens PE=2 SV=1 - (B2R5V0_HUMAN)
G3V2B8	Methylenetetrahydrofolate dehydrogenase OS=Homo sapiens GN=MTHFD1 PE=3 SV=1 - (G3V2B8_HUMAN)
Q9NS13	Placenta apolipoprotein B48 receptor type 2 OS=Homo sapiens GN=APOB48R PE=2 SV=1 - (Q9NS13_HUMAN)
B1AN92	Eukaryotic translation initiation factor 4 gamma 3 (Fragment) OS=Homo sapiens GN=EIF4G3 PE=4 SV=1 - (B1AN92_HUMAN)
A6H8H3	C14orf49 protein OS=Homo sapiens GN=C14orf49 PE=2 SV=1 - (A6H8H3_HUMAN)
B4DFR2	Dynein light chain roadblock-type 1 OS=Homo sapiens GN=DYNLRB1 PE=2 SV=1 - (B4DFR2_HUMAN)
Q96P63	Serpin B12 OS=Homo sapiens GN=SERPINB12 PE=1 SV=1 - (SPB12_HUMAN)
F5H0X6	Proactivator polypeptide OS=Homo sapiens GN=PSAP PE=4 SV=1 - (F5H0X6_HUMAN)
Q9Y3A5	Ribosome maturation protein SBDS OS=Homo sapiens GN=SBDS PE=1 SV=4 - (SBDS_HUMAN)
H0Y2S5	LIM and calponin homology domains-containing protein 1 OS=Homo sapiens GN=LIMCH1 PE=4 SV=1 - (H0Y2S5_HUMAN)
Q9UJZ1	Stomatin-like protein 2 OS=Homo sapiens GN=STOML2 PE=1 SV=1 - (STML2_HUMAN)
P48637	Glutathione synthetase OS=Homo sapiens GN=GSS PE=1 SV=1 - (GSHB_HUMAN)
B4E0Z6	cDNA FLJ59809, highly similar to Bone marrow stromal antigen 2 OS=Homo sapiens PE=2 SV=1 - (B4E0Z6_HUMAN)
B7Z6D1	cDNA FLJ57430, highly similar to DNA-directed RNA polymerase II 33 kDa polypeptide (EC 2.7.7.6) OS=Homo sapiens PE=2 SV=1 - (B7Z6D1_HUMAN)
E7ERI3	Threonine--tRNA ligase, cytoplasmic OS=Homo sapiens GN=TARS PE=3 SV=1 - (E7ERI3_HUMAN)
Q9NQ38-3	Isoform long of Serine protease inhibitor Kazal-type 5 OS=Homo sapiens GN=SPINK5 - (ISK5_HUMAN)
P17900	Ganglioside GM2 activator OS=Homo sapiens GN=GM2A PE=1 SV=4 - (SAP3_HUMAN)
F5H1F8	Chloride intracellular channel protein 4 OS=Homo sapiens GN=CLIC4 PE=4 SV=1 - (F5H1F8_HUMAN)
Q8N7R1	POM121-like protein 12 OS=Homo sapiens GN=POM121L12 PE=2 SV=3 - (P1L12_HUMAN)
Q99714-2	Isoform 2 of 3-hydroxyacyl-CoA dehydrogenase type-2 OS=Homo sapiens GN=HSD17B10 - (HCD2_HUMAN)
D3DPB7	Sodium channel, voltage-gated, type IX, alpha, isoform CRA_a OS=Homo sapiens GN=SCN9A PE=3 SV=1 - (D3DPB7_HUMAN)
Q6ZMU0	Delta-aminolevulinic acid dehydratase OS=Homo sapiens PE=2 SV=1 - (Q6ZMU0_HUMAN)
Q6P5X8	DNA-directed DNA/RNA polymerase mu OS=Homo sapiens GN=POLM PE=2 SV=1 - (Q6P5X8_HUMAN)
H0YGV0	DnaJ homolog subfamily C member 4 (Fragment) OS=Homo sapiens GN=DNAJC4 PE=4 SV=1 - (H0YGV0_HUMAN)
Q2YDC2	HCG1985580, isoform CRA_f OS=Homo sapiens GN=PDCD6 PE=2 SV=1 - (Q2YDC2_HUMAN)
H0YLM9	Proline-serine-threonine phosphatase-interacting protein 1 (Fragment) OS=Homo sapiens GN=PSTPIP1 PE=4 SV=1 - (H0YLM9_HUMAN)
Q9Y305	Acyl-coenzyme A thioesterase 9, mitochondrial OS=Homo sapiens GN=ACOT9 PE=1 SV=2 - (ACOT9_HUMAN)
Q8NC51-4	Isoform 4 of Plasminogen activator inhibitor 1 RNA-binding protein OS=Homo sapiens GN=SERBP1 - (PAIRB_HUMAN)
A7E2A5	ARAP2 protein (Fragment) OS=Homo sapiens GN=ARAP2 PE=2 SV=1 - (A7E2A5_HUMAN)
Q9BVC6	Transmembrane protein 109 OS=Homo sapiens GN=TMEM109 PE=1 SV=1 - (TM109_HUMAN)
G3V5Q1	DNA-(apurinic or apyrimidinic site) lyase (Fragment) OS=Homo sapiens GN=APEX1 PE=4 SV=1 - (G3V5Q1_HUMAN)
Q6ECI4	Zinc finger protein 470 OS=Homo sapiens GN=ZNF470 PE=1 SV=3 - (ZN470_HUMAN)
C9J8H1	V-type proton ATPase subunit E 1 (Fragment) OS=Homo sapiens GN=ATP6V1E1 PE=4 SV=1 - (C9J8H1_HUMAN)
Q9C0F0	Putative Polycomb group protein ASXL3 OS=Homo sapiens GN=ASXL3 PE=2 SV=3 - (ASXL3_HUMAN)
Q6P179-3	Isoform 3 of Endoplasmic reticulum aminopeptidase 2 OS=Homo sapiens GN=ERAP2 - (ERAP2_HUMAN)
Q1JUQ5	Peptidyl-prolyl cis-trans isomerase OS=Homo sapiens GN=FKBP12-Exip2 PE=2 SV=1 - (Q1JUQ5_HUMAN)
P46778	60S ribosomal protein L21 OS=Homo sapiens GN=RPL21 PE=1 SV=2 - (RL21_HUMAN)
Q01664	Transcription factor AP-4 OS=Homo sapiens GN=TFAP4 PE=1 SV=2 - (TFAP4_HUMAN)
Q05DH1	Proteasome subunit alpha type (Fragment) OS=Homo sapiens GN=PSMA7 PE=2 SV=1 - (Q05DH1_HUMAN)
F6USW4	F-actin-capping protein subunit beta (Fragment) OS=Homo sapiens GN=CAPZB PE=4 SV=1 - (F6USW4_HUMAN)
Q6ZRK6	Coiled-coil domain-containing protein 73 OS=Homo sapiens GN=CCDC73 PE=1 SV=2 - (CCD73_HUMAN)
B3KWD0	cDNA FLJ42779 fis, clone BRAWH3005300, highly similar to Exportin-1 OS=Homo sapiens PE=2 SV=1 - (B3KWD0_HUMAN)
P23297	Protein S100-A1 OS=Homo sapiens GN=S100A1 PE=1 SV=2 - (S10A1_HUMAN)
Q5W0H4	Tumor protein, translationally-controlled 1 OS=Homo sapiens GN=TPT1 PE=2 SV=1 - (Q5W0H4_HUMAN)
Q8IZF2-3	Isoform 3 of Probable G-protein coupled receptor 116 OS=Homo sapiens GN=GPR116 - (GP116_HUMAN)
Q38G99	C-myc intron-binding protein 1 OS=Homo sapiens GN=MIBP1 PE=2 SV=1 - (Q38G99_HUMAN)
Q96E29	mTERF domain-containing protein 1, mitochondrial OS=Homo sapiens GN=MTERFD1 PE=1 SV=2 - (MTER1_HUMAN)
Q7RTM1	Otopetrin-1 OS=Homo sapiens GN=OTOP1 PE=2 SV=1 - (OTOP1_HUMAN)
Q96RP6	Ubiquitin-conjugating enzyme OS=Homo sapiens GN=PUBC1 PE=2 SV=1 - (Q96RP6_HUMAN)
Q13011	Delta(3,5)-Delta(2,4)-dienoyl-CoA isomerase, mitochondrial OS=Homo sapiens GN=ECH1 PE=1 SV=2 - (ECH1_HUMAN)
Q02156	Protein kinase C epsilon type OS=Homo sapiens GN=PRKCE PE=1 SV=1 - (KPCE_HUMAN)
Q03252	Lamin-B2 OS=Homo sapiens GN=LMNB2 PE=1 SV=3 - (LMNB2_HUMAN)
B4DTD3	cDNA FLJ50209, highly similar to DNA-repair protein XRCC1 OS=Homo sapiens PE=2 SV=1 - (B4DTD3_HUMAN)
Q86UF4	Coiled-coil domain-containing protein C1orf110 OS=Homo sapiens GN=C1orf110 PE=2 SV=2 - (CA110_HUMAN)
Q9HAU6	Putative apoptosis inhibitor FKSG2 OS=Homo sapiens GN=FKSG2 PE=5 SV=1 - (FKSG2_HUMAN)
Q49MI3-4	Isoform 4 of Ceramide kinase-like protein OS=Homo sapiens GN=CERKL - (CERKL_HUMAN)
F8WDL0	AP-1 complex subunit beta-1 OS=Homo sapiens GN=AP1B1 PE=4 SV=1 - (F8WDL0_HUMAN)
A8K202	5-aminoimidazole-4-carboxamide ribonucleotide formyltransferase/IMP cyclohydrolase, isoform CRA_g OS=Homo sapiens GN=ATIC PE=2 SV=1 - (A8K202_HUMAN)
O75410-5	Isoform 5 of Transforming acidic coiled-coil-containing protein 1 OS=Homo sapiens GN=TACC1 - (TACC1_HUMAN)
Q9UPR0	Inactive phospholipase C-like protein 2 OS=Homo sapiens GN=PLCL2 PE=1 SV=2 - (PLCL2_HUMAN)
B7ZBG9	Eukaryotic translation initiation factor 6 OS=Homo sapiens GN=EIF6 PE=3 SV=1 - (B7ZBG9_HUMAN)
Q14591-2	Isoform 2 of Zinc finger protein 271 OS=Homo sapiens GN=ZNF271 - (ZN271_HUMAN)
A8MVP6	T-cell surface glycoprotein CD3 delta chain OS=Homo sapiens GN=CD3D PE=4 SV=1 - (A8MVP6_HUMAN)
P14314-2	Isoform 2 of Glucosidase 2 subunit beta OS=Homo sapiens GN=PRKCSH - (GLU2B_HUMAN)
E9PNX0	FYVE, RhoGEF and PH domain-containing protein 4 OS=Homo sapiens GN=FGD4 PE=4 SV=1 - (E9PNX0_HUMAN)
Q5VZ89-6	Isoform 6 of DENN domain-containing protein 4C OS=Homo sapiens GN=DENND4C - (DEN4C_HUMAN)
O00425	Insulin-like growth factor 2 mRNA-binding protein 3 OS=Homo sapiens GN=IGF2BP3 PE=1 SV=2 - (IF2B3_HUMAN)
B2R806	Eukaryotic translation initiation factor 3 subunit E OS=Homo sapiens PE=2 SV=1 - (B2R806_HUMAN)
B2R8A2	cDNA, FLJ93804, highly similar to Homo sapiens gp25L2 protein (HSGP25L2G), mRNA OS=Homo sapiens PE=2 SV=1 - (B2R8A2_HUMAN)
B4DWL3	Lysosome-associated membrane glycoprotein 1 OS=Homo sapiens GN=LAMP1 PE=2 SV=1 - (B4DWL3_HUMAN)
Q59GB4	Dihydropyrimidinase-like 2 variant (Fragment) OS=Homo sapiens PE=2 SV=1 - (Q59GB4_HUMAN)
Q9P278	Folliculin-interacting protein 2 OS=Homo sapiens GN=FNIP2 PE=1 SV=2 - (FNIP2_HUMAN)
Q13185	Chromobox protein homolog 3 OS=Homo sapiens GN=CBX3 PE=1 SV=4 - (CBX3_HUMAN)
Q9H501	ESF1 homolog OS=Homo sapiens GN=ESF1 PE=1 SV=1 - (ESF1_HUMAN)
Q5H9U9	Probable ATP-dependent RNA helicase DDX60-like OS=Homo sapiens GN=DDX60L PE=2 SV=2 - (DDX6L_HUMAN)
B2RTX8	WAPAL protein OS=Homo sapiens GN=WAPAL PE=2 SV=1 - (B2RTX8_HUMAN)
Q70EL4-2	Isoform 2 of Ubiquitin carboxyl-terminal hydrolase 43 OS=Homo sapiens GN=USP43 - (UBP43_HUMAN)
Q96PU5-3	Isoform 3 of E3 ubiquitin-protein ligase NEDD4-like OS=Homo sapiens GN=NEDD4L - (NED4L_HUMAN)
P61129	Zinc finger CCCH domain-containing protein 6 OS=Homo sapiens GN=ZC3H6 PE=2 SV=2 - (ZC3H6_HUMAN)
I1SRC5	UBE2L3/KRAS fusion protein OS=Homo sapiens PE=2 SV=1 - (I1SRC5_HUMAN)
Q9UFP1	Protein FAM198A OS=Homo sapiens GN=FAM198A PE=1 SV=3 - (F198A_HUMAN)
P36405	ADP-ribosylation factor-like protein 3 OS=Homo sapiens GN=ARL3 PE=1 SV=2 - (ARL3_HUMAN)
Q56VL3	OCIA domain-containing protein 2 OS=Homo sapiens GN=OCIAD2 PE=1 SV=1 - (OCAD2_HUMAN)
O00410	Importin-5 OS=Homo sapiens GN=IPO5 PE=1 SV=4 - (IPO5_HUMAN)
Q9Y6W5	Wiskott-Aldrich syndrome protein family member 2 OS=Homo sapiens GN=WASF2 PE=1 SV=3 - (WASF2_HUMAN)
A7E261	5-oxoprolinase (ATP-hydrolysing) OS=Homo sapiens GN=OPLAH PE=2 SV=1 - (A7E261_HUMAN)
B3KPE5	cDNA FLJ31682 fis, clone NT2RI2005335, highly similar to MMS19-like protein OS=Homo sapiens PE=2 SV=1 - (B3KPE5_HUMAN)
B4DKB2	Endothelin-converting enzyme 1 OS=Homo sapiens GN=ECE1 PE=2 SV=1 - (B4DKB2_HUMAN)
G3XAD8	Stress-induced-phosphoprotein 1 OS=Homo sapiens GN=STIP1 PE=4 SV=1 - (G3XAD8_HUMAN)
Q96JD6-4	Isoform 4 of 1,5-anhydro-D-fructose reductase OS=Homo sapiens GN=AKR1E2 - (AKCL2_HUMAN)
P25788-2	Isoform 2 of Proteasome subunit alpha type-3 OS=Homo sapiens GN=PSMA3 - (PSA3_HUMAN)
Q8N0W0	Seven transmembrane helix receptor OS=Homo sapiens PE=2 SV=1 - (Q8N0W0_HUMAN)
E9PKV2	39S ribosomal protein L17, mitochondrial (Fragment) OS=Homo sapiens GN=MRPL17 PE=4 SV=1 - (E9PKV2_HUMAN)
Q5XUX1-3	Isoform 3 of F-box/WD repeat-containing protein 9 OS=Homo sapiens GN=FBXW9 - (FBXW9_HUMAN)
H7C0Q6	Tripartite motif-containing protein 4 (Fragment) OS=Homo sapiens GN=TRIM4 PE=4 SV=1 - (H7C0Q6_HUMAN)
Q8WX93	Palladin OS=Homo sapiens GN=PALLD PE=1 SV=3 - (PALLD_HUMAN)
B1Q3B3	Ferritin (Fragment) OS=Homo sapiens GN=FTL PE=3 SV=1 - (B1Q3B3_HUMAN)
B4DXN6	cDNA FLJ53461, highly similar to Eukaryotic translation initiation factor 3 subunit 9 OS=Homo sapiens PE=2 SV=1 - (B4DXN6_HUMAN)
Q5U676	B3GAT3 protein (Fragment) OS=Homo sapiens GN=B3GAT3 PE=2 SV=1 - (Q5U676_HUMAN)
O75531	Barrier-to-autointegration factor OS=Homo sapiens GN=BANF1 PE=1 SV=1 - (BAF_HUMAN)
O95373	Importin-7 OS=Homo sapiens GN=IPO7 PE=1 SV=1 - (IPO7_HUMAN)
Q6WCQ1-3	Isoform 3 of Myosin phosphatase Rho-interacting protein OS=Homo sapiens GN=MPRIP - (MPRIP_HUMAN)
P12273	Prolactin-inducible protein OS=Homo sapiens GN=PIP PE=1 SV=1 - (PIP_HUMAN)
Q92945-2	Isoform 2 of Far upstream element-binding protein 2 OS=Homo sapiens GN=KHSRP - (FUBP2_HUMAN)
B7Z6L2	cDNA FLJ54156, highly similar to ADP-ribosylation factor GTPase-activating protein 3 OS=Homo sapiens PE=2 SV=1 - (B7Z6L2_HUMAN)
Q9Y2U5	Mitogen-activated protein kinase kinase kinase 2 OS=Homo sapiens GN=MAP3K2 PE=1 SV=2 - (M3K2_HUMAN)
Q9Y613	FH1/FH2 domain-containing protein 1 OS=Homo sapiens GN=FHOD1 PE=1 SV=3 - (FHOD1_HUMAN)
P20290-2	Isoform 2 of Transcription factor BTF3 OS=Homo sapiens GN=BTF3 - (BTF3_HUMAN)
Q15751	Probable E3 ubiquitin-protein ligase HERC1 OS=Homo sapiens GN=HERC1 PE=1 SV=2 - (HERC1_HUMAN)
A8KAP9	cDNA FLJ78448, highly similar to Homo sapiens argininosuccinate synthetase (ASS), transcript variant 1, mRNA OS=Homo sapiens PE=2 SV=1 - (A8KAP9_HUMAN)
Q14247-2	Isoform 2 of Src substrate cortactin OS=Homo sapiens GN=CTTN - (SRC8_HUMAN)
Q92508	Piezo-type mechanosensitive ion channel component 1 OS=Homo sapiens GN=PIEZO1 PE=1 SV=4 - (PIEZ1_HUMAN)
Q15257-3	Isoform 3 of Serine/threonine-protein phosphatase 2A activator OS=Homo sapiens GN=PPP2R4 - (PTPA_HUMAN)
B4DP21	Prostaglandin E synthase 3 OS=Homo sapiens GN=PTGES3 PE=2 SV=1 - (B4DP21_HUMAN)
P15374	Ubiquitin carboxyl-terminal hydrolase isozyme L3 OS=Homo sapiens GN=UCHL3 PE=1 SV=1 - (UCHL3_HUMAN)
Q96M91	Coiled-coil domain-containing protein 11 OS=Homo sapiens GN=CCDC11 PE=2 SV=2 - (CCD11_HUMAN)
B7Z9G1	cDNA, FLJ78825 OS=Homo sapiens PE=4 SV=1 - (B7Z9G1_HUMAN)
A0AVI2	Fer-1-like protein 5 OS=Homo sapiens GN=FER1L5 PE=2 SV=2 - (FR1L5_HUMAN)
E9PKS9	Trafficking protein particle complex subunit 4 OS=Homo sapiens GN=TRAPPC4 PE=4 SV=1 - (E9PKS9_HUMAN)
Q5EFE6	Anti-RhD monoclonal T125 kappa light chain OS=Homo sapiens PE=2 SV=1 - (Q5EFE6_HUMAN)
B4DL80	Cell division cycle protein 27 homolog OS=Homo sapiens GN=CDC27 PE=2 SV=1 - (B4DL80_HUMAN)
B4DJ44	cDNA FLJ54716, highly similar to Target of Myb protein 1 OS=Homo sapiens PE=2 SV=1 - (B4DJ44_HUMAN)
Q7Z3Z4	Piwi-like protein 4 OS=Homo sapiens GN=PIWIL4 PE=2 SV=2 - (PIWL4_HUMAN)
P58546	Myotrophin OS=Homo sapiens GN=MTPN PE=1 SV=2 - (MTPN_HUMAN)
P22531	Small proline-rich protein 2E OS=Homo sapiens GN=SPRR2E PE=2 SV=2 - (SPR2E_HUMAN)
B4DV28	cDNA FLJ54170, highly similar to Cytosolic nonspecific dipeptidase OS=Homo sapiens PE=2 SV=1 - (B4DV28_HUMAN)
B2R4D8	60S ribosomal protein L27 OS=Homo sapiens PE=2 SV=1 - (B2R4D8_HUMAN)
P49761	Dual specificity protein kinase CLK3 OS=Homo sapiens GN=CLK3 PE=1 SV=3 - (CLK3_HUMAN)
Q96JS4	Cerebral protein-3 OS=Homo sapiens GN=hucep-3 PE=2 SV=1 - (Q96JS4_HUMAN)
A6NMP4	Uncharacterized PRO1102-like protein LOC100132418 OS=Homo sapiens PE=4 SV=1 - (YJ010_HUMAN)
G3V0E5	Transferrin receptor (P90, CD71), isoform CRA_c OS=Homo sapiens GN=TFRC PE=4 SV=1 - (G3V0E5_HUMAN)
Q14730	La 4.1 protein (Fragment) OS=Homo sapiens PE=2 SV=1 - (Q14730_HUMAN)
Q15834	Coiled-coil domain-containing protein 85B OS=Homo sapiens GN=CCDC85B PE=1 SV=2 - (CC85B_HUMAN)
H7C2W1	D-beta-hydroxybutyrate dehydrogenase, mitochondrial (Fragment) OS=Homo sapiens GN=BDH1 PE=4 SV=1 - (H7C2W1_HUMAN)
B2R7R2	cDNA, FLJ93566, highly similar to Homo sapiens RWD domain containing 2 (RWDD2), mRNA OS=Homo sapiens PE=2 SV=1 - (B2R7R2_HUMAN)
H0YCD4	T-lymphoma invasion and metastasis-inducing protein 2 OS=Homo sapiens GN=TIAM2 PE=4 SV=1 - (H0YCD4_HUMAN)
E7EUJ8	Creatine kinase B-type OS=Homo sapiens GN=CKB PE=3 SV=2 - (E7EUJ8_HUMAN)
A8K4E4	cDNA FLJ75360, highly similar to Homo sapiens egf-like module containing, mucin-like, hormone receptor-like 3 (EMR3), transcript variant 1, mRNA OS=Homo sapiens PE=2 SV=1 - (A8K4E4_HUMAN)
E7EQV9	Ribosomal protein L15 (Fragment) OS=Homo sapiens GN=RPL15 PE=3 SV=1 - (E7EQV9_HUMAN)
Q96G46-2	Isoform 2 of tRNA-dihydrouridine(47) synthase (NAD(P)(+))-like OS=Homo sapiens GN=DUS3L - (DUS3L_HUMAN)
B7Z505	Protein NDRG1 OS=Homo sapiens GN=NDRG1 PE=2 SV=1 - (B7Z505_HUMAN)
H7C2M7	Armadillo repeat-containing protein 10 (Fragment) OS=Homo sapiens GN=ARMC10 PE=4 SV=1 - (H7C2M7_HUMAN)
O43598-2	Isoform 2 of Deoxyribonucleoside 5'-monophosphate N-glycosidase OS=Homo sapiens GN=RCL - (RCL_HUMAN)
A4D1I1	Similar to ribosomal protein S3a; 40S ribosomal protein S3a; v-fos transformation effector protein 1 OS=Homo sapiens GN=LOC402287 PE=4 SV=1 - (A4D1I1_HUMAN)
E9PRQ6	Acetyl-CoA acetyltransferase, mitochondrial OS=Homo sapiens GN=ACAT1 PE=4 SV=1 - (E9PRQ6_HUMAN)
Q49AN9	SNRPG protein OS=Homo sapiens GN=SNRPG PE=4 SV=1 - (Q49AN9_HUMAN)
H0UI63	Zinc finger, NFX1-type containing 1, isoform CRA_b OS=Homo sapiens GN=ZNFX1 PE=4 SV=1 - (H0UI63_HUMAN)
P17022-2	Isoform 2 of Zinc finger protein 18 OS=Homo sapiens GN=ZNF18 - (ZNF18_HUMAN)
Q2VEU1	Sulfurtransferase (Fragment) OS=Homo sapiens GN=MPST PE=2 SV=1 - (Q2VEU1_HUMAN)
H0YIB4	Serine/arginine-rich-splicing factor 9 (Fragment) OS=Homo sapiens GN=SRSF9 PE=4 SV=1 - (H0YIB4_HUMAN)
O43399-4	Isoform 4 of Tumor protein D54 OS=Homo sapiens GN=TPD52L2 - (TPD54_HUMAN)
P20962	Parathymosin OS=Homo sapiens GN=PTMS PE=1 SV=2 - (PTMS_HUMAN)
Q13151	Heterogeneous nuclear ribonucleoprotein A0 OS=Homo sapiens GN=HNRNPA0 PE=1 SV=1 - (ROA0_HUMAN)
E9PM69	26S protease regulatory subunit 6A OS=Homo sapiens GN=PSMC3 PE=3 SV=1 - (E9PM69_HUMAN)
Q6IPM2-2	Isoform 2 of IQ domain-containing protein E OS=Homo sapiens GN=IQCE - (IQCE_HUMAN)
P51148	Ras-related protein Rab-5C OS=Homo sapiens GN=RAB5C PE=1 SV=2 - (RAB5C_HUMAN)
B4E1W7	cDNA FLJ59114, highly similar to Amyotrophic lateral sclerosis 2 chromosomalregion candidate gene 8 protein (Calcium-responsefactor) (CaRF) (Protein NYD-SP24) OS=Homo sapiens PE=2 SV=1 - (B4E1W7_HUMAN)
B2R9G9	cDNA, FLJ94385, highly similar to Homo sapiens cofactor required for Sp1 transcriptional activation, subunit 7, 70kDa (CRSP7), mRNA OS=Homo sapiens PE=2 SV=1 - (B2R9G9_HUMAN)
P42694	Probable helicase with zinc finger domain OS=Homo sapiens GN=HELZ PE=1 SV=2 - (HELZ_HUMAN)
O00483	NADH dehydrogenase (ubiquinone) 1 alpha subcomplex subunit 4 OS=Homo sapiens GN=NDUFA4 PE=1 SV=1 - (NDUA4_HUMAN)
P53396-2	Isoform 2 of ATP-citrate synthase OS=Homo sapiens GN=ACLY - (ACLY_HUMAN)
Q53HL1	Myosin regulatory light chain MRCL3 variant (Fragment) OS=Homo sapiens PE=2 SV=1 - (Q53HL1_HUMAN)
O15547	P2X purinoceptor 6 OS=Homo sapiens GN=P2RX6 PE=1 SV=2 - (P2RX6_HUMAN)
B4DLW8	cDNA FLJ59339, highly similar to Probable ATP-dependent RNA helicase DDX5 (EC 3.6.1.-) OS=Homo sapiens PE=2 SV=1 - (B4DLW8_HUMAN)
Q96RX5	NADH ubiquinone oxidoreductase PDSW subunit (RH 16p13.3) (Fragment) OS=Homo sapiens GN=NDUFB10 PE=2 SV=1 - (Q96RX5_HUMAN)
Q59EW3	RAB18, member RAS oncogene family variant (Fragment) OS=Homo sapiens PE=2 SV=1 - (Q59EW3_HUMAN)
B7Z8R7	Nuclear receptor subfamily 1 group I member 3 OS=Homo sapiens GN=NR1I3 PE=2 SV=1 - (B7Z8R7_HUMAN)
Q9H6X5-2	Isoform 2 of Uncharacterized protein C19orf44 OS=Homo sapiens GN=C19orf44 - (CS044_HUMAN)
Q9NY47-4	Isoform 4 of Voltage-dependent calcium channel subunit alpha-2/delta-2 OS=Homo sapiens GN=CACNA2D2 - (CA2D2_HUMAN)
Q2F838	Eukaryotic translation elongation factor 1 gamma (Fragment) OS=Homo sapiens PE=2 SV=1 - (Q2F838_HUMAN)
B4DM22	cDNA FLJ53357, highly similar to 26S proteasome non-ATPase regulatory subunit 2 OS=Homo sapiens PE=2 SV=1 - (B4DM22_HUMAN)
B3VRC5	5-hydroxytryptamine (Serotonin) receptor 2B (Fragment) OS=Homo sapiens GN=HTR2B PE=4 SV=1 - (B3VRC5_HUMAN)
H0YN18	Proteasome subunit alpha type-4 OS=Homo sapiens GN=PSMA4 PE=4 SV=1 - (H0YN18_HUMAN)
O94929-2	Isoform 2 of Actin-binding LIM protein 3 OS=Homo sapiens GN=ABLIM3 - (ABLM3_HUMAN)
G3V4P8	Glia maturation factor beta (Fragment) OS=Homo sapiens GN=GMFB PE=4 SV=1 - (G3V4P8_HUMAN)
B4E0J3	cDNA FLJ58965, highly similar to Nonspecific lipid-transfer protein (EC 2.3.1.176) OS=Homo sapiens PE=2 SV=1 - (B4E0J3_HUMAN)
G3V117	Enhancer of yellow 2 homolog (Drosophila), isoform CRA_c OS=Homo sapiens GN=ENY2 PE=4 SV=1 - (G3V117_HUMAN)
O00151	PDZ and LIM domain protein 1 OS=Homo sapiens GN=PDLIM1 PE=1 SV=4 - (PDLI1_HUMAN)
Q5HYK3-2	Isoform 2 of 2-methoxy-6-polyprenyl-1,4-benzoquinol methylase, mitochondrial OS=Homo sapiens GN=COQ5 - (COQ5_HUMAN)
P19623	Spermidine synthase OS=Homo sapiens GN=SRM PE=1 SV=1 - (SPEE_HUMAN)
Q63HQ8	Putative uncharacterized protein DKFZp686M21196 OS=Homo sapiens GN=DKFZp686M21196 PE=2 SV=1 - (Q63HQ8_HUMAN)
Q8NHP1	Aflatoxin B1 aldehyde reductase member 4 OS=Homo sapiens GN=AKR7L PE=2 SV=6 - (ARK74_HUMAN)
Q8N2H2	cDNA FLJ90785 fis, clone THYRO1001457, moderately similar to H.sapiens protein kinase C mu OS=Homo sapiens PE=2 SV=1 - (Q8N2H2_HUMAN)
B4DMK9	cDNA FLJ56112, highly similar to Dihydrolipoyl dehydrogenase, mitochondrial (EC 1.8.1.4) OS=Homo sapiens PE=2 SV=1 - (B4DMK9_HUMAN)
Q8N7R7-3	Isoform 3 of Cyclin-Y-like protein 1 OS=Homo sapiens GN=CCNYL1 - (CCYL1_HUMAN)
E5RFM1	Uncharacterized protein OS=Homo sapiens PE=4 SV=1 - (E5RFM1_HUMAN)
F8VY04	Adenylate kinase 2, mitochondrial OS=Homo sapiens GN=AK2 PE=3 SV=1 - (F8VY04_HUMAN)
Q5QPM0	RNA binding protein, autoantigenic (HnRNP-associated with lethal yellow homolog (Mouse)) (Fragment) OS=Homo sapiens GN=RALY PE=2 SV=1 - (Q5QPM0_HUMAN)
B7Z1R5	V-type proton ATPase catalytic subunit A OS=Homo sapiens GN=ATP6V1A PE=2 SV=1 - (B7Z1R5_HUMAN)
Q9Y349	KIAA0232 protein OS=Homo sapiens GN=KIAA0232 PE=2 SV=1 - (Q9Y349_HUMAN)
H0Y3H1	Toll-interacting protein OS=Homo sapiens GN=TOLLIP PE=4 SV=1 - (H0Y3H1_HUMAN)
Q9P2F5-2	Isoform 2 of Storkhead-box protein 2 OS=Homo sapiens GN=STOX2 - (STOX2_HUMAN)
Q9NRS6-2	Isoform 2 of Sorting nexin-15 OS=Homo sapiens GN=SNX15 - (SNX15_HUMAN)
H0YLF3	Beta-2-microglobulin (Fragment) OS=Homo sapiens GN=B2M PE=3 SV=1 - (H0YLF3_HUMAN)
A8K6Y1	cDNA FLJ75526, highly similar to Homo sapiens proliferation-associated 2G4, 38kDa (PA2G4), mRNA (Fragment) OS=Homo sapiens PE=2 SV=1 - (A8K6Y1_HUMAN)
Q9NSA3	Beta-catenin-interacting protein 1 OS=Homo sapiens GN=CTNNBIP1 PE=1 SV=1 - (CNBP1_HUMAN)
Q6ZUG2	cDNA FLJ43743 fis, clone TESTI2019042 OS=Homo sapiens PE=2 SV=1 - (Q6ZUG2_HUMAN)
B4DF39	cDNA FLJ59119, highly similar to Receptor expression-enhancing protein 6 OS=Homo sapiens PE=2 SV=1 - (B4DF39_HUMAN)
Q9H875	PRKR-interacting protein 1 OS=Homo sapiens GN=PRKRIP1 PE=1 SV=1 - (PKRI1_HUMAN)
B1AH89	Tubulin tyrosine ligase-like family, member 12 OS=Homo sapiens GN=TTLL12 PE=4 SV=1 - (B1AH89_HUMAN)
F5H1H8	Activated RNA polymerase II transcriptional coactivator p15 OS=Homo sapiens GN=SUB1 PE=4 SV=1 - (F5H1H8_HUMAN)
P51178-2	Isoform 2 of 1-phosphatidylinositol 4,5-bisphosphate phosphodiesterase delta-1 OS=Homo sapiens GN=PLCD1 - (PLCD1_HUMAN)
Q9BU79	Transmembrane protein C7orf23 OS=Homo sapiens GN=C7orf23 PE=2 SV=1 - (CG023_HUMAN)
Q7LAX7	CKII beta binding protein 2 OS=Homo sapiens PE=2 SV=1 - (Q7LAX7_HUMAN)
H0Y8J7	Eukaryotic translation initiation factor 4E (Fragment) OS=Homo sapiens GN=EIF4E PE=3 SV=1 - (H0Y8J7_HUMAN)
Q8NGP8	Olfactory receptor 5M1 OS=Homo sapiens GN=OR5M1 PE=2 SV=1 - (OR5M1_HUMAN)
B7Z3W8	Transporter OS=Homo sapiens PE=2 SV=1 - (B7Z3W8_HUMAN)
Q9Y5Z4-2	Isoform 2 of Heme-binding protein 2 OS=Homo sapiens GN=HEBP2 - (HEBP2_HUMAN)
Q96L46	Calpain small subunit 2 OS=Homo sapiens GN=CAPNS2 PE=2 SV=2 - (CPNS2_HUMAN)
P35321	Cornifin-A OS=Homo sapiens GN=SPRR1A PE=1 SV=2 - (SPR1A_HUMAN)
Q9ULC4-2	Isoform 2 of Malignant T-cell-amplified sequence 1 OS=Homo sapiens GN=MCTS1 - (MCTS1_HUMAN)
C9JGI8	MAGUK p55 subfamily member 6 (Fragment) OS=Homo sapiens GN=MPP6 PE=4 SV=1 - (C9JGI8_HUMAN)
Q7Z739	YTH domain family protein 3 OS=Homo sapiens GN=YTHDF3 PE=1 SV=1 - (YTHD3_HUMAN)
B4E2I4	cDNA FLJ58227, highly similar to Glutamate--cysteine ligase catalytic subunit (EC 6.3.2.2) OS=Homo sapiens PE=2 SV=1 - (B4E2I4_HUMAN)
Q6NVY0	Calcyclin binding protein OS=Homo sapiens GN=CACYBP PE=2 SV=1 - (Q6NVY0_HUMAN)
P08621-2	Isoform 2 of U1 small nuclear ribonucleoprotein 70 kDa OS=Homo sapiens GN=SNRNP70 - (RU17_HUMAN)
B4DI45	cDNA FLJ56612, highly similar to Inner nuclear membrane protein Man1 OS=Homo sapiens PE=2 SV=1 - (B4DI45_HUMAN)
Q8NAN7	Very low density lipoprotein receptor OS=Homo sapiens GN=VLDLR PE=2 SV=1 - (Q8NAN7_HUMAN)
Q99704-3	Isoform 3 of Docking protein 1 OS=Homo sapiens GN=DOK1 - (DOK1_HUMAN)
E9PKL8	NADH dehydrogenase (ubiquinone) iron-sulfur protein 3, mitochondrial (Fragment) OS=Homo sapiens GN=NDUFS3 PE=4 SV=1 - (E9PKL8_HUMAN)
O75691	Small subunit processome component 20 homolog OS=Homo sapiens GN=UTP20 PE=1 SV=3 - (UTP20_HUMAN)
Q9Y3C4-2	Isoform 2 of TP53RK-binding protein OS=Homo sapiens GN=TPRKB - (TPRKB_HUMAN)
H7C125	Ras-related protein Rab-2A (Fragment) OS=Homo sapiens GN=RAB2A PE=4 SV=1 - (H7C125_HUMAN)
P24534	Elongation factor 1-beta OS=Homo sapiens GN=EEF1B2 PE=1 SV=3 - (EF1B_HUMAN)
E5RJ48	Acyl-protein thioesterase 1 (Fragment) OS=Homo sapiens GN=LYPLA1 PE=4 SV=1 - (E5RJ48_HUMAN)
O00487	26S proteasome non-ATPase regulatory subunit 14 OS=Homo sapiens GN=PSMD14 PE=1 SV=1 - (PSDE_HUMAN)
Q9BTV4	Transmembrane protein 43 OS=Homo sapiens GN=TMEM43 PE=1 SV=1 - (TMM43_HUMAN)
G3V5F2	Peptidyl-prolyl cis-trans isomerase FKBP3 OS=Homo sapiens GN=FKBP3 PE=4 SV=1 - (G3V5F2_HUMAN)
H0YHG0	Uncharacterized protein (Fragment) OS=Homo sapiens PE=4 SV=1 - (H0YHG0_HUMAN)
Q9Y6D5	Brefeldin A-inhibited guanine nucleotide-exchange protein 2 OS=Homo sapiens GN=ARFGEF2 PE=1 SV=3 - (BIG2_HUMAN)
A8K4W2	cDNA FLJ78635, highly similar to Homo sapiens ATP synthase, H+ transporting, mitochondrial F0 complex, subunit b, isoform 1 (ATP5F1), transcript variant 1, mRNA OS=Homo sapiens PE=2 SV=1 - (A8K4W2_HUMAN)
Q96LZ7-4	Isoform 4 of Regulator of microtubule dynamics protein 2 OS=Homo sapiens GN=FAM82A1 - (RMD2_HUMAN)
Q9Y6K8-3	Isoform 3 of Adenylate kinase isoenzyme 5 OS=Homo sapiens GN=AK5 - (KAD5_HUMAN)
B4DPD5	cDNA FLJ56307, highly similar to Ubiquitin thioesterase protein OTUB1 (EC 3.4.-.-) OS=Homo sapiens PE=2 SV=1 - (B4DPD5_HUMAN)
B4DFL3	cDNA FLJ56661, highly similar to Proteasome subunit beta type 4 (EC 3.4.25.1) OS=Homo sapiens PE=2 SV=1 - (B4DFL3_HUMAN)
B4DEE5	cDNA FLJ60230, highly similar to Arsenical pump-driving ATPase (EC 3.6.3.16) OS=Homo sapiens PE=2 SV=1 - (B4DEE5_HUMAN)
A6NHJ4	Zinc finger protein 860 OS=Homo sapiens GN=ZNF860 PE=2 SV=3 - (ZN860_HUMAN)
H7C547	Sodium/potassium-transporting ATPase subunit beta-3 (Fragment) OS=Homo sapiens GN=ATP1B3 PE=3 SV=1 - (H7C547_HUMAN)
A5YM41	MTSS1 protein OS=Homo sapiens GN=MTSS1 PE=2 SV=1 - (A5YM41_HUMAN)
O00300	Tumor necrosis factor receptor superfamily member 11B OS=Homo sapiens GN=TNFRSF11B PE=1 SV=3 - (TR11B_HUMAN)
Q5VYB5	Nucleoporin 43kDa OS=Homo sapiens GN=NUP43 PE=2 SV=1 - (Q5VYB5_HUMAN)
P83881	60S ribosomal protein L36a OS=Homo sapiens GN=RPL36A PE=1 SV=2 - (RL36A_HUMAN)
H0YCR4	Post-GPI attachment to proteins factor 2 (Fragment) OS=Homo sapiens GN=PGAP2 PE=4 SV=1 - (H0YCR4_HUMAN)
O60493-3	Isoform 3 of Sorting nexin-3 OS=Homo sapiens GN=SNX3 - (SNX3_HUMAN)
Q5SSJ5-2	Isoform 2 of Heterochromatin protein 1-binding protein 3 OS=Homo sapiens GN=HP1BP3 - (HP1B3_HUMAN)
E7EWI9	Heterogeneous nuclear ribonucleoprotein A3 OS=Homo sapiens GN=HNRNPA3 PE=4 SV=1 - (E7EWI9_HUMAN)
H0YLX4	WD repeat-containing protein 72 OS=Homo sapiens GN=WDR72 PE=4 SV=1 - (H0YLX4_HUMAN)
D6RAG5	S-phase kinase-associated protein 2 OS=Homo sapiens GN=SKP2 PE=4 SV=1 - (D6RAG5_HUMAN)
H3BQZ7	HCG2044799 OS=Homo sapiens GN=hCG_2044799 PE=4 SV=1 - (H3BQZ7_HUMAN)
Q50KG7	Taste receptor type 2 (Fragment) OS=Homo sapiens GN=Hosa(Biaka)-T2R47 PE=3 SV=1 - (Q50KG7_HUMAN)
P53618	Coatomer subunit beta OS=Homo sapiens GN=COPB1 PE=1 SV=3 - (COPB_HUMAN)
Q96GI7	Protein FAM89A OS=Homo sapiens GN=FAM89A PE=2 SV=1 - (FA89A_HUMAN)
P05204	Non-histone chromosomal protein HMG-17 OS=Homo sapiens GN=HMGN2 PE=1 SV=3 - (HMGN2_HUMAN)
E5RIY5	Serine protease 45 OS=Homo sapiens GN=PRSS45 PE=3 SV=1 - (E5RIY5_HUMAN)
Q9NRX4-2	Isoform 2 of 14 kDa phosphohistidine phosphatase OS=Homo sapiens GN=PHPT1 - (PHP14_HUMAN)
A8K335	cDNA FLJ76254, highly similar to Homo sapiens gamma-glutamyl hydrolase (GGH), mRNA OS=Homo sapiens PE=2 SV=1 - (A8K335_HUMAN)
P07359	Platelet glycoprotein Ib alpha chain OS=Homo sapiens GN=GP1BA PE=1 SV=1 - (GP1BA_HUMAN)
H7C1C6	Translocon-associated protein subunit delta (Fragment) OS=Homo sapiens GN=SSR4 PE=4 SV=1 - (H7C1C6_HUMAN)
Q6GMR7	Fatty-acid amide hydrolase 2 OS=Homo sapiens GN=FAAH2 PE=2 SV=1 - (FAAH2_HUMAN)
Q8N8A7	HCG38579, isoform CRA_c OS=Homo sapiens GN=TM6SF2 PE=2 SV=1 - (Q8N8A7_HUMAN)
C9JYN0	Synaptophysin-like protein 1 OS=Homo sapiens GN=SYPL1 PE=4 SV=1 - (C9JYN0_HUMAN)
Q9Y5S9	RNA-binding protein 8A OS=Homo sapiens GN=RBM8A PE=1 SV=1 - (RBM8A_HUMAN)
Q6LES1	TYRP1 protein (Fragment) OS=Homo sapiens GN=TYRP1 PE=2 SV=1 - (Q6LES1_HUMAN)
C9J0K6	Sorcin OS=Homo sapiens GN=SRI PE=4 SV=1 - (C9J0K6_HUMAN)
F5H3J5	Protein S100-A4 OS=Homo sapiens GN=S100A4 PE=4 SV=1 - (F5H3J5_HUMAN)
P14854	Cytochrome c oxidase subunit 6B1 OS=Homo sapiens GN=COX6B1 PE=1 SV=2 - (CX6B1_HUMAN)
Q86UJ1	Putative uncharacterized protein CNTNAP2 (Fragment) OS=Homo sapiens GN=CNTNAP2 PE=2 SV=1 - (Q86UJ1_HUMAN)
P36969-2	Isoform Cytoplasmic of Phospholipid hydroperoxide glutathione peroxidase, mitochondrial OS=Homo sapiens GN=GPX4 - (GPX4_HUMAN)
P55010	Eukaryotic translation initiation factor 5 OS=Homo sapiens GN=EIF5 PE=1 SV=2 - (IF5_HUMAN)
B5BUG9	Bone morphogenetic protein receptor type IA (Fragment) OS=Homo sapiens GN=BMPR1A PE=2 SV=1 - (B5BUG9_HUMAN)
G3V4R8	Tubulin polyglutamylase TTLL5 OS=Homo sapiens GN=TTLL5 PE=4 SV=1 - (G3V4R8_HUMAN)
B4DFN5	cDNA FLJ56769, weakly similar to Nuclear pore complex-interacting protein OS=Homo sapiens PE=4 SV=1 - (B4DFN5_HUMAN)
F2Z317	Histone-lysine N-methyltransferase SETD2 OS=Homo sapiens GN=SETD2 PE=4 SV=2 - (F2Z317_HUMAN)
Q5TEU4	NADH dehydrogenase (ubiquinone) 1 alpha subcomplex assembly factor 5 OS=Homo sapiens GN=NDUFAF5 PE=1 SV=1 - (NDUF5_HUMAN)
B7Z8G2	Peptidyl-prolyl cis-trans isomerase FKBP5 OS=Homo sapiens GN=FKBP5 PE=2 SV=1 - (B7Z8G2_HUMAN)
E5RHK5	Glutathione peroxidase 3 OS=Homo sapiens GN=GPX3 PE=4 SV=1 - (E5RHK5_HUMAN)
Q53FX5	Lin-7 homolog C variant (Fragment) OS=Homo sapiens PE=2 SV=1 - (Q53FX5_HUMAN)
H7C0K5	Enhancer of polycomb homolog 2 (Fragment) OS=Homo sapiens GN=EPC2 PE=4 SV=1 - (H7C0K5_HUMAN)
Q9NZZ3-2	Isoform 2 of Charged multivesicular body protein 5 OS=Homo sapiens GN=CHMP5 - (CHMP5_HUMAN)
Q6UXN9	WD repeat-containing protein 82 OS=Homo sapiens GN=WDR82 PE=1 SV=1 - (WDR82_HUMAN)
Q15776	Zinc finger protein 192 OS=Homo sapiens GN=ZNF192 PE=1 SV=2 - (ZN192_HUMAN)
Q9NX07-2	Isoform 2 of tRNA selenocysteine 1-associated protein 1 OS=Homo sapiens GN=TRNAU1AP - (TSAP1_HUMAN)
H3BSP9	Metallothionein OS=Homo sapiens GN=MT2A PE=3 SV=1 - (H3BSP9_HUMAN)
P56385	ATP synthase subunit e, mitochondrial OS=Homo sapiens GN=ATP5I PE=1 SV=2 - (ATP5I_HUMAN)
Q6ZNE9	RUN and FYVE domain-containing protein 4 OS=Homo sapiens GN=RUFY4 PE=2 SV=2 - (RUFY4_HUMAN)
H0Y9T8	Protein PRR5-ARHGAP8 (Fragment) OS=Homo sapiens GN=PRR5-ARHGAP8 PE=4 SV=1 - (H0Y9T8_HUMAN)
B4DYI2	cDNA FLJ59639 OS=Homo sapiens PE=2 SV=1 - (B4DYI2_HUMAN)
H7C4C8	T-complex protein 1 subunit theta (Fragment) OS=Homo sapiens GN=CCT8 PE=3 SV=1 - (H7C4C8_HUMAN)
Q8WUW1	Protein BRICK1 OS=Homo sapiens GN=BRK1 PE=1 SV=1 - (BRK1_HUMAN)
H0YCU2	Sharpin (Fragment) OS=Homo sapiens GN=SHARPIN PE=4 SV=1 - (H0YCU2_HUMAN)
Q8N2E1	cDNA PSEC0226 fis, clone HEMBA1005833, moderately similar to Mus musculus carboxypeptidase X2 mRNA OS=Homo sapiens PE=2 SV=1 - (Q8N2E1_HUMAN)
A6NLI5	Putative tripartite motif-containing protein 64C OS=Homo sapiens GN=TRIM64C PE=5 SV=3 - (TR64C_HUMAN)
Q92574	Hamartin OS=Homo sapiens GN=TSC1 PE=1 SV=2 - (TSC1_HUMAN)
A6NJH9	Eukaryotic translation initiation factor 1A, Y-chromosomal OS=Homo sapiens GN=EIF1AY PE=4 SV=1 - (A6NJH9_HUMAN)
Q9HAC7-2	Isoform 2 of CaiB/baiF CoA-transferase family protein C7orf10 OS=Homo sapiens GN=C7orf10 - (CG010_HUMAN)
B4DQI0	cDNA FLJ59385 OS=Homo sapiens PE=2 SV=1 - (B4DQI0_HUMAN)
O43781	Dual specificity tyrosine-phosphorylation-regulated kinase 3 OS=Homo sapiens GN=DYRK3 PE=1 SV=3 - (DYRK3_HUMAN)
Q9HBA9	Putative N-acetylated-alpha-linked acidic dipeptidase OS=Homo sapiens GN=FOLH1B PE=2 SV=1 - (FOH1B_HUMAN)
O95741	Copine-6 OS=Homo sapiens GN=CPNE6 PE=1 SV=3 - (CPNE6_HUMAN)
B4DI85	ADP-ribosylation factor-like 8B, isoform CRA_a OS=Homo sapiens GN=ARL8B PE=2 SV=1 - (B4DI85_HUMAN)
H3BMX4	Protein FAM188B2 OS=Homo sapiens GN=FAM188B2 PE=4 SV=1 - (H3BMX4_HUMAN)
H0YAR1	Lysyl oxidase homolog 2 (Fragment) OS=Homo sapiens GN=LOXL2 PE=4 SV=1 - (H0YAR1_HUMAN)
F1T0L1	Hypothetical LOC645967 OS=Homo sapiens GN=LOC645967 PE=2 SV=1 - (F1T0L1_HUMAN)
G3V1D7	Reticulon 4 receptor-like 2, isoform CRA_a OS=Homo sapiens GN=RTN4RL2 PE=4 SV=1 - (G3V1D7_HUMAN)
H0YG18	Mesothelin-like protein (Fragment) OS=Homo sapiens GN=MSLNL PE=4 SV=1 - (H0YG18_HUMAN)
D6RJD6	NADH dehydrogenase (ubiquinone) 1 alpha subcomplex subunit 2 OS=Homo sapiens GN=NDUFA2 PE=4 SV=1 - (D6RJD6_HUMAN)
C9J7D4	Uncharacterized protein OS=Homo sapiens GN=REPIN1 PE=4 SV=2 - (C9J7D4_HUMAN)
O43660-2	Isoform 2 of Pleiotropic regulator 1 OS=Homo sapiens GN=PLRG1 - (PLRG1_HUMAN)
B4DF97	cDNA FLJ59673, highly similar to Homo sapiens growth and transformation-dependent protein (E2IG5), mRNA OS=Homo sapiens PE=2 SV=1 - (B4DF97_HUMAN)
Q86UD0	Suppressor APC domain-containing protein 2 OS=Homo sapiens GN=SAPCD2 PE=2 SV=2 - (SAPC2_HUMAN)
Q9BV35	Calcium-binding mitochondrial carrier protein SCaMC-3 OS=Homo sapiens GN=SLC25A23 PE=1 SV=2 - (SCMC3_HUMAN)
P36639	7,8-dihydro-8-oxoguanine triphosphatase OS=Homo sapiens GN=NUDT1 PE=1 SV=3 - (8ODP_HUMAN)
Q9Y4X0-2	Isoform 2 of AMME syndrome candidate gene 1 protein OS=Homo sapiens GN=AMMECR1 - (AMMR1_HUMAN)
P35626	Beta-adrenergic receptor kinase 2 OS=Homo sapiens GN=ADRBK2 PE=2 SV=2 - (ARBK2_HUMAN)
P56381	ATP synthase subunit epsilon, mitochondrial OS=Homo sapiens GN=ATP5E PE=1 SV=2 - (ATP5E_HUMAN)
Q6ZTY9	Uncharacterized protein C7orf65 OS=Homo sapiens GN=C7orf65 PE=2 SV=1 - (CG065_HUMAN)
Q03013-2	Isoform 2 of Glutathione S-transferase Mu 4 OS=Homo sapiens GN=GSTM4 - (GSTM4_HUMAN)
Q6JEL2	Kelch-like protein 10 OS=Homo sapiens GN=KLHL10 PE=2 SV=1 - (KLH10_HUMAN)
Q8WVC0-2	Isoform 2 of RNA polymerase-associated protein LEO1 OS=Homo sapiens GN=LEO1 - (LEO1_HUMAN)
Q86VX9-3	Isoform 3 of Vacuolar fusion protein MON1 homolog A OS=Homo sapiens GN=MON1A - (MON1A_HUMAN)
Q5TC84	Opioid growth factor receptor-like protein 1 OS=Homo sapiens GN=OGFRL1 PE=2 SV=1 - (OGRL1_HUMAN)
Q9NR45	Sialic acid synthase OS=Homo sapiens GN=NANS PE=1 SV=2 - (SIAS_HUMAN)
Q9Y3Q8	TSC22 domain family protein 4 OS=Homo sapiens GN=TSC22D4 PE=1 SV=2 - (T22D4_HUMAN)
Q8N7K0	Zinc finger protein 433 OS=Homo sapiens GN=ZNF433 PE=2 SV=1 - (ZN433_HUMAN)
Q8IW36	Zinc finger protein 695 OS=Homo sapiens GN=ZNF695 PE=2 SV=4 - (ZN695_HUMAN)
B3KUY1	Splicing factor, arginine/serine-rich 2, isoform CRA_d OS=Homo sapiens GN=SFRS2 PE=2 SV=1 - (B3KUY1_HUMAN)
D3DSM3	HCG1749250, isoform CRA_b OS=Homo sapiens GN=hCG_1749250 PE=4 SV=1 - (D3DSM3_HUMAN)
B4DY47	Prohibitin OS=Homo sapiens GN=PHB PE=2 SV=1 - (B4DY47_HUMAN)
Q7Z4B2	MSTP161 OS=Homo sapiens GN=MST161 PE=2 SV=1 - (Q7Z4B2_HUMAN)
A4UCS3	NADH:ubiquinone oxidoreductase SDAP subunit (Fragment) OS=Homo sapiens PE=2 SV=1 - (A4UCS3_HUMAN)
C9JUL6	Uncharacterized protein OS=Homo sapiens PE=4 SV=1 - (C9JUL6_HUMAN)
G1UI17	Glycogen debranching enzyme (Fragment) OS=Homo sapiens GN=AGL PE=2 SV=1 - (G1UI17_HUMAN)
B4DQG8	cDNA FLJ54124, highly similar to DNA methyltransferase 1-associated protein 1 OS=Homo sapiens PE=2 SV=1 - (B4DQG8_HUMAN)
Q8TAK2	Similar to catalase (Fragment) OS=Homo sapiens PE=2 SV=1 - (Q8TAK2_HUMAN)
A8MXP7	Major facilitator superfamily domain-containing protein 12 OS=Homo sapiens GN=MFSD12 PE=4 SV=1 - (A8MXP7_HUMAN)
D0EZN6	Cytochrome p450 (Fragment) OS=Homo sapiens GN=CYP1B1 PE=4 SV=1 - (D0EZN6_HUMAN)
I3L1M9	Diphthamide biosynthesis protein 1 (Fragment) OS=Homo sapiens GN=DPH1 PE=4 SV=1 - (I3L1M9_HUMAN)
Q5RLJ0	CLE OS=Homo sapiens PE=2 SV=1 - (Q5RLJ0_HUMAN)
Q9UG64	Putative uncharacterized protein DKFZp586I1223 (Fragment) OS=Homo sapiens GN=DKFZp586I1223 PE=2 SV=1 - (Q9UG64_HUMAN)
F8WF17	Galectin-related protein OS=Homo sapiens GN=LGALSL PE=4 SV=1 - (F8WF17_HUMAN)
H0YCE4	Uncharacterized protein C1orf105 (Fragment) OS=Homo sapiens GN=C1orf105 PE=4 SV=1 - (H0YCE4_HUMAN)
F5GZZ9	Soluble CD163 OS=Homo sapiens GN=CD163 PE=4 SV=1 - (F5GZZ9_HUMAN)
A0N5T0	V1 protein (Fragment) OS=Homo sapiens GN=V1 PE=4 SV=1 - (A0N5T0_HUMAN)
B7Z8Q1	cDNA FLJ53690, highly similar to SEC14-like protein 2 OS=Homo sapiens PE=2 SV=1 - (B7Z8Q1_HUMAN)
Q658V8	Putative uncharacterized protein DKFZp666C182 (Fragment) OS=Homo sapiens GN=DKFZp666C182 PE=2 SV=1 - (Q658V8_HUMAN)
E7BYD6	MHC class II antigen (Fragment) OS=Homo sapiens GN=HLA-DRB1 PE=4 SV=1 - (E7BYD6_HUMAN)
A0JNT2	KRT83 protein OS=Homo sapiens GN=KRT83 PE=2 SV=1 - (A0JNT2_HUMAN)
I3L2N9	Uncharacterized protein OS=Homo sapiens PE=4 SV=1 - (I3L2N9_HUMAN)
P19012	Keratin, type I cytoskeletal 15 OS=Homo sapiens GN=KRT15 PE=1 SV=3 - (K1C15_HUMAN)
O76013-2	Isoform 2 of Keratin, type I cuticular Ha6 OS=Homo sapiens GN=KRT36 - (KRT36_HUMAN)
A1A4E9	Keratin 13 OS=Homo sapiens GN=KRT13 PE=2 SV=1 - (A1A4E9_HUMAN)
Q0IIN1	Keratin 77 OS=Homo sapiens GN=KRT77 PE=2 SV=1 - (Q0IIN1_HUMAN)
F5H5D3	Tubulin alpha-1C chain OS=Homo sapiens GN=TUBA1C PE=3 SV=1 - (F5H5D3_HUMAN)
O76015	Keratin, type I cuticular Ha8 OS=Homo sapiens GN=KRT38 PE=2 SV=3 - (KRT38_HUMAN)
E7ENN3	Nesprin-1 OS=Homo sapiens GN=SYNE1 PE=4 SV=1 - (E7ENN3_HUMAN)
Q96KK5	Histone H2A type 1-H OS=Homo sapiens GN=HIST1H2AH PE=1 SV=3 - (H2A1H_HUMAN)
B2R6L0	cDNA, FLJ93005, highly similar to Homo sapiens tubulin, beta polypeptide (TUBB), mRNA OS=Homo sapiens PE=2 SV=1 - (B2R6L0_HUMAN)
F8VZY9	Keratin, type I cytoskeletal 18 OS=Homo sapiens GN=KRT18 PE=3 SV=1 - (F8VZY9_HUMAN)
Q15149-4	Isoform 4 of Plectin OS=Homo sapiens GN=PLEC - (PLEC_HUMAN)
Q6A162	Keratin, type I cytoskeletal 40 OS=Homo sapiens GN=KRT40 PE=1 SV=2 - (K1C40_HUMAN)
Q7Z3Y9	Keratin, type I cytoskeletal 26 OS=Homo sapiens GN=KRT26 PE=1 SV=2 - (K1C26_HUMAN)
Q6DRA6	Putative histone H2B type 2-D OS=Homo sapiens GN=HIST2H2BD PE=5 SV=3 - (H2B2D_HUMAN)
P08670	Vimentin OS=Homo sapiens GN=VIM PE=1 SV=4 - (VIME_HUMAN)
Q5I6Y6	Lamin A/C transcript variant 1 OS=Homo sapiens GN=LMNA PE=2 SV=1 - (Q5I6Y6_HUMAN)
Q9HCF6-8	Isoform 8 of Transient receptor potential cation channel subfamily M member 3 OS=Homo sapiens GN=TRPM3 - (TRPM3_HUMAN)
O94822	E3 ubiquitin-protein ligase listerin OS=Homo sapiens GN=LTN1 PE=1 SV=6 - (LTN1_HUMAN)
B4E380	Histone H3 OS=Homo sapiens PE=3 SV=1 - (B4E380_HUMAN)
P60174-1	Isoform 2 of Triosephosphate isomerase OS=Homo sapiens GN=TPI1 - (TPIS_HUMAN)
Q92794	Histone acetyltransferase KAT6A OS=Homo sapiens GN=KAT6A PE=1 SV=2 - (KAT6A_HUMAN)
B3KSC3	cDNA FLJ35987 fis, clone TESTI2014269, highly similar to D-3-phosphoglycerate dehydrogenase (EC 1.1.1.95) OS=Homo sapiens PE=2 SV=1 - (B3KSC3_HUMAN)
B7Z2N5	cDNA FLJ51840, highly similar to Alpha-actinin-2 OS=Homo sapiens PE=2 SV=1 - (B7Z2N5_HUMAN)
Q5TEC6	Histone H3 OS=Homo sapiens GN=HIST2H3PS2 PE=3 SV=1 - (Q5TEC6_HUMAN)
Q14134-2	Isoform Beta of Tripartite motif-containing protein 29 OS=Homo sapiens GN=TRIM29 - (TRI29_HUMAN)
A6NC98	Coiled-coil domain-containing protein 88B OS=Homo sapiens GN=CCDC88B PE=1 SV=1 - (CC88B_HUMAN)
F8VS61	Keratin, type II cytoskeletal 1b OS=Homo sapiens GN=KRT77 PE=4 SV=1 - (F8VS61_HUMAN)
F5H335	Eukaryotic translation initiation factor 3 subunit A OS=Homo sapiens GN=EIF3A PE=4 SV=1 - (F5H335_HUMAN)
H3BTN5	Pyruvate kinase (Fragment) OS=Homo sapiens GN=PKM PE=3 SV=1 - (H3BTN5_HUMAN)
Q86Y33-3	Isoform 3 of Cell division cycle protein 20 homolog B OS=Homo sapiens GN=CDC20B - (CD20B_HUMAN)
Q0QF37	Malate dehydrogenase (Fragment) OS=Homo sapiens GN=MDH2 PE=2 SV=1 - (Q0QF37_HUMAN)
F8W6W4	Voltage-gated potassium channel subunit beta-1 OS=Homo sapiens GN=KCNAB1 PE=4 SV=1 - (F8W6W4_HUMAN)
B4DMA2	cDNA FLJ54023, highly similar to Heat shock protein HSP 90-beta OS=Homo sapiens PE=2 SV=1 - (B4DMA2_HUMAN)
H3BST3	Stomatin-like protein 1 OS=Homo sapiens GN=STOML1 PE=4 SV=1 - (H3BST3_HUMAN)
Q6MZM0	Hephaestin-like protein 1 OS=Homo sapiens GN=HEPHL1 PE=2 SV=2 - (HPHL1_HUMAN)
Q14574	Desmocollin-3 OS=Homo sapiens GN=DSC3 PE=1 SV=3 - (DSC3_HUMAN)
O14863	Zinc transporter 4 OS=Homo sapiens GN=SLC30A4 PE=2 SV=2 - (ZNT4_HUMAN)
Q93034	Cullin-5 OS=Homo sapiens GN=CUL5 PE=1 SV=4 - (CUL5_HUMAN)
H7C469	Uncharacterized protein (Fragment) OS=Homo sapiens PE=3 SV=1 - (H7C469_HUMAN)
F8WCP0	Nebulin OS=Homo sapiens GN=NEB PE=4 SV=1 - (F8WCP0_HUMAN)
Q15059	Bromodomain-containing protein 3 OS=Homo sapiens GN=BRD3 PE=1 SV=1 - (BRD3_HUMAN)
P49454	Centromere protein F OS=Homo sapiens GN=CENPF PE=1 SV=2 - (CENPF_HUMAN)
Q2LD37-4	Isoform 4 of Uncharacterized protein KIAA1109 OS=Homo sapiens GN=KIAA1109 - (K1109_HUMAN)
Q9UMY1	Nucleolar protein 7 OS=Homo sapiens GN=NOL7 PE=1 SV=2 - (NOL7_HUMAN)
B2RUU3	Dedicator of cytokinesis 1 OS=Homo sapiens GN=DOCK1 PE=2 SV=1 - (B2RUU3_HUMAN)
Q32MZ4-3	Isoform 3 of Leucine-rich repeat flightless-interacting protein 1 OS=Homo sapiens GN=LRRFIP1 - (LRRF1_HUMAN)
Q13813	Spectrin alpha chain, brain OS=Homo sapiens GN=SPTAN1 PE=1 SV=3 - (SPTA2_HUMAN)
B2RBE5	cDNA, FLJ95468, highly similar to Homo sapiens transcriptional coactivator tubedown-100 (TBDN100),transcript variant 1, mRNA OS=Homo sapiens PE=2 SV=1 - (B2RBE5_HUMAN)
Q5TZA2-2	Isoform 2 of Rootletin OS=Homo sapiens GN=CROCC - (CROCC_HUMAN)
Q96AQ8	Coiled-coil domain-containing protein 90A, mitochondrial OS=Homo sapiens GN=CCDC90A PE=2 SV=1 - (CC90A_HUMAN)
A1L390	Pleckstrin homology domain-containing family G member 3 OS=Homo sapiens GN=PLEKHG3 PE=1 SV=1 - (PKHG3_HUMAN)
B2RA91	cDNA, FLJ94773, highly similar to Homo sapiens splicing factor, arginine/serine-rich 2, interacting protein (SFRS2IP), mRNA OS=Homo sapiens PE=2 SV=1 - (B2RA91_HUMAN)
A4FUJ8	MKL1 protein OS=Homo sapiens GN=MKL1 PE=2 SV=1 - (A4FUJ8_HUMAN)
Q9BYR4	Keratin-associated protein 4-3 OS=Homo sapiens GN=KRTAP4-3 PE=2 SV=2 - (KRA43_HUMAN)
Q5THJ4	Vacuolar protein sorting-associated protein 13D OS=Homo sapiens GN=VPS13D PE=1 SV=1 - (VP13D_HUMAN)
Q0VF96	Cingulin-like protein 1 OS=Homo sapiens GN=CGNL1 PE=1 SV=2 - (CGNL1_HUMAN)
F8WAH5	Laminin subunit alpha-2 OS=Homo sapiens GN=LAMA2 PE=4 SV=1 - (F8WAH5_HUMAN)
Q8N1C8	HSPA9 protein (Fragment) OS=Homo sapiens GN=HSPA9 PE=2 SV=1 - (Q8N1C8_HUMAN)
P0CG32	Putative zinc finger CCHC domain-containing protein 18 OS=Homo sapiens GN=ZCCHC18 PE=5 SV=1 - (ZCC18_HUMAN)
G3V388	Coiled-coil domain-containing protein 18 OS=Homo sapiens GN=CCDC18 PE=4 SV=1 - (G3V388_HUMAN)
Q9BU89	Deoxyhypusine hydroxylase OS=Homo sapiens GN=DOHH PE=1 SV=1 - (DOHH_HUMAN)
O14777	Kinetochore protein NDC80 homolog OS=Homo sapiens GN=NDC80 PE=1 SV=1 - (NDC80_HUMAN)
P35580	Myosin-10 OS=Homo sapiens GN=MYH10 PE=1 SV=3 - (MYH10_HUMAN)
F5H0C8	Enolase OS=Homo sapiens GN=ENO2 PE=3 SV=1 - (F5H0C8_HUMAN)
F8VZV5	Myosin light polypeptide 6 OS=Homo sapiens GN=MYL6 PE=4 SV=1 - (F8VZV5_HUMAN)
Q8IW70	Transmembrane protein 151B OS=Homo sapiens GN=TMEM151B PE=2 SV=2 - (T151B_HUMAN)
P36543-2	Isoform 2 of V-type proton ATPase subunit E 1 OS=Homo sapiens GN=ATP6V1E1 - (VATE1_HUMAN)
E5RGV0	Heterogeneous nuclear ribonucleoprotein H, N-terminally processed (Fragment) OS=Homo sapiens GN=HNRNPH1 PE=4 SV=1 - (E5RGV0_HUMAN)
F8W7L8	Intersectin-1 OS=Homo sapiens GN=ITSN1 PE=4 SV=1 - (F8W7L8_HUMAN)
Q86YN6-4	Isoform 4 of Peroxisome proliferator-activated receptor gamma coactivator 1-beta OS=Homo sapiens GN=PPARGC1B - (PRGC2_HUMAN)
Q96JB1-2	Isoform 2 of Dynein heavy chain 8, axonemal OS=Homo sapiens GN=DNAH8 - (DYH8_HUMAN)
B4E1M1	cDNA FLJ60391, highly similar to Lactoperoxidase (EC 1.11.1.7) OS=Homo sapiens PE=2 SV=1 - (B4E1M1_HUMAN)
P20592	Interferon-induced GTP-binding protein Mx2 OS=Homo sapiens GN=MX2 PE=1 SV=1 - (MX2_HUMAN)
Q53F18	Homer 1 variant (Fragment) OS=Homo sapiens PE=2 SV=1 - (Q53F18_HUMAN)
A8MXQ7	Putative IQ motif and ankyrin repeat domain-containing protein LOC642574 OS=Homo sapiens PE=5 SV=2 - (YH010_HUMAN)
P42704	Leucine-rich PPR motif-containing protein, mitochondrial OS=Homo sapiens GN=LRPPRC PE=1 SV=3 - (LPPRC_HUMAN)
Q9Y2F5	Uncharacterized protein KIAA0947 OS=Homo sapiens GN=KIAA0947 PE=1 SV=5 - (K0947_HUMAN)
A8K884	cDNA FLJ77178 OS=Homo sapiens PE=2 SV=1 - (A8K884_HUMAN)
Q9Y2D5-4	Isoform 2 of A-kinase anchor protein 2 OS=Homo sapiens GN=AKAP2 - (AKAP2_HUMAN)
P04150-2	Isoform Beta of Glucocorticoid receptor OS=Homo sapiens GN=NR3C1 - (GCR_HUMAN)
P62136-2	Isoform 2 of Serine/threonine-protein phosphatase PP1-alpha catalytic subunit OS=Homo sapiens GN=PPP1CA - (PP1A_HUMAN)
B2R8Z8	cDNA, FLJ94136, highly similar to Homo sapiens synaptotagmin binding, cytoplasmic RNA interacting protein (SYNCRIP), mRNA OS=Homo sapiens PE=2 SV=1 - (B2R8Z8_HUMAN)
B4DMH5	cDNA FLJ55107, highly similar to Cell division control protein 42 homolog OS=Homo sapiens PE=2 SV=1 - (B4DMH5_HUMAN)
D6W5D1	KIAA1212, isoform CRA_a OS=Homo sapiens GN=KIAA1212 PE=4 SV=1 - (D6W5D1_HUMAN)
B9EG55	GRB10 interacting GYF protein 2 OS=Homo sapiens GN=GIGYF2 PE=2 SV=1 - (B9EG55_HUMAN)
P21796	Voltage-dependent anion-selective channel protein 1 OS=Homo sapiens GN=VDAC1 PE=1 SV=2 - (VDAC1_HUMAN)
Q96Q89-4	Isoform 4 of Kinesin-like protein KIF20B OS=Homo sapiens GN=KIF20B - (KI20B_HUMAN)
O96011-2	Isoform 2 of Peroxisomal membrane protein 11B OS=Homo sapiens GN=PEX11B - (PX11B_HUMAN)
Q3SAH0	G-protein-coupled receptor 34 splice variant OS=Homo sapiens GN=GPR34 PE=2 SV=1 - (Q3SAH0_HUMAN)
B8XPJ7	Soluble catechol-O-methyltransferase OS=Homo sapiens GN=COMT PE=2 SV=1 - (B8XPJ7_HUMAN)
B3KX96	cDNA FLJ45003 fis, clone BRAWH3011623, highly similar to Heterogeneous nuclear ribonucleoproteins C OS=Homo sapiens PE=2 SV=1 - (B3KX96_HUMAN)
C9JYJ6	Filamin A-interacting protein 1-like (Fragment) OS=Homo sapiens GN=FILIP1L PE=4 SV=1 - (C9JYJ6_HUMAN)
Q9BYF7	SCCA2b OS=Homo sapiens GN=SCCA2 PE=2 SV=1 - (Q9BYF7_HUMAN)
C9JXB8	60S ribosomal protein L24 OS=Homo sapiens GN=RPL24 PE=4 SV=1 - (C9JXB8_HUMAN)
Q9Y3P9	Rab GTPase-activating protein 1 OS=Homo sapiens GN=RABGAP1 PE=1 SV=3 - (RBGP1_HUMAN)
O60744	Thioredoxin delta 3 (Fragment) OS=Homo sapiens GN=TXN delta 3 PE=2 SV=1 - (O60744_HUMAN)
H0YN65	Carbohydrate sulfotransferase 14 OS=Homo sapiens GN=CHST14 PE=4 SV=1 - (H0YN65_HUMAN)
B7Z3T9	cDNA FLJ53345, highly similar to Leucine-zipper-like transcriptional regulator1 OS=Homo sapiens PE=2 SV=1 - (B7Z3T9_HUMAN)
E0AF97	Cytochrome c oxidase subunit 2 OS=Homo sapiens GN=COX2 PE=3 SV=1 - (E0AF97_HUMAN)
Q9NWH9	SAFB-like transcription modulator OS=Homo sapiens GN=SLTM PE=1 SV=2 - (SLTM_HUMAN)
I3L397	Eukaryotic translation initiation factor 5A-1 (Fragment) OS=Homo sapiens GN=EIF5A PE=4 SV=1 - (I3L397_HUMAN)
Q8IWB6-3	Isoform 3 of Inactive serine/threonine-protein kinase TEX14 OS=Homo sapiens GN=TEX14 - (TEX14_HUMAN)
A8MXJ1	Protein STON1-GTF2A1L OS=Homo sapiens GN=STON1-GTF2A1L PE=4 SV=3 - (A8MXJ1_HUMAN)
Q8IZC4-2	Isoform 2 of Rhotekin-2 OS=Homo sapiens GN=RTKN2 - (RTKN2_HUMAN)
A8MW50	L-lactate dehydrogenase (Fragment) OS=Homo sapiens GN=LDHB PE=3 SV=1 - (A8MW50_HUMAN)
B4DFX6	cDNA FLJ58791, highly similar to Ankyrin repeat domain-containing protein 35 OS=Homo sapiens PE=2 SV=1 - (B4DFX6_HUMAN)
Q71F23-2	Isoform 2 of Centromere protein U OS=Homo sapiens GN=MLF1IP - (CENPU_HUMAN)
H3BMR2	Heparan sulfate glucosamine 3-O-sulfotransferase 2 OS=Homo sapiens GN=HS3ST2 PE=4 SV=1 - (H3BMR2_HUMAN)
Q9H3Q3	Galactose-3-O-sulfotransferase 2 OS=Homo sapiens GN=GAL3ST2 PE=1 SV=2 - (G3ST2_HUMAN)
Q96N67-4	Isoform 4 of Dedicator of cytokinesis protein 7 OS=Homo sapiens GN=DOCK7 - (DOCK7_HUMAN)
P30101	Protein disulfide-isomerase A3 OS=Homo sapiens GN=PDIA3 PE=1 SV=4 - (PDIA3_HUMAN)
Q96LA8	Protein arginine N-methyltransferase 6 OS=Homo sapiens GN=PRMT6 PE=1 SV=1 - (ANM6_HUMAN)
Q86SQ0-2	Isoform 2 of Pleckstrin homology-like domain family B member 2 OS=Homo sapiens GN=PHLDB2 - (PHLB2_HUMAN)
O00186	Syntaxin-binding protein 3 OS=Homo sapiens GN=STXBP3 PE=1 SV=2 - (STXB3_HUMAN)
Q0VF49	Uncharacterized protein KIAA2012 OS=Homo sapiens GN=KIAA2012 PE=2 SV=1 - (K2012_HUMAN)
Q8TAB0	CLOCK protein (Fragment) OS=Homo sapiens GN=clock PE=2 SV=1 - (Q8TAB0_HUMAN)
B3KVR1	Small nuclear ribonucleoprotein-associated protein OS=Homo sapiens GN=SNRPN PE=2 SV=1 - (B3KVR1_HUMAN)
B4DDF9	Annexin OS=Homo sapiens GN=ANXA4 PE=2 SV=1 - (B4DDF9_HUMAN)
Q8TCG1	Protein CIP2A OS=Homo sapiens GN=KIAA1524 PE=1 SV=2 - (CIP2A_HUMAN)
Q9NVR5	Protein kintoun OS=Homo sapiens GN=DNAAF2 PE=1 SV=2 - (KTU_HUMAN)
Q8NE70	Retinoblastoma-like 2 (P130) OS=Homo sapiens GN=RBL2 PE=2 SV=1 - (Q8NE70_HUMAN)
C9K0X2	Uncharacterized protein OS=Homo sapiens PE=4 SV=1 - (C9K0X2_HUMAN)
Q05DB9	PAPOLG protein (Fragment) OS=Homo sapiens GN=PAPOLG PE=2 SV=1 - (Q05DB9_HUMAN)
A0MZ66	Shootin-1 OS=Homo sapiens GN=KIAA1598 PE=1 SV=4 - (SHOT1_HUMAN)
A6NJZ7	RIMS-binding protein 3C OS=Homo sapiens GN=RIMBP3C PE=1 SV=3 - (RIM3C_HUMAN)
Q96L21	60S ribosomal protein L10-like OS=Homo sapiens GN=RPL10L PE=1 SV=3 - (RL10L_HUMAN)
F8VU65	60S acidic ribosomal protein P0 (Fragment) OS=Homo sapiens GN=RPLP0 PE=3 SV=1 - (F8VU65_HUMAN)
I3L2P8	Protein disulfide-isomerase OS=Homo sapiens GN=P4HB PE=4 SV=1 - (I3L2P8_HUMAN)
Q6NXR8	Ribosomal protein S3A OS=Homo sapiens GN=RPS3A PE=2 SV=1 - (Q6NXR8_HUMAN)
P50395-2	Isoform 2 of Rab GDP dissociation inhibitor beta OS=Homo sapiens GN=GDI2 - (GDIB_HUMAN)
H0Y4M1	EF-hand calcium-binding domain-containing protein 5 OS=Homo sapiens GN=EFCAB5 PE=4 SV=1 - (H0Y4M1_HUMAN)
Q6ZV29-3	Isoform 3 of Patatin-like phospholipase domain-containing protein 7 OS=Homo sapiens GN=PNPLA7 - (PLPL7_HUMAN)
Q8WUQ7	Uncharacterized protein C19orf29 OS=Homo sapiens GN=C19orf29 PE=1 SV=3 - (CS029_HUMAN)
B7ZLP0	DOCK8 protein OS=Homo sapiens GN=DOCK8 PE=2 SV=1 - (B7ZLP0_HUMAN)
H0YG24	Coiled-coil domain-containing protein 151 OS=Homo sapiens GN=CCDC151 PE=4 SV=1 - (H0YG24_HUMAN)
A7BI36	p180/ribosome receptor OS=Homo sapiens GN=RRBP1 PE=2 SV=2 - (A7BI36_HUMAN)
Q7KZ85	Transcription elongation factor SPT6 OS=Homo sapiens GN=SUPT6H PE=1 SV=2 - (SPT6H_HUMAN)
Q92830	Histone acetyltransferase KAT2A OS=Homo sapiens GN=KAT2A PE=1 SV=3 - (KAT2A_HUMAN)
Q5T0Z8	Uncharacterized protein C6orf132 OS=Homo sapiens GN=C6orf132 PE=1 SV=4 - (CF132_HUMAN)
F8W8F5	Calpain-3 OS=Homo sapiens GN=CAPN3 PE=4 SV=1 - (F8W8F5_HUMAN)
H7C0E8	Probable phospholipid-transporting ATPase IG (Fragment) OS=Homo sapiens GN=ATP11C PE=4 SV=1 - (H7C0E8_HUMAN)
Q9UJL9	Zinc finger protein 643 OS=Homo sapiens GN=ZNF643 PE=2 SV=2 - (ZN643_HUMAN)
Q9UKK3	Poly (ADP-ribose) polymerase 4 OS=Homo sapiens GN=PARP4 PE=1 SV=3 - (PARP4_HUMAN)
Q9UJX2	Cell division cycle protein 23 homolog OS=Homo sapiens GN=CDC23 PE=1 SV=3 - (CDC23_HUMAN)
A8K2Q8	Serpin peptidase inhibitor, clade B (Ovalbumin), member 13, isoform CRA_d OS=Homo sapiens GN=SERPINB13 PE=2 SV=1 - (A8K2Q8_HUMAN)
E7ENJ7	Translin-associated factor X-interacting protein 1 OS=Homo sapiens GN=TSNAXIP1 PE=4 SV=1 - (E7ENJ7_HUMAN)
P11047	Laminin subunit gamma-1 OS=Homo sapiens GN=LAMC1 PE=1 SV=3 - (LAMC1_HUMAN)
Q96FL8	Multidrug and toxin extrusion protein 1 OS=Homo sapiens GN=SLC47A1 PE=1 SV=1 - (S47A1_HUMAN)
P12111-2	Isoform 2 of Collagen alpha-3(VI) chain OS=Homo sapiens GN=COL6A3 - (CO6A3_HUMAN)
B4DE24	cDNA FLJ52155, moderately similar to Rattus norvegicus ring finger protein 34 (Rnf34), mRNA OS=Homo sapiens PE=2 SV=1 - (B4DE24_HUMAN)
B3KX72	Heterogeneous nuclear ribonucleoprotein U OS=Homo sapiens GN=HNRNPU PE=2 SV=1 - (B3KX72_HUMAN)
A4GWZ0	MHC class I antigen (Fragment) OS=Homo sapiens GN=HLA-A PE=3 SV=1 - (A4GWZ0_HUMAN)
C9J1I8	Transcription factor TFIIIB component B'' homolog OS=Homo sapiens GN=BDP1 PE=4 SV=2 - (C9J1I8_HUMAN)
B4DMK0	cDNA FLJ61134, highly similar to Ras-related protein Rab-11B OS=Homo sapiens PE=2 SV=1 - (B4DMK0_HUMAN)
A8MYX0	Protein diaphanous homolog 3 OS=Homo sapiens GN=DIAPH3 PE=4 SV=3 - (A8MYX0_HUMAN)
H0YMZ1	Proteasome subunit alpha type (Fragment) OS=Homo sapiens GN=PSMA4 PE=3 SV=1 - (H0YMZ1_HUMAN)
O95229	ZW10 interactor OS=Homo sapiens GN=ZWINT PE=1 SV=2 - (ZWINT_HUMAN)
Q96M42	Putative uncharacterized protein encoded by LINC00479 OS=Homo sapiens GN=LINC00479 PE=5 SV=2 - (CU129_HUMAN)
B4DXW2	cDNA FLJ60947, highly similar to Coiled-coil domain-containing protein 9 OS=Homo sapiens PE=2 SV=1 - (B4DXW2_HUMAN)
D6REM6	Matrin-3 OS=Homo sapiens GN=MATR3 PE=4 SV=1 - (D6REM6_HUMAN)
Q96L12	Calreticulin-3 OS=Homo sapiens GN=CALR3 PE=1 SV=2 - (CALR3_HUMAN)
Q5SW79	Centrosomal protein of 170 kDa OS=Homo sapiens GN=CEP170 PE=1 SV=1 - (CE170_HUMAN)
Q6IPM2-4	Isoform 4 of IQ domain-containing protein E OS=Homo sapiens GN=IQCE - (IQCE_HUMAN)
A4D218	MAD1 mitotic arrest deficient-like 1 (Yeast) OS=Homo sapiens GN=MAD1L1 PE=4 SV=1 - (A4D218_HUMAN)
Q86UK0	ATP-binding cassette sub-family A member 12 OS=Homo sapiens GN=ABCA12 PE=1 SV=3 - (ABCAC_HUMAN)
P06753	Tropomyosin alpha-3 chain OS=Homo sapiens GN=TPM3 PE=1 SV=1 - (TPM3_HUMAN)
A7KAX9	Rho GTPase-activating protein 32 OS=Homo sapiens GN=ARHGAP32 PE=1 SV=1 - (RHG32_HUMAN)
Q68D86	Coiled-coil domain-containing protein 102B OS=Homo sapiens GN=CCDC102B PE=2 SV=4 - (C102B_HUMAN)
D3DRN8	Putative uncharacterized protein LOC158381 (Fragment) OS=Homo sapiens GN=LOC158381 PE=4 SV=1 - (D3DRN8_HUMAN)
Q99797	Mitochondrial intermediate peptidase OS=Homo sapiens GN=MIPEP PE=1 SV=2 - (MIPEP_HUMAN)
Q9ULZ3-2	Isoform 2 of Apoptosis-associated speck-like protein containing a CARD OS=Homo sapiens GN=PYCARD - (ASC_HUMAN)
P62913-2	Isoform 2 of 60S ribosomal protein L11 OS=Homo sapiens GN=RPL11 - (RL11_HUMAN)
H0YHU6	Uncharacterized protein OS=Homo sapiens PE=4 SV=1 - (H0YHU6_HUMAN)
B4E343	cDNA FLJ54249, highly similar to U3 small nucleolar RNA-associated protein 14 homolog A OS=Homo sapiens PE=2 SV=1 - (B4E343_HUMAN)
Q6QEF7	Breast cancer anti-estrogen resistance 1 OS=Homo sapiens GN=BCAR1 PE=2 SV=1 - (Q6QEF7_HUMAN)
Q8NEM0	Microcephalin OS=Homo sapiens GN=MCPH1 PE=1 SV=3 - (MCPH1_HUMAN)
P62847-2	Isoform 2 of 40S ribosomal protein S24 OS=Homo sapiens GN=RPS24 - (RS24_HUMAN)
Q96L91-4	Isoform 4 of E1A-binding protein p400 OS=Homo sapiens GN=EP400 - (EP400_HUMAN)
P08195-2	Isoform 2 of 4F2 cell-surface antigen heavy chain OS=Homo sapiens GN=SLC3A2 - (4F2_HUMAN)
Q9Y4W2-3	Isoform 3 of Ribosomal biogenesis protein LAS1L OS=Homo sapiens GN=LAS1L - (LAS1L_HUMAN)
Q9UMN6	Histone-lysine N-methyltransferase MLL4 OS=Homo sapiens GN=WBP7 PE=1 SV=1 - (MLL4_HUMAN)
Q8NHS4-2	Isoform 2 of Uncharacterized protein C2orf63 OS=Homo sapiens GN=C2orf63 - (CB063_HUMAN)
G3XAM7	Catenin (Cadherin-associated protein), alpha 1, 102kDa, isoform CRA_a OS=Homo sapiens GN=CTNNA1 PE=4 SV=1 - (G3XAM7_HUMAN)
Q14997	Proteasome activator complex subunit 4 OS=Homo sapiens GN=PSME4 PE=1 SV=2 - (PSME4_HUMAN)
Q14191	Werner syndrome ATP-dependent helicase OS=Homo sapiens GN=WRN PE=1 SV=2 - (WRN_HUMAN)
H7BY10	60S ribosomal protein L23a (Fragment) OS=Homo sapiens GN=RPL23A PE=3 SV=1 - (H7BY10_HUMAN)
B7Z848	DNA-binding protein RFX5 OS=Homo sapiens GN=RFX5 PE=2 SV=1 - (B7Z848_HUMAN)
Q9BT92	Trichoplein keratin filament-binding protein OS=Homo sapiens GN=TCHP PE=1 SV=1 - (TCHP_HUMAN)
H0Y5J4	Drebrin-like protein (Fragment) OS=Homo sapiens GN=DBNL PE=4 SV=1 - (H0Y5J4_HUMAN)
B3KXY9	cDNA FLJ46359 fis, clone TESTI4049786, highly similar to Hexokinase-1 (EC 2.7.1.1) OS=Homo sapiens PE=2 SV=1 - (B3KXY9_HUMAN)
B4DNP0	cDNA FLJ59362, highly similar to Signal transducer and activator of transcription 3 OS=Homo sapiens PE=2 SV=1 - (B4DNP0_HUMAN)
Q53SF7-2	Isoform 2 of Cordon-bleu protein-like 1 OS=Homo sapiens GN=COBLL1 - (COBL1_HUMAN)
G3V533	Nesprin-3 OS=Homo sapiens GN=C14orf49 PE=4 SV=1 - (G3V533_HUMAN)
Q8IUC6	TIR domain-containing adapter molecule 1 OS=Homo sapiens GN=TICAM1 PE=1 SV=1 - (TCAM1_HUMAN)
Q5T024	Centrosomal protein C10orf90 OS=Homo sapiens GN=RP11-422P15.2 PE=2 SV=1 - (Q5T024_HUMAN)
G5EA45	Disheveled-associated activator of morphogenesis 2 OS=Homo sapiens GN=DAAM2 PE=4 SV=1 - (G5EA45_HUMAN)
C9JTA6	SWI/SNF-related matrix-associated actin-dependent regulator of chromatin subfamily B member 1 (Fragment) OS=Homo sapiens GN=SMARCB1 PE=4 SV=1 - (C9JTA6_HUMAN)
B3KRS1	cDNA FLJ34794 fis, clone NT2NE2005676, highly similar to Gamma-tubulin complex component 2 OS=Homo sapiens PE=2 SV=1 - (B3KRS1_HUMAN)
A8MWV3	Osteoclast-associated immunoglobulin-like receptor OS=Homo sapiens GN=OSCAR PE=4 SV=1 - (A8MWV3_HUMAN)
P98161-2	Isoform 2 of Polycystin-1 OS=Homo sapiens GN=PKD1 - (PKD1_HUMAN)
B4DEA8	cDNA FLJ56425, highly similar to Very-long-chain specific acyl-CoAdehydrogenase, mitochondrial (EC 1.3.99.-) OS=Homo sapiens PE=2 SV=1 - (B4DEA8_HUMAN)
F5GX67	Keratin-associated protein 1-5 OS=Homo sapiens GN=KRTAP1-5 PE=4 SV=1 - (F5GX67_HUMAN)
H0YL91	Activating signal cointegrator 1 OS=Homo sapiens GN=TRIP4 PE=4 SV=1 - (H0YL91_HUMAN)
Q8NEY1-2	Isoform 2 of Neuron navigator 1 OS=Homo sapiens GN=NAV1 - (NAV1_HUMAN)
B2R608	cDNA, FLJ92718, highly similar to Homo sapiens tripeptidyl peptidase I (TPP1), mRNA OS=Homo sapiens PE=2 SV=1 - (B2R608_HUMAN)
B2R983	cDNA, FLJ94267, highly similar to Homo sapiens glutathione S-transferase omega 1 (GSTO1), mRNA OS=Homo sapiens PE=2 SV=1 - (B2R983_HUMAN)
Q5T0V4	Slit homolog 1 (Drosophila) (Fragment) OS=Homo sapiens GN=SLIT1 PE=2 SV=1 - (Q5T0V4_HUMAN)
A8K8P3-10	Isoform 10 of Protein SFI1 homolog OS=Homo sapiens GN=SFI1 - (SFI1_HUMAN)
Q9P153	PRO2729 OS=Homo sapiens PE=2 SV=1 - (Q9P153_HUMAN)
Q59G27	Stathmin (Fragment) OS=Homo sapiens PE=2 SV=1 - (Q59G27_HUMAN)
B3KXQ9	cDNA FLJ45894 fis, clone OCBBF3023913, highly similar to R3H domain-containing protein 1 OS=Homo sapiens PE=2 SV=1 - (B3KXQ9_HUMAN)
P20290	Transcription factor BTF3 OS=Homo sapiens GN=BTF3 PE=1 SV=1 - (BTF3_HUMAN)
P10266	HERV-K_5q33.3 provirus ancestral Pol protein OS=Homo sapiens PE=3 SV=2 - (POK10_HUMAN)
Q9NY97	UDP-GlcNAc:betaGal beta-1,3-N-acetylglucosaminyltransferase 2 OS=Homo sapiens GN=B3GNT2 PE=1 SV=2 - (B3GN2_HUMAN)
Q9Y6N8	Cadherin-10 OS=Homo sapiens GN=CDH10 PE=1 SV=2 - (CAD10_HUMAN)
Q07DS4	Envelope glycoprotein OS=Homo sapiens GN=env PE=4 SV=1 - (Q07DS4_HUMAN)
Q8TAS0	ATP synthase gamma chain (Fragment) OS=Homo sapiens PE=2 SV=1 - (Q8TAS0_HUMAN)
H0YH34	ADP-ribosylation factor-like protein 13A (Fragment) OS=Homo sapiens GN=ARL13A PE=4 SV=1 - (H0YH34_HUMAN)
H7BXN2	Heterogeneous nuclear ribonucleoprotein D0 OS=Homo sapiens GN=HNRNPD PE=4 SV=1 - (H7BXN2_HUMAN)
Q7Z5A3	Small nuclear ribonucleoprotein Sm D1 OS=Homo sapiens GN=SNRPD1 PE=2 SV=1 - (Q7Z5A3_HUMAN)
Q15008	26S proteasome non-ATPase regulatory subunit 6 OS=Homo sapiens GN=PSMD6 PE=1 SV=1 - (PSMD6_HUMAN)
Q8N1G2	Cap-specific mRNA (nucleoside-2'-O-)-methyltransferase 1 OS=Homo sapiens GN=FTSJD2 PE=1 SV=1 - (MTR1_HUMAN)
Q96E11-7	Isoform 7 of Ribosome-recycling factor, mitochondrial OS=Homo sapiens GN=MRRF - (RRFM_HUMAN)
P28702	Retinoic acid receptor RXR-beta OS=Homo sapiens GN=RXRB PE=1 SV=2 - (RXRB_HUMAN)
Q6AI18	Putative uncharacterized protein DKFZp686M148 (Fragment) OS=Homo sapiens GN=DKFZp686M148 PE=2 SV=1 - (Q6AI18_HUMAN)
B4DPW9	cDNA FLJ51929, highly similar to Plastin-3 OS=Homo sapiens PE=2 SV=1 - (B4DPW9_HUMAN)
C9J592	Ras-related protein Rab-7a (Fragment) OS=Homo sapiens GN=RAB7A PE=3 SV=1 - (C9J592_HUMAN)
B1AHC7	X-ray repair complementing defective repair in Chinese hamster cells 6 (Ku autoantigen, 70kDa) OS=Homo sapiens GN=XRCC6 PE=4 SV=1 - (B1AHC7_HUMAN)
B2RBF9	cDNA, FLJ95487, highly similar to Homo sapiens peptidyl arginine deiminase, type I (PADI1), mRNA OS=Homo sapiens PE=2 SV=1 - (B2RBF9_HUMAN)
Q92928	Putative Ras-related protein Rab-1C OS=Homo sapiens GN=RAB1C PE=5 SV=2 - (RAB1C_HUMAN)
Q9UGU0-2	Isoform 2 of Transcription factor 20 OS=Homo sapiens GN=TCF20 - (TCF20_HUMAN)
Q9P0L2	Serine/threonine-protein kinase MARK1 OS=Homo sapiens GN=MARK1 PE=1 SV=2 - (MARK1_HUMAN)
P15822	Zinc finger protein 40 OS=Homo sapiens GN=HIVEP1 PE=1 SV=3 - (ZEP1_HUMAN)
Q86YL3	INM04 OS=Homo sapiens PE=2 SV=1 - (Q86YL3_HUMAN)
B2RBL3	cDNA, FLJ95575, highly similar to Homo sapiens endothelial cell growth factor 1 (platelet-derived) (ECGF1), mRNA OS=Homo sapiens PE=2 SV=1 - (B2RBL3_HUMAN)
Q9Y5H0	Protocadherin gamma-A3 OS=Homo sapiens GN=PCDHGA3 PE=2 SV=2 - (PCDG3_HUMAN)
Q9BYR2	Keratin-associated protein 4-5 OS=Homo sapiens GN=KRTAP4-5 PE=2 SV=4 - (KRA45_HUMAN)
B4DY09	cDNA FLJ51660, highly similar to Interleukin enhancer-binding factor 2 OS=Homo sapiens PE=2 SV=1 - (B4DY09_HUMAN)
F8VSZ4	Plexin-A1 OS=Homo sapiens GN=PLXNA1 PE=4 SV=1 - (F8VSZ4_HUMAN)
A8K8P1	cDNA FLJ78752, highly similar to Homo sapiens centromere protein J (CENPJ), mRNA OS=Homo sapiens PE=2 SV=1 - (A8K8P1_HUMAN)
Q13535-2	Isoform 2 of Serine/threonine-protein kinase ATR OS=Homo sapiens GN=ATR - (ATR_HUMAN)
Q7Z353	Highly divergent homeobox OS=Homo sapiens GN=HDX PE=1 SV=1 - (HDX_HUMAN)
B4DPU7	cDNA FLJ53623, moderately similar to Homo sapiens cytidylate kinase (CMPK), mRNA OS=Homo sapiens PE=2 SV=1 - (B4DPU7_HUMAN)
D6RD18	Heterogeneous nuclear ribonucleoprotein A/B OS=Homo sapiens GN=HNRNPAB PE=4 SV=1 - (D6RD18_HUMAN)
E7ETU9	Procollagen-lysine,2-oxoglutarate 5-dioxygenase 2 OS=Homo sapiens GN=PLOD2 PE=4 SV=1 - (E7ETU9_HUMAN)
B7Z948	cDNA FLJ53589, highly similar to Tyrosine-protein phosphatase non-receptor type3 (EC 3.1.3.48) OS=Homo sapiens PE=2 SV=1 - (B7Z948_HUMAN)
A8K781	cDNA FLJ75299, highly similar to Xenopus laevis proteasome (prosome, macropain) 26S subunit, ATPase 3, mRNA OS=Homo sapiens PE=2 SV=1 - (A8K781_HUMAN)
Q16630	Cleavage and polyadenylation specificity factor subunit 6 OS=Homo sapiens GN=CPSF6 PE=1 SV=2 - (CPSF6_HUMAN)
B4DJV9	cDNA FLJ60607, highly similar to Acyl-protein thioesterase 1 (EC 3.1.2.-) OS=Homo sapiens PE=2 SV=1 - (B4DJV9_HUMAN)
Q9P265	Disco-interacting protein 2 homolog B OS=Homo sapiens GN=DIP2B PE=1 SV=3 - (DIP2B_HUMAN)
P46779-2	Isoform 2 of 60S ribosomal protein L28 OS=Homo sapiens GN=RPL28 - (RL28_HUMAN)
H0Y9N1	Programmed cell death protein 6 (Fragment) OS=Homo sapiens GN=PDCD6 PE=4 SV=1 - (H0Y9N1_HUMAN)
A4QN25	NBPF6 protein (Fragment) OS=Homo sapiens GN=NBPF6 PE=2 SV=1 - (A4QN25_HUMAN)
Q7L5L3-1	Isoform 2 of Glycerophosphodiester phosphodiesterase domain-containing protein 3 OS=Homo sapiens GN=GDPD3 - (GDPD3_HUMAN)
I3L4X2	Multidrug resistance-associated protein 1 (Fragment) OS=Homo sapiens GN=ABCC1 PE=4 SV=1 - (I3L4X2_HUMAN)
B4DW36	cDNA FLJ58862, highly similar to THUMP domain-containing protein 2 OS=Homo sapiens PE=2 SV=1 - (B4DW36_HUMAN)
G3V1B3	60S ribosomal protein L21 OS=Homo sapiens GN=RPL21 PE=4 SV=1 - (G3V1B3_HUMAN)
Q5T8Y7	COMM domain containing 3 (Fragment) OS=Homo sapiens GN=COMMD3 PE=2 SV=1 - (Q5T8Y7_HUMAN)
H7C2W9	60S ribosomal protein L31 (Fragment) OS=Homo sapiens GN=RPL31 PE=4 SV=1 - (H7C2W9_HUMAN)
Q8WXC6-1	Isoform 2 of Myeloma-overexpressed gene 2 protein OS=Homo sapiens GN=MYEOV2 - (MYOV2_HUMAN)
F8W1S7	Heterogeneous nuclear ribonucleoprotein A1 OS=Homo sapiens GN=HNRNPA1 PE=4 SV=1 - (F8W1S7_HUMAN)
B4E2V4	Proteasome (Prosome, macropain) subunit, alpha type, 5, isoform CRA_c OS=Homo sapiens GN=PSMA5 PE=2 SV=1 - (B4E2V4_HUMAN)
F8W651	Coatomer subunit zeta-1 OS=Homo sapiens GN=COPZ1 PE=4 SV=1 - (F8W651_HUMAN)
Q6GX22	Midline 2 OS=Homo sapiens GN=MID2 PE=2 SV=1 - (Q6GX22_HUMAN)
B3KUI2	cDNA FLJ39965 fis, clone SPLEN2027157, highly similar to Homo sapiens tumor necrosis factor, alpha-induced protein 8 (TNFAIP8), mRNA OS=Homo sapiens PE=2 SV=1 - (B3KUI2_HUMAN)
O43594	Ku86 autoantigen related protein 1 (Fragment) OS=Homo sapiens GN=KARP-1 PE=1 SV=1 - (O43594_HUMAN)
P18510-2	Isoform 2 of Interleukin-1 receptor antagonist protein OS=Homo sapiens GN=IL1RN - (IL1RA_HUMAN)
B4DKX4	cDNA FLJ58014, highly similar to Homo sapiens programmed cell death 4, transcript variant 1, mRNA OS=Homo sapiens PE=2 SV=1 - (B4DKX4_HUMAN)
Q9BQQ5	Ribosomal protein L27a OS=Homo sapiens GN=L27a PE=2 SV=1 - (Q9BQQ5_HUMAN)
A8K1B1	cDNA FLJ75211, highly similar to Homo sapiens ubiquitin specific peptidase like 1, mRNA OS=Homo sapiens PE=2 SV=1 - (A8K1B1_HUMAN)
H0YB22	40S ribosomal protein S14 (Fragment) OS=Homo sapiens GN=RPS14 PE=4 SV=1 - (H0YB22_HUMAN)
Q96PI1	Small proline-rich protein 4 OS=Homo sapiens GN=SPRR4 PE=2 SV=1 - (SPRR4_HUMAN)
Q6NWZ1	CKAP4 protein (Fragment) OS=Homo sapiens GN=CKAP4 PE=2 SV=1 - (Q6NWZ1_HUMAN)
B4DN59	cDNA FLJ52702, highly similar to Homo sapiens CD44 antigen (homing function and Indian blood group system) (CD44), transcript variant 4, mRNA OS=Homo sapiens PE=2 SV=1 - (B4DN59_HUMAN)
E9PDE0	Uncharacterized protein KIAA1683 OS=Homo sapiens GN=KIAA1683 PE=4 SV=1 - (E9PDE0_HUMAN)
Q5T123	SH3 domain binding glutamic acid-rich protein like 3 OS=Homo sapiens GN=SH3BGRL3 PE=2 SV=1 - (Q5T123_HUMAN)
E9PE88	cGMP-dependent 3',5'-cyclic phosphodiesterase OS=Homo sapiens GN=PDE2A PE=4 SV=2 - (E9PE88_HUMAN)
Q5H9J9	Novel protein similar to t-complex 11 homolog (Mouse) TCP11 OS=Homo sapiens GN=LL0XNC01-19D8.1 PE=4 SV=1 - (Q5H9J9_HUMAN)
H7BZ60	Protein kinase C gamma type (Fragment) OS=Homo sapiens GN=PRKCG PE=4 SV=1 - (H7BZ60_HUMAN)
B2R761	cDNA, FLJ93299, highly similar to Homo sapiens sterol carrier protein 2 (SCP2), mRNA OS=Homo sapiens PE=2 SV=1 - (B2R761_HUMAN)
P27824	Calnexin OS=Homo sapiens GN=CANX PE=1 SV=2 - (CALX_HUMAN)
Q12906	Interleukin enhancer-binding factor 3 OS=Homo sapiens GN=ILF3 PE=1 SV=3 - (ILF3_HUMAN)
B4E0B1	cDNA FLJ52100 OS=Homo sapiens PE=2 SV=1 - (B4E0B1_HUMAN)
P10909-4	Isoform 4 of Clusterin OS=Homo sapiens GN=CLU - (CLUS_HUMAN)
Q9BPX5	Actin-related protein 2/3 complex subunit 5-like protein OS=Homo sapiens GN=ARPC5L PE=1 SV=1 - (ARP5L_HUMAN)
Q5HYC5	Putative uncharacterized protein DKFZp686L08115 (Fragment) OS=Homo sapiens GN=DKFZp686L08115 PE=2 SV=1 - (Q5HYC5_HUMAN)
B4DYI3	cDNA FLJ57061, highly similar to NADH-ubiquinone oxidoreductase 23 kDa subunit, mitochondrial (EC 1.6.5.3) OS=Homo sapiens PE=2 SV=1 - (B4DYI3_HUMAN)
B4E1M5	60S ribosomal protein L9 OS=Homo sapiens GN=RPL9 PE=2 SV=1 - (B4E1M5_HUMAN)
G5E9Y3	Arf-GAP with Rho-GAP domain, ANK repeat and PH domain-containing protein 3 OS=Homo sapiens GN=ARAP3 PE=4 SV=1 - (G5E9Y3_HUMAN)
B2R862	cDNA, FLJ93754, highly similar to Homo sapiens tripartite motif-containing 38 (TRIM38), mRNA OS=Homo sapiens PE=2 SV=1 - (B2R862_HUMAN)
F8WCF6	Actin-related protein 2/3 complex subunit 4 OS=Homo sapiens GN=ARPC4 PE=4 SV=1 - (F8WCF6_HUMAN)
Q8NHY3	GAS2-like protein 2 OS=Homo sapiens GN=GAS2L2 PE=2 SV=1 - (GA2L2_HUMAN)
B3KPG6	cDNA FLJ31769 fis, clone NT2RI2007956, highly similar to Chondroitin sulfate glucuronyltransferase (EC 2.4.1.226) OS=Homo sapiens PE=2 SV=1 - (B3KPG6_HUMAN)
B4E190	cDNA FLJ57770, moderately similar to ADP-ribosylation factor 3 OS=Homo sapiens PE=2 SV=1 - (B4E190_HUMAN)
E9PF10	Nuclear pore complex protein Nup155 OS=Homo sapiens GN=NUP155 PE=4 SV=1 - (E9PF10_HUMAN)
Q53GD1	Guanine nucleotide-binding protein subunit gamma (Fragment) OS=Homo sapiens PE=2 SV=1 - (Q53GD1_HUMAN)
E9PI54	DNA repair protein RAD51 homolog 1 OS=Homo sapiens GN=RAD51 PE=4 SV=1 - (E9PI54_HUMAN)
O60658-2	Isoform 2 of High affinity cAMP-specific and IBMX-insensitive 3',5'-cyclic phosphodiesterase 8A OS=Homo sapiens GN=PDE8A - (PDE8A_HUMAN)
O95601	Mih1/Tx protein OS=Homo sapiens GN=mih1/Tx PE=2 SV=1 - (O95601_HUMAN)
F4NBY2	MHC class I antigen (Fragment) OS=Homo sapiens GN=HLA-B PE=3 SV=1 - (F4NBY2_HUMAN)
Q96C19	EF-hand domain-containing protein D2 OS=Homo sapiens GN=EFHD2 PE=1 SV=1 - (EFHD2_HUMAN)
P61081	NEDD8-conjugating enzyme Ubc12 OS=Homo sapiens GN=UBE2M PE=1 SV=1 - (UBC12_HUMAN)
Q6UXL0	Interleukin-20 receptor subunit beta OS=Homo sapiens GN=IL20RB PE=1 SV=1 - (I20RB_HUMAN)
B7Z3I7	Malate dehydrogenase OS=Homo sapiens PE=2 SV=1 - (B7Z3I7_HUMAN)
Q8WXF0	Serine/arginine-rich splicing factor 12 OS=Homo sapiens GN=SRSF12 PE=1 SV=1 - (SRS12_HUMAN)
C9J1E7	AP-1 complex subunit beta-1 (Fragment) OS=Homo sapiens GN=AP1B1 PE=4 SV=1 - (C9J1E7_HUMAN)
E7EPR8	Breast carcinoma-amplified sequence 1 OS=Homo sapiens GN=BCAS1 PE=4 SV=1 - (E7EPR8_HUMAN)
Q9UNN8	Endothelial protein C receptor OS=Homo sapiens GN=PROCR PE=1 SV=1 - (EPCR_HUMAN)
Q9NQ94-2	Isoform 2 of APOBEC1 complementation factor OS=Homo sapiens GN=A1CF - (A1CF_HUMAN)
O14497-2	Isoform 2 of AT-rich interactive domain-containing protein 1A OS=Homo sapiens GN=ARID1A - (ARI1A_HUMAN)
Q05CV8	PRPF39 protein (Fragment) OS=Homo sapiens GN=PRPF39 PE=2 SV=1 - (Q05CV8_HUMAN)
Q5T8U3	60S ribosomal protein L7a (Fragment) OS=Homo sapiens GN=RPL7A PE=4 SV=1 - (Q5T8U3_HUMAN)
Q5UBV6	NIN/PDGFRB fusion protein (Fragment) OS=Homo sapiens GN=NIN/PDGFRB fusion PE=2 SV=1 - (Q5UBV6_HUMAN)
Q5MCW4	Zinc finger protein 569 OS=Homo sapiens GN=ZNF569 PE=2 SV=1 - (ZN569_HUMAN)
B7Z993	Zinc finger protein 418 OS=Homo sapiens GN=ZNF418 PE=2 SV=1 - (B7Z993_HUMAN)
Q53FL7	Vascular cell adhesion molecule 1 isoform a variant (Fragment) OS=Homo sapiens PE=2 SV=1 - (Q53FL7_HUMAN)
Q9P1U1-3	Isoform 3 of Actin-related protein 3B OS=Homo sapiens GN=ACTR3B - (ARP3B_HUMAN)
Q9P1W3	Transmembrane protein 63C OS=Homo sapiens GN=TMEM63C PE=2 SV=1 - (TM63C_HUMAN)
E7EQY4	Metastasis-associated protein MTA3 OS=Homo sapiens GN=MTA3 PE=4 SV=1 - (E7EQY4_HUMAN)
Q5JW98	Protein FAM26D OS=Homo sapiens GN=FAM26D PE=1 SV=1 - (FA26D_HUMAN)
E5RGF0	Gamma-glutamyl hydrolase (Fragment) OS=Homo sapiens GN=GGH PE=4 SV=1 - (E5RGF0_HUMAN)
A1L3A9	TBC1 domain family, member 9B (With GRAM domain) OS=Homo sapiens GN=TBC1D9B PE=2 SV=1 - (A1L3A9_HUMAN)
Q8TDY8	Immunoglobulin superfamily DCC subclass member 4 OS=Homo sapiens GN=IGDCC4 PE=2 SV=1 - (IGDC4_HUMAN)
O15054	Lysine-specific demethylase 6B OS=Homo sapiens GN=KDM6B PE=1 SV=4 - (KDM6B_HUMAN)
Q8IU60	m7GpppN-mRNA hydrolase OS=Homo sapiens GN=DCP2 PE=1 SV=2 - (DCP2_HUMAN)
Q2NLA0	Coatomer subunit gamma OS=Homo sapiens GN=COPG2 PE=2 SV=1 - (Q2NLA0_HUMAN)
Q5JXB2	Putative ubiquitin-conjugating enzyme E2 N-like OS=Homo sapiens GN=UBE2NL PE=5 SV=1 - (UE2NL_HUMAN)
O75154	Rab11 family-interacting protein 3 OS=Homo sapiens GN=RAB11FIP3 PE=1 SV=1 - (RFIP3_HUMAN)
Q8N7Y3	cDNA FLJ40219 fis, clone TESTI2021637, moderately similar to LIVER CARBOXYLESTERASE (EC 3.1.1.1) OS=Homo sapiens PE=2 SV=1 - (Q8N7Y3_HUMAN)
A8K5A5	cDNA FLJ75393, highly similar to Homo sapiens PHD finger protein 16 (PHF16), mRNA OS=Homo sapiens PE=2 SV=1 - (A8K5A5_HUMAN)
D9MWM3	Soluble Fc mu receptor OS=Homo sapiens GN=FCMR PE=2 SV=1 - (D9MWM3_HUMAN)
Q96PT7	Integrin beta 2 (Fragment) OS=Homo sapiens GN=ITGB2 PE=2 SV=1 - (Q96PT7_HUMAN)
Q8NFQ5	BPI fold-containing family B member 6 OS=Homo sapiens GN=BPIFB6 PE=2 SV=1 - (BPIB6_HUMAN)
B7Z5E7	cDNA FLJ51046, highly similar to 60 kDa heat shock protein, mitochondrial OS=Homo sapiens PE=2 SV=1 - (B7Z5E7_HUMAN)
Q8NFU7	Methylcytosine dioxygenase TET1 OS=Homo sapiens GN=TET1 PE=1 SV=2 - (TET1_HUMAN)
Q5JPJ9	Putative uncharacterized protein DKFZp686D0114 OS=Homo sapiens GN=DKFZp686D0114 PE=2 SV=1 - (Q5JPJ9_HUMAN)
Q5HYE3	Putative uncharacterized protein DKFZp686H1812 OS=Homo sapiens GN=DKFZp686H1812 PE=2 SV=1 - (Q5HYE3_HUMAN)
B2R679	cDNA, FLJ92825, highly similar to Homo sapiens SAR1a gene homolog 1 (S. cerevisiae) (SARA1), mRNA OS=Homo sapiens PE=2 SV=1 - (B2R679_HUMAN)
E9PCW0	Prohibitin OS=Homo sapiens GN=PHB PE=4 SV=1 - (E9PCW0_HUMAN)
E1V2K1	MHC class II antigen (Fragment) OS=Homo sapiens GN=HLA-DRB1 PE=4 SV=1 - (E1V2K1_HUMAN)
Q70V32	ABO glycosyltransferase (Fragment) OS=Homo sapiens GN=ABO PE=4 SV=1 - (Q70V32_HUMAN)
B4DJV2	Citrate synthase OS=Homo sapiens GN=CS PE=2 SV=1 - (B4DJV2_HUMAN)
Q9UKB3	DnaJ homolog subfamily C member 12 OS=Homo sapiens GN=DNAJC12 PE=1 SV=1 - (DJC12_HUMAN)
Q86XR8-4	Isoform 4 of Centrosomal protein of 57 kDa OS=Homo sapiens GN=CEP57 - (CEP57_HUMAN)
E9PRA1	Putative deoxyribonuclease TATDN3 (Fragment) OS=Homo sapiens GN=TATDN3 PE=4 SV=1 - (E9PRA1_HUMAN)
P02792	Ferritin light chain OS=Homo sapiens GN=FTL PE=1 SV=2 - (FRIL_HUMAN)
B8ZZL8	10 kDa heat shock protein, mitochondrial OS=Homo sapiens GN=HSPE1 PE=3 SV=1 - (B8ZZL8_HUMAN)
Q3HKR1	BTB/POZ domain-containing protein KCTD7 OS=Homo sapiens GN=KCTD7 PE=2 SV=1 - (Q3HKR1_HUMAN)
Q8TEU7	Rap guanine nucleotide exchange factor 6 OS=Homo sapiens GN=RAPGEF6 PE=1 SV=2 - (RPGF6_HUMAN)
P41252	Isoleucine--tRNA ligase, cytoplasmic OS=Homo sapiens GN=IARS PE=1 SV=2 - (SYIC_HUMAN)
Q8N8D1	Programmed cell death protein 7 OS=Homo sapiens GN=PDCD7 PE=1 SV=1 - (PDCD7_HUMAN)
B4DPT5	cDNA FLJ50023, highly similar to Alanine aminotransferase 1 (EC 2.6.1.2) OS=Homo sapiens PE=2 SV=1 - (B4DPT5_HUMAN)
B0YJ28	Interleukin-18 OS=Homo sapiens GN=IL18 PE=3 SV=1 - (B0YJ28_HUMAN)
B1AJY7	26S proteasome non-ATPase regulatory subunit 10 OS=Homo sapiens GN=PSMD10 PE=4 SV=1 - (B1AJY7_HUMAN)
Q14CB2	DOK1 protein OS=Homo sapiens GN=DOK1 PE=2 SV=1 - (Q14CB2_HUMAN)
Q86TR6	Full-length cDNA 5-PRIME end of clone CS0DF014YL24 of Fetal brain of Homo sapiens (human) (Fragment) OS=Homo sapiens PE=2 SV=1 - (Q86TR6_HUMAN)
Q96KP4-2	Isoform 2 of Cytosolic non-specific dipeptidase OS=Homo sapiens GN=CNDP2 - (CNDP2_HUMAN)
O96013	Serine/threonine-protein kinase PAK 4 OS=Homo sapiens GN=PAK4 PE=1 SV=1 - (PAK4_HUMAN)
E5RGX1	Transcription factor IIIB 50 kDa subunit OS=Homo sapiens GN=BRF2 PE=4 SV=1 - (E5RGX1_HUMAN)
F8W543	Nucleosome assembly protein 1-like 1 OS=Homo sapiens GN=NAP1L1 PE=3 SV=1 - (F8W543_HUMAN)
F8WF32	Dolichyl-diphosphooligosaccharide--protein glycosyltransferase subunit 1 OS=Homo sapiens GN=RPN1 PE=4 SV=1 - (F8WF32_HUMAN)
B2RDY0	cDNA, FLJ96821, highly similar to Homo sapiens solute carrier family 35, member F5 (SLC35F5), mRNA OS=Homo sapiens PE=2 SV=1 - (B2RDY0_HUMAN)
B4DVR5	cDNA FLJ56251, highly similar to Cyclin-L2 OS=Homo sapiens PE=2 SV=1 - (B4DVR5_HUMAN)
Q92841-1	Isoform 2 of Probable ATP-dependent RNA helicase DDX17 OS=Homo sapiens GN=DDX17 - (DDX17_HUMAN)
B3KX75	cDNA FLJ44930 fis, clone BRAMY3015549, highly similar to Neural cell adhesion molecule L1-like protein (Fragment) OS=Homo sapiens PE=2 SV=1 - (B3KX75_HUMAN)
E9PI41	Exosome complex component RRP41 (Fragment) OS=Homo sapiens GN=EXOSC4 PE=4 SV=1 - (E9PI41_HUMAN)
E9PJ67	Cathepsin B (Fragment) OS=Homo sapiens GN=CTSB PE=4 SV=1 - (E9PJ67_HUMAN)
B3KX08	cDNA FLJ44420 fis, clone UTERU2002198, highly similar to RNA polymerase II elongation factor ELL3 OS=Homo sapiens PE=2 SV=1 - (B3KX08_HUMAN)
B3KMV5	cDNA FLJ12728 fis, clone NT2RP2000040, highly similar to Protein FAM62A OS=Homo sapiens PE=2 SV=1 - (B3KMV5_HUMAN)
A8K670	cDNA FLJ75703, highly similar to Homo sapiens nitric oxide synthase interacting protein (NOSIP), mRNA OS=Homo sapiens PE=2 SV=1 - (A8K670_HUMAN)
B0I1R4	KIF26A variant protein (Fragment) OS=Homo sapiens PE=2 SV=1 - (B0I1R4_HUMAN)
E9PCA6	Protocadherin-10 OS=Homo sapiens GN=PCDH10 PE=3 SV=1 - (E9PCA6_HUMAN)
Q59G39	G protein-coupled receptor 22 variant (Fragment) OS=Homo sapiens PE=2 SV=1 - (Q59G39_HUMAN)
Q13098-5	Isoform 4 of COP9 signalosome complex subunit 1 OS=Homo sapiens GN=GPS1 - (CSN1_HUMAN)
P49721	Proteasome subunit beta type-2 OS=Homo sapiens GN=PSMB2 PE=1 SV=1 - (PSB2_HUMAN)
D3DNI3	Profilin (Fragment) OS=Homo sapiens GN=PFN2 PE=3 SV=1 - (D3DNI3_HUMAN)
Q3SYB5	SERPINB12 protein OS=Homo sapiens GN=SERPINB12 PE=2 SV=1 - (Q3SYB5_HUMAN)
O14957	Cytochrome b-c1 complex subunit 10 OS=Homo sapiens GN=UQCR11 PE=2 SV=1 - (QCR10_HUMAN)
Q59FJ2	Ubiquilin 1 isoform 1 variant (Fragment) OS=Homo sapiens PE=2 SV=1 - (Q59FJ2_HUMAN)
A8K7S0	cDNA FLJ75478, highly similar to Homo sapiens splicing factor 4 (SF4), transcript variant a, mRNA OS=Homo sapiens PE=2 SV=1 - (A8K7S0_HUMAN)
Q8NHH1	Tubulin polyglutamylase TTLL11 OS=Homo sapiens GN=TTLL11 PE=2 SV=1 - (TTL11_HUMAN)
Q9HB15	Potassium channel subfamily K member 12 OS=Homo sapiens GN=KCNK12 PE=1 SV=1 - (KCNKC_HUMAN)
C9JLK5	Eukaryotic translation elongation factor 1 epsilon-1 OS=Homo sapiens GN=EEF1E1 PE=4 SV=1 - (C9JLK5_HUMAN)
Q5JYB1	Zinc finger and BTB domain containing 17 (Fragment) OS=Homo sapiens GN=ZBTB17 PE=2 SV=1 - (Q5JYB1_HUMAN)
Q9H6F5	Coiled-coil domain-containing protein 86 OS=Homo sapiens GN=CCDC86 PE=1 SV=1 - (CCD86_HUMAN)
O00470-2	Isoform 2 of Homeobox protein Meis1 OS=Homo sapiens GN=MEIS1 - (MEIS1_HUMAN)
B1AK87	Capping protein (Actin filament) muscle Z-line, beta OS=Homo sapiens GN=CAPZB PE=4 SV=1 - (B1AK87_HUMAN)
P62699	Protein yippee-like 5 OS=Homo sapiens GN=YPEL5 PE=2 SV=1 - (YPEL5_HUMAN)
O75445	Usherin OS=Homo sapiens GN=USH2A PE=1 SV=3 - (USH2A_HUMAN)
P62633-2	Isoform 2 of Cellular nucleic acid-binding protein OS=Homo sapiens GN=CNBP - (CNBP_HUMAN)
F5H4D0	Protein CLEC16A OS=Homo sapiens GN=CLEC16A PE=4 SV=1 - (F5H4D0_HUMAN)
Q6NWR3	G protein-coupled receptor 83 OS=Homo sapiens GN=GPR83 PE=2 SV=1 - (Q6NWR3_HUMAN)
P00441	Superoxide dismutase (Cu-Zn) OS=Homo sapiens GN=SOD1 PE=1 SV=2 - (SODC_HUMAN)
O15399	Glutamate (NMDA) receptor subunit epsilon-4 OS=Homo sapiens GN=GRIN2D PE=2 SV=2 - (NMDE4_HUMAN)
F8WAS3	NADH dehydrogenase (ubiquinone) 1 alpha subcomplex subunit 5 OS=Homo sapiens GN=NDUFA5 PE=4 SV=1 - (F8WAS3_HUMAN)
H7C2V0	Fatty acid desaturase 1 (Fragment) OS=Homo sapiens GN=FADS1 PE=3 SV=1 - (H7C2V0_HUMAN)
B4DGV0	cDNA FLJ60251, highly similar to Contactin-6 OS=Homo sapiens PE=2 SV=1 - (B4DGV0_HUMAN)
B4DMT0	Transmembrane protein 143 OS=Homo sapiens GN=TMEM143 PE=2 SV=1 - (B4DMT0_HUMAN)
Q5TA44	Cleavage and polyadenylation specific factor 3-like (Fragment) OS=Homo sapiens GN=CPSF3L PE=2 SV=1 - (Q5TA44_HUMAN)
Q96HE7	ERO1-like protein alpha OS=Homo sapiens GN=ERO1L PE=1 SV=2 - (ERO1A_HUMAN)
A6NM62	Leucine-rich repeat-containing protein 53 OS=Homo sapiens GN=LRRC53 PE=4 SV=2 - (LRC53_HUMAN)
B7ZBU0	Novel protein (Fragment) OS=Homo sapiens GN=RP11-60H7.2-001 PE=4 SV=1 - (B7ZBU0_HUMAN)
H0YJG0	Creatine kinase B-type (Fragment) OS=Homo sapiens GN=CKB PE=3 SV=1 - (H0YJG0_HUMAN)
Q14697-2	Isoform 2 of Neutral alpha-glucosidase AB OS=Homo sapiens GN=GANAB - (GANAB_HUMAN)
Q5VXS2	Ubiquitin-fold modifier 1 (Fragment) OS=Homo sapiens GN=UFM1 PE=2 SV=1 - (Q5VXS2_HUMAN)
B2RD96	cDNA, FLJ96514 OS=Homo sapiens PE=2 SV=1 - (B2RD96_HUMAN)
Q9NP97	Dynein light chain roadblock-type 1 OS=Homo sapiens GN=DYNLRB1 PE=1 SV=3 - (DLRB1_HUMAN)
Q6IPX5	Ribosomal protein S2 OS=Homo sapiens GN=RPS2 PE=2 SV=1 - (Q6IPX5_HUMAN)
B4E3K9	Superoxide dismutase OS=Homo sapiens GN=SOD2 PE=2 SV=1 - (B4E3K9_HUMAN)
E5RJY1	Protein NDRG1 OS=Homo sapiens GN=NDRG1 PE=4 SV=1 - (E5RJY1_HUMAN)
E9PNW4	CD59 glycoprotein OS=Homo sapiens GN=CD59 PE=4 SV=1 - (E9PNW4_HUMAN)
P62888	60S ribosomal protein L30 OS=Homo sapiens GN=RPL30 PE=1 SV=2 - (RL30_HUMAN)
H0YLR1	Protein FAM63B (Fragment) OS=Homo sapiens GN=FAM63B PE=4 SV=1 - (H0YLR1_HUMAN)
Q6L8H4	Keratin-associated protein 5-1 OS=Homo sapiens GN=KRTAP5-1 PE=2 SV=1 - (KRA51_HUMAN)
B4DFV7	cDNA FLJ60481 OS=Homo sapiens PE=2 SV=1 - (B4DFV7_HUMAN)
B7Z4B2	cDNA FLJ56108, highly similar to Puromycin-sensitive aminopeptidase (EC 3.4.11.-) OS=Homo sapiens PE=2 SV=1 - (B7Z4B2_HUMAN)
P04626-4	Isoform 4 of Receptor tyrosine-protein kinase erbB-2 OS=Homo sapiens GN=ERBB2 - (ERBB2_HUMAN)
E9PNA4	Transcriptional activator Myb OS=Homo sapiens GN=MYB PE=4 SV=1 - (E9PNA4_HUMAN)
Q96AZ6	Interferon-stimulated gene 20 kDa protein OS=Homo sapiens GN=ISG20 PE=1 SV=2 - (ISG20_HUMAN)
Q00688	Peptidyl-prolyl cis-trans isomerase FKBP3 OS=Homo sapiens GN=FKBP3 PE=1 SV=1 - (FKBP3_HUMAN)
Q96BK5	PIN2/TERF1-interacting telomerase inhibitor 1 OS=Homo sapiens GN=PINX1 PE=1 SV=2 - (PINX1_HUMAN)
Q53ER2	Cyclin D2 variant (Fragment) OS=Homo sapiens PE=2 SV=1 - (Q53ER2_HUMAN)
B5MD17	Chromobox protein homolog 1 (Fragment) OS=Homo sapiens GN=CBX1 PE=4 SV=1 - (B5MD17_HUMAN)
A8K3Y8	cDNA FLJ76141 OS=Homo sapiens PE=2 SV=1 - (A8K3Y8_HUMAN)
Q14161-11	Isoform 11 of ARF GTPase-activating protein GIT2 OS=Homo sapiens GN=GIT2 - (GIT2_HUMAN)
Q9Y5P6-2	Isoform 2 of Mannose-1-phosphate guanyltransferase beta OS=Homo sapiens GN=GMPPB - (GMPPB_HUMAN)
Q5JQR1	Testis expressed 11 (Fragment) OS=Homo sapiens GN=TEX11 PE=2 SV=1 - (Q5JQR1_HUMAN)
Q9H3A6	PRO2179 OS=Homo sapiens PE=2 SV=1 - (Q9H3A6_HUMAN)
I3L1R8	Trafficking protein particle complex subunit 1 OS=Homo sapiens GN=TRAPPC1 PE=4 SV=1 - (I3L1R8_HUMAN)
A4VCH6	LONP1 protein (Fragment) OS=Homo sapiens GN=LONP1 PE=2 SV=1 - (A4VCH6_HUMAN)
H7BZJ8	Zinc-alpha-2-glycoprotein (Fragment) OS=Homo sapiens GN=AZGP1 PE=4 SV=1 - (H7BZJ8_HUMAN)
C6K4L3	MHC class I antigen (Fragment) OS=Homo sapiens GN=HLA-C PE=3 SV=1 - (C6K4L3_HUMAN)
H0YEM8	Uncharacterized protein KIAA0408 (Fragment) OS=Homo sapiens GN=KIAA0408 PE=4 SV=1 - (H0YEM8_HUMAN)
H0YDB2	Stromal interaction molecule 1 (Fragment) OS=Homo sapiens GN=STIM1 PE=4 SV=1 - (H0YDB2_HUMAN)
Q8TE77-3	Isoform 3 of Protein phosphatase Slingshot homolog 3 OS=Homo sapiens GN=SSH3 - (SSH3_HUMAN)
Q13018-2	Isoform 2 of Secretory phospholipase A2 receptor OS=Homo sapiens GN=PLA2R1 - (PLA2R_HUMAN)
A8K6K4	cDNA FLJ77565, highly similar to Homo sapiens interleukin 1 receptor accessory protein (IL1RAP), transcript variant 1, mRNA OS=Homo sapiens PE=2 SV=1 - (A8K6K4_HUMAN)
B7Z1K1	cDNA FLJ60431, highly similar to Regulator of G-protein signaling 11 OS=Homo sapiens PE=2 SV=1 - (B7Z1K1_HUMAN)
A8MUL3	Putative uncharacterized protein ADARB2-AS1 OS=Homo sapiens GN=ADARB2-AS1 PE=5 SV=2 - (ADAS1_HUMAN)
F2Z2D5	Protein POF1B OS=Homo sapiens GN=POF1B PE=4 SV=1 - (F2Z2D5_HUMAN)
H0YJM8	Proteasome subunit beta type-5 (Fragment) OS=Homo sapiens GN=PSMB5 PE=3 SV=1 - (H0YJM8_HUMAN)
Q9BY89	Uncharacterized protein KIAA1671 OS=Homo sapiens GN=KIAA1671 PE=1 SV=2 - (K1671_HUMAN)
Q9H0R4-2	Isoform 2 of Haloacid dehalogenase-like hydrolase domain-containing protein 2 OS=Homo sapiens GN=HDHD2 - (HDHD2_HUMAN)
Q59EJ5	Glutathione S-transferase M3 variant (Fragment) OS=Homo sapiens PE=2 SV=1 - (Q59EJ5_HUMAN)
E0YMJ4	HNF1 alpha A splice variant 8 OS=Homo sapiens GN=HNF1A PE=2 SV=1 - (E0YMJ4_HUMAN)
B7ZLL0	KIAA0528 protein OS=Homo sapiens GN=KIAA0528 PE=2 SV=1 - (B7ZLL0_HUMAN)
A6NMS2	Ribose-phosphate pyrophosphokinase 2 (Fragment) OS=Homo sapiens GN=PRPS2 PE=4 SV=2 - (A6NMS2_HUMAN)
O95803	Bifunctional heparan sulfate N-deacetylase/N-sulfotransferase 3 OS=Homo sapiens GN=NDST3 PE=2 SV=1 - (NDST3_HUMAN)
B2RAN0	cDNA, FLJ95010, highly similar to Homo sapiens Bloom syndrome (BLM), mRNA OS=Homo sapiens PE=2 SV=1 - (B2RAN0_HUMAN)
Q9NSK3	Putative uncharacterized protein DKFZp762I166 (Fragment) OS=Homo sapiens GN=DKFZp762I166 PE=2 SV=1 - (Q9NSK3_HUMAN)
A8K9U7	cDNA FLJ75829, highly similar to Homo sapiens potassium inwardly-rectifying channel, subfamily J, member 15 (KCNJ15), transcript variant 2, mRNA OS=Homo sapiens PE=2 SV=1 - (A8K9U7_HUMAN)
B2REA7	Ribosomal protein L36a OS=Homo sapiens GN=RPL36A PE=3 SV=1 - (B2REA7_HUMAN)
B7Z2T1	cDNA FLJ58061, highly similar to Eukaryotic translation initiation factor 4E OS=Homo sapiens PE=2 SV=1 - (B7Z2T1_HUMAN)
H3BLV8	Vacuolar protein sorting-associated protein 29 (Fragment) OS=Homo sapiens GN=VPS29 PE=4 SV=1 - (H3BLV8_HUMAN)
D3VVI3	Ataxin 3 variant ref (Fragment) OS=Homo sapiens GN=ATXN3 PE=2 SV=1 - (D3VVI3_HUMAN)
E3VWE2	Regulator of calcineurin 3 isoform 1,3,4,5 OS=Homo sapiens GN=RCAN3 PE=4 SV=1 - (E3VWE2_HUMAN)
F5GZ04	Zinc finger and SCAN domain-containing protein 2 OS=Homo sapiens GN=ZSCAN2 PE=4 SV=1 - (F5GZ04_HUMAN)
Q56VW4	Transformation-related gene 6 OS=Homo sapiens PE=4 SV=1 - (Q56VW4_HUMAN)
E9PN70	Trafficking protein particle complex subunit 4 OS=Homo sapiens GN=TRAPPC4 PE=4 SV=1 - (E9PN70_HUMAN)
B4DNT4	Cardiotrophin-like cytokine factor 1 OS=Homo sapiens GN=CLCF1 PE=2 SV=1 - (B4DNT4_HUMAN)
B4DWH7	V-type proton ATPase subunit B, kidney isoform OS=Homo sapiens GN=ATP6V1B1 PE=2 SV=1 - (B4DWH7_HUMAN)
E9PJ29	Out at first protein homolog OS=Homo sapiens GN=OAF PE=4 SV=1 - (E9PJ29_HUMAN)
P25786-2	Isoform Long of Proteasome subunit alpha type-1 OS=Homo sapiens GN=PSMA1 - (PSA1_HUMAN)
A8MWY0-2	Isoform 2 of UPF0577 protein KIAA1324-like OS=Homo sapiens GN=KIAA1324L - (K132L_HUMAN)
P05089-3	Isoform 3 of Arginase-1 OS=Homo sapiens GN=ARG1 - (ARGI1_HUMAN)
O95835	Serine/threonine-protein kinase LATS1 OS=Homo sapiens GN=LATS1 PE=1 SV=1 - (LATS1_HUMAN)
D6RB53	Ufm1-specific protease 2 OS=Homo sapiens GN=UFSP2 PE=4 SV=1 - (D6RB53_HUMAN)
H3BQF3	S phase cyclin A-associated protein in the endoplasmic reticulum (Fragment) OS=Homo sapiens GN=SCAPER PE=4 SV=1 - (H3BQF3_HUMAN)
Q9H246	Uncharacterized protein C1orf21 OS=Homo sapiens GN=C1orf21 PE=1 SV=1 - (CA021_HUMAN)
B4E0H6	cDNA FLJ52584, highly similar to Heat shock 70 kDa protein 4 OS=Homo sapiens PE=2 SV=1 - (B4E0H6_HUMAN)
O75844	CAAX prenyl protease 1 homolog OS=Homo sapiens GN=ZMPSTE24 PE=1 SV=2 - (FACE1_HUMAN)
Q3SWV3	LOC389833 protein (Fragment) OS=Homo sapiens GN=LOC389833 PE=2 SV=1 - (Q3SWV3_HUMAN)
A8K4K5	cDNA FLJ77843, highly similar to Homo sapiens TATA box binding protein (TBP)-associated factor, RNA polymerase I, A, 48kDa (TAF1A), transcript variant 1, mRNA OS=Homo sapiens PE=2 SV=1 - (A8K4K5_HUMAN)
I3NI19	Neuropeptide B-29 OS=Homo sapiens GN=NPB PE=4 SV=1 - (I3NI19_HUMAN)
Q96QS1-3	Isoform 3 of Tetraspanin-32 OS=Homo sapiens GN=TSPAN32 - (TSN32_HUMAN)
E9PNP2	Translocon-associated protein subunit beta OS=Homo sapiens GN=SSR2 PE=4 SV=1 - (E9PNP2_HUMAN)
O14523	C2 domain-containing protein 2-like OS=Homo sapiens GN=C2CD2L PE=1 SV=3 - (C2C2L_HUMAN)
Q03692	Collagen alpha-1(X) chain OS=Homo sapiens GN=COL10A1 PE=1 SV=2 - (COAA1_HUMAN)
Q8IUH2	Protein CREG2 OS=Homo sapiens GN=CREG2 PE=1 SV=1 - (CREG2_HUMAN)
P21802-11	Isoform 11 of Fibroblast growth factor receptor 2 OS=Homo sapiens GN=FGFR2 - (FGFR2_HUMAN)
Q12967-2	Isoform 2 of Ral guanine nucleotide dissociation stimulator OS=Homo sapiens GN=RALGDS - (GNDS_HUMAN)
O75525	KH domain-containing, RNA-binding, signal transduction-associated protein 3 OS=Homo sapiens GN=KHDRBS3 PE=1 SV=1 - (KHDR3_HUMAN)
P01704	Ig lambda chain V-II region TOG OS=Homo sapiens PE=1 SV=1 - (LV201_HUMAN)
P29966	Myristoylated alanine-rich C-kinase substrate OS=Homo sapiens GN=MARCKS PE=1 SV=4 - (MARCS_HUMAN)
O75448-2	Isoform 2 of Mediator of RNA polymerase II transcription subunit 24 OS=Homo sapiens GN=MED24 - (MED24_HUMAN)
Q6W4X9	Mucin-6 OS=Homo sapiens GN=MUC6 PE=1 SV=3 - (MUC6_HUMAN)
Q6ZVX7	Non-specific cytotoxic cell receptor protein 1 homolog OS=Homo sapiens GN=NCCRP1 PE=1 SV=1 - (NCRP1_HUMAN)
O14788	Tumor necrosis factor ligand superfamily member 11 OS=Homo sapiens GN=TNFSF11 PE=1 SV=1 - (TNF11_HUMAN)
Q9UGI0	Ubiquitin thioesterase ZRANB1 OS=Homo sapiens GN=ZRANB1 PE=1 SV=2 - (ZRAN1_HUMAN)
B4DQB5	Leukocyte surface antigen CD53 OS=Homo sapiens GN=CD53 PE=2 SV=1 - (B4DQB5_HUMAN)
B7Z4K4	Putative ribosomal RNA methyltransferase 1 OS=Homo sapiens GN=FTSJ1 PE=2 SV=1 - (B7Z4K4_HUMAN)
Q5T446	Uroporphyrinogen decarboxylase (Fragment) OS=Homo sapiens GN=UROD PE=2 SV=1 - (Q5T446_HUMAN)
I3NI52	Dehydrogenase/reductase SDR family member 7C (Fragment) OS=Homo sapiens GN=DHRS7C PE=4 SV=1 - (I3NI52_HUMAN)
Q9BS84	STK4 protein OS=Homo sapiens PE=2 SV=1 - (Q9BS84_HUMAN)
B4DEE1	cDNA FLJ56055, highly similar to ER degradation-enhancingalpha-mannosidase-like 2 OS=Homo sapiens PE=2 SV=1 - (B4DEE1_HUMAN)
B3KWY2	cDNA FLJ44205 fis, clone THYMU3001379, highly similar to 116 kDa U5 small nuclear ribonucleoprotein component OS=Homo sapiens PE=2 SV=1 - (B3KWY2_HUMAN)
Q8NF00	FLJ00404 protein (Fragment) OS=Homo sapiens GN=FLJ00404 PE=2 SV=1 - (Q8NF00_HUMAN)
B7ZLZ5	TMEM44 protein OS=Homo sapiens GN=TMEM44 PE=2 SV=1 - (B7ZLZ5_HUMAN)
Q5HYI2	Putative uncharacterized protein DKFZp313N0733 OS=Homo sapiens GN=DKFZp313N0733 PE=2 SV=1 - (Q5HYI2_HUMAN)
Q3KNR5	PAX4 protein OS=Homo sapiens GN=PAX4 PE=2 SV=1 - (Q3KNR5_HUMAN)
Q6UWG2	ALLW1950 OS=Homo sapiens GN=UNQ1950 PE=4 SV=1 - (Q6UWG2_HUMAN)
B2RB24	cDNA, FLJ95266 OS=Homo sapiens PE=2 SV=1 - (B2RB24_HUMAN)
Q65ZQ3	FBRNP OS=Homo sapiens GN=D10S102 PE=2 SV=1 - (Q65ZQ3_HUMAN)
B7Z4R9	cDNA FLJ59037 OS=Homo sapiens PE=2 SV=1 - (B7Z4R9_HUMAN)
B4DZ94	cDNA FLJ57095 OS=Homo sapiens PE=2 SV=1 - (B4DZ94_HUMAN)
F5GWE1	Glycine receptor subunit beta OS=Homo sapiens GN=GLRB PE=3 SV=1 - (F5GWE1_HUMAN)
B3Y1W9	Wings apart-like homolog variant 8 (Fragment) OS=Homo sapiens GN=WAPL PE=2 SV=1 - (B3Y1W9_HUMAN)
E9PP00	Probable ribonuclease ZC3H12C OS=Homo sapiens GN=ZC3H12C PE=4 SV=1 - (E9PP00_HUMAN)
B1AHM3	Transmembrane protein 8 (Five membrane-spanning domains) (Fragment) OS=Homo sapiens GN=TMEM8 PE=4 SV=1 - (B1AHM3_HUMAN)
Q701L7	Type II hair keratin 2 OS=Homo sapiens GN=KRTHB2 PE=2 SV=1 - (Q701L7_HUMAN)
B3KY79	cDNA FLJ46620 fis, clone TLUNG2000654, highly similar to Keratin, type II cytoskeletal 7 OS=Homo sapiens PE=2 SV=1 - (B3KY79_HUMAN)
Q53GA7	Tubulin alpha 6 variant (Fragment) OS=Homo sapiens PE=2 SV=1 - (Q53GA7_HUMAN)
B7TY16	Actinin alpha 1 isoform 3 OS=Homo sapiens GN=ACTN1 PE=2 SV=1 - (B7TY16_HUMAN)
O15446-2	Isoform 2 of DNA-directed RNA polymerase I subunit RPA34 OS=Homo sapiens GN=CD3EAP - (RPA34_HUMAN)
Q8WZ42-2	Isoform 2 of Titin OS=Homo sapiens GN=TTN - (TITIN_HUMAN)
H0YA55	Serum albumin (Fragment) OS=Homo sapiens GN=ALB PE=4 SV=1 - (H0YA55_HUMAN)
O94868-3	Isoform 3 of FCH and double SH3 domains protein 2 OS=Homo sapiens GN=FCHSD2 - (FCSD2_HUMAN)
Q13748	Tubulin alpha-3C/D chain OS=Homo sapiens GN=TUBA3C PE=1 SV=3 - (TBA3C_HUMAN)
O75369-6	Isoform 6 of Filamin-B OS=Homo sapiens GN=FLNB - (FLNB_HUMAN)
A2A3F7	Transient receptor potential cation channel, subfamily M, member 3 OS=Homo sapiens GN=TRPM3 PE=4 SV=1 - (A2A3F7_HUMAN)
Q14980-2	Isoform 2 of Nuclear mitotic apparatus protein 1 OS=Homo sapiens GN=NUMA1 - (NUMA1_HUMAN)
Q13136-2	Isoform 2 of Liprin-alpha-1 OS=Homo sapiens GN=PPFIA1 - (LIPA1_HUMAN)
P46939	Utrophin OS=Homo sapiens GN=UTRN PE=1 SV=2 - (UTRO_HUMAN)
F8VZ45	60S ribosomal protein L6 (Fragment) OS=Homo sapiens GN=RPL6 PE=4 SV=1 - (F8VZ45_HUMAN)
B3KXE7	cDNA FLJ45304 fis, clone BRHIP3003984, highly similar to IkappaB kinase complex-associated protein OS=Homo sapiens PE=2 SV=1 - (B3KXE7_HUMAN)
Q15906	Vacuolar protein sorting-associated protein 72 homolog OS=Homo sapiens GN=VPS72 PE=1 SV=1 - (VPS72_HUMAN)
B3KTV0	cDNA FLJ38781 fis, clone LIVER2000216, highly similar to HEAT SHOCK COGNATE 71 kDa PROTEIN OS=Homo sapiens PE=2 SV=1 - (B3KTV0_HUMAN)
Q6AWC2-4	Isoform 4 of Protein WWC2 OS=Homo sapiens GN=WWC2 - (WWC2_HUMAN)
O95613-2	Isoform 2 of Pericentrin OS=Homo sapiens GN=PCNT - (PCNT_HUMAN)
Q6P1J9	Parafibromin OS=Homo sapiens GN=CDC73 PE=1 SV=1 - (CDC73_HUMAN)
Q5UGI3	Ubiquitin C splice variant OS=Homo sapiens GN=UBC PE=2 SV=1 - (Q5UGI3_HUMAN)
H0YM23	Ankyrin repeat domain-containing protein 17 (Fragment) OS=Homo sapiens GN=ANKRD17 PE=4 SV=1 - (H0YM23_HUMAN)
Q8NEY8-5	Isoform 5 of Periphilin-1 OS=Homo sapiens GN=PPHLN1 - (PPHLN_HUMAN)
B4E3S1	cDNA FLJ57040, highly similar to Myosin-9 OS=Homo sapiens PE=2 SV=1 - (B4E3S1_HUMAN)
Q3V6T2-4	Isoform 4 of Girdin OS=Homo sapiens GN=CCDC88A - (GRDN_HUMAN)
B3KTL8	cDNA FLJ38476 fis, clone FEBRA2022504, highly similar to YTH domain protein 1 OS=Homo sapiens PE=2 SV=1 - (B3KTL8_HUMAN)
B4DJI1	L-lactate dehydrogenase OS=Homo sapiens PE=2 SV=1 - (B4DJI1_HUMAN)
H0Y4W4	NF-kappa-B inhibitor epsilon (Fragment) OS=Homo sapiens GN=NFKBIE PE=4 SV=1 - (H0Y4W4_HUMAN)
Q8IUE6	Histone H2A type 2-B OS=Homo sapiens GN=HIST2H2AB PE=1 SV=3 - (H2A2B_HUMAN)
Q5T655	Coiled-coil domain-containing protein 147 OS=Homo sapiens GN=CCDC147 PE=2 SV=1 - (CC147_HUMAN)
Q9NS87	Kinesin-like protein KIF15 OS=Homo sapiens GN=KIF15 PE=1 SV=1 - (KIF15_HUMAN)
Q96LA8-2	Isoform 2 of Protein arginine N-methyltransferase 6 OS=Homo sapiens GN=PRMT6 - (ANM6_HUMAN)
Q7RTZ2	Putative ubiquitin carboxyl-terminal hydrolase 17-like protein 1 OS=Homo sapiens GN=USP17L1P PE=5 SV=1 - (U17L1_HUMAN)
Q86Y33-5	Isoform 5 of Cell division cycle protein 20 homolog B OS=Homo sapiens GN=CDC20B - (CD20B_HUMAN)
Q15853-2	Isoform USF2A-delta-H of Upstream stimulatory factor 2 OS=Homo sapiens GN=USF2 - (USF2_HUMAN)
Q53G25	Ribosomal protein S5 variant (Fragment) OS=Homo sapiens PE=2 SV=1 - (Q53G25_HUMAN)
B4E3Q9	cDNA FLJ59659, highly similar to Vinculin OS=Homo sapiens PE=2 SV=1 - (B4E3Q9_HUMAN)
Q32MZ4-2	Isoform 2 of Leucine-rich repeat flightless-interacting protein 1 OS=Homo sapiens GN=LRRFIP1 - (LRRF1_HUMAN)
Q7L7X3	Serine/threonine-protein kinase TAO1 OS=Homo sapiens GN=TAOK1 PE=1 SV=1 - (TAOK1_HUMAN)
G3V1A4	Cofilin 1 (Non-muscle), isoform CRA_a OS=Homo sapiens GN=CFL1 PE=4 SV=1 - (G3V1A4_HUMAN)
Q99996-6	Isoform 6 of A-kinase anchor protein 9 OS=Homo sapiens GN=AKAP9 - (AKAP9_HUMAN)
Q5T011-5	Isoform 3 of Protein SZT2 OS=Homo sapiens GN=SZT2 - (SZT2_HUMAN)
Q96CS3	FAS-associated factor 2 OS=Homo sapiens GN=FAF2 PE=1 SV=2 - (FAF2_HUMAN)
Q02388	Collagen alpha-1(VII) chain OS=Homo sapiens GN=COL7A1 PE=1 SV=2 - (CO7A1_HUMAN)
D3YTG3	Target of Nesh-SH3 OS=Homo sapiens GN=ABI3BP PE=4 SV=1 - (D3YTG3_HUMAN)
H0YJZ4	Striatin-3 (Fragment) OS=Homo sapiens GN=STRN3 PE=4 SV=1 - (H0YJZ4_HUMAN)
B4DE44	cDNA FLJ52880, highly similar to Malate dehydrogenase, mitochondrial (EC 1.1.1.37) OS=Homo sapiens PE=2 SV=1 - (B4DE44_HUMAN)
H0YIV1	Nuclear receptor subfamily 2 group C member 1 (Fragment) OS=Homo sapiens GN=NR2C1 PE=4 SV=1 - (H0YIV1_HUMAN)
Q5SQQ9-2	Isoform 2 of Ventral anterior homeobox 1 OS=Homo sapiens GN=VAX1 - (VAX1_HUMAN)
Q86Y02	FAM167A protein OS=Homo sapiens GN=FAM167A PE=2 SV=1 - (Q86Y02_HUMAN)
I3L2X7	5-azacytidine-induced protein 1 (Fragment) OS=Homo sapiens GN=AZI1 PE=4 SV=1 - (I3L2X7_HUMAN)
Q96HA7	Tonsoku-like protein OS=Homo sapiens GN=TONSL PE=1 SV=2 - (TONSL_HUMAN)
F8VVB8	Meiosis arrest female protein 1 OS=Homo sapiens GN=KIAA0430 PE=4 SV=1 - (F8VVB8_HUMAN)
A8TX70-2	Isoform 2 of Collagen alpha-5(VI) chain OS=Homo sapiens GN=COL6A5 - (CO6A5_HUMAN)
H0Y5Q9	Phosphatidylinositol 3,4,5-trisphosphate 5-phosphatase 1 (Fragment) OS=Homo sapiens GN=INPP5D PE=4 SV=1 - (H0Y5Q9_HUMAN)
Q9Y6V0	Protein piccolo OS=Homo sapiens GN=PCLO PE=1 SV=4 - (PCLO_HUMAN)
O75185	Calcium-transporting ATPase type 2C member 2 OS=Homo sapiens GN=ATP2C2 PE=1 SV=2 - (AT2C2_HUMAN)
E5RGT7	Uncharacterized protein OS=Homo sapiens PE=4 SV=1 - (E5RGT7_HUMAN)
A4UGR9-2	Isoform 2 of Xin actin-binding repeat-containing protein 2 OS=Homo sapiens GN=XIRP2 - (XIRP2_HUMAN)
P25705	ATP synthase subunit alpha, mitochondrial OS=Homo sapiens GN=ATP5A1 PE=1 SV=1 - (ATPA_HUMAN)
A8K2G5	Rap guanine nucleotide exchange factor (GEF) 3, isoform CRA_a OS=Homo sapiens GN=RAPGEF3 PE=2 SV=1 - (A8K2G5_HUMAN)
B4DK00	cDNA FLJ58506 OS=Homo sapiens PE=2 SV=1 - (B4DK00_HUMAN)
Q1MSJ5-1	Isoform 1 of Centrosome and spindle pole-associated protein 1 OS=Homo sapiens GN=CSPP1 - (CSPP1_HUMAN)
Q6UXS9-3	Isoform 3 of Inactive caspase-12 OS=Homo sapiens GN=CASP12 - (CASPC_HUMAN)
Q8NF50-2	Isoform 2 of Dedicator of cytokinesis protein 8 OS=Homo sapiens GN=DOCK8 - (DOCK8_HUMAN)
Q9Y4C2-2	Isoform 2 of Protein FAM115A OS=Homo sapiens GN=FAM115A - (F115A_HUMAN)
P06734-2	Isoform B of Low affinity immunoglobulin epsilon Fc receptor OS=Homo sapiens GN=FCER2 - (FCER2_HUMAN)
Q9H8X9	Probable palmitoyltransferase ZDHHC11 OS=Homo sapiens GN=ZDHHC11 PE=2 SV=1 - (ZDH11_HUMAN)
C9JQD1	Glucokinase OS=Homo sapiens GN=GCK PE=3 SV=1 - (C9JQD1_HUMAN)
Q9H2L5	Ras association domain-containing protein 4 OS=Homo sapiens GN=RASSF4 PE=1 SV=2 - (RASF4_HUMAN)
B2RAP5	cDNA, FLJ95039, highly similar to Homo sapiens peroxisomal biogenesis factor 5-like (PEX5L), mRNA OS=Homo sapiens PE=2 SV=1 - (B2RAP5_HUMAN)
Q9UGL1-2	Isoform 2 of Lysine-specific demethylase 5B OS=Homo sapiens GN=KDM5B - (KDM5B_HUMAN)
F8WD27	Ryanodine receptor 3 OS=Homo sapiens GN=RYR3 PE=4 SV=1 - (F8WD27_HUMAN)
B4DWN9	cDNA FLJ55258, highly similar to Homo sapiens pleckstrin homology domain containing, family F member 1, mRNA OS=Homo sapiens PE=2 SV=1 - (B4DWN9_HUMAN)
H0YGD3	Nesprin-1 (Fragment) OS=Homo sapiens GN=SYNE1 PE=4 SV=1 - (H0YGD3_HUMAN)
P18124	60S ribosomal protein L7 OS=Homo sapiens GN=RPL7 PE=1 SV=1 - (RL7_HUMAN)
E5RFJ1	E3 SUMO-protein ligase NSE2 OS=Homo sapiens GN=NSMCE2 PE=4 SV=1 - (E5RFJ1_HUMAN)
G8JLF2	Coiled-coil and C2 domain-containing protein 1B OS=Homo sapiens GN=CC2D1B PE=4 SV=1 - (G8JLF2_HUMAN)
Q6YHK3-4	Isoform 4 of CD109 antigen OS=Homo sapiens GN=CD109 - (CD109_HUMAN)
B4DEB9	cDNA FLJ61099, highly similar to ADP-ribosylation factor 1 OS=Homo sapiens PE=2 SV=1 - (B4DEB9_HUMAN)
H9C5F6	MHC class I antigen (Fragment) OS=Homo sapiens GN=HLA-A PE=3 SV=1 - (H9C5F6_HUMAN)
F5GZX2	Tektin-1 OS=Homo sapiens GN=TEKT1 PE=4 SV=1 - (F5GZX2_HUMAN)
A8K1U9	cDNA FLJ76762, highly similar to Homo sapiens suppressor of zeste 12 homolog (Drosophila) (SUZ12), mRNA OS=Homo sapiens PE=2 SV=1 - (A8K1U9_HUMAN)
P30613-2	Isoform L-type of Pyruvate kinase isozymes R/L OS=Homo sapiens GN=PKLR - (KPYR_HUMAN)
B4DXF3	cDNA FLJ57650, highly similar to Bleomycin hydrolase (EC 3.4.22.40) OS=Homo sapiens PE=2 SV=1 - (B4DXF3_HUMAN)
E7EVW9	Oxygen-regulated protein 1 OS=Homo sapiens GN=RP1 PE=4 SV=1 - (E7EVW9_HUMAN)
Q8N6N2	Tetratricopeptide repeat protein 9B OS=Homo sapiens GN=TTC9B PE=2 SV=1 - (TTC9B_HUMAN)
I3L3E2	Multidrug and toxin extrusion protein 1 (Fragment) OS=Homo sapiens GN=SLC47A1 PE=4 SV=1 - (I3L3E2_HUMAN)
Q14966-3	Isoform 3 of Zinc finger protein 638 OS=Homo sapiens GN=ZNF638 - (ZN638_HUMAN)
H0YFW4	Low-density lipoprotein receptor-related protein 1B OS=Homo sapiens GN=LRP1B PE=4 SV=1 - (H0YFW4_HUMAN)
D3DPI2	HCG1641229, isoform CRA_a OS=Homo sapiens GN=hCG_1641229 PE=4 SV=1 - (D3DPI2_HUMAN)
B4E2M0	cDNA FLJ55443, highly similar to Ras GTPase-activating-like protein IQGAP1 OS=Homo sapiens PE=2 SV=1 - (B4E2M0_HUMAN)
Q9BYX2-4	Isoform 4 of TBC1 domain family member 2A OS=Homo sapiens GN=TBC1D2 - (TBD2A_HUMAN)
B4DNL5	cDNA FLJ59361, highly similar to Protein disulfide-isomerase (EC 5.3.4.1) OS=Homo sapiens PE=2 SV=1 - (B4DNL5_HUMAN)
A4D1E1	Zinc finger protein 804B OS=Homo sapiens GN=ZNF804B PE=1 SV=2 - (Z804B_HUMAN)
B4DDM6	Mitotic checkpoint protein BUB3 OS=Homo sapiens GN=BUB3 PE=2 SV=1 - (B4DDM6_HUMAN)
G3V492	Uncharacterized protein OS=Homo sapiens PE=4 SV=1 - (G3V492_HUMAN)
B4DYN3	cDNA FLJ55634, highly similar to Autophagy-related protein 9A OS=Homo sapiens PE=2 SV=1 - (B4DYN3_HUMAN)
F8WCJ1	Eukaryotic translation initiation factor 5A-2 OS=Homo sapiens GN=EIF5A2 PE=4 SV=1 - (F8WCJ1_HUMAN)
Q86VG7	Uncharacterized protein OS=Homo sapiens PE=2 SV=1 - (Q86VG7_HUMAN)
Q9NY97-2	Isoform 2 of UDP-GlcNAc:betaGal beta-1,3-N-acetylglucosaminyltransferase 2 OS=Homo sapiens GN=B3GNT2 - (B3GN2_HUMAN)
Q9UG77	Putative uncharacterized protein DKFZp586K091 (Fragment) OS=Homo sapiens GN=DKFZp586K091 PE=2 SV=1 - (Q9UG77_HUMAN)
P18084	Integrin beta-5 OS=Homo sapiens GN=ITGB5 PE=1 SV=1 - (ITB5_HUMAN)
Q7L5Y1-2	Isoform 2 of Mitochondrial enolase superfamily member 1 OS=Homo sapiens GN=ENOSF1 - (ENOF1_HUMAN)
E9PGT1	Translin OS=Homo sapiens GN=TSN PE=4 SV=1 - (E9PGT1_HUMAN)
Q8NFC6	Biorientation of chromosomes in cell division protein 1-like 1 OS=Homo sapiens GN=BOD1L1 PE=1 SV=2 - (BD1L1_HUMAN)
B3KWQ6	cDNA FLJ43599 fis, clone SMINT2017781, highly similar to PERIPHERIN OS=Homo sapiens PE=2 SV=1 - (B3KWQ6_HUMAN)
B4DRA1	cDNA FLJ58609, highly similar to SH3-containing GRB2-like protein 1 OS=Homo sapiens PE=2 SV=1 - (B4DRA1_HUMAN)
B3KXG3	cDNA FLJ45362 fis, clone BRHIP3014675, highly similar to Homo sapiens BTB (POZ) domain containing 11 (BTBD11), transcript variant 2, mRNA OS=Homo sapiens PE=2 SV=1 - (B3KXG3_HUMAN)
Q5BJF6-2	Isoform 2 of Outer dense fiber protein 2 OS=Homo sapiens GN=ODF2 - (ODFP2_HUMAN)
Q96PN6	Adenylate cyclase type 10 OS=Homo sapiens GN=ADCY10 PE=1 SV=3 - (ADCYA_HUMAN)
B4DE36	Glucose-6-phosphate isomerase OS=Homo sapiens PE=2 SV=1 - (B4DE36_HUMAN)
P16157-7	Isoform Er6 of Ankyrin-1 OS=Homo sapiens GN=ANK1 - (ANK1_HUMAN)
B4DLQ3	cDNA FLJ52326, highly similar to Oral-facial-digital syndrome 1 protein OS=Homo sapiens PE=2 SV=1 - (B4DLQ3_HUMAN)
Q5M9N0	Coiled-coil domain-containing protein 158 OS=Homo sapiens GN=CCDC158 PE=2 SV=2 - (CD158_HUMAN)
Q9UK61	Protein FAM208A OS=Homo sapiens GN=FAM208A PE=1 SV=3 - (F208A_HUMAN)
E5RGC2	Zinc finger protein 354A OS=Homo sapiens GN=ZNF354A PE=4 SV=1 - (E5RGC2_HUMAN)
Q6ZUS5	Coiled-coil domain-containing protein 121 OS=Homo sapiens GN=CCDC121 PE=2 SV=1 - (CC121_HUMAN)
Q5VVM6	Coiled-coil domain-containing protein 30 OS=Homo sapiens GN=CCDC30 PE=2 SV=1 - (CCD30_HUMAN)
Q53SV2	Putative uncharacterized protein FLJ10035 OS=Homo sapiens GN=FLJ10035 PE=2 SV=1 - (Q53SV2_HUMAN)
A7KAX9-2	Isoform 2 of Rho GTPase-activating protein 32 OS=Homo sapiens GN=ARHGAP32 - (RHG32_HUMAN)
H0YA96	Heterogeneous nuclear ribonucleoprotein D0 (Fragment) OS=Homo sapiens GN=HNRNPD PE=4 SV=1 - (H0YA96_HUMAN)
Q6AWC6	Putative uncharacterized protein DKFZp686N14223 OS=Homo sapiens GN=DKFZp686N14223 PE=2 SV=1 - (Q6AWC6_HUMAN)
P35442	Thrombospondin-2 OS=Homo sapiens GN=THBS2 PE=1 SV=2 - (TSP2_HUMAN)
E9PP36	60S ribosomal protein L8 OS=Homo sapiens GN=RPL8 PE=4 SV=1 - (E9PP36_HUMAN)
B9EK47	HEAT repeat containing 5B OS=Homo sapiens GN=HEATR5B PE=2 SV=1 - (B9EK47_HUMAN)
Q9H0C2	ADP/ATP translocase 4 OS=Homo sapiens GN=SLC25A31 PE=1 SV=1 - (ADT4_HUMAN)
B7Z688	cDNA FLJ57192, highly similar to Tuftelin OS=Homo sapiens PE=2 SV=1 - (B7Z688_HUMAN)
Q9Y4D8-4	Isoform 4 of Probable E3 ubiquitin-protein ligase C12orf51 OS=Homo sapiens GN=C12orf51 - (K0614_HUMAN)
B9EGR0	C4orf8 protein OS=Homo sapiens GN=C4orf8 PE=2 SV=1 - (B9EGR0_HUMAN)
B4E3K4	cDNA FLJ57020, highly similar to Rho-guanine nucleotide exchange factor OS=Homo sapiens PE=2 SV=1 - (B4E3K4_HUMAN)
Q13451	Peptidyl-prolyl cis-trans isomerase FKBP5 OS=Homo sapiens GN=FKBP5 PE=1 SV=2 - (FKBP5_HUMAN)
B7Z5R8	cDNA FLJ61653, highly similar to Potassium/sodium hyperpolarization-activated cyclic nucleotide-gated channel 3 OS=Homo sapiens PE=2 SV=1 - (B7Z5R8_HUMAN)
Q9UK96	F-box only protein 10 OS=Homo sapiens GN=FBXO10 PE=2 SV=3 - (FBX10_HUMAN)
A7E2C5	BZRAP1 protein OS=Homo sapiens GN=BZRAP1 PE=2 SV=1 - (A7E2C5_HUMAN)
Q93084-7	Isoform SERCA3F of Sarcoplasmic/endoplasmic reticulum calcium ATPase 3 OS=Homo sapiens GN=ATP2A3 - (AT2A3_HUMAN)
Q8N4Y2	EF-hand calcium-binding domain-containing protein 4A OS=Homo sapiens GN=EFCAB4A PE=2 SV=3 - (EFC4A_HUMAN)
B7ZLJ4	Peroxiredoxin 5 OS=Homo sapiens GN=PRDX5 PE=2 SV=1 - (B7ZLJ4_HUMAN)
F8W6N3	Ubiquitin carboxyl-terminal hydrolase BAP1 OS=Homo sapiens GN=BAP1 PE=4 SV=1 - (F8W6N3_HUMAN)
Q69YN2	CWF19-like protein 1 OS=Homo sapiens GN=CWF19L1 PE=1 SV=2 - (C19L1_HUMAN)
A6NJ78	Probable methyltransferase-like protein 15 OS=Homo sapiens GN=METTL15 PE=2 SV=1 - (MET15_HUMAN)
Q8IY92	Structure-specific endonuclease subunit SLX4 OS=Homo sapiens GN=SLX4 PE=1 SV=3 - (SLX4_HUMAN)
B4DHC1	cDNA FLJ53868 OS=Homo sapiens PE=2 SV=1 - (B4DHC1_HUMAN)
Q9NQ90-3	Isoform 3 of Anoctamin-2 OS=Homo sapiens GN=ANO2 - (ANO2_HUMAN)
B9EJE3	Putative uncharacterized protein OS=Homo sapiens PE=2 SV=1 - (B9EJE3_HUMAN)
B2R646	cDNA, FLJ92780, highly similar to Homo sapiens phosphodiesterase 2A, cGMP-stimulated (PDE2A), mRNA OS=Homo sapiens PE=2 SV=1 - (B2R646_HUMAN)
Q96JP0	Protein fem-1 homolog C OS=Homo sapiens GN=FEM1C PE=2 SV=1 - (FEM1C_HUMAN)
Q9NYQ6	Cadherin EGF LAG seven-pass G-type receptor 1 OS=Homo sapiens GN=CELSR1 PE=1 SV=1 - (CELR1_HUMAN)
B4DMB1	cDNA FLJ53358, highly similar to Heterogeneous nuclear ribonucleoprotein R OS=Homo sapiens PE=2 SV=1 - (B4DMB1_HUMAN)
B4DPU9	cDNA FLJ55129, highly similar to HMG box-containing protein 1 OS=Homo sapiens PE=2 SV=1 - (B4DPU9_HUMAN)
E7EWK6	Uncharacterized protein OS=Homo sapiens PE=4 SV=1 - (E7EWK6_HUMAN)
B4DS76	Mitogen-activated protein kinase kinase kinase 11 OS=Homo sapiens GN=MAP3K11 PE=2 SV=1 - (B4DS76_HUMAN)
B2RDM2	HCG1811539, isoform CRA_b OS=Homo sapiens GN=TXNDC5 PE=2 SV=1 - (B2RDM2_HUMAN)
P35712-4	Isoform 4 of Transcription factor SOX-6 OS=Homo sapiens GN=SOX6 - (SOX6_HUMAN)
Q59FN4	LATS homolog 1 variant (Fragment) OS=Homo sapiens PE=2 SV=1 - (Q59FN4_HUMAN)
O94804	Serine/threonine-protein kinase 10 OS=Homo sapiens GN=STK10 PE=1 SV=1 - (STK10_HUMAN)
Q8NGI6	Olfactory receptor 4D10 OS=Homo sapiens GN=OR4D10 PE=2 SV=1 - (OR4DA_HUMAN)
Q02040	A-kinase anchor protein 17A OS=Homo sapiens GN=AKAP17A PE=1 SV=2 - (AK17A_HUMAN)
B4DLS2	cDNA FLJ50803, highly similar to Zinc finger protein 23 OS=Homo sapiens PE=2 SV=1 - (B4DLS2_HUMAN)
Q70J99	Protein unc-13 homolog D OS=Homo sapiens GN=UNC13D PE=1 SV=1 - (UN13D_HUMAN)
D6RER6	Coproporphyrinogen-III oxidase, mitochondrial OS=Homo sapiens GN=CPOX PE=4 SV=1 - (D6RER6_HUMAN)
Q92623	Tetratricopeptide repeat protein 9A OS=Homo sapiens GN=TTC9 PE=2 SV=3 - (TTC9A_HUMAN)
Q9ULV0	Unconventional myosin-Vb OS=Homo sapiens GN=MYO5B PE=1 SV=3 - (MYO5B_HUMAN)
Q16658	Fascin OS=Homo sapiens GN=FSCN1 PE=1 SV=3 - (FSCN1_HUMAN)
B7Z9M9	cDNA, FLJ78893, highly similar to Destrin OS=Homo sapiens PE=2 SV=1 - (B7Z9M9_HUMAN)
B4E0S6	cDNA FLJ55635, highly similar to pre-mRNA-splicing factorATP-dependent RNA helicase DHX15 (EC 3.6.1.-) OS=Homo sapiens PE=2 SV=1 - (B4E0S6_HUMAN)
Q9H0E3-2	Isoform 2 of Histone deacetylase complex subunit SAP130 OS=Homo sapiens GN=SAP130 - (SP130_HUMAN)
E9PJD9	60S ribosomal protein L27a OS=Homo sapiens GN=RPL27A PE=3 SV=1 - (E9PJD9_HUMAN)
Q5T0R7	Adenylyl cyclase-associated protein (Fragment) OS=Homo sapiens GN=CAP1 PE=2 SV=1 - (Q5T0R7_HUMAN)
B7Z1C9	Chaperonin containing TCP1, subunit 7 (Eta), isoform CRA_a OS=Homo sapiens GN=CCT7 PE=2 SV=1 - (B7Z1C9_HUMAN)
A9UFC0	Caspase 14 OS=Homo sapiens GN=CASP14 PE=2 SV=1 - (A9UFC0_HUMAN)
D3WYY8	Cytochrome c oxidase subunit 2 (Fragment) OS=Homo sapiens PE=4 SV=1 - (D3WYY8_HUMAN)
F2Z393	Transaldolase OS=Homo sapiens GN=TALDO1 PE=3 SV=1 - (F2Z393_HUMAN)
B2R8A3	cDNA, FLJ93805, highly similar to Homo sapiens creatine kinase, mitochondrial 2 (sarcomeric) (CKMT2), nuclear gene encoding mitochondrial protein, mRNA OS=Homo sapiens PE=2 SV=1 - (B2R8A3_HUMAN)
Q12981	Vesicle transport protein SEC20 OS=Homo sapiens GN=BNIP1 PE=1 SV=3 - (SEC20_HUMAN)
Q8IYE0	Coiled-coil domain-containing protein 146 OS=Homo sapiens GN=CCDC146 PE=2 SV=2 - (CC146_HUMAN)
Q68DE3	Basic helix-loop-helix domain-containing protein KIAA2018 OS=Homo sapiens GN=KIAA2018 PE=1 SV=3 - (K2018_HUMAN)
Q8NE71-2	Isoform 2 of ATP-binding cassette sub-family F member 1 OS=Homo sapiens GN=ABCF1 - (ABCF1_HUMAN)
A5YM43	PHC3 protein OS=Homo sapiens GN=PHC3 PE=2 SV=1 - (A5YM43_HUMAN)
G3V2K4	Brain-enriched guanylate kinase-associated protein (Fragment) OS=Homo sapiens GN=BEGAIN PE=4 SV=1 - (G3V2K4_HUMAN)
Q9Y4K1	Absent in melanoma 1 protein OS=Homo sapiens GN=AIM1 PE=1 SV=3 - (AIM1_HUMAN)
G3V3A0	Alpha-1-antichymotrypsin OS=Homo sapiens GN=SERPINA3 PE=3 SV=1 - (G3V3A0_HUMAN)
A8K2E6	Hermansky-Pudlak syndrome 4, isoform CRA_b OS=Homo sapiens GN=HPS4 PE=2 SV=1 - (A8K2E6_HUMAN)
E9PG49	Sodium channel protein type 1 subunit alpha OS=Homo sapiens GN=SCN1A PE=3 SV=1 - (E9PG49_HUMAN)
Q9UKF2-2	Isoform Beta of Disintegrin and metalloproteinase domain-containing protein 30 OS=Homo sapiens GN=ADAM30 - (ADA30_HUMAN)
O15013-4	Isoform 4 of Rho guanine nucleotide exchange factor 10 OS=Homo sapiens GN=ARHGEF10 - (ARHGA_HUMAN)
Q59H50	Integrin beta (Fragment) OS=Homo sapiens PE=1 SV=1 - (Q59H50_HUMAN)
Q08211	ATP-dependent RNA helicase A OS=Homo sapiens GN=DHX9 PE=1 SV=4 - (DHX9_HUMAN)
Q9HA11	Histone H2A OS=Homo sapiens PE=2 SV=1 - (Q9HA11_HUMAN)
P11217-2	Isoform 2 of Glycogen phosphorylase, muscle form OS=Homo sapiens GN=PYGM - (PYGM_HUMAN)
Q9NRZ7-2	Isoform 2 of 1-acyl-sn-glycerol-3-phosphate acyltransferase gamma OS=Homo sapiens GN=AGPAT3 - (PLCC_HUMAN)
Q13422-7	Isoform Ik7 of DNA-binding protein Ikaros OS=Homo sapiens GN=IKZF1 - (IKZF1_HUMAN)
Q5TG40	DNA methyltransferase 1 associated protein 1 (Fragment) OS=Homo sapiens GN=DMAP1 PE=2 SV=1 - (Q5TG40_HUMAN)
B3KN82	cDNA FLJ13913 fis, clone Y79AA1000231, highly similar to Nucleolar protein NOP5 OS=Homo sapiens PE=2 SV=1 - (B3KN82_HUMAN)
B2RBA0	cDNA, FLJ95388, highly similar to Homo sapiens step II splicing factor SLU7 (SLU7), mRNA OS=Homo sapiens PE=2 SV=1 - (B2RBA0_HUMAN)
F4MH93	Ubiquitously transcribed tetratricopeptide repeat protein Y-linked transcript variant 246 OS=Homo sapiens GN=UTY PE=2 SV=1 - (F4MH93_HUMAN)
Q76N57	Ribosomal protein S26 (Fragment) OS=Homo sapiens PE=2 SV=1 - (Q76N57_HUMAN)
E9PQM0	Protein-tyrosine phosphatase mitochondrial 1 OS=Homo sapiens GN=PTPMT1 PE=4 SV=1 - (E9PQM0_HUMAN)
Q9P241	Probable phospholipid-transporting ATPase VD OS=Homo sapiens GN=ATP10D PE=2 SV=3 - (AT10D_HUMAN)
Q4G134	NUDT13 protein OS=Homo sapiens GN=NUDT13 PE=2 SV=1 - (Q4G134_HUMAN)
B2R5M1	cDNA, FLJ92527, highly similar to Homo sapiens cytochrome P450, family 17, subfamily A, polypeptide 1 (CYP17A1), mRNA OS=Homo sapiens PE=2 SV=1 - (B2R5M1_HUMAN)
Q8IYY4-2	Isoform 2 of Zinc finger protein DZIP1L OS=Homo sapiens GN=DZIP1L - (DZI1L_HUMAN)
Q5SYZ4	Cellular retinoic acid binding protein 2 (Fragment) OS=Homo sapiens GN=CRABP2 PE=2 SV=1 - (Q5SYZ4_HUMAN)
B3KTF6	cDNA FLJ38174 fis, clone FCBBF1000061, highly similar to Antizyme inhibitor 1 OS=Homo sapiens PE=2 SV=1 - (B3KTF6_HUMAN)
Q9UPP2	IQ motif and SEC7 domain-containing protein 3 OS=Homo sapiens GN=IQSEC3 PE=2 SV=3 - (IQEC3_HUMAN)
I3NI21	Promethin (Fragment) OS=Homo sapiens GN=TMEM159 PE=4 SV=1 - (I3NI21_HUMAN)
B3KQ30	Iron-sulfur cluster assembly enzyme ISCU, mitochondrial OS=Homo sapiens GN=ISCU PE=2 SV=1 - (B3KQ30_HUMAN)
Q9Y5Z0-4	Isoform 4 of Beta-secretase 2 OS=Homo sapiens GN=BACE2 - (BACE2_HUMAN)
P61077-3	Isoform 3 of Ubiquitin-conjugating enzyme E2 D3 OS=Homo sapiens GN=UBE2D3 - (UB2D3_HUMAN)
H0YCY8	Dipeptidyl peptidase 1 light chain (Fragment) OS=Homo sapiens GN=CTSC PE=4 SV=1 - (H0YCY8_HUMAN)
B8X2Z3	CARMIL2b OS=Homo sapiens GN=RLTPR PE=2 SV=1 - (B8X2Z3_HUMAN)
Q9NS37	CREB/ATF bZIP transcription factor OS=Homo sapiens GN=CREBZF PE=1 SV=2 - (ZHANG_HUMAN)
P48739-2	Isoform 2 of Phosphatidylinositol transfer protein beta isoform OS=Homo sapiens GN=PITPNB - (PIPNB_HUMAN)
A8K132	cDNA FLJ75476, highly similar to Homo sapiens glutaminase (GLS), mRNA OS=Homo sapiens PE=2 SV=1 - (A8K132_HUMAN)
E9PLD0	Ras-related protein Rab-1B OS=Homo sapiens GN=RAB1B PE=3 SV=1 - (E9PLD0_HUMAN)
B4E0W3	cDNA FLJ60386, moderately similar to Signal-induced proliferation-associated protein 1 OS=Homo sapiens PE=2 SV=1 - (B4E0W3_HUMAN)
D6W5P0	Cyclin D binding myb-like transcription factor 1, isoform CRA_a OS=Homo sapiens GN=DMTF1 PE=4 SV=1 - (D6W5P0_HUMAN)
C9JZ20	Prohibitin (Fragment) OS=Homo sapiens GN=PHB PE=4 SV=1 - (C9JZ20_HUMAN)
P42695	Condensin-2 complex subunit D3 OS=Homo sapiens GN=NCAPD3 PE=1 SV=2 - (CNDD3_HUMAN)
Q5QNZ2	ATP synthase subunit b, mitochondrial OS=Homo sapiens GN=ATP5F1 PE=2 SV=1 - (Q5QNZ2_HUMAN)
H7BY24	Centrosomal protein of 55 kDa OS=Homo sapiens GN=CEP55 PE=4 SV=1 - (H7BY24_HUMAN)
Q8TCA0-3	Isoform 3 of Leucine-rich repeat-containing protein 20 OS=Homo sapiens GN=LRRC20 - (LRC20_HUMAN)
Q7Z5N2	Kangai 1 OS=Homo sapiens GN=KAI1 PE=2 SV=1 - (Q7Z5N2_HUMAN)
E5RIW3	Tubulin-specific chaperone A OS=Homo sapiens GN=TBCA PE=4 SV=1 - (E5RIW3_HUMAN)
Q59FS3	Ribosomal protein S7 variant (Fragment) OS=Homo sapiens PE=2 SV=1 - (Q59FS3_HUMAN)
B7Z7G8	cDNA FLJ53530 OS=Homo sapiens PE=2 SV=1 - (B7Z7G8_HUMAN)
A7E2S3	RPS3 protein (Fragment) OS=Homo sapiens GN=RPS3 PE=2 SV=1 - (A7E2S3_HUMAN)
B4DUZ6	cDNA FLJ56608, highly similar to Homo sapiens chloride channel, calcium activated, family member 1 (CLCA1), mRNA OS=Homo sapiens PE=2 SV=1 - (B4DUZ6_HUMAN)
H3BPQ4	Hydroxyacylglutathione hydrolase, mitochondrial OS=Homo sapiens GN=HAGH PE=3 SV=1 - (H3BPQ4_HUMAN)
Q6ZUA9	cDNA FLJ43860 fis, clone TESTI4007404 OS=Homo sapiens PE=2 SV=1 - (Q6ZUA9_HUMAN)
Q9NQR4	Omega-amidase NIT2 OS=Homo sapiens GN=NIT2 PE=1 SV=1 - (NIT2_HUMAN)
E9PJP5	Junctional adhesion molecule B OS=Homo sapiens GN=JAM2 PE=4 SV=1 - (E9PJP5_HUMAN)
F8W9N0	Tubulin polyglutamylase TTLL6 OS=Homo sapiens GN=TTLL6 PE=4 SV=1 - (F8W9N0_HUMAN)
O00515	Ladinin-1 OS=Homo sapiens GN=LAD1 PE=1 SV=2 - (LAD1_HUMAN)
Q8NCA9	Zinc finger protein 784 OS=Homo sapiens GN=ZNF784 PE=2 SV=1 - (ZN784_HUMAN)
E9PBH2	Leucine-rich repeat-containing protein 34 OS=Homo sapiens GN=LRRC34 PE=4 SV=1 - (E9PBH2_HUMAN)
Q8IWV2-3	Isoform 3 of Contactin-4 OS=Homo sapiens GN=CNTN4 - (CNTN4_HUMAN)
A8K1I4	cDNA FLJ78426 OS=Homo sapiens PE=2 SV=1 - (A8K1I4_HUMAN)
B4E2Q6	Regulation of nuclear pre-mRNA domain-containing protein 2 OS=Homo sapiens GN=RPRD2 PE=2 SV=1 - (B4E2Q6_HUMAN)
Q9UJW2-2	Isoform 2 of Tubulointerstitial nephritis antigen OS=Homo sapiens GN=TINAG - (TINAG_HUMAN)
Q9Y520-6	Isoform 6 of Protein PRRC2C OS=Homo sapiens GN=PRRC2C - (PRC2C_HUMAN)
Q8TE68-2	Isoform 2 of Epidermal growth factor receptor kinase substrate 8-like protein 1 OS=Homo sapiens GN=EPS8L1 - (ES8L1_HUMAN)
F8VQQ8	Ubiquitin-conjugating enzyme E2 N OS=Homo sapiens GN=UBE2N PE=3 SV=1 - (F8VQQ8_HUMAN)
O60518	Ran-binding protein 6 OS=Homo sapiens GN=RANBP6 PE=1 SV=2 - (RNBP6_HUMAN)
B7Z2Z8	T-complex protein 1 subunit delta OS=Homo sapiens PE=2 SV=1 - (B7Z2Z8_HUMAN)
H0YFX4	4F2 cell-surface antigen heavy chain (Fragment) OS=Homo sapiens GN=SLC3A2 PE=4 SV=1 - (H0YFX4_HUMAN)
C9J6I7	UDP-GalNAc:beta-1,3-N-acetylgalactosaminyltransferase 1 (Fragment) OS=Homo sapiens GN=B3GALNT1 PE=4 SV=1 - (C9J6I7_HUMAN)
Q59GW1	High-mobility group box 1 variant (Fragment) OS=Homo sapiens PE=2 SV=1 - (Q59GW1_HUMAN)
P68036	Ubiquitin-conjugating enzyme E2 L3 OS=Homo sapiens GN=UBE2L3 PE=1 SV=1 - (UB2L3_HUMAN)
B4DLC0	Poly(rC)-binding protein 2 OS=Homo sapiens GN=PCBP2 PE=2 SV=1 - (B4DLC0_HUMAN)
E9PH29	Thioredoxin-dependent peroxide reductase, mitochondrial OS=Homo sapiens GN=PRDX3 PE=4 SV=1 - (E9PH29_HUMAN)
G3V5M2	Purine nucleoside phosphorylase (Fragment) OS=Homo sapiens GN=PNP PE=4 SV=1 - (G3V5M2_HUMAN)
A8K0D2	cDNA FLJ77740, highly similar to Homo sapiens 7-dehydrocholesterol reductase, mRNA OS=Homo sapiens PE=2 SV=1 - (A8K0D2_HUMAN)
B7ZKY6	Membrane metallo-endopeptidase OS=Homo sapiens GN=MME PE=2 SV=1 - (B7ZKY6_HUMAN)
Q04941	Proteolipid protein 2 OS=Homo sapiens GN=PLP2 PE=1 SV=1 - (PLP2_HUMAN)
Q8NFI4	Putative protein FAM10A5 OS=Homo sapiens GN=ST13P5 PE=5 SV=1 - (F10A5_HUMAN)
P51801	Chloride channel protein ClC-Kb OS=Homo sapiens GN=CLCNKB PE=1 SV=3 - (CLCKB_HUMAN)
E9PJU8	Ester hydrolase C11orf54 (Fragment) OS=Homo sapiens GN=C11orf54 PE=4 SV=1 - (E9PJU8_HUMAN)
Q9NTX5-6	Isoform 6 of Ethylmalonyl-CoA decarboxylase OS=Homo sapiens GN=ECHDC1 - (ECHD1_HUMAN)
H0YDQ0	FYVE, RhoGEF and PH domain-containing protein 4 (Fragment) OS=Homo sapiens GN=FGD4 PE=4 SV=1 - (H0YDQ0_HUMAN)
B3KXK4	cDNA FLJ45605 fis, clone BRTHA3021971, highly similar to Nuclear receptor coactivator 7 OS=Homo sapiens PE=2 SV=1 - (B3KXK4_HUMAN)
Q8N5Z0-2	Isoform 2 of Kynurenine/alpha-aminoadipate aminotransferase, mitochondrial OS=Homo sapiens GN=AADAT - (AADAT_HUMAN)
H0YE60	UPF0722 protein C11orf88 (Fragment) OS=Homo sapiens GN=C11orf88 PE=4 SV=1 - (H0YE60_HUMAN)
Q6UXN8	C-type lectin domain family 9 member A OS=Homo sapiens GN=CLEC9A PE=1 SV=1 - (CLC9A_HUMAN)
Q96IV0-5	Isoform 5 of Peptide-N(4)-(N-acetyl-beta-glucosaminyl)asparagine amidase OS=Homo sapiens GN=NGLY1 - (NGLY1_HUMAN)
Q8WU49	Uncharacterized protein C7orf33 OS=Homo sapiens GN=C7orf33 PE=2 SV=1 - (CG033_HUMAN)
Q9BRA2	Thioredoxin domain-containing protein 17 OS=Homo sapiens GN=TXNDC17 PE=1 SV=1 - (TXD17_HUMAN)
Q6WQI6	Putative cancer susceptibility gene HEPN1 protein OS=Homo sapiens GN=HEPN1 PE=5 SV=2 - (HEPN1_HUMAN)
H0YCU9	Transgelin (Fragment) OS=Homo sapiens GN=TAGLN PE=4 SV=1 - (H0YCU9_HUMAN)
O75051	Plexin-A2 OS=Homo sapiens GN=PLXNA2 PE=1 SV=4 - (PLXA2_HUMAN)
P51814-4	Isoform 4 of Zinc finger protein 41 OS=Homo sapiens GN=ZNF41 - (ZNF41_HUMAN)
O00522-3	Isoform 3 of Krev interaction trapped protein 1 OS=Homo sapiens GN=KRIT1 - (KRIT1_HUMAN)
P61106	Ras-related protein Rab-14 OS=Homo sapiens GN=RAB14 PE=1 SV=4 - (RAB14_HUMAN)
Q9UJA3-2	Isoform 2 of DNA replication licensing factor MCM8 OS=Homo sapiens GN=MCM8 - (MCM8_HUMAN)
F8W7I0	2',3'-cyclic-nucleotide 3'-phosphodiesterase OS=Homo sapiens GN=CNP PE=4 SV=1 - (F8W7I0_HUMAN)
Q14427	GM2-activator protein (Fragment) OS=Homo sapiens PE=2 SV=1 - (Q14427_HUMAN)
H0Y9B1	Interleukin-6 receptor subunit alpha (Fragment) OS=Homo sapiens GN=IL6R PE=4 SV=1 - (H0Y9B1_HUMAN)
H0Y9T5	m7GpppN-mRNA hydrolase (Fragment) OS=Homo sapiens GN=DCP2 PE=3 SV=1 - (H0Y9T5_HUMAN)
O15482	Testis-specific protein TEX28 OS=Homo sapiens GN=TEX28 PE=2 SV=1 - (TEX28_HUMAN)
D0QYQ8	MYB/NFIB fusion protein variant 1 (Fragment) OS=Homo sapiens GN=MYB/NFIB fusion PE=2 SV=1 - (D0QYQ8_HUMAN)
A9XAC0	Alpha-helix coiled-coil rod homologue (Fragment) OS=Homo sapiens GN=HCR PE=4 SV=1 - (A9XAC0_HUMAN)
H3BNI0	Zinc finger protein 19 OS=Homo sapiens GN=ZNF19 PE=4 SV=1 - (H3BNI0_HUMAN)
Q5H9S0	Putative uncharacterized protein DKFZp781N1974 OS=Homo sapiens GN=DKFZp781N1974 PE=2 SV=1 - (Q5H9S0_HUMAN)
Q86VY2	Copine-8 OS=Homo sapiens GN=CPNE8 PE=2 SV=1 - (Q86VY2_HUMAN)
Q7L8L6	FAST kinase domain-containing protein 5 OS=Homo sapiens GN=FASTKD5 PE=1 SV=1 - (FAKD5_HUMAN)
P22460	Potassium voltage-gated channel subfamily A member 5 OS=Homo sapiens GN=KCNA5 PE=1 SV=4 - (KCNA5_HUMAN)
Q6PJI0	Potassium intermediate/small conductance calcium-activated channel, subfamily N, member 2 OS=Homo sapiens GN=KCNN2 PE=2 SV=1 - (Q6PJI0_HUMAN)
Q53HD3	60S ribosomal protein L18a (Fragment) OS=Homo sapiens PE=2 SV=1 - (Q53HD3_HUMAN)
Q9H869	YY1-associated protein 1 OS=Homo sapiens GN=YY1AP1 PE=1 SV=2 - (YYAP1_HUMAN)
H7C2D4	Nuclear receptor corepressor 2 (Fragment) OS=Homo sapiens GN=NCOR2 PE=4 SV=1 - (H7C2D4_HUMAN)
Q3LTJ6	MHC class II antigen (Fragment) OS=Homo sapiens GN=HLA-DRB1 PE=4 SV=1 - (Q3LTJ6_HUMAN)
P62316	Small nuclear ribonucleoprotein Sm D2 OS=Homo sapiens GN=SNRPD2 PE=1 SV=1 - (SMD2_HUMAN)
P24588	A-kinase anchor protein 5 OS=Homo sapiens GN=AKAP5 PE=1 SV=3 - (AKAP5_HUMAN)
Q8N8A2	Serine/threonine-protein phosphatase 6 regulatory ankyrin repeat subunit B OS=Homo sapiens GN=ANKRD44 PE=1 SV=3 - (ANR44_HUMAN)
O00548	Delta-like protein 1 OS=Homo sapiens GN=DLL1 PE=2 SV=2 - (DLL1_HUMAN)
P54851	Epithelial membrane protein 2 OS=Homo sapiens GN=EMP2 PE=2 SV=1 - (EMP2_HUMAN)
Q9NQE9	Histidine triad nucleotide-binding protein 3 OS=Homo sapiens GN=HINT3 PE=1 SV=1 - (HINT3_HUMAN)
Q4G112	Heat shock factor protein 5 OS=Homo sapiens GN=HSF5 PE=2 SV=2 - (HSF5_HUMAN)
Q5JT82	Krueppel-like factor 17 OS=Homo sapiens GN=KLF17 PE=1 SV=1 - (KLF17_HUMAN)
Q8TE12	LIM homeobox transcription factor 1-alpha OS=Homo sapiens GN=LMX1A PE=2 SV=1 - (LMX1A_HUMAN)
Q9BT67	NEDD4 family-interacting protein 1 OS=Homo sapiens GN=NDFIP1 PE=1 SV=1 - (NFIP1_HUMAN)
Q0D2K0-2	Isoform 2 of Magnesium transporter NIPA4 OS=Homo sapiens GN=NIPAL4 - (NIPA4_HUMAN)
Q92932-2	Isoform 2 of Receptor-type tyrosine-protein phosphatase N2 OS=Homo sapiens GN=PTPRN2 - (PTPR2_HUMAN)
Q9HAU5	Regulator of nonsense transcripts 2 OS=Homo sapiens GN=UPF2 PE=1 SV=1 - (RENT2_HUMAN)
Q96SB8	Structural maintenance of chromosomes protein 6 OS=Homo sapiens GN=SMC6 PE=1 SV=2 - (SMC6_HUMAN)
Q13573	SNW domain-containing protein 1 OS=Homo sapiens GN=SNW1 PE=1 SV=1 - (SNW1_HUMAN)
Q86UX6	Serine/threonine-protein kinase 32C OS=Homo sapiens GN=STK32C PE=1 SV=1 - (ST32C_HUMAN)
Q9UJW7	Zinc finger protein 229 OS=Homo sapiens GN=ZNF229 PE=2 SV=3 - (ZN229_HUMAN)
Q8N184-2	Isoform 2 of Zinc finger protein 567 OS=Homo sapiens GN=ZNF567 - (ZN567_HUMAN)
Q6PDB4	Zinc finger protein 880 OS=Homo sapiens GN=ZNF880 PE=2 SV=2 - (ZN880_HUMAN)
B4DP65	EMG1 nucleolar protein homolog (S. cerevisiae), isoform CRA_a OS=Homo sapiens GN=EMG1 PE=2 SV=1 - (B4DP65_HUMAN)
Q7L3Y3	HCG1785781 OS=Homo sapiens GN=PSD3 PE=4 SV=1 - (Q7L3Y3_HUMAN)
C9JD75	Biotin--(acetyl-CoA-carboxylase) ligase (Fragment) OS=Homo sapiens GN=HLCS PE=4 SV=1 - (C9JD75_HUMAN)
B4DQK8	cDNA FLJ54750, moderately similar to Pyrroline-5-carboxylate reductase 2 (EC 1.5.1.2) OS=Homo sapiens PE=2 SV=1 - (B4DQK8_HUMAN)
E7EWB4	Dihydropyrimidinase-related protein 5 (Fragment) OS=Homo sapiens GN=DPYSL5 PE=4 SV=1 - (E7EWB4_HUMAN)
A2BF29	Major histocompatibility complex class I B (Fragment) OS=Homo sapiens GN=HLA-B PE=3 SV=1 - (A2BF29_HUMAN)
B4DDX6	cDNA FLJ57296 OS=Homo sapiens PE=2 SV=1 - (B4DDX6_HUMAN)
C9JZZ1	Rabenosyn-5 OS=Homo sapiens GN=ZFYVE20 PE=4 SV=1 - (C9JZZ1_HUMAN)
B7Z284	DNA-directed RNA polymerase OS=Homo sapiens PE=2 SV=1 - (B7Z284_HUMAN)
B4E2C8	cDNA FLJ61064, highly similar to Homo sapiens DEAD/H (Asp-Glu-Ala-Asp/His) box polypeptide 26 (DDX26), mRNA OS=Homo sapiens PE=2 SV=1 - (B4E2C8_HUMAN)
B4E0E0	cDNA FLJ54898, highly similar to Homo sapiens transmembrane protein 41B (TMEM41B), mRNA OS=Homo sapiens PE=2 SV=1 - (B4E0E0_HUMAN)
B4E053	cDNA FLJ61195, highly similar to Ubiquitin thioesterase protein OTUB1 (EC 3.4.-.-) OS=Homo sapiens PE=2 SV=1 - (B4E053_HUMAN)
B4E2F2	cDNA FLJ61068, highly similar to Amyloid beta A4 protein-bindingfamily B member 2 (Fe65-like protein) OS=Homo sapiens PE=2 SV=1 - (B4E2F2_HUMAN)
H0YDB6	PIH1 domain-containing protein 2 (Fragment) OS=Homo sapiens GN=PIH1D2 PE=4 SV=1 - (H0YDB6_HUMAN)
Q8NAG5	cDNA FLJ35389 fis, clone SKNMC2000635, moderately similar to 65 kDa FK506-BINDING PROTEIN (EC 5.2.1.8) OS=Homo sapiens PE=2 SV=1 - (Q8NAG5_HUMAN)
E7EXA5	Exocyst complex component 3-like protein OS=Homo sapiens GN=EXOC3L1 PE=4 SV=1 - (E7EXA5_HUMAN)
Q16234	HuD protein OS=Homo sapiens GN=HuD PE=4 SV=1 - (Q16234_HUMAN)
Q6PI41	AURKAIP1 protein (Fragment) OS=Homo sapiens GN=AURKAIP1 PE=2 SV=1 - (Q6PI41_HUMAN)
O76013	Keratin, type I cuticular Ha6 OS=Homo sapiens GN=KRT36 PE=1 SV=1 - (KRT36_HUMAN)
B0YJC4	Vimentin OS=Homo sapiens GN=VIM PE=3 SV=1 - (B0YJC4_HUMAN)
Q05BI1	CD3EAP protein (Fragment) OS=Homo sapiens GN=CD3EAP PE=2 SV=1 - (Q05BI1_HUMAN)
O43149	Zinc finger ZZ-type and EF-hand domain-containing protein 1 OS=Homo sapiens GN=ZZEF1 PE=1 SV=6 - (ZZEF1_HUMAN)
Q8IZT6	Abnormal spindle-like microcephaly-associated protein OS=Homo sapiens GN=ASPM PE=1 SV=2 - (ASPM_HUMAN)
P12036-2	Isoform 2 of Neurofilament heavy polypeptide OS=Homo sapiens GN=NEFH - (NFH_HUMAN)
Q2TSD0	Glyceraldehyde-3-phosphate dehydrogenase OS=Homo sapiens PE=2 SV=1 - (Q2TSD0_HUMAN)
O75369-8	Isoform 8 of Filamin-B OS=Homo sapiens GN=FLNB - (FLNB_HUMAN)
B4DYS1	cDNA FLJ50921, highly similar to Eukaryotic translation initiation factor 3 subunit 10 OS=Homo sapiens PE=2 SV=1 - (B4DYS1_HUMAN)
Q8NCY6	Myb/SANT-like DNA-binding domain-containing protein 4 OS=Homo sapiens GN=MSANTD4 PE=1 SV=1 - (MSD4_HUMAN)
Q8N5Z7	60S ribosomal protein L6 OS=Homo sapiens GN=RPL6 PE=2 SV=1 - (Q8N5Z7_HUMAN)
P62937	Peptidyl-prolyl cis-trans isomerase A OS=Homo sapiens GN=PPIA PE=1 SV=2 - (PPIA_HUMAN)
C0JYZ1	Dynein, axonemal, heavy chain 11 OS=Homo sapiens GN=DNAH11 PE=4 SV=1 - (C0JYZ1_HUMAN)
A2RUB1	Uncharacterized protein C17orf104 OS=Homo sapiens GN=C17orf104 PE=2 SV=3 - (CQ104_HUMAN)
Q8TDJ6-2	Isoform 2 of DmX-like protein 2 OS=Homo sapiens GN=DMXL2 - (DMXL2_HUMAN)
Q3V6T2-2	Isoform 2 of Girdin OS=Homo sapiens GN=CCDC88A - (GRDN_HUMAN)
B7Z4V2	Stress-70 protein, mitochondrial OS=Homo sapiens GN=HSPA9 PE=2 SV=1 - (B7Z4V2_HUMAN)
E7ETI5	G-protein-coupled receptor 98 OS=Homo sapiens GN=GPR98 PE=4 SV=1 - (E7ETI5_HUMAN)
P08107	Heat shock 70 kDa protein 1A/1B OS=Homo sapiens GN=HSPA1A PE=1 SV=5 - (HSP71_HUMAN)
Q5MJQ3	Potassium voltage-gated channel beta subunit OS=Homo sapiens GN=KCNAB1 PE=2 SV=1 - (Q5MJQ3_HUMAN)
Q8WZ74	Cortactin-binding protein 2 OS=Homo sapiens GN=CTTNBP2 PE=1 SV=1 - (CTTB2_HUMAN)
Q6ZNS7	CDNA FLJ27229 fis, clone SYN05701 OS=Homo sapiens PE=2 SV=1 - (Q6ZNS7_HUMAN)
O94832	Unconventional myosin-Id OS=Homo sapiens GN=MYO1D PE=1 SV=2 - (MYO1D_HUMAN)
Q6IQ47	PARD3 protein OS=Homo sapiens GN=PARD3 PE=2 SV=1 - (Q6IQ47_HUMAN)
Q2M1J6	Oxidase (Cytochrome c) assembly 1-like OS=Homo sapiens GN=OXA1L PE=2 SV=1 - (Q2M1J6_HUMAN)
Q6ZMD1	cDNA FLJ23994 fis, clone HRC11286 OS=Homo sapiens PE=2 SV=1 - (Q6ZMD1_HUMAN)
E7EP17	Dynein heavy chain 9, axonemal OS=Homo sapiens GN=DNAH9 PE=4 SV=1 - (E7EP17_HUMAN)
Q13332-6	Isoform 2 of Receptor-type tyrosine-protein phosphatase S OS=Homo sapiens GN=PTPRS - (PTPRS_HUMAN)
Q8TE73	Dynein heavy chain 5, axonemal OS=Homo sapiens GN=DNAH5 PE=1 SV=3 - (DYH5_HUMAN)
P42858	Huntingtin OS=Homo sapiens GN=HTT PE=1 SV=2 - (HD_HUMAN)
B4DRT3	Pyruvate kinase OS=Homo sapiens PE=2 SV=1 - (B4DRT3_HUMAN)
F8WCW5	Hyaluronan mediated motility receptor OS=Homo sapiens GN=HMMR PE=4 SV=1 - (F8WCW5_HUMAN)
Q5SQQ9	Ventral anterior homeobox 1 OS=Homo sapiens GN=VAX1 PE=1 SV=1 - (VAX1_HUMAN)
Q59FI9	Ribosomal protein L12 variant (Fragment) OS=Homo sapiens PE=2 SV=1 - (Q59FI9_HUMAN)
Q08378-2	Isoform 2 of Golgin subfamily A member 3 OS=Homo sapiens GN=GOLGA3 - (GOGA3_HUMAN)
Q59FC6	Tumor rejection antigen (Gp96) 1 variant (Fragment) OS=Homo sapiens PE=2 SV=1 - (Q59FC6_HUMAN)
Q9NZE6	BM-010 OS=Homo sapiens GN=EIF4A2 PE=2 SV=1 - (Q9NZE6_HUMAN)
C9K0F9	Histone acetyltransferase KAT6A (Fragment) OS=Homo sapiens GN=KAT6A PE=4 SV=1 - (C9K0F9_HUMAN)
H0YL80	Tropomyosin alpha-1 chain (Fragment) OS=Homo sapiens GN=TPM1 PE=4 SV=1 - (H0YL80_HUMAN)
Q15058	Kinesin-like protein KIF14 OS=Homo sapiens GN=KIF14 PE=1 SV=1 - (KIF14_HUMAN)
B2R4P2	cDNA, FLJ92164, highly similar to Homo sapiens peroxiredoxin 1 (PRDX1), mRNA OS=Homo sapiens PE=2 SV=1 - (B2R4P2_HUMAN)
Q8TBY8	Polyamine-modulated factor 1-binding protein 1 OS=Homo sapiens GN=PMFBP1 PE=2 SV=1 - (PMFBP_HUMAN)
E9PJL5	Putative uncharacterized protein C12orf63 OS=Homo sapiens GN=C12orf63 PE=4 SV=1 - (E9PJL5_HUMAN)
I1E4Y4	Meiosis arrest female protein 1 OS=Homo sapiens GN=KIAA0430 PE=4 SV=1 - (I1E4Y4_HUMAN)
F5GZT0	Meiosis inhibitor protein 1 OS=Homo sapiens GN=MEI1 PE=4 SV=1 - (F5GZT0_HUMAN)
Q8NB66	Protein unc-13 homolog C OS=Homo sapiens GN=UNC13C PE=1 SV=3 - (UN13C_HUMAN)
Q96NX9-2	Isoform 2 of Dachshund homolog 2 OS=Homo sapiens GN=DACH2 - (DACH2_HUMAN)
Q01082	Spectrin beta chain, brain 1 OS=Homo sapiens GN=SPTBN1 PE=1 SV=2 - (SPTB2_HUMAN)
D6R9P1	Ectonucleotide pyrophosphatase/phosphodiesterase family member 6 soluble form (Fragment) OS=Homo sapiens GN=ENPP6 PE=4 SV=1 - (D6R9P1_HUMAN)
H0YF27	Tripartite motif-containing protein 29 (Fragment) OS=Homo sapiens GN=TRIM29 PE=4 SV=1 - (H0YF27_HUMAN)
Q9P2K1	Coiled-coil and C2 domain-containing protein 2A OS=Homo sapiens GN=CC2D2A PE=1 SV=3 - (C2D2A_HUMAN)
Q6P2D8-3	Isoform 3 of X-ray radiation resistance-associated protein 1 OS=Homo sapiens GN=XRRA1 - (XRRA1_HUMAN)
B7ZVZ7	BZRAP1 protein OS=Homo sapiens GN=BZRAP1 PE=2 SV=1 - (B7ZVZ7_HUMAN)
B7Z2X4	cDNA FLJ53327, highly similar to Gelsolin OS=Homo sapiens PE=2 SV=1 - (B7Z2X4_HUMAN)
Q9Y3P9-3	Isoform 3 of Rab GTPase-activating protein 1 OS=Homo sapiens GN=RABGAP1 - (RBGP1_HUMAN)
Q8N4C6-10	Isoform 3 of Ninein OS=Homo sapiens GN=NIN - (NIN_HUMAN)
B2R7G4	cDNA, FLJ93435, highly similar to Homo sapiens general transcription factor IIH, polypeptide 1 (62kD subunit) (GTF2H1), mRNA OS=Homo sapiens PE=2 SV=1 - (B2R7G4_HUMAN)
Q59GY2	Ribosomal protein L4 variant (Fragment) OS=Homo sapiens PE=2 SV=1 - (Q59GY2_HUMAN)
B3KX91	cDNA FLJ44992 fis, clone BRAWH3009961, highly similar to Regulating synaptic membrane exocytosis protein 2 OS=Homo sapiens PE=2 SV=1 - (B3KX91_HUMAN)
B7ZW38	LOC649330 protein OS=Homo sapiens GN=LOC649330 PE=2 SV=1 - (B7ZW38_HUMAN)
Q5VT06	Centrosome-associated protein 350 OS=Homo sapiens GN=CEP350 PE=1 SV=1 - (CE350_HUMAN)
F8W805	Metabotropic glutamate receptor 1 OS=Homo sapiens GN=GRM1 PE=3 SV=1 - (F8W805_HUMAN)
Q6IBG5	MYL6 protein OS=Homo sapiens GN=MYL6 PE=2 SV=1 - (Q6IBG5_HUMAN)
O14578	Citron Rho-interacting kinase OS=Homo sapiens GN=CIT PE=1 SV=2 - (CTRO_HUMAN)
A5D8W1-2	Isoform 2 of Uncharacterized protein C7orf63 OS=Homo sapiens GN=C7orf63 - (CG063_HUMAN)
Q9Y493-7	Isoform 7 of Zonadhesin OS=Homo sapiens GN=ZAN - (ZAN_HUMAN)
B3KXD3	cDNA FLJ45230 fis, clone BRCAN2021325, highly similar to Carboxypeptidase E (EC 3.4.17.10) OS=Homo sapiens PE=2 SV=1 - (B3KXD3_HUMAN)
Q8TCY9-3	Isoform 3 of Up-regulator of cell proliferation OS=Homo sapiens GN=URGCP - (URGCP_HUMAN)
A8K7H3	cDNA FLJ77670, highly similar to Homo sapiens ribosomal protein S15a (RPS15A), mRNA OS=Homo sapiens PE=2 SV=1 - (A8K7H3_HUMAN)
Q9ULT8	E3 ubiquitin-protein ligase HECTD1 OS=Homo sapiens GN=HECTD1 PE=1 SV=3 - (HECD1_HUMAN)
F8VWT9	Probable E3 ubiquitin-protein ligase C12orf51 OS=Homo sapiens GN=C12orf51 PE=4 SV=1 - (F8VWT9_HUMAN)
B7ZLW1	CAMSAP1 protein OS=Homo sapiens GN=CAMSAP1 PE=2 SV=1 - (B7ZLW1_HUMAN)
A8MYJ7	Tetratricopeptide repeat protein 34 OS=Homo sapiens GN=TTC34 PE=2 SV=2 - (TTC34_HUMAN)
H7C292	Phosphatidylinositol 3,4,5-trisphosphate 5-phosphatase 1 OS=Homo sapiens GN=INPP5D PE=4 SV=1 - (H7C292_HUMAN)
E7ENF9	Isochorismatase domain-containing protein 1 (Fragment) OS=Homo sapiens GN=ISOC1 PE=4 SV=1 - (E7ENF9_HUMAN)
O95466-2	Isoform 2 of Formin-like protein 1 OS=Homo sapiens GN=FMNL1 - (FMNL_HUMAN)
Q9NT68	Teneurin-2 OS=Homo sapiens GN=ODZ2 PE=1 SV=3 - (TEN2_HUMAN)
Q9C0C7-2	Isoform 2 of Activating molecule in BECN1-regulated autophagy protein 1 OS=Homo sapiens GN=AMBRA1 - (AMRA1_HUMAN)
Q53GR6	SH3 domain and tetratricopeptide repeats 1 variant (Fragment) OS=Homo sapiens PE=2 SV=1 - (Q53GR6_HUMAN)
Q6PIB3	KN motif and ankyrin repeat domains 1 OS=Homo sapiens GN=KANK1 PE=2 SV=1 - (Q6PIB3_HUMAN)
Q12766	HMG domain-containing protein 3 OS=Homo sapiens GN=HMGXB3 PE=2 SV=2 - (HMGX3_HUMAN)
O75976	Carboxypeptidase D OS=Homo sapiens GN=CPD PE=1 SV=2 - (CBPD_HUMAN)
Q53EQ3	Tubulin, gamma complex associated protein 2 variant (Fragment) OS=Homo sapiens PE=2 SV=1 - (Q53EQ3_HUMAN)
Q659C4-9	Isoform 9 of La-related protein 1B OS=Homo sapiens GN=LARP1B - (LAR1B_HUMAN)
Q8N1A4	Similar to phospholipase C, beta 3 (Phosphatidylinositol-specific) (Fragment) OS=Homo sapiens PE=2 SV=1 - (Q8N1A4_HUMAN)
Q6FG99	RPLP1 protein OS=Homo sapiens GN=RPLP1 PE=4 SV=1 - (Q6FG99_HUMAN)
Q92805	Golgin subfamily A member 1 OS=Homo sapiens GN=GOLGA1 PE=1 SV=3 - (GOGA1_HUMAN)
Q5T653	39S ribosomal protein L2, mitochondrial OS=Homo sapiens GN=MRPL2 PE=1 SV=2 - (RM02_HUMAN)
Q9ULL5-2	Isoform 2 of Proline-rich protein 12 OS=Homo sapiens GN=PRR12 - (PRR12_HUMAN)
Q6PCE3	Glucose 1,6-bisphosphate synthase OS=Homo sapiens GN=PGM2L1 PE=1 SV=3 - (PGM2L_HUMAN)
P55157	Microsomal triglyceride transfer protein large subunit OS=Homo sapiens GN=MTTP PE=1 SV=1 - (MTP_HUMAN)
G3V5X3	Cochlin (Fragment) OS=Homo sapiens GN=COCH PE=4 SV=1 - (G3V5X3_HUMAN)
H0YF99	Heparan sulfate glucosamine 3-O-sulfotransferase 2 OS=Homo sapiens GN=HS3ST2 PE=4 SV=1 - (H0YF99_HUMAN)
Q96NL6-3	Isoform 3 of Sodium channel and clathrin linker 1 OS=Homo sapiens GN=SCLT1 - (SCLT1_HUMAN)
Q6ZMW3-2	Isoform 2 of Echinoderm microtubule-associated protein-like 6 OS=Homo sapiens GN=EML6 - (EMAL6_HUMAN)
P63162	Small nuclear ribonucleoprotein-associated protein N OS=Homo sapiens GN=SNRPN PE=1 SV=1 - (RSMN_HUMAN)
D5FHQ7	MHC class I antigen (Fragment) OS=Homo sapiens GN=HLA-A PE=3 SV=1 - (D5FHQ7_HUMAN)
B3KWN0	cDNA FLJ43389 fis, clone OCBBF2007068, highly similar to Ankyrin repeat domain-containing protein 52 OS=Homo sapiens PE=2 SV=1 - (B3KWN0_HUMAN)
F1T0K4	DmX-like protein 1 OS=Homo sapiens GN=DMXL1 PE=2 SV=1 - (F1T0K4_HUMAN)
F5GXU8	Leucine-zipper-like transcriptional regulator 1 OS=Homo sapiens GN=LZTR1 PE=4 SV=1 - (F5GXU8_HUMAN)
B1AMD2	Ring finger and WD repeat domain 2 OS=Homo sapiens GN=RFWD2 PE=4 SV=1 - (B1AMD2_HUMAN)
Q86Y13	E3 ubiquitin-protein ligase DZIP3 OS=Homo sapiens GN=DZIP3 PE=1 SV=2 - (DZIP3_HUMAN)
B4DEI3	cDNA FLJ57715, highly similar to Voltage-dependent anion-selective channel protein 1 OS=Homo sapiens PE=2 SV=1 - (B4DEI3_HUMAN)
Q96BA7	HNRPU protein OS=Homo sapiens PE=2 SV=1 - (Q96BA7_HUMAN)
C9JNV4	Tripartite motif-containing protein 15 OS=Homo sapiens GN=TRIM15 PE=4 SV=1 - (C9JNV4_HUMAN)
B4DDC4	cDNA FLJ50435, highly similar to Homo sapiens nuclear domain 10 protein (NDP52), mRNA OS=Homo sapiens PE=2 SV=1 - (B4DDC4_HUMAN)
Q7Z6R9	Transcription factor AP-2-delta OS=Homo sapiens GN=TFAP2D PE=2 SV=1 - (AP2D_HUMAN)
H3BRU6	Poly(rC)-binding protein 2 (Fragment) OS=Homo sapiens GN=PCBP2 PE=4 SV=1 - (H3BRU6_HUMAN)
Q9BZW7	Testis-specific gene 10 protein OS=Homo sapiens GN=TSGA10 PE=1 SV=1 - (TSG10_HUMAN)
O14770-5	Isoform 5 of Homeobox protein Meis2 OS=Homo sapiens GN=MEIS2 - (MEIS2_HUMAN)
B5BTY4	ATP-dependent RNA helicase DDX3X OS=Homo sapiens GN=DDX3X PE=2 SV=1 - (B5BTY4_HUMAN)
F8WEV3	PR domain zinc finger protein 16 OS=Homo sapiens GN=PRDM16 PE=4 SV=1 - (F8WEV3_HUMAN)
Q4LDG9-3	Isoform 3 of Dynein light chain 1, axonemal OS=Homo sapiens GN=DNAL1 - (DNAL1_HUMAN)
B2RWP4	TACC2 protein OS=Homo sapiens GN=TACC2 PE=2 SV=1 - (B2RWP4_HUMAN)
B4E2K4	cDNA FLJ54576, highly similar to Aspartyl/asparaginyl beta-hydroxylase (EC 1.14.11.16) OS=Homo sapiens PE=2 SV=1 - (B4E2K4_HUMAN)
H7C346	Protein FAM9C (Fragment) OS=Homo sapiens GN=FAM9C PE=4 SV=1 - (H7C346_HUMAN)
H0YLN8	Transient receptor potential cation channel subfamily M member 7 OS=Homo sapiens GN=TRPM7 PE=4 SV=1 - (H0YLN8_HUMAN)
C9JDG1	Protein diaphanous homolog 3 OS=Homo sapiens GN=DIAPH3 PE=4 SV=1 - (C9JDG1_HUMAN)
Q8TAF3-3	Isoform 3 of WD repeat-containing protein 48 OS=Homo sapiens GN=WDR48 - (WDR48_HUMAN)
F5H816	Phosphorylase OS=Homo sapiens GN=PYGL PE=3 SV=1 - (F5H816_HUMAN)
B3KX35	cDNA FLJ44610 fis, clone BRACE2012317, highly similar to Sodium/potassium-transporting ATPase alpha-2 chain (EC 3.6.3.9) OS=Homo sapiens PE=2 SV=1 - (B3KX35_HUMAN)
Q8IZQ8	Myocardin OS=Homo sapiens GN=MYOCD PE=1 SV=1 - (MYCD_HUMAN)
B7Z6H4	DNA-directed RNA polymerase OS=Homo sapiens PE=2 SV=1 - (B7Z6H4_HUMAN)
Q7Z2Y8	Interferon-induced very large GTPase 1 OS=Homo sapiens GN=GVINP1 PE=2 SV=2 - (GVIN1_HUMAN)
Q9Y5J6	Mitochondrial import inner membrane translocase subunit Tim9 B OS=Homo sapiens GN=FXC1 PE=1 SV=1 - (TIM9B_HUMAN)
Q5T5N4	Uncharacterized protein C6orf118 OS=Homo sapiens GN=C6orf118 PE=2 SV=1 - (CF118_HUMAN)
B4DV51	GTP-binding nuclear protein Ran OS=Homo sapiens GN=RAN PE=2 SV=1 - (B4DV51_HUMAN)
G3V3A5	Tetratricopeptide repeat protein 6 OS=Homo sapiens GN=TTC6 PE=4 SV=1 - (G3V3A5_HUMAN)
P02452	Collagen alpha-1(I) chain OS=Homo sapiens GN=COL1A1 PE=1 SV=5 - (CO1A1_HUMAN)
O94844	Rho-related BTB domain-containing protein 1 OS=Homo sapiens GN=RHOBTB1 PE=1 SV=2 - (RHBT1_HUMAN)
P26599	Polypyrimidine tract-binding protein 1 OS=Homo sapiens GN=PTBP1 PE=1 SV=1 - (PTBP1_HUMAN)
O14979-3	Isoform 3 of Heterogeneous nuclear ribonucleoprotein D-like OS=Homo sapiens GN=HNRPDL - (HNRDL_HUMAN)
Q13620-3	Isoform 3 of Cullin-4B OS=Homo sapiens GN=CUL4B - (CUL4B_HUMAN)
E9PIU1	N-acetylated-alpha-linked acidic dipeptidase-like protein (Fragment) OS=Homo sapiens GN=NAALADL1 PE=4 SV=1 - (E9PIU1_HUMAN)
Q8WU76-2	Isoform 2 of Sec1 family domain-containing protein 2 OS=Homo sapiens GN=SCFD2 - (SCFD2_HUMAN)
O75031	Heat shock factor 2-binding protein OS=Homo sapiens GN=HSF2BP PE=1 SV=1 - (HSF2B_HUMAN)
Q01668-2	Isoform Beta-cell-type of Voltage-dependent L-type calcium channel subunit alpha-1D OS=Homo sapiens GN=CACNA1D - (CAC1D_HUMAN)
B7ZMB1	ZNF214 protein OS=Homo sapiens GN=ZNF214 PE=2 SV=1 - (B7ZMB1_HUMAN)
Q6UWV6	Ectonucleotide pyrophosphatase/phosphodiesterase family member 7 OS=Homo sapiens GN=ENPP7 PE=1 SV=3 - (ENPP7_HUMAN)
Q9BZ22	Solute carrier family 15 (Oligopeptide transporter), member 1 OS=Homo sapiens GN=SLC15A1 PE=2 SV=1 - (Q9BZ22_HUMAN)
Q53G17	NADH dehydrogenase (Ubiquinone) Fe-S protein 8, 23kDa (NADH-coenzyme Q reductase) variant (Fragment) OS=Homo sapiens PE=2 SV=1 - (Q53G17_HUMAN)
D3TTZ3	Tumor necrosis factor alpha-induced protein 3 OS=Homo sapiens GN=TNFAIP3 PE=2 SV=1 - (D3TTZ3_HUMAN)
E0Z3F3	DUX4 (Fragment) OS=Homo sapiens GN=DUX4 PE=3 SV=1 - (E0Z3F3_HUMAN)
C9JZ61	Ankyrin repeat domain-containing protein 53 OS=Homo sapiens GN=ANKRD53 PE=4 SV=1 - (C9JZ61_HUMAN)
O95235	Kinesin-like protein KIF20A OS=Homo sapiens GN=KIF20A PE=1 SV=1 - (KI20A_HUMAN)
A1A5D9	Bicaudal D-related protein 2 OS=Homo sapiens GN=CCDC64B PE=2 SV=2 - (BICR2_HUMAN)
Q9UKJ3	G patch domain-containing protein 8 OS=Homo sapiens GN=GPATCH8 PE=1 SV=2 - (GPTC8_HUMAN)
B5MC21	Interleukin-6 OS=Homo sapiens GN=IL6 PE=4 SV=1 - (B5MC21_HUMAN)
B4DX78	cDNA FLJ55484, highly similar to ATP-dependent RNA helicase DDX39 (EC 3.6.1.-) OS=Homo sapiens PE=2 SV=1 - (B4DX78_HUMAN)
P35030	Trypsin-3 OS=Homo sapiens GN=PRSS3 PE=1 SV=2 - (TRY3_HUMAN)
B7ZAH9	cDNA, FLJ79193, highly similar to Beta-1,4-galactosyltransferase 1 (EC 2.4.1.-) OS=Homo sapiens PE=2 SV=1 - (B7ZAH9_HUMAN)
Q86YV0-2	Isoform 2 of RAS protein activator like-3 OS=Homo sapiens GN=RASAL3 - (RASL3_HUMAN)
Q6ZV71	cDNA FLJ42918 fis, clone BRHIP3027137, highly similar to 10-formyltetrahydrofolate dehydrogenase OS=Homo sapiens PE=2 SV=1 - (Q6ZV71_HUMAN)
B7ZL41	ABCG8 protein OS=Homo sapiens GN=ABCG8 PE=2 SV=1 - (B7ZL41_HUMAN)
I1YDD2	2'-5'-oligoadenylate synthetase-like transcript variant 4 OS=Homo sapiens GN=OASLd PE=2 SV=1 - (I1YDD2_HUMAN)
B6ZGS4	Retinoid-related orphan receptor alpha OS=Homo sapiens GN=NR1F1 PE=2 SV=1 - (B6ZGS4_HUMAN)
P29992	Guanine nucleotide-binding protein subunit alpha-11 OS=Homo sapiens GN=GNA11 PE=1 SV=2 - (GNA11_HUMAN)
Q9Y2I9	TBC1 domain family member 30 OS=Homo sapiens GN=TBC1D30 PE=1 SV=2 - (TBC30_HUMAN)
B4DX24	NADPH:adrenodoxin oxidoreductase, mitochondrial OS=Homo sapiens PE=2 SV=1 - (B4DX24_HUMAN)
Q9H091-2	Isoform 2 of Zinc finger MYND domain-containing protein 15 OS=Homo sapiens GN=ZMYND15 - (ZMY15_HUMAN)
Q9H2K8	Serine/threonine-protein kinase TAO3 OS=Homo sapiens GN=TAOK3 PE=1 SV=2 - (TAOK3_HUMAN)
Q6MZV7	Putative uncharacterized protein DKFZp686C11235 OS=Homo sapiens GN=DKFZp686C11235 PE=1 SV=1 - (Q6MZV7_HUMAN)
B4DT13	Ras-related protein Rab-11A OS=Homo sapiens GN=RAB11A PE=2 SV=1 - (B4DT13_HUMAN)
Q4J6C6	Prolyl endopeptidase-like OS=Homo sapiens GN=PREPL PE=1 SV=1 - (PPCEL_HUMAN)
Q2TAC6-2	Isoform 2 of Kinesin-like protein KIF19 OS=Homo sapiens GN=KIF19 - (KIF19_HUMAN)
Q5SXQ8	Novel protein (Fragment) OS=Homo sapiens GN=RP11-792P23.2 PE=2 SV=1 - (Q5SXQ8_HUMAN)
B4DN33	Transmembrane protein 35 OS=Homo sapiens GN=TMEM35 PE=2 SV=1 - (B4DN33_HUMAN)
Q86XP0	Cytosolic phospholipase A2 delta OS=Homo sapiens GN=PLA2G4D PE=2 SV=2 - (PA24D_HUMAN)
P49368-2	Isoform 2 of T-complex protein 1 subunit gamma OS=Homo sapiens GN=CCT3 - (TCPG_HUMAN)
Q96FJ2	Dynein light chain 2, cytoplasmic OS=Homo sapiens GN=DYNLL2 PE=1 SV=1 - (DYL2_HUMAN)
Q5VSX7	Tripartite motif-containing 62 OS=Homo sapiens GN=TRIM62 PE=2 SV=1 - (Q5VSX7_HUMAN)
B2YKU2	Cytochrome c oxidase subunit 2 (Fragment) OS=Homo sapiens GN=COX2 PE=3 SV=1 - (B2YKU2_HUMAN)
B5BTZ6	Signal transducer and activator of transcription 3 isoform 2 OS=Homo sapiens GN=STAT3 PE=2 SV=1 - (B5BTZ6_HUMAN)
E2GH19	T-cell factor-4 variant I OS=Homo sapiens GN=TCF7L2 PE=2 SV=1 - (E2GH19_HUMAN)
P78509-2	Isoform 2 of Reelin OS=Homo sapiens GN=RELN - (RELN_HUMAN)
Q5SSD1	Synaptotagmin-like 1 OS=Homo sapiens GN=SYTL1 PE=4 SV=1 - (Q5SSD1_HUMAN)
Q15181	Inorganic pyrophosphatase OS=Homo sapiens GN=PPA1 PE=1 SV=2 - (IPYR_HUMAN)
P17706-2	Isoform PTPA of Tyrosine-protein phosphatase non-receptor type 2 OS=Homo sapiens GN=PTPN2 - (PTN2_HUMAN)
C9JJR0	Neurexin-3-beta, soluble form OS=Homo sapiens GN=NRXN3 PE=4 SV=2 - (C9JJR0_HUMAN)
Q96BD5	PHD finger protein 21A OS=Homo sapiens GN=PHF21A PE=1 SV=1 - (PF21A_HUMAN)
A8MWD9	Small nuclear ribonucleoprotein G-like protein OS=Homo sapiens PE=3 SV=2 - (RUXGL_HUMAN)
Q15650	Activating signal cointegrator 1 OS=Homo sapiens GN=TRIP4 PE=1 SV=4 - (TRIP4_HUMAN)
D6RF23	Guanine nucleotide-binding protein subunit beta-2-like 1 OS=Homo sapiens GN=GNB2L1 PE=4 SV=1 - (D6RF23_HUMAN)
Q53GF9	Full-length cDNA 5-PRIME end of clone CS0DF013YM24 of Fetal brain of Homo sapiens (Human) variant (Fragment) OS=Homo sapiens PE=2 SV=1 - (Q53GF9_HUMAN)
Q14330	N-arachidonyl glycine receptor OS=Homo sapiens GN=GPR18 PE=2 SV=2 - (GPR18_HUMAN)
A8K3R3	cDNA FLJ75284, highly similar to Homo sapiens RAB guanine nucleotide exchange factor (GEF) 1 (RABGEF1), mRNA OS=Homo sapiens PE=2 SV=1 - (A8K3R3_HUMAN)
A6NG06	Peroxiredoxin-5, mitochondrial OS=Homo sapiens GN=PRDX5 PE=4 SV=1 - (A6NG06_HUMAN)
Q86V48-2	Isoform 2 of Leucine zipper protein 1 OS=Homo sapiens GN=LUZP1 - (LUZP1_HUMAN)
B4DE53	cDNA FLJ61213, highly similar to Tubulin-specific chaperone D OS=Homo sapiens PE=2 SV=1 - (B4DE53_HUMAN)
Q99935	Proline-rich protein 1 OS=Homo sapiens GN=PROL1 PE=1 SV=2 - (PROL1_HUMAN)
Q5JNZ5	Putative 40S ribosomal protein S26-like 1 OS=Homo sapiens GN=RPS26P11 PE=5 SV=1 - (RS26L_HUMAN)
Q9H3E7	MSTP038 OS=Homo sapiens PE=4 SV=1 - (Q9H3E7_HUMAN)
B7Z815	Ubiquitin carboxyl-terminal hydrolase OS=Homo sapiens GN=USP7 PE=2 SV=1 - (B7Z815_HUMAN)
B4DE15	cDNA FLJ53300, highly similar to UDP-galactose translocator OS=Homo sapiens PE=2 SV=1 - (B4DE15_HUMAN)
Q9BYQ3	Keratin-associated protein 9-3 OS=Homo sapiens GN=KRTAP9-3 PE=1 SV=1 - (KRA93_HUMAN)
D3DSL5	Chromosome 21 open reading frame 2, isoform CRA_a OS=Homo sapiens GN=C21orf2 PE=4 SV=1 - (D3DSL5_HUMAN)
Q05BX4	PSMD1 protein (Fragment) OS=Homo sapiens GN=PSMD1 PE=2 SV=1 - (Q05BX4_HUMAN)
Q59GK9	Ribosomal protein L21 variant (Fragment) OS=Homo sapiens PE=2 SV=1 - (Q59GK9_HUMAN)
D6RIG4	Protocadherin-18 OS=Homo sapiens GN=PCDH18 PE=4 SV=1 - (D6RIG4_HUMAN)
C0IMJ4	Periostin isoform thy8 OS=Homo sapiens PE=2 SV=1 - (C0IMJ4_HUMAN)
H3BQV3	Conserved oligomeric Golgi complex subunit 8 OS=Homo sapiens GN=COG8 PE=4 SV=1 - (H3BQV3_HUMAN)
Q5H9L4	Transcription initiation factor TFIID subunit 7-like OS=Homo sapiens GN=TAF7L PE=2 SV=1 - (TAF7L_HUMAN)
H8WV78	MHC class II antigen (Fragment) OS=Homo sapiens GN=HLA-DRB1 PE=4 SV=1 - (H8WV78_HUMAN)
Q5VWK0	Neuroblastoma breakpoint family member 6 OS=Homo sapiens GN=NBPF6 PE=2 SV=2 - (NBPF6_HUMAN)
I3L398	Protein disulfide-isomerase (Fragment) OS=Homo sapiens GN=P4HB PE=4 SV=1 - (I3L398_HUMAN)
Q5JU85	IQ motif and SEC7 domain-containing protein 2 OS=Homo sapiens GN=IQSEC2 PE=1 SV=1 - (IQEC2_HUMAN)
B4DK14	cDNA FLJ58890, highly similar to GTP-binding protein PTD004 OS=Homo sapiens PE=2 SV=1 - (B4DK14_HUMAN)
Q49A13	ZNF800 protein OS=Homo sapiens GN=ZNF800 PE=2 SV=1 - (Q49A13_HUMAN)
B4DFE6	cDNA FLJ59861, highly similar to ATP synthase gamma chain, mitochondrial (EC 3.6.3.14) OS=Homo sapiens PE=2 SV=1 - (B4DFE6_HUMAN)
Q9Y2G7	Zinc finger protein 30 homolog OS=Homo sapiens GN=ZFP30 PE=2 SV=1 - (ZFP30_HUMAN)
Q96DX7	Tripartite motif-containing protein 44 OS=Homo sapiens GN=TRIM44 PE=1 SV=1 - (TRI44_HUMAN)
A8K0D4	cDNA FLJ78693, highly similar to Homo sapiens TAF6-like RNA polymerase II, p300/CBP-associated factor (PCAF)-associated factor, 65kDa (TAF6L), mRNA OS=Homo sapiens PE=2 SV=1 - (A8K0D4_HUMAN)
Q9UM13	Anaphase-promoting complex subunit 10 OS=Homo sapiens GN=ANAPC10 PE=1 SV=1 - (APC10_HUMAN)
O94761	ATP-dependent DNA helicase Q4 OS=Homo sapiens GN=RECQL4 PE=1 SV=1 - (RECQ4_HUMAN)
Q5VWQ0	Round spermatid basic protein 1 OS=Homo sapiens GN=RSBN1 PE=2 SV=2 - (RSBN1_HUMAN)
B4DLJ5	cDNA FLJ55716, highly similar to Desmocollin-2 OS=Homo sapiens PE=2 SV=1 - (B4DLJ5_HUMAN)
Q6PHZ4	GON4L protein (Fragment) OS=Homo sapiens GN=GON4L PE=2 SV=1 - (Q6PHZ4_HUMAN)
Q8N5P7	MGC50722 protein (Fragment) OS=Homo sapiens GN=MGC50722 PE=2 SV=1 - (Q8N5P7_HUMAN)
Q8NAV1	Pre-mRNA-splicing factor 38A OS=Homo sapiens GN=PRPF38A PE=1 SV=1 - (PR38A_HUMAN)
H0YJA4	Chromodomain-helicase-DNA-binding protein 8 (Fragment) OS=Homo sapiens GN=CHD8 PE=4 SV=1 - (H0YJA4_HUMAN)
Q7RTP6	Protein-methionine sulfoxide oxidase MICAL3 OS=Homo sapiens GN=MICAL3 PE=1 SV=2 - (MICA3_HUMAN)
H7C146	Microtubule-associated serine/threonine-protein kinase 4 (Fragment) OS=Homo sapiens GN=MAST4 PE=4 SV=1 - (H7C146_HUMAN)
Q6IAG3	IL17B protein OS=Homo sapiens GN=IL17B PE=2 SV=1 - (Q6IAG3_HUMAN)
Q05BU7	WASF2 protein (Fragment) OS=Homo sapiens GN=WASF2 PE=2 SV=1 - (Q05BU7_HUMAN)
Q6N069	N-alpha-acetyltransferase 16, NatA auxiliary subunit OS=Homo sapiens GN=NAA16 PE=1 SV=2 - (NAA16_HUMAN)
Q9BY12	S phase cyclin A-associated protein in the endoplasmic reticulum OS=Homo sapiens GN=SCAPER PE=1 SV=1 - (SCAPE_HUMAN)
B4DP80	cDNA FLJ56357, highly similar to Homo sapiens apolipoprotein A-I binding protein (APOA1BP), mRNA OS=Homo sapiens PE=2 SV=1 - (B4DP80_HUMAN)
B4E1B2	cDNA FLJ53691, highly similar to Serotransferrin OS=Homo sapiens PE=2 SV=1 - (B4E1B2_HUMAN)
B4DZC0	cDNA FLJ51771, highly similar to SWI/SNF-related matrix-associatedactin-dependent regulator of chromatin subfamily A member5 (EC 3.6.1.-) OS=Homo sapiens PE=2 SV=1 - (B4DZC0_HUMAN)
F2Z2S8	40S ribosomal protein S3 OS=Homo sapiens GN=RPS3 PE=4 SV=1 - (F2Z2S8_HUMAN)
B9EK40	SPATA21 protein OS=Homo sapiens GN=SPATA21 PE=2 SV=1 - (B9EK40_HUMAN)
O60809	PRAME family member 10 OS=Homo sapiens GN=PRAMEF10 PE=1 SV=3 - (PRA10_HUMAN)
D6RFM0	Ubiquitin-conjugating enzyme E2 D2 (Fragment) OS=Homo sapiens GN=UBE2D2 PE=3 SV=1 - (D6RFM0_HUMAN)
Q9NZD1-2	Isoform 2 of G-protein coupled receptor family C group 5 member D OS=Homo sapiens GN=GPRC5D - (GPC5D_HUMAN)
O75340	Programmed cell death protein 6 OS=Homo sapiens GN=PDCD6 PE=1 SV=1 - (PDCD6_HUMAN)
Q7RTQ0	Kinesin light chain 1N OS=Homo sapiens GN=KNS2 PE=2 SV=1 - (Q7RTQ0_HUMAN)
B4DPP0	CD9 antigen OS=Homo sapiens GN=CD9 PE=2 SV=1 - (B4DPP0_HUMAN)
H0Y8I5	Transmembrane protein 184C (Fragment) OS=Homo sapiens GN=TMEM184C PE=4 SV=1 - (H0Y8I5_HUMAN)
Q68DN7	Putative uncharacterized protein DKFZp781P1017 OS=Homo sapiens GN=DKFZp781P1017 PE=2 SV=1 - (Q68DN7_HUMAN)
C9J326	Probable guanine nucleotide exchange factor MCF2L2 (Fragment) OS=Homo sapiens GN=MCF2L2 PE=4 SV=1 - (C9J326_HUMAN)
P20933	N(4)-(beta-N-acetylglucosaminyl)-L-asparaginase OS=Homo sapiens GN=AGA PE=1 SV=2 - (ASPG_HUMAN)
Q9BTA9-2	Isoform 2 of WW domain-containing adapter protein with coiled-coil OS=Homo sapiens GN=WAC - (WAC_HUMAN)
Q86WV2	COX4I1 protein OS=Homo sapiens GN=COX4I1 PE=2 SV=1 - (Q86WV2_HUMAN)
Q5U5X8	Protein FAM222A OS=Homo sapiens GN=FAM222A PE=2 SV=1 - (F222A_HUMAN)
Q96P20-2	Isoform 1 of NACHT, LRR and PYD domains-containing protein 3 OS=Homo sapiens GN=NLRP3 - (NALP3_HUMAN)
P09669	Cytochrome c oxidase subunit 6C OS=Homo sapiens GN=COX6C PE=1 SV=2 - (COX6C_HUMAN)
Q04671-3	Isoform 3 of P protein OS=Homo sapiens GN=OCA2 - (P_HUMAN)
Q9P0J0	NADH dehydrogenase (ubiquinone) 1 alpha subcomplex subunit 13 OS=Homo sapiens GN=NDUFA13 PE=1 SV=3 - (NDUAD_HUMAN)
Q4VAN1	BTN1A1 protein OS=Homo sapiens GN=BTN1A1 PE=2 SV=1 - (Q4VAN1_HUMAN)
H7C2D7	Claudin-15 (Fragment) OS=Homo sapiens GN=CLDN15 PE=4 SV=1 - (H7C2D7_HUMAN)
O15277	Matrix metalloproteinase RASI-1 OS=Homo sapiens PE=4 SV=1 - (O15277_HUMAN)
C9JUG7	F-actin-capping protein subunit alpha-2 OS=Homo sapiens GN=CAPZA2 PE=4 SV=1 - (C9JUG7_HUMAN)
Q9NQC3	Reticulon-4 OS=Homo sapiens GN=RTN4 PE=1 SV=2 - (RTN4_HUMAN)
Q9ULE0	Protein WWC3 OS=Homo sapiens GN=WWC3 PE=2 SV=3 - (WWC3_HUMAN)
Q9NQ66-2	Isoform B of 1-phosphatidylinositol 4,5-bisphosphate phosphodiesterase beta-1 OS=Homo sapiens GN=PLCB1 - (PLCB1_HUMAN)
Q9NZM4-2	Isoform 2 of Glioma tumor suppressor candidate region gene 1 protein OS=Homo sapiens GN=GLTSCR1 - (GSCR1_HUMAN)
Q71RD3	PP11517 OS=Homo sapiens PE=2 SV=1 - (Q71RD3_HUMAN)
Q96EG3	Zinc finger protein 837 OS=Homo sapiens GN=ZNF837 PE=2 SV=2 - (ZN837_HUMAN)
H7C5G3	F-box only protein 9 (Fragment) OS=Homo sapiens GN=FBXO9 PE=4 SV=1 - (H7C5G3_HUMAN)
O43313	ATM interactor OS=Homo sapiens GN=ATMIN PE=1 SV=2 - (ATMIN_HUMAN)
E5G0Z0	MHC class I antigen (Fragment) OS=Homo sapiens GN=HLA-Cw PE=3 SV=1 - (E5G0Z0_HUMAN)
Q9NXW9	Probable alpha-ketoglutarate-dependent dioxygenase ABH4 OS=Homo sapiens GN=ALKBH4 PE=1 SV=1 - (ALKB4_HUMAN)
Q9BTF0	THUMP domain-containing protein 2 OS=Homo sapiens GN=THUMPD2 PE=2 SV=2 - (THUM2_HUMAN)
Q6ZRK0	Uncharacterized protein OS=Homo sapiens PE=2 SV=1 - (Q6ZRK0_HUMAN)
B4DVX8	cDNA FLJ53566 OS=Homo sapiens PE=2 SV=1 - (B4DVX8_HUMAN)
G3V3E8	Epididymal secretory protein E1 OS=Homo sapiens GN=NPC2 PE=4 SV=1 - (G3V3E8_HUMAN)
E9PNX2	Neuronal acetylcholine receptor subunit alpha-10 OS=Homo sapiens GN=CHRNA10 PE=4 SV=1 - (E9PNX2_HUMAN)
Q5TA51	Cleavage and polyadenylation specific factor 3-like (Fragment) OS=Homo sapiens GN=CPSF3L PE=2 SV=1 - (Q5TA51_HUMAN)
Q8IUC2	Keratin-associated protein 8-1 OS=Homo sapiens GN=KRTAP8-1 PE=2 SV=1 - (KRA81_HUMAN)
Q6UW63	KDEL motif-containing protein 1 OS=Homo sapiens GN=KDELC1 PE=1 SV=1 - (KDEL1_HUMAN)
Q6ZP98	Putative uncharacterized protein C16orf47 OS=Homo sapiens GN=C16orf47 PE=2 SV=1 - (CP047_HUMAN)
B4DT92	Acetylcholine receptor subunit delta OS=Homo sapiens GN=CHRND PE=2 SV=1 - (B4DT92_HUMAN)
F6KCZ3	Phosphatase and tensin-like protein OS=Homo sapiens PE=4 SV=1 - (F6KCZ3_HUMAN)
B4DUR3	cDNA FLJ55386, highly similar to Homo sapiens methyl-CpG binding domain protein 1 (MBD1), transcript variant 4, mRNA OS=Homo sapiens PE=2 SV=1 - (B4DUR3_HUMAN)
A8K0S5	cDNA FLJ77003, highly similar to Homo sapiens cerebellin 4 (CBLN4), mRNA OS=Homo sapiens PE=2 SV=1 - (A8K0S5_HUMAN)
A8MT70	Zinc finger B-box domain-containing protein 1 OS=Homo sapiens GN=ZBBX PE=2 SV=3 - (ZBBX_HUMAN)
Q8TB52	F-box only protein 30 OS=Homo sapiens GN=FBXO30 PE=1 SV=3 - (FBX30_HUMAN)
Q8NEP3-3	Isoform 3 of Dynein assembly factor 1, axonemal OS=Homo sapiens GN=DNAAF1 - (DAAF1_HUMAN)
Q6ZW33	MICAL C-terminal-like protein OS=Homo sapiens GN=MICALCL PE=2 SV=3 - (MICLK_HUMAN)
Q9XRX5	HERV-H LTR-associating protein 3 OS=Homo sapiens GN=HHLA3 PE=1 SV=3 - (HHLA3_HUMAN)
O95429-2	Isoform 2 of BAG family molecular chaperone regulator 4 OS=Homo sapiens GN=BAG4 - (BAG4_HUMAN)
Q9BRK5-4	Isoform 4 of 45 kDa calcium-binding protein OS=Homo sapiens GN=SDF4 - (CAB45_HUMAN)
Q6SJ93	Protein FAM111B OS=Homo sapiens GN=FAM111B PE=2 SV=1 - (F111B_HUMAN)
Q9Y5L2	Hypoxia-inducible lipid droplet-associated protein OS=Homo sapiens GN=HILPDA PE=1 SV=1 - (HLPDA_HUMAN)
P48735	Isocitrate dehydrogenase (NADP), mitochondrial OS=Homo sapiens GN=IDH2 PE=1 SV=2 - (IDHP_HUMAN)
P78414	Iroquois-class homeodomain protein IRX-1 OS=Homo sapiens GN=IRX1 PE=1 SV=3 - (IRX1_HUMAN)
P01597	Ig kappa chain V-I region DEE OS=Homo sapiens PE=1 SV=1 - (KV105_HUMAN)
Q96G30	Melanocortin-2 receptor accessory protein 2 OS=Homo sapiens GN=MRAP2 PE=2 SV=2 - (MRAP2_HUMAN)
O76036-5	Isoform 5 of Natural cytotoxicity triggering receptor 1 OS=Homo sapiens GN=NCR1 - (NCTR1_HUMAN)
Q15858-3	Isoform 3 of Sodium channel protein type 9 subunit alpha OS=Homo sapiens GN=SCN9A - (SCN9A_HUMAN)
Q9GZM7-3	Isoform 3 of Tubulointerstitial nephritis antigen-like OS=Homo sapiens GN=TINAGL1 - (TINAL_HUMAN)
Q8NC26	Zinc finger protein 114 OS=Homo sapiens GN=ZNF114 PE=2 SV=1 - (ZN114_HUMAN)
F8W1E4	Protein G8 OS=Homo sapiens GN=C6orf48 PE=4 SV=1 - (F8W1E4_HUMAN)
B7Z7G5	Probable tRNA pseudouridine synthase 2 OS=Homo sapiens GN=TRUB2 PE=2 SV=1 - (B7Z7G5_HUMAN)
B7Z7N4	Poly(U)-specific endoribonuclease OS=Homo sapiens GN=ENDOU PE=2 SV=1 - (B7Z7N4_HUMAN)
A1BQX2	MARVEL domain-containing protein 2 OS=Homo sapiens GN=MARVELD2 PE=2 SV=1 - (A1BQX2_HUMAN)
E9PAT1	Protein phosphatase 1 regulatory subunit 14D OS=Homo sapiens GN=PPP1R14D PE=4 SV=1 - (E9PAT1_HUMAN)
Q5GRG0	ESC42-RELB OS=Homo sapiens PE=2 SV=1 - (Q5GRG0_HUMAN)
B5MDV0	Protein monoglycylase TTLL8 OS=Homo sapiens GN=TTLL8 PE=4 SV=2 - (B5MDV0_HUMAN)
I3L3D7	Serine protease 33 (Fragment) OS=Homo sapiens GN=PRSS33 PE=4 SV=1 - (I3L3D7_HUMAN)
I3L3X7	Proteasome subunit beta type-6 (Fragment) OS=Homo sapiens GN=PSMB6 PE=4 SV=1 - (I3L3X7_HUMAN)
E9PQT9	Disks large homolog 2 (Fragment) OS=Homo sapiens GN=DLG2 PE=4 SV=1 - (E9PQT9_HUMAN)
Q8N730	Peripheral-type Benzodiazepine Receptor (Fragment) OS=Homo sapiens GN=PBR PE=2 SV=1 - (Q8N730_HUMAN)
B2RD27	cDNA, FLJ96428, highly similar to Homo sapiens proteasome (prosome, macropain) 26S subunit, non-ATPase, 7 (Mov34 homolog) (PSMD7), mRNA OS=Homo sapiens PE=2 SV=1 - (B2RD27_HUMAN)
D9MXF4	Fanconi anemia associated nuclease 1 OS=Homo sapiens GN=FAN1 PE=2 SV=1 - (D9MXF4_HUMAN)
E9PL38	26S proteasome non-ATPase regulatory subunit 13 (Fragment) OS=Homo sapiens GN=PSMD13 PE=4 SV=1 - (E9PL38_HUMAN)
Q8NF08	FLJ00396 protein (Fragment) OS=Homo sapiens GN=FLJ00396 PE=2 SV=1 - (Q8NF08_HUMAN)
F5H402	Aryl hydrocarbon receptor nuclear translocator-like protein 2 OS=Homo sapiens GN=ARNTL2 PE=4 SV=1 - (F5H402_HUMAN)
H0Y6B5	Abl interactor 2 (Fragment) OS=Homo sapiens GN=ABI2 PE=4 SV=1 - (H0Y6B5_HUMAN)
A8K8K6	cDNA FLJ76430, highly similar to Homo sapiens zinc finger protein 165 (ZNF165), mRNA OS=Homo sapiens PE=2 SV=1 - (A8K8K6_HUMAN)
B7Z210	cDNA FLJ50128, highly similar to Homo sapiens acyl-CoA synthetase bubblegum family member 1 (ACSBG1), mRNA OS=Homo sapiens PE=2 SV=1 - (B7Z210_HUMAN)
E9PEN5	Intermediate filament family orphan 2 OS=Homo sapiens GN=IFFO2 PE=4 SV=1 - (E9PEN5_HUMAN)
E9PIE0	Connector enhancer of kinase suppressor of ras 1 OS=Homo sapiens GN=CNKSR1 PE=4 SV=1 - (E9PIE0_HUMAN)
C9JQH3	Tripartite motif-containing protein 73 OS=Homo sapiens GN=TRIM73 PE=4 SV=1 - (C9JQH3_HUMAN)
Q86W19	Protease serine 1 (Fragment) OS=Homo sapiens GN=PRSS1 PE=2 SV=1 - (Q86W19_HUMAN)
F6XQ08	Lymphocyte antigen 6 complex locus protein G6d (Fragment) OS=Homo sapiens GN=LY6G6D PE=4 SV=1 - (F6XQ08_HUMAN)

Appendix B

**Table 4:** 190 common proteins identified in all five subjects and comparisons with earlier reports.

Intersection of all five samples - 190 proteins and comparisons with earlier reports				
Accession	Protein description	Lee et al. (2006) [9]	Rice (2011) [10]	Nasir et al. (2020) [30]
O43790	Keratin, type II cuticular Hb6 OS=Homo sapiens GN=KRT86 PE=1 SV=1 - (KRT86_HUMAN)	+	+	+
F8W6V8	Keratin, type II cuticular Hb1 OS=Homo sapiens GN=KRT81 PE=3 SV=1 - (F8W6V8_HUMAN)	−	−	
P78386	Keratin, type II cuticular Hb5 OS=Homo sapiens GN=KRT85 PE=1 SV=1 - (KRT85_HUMAN)	+	+	+
P78385	Keratin, type II cuticular Hb3 OS=Homo sapiens GN=KRT83 PE=1 SV=2 - (KRT83_HUMAN)	−	−	+
Q15323	Keratin, type I cuticular Ha1 OS=Homo sapiens GN=KRT31 PE=2 SV=3 - (K1H1_HUMAN)	−	+	
O76009	Keratin, type I cuticular Ha3-I OS=Homo sapiens GN=KRT33A PE=2 SV=2 - (KT33A_HUMAN)	−	−	
Q14525	Keratin, type I cuticular Ha3-II OS=Homo sapiens GN=KRT33B PE=2 SV=3 - (KT33B_HUMAN)	+	+	+
H6VRF8	Keratin 1 OS=Homo sapiens GN=KRT1 PE=3 SV=1 - (H6VRF8_HUMAN)	−	−	
P13647	Keratin, type II cytoskeletal 5 OS=Homo sapiens GN=KRT5 PE=1 SV=3 - (K2C5_HUMAN)	−	−	
P35908	Keratin, type II cytoskeletal 2 epidermal OS=Homo sapiens GN=KRT2 PE=1 SV=2 - (K22E_HUMAN)	−	−	
P04259	Keratin, type II cytoskeletal 6B OS=Homo sapiens GN=KRT6B PE=1 SV=5 - (K2C6B_HUMAN)	−	−	
B4DRR0	cDNA FLJ53910, highly similar to Keratin, type II cytoskeletal 6A OS=Homo sapiens PE=2 SV=1 - (B4DRR0_HUMAN)	−	−	
P13645	Keratin, type I cytoskeletal 10 OS=Homo sapiens GN=KRT10 PE=1 SV=6 - (K1C10_HUMAN)	−	−	
B4DKV4	cDNA FLJ60647, highly similar to Keratin, type II cytoskeletal 6B OS=Homo sapiens PE=2 SV=1 - (B4DKV4_HUMAN)	−	−	
P02533	Keratin, type I cytoskeletal 14 OS=Homo sapiens GN=KRT14 PE=1 SV=4 - (K1C14_HUMAN)	−	−	
Q92764	Keratin, type I cuticular Ha5 OS=Homo sapiens GN=KRT35 PE=2 SV=5 - (KRT35_HUMAN)	+	−	
P35527	Keratin, type I cytoskeletal 9 OS=Homo sapiens GN=KRT9 PE=1 SV=3 - (K1C9_HUMAN)	−	−	
O95678	Keratin, type II cytoskeletal 75 OS=Homo sapiens GN=KRT75 PE=1 SV=2 - (K2C75_HUMAN)	−	−	
P08779	Keratin, type I cytoskeletal 16 OS=Homo sapiens GN=KRT16 PE=1 SV=4 - (K1C16_HUMAN)	−	−	
Q04695	Keratin, type I cytoskeletal 17 OS=Homo sapiens GN=KRT17 PE=1 SV=2 - (K1C17_HUMAN)	−	−	
Q14532	Keratin, type I cuticular Ha2 OS=Homo sapiens GN=KRT32 PE=1 SV=3 - (K1H2_HUMAN)	+	−	
Q9NSB2	Keratin, type II cuticular Hb4 OS=Homo sapiens GN=KRT84 PE=1 SV=2 - (KRT84_HUMAN)	−	−	
P15924	Desmoplakin OS=Homo sapiens GN=DSP PE=1 SV=3 - (DESP_HUMAN)	+	+	
F5GZD1	Keratin, type II cytoskeletal 7 OS=Homo sapiens GN=KRT7 PE=4 SV=1 - (F5GZD1_HUMAN)	−	−	
B3KVF5	cDNA FLJ16494 fis, clone CTONG3004576, highly similar to Keratin, type I cytoskeletal 15 OS=Homo sapiens PE=2 SV=1 - (B3KVF5_HUMAN)	−	−	
Q3SY84	Keratin, type II cytoskeletal 71 OS=Homo sapiens GN=KRT71 PE=1 SV=3 - (K2C71_HUMAN)	−	−	
Q86Y46	Keratin, type II cytoskeletal 73 OS=Homo sapiens GN=KRT73 PE=1 SV=1 - (K2C73_HUMAN)	−	−	
Q7RTS7	Keratin, type II cytoskeletal 74 OS=Homo sapiens GN=KRT74 PE=1 SV=2 - (K2C74_HUMAN)	−	−	
Q07283	Trichohyalin OS=Homo sapiens GN=TCHH PE=1 SV=2 - (TRHY_HUMAN)	−	−	
Q7Z3Y7	Keratin, type I cytoskeletal 28 OS=Homo sapiens GN=KRT28 PE=1 SV=2 - (K1C28_HUMAN)	−	−	
B4DRW1	cDNA FLJ55805, highly similar to Keratin, type II cytoskeletal 4 OS=Homo sapiens PE=2 SV=1 - (B4DRW1_HUMAN)	−	−	
Q7Z3Y8	Keratin, type I cytoskeletal 27 OS=Homo sapiens GN=KRT27 PE=1 SV=2 - (K1C27_HUMAN)	−	−	
Q7Z3Z0	Keratin, type I cytoskeletal 25 OS=Homo sapiens GN=KRT25 PE=1 SV=1 - (K1C25_HUMAN)	−	−	
Q8WVW5	Putative uncharacterized protein (Fragment) OS=Homo sapiens PE=2 SV=1 - (Q8WVW5_HUMAN)	−	−	
P14923	Junction plakoglobin OS=Homo sapiens GN=JUP PE=1 SV=3 - (PLAK_HUMAN)	+	+	
P62805	Histone H4 OS=Homo sapiens GN=HIST1H4A PE=1 SV=2 - (H4_HUMAN)	−	−	
Q2M2I5	Keratin, type I cytoskeletal 24 OS=Homo sapiens GN=KRT24 PE=1 SV=1 - (K1C24_HUMAN)	−	−	
Q8N1N4	Keratin, type II cytoskeletal 78 OS=Homo sapiens GN=KRT78 PE=2 SV=2 - (K2C78_HUMAN)	−	−	
Q9BYR8	Keratin-associated protein 3-1 OS=Homo sapiens GN=KRTAP3-1 PE=1 SV=1 - (KRA31_HUMAN)	−	−	
Q93077	Histone H2A type 1-C OS=Homo sapiens GN=HIST1H2AC PE=1 SV=3 - (H2A1C_HUMAN)	+	−	
Q8TC04	Keratin 23 (Histone deacetylase inducible) OS=Homo sapiens GN=KRT23 PE=2 SV=1 - (Q8TC04_HUMAN)	−	−	
P04792	Heat shock protein beta-1 OS=Homo sapiens GN=HSPB1 PE=1 SV=2 - (HSPB1_HUMAN)	−	−	
P31947	14-3-3 protein sigma OS=Homo sapiens GN=SFN PE=1 SV=1 - (1433S_HUMAN)	−	+	
A8MUB1	Tubulin alpha-4A chain OS=Homo sapiens GN=TUBA4A PE=2 SV=1 - (A8MUB1_HUMAN)	−	−	
P47929	Galectin-7 OS=Homo sapiens GN=LGALS7 PE=1 SV=2 - (LEG7_HUMAN)	−	−	
O43707	Alpha-actinin-4 OS=Homo sapiens GN=ACTN4 PE=1 SV=2 - (ACTN4_HUMAN)	−	−	
F8VW92	Tubulin beta chain OS=Homo sapiens GN=TUBB PE=3 SV=1 - (F8VW92_HUMAN)	+	−	
A0N4V7	HCG2039797 (Fragment) OS=Homo sapiens GN=Tcr-alpha PE=4 SV=1 - (A0N4V7_HUMAN)	−	−	
Q562R1	Beta-actin-like protein 2 OS=Homo sapiens GN=ACTBL2 PE=1 SV=2 - (ACTBL_HUMAN)	−	+	
Q6A163	Keratin, type I cytoskeletal 39 OS=Homo sapiens GN=KRT39 PE=1 SV=2 - (K1C39_HUMAN)	−	+	
A8MTN3	Putative keratin-associated protein ENSP00000381495 OS=Homo sapiens PE=3 SV=2 - (KRA2X_HUMAN)	−	−	
Q13835-2	Isoform 1 of Plakophilin-1 OS=Homo sapiens GN=PKP1 - (PKP1_HUMAN)	−	+	
Q86UQ4	ATP-binding cassette sub-family A member 13 OS=Homo sapiens GN=ABCA13 PE=2 SV=3 - (ABCAD_HUMAN)	−	−	
P35579	Myosin-9 OS=Homo sapiens GN=MYH9 PE=1 SV=4 - (MYH9_HUMAN)	−	−	
Q6PK75	PRSS2 protein (Fragment) OS=Homo sapiens GN=PRSS2 PE=2 SV=1 - (Q6PK75_HUMAN)	−	−	
Q9BYR6	Keratin-associated protein 3-3 OS=Homo sapiens GN=KRTAP3-3 PE=1 SV=1 - (KRA33_HUMAN)	−	−	
Q01469	Fatty acid-binding protein, epidermal OS=Homo sapiens GN=FABP5 PE=1 SV=3 - (FABP5_HUMAN)	−	−	
Q02413	Desmoglein-1 OS=Homo sapiens GN=DSG1 PE=1 SV=2 - (DSG1_HUMAN)	−	+	
Q2VIP3	PABP3 (Fragment) OS=Homo sapiens PE=2 SV=1 - (Q2VIP3_HUMAN)	−	−	
P27348	14-3-3 protein theta OS=Homo sapiens GN=YWHAQ PE=1 SV=1 - (1433T_HUMAN)	−	−	
Q9BYR7	Keratin-associated protein 3-2 OS=Homo sapiens GN=KRTAP3-2 PE=1 SV=1 - (KRA32_HUMAN)	−	−	
B2RAW1	cDNA, FLJ95155, highly similar to Homo sapiens Bruton agammaglobulinemia tyrosine kinase (BTK), mRNA OS=Homo sapiens PE=2 SV=1 - (B2RAW1_HUMAN)	−	−	
F8W8Y7	Armadillo repeat-containing X-linked protein 4 OS=Homo sapiens GN=ARMCX4 PE=4 SV=1 - (F8W8Y7_HUMAN)	−	−	
P63104	14-3-3 protein zeta/delta OS=Homo sapiens GN=YWHAZ PE=1 SV=1 - (1433Z_HUMAN)	−	−	
P81605	Dermcidin OS=Homo sapiens GN=DCD PE=1 SV=2 - (DCD_HUMAN)	−	−	
P61981	14-3-3 protein gamma OS=Homo sapiens GN=YWHAG PE=1 SV=2 - (1433G_HUMAN)	−	−	
Q86TY5	Full-length cDNA clone CS0DI041YE05 of Placenta of Homo sapiens (human) OS=Homo sapiens PE=2 SV=1 - (Q86TY5_HUMAN)	−	−	
P09382	Galectin-1 OS=Homo sapiens GN=LGALS1 PE=1 SV=2 - (LEG1_HUMAN)	−	−	
Q09666	Neuroblast differentiation-associated protein AHNAK OS=Homo sapiens GN=AHNAK PE=1 SV=2 - (AHNK_HUMAN)	−	−	
Q8NGB0	Seven transmembrane helix receptor OS=Homo sapiens GN=GPR142 PE=2 SV=1 - (Q8NGB0_HUMAN)	−	−	
P54652	Heat shock-related 70 kDa protein 2 OS=Homo sapiens GN=HSPA2 PE=1 SV=1 - (HSP72_HUMAN)	+	−	
B4DHC2	cDNA FLJ56434, highly similar to p130Cas-associated protein OS=Homo sapiens PE=2 SV=1 - (B4DHC2_HUMAN)	−	−	
A0PK11	Clarin-2 OS=Homo sapiens GN=CLRN2 PE=2 SV=1 - (CLRN2_HUMAN)	−	−	
Q0Z7S8	Fatty acid-binding protein 9 OS=Homo sapiens GN=FABP9 PE=1 SV=1 - (FABP9_HUMAN)	−	−	
P33764	Protein S100-A3 OS=Homo sapiens GN=S100A3 PE=1 SV=1 - (S10A3_HUMAN)	+	−	
Q7Z406	Myosin-14 OS=Homo sapiens GN=MYH14 PE=1 SV=2 - (MYH14_HUMAN)	−	−	
O95147	Dual specificity protein phosphatase 14 OS=Homo sapiens GN=DUSP14 PE=1 SV=1 - (DUS14_HUMAN)	−	−	
Q6ISF6	KRTAP9-2 protein (Fragment) OS=Homo sapiens GN=KRTAP9-2 PE=2 SV=1 - (Q6ISF6_HUMAN)	−	−	
P07737	Profilin-1 OS=Homo sapiens GN=PFN1 PE=1 SV=2 - (PROF1_HUMAN)	−	−	
P27482	Calmodulin-like protein 3 OS=Homo sapiens GN=CALML3 PE=1 SV=2 - (CALL3_HUMAN)	−	−	
B4DF70	cDNA FLJ60461, highly similar to Peroxiredoxin-2 (EC 1.11.1.15) OS=Homo sapiens PE=2 SV=1 - (B4DF70_HUMAN)	−	−	
Q86SJ6	Desmoglein-4 OS=Homo sapiens GN=DSG4 PE=1 SV=1 - (DSG4_HUMAN)	+	+	
B3KVD6	cDNA FLJ16433 fis, clone BRACE3013936, highly similar to Cell death activator CIDE-B OS=Homo sapiens PE=2 SV=1 - (B3KVD6_HUMAN)	−	−	
Q13228	Selenium-binding protein 1 OS=Homo sapiens GN=SELENBP1 PE=1 SV=2 - (SBP1_HUMAN)	+	+	
P62258	14-3-3 protein epsilon OS=Homo sapiens GN=YWHAE PE=1 SV=1 - (1433E_HUMAN)	−	−	
O94819	Kelch repeat and BTB domain-containing protein 11 OS=Homo sapiens GN=KBTBD11 PE=1 SV=1 - (KBTBB_HUMAN)	−	−	
B0AZQ4	cDNA, FLJ79494, highly similar to Structural maintenance of chromosome 3 OS=Homo sapiens PE=2 SV=1 - (B0AZQ4_HUMAN)	−	−	
Q15828	Cystatin-M OS=Homo sapiens GN=CST6 PE=1 SV=1 - (CYTM_HUMAN)	−	−	
B4DVQ3	Calcium/calmodulin-dependent protein kinase type II subunit gamma OS=Homo sapiens GN=CAMK2G PE=2 SV=1 - (B4DVQ3_HUMAN)	−	−	
O15173	Membrane-associated progesterone receptor component 2 OS=Homo sapiens GN=PGRMC2 PE=1 SV=1 - (PGRC2_HUMAN)	−	−	
P58107	Epiplakin OS=Homo sapiens GN=EPPK1 PE=1 SV=2 - (EPIPL_HUMAN)	−	−	
P13639	Elongation factor 2 OS=Homo sapiens GN=EEF2 PE=1 SV=4 - (EF2_HUMAN)	−	−	
P37802	Transgelin-2 OS=Homo sapiens GN=TAGLN2 PE=1 SV=3 - (TAGL2_HUMAN)	−	−	
P31949	Protein S100-A11 OS=Homo sapiens GN=S100A11 PE=1 SV=2 - (S10AB_HUMAN)	−	−	
P55072	Transitional endoplasmic reticulum ATPase OS=Homo sapiens GN=VCP PE=1 SV=4 - (TERA_HUMAN)	−	−	
B7Z7A9	Phosphoglycerate kinase OS=Homo sapiens GN=PGK1 PE=2 SV=1 - (B7Z7A9_HUMAN)	−	−	
Q9Y6R9	Coiled-coil domain-containing protein 61 OS=Homo sapiens GN=CCDC61 PE=1 SV=2 - (CCD61_HUMAN)	−	−	
P06733	Alpha-enolase OS=Homo sapiens GN=ENO1 PE=1 SV=2 - (ENOA_HUMAN)	+	−	
P36952	Serpin B5 OS=Homo sapiens GN=SERPINB5 PE=1 SV=2 - (SPB5_HUMAN)	−	−	
E5KNB2	DNA-apurinic or apyrimidinic site lyase 2 OS=Homo sapiens PE=4 SV=1 - (E5KNB2_HUMAN)	−	−	
Q6ZQQ6	WD repeat-containing protein 87 OS=Homo sapiens GN=WDR87 PE=2 SV=3 - (WDR87_HUMAN)	−	−	
P15090	Fatty acid-binding protein, adipocyte OS=Homo sapiens GN=FABP4 PE=1 SV=3 - (FABP4_HUMAN)	−	−	
B3KWI4	cDNA FLJ43122 fis, clone CTONG3003737, highly similar to Leucine-rich repeat-containing protein 15 OS=Homo sapiens PE=2 SV=1 - (B3KWI4_HUMAN)	+	−	
Q9H9L3	Interferon-stimulated 20 kDa exonuclease-like 2 OS=Homo sapiens GN=ISG20L2 PE=1 SV=1 - (I20L2_HUMAN)	−	−	
Q5T749	Keratinocyte proline-rich protein OS=Homo sapiens GN=KPRP PE=1 SV=1 - (KPRP_HUMAN)	−	+	
Q562Q2	Actin-like protein (Fragment) OS=Homo sapiens GN=ACT PE=3 SV=1 - (Q562Q2_HUMAN)	−	−	
Q5D862	Filaggrin-2 OS=Homo sapiens GN=FLG2 PE=1 SV=1 - (FILA2_HUMAN)	−	−	
E9PR44	Alpha-crystallin B chain (Fragment) OS=Homo sapiens GN=CRYAB PE=3 SV=1 - (E9PR44_HUMAN)	−	−	
P62249	40S ribosomal protein S16 OS=Homo sapiens GN=RPS16 PE=1 SV=2 - (RS16_HUMAN)	−	−	
P09211	Glutathione S-transferase P OS=Homo sapiens GN=GSTP1 PE=1 SV=2 - (GSTP1_HUMAN)	+	−	
P22392-2	Isoform 3 of Nucleoside diphosphate kinase B OS=Homo sapiens GN=NME2 - (NDKB_HUMAN)	−	−	
O60437	Periplakin OS=Homo sapiens GN=PPL PE=1 SV=4 - (PEPL_HUMAN)	−	−	
P05109	Protein S100-A8 OS=Homo sapiens GN=S100A8 PE=1 SV=1 - (S10A8_HUMAN)	−	−	
Q00610-2	Isoform 2 of Clathrin heavy chain 1 OS=Homo sapiens GN=CLTC - (CLH1_HUMAN)	+	−	
B4DLJ7	cDNA FLJ59334, highly similar to Transmembrane glycoprotein NMB OS=Homo sapiens PE=2 SV=1 - (B4DLJ7_HUMAN)	−	−	
P45880-2	Isoform 2 of Voltage-dependent anion-selective channel protein 2 OS=Homo sapiens GN=VDAC2 - (VDAC2_HUMAN)	−	−	
P62269	40S ribosomal protein S18 OS=Homo sapiens GN=RPS18 PE=1 SV=3 - (RS18_HUMAN)	−	−	
Q9Y446	Plakophilin-3 OS=Homo sapiens GN=PKP3 PE=1 SV=1 - (PKP3_HUMAN)	+	−	
P04075	Fructose-bisphosphate aldolase A OS=Homo sapiens GN=ALDOA PE=1 SV=2 - (ALDOA_HUMAN)	−	−	
P11021	78 kDa glucose-regulated protein OS=Homo sapiens GN=HSPA5 PE=1 SV=2 - (GRP78_HUMAN)	+	+	
P62851	40S ribosomal protein S25 OS=Homo sapiens GN=RPS25 PE=1 SV=1 - (RS25_HUMAN)	−	−	
P62277	40S ribosomal protein S13 OS=Homo sapiens GN=RPS13 PE=1 SV=2 - (RS13_HUMAN)	−	−	
E9PBV3	Suprabasin OS=Homo sapiens GN=SBSN PE=4 SV=1 - (E9PBV3_HUMAN)	−	−	
P68402	Platelet-activating factor acetylhydrolase IB subunit beta OS=Homo sapiens GN=PAFAH1B2 PE=1 SV=1 - (PA1B2_HUMAN)	−	−	
P28838-2	Isoform 2 of Cytosol aminopeptidase OS=Homo sapiens GN=LAP3 - (AMPL_HUMAN)	+	−	
Q5VU13	V-set and immunoglobulin domain-containing protein 8 OS=Homo sapiens GN=VSIG8 PE=1 SV=1 - (VSIG8_HUMAN)	−	−	
P07476	Involucrin OS=Homo sapiens GN=IVL PE=1 SV=2 - (INVO_HUMAN)	−	−	
Q15365	Poly(rC)-binding protein 1 OS=Homo sapiens GN=PCBP1 PE=1 SV=2 - (PCBP1_HUMAN)	−	−	
E9PI98	Layilin (Fragment) OS=Homo sapiens GN=LAYN PE=4 SV=1 - (E9PI98_HUMAN)	−	−	
P23284	Peptidyl-prolyl cis-trans isomerase B OS=Homo sapiens GN=PPIB PE=1 SV=2 - (PPIB_HUMAN)	−	−	
B2RBR9	cDNA, FLJ95650, highly similar to Homo sapiens karyopherin (importin) beta 1 (KPNB1), mRNA OS=Homo sapiens PE=2 SV=1 - (B2RBR9_HUMAN)	−	−	
Q9BTQ7	Similar to ribosomal protein L23 (Fragment) OS=Homo sapiens PE=1 SV=1 - (Q9BTQ7_HUMAN)	−	−	
Q96IR1	RPS4X protein (Fragment) OS=Homo sapiens GN=RPS4X PE=2 SV=2 - (Q96IR1_HUMAN)	−	−	
Q86VG2	Splicing factor proline/glutamine-rich (Polypyrimidine tract binding protein associated) OS=Homo sapiens GN=SFPQ PE=2 SV=1 - (Q86VG2_HUMAN)	−	−	
B2R4M6	cDNA, FLJ92148, highly similar to Homo sapiens S100 calcium binding protein A9 (calgranulin B) (S100A9), mRNA OS=Homo sapiens PE=4 SV=1 - (B2R4M6_HUMAN)	−	−	
P04083	Annexin A1 OS=Homo sapiens GN=ANXA1 PE=1 SV=2 - (ANXA1_HUMAN)	−	−	
Q5VYY1	Ankyrin repeat domain-containing protein 22 OS=Homo sapiens GN=ANKRD22 PE=2 SV=1 - (ANR22_HUMAN)	−	−	
P62857	40S ribosomal protein S28 OS=Homo sapiens GN=RPS28 PE=1 SV=1 - (RS28_HUMAN)	−	−	
A8MXN1	Receptor expression-enhancing protein 6 OS=Homo sapiens GN=REEP6 PE=4 SV=1 - (A8MXN1_HUMAN)	−	−	
Q96QA5	Gasdermin-A OS=Homo sapiens GN=GSDMA PE=2 SV=4 - (GSDMA_HUMAN)	−	−	
Q86SG7	Lysozyme g-like protein 2 OS=Homo sapiens GN=LYG2 PE=1 SV=2 - (LYG2_HUMAN)	+	−	
P60866	40S ribosomal protein S20 OS=Homo sapiens GN=RPS20 PE=1 SV=1 - (RS20_HUMAN)	−	−	
P04080	Cystatin-B OS=Homo sapiens GN=CSTB PE=1 SV=2 - (CYTB_HUMAN)	−	−	
Q9HCY8	Protein S100-A14 OS=Homo sapiens GN=S100A14 PE=1 SV=1 - (S10AE_HUMAN)	−	−	
P22314	Ubiquitin-like modifier-activating enzyme 1 OS=Homo sapiens GN=UBA1 PE=1 SV=3 - (UBA1_HUMAN)	−	−	
Q96FQ6	Protein S100-A16 OS=Homo sapiens GN=S100A16 PE=1 SV=1 - (S10AG_HUMAN)	−	−	
P06703	Protein S100-A6 OS=Homo sapiens GN=S100A6 PE=1 SV=1 - (S10A6_HUMAN)	−	−	
P31151	Protein S100-A7 OS=Homo sapiens GN=S100A7 PE=1 SV=4 - (S10A7_HUMAN)	−	−	
P25787	Proteasome subunit alpha type-2 OS=Homo sapiens GN=PSMA2 PE=1 SV=2 - (PSA2_HUMAN)	−	−	
Q04760-2	Isoform 2 of Lactoylglutathione lyase OS=Homo sapiens GN=GLO1 - (LGUL_HUMAN)	−	−	
P61254	60S ribosomal protein L26 OS=Homo sapiens GN=RPL26 PE=1 SV=1 - (RL26_HUMAN)	−	−	
P46783	40S ribosomal protein S10 OS=Homo sapiens GN=RPS10 PE=1 SV=1 - (RS10_HUMAN)	−	−	
P32926	Desmoglein-3 OS=Homo sapiens GN=DSG3 PE=1 SV=2 - (DSG3_HUMAN)	−	−	
B2R717	cDNA, FLJ93235, highly similar to Homo sapiens cathepsin L2 (CTSL2), mRNA OS=Homo sapiens PE=2 SV=1 - (B2R717_HUMAN)	−	−	
P14174	Macrophage migration inhibitory factor OS=Homo sapiens GN=MIF PE=1 SV=4 - (MIF_HUMAN)	−	−	
P62266	40S ribosomal protein S23 OS=Homo sapiens GN=RPS23 PE=1 SV=3 - (RS23_HUMAN)	−	−	
Q53RT3	Retroviral-like aspartic protease 1 OS=Homo sapiens GN=ASPRV1 PE=1 SV=1 - (APRV1_HUMAN)	−	−	
E7EPB3	60S ribosomal protein L14 OS=Homo sapiens GN=RPL14 PE=4 SV=1 - (E7EPB3_HUMAN)	−	−	
D3DV26	S100 calcium binding protein A10 (Annexin II ligand, calpactin I, light polypeptide (P11)), isoform CRA_b (Fragment) OS=Homo sapiens GN=S100A10 PE=4 SV=1 - (D3DV26_HUMAN)	−	−	
P29034	Protein S100-A2 OS=Homo sapiens GN=S100A2 PE=1 SV=3 - (S10A2_HUMAN)	−	−	
P26641	Elongation factor 1-gamma OS=Homo sapiens GN=EEF1G PE=1 SV=3 - (EF1G_HUMAN)	−	−	
P49207	60S ribosomal protein L34 OS=Homo sapiens GN=RPL34 PE=1 SV=3 - (RL34_HUMAN)	−	−	
P60900	Proteasome subunit alpha type-6 OS=Homo sapiens GN=PSMA6 PE=1 SV=1 - (PSA6_HUMAN)	−	−	
P62273	40S ribosomal protein S29 OS=Homo sapiens GN=RPS29 PE=1 SV=2 - (RS29_HUMAN)	−	−	
Q9Y3D6	Mitochondrial fission 1 protein OS=Homo sapiens GN=FIS1 PE=1 SV=2 - (FIS1_HUMAN)	−	−	
F5GY37	Prohibitin-2 OS=Homo sapiens GN=PHB2 PE=4 SV=1 - (F5GY37_HUMAN)	−	−	
P62304	Small nuclear ribonucleoprotein E OS=Homo sapiens GN=SNRPE PE=1 SV=1 - (RUXE_HUMAN)	−	−	
C9JFR7	Cytochrome c (Fragment) OS=Homo sapiens GN=CYCS PE=3 SV=1 - (C9JFR7_HUMAN)	−	−	
P00918	Carbonic anhydrase 2 OS=Homo sapiens GN=CA2 PE=1 SV=2 - (CAH2_HUMAN)	−	−	
P42766	60S ribosomal protein L35 OS=Homo sapiens GN=RPL35 PE=1 SV=2 - (RL35_HUMAN)	−	−	
Q96AG4	Leucine-rich repeat-containing protein 59 OS=Homo sapiens GN=LRRC59 PE=1 SV=1 - (LRC59_HUMAN)	−	−	
P48047	ATP synthase subunit O, mitochondrial OS=Homo sapiens GN=ATP5O PE=1 SV=1 - (ATPO_HUMAN)	+	−	
P80188	Neutrophil gelatinase-associated lipocalin OS=Homo sapiens GN=LCN2 PE=1 SV=2 - (NGAL_HUMAN)	−	−	
C9J9K3	40S ribosomal protein SA (Fragment) OS=Homo sapiens GN=RPSA PE=3 SV=1 - (C9J9K3_HUMAN)	−	−	
Q9P1F3	Costars family protein ABRACL OS=Homo sapiens GN=ABRACL PE=1 SV=1 - (ABRAL_HUMAN)	−	−	
P62280	40S ribosomal protein S11 OS=Homo sapiens GN=RPS11 PE=1 SV=3 - (RS11_HUMAN)	−	−	
Q8IXI1	Mitochondrial Rho GTPase 2 OS=Homo sapiens GN=RHOT2 PE=1 SV=2 - (MIRO2_HUMAN)	−	−	
P30041	Peroxiredoxin-6 OS=Homo sapiens GN=PRDX6 PE=1 SV=3 - (PRDX6_HUMAN)	+	−	
Q3LI83	Keratin-associated protein 24-1 OS=Homo sapiens GN=KRTAP24-1 PE=1 SV=1 - (KR241_HUMAN)	−	−	
P0CW22	40S ribosomal protein S17-like OS=Homo sapiens GN=RPS17L PE=3 SV=1 - (RS17L_HUMAN)	−	−	
Q15102	Platelet-activating factor acetylhydrolase IB subunit gamma OS=Homo sapiens GN=PAFAH1B3 PE=1 SV=1 - (PA1B3_HUMAN)	−	−	
P18621	60S ribosomal protein L17 OS=Homo sapiens GN=RPL17 PE=1 SV=3 - (RL17_HUMAN)	−	−	
Q9Y3U8	60S ribosomal protein L36 OS=Homo sapiens GN=RPL36 PE=1 SV=3 - (RL36_HUMAN)	−	−	
Q53FH6	Mitogen-activated protein kinase kinase 1 interacting protein 1 variant (Fragment) OS=Homo sapiens PE=2 SV=1 - (Q53FH6_HUMAN)	−	−	
P16402	Histone H1.3 OS=Homo sapiens GN=HIST1H1D PE=1 SV=2 - (H13_HUMAN)	−	−	
P16152	Carbonyl reductase (NADPH) 1 OS=Homo sapiens GN=CBR1 PE=1 SV=3 - (CBR1_HUMAN)	−	−	
Q99497	Protein DJ-1 OS=Homo sapiens GN=PARK7 PE=1 SV=2 - (PARK7_HUMAN)	−	−	
P04632	Calpain small subunit 1 OS=Homo sapiens GN=CAPNS1 PE=1 SV=1 - (CPNS1_HUMAN)	−	−	
Q96QR8	Transcriptional activator protein Pur-beta OS=Homo sapiens GN=PURB PE=1 SV=3 - (PURB_HUMAN)	−	−	
A8K0H3	cDNA FLJ78093, highly similar to Homo sapiens ribosomal protein L29 (RPL29), mRNA OS=Homo sapiens PE=2 SV=1 - (A8K0H3_HUMAN)	−	−	

Appendix C 

**Table 5:** Proteins identified in Subject 1 (N1).

Accession	Description	Score	Coverage	# Proteins	# Unique Peptides	# Peptides	# PSMs	Area	# AAs	MW (kDa)	calc. pI
P13647	Keratin, type II cytoskeletal 5 OS=Homo sapiens GN=KRT5 PE=1 SV=3 - (K2C5_HUMAN)	8029.67	66.95	26	20	86	3805	1.515E10	590	62.3	7.74
P02533	Keratin, type I cytoskeletal 14 OS=Homo sapiens GN=KRT14 PE=1 SV=4 - (K1C14_HUMAN)	7881.19	85.38	12	26	70	3370	1.841E10	472	51.5	5.16
P04259	Keratin, type II cytoskeletal 6B OS=Homo sapiens GN=KRT6B PE=1 SV=5 - (K2C6B_HUMAN)	7659.53	71.10	21	3	84	3624	1.472E10	564	60.0	8.00
B4DRR0	cDNA FLJ53910, highly similar to Keratin, type II cytoskeletal 6A OS=Homo sapiens PE=2 SV=1 - (B4DRR0_HUMAN)	7655.37	76.82	25	4	85	3572	1.472E10	535	57.8	8.00
B4DKV4	cDNA FLJ60647, highly similar to Keratin, type II cytoskeletal 6B OS=Homo sapiens PE=2 SV=1 - (B4DKV4_HUMAN)	6363.53	66.92	18	2	71	3040	1.472E10	526	55.7	8.43
Q04695	Keratin, type I cytoskeletal 17 OS=Homo sapiens GN=KRT17 PE=1 SV=2 - (K1C17_HUMAN)	5740.53	96.99	8	22	60	2500	1.787E10	432	48.1	5.02
P08779	Keratin, type I cytoskeletal 16 OS=Homo sapiens GN=KRT16 PE=1 SV=4 - (K1C16_HUMAN)	5449.79	84.99	11	28	57	2291	1.787E10	473	51.2	5.05
O43790	Keratin, type II cuticular Hb6 OS=Homo sapiens GN=KRT86 PE=1 SV=1 - (KRT86_HUMAN)	5210.58	94.65	14	3	70	2151	1.155E10	486	53.5	5.66
H6VRF8	Keratin 1 OS=Homo sapiens GN=KRT1 PE=3 SV=1 - (H6VRF8_HUMAN)	5139.20	70.19	16	49	64	1892	3.644E9	644	66.0	8.12
P78386	Keratin, type II cuticular Hb5 OS=Homo sapiens GN=KRT85 PE=1 SV=1 - (KRT85_HUMAN)	4870.88	92.70	7	28	71	2000	1.146E10	507	55.8	6.55
P35908	Keratin, type II cytoskeletal 2 epidermal OS=Homo sapiens GN=KRT2 PE=1 SV=2 - (K22E_HUMAN)	4716.67	77.78	8	40	66	2095	1.006E10	639	65.4	8.00
Q15323	Keratin, type I cuticular Ha1 OS=Homo sapiens GN=KRT31 PE=2 SV=3 - (K1H1_HUMAN)	4509.35	74.28	6	12	52	1903	1.482E10	416	47.2	4.88
F8W6V8	Keratin, type II cuticular Hb1 OS=Homo sapiens GN=KRT81 PE=3 SV=1 - (F8W6V8_HUMAN)	4444.03	79.75	13	1	64	1939	1.133E10	484	53.1	5.47
P78385	Keratin, type II cuticular Hb3 OS=Homo sapiens GN=KRT83 PE=1 SV=2 - (KRT83_HUMAN)	4120.28	79.72	11	5	64	1819	1.155E10	493	54.2	5.64
P13645	Keratin, type I cytoskeletal 10 OS=Homo sapiens GN=KRT10 PE=1 SV=6 - (K1C10_HUMAN)	4005.39	69.69	1	34	47	1667	1.106E10	584	58.8	5.21
O95678	Keratin, type II cytoskeletal 75 OS=Homo sapiens GN=KRT75 PE=1 SV=2 - (K2C75_HUMAN)	3932.11	65.15	18	28	63	1916	1.201E10	551	59.5	7.74
H0YI76	Keratin, type II cytoskeletal 5 (Fragment) OS=Homo sapiens GN=KRT5 PE=3 SV=1 - (H0YI76_HUMAN)	3541.80	77.61	13	1	36	1450	7.802E9	201	23.1	4.97
Q14525	Keratin, type I cuticular Ha3-II OS=Homo sapiens GN=KRT33B PE=2 SV=3 - (KT33B_HUMAN)	3312.24	81.93	5	11	45	1382	1.482E10	404	46.2	4.84
O76009	Keratin, type I cuticular Ha3-I OS=Homo sapiens GN=KRT33A PE=2 SV=2 - (KT33A_HUMAN)	3258.16	81.93	2	9	44	1385	1.107E10	404	45.9	4.82
Q07283	Trichohyalin OS=Homo sapiens GN=TCHH PE=1 SV=2 - (TRHY_HUMAN)	2918.82	76.22	31	162	234	1896	1.257E9	1943	253.8	5.78
A6NCN2	Keratin-81-like protein OS=Homo sapiens PE=2 SV=3 - (KT81L_HUMAN)	2765.47	59.47	5	2	46	1215	1.129E10	486	53.4	6.35
Q5XKE5	Keratin, type II cytoskeletal 79 OS=Homo sapiens GN=KRT79 PE=1 SV=2 - (K2C79_HUMAN)	2629.46	29.72	7	1	25	1578	1.210E10	535	57.8	7.20
P15924	Desmoplakin OS=Homo sapiens GN=DSP PE=1 SV=3 - (DESP_HUMAN)	2610.69	59.35	15	147	211	1309	2.375E9	2871	331.6	6.81
Q3SY84	Keratin, type II cytoskeletal 71 OS=Homo sapiens GN=KRT71 PE=1 SV=3 - (K2C71_HUMAN)	2509.80	69.22	2	28	66	1487	7.161E9	523	57.3	6.61
Q01546	Keratin, type II cytoskeletal 2 oral OS=Homo sapiens GN=KRT76 PE=1 SV=2 - (K22O_HUMAN)	2337.33	33.86	19	3	39	1521	1.027E10	638	65.8	8.12
B3KVF5	cDNA FLJ16494 fis, clone CTONG3004576, highly similar to Keratin, type I cytoskeletal 15 OS=Homo sapiens PE=2 SV=1 - (B3KVF5_HUMAN)	2069.44	59.65	8	9	30	765	1.787E10	456	49.1	4.79
P35527	Keratin, type I cytoskeletal 9 OS=Homo sapiens GN=KRT9 PE=1 SV=3 - (K1C9_HUMAN)	2044.56	61.32	1	31	34	736	3.593E9	623	62.0	5.24
H9KV34	Uncharacterized protein OS=Homo sapiens PE=3 SV=1 - (H9KV34_HUMAN)	2043.60	43.71	8	2	25	983	1.360E10	437	48.2	4.97
C4AMA3	Keratin, type I cuticular Ha4 OS=Homo sapiens GN=KRT34 PE=3 SV=1 - (C4AMA3_HUMAN)	1910.28	80.71	3	20	39	807	1.065E10	394	44.7	4.78
P08727	Keratin, type I cytoskeletal 19 OS=Homo sapiens GN=KRT19 PE=1 SV=4 - (K1C19_HUMAN)	1877.88	39.50	7	3	22	940	1.679E10	400	44.1	5.14
Q92764	Keratin, type I cuticular Ha5 OS=Homo sapiens GN=KRT35 PE=2 SV=5 - (KRT35_HUMAN)	1769.95	75.60	2	24	38	765	1.228E10	455	50.3	4.91
Q7Z3Y8	Keratin, type I cytoskeletal 27 OS=Homo sapiens GN=KRT27 PE=1 SV=2 - (K1C27_HUMAN)	1714.16	69.28	2	16	34	762	2.624E9	459	49.8	5.05
Q7Z3Z0	Keratin, type I cytoskeletal 25 OS=Homo sapiens GN=KRT25 PE=1 SV=1 - (K1C25_HUMAN)	1705.68	72.44	4	17	37	762	2.624E9	450	49.3	5.08
Q86Y46	Keratin, type II cytoskeletal 73 OS=Homo sapiens GN=KRT73 PE=1 SV=1 - (K2C73_HUMAN)	1673.20	39.26	7	2	37	1139	9.282E9	540	58.9	7.23
Q7RTS7	Keratin, type II cytoskeletal 74 OS=Homo sapiens GN=KRT74 PE=1 SV=2 - (K2C74_HUMAN)	1671.85	73.72	6	17	52	1124	7.575E9	529	57.8	7.71
Q14CN4-2	Isoform 2 of Keratin, type II cytoskeletal 72 OS=Homo sapiens GN=KRT72 - (K2C72_HUMAN)	1668.65	40.78	7	1	34	1168	7.970E9	510	55.7	6.89
P13646-3	Isoform 3 of Keratin, type I cytoskeletal 13 OS=Homo sapiens GN=KRT13 - (K1C13_HUMAN)	1407.89	35.71	11	2	21	625	1.300E10	420	45.8	4.88
Q8WVW5	Putative uncharacterized protein (Fragment) OS=Homo sapiens PE=2 SV=1 - (Q8WVW5_HUMAN)	1344.03	78.24	82	12	37	544	4.580E9	363	40.5	6.14
Q9NSB2	Keratin, type II cuticular Hb4 OS=Homo sapiens GN=KRT84 PE=1 SV=2 - (KRT84_HUMAN)	1165.89	54.67	15	16	35	731	1.027E10	600	64.8	7.56
Q7Z3Y7	Keratin, type I cytoskeletal 28 OS=Homo sapiens GN=KRT28 PE=1 SV=2 - (K1C28_HUMAN)	1070.14	43.53	1	9	24	585	8.767E9	464	50.5	5.47
Q9NSB4	Keratin, type II cuticular Hb2 OS=Homo sapiens GN=KRT82 PE=1 SV=3 - (KRT82_HUMAN)	1012.68	38.60	2	8	21	727	7.686E9	513	56.6	6.74
Q7Z794	Keratin, type II cytoskeletal 1b OS=Homo sapiens GN=KRT77 PE=1 SV=3 - (K2C1B_HUMAN)	925.45	24.05	5	1	14	571	8.071E9	578	61.9	5.99
F5GZD1	Keratin, type II cytoskeletal 7 OS=Homo sapiens GN=KRT7 PE=4 SV=1 - (F5GZD1_HUMAN)	888.38	15.88	3	1	13	676	1.027E10	403	44.9	5.55
Q14532	Keratin, type I cuticular Ha2 OS=Homo sapiens GN=KRT32 PE=1 SV=3 - (K1H2_HUMAN)	871.78	45.09	5	7	20	396	1.147E10	448	50.3	4.84
B4DRW1	cDNA FLJ55805, highly similar to Keratin, type II cytoskeletal 4 OS=Homo sapiens PE=2 SV=1 - (B4DRW1_HUMAN)	871.46	34.18	10	2	22	484	4.442E9	474	51.7	6.81
P07355-2	Isoform 2 of Annexin A2 OS=Homo sapiens GN=ANXA2 - (ANXA2_HUMAN)	866.40	85.71	27	41	48	325	2.318E9	357	40.4	8.37
P14923	Junction plakoglobin OS=Homo sapiens GN=JUP PE=1 SV=3 - (PLAK_HUMAN)	669.35	60.81	8	29	38	291	4.980E8	745	81.7	6.14
Q99456	Keratin, type I cytoskeletal 12 OS=Homo sapiens GN=KRT12 PE=1 SV=1 - (K1C12_HUMAN)	657.34	30.97	1	1	18	336	5.056E9	494	53.5	4.78
B7Z6P1	cDNA FLJ53662, highly similar to Actin, alpha skeletal muscle OS=Homo sapiens PE=2 SV=1 - (B7Z6P1_HUMAN)	633.91	40.23	47	1	22	339	2.608E9	343	38.6	5.38
F8VW92	Tubulin beta chain OS=Homo sapiens GN=TUBB PE=3 SV=1 - (F8VW92_HUMAN)	619.45	61.38	42	3	23	231	4.338E8	435	48.5	5.20
P31947	14-3-3 protein sigma OS=Homo sapiens GN=SFN PE=1 SV=1 - (1433S_HUMAN)	593.91	84.27	5	22	29	270	2.424E9	248	27.8	4.74
P04792	Heat shock protein beta-1 OS=Homo sapiens GN=HSPB1 PE=1 SV=2 - (HSPB1_HUMAN)	593.74	94.15	4	20	20	253	2.843E9	205	22.8	6.40
B7Z565	cDNA FLJ54739, highly similar to Alpha-actinin-1 OS=Homo sapiens PE=2 SV=1 - (B7Z565_HUMAN)	560.59	50.36	27	13	37	204	4.052E8	822	94.7	5.69
O43707	Alpha-actinin-4 OS=Homo sapiens GN=ACTN4 PE=1 SV=2 - (ACTN4_HUMAN)	549.83	47.97	18	14	41	195	4.293E8	911	104.8	5.44
A8MUB1	Tubulin alpha-4A chain OS=Homo sapiens GN=TUBA4A PE=2 SV=1 - (A8MUB1_HUMAN)	541.77	48.96	14	5	21	182	9.013E8	433	48.3	5.01
G3V1U9	Tubulin alpha-1A chain OS=Homo sapiens GN=TUBA1A PE=3 SV=1 - (G3V1U9_HUMAN)	541.48	55.77	30	6	23	182	1.011E9	416	46.3	5.08
Q13885	Tubulin beta-2A chain OS=Homo sapiens GN=TUBB2A PE=1 SV=1 - (TBB2A_HUMAN)	525.22	57.30	24	4	22	199	4.315E8	445	49.9	4.89
B4DE77	cDNA FLJ55189, highly similar to Tubulin beta-4 chain OS=Homo sapiens PE=2 SV=1 - (B4DE77_HUMAN)	513.94	54.03	7	1	19	196	4.315E8	409	45.8	4.87
B7ZBC3	Spectrin repeat containing, nuclear envelope 1 OS=Homo sapiens GN=SYNE1 PE=4 SV=1 - (B7ZBC3_HUMAN)	448.66	7.39	22	3	58	268	5.224E9	8797	1010.4	5.53
Q5QNW6	Histone H2B type 2-F OS=Homo sapiens GN=HIST2H2BF PE=1 SV=3 - (H2B2F_HUMAN)	446.02	71.43	32	9	14	215	4.264E9	126	13.9	10.32
P04406	Glyceraldehyde-3-phosphate dehydrogenase OS=Homo sapiens GN=GAPDH PE=1 SV=3 - (G3P_HUMAN)	445.13	79.10	11	20	26	204	6.559E9	335	36.0	8.46
Q562R1	Beta-actin-like protein 2 OS=Homo sapiens GN=ACTBL2 PE=1 SV=2 - (ACTBL_HUMAN)	427.38	22.87	13	1	15	232	2.987E9	376	42.0	5.59
P62805	Histone H4 OS=Homo sapiens GN=HIST1H4A PE=1 SV=2 - (H4_HUMAN)	412.22	66.99	3	13	15	186	3.559E9	103	11.4	11.36
Q13835-2	Isoform 1 of Plakophilin-1 OS=Homo sapiens GN=PKP1 - (PKP1_HUMAN)	402.93	54.41	4	28	38	194	3.788E8	726	80.4	8.97
Q2M2I5	Keratin, type I cytoskeletal 24 OS=Homo sapiens GN=KRT24 PE=1 SV=1 - (K1C24_HUMAN)	399.80	24.76	1	2	12	219	6.989E9	525	55.1	4.96
Q8TC04	Keratin 23 (Histone deacetylase inducible) OS=Homo sapiens GN=KRT23 PE=2 SV=1 - (Q8TC04_HUMAN)	380.37	10.43	1	1	6	292	4.520E9	422	48.1	6.54
P47929	Galectin-7 OS=Homo sapiens GN=LGALS7 PE=1 SV=2 - (LEG7_HUMAN)	368.05	98.53	1	14	17	142	4.397E9	136	15.1	7.62
Q5VTE0	Putative elongation factor 1-alpha-like 3 OS=Homo sapiens GN=EEF1A1P5 PE=5 SV=1 - (EF1A3_HUMAN)	363.69	48.92	40	18	25	167	9.303E8	462	50.2	9.07
Q15149-3	Isoform 3 of Plectin OS=Homo sapiens GN=PLEC - (PLEC_HUMAN)	361.71	27.37	16	49	127	244	3.533E9	4570	517.7	5.74
Q8N1N4	Keratin, type II cytoskeletal 78 OS=Homo sapiens GN=KRT78 PE=2 SV=2 - (K2C78_HUMAN)	360.40	27.69	4	3	16	230	5.397E9	520	56.8	6.02
Q9BTM1	Histone H2A.J OS=Homo sapiens GN=H2AFJ PE=1 SV=1 - (H2AJ_HUMAN)	348.77	48.06	16	3	11	135	3.631E9	129	14.0	10.90
Q93077	Histone H2A type 1-C OS=Homo sapiens GN=HIST1H2AC PE=1 SV=3 - (H2A1C_HUMAN)	339.76	41.54	6	2	9	126	3.631E9	130	14.1	11.05
Q13509	Tubulin beta-3 chain OS=Homo sapiens GN=TUBB3 PE=1 SV=2 - (TBB3_HUMAN)	334.06	42.00	11	1	17	135	4.012E8	450	50.4	4.93
P63104	14-3-3 protein zeta/delta OS=Homo sapiens GN=YWHAZ PE=1 SV=1 - (1433Z_HUMAN)	320.31	70.61	17	10	23	152	1.452E9	245	27.7	4.79
P27348	14-3-3 protein theta OS=Homo sapiens GN=YWHAQ PE=1 SV=1 - (1433T_HUMAN)	290.69	63.67	9	9	20	127	1.705E9	245	27.7	4.78
P35579	Myosin-9 OS=Homo sapiens GN=MYH9 PE=1 SV=4 - (MYH9_HUMAN)	246.96	29.23	15	25	61	150	2.564E8	1960	226.4	5.60
B5BU24	14-3-3 protein beta/alpha OS=Homo sapiens GN=YWHAB PE=2 SV=1 - (B5BU24_HUMAN)	242.18	51.22	8	2	15	112	1.897E9	246	28.1	4.83
Q01469	Fatty acid-binding protein, epidermal OS=Homo sapiens GN=FABP5 PE=1 SV=3 - (FABP5_HUMAN)	228.02	92.59	2	14	20	107	1.391E9	135	15.2	7.01
P11142	Heat shock cognate 71 kDa protein OS=Homo sapiens GN=HSPA8 PE=1 SV=1 - (HSP7C_HUMAN)	219.27	43.96	28	12	30	129	1.391E9	646	70.9	5.52
P61981	14-3-3 protein gamma OS=Homo sapiens GN=YWHAG PE=1 SV=2 - (1433G_HUMAN)	218.71	42.11	10	3	14	110	1.705E9	247	28.3	4.89
D3DPG0	Titin, isoform CRA_a OS=Homo sapiens GN=TTN PE=4 SV=1 - (D3DPG0_HUMAN)	216.70	4.51	15	1	143	305	2.813E9	34942	3878.8	6.38
Q562V5	Actin-like protein (Fragment) OS=Homo sapiens GN=ACT PE=2 SV=1 - (Q562V5_HUMAN)	192.09	52.43	14	1	3	70	2.371E9	103	11.4	6.35
P08238	Heat shock protein HSP 90-beta OS=Homo sapiens GN=HSP90AB1 PE=1 SV=4 - (HS90B_HUMAN)	191.14	29.14	40	9	20	83	2.097E8	724	83.2	5.03
Q9HD64	G antigen family D member 2 OS=Homo sapiens GN=XAGE1A PE=2 SV=3 - (GAGD2_HUMAN)	189.50	45.68	7	1	3	89	9.636E7	81	9.1	9.58
F5H288	Vimentin OS=Homo sapiens GN=VIM PE=3 SV=1 - (F5H288_HUMAN)	181.47	14.74	20	1	9	124	5.645E9	407	47.0	5.00
P54652	Heat shock-related 70 kDa protein 2 OS=Homo sapiens GN=HSPA2 PE=1 SV=1 - (HSP72_HUMAN)	180.69	48.67	3	11	31	109	1.391E9	639	70.0	5.74
O75116	Rho-associated protein kinase 2 OS=Homo sapiens GN=ROCK2 PE=1 SV=4 - (ROCK2_HUMAN)	179.25	14.55	4	1	21	107	4.433E9	1388	160.8	6.02
Q6KB66-2	Isoform 2 of Keratin, type II cytoskeletal 80 OS=Homo sapiens GN=KRT80 - (K2C80_HUMAN)	179.13	29.86	5	4	10	98	3.851E9	422	47.2	5.30
P06733	Alpha-enolase OS=Homo sapiens GN=ENO1 PE=1 SV=2 - (ENOA_HUMAN)	173.77	65.90	25	18	27	88	9.186E8	434	47.1	7.39
Q7Z406	Myosin-14 OS=Homo sapiens GN=MYH14 PE=1 SV=2 - (MYH14_HUMAN)	171.06	21.55	17	11	44	107	3.533E8	1995	227.7	5.60
P62258	14-3-3 protein epsilon OS=Homo sapiens GN=YWHAE PE=1 SV=1 - (1433E_HUMAN)	165.93	41.96	12	7	12	75	1.345E9	255	29.2	4.74
P36952	Serpin B5 OS=Homo sapiens GN=SERPINB5 PE=1 SV=2 - (SPB5_HUMAN)	162.49	59.47	3	8	18	64	4.863E8	375	42.1	6.05
O75369-2	Isoform 2 of Filamin-B OS=Homo sapiens GN=FLNB - (FLNB_HUMAN)	161.73	15.44	15	14	39	86	9.503E8	2578	275.5	5.78
P07900	Heat shock protein HSP 90-alpha OS=Homo sapiens GN=HSP90AA1 PE=1 SV=5 - (HS90A_HUMAN)	160.47	22.95	33	6	17	71	2.068E8	732	84.6	5.02
P68431	Histone H3.1 OS=Homo sapiens GN=HIST1H3A PE=1 SV=2 - (H31_HUMAN)	158.17	87.50	12	5	18	123	3.362E9	136	15.4	11.12
Q96QV6	Histone H2A type 1-A OS=Homo sapiens GN=HIST1H2AA PE=1 SV=3 - (H2A1A_HUMAN)	156.57	40.46	6	1	6	72	3.013E9	131	14.2	10.86
B4DPP6	cDNA FLJ54371, highly similar to Serum albumin OS=Homo sapiens PE=2 SV=1 - (B4DPP6_HUMAN)	156.23	27.83	15	11	16	76	1.364E8	618	70.3	6.09
P62979	Ubiquitin-40S ribosomal protein S27a OS=Homo sapiens GN=RPS27A PE=1 SV=2 - (RS27A_HUMAN)	155.49	53.85	28	1	13	101	4.568E8	156	18.0	9.64
A0N4V7	HCG2039797 (Fragment) OS=Homo sapiens GN=Tcr-alpha PE=4 SV=1 - (A0N4V7_HUMAN)	153.79	38.10	1	1	1	176	1.191E10	21	2.2	9.74
F8W8Y7	Armadillo repeat-containing X-linked protein 4 OS=Homo sapiens GN=ARMCX4 PE=4 SV=1 - (F8W8Y7_HUMAN)	153.56	7.81	3	2	15	110	8.814E8	2394	247.7	5.95
P62987	Ubiquitin-60S ribosomal protein L40 OS=Homo sapiens GN=UBA52 PE=1 SV=2 - (RL40_HUMAN)	153.06	62.50	26	1	11	98	4.568E8	128	14.7	9.83
Q0Z7S8	Fatty acid-binding protein 9 OS=Homo sapiens GN=FABP9 PE=1 SV=1 - (FABP9_HUMAN)	145.03	86.36	1	11	13	68	4.307E8	132	15.1	8.09
Q86TY5	Full-length cDNA clone CS0DI041YE05 of Placenta of Homo sapiens (human) OS=Homo sapiens PE=2 SV=1 - (Q86TY5_HUMAN)	144.22	84.30	16	12	12	87	4.942E8	121	13.9	9.16
P13639	Elongation factor 2 OS=Homo sapiens GN=EEF2 PE=1 SV=4 - (EF2_HUMAN)	143.12	27.16	16	14	20	65	2.694E8	858	95.3	6.83
Q8WVJ2	NudC domain-containing protein 2 OS=Homo sapiens GN=NUDCD2 PE=1 SV=1 - (NUDC2_HUMAN)	139.11	21.66	1	1	3	88	1.090E8	157	17.7	5.07
Q6DN03	Putative histone H2B type 2-C OS=Homo sapiens GN=HIST2H2BC PE=5 SV=3 - (H2B2C_HUMAN)	138.67	30.57	20	2	9	79	7.440E8	193	21.5	10.70
P04083	Annexin A1 OS=Homo sapiens GN=ANXA1 PE=1 SV=2 - (ANXA1_HUMAN)	136.51	55.49	6	16	18	55	3.219E8	346	38.7	7.02
H0YG33	Heat shock 70 kDa protein 1A/1B OS=Homo sapiens GN=HSPA1B PE=3 SV=1 - (H0YG33_HUMAN)	136.12	32.91	22	14	25	83	5.262E8	708	77.4	6.99
Q15828	Cystatin-M OS=Homo sapiens GN=CST6 PE=1 SV=1 - (CYTM_HUMAN)	131.21	71.14	6	9	11	49	6.223E8	149	16.5	8.09
Q562Q2	Actin-like protein (Fragment) OS=Homo sapiens GN=ACT PE=3 SV=1 - (Q562Q2_HUMAN)	130.10	33.98	10	1	3	70	1.096E9	103	11.5	6.68
Q09666	Neuroblast differentiation-associated protein AHNAK OS=Homo sapiens GN=AHNAK PE=1 SV=2 - (AHNK_HUMAN)	127.29	29.78	5	22	56	100	3.083E8	5890	628.7	6.15
Q6PK75	PRSS2 protein (Fragment) OS=Homo sapiens GN=PRSS2 PE=2 SV=1 - (Q6PK75_HUMAN)	127.11	16.04	16	1	2	60	8.433E8	187	20.1	4.87
Q5HY54	Filamin-A OS=Homo sapiens GN=FLNA PE=2 SV=1 - (Q5HY54_HUMAN)	122.82	11.32	18	13	29	70	1.630E8	2607	276.4	6.05
P60174	Triosephosphate isomerase OS=Homo sapiens GN=TPI1 PE=1 SV=3 - (TPIS_HUMAN)	121.57	83.22	6	15	20	46	3.783E8	286	30.8	5.92
P02545-2	Isoform C of Prelamin-A/C OS=Homo sapiens GN=LMNA - (LMNA_HUMAN)	121.20	50.70	17	17	26	63	5.790E8	572	65.1	6.84
B3KWI4	cDNA FLJ43122 fis, clone CTONG3003737, highly similar to Leucine-rich repeat-containing protein 15 OS=Homo sapiens PE=2 SV=1 - (B3KWI4_HUMAN)	119.53	21.17	3	8	11	51	1.556E8	581	64.4	6.71
F8WA45	Gelsolin OS=Homo sapiens GN=GSN PE=4 SV=1 - (F8WA45_HUMAN)	118.75	22.38	19	9	18	61	1.588E8	715	78.9	6.01
O43175	D-3-phosphoglycerate dehydrogenase OS=Homo sapiens GN=PHGDH PE=1 SV=4 - (SERA_HUMAN)	116.51	23.64	11	8	12	52	1.368E8	533	56.6	6.71
Q02413	Desmoglein-1 OS=Homo sapiens GN=DSG1 PE=1 SV=2 - (DSG1_HUMAN)	113.81	17.45	2	11	15	50	1.180E8	1049	113.7	5.03
P37802	Transgelin-2 OS=Homo sapiens GN=TAGLN2 PE=1 SV=3 - (TAGL2_HUMAN)	113.69	75.38	2	9	14	40	1.498E8	199	22.4	8.25
P00338-3	Isoform 3 of L-lactate dehydrogenase A chain OS=Homo sapiens GN=LDHA - (LDHA_HUMAN)	113.01	39.89	25	8	16	61	2.489E8	361	39.8	8.43
O15066	Kinesin-like protein KIF3B OS=Homo sapiens GN=KIF3B PE=1 SV=1 - (KIF3B_HUMAN)	111.31	9.10	2	1	8	91	7.903E8	747	85.1	7.69
O94819	Kelch repeat and BTB domain-containing protein 11 OS=Homo sapiens GN=KBTBD11 PE=1 SV=1 - (KBTBB_HUMAN)	109.59	3.21	1	1	3	57	1.359E9	623	65.7	6.07
P0C0S5	Histone H2A.Z OS=Homo sapiens GN=H2AFZ PE=1 SV=2 - (H2AZ_HUMAN)	108.79	31.25	9	2	4	50	2.868E9	128	13.5	10.58
Q86UQ4	ATP-binding cassette sub-family A member 13 OS=Homo sapiens GN=ABCA13 PE=2 SV=3 - (ABCAD_HUMAN)	106.87	4.76	4	2	23	64	1.174E9	5058	575.8	6.46
P09211	Glutathione S-transferase P OS=Homo sapiens GN=GSTP1 PE=1 SV=2 - (GSTP1_HUMAN)	106.33	67.14	4	8	9	39	6.739E8	210	23.3	5.64
P25054-2	Isoform Short of Adenomatous polyposis coli protein OS=Homo sapiens GN=APC - (APC_HUMAN)	106.16	9.66	5	1	22	78	1.742E9	2742	300.3	7.77
Q8IUX7	Adipocyte enhancer-binding protein 1 OS=Homo sapiens GN=AEBP1 PE=1 SV=1 - (AEBP1_HUMAN)	102.85	5.96	3	1	7	138	8.363E8	1158	130.8	5.11
B4DF70	cDNA FLJ60461, highly similar to Peroxiredoxin-2 (EC 1.11.1.15) OS=Homo sapiens PE=2 SV=1 - (B4DF70_HUMAN)	100.37	38.80	3	6	9	38	2.227E8	183	20.1	8.78
Q3LI55	Keratin associated protein OS=Homo sapiens GN=KRTAP11-1 PE=2 SV=1 - (Q3LI55_HUMAN)	99.29	26.99	2	3	5	83	5.988E8	163	17.0	7.78
P15311	Ezrin OS=Homo sapiens GN=EZR PE=1 SV=4 - (EZRI_HUMAN)	97.95	29.86	32	11	24	66	1.364E9	586	69.4	6.27
P40926	Malate dehydrogenase, mitochondrial OS=Homo sapiens GN=MDH2 PE=1 SV=3 - (MDHM_HUMAN)	96.85	59.47	8	15	16	35	1.183E8	338	35.5	8.68
B4DWS9	cDNA FLJ57640, highly similar to Serpin B5 OS=Homo sapiens PE=2 SV=1 - (B4DWS9_HUMAN)	96.05	42.86	1	1	11	47	4.863E8	287	32.4	6.54
A8K486	Peptidyl-prolyl cis-trans isomerase OS=Homo sapiens PE=2 SV=1 - (A8K486_HUMAN)	92.95	67.27	9	10	12	50	6.838E8	165	18.0	6.90
C9J1U3	Protein unc-80 homolog OS=Homo sapiens GN=UNC80 PE=4 SV=1 - (C9J1U3_HUMAN)	91.34	4.24	4	1	13	54	1.429E9	3234	360.6	6.92
P05109	Protein S100-A8 OS=Homo sapiens GN=S100A8 PE=1 SV=1 - (S10A8_HUMAN)	91.28	60.22	1	12	13	46	8.041E8	93	10.8	7.03
Q86YZ3	Hornerin OS=Homo sapiens GN=HRNR PE=1 SV=2 - (HORN_HUMAN)	90.91	22.25	2	11	18	50	2.446E8	2850	282.2	10.04
Q15057	Arf-GAP with coiled-coil, ANK repeat and PH domain-containing protein 2 OS=Homo sapiens GN=ACAP2 PE=1 SV=3 - (ACAP2_HUMAN)	90.36	6.68	2	1	5	60	1.113E8	778	88.0	6.80
Q92817	Envoplakin OS=Homo sapiens GN=EVPL PE=1 SV=3 - (EVPL_HUMAN)	89.88	15.20	2	3	34	66	1.094E9	2033	231.5	6.96
P27482	Calmodulin-like protein 3 OS=Homo sapiens GN=CALML3 PE=1 SV=2 - (CALL3_HUMAN)	88.20	53.69	1	5	7	33	5.639E8	149	16.9	4.42
P14618	Pyruvate kinase isozymes M1/M2 OS=Homo sapiens GN=PKM2 PE=1 SV=4 - (KPYM_HUMAN)	85.15	28.63	27	11	14	40	2.112E8	531	57.9	7.84
P55072	Transitional endoplasmic reticulum ATPase OS=Homo sapiens GN=VCP PE=1 SV=4 - (TERA_HUMAN)	85.05	28.41	11	13	19	47	1.249E8	806	89.3	5.26
P16402	Histone H1.3 OS=Homo sapiens GN=HIST1H1D PE=1 SV=2 - (H13_HUMAN)	84.04	34.39	9	5	14	78	3.958E9	221	22.3	11.02
P31949	Protein S100-A11 OS=Homo sapiens GN=S100A11 PE=1 SV=2 - (S10AB_HUMAN)	81.56	56.19	2	7	8	28	2.531E8	105	11.7	7.12
Q06830	Peroxiredoxin-1 OS=Homo sapiens GN=PRDX1 PE=1 SV=1 - (PRDX1_HUMAN)	79.71	46.73	4	8	11	35	2.113E8	199	22.1	8.13
Q14204	Cytoplasmic dynein 1 heavy chain 1 OS=Homo sapiens GN=DYNC1H1 PE=1 SV=5 - (DYHC1_HUMAN)	77.41	7.17	1	2	30	90	2.797E9	4646	532.1	6.40
B0AZQ4	cDNA, FLJ79494, highly similar to Structural maintenance of chromosome 3 OS=Homo sapiens PE=2 SV=1 - (B0AZQ4_HUMAN)	77.10	16.43	3	1	21	71	1.802E9	1217	141.4	7.18
H0YIG3	Keratin, type II cytoskeletal 72 (Fragment) OS=Homo sapiens GN=KRT72 PE=4 SV=1 - (H0YIG3_HUMAN)	75.96	14.58	1	1	4	42	7.342E8	192	21.7	11.19
P33764	Protein S100-A3 OS=Homo sapiens GN=S100A3 PE=1 SV=1 - (S10A3_HUMAN)	75.72	72.28	1	8	8	32	3.623E8	101	11.7	4.78
C9JH62	Zinc finger protein 277 (Fragment) OS=Homo sapiens GN=ZNF277 PE=4 SV=1 - (C9JH62_HUMAN)	75.38	28.14	11	1	4	39	1.291E9	199	22.7	6.16
P09382	Galectin-1 OS=Homo sapiens GN=LGALS1 PE=1 SV=2 - (LEG1_HUMAN)	73.98	61.48	2	6	10	28	2.224E8	135	14.7	5.50
Q8WUT4	Leucine-rich repeat neuronal protein 4 OS=Homo sapiens GN=LRRN4 PE=1 SV=3 - (LRRN4_HUMAN)	73.24	6.62	2	2	4	51	3.112E8	740	78.8	7.20
P28838-2	Isoform 2 of Cytosol aminopeptidase OS=Homo sapiens GN=LAP3 - (AMPL_HUMAN)	72.39	39.55	5	12	17	46	9.352E8	488	52.7	6.74
B2R4K7	60S ribosomal protein L6 OS=Homo sapiens PE=2 SV=1 - (B2R4K7_HUMAN)	71.43	31.25	13	4	11	56	4.086E8	288	32.7	10.62
Q14134	Tripartite motif-containing protein 29 OS=Homo sapiens GN=TRIM29 PE=1 SV=2 - (TRI29_HUMAN)	71.33	32.82	18	14	20	44	2.043E8	588	65.8	7.15
Q96IR1	RPS4X protein (Fragment) OS=Homo sapiens GN=RPS4X PE=2 SV=2 - (Q96IR1_HUMAN)	70.94	51.85	11	12	14	33	1.259E8	243	27.2	9.94
P07737	Profilin-1 OS=Homo sapiens GN=PFN1 PE=1 SV=2 - (PROF1_HUMAN)	69.56	55.71	2	7	8	30	2.151E8	140	15.0	8.27
F5H6K1	Dystrophin OS=Homo sapiens GN=DMD PE=4 SV=1 - (F5H6K1_HUMAN)	69.09	9.17	13	1	34	75	6.435E8	3685	426.4	5.91
Q9UPN3	Microtubule-actin cross-linking factor 1, isoforms 1/2/3/5 OS=Homo sapiens GN=MACF1 PE=1 SV=4 - (MACF1_HUMAN)	68.87	7.70	11	1	50	104	2.116E9	7388	837.8	5.39
P46782	40S ribosomal protein S5 OS=Homo sapiens GN=RPS5 PE=1 SV=4 - (RS5_HUMAN)	66.80	39.22	3	5	9	27	4.830E8	204	22.9	9.72
A3RLL6	Cellular apoptosis susceptibility protein variant 2 OS=Homo sapiens GN=CSE1L-2 PE=2 SV=1 - (A3RLL6_HUMAN)	66.80	2.08	9	1	2	45	4.578E9	915	103.8	6.11
P07195	L-lactate dehydrogenase B chain OS=Homo sapiens GN=LDHB PE=1 SV=2 - (LDHB_HUMAN)	66.22	32.93	12	6	13	32	2.363E8	334	36.6	6.05
F8VW21	60S acidic ribosomal protein P0 (Fragment) OS=Homo sapiens GN=RPLP0 PE=3 SV=1 - (F8VW21_HUMAN)	65.84	32.38	5	1	9	30	2.332E8	244	26.9	8.97
Q8NAM9	cDNA FLJ35099 fis, clone PLACE6006287 OS=Homo sapiens PE=2 SV=1 - (Q8NAM9_HUMAN)	64.57	8.93	1	1	3	52	1.633E9	224	24.0	11.75
A5D8V7	Coiled-coil domain-containing protein 151 OS=Homo sapiens GN=CCDC151 PE=2 SV=1 - (CC151_HUMAN)	63.92	15.63	6	1	8	56	4.306E8	595	69.1	9.07
O95613	Pericentrin OS=Homo sapiens GN=PCNT PE=1 SV=4 - (PCNT_HUMAN)	63.79	11.33	3	1	35	86	8.216E8	3336	377.8	5.55
B7Z7A9	Phosphoglycerate kinase OS=Homo sapiens GN=PGK1 PE=2 SV=1 - (B7Z7A9_HUMAN)	63.78	37.28	7	8	11	34	2.554E8	389	41.4	7.33
A2RRR7	CCDC46 protein OS=Homo sapiens GN=CCDC46 PE=2 SV=1 - (A2RRR7_HUMAN)	63.44	13.80	6	1	14	50	4.199E8	913	107.6	6.34
Q96M86	Dynein heavy chain domain-containing protein 1 OS=Homo sapiens GN=DNHD1 PE=1 SV=2 - (DNHD1_HUMAN)	61.96	3.68	4	1	19	53	4.197E8	4753	533.3	6.71
O15230	Laminin subunit alpha-5 OS=Homo sapiens GN=LAMA5 PE=1 SV=8 - (LAMA5_HUMAN)	61.82	3.22	2	1	10	40	2.699E8	3695	399.5	7.02
A8K4Z4	cDNA FLJ75549, highly similar to Homo sapiens ribosomal protein, large, P0 (RPLP0), transcript variant 1, mRNA OS=Homo sapiens PE=2 SV=1 - (A8K4Z4_HUMAN)	61.51	28.71	19	1	9	28	1.321E8	317	34.2	5.97
O60437	Periplakin OS=Homo sapiens GN=PPL PE=1 SV=4 - (PEPL_HUMAN)	60.87	11.96	1	5	25	50	2.718E8	1756	204.6	5.60
Q9UKE5-4	Isoform 4 of TRAF2 and NCK-interacting protein kinase OS=Homo sapiens GN=TNIK - (TNIK_HUMAN)	60.12	10.36	40	2	18	55	1.205E9	1352	153.9	7.17
I0B0K8	Truncated profilaggrin OS=Homo sapiens GN=FLG PE=4 SV=1 - (I0B0K8_HUMAN)	59.88	16.31	16	4	26	66	2.869E8	4021	430.2	9.29
P07476	Involucrin OS=Homo sapiens GN=IVL PE=1 SV=2 - (INVO_HUMAN)	59.53	21.37	3	8	12	50	1.460E8	585	68.4	4.61
A8K4W0	cDNA FLJ78591, highly similar to Homo sapiens ribosomal protein S3A (RPS3A), mRNA OS=Homo sapiens PE=2 SV=1 - (A8K4W0_HUMAN)	59.21	45.45	16	11	16	37	1.456E8	264	29.9	9.73
Q8IWY4	Signal peptide, CUB and EGF-like domain-containing protein 1 OS=Homo sapiens GN=SCUBE1 PE=1 SV=3 - (SCUB1_HUMAN)	58.98	5.87	3	1	4	34	5.255E8	988	107.8	6.83
B2RAW1	cDNA, FLJ95155, highly similar to Homo sapiens Bruton agammaglobulinemia tyrosine kinase (BTK), mRNA OS=Homo sapiens PE=2 SV=1 - (B2RAW1_HUMAN)	58.79	8.95	14	1	5	39	5.526E8	659	76.2	7.77
B4DSW9	cDNA FLJ59415, highly similar to Beta-catenin OS=Homo sapiens PE=2 SV=1 - (B4DSW9_HUMAN)	58.70	19.89	6	2	11	47	3.543E8	709	77.5	6.38
P07339	Cathepsin D OS=Homo sapiens GN=CTSD PE=1 SV=1 - (CATD_HUMAN)	57.25	28.88	6	8	12	23	1.392E8	412	44.5	6.54
B4DM97	cDNA FLJ55002, highly similar to Alpha-centractin OS=Homo sapiens PE=2 SV=1 - (B4DM97_HUMAN)	57.04	17.37	20	3	6	31	1.033E9	334	38.3	6.96
Q9P2M7	Cingulin OS=Homo sapiens GN=CGN PE=1 SV=2 - (CING_HUMAN)	56.62	17.79	3	1	23	53	4.326E8	1197	136.3	5.54
Q96JS0	Tubby homologue (Fragment) OS=Homo sapiens GN=TUB PE=2 SV=1 - (Q96JS0_HUMAN)	56.57	10.19	6	1	3	29	2.263E8	373	40.4	9.23
E9PDB1	Non-erythrocytic beta-spectrin 4 OS=Homo sapiens GN=SPTBN4 PE=2 SV=2 - (E9PDB1_HUMAN)	54.26	11.43	6	1	29	77	8.671E8	2564	288.9	6.06
G8JLA2	Myosin light polypeptide 6 OS=Homo sapiens GN=MYL6 PE=4 SV=1 - (G8JLA2_HUMAN)	53.60	75.66	21	7	11	27	3.004E8	152	17.1	4.55
P11021	78 kDa glucose-regulated protein OS=Homo sapiens GN=HSPA5 PE=1 SV=2 - (GRP78_HUMAN)	53.39	28.75	2	7	17	27	2.553E8	654	72.3	5.16
P49327	Fatty acid synthase OS=Homo sapiens GN=FASN PE=1 SV=3 - (FAS_HUMAN)	52.69	4.26	1	1	10	39	2.936E9	2511	273.3	6.44
Q9Y446	Plakophilin-3 OS=Homo sapiens GN=PKP3 PE=1 SV=1 - (PKP3_HUMAN)	52.53	25.85	7	7	19	48	7.712E8	797	87.0	9.32
Q9NS87-2	Isoform 2 of Kinesin-like protein KIF15 OS=Homo sapiens GN=KIF15 - (KIF15_HUMAN)	52.35	5.58	8	2	8	29	5.299E8	1291	149.7	6.05
O95447	Lebercilin-like protein OS=Homo sapiens GN=LCA5L PE=2 SV=1 - (LCA5L_HUMAN)	52.27	16.42	1	1	8	29	1.175E9	670	76.5	9.48
Q14152	Eukaryotic translation initiation factor 3 subunit A OS=Homo sapiens GN=EIF3A PE=1 SV=1 - (EIF3A_HUMAN)	50.95	25.90	8	6	37	51	2.713E8	1382	166.5	6.79
P15090	Fatty acid-binding protein, adipocyte OS=Homo sapiens GN=FABP4 PE=1 SV=3 - (FABP4_HUMAN)	50.87	52.27	4	6	8	21	1.738E8	132	14.7	7.14
Q53TK3	Putative uncharacterized protein PRO2015 OS=Homo sapiens GN=PRO2015 PE=4 SV=1 - (Q53TK3_HUMAN)	50.35	28.72	2	1	2	26	2.130E8	94	10.8	11.59
Q5T6C5	Ataxin-7-like protein 2 OS=Homo sapiens GN=ATXN7L2 PE=2 SV=1 - (AT7L2_HUMAN)	50.21	9.14	2	1	7	34	2.241E8	722	77.1	9.23
E1NZA1	Peroxisome proliferator activated receptor interacting complex protein OS=Homo sapiens GN=PRIC295 PE=2 SV=1 - (E1NZA1_HUMAN)	50.02	7.97	3	1	20	37	2.268E8	2671	292.6	7.43
Q99968	Tpr OS=Homo sapiens GN=tpr PE=2 SV=1 - (Q99968_HUMAN)	48.57	9.90	6	1	24	60	4.980E8	2363	267.2	5.03
P04075	Fructose-bisphosphate aldolase A OS=Homo sapiens GN=ALDOA PE=1 SV=2 - (ALDOA_HUMAN)	48.20	43.41	22	9	17	39	2.363E8	364	39.4	8.09
P08758	Annexin A5 OS=Homo sapiens GN=ANXA5 PE=1 SV=2 - (ANXA5_HUMAN)	47.97	44.38	12	11	15	23	1.103E8	320	35.9	5.05
Q8WXH0	Nesprin-2 OS=Homo sapiens GN=SYNE2 PE=1 SV=3 - (SYNE2_HUMAN)	47.39	8.03	9	1	53	93	8.319E8	6885	795.9	5.36
Q99996-5	Isoform 5 of A-kinase anchor protein 9 OS=Homo sapiens GN=AKAP9 - (AKAP9_HUMAN)	47.10	9.15	13	2	32	49	3.116E8	3857	447.5	4.98
Q9NRC6	Spectrin beta chain, brain 4 OS=Homo sapiens GN=SPTBN5 PE=1 SV=1 - (SPTN5_HUMAN)	47.04	9.09	1	1	34	61	2.215E9	3674	416.6	6.67
Q8IWJ2	GRIP and coiled-coil domain-containing protein 2 OS=Homo sapiens GN=GCC2 PE=1 SV=4 - (GCC2_HUMAN)	47.02	10.27	2	1	18	42	4.602E8	1684	195.8	5.14
H3BLZ6	Sterol regulatory element-binding protein 1 (Fragment) OS=Homo sapiens GN=SREBF1 PE=4 SV=1 - (H3BLZ6_HUMAN)	46.98	11.43	13	1	16	75	4.072E9	1155	122.3	8.88
P22626-2	Isoform A2 of Heterogeneous nuclear ribonucleoproteins A2/B1 OS=Homo sapiens GN=HNRNPA2B1 - (ROA2_HUMAN)	46.23	30.21	2	6	10	29	1.732E8	341	36.0	8.65
Q92556	Engulfment and cell motility protein 1 OS=Homo sapiens GN=ELMO1 PE=1 SV=2 - (ELMO1_HUMAN)	46.21	6.74	5	1	6	29	1.414E8	727	83.8	6.28
Q0QEN7	ATP synthase subunit beta (Fragment) OS=Homo sapiens GN=ATP5B PE=2 SV=1 - (Q0QEN7_HUMAN)	46.17	28.99	8	7	10	19	1.308E8	445	48.1	5.07
Q8NGB0	Seven transmembrane helix receptor OS=Homo sapiens GN=GPR142 PE=2 SV=1 - (Q8NGB0_HUMAN)	45.36	9.22	1	1	13	37	1.044E9	1464	156.4	10.95
Q5T7N8	Protein FAM27D1 OS=Homo sapiens GN=FAM27D1 PE=3 SV=2 - (F27D1_HUMAN)	44.93	27.44	5	1	8	30	1.243E8	215	24.9	11.93
Q5CAQ5	Tumor rejection antigen (Gp96) 1 OS=Homo sapiens GN=TRA1 PE=2 SV=1 - (Q5CAQ5_HUMAN)	44.65	20.57	12	9	16	23	8.732E7	802	92.3	4.86
P22392-2	Isoform 3 of Nucleoside diphosphate kinase B OS=Homo sapiens GN=NME2 - (NDKB_HUMAN)	44.35	49.06	10	7	9	22	2.623E8	267	30.1	8.92
B2RDE1	cDNA, FLJ96568, highly similar to Homo sapiens tropomyosin 3 (TPM3), mRNA OS=Homo sapiens PE=2 SV=1 - (B2RDE1_HUMAN)	44.19	46.37	40	6	15	35	2.688E8	248	29.0	4.75
B4DHC2	cDNA FLJ56434, highly similar to p130Cas-associated protein OS=Homo sapiens PE=2 SV=1 - (B4DHC2_HUMAN)	44.02	13.33	1	1	12	49	1.132E9	1035	111.0	9.41
O00362	Putative p150 OS=Homo sapiens PE=4 SV=1 - (O00362_HUMAN)	43.58	9.49	20	1	13	43	4.312E8	1275	149.1	9.69
B2RTY4-4	Isoform 4 of Unconventional myosin-IXa OS=Homo sapiens GN=MYO9A - (MYO9A_HUMAN)	43.49	10.04	8	1	24	48	6.202E8	2619	300.2	8.75
Q32MZ4	Leucine-rich repeat flightless-interacting protein 1 OS=Homo sapiens GN=LRRFIP1 PE=1 SV=2 - (LRRF1_HUMAN)	42.90	15.59	8	1	11	50	8.192E8	808	89.2	4.65
Q6ZQQ6	WD repeat-containing protein 87 OS=Homo sapiens GN=WDR87 PE=2 SV=3 - (WDR87_HUMAN)	42.80	9.36	3	1	28	47	2.510E8	2873	333.0	7.28
C9J9K3	40S ribosomal protein SA (Fragment) OS=Homo sapiens GN=RPSA PE=3 SV=1 - (C9J9K3_HUMAN)	42.63	20.83	11	5	5	16	1.152E8	264	29.5	5.25
Q2VIP3	PABP3 (Fragment) OS=Homo sapiens PE=2 SV=1 - (Q2VIP3_HUMAN)	42.46	18.57	5	1	10	41	4.272E8	630	69.9	9.67
A6NCW0	Ubiquitin carboxyl-terminal hydrolase 17-like protein 3 OS=Homo sapiens GN=USP17L3 PE=3 SV=1 - (U17L3_HUMAN)	42.05	7.55	16	1	5	50	3.933E9	530	59.5	7.74
P58107	Epiplakin OS=Homo sapiens GN=EPPK1 PE=1 SV=2 - (EPIPL_HUMAN)	42.01	19.00	3	4	23	55	4.224E8	5090	555.3	5.60
A8MTN3	Putative keratin-associated protein ENSP00000381495 OS=Homo sapiens PE=3 SV=2 - (KRA2X_HUMAN)	41.57	26.83	7	2	2	23	1.281E9	123	12.9	7.81
B7Z4T3	cDNA FLJ51903, highly similar to Stress-70 protein, mitochondrial OS=Homo sapiens PE=2 SV=1 - (B7Z4T3_HUMAN)	41.35	18.51	8	1	10	28	9.744E8	632	69.9	6.34
O60486	Plexin-C1 OS=Homo sapiens GN=PLXNC1 PE=1 SV=1 - (PLXC1_HUMAN)	41.19	3.44	1	1	5	28	5.757E8	1568	175.6	7.61
Q6ZS99	cDNA FLJ45706 fis, clone FEBRA2028457, highly similar to Nucleolin OS=Homo sapiens PE=2 SV=1 - (Q6ZS99_HUMAN)	40.89	22.55	7	5	15	33	3.481E8	603	65.9	4.55
Q96Q15	Serine/threonine-protein kinase SMG1 OS=Homo sapiens GN=SMG1 PE=1 SV=3 - (SMG1_HUMAN)	40.56	6.34	5	1	21	35	6.014E8	3661	410.2	6.46
P31151	Protein S100-A7 OS=Homo sapiens GN=S100A7 PE=1 SV=4 - (S10A7_HUMAN)	40.33	48.51	2	5	5	17	4.345E7	101	11.5	6.77
P81605	Dermcidin OS=Homo sapiens GN=DCD PE=1 SV=2 - (DCD_HUMAN)	39.96	34.55	2	2	4	17	6.544E7	110	11.3	6.54
A7MCY6	TANK-binding kinase 1-binding protein 1 OS=Homo sapiens GN=TBKBP1 PE=1 SV=1 - (TBKB1_HUMAN)	39.77	8.13	4	1	6	40	3.476E8	615	67.7	5.86
F5H7F0	Protein unc-13 homolog C OS=Homo sapiens GN=UNC13C PE=4 SV=1 - (F5H7F0_HUMAN)	39.71	6.10	14	1	15	37	1.071E9	2212	250.6	5.92
P18669	Phosphoglycerate mutase 1 OS=Homo sapiens GN=PGAM1 PE=1 SV=2 - (PGAM1_HUMAN)	39.28	36.61	14	5	9	23	1.450E8	254	28.8	7.18
Q14574-2	Isoform 3B of Desmocollin-3 OS=Homo sapiens GN=DSC3 - (DSC3_HUMAN)	39.19	20.38	5	8	15	25	3.466E8	839	93.4	6.32
Q08ES8	Cell growth-inhibiting protein 34 OS=Homo sapiens PE=2 SV=1 - (Q08ES8_HUMAN)	38.90	48.02	5	3	8	22	1.460E8	177	20.1	9.60
Q96AG4	Leucine-rich repeat-containing protein 59 OS=Homo sapiens GN=LRRC59 PE=1 SV=1 - (LRC59_HUMAN)	38.14	21.50	1	1	8	28	3.157E7	307	34.9	9.57
B2R4M6	cDNA, FLJ92148, highly similar to Homo sapiens S100 calcium binding protein A9 (calgranulin B) (S100A9), mRNA OS=Homo sapiens PE=4 SV=1 - (B2R4M6_HUMAN)	37.85	51.75	2	4	7	22	1.791E8	114	13.2	6.13
Q4W4Y1	Dopamine receptor interacting protein 4 OS=Homo sapiens GN=DRIP4 PE=2 SV=1 - (Q4W4Y1_HUMAN)	37.83	12.44	7	2	12	29	2.076E8	868	96.0	6.52
O00221	NF-kappa-B inhibitor epsilon OS=Homo sapiens GN=NFKBIE PE=1 SV=3 - (IKBE_HUMAN)	37.55	7.20	6	1	3	25	2.740E7	500	52.8	6.68
Q99575	Ribonucleases P/MRP protein subunit POP1 OS=Homo sapiens GN=POP1 PE=1 SV=2 - (POP1_HUMAN)	37.52	10.06	2	1	12	40	1.206E9	1024	114.6	9.22
F5H7T3	Vinculin OS=Homo sapiens GN=VCL PE=4 SV=1 - (F5H7T3_HUMAN)	37.42	14.21	8	2	15	26	2.017E8	1006	110.2	6.34
A4QPB0	IQ motif containing GTPase activating protein 1 OS=Homo sapiens GN=IQGAP1 PE=2 SV=1 - (A4QPB0_HUMAN)	37.23	11.29	5	4	17	24	8.519E7	1657	189.2	6.48
A6H8Y1-7	Isoform 7 of Transcription factor TFIIIB component B'' homolog OS=Homo sapiens GN=BDP1 - (BDP1_HUMAN)	37.10	6.94	9	1	17	54	2.856E8	2552	286.0	5.17
P30050	60S ribosomal protein L12 OS=Homo sapiens GN=RPL12 PE=1 SV=1 - (RL12_HUMAN)	37.05	55.76	4	5	9	14	1.028E8	165	17.8	9.42
Q9P219	Protein Daple OS=Homo sapiens GN=CCDC88C PE=1 SV=3 - (DAPLE_HUMAN)	36.73	10.11	1	1	19	30	1.327E9	2028	228.1	6.23
P62277	40S ribosomal protein S13 OS=Homo sapiens GN=RPS13 PE=1 SV=2 - (RS13_HUMAN)	36.54	50.99	2	6	9	18	1.339E8	151	17.2	10.54
B4DXS5	cDNA FLJ57802, highly similar to Zinc finger protein 225 OS=Homo sapiens PE=2 SV=1 - (B4DXS5_HUMAN)	36.52	10.30	1	1	8	30	4.130E8	670	78.1	8.92
P98164	Low-density lipoprotein receptor-related protein 2 OS=Homo sapiens GN=LRP2 PE=1 SV=3 - (LRP2_HUMAN)	36.45	4.68	4	1	21	40	2.811E8	4655	521.6	5.08
E9PK25	Cofilin-1 OS=Homo sapiens GN=CFL1 PE=4 SV=1 - (E9PK25_HUMAN)	36.31	45.10	10	5	7	14	4.078E8	204	22.7	8.34
E1U1N1	Ubiquitously transcribed tetratricopeptide repeat protein Y-linked transcript variant 252 OS=Homo sapiens GN=UTY PE=2 SV=1 - (E1U1N1_HUMAN)	35.87	5.75	85	1	6	35	3.839E9	887	96.9	7.47
Q9H694	Protein bicaudal C homolog 1 OS=Homo sapiens GN=BICC1 PE=1 SV=2 - (BICC1_HUMAN)	35.75	7.70	3	1	7	37	5.436E7	974	104.8	8.54
F5GX82	Protein furry homolog-like OS=Homo sapiens GN=FRYL PE=4 SV=1 - (F5GX82_HUMAN)	35.67	3.15	5	1	13	33	1.103E9	3013	339.4	5.58
P51587	Breast cancer type 2 susceptibility protein OS=Homo sapiens GN=BRCA2 PE=1 SV=2 - (BRCA2_HUMAN)	34.91	5.41	4	1	17	45	7.629E8	3418	384.0	6.73
Q9P2D1	Chromodomain-helicase-DNA-binding protein 7 OS=Homo sapiens GN=CHD7 PE=1 SV=3 - (CHD7_HUMAN)	34.52	6.57	2	1	18	34	3.012E8	2997	335.7	6.34
Q5U5K4	Homer homolog 1 (Drosophila) OS=Homo sapiens GN=HOMER1 PE=2 SV=1 - (Q5U5K4_HUMAN)	34.47	13.56	5	2	5	33	1.506E8	354	40.3	5.44
Q4L180-2	Isoform 2 of Filamin A-interacting protein 1-like OS=Homo sapiens GN=FILIP1L - (FIL1L_HUMAN)	34.33	8.91	7	1	8	25	2.118E9	1133	130.1	6.61
Q5BJF6	Outer dense fiber protein 2 OS=Homo sapiens GN=ODF2 PE=1 SV=1 - (ODFP2_HUMAN)	33.87	14.60	6	1	14	40	7.639E8	829	95.3	7.62
P26641	Elongation factor 1-gamma OS=Homo sapiens GN=EEF1G PE=1 SV=3 - (EF1G_HUMAN)	33.74	12.13	6	4	6	20	5.233E7	437	50.1	6.67
Q86SG7	Lysozyme g-like protein 2 OS=Homo sapiens GN=LYG2 PE=1 SV=2 - (LYG2_HUMAN)	33.61	38.21	4	7	8	17	3.039E7	212	23.5	8.87
O75182-2	Isoform 2 of Paired amphipathic helix protein Sin3b OS=Homo sapiens GN=SIN3B - (SIN3B_HUMAN)	33.36	4.78	3	1	7	49	1.375E8	1130	129.3	6.98
Q5HYW3	Retrotransposon gag domain-containing protein 4 OS=Homo sapiens GN=RGAG4 PE=2 SV=1 - (RGAG4_HUMAN)	33.24	10.54	1	1	6	26	2.971E8	569	64.7	4.75
P63244	Guanine nucleotide-binding protein subunit beta-2-like 1 OS=Homo sapiens GN=GNB2L1 PE=1 SV=3 - (GBLP_HUMAN)	33.06	38.80	24	10	11	15	1.550E8	317	35.1	7.69
Q92833	Protein Jumonji OS=Homo sapiens GN=JARID2 PE=1 SV=2 - (JARD2_HUMAN)	32.88	8.67	2	1	13	30	3.738E8	1246	138.6	9.38
P16401	Histone H1.5 OS=Homo sapiens GN=HIST1H1B PE=1 SV=3 - (H15_HUMAN)	32.87	22.57	1	2	8	20	2.723E8	226	22.6	10.92
Q9HBI0-3	Isoform 3 of Gamma-parvin OS=Homo sapiens GN=PARVG - (PARVG_HUMAN)	32.75	7.49	3	1	1	15	3.794E8	187	21.4	5.47
P62249	40S ribosomal protein S16 OS=Homo sapiens GN=RPS16 PE=1 SV=2 - (RS16_HUMAN)	32.62	44.52	3	6	7	15	8.141E8	146	16.4	10.21
Q15365	Poly(rC)-binding protein 1 OS=Homo sapiens GN=PCBP1 PE=1 SV=2 - (PCBP1_HUMAN)	32.46	20.22	10	5	7	16	1.503E8	356	37.5	7.09
P07237	Protein disulfide-isomerase OS=Homo sapiens GN=P4HB PE=1 SV=3 - (PDIA1_HUMAN)	32.46	19.29	20	5	13	24	5.178E7	508	57.1	4.87
H0Y2N2	Ribonuclease 3 OS=Homo sapiens GN=DROSHA PE=4 SV=1 - (H0Y2N2_HUMAN)	32.42	9.08	7	1	8	29	7.643E8	1299	150.4	7.65
E9PCF4	Kelch-like protein 5 OS=Homo sapiens GN=KLHL5 PE=4 SV=1 - (E9PCF4_HUMAN)	32.41	5.99	10	1	3	47	8.313E7	568	63.5	6.98
Q86SJ6	Desmoglein-4 OS=Homo sapiens GN=DSG4 PE=1 SV=1 - (DSG4_HUMAN)	32.40	9.23	2	5	10	21	6.613E7	1040	113.8	4.56
P62269	40S ribosomal protein S18 OS=Homo sapiens GN=RPS18 PE=1 SV=3 - (RS18_HUMAN)	32.33	45.39	2	8	11	18	2.240E8	152	17.7	10.99
A0PK11	Clarin-2 OS=Homo sapiens GN=CLRN2 PE=2 SV=1 - (CLRN2_HUMAN)	31.91	3.02	1	1	1	25	4.732E8	232	25.4	6.99
Q04656-5	Isoform 5 of Copper-transporting ATPase 1 OS=Homo sapiens GN=ATP7A - (ATP7A_HUMAN)	31.82	3.66	6	1	5	24	1.541E9	1422	154.3	6.05
Q68CX2	Putative uncharacterized protein DKFZp761A1822 OS=Homo sapiens GN=DKFZp761A1822 PE=2 SV=2 - (Q68CX2_HUMAN)	31.79	4.74	1	1	1	21	2.151E8	211	22.7	7.46
Q71RH4	FP1047 OS=Homo sapiens PE=2 SV=1 - (Q71RH4_HUMAN)	31.30	19.46	29	5	11	19	1.512E9	632	69.4	7.21
Q96QE3	ATPase family AAA domain-containing protein 5 OS=Homo sapiens GN=ATAD5 PE=1 SV=4 - (ATAD5_HUMAN)	31.14	15.46	3	1	25	158	4.233E9	1844	207.4	9.19
Q9BTQ7	Similar to ribosomal protein L23 (Fragment) OS=Homo sapiens PE=1 SV=1 - (Q9BTQ7_HUMAN)	30.96	49.25	4	7	7	9	1.201E8	134	14.1	10.26
B2R717	cDNA, FLJ93235, highly similar to Homo sapiens cathepsin L2 (CTSL2), mRNA OS=Homo sapiens PE=2 SV=1 - (B2R717_HUMAN)	30.80	19.46	2	5	6	14	9.967E7	334	37.3	8.76
B2R5W2	cDNA, FLJ92657, highly similar to Homo sapiens heterogeneous nuclear ribonucleoprotein C (C1/C2) (HNRPC), transcript variant 2, mRNA OS=Homo sapiens PE=2 SV=1 - (B2R5W2_HUMAN)	30.75	23.79	33	4	8	20	2.273E8	290	31.9	5.24
Q13228	Selenium-binding protein 1 OS=Homo sapiens GN=SELENBP1 PE=1 SV=2 - (SBP1_HUMAN)	30.71	20.76	14	7	9	21	2.092E8	472	52.4	6.37
Q5JR95	40S ribosomal protein S8 OS=Homo sapiens GN=RPS8 PE=4 SV=1 - (Q5JR95_HUMAN)	30.70	56.38	3	7	10	15	1.513E8	188	21.9	10.36
Q8NI35	InaD-like protein OS=Homo sapiens GN=INADL PE=1 SV=3 - (INADL_HUMAN)	30.57	5.44	8	1	9	24	2.535E8	1801	196.2	4.94
Q96IV0-3	Isoform 3 of Peptide-N(4)-(N-acetyl-beta-glucosaminyl)asparagine amidase OS=Homo sapiens GN=NGLY1 - (NGLY1_HUMAN)	30.56	9.14	4	1	6	21	9.063E8	558	63.8	8.10
P09651-3	Isoform 2 of Heterogeneous nuclear ribonucleoprotein A1 OS=Homo sapiens GN=HNRNPA1 - (ROA1_HUMAN)	30.33	29.59	19	3	7	16	5.053E8	267	29.4	9.14
Q5VYY1	Ankyrin repeat domain-containing protein 22 OS=Homo sapiens GN=ANKRD22 PE=2 SV=1 - (ANR22_HUMAN)	30.27	20.94	1	2	6	26	2.390E8	191	21.8	8.84
O75145-2	Isoform 2 of Liprin-alpha-3 OS=Homo sapiens GN=PPFIA3 - (LIPA3_HUMAN)	30.06	13.50	3	2	16	32	5.115E8	1185	132.3	5.74
Q9UKN7	Unconventional myosin-XV OS=Homo sapiens GN=MYO15A PE=1 SV=2 - (MYO15_HUMAN)	29.91	8.10	1	1	23	43	6.776E8	3530	395.0	9.17
P29034	Protein S100-A2 OS=Homo sapiens GN=S100A2 PE=1 SV=3 - (S10A2_HUMAN)	29.84	36.73	2	7	7	17	4.445E8	98	11.1	4.78
P50395	Rab GDP dissociation inhibitor beta OS=Homo sapiens GN=GDI2 PE=1 SV=2 - (GDIB_HUMAN)	29.81	18.20	14	5	6	9	4.037E7	445	50.6	6.47
Q96IU2	Zinc finger BED domain-containing protein 3 OS=Homo sapiens GN=ZBED3 PE=1 SV=1 - (ZBED3_HUMAN)	29.76	19.66	2	1	5	19	3.849E8	234	25.1	8.31
P62851	40S ribosomal protein S25 OS=Homo sapiens GN=RPS25 PE=1 SV=1 - (RS25_HUMAN)	29.68	46.40	1	5	8	15	3.813E8	125	13.7	10.11
C9JNW5	60S ribosomal protein L24 OS=Homo sapiens GN=RPL24 PE=4 SV=1 - (C9JNW5_HUMAN)	29.50	50.67	3	6	10	17	1.635E9	150	17.5	10.78
Q8NEZ4	Histone-lysine N-methyltransferase MLL3 OS=Homo sapiens GN=MLL3 PE=1 SV=3 - (MLL3_HUMAN)	29.48	3.97	6	1	19	26	6.461E7	4911	541.0	6.49
Q9NZM3-3	Isoform 3 of Intersectin-2 OS=Homo sapiens GN=ITSN2 - (ITSN2_HUMAN)	29.44	11.05	10	1	14	32	5.639E8	1249	141.7	7.75
Q6DT37	Serine/threonine-protein kinase MRCK gamma OS=Homo sapiens GN=CDC42BPG PE=1 SV=2 - (MRCKG_HUMAN)	29.29	6.38	1	1	10	29	2.561E8	1551	172.4	6.28
P13928	Annexin A8 OS=Homo sapiens GN=ANXA8 PE=1 SV=3 - (ANXA8_HUMAN)	29.19	29.97	15	6	12	19	1.061E8	327	36.9	5.78
O95759	TBC1 domain family member 8 OS=Homo sapiens GN=TBC1D8 PE=1 SV=3 - (TBCD8_HUMAN)	28.99	6.84	2	1	8	31	8.470E8	1140	130.8	5.52
P16152	Carbonyl reductase (NADPH) 1 OS=Homo sapiens GN=CBR1 PE=1 SV=3 - (CBR1_HUMAN)	28.94	34.66	4	6	9	16	1.904E8	277	30.4	8.32
E7ETZ4	Basic leucine zipper and W2 domain-containing protein 2 (Fragment) OS=Homo sapiens GN=BZW2 PE=4 SV=1 - (E7ETZ4_HUMAN)	28.73	8.58	8	1	4	19	1.820E8	408	46.9	8.44
Q5VU13	V-set and immunoglobulin domain-containing protein 8 OS=Homo sapiens GN=VSIG8 PE=1 SV=1 - (VSIG8_HUMAN)	28.70	18.36	1	5	6	20	4.734E7	414	43.9	7.20
Q96QA5	Gasdermin-A OS=Homo sapiens GN=GSDMA PE=2 SV=4 - (GSDMA_HUMAN)	28.67	19.55	1	4	7	13	2.074E8	445	49.3	5.29
F5H7C4	Methionine-R-sulfoxide reductase B3 (Fragment) OS=Homo sapiens GN=MSRB3 PE=4 SV=1 - (F5H7C4_HUMAN)	28.39	7.69	6	1	1	11	8.816E9	117	13.1	7.78
E9PC74	Translation initiation factor eIF-2B subunit epsilon OS=Homo sapiens GN=EIF2B5 PE=4 SV=1 - (E9PC74_HUMAN)	28.32	13.05	4	1	5	21	3.281E8	705	78.3	5.21
Q8NGN2	Olfactory receptor 10S1 OS=Homo sapiens GN=OR10S1 PE=2 SV=2 - (O10S1_HUMAN)	28.04	9.06	1	1	3	22	2.092E9	331	36.5	8.12
P04080	Cystatin-B OS=Homo sapiens GN=CSTB PE=1 SV=2 - (CYTB_HUMAN)	27.75	61.22	1	4	5	14	9.568E7	98	11.1	7.56
Q8WVX7	Ribosomal protein S19 (Fragment) OS=Homo sapiens PE=2 SV=1 - (Q8WVX7_HUMAN)	27.67	71.97	2	8	16	28	1.709E8	157	17.3	10.52
P62857	40S ribosomal protein S28 OS=Homo sapiens GN=RPS28 PE=1 SV=1 - (RS28_HUMAN)	27.59	52.17	1	4	4	12	5.538E7	69	7.8	10.70
Q9ULL8-2	Isoform 2 of Protein Shroom4 OS=Homo sapiens GN=SHROOM4 - (SHRM4_HUMAN)	27.55	12.85	2	1	15	30	1.492E9	1377	152.1	6.47
B4DLZ5	N-acetylglucosamine kinase OS=Homo sapiens GN=NAGK PE=2 SV=1 - (B4DLZ5_HUMAN)	27.35	28.97	10	9	11	20	6.010E7	390	42.0	6.68
Q9UJ55	MAGE-like protein 2 OS=Homo sapiens GN=MAGEL2 PE=1 SV=1 - (MAGL2_HUMAN)	27.34	6.43	5	1	2	14	8.215E7	529	58.6	7.99
Q17RC7	Exocyst complex component 3-like protein 4 OS=Homo sapiens GN=EXOC3L4 PE=2 SV=2 - (EX3L4_HUMAN)	27.29	8.59	1	2	7	25	6.130E8	722	79.8	6.32
Q66LE6	Serine/threonine-protein phosphatase 2A 55 kDa regulatory subunit B delta isoform OS=Homo sapiens GN=PPP2R2D PE=1 SV=1 - (2ABD_HUMAN)	27.21	11.70	17	2	5	14	4.968E8	453	52.0	6.39
D6RAD4	Cyclin-dependent kinase 7 OS=Homo sapiens GN=CDK7 PE=4 SV=1 - (D6RAD4_HUMAN)	27.20	7.44	3	1	4	11	1.400E8	309	34.6	8.28
Q9BYR8	Keratin-associated protein 3-1 OS=Homo sapiens GN=KRTAP3-1 PE=1 SV=1 - (KRA31_HUMAN)	27.10	24.49	1	2	2	9	3.454E8	98	10.5	6.40
Q8N3C0	Activating signal cointegrator 1 complex subunit 3 OS=Homo sapiens GN=ASCC3 PE=1 SV=3 - (ASCC3_HUMAN)	26.99	6.36	1	1	12	40	1.597E9	2202	251.3	7.09
O60282	Kinesin heavy chain isoform 5C OS=Homo sapiens GN=KIF5C PE=1 SV=1 - (KIF5C_HUMAN)	26.91	15.46	19	1	14	24	8.045E8	957	109.4	6.19
F8WD26	LIM domain only protein 7 OS=Homo sapiens GN=LMO7 PE=4 SV=1 - (F8WD26_HUMAN)	26.56	15.30	25	1	26	50	6.065E8	1614	185.0	7.97
A2ABS5	Dimethylarginine dimethylaminohydrolase 2 (Fragment) OS=Homo sapiens GN=DDAH2 PE=4 SV=1 - (A2ABS5_HUMAN)	26.56	11.23	6	1	2	28	4.428E9	187	19.9	8.09
Q8N264	Rho GTPase-activating protein 24 OS=Homo sapiens GN=ARHGAP24 PE=1 SV=2 - (RHG24_HUMAN)	26.44	10.03	7	2	8	19	1.737E8	748	84.2	6.67
B2RCA2	cDNA, FLJ95932, Homo sapiens polyamine modulated factor 1 binding protein 1(PMFBP1), mRNA OS=Homo sapiens PE=2 SV=1 - (B2RCA2_HUMAN)	26.21	13.01	27	1	17	43	5.760E8	1007	117.4	6.25
O43497-5	Isoform 4 of Voltage-dependent T-type calcium channel subunit alpha-1G OS=Homo sapiens GN=CACNA1G - (CAC1G_HUMAN)	26.15	4.79	35	1	11	26	8.528E7	2171	240.8	7.08
H3BTF6	Uncharacterized protein C16orf59 (Fragment) OS=Homo sapiens GN=C16orf59 PE=4 SV=1 - (H3BTF6_HUMAN)	26.04	16.84	7	1	5	15	8.881E8	285	30.4	9.88
Q8IYB4-2	Isoform 2 of PEX5-related protein OS=Homo sapiens GN=PEX5L - (PEX5R_HUMAN)	25.83	10.26	15	1	6	17	9.638E7	624	69.4	5.59
O75629	Protein CREG1 OS=Homo sapiens GN=CREG1 PE=1 SV=1 - (CREG1_HUMAN)	25.69	16.82	2	2	3	18	1.144E8	220	24.1	7.59
B3W5Y6	Squamous cell carcinoma antigen-1 isoform SCCA-PD OS=Homo sapiens GN=SERPINB3 PE=2 SV=1 - (B3W5Y6_HUMAN)	25.65	26.92	5	1	9	22	6.411E7	390	44.6	6.81
Q149N8-4	Isoform 3 of E3 ubiquitin-protein ligase SHPRH OS=Homo sapiens GN=SHPRH - (SHPRH_HUMAN)	25.59	9.76	3	1	14	28	1.820E9	1659	190.4	7.62
Q86VG2	Splicing factor proline/glutamine-rich (Polypyrimidine tract binding protein associated) OS=Homo sapiens GN=SFPQ PE=2 SV=1 - (Q86VG2_HUMAN)	25.55	17.26	5	2	14	26	2.229E8	707	76.1	9.48
A8K092	ATP synthase subunit alpha OS=Homo sapiens GN=ATP5A1 PE=2 SV=1 - (A8K092_HUMAN)	25.50	21.67	4	5	9	11	8.607E7	503	54.5	8.24
Q13515	Phakinin OS=Homo sapiens GN=BFSP2 PE=1 SV=1 - (BFSP2_HUMAN)	25.49	11.33	1	1	5	13	9.755E7	415	45.9	5.55
P01024	Complement C3 OS=Homo sapiens GN=C3 PE=1 SV=2 - (CO3_HUMAN)	25.43	8.00	2	1	17	26	1.161E8	1663	187.0	6.40
Q5T7W0-2	Isoform 2 of Zinc finger protein 618 OS=Homo sapiens GN=ZNF618 - (ZN618_HUMAN)	25.33	4.18	3	1	5	24	9.047E7	861	95.6	7.12
Q9BTI9	NPM1 protein (Fragment) OS=Homo sapiens GN=NPM1 PE=2 SV=2 - (Q9BTI9_HUMAN)	25.30	28.95	8	4	6	23	4.998E7	228	25.0	4.86
B4DLR3	cDNA FLJ54020, highly similar to Heterogeneous nuclear ribonucleoprotein U OS=Homo sapiens PE=2 SV=1 - (B4DLR3_HUMAN)	25.25	13.78	8	1	12	36	6.901E8	784	86.8	6.43
B3KS79	cDNA FLJ35730 fis, clone TESTI2003131, highly similar to ALPHA-1-ANTICHYMOTRYPSIN OS=Homo sapiens PE=2 SV=1 - (B3KS79_HUMAN)	25.03	14.73	4	4	6	13	1.471E8	448	50.6	5.63
B3KVD6	cDNA FLJ16433 fis, clone BRACE3013936, highly similar to Cell death activator CIDE-B OS=Homo sapiens PE=2 SV=1 - (B3KVD6_HUMAN)	25.02	3.20	2	1	1	26	8.751E8	219	24.8	9.03
Q5K634	SCCA2/SCCA1 fusion protein isoform 1 OS=Homo sapiens PE=2 SV=1 - (Q5K634_HUMAN)	24.98	15.64	13	1	7	20	5.998E7	390	44.6	6.39
Q00610-2	Isoform 2 of Clathrin heavy chain 1 OS=Homo sapiens GN=CLTC - (CLH1_HUMAN)	24.92	8.79	2	3	13	24	3.413E8	1639	187.8	5.69
O60930	Ribonuclease H1 OS=Homo sapiens GN=RNASEH1 PE=1 SV=2 - (RNH1_HUMAN)	24.82	12.94	2	1	4	19	1.250E8	286	32.0	9.16
P12236	ADP/ATP translocase 3 OS=Homo sapiens GN=SLC25A6 PE=1 SV=4 - (ADT3_HUMAN)	24.73	25.84	9	3	8	17	3.311E8	298	32.8	9.74
E9PBB0	PERQ amino acid-rich with GYF domain-containing protein 2 OS=Homo sapiens GN=GIGYF2 PE=4 SV=1 - (E9PBB0_HUMAN)	24.44	15.00	5	1	21	29	3.452E8	1293	149.4	5.54
Q71UH4	DNA topoisomerase 2 (Fragment) OS=Homo sapiens GN=TOP2B PE=2 SV=1 - (Q71UH4_HUMAN)	24.38	6.82	9	1	14	46	2.042E9	1598	180.5	7.94
P09467	Fructose-1,6-bisphosphatase 1 OS=Homo sapiens GN=FBP1 PE=1 SV=5 - (F16P1_HUMAN)	24.23	11.83	4	2	3	7	3.289E7	338	36.8	6.99
P45880-2	Isoform 2 of Voltage-dependent anion-selective channel protein 2 OS=Homo sapiens GN=VDAC2 - (VDAC2_HUMAN)	24.18	25.44	7	5	7	17	7.265E7	283	30.4	7.20
Q9NYJ1	Coiled-coil-helix-coiled-coil-helix domain-containing protein 8 OS=Homo sapiens GN=CHCHD8 PE=1 SV=2 - (CHCH8_HUMAN)	24.16	31.03	2	1	4	15	1.536E8	87	10.1	6.04
B3KMF7	cDNA FLJ10863 fis, clone NT2RP4001575, highly similar to Vacuolar protein sorting protein 52 OS=Homo sapiens PE=2 SV=1 - (B3KMF7_HUMAN)	24.06	9.18	1	1	4	17	1.294E8	534	61.3	8.60
E7EQG2	Eukaryotic initiation factor 4A-II OS=Homo sapiens GN=EIF4A2 PE=3 SV=1 - (E7EQG2_HUMAN)	23.95	13.54	14	2	5	11	3.034E7	362	41.3	5.64
P52272-2	Isoform 2 of Heterogeneous nuclear ribonucleoprotein M OS=Homo sapiens GN=HNRNPM - (HNRPM_HUMAN)	23.60	28.94	7	4	18	26	5.537E8	691	73.6	8.82
B4DSG0	Janus kinase and microtubule-interacting protein 2 OS=Homo sapiens GN=JAKMIP2 PE=2 SV=1 - (B4DSG0_HUMAN)	23.55	10.15	3	1	10	62	3.667E8	778	91.5	5.78
Q3LIE3	Putative uncharacterized protein Nbla04137 (Fragment) OS=Homo sapiens GN=Nbla04137 PE=2 SV=1 - (Q3LIE3_HUMAN)	23.46	10.73	1	1	2	17	1.702E8	289	33.3	5.21
B3KX11	cDNA FLJ44436 fis, clone UTERU2019706, highly similar to T-complex protein 1 subunit gamma OS=Homo sapiens PE=2 SV=1 - (B3KX11_HUMAN)	23.44	14.75	7	2	9	18	8.300E7	522	57.9	6.93
P05090	Apolipoprotein D OS=Homo sapiens GN=APOD PE=1 SV=1 - (APOD_HUMAN)	23.40	30.16	4	5	5	11	8.623E7	189	21.3	5.15
Q9HCY8	Protein S100-A14 OS=Homo sapiens GN=S100A14 PE=1 SV=1 - (S10AE_HUMAN)	23.37	52.88	1	5	5	8	8.997E7	104	11.7	5.24
H0YFF8	UPF0606 protein C11orf41 OS=Homo sapiens GN=C11orf41 PE=4 SV=1 - (H0YFF8_HUMAN)	23.32	3.50	6	1	5	18	2.164E8	1688	182.6	8.88
Q9H7E2-3	Isoform 3 of Tudor domain-containing protein 3 OS=Homo sapiens GN=TDRD3 - (TDRD3_HUMAN)	23.23	9.14	3	1	8	79	1.778E8	744	83.1	9.00
P10599	Thioredoxin OS=Homo sapiens GN=TXN PE=1 SV=3 - (THIO_HUMAN)	23.15	54.29	3	4	8	16	1.312E8	105	11.7	4.92
P29373	Cellular retinoic acid-binding protein 2 OS=Homo sapiens GN=CRABP2 PE=1 SV=2 - (RABP2_HUMAN)	23.06	47.83	2	3	6	15	1.471E8	138	15.7	5.40
Q9Y6R9	Coiled-coil domain-containing protein 61 OS=Homo sapiens GN=CCDC61 PE=1 SV=2 - (CCD61_HUMAN)	23.04	21.10	1	1	12	25	5.447E8	474	53.2	9.95
P25787	Proteasome subunit alpha type-2 OS=Homo sapiens GN=PSMA2 PE=1 SV=2 - (PSA2_HUMAN)	22.85	30.77	5	3	5	8	7.419E7	234	25.9	7.43
P39023	60S ribosomal protein L3 OS=Homo sapiens GN=RPL3 PE=1 SV=2 - (RL3_HUMAN)	22.78	22.08	13	2	11	19	2.851E8	403	46.1	10.18
P31944	Caspase-14 OS=Homo sapiens GN=CASP14 PE=1 SV=2 - (CASPE_HUMAN)	22.78	23.97	2	3	7	16	1.351E8	242	27.7	5.58
A6NGS5	Laminin subunit alpha-2 OS=Homo sapiens GN=LAMA2 PE=4 SV=2 - (A6NGS5_HUMAN)	22.77	6.69	5	1	18	41	5.696E8	3122	343.8	6.40
B4DL86	6-phosphogluconate dehydrogenase, decarboxylating OS=Homo sapiens PE=2 SV=1 - (B4DL86_HUMAN)	22.37	15.85	6	1	5	14	1.468E8	429	47.4	6.47
P0CG31	Putative zinc finger protein 286B OS=Homo sapiens GN=ZNF286B PE=5 SV=1 - (Z286B_HUMAN)	22.25	10.73	5	1	5	15	5.850E8	522	59.5	8.35
Q8TDW7-3	Isoform 3 of Protocadherin Fat 3 OS=Homo sapiens GN=FAT3 - (FAT3_HUMAN)	22.18	2.15	3	1	9	21	2.280E8	4557	501.7	4.87
B4DSU0	cDNA FLJ61379, highly similar to Sterol regulatory element-binding protein 2 OS=Homo sapiens PE=2 SV=1 - (B4DSU0_HUMAN)	22.17	4.54	1	1	4	12	1.003E9	1102	119.5	8.37
P00918	Carbonic anhydrase 2 OS=Homo sapiens GN=CA2 PE=1 SV=2 - (CAH2_HUMAN)	22.05	20.77	3	4	4	6	4.207E7	260	29.2	7.40
F8VXI9	ARF GTPase-activating protein GIT2 OS=Homo sapiens GN=GIT2 PE=4 SV=1 - (F8VXI9_HUMAN)	22.04	7.06	1	1	4	25	2.465E8	708	78.8	7.46
Q8WXX0	Dynein heavy chain 7, axonemal OS=Homo sapiens GN=DNAH7 PE=1 SV=2 - (DYH7_HUMAN)	22.00	4.10	1	1	15	25	1.142E9	4024	460.9	6.00
A2A3R5	40S ribosomal protein S6 OS=Homo sapiens GN=RPS6 PE=3 SV=1 - (A2A3R5_HUMAN)	21.78	36.24	4	3	8	14	4.391E7	218	25.0	11.14
Q13867	Bleomycin hydrolase OS=Homo sapiens GN=BLMH PE=1 SV=1 - (BLMH_HUMAN)	21.74	10.11	3	3	4	12	4.979E7	455	52.5	6.27
Q9BVP2-2	Isoform 2 of Guanine nucleotide-binding protein-like 3 OS=Homo sapiens GN=GNL3 - (GNL3_HUMAN)	21.69	8.57	4	1	5	16	2.133E8	537	60.5	8.79
Q5JPC5	Putative uncharacterized protein DKFZp667E1714 OS=Homo sapiens GN=DKFZp667E1714 PE=2 SV=1 - (Q5JPC5_HUMAN)	21.56	12.97	4	1	7	16	2.416E8	424	49.2	8.60
Q5VTQ0-6	Isoform 6 of Tetratricopeptide repeat protein 39B OS=Homo sapiens GN=TTC39B - (TT39B_HUMAN)	21.55	5.55	7	1	3	11	1.708E8	613	69.5	7.55
Q8IY63-2	Isoform 2 of Angiomotin-like protein 1 OS=Homo sapiens GN=AMOTL1 - (AMOL1_HUMAN)	21.52	9.93	4	1	10	19	9.267E8	906	101.1	7.33
Q8N3D4	EH domain-binding protein 1-like protein 1 OS=Homo sapiens GN=EHBP1L1 PE=1 SV=2 - (EH1L1_HUMAN)	21.48	10.44	1	1	12	22	8.874E7	1523	161.8	4.83
H9KVA1	Solute carrier family 22 member 17 OS=Homo sapiens GN=SLC22A17 PE=4 SV=1 - (H9KVA1_HUMAN)	21.41	3.42	3	1	2	17	8.621E8	409	43.9	8.73
O15173	Membrane-associated progesterone receptor component 2 OS=Homo sapiens GN=PGRMC2 PE=1 SV=1 - (PGRC2_HUMAN)	21.24	6.73	3	2	2	14	9.183E8	223	23.8	4.88
B4DI38	Adenylyl cyclase-associated protein OS=Homo sapiens PE=2 SV=1 - (B4DI38_HUMAN)	21.24	26.55	18	4	8	10	3.910E7	452	49.0	8.21
P32926	Desmoglein-3 OS=Homo sapiens GN=DSG3 PE=1 SV=2 - (DSG3_HUMAN)	21.22	6.21	1	2	6	11	4.444E7	999	107.5	5.00
Q08188	Protein-glutamine gamma-glutamyltransferase E OS=Homo sapiens GN=TGM3 PE=1 SV=4 - (TGM3_HUMAN)	21.20	15.44	3	6	12	20	7.257E7	693	76.6	5.86
P06744-2	Isoform 2 of Glucose-6-phosphate isomerase OS=Homo sapiens GN=GPI - (G6PI_HUMAN)	21.08	12.83	4	2	6	23	2.941E8	569	64.3	8.91
P46783	40S ribosomal protein S10 OS=Homo sapiens GN=RPS10 PE=1 SV=1 - (RS10_HUMAN)	20.97	49.70	4	3	8	21	6.006E7	165	18.9	10.15
P80188	Neutrophil gelatinase-associated lipocalin OS=Homo sapiens GN=LCN2 PE=1 SV=2 - (NGAL_HUMAN)	20.95	34.85	4	4	6	10	6.721E7	198	22.6	8.91
Q99714	3-hydroxyacyl-CoA dehydrogenase type-2 OS=Homo sapiens GN=HSD17B10 PE=1 SV=3 - (HCD2_HUMAN)	20.95	31.42	3	3	5	10	4.305E7	261	26.9	7.78
E7ENM8	Tyrosine-protein kinase Fes/Fps OS=Homo sapiens GN=FES PE=3 SV=1 - (E7ENM8_HUMAN)	20.90	7.64	8	1	4	15	8.159E7	681	76.8	6.71
E9PL10	Transcription factor BTF3 homolog 4 OS=Homo sapiens GN=BTF3L4 PE=4 SV=1 - (E9PL10_HUMAN)	20.82	17.93	7	1	4	14	1.527E8	145	15.8	9.14
Q08999	Retinoblastoma-like protein 2 OS=Homo sapiens GN=RBL2 PE=1 SV=3 - (RBL2_HUMAN)	20.82	8.34	4	1	11	22	1.641E8	1139	128.3	7.44
Q96BY7	Autophagy-related protein 2 homolog B OS=Homo sapiens GN=ATG2B PE=1 SV=5 - (ATG2B_HUMAN)	20.66	7.46	1	1	13	26	2.259E8	2078	232.6	5.76
C9J5W6	Transgelin-3 OS=Homo sapiens GN=TAGLN3 PE=4 SV=1 - (C9J5W6_HUMAN)	20.62	25.22	4	1	4	10	6.088E7	115	12.9	9.19
P02649	Apolipoprotein E OS=Homo sapiens GN=APOE PE=1 SV=1 - (APOE_HUMAN)	20.58	33.75	8	4	15	19	1.005E8	317	36.1	5.73
A8MXP9	Matrin-3 OS=Homo sapiens GN=MATR3 PE=4 SV=1 - (A8MXP9_HUMAN)	20.57	15.87	12	1	20	36	3.681E8	895	99.9	6.04
Q17RV3	Leucine-rich repeat kinase 2 OS=Homo sapiens GN=LRRK2 PE=2 SV=1 - (Q17RV3_HUMAN)	20.50	6.05	2	1	14	38	5.659E8	2527	285.9	6.79
P12532	Creatine kinase U-type, mitochondrial OS=Homo sapiens GN=CKMT1A PE=1 SV=1 - (KCRU_HUMAN)	20.46	14.15	12	1	8	15	6.942E7	417	47.0	8.34
Q7Z3D4	LysM and putative peptidoglycan-binding domain-containing protein 3 OS=Homo sapiens GN=LYSMD3 PE=2 SV=2 - (LYSM3_HUMAN)	20.40	6.86	1	1	2	9	3.277E5	306	34.5	5.97
P23396	40S ribosomal protein S3 OS=Homo sapiens GN=RPS3 PE=1 SV=2 - (RS3_HUMAN)	20.18	44.86	18	7	11	17	3.178E9	243	26.7	9.66
E9PIY1	Protein kinase C and casein kinase substrate in neurons protein 3 OS=Homo sapiens GN=PACSIN3 PE=4 SV=1 - (E9PIY1_HUMAN)	20.04	8.44	4	1	3	18	2.530E7	320	37.2	6.90
O60502-2	Isoform 2 of Bifunctional protein NCOAT OS=Homo sapiens GN=MGEA5 - (NCOAT_HUMAN)	20.03	3.42	5	1	4	12	1.018E8	849	95.3	5.03
B0QY59	GTP binding protein 1 (Fragment) OS=Homo sapiens GN=GTPBP1 PE=4 SV=1 - (B0QY59_HUMAN)	19.89	8.88	3	1	4	33	2.522E8	349	37.8	10.56
E7EVA0	Microtubule-associated protein OS=Homo sapiens GN=MAP4 PE=4 SV=1 - (E7EVA0_HUMAN)	19.86	8.92	13	1	15	24	7.435E8	2297	245.3	6.23
P61088	Ubiquitin-conjugating enzyme E2 N OS=Homo sapiens GN=UBE2N PE=1 SV=1 - (UBE2N_HUMAN)	19.81	34.87	6	4	6	9	1.321E8	152	17.1	6.57
P15880	40S ribosomal protein S2 OS=Homo sapiens GN=RPS2 PE=1 SV=2 - (RS2_HUMAN)	19.63	33.45	18	4	10	14	1.714E8	293	31.3	10.24
P35913	Rod cGMP-specific 3',5'-cyclic phosphodiesterase subunit beta OS=Homo sapiens GN=PDE6B PE=1 SV=2 - (PDE6B_HUMAN)	19.60	11.01	2	1	9	19	1.629E8	854	98.3	5.24
I3L0X1	Fatty aldehyde dehydrogenase OS=Homo sapiens GN=ALDH3A2 PE=4 SV=1 - (I3L0X1_HUMAN)	19.58	14.29	4	1	3	16	4.708E8	182	20.7	5.03
Q6P452	Annexin OS=Homo sapiens GN=ANXA4 PE=2 SV=1 - (Q6P452_HUMAN)	19.49	31.44	14	5	8	8	1.209E8	299	33.5	5.86
P60866	40S ribosomal protein S20 OS=Homo sapiens GN=RPS20 PE=1 SV=1 - (RS20_HUMAN)	19.39	42.02	6	6	7	8	2.010E8	119	13.4	9.94
D6RAF8	Heterogeneous nuclear ribonucleoprotein D0 (Fragment) OS=Homo sapiens GN=HNRNPD PE=4 SV=1 - (D6RAF8_HUMAN)	19.28	21.27	18	2	4	10	1.926E8	221	23.1	5.11
Q6A163	Keratin, type I cytoskeletal 39 OS=Homo sapiens GN=KRT39 PE=1 SV=2 - (K1C39_HUMAN)	19.07	13.65	7	1	10	29	3.410E8	491	55.6	5.26
Q9UPY3	Endoribonuclease Dicer OS=Homo sapiens GN=DICER1 PE=1 SV=3 - (DICER_HUMAN)	19.04	5.57	2	1	10	18	1.747E9	1922	218.5	5.68
Q9Y2I6	Ninein-like protein OS=Homo sapiens GN=NINL PE=1 SV=2 - (NINL_HUMAN)	19.01	9.55	3	1	13	21	3.938E8	1382	156.2	5.06
P56715	Oxygen-regulated protein 1 OS=Homo sapiens GN=RP1 PE=1 SV=1 - (RP1_HUMAN)	18.93	6.96	2	1	14	28	2.186E8	2156	240.5	5.80
D3DUP2	WNK lysine deficient protein kinase 1, isoform CRA_d OS=Homo sapiens GN=WNK1 PE=4 SV=1 - (D3DUP2_HUMAN)	18.92	5.22	11	1	12	17	1.227E9	2107	222.6	6.43
P02549-2	Isoform 2 of Spectrin alpha chain, erythrocyte OS=Homo sapiens GN=SPTA1 - (SPTA1_HUMAN)	18.54	5.26	2	1	12	20	2.431E8	2416	279.5	5.05
A8K8P3-9	Isoform 9 of Protein SFI1 homolog OS=Homo sapiens GN=SFI1 - (SFI1_HUMAN)	18.27	9.94	11	1	13	26	3.555E8	1187	141.0	10.76
Q9P2D3-3	Isoform 3 of HEAT repeat-containing protein 5B OS=Homo sapiens GN=HEATR5B - (HTR5B_HUMAN)	18.27	4.34	3	1	10	19	1.083E8	1982	214.9	7.42
P35221	Catenin alpha-1 OS=Homo sapiens GN=CTNNA1 PE=1 SV=1 - (CTNA1_HUMAN)	18.21	14.79	15	1	11	26	1.654E9	906	100.0	6.29
F5GY37	Prohibitin-2 OS=Homo sapiens GN=PHB2 PE=4 SV=1 - (F5GY37_HUMAN)	18.13	33.33	9	3	9	10	1.412E8	267	29.7	9.88
F6RFD5	Destrin OS=Homo sapiens GN=DSTN PE=4 SV=1 - (F6RFD5_HUMAN)	18.11	40.74	4	2	5	9	8.943E7	135	15.4	8.59
Q96EY7	Pentatricopeptide repeat-containing protein 3, mitochondrial OS=Homo sapiens GN=PTCD3 PE=1 SV=3 - (PTCD3_HUMAN)	18.06	8.85	5	1	9	18	2.896E8	689	78.5	6.42
B4DL14	ATP synthase gamma chain OS=Homo sapiens GN=ATP5C1 PE=2 SV=1 - (B4DL14_HUMAN)	17.99	33.60	5	3	7	13	1.703E8	250	27.5	7.42
P25391	Laminin subunit alpha-1 OS=Homo sapiens GN=LAMA1 PE=1 SV=2 - (LAMA1_HUMAN)	17.91	3.90	5	2	9	23	1.048E8	3075	336.9	6.35
B4DMP5	cDNA FLJ60050, highly similar to Vacuolar protein sorting protein 72 homolog OS=Homo sapiens PE=2 SV=1 - (B4DMP5_HUMAN)	17.88	9.75	3	1	3	14	1.521E8	236	26.7	5.33
E7EVE5	Rap guanine nucleotide exchange factor 4 OS=Homo sapiens GN=RAPGEF4 PE=4 SV=1 - (E7EVE5_HUMAN)	17.62	7.23	11	1	9	36	6.883E8	1010	115.4	6.84
B4DG54	cDNA FLJ56635 OS=Homo sapiens PE=2 SV=1 - (B4DG54_HUMAN)	17.56	9.76	2	1	9	21	1.054E9	922	105.2	5.76
P63173	60S ribosomal protein L38 OS=Homo sapiens GN=RPL38 PE=1 SV=2 - (RL38_HUMAN)	17.38	28.57	1	2	3	5	5.825E7	70	8.2	10.10
P30041	Peroxiredoxin-6 OS=Homo sapiens GN=PRDX6 PE=1 SV=3 - (PRDX6_HUMAN)	17.36	19.64	2	5	5	11	1.527E8	224	25.0	6.38
P37837	Transaldolase OS=Homo sapiens GN=TALDO1 PE=1 SV=2 - (TALDO_HUMAN)	17.34	29.67	4	5	11	15	7.485E7	337	37.5	6.81
H0YKY3	Proline-serine-threonine phosphatase-interacting protein 1 OS=Homo sapiens GN=PSTPIP1 PE=4 SV=1 - (H0YKY3_HUMAN)	17.22	20.14	2	1	7	20	1.507E7	417	47.6	7.59
Q02487-2	Isoform 2B of Desmocollin-2 OS=Homo sapiens GN=DSC2 - (DSC2_HUMAN)	17.05	11.33	10	1	9	15	1.966E7	847	93.7	5.47
P54920	Alpha-soluble NSF attachment protein OS=Homo sapiens GN=NAPA PE=1 SV=3 - (SNAA_HUMAN)	17.02	14.58	6	2	4	12	8.261E7	295	33.2	5.36
Q9BV44	THUMP domain-containing protein 3 OS=Homo sapiens GN=THUMPD3 PE=1 SV=1 - (THUM3_HUMAN)	17.02	12.62	2	2	7	14	3.183E8	507	57.0	6.37
Q96FQ6	Protein S100-A16 OS=Homo sapiens GN=S100A16 PE=1 SV=1 - (S10AG_HUMAN)	17.00	36.89	1	3	3	8	3.844E7	103	11.8	6.79
Q6ZMS3	cDNA FLJ16726 fis, clone UTERU3014791 OS=Homo sapiens PE=2 SV=1 - (Q6ZMS3_HUMAN)	16.95	23.56	13	3	4	10	1.482E8	191	20.4	9.16
P28062-2	Isoform 2 of Proteasome subunit beta type-8 OS=Homo sapiens GN=PSMB8 - (PSB8_HUMAN)	16.86	8.46	3	1	3	12	1.572E8	272	29.8	5.82
B7Z478	Proteasome (Prosome, macropain) subunit, beta type, 2, isoform CRA_b OS=Homo sapiens GN=PSMB2 PE=2 SV=1 - (B7Z478_HUMAN)	16.75	19.89	2	1	3	6	1.987E7	176	20.2	7.44
Q9NVU3	cDNA FLJ10506 fis, clone NT2RP2000510 OS=Homo sapiens PE=2 SV=1 - (Q9NVU3_HUMAN)	16.69	7.18	5	1	7	17	6.856E8	864	96.3	6.65
Q7Z612	Acidic ribosomal phosphoprotein P1 OS=Homo sapiens PE=4 SV=1 - (Q7Z612_HUMAN)	16.66	14.16	4	1	1	5	1.306E8	113	11.4	4.36
B4DL49	cDNA FLJ58073, moderately similar to Cathepsin B (EC 3.4.22.1) OS=Homo sapiens PE=2 SV=1 - (B4DL49_HUMAN)	16.64	24.54	20	4	6	9	4.854E7	273	30.7	6.62
Q6YHK3	CD109 antigen OS=Homo sapiens GN=CD109 PE=1 SV=2 - (CD109_HUMAN)	16.46	8.17	4	6	11	14	3.423E7	1445	161.6	5.85
B4DMN1	cDNA FLJ61136, highly similar to Ras-related protein Rab-11A OS=Homo sapiens PE=2 SV=1 - (B4DMN1_HUMAN)	16.44	28.74	12	5	8	14	5.111E8	261	28.8	8.38
Q9NVE4	Coiled-coil domain-containing protein 87 OS=Homo sapiens GN=CCDC87 PE=2 SV=2 - (CCD87_HUMAN)	16.44	6.83	1	1	7	33	4.123E7	849	96.3	8.59
C9JP48	Serine/threonine-protein phosphatase (Fragment) OS=Homo sapiens GN=PPP1CB PE=3 SV=1 - (C9JP48_HUMAN)	16.39	34.78	22	4	4	5	2.509E7	138	15.8	5.15
P14735	Insulin-degrading enzyme OS=Homo sapiens GN=IDE PE=1 SV=4 - (IDE_HUMAN)	16.39	6.87	2	1	6	12	6.091E8	1019	117.9	6.61
Q0IJ56	ST13 protein (Fragment) OS=Homo sapiens GN=ST13 PE=2 SV=1 - (Q0IJ56_HUMAN)	16.36	24.19	11	2	6	9	4.584E7	310	35.0	5.35
Q5JWF2-2	Isoform XLas-2 of Guanine nucleotide-binding protein G(s) subunit alpha isoforms XLas OS=Homo sapiens GN=GNAS - (GNAS1_HUMAN)	16.35	2.93	38	2	3	9	1.332E8	1023	109.6	5.07
Q9P2Z0	THAP domain-containing protein 10 OS=Homo sapiens GN=THAP10 PE=2 SV=1 - (THA10_HUMAN)	16.32	15.95	2	1	7	20	3.606E8	257	28.3	8.60
Q9UIS4	Small nuclear ribonucleoprotein-associated protein OS=Homo sapiens GN=SNRPB PE=2 SV=1 - (Q9UIS4_HUMAN)	16.29	16.87	12	2	5	13	5.783E7	243	27.2	9.41
E9PR44	Alpha-crystallin B chain (Fragment) OS=Homo sapiens GN=CRYAB PE=3 SV=1 - (E9PR44_HUMAN)	16.27	38.51	10	5	7	14	4.561E7	174	20.0	7.03
Q8NFA0	Ubiquitin carboxyl-terminal hydrolase 32 OS=Homo sapiens GN=USP32 PE=1 SV=1 - (UBP32_HUMAN)	16.12	5.36	2	1	8	27	8.431E7	1604	181.5	6.44
Q86TB3	Alpha-protein kinase 2 OS=Homo sapiens GN=ALPK2 PE=1 SV=3 - (ALPK2_HUMAN)	16.10	7.56	1	1	16	24	5.350E8	2170	236.9	5.24
P60900	Proteasome subunit alpha type-6 OS=Homo sapiens GN=PSMA6 PE=1 SV=1 - (PSA6_HUMAN)	16.04	26.83	11	4	6	7	1.748E8	246	27.4	6.76
Q86Z22	Putative uncharacterized protein OS=Homo sapiens PE=2 SV=1 - (Q86Z22_HUMAN)	16.04	23.89	4	3	5	9	3.188E7	226	23.8	9.06
P0CW22	40S ribosomal protein S17-like OS=Homo sapiens GN=RPS17L PE=3 SV=1 - (RS17L_HUMAN)	15.71	40.00	5	4	5	6	5.947E7	135	15.5	9.85
B4DRD7	Poly(rC)-binding protein 2 OS=Homo sapiens GN=PCBP2 PE=2 SV=1 - (B4DRD7_HUMAN)	15.62	15.25	24	1	4	6	6.084E7	282	29.7	7.71
Q13435	Splicing factor 3B subunit 2 OS=Homo sapiens GN=SF3B2 PE=1 SV=2 - (SF3B2_HUMAN)	15.54	10.95	5	1	9	17	1.759E8	895	100.2	5.67
F5H1Y4	Golgi-associated PDZ and coiled-coil motif-containing protein OS=Homo sapiens GN=GOPC PE=4 SV=1 - (F5H1Y4_HUMAN)	15.53	12.99	4	1	4	15	5.282E7	462	50.6	6.23
O00299	Chloride intracellular channel protein 1 OS=Homo sapiens GN=CLIC1 PE=1 SV=4 - (CLIC1_HUMAN)	15.48	26.14	2	3	7	8	6.007E7	241	26.9	5.17
P62280	40S ribosomal protein S11 OS=Homo sapiens GN=RPS11 PE=1 SV=3 - (RS11_HUMAN)	15.43	34.18	1	3	7	12	4.435E7	158	18.4	10.30
B4DRM3	cDNA FLJ54492, highly similar to Eukaryotic translation initiation factor 4B OS=Homo sapiens PE=2 SV=1 - (B4DRM3_HUMAN)	15.36	10.88	12	1	8	58	8.480E7	616	69.7	5.67
P11940-2	Isoform 2 of Polyadenylate-binding protein 1 OS=Homo sapiens GN=PABPC1 - (PABP1_HUMAN)	15.32	15.17	12	1	8	16	1.353E8	547	61.1	9.07
B8ZZK4	60S ribosomal protein L31 OS=Homo sapiens GN=RPL31 PE=4 SV=1 - (B8ZZK4_HUMAN)	15.31	53.16	9	3	6	13	2.544E8	79	9.0	10.89
P59998	Actin-related protein 2/3 complex subunit 4 OS=Homo sapiens GN=ARPC4 PE=1 SV=3 - (ARPC4_HUMAN)	15.07	24.40	8	2	6	11	5.166E7	168	19.7	8.43
P84077	ADP-ribosylation factor 1 OS=Homo sapiens GN=ARF1 PE=1 SV=2 - (ARF1_HUMAN)	15.05	37.57	16	2	7	8	1.556E8	181	20.7	6.80
Q8N300	Coiled-coil domain-containing protein 23 OS=Homo sapiens GN=CCDC23 PE=2 SV=1 - (CCD23_HUMAN)	15.00	16.67	1	1	2	16	8.009E7	66	7.8	9.16
P46781	40S ribosomal protein S9 OS=Homo sapiens GN=RPS9 PE=1 SV=3 - (RS9_HUMAN)	14.90	48.45	7	5	10	13	1.031E8	194	22.6	10.65
P30044	Peroxiredoxin-5, mitochondrial OS=Homo sapiens GN=PRDX5 PE=1 SV=4 - (PRDX5_HUMAN)	14.90	41.12	6	5	6	6	4.555E7	214	22.1	8.70
Q92788	Rad GTPase OS=Homo sapiens PE=2 SV=1 - (Q92788_HUMAN)	14.89	14.94	3	1	5	38	3.414E7	308	33.2	8.68
H3BQZ9	Adenine phosphoribosyltransferase OS=Homo sapiens GN=APRT PE=4 SV=1 - (H3BQZ9_HUMAN)	14.88	28.10	7	3	4	7	2.276E7	153	16.6	5.59
B7Z382	Cytosolic purine 5'-nucleotidase OS=Homo sapiens GN=NT5C2 PE=2 SV=1 - (B7Z382_HUMAN)	14.85	5.45	7	1	3	24	1.240E8	532	61.4	5.74
Q5Y190	Anchor protein OS=Homo sapiens GN=RESDA1 PE=2 SV=1 - (Q5Y190_HUMAN)	14.77	4.42	1	1	16	25	2.730E8	4186	453.0	6.83
P18077	60S ribosomal protein L35a OS=Homo sapiens GN=RPL35A PE=1 SV=2 - (RL35A_HUMAN)	14.75	38.18	2	2	5	11	5.019E7	110	12.5	11.06
Q53RT3	Retroviral-like aspartic protease 1 OS=Homo sapiens GN=ASPRV1 PE=1 SV=1 - (APRV1_HUMAN)	14.71	14.29	1	4	4	7	5.867E7	343	37.0	5.44
Q8TCJ6	Putative uncharacterized protein DKFZp667C0210 (Fragment) OS=Homo sapiens GN=DKFZp667C0210 PE=2 SV=1 - (Q8TCJ6_HUMAN)	14.68	9.76	3	1	5	12	6.172E7	461	52.1	7.33
F2Z2C0	Exosome complex exonuclease RRP44 OS=Homo sapiens GN=DIS3 PE=4 SV=1 - (F2Z2C0_HUMAN)	14.68	3.04	6	1	2	13	2.185E6	559	63.6	6.40
O60506-2	Isoform 2 of Heterogeneous nuclear ribonucleoprotein Q OS=Homo sapiens GN=SYNCRIP - (HNRPQ_HUMAN)	14.56	24.15	18	2	14	33	1.253E8	588	65.6	8.60
Q9H2F9	Coiled-coil domain-containing protein 68 OS=Homo sapiens GN=CCDC68 PE=2 SV=1 - (CCD68_HUMAN)	14.56	13.13	1	1	5	19	4.407E8	335	38.8	8.65
Q9NVK5-2	Isoform 2 of FGFR1 oncogene partner 2 OS=Homo sapiens GN=FGFR1OP2 - (FGOP2_HUMAN)	14.54	27.44	3	1	6	20	2.476E8	215	25.0	5.57
Q9BUP0	EF-hand domain-containing protein D1 OS=Homo sapiens GN=EFHD1 PE=1 SV=1 - (EFHD1_HUMAN)	14.49	26.36	4	2	7	7	3.051E7	239	26.9	5.39
Q05877	Hepatocellular carcinoma protein HHCM OS=Homo sapiens PE=4 SV=1 - (HHCM_HUMAN)	14.45	8.14	1	1	3	13	8.397E8	467	52.1	9.54
Q9Y4F4	Protein FAM179B OS=Homo sapiens GN=FAM179B PE=1 SV=4 - (F179B_HUMAN)	14.30	8.84	1	1	12	16	5.340E8	1720	189.2	8.50
A8MUD9	60S ribosomal protein L7 OS=Homo sapiens GN=RPL7 PE=3 SV=1 - (A8MUD9_HUMAN)	14.30	27.40	5	3	6	8	5.214E7	208	24.4	10.67
Q16176	Lsk protein OS=Homo sapiens GN=lsk PE=2 SV=1 - (Q16176_HUMAN)	14.28	9.89	1	1	4	10	7.793E7	465	51.9	8.51
P62263	40S ribosomal protein S14 OS=Homo sapiens GN=RPS14 PE=1 SV=3 - (RS14_HUMAN)	14.26	24.50	3	4	5	7	1.242E8	151	16.3	10.05
P62854	40S ribosomal protein S26 OS=Homo sapiens GN=RPS26 PE=1 SV=3 - (RS26_HUMAN)	14.26	37.39	8	4	4	6	1.173E8	115	13.0	11.00
F5GX11	Proteasome subunit alpha type-1 OS=Homo sapiens GN=PSMA1 PE=4 SV=1 - (F5GX11_HUMAN)	14.18	40.34	6	4	9	9	1.296E8	238	26.5	6.73
P36543	V-type proton ATPase subunit E 1 OS=Homo sapiens GN=ATP6V1E1 PE=1 SV=1 - (VATE1_HUMAN)	14.15	40.27	3	1	7	14	1.303E9	226	26.1	8.00
Q15126	Phosphomevalonate kinase OS=Homo sapiens GN=PMVK PE=1 SV=3 - (PMVK_HUMAN)	14.15	17.19	1	3	4	11	7.710E7	192	22.0	5.73
P23284	Peptidyl-prolyl cis-trans isomerase B OS=Homo sapiens GN=PPIB PE=1 SV=2 - (PPIB_HUMAN)	14.12	25.93	1	4	6	9	8.630E7	216	23.7	9.41
Q6ZME5	cDNA FLJ23974 fis, clone HEP18860 OS=Homo sapiens PE=2 SV=1 - (Q6ZME5_HUMAN)	14.06	15.79	7	1	2	9	2.025E8	133	15.3	9.86
Q8NDA2	Hemicentin-2 OS=Homo sapiens GN=HMCN2 PE=2 SV=2 - (HMCN2_HUMAN)	14.05	4.60	3	1	18	20	2.871E8	5065	542.3	5.83
E7EPK6	40S ribosomal protein S24 OS=Homo sapiens GN=RPS24 PE=3 SV=1 - (E7EPK6_HUMAN)	14.04	22.15	5	3	6	10	2.131E8	289	32.4	10.15
P25398	40S ribosomal protein S12 OS=Homo sapiens GN=RPS12 PE=1 SV=3 - (RS12_HUMAN)	14.03	28.79	1	3	4	8	5.586E7	132	14.5	7.21
B4DJP7	Small nuclear ribonucleoprotein Sm D3 OS=Homo sapiens GN=SNRPD3 PE=2 SV=1 - (B4DJP7_HUMAN)	14.00	23.33	2	1	3	9	2.956E7	120	13.3	8.91
P63241-2	Isoform 2 of Eukaryotic translation initiation factor 5A-1 OS=Homo sapiens GN=EIF5A - (IF5A1_HUMAN)	13.95	30.98	9	2	5	11	5.386E7	184	20.2	7.01
B7Z399	cDNA FLJ50162, highly similar to Glycosyltransferase-like protein LARGE1 (EC 2.4.-.-) OS=Homo sapiens PE=2 SV=1 - (B7Z399_HUMAN)	13.92	7.27	7	1	5	11	7.058E7	688	80.5	7.36
H0YJA0	Putative E3 ubiquitin-protein ligase UBR7 (Fragment) OS=Homo sapiens GN=UBR7 PE=4 SV=1 - (H0YJA0_HUMAN)	13.85	11.59	1	1	2	9	1.620E7	164	18.0	4.64
Q5T321	Neurobeachin OS=Homo sapiens GN=NBEA PE=2 SV=1 - (Q5T321_HUMAN)	13.82	4.62	10	1	13	27	1.374E8	2943	327.3	6.16
Q9ULG6-4	Isoform 4 of Cell cycle progression protein 1 OS=Homo sapiens GN=CCPG1 - (CCPG1_HUMAN)	13.81	10.37	5	1	6	10	2.312E8	598	68.4	5.67
P05089	Arginase-1 OS=Homo sapiens GN=ARG1 PE=1 SV=2 - (ARGI1_HUMAN)	13.72	21.43	3	2	5	7	4.531E7	322	34.7	7.21
B4DHK5	cDNA FLJ61590, moderately similar to Pleckstrin homology domain-containing family A member 5 (Fragment) OS=Homo sapiens PE=2 SV=1 - (B4DHK5_HUMAN)	13.68	7.78	5	1	11	19	1.652E8	1183	135.9	8.53
P09493	Tropomyosin alpha-1 chain OS=Homo sapiens GN=TPM1 PE=1 SV=2 - (TPM1_HUMAN)	13.66	37.32	41	2	11	13	3.736E8	284	32.7	4.74
A4QN26	NBPF4 protein (Fragment) OS=Homo sapiens GN=NBPF4 PE=2 SV=1 - (A4QN26_HUMAN)	13.57	7.54	61	1	4	19	1.867E8	544	61.6	4.78
D3DV26	S100 calcium binding protein A10 (Annexin II ligand, calpactin I, light polypeptide (P11)), isoform CRA_b (Fragment) OS=Homo sapiens GN=S100A10 PE=4 SV=1 - (D3DV26_HUMAN)	13.56	40.98	3	3	7	15	1.027E8	205	22.3	10.33
P56730	Neurotrypsin OS=Homo sapiens GN=PRSS12 PE=2 SV=2 - (NETR_HUMAN)	13.43	8.11	1	1	9	13	5.037E7	875	97.0	8.03
E9PI98	Layilin (Fragment) OS=Homo sapiens GN=LAYN PE=4 SV=1 - (E9PI98_HUMAN)	13.37	14.06	1	1	1	8	3.184E8	64	7.4	8.00
P40925	Malate dehydrogenase, cytoplasmic OS=Homo sapiens GN=MDH1 PE=1 SV=4 - (MDHC_HUMAN)	13.36	28.14	8	5	10	10	8.211E7	334	36.4	7.36
O14662-2	Isoform A of Syntaxin-16 OS=Homo sapiens GN=STX16 - (STX16_HUMAN)	13.31	5.92	10	1	2	9	7.980E7	304	34.8	6.20
I3L3P7	40S ribosomal protein S15a OS=Homo sapiens GN=RPS15A PE=4 SV=1 - (I3L3P7_HUMAN)	13.30	66.00	6	4	8	10	2.308E8	100	11.5	10.15
Q5T6W2	Heterogeneous nuclear ribonucleoprotein K (Fragment) OS=Homo sapiens GN=HNRNPK PE=2 SV=1 - (Q5T6W2_HUMAN)	13.28	27.44	9	5	12	18	1.722E8	379	41.8	5.59
O14497	AT-rich interactive domain-containing protein 1A OS=Homo sapiens GN=ARID1A PE=1 SV=3 - (ARI1A_HUMAN)	13.17	3.68	2	1	8	24	5.035E7	2285	241.9	6.70
Q8NEY1-7	Isoform 7 of Neuron navigator 1 OS=Homo sapiens GN=NAV1 - (NAV1_HUMAN)	13.16	9.01	9	1	13	25	9.307E8	1809	194.9	8.28
Q9BWC7	COMT protein OS=Homo sapiens GN=COMT PE=2 SV=2 - (Q9BWC7_HUMAN)	12.91	17.03	10	2	2	3	2.778E7	182	20.0	5.60
P62750	60S ribosomal protein L23a OS=Homo sapiens GN=RPL23A PE=1 SV=1 - (RL23A_HUMAN)	12.87	24.36	6	3	4	6	7.971E7	156	17.7	10.45
E7EPB3	60S ribosomal protein L14 OS=Homo sapiens GN=RPL14 PE=4 SV=1 - (E7EPB3_HUMAN)	12.86	58.06	7	5	8	12	2.107E9	124	14.5	10.21
Q9NNZ3	DnaJ homolog subfamily C member 4 OS=Homo sapiens GN=DNAJC4 PE=2 SV=1 - (DNJC4_HUMAN)	12.75	20.33	2	1	5	11	7.987E7	241	27.6	10.55
B4E086	cDNA FLJ51584, highly similar to Homo sapiens spindle pole body component 24 homolog (SPBC24), mRNA OS=Homo sapiens PE=2 SV=1 - (B4E086_HUMAN)	12.74	15.00	1	1	2	8	2.458E8	140	16.0	4.55
Q7Z4W8	Heparin-binding protein HBp15 OS=Homo sapiens PE=2 SV=1 - (Q7Z4W8_HUMAN)	12.62	28.91	2	2	5	11	2.203E8	128	14.8	9.32
H7BXZ3	Unconventional myosin-VIIa OS=Homo sapiens GN=MYO7A PE=4 SV=1 - (H7BXZ3_HUMAN)	12.58	5.50	17	1	12	18	1.195E9	2091	240.3	8.78
Q5D862	Filaggrin-2 OS=Homo sapiens GN=FLG2 PE=1 SV=1 - (FILA2_HUMAN)	12.54	2.63	1	4	7	9	3.459E7	2391	247.9	8.31
P49207	60S ribosomal protein L34 OS=Homo sapiens GN=RPL34 PE=1 SV=3 - (RL34_HUMAN)	12.49	37.61	2	5	7	7	1.565E8	117	13.3	11.47
P20618	Proteasome subunit beta type-1 OS=Homo sapiens GN=PSMB1 PE=1 SV=2 - (PSB1_HUMAN)	12.49	17.01	4	2	3	7	8.551E6	241	26.5	8.13
Q96HZ4-2	Isoform 2 of Transcription cofactor HES-6 OS=Homo sapiens GN=HES6 - (HES6_HUMAN)	12.47	26.64	2	1	5	9	4.132E8	214	23.5	11.94
B7ZAV1	cDNA, FLJ79315, highly similar to Proto-oncogene DBL OS=Homo sapiens PE=2 SV=1 - (B7ZAV1_HUMAN)	12.46	8.89	16	1	8	15	1.174E8	1001	116.1	5.96
Q8NEY8-3	Isoform 3 of Periphilin-1 OS=Homo sapiens GN=PPHLN1 - (PPHLN_HUMAN)	12.40	8.06	5	1	4	28	2.194E8	434	49.6	8.72
P61077	Ubiquitin-conjugating enzyme E2 D3 OS=Homo sapiens GN=UBE2D3 PE=1 SV=1 - (UB2D3_HUMAN)	12.35	45.58	15	3	5	7	3.487E7	147	16.7	7.80
P07108-4	Isoform 4 of Acyl-CoA-binding protein OS=Homo sapiens GN=DBI - (ACBP_HUMAN)	12.29	44.96	8	3	5	13	5.589E7	129	14.4	9.42
Q9UBT7-3	Isoform 3 of Alpha-catulin OS=Homo sapiens GN=CTNNAL1 - (CTNL1_HUMAN)	12.29	10.46	6	1	6	11	5.018E7	650	72.6	7.06
Q6ZPB4	cDNA FLJ26129 fis, clone TMS02971 OS=Homo sapiens PE=2 SV=1 - (Q6ZPB4_HUMAN)	12.22	35.00	1	1	4	12	1.108E8	140	16.1	6.77
B3KTS5	cDNA FLJ38670 fis, clone HSYRA2000190, highly similar to Voltage-dependent anion-selective channel protein 1 OS=Homo sapiens PE=2 SV=1 - (B3KTS5_HUMAN)	12.21	14.49	6	1	5	10	4.918E7	283	30.7	7.90
P26373	60S ribosomal protein L13 OS=Homo sapiens GN=RPL13 PE=1 SV=4 - (RL13_HUMAN)	12.17	27.49	4	4	7	17	2.209E8	211	24.2	11.65
O75962	Triple functional domain protein OS=Homo sapiens GN=TRIO PE=1 SV=2 - (TRIO_HUMAN)	12.12	4.20	8	1	13	34	1.787E9	3097	346.7	6.37
E5RI99	60S ribosomal protein L30 (Fragment) OS=Homo sapiens GN=RPL30 PE=3 SV=1 - (E5RI99_HUMAN)	12.11	42.11	9	4	5	8	7.930E7	114	12.6	9.55
P62424	60S ribosomal protein L7a OS=Homo sapiens GN=RPL7A PE=1 SV=2 - (RL7A_HUMAN)	12.11	30.83	7	3	9	10	1.604E8	266	30.0	10.61
G3V193	Activating molecule in BECN1-regulated autophagy protein 1 OS=Homo sapiens GN=AMBRA1 PE=4 SV=1 - (G3V193_HUMAN)	12.10	8.65	7	1	10	19	1.758E8	1179	129.5	6.98
P28074	Proteasome subunit beta type-5 OS=Homo sapiens GN=PSMB5 PE=1 SV=3 - (PSB5_HUMAN)	12.06	20.15	4	3	5	6	2.179E8	263	28.5	6.92
Q8WX93-2	Isoform 2 of Palladin OS=Homo sapiens GN=PALLD - (PALLD_HUMAN)	12.04	9.31	8	1	9	14	1.666E8	1106	122.0	6.92
B3KUZ8	Aspartate aminotransferase OS=Homo sapiens PE=2 SV=1 - (B3KUZ8_HUMAN)	11.91	20.49	5	2	6	17	2.964E8	371	41.3	8.84
P18621	60S ribosomal protein L17 OS=Homo sapiens GN=RPL17 PE=1 SV=3 - (RL17_HUMAN)	11.88	25.54	2	3	7	9	2.053E7	184	21.4	10.17
P61626	Lysozyme C OS=Homo sapiens GN=LYZ PE=1 SV=1 - (LYSC_HUMAN)	11.85	17.57	2	2	3	9	8.573E6	148	16.5	9.16
P62826	GTP-binding nuclear protein Ran OS=Homo sapiens GN=RAN PE=1 SV=3 - (RAN_HUMAN)	11.71	23.15	4	2	4	6	5.983E7	216	24.4	7.49
Q2M1Z3	Rho GTPase-activating protein 31 OS=Homo sapiens GN=ARHGAP31 PE=1 SV=2 - (RHG31_HUMAN)	11.68	3.12	4	1	6	9	3.921E8	1444	156.9	5.76
Q9UNM1	Chaperonin 10-related protein (Fragment) OS=Homo sapiens GN=EPFP1 PE=2 SV=1 - (Q9UNM1_HUMAN)	11.59	37.11	4	2	4	6	8.475E7	97	10.3	9.00
Q8ND71	GTPase IMAP family member 8 OS=Homo sapiens GN=GIMAP8 PE=1 SV=2 - (GIMA8_HUMAN)	11.54	12.93	1	1	9	17	8.794E8	665	74.8	8.34
B7Z539	cDNA FLJ56954, highly similar to Inter-alpha-trypsin inhibitor heavy chain H1 OS=Homo sapiens PE=2 SV=1 - (B7Z539_HUMAN)	11.52	4.50	10	1	2	7	2.624E8	645	72.1	7.68
P46776	60S ribosomal protein L27a OS=Homo sapiens GN=RPL27A PE=1 SV=2 - (RL27A_HUMAN)	11.46	38.51	6	3	7	11	1.359E8	148	16.6	11.00
O14818	Proteasome subunit alpha type-7 OS=Homo sapiens GN=PSMA7 PE=1 SV=1 - (PSA7_HUMAN)	11.45	24.60	14	2	7	12	7.797E7	248	27.9	8.46
A8MXN1	Receptor expression-enhancing protein 6 OS=Homo sapiens GN=REEP6 PE=4 SV=1 - (A8MXN1_HUMAN)	11.44	13.13	1	1	8	23	2.532E8	480	49.7	11.24
F5GY69	Serpin B11 OS=Homo sapiens GN=SERPINB11 PE=3 SV=1 - (F5GY69_HUMAN)	11.38	12.63	3	1	2	5	1.433E8	190	21.1	6.28
Q9NTK5	Obg-like ATPase 1 OS=Homo sapiens GN=OLA1 PE=1 SV=2 - (OLA1_HUMAN)	11.33	7.07	5	1	2	4	4.577E7	396	44.7	7.81
B9A6J0	Ecotropic viral integration site 5 OS=Homo sapiens GN=EVI5 PE=2 SV=1 - (B9A6J0_HUMAN)	11.30	9.87	4	1	7	20	3.378E8	821	94.0	6.04
O60284	Suppression of tumorigenicity 18 protein OS=Homo sapiens GN=ST18 PE=1 SV=1 - (ST18_HUMAN)	11.28	4.78	1	1	5	8	8.400E7	1047	115.1	6.06
B4DUB7	Phosphorylase OS=Homo sapiens PE=2 SV=1 - (B4DUB7_HUMAN)	11.27	10.70	15	2	9	13	1.008E8	813	92.9	7.17
P00491	Purine nucleoside phosphorylase OS=Homo sapiens GN=PNP PE=1 SV=2 - (PNPH_HUMAN)	11.26	12.80	5	2	4	12	1.897E7	289	32.1	6.95
A8K0T9	cDNA FLJ75422, highly similar to Homo sapiens capping protein (actin filament) muscle Z-line, alpha 1, mRNA OS=Homo sapiens PE=2 SV=1 - (A8K0T9_HUMAN)	11.25	11.19	7	2	4	8	5.490E7	286	32.9	5.69
Q96EA4	Protein Spindly OS=Homo sapiens GN=CCDC99 PE=1 SV=2 - (SPDLY_HUMAN)	11.22	15.87	6	1	8	14	5.330E8	605	70.1	5.47
Q9H9L3	Interferon-stimulated 20 kDa exonuclease-like 2 OS=Homo sapiens GN=ISG20L2 PE=1 SV=1 - (I20L2_HUMAN)	11.17	20.11	1	1	7	21	9.451E7	353	39.1	9.94
F8VUA6	60S ribosomal protein L18 (Fragment) OS=Homo sapiens GN=RPL18 PE=4 SV=1 - (F8VUA6_HUMAN)	11.15	30.00	9	2	4	7	2.765E7	130	14.5	11.75
B4DJQ8	cDNA FLJ55694, highly similar to Dipeptidyl-peptidase 1 (EC 3.4.14.1) OS=Homo sapiens PE=2 SV=1 - (B4DJQ8_HUMAN)	11.12	13.00	4	2	6	7	2.171E7	446	50.1	6.99
B2RBG3	cDNA, FLJ95493, highly similar to Homo sapiens fucosidase, alpha-L- 1, tissue (FUCA1), mRNA OS=Homo sapiens PE=2 SV=1 - (B2RBG3_HUMAN)	11.08	6.94	2	1	2	5	1.066E8	461	53.2	6.84
B7Z233	Acetyl-CoA acetyltransferase, cytosolic OS=Homo sapiens GN=ACAT2 PE=2 SV=1 - (B7Z233_HUMAN)	11.00	17.84	4	3	6	8	1.291E8	426	44.6	8.46
P62266	40S ribosomal protein S23 OS=Homo sapiens GN=RPS23 PE=1 SV=3 - (RS23_HUMAN)	11.00	20.98	1	1	3	5	4.160E7	143	15.8	10.49
E9PIZ3	60S ribosomal protein L8 OS=Homo sapiens GN=RPL8 PE=4 SV=1 - (E9PIZ3_HUMAN)	10.96	18.55	7	3	4	4	1.210E8	221	23.9	11.24
F5H4R1	Mitogen-activated protein kinase kinase kinase 4 OS=Homo sapiens GN=MAP3K4 PE=4 SV=1 - (F5H4R1_HUMAN)	10.95	6.17	6	1	9	17	1.893E8	1442	162.9	6.30
Q5T749	Keratinocyte proline-rich protein OS=Homo sapiens GN=KPRP PE=1 SV=1 - (KPRP_HUMAN)	10.94	5.01	1	2	4	10	6.501E7	579	64.1	8.27
Q2TNB3	Cell migration-inducing protein 22 OS=Homo sapiens PE=2 SV=1 - (Q2TNB3_HUMAN)	10.91	25.52	5	3	5	6	5.155E7	239	27.3	5.58
B3KUT2	cDNA FLJ40562 fis, clone THYMU2003981, moderately similar to Rattus norvegicus macrophage expressed gene 1 (Mpeg1), mRNA OS=Homo sapiens PE=2 SV=1 - (B3KUT2_HUMAN)	10.87	3.63	2	1	2	7	1.630E8	716	78.5	7.65
Q8IYY4	Zinc finger protein DZIP1L OS=Homo sapiens GN=DZIP1L PE=2 SV=2 - (DZI1L_HUMAN)	10.85	11.99	3	1	7	12	2.889E8	767	86.8	7.28
Q0D2H7	PCF11 protein (Fragment) OS=Homo sapiens GN=PCF11 PE=2 SV=1 - (Q0D2H7_HUMAN)	10.82	19.62	1	1	2	9	2.271E8	158	17.0	8.02
Q5VX52	Spermatogenesis-associated protein 1 OS=Homo sapiens GN=SPATA1 PE=2 SV=3 - (SPAT1_HUMAN)	10.82	27.92	3	1	11	24	1.065E9	437	50.3	8.43
Q86UY0	TXNDC5 protein OS=Homo sapiens GN=TXNDC5 PE=2 SV=1 - (Q86UY0_HUMAN)	10.79	14.72	5	1	8	11	4.076E8	360	40.3	5.83
B4E1U0	cDNA FLJ59148, highly similar to RNA-binding protein 4 OS=Homo sapiens PE=2 SV=1 - (B4E1U0_HUMAN)	10.78	6.94	2	1	1	4	4.873E7	173	19.8	7.39
Q9UMZ2-6	Isoform 5 of Synergin gamma OS=Homo sapiens GN=SYNRG - (SYNRG_HUMAN)	10.76	6.23	7	1	7	12	7.902E7	1108	119.4	5.12
D6RA82	Annexin OS=Homo sapiens GN=ANXA3 PE=3 SV=1 - (D6RA82_HUMAN)	10.74	11.97	5	2	4	11	5.918E8	284	32.1	5.94
B4DYC8	cDNA FLJ52361, highly similar to T-complex protein 1 subunit epsilon OS=Homo sapiens PE=2 SV=1 - (B4DYC8_HUMAN)	10.73	18.31	16	2	7	7	1.086E8	486	53.9	6.55
Q9HD67	Unconventionnal myosin-X OS=Homo sapiens GN=MYO10 PE=1 SV=3 - (MYO10_HUMAN)	10.68	5.88	4	1	13	24	2.151E8	2058	237.2	6.21
Q5SSJ5-3	Isoform 3 of Heterochromatin protein 1-binding protein 3 OS=Homo sapiens GN=HP1BP3 - (HP1B3_HUMAN)	10.61	20.20	5	1	8	9	1.055E9	401	44.4	10.10
B3KSL2	cDNA FLJ36533 fis, clone TRACH2004428, highly similar to Lactotransferrin (EC 3.4.21.-) (Fragment) OS=Homo sapiens PE=2 SV=1 - (B3KSL2_HUMAN)	10.58	9.47	14	1	5	7	1.517E8	697	76.7	8.02
B4DTN4	N6-adenosine-methyltransferase 70 kDa subunit OS=Homo sapiens GN=METTL3 PE=2 SV=1 - (B4DTN4_HUMAN)	10.56	25.97	1	1	6	11	2.639E8	258	27.6	8.02
Q63HR1	Putative uncharacterized protein DKFZp686P17171 OS=Homo sapiens GN=DKFZp686P17171 PE=2 SV=1 - (Q63HR1_HUMAN)	10.52	17.05	7	1	5	7	8.728E7	387	42.4	8.44
O60667-2	Isoform 2 of Fas apoptotic inhibitory molecule 3 OS=Homo sapiens GN=FAIM3 - (FAIM3_HUMAN)	10.48	12.23	4	1	3	7	5.776E7	278	31.0	10.11
A6NCL7	Ankyrin repeat domain-containing protein 33B OS=Homo sapiens GN=ANKRD33B PE=4 SV=1 - (AN33B_HUMAN)	10.36	4.45	2	1	2	6	2.692E7	494	53.9	8.00
F4ZW63	NF45 OS=Homo sapiens PE=2 SV=1 - (F4ZW63_HUMAN)	10.36	4.87	4	1	2	7	2.221E8	390	43.0	5.34
B3KR97	cDNA FLJ33900 fis, clone CTONG2008262, highly similar to Low density lipoprotein receptor adapter protein 1 OS=Homo sapiens PE=2 SV=1 - (B3KR97_HUMAN)	10.35	13.31	2	1	4	8	1.424E8	308	33.9	6.70
H7C4J1	Actin-related protein 3C (Fragment) OS=Homo sapiens GN=ACTR3C PE=4 SV=1 - (H7C4J1_HUMAN)	10.34	30.29	13	1	5	11	1.184E8	208	23.8	5.86
E9PBV3	Suprabasin OS=Homo sapiens GN=SBSN PE=4 SV=1 - (E9PBV3_HUMAN)	10.34	16.44	1	1	4	7	1.824E8	590	60.5	7.01
Q8N7B9	EF-hand calcium-binding domain-containing protein 3 OS=Homo sapiens GN=EFCAB3 PE=1 SV=1 - (EFCB3_HUMAN)	10.27	12.56	3	1	5	8	1.489E8	438	50.1	9.22
C9JA38	DALR anticodon-binding domain-containing protein 3 (Fragment) OS=Homo sapiens GN=DALRD3 PE=4 SV=2 - (C9JA38_HUMAN)	10.27	10.88	6	1	2	6	4.408E7	294	31.9	7.97
P13010	X-ray repair cross-complementing protein 5 OS=Homo sapiens GN=XRCC5 PE=1 SV=3 - (XRCC5_HUMAN)	10.26	14.75	2	2	9	10	1.782E8	732	82.7	5.81
P21108	Ribose-phosphate pyrophosphokinase 3 OS=Homo sapiens GN=PRPS1L1 PE=1 SV=2 - (PRPS3_HUMAN)	10.23	10.38	12	1	3	6	1.060E9	318	34.8	6.35
B4DLJ7	cDNA FLJ59334, highly similar to Transmembrane glycoprotein NMB OS=Homo sapiens PE=2 SV=1 - (B4DLJ7_HUMAN)	10.17	14.98	11	1	6	12	4.305E8	514	57.2	6.77
A8MUQ0	Putative paraneoplastic antigen-like protein 6B-like protein LOC649238 OS=Homo sapiens PE=3 SV=1 - (PN6BL_HUMAN)	10.04	5.09	1	1	2	6	7.607E7	334	37.3	7.33
Q9H9B7	Coatomer subunit gamma OS=Homo sapiens PE=2 SV=1 - (Q9H9B7_HUMAN)	9.95	7.76	8	1	6	21	3.164E7	799	89.3	5.68
Q08257	Quinone oxidoreductase OS=Homo sapiens GN=CRYZ PE=1 SV=1 - (QOR_HUMAN)	9.95	18.84	5	4	6	8	5.134E7	329	35.2	8.44
Q6ZU59	cDNA FLJ43980 fis, clone TESTI4018806 OS=Homo sapiens PE=2 SV=1 - (Q6ZU59_HUMAN)	9.95	13.29	1	1	2	8	2.518E8	143	16.6	6.80
O14647-2	Isoform 2 of Chromodomain-helicase-DNA-binding protein 2 OS=Homo sapiens GN=CHD2 - (CHD2_HUMAN)	9.93	8.63	3	1	13	23	4.092E8	1739	200.4	7.84
H0YKD8	60S ribosomal protein L28 OS=Homo sapiens GN=RPL28 PE=4 SV=1 - (H0YKD8_HUMAN)	9.91	20.59	9	1	3	9	9.010E7	170	19.1	11.46
P68402	Platelet-activating factor acetylhydrolase IB subunit beta OS=Homo sapiens GN=PAFAH1B2 PE=1 SV=1 - (PA1B2_HUMAN)	9.91	8.30	1	1	2	5	5.730E7	229	25.6	5.92
Q52LG2	Keratin-associated protein 13-2 OS=Homo sapiens GN=KRTAP13-2 PE=1 SV=1 - (KR132_HUMAN)	9.90	26.29	2	4	4	7	3.777E7	175	18.7	8.29
Q9UKS7	Zinc finger protein Helios OS=Homo sapiens GN=IKZF2 PE=1 SV=2 - (IKZF2_HUMAN)	9.89	10.08	14	1	7	21	3.749E8	526	59.5	6.77
Q13239	Src-like-adapter OS=Homo sapiens GN=SLA PE=1 SV=3 - (SLAP1_HUMAN)	9.88	16.67	4	1	3	8	9.767E8	276	31.1	7.74
Q59GJ9	Acetyl-CoA carboxylase 2 variant (Fragment) OS=Homo sapiens PE=1 SV=1 - (Q59GJ9_HUMAN)	9.88	7.76	9	1	10	33	4.201E8	1689	192.2	6.60
A6QKW0	SHINC3 OS=Homo sapiens GN=SHINC3 PE=2 SV=1 - (A6QKW0_HUMAN)	9.86	25.10	3	1	5	8	4.697E7	247	27.9	9.70
E7EWF1	60S ribosomal protein L4 OS=Homo sapiens GN=RPL4 PE=4 SV=1 - (E7EWF1_HUMAN)	9.86	16.50	6	1	6	15	5.169E8	406	45.5	11.03
B3KPZ8	cDNA FLJ32530 fis, clone SMINT2000185, highly similar to TRANSKETOLASE (EC 2.2.1.1) OS=Homo sapiens PE=2 SV=1 - (B3KPZ8_HUMAN)	9.74	16.76	8	1	5	13	4.769E8	358	38.9	7.72
B3KTT6	cDNA FLJ38699 fis, clone KIDNE2002168, highly similar to Short chain 3-hydroxyacyl-CoA dehydrogenase, mitochondrial (EC 1.1.1.35) OS=Homo sapiens PE=2 SV=1 - (B3KTT6_HUMAN)	9.72	15.72	5	2	3	4	1.280E7	318	35.2	7.21
Q7Z3U5	Putative uncharacterized protein DKFZp686L0869 OS=Homo sapiens GN=DKFZp686L0869 PE=2 SV=1 - (Q7Z3U5_HUMAN)	9.72	8.86	6	2	10	15	1.129E8	1242	142.2	6.04
D6RA63	Protein GAPT (Fragment) OS=Homo sapiens GN=GAPT PE=4 SV=1 - (D6RA63_HUMAN)	9.70	35.29	3	1	3	7	1.935E8	68	7.6	11.80
C9JSL4	Prolyl 3-hydroxylase 2 (Fragment) OS=Homo sapiens GN=LEPREL1 PE=4 SV=1 - (C9JSL4_HUMAN)	9.67	8.21	3	1	1	10	8.667E7	134	15.8	4.81
Q15276-2	Isoform 2 of Rab GTPase-binding effector protein 1 OS=Homo sapiens GN=RABEP1 - (RABE1_HUMAN)	9.66	14.96	6	1	13	22	1.305E8	829	95.4	5.00
P30084	Enoyl-CoA hydratase, mitochondrial OS=Homo sapiens GN=ECHS1 PE=1 SV=4 - (ECHM_HUMAN)	9.61	14.48	1	2	3	3	5.742E6	290	31.4	8.07
H3BPC7	Inositol 1,4,5-trisphosphate receptor-interacting protein-like 2 (Fragment) OS=Homo sapiens GN=ITPRIPL2 PE=4 SV=2 - (H3BPC7_HUMAN)	9.59	28.35	2	1	3	6	3.723E8	127	13.5	11.80
Q5BKY2	EIF3H protein OS=Homo sapiens GN=EIF3H PE=2 SV=1 - (Q5BKY2_HUMAN)	9.57	11.75	11	1	4	8	5.815E6	349	39.6	6.39
Q99439-2	Isoform 2 of Calponin-2 OS=Homo sapiens GN=CNN2 - (CNN2_HUMAN)	9.56	16.30	8	1	2	3	1.011E8	270	29.5	6.99
Q92835-2	Isoform 2 of Phosphatidylinositol 3,4,5-trisphosphate 5-phosphatase 1 OS=Homo sapiens GN=INPP5D - (SHIP1_HUMAN)	9.51	7.15	8	1	9	19	4.750E8	1188	133.1	7.59
A2VDJ1	GOLGA6 protein (Fragment) OS=Homo sapiens GN=GOLGA6 PE=2 SV=1 - (A2VDJ1_HUMAN)	9.50	8.86	5	1	8	11	4.133E8	632	73.6	5.78
P48047	ATP synthase subunit O, mitochondrial OS=Homo sapiens GN=ATP5O PE=1 SV=1 - (ATPO_HUMAN)	9.47	19.72	4	3	4	5	3.324E7	213	23.3	9.96
E9PN51	NADH dehydrogenase (ubiquinone) iron-sulfur protein 8, mitochondrial (Fragment) OS=Homo sapiens GN=NDUFS8 PE=4 SV=1 - (E9PN51_HUMAN)	9.40	18.18	7	1	2	5	2.351E8	110	12.4	9.98
D6RF73	Aryl hydrocarbon receptor repressor OS=Homo sapiens GN=AHRR PE=4 SV=1 - (D6RF73_HUMAN)	9.32	6.76	5	1	3	7	9.939E8	547	59.4	8.57
Q14692	Ribosome biogenesis protein BMS1 homolog OS=Homo sapiens GN=BMS1 PE=1 SV=1 - (BMS1_HUMAN)	9.30	5.38	1	1	7	33	4.076E8	1282	145.7	6.44
Q9NXR1-2	Isoform 2 of Nuclear distribution protein nudE homolog 1 OS=Homo sapiens GN=NDE1 - (NDE1_HUMAN)	9.19	17.61	11	1	7	8	1.183E8	335	37.7	5.15
O00571-2	Isoform 2 of ATP-dependent RNA helicase DDX3X OS=Homo sapiens GN=DDX3X - (DDX3X_HUMAN)	9.18	14.24	9	3	8	14	1.213E8	646	71.3	6.62
I3L518	Cytosolic Fe-S cluster assembly factor NUBP1 (Fragment) OS=Homo sapiens GN=NUBP1 PE=4 SV=1 - (I3L518_HUMAN)	9.15	22.22	1	1	1	6	1.552E8	63	7.1	9.74
O60303	Uncharacterized protein KIAA0556 OS=Homo sapiens GN=KIAA0556 PE=1 SV=4 - (K0556_HUMAN)	9.12	6.61	2	1	12	19	3.279E8	1618	180.8	5.87
Q9NPP6	Immunoglobulin heavy chain variant (Fragment) OS=Homo sapiens PE=2 SV=1 - (Q9NPP6_HUMAN)	9.10	9.13	18	1	3	9	1.281E8	416	44.8	6.13
A7BKA4	ATP-binding cassette protein OS=Homo sapiens GN=ABCB5 PE=2 SV=1 - (A7BKA4_HUMAN)	9.09	4.53	5	1	6	13	1.217E8	1257	138.6	7.77
A8K865	cDNA FLJ75789, highly similar to Homo sapiens synaptophysin-like 1 (SYPL1), transcript variant 1, mRNA OS=Homo sapiens PE=2 SV=1 - (A8K865_HUMAN)	9.06	11.20	4	1	3	5	2.246E8	259	28.5	7.99
Q5HYM2	Putative uncharacterized protein DKFZp686O2462 (Fragment) OS=Homo sapiens GN=DKFZp686O2462 PE=2 SV=1 - (Q5HYM2_HUMAN)	9.06	13.58	4	2	8	9	1.053E8	626	72.0	5.36
D7PBN3	ESRP1/RAF1 fusion protein OS=Homo sapiens PE=2 SV=1 - (D7PBN3_HUMAN)	9.04	6.22	14	1	7	12	2.050E8	1061	118.5	8.22
P53621	Coatomer subunit alpha OS=Homo sapiens GN=COPA PE=1 SV=2 - (COPA_HUMAN)	9.01	4.49	2	1	8	14	8.596E7	1224	138.3	7.66
P63167	Dynein light chain 1, cytoplasmic OS=Homo sapiens GN=DYNLL1 PE=1 SV=1 - (DYL1_HUMAN)	9.01	39.33	4	5	6	7	5.767E7	89	10.4	7.40
A8K920	cDNA FLJ76953, highly similar to Homo sapiens plexin B3 (PLXNB3), mRNA OS=Homo sapiens PE=2 SV=1 - (A8K920_HUMAN)	8.96	2.30	6	1	4	15	6.120E8	1909	206.6	6.43
A8KA04	cDNA FLJ77539 OS=Homo sapiens PE=2 SV=1 - (A8KA04_HUMAN)	8.95	6.80	3	1	4	8	1.297E8	515	55.8	9.33
Q5VY36	Novel protein similar to ribosomal protein L13a RPL13A OS=Homo sapiens GN=RP11-365K22.1-001 PE=4 SV=1 - (Q5VY36_HUMAN)	8.89	21.18	7	1	5	14	4.808E7	203	23.4	10.77
P07814	Bifunctional glutamate/proline--tRNA ligase OS=Homo sapiens GN=EPRS PE=1 SV=5 - (SYEP_HUMAN)	8.88	8.80	1	1	13	24	2.843E8	1512	170.5	7.33
B7Z1I2	Aspartate aminotransferase OS=Homo sapiens GN=GOT1 PE=2 SV=1 - (B7Z1I2_HUMAN)	8.88	9.56	4	1	4	7	4.568E8	366	41.0	7.18
A6NKB8	Aminopeptidase B OS=Homo sapiens GN=RNPEP PE=4 SV=1 - (A6NKB8_HUMAN)	8.87	9.82	4	3	5	7	6.134E7	611	68.1	6.01
E9PRB8	26S protease regulatory subunit 6A OS=Homo sapiens GN=PSMC3 PE=4 SV=1 - (E9PRB8_HUMAN)	8.86	28.33	10	1	5	22	7.640E7	120	13.6	4.88
P05091	Aldehyde dehydrogenase, mitochondrial OS=Homo sapiens GN=ALDH2 PE=1 SV=2 - (ALDH2_HUMAN)	8.84	11.22	8	2	5	7	3.268E7	517	56.3	7.05
A0AVK6	Transcription factor E2F8 OS=Homo sapiens GN=E2F8 PE=1 SV=1 - (E2F8_HUMAN)	8.82	10.61	3	1	7	8	1.283E8	867	94.1	8.98
E7ESV6	Latrophilin-3 OS=Homo sapiens GN=LPHN3 PE=3 SV=1 - (E7ESV6_HUMAN)	8.82	4.23	19	1	7	17	3.511E8	1299	145.0	7.53
Q86XL3	Ankyrin repeat and LEM domain-containing protein 2 OS=Homo sapiens GN=ANKLE2 PE=1 SV=4 - (ANKL2_HUMAN)	8.80	6.29	4	1	5	7	4.883E7	938	104.0	7.09
B7Z7I4	cDNA FLJ54568, highly similar to T-complex protein 1 subunit eta OS=Homo sapiens PE=2 SV=1 - (B7Z7I4_HUMAN)	8.78	16.03	10	1	5	7	1.645E8	443	49.0	8.22
H0YDT6	Eukaryotic translation initiation factor 3 subunit F (Fragment) OS=Homo sapiens GN=EIF3F PE=4 SV=1 - (H0YDT6_HUMAN)	8.71	19.00	5	1	1	2	5.542E6	100	10.8	5.81
Q4ZG57	Putative uncharacterized protein MCM6 (Fragment) OS=Homo sapiens GN=MCM6 PE=2 SV=1 - (Q4ZG57_HUMAN)	8.71	10.19	3	1	7	10	2.911E8	785	88.9	5.60
B4DQZ2	cDNA FLJ50621, highly similar to DNA (cytosine-5)-methyltransferase-like protein 2 OS=Homo sapiens PE=2 SV=1 - (B4DQZ2_HUMAN)	8.69	11.29	7	1	3	7	7.713E7	310	35.6	7.40
O00562-2	Isoform 2 of Membrane-associated phosphatidylinositol transfer protein 1 OS=Homo sapiens GN=PITPNM1 - (PITM1_HUMAN)	8.67	5.15	3	1	6	12	5.775E8	1243	134.7	5.95
B7Z5N7	Sec1 family domain-containing protein 1 OS=Homo sapiens GN=SCFD1 PE=2 SV=1 - (B7Z5N7_HUMAN)	8.65	6.56	6	1	3	5	2.027E7	457	51.5	6.96
Q6ISF6	KRTAP9-2 protein (Fragment) OS=Homo sapiens GN=KRTAP9-2 PE=2 SV=1 - (Q6ISF6_HUMAN)	8.64	8.09	1	1	1	2	8.116E6	173	18.1	7.69
P49458	Signal recognition particle 9 kDa protein OS=Homo sapiens GN=SRP9 PE=1 SV=2 - (SRP09_HUMAN)	8.53	70.93	2	1	6	6	7.913E7	86	10.1	7.97
B4E054	cDNA FLJ58444, highly similar to Vacuolar ATP synthase subunit H (EC 3.6.3.14) OS=Homo sapiens PE=2 SV=1 - (B4E054_HUMAN)	8.52	4.81	5	1	2	10	3.254E8	457	52.7	6.74
A8K321	cDNA FLJ78524, highly similar to Homo sapiens SMILE protein (SMILE), mRNA OS=Homo sapiens PE=2 SV=1 - (A8K321_HUMAN)	8.48	7.01	4	1	7	16	4.474E8	899	102.0	8.84
Q6ZWJ8	Kielin/chordin-like protein OS=Homo sapiens GN=KCP PE=2 SV=2 - (KCP_HUMAN)	8.43	1.93	1	1	3	14	3.307E8	1503	159.8	5.74
B3KVK3	cDNA FLJ16666 fis, clone THYMU2032732, highly similar to Forkhead box protein D4-like 1 OS=Homo sapiens PE=2 SV=1 - (B3KVK3_HUMAN)	8.43	4.90	2	1	2	5	1.520E7	408	43.6	9.38
Q9NYV4	Cyclin-dependent kinase 12 OS=Homo sapiens GN=CDK12 PE=1 SV=2 - (CDK12_HUMAN)	8.38	7.58	176	1	12	18	2.302E8	1490	164.1	9.44
B1AK88	Capping protein (Actin filament) muscle Z-line, beta OS=Homo sapiens GN=CAPZB PE=4 SV=1 - (B1AK88_HUMAN)	8.35	11.63	8	1	4	5	2.102E7	301	33.8	6.43
C5H6M8	Cytochrome c oxidase subunit 2 OS=Homo sapiens GN=COII PE=3 SV=1 - (C5H6M8_HUMAN)	8.30	11.89	175	2	3	5	4.092E7	227	25.6	4.82
Q9NS56-2	Isoform 2 of E3 ubiquitin-protein ligase Topors OS=Homo sapiens GN=TOPORS - (TOPRS_HUMAN)	8.28	9.49	2	1	12	17	2.385E8	980	112.3	9.41
Q8IZH2-2	Isoform 2 of 5'-3' exoribonuclease 1 OS=Homo sapiens GN=XRN1 - (XRN1_HUMAN)	8.26	5.61	3	1	8	13	9.026E7	1694	192.7	7.30
B5B2P0	Nuclear factor of activated T-cells c2 isoform IA-IIS-Xa OS=Homo sapiens GN=NFATC2 PE=2 SV=1 - (B5B2P0_HUMAN)	8.23	1.85	11	1	2	31	1.121E7	702	76.7	8.46
B2R7C2	cDNA, FLJ93375, highly similar to Homo sapiens ZW10, kinetochore associated, homolog (Drosophila) (ZW10), mRNA OS=Homo sapiens PE=2 SV=1 - (B2R7C2_HUMAN)	8.23	5.13	4	1	4	6	4.927E7	779	88.8	6.27
P28066	Proteasome subunit alpha type-5 OS=Homo sapiens GN=PSMA5 PE=1 SV=3 - (PSA5_HUMAN)	8.21	15.77	3	3	3	3	6.830E7	241	26.4	4.79
Q7Z3C0	Putative uncharacterized protein DKFZp686A04192 (Fragment) OS=Homo sapiens GN=DKFZp686A04192 PE=2 SV=1 - (Q7Z3C0_HUMAN)	8.19	10.54	2	1	5	12	1.057E8	626	70.2	9.23
A6NDW3	Lipolysis-stimulated lipoprotein receptor OS=Homo sapiens GN=LSR PE=4 SV=1 - (A6NDW3_HUMAN)	8.13	14.29	5	1	11	14	7.041E7	581	64.0	8.78
I3L0T4	Peroxisomal acyl-coenzyme A oxidase 1 OS=Homo sapiens GN=ACOX1 PE=4 SV=1 - (I3L0T4_HUMAN)	8.12	17.04	1	1	4	7	7.126E7	223	25.9	9.19
B4DGP8	cDNA FLJ55574, highly similar to Calnexin OS=Homo sapiens PE=2 SV=1 - (B4DGP8_HUMAN)	8.11	7.34	3	1	4	6	9.980E7	627	71.5	4.70
E9PE28	X-linked retinitis pigmentosa GTPase regulator OS=Homo sapiens GN=RPGR PE=4 SV=1 - (E9PE28_HUMAN)	8.08	5.64	3	1	7	21	1.618E8	1152	127.0	4.23
B2R8E1	Ran-binding protein 9 OS=Homo sapiens GN=RANBP9 PE=2 SV=1 - (B2R8E1_HUMAN)	8.03	10.00	3	1	4	8	1.583E8	500	55.0	6.32
E5KNB2	DNA-apurinic or apyrimidinic site lyase 2 OS=Homo sapiens PE=4 SV=1 - (E5KNB2_HUMAN)	7.99	1.93	3	1	1	6	2.922E8	518	57.4	8.29
E9PPP7	Putative uncharacterized protein C8orf73 (Fragment) OS=Homo sapiens GN=C8orf73 PE=4 SV=1 - (E9PPP7_HUMAN)	7.96	13.48	1	1	6	40	1.112E8	549	56.2	9.79
A6NCD2	T-complex protein 1 subunit zeta OS=Homo sapiens GN=CCT6A PE=3 SV=1 - (A6NCD2_HUMAN)	7.95	10.70	7	1	6	6	1.118E8	486	53.3	7.27
Q9BYR7	Keratin-associated protein 3-2 OS=Homo sapiens GN=KRTAP3-2 PE=1 SV=1 - (KRA32_HUMAN)	7.94	17.35	1	1	1	2	1.883E8	98	10.4	5.69
B4DND6	cDNA FLJ55804, highly similar to COP9 signalosome complex subunit 1 OS=Homo sapiens PE=2 SV=1 - (B4DND6_HUMAN)	7.94	13.66	8	1	6	9	1.057E8	483	54.6	6.90
Q5T0N5-2	Isoform 2 of Formin-binding protein 1-like OS=Homo sapiens GN=FNBP1L - (FBP1L_HUMAN)	7.89	10.00	8	1	7	14	4.216E7	600	69.4	6.55
A4D160	Serine/threonine kinase 31 OS=Homo sapiens GN=STK31 PE=4 SV=1 - (A4D160_HUMAN)	7.88	4.82	7	1	4	4	6.537E7	996	113.2	5.14
P30086	Phosphatidylethanolamine-binding protein 1 OS=Homo sapiens GN=PEBP1 PE=1 SV=3 - (PEBP1_HUMAN)	7.84	14.97	2	2	2	2	2.952E7	187	21.0	7.53
B2RBR9	cDNA, FLJ95650, highly similar to Homo sapiens karyopherin (importin) beta 1 (KPNB1), mRNA OS=Homo sapiens PE=2 SV=1 - (B2RBR9_HUMAN)	7.83	3.88	6	1	4	4	1.737E8	876	97.1	4.78
Q53T70	Putative uncharacterized protein RAB10 (Fragment) OS=Homo sapiens GN=RAB10 PE=2 SV=1 - (Q53T70_HUMAN)	7.77	31.85	86	2	4	4	3.071E7	157	17.8	8.25
Q15102	Platelet-activating factor acetylhydrolase IB subunit gamma OS=Homo sapiens GN=PAFAH1B3 PE=1 SV=1 - (PA1B3_HUMAN)	7.76	15.15	1	2	4	5	5.400E7	231	25.7	6.84
Q5T5C0-3	Isoform 3 of Syntaxin-binding protein 5 OS=Homo sapiens GN=STXBP5 - (STXB5_HUMAN)	7.63	5.28	7	1	5	7	2.761E8	1098	121.5	6.96
H3BNA1	Ubiquitin carboxyl-terminal hydrolase 10 (Fragment) OS=Homo sapiens GN=USP10 PE=4 SV=1 - (H3BNA1_HUMAN)	7.62	21.33	1	1	3	6	1.206E8	150	17.0	4.58
Q9BYR6	Keratin-associated protein 3-3 OS=Homo sapiens GN=KRTAP3-3 PE=1 SV=1 - (KRA33_HUMAN)	7.54	17.35	1	1	1	2	1.213E8	98	10.4	5.69
Q96HX3	Similar to ribophorin I (Fragment) OS=Homo sapiens PE=2 SV=1 - (Q96HX3_HUMAN)	7.52	7.92	7	1	4	7	1.819E8	568	64.5	6.55
B0VJZ1	Leukocyte receptor cluster (LRC) member 9 OS=Homo sapiens GN=LENG9 PE=4 SV=1 - (B0VJZ1_HUMAN)	7.52	9.41	2	1	4	7	1.991E8	478	50.7	7.50
Q9UNX4	WD repeat-containing protein 3 OS=Homo sapiens GN=WDR3 PE=1 SV=1 - (WDR3_HUMAN)	7.50	7.74	3	1	8	29	5.477E8	943	106.0	6.64
C9J1Z8	ADP-ribosylation factor 5 (Fragment) OS=Homo sapiens GN=ARF5 PE=3 SV=1 - (C9J1Z8_HUMAN)	7.48	20.67	9	1	4	4	1.556E8	150	17.1	7.34
B4DII5	Importin subunit alpha OS=Homo sapiens PE=2 SV=1 - (B4DII5_HUMAN)	7.47	3.00	5	1	2	6	7.894E7	533	59.6	5.01
E5KSX8	Mitochondrial transcription factor A OS=Homo sapiens PE=4 SV=1 - (E5KSX8_HUMAN)	7.46	20.73	4	1	4	5	3.824E7	246	29.1	9.72
Q9NVM6	DnaJ homolog subfamily C member 17 OS=Homo sapiens GN=DNAJC17 PE=1 SV=1 - (DJC17_HUMAN)	7.46	19.08	1	1	6	16	7.045E8	304	34.7	8.53
P40939	Trifunctional enzyme subunit alpha, mitochondrial OS=Homo sapiens GN=HADHA PE=1 SV=2 - (ECHA_HUMAN)	7.46	6.16	6	2	3	3	2.194E7	763	82.9	9.04
Q9HDC9	Adipocyte plasma membrane-associated protein OS=Homo sapiens GN=APMAP PE=1 SV=2 - (APMAP_HUMAN)	7.46	5.05	2	1	2	8	1.234E8	416	46.5	6.16
P62273	40S ribosomal protein S29 OS=Homo sapiens GN=RPS29 PE=1 SV=2 - (RS29_HUMAN)	7.45	39.29	2	2	3	7	4.722E7	56	6.7	10.13
B7Z6B8	2,4-dienoyl-CoA reductase, mitochondrial OS=Homo sapiens GN=DECR1 PE=2 SV=1 - (B7Z6B8_HUMAN)	7.41	15.64	6	1	3	4	2.096E7	326	35.0	8.90
F5H5L3	Ankyrin repeat domain-containing protein 36A OS=Homo sapiens GN=ANKRD36 PE=4 SV=1 - (F5H5L3_HUMAN)	7.36	12.17	1	1	15	21	1.984E8	1208	135.6	8.78
B3KMX0	cDNA FLJ12837 fis, clone NT2RP2003228, highly similar to DNA replication licensing factor MCM4 OS=Homo sapiens PE=2 SV=1 - (B3KMX0_HUMAN)	7.33	8.57	4	1	7	12	2.801E8	863	96.5	6.74
Q08AD1-2	Isoform 2 of Calmodulin-regulated spectrin-associated protein 2 OS=Homo sapiens GN=CAMSAP2 - (CAMP2_HUMAN)	7.31	7.39	4	1	9	11	6.711E7	1462	165.0	6.71
P06703	Protein S100-A6 OS=Homo sapiens GN=S100A6 PE=1 SV=1 - (S10A6_HUMAN)	7.30	16.67	1	2	3	4	1.751E8	90	10.2	5.48
Q9ULW8	Protein-arginine deiminase type-3 OS=Homo sapiens GN=PADI3 PE=1 SV=2 - (PADI3_HUMAN)	7.30	6.02	3	2	3	3	4.329E7	664	74.7	5.54
P14174	Macrophage migration inhibitory factor OS=Homo sapiens GN=MIF PE=1 SV=4 - (MIF_HUMAN)	7.30	13.91	2	2	2	6	2.886E8	115	12.5	7.88
P42766	60S ribosomal protein L35 OS=Homo sapiens GN=RPL35 PE=1 SV=2 - (RL35_HUMAN)	7.27	39.84	4	2	5	7	6.418E7	123	14.5	11.05
Q9P1F3	Costars family protein ABRACL OS=Homo sapiens GN=ABRACL PE=1 SV=1 - (ABRAL_HUMAN)	7.26	27.16	1	3	4	4	1.270E7	81	9.1	6.29
Q9NTN9	Semaphorin-4G OS=Homo sapiens GN=SEMA4G PE=2 SV=1 - (SEM4G_HUMAN)	7.18	5.13	3	1	4	8	4.317E8	838	91.4	7.84
Q8IZI4	B-lymphocyte stimulator (Fragment) OS=Homo sapiens GN=TNFSF13B PE=2 SV=1 - (Q8IZI4_HUMAN)	7.18	6.33	5	1	1	4	1.846E6	158	17.8	5.59
E5RI90	Uncharacterized protein C1orf198 (Fragment) OS=Homo sapiens GN=C1orf198 PE=4 SV=1 - (E5RI90_HUMAN)	7.15	21.25	5	1	4	7	3.618E7	160	17.7	5.27
Q9UBI6	Guanine nucleotide-binding protein G(I)/G(S)/G(O) subunit gamma-12 OS=Homo sapiens GN=GNG12 PE=1 SV=3 - (GBG12_HUMAN)	7.13	30.56	2	2	3	4	8.977E6	72	8.0	8.97
Q16832	Discoidin domain-containing receptor 2 OS=Homo sapiens GN=DDR2 PE=1 SV=2 - (DDR2_HUMAN)	7.11	12.16	2	1	7	19	9.410E7	855	96.7	5.36
C9J4N6	Isocitrate dehydrogenase (NADP) cytoplasmic (Fragment) OS=Homo sapiens GN=IDH1 PE=3 SV=1 - (C9J4N6_HUMAN)	7.08	28.66	7	1	4	5	1.804E7	157	17.7	8.38
B7Z589	Transporter OS=Homo sapiens GN=SLC6A9 PE=2 SV=1 - (B7Z589_HUMAN)	7.02	1.34	5	1	1	4	6.622E7	522	58.3	7.02
Q495G0	V-myb myeloblastosis viral oncogene homolog (Avian)-like 1 OS=Homo sapiens GN=MYBL1 PE=2 SV=1 - (Q495G0_HUMAN)	7.00	11.58	3	1	6	9	1.675E9	691	78.8	6.68
B2RCY5	cDNA, FLJ96373, highly similar to Homo sapiens X-ray repair complementing defective repair in Chinese hamster cells 1 (XRCC1), mRNA OS=Homo sapiens PE=2 SV=1 - (B2RCY5_HUMAN)	6.99	6.00	6	1	4	7	4.481E7	633	69.5	6.34
O95976	Immunoglobulin superfamily member 6 OS=Homo sapiens GN=IGSF6 PE=2 SV=2 - (IGSF6_HUMAN)	6.99	9.13	1	1	2	5	1.062E8	241	27.0	8.65
P48729	Casein kinase I isoform alpha OS=Homo sapiens GN=CSNK1A1 PE=1 SV=2 - (KC1A_HUMAN)	6.98	15.13	10	2	5	5	7.582E7	337	38.9	9.57
B7Z254	Protein disulfide-isomerase A6 OS=Homo sapiens GN=PDIA6 PE=2 SV=1 - (B7Z254_HUMAN)	6.95	9.61	7	2	4	6	7.541E8	437	47.8	5.08
Q86VP6-2	Isoform 2 of Cullin-associated NEDD8-dissociated protein 1 OS=Homo sapiens GN=CAND1 - (CAND1_HUMAN)	6.94	6.50	5	2	7	9	3.228E8	1062	117.8	5.69
Q59H46	Integrin beta (Fragment) OS=Homo sapiens PE=2 SV=1 - (Q59H46_HUMAN)	6.93	5.28	8	1	9	13	1.263E8	1515	169.0	6.49
H7BZW3	Voltage-dependent calcium channel subunit alpha-2-3 OS=Homo sapiens GN=CACNA2D3 PE=4 SV=1 - (H7BZW3_HUMAN)	6.86	5.21	1	1	2	9	3.501E8	518	59.4	5.86
O60716-24	Isoform 3 of Catenin delta-1 OS=Homo sapiens GN=CTNND1 - (CTND1_HUMAN)	6.83	8.25	38	1	7	14	5.873E7	812	90.3	7.03
O75820-3	Isoform 3 of Zinc finger protein 189 OS=Homo sapiens GN=ZNF189 - (ZN189_HUMAN)	6.73	4.52	6	1	4	12	3.567E8	531	62.0	9.09
Q13283	Ras GTPase-activating protein-binding protein 1 OS=Homo sapiens GN=G3BP1 PE=1 SV=1 - (G3BP1_HUMAN)	6.70	13.73	9	2	6	29	3.872E8	466	52.1	5.52
B3KP61	RAC-beta serine/threonine-protein kinase OS=Homo sapiens GN=AKT2 PE=2 SV=1 - (B3KP61_HUMAN)	6.67	6.81	1	1	3	4	2.176E8	382	43.9	6.18
Q32XH3	Ribosomal protein L18a-like protein OS=Homo sapiens PE=2 SV=1 - (Q32XH3_HUMAN)	6.67	25.74	7	2	4	5	4.655E7	136	15.8	10.27
B4DFL2	Isocitrate dehydrogenase (NADP) OS=Homo sapiens GN=IDH2 PE=2 SV=1 - (B4DFL2_HUMAN)	6.66	14.00	7	2	5	7	1.662E7	400	45.2	7.75
E9PAV3	Nascent polypeptide-associated complex subunit alpha OS=Homo sapiens GN=NACA PE=4 SV=1 - (E9PAV3_HUMAN)	6.64	2.79	9	1	5	8	1.883E8	2078	205.3	9.58
Q8WVV4	Protein POF1B OS=Homo sapiens GN=POF1B PE=1 SV=3 - (POF1B_HUMAN)	6.62	7.64	4	1	5	8	5.264E7	589	68.0	6.32
P42677	40S ribosomal protein S27 OS=Homo sapiens GN=RPS27 PE=1 SV=3 - (RS27_HUMAN)	6.59	25.00	7	2	2	3	3.567E7	84	9.5	9.45
F5GYN4	Ubiquitin thioesterase OTUB1 OS=Homo sapiens GN=OTUB1 PE=4 SV=1 - (F5GYN4_HUMAN)	6.53	16.60	10	2	3	4	4.353E7	241	28.0	5.29
D3YTB1	60S ribosomal protein L32 (Fragment) OS=Homo sapiens GN=RPL32 PE=4 SV=1 - (D3YTB1_HUMAN)	6.50	12.03	3	1	2	3	2.421E7	133	15.6	11.44
P49411	Elongation factor Tu, mitochondrial OS=Homo sapiens GN=TUFM PE=1 SV=2 - (EFTU_HUMAN)	6.49	11.28	1	1	4	4	2.288E7	452	49.5	7.61
A8K646	cDNA FLJ75699, highly similar to Homo sapiens osteoclast stimulating factor 1 (OSTF1), mRNA OS=Homo sapiens PE=2 SV=1 - (A8K646_HUMAN)	6.49	9.35	2	1	2	3	5.900E6	214	23.7	5.68
Q8IV16	Glycosylphosphatidylinositol-anchored high density lipoprotein-binding protein 1 OS=Homo sapiens GN=GPIHBP1 PE=1 SV=2 - (HDBP1_HUMAN)	6.46	3.26	1	1	1	4	3.066E8	184	19.8	4.79
B7Z6Q1	cDNA FLJ51102, highly similar to DEP domain-containing protein 5 OS=Homo sapiens PE=2 SV=1 - (B7Z6Q1_HUMAN)	6.45	8.13	13	2	5	24	5.317E7	664	75.2	8.18
B7ZKX2	Putative uncharacterized protein OS=Homo sapiens PE=2 SV=1 - (B7ZKX2_HUMAN)	6.43	7.36	2	1	7	7	4.395E7	1019	117.6	9.38
Q15406-3	Isoform 3 of Nuclear receptor subfamily 6 group A member 1 OS=Homo sapiens GN=NR6A1 - (NR6A1_HUMAN)	6.38	7.71	5	1	5	7	5.174E7	454	52.1	6.47
P22079	Lactoperoxidase OS=Homo sapiens GN=LPO PE=1 SV=2 - (PERL_HUMAN)	6.33	10.25	3	1	7	19	5.977E8	712	80.2	8.62
O95147	Dual specificity protein phosphatase 14 OS=Homo sapiens GN=DUSP14 PE=1 SV=1 - (DUS14_HUMAN)	6.29	26.77	1	1	4	4	7.168E6	198	22.2	9.57
F8W6V3	Protein TSPEAR OS=Homo sapiens GN=TSPEAR PE=4 SV=1 - (F8W6V3_HUMAN)	6.25	6.84	4	1	3	5	2.726E8	599	66.8	6.73
B2R9T9	cDNA, FLJ94551 OS=Homo sapiens PE=2 SV=1 - (B2R9T9_HUMAN)	6.24	12.35	4	1	3	4	1.037E7	243	26.2	10.67
Q2NKY6	Putative uncharacterized protein OS=Homo sapiens PE=2 SV=1 - (Q2NKY6_HUMAN)	6.22	17.19	5	1	3	4	4.887E7	192	21.7	10.07
Q9BX68	Histidine triad nucleotide-binding protein 2, mitochondrial OS=Homo sapiens GN=HINT2 PE=1 SV=1 - (HINT2_HUMAN)	6.17	18.40	2	1	4	7	5.711E7	163	17.2	9.16
Q5SQ08	Complement component 8, gamma polypeptide (Fragment) OS=Homo sapiens GN=C8G PE=2 SV=1 - (Q5SQ08_HUMAN)	6.15	17.07	2	1	3	6	2.108E7	164	17.5	9.79
E9PN17	ATP synthase subunit g, mitochondrial OS=Homo sapiens GN=ATP5L PE=4 SV=1 - (E9PN17_HUMAN)	6.08	36.84	2	2	2	2	5.684E7	76	8.4	10.29
A8K8S6	cDNA FLJ77231, highly similar to Homo sapiens hemogen (HEMGN), transcript variant 1, mRNA OS=Homo sapiens PE=2 SV=1 - (A8K8S6_HUMAN)	5.94	4.75	2	1	3	7	2.817E8	484	55.3	4.87
P62314	Small nuclear ribonucleoprotein Sm D1 OS=Homo sapiens GN=SNRPD1 PE=1 SV=1 - (SMD1_HUMAN)	5.89	25.21	2	1	2	2	3.780E7	119	13.3	11.56
P60985-2	Isoform 2 of Keratinocyte differentiation-associated protein OS=Homo sapiens GN=KRTDAP - (KTDAP_HUMAN)	5.88	14.12	2	1	1	2	9.987E6	85	9.4	6.54
Q86YF9-2	Isoform 2 of Zinc finger protein DZIP1 OS=Homo sapiens GN=DZIP1 - (DZIP1_HUMAN)	5.87	7.08	2	1	7	9	1.799E8	848	96.6	6.20
P13489	Ribonuclease inhibitor OS=Homo sapiens GN=RNH1 PE=1 SV=2 - (RINI_HUMAN)	5.86	12.80	4	1	5	7	1.105E8	461	49.9	4.82
P60369	Keratin-associated protein 10-3 OS=Homo sapiens GN=KRTAP10-3 PE=1 SV=2 - (KR103_HUMAN)	5.81	5.88	1	1	1	2	1.200E7	221	22.3	7.55
P27635	60S ribosomal protein L10 OS=Homo sapiens GN=RPL10 PE=1 SV=4 - (RL10_HUMAN)	5.80	15.89	8	4	4	7	3.336E7	214	24.6	10.08
Q0VDC6	Peptidyl-prolyl cis-trans isomerase OS=Homo sapiens GN=FKBP1A PE=2 SV=1 - (Q0VDC6_HUMAN)	5.79	25.52	4	1	3	5	1.271E8	145	15.7	9.17
H3BLU7	Aflatoxin B1 aldehyde reductase member 2 (Fragment) OS=Homo sapiens GN=AKR7A2 PE=4 SV=1 - (H3BLU7_HUMAN)	5.79	11.46	3	1	3	3	2.625E7	314	34.7	7.18
A8K5N5	cDNA FLJ78142, highly similar to Homo sapiens transglutaminase 1 (K polypeptide epidermal type I, protein-glutamine-gamma-glutamyltransferase), mRNA OS=Homo sapiens PE=2 SV=1 - (A8K5N5_HUMAN)	5.74	9.67	6	1	6	7	1.796E8	817	89.7	6.04
E2QRG3	Kinesin-like protein KIF6 OS=Homo sapiens GN=KIF6 PE=3 SV=2 - (E2QRG3_HUMAN)	5.74	9.80	12	1	5	9	4.883E7	653	74.2	7.12
Q92688-2	Isoform 2 of Acidic leucine-rich nuclear phosphoprotein 32 family member B OS=Homo sapiens GN=ANP32B - (AN32B_HUMAN)	5.70	24.10	10	2	6	7	5.209E7	195	22.3	4.30
F8VR50	Actin-related protein 2/3 complex subunit 3 (Fragment) OS=Homo sapiens GN=ARPC3 PE=4 SV=1 - (F8VR50_HUMAN)	5.70	13.10	6	1	1	2	4.312E7	84	9.7	7.93
Q1JQ76	Ribosomal protein (Fragment) OS=Homo sapiens GN=RPL10A PE=2 SV=1 - (Q1JQ76_HUMAN)	5.67	19.42	2	1	4	6	1.875E7	206	23.5	10.07
Q5T949	Protein phosphatase 2A activator, regulatory subunit 4 (Fragment) OS=Homo sapiens GN=PPP2R4 PE=2 SV=1 - (Q5T949_HUMAN)	5.66	10.00	12	1	3	10	6.426E7	250	28.1	5.40
G5E9R3	60S ribosomal protein L37a OS=Homo sapiens GN=RPL37A PE=4 SV=1 - (G5E9R3_HUMAN)	5.62	41.38	6	1	3	3	1.707E7	58	6.6	10.45
Q13946-2	Isoform PDE7A2 of High affinity cAMP-specific 3',5'-cyclic phosphodiesterase 7A OS=Homo sapiens GN=PDE7A - (PDE7A_HUMAN)	5.61	4.61	2	1	3	5	1.286E8	456	52.7	7.50
P49748-2	Isoform 2 of Very long-chain specific acyl-CoA dehydrogenase, mitochondrial OS=Homo sapiens GN=ACADVL - (ACADV_HUMAN)	5.60	7.27	8	1	5	10	1.223E8	633	68.0	8.56
Q9UIV8-2	Isoform 2 of Serpin B13 OS=Homo sapiens GN=SERPINB13 - (SPB13_HUMAN)	5.56	10.03	11	3	5	5	1.937E7	339	38.4	5.81
P18827	Syndecan-1 OS=Homo sapiens GN=SDC1 PE=1 SV=3 - (SDC1_HUMAN)	5.56	9.68	5	1	2	2	1.204E8	310	32.4	4.63
Q5H8C1	FRAS1-related extracellular matrix protein 1 OS=Homo sapiens GN=FREM1 PE=1 SV=3 - (FREM1_HUMAN)	5.51	2.48	4	1	5	6	2.486E8	2179	244.0	5.90
H7BYH4	Superoxide dismutase (Cu-Zn) OS=Homo sapiens GN=SOD1 PE=3 SV=1 - (H7BYH4_HUMAN)	5.46	36.30	2	1	2	3	5.571E7	135	13.9	6.11
D6RBW1	Eukaryotic translation initiation factor 4E OS=Homo sapiens GN=EIF4E PE=3 SV=1 - (D6RBW1_HUMAN)	5.46	10.61	7	1	4	5	5.034E7	245	28.5	8.12
Q14651	Plastin-1 OS=Homo sapiens GN=PLS1 PE=1 SV=2 - (PLSI_HUMAN)	5.46	6.84	10	1	4	6	8.547E7	629	70.2	5.41
B4DU58	Macrophage-capping protein OS=Homo sapiens GN=CAPG PE=2 SV=1 - (B4DU58_HUMAN)	5.45	13.15	5	1	4	10	1.594E7	327	36.2	6.25
Q3LI83	Keratin-associated protein 24-1 OS=Homo sapiens GN=KRTAP24-1 PE=1 SV=1 - (KR241_HUMAN)	5.45	9.06	1	1	2	3	4.333E7	254	27.7	8.29
C5IX07	Tumor-associated calcium signal transducer 2 OS=Homo sapiens GN=TACSTD2 PE=4 SV=1 - (C5IX07_HUMAN)	5.43	11.15	2	1	5	6	3.349E7	323	35.7	8.87
Q59FM5	Guanine nucleotide-binding protein G, alpha subunit variant (Fragment) OS=Homo sapiens PE=2 SV=1 - (Q59FM5_HUMAN)	5.43	7.00	2	1	3	4	1.740E7	357	41.9	5.69
Q06323	Proteasome activator complex subunit 1 OS=Homo sapiens GN=PSME1 PE=1 SV=1 - (PSME1_HUMAN)	5.42	20.88	7	3	5	5	2.105E7	249	28.7	6.02
Q6UWL6-3	Isoform 3 of Kin of IRRE-like protein 2 OS=Homo sapiens GN=KIRREL2 - (KIRR2_HUMAN)	5.41	4.97	4	1	3	6	5.162E7	583	61.7	6.74
B4DIX5	Transient receptor potential cation channel subfamily M member 4 OS=Homo sapiens GN=TRPM4 PE=2 SV=1 - (B4DIX5_HUMAN)	5.38	8.37	4	1	7	10	5.328E8	860	95.5	8.41
B4DXN1	cDNA FLJ53236, highly similar to Homo sapiens IQ motif containing E (IQCE), mRNA OS=Homo sapiens PE=2 SV=1 - (B4DXN1_HUMAN)	5.36	10.52	14	1	8	11	2.107E8	637	71.1	9.38
P30046	D-dopachrome decarboxylase OS=Homo sapiens GN=DDT PE=1 SV=3 - (DOPD_HUMAN)	5.34	29.66	7	2	3	3	1.574E8	118	12.7	7.30
E9PPC4	E3 ubiquitin-protein ligase RAG1 OS=Homo sapiens GN=RAG1 PE=4 SV=1 - (E9PPC4_HUMAN)	5.32	5.16	3	1	6	9	4.847E6	931	106.1	8.75
A4D2P2	Ras-related C3 botulinum toxin substrate 1 (Rho family, small GTP binding protein Rac1) OS=Homo sapiens GN=RAC1 PE=3 SV=1 - (A4D2P2_HUMAN)	5.26	16.22	3	1	3	4	4.389E7	148	16.8	8.91
Q6NUJ1	Proactivator polypeptide-like 1 OS=Homo sapiens GN=PSAPL1 PE=2 SV=2 - (SAPL1_HUMAN)	5.22	7.10	1	1	3	3	6.741E7	521	56.6	7.27
P47870	Gamma-aminobutyric acid receptor subunit beta-2 OS=Homo sapiens GN=GABRB2 PE=1 SV=2 - (GBRB2_HUMAN)	5.22	13.67	18	1	6	13	2.346E8	512	59.1	9.36
O75582-2	Isoform 2 of Ribosomal protein S6 kinase alpha-5 OS=Homo sapiens GN=RPS6KA5 - (KS6A5_HUMAN)	5.17	4.74	3	1	3	7	7.719E7	549	61.7	6.90
H0YKT8	Proteasome subunit beta type (Fragment) OS=Homo sapiens GN=PSMA4 PE=3 SV=1 - (H0YKT8_HUMAN)	5.14	9.89	13	2	2	2	3.728E7	182	20.1	6.76
A8K8F9	cDNA FLJ75064, highly similar to Homo sapiens phospholipase C, delta 1 (PLCD1), mRNA OS=Homo sapiens PE=2 SV=1 - (A8K8F9_HUMAN)	5.12	6.35	6	1	5	7	7.163E7	756	85.6	6.70
F8WC88	Cyclin-dependent kinase-like 4 OS=Homo sapiens GN=CDKL4 PE=4 SV=1 - (F8WC88_HUMAN)	5.12	36.21	3	1	3	5	4.559E7	58	6.5	9.36
O75828	Carbonyl reductase (NADPH) 3 OS=Homo sapiens GN=CBR3 PE=1 SV=3 - (CBR3_HUMAN)	5.10	9.75	2	1	3	6	3.408E8	277	30.8	6.18
B0V2L2	HLA-B associated transcript 1 (Fragment) OS=Homo sapiens GN=BAT1 PE=4 SV=1 - (B0V2L2_HUMAN)	5.07	14.51	34	2	6	13	8.846E7	324	37.9	8.95
P49961-2	Isoform Placental I of Ectonucleoside triphosphate diphosphohydrolase 1 OS=Homo sapiens GN=ENTPD1 - (ENTP1_HUMAN)	5.06	8.51	6	1	4	8	2.018E8	517	58.7	6.74
D6RBK0	Prohibitin (Fragment) OS=Homo sapiens GN=PHB PE=4 SV=1 - (D6RBK0_HUMAN)	5.05	9.68	10	1	1	2	1.061E8	124	13.7	9.36
A8K6Q5	cDNA FLJ77327, highly similar to Homo sapiens cyclin H (CCNH), mRNA OS=Homo sapiens PE=2 SV=1 - (A8K6Q5_HUMAN)	5.05	16.10	5	1	4	5	2.481E7	323	37.6	7.15
Q9BYD5	Cornifelin OS=Homo sapiens GN=CNFN PE=1 SV=2 - (CNFN_HUMAN)	5.00	19.64	1	1	1	1	1.310E8	112	12.4	6.10
H0Y9D8	Inorganic pyrophosphatase 2, mitochondrial (Fragment) OS=Homo sapiens GN=PPA2 PE=4 SV=1 - (H0Y9D8_HUMAN)	4.98	13.72	13	1	4	13	9.358E7	226	25.6	7.77
Q6P6C2-2	Isoform 2 of Probable alpha-ketoglutarate-dependent dioxygenase ABH5 OS=Homo sapiens GN=ALKBH5 - (ALKB5_HUMAN)	4.96	23.86	4	1	9	15	1.232E8	394	44.2	9.09
B2RDB6	cDNA, FLJ96540, highly similar to Homo sapiens zinc finger protein 3 (A8-51) (ZNF3), transcript variant 2, mRNA OS=Homo sapiens PE=2 SV=1 - (B2RDB6_HUMAN)	4.89	5.37	9	1	3	5	1.934E7	410	47.1	8.32
P22314	Ubiquitin-like modifier-activating enzyme 1 OS=Homo sapiens GN=UBA1 PE=1 SV=3 - (UBA1_HUMAN)	4.88	3.97	3	1	4	5	2.014E7	1058	117.8	5.76
Q96AU7	Thymic stromal lymphopoietin OS=Homo sapiens GN=TSLP PE=2 SV=1 - (Q96AU7_HUMAN)	4.86	31.67	3	1	1	2	1.128E8	60	7.1	11.09
D6RGH1	Uncharacterized protein OS=Homo sapiens PE=4 SV=1 - (D6RGH1_HUMAN)	4.85	13.46	1	1	2	5	4.896E7	156	17.5	8.19
I3L4A2	Leucine carboxyl methyltransferase 1 (Fragment) OS=Homo sapiens GN=LCMT1 PE=4 SV=1 - (I3L4A2_HUMAN)	4.80	15.82	3	1	2	2	1.012E8	158	18.2	5.60
Q53TD0	Putative uncharacterized protein SP100 (Fragment) OS=Homo sapiens GN=SP100 PE=2 SV=1 - (Q53TD0_HUMAN)	4.80	8.78	11	1	8	12	1.053E8	843	96.5	8.31
B2RAZ4	cDNA, FLJ95208, highly similar to Homo sapiens PYD and CARD domain containing (PYCARD), transcript variant 1, mRNA OS=Homo sapiens PE=2 SV=1 - (B2RAZ4_HUMAN)	4.78	20.00	4	1	3	4	7.011E6	195	21.6	5.64
H0YN94	Protein FAM81A (Fragment) OS=Homo sapiens GN=FAM81A PE=4 SV=1 - (H0YN94_HUMAN)	4.75	23.15	1	1	3	3	7.514E7	108	12.6	8.88
P84103	Serine/arginine-rich splicing factor 3 OS=Homo sapiens GN=SRSF3 PE=1 SV=1 - (SRSF3_HUMAN)	4.74	20.73	2	1	3	8	1.920E7	164	19.3	11.65
Q5T946	Glyoxylate reductase/hydroxypyruvate reductase OS=Homo sapiens GN=GRHPR PE=2 SV=1 - (Q5T946_HUMAN)	4.71	10.89	3	1	4	5	6.593E7	395	43.6	7.42
B7WNW7	HEAT repeat-containing protein 3 OS=Homo sapiens GN=HEATR3 PE=4 SV=1 - (B7WNW7_HUMAN)	4.70	3.70	3	1	3	6	3.348E7	594	65.8	5.11
C9JGI3	Thymidine phosphorylase (Fragment) OS=Homo sapiens GN=TYMP PE=4 SV=1 - (C9JGI3_HUMAN)	4.70	14.83	4	1	6	8	1.165E8	445	46.1	5.52
Q53F37	SAR1a gene homolog 2 variant (Fragment) OS=Homo sapiens PE=2 SV=1 - (Q53F37_HUMAN)	4.68	17.68	10	1	3	3	3.444E7	198	22.4	6.11
F8VVM2	Phosphate carrier protein, mitochondrial OS=Homo sapiens GN=SLC25A3 PE=3 SV=1 - (F8VVM2_HUMAN)	4.65	8.64	6	1	3	3	4.902E7	324	36.1	9.26
F5H022	Histone H1.0 OS=Homo sapiens GN=H1F0 PE=3 SV=1 - (F5H022_HUMAN)	4.64	5.65	3	1	1	2	1.326E7	177	19.2	10.76
P62308	Small nuclear ribonucleoprotein G OS=Homo sapiens GN=SNRPG PE=1 SV=1 - (RUXG_HUMAN)	4.63	38.16	6	2	3	3	1.460E7	76	8.5	8.88
Q6TGM5	Mutant NADH-cytochrome b5 reductase (Fragment) OS=Homo sapiens PE=2 SV=1 - (Q6TGM5_HUMAN)	4.62	31.67	1	1	1	4	1.579E8	60	6.5	10.01
O60493	Sorting nexin-3 OS=Homo sapiens GN=SNX3 PE=1 SV=3 - (SNX3_HUMAN)	4.60	19.14	4	1	3	3	1.958E7	162	18.8	8.66
H7C0M9	Uncharacterized protein (Fragment) OS=Homo sapiens PE=4 SV=1 - (H7C0M9_HUMAN)	4.56	5.38	1	1	1	2	1.198E8	186	20.7	4.96
Q6WWJ7	DELTA3PRO-IL-18 OS=Homo sapiens GN=IL18 PE=2 SV=1 - (Q6WWJ7_HUMAN)	4.56	20.63	4	2	4	4	6.747E8	189	21.9	4.78
Q96MT8	Centrosomal protein of 63 kDa OS=Homo sapiens GN=CEP63 PE=1 SV=1 - (CEP63_HUMAN)	4.53	10.67	4	1	7	8	3.879E8	703	81.3	6.13
H0YBX3	Ribonuclease UK114 (Fragment) OS=Homo sapiens GN=HRSP12 PE=4 SV=1 - (H0YBX3_HUMAN)	4.52	30.71	4	1	3	3	2.270E8	127	13.8	9.31
Q5J7W2	Migration-inducing gene 9 protein OS=Homo sapiens PE=2 SV=1 - (Q5J7W2_HUMAN)	4.47	14.77	2	1	1	1	2.905E7	88	9.6	5.17
Q9Y3U8	60S ribosomal protein L36 OS=Homo sapiens GN=RPL36 PE=1 SV=3 - (RL36_HUMAN)	4.43	23.81	6	2	3	4	8.402E7	105	12.2	11.59
Q08554-2	Isoform 1B of Desmocollin-1 OS=Homo sapiens GN=DSC1 - (DSC1_HUMAN)	4.42	9.05	2	1	7	15	9.417E7	840	93.8	5.53
H3BPU8	Deoxyribonuclease-1-like 2 (Fragment) OS=Homo sapiens GN=DNASE1L2 PE=4 SV=1 - (H3BPU8_HUMAN)	4.42	7.87	3	1	2	3	1.121E7	254	27.8	4.98
B2R4V2	cDNA, FLJ92227, highly similar to Homo sapiens ribosomal protein L36a-like (RPL36AL), mRNA OS=Homo sapiens PE=3 SV=1 - (B2R4V2_HUMAN)	4.40	18.87	6	1	2	5	3.862E7	106	12.5	10.65
O94933	SLIT and NTRK-like protein 3 OS=Homo sapiens GN=SLITRK3 PE=2 SV=2 - (SLIK3_HUMAN)	4.36	2.25	1	1	1	7	1.126E8	977	108.9	7.37
P05387	60S acidic ribosomal protein P2 OS=Homo sapiens GN=RPLP2 PE=1 SV=1 - (RLA2_HUMAN)	4.35	17.39	1	1	1	2	7.093E6	115	11.7	4.54
A8K2Q6	Peptidyl-prolyl cis-trans isomerase OS=Homo sapiens PE=2 SV=1 - (A8K2Q6_HUMAN)	4.34	8.96	2	1	2	2	1.659E7	212	22.7	8.40
F8W950	Aminoacyl tRNA synthase complex-interacting multifunctional protein 2 OS=Homo sapiens GN=AIMP2 PE=4 SV=1 - (F8W950_HUMAN)	4.33	15.94	4	1	3	3	5.231E7	251	27.8	9.32
A6NM69	Non-specific lipid-transfer protein OS=Homo sapiens GN=SCP2 PE=4 SV=2 - (A6NM69_HUMAN)	4.33	10.54	10	1	5	10	3.943E7	503	54.4	6.68
B5MCP9	40S ribosomal protein S7 OS=Homo sapiens GN=RPS7 PE=4 SV=1 - (B5MCP9_HUMAN)	4.32	11.23	2	1	2	2	1.007E7	187	21.3	10.27
B7Z6B3	cDNA FLJ53094, highly similar to Receptor expression-enhancing protein 5 OS=Homo sapiens PE=2 SV=1 - (B7Z6B3_HUMAN)	4.28	5.13	4	1	1	2	2.082E7	156	17.7	8.65
E9PQH6	Rho-related GTP-binding protein RhoC (Fragment) OS=Homo sapiens GN=RHOC PE=3 SV=1 - (E9PQH6_HUMAN)	4.28	10.65	14	1	2	4	4.559E7	169	19.2	5.10
E4W6B6	RPL27/NME2 fusion protein (Fragment) OS=Homo sapiens GN=RPL27 PE=2 SV=1 - (E4W6B6_HUMAN)	4.25	7.14	3	1	1	3	5.554E7	126	14.2	10.46
Q99497	Protein DJ-1 OS=Homo sapiens GN=PARK7 PE=1 SV=2 - (PARK7_HUMAN)	4.23	15.34	1	1	3	3	2.314E7	189	19.9	6.79
C9JFR7	Cytochrome c (Fragment) OS=Homo sapiens GN=CYCS PE=3 SV=1 - (C9JFR7_HUMAN)	4.19	41.58	2	1	3	4	1.573E8	101	11.3	9.66
P0C7V6	Putative protein FAM48B2 OS=Homo sapiens GN=FAM48B2 PE=5 SV=1 - (F48B2_HUMAN)	4.19	6.24	1	1	5	11	4.964E7	817	87.5	8.79
Q8N1F8-2	Isoform 2 of Serine/threonine-protein kinase 11-interacting protein OS=Homo sapiens GN=STK11IP - (S11IP_HUMAN)	4.18	4.28	3	1	3	5	3.364E7	747	82.3	5.50
P28070	Proteasome subunit beta type-4 OS=Homo sapiens GN=PSMB4 PE=1 SV=4 - (PSB4_HUMAN)	4.16	11.74	2	1	3	3	1.056E8	264	29.2	5.97
Q9Y3D6	Mitochondrial fission 1 protein OS=Homo sapiens GN=FIS1 PE=1 SV=2 - (FIS1_HUMAN)	4.15	7.24	1	1	1	2	1.827E7	152	16.9	8.79
P61254	60S ribosomal protein L26 OS=Homo sapiens GN=RPL26 PE=1 SV=1 - (RL26_HUMAN)	4.15	35.86	4	4	6	7	3.292E7	145	17.2	10.55
P28072	Proteasome subunit beta type-6 OS=Homo sapiens GN=PSMB6 PE=1 SV=4 - (PSB6_HUMAN)	4.14	17.15	2	1	3	3	1.273E8	239	25.3	4.92
A6H8Y5	Nibrin OS=Homo sapiens GN=NBN PE=2 SV=1 - (A6H8Y5_HUMAN)	4.13	7.03	7	1	5	6	1.176E8	754	84.9	7.06
E9PCY7	Heterogeneous nuclear ribonucleoprotein H, N-terminally processed OS=Homo sapiens GN=HNRNPH1 PE=4 SV=1 - (E9PCY7_HUMAN)	4.12	9.79	19	3	4	6	7.861E7	429	47.1	6.34
E7EUY0	DNA-dependent protein kinase catalytic subunit OS=Homo sapiens GN=PRKDC PE=4 SV=1 - (E7EUY0_HUMAN)	4.10	4.76	4	2	20	37	4.607E8	4096	465.1	7.17
Q9BXG0	P231 OS=Homo sapiens PE=2 SV=1 - (Q9BXG0_HUMAN)	4.09	15.58	1	1	3	4	1.594E8	231	26.7	5.95
H0YFA4	Cysteine-rich protein 2 (Fragment) OS=Homo sapiens GN=CRIP2 PE=4 SV=1 - (H0YFA4_HUMAN)	4.05	13.54	4	1	2	2	4.413E7	192	20.7	8.94
Q0VD83-3	Isoform 3 of Apolipoprotein B receptor OS=Homo sapiens GN=APOBR - (APOBR_HUMAN)	4.05	15.53	5	1	14	24	1.331E8	1069	112.9	4.37
B7ZW58	C13orf38 protein OS=Homo sapiens GN=C13orf38 PE=4 SV=1 - (B7ZW58_HUMAN)	4.00	15.38	1	1	2	3	1.367E7	91	10.7	9.77
Q04941-2	Isoform 2 of Proteolipid protein 2 OS=Homo sapiens GN=PLP2 - (PLP2_HUMAN)	3.99	10.34	2	1	1	1	2.313E7	145	15.9	8.87
F8W7F1	p53 apoptosis effector-related to PMP-22 OS=Homo sapiens GN=PERP PE=4 SV=1 - (F8W7F1_HUMAN)	3.99	4.00	4	1	1	2	4.965E7	175	19.5	7.03
Q9Y3R5-2	Isoform 2 of Protein dopey-2 OS=Homo sapiens GN=DOPEY2 - (DOP2_HUMAN)	3.96	2.88	2	1	7	24	6.080E7	2291	257.2	6.40
Q6KC79-2	Isoform 2 of Nipped-B-like protein OS=Homo sapiens GN=NIPBL - (NIPBL_HUMAN)	3.95	5.78	1	1	14	25	5.770E8	2697	304.2	7.81
Q9H116	GDNF-inducible zinc finger protein 1 OS=Homo sapiens GN=GZF1 PE=1 SV=1 - (GZF1_HUMAN)	3.94	7.88	1	1	5	8	1.509E8	711	80.4	7.87
Q69YN4-3	Isoform 3 of Protein virilizer homolog OS=Homo sapiens GN=KIAA1429 - (VIR_HUMAN)	3.89	6.07	4	1	10	24	5.398E7	1797	200.9	4.82
B1ALC0	Actin-related protein 2/3 complex subunit 5 OS=Homo sapiens GN=ARPC5 PE=3 SV=1 - (B1ALC0_HUMAN)	3.87	28.15	4	1	5	5	4.750E7	135	14.8	5.06
P84090	Enhancer of rudimentary homolog OS=Homo sapiens GN=ERH PE=1 SV=1 - (ERH_HUMAN)	3.85	22.12	1	1	2	2	2.157E7	104	12.3	5.92
B3KY56	Extended synaptotagmin-1 OS=Homo sapiens GN=ESYT1 PE=2 SV=1 - (B3KY56_HUMAN)	3.85	5.39	4	1	7	9	8.705E7	1058	117.3	5.90
G3V5R8	Alpha-1-antitrypsin (Fragment) OS=Homo sapiens GN=SERPINA1 PE=4 SV=1 - (G3V5R8_HUMAN)	3.82	15.22	6	1	1	1	1.580E7	92	9.9	5.58
A2RRP1	Neuroblastoma-amplified sequence OS=Homo sapiens GN=NBAS PE=1 SV=2 - (NBAS_HUMAN)	3.81	3.08	3	1	9	22	2.492E9	2371	268.4	5.96
A8MUX0	Putative keratin-associated protein 10-like ENSP00000375147 OS=Homo sapiens PE=3 SV=1 - (KR10D_HUMAN)	3.81	2.90	1	1	1	1	1.947E7	517	53.9	5.39
F8WEV1	Protein C3orf15 OS=Homo sapiens GN=C3orf15 PE=4 SV=1 - (F8WEV1_HUMAN)	3.80	13.74	2	1	2	6	1.655E8	131	15.5	10.23
B4E2Y4	cDNA FLJ52462, moderately similar to Mus musculus proteasome (prosome, macropain) 26S subunit, non-ATPase, 8 (Psmd8), mRNA OS=Homo sapiens PE=2 SV=1 - (B4E2Y4_HUMAN)	3.79	10.60	3	1	2	2	5.740E7	217	23.8	8.85
E9PF49	NADH dehydrogenase (ubiquinone) 1 beta subcomplex subunit 9 OS=Homo sapiens GN=NDUFB9 PE=4 SV=1 - (E9PF49_HUMAN)	3.77	7.69	1	1	2	3	2.093E8	221	26.6	8.87
B4DV06	cDNA FLJ55904, highly similar to Homo sapiens leucine rich repeat and coiled-coil domain containing 1 (LRRCC1), mRNA (Fragment) OS=Homo sapiens PE=2 SV=1 - (B4DV06_HUMAN)	3.75	3.20	3	1	4	17	1.017E9	751	87.4	6.24
Q9UBS4	DnaJ homolog subfamily B member 11 OS=Homo sapiens GN=DNAJB11 PE=1 SV=1 - (DJB11_HUMAN)	3.74	5.59	1	1	2	11	1.713E8	358	40.5	6.18
Q59H49	Polypyrimidine tract-binding protein 1 isoform c variant (Fragment) OS=Homo sapiens PE=2 SV=1 - (Q59H49_HUMAN)	3.74	6.38	4	1	1	1	1.720E7	329	35.5	8.98
Q5JU30	SH2 domain containing 3C (Fragment) OS=Homo sapiens GN=SH2D3C PE=2 SV=1 - (Q5JU30_HUMAN)	3.73	11.11	1	1	3	6	2.876E7	324	35.2	8.82
Q16186	Proteasomal ubiquitin receptor ADRM1 OS=Homo sapiens GN=ADRM1 PE=1 SV=2 - (ADRM1_HUMAN)	3.72	8.35	4	2	3	4	1.814E6	407	42.1	5.07
P22528	Cornifin-B OS=Homo sapiens GN=SPRR1B PE=1 SV=2 - (SPR1B_HUMAN)	3.71	26.97	3	1	2	5	1.272E7	89	9.9	8.48
P52565	Rho GDP-dissociation inhibitor 1 OS=Homo sapiens GN=ARHGDIA PE=1 SV=3 - (GDIR1_HUMAN)	3.71	22.55	1	1	6	8	4.792E7	204	23.2	5.11
Q6ZUB1	Protein FAM75E1 OS=Homo sapiens GN=FAM75E1 PE=2 SV=2 - (F75E1_HUMAN)	3.70	4.50	1	1	6	13	1.215E8	1445	157.0	9.10
O60216	Double-strand-break repair protein rad21 homolog OS=Homo sapiens GN=RAD21 PE=1 SV=2 - (RAD21_HUMAN)	3.67	8.24	1	1	3	5	3.779E8	631	71.6	4.65
O94907	Dickkopf-related protein 1 OS=Homo sapiens GN=DKK1 PE=1 SV=1 - (DKK1_HUMAN)	3.66	10.53	1	1	3	15	1.142E7	266	28.7	8.40
O60336-3	Isoform 3 of Mitogen-activated protein kinase-binding protein 1 OS=Homo sapiens GN=MAPKBP1 - (MABP1_HUMAN)	3.63	7.99	8	1	10	46	3.448E8	1389	149.9	6.87
A6NKE1	Trafficking protein particle complex subunit 3 OS=Homo sapiens GN=TRAPPC3 PE=4 SV=1 - (A6NKE1_HUMAN)	3.62	16.95	3	1	2	2	2.816E8	118	13.3	8.87
Q96MR7	Putative uncharacterized protein C1orf145 OS=Homo sapiens GN=C1orf145 PE=5 SV=1 - (CA145_HUMAN)	3.62	22.78	2	1	3	6	2.286E8	158	17.5	11.81
A8K4I8	cDNA FLJ78131, highly similar to Homo sapiens nipsnap homolog 1 (C. elegans) (NIPSNAP1), mRNA OS=Homo sapiens PE=2 SV=1 - (A8K4I8_HUMAN)	3.61	10.21	5	1	3	5	4.189E7	284	33.2	9.39
E7EX53	Ribosomal protein L15 (Fragment) OS=Homo sapiens GN=RPL15 PE=3 SV=1 - (E7EX53_HUMAN)	3.54	15.04	5	1	2	2	2.352E7	133	15.7	11.00
Q07955	Serine/arginine-rich splicing factor 1 OS=Homo sapiens GN=SRSF1 PE=1 SV=2 - (SRSF1_HUMAN)	3.54	26.21	3	1	7	9	4.083E7	248	27.7	10.36
Q9H0R4	Haloacid dehalogenase-like hydrolase domain-containing protein 2 OS=Homo sapiens GN=HDHD2 PE=1 SV=1 - (HDHD2_HUMAN)	3.52	6.18	2	1	2	2	7.273E6	259	28.5	6.24
B3KRM2	Serine/threonine-protein phosphatase OS=Homo sapiens PE=2 SV=1 - (B3KRM2_HUMAN)	3.48	13.59	10	3	6	8	1.658E7	309	35.5	5.43
Q5T3W0	Absent in melanoma 2 (Fragment) OS=Homo sapiens GN=AIM2 PE=2 SV=1 - (Q5T3W0_HUMAN)	3.48	14.97	1	1	3	9	1.296E8	167	19.1	9.51
Q8IYS2-2	Isoform 2 of Uncharacterized protein KIAA2013 OS=Homo sapiens GN=KIAA2013 - (K2013_HUMAN)	3.47	2.40	1	1	2	3	9.795E7	667	72.6	8.84
C9JJ54	WD repeat and FYVE domain-containing protein 1 (Fragment) OS=Homo sapiens GN=WDFY1 PE=4 SV=1 - (C9JJ54_HUMAN)	3.47	9.77	3	1	2	3	4.498E7	215	24.2	7.80
P51572	B-cell receptor-associated protein 31 OS=Homo sapiens GN=BCAP31 PE=1 SV=3 - (BAP31_HUMAN)	3.45	19.51	9	1	6	16	3.120E8	246	28.0	8.44
A8K3Z3	26S protease regulatory subunit 8 OS=Homo sapiens GN=PSMC5 PE=2 SV=1 - (A8K3Z3_HUMAN)	3.44	12.81	3	2	5	6	2.583E7	398	44.8	8.18
O75368	SH3 domain-binding glutamic acid-rich-like protein OS=Homo sapiens GN=SH3BGRL PE=1 SV=1 - (SH3L1_HUMAN)	3.43	11.40	1	1	1	1	2.001E7	114	12.8	5.25
A8K0H3	cDNA FLJ78093, highly similar to Homo sapiens ribosomal protein L29 (RPL29), mRNA OS=Homo sapiens PE=2 SV=1 - (A8K0H3_HUMAN)	3.37	24.53	4	2	4	5	2.955E7	159	17.8	11.74
P98088	Mucin-5AC (Fragments) OS=Homo sapiens GN=MUC5AC PE=1 SV=3 - (MUC5A_HUMAN)	3.35	3.30	2	1	8	21	1.229E8	5030	526.3	6.90
Q6GMX0	Putative uncharacterized protein OS=Homo sapiens PE=2 SV=1 - (Q6GMX0_HUMAN)	3.33	18.64	10	1	2	3	1.211E8	236	25.8	7.97
E5RIV2	Centrosomal protein CEP57L1 OS=Homo sapiens GN=CEP57L1 PE=4 SV=1 - (E5RIV2_HUMAN)	3.30	13.76	17	1	7	7	1.044E8	407	47.5	7.80
B7Z7X0	cDNA FLJ51151, highly similar to Receptor-interacting serine/threonine-protein kinase 4 (EC 2.7.11.1) OS=Homo sapiens PE=2 SV=1 - (B7Z7X0_HUMAN)	3.30	5.27	9	1	3	6	3.348E7	721	79.3	7.06
Q8NE71	ATP-binding cassette sub-family F member 1 OS=Homo sapiens GN=ABCF1 PE=1 SV=2 - (ABCF1_HUMAN)	3.25	7.46	5	1	6	12	1.494E8	845	95.9	6.80
P62304	Small nuclear ribonucleoprotein E OS=Homo sapiens GN=SNRPE PE=1 SV=1 - (RUXE_HUMAN)	3.23	21.74	1	1	2	2	3.944E7	92	10.8	9.44
Q96MH7	Uncharacterized protein C5orf34 OS=Homo sapiens GN=C5orf34 PE=2 SV=2 - (CE034_HUMAN)	3.22	3.45	1	1	3	8	6.813E8	638	72.9	7.93
Q9H293-2	Isoform 2 of Interleukin-25 OS=Homo sapiens GN=IL25 - (IL25_HUMAN)	3.21	6.21	2	1	1	7	2.499E7	161	18.5	8.35
B3KPC7	Actin-related protein 2/3 complex subunit 5 OS=Homo sapiens PE=2 SV=1 - (B3KPC7_HUMAN)	3.15	28.76	2	1	4	5	1.471E7	153	17.0	6.02
H0YJB9	UPF0568 protein C14orf166 (Fragment) OS=Homo sapiens GN=C14orf166 PE=4 SV=1 - (H0YJB9_HUMAN)	3.15	10.85	4	1	1	1		129	14.7	5.60
Q53H37	Calmodulin-like skin protein variant (Fragment) OS=Homo sapiens PE=2 SV=1 - (Q53H37_HUMAN)	3.12	7.53	2	1	1	1	3.145E6	146	15.9	4.44
C9IZG4	Protein CutA OS=Homo sapiens GN=CUTA PE=4 SV=1 - (C9IZG4_HUMAN)	3.06	10.37	4	1	1	1	3.736E7	135	14.4	5.45
O00231	26S proteasome non-ATPase regulatory subunit 11 OS=Homo sapiens GN=PSMD11 PE=1 SV=3 - (PSD11_HUMAN)	3.06	7.58	3	1	2	2	6.017E7	422	47.4	6.48
B4DVQ3	Calcium/calmodulin-dependent protein kinase type II subunit gamma OS=Homo sapiens GN=CAMK2G PE=2 SV=1 - (B4DVQ3_HUMAN)	3.01	9.44	1	1	1	2	1.108E8	180	20.5	8.75
E9PR17	CD59 glycoprotein OS=Homo sapiens GN=CD59 PE=4 SV=1 - (E9PR17_HUMAN)	3.00	11.54	3	1	2	2	2.051E7	130	14.5	7.77
P61960	Ubiquitin-fold modifier 1 OS=Homo sapiens GN=UFM1 PE=1 SV=1 - (UFM1_HUMAN)	2.99	24.71	3	1	2	3	1.368E7	85	9.1	9.31
B4DEL6	Phospholipase D3 OS=Homo sapiens GN=PLD3 PE=2 SV=1 - (B4DEL6_HUMAN)	2.96	2.49	2	1	1	1	2.297E6	402	44.8	6.46
Q3LI72	Keratin-associated protein 19-5 OS=Homo sapiens GN=KRTAP19-5 PE=1 SV=1 - (KR195_HUMAN)	2.94	22.22	1	1	1	1	5.919E7	72	7.6	8.13
B2R4N3	cDNA, FLJ92155, highly similar to Homo sapiens ubiquitin-like 5 (UBL5), mRNA OS=Homo sapiens PE=4 SV=1 - (B2R4N3_HUMAN)	2.87	12.33	2	1	1	1	2.237E7	73	8.5	7.28
H7BYG3	Leucine-rich repeats and immunoglobulin-like domains protein 1 OS=Homo sapiens GN=LRIG1 PE=4 SV=1 - (H7BYG3_HUMAN)	2.84	3.61	2	1	4	4	2.066E7	996	109.0	6.80
Q5TA02	Glutathione S-transferase omega 1 (Fragment) OS=Homo sapiens GN=GSTO1 PE=2 SV=1 - (Q5TA02_HUMAN)	2.84	14.00	4	1	3	3	7.747E7	200	23.3	7.21
E7ET82	Ly6/PLAUR domain-containing protein 3 OS=Homo sapiens GN=LYPD3 PE=4 SV=1 - (E7ET82_HUMAN)	2.83	17.35	4	1	5	12	7.669E7	294	30.5	8.27
Q4J6C6-4	Isoform 4 of Prolyl endopeptidase-like OS=Homo sapiens GN=PREPL - (PPCEL_HUMAN)	2.73	3.29	3	1	2	5	4.889E7	638	73.3	5.44
G3V2K7	Transmembrane emp24 domain-containing protein 10 OS=Homo sapiens GN=TMED10 PE=3 SV=1 - (G3V2K7_HUMAN)	2.72	7.19	4	1	1	1	2.983E6	153	16.9	6.52
O15144	Actin-related protein 2/3 complex subunit 2 OS=Homo sapiens GN=ARPC2 PE=1 SV=1 - (ARPC2_HUMAN)	2.68	12.67	2	1	4	4	2.815E7	300	34.3	7.36
Q8IV48	3'-5' exoribonuclease 1 OS=Homo sapiens GN=ERI1 PE=1 SV=3 - (ERI1_HUMAN)	2.68	5.44	2	1	3	3	6.608E7	349	40.0	6.70
Q4ZG77	Putative uncharacterized protein PSMD14 (Fragment) OS=Homo sapiens GN=PSMD14 PE=2 SV=1 - (Q4ZG77_HUMAN)	2.68	8.44	2	1	1	1	3.510E7	154	16.5	5.24
B3KU40	cDNA FLJ39163 fis, clone OCBBF2002615, highly similar to Low-density lipoprotein receptor-related protein 8 OS=Homo sapiens PE=2 SV=1 - (B3KU40_HUMAN)	2.66	8.75	2	1	3	5	6.030E7	457	50.9	6.21
P62861	40S ribosomal protein S30 OS=Homo sapiens GN=FAU PE=1 SV=1 - (RS30_HUMAN)	2.65	42.37	2	2	4	5	4.463E8	59	6.6	12.15
C9J7D1	Ras-related protein Rab-7a OS=Homo sapiens GN=RAB7A PE=4 SV=1 - (C9J7D1_HUMAN)	2.59	12.09	8	1	1	1	1.980E7	91	10.2	7.93
Q9BRL5	CALM3 protein OS=Homo sapiens PE=2 SV=1 - (Q9BRL5_HUMAN)	2.54	15.65	8	1	2	3	5.256E7	147	16.5	4.46
Q05CP6	ZNF140 protein OS=Homo sapiens GN=ZNF140 PE=2 SV=1 - (Q05CP6_HUMAN)	2.53	6.21	1	1	2	2	6.593E7	354	41.1	8.91
A8K4W8	cDNA FLJ77917, highly similar to Homo sapiens ubiquitin-conjugating enzyme E2L 3 (UBE2L3), transcript variant 1, mRNA OS=Homo sapiens PE=2 SV=1 - (A8K4W8_HUMAN)	2.52	14.29	6	1	2	8	8.062E7	154	17.8	8.75
Q9BYR3	Keratin-associated protein 4-4 OS=Homo sapiens GN=KRTAP4-4 PE=2 SV=1 - (KRA44_HUMAN)	2.44	7.23	1	1	1	3	3.926E7	166	18.0	7.91
Q8IX95	Putative cutaneous T-cell lymphoma-associated antigen 3 OS=Homo sapiens GN=CTAGE3P PE=5 SV=1 - (CTGE3_HUMAN)	2.43	15.82	1	1	1	7	6.796E7	158	18.0	4.92
F8VRZ3	Proliferation-associated protein 2G4 OS=Homo sapiens GN=PA2G4 PE=4 SV=1 - (F8VRZ3_HUMAN)	2.42	10.88	10	1	4	8	5.137E7	294	32.6	7.53
P49773	Histidine triad nucleotide-binding protein 1 OS=Homo sapiens GN=HINT1 PE=1 SV=2 - (HINT1_HUMAN)	2.36	18.25	6	2	2	2	4.753E7	126	13.8	6.95
G5EA35	Caspase recruitment domain-containing protein 18 OS=Homo sapiens GN=CARD18 PE=4 SV=1 - (G5EA35_HUMAN)	2.35	17.65	2	1	1	1	2.919E7	51	5.7	7.88
B4DHP2	Prostaglandin E synthase 3 OS=Homo sapiens GN=PTGES3 PE=2 SV=1 - (B4DHP2_HUMAN)	2.34	10.24	5	1	1	1	1.305E7	127	14.8	4.11
P04632	Calpain small subunit 1 OS=Homo sapiens GN=CAPNS1 PE=1 SV=1 - (CPNS1_HUMAN)	2.33	16.42	1	1	3	3	2.922E6	268	28.3	5.20
Q8NA70	Protein FAM47B OS=Homo sapiens GN=FAM47B PE=2 SV=2 - (FA47B_HUMAN)	2.30	1.86	1	1	1	1	5.677E7	645	73.9	8.73
G3V1C1	1,5-anhydro-D-fructose reductase OS=Homo sapiens GN=AKR1E2 PE=4 SV=1 - (G3V1C1_HUMAN)	2.29	8.37	4	1	2	5	3.158E7	203	23.1	6.20
B4DF76	cDNA FLJ59191, highly similar to NADH dehydrogenase (ubiquinone) 1 alpha subcomplex subunit 13 (EC 1.6.5.3) OS=Homo sapiens PE=2 SV=1 - (B4DF76_HUMAN)	2.27	30.18	2	1	4	5	7.718E7	222	24.9	9.70
P19105	Myosin regulatory light chain 12A OS=Homo sapiens GN=MYL12A PE=1 SV=2 - (ML12A_HUMAN)	2.26	18.71	3	1	3	3	9.813E7	171	19.8	4.81
B4DZF3	cDNA FLJ60995 OS=Homo sapiens PE=2 SV=1 - (B4DZF3_HUMAN)	2.25	6.13	1	1	2	3	2.550E8	359	38.4	6.64
Q9Y5G4-2	Isoform 2 of Protocadherin gamma-A9 OS=Homo sapiens GN=PCDHGA9 - (PCDG9_HUMAN)	2.24	2.29	2	1	2	3	5.501E7	828	91.2	4.88
Q04760-2	Isoform 2 of Lactoylglutathione lyase OS=Homo sapiens GN=GLO1 - (LGUL_HUMAN)	2.22	8.28	2	1	2	2	1.795E7	169	19.0	6.05
Q86YI3	PSD protein (Fragment) OS=Homo sapiens GN=PSD PE=2 SV=2 - (Q86YI3_HUMAN)	2.22	10.03	1	1	10	10	6.386E7	927	99.7	6.19
O75223	Gamma-glutamylcyclotransferase OS=Homo sapiens GN=GGCT PE=1 SV=1 - (GGCT_HUMAN)	2.22	21.28	5	1	3	3	1.177E7	188	21.0	5.14
H3BPG0	Cytochrome c oxidase subunit 4 isoform 1, mitochondrial (Fragment) OS=Homo sapiens GN=COX4I1 PE=4 SV=1 - (H3BPG0_HUMAN)	2.22	17.59	5	1	2	2	1.115E8	108	12.6	9.52
C1PHC4	CD44 molecule (Fragment) OS=Homo sapiens GN=CD44 PE=4 SV=1 - (C1PHC4_HUMAN)	2.21	27.27	32	1	1	1	6.204E6	44	4.8	6.47
I3L0K2	Thioredoxin domain-containing protein 17 OS=Homo sapiens GN=TXNDC17 PE=4 SV=1 - (I3L0K2_HUMAN)	2.21	13.33	3	1	1	1	1.579E7	75	8.4	5.76
Q8WZ58	Achaete-scute associated protein OS=Homo sapiens GN=hCG_1648122 PE=4 SV=1 - (Q8WZ58_HUMAN)	2.19	10.60	1	1	5	6	2.375E7	368	39.1	12.04
C9JR72	Kelch repeat and BTB domain-containing protein 13 OS=Homo sapiens GN=KBTBD13 PE=1 SV=1 - (KBTBD_HUMAN)	2.17	4.15	1	1	2	8	2.822E8	458	49.5	5.71
F2VNQ2	MHC class I antigen (Fragment) OS=Homo sapiens GN=HLA-C PE=3 SV=1 - (F2VNQ2_HUMAN)	2.16	10.50	1	1	2	4	4.304E7	181	21.1	7.37
Q59GT9	Gap junction protein (Fragment) OS=Homo sapiens PE=2 SV=1 - (Q59GT9_HUMAN)	2.16	5.50	6	1	3	4	2.703E7	600	66.9	6.46
I3L1H9	Zymogen granule protein 16 homolog B (Fragment) OS=Homo sapiens GN=ZG16B PE=4 SV=1 - (I3L1H9_HUMAN)	2.16	13.04	4	1	1	1	2.174E7	69	7.5	9.32
Q4QRI4	RGS9 protein OS=Homo sapiens GN=RGS9 PE=2 SV=1 - (Q4QRI4_HUMAN)	2.16	11.24	1	1	4	4	5.806E7	445	49.7	9.42
P62306	Small nuclear ribonucleoprotein F OS=Homo sapiens GN=SNRPF PE=1 SV=1 - (RUXF_HUMAN)	2.15	9.30	1	1	1	1	2.055E7	86	9.7	4.67
B3KTA3	cDNA FLJ37935 fis, clone CTONG2005290, highly similar to FASCIN OS=Homo sapiens PE=2 SV=1 - (B3KTA3_HUMAN)	2.15	12.50	7	1	6	8	8.550E7	472	52.2	7.65
P26998	Beta-crystallin B3 OS=Homo sapiens GN=CRYBB3 PE=1 SV=4 - (CRBB3_HUMAN)	2.14	12.32	2	1	2	2	2.708E7	211	24.2	6.73
P56556	NADH dehydrogenase (ubiquinone) 1 alpha subcomplex subunit 6 OS=Homo sapiens GN=NDUFA6 PE=1 SV=3 - (NDUA6_HUMAN)	2.14	5.19	1	1	1	1	1.210E7	154	17.9	10.14
B1N7B6	Cryocrystalglobulin CC1 heavy chain variable region (Fragment) OS=Homo sapiens PE=2 SV=1 - (B1N7B6_HUMAN)	2.13	12.50	1	1	2	3	2.171E7	120	13.2	8.48
Q5VTU8	ATP synthase subunit epsilon-like protein, mitochondrial OS=Homo sapiens GN=ATP5EP2 PE=1 SV=1 - (AT5EL_HUMAN)	2.13	35.29	2	1	2	2	1.773E8	51	5.8	10.14
B7Z3I9	Delta-aminolevulinic acid dehydratase OS=Homo sapiens PE=2 SV=1 - (B7Z3I9_HUMAN)	2.11	11.50	5	1	2	2	3.899E7	313	34.5	7.20
H7BY78	Hypoxanthine-guanine phosphoribosyltransferase OS=Homo sapiens GN=HPRT1 PE=4 SV=1 - (H7BY78_HUMAN)	2.11	8.26	3	1	2	2	1.421E6	218	24.6	7.02
B4DX37	cDNA FLJ58296, highly similar to Kelch-like protein 10 OS=Homo sapiens PE=2 SV=1 - (B4DX37_HUMAN)	2.10	5.77	2	1	3	7	5.501E7	520	58.9	5.41
Q9UI42-2	Isoform 2 of Carboxypeptidase A4 OS=Homo sapiens GN=CPA4 - (CBPA4_HUMAN)	2.09	11.60	5	1	3	3	4.918E7	388	43.5	8.34
A8K6K8	cDNA FLJ77325 OS=Homo sapiens PE=2 SV=1 - (A8K6K8_HUMAN)	2.09	4.60	2	1	2	2	2.602E7	500	55.0	5.88
Q8WV83-2	Isoform 2 of Solute carrier family 35 member F5 OS=Homo sapiens GN=SLC35F5 - (S35F5_HUMAN)	2.09	8.20	5	1	3	4	2.713E8	244	27.8	9.99
Q9Y6X9	MORC family CW-type zinc finger protein 2 OS=Homo sapiens GN=MORC2 PE=1 SV=2 - (MORC2_HUMAN)	2.06	7.07	2	1	6	6	8.324E7	1032	117.7	8.38
D6R9P3	Heterogeneous nuclear ribonucleoprotein A/B OS=Homo sapiens GN=HNRNPAB PE=4 SV=1 - (D6R9P3_HUMAN)	2.04	7.86	9	1	3	3	6.927E7	280	30.3	7.91
Q14393-2	Isoform 2 of Growth arrest-specific protein 6 OS=Homo sapiens GN=GAS6 - (GAS6_HUMAN)	2.03	3.54	6	1	3	9	5.754E8	678	74.9	5.69
B3KT45	cDNA FLJ37607 fis, clone BRCOC2010980, highly similar to Thioredoxin-like protein 1 OS=Homo sapiens PE=2 SV=1 - (B3KT45_HUMAN)	2.03	4.22	5	1	1	1	3.413E7	166	18.9	4.78
A8K9G4	cDNA FLJ77745, highly similar to Homo sapiens neutral sphingomyelinase (N-SMase) activation associated factor (NSMAF), mRNA OS=Homo sapiens PE=2 SV=1 - (A8K9G4_HUMAN)	2.02	6.11	4	1	4	6	5.937E8	917	104.3	6.23
H3BPK3	Hydroxyacylglutathione hydrolase, mitochondrial (Fragment) OS=Homo sapiens GN=HAGH PE=3 SV=1 - (H3BPK3_HUMAN)	2.02	7.53	5	1	2	2	6.750E6	239	26.4	8.24
Q53FH6	Mitogen-activated protein kinase kinase 1 interacting protein 1 variant (Fragment) OS=Homo sapiens PE=2 SV=1 - (Q53FH6_HUMAN)	2.01	8.06	2	1	1	1	8.132E6	124	13.6	7.34
F6S6P2	Large proline-rich protein BAG6 (Fragment) OS=Homo sapiens GN=BAG6 PE=4 SV=1 - (F6S6P2_HUMAN)	2.00	2.52	12	1	1	6	1.387E8	515	54.6	5.77
Q8IWR8	Ribosomal protein L19 (Fragment) OS=Homo sapiens PE=2 SV=1 - (Q8IWR8_HUMAN)	1.99	28.32	3	1	7	7	3.383E8	173	20.8	11.44
B7Z2R9	Lysosome-associated membrane glycoprotein 2 OS=Homo sapiens GN=LAMP2 PE=2 SV=1 - (B7Z2R9_HUMAN)	1.97	2.67	5	1	1	1	8.664E6	300	33.0	6.87
Q96QR8	Transcriptional activator protein Pur-beta OS=Homo sapiens GN=PURB PE=1 SV=3 - (PURB_HUMAN)	1.96	6.09	1	1	2	12	9.303E6	312	33.2	5.43
B2REB8	Protein SET OS=Homo sapiens GN=SET PE=3 SV=1 - (B2REB8_HUMAN)	1.96	7.92	6	1	2	2	2.593E7	265	31.0	4.23
H0Y3U0	Parvalbumin alpha (Fragment) OS=Homo sapiens GN=PVALB PE=4 SV=1 - (H0Y3U0_HUMAN)	1.95	12.26	1	1	2	2	3.630E8	106	11.6	5.07
Q6IFR1	Olfactory receptor OR11-87 OS=Homo sapiens PE=2 SV=1 - (Q6IFR1_HUMAN)	1.93	12.96	1	1	5	5	2.165E7	324	36.1	7.87
P62841	40S ribosomal protein S15 OS=Homo sapiens GN=RPS15 PE=1 SV=2 - (RS15_HUMAN)	1.92	13.10	1	1	2	3	6.709E7	145	17.0	10.39
Q96K62	Zinc finger and BTB domain-containing protein 45 OS=Homo sapiens GN=ZBTB45 PE=2 SV=1 - (ZBT45_HUMAN)	1.89	3.72	1	1	2	2	2.735E7	511	54.0	6.92
C9K0U8	Single-stranded DNA-binding protein, mitochondrial (Fragment) OS=Homo sapiens GN=SSBP1 PE=4 SV=1 - (C9K0U8_HUMAN)	1.87	8.26	5	1	2	2	2.073E7	121	14.1	9.57
O95833	Chloride intracellular channel protein 3 OS=Homo sapiens GN=CLIC3 PE=1 SV=2 - (CLIC3_HUMAN)	1.86	7.63	1	1	2	2	5.273E7	236	26.6	6.43
F8VU40	Proline-rich protein 13 OS=Homo sapiens GN=PRR13 PE=4 SV=1 - (F8VU40_HUMAN)	1.85	5.96	1	1	1	1	3.396E7	151	16.0	10.01
F8VS20	Transmembrane protein 19 (Fragment) OS=Homo sapiens GN=TMEM19 PE=4 SV=1 - (F8VS20_HUMAN)	1.84	6.47	3	1	1	1	1.537E7	170	19.1	9.22
F5H5Y2	Translocon-associated protein subunit alpha OS=Homo sapiens GN=SSR1 PE=4 SV=1 - (F5H5Y2_HUMAN)	1.84	3.09	8	1	1	1	1.576E7	259	29.4	4.72
Q14DE1	GLDN protein OS=Homo sapiens GN=GLDN PE=2 SV=1 - (Q14DE1_HUMAN)	1.82	4.22	3	1	2	3	1.748E7	427	45.8	6.95
F8W9J5	Homeobox even-skipped homolog protein 1 OS=Homo sapiens GN=EVX1 PE=4 SV=1 - (F8W9J5_HUMAN)	1.80	26.87	1	1	6	8	3.147E8	201	20.8	9.00
P15121	Aldose reductase OS=Homo sapiens GN=AKR1B1 PE=1 SV=3 - (ALDR_HUMAN)	1.78	7.28	1	1	2	2	1.273E7	316	35.8	6.98
Q8NEJ1	Putative uncharacterized protein OS=Homo sapiens PE=2 SV=1 - (Q8NEJ1_HUMAN)	1.78	8.90	21	1	2	3	6.458E6	236	25.0	7.69
Q96NG3	Tetratricopeptide repeat protein 25 OS=Homo sapiens GN=TTC25 PE=1 SV=2 - (TTC25_HUMAN)	1.78	3.87	1	1	3	3	8.936E7	672	76.6	5.60
Q0EFA5	S protein OS=Homo sapiens GN=S PE=2 SV=1 - (Q0EFA5_HUMAN)	1.78	1.76	6	1	1	1	9.498E5	512	49.9	8.13
F8VQ14	T-complex protein 1 subunit beta OS=Homo sapiens GN=CCT2 PE=3 SV=1 - (F8VQ14_HUMAN)	1.78	5.53	4	1	2	2	1.664E7	416	44.8	6.54
B4E1K5	Uncharacterized protein OS=Homo sapiens GN=DHCR7 PE=2 SV=1 - (B4E1K5_HUMAN)	1.77	5.36	3	1	2	2	1.365E7	280	32.7	8.94
A6NKB5-2	Isoform 2 of Pecanex-like protein 2 OS=Homo sapiens GN=PCNXL2 - (PCX2_HUMAN)	1.76	6.99	1	1	2	2	1.822E7	286	32.2	8.27
B4E2W0	3-ketoacyl-CoA thiolase OS=Homo sapiens GN=HADHB PE=2 SV=1 - (B4E2W0_HUMAN)	1.75	11.73	21	2	6	7	3.326E7	452	48.8	9.17
I3L0X8	Coronin-7 (Fragment) OS=Homo sapiens GN=CORO7 PE=4 SV=1 - (I3L0X8_HUMAN)	1.73	28.40	10	1	5	15	5.543E8	162	18.4	9.41
C9J5N9	Apolipoprotein O-like OS=Homo sapiens GN=APOOL PE=4 SV=2 - (C9J5N9_HUMAN)	1.73	6.74	2	1	2	7	1.753E8	267	29.1	9.60
G3V3Q9	Paired box protein Pax-6 (Fragment) OS=Homo sapiens GN=PAX6 PE=3 SV=1 - (G3V3Q9_HUMAN)	1.73	7.16	8	1	2	5	1.899E7	377	41.8	9.26
A8K5Q6	cDNA FLJ75694, highly similar to Homo sapiens G protein-coupled receptor 44 (GPR44), mRNA OS=Homo sapiens PE=2 SV=1 - (A8K5Q6_HUMAN)	1.72	4.30	2	1	2	2	9.244E7	395	43.3	10.49
Q68CR9	Aspartate--tRNA ligase, cytoplasmic OS=Homo sapiens GN=DKFZp781B11202 PE=2 SV=1 - (Q68CR9_HUMAN)	1.71	3.99	4	1	2	2	5.806E7	401	45.7	5.71
O95336	6-phosphogluconolactonase OS=Homo sapiens GN=PGLS PE=1 SV=2 - (6PGL_HUMAN)	1.70	6.98	1	1	2	2	4.978E6	258	27.5	6.05
E7EUN2	Arf-GAP with GTPase, ANK repeat and PH domain-containing protein 1 OS=Homo sapiens GN=AGAP1 PE=4 SV=1 - (E7EUN2_HUMAN)	1.70	6.36	8	1	6	9	2.973E8	1069	116.5	8.72
Q96SM3	Probable carboxypeptidase X1 OS=Homo sapiens GN=CPXM1 PE=2 SV=2 - (CPXM1_HUMAN)	1.70	3.27	2	1	3	12	5.933E8	734	81.6	6.67
Q53SX6	Putative uncharacterized protein KYNU (Fragment) OS=Homo sapiens GN=KYNU PE=2 SV=1 - (Q53SX6_HUMAN)	1.68	2.33	6	1	1	1	3.079E7	301	33.8	5.85
B2RAH1	cDNA, FLJ94913, highly similar to Homo sapiens holocarboxylase synthetase(biotin-(proprionyl-Coenzyme A-carboxylase (ATP-hydrolysing))ligase) (HLCS), mRNA OS=Homo sapiens PE=2 SV=1 - (B2RAH1_HUMAN)	1.68	2.20	2	1	2	4	6.473E6	726	80.7	5.62
Q6WG76	Antigen MLAA-20 (Fragment) OS=Homo sapiens PE=2 SV=1 - (Q6WG76_HUMAN)	1.66	13.11	1	1	3	4	4.726E7	244	28.1	4.96
B4DT68	Myotilin OS=Homo sapiens GN=MYOT PE=2 SV=1 - (B4DT68_HUMAN)	1.66	9.40	3	1	3	4	6.253E8	383	42.9	8.75
B4DET6	cDNA FLJ59314, highly similar to UV radiation resistance-associated gene protein OS=Homo sapiens PE=2 SV=1 - (B4DET6_HUMAN)	1.66	3.68	7	1	3	4	7.268E7	598	67.0	7.65
B3KY44	Solute carrier family 11 (Proton-coupled divalent metal ion transporters), member 2, isoform CRA_c OS=Homo sapiens GN=SLC11A2 PE=2 SV=1 - (B3KY44_HUMAN)	1.66	1.71	9	1	1	1	9.199E6	410	44.9	7.65
A8K8F6	cDNA FLJ78417, highly similar to Homo sapiens low density lipoprotein receptor-related protein associated protein 1 (LRPAP1), mRNA OS=Homo sapiens PE=2 SV=1 - (A8K8F6_HUMAN)	1.65	21.57	3	1	8	11	8.938E7	357	41.4	9.06
Q6AHY1	Putative uncharacterized protein DKFZp686O2142 OS=Homo sapiens GN=DKFZp686O2142 PE=2 SV=1 - (Q6AHY1_HUMAN)	1.65	11.28	1	1	2	3	5.649E7	195	21.5	8.62
Q9Y2S2-2	Isoform 2 of Lambda-crystallin homolog OS=Homo sapiens GN=CRYL1 - (CRYL1_HUMAN)	1.65	15.15	2	1	5	5	1.486E8	297	33.3	6.00
P30414	NK-tumor recognition protein OS=Homo sapiens GN=NKTR PE=1 SV=2 - (NKTR_HUMAN)	1.65	6.91	2	1	10	16	4.775E8	1462	165.6	9.99
G3V1A8	Lymphocyte antigen 6 complex locus protein G6c OS=Homo sapiens GN=LY6G6C PE=4 SV=1 - (G3V1A8_HUMAN)	1.63	11.59	2	1	1	2	1.472E7	69	7.6	7.71
Q8WYK1	Contactin-associated protein-like 5 OS=Homo sapiens GN=CNTNAP5 PE=2 SV=1 - (CNTP5_HUMAN)	1.63	2.53	1	1	3	3	2.502E7	1306	145.5	6.29
Q6T4R5	Nance-Horan syndrome protein OS=Homo sapiens GN=NHS PE=1 SV=1 - (NHS_HUMAN)	1.63	3.99	7	1	7	14	9.122E7	1630	176.6	6.79
Q5EBM0	UMP-CMP kinase 2, mitochondrial OS=Homo sapiens GN=CMPK2 PE=1 SV=3 - (CMPK2_HUMAN)	1.60	7.13	1	1	3	3	7.873E8	449	49.4	7.01
P0C6C1	Ankyrin repeat domain-containing protein 34C OS=Homo sapiens GN=ANKRD34C PE=2 SV=1 - (AN34C_HUMAN)	0.00	5.28	2	1	3	4	4.177E7	530	57.6	9.17
Q96PC3-2	Isoform 2 of AP-1 complex subunit sigma-3 OS=Homo sapiens GN=AP1S3 - (AP1S3_HUMAN)	0.00	7.32	1	1	1	7	8.589E6	164	19.4	7.69
Q5T0J7-4	Isoform 4 of Uncharacterized protein C1orf49 OS=Homo sapiens GN=C1orf49 - (CA049_HUMAN)	0.00	4.47	1	1	1	1	4.484E6	179	20.2	8.69
Q8N998	Coiled-coil domain-containing protein 89 OS=Homo sapiens GN=CCDC89 PE=2 SV=1 - (CCD89_HUMAN)	0.00	10.96	2	1	5	8	1.480E8	374	43.8	5.36
Q0VDD5	Putative uncharacterized protein encoded by MIR22HG OS=Homo sapiens GN=MIR22HG PE=5 SV=1 - (CQ091_HUMAN)	0.00	21.05	1	1	1	1	1.652E8	57	6.5	11.21
O14602	Eukaryotic translation initiation factor 1A, Y-chromosomal OS=Homo sapiens GN=EIF1AY PE=1 SV=4 - (IF1AY_HUMAN)	0.00	27.78	3	1	6	9	3.487E7	144	16.4	5.24
Q3MIP1	Inositol 1,4,5-trisphosphate receptor-interacting protein-like 2 OS=Homo sapiens GN=ITPRIPL2 PE=2 SV=1 - (IPIL2_HUMAN)	0.00	8.60	6	1	4	4	8.586E7	535	58.4	9.42
Q8IXI1	Mitochondrial Rho GTPase 2 OS=Homo sapiens GN=RHOT2 PE=1 SV=2 - (MIRO2_HUMAN)	0.00	2.59	1	1	2	5	9.842E7	618	68.1	5.86
Q99542-3	Isoform 2 of Matrix metalloproteinase-19 OS=Homo sapiens GN=MMP19 - (MMP19_HUMAN)	0.00	4.50	3	1	1	1	3.696E7	222	25.3	9.60
Q9BV57-2	Isoform 2 of 1,2-dihydroxy-3-keto-5-methylthiopentene dioxygenase OS=Homo sapiens GN=ADI1 - (MTND_HUMAN)	0.00	3.47	2	1	1	2	1.417E8	173	20.3	6.29
P98179	Putative RNA-binding protein 3 OS=Homo sapiens GN=RBM3 PE=1 SV=1 - (RBM3_HUMAN)	0.00	36.94	1	1	4	4	4.922E7	157	17.2	8.91
Q9P1V8-2	Isoform 2 of Sterile alpha motif domain-containing protein 15 OS=Homo sapiens GN=SAMD15 - (SAM15_HUMAN)	0.00	7.37	3	1	3	4	4.798E8	570	65.5	4.44
Q9P0U3-2	Isoform 2 of Sentrin-specific protease 1 OS=Homo sapiens GN=SENP1 - (SENP1_HUMAN)	0.00	4.51	2	1	3	4	1.444E8	643	73.3	8.56
Q969X2-2	Isoform 2 of Alpha-N-acetylgalactosaminide alpha-2,6-sialyltransferase 6 OS=Homo sapiens GN=ST6GALNAC6 - (SIA7F_HUMAN)	0.00	10.03	2	1	3	3	1.006E5	299	34.3	9.67
Q8IWV8-3	Isoform 3 of E3 ubiquitin-protein ligase UBR2 OS=Homo sapiens GN=UBR2 - (UBR2_HUMAN)	0.00	2.09	1	1	2	8	6.504E7	575	66.0	7.42
Q9Y2H8	Zinc finger protein 510 OS=Homo sapiens GN=ZNF510 PE=2 SV=1 - (ZN510_HUMAN)	0.00	8.49	2	1	7	17	3.010E8	683	79.1	8.97
Q8NEK5	Zinc finger protein 548 OS=Homo sapiens GN=ZNF548 PE=2 SV=2 - (ZN548_HUMAN)	0.00	1.69	3	1	1	1	1.814E7	533	62.7	8.03
E9PEN1	Ankyrin repeat and SOCS box protein 11 OS=Homo sapiens GN=ASB11 PE=4 SV=1 - (E9PEN1_HUMAN)	0.00	2.98	2	1	1	5	4.736E7	302	32.9	7.62
B5BU41	Calcium/calmodulin-dependent protein kinase I OS=Homo sapiens GN=CAMK1 PE=2 SV=1 - (B5BU41_HUMAN)	0.00	3.51	2	1	1	5	5.123E7	370	41.3	5.35
B7ZAM9	Eukaryotic translation initiation factor 3 subunit K OS=Homo sapiens GN=EIF3K PE=2 SV=1 - (B7ZAM9_HUMAN)	0.00	7.11	2	1	2	2	6.570E7	211	24.5	4.87
B3KNR6	cDNA FLJ30255 fis, clone BRACE2002444, highly similar to LAS1-like protein OS=Homo sapiens PE=2 SV=1 - (B3KNR6_HUMAN)	0.00	6.07	1	1	1	2	3.342E7	247	27.4	4.07
A8KA83	cDNA FLJ78586, highly similar to Homo sapiens VAMP (vesicle-associated membrane protein)-associated protein A, 33kDa (VAPA), mRNA OS=Homo sapiens PE=2 SV=1 - (A8KA83_HUMAN)	0.00	6.61	3	1	2	6	1.490E7	242	27.3	8.62
B9EIM4	Olfactory receptor, family 4, subfamily A, member 47 OS=Homo sapiens GN=OR4A47 PE=2 SV=1 - (B9EIM4_HUMAN)	0.00	5.50	2	1	2	4	2.283E7	309	34.8	8.46
I3L3L9	A-kinase anchor protein 1, mitochondrial (Fragment) OS=Homo sapiens GN=AKAP1 PE=4 SV=1 - (I3L3L9_HUMAN)	0.00	38.30	1	1	1	1	2.447E6	47	5.4	8.85
B4DZZ0	cDNA FLJ52128, highly similar to PRA1 family protein 3 OS=Homo sapiens PE=2 SV=1 - (B4DZZ0_HUMAN)	0.00	7.88	7	1	2	3	5.856E6	165	19.2	9.77
F5H2Q4	Tyrosine-protein phosphatase non-receptor type 9 OS=Homo sapiens GN=PTPN9 PE=4 SV=1 - (F5H2Q4_HUMAN)	0.00	5.32	3	1	3	5	1.530E8	583	66.9	8.15
A8K3G7	cDNA FLJ75262, highly similar to Homo sapiens cAMP specific phosphodiesterase 7B (PDE7B gene) OS=Homo sapiens PE=2 SV=1 - (A8K3G7_HUMAN)	0.00	1.56	4	1	1	2	2.023E6	450	51.8	7.03
Q53H03	Nuclear autoantigenic sperm protein isoform 2 variant (Fragment) OS=Homo sapiens PE=2 SV=1 - (Q53H03_HUMAN)	0.00	2.03	1	1	2	3	1.195E7	788	85.2	4.30
E5RI82	Inositol monophosphatase 1 (Fragment) OS=Homo sapiens GN=IMPA1 PE=4 SV=1 - (E5RI82_HUMAN)	0.00	19.54	1	1	3	3	3.565E7	174	19.2	6.54
Q6PJ68	ZNF134 protein (Fragment) OS=Homo sapiens GN=ZNF134 PE=2 SV=1 - (Q6PJ68_HUMAN)	0.00	5.52	2	1	2	2	7.256E7	489	54.8	8.91
A4D142	Similar to ribosomal protein L23 OS=Homo sapiens GN=LOC222901 PE=3 SV=1 - (A4D142_HUMAN)	0.00	19.63	1	1	2	2	3.174E8	107	11.0	10.40
Q8WYX5	Putative uncharacterized protein pp10472 OS=Homo sapiens GN=pp10472 PE=2 SV=1 - (Q8WYX5_HUMAN)	0.00	4.94	1	1	1	1	5.492E6	162	17.9	9.64
A8K874	cDNA FLJ77588, highly similar to Homo sapiens MAK10 homolog, amino-acid N-acetyltransferase subunit, (S. cerevisiae) (MAK10), mRNA OS=Homo sapiens PE=2 SV=1 - (A8K874_HUMAN)	0.00	2.21	2	1	2	3	8.390E7	725	83.6	7.05
B4DP56	cDNA FLJ52237, highly similar to Creatine kinase B-type (EC 2.7.3.2) OS=Homo sapiens PE=2 SV=1 - (B4DP56_HUMAN)	0.00	15.61	4	1	3	3	7.822E7	346	38.7	5.39
B4DVS4	cDNA FLJ57619, highly similar to Delta 3,5-delta 2,4-dienoyl-CoA isomerase, mitochondrial (EC 5.3.3.-) OS=Homo sapiens PE=2 SV=1 - (B4DVS4_HUMAN)	0.00	13.61	2	1	3	4	3.658E7	191	21.0	9.82
E5RK52	Replication initiator 1 OS=Homo sapiens GN=REPIN1 PE=4 SV=1 - (E5RK52_HUMAN)	0.00	18.38	2	1	2	38	3.521E7	136	13.6	11.49
F5H686	Ras-related and estrogen-regulated growth inhibitor-like protein OS=Homo sapiens GN=RERGL PE=4 SV=1 - (F5H686_HUMAN)	0.00	15.20	2	1	3	3	1.009E7	204	23.4	9.36
F5H110	Zinc finger CCCH domain-containing protein 6 OS=Homo sapiens GN=ZC3H6 PE=4 SV=1 - (F5H110_HUMAN)	0.00	4.71	3	1	3	9	6.734E7	892	100.7	8.82
B7Z850	cDNA FLJ54514 OS=Homo sapiens PE=2 SV=1 - (B7Z850_HUMAN)	0.00	4.37	7	1	6	7	5.180E7	961	105.6	5.62
B4DWB4	cDNA FLJ58332, highly similar to MBD2-interacting zinc finger protein OS=Homo sapiens PE=2 SV=1 - (B4DWB4_HUMAN)	0.00	4.53	3	1	2	6	8.912E7	419	48.9	7.28
Q8NAV9	cDNA FLJ34690 fis, clone MESAN2000894 OS=Homo sapiens PE=2 SV=1 - (Q8NAV9_HUMAN)	0.00	6.15	1	1	1	1		130	15.0	10.67
A8K8R2	cDNA FLJ77381, highly similar to Homo sapiens cation channel, sperm associated 3 (CATSPER3), mRNA OS=Homo sapiens PE=2 SV=1 - (A8K8R2_HUMAN)	0.00	6.53	1	1	2	3	8.309E7	398	46.4	6.30
Q9Y2S1	Galanin-related peptide OS=Homo sapiens PE=4 SV=1 - (Q9Y2S1_HUMAN)	0.00	9.43	1	1	1	4	6.953E7	106	11.8	5.97
A8K0P8	cDNA FLJ78223 OS=Homo sapiens PE=2 SV=1 - (A8K0P8_HUMAN)	0.00	2.38	2	1	2	6	2.138E8	756	87.0	5.72
B3KMR4	cDNA FLJ12419 fis, clone MAMMA1003047, highly similar to Protein inhibitor of activated STAT protein 4 OS=Homo sapiens PE=2 SV=1 - (B3KMR4_HUMAN)	0.00	1.37	3	1	1	3		510	56.4	6.29
G3V4X5	Proteasome subunit alpha type-3 OS=Homo sapiens GN=PSMA3 PE=4 SV=1 - (G3V4X5_HUMAN)	0.00	16.11	4	2	3	3	1.616E7	180	20.3	5.10

Appendix D 

**Table 6:** Proteins identified in Subject 2 (N2).

Accession	Description	Score	Coverage	# Proteins	# Unique Peptides	# Peptides	# PSMs	Area	# AAs	MW (kDa)	calc. pI
P02533	Keratin, type I cytoskeletal 14 OS=Homo sapiens GN=KRT14 PE=1 SV=4 - (K1C14_HUMAN)	10202.54	85.38	11	25	76	4159	3.351E10	472	51.5	5.16
P13647	Keratin, type II cytoskeletal 5 OS=Homo sapiens GN=KRT5 PE=1 SV=3 - (K2C5_HUMAN)	10116.88	72.37	19	19	83	4720	2.080E10	590	62.3	7.74
B4DRR0	cDNA FLJ53910, highly similar to Keratin, type II cytoskeletal 6A OS=Homo sapiens PE=2 SV=1 - (B4DRR0_HUMAN)	9085.30	77.57	18	4	85	4185	1.905E10	535	57.8	8.00
P04259	Keratin, type II cytoskeletal 6B OS=Homo sapiens GN=KRT6B PE=1 SV=5 - (K2C6B_HUMAN)	8986.81	73.58	14	3	82	4192	1.905E10	564	60.0	8.00
B2R853	cDNA, FLJ93744, highly similar to Homo sapiens keratin 6E (KRT6E), mRNA OS=Homo sapiens PE=2 SV=1 - (B2R853_HUMAN)	8518.92	73.58	12	2	76	3697	1.905E10	564	60.0	7.72
B4DKV4	cDNA FLJ60647, highly similar to Keratin, type II cytoskeletal 6B OS=Homo sapiens PE=2 SV=1 - (B4DKV4_HUMAN)	7412.83	71.67	13	2	72	3456	1.887E10	526	55.7	8.43
Q04695	Keratin, type I cytoskeletal 17 OS=Homo sapiens GN=KRT17 PE=1 SV=2 - (K1C17_HUMAN)	6081.66	97.69	11	23	67	2570	3.351E10	432	48.1	5.02
P08779	Keratin, type I cytoskeletal 16 OS=Homo sapiens GN=KRT16 PE=1 SV=4 - (K1C16_HUMAN)	5860.99	85.41	11	32	61	2420	3.351E10	473	51.2	5.05
H6VRF8	Keratin 1 OS=Homo sapiens GN=KRT1 PE=3 SV=1 - (H6VRF8_HUMAN)	5375.30	69.72	13	48	58	1959	4.675E9	644	66.0	8.12
P35908	Keratin, type II cytoskeletal 2 epidermal OS=Homo sapiens GN=KRT2 PE=1 SV=2 - (K22E_HUMAN)	5116.80	77.78	6	38	63	2183	1.385E10	639	65.4	8.00
H0YI76	Keratin, type II cytoskeletal 5 (Fragment) OS=Homo sapiens GN=KRT5 PE=3 SV=1 - (H0YI76_HUMAN)	4905.17	93.53	11	1	37	1960	1.427E10	201	23.1	4.97
O95678	Keratin, type II cytoskeletal 75 OS=Homo sapiens GN=KRT75 PE=1 SV=2 - (K2C75_HUMAN)	4238.63	65.88	13	28	65	2015	1.444E10	551	59.5	7.74
P15924	Desmoplakin OS=Homo sapiens GN=DSP PE=1 SV=3 - (DESP_HUMAN)	3710.32	72.17	10	186	250	1756	1.468E10	2871	331.6	6.81
O43790	Keratin, type II cuticular Hb6 OS=Homo sapiens GN=KRT86 PE=1 SV=1 - (KRT86_HUMAN)	3648.96	94.44	10	3	64	1589	8.732E9	486	53.5	5.66
P13645	Keratin, type I cytoskeletal 10 OS=Homo sapiens GN=KRT10 PE=1 SV=6 - (K1C10_HUMAN)	3595.31	67.64	1	34	46	1542	2.366E10	584	58.8	5.21
P78386	Keratin, type II cuticular Hb5 OS=Homo sapiens GN=KRT85 PE=1 SV=1 - (KRT85_HUMAN)	3534.03	92.50	4	24	64	1533	8.732E9	507	55.8	6.55
F8W6V8	Keratin, type II cuticular Hb1 OS=Homo sapiens GN=KRT81 PE=3 SV=1 - (F8W6V8_HUMAN)	3112.80	79.55	9	1	57	1428	8.688E9	484	53.1	5.47
Q15323	Keratin, type I cuticular Ha1 OS=Homo sapiens GN=KRT31 PE=2 SV=3 - (K1H1_HUMAN)	3107.95	72.60	9	11	45	1321	1.962E10	416	47.2	4.88
Q07283	Trichohyalin OS=Homo sapiens GN=TCHH PE=1 SV=2 - (TRHY_HUMAN)	2937.63	81.27	26	179	242	1973	2.680E9	1943	253.8	5.78
Q5XKE5	Keratin, type II cytoskeletal 79 OS=Homo sapiens GN=KRT79 PE=1 SV=2 - (K2C79_HUMAN)	2870.72	34.39	4	3	25	1678	1.559E10	535	57.8	7.20
P78385	Keratin, type II cuticular Hb3 OS=Homo sapiens GN=KRT83 PE=1 SV=2 - (KRT83_HUMAN)	2818.46	76.06	8	3	55	1317	8.732E9	493	54.2	5.64
B3KVF5	cDNA FLJ16494 fis, clone CTONG3004576, highly similar to Keratin, type I cytoskeletal 15 OS=Homo sapiens PE=2 SV=1 - (B3KVF5_HUMAN)	2642.67	72.37	6	25	37	1000	2.963E10	456	49.1	4.79
P35527	Keratin, type I cytoskeletal 9 OS=Homo sapiens GN=KRT9 PE=1 SV=3 - (K1C9_HUMAN)	2638.03	71.27	9	35	38	936	4.024E9	623	62.0	5.24
Q13092	Epidermal type I keratin (Fragment) OS=Homo sapiens GN=K14 PE=1 SV=1 - (Q13092_HUMAN)	2478.84	85.35	3	1	22	1100	2.084E10	157	17.7	5.08
Q01546	Keratin, type II cytoskeletal 2 oral OS=Homo sapiens GN=KRT76 PE=1 SV=2 - (K22O_HUMAN)	2402.65	41.07	14	5	42	1532	1.194E10	638	65.8	8.12
Q14525	Keratin, type I cuticular Ha3-II OS=Homo sapiens GN=KRT33B PE=2 SV=3 - (KT33B_HUMAN)	2261.74	84.16	8	11	42	965	1.962E10	404	46.2	4.84
P12035	Keratin, type II cytoskeletal 3 OS=Homo sapiens GN=KRT3 PE=1 SV=3 - (K2C3_HUMAN)	2172.78	30.89	18	1	32	1444	1.216E10	628	64.4	6.48
O76009	Keratin, type I cuticular Ha3-I OS=Homo sapiens GN=KRT33A PE=2 SV=2 - (KT33A_HUMAN)	2130.93	80.94	5	7	40	898	1.018E10	404	45.9	4.82
H9KV34	Uncharacterized protein OS=Homo sapiens PE=3 SV=1 - (H9KV34_HUMAN)	2047.12	42.11	8	1	26	1003	2.541E10	437	48.2	4.97
A6NCN2	Keratin-81-like protein OS=Homo sapiens PE=2 SV=3 - (KT81L_HUMAN)	1859.86	55.56	2	1	42	875	5.754E9	486	53.4	6.35
Q3SY84	Keratin, type II cytoskeletal 71 OS=Homo sapiens GN=KRT71 PE=1 SV=3 - (K2C71_HUMAN)	1818.14	68.07	2	23	59	1233	8.482E9	523	57.3	6.61
P08727	Keratin, type I cytoskeletal 19 OS=Homo sapiens GN=KRT19 PE=1 SV=4 - (K1C19_HUMAN)	1799.38	37.25	7	3	23	963	3.144E10	400	44.1	5.14
I3L1M0	Uncharacterized protein OS=Homo sapiens PE=4 SV=1 - (I3L1M0_HUMAN)	1762.18	72.94	8	16	42	715	9.720E9	436	49.4	5.06
H9KV51	Keratin, type II cytoskeletal 72 OS=Homo sapiens GN=KRT72 PE=3 SV=1 - (H9KV51_HUMAN)	1695.68	33.69	7	1	29	1223	8.482E9	469	51.1	6.46
Q8WVW5	Putative uncharacterized protein (Fragment) OS=Homo sapiens PE=2 SV=1 - (Q8WVW5_HUMAN)	1645.14	79.34	78	11	39	658	1.074E10	363	40.5	6.14
Q7RTS7	Keratin, type II cytoskeletal 74 OS=Homo sapiens GN=KRT74 PE=1 SV=2 - (K2C74_HUMAN)	1447.94	68.43	6	16	50	1053	9.825E9	529	57.8	7.71
Q86Y46	Keratin, type II cytoskeletal 73 OS=Homo sapiens GN=KRT73 PE=1 SV=1 - (K2C73_HUMAN)	1442.16	43.15	7	2	35	1062	9.825E9	540	58.9	7.23
Q7Z3Z0	Keratin, type I cytoskeletal 25 OS=Homo sapiens GN=KRT25 PE=1 SV=1 - (K1C25_HUMAN)	1326.45	74.67	4	17	36	609	4.512E9	450	49.3	5.08
Q7Z3Y8	Keratin, type I cytoskeletal 27 OS=Homo sapiens GN=KRT27 PE=1 SV=2 - (K1C27_HUMAN)	1265.40	65.80	2	14	31	552	4.512E9	459	49.8	5.05
Q92764	Keratin, type I cuticular Ha5 OS=Homo sapiens GN=KRT35 PE=2 SV=5 - (KRT35_HUMAN)	1187.19	62.86	2	18	31	539	1.784E10	455	50.3	4.91
P05787	Keratin, type II cytoskeletal 8 OS=Homo sapiens GN=KRT8 PE=1 SV=7 - (K2C8_HUMAN)	958.49	46.79	45	5	28	759	1.194E10	483	53.7	5.59
P07355	Annexin A2 OS=Homo sapiens GN=ANXA2 PE=1 SV=2 - (ANXA2_HUMAN)	954.61	89.09	27	36	44	356	4.917E9	339	38.6	7.75
F8VW92	Tubulin beta chain OS=Homo sapiens GN=TUBB PE=3 SV=1 - (F8VW92_HUMAN)	941.58	68.97	38	5	26	334	1.008E9	435	48.5	5.20
Q9NSB2	Keratin, type II cuticular Hb4 OS=Homo sapiens GN=KRT84 PE=1 SV=2 - (KRT84_HUMAN)	924.11	52.83	18	16	32	668	1.194E10	600	64.8	7.56
Q7Z3Y7	Keratin, type I cytoskeletal 28 OS=Homo sapiens GN=KRT28 PE=1 SV=2 - (K1C28_HUMAN)	914.85	51.51	1	8	24	521	1.667E10	464	50.5	5.47
B4DRW1	cDNA FLJ55805, highly similar to Keratin, type II cytoskeletal 4 OS=Homo sapiens PE=2 SV=1 - (B4DRW1_HUMAN)	881.35	40.51	10	2	22	536	7.582E9	474	51.7	6.81
P68371	Tubulin beta-4B chain OS=Homo sapiens GN=TUBB4B PE=1 SV=1 - (TBB4B_HUMAN)	860.54	61.35	19	1	25	315	1.004E9	445	49.8	4.89
Q13885	Tubulin beta-2A chain OS=Homo sapiens GN=TUBB2A PE=1 SV=1 - (TBB2A_HUMAN)	811.16	68.31	24	4	26	300	9.132E8	445	49.9	4.89
Q9NSB4	Keratin, type II cuticular Hb2 OS=Homo sapiens GN=KRT82 PE=1 SV=3 - (KRT82_HUMAN)	810.37	43.66	2	7	20	684	1.029E10	513	56.6	6.74
Q99456	Keratin, type I cytoskeletal 12 OS=Homo sapiens GN=KRT12 PE=1 SV=1 - (K1C12_HUMAN)	808.21	26.72	1	2	15	388	1.285E10	494	53.5	4.78
P68032	Actin, alpha cardiac muscle 1 OS=Homo sapiens GN=ACTC1 PE=1 SV=1 - (ACTC_HUMAN)	784.67	50.66	47	2	25	430	6.082E9	377	42.0	5.39
P12814-2	Isoform 2 of Alpha-actinin-1 OS=Homo sapiens GN=ACTN1 - (ACTN1_HUMAN)	731.60	63.02	22	21	54	277	1.037E9	887	102.6	5.50
G3V1U9	Tubulin alpha-1A chain OS=Homo sapiens GN=TUBA1A PE=3 SV=1 - (G3V1U9_HUMAN)	730.65	55.29	28	7	23	240	1.895E9	416	46.3	5.08
O43707	Alpha-actinin-4 OS=Homo sapiens GN=ACTN4 PE=1 SV=2 - (ACTN4_HUMAN)	713.53	63.67	9	24	57	285	1.037E9	911	104.8	5.44
F5GZD1	Keratin, type II cytoskeletal 7 OS=Homo sapiens GN=KRT7 PE=4 SV=1 - (F5GZD1_HUMAN)	700.78	30.77	3	1	17	634	1.194E10	403	44.9	5.55
P08729	Keratin, type II cytoskeletal 7 OS=Homo sapiens GN=KRT7 PE=1 SV=5 - (K2C7_HUMAN)	696.68	35.18	39	1	23	639	1.194E10	469	51.4	5.48
O60814	Histone H2B type 1-K OS=Homo sapiens GN=HIST1H2BK PE=1 SV=3 - (H2B1K_HUMAN)	678.35	59.52	32	8	11	315	6.268E9	126	13.9	10.32
P31947	14-3-3 protein sigma OS=Homo sapiens GN=SFN PE=1 SV=1 - (1433S_HUMAN)	672.15	84.27	8	23	34	288	3.540E9	248	27.8	4.74
A8MUB1	Tubulin alpha-4A chain OS=Homo sapiens GN=TUBA4A PE=2 SV=1 - (A8MUB1_HUMAN)	646.93	48.50	11	4	21	220	1.817E9	433	48.3	5.01
B7ZBC3	Spectrin repeat containing, nuclear envelope 1 OS=Homo sapiens GN=SYNE1 PE=4 SV=1 - (B7ZBC3_HUMAN)	628.19	8.91	21	2	67	367	8.395E9	8797	1010.4	5.53
Q14532	Keratin, type I cuticular Ha2 OS=Homo sapiens GN=KRT32 PE=1 SV=3 - (K1H2_HUMAN)	627.86	33.71	5	4	17	328	1.792E10	448	50.3	4.84
O75369-2	Isoform 2 of Filamin-B OS=Homo sapiens GN=FLNB - (FLNB_HUMAN)	626.55	39.99	16	62	83	261	3.713E8	2578	275.5	5.78
P04792	Heat shock protein beta-1 OS=Homo sapiens GN=HSPB1 PE=1 SV=2 - (HSPB1_HUMAN)	620.88	94.15	4	19	20	260	5.429E9	205	22.8	6.40
Q09666	Neuroblast differentiation-associated protein AHNAK OS=Homo sapiens GN=AHNAK PE=1 SV=2 - (AHNK_HUMAN)	615.95	50.41	8	104	159	461	2.245E9	5890	628.7	6.15
Q15149	Plectin OS=Homo sapiens GN=PLEC PE=1 SV=3 - (PLEC_HUMAN)	581.38	33.01	16	74	165	355	2.003E9	4684	531.5	5.96
P14923	Junction plakoglobin OS=Homo sapiens GN=JUP PE=1 SV=3 - (PLAK_HUMAN)	571.86	68.05	13	33	38	281	5.717E8	745	81.7	6.14
P04406	Glyceraldehyde-3-phosphate dehydrogenase OS=Homo sapiens GN=GAPDH PE=1 SV=3 - (G3P_HUMAN)	533.28	80.60	11	24	29	248	1.584E10	335	36.0	8.46
Q13835-2	Isoform 1 of Plakophilin-1 OS=Homo sapiens GN=PKP1 - (PKP1_HUMAN)	529.19	61.71	4	32	46	265	6.597E8	726	80.4	8.97
B2RBD5	cDNA, FLJ95457, highly similar to Homo sapiens tubulin, beta, 4 (TUBB4), mRNA OS=Homo sapiens PE=2 SV=1 - (B2RBD5_HUMAN)	528.55	44.44	14	1	20	202	8.605E8	450	50.4	4.93
Q9BTM1	Histone H2A.J OS=Homo sapiens GN=H2AFJ PE=1 SV=1 - (H2AJ_HUMAN)	516.22	48.06	19	4	13	189	7.260E9	129	14.0	10.90
P62805	Histone H4 OS=Homo sapiens GN=HIST1H4A PE=1 SV=2 - (H4_HUMAN)	503.30	59.22	3	13	14	212	9.332E9	103	11.4	11.36
Q562R1	Beta-actin-like protein 2 OS=Homo sapiens GN=ACTBL2 PE=1 SV=2 - (ACTBL_HUMAN)	494.96	22.87	13	2	15	286	8.094E9	376	42.0	5.59
Q93077	Histone H2A type 1-C OS=Homo sapiens GN=HIST1H2AC PE=1 SV=3 - (H2A1C_HUMAN)	483.42	41.54	9	2	10	169	7.260E9	130	14.1	11.05
P35579	Myosin-9 OS=Homo sapiens GN=MYH9 PE=1 SV=4 - (MYH9_HUMAN)	482.02	48.01	15	47	98	278	9.119E8	1960	226.4	5.60
P06733	Alpha-enolase OS=Homo sapiens GN=ENO1 PE=1 SV=2 - (ENOA_HUMAN)	459.90	72.58	25	23	30	174	1.134E9	434	47.1	7.39
P47929	Galectin-7 OS=Homo sapiens GN=LGALS7 PE=1 SV=2 - (LEG7_HUMAN)	453.81	98.53	1	14	17	177	4.674E9	136	15.1	7.62
P63104	14-3-3 protein zeta/delta OS=Homo sapiens GN=YWHAZ PE=1 SV=1 - (1433Z_HUMAN)	416.63	72.24	20	12	22	175	2.496E9	245	27.7	4.79
P21333-2	Isoform 2 of Filamin-A OS=Homo sapiens GN=FLNA - (FLNA_HUMAN)	403.22	30.01	18	44	60	206	2.231E8	2639	279.8	6.05
Q2M2I5	Keratin, type I cytoskeletal 24 OS=Homo sapiens GN=KRT24 PE=1 SV=1 - (K1C24_HUMAN)	357.76	20.76	1	1	10	219	1.293E10	525	55.1	4.96
P27348	14-3-3 protein theta OS=Homo sapiens GN=YWHAQ PE=1 SV=1 - (1433T_HUMAN)	349.82	67.35	12	13	23	140	2.580E9	245	27.7	4.78
H0YG33	Heat shock 70 kDa protein 1A/1B OS=Homo sapiens GN=HSPA1B PE=3 SV=1 - (H0YG33_HUMAN)	319.97	42.51	22	20	37	177	8.799E8	708	77.4	6.99
Q8TC04	Keratin 23 (Histone deacetylase inducible) OS=Homo sapiens GN=KRT23 PE=2 SV=1 - (Q8TC04_HUMAN)	302.08	11.37	1	1	7	264	1.089E10	422	48.1	6.54
Q3ZCM7	Tubulin beta-8 chain OS=Homo sapiens GN=TUBB8 PE=1 SV=2 - (TBB8_HUMAN)	299.51	22.52	11	1	11	134	6.829E8	444	49.7	4.89
P37802	Transgelin-2 OS=Homo sapiens GN=TAGLN2 PE=1 SV=3 - (TAGL2_HUMAN)	290.17	79.90	2	15	19	101	5.492E8	199	22.4	8.25
Q8N1N4	Keratin, type II cytoskeletal 78 OS=Homo sapiens GN=KRT78 PE=2 SV=2 - (K2C78_HUMAN)	283.12	17.50	4	2	11	180	6.573E9	520	56.8	6.02
P11142	Heat shock cognate 71 kDa protein OS=Homo sapiens GN=HSPA8 PE=1 SV=1 - (HSP7C_HUMAN)	279.86	46.44	26	11	33	178	2.435E9	646	70.9	5.52
P54652	Heat shock-related 70 kDa protein 2 OS=Homo sapiens GN=HSPA2 PE=1 SV=1 - (HSP72_HUMAN)	273.48	46.32	3	13	30	164	1.140E9	639	70.0	5.74
P31946-2	Isoform Short of 14-3-3 protein beta/alpha OS=Homo sapiens GN=YWHAB - (1433B_HUMAN)	266.44	51.64	11	3	15	120	2.825E9	244	27.8	4.83
P68104	Elongation factor 1-alpha 1 OS=Homo sapiens GN=EEF1A1 PE=1 SV=1 - (EF1A1_HUMAN)	264.87	29.44	40	12	16	115	8.450E8	462	50.1	9.01
Q01469	Fatty acid-binding protein, epidermal OS=Homo sapiens GN=FABP5 PE=1 SV=3 - (FABP5_HUMAN)	256.45	90.37	2	17	20	111	1.789E9	135	15.2	7.01
P61981	14-3-3 protein gamma OS=Homo sapiens GN=YWHAG PE=1 SV=2 - (1433G_HUMAN)	255.39	56.68	13	5	15	119	2.580E9	247	28.3	4.89
P02545-3	Isoform ADelta10 of Prelamin-A/C OS=Homo sapiens GN=LMNA - (LMNA_HUMAN)	253.47	59.78	17	25	37	123	4.727E8	634	70.6	8.40
O43175	D-3-phosphoglycerate dehydrogenase OS=Homo sapiens GN=PHGDH PE=1 SV=4 - (SERA_HUMAN)	253.20	39.96	11	14	14	98	2.784E8	533	56.6	6.71
Q9NSB5	Type II hair keratin 1 (Fragment) OS=Homo sapiens GN=KRTHB1 PE=2 SV=1 - (Q9NSB5_HUMAN)	243.13	76.42	1	1	8	138	3.424E9	123	12.9	8.57
P15311	Ezrin OS=Homo sapiens GN=EZR PE=1 SV=4 - (EZRI_HUMAN)	241.06	40.61	25	12	29	120	2.684E8	586	69.4	6.27
P62987	Ubiquitin-60S ribosomal protein L40 OS=Homo sapiens GN=UBA52 PE=1 SV=2 - (RL40_HUMAN)	230.43	58.59	26	1	10	135	4.213E8	128	14.7	9.83
F8WA45	Gelsolin OS=Homo sapiens GN=GSN PE=4 SV=1 - (F8WA45_HUMAN)	226.47	33.43	19	14	22	95	6.614E8	715	78.9	6.01
P62258	14-3-3 protein epsilon OS=Homo sapiens GN=YWHAE PE=1 SV=1 - (1433E_HUMAN)	223.10	41.96	12	7	12	89	2.168E9	255	29.2	4.74
P62979	Ubiquitin-40S ribosomal protein S27a OS=Homo sapiens GN=RPS27A PE=1 SV=2 - (RS27A_HUMAN)	221.00	62.18	28	2	11	131	4.213E8	156	18.0	9.64
P09211	Glutathione S-transferase P OS=Homo sapiens GN=GSTP1 PE=1 SV=2 - (GSTP1_HUMAN)	220.02	68.10	4	7	12	80	1.141E9	210	23.3	5.64
P55072	Transitional endoplasmic reticulum ATPase OS=Homo sapiens GN=VCP PE=1 SV=4 - (TERA_HUMAN)	207.65	32.75	11	18	26	93	2.545E8	806	89.3	5.26
Q9H853	Putative tubulin-like protein alpha-4B OS=Homo sapiens GN=TUBA4B PE=5 SV=2 - (TBA4B_HUMAN)	202.11	14.11	1	1	3	65	2.582E8	241	27.5	7.83
Q6DN03	Putative histone H2B type 2-C OS=Homo sapiens GN=HIST2H2BC PE=5 SV=3 - (H2B2C_HUMAN)	187.22	26.42	20	3	7	106	1.529E9	193	21.5	10.70
Q562V5	Actin-like protein (Fragment) OS=Homo sapiens GN=ACT PE=2 SV=1 - (Q562V5_HUMAN)	183.87	52.43	14	1	3	72	6.315E9	103	11.4	6.35
P31949	Protein S100-A11 OS=Homo sapiens GN=S100A11 PE=1 SV=2 - (S10AB_HUMAN)	183.48	56.19	2	7	9	59	3.710E8	105	11.7	7.12
P13639	Elongation factor 2 OS=Homo sapiens GN=EEF2 PE=1 SV=4 - (EF2_HUMAN)	180.95	40.68	16	20	26	78	4.456E8	858	95.3	6.83
Q96QV6	Histone H2A type 1-A OS=Homo sapiens GN=HIST1H2AA PE=1 SV=3 - (H2A1A_HUMAN)	178.74	40.46	9	2	8	81	5.141E9	131	14.2	10.86
Q7Z406	Myosin-14 OS=Homo sapiens GN=MYH14 PE=1 SV=2 - (MYH14_HUMAN)	177.44	27.07	17	10	53	120	3.989E8	1995	227.7	5.60
P36952	Serpin B5 OS=Homo sapiens GN=SERPINB5 PE=1 SV=2 - (SPB5_HUMAN)	174.39	56.53	4	16	17	75	4.487E8	375	42.1	6.05
B4DF70	cDNA FLJ60461, highly similar to Peroxiredoxin-2 (EC 1.11.1.15) OS=Homo sapiens PE=2 SV=1 - (B4DF70_HUMAN)	163.27	51.37	4	11	13	67	4.804E8	183	20.1	8.78
P09382	Galectin-1 OS=Homo sapiens GN=LGALS1 PE=1 SV=2 - (LEG1_HUMAN)	162.87	60.00	2	7	9	65	8.365E8	135	14.7	5.50
D3DPU2	Adenylyl cyclase-associated protein OS=Homo sapiens GN=CAP1 PE=3 SV=1 - (D3DPU2_HUMAN)	159.45	32.00	24	2	15	49	1.322E8	475	51.6	8.02
P35609	Alpha-actinin-2 OS=Homo sapiens GN=ACTN2 PE=1 SV=1 - (ACTN2_HUMAN)	151.57	30.65	9	1	26	68	3.887E8	894	103.8	5.45
Q6PK75	PRSS2 protein (Fragment) OS=Homo sapiens GN=PRSS2 PE=2 SV=1 - (Q6PK75_HUMAN)	149.56	17.65	16	1	3	69	4.275E8	187	20.1	4.87
O75116	Rho-associated protein kinase 2 OS=Homo sapiens GN=ROCK2 PE=1 SV=4 - (ROCK2_HUMAN)	149.48	12.61	5	2	19	102	4.649E9	1388	160.8	6.02
P14618	Pyruvate kinase isozymes M1/M2 OS=Homo sapiens GN=PKM2 PE=1 SV=4 - (KPYM_HUMAN)	148.68	45.76	20	15	21	64	2.711E8	531	57.9	7.84
Q9P225-2	Isoform 2 of Dynein heavy chain 2, axonemal OS=Homo sapiens GN=DNAH2 - (DYH2_HUMAN)	144.77	6.27	3	1	21	79	1.274E9	4196	481.7	6.43
A8K486	Peptidyl-prolyl cis-trans isomerase OS=Homo sapiens PE=2 SV=1 - (A8K486_HUMAN)	144.12	71.52	31	11	14	72	1.118E9	165	18.0	6.90
P26038	Moesin OS=Homo sapiens GN=MSN PE=1 SV=3 - (MOES_HUMAN)	142.95	30.68	19	1	21	88	3.825E8	577	67.8	6.40
P84243	Histone H3.3 OS=Homo sapiens GN=H3F3A PE=1 SV=2 - (H33_HUMAN)	141.46	82.35	9	1	17	108	3.758E9	136	15.3	11.27
Q5DT20	Hornerin OS=Homo sapiens GN=HRNR PE=2 SV=1 - (Q5DT20_HUMAN)	137.55	18.42	2	8	17	79	2.432E8	2850	282.2	10.02
P68431	Histone H3.1 OS=Homo sapiens GN=HIST1H3A PE=1 SV=2 - (H31_HUMAN)	136.84	75.00	7	1	16	105	3.758E9	136	15.4	11.12
B2R8Y4	cDNA, FLJ94117, highly similar to Homo sapiens actinin, alpha 3 (ACTN3), mRNA OS=Homo sapiens PE=2 SV=1 - (B2R8Y4_HUMAN)	136.16	27.08	5	1	23	68	8.566E8	901	103.2	5.60
Q02413	Desmoglein-1 OS=Homo sapiens GN=DSG1 PE=1 SV=2 - (DSG1_HUMAN)	135.11	27.74	2	14	20	58	1.512E8	1049	113.7	5.03
P60174	Triosephosphate isomerase OS=Homo sapiens GN=TPI1 PE=1 SV=3 - (TPIS_HUMAN)	134.56	80.42	6	12	19	52	5.992E8	286	30.8	5.92
F8W8Y7	Armadillo repeat-containing X-linked protein 4 OS=Homo sapiens GN=ARMCX4 PE=4 SV=1 - (F8W8Y7_HUMAN)	134.22	10.61	3	1	18	123	1.127E9	2394	247.7	5.95
A8K6T3	cDNA FLJ78674, highly similar to Homo sapiens desmocollin type 4 OS=Homo sapiens PE=2 SV=1 - (A8K6T3_HUMAN)	133.77	28.68	4	14	24	60	3.370E8	896	99.9	6.15
B7Z7A9	Phosphoglycerate kinase OS=Homo sapiens GN=PGK1 PE=2 SV=1 - (B7Z7A9_HUMAN)	133.20	61.44	8	15	21	51	4.525E8	389	41.4	7.33
Q8IUX7	Adipocyte enhancer-binding protein 1 OS=Homo sapiens GN=AEBP1 PE=1 SV=1 - (AEBP1_HUMAN)	129.76	4.75	3	1	4	149	1.274E9	1158	130.8	5.11
P0C0S5	Histone H2A.Z OS=Homo sapiens GN=H2AFZ PE=1 SV=2 - (H2AZ_HUMAN)	128.97	26.56	9	1	4	55	4.893E9	128	13.5	10.58
P06576	ATP synthase subunit beta, mitochondrial OS=Homo sapiens GN=ATP5B PE=1 SV=3 - (ATPB_HUMAN)	128.91	40.08	8	13	17	46	3.013E8	529	56.5	5.40
Q53HU8	Vimentin variant (Fragment) OS=Homo sapiens PE=2 SV=1 - (Q53HU8_HUMAN)	128.47	31.55	20	2	14	105	5.427E9	466	53.7	5.12
Q562Q2	Actin-like protein (Fragment) OS=Homo sapiens GN=ACT PE=3 SV=1 - (Q562Q2_HUMAN)	127.19	33.98	10	1	3	71	3.349E9	103	11.5	6.68
Q8WVJ2	NudC domain-containing protein 2 OS=Homo sapiens GN=NUDCD2 PE=1 SV=1 - (NUDC2_HUMAN)	126.61	17.83	1	1	2	92	1.745E8	157	17.7	5.07
Q15828	Cystatin-M OS=Homo sapiens GN=CST6 PE=1 SV=1 - (CYTM_HUMAN)	125.17	72.48	6	12	12	56	3.044E8	149	16.5	8.09
Q71DI3	Histone H3.2 OS=Homo sapiens GN=HIST2H3A PE=1 SV=3 - (H32_HUMAN)	124.97	75.00	7	1	16	102	3.758E9	136	15.4	11.27
Q9HD64-4	Isoform D of G antigen family D member 2 OS=Homo sapiens GN=XAGE1A - (GAGD2_HUMAN)	121.02	24.64	7	1	2	59	1.440E8	69	8.0	10.11
F6KPG5	Albumin (Fragment) OS=Homo sapiens PE=2 SV=1 - (F6KPG5_HUMAN)	118.38	39.15	15	19	20	52	2.191E8	585	66.5	6.04
P07737	Profilin-1 OS=Homo sapiens GN=PFN1 PE=1 SV=2 - (PROF1_HUMAN)	118.22	72.86	2	9	11	48	5.706E8	140	15.0	8.27
P27482	Calmodulin-like protein 3 OS=Homo sapiens GN=CALML3 PE=1 SV=2 - (CALL3_HUMAN)	117.52	81.21	1	9	11	53	8.426E8	149	16.9	4.42
A0N4V7	HCG2039797 (Fragment) OS=Homo sapiens GN=Tcr-alpha PE=4 SV=1 - (A0N4V7_HUMAN)	117.01	38.10	1	1	1	203	1.848E10	21	2.2	9.74
B7ZLH8	EVPL protein OS=Homo sapiens GN=EVPL PE=2 SV=1 - (B7ZLH8_HUMAN)	116.20	21.61	3	9	44	86	1.448E9	2055	233.7	7.25
P16402	Histone H1.3 OS=Homo sapiens GN=HIST1H1D PE=1 SV=2 - (H13_HUMAN)	112.67	36.65	9	8	16	100	1.479E9	221	22.3	11.02
P00338	L-lactate dehydrogenase A chain OS=Homo sapiens GN=LDHA PE=1 SV=2 - (LDHA_HUMAN)	110.37	57.23	23	11	21	59	3.933E8	332	36.7	8.27
Q0Z7S8	Fatty acid-binding protein 9 OS=Homo sapiens GN=FABP9 PE=1 SV=1 - (FABP9_HUMAN)	107.52	75.76	1	11	11	49	3.681E8	132	15.1	8.09
P23528	Cofilin-1 OS=Homo sapiens GN=CFL1 PE=1 SV=3 - (COF1_HUMAN)	107.01	81.33	12	11	13	40	8.406E8	166	18.5	8.09
H0Y2N2	Ribonuclease 3 OS=Homo sapiens GN=DROSHA PE=4 SV=1 - (H0Y2N2_HUMAN)	106.10	5.23	7	1	7	77	1.240E9	1299	150.4	7.65
P07900-2	Isoform 2 of Heat shock protein HSP 90-alpha OS=Homo sapiens GN=HSP90AA1 - (HS90A_HUMAN)	101.44	24.12	42	8	22	63	3.651E8	854	98.1	5.16
P04083	Annexin A1 OS=Homo sapiens GN=ANXA1 PE=1 SV=2 - (ANXA1_HUMAN)	99.71	64.16	6	17	22	46	6.989E8	346	38.7	7.02
P29034	Protein S100-A2 OS=Homo sapiens GN=S100A2 PE=1 SV=3 - (S10A2_HUMAN)	98.08	39.80	2	8	8	52	3.474E8	98	11.1	4.78
P08238	Heat shock protein HSP 90-beta OS=Homo sapiens GN=HSP90AB1 PE=1 SV=4 - (HS90B_HUMAN)	97.62	28.04	39	5	24	59	3.688E8	724	83.2	5.03
P05109	Protein S100-A8 OS=Homo sapiens GN=S100A8 PE=1 SV=1 - (S10A8_HUMAN)	97.01	60.22	1	9	12	46	8.360E8	93	10.8	7.03
D3DVC4	Nestin, isoform CRA_c OS=Homo sapiens GN=NES PE=3 SV=1 - (D3DVC4_HUMAN)	96.98	7.28	5	2	11	56	1.575E9	1621	177.3	4.36
Q9UPN3	Microtubule-actin cross-linking factor 1, isoforms 1/2/3/5 OS=Homo sapiens GN=MACF1 PE=1 SV=4 - (MACF1_HUMAN)	93.38	8.76	11	4	58	106	2.652E9	7388	837.8	5.39
P50607-2	Isoform 2 of Tubby protein homolog OS=Homo sapiens GN=TUB - (TUB_HUMAN)	93.18	13.90	6	1	7	52	5.989E8	561	62.1	9.54
Q6YHK3	CD109 antigen OS=Homo sapiens GN=CD109 PE=1 SV=2 - (CD109_HUMAN)	92.88	19.10	4	16	24	50	5.537E8	1445	161.6	5.85
P32926	Desmoglein-3 OS=Homo sapiens GN=DSG3 PE=1 SV=2 - (DSG3_HUMAN)	92.75	16.72	1	11	13	33	2.210E8	999	107.5	5.00
P04075	Fructose-bisphosphate aldolase A OS=Homo sapiens GN=ALDOA PE=1 SV=2 - (ALDOA_HUMAN)	91.54	53.02	18	14	20	54	2.946E8	364	39.4	8.09
P18206-2	Isoform 1 of Vinculin OS=Homo sapiens GN=VCL - (VINC_HUMAN)	90.88	21.86	9	4	18	41	1.718E8	1066	116.6	6.09
B4DPS6	cDNA FLJ59371, highly similar to Exportin-2 OS=Homo sapiens PE=2 SV=1 - (B4DPS6_HUMAN)	90.73	5.36	9	1	3	53	6.618E9	634	71.7	6.20
Q06830	Peroxiredoxin-1 OS=Homo sapiens GN=PRDX1 PE=1 SV=1 - (PRDX1_HUMAN)	88.76	63.32	4	11	14	50	1.392E9	199	22.1	8.13
Q14204	Cytoplasmic dynein 1 heavy chain 1 OS=Homo sapiens GN=DYNC1H1 PE=1 SV=5 - (DYHC1_HUMAN)	88.58	9.19	1	1	37	88	3.270E9	4646	532.1	6.40
Q5CAQ5	Tumor rejection antigen (Gp96) 1 OS=Homo sapiens GN=TRA1 PE=2 SV=1 - (Q5CAQ5_HUMAN)	87.89	28.80	12	12	21	49	4.479E9	802	92.3	4.86
O94819	Kelch repeat and BTB domain-containing protein 11 OS=Homo sapiens GN=KBTBD11 PE=1 SV=1 - (KBTBB_HUMAN)	87.84	6.42	1	1	3	48	1.171E9	623	65.7	6.07
O15078	Centrosomal protein of 290 kDa OS=Homo sapiens GN=CEP290 PE=1 SV=2 - (CE290_HUMAN)	87.18	12.14	2	1	34	97	1.128E9	2479	290.2	5.95
H0YIG3	Keratin, type II cytoskeletal 72 (Fragment) OS=Homo sapiens GN=KRT72 PE=4 SV=1 - (H0YIG3_HUMAN)	86.19	18.75	1	2	4	48	6.933E8	192	21.7	11.19
Q02878	60S ribosomal protein L6 OS=Homo sapiens GN=RPL6 PE=1 SV=3 - (RL6_HUMAN)	86.08	36.81	13	6	12	56	3.052E8	288	32.7	10.58
Q6KB66	Keratin, type II cytoskeletal 80 OS=Homo sapiens GN=KRT80 PE=1 SV=2 - (K2C80_HUMAN)	85.93	31.42	5	4	12	52	6.638E9	452	50.5	5.67
P40926	Malate dehydrogenase, mitochondrial OS=Homo sapiens GN=MDH2 PE=1 SV=3 - (MDHM_HUMAN)	85.73	52.37	8	12	14	31	2.644E8	338	35.5	8.68
A4QPB0	IQ motif containing GTPase activating protein 1 OS=Homo sapiens GN=IQGAP1 PE=2 SV=1 - (A4QPB0_HUMAN)	85.07	15.15	22	9	20	41	9.938E7	1657	189.2	6.48
Q8N2C7-4	Isoform 4 of Protein unc-80 homolog OS=Homo sapiens GN=UNC80 - (UNC80_HUMAN)	84.74	4.37	4	1	12	45	1.795E9	3020	337.6	7.06
C7DJS2	Glutathione S-transferase pi (Fragment) OS=Homo sapiens GN=GSTP1 PE=2 SV=1 - (C7DJS2_HUMAN)	82.55	58.28	2	1	7	36	6.957E8	151	16.7	5.10
G9JV18	Coiled-coil domain containing 154 OS=Homo sapiens GN=CCDC154 PE=2 SV=1 - (G9JV18_HUMAN)	82.09	14.54	3	1	11	84	5.218E9	667	75.4	8.24
Q14152	Eukaryotic translation initiation factor 3 subunit A OS=Homo sapiens GN=EIF3A PE=1 SV=1 - (EIF3A_HUMAN)	80.71	29.74	8	9	41	76	9.895E8	1382	166.5	6.79
Q8NEH6	Meiosis-specific nuclear structural protein 1 OS=Homo sapiens GN=MNS1 PE=2 SV=2 - (MNS1_HUMAN)	80.70	26.67	2	2	14	60	1.597E9	495	60.5	7.12
Q8IUC1	Keratin-associated protein 11-1 OS=Homo sapiens GN=KRTAP11-1 PE=1 SV=1 - (KR111_HUMAN)	79.87	27.61	2	4	6	59	1.873E8	163	17.1	7.90
P46782	40S ribosomal protein S5 OS=Homo sapiens GN=RPS5 PE=1 SV=4 - (RS5_HUMAN)	79.61	36.27	2	5	10	25	3.205E8	204	22.9	9.72
P25054-2	Isoform Short of Adenomatous polyposis coli protein OS=Homo sapiens GN=APC - (APC_HUMAN)	79.50	10.76	5	1	23	76	1.067E9	2742	300.3	7.77
Q6TXQ4	H3L-like histone (Fragment) OS=Homo sapiens PE=2 SV=1 - (Q6TXQ4_HUMAN)	77.61	78.50	1	1	9	68	2.622E9	107	12.1	10.59
Q5VU43-4	Isoform 4 of Myomegalin OS=Homo sapiens GN=PDE4DIP - (MYOME_HUMAN)	74.59	13.55	26	3	27	70	9.973E8	2362	267.0	5.48
P18669	Phosphoglycerate mutase 1 OS=Homo sapiens GN=PGAM1 PE=1 SV=2 - (PGAM1_HUMAN)	74.13	66.93	14	9	12	32	3.364E8	254	28.8	7.18
B7Z6Z4	Myosin light polypeptide 6 OS=Homo sapiens GN=MYL6 PE=2 SV=1 - (B7Z6Z4_HUMAN)	74.13	43.28	20	6	9	29	4.957E8	238	26.7	5.08
Q01518-2	Isoform 2 of Adenylyl cyclase-associated protein 1 OS=Homo sapiens GN=CAP1 - (CAP1_HUMAN)	73.93	28.06	11	1	14	32	8.863E7	474	51.8	8.06
B2R5W2	cDNA, FLJ92657, highly similar to Homo sapiens heterogeneous nuclear ribonucleoprotein C (C1/C2) (HNRPC), transcript variant 2, mRNA OS=Homo sapiens PE=2 SV=1 - (B2R5W2_HUMAN)	73.58	36.21	33	6	11	33	1.496E8	290	31.9	5.24
Q6P9G8	Family with sequence similarity 184, member A OS=Homo sapiens GN=FAM184A PE=2 SV=2 - (Q6P9G8_HUMAN)	72.26	17.16	12	1	17	57	5.058E9	1107	129.4	5.72
Q5TZA2	Rootletin OS=Homo sapiens GN=CROCC PE=1 SV=1 - (CROCC_HUMAN)	71.89	18.84	5	2	40	68	6.692E8	2017	228.4	5.50
Q14134	Tripartite motif-containing protein 29 OS=Homo sapiens GN=TRIM29 PE=1 SV=2 - (TRI29_HUMAN)	71.66	39.97	19	18	25	53	3.405E8	588	65.8	7.15
B0AZQ4	cDNA, FLJ79494, highly similar to Structural maintenance of chromosome 3 OS=Homo sapiens PE=2 SV=1 - (B0AZQ4_HUMAN)	71.42	12.08	3	1	16	84	1.731E9	1217	141.4	7.18
P30050	60S ribosomal protein L12 OS=Homo sapiens GN=RPL12 PE=1 SV=1 - (RL12_HUMAN)	71.05	72.73	4	7	11	27	2.522E8	165	17.8	9.42
Q9Y490	Talin-1 OS=Homo sapiens GN=TLN1 PE=1 SV=3 - (TLN1_HUMAN)	69.72	7.99	2	5	18	44	6.110E8	2541	269.6	6.07
P11021	78 kDa glucose-regulated protein OS=Homo sapiens GN=HSPA5 PE=1 SV=2 - (GRP78_HUMAN)	69.14	29.82	2	8	17	40	4.696E8	654	72.3	5.16
Q4W4Y1	Dopamine receptor interacting protein 4 OS=Homo sapiens GN=DRIP4 PE=2 SV=1 - (Q4W4Y1_HUMAN)	68.21	16.13	6	6	14	42	2.925E8	868	96.0	6.52
P13489	Ribonuclease inhibitor OS=Homo sapiens GN=RNH1 PE=1 SV=2 - (RINI_HUMAN)	67.34	29.72	10	8	11	22	2.546E8	461	49.9	4.82
Q75MZ1	Putative uncharacterized protein ZNF277 (Fragment) OS=Homo sapiens GN=ZNF277 PE=2 SV=1 - (Q75MZ1_HUMAN)	66.72	11.58	11	1	3	34	1.727E9	259	30.7	7.15
Q96IR1	RPS4X protein (Fragment) OS=Homo sapiens GN=RPS4X PE=2 SV=2 - (Q96IR1_HUMAN)	66.35	53.50	11	12	15	32	1.866E8	243	27.2	9.94
P08758	Annexin A5 OS=Homo sapiens GN=ANXA5 PE=1 SV=2 - (ANXA5_HUMAN)	66.13	43.75	7	10	13	34	3.844E8	320	35.9	5.05
P16152	Carbonyl reductase (NADPH) 1 OS=Homo sapiens GN=CBR1 PE=1 SV=3 - (CBR1_HUMAN)	65.85	48.74	4	10	11	29	1.849E8	277	30.4	8.32
B4DUQ1	cDNA FLJ54552, highly similar to Heterogeneous nuclear ribonucleoprotein K OS=Homo sapiens PE=2 SV=1 - (B4DUQ1_HUMAN)	65.64	45.79	10	7	21	49	5.334E8	439	48.5	5.92
Q9Y446	Plakophilin-3 OS=Homo sapiens GN=PKP3 PE=1 SV=1 - (PKP3_HUMAN)	65.55	31.24	8	13	22	48	4.657E8	797	87.0	9.32
Q9HCY8	Protein S100-A14 OS=Homo sapiens GN=S100A14 PE=1 SV=1 - (S10AE_HUMAN)	65.18	93.27	1	8	10	23	1.424E8	104	11.7	5.24
P28838-2	Isoform 2 of Cytosol aminopeptidase OS=Homo sapiens GN=LAP3 - (AMPL_HUMAN)	64.89	29.51	4	7	13	36	1.591E8	488	52.7	6.74
P81605	Dermcidin OS=Homo sapiens GN=DCD PE=1 SV=2 - (DCD_HUMAN)	63.73	41.82	2	3	4	23	7.844E7	110	11.3	6.54
C9J9K3	40S ribosomal protein SA (Fragment) OS=Homo sapiens GN=RPSA PE=3 SV=1 - (C9J9K3_HUMAN)	62.37	30.68	11	6	8	23	1.948E8	264	29.5	5.25
P33764	Protein S100-A3 OS=Homo sapiens GN=S100A3 PE=1 SV=1 - (S10A3_HUMAN)	62.07	62.38	1	7	7	29	1.910E8	101	11.7	4.78
B9EK46	Cingulin OS=Homo sapiens GN=CGN PE=2 SV=1 - (B9EK46_HUMAN)	61.80	13.72	3	1	21	57	4.851E8	1203	137.0	5.52
A8K092	ATP synthase subunit alpha OS=Homo sapiens GN=ATP5A1 PE=2 SV=1 - (A8K092_HUMAN)	61.54	29.42	4	12	14	25	1.682E8	503	54.5	8.24
Q6ZQQ6	WD repeat-containing protein 87 OS=Homo sapiens GN=WDR87 PE=2 SV=3 - (WDR87_HUMAN)	60.87	9.75	4	2	29	62	4.212E8	2873	333.0	7.28
Q2Q9B7	Glucose-6-phosphate 1-dehydrogenase (Fragment) OS=Homo sapiens GN=G6PD PE=2 SV=1 - (Q2Q9B7_HUMAN)	60.34	14.95	13	1	7	42	3.145E8	475	54.8	7.08
Q5T6C5	Ataxin-7-like protein 2 OS=Homo sapiens GN=ATXN7L2 PE=2 SV=1 - (AT7L2_HUMAN)	60.13	10.11	2	1	9	45	9.465E8	722	77.1	9.23
Q6ZNE1	cDNA FLJ16186 fis, clone BRTHA2007060, moderately similar to EUKARYOTIC TRANSLATION INITIATION FACTOR 3 SUBUNIT 10 OS=Homo sapiens PE=2 SV=1 - (Q6ZNE1_HUMAN)	59.58	11.18	1	1	12	52	6.182E8	957	111.7	9.33
A6NHL2-2	Isoform 2 of Tubulin alpha chain-like 3 OS=Homo sapiens GN=TUBAL3 - (TBAL3_HUMAN)	59.07	24.14	2	1	10	30	4.202E8	406	45.5	6.32
O95613	Pericentrin OS=Homo sapiens GN=PCNT PE=1 SV=4 - (PCNT_HUMAN)	58.36	12.32	3	1	35	82	2.033E9	3336	377.8	5.55
P06748	Nucleophosmin OS=Homo sapiens GN=NPM1 PE=1 SV=2 - (NPM_HUMAN)	57.68	39.46	17	8	11	35	2.010E8	294	32.6	4.78
E9PKF4	Four and a half LIM domains protein 2 OS=Homo sapiens GN=FHL2 PE=4 SV=1 - (E9PKF4_HUMAN)	57.55	22.22	2	1	2	86	9.531E7	144	15.5	11.14
P07237	Protein disulfide-isomerase OS=Homo sapiens GN=P4HB PE=1 SV=3 - (PDIA1_HUMAN)	57.18	37.20	22	8	17	31	6.158E7	508	57.1	4.87
Q86TY5	Full-length cDNA clone CS0DI041YE05 of Placenta of Homo sapiens (human) OS=Homo sapiens PE=2 SV=1 - (Q86TY5_HUMAN)	57.16	76.03	16	8	10	49	4.079E8	121	13.9	9.16
F8VW21	60S acidic ribosomal protein P0 (Fragment) OS=Homo sapiens GN=RPLP0 PE=3 SV=1 - (F8VW21_HUMAN)	56.98	26.64	3	1	7	26	4.084E8	244	26.9	8.97
P62081	40S ribosomal protein S7 OS=Homo sapiens GN=RPS7 PE=1 SV=1 - (RS7_HUMAN)	56.51	58.25	3	8	11	22	1.450E8	194	22.1	10.10
F8W7K3	Spectrin alpha chain, brain OS=Homo sapiens GN=SPTAN1 PE=4 SV=1 - (F8W7K3_HUMAN)	56.35	10.20	10	6	19	36	3.852E8	2452	282.0	5.34
C9JKA9	Kinesin-like protein KIF15 OS=Homo sapiens GN=KIF15 PE=4 SV=1 - (C9JKA9_HUMAN)	55.75	13.00	8	2	12	38	1.878E9	1023	120.3	5.54
D3DX70	GRIP and coiled-coil domain containing 2, isoform CRA_a OS=Homo sapiens GN=GCC2 PE=4 SV=1 - (D3DX70_HUMAN)	55.35	10.11	6	2	18	49	5.991E8	1583	184.5	5.10
O15230	Laminin subunit alpha-5 OS=Homo sapiens GN=LAMA5 PE=1 SV=8 - (LAMA5_HUMAN)	54.87	4.30	2	1	15	50	6.431E8	3695	399.5	7.02
P06753-3	Isoform 3 of Tropomyosin alpha-3 chain OS=Homo sapiens GN=TPM3 - (TPM3_HUMAN)	54.19	58.70	53	4	18	31	4.105E8	247	28.9	4.81
P22314	Ubiquitin-like modifier-activating enzyme 1 OS=Homo sapiens GN=UBA1 PE=1 SV=3 - (UBA1_HUMAN)	54.18	13.52	5	6	10	21	5.453E8	1058	117.8	5.76
P07339	Cathepsin D OS=Homo sapiens GN=CTSD PE=1 SV=1 - (CATD_HUMAN)	54.16	20.63	6	7	7	25	1.421E8	412	44.5	6.54
P46783	40S ribosomal protein S10 OS=Homo sapiens GN=RPS10 PE=1 SV=1 - (RS10_HUMAN)	54.02	44.85	4	5	9	34	1.530E8	165	18.9	10.15
Q15075	Early endosome antigen 1 OS=Homo sapiens GN=EEA1 PE=1 SV=2 - (EEA1_HUMAN)	53.91	18.92	2	2	27	53	9.109E8	1411	162.4	5.68
Q86UP2-3	Isoform 3 of Kinectin OS=Homo sapiens GN=KTN1 - (KTN1_HUMAN)	53.16	13.94	11	4	15	53	9.742E8	1306	150.3	5.74
P58107	Epiplakin OS=Homo sapiens GN=EPPK1 PE=1 SV=2 - (EPIPL_HUMAN)	53.13	15.46	3	6	23	52	2.670E8	5090	555.3	5.60
E9PDB1	Non-erythrocytic beta-spectrin 4 OS=Homo sapiens GN=SPTBN4 PE=2 SV=2 - (E9PDB1_HUMAN)	53.04	10.34	6	2	24	60	1.410E9	2564	288.9	6.06
Q5U5K4	Homer homolog 1 (Drosophila) OS=Homo sapiens GN=HOMER1 PE=2 SV=1 - (Q5U5K4_HUMAN)	52.91	11.02	5	2	5	53	2.743E8	354	40.3	5.44
E9PRY8	Elongation factor 1-delta OS=Homo sapiens GN=EEF1D PE=3 SV=1 - (E9PRY8_HUMAN)	52.58	26.26	31	5	16	41	2.580E9	697	76.5	7.05
I3L3T4	TOM1-like protein 1 OS=Homo sapiens GN=TOM1L1 PE=4 SV=1 - (I3L3T4_HUMAN)	52.48	11.56	9	1	2	25	4.353E9	225	25.2	7.36
D3DTT5	TBK1 binding protein 1, isoform CRA_a OS=Homo sapiens GN=TBKBP1 PE=4 SV=1 - (D3DTT5_HUMAN)	52.28	18.79	4	2	12	56	5.593E8	463	50.3	5.26
B4DXS5	cDNA FLJ57802, highly similar to Zinc finger protein 225 OS=Homo sapiens PE=2 SV=1 - (B4DXS5_HUMAN)	52.28	1.49	1	2	2	29	6.946E7	670	78.1	8.92
P22626	Heterogeneous nuclear ribonucleoproteins A2/B1 OS=Homo sapiens GN=HNRNPA2B1 PE=1 SV=2 - (ROA2_HUMAN)	52.15	48.44	2	8	16	38	3.582E8	353	37.4	8.95
B3KWI4	cDNA FLJ43122 fis, clone CTONG3003737, highly similar to Leucine-rich repeat-containing protein 15 OS=Homo sapiens PE=2 SV=1 - (B3KWI4_HUMAN)	51.74	14.63	3	5	9	27	1.387E8	581	64.4	6.71
P67936-2	Isoform 2 of Tropomyosin alpha-4 chain OS=Homo sapiens GN=TPM4 - (TPM4_HUMAN)	51.52	51.76	44	3	17	30	4.060E8	284	32.7	4.73
Q8WUT4	Leucine-rich repeat neuronal protein 4 OS=Homo sapiens GN=LRRN4 PE=1 SV=3 - (LRRN4_HUMAN)	50.89	5.00	2	1	2	30	6.471E8	740	78.8	7.20
P07195	L-lactate dehydrogenase B chain OS=Homo sapiens GN=LDHB PE=1 SV=2 - (LDHB_HUMAN)	50.00	26.35	12	5	9	22	2.874E8	334	36.6	6.05
Q13439-3	Isoform 3 of Golgin subfamily A member 4 OS=Homo sapiens GN=GOLGA4 - (GOGA4_HUMAN)	49.88	14.53	5	1	30	59	5.145E8	2223	260.2	5.40
F5H3I4	Alpha-centractin OS=Homo sapiens GN=ACTR1A PE=3 SV=1 - (F5H3I4_HUMAN)	48.99	20.20	17	1	5	33	9.816E8	302	34.5	6.46
E9PR44	Alpha-crystallin B chain (Fragment) OS=Homo sapiens GN=CRYAB PE=3 SV=1 - (E9PR44_HUMAN)	48.86	72.41	10	8	14	20	1.448E8	174	20.0	7.03
E7EV56	Pericentriolar material 1 protein OS=Homo sapiens GN=PCM1 PE=4 SV=1 - (E7EV56_HUMAN)	48.64	8.76	18	2	16	49	3.067E8	1860	210.0	5.00
Q6ZRS2-3	Isoform 3 of Helicase SRCAP OS=Homo sapiens GN=SRCAP - (SRCAP_HUMAN)	48.61	5.92	3	1	18	51	1.821E9	3072	327.6	5.76
F8VXY0	Heterogeneous nuclear ribonucleoprotein A1 OS=Homo sapiens GN=HNRNPA1 PE=4 SV=1 - (F8VXY0_HUMAN)	47.94	62.83	20	9	16	25	1.575E8	304	32.7	9.44
B2RBR9	cDNA, FLJ95650, highly similar to Homo sapiens karyopherin (importin) beta 1 (KPNB1), mRNA OS=Homo sapiens PE=2 SV=1 - (B2RBR9_HUMAN)	47.84	11.87	6	4	8	18	1.142E8	876	97.1	4.78
P63241-2	Isoform 2 of Eukaryotic translation initiation factor 5A-1 OS=Homo sapiens GN=EIF5A - (IF5A1_HUMAN)	47.80	56.52	8	6	9	22	8.916E7	184	20.2	7.01
B2R9K8	cDNA, FLJ94440, highly similar to Homo sapiens chaperonin containing TCP1, subunit 6A (zeta 1)(CCT6A), mRNA OS=Homo sapiens PE=2 SV=1 - (B2R9K8_HUMAN)	47.65	24.48	7	3	13	23	6.201E8	531	57.9	6.80
F8WAQ9	Microtubule-associated serine/threonine-protein kinase 4 OS=Homo sapiens GN=MAST4 PE=4 SV=1 - (F8WAQ9_HUMAN)	47.46	10.47	9	1	22	43	1.603E9	2626	284.2	8.62
Q15365	Poly(rC)-binding protein 1 OS=Homo sapiens GN=PCBP1 PE=1 SV=2 - (PCBP1_HUMAN)	46.93	17.98	10	4	6	18	1.470E8	356	37.5	7.09
Q86UQ4	ATP-binding cassette sub-family A member 13 OS=Homo sapiens GN=ABCA13 PE=2 SV=3 - (ABCAD_HUMAN)	46.82	4.41	2	2	18	36	6.066E8	5058	575.8	6.46
Q8NGB0	Seven transmembrane helix receptor OS=Homo sapiens GN=GPR142 PE=2 SV=1 - (Q8NGB0_HUMAN)	46.73	10.11	1	1	13	46	2.562E9	1464	156.4	10.95
Q08ES8	Cell growth-inhibiting protein 34 OS=Homo sapiens PE=2 SV=1 - (Q08ES8_HUMAN)	46.47	41.81	5	6	8	25	2.904E8	177	20.1	9.60
P02461	Collagen alpha-1(III) chain OS=Homo sapiens GN=COL3A1 PE=1 SV=4 - (CO3A1_HUMAN)	45.62	5.12	1	1	7	29	9.376E7	1466	138.5	6.61
P11532-4	Isoform 3 of Dystrophin OS=Homo sapiens GN=DMD - (DMD_HUMAN)	45.52	8.40	11	1	29	74	6.799E8	3677	425.4	5.91
P16401	Histone H1.5 OS=Homo sapiens GN=HIST1H1B PE=1 SV=3 - (H15_HUMAN)	45.49	29.20	1	4	12	30	4.080E8	226	22.6	10.92
P06744-2	Isoform 2 of Glucose-6-phosphate isomerase OS=Homo sapiens GN=GPI - (G6PI_HUMAN)	45.11	15.82	5	4	8	30	1.510E9	569	64.3	8.91
B3KTP9	cDNA FLJ38578 fis, clone HCHON2007674, highly similar to NUCLEOLIN OS=Homo sapiens PE=2 SV=1 - (B3KTP9_HUMAN)	45.07	28.28	7	9	19	34	4.018E8	633	68.4	4.65
A4D1E4	A kinase (PRKA) anchor protein (Yotiao) 9 OS=Homo sapiens GN=AKAP9 PE=4 SV=1 - (A4D1E4_HUMAN)	45.07	8.64	10	2	13	33	3.577E8	1655	192.7	4.91
O60437	Periplakin OS=Homo sapiens GN=PPL PE=1 SV=4 - (PEPL_HUMAN)	44.99	13.84	1	7	25	35	4.749E8	1756	204.6	5.60
E7ETZ4	Basic leucine zipper and W2 domain-containing protein 2 (Fragment) OS=Homo sapiens GN=BZW2 PE=4 SV=1 - (E7ETZ4_HUMAN)	44.51	20.10	21	1	10	34	3.474E8	408	46.9	8.44
Q96Q15	Serine/threonine-protein kinase SMG1 OS=Homo sapiens GN=SMG1 PE=1 SV=3 - (SMG1_HUMAN)	44.44	4.64	5	1	16	44	1.264E9	3661	410.2	6.46
Q02487-2	Isoform 2B of Desmocollin-2 OS=Homo sapiens GN=DSC2 - (DSC2_HUMAN)	44.31	15.23	10	5	11	24	4.449E8	847	93.7	5.47
Q5JR95	40S ribosomal protein S8 OS=Homo sapiens GN=RPS8 PE=4 SV=1 - (Q5JR95_HUMAN)	44.16	56.38	3	6	9	17	5.052E8	188	21.9	10.36
Q9P2P6	StAR-related lipid transfer protein 9 OS=Homo sapiens GN=STARD9 PE=1 SV=3 - (STAR9_HUMAN)	43.82	6.85	2	1	29	58	2.364E9	4700	516.0	6.32
Q8NGR6	Olfactory receptor 1B1 OS=Homo sapiens GN=OR1B1 PE=2 SV=2 - (OR1B1_HUMAN)	43.66	3.46	1	1	1	19	1.165E8	318	35.3	8.16
B2RUT2	Thyroid hormone receptor interactor 11 OS=Homo sapiens GN=TRIP11 PE=2 SV=1 - (B2RUT2_HUMAN)	43.29	11.72	6	1	21	37	3.306E8	1979	227.5	5.26
Q9BV73-2	Isoform 2 of Centrosome-associated protein CEP250 OS=Homo sapiens GN=CEP250 - (CP250_HUMAN)	43.18	13.41	5	1	32	60	6.283E8	2386	274.2	5.03
Q8IVF2-3	Isoform 3 of Protein AHNAK2 OS=Homo sapiens GN=AHNAK2 - (AHNK2_HUMAN)	42.78	19.44	2	5	35	54	4.315E8	5695	605.3	5.36
Q8N9W4	Golgin subfamily A member 6-like protein 2 OS=Homo sapiens GN=GOLGA6L2 PE=2 SV=2 - (GG6L2_HUMAN)	42.75	19.08	3	1	11	47	4.061E9	650	79.3	5.54
F4MH43	Ubiquitously transcribed tetratricopeptide repeat protein Y-linked transcript variant 4 OS=Homo sapiens GN=UTY PE=2 SV=1 - (F4MH43_HUMAN)	42.40	2.72	1	1	3	23	5.648E9	955	105.1	7.78
B2R4M6	cDNA, FLJ92148, highly similar to Homo sapiens S100 calcium binding protein A9 (calgranulin B) (S100A9), mRNA OS=Homo sapiens PE=4 SV=1 - (B2R4M6_HUMAN)	42.30	48.25	2	6	7	22	2.577E8	114	13.2	6.13
Q7Z612	Acidic ribosomal phosphoprotein P1 OS=Homo sapiens PE=4 SV=1 - (Q7Z612_HUMAN)	41.31	14.16	4	1	1	12	3.380E8	113	11.4	4.36
P62857	40S ribosomal protein S28 OS=Homo sapiens GN=RPS28 PE=1 SV=1 - (RS28_HUMAN)	41.23	52.17	1	4	4	16	1.134E8	69	7.8	10.70
P49327	Fatty acid synthase OS=Homo sapiens GN=FASN PE=1 SV=3 - (FAS_HUMAN)	41.23	4.66	1	1	12	37	3.371E9	2511	273.3	6.44
Q9BYR8	Keratin-associated protein 3-1 OS=Homo sapiens GN=KRTAP3-1 PE=1 SV=1 - (KRA31_HUMAN)	41.13	24.49	1	2	2	11	2.410E8	98	10.5	6.40
P23284	Peptidyl-prolyl cis-trans isomerase B OS=Homo sapiens GN=PPIB PE=1 SV=2 - (PPIB_HUMAN)	41.09	38.89	3	6	8	19	1.524E8	216	23.7	9.41
P50395	Rab GDP dissociation inhibitor beta OS=Homo sapiens GN=GDI2 PE=1 SV=2 - (GDIB_HUMAN)	40.80	26.07	15	6	8	16	9.661E7	445	50.6	6.47
P61204	ADP-ribosylation factor 3 OS=Homo sapiens GN=ARF3 PE=1 SV=2 - (ARF3_HUMAN)	40.79	38.67	32	5	5	13	7.250E7	181	20.6	7.43
P01011	Alpha-1-antichymotrypsin OS=Homo sapiens GN=SERPINA3 PE=1 SV=2 - (AACT_HUMAN)	40.49	21.75	6	6	8	16	1.828E8	423	47.6	5.52
B2RAW1	cDNA, FLJ95155, highly similar to Homo sapiens Bruton agammaglobulinemia tyrosine kinase (BTK), mRNA OS=Homo sapiens PE=2 SV=1 - (B2RAW1_HUMAN)	40.48	8.80	14	1	4	30	9.119E8	659	76.2	7.77
Q4L180-3	Isoform 3 of Filamin A-interacting protein 1-like OS=Homo sapiens GN=FILIP1L - (FIL1L_HUMAN)	40.46	12.54	7	1	9	23	8.332E8	893	102.2	6.58
D3DV26	S100 calcium binding protein A10 (Annexin II ligand, calpactin I, light polypeptide (P11)), isoform CRA_b (Fragment) OS=Homo sapiens GN=S100A10 PE=4 SV=1 - (D3DV26_HUMAN)	40.44	29.76	3	5	7	40	3.952E8	205	22.3	10.33
P22392-2	Isoform 3 of Nucleoside diphosphate kinase B OS=Homo sapiens GN=NME2 - (NDKB_HUMAN)	40.41	59.55	10	7	10	24	1.496E8	267	30.1	8.92
B2R643	cDNA, FLJ92777, highly similar to Homo sapiens myosin IA (MYO1A), mRNA OS=Homo sapiens PE=2 SV=1 - (B2R643_HUMAN)	39.91	15.44	11	2	13	40	4.503E8	1043	118.3	9.31
H0YC11	Alpha-1,3-mannosyl-glycoprotein 4-beta-N-acetylglucosaminyltransferase B (Fragment) OS=Homo sapiens GN=MGAT4B PE=4 SV=1 - (H0YC11_HUMAN)	39.62	12.12	6	1	5	25	1.852E8	429	47.2	7.64
Q9UIF8-4	Isoform 4 of Bromodomain adjacent to zinc finger domain protein 2B OS=Homo sapiens GN=BAZ2B - (BAZ2B_HUMAN)	39.47	5.27	5	1	14	44	5.900E8	1972	220.6	6.37
P62851	40S ribosomal protein S25 OS=Homo sapiens GN=RPS25 PE=1 SV=1 - (RS25_HUMAN)	39.43	40.00	1	6	7	19	2.979E8	125	13.7	10.11
H7C181	Sialic acid-binding Ig-like lectin 11 (Fragment) OS=Homo sapiens GN=SIGLEC11 PE=4 SV=1 - (H7C181_HUMAN)	39.24	6.08	3	1	3	28	2.213E8	592	64.8	7.80
B4DQY1	cDNA FLJ56133, highly similar to Serine/threonine-protein phosphatase 2A 65 kDa regulatory subunit A alpha isoform OS=Homo sapiens PE=2 SV=1 - (B4DQY1_HUMAN)	39.17	14.59	12	1	6	14	6.470E7	555	61.5	5.12
Q9UFD9	RIMS-binding protein 3A OS=Homo sapiens GN=RIMBP3 PE=1 SV=4 - (RIM3A_HUMAN)	39.12	7.99	9	1	15	45	1.771E8	1639	180.6	6.89
B4DLR3	cDNA FLJ54020, highly similar to Heterogeneous nuclear ribonucleoprotein U OS=Homo sapiens PE=2 SV=1 - (B4DLR3_HUMAN)	39.03	15.94	8	3	13	42	1.498E9	784	86.8	6.43
E9PL22	Hypoxia up-regulated protein 1 OS=Homo sapiens GN=HYOU1 PE=3 SV=1 - (E9PL22_HUMAN)	38.96	14.41	12	1	11	32	1.305E9	937	104.7	5.72
B2R717	cDNA, FLJ93235, highly similar to Homo sapiens cathepsin L2 (CTSL2), mRNA OS=Homo sapiens PE=2 SV=1 - (B2R717_HUMAN)	38.95	23.05	2	6	8	21	2.804E8	334	37.3	8.76
Q86VG2	Splicing factor proline/glutamine-rich (Polypyrimidine tract binding protein associated) OS=Homo sapiens GN=SFPQ PE=2 SV=1 - (Q86VG2_HUMAN)	38.93	27.86	11	5	22	37	2.408E8	707	76.1	9.48
P62269	40S ribosomal protein S18 OS=Homo sapiens GN=RPS18 PE=1 SV=3 - (RS18_HUMAN)	38.82	43.42	2	6	10	25	3.423E8	152	17.7	10.99
Q8NHW5	60S acidic ribosomal protein P0-like OS=Homo sapiens GN=RPLP0P6 PE=5 SV=1 - (RLA0L_HUMAN)	38.69	32.18	17	1	10	22	3.204E8	317	34.3	5.55
P83731	60S ribosomal protein L24 OS=Homo sapiens GN=RPL24 PE=1 SV=1 - (RL24_HUMAN)	38.60	53.50	3	7	12	25	2.610E8	157	17.8	11.25
A8K4W0	cDNA FLJ78591, highly similar to Homo sapiens ribosomal protein S3A (RPS3A), mRNA OS=Homo sapiens PE=2 SV=1 - (A8K4W0_HUMAN)	38.48	54.17	16	10	19	31	2.339E8	264	29.9	9.73
P52272-2	Isoform 2 of Heterogeneous nuclear ribonucleoprotein M OS=Homo sapiens GN=HNRNPM - (HNRPM_HUMAN)	38.45	28.08	7	8	21	51	9.557E8	691	73.6	8.82
B4DSG0	Janus kinase and microtubule-interacting protein 2 OS=Homo sapiens GN=JAKMIP2 PE=2 SV=1 - (B4DSG0_HUMAN)	38.10	19.67	7	2	15	68	4.227E8	778	91.5	5.78
Q96L96	Alpha-protein kinase 3 OS=Homo sapiens GN=ALPK3 PE=2 SV=2 - (ALPK3_HUMAN)	38.01	9.02	1	1	13	40	1.539E9	1907	201.1	7.58
P30622-1	Isoform 2 of CAP-Gly domain-containing linker protein 1 OS=Homo sapiens GN=CLIP1 - (CLIP1_HUMAN)	37.58	10.02	3	1	14	35	1.028E9	1427	160.9	5.39
Q96F88	Processing of 1, ribonuclease P/MRP subunit (S. cerevisiae) OS=Homo sapiens GN=POP1 PE=2 SV=1 - (Q96F88_HUMAN)	37.46	12.50	2	1	12	40	2.074E9	1024	114.6	9.20
Q96GM5-2	Isoform 2 of SWI/SNF-related matrix-associated actin-dependent regulator of chromatin subfamily D member 1 OS=Homo sapiens GN=SMARCD1 - (SMRD1_HUMAN)	37.33	5.06	6	1	4	20	5.138E8	474	53.3	9.39
Q5BJF6-3	Isoform 3 of Outer dense fiber protein 2 OS=Homo sapiens GN=ODF2 - (ODFP2_HUMAN)	37.11	24.35	6	1	17	45	9.830E8	805	93.2	7.08
P11055	Myosin-3 OS=Homo sapiens GN=MYH3 PE=1 SV=3 - (MYH3_HUMAN)	37.10	9.54	2	1	18	43	8.155E8	1940	223.8	5.81
P31944	Caspase-14 OS=Homo sapiens GN=CASP14 PE=1 SV=2 - (CASPE_HUMAN)	37.01	24.79	2	6	7	19	1.323E8	242	27.7	5.58
O15231-3	Isoform 3 of Zinc finger protein 185 OS=Homo sapiens GN=ZNF185 - (ZN185_HUMAN)	36.97	13.91	17	1	9	27	6.836E8	690	73.6	7.01
P46781	40S ribosomal protein S9 OS=Homo sapiens GN=RPS9 PE=1 SV=3 - (RS9_HUMAN)	36.89	60.82	7	10	14	21	1.534E8	194	22.6	10.65
Q05682	Caldesmon OS=Homo sapiens GN=CALD1 PE=1 SV=3 - (CALD1_HUMAN)	36.34	15.64	17	2	12	30	1.301E8	793	93.2	5.66
Q9BYK8	Peroxisomal proliferator-activated receptor A-interacting complex 285 kDa protein OS=Homo sapiens GN=PRIC285 PE=1 SV=6 - (PR285_HUMAN)	35.80	6.12	4	1	14	33	6.218E8	2649	294.5	7.49
O00221	NF-kappa-B inhibitor epsilon OS=Homo sapiens GN=NFKBIE PE=1 SV=3 - (IKBE_HUMAN)	35.69	11.40	6	1	4	22	4.973E7	500	52.8	6.68
P02549-2	Isoform 2 of Spectrin alpha chain, erythrocyte OS=Homo sapiens GN=SPTA1 - (SPTA1_HUMAN)	35.64	10.18	2	1	26	41	2.318E8	2416	279.5	5.05
Q5VZ66	Janus kinase and microtubule-interacting protein 3 OS=Homo sapiens GN=JAKMIP3 PE=2 SV=2 - (JKIP3_HUMAN)	35.45	20.38	1	1	18	37	8.831E8	844	98.5	5.66
O43497-5	Isoform 4 of Voltage-dependent T-type calcium channel subunit alpha-1G OS=Homo sapiens GN=CACNA1G - (CAC1G_HUMAN)	35.23	5.62	35	1	11	26	9.072E7	2171	240.8	7.08
B3KMF7	cDNA FLJ10863 fis, clone NT2RP4001575, highly similar to Vacuolar protein sorting protein 52 OS=Homo sapiens PE=2 SV=1 - (B3KMF7_HUMAN)	34.89	6.55	1	1	3	26	2.113E8	534	61.3	8.60
P35221	Catenin alpha-1 OS=Homo sapiens GN=CTNNA1 PE=1 SV=1 - (CTNA1_HUMAN)	34.76	21.19	24	7	19	36	1.828E8	906	100.0	6.29
Q2M329	Coiled-coil domain-containing protein 96 OS=Homo sapiens GN=CCDC96 PE=2 SV=2 - (CCD96_HUMAN)	34.75	10.63	1	1	5	41	2.206E8	555	62.7	4.94
D3DSS6	Dedicator of cytokinesis 5, isoform CRA_a OS=Homo sapiens GN=DOCK5 PE=4 SV=1 - (D3DSS6_HUMAN)	34.68	9.09	5	1	15	41	6.187E9	1860	213.9	7.90
P26641	Elongation factor 1-gamma OS=Homo sapiens GN=EEF1G PE=1 SV=3 - (EF1G_HUMAN)	34.45	19.45	5	4	9	21	1.056E8	437	50.1	6.67
A8MY67	Serine/threonine-protein phosphatase 2A 65 kDa regulatory subunit A beta isoform OS=Homo sapiens GN=PPP2R1B PE=4 SV=2 - (A8MY67_HUMAN)	34.40	18.14	11	1	5	11	6.446E7	474	52.2	4.84
P78347-2	Isoform 2 of General transcription factor II-I OS=Homo sapiens GN=GTF2I - (GTF2I_HUMAN)	34.36	7.21	8	1	8	24	1.063E9	957	107.9	7.94
Q8N300	Coiled-coil domain-containing protein 23 OS=Homo sapiens GN=CCDC23 PE=2 SV=1 - (CCD23_HUMAN)	34.35	19.70	1	2	4	28	3.304E8	66	7.8	9.16
P12270	Nucleoprotein TPR OS=Homo sapiens GN=TPR PE=1 SV=3 - (TPR_HUMAN)	34.08	12.19	2	1	26	52	6.941E8	2363	267.1	5.02
Q9UJ55	MAGE-like protein 2 OS=Homo sapiens GN=MAGEL2 PE=1 SV=1 - (MAGL2_HUMAN)	34.08	8.51	5	1	3	16	6.370E7	529	58.6	7.99
B3KPM8	cDNA FLJ31974 fis, clone NT2RP7008167, weakly similar to 85.1 kDa PROTEIN IN GREB-FEOA INTERGENIC REGION OS=Homo sapiens PE=2 SV=1 - (B3KPM8_HUMAN)	34.01	6.73	2	1	6	21	4.621E9	995	111.7	8.72
Q9NVU3	cDNA FLJ10506 fis, clone NT2RP2000510 OS=Homo sapiens PE=2 SV=1 - (Q9NVU3_HUMAN)	33.93	10.65	5	1	9	32	1.145E9	864	96.3	6.65
P07741	Adenine phosphoribosyltransferase OS=Homo sapiens GN=APRT PE=1 SV=2 - (APT_HUMAN)	33.84	41.67	7	6	7	12	2.059E8	180	19.6	6.02
P14174	Macrophage migration inhibitory factor OS=Homo sapiens GN=MIF PE=1 SV=4 - (MIF_HUMAN)	33.62	17.39	1	1	3	14	4.516E8	115	12.5	7.88
P62249	40S ribosomal protein S16 OS=Homo sapiens GN=RPS16 PE=1 SV=2 - (RS16_HUMAN)	33.54	34.93	3	6	6	16	2.334E8	146	16.4	10.21
Q504W7	Oculocerebrorenal syndrome of Lowe OS=Homo sapiens GN=OCRL PE=2 SV=1 - (Q504W7_HUMAN)	33.25	12.88	3	1	9	35	5.608E8	893	103.1	6.77
G3V203	60S ribosomal protein L18 OS=Homo sapiens GN=RPL18 PE=3 SV=1 - (G3V203_HUMAN)	33.14	51.22	9	6	9	17	1.411E8	164	18.7	11.59
Q96FQ6	Protein S100-A16 OS=Homo sapiens GN=S100A16 PE=1 SV=1 - (S10AG_HUMAN)	33.11	76.70	1	6	7	15	3.991E7	103	11.8	6.79
A8MTN3	Putative keratin-associated protein ENSP00000381495 OS=Homo sapiens PE=3 SV=2 - (KRA2X_HUMAN)	32.96	26.83	7	2	2	17	2.724E8	123	12.9	7.81
P07476	Involucrin OS=Homo sapiens GN=IVL PE=1 SV=2 - (INVO_HUMAN)	32.86	26.84	3	6	13	27	6.841E7	585	68.4	4.61
Q66LE6	Serine/threonine-protein phosphatase 2A 55 kDa regulatory subunit B delta isoform OS=Homo sapiens GN=PPP2R2D PE=1 SV=1 - (2ABD_HUMAN)	32.80	9.93	17	2	4	14	7.772E7	453	52.0	6.39
Q3LIE3	Putative uncharacterized protein Nbla04137 (Fragment) OS=Homo sapiens GN=Nbla04137 PE=2 SV=1 - (Q3LIE3_HUMAN)	32.75	24.57	1	1	5	19	4.604E8	289	33.3	5.21
P15880	40S ribosomal protein S2 OS=Homo sapiens GN=RPS2 PE=1 SV=2 - (RS2_HUMAN)	32.62	39.25	19	6	13	24	5.655E8	293	31.3	10.24
O00571-2	Isoform 2 of ATP-dependent RNA helicase DDX3X OS=Homo sapiens GN=DDX3X - (DDX3X_HUMAN)	32.61	22.14	11	7	11	16	3.065E8	646	71.3	6.62
Q04656-5	Isoform 5 of Copper-transporting ATPase 1 OS=Homo sapiens GN=ATP7A - (ATP7A_HUMAN)	32.58	4.43	18	2	7	26	5.715E8	1422	154.3	6.05
Q9BTQ7	Similar to ribosomal protein L23 (Fragment) OS=Homo sapiens PE=1 SV=1 - (Q9BTQ7_HUMAN)	32.54	42.54	4	4	5	9	1.216E8	134	14.1	10.26
P40925	Malate dehydrogenase, cytoplasmic OS=Homo sapiens GN=MDH1 PE=1 SV=4 - (MDHC_HUMAN)	32.35	37.72	11	5	10	13	1.504E8	334	36.4	7.36
Q3KNW1	Zinc finger protein SNAI3 OS=Homo sapiens GN=SNAI3 PE=2 SV=1 - (SNAI3_HUMAN)	32.14	10.62	1	1	4	27	1.224E8	292	32.5	9.22
Q05BA3	CCDC19 protein OS=Homo sapiens GN=CCDC19 PE=2 SV=1 - (Q05BA3_HUMAN)	31.95	34.98	3	1	17	38	3.576E8	466	56.5	8.41
Q2VIP3	PABP3 (Fragment) OS=Homo sapiens PE=2 SV=1 - (Q2VIP3_HUMAN)	31.85	21.43	9	1	11	32	8.401E8	630	69.9	9.67
P38646	Stress-70 protein, mitochondrial OS=Homo sapiens GN=HSPA9 PE=1 SV=2 - (GRP75_HUMAN)	31.85	16.64	9	4	9	19	9.484E8	679	73.6	6.16
P60953	Cell division control protein 42 homolog OS=Homo sapiens GN=CDC42 PE=1 SV=2 - (CDC42_HUMAN)	31.79	30.89	8	4	4	11	4.946E7	191	21.2	6.55
A7E2Y1	Myosin-7B OS=Homo sapiens GN=MYH7B PE=2 SV=3 - (MYH7B_HUMAN)	31.66	14.73	1	1	28	47	1.143E9	1941	221.3	6.01
B3KSS3	cDNA FLJ36854 fis, clone ASTRO2014561, highly similar to Peroxisomal membrane protein 11B OS=Homo sapiens PE=2 SV=1 - (B3KSS3_HUMAN)	31.42	18.11	4	1	2	17	9.058E7	127	13.4	9.48
Q9P2K8-2	Isoform 2 of Eukaryotic translation initiation factor 2-alpha kinase 4 OS=Homo sapiens GN=EIF2AK4 - (E2AK4_HUMAN)	31.29	8.95	3	1	12	33	9.711E8	1621	183.6	6.42
B7Z400	cDNA FLJ50558, highly similar to Homo sapiens microtubule-associated protein 7 (MAP7), mRNA OS=Homo sapiens PE=2 SV=1 - (B7Z400_HUMAN)	31.28	12.97	14	1	10	27	4.251E8	771	87.0	9.77
Q9P1F3	Costars family protein ABRACL OS=Homo sapiens GN=ABRACL PE=1 SV=1 - (ABRAL_HUMAN)	31.21	27.16	1	3	4	11	1.718E7	81	9.1	6.29
Q6NS36	Ferritin (Fragment) OS=Homo sapiens GN=FTH1 PE=2 SV=1 - (Q6NS36_HUMAN)	31.09	25.00	14	2	6	27	8.673E8	232	26.2	6.52
H3BLZ6	Sterol regulatory element-binding protein 1 (Fragment) OS=Homo sapiens GN=SREBF1 PE=4 SV=1 - (H3BLZ6_HUMAN)	30.72	6.93	13	1	10	44	6.196E9	1155	122.3	8.88
B4E060	Coiled-coil domain-containing protein 150 OS=Homo sapiens GN=CCDC150 PE=2 SV=1 - (B4E060_HUMAN)	30.64	28.91	1	1	2	17	2.441E9	128	15.2	9.50
O60486	Plexin-C1 OS=Homo sapiens GN=PLXNC1 PE=1 SV=1 - (PLXC1_HUMAN)	30.57	6.70	1	1	8	28	1.452E9	1568	175.6	7.61
F8WBW2	Coiled-coil domain-containing protein 39 OS=Homo sapiens GN=CCDC39 PE=4 SV=1 - (F8WBW2_HUMAN)	30.38	7.30	3	1	5	18	4.950E8	671	79.0	6.13
O43566-5	Isoform 3 of Regulator of G-protein signaling 14 OS=Homo sapiens GN=RGS14 - (RGS14_HUMAN)	30.10	7.00	4	1	3	18	6.698E8	414	44.8	9.20
P15090	Fatty acid-binding protein, adipocyte OS=Homo sapiens GN=FABP4 PE=1 SV=3 - (FABP4_HUMAN)	30.05	67.42	4	5	7	14	3.681E8	132	14.7	7.14
P78371	T-complex protein 1 subunit beta OS=Homo sapiens GN=CCT2 PE=1 SV=4 - (TCPB_HUMAN)	30.01	25.42	6	4	12	16	3.108E8	535	57.5	6.46
E7EVA0	Microtubule-associated protein OS=Homo sapiens GN=MAP4 PE=4 SV=1 - (E7EVA0_HUMAN)	29.96	10.36	14	2	19	29	6.703E8	2297	245.3	6.23
H3BUB1	Leucine-rich repeat-containing protein 9 (Fragment) OS=Homo sapiens GN=LRRC9 PE=4 SV=1 - (H3BUB1_HUMAN)	29.79	25.00	1	1	2	19	3.087E9	112	13.3	9.94
Q5CZC0	Fibrous sheath-interacting protein 2 OS=Homo sapiens GN=FSIP2 PE=1 SV=4 - (FSIP2_HUMAN)	29.66	6.05	4	2	37	63	1.091E9	6907	780.1	6.71
E9PCF4	Kelch-like protein 5 OS=Homo sapiens GN=KLHL5 PE=4 SV=1 - (E9PCF4_HUMAN)	29.66	3.70	10	1	2	46	9.612E7	568	63.5	6.98
E7EUE6	Sodium channel protein type 3 subunit alpha (Fragment) OS=Homo sapiens GN=SCN3A PE=4 SV=1 - (E7EUE6_HUMAN)	29.65	7.18	16	1	8	53	2.444E8	1364	154.4	5.55
O00362	Putative p150 OS=Homo sapiens PE=4 SV=1 - (O00362_HUMAN)	29.55	15.45	20	1	23	37	1.698E9	1275	149.1	9.69
B4DRD7	Poly(rC)-binding protein 2 OS=Homo sapiens GN=PCBP2 PE=2 SV=1 - (B4DRD7_HUMAN)	29.46	18.09	28	3	4	10	7.450E7	282	29.7	7.71
P04080	Cystatin-B OS=Homo sapiens GN=CSTB PE=1 SV=2 - (CYTB_HUMAN)	29.40	73.47	1	3	5	17	1.329E8	98	11.1	7.56
Q96QA5	Gasdermin-A OS=Homo sapiens GN=GSDMA PE=2 SV=4 - (GSDMA_HUMAN)	29.39	22.25	1	4	8	13	4.984E7	445	49.3	5.29
Q15057	Arf-GAP with coiled-coil, ANK repeat and PH domain-containing protein 2 OS=Homo sapiens GN=ACAP2 PE=1 SV=3 - (ACAP2_HUMAN)	29.31	11.05	2	1	8	41	1.693E8	778	88.0	6.80
Q8N6N2-2	Isoform 2 of Tetratricopeptide repeat protein 9B OS=Homo sapiens GN=TTC9B - (TTC9B_HUMAN)	29.31	9.90	2	1	2	19	3.961E7	192	19.5	9.50
P31151	Protein S100-A7 OS=Homo sapiens GN=S100A7 PE=1 SV=4 - (S10A7_HUMAN)	29.29	35.64	2	4	4	14	9.521E7	101	11.5	6.77
Q8IY18	Structural maintenance of chromosomes protein 5 OS=Homo sapiens GN=SMC5 PE=1 SV=2 - (SMC5_HUMAN)	29.10	19.80	2	1	18	34	8.568E8	1101	128.7	8.38
Q53FR4	Vacuolar protein sorting 35 variant (Fragment) OS=Homo sapiens PE=2 SV=1 - (Q53FR4_HUMAN)	28.94	18.59	4	5	12	18	1.249E8	796	91.6	5.49
P39019	40S ribosomal protein S19 OS=Homo sapiens GN=RPS19 PE=1 SV=2 - (RS19_HUMAN)	28.92	66.21	2	8	12	20	2.480E8	145	16.1	10.32
Q8IZX4	Transcription initiation factor TFIID subunit 1-like OS=Homo sapiens GN=TAF1L PE=1 SV=1 - (TAF1L_HUMAN)	28.79	9.97	6	1	19	50	4.610E8	1826	207.2	5.40
B4DX08	cDNA FLJ58784, highly similar to T-complex protein 1 subunit epsilon OS=Homo sapiens PE=2 SV=1 - (B4DX08_HUMAN)	28.78	12.88	17	2	5	14	3.088E7	520	57.1	5.81
Q86SJ6	Desmoglein-4 OS=Homo sapiens GN=DSG4 PE=1 SV=1 - (DSG4_HUMAN)	28.78	11.06	2	5	11	21	9.047E7	1040	113.8	4.56
E9PCY7	Heterogeneous nuclear ribonucleoprotein H, N-terminally processed OS=Homo sapiens GN=HNRNPH1 PE=4 SV=1 - (E9PCY7_HUMAN)	28.75	15.62	26	2	7	13	8.190E7	429	47.1	6.34
O75629	Protein CREG1 OS=Homo sapiens GN=CREG1 PE=1 SV=1 - (CREG1_HUMAN)	28.67	25.91	1	1	5	17	4.085E7	220	24.1	7.59
P63173	60S ribosomal protein L38 OS=Homo sapiens GN=RPL38 PE=1 SV=2 - (RL38_HUMAN)	28.62	50.00	1	4	4	9	1.258E8	70	8.2	10.10
E9PGH8	TBC1 domain family member 1 OS=Homo sapiens GN=TBC1D1 PE=4 SV=1 - (E9PGH8_HUMAN)	28.53	13.03	8	1	13	26	2.099E8	1159	131.3	7.58
Q86WN1	FCH and double SH3 domains protein 1 OS=Homo sapiens GN=FCHSD1 PE=1 SV=1 - (FCSD1_HUMAN)	28.49	15.36	4	1	11	23	1.408E8	690	76.9	5.34
Q12914	G2 (Fragment) OS=Homo sapiens PE=2 SV=1 - (Q12914_HUMAN)	28.39	7.80	6	1	10	23	2.125E8	1692	183.1	8.88
B4DZQ5	cDNA FLJ51417, highly similar to Serine/threonine-protein kinase PRP4 homolog (EC 2.7.11.1) OS=Homo sapiens PE=2 SV=1 - (B4DZQ5_HUMAN)	28.28	7.85	3	1	10	26	1.124E9	993	115.4	10.27
Q07157-2	Isoform Short of Tight junction protein ZO-1 OS=Homo sapiens GN=TJP1 - (ZO1_HUMAN)	28.15	8.21	7	1	15	23	1.548E8	1668	186.9	6.79
P20930	Filaggrin OS=Homo sapiens GN=FLG PE=1 SV=3 - (FILA_HUMAN)	27.97	20.51	11	2	37	65	2.710E8	4061	434.9	9.25
Q08999	Retinoblastoma-like protein 2 OS=Homo sapiens GN=RBL2 PE=1 SV=3 - (RBL2_HUMAN)	27.92	6.06	4	1	8	27	2.638E8	1139	128.3	7.44
Q5JPC5	Putative uncharacterized protein DKFZp667E1714 OS=Homo sapiens GN=DKFZp667E1714 PE=2 SV=1 - (Q5JPC5_HUMAN)	27.84	8.96	4	1	5	19	3.549E8	424	49.2	8.60
B4DFZ6	cDNA FLJ51823, highly similar to Homo sapiens BRF2, subunit of RNA polymerase III transcription initiation factor, BRF1-like (BRF2), mRNA OS=Homo sapiens PE=2 SV=1 - (B4DFZ6_HUMAN)	27.71	9.09	4	1	3	42	9.828E7	396	44.1	8.50
Q9H1R3	Myosin light chain kinase 2, skeletal/cardiac muscle OS=Homo sapiens GN=MYLK2 PE=1 SV=3 - (MYLK2_HUMAN)	27.66	5.20	1	1	4	17	3.186E8	596	64.6	7.02
Q14004	Cyclin-dependent kinase 13 OS=Homo sapiens GN=CDK13 PE=1 SV=2 - (CDK13_HUMAN)	27.40	10.58	176	1	15	28	9.047E8	1512	164.8	9.69
P63244	Guanine nucleotide-binding protein subunit beta-2-like 1 OS=Homo sapiens GN=GNB2L1 PE=1 SV=3 - (GBLP_HUMAN)	27.29	32.81	23	8	10	12	2.099E8	317	35.1	7.69
P32456	Interferon-induced guanylate-binding protein 2 OS=Homo sapiens GN=GBP2 PE=2 SV=3 - (GBP2_HUMAN)	27.22	6.26	1	1	4	20	6.340E9	591	67.2	5.71
A6H8W8	Intersectin 2 OS=Homo sapiens GN=ITSN2 PE=2 SV=1 - (A6H8W8_HUMAN)	27.02	10.78	10	1	18	33	6.763E8	1669	190.2	8.18
B4DJ75	Serine/threonine-protein phosphatase OS=Homo sapiens PE=2 SV=1 - (B4DJ75_HUMAN)	26.77	25.42	24	4	7	12	6.351E7	299	34.1	6.16
P51648	Fatty aldehyde dehydrogenase OS=Homo sapiens GN=ALDH3A2 PE=1 SV=1 - (AL3A2_HUMAN)	26.75	6.39	6	2	3	11	2.692E8	485	54.8	7.88
B4DJ30	cDNA FLJ61290, highly similar to Neutral alpha-glucosidase AB OS=Homo sapiens PE=2 SV=1 - (B4DJ30_HUMAN)	26.69	11.76	11	6	10	13	9.705E8	995	112.9	6.06
P30086	Phosphatidylethanolamine-binding protein 1 OS=Homo sapiens GN=PEBP1 PE=1 SV=3 - (PEBP1_HUMAN)	26.61	40.11	2	5	5	7	1.088E8	187	21.0	7.53
B4DG54	cDNA FLJ56635 OS=Homo sapiens PE=2 SV=1 - (B4DG54_HUMAN)	26.41	9.11	2	1	7	18	1.533E9	922	105.2	5.76
O15085-2	Isoform 2 of Rho guanine nucleotide exchange factor 11 OS=Homo sapiens GN=ARHGEF11 - (ARHGB_HUMAN)	26.39	9.92	2	1	11	23	3.432E8	1562	172.1	5.54
H0Y8G5	Heterogeneous nuclear ribonucleoprotein D0 (Fragment) OS=Homo sapiens GN=HNRNPD PE=4 SV=1 - (H0Y8G5_HUMAN)	26.35	22.22	19	3	6	13	1.267E8	261	29.7	9.16
Q5VX52	Spermatogenesis-associated protein 1 OS=Homo sapiens GN=SPATA1 PE=2 SV=3 - (SPAT1_HUMAN)	26.12	20.37	3	2	9	20	1.523E8	437	50.3	8.43
Q96L19	L-lactate dehydrogenase OS=Homo sapiens GN=LDHAL6A PE=2 SV=1 - (Q96L19_HUMAN)	25.98	20.51	10	1	5	13	1.895E8	234	25.6	7.87
B4DTQ2	cDNA FLJ56889, moderately similar to Vigilin OS=Homo sapiens PE=2 SV=1 - (B4DTQ2_HUMAN)	25.58	9.23	17	2	12	22	9.233E7	1235	137.9	6.89
O00299	Chloride intracellular channel protein 1 OS=Homo sapiens GN=CLIC1 PE=1 SV=4 - (CLIC1_HUMAN)	25.41	27.80	10	2	6	12	8.827E7	241	26.9	5.17
B4DHC2	cDNA FLJ56434, highly similar to p130Cas-associated protein OS=Homo sapiens PE=2 SV=1 - (B4DHC2_HUMAN)	25.34	9.95	1	1	9	34	2.551E8	1035	111.0	9.41
C9JYT9	Vacuolar protein sorting-associated protein 37D OS=Homo sapiens GN=VPS37D PE=4 SV=1 - (C9JYT9_HUMAN)	25.32	15.66	2	1	2	12	5.040E8	166	18.0	11.27
P39023	60S ribosomal protein L3 OS=Homo sapiens GN=RPL3 PE=1 SV=2 - (RL3_HUMAN)	25.26	18.11	13	2	9	17	2.128E8	403	46.1	10.18
Q8IVI3	Macrophage migration inhibitory factor homolog/MIF homolog (Fragment) OS=Homo sapiens PE=2 SV=1 - (Q8IVI3_HUMAN)	25.19	18.18	1	1	1	18	5.427E6	55	6.0	8.03
Q9HCD5	Nuclear receptor coactivator 5 OS=Homo sapiens GN=NCOA5 PE=1 SV=2 - (NCOA5_HUMAN)	25.17	14.51	1	1	12	36	8.902E8	579	65.5	9.60
Q5JPF3	Ankyrin repeat domain-containing protein 36C OS=Homo sapiens GN=ANKRD36C PE=2 SV=3 - (AN36C_HUMAN)	24.98	15.35	4	1	22	37	5.051E8	1778	199.6	7.83
B7ZAX3	cDNA, FLJ79337, weakly similar to Homo sapiens phosphodiesterase 4D interacting protein, transcript variant 1, mRNA OS=Homo sapiens PE=2 SV=1 - (B7ZAX3_HUMAN)	24.93	11.13	7	1	5	23	4.224E8	638	72.0	4.89
D3DPK5	SH3 domain binding glutamic acid-rich protein like 3, isoform CRA_a (Fragment) OS=Homo sapiens GN=SH3BGRL3 PE=4 SV=1 - (D3DPK5_HUMAN)	24.84	29.57	4	4	9	16	7.806E7	257	26.8	8.38
P00918	Carbonic anhydrase 2 OS=Homo sapiens GN=CA2 PE=1 SV=2 - (CAH2_HUMAN)	24.71	13.46	1	2	3	7	6.134E7	260	29.2	7.40
Q15911	Zinc finger homeobox protein 3 OS=Homo sapiens GN=ZFHX3 PE=1 SV=2 - (ZFHX3_HUMAN)	24.70	6.08	2	1	20	37	5.098E8	3703	404.2	6.20
Q4LE33	TNC variant protein (Fragment) OS=Homo sapiens GN=TNC variant protein PE=2 SV=1 - (Q4LE33_HUMAN)	24.70	5.11	11	1	9	36	1.548E8	2233	244.2	4.94
P26373	60S ribosomal protein L13 OS=Homo sapiens GN=RPL13 PE=1 SV=4 - (RL13_HUMAN)	24.65	32.23	4	6	10	18	1.274E8	211	24.2	11.65
A8K8P3-2	Isoform 2 of Protein SFI1 homolog OS=Homo sapiens GN=SFI1 - (SFI1_HUMAN)	24.61	12.80	11	1	19	38	4.774E8	1211	143.9	10.81
P48047	ATP synthase subunit O, mitochondrial OS=Homo sapiens GN=ATP5O PE=1 SV=1 - (ATPO_HUMAN)	24.42	23.94	4	4	4	9	5.976E7	213	23.3	9.96
B7Z3K3	cDNA FLJ57998, highly similar to Homo sapiens myo-inositol 1-phosphate synthase A1 (ISYNA1), mRNA OS=Homo sapiens PE=2 SV=1 - (B7Z3K3_HUMAN)	24.41	3.65	1	1	2	16	1.549E9	356	38.8	5.40
P21964-2	Isoform Soluble of Catechol O-methyltransferase OS=Homo sapiens GN=COMT - (COMT_HUMAN)	24.25	37.10	6	4	7	9	1.059E8	221	24.4	5.33
Q6URC4	Diaphanous 1 OS=Homo sapiens PE=2 SV=1 - (Q6URC4_HUMAN)	24.16	8.41	17	1	11	22	5.359E8	1272	141.2	5.47
Q0IJ56	ST13 protein (Fragment) OS=Homo sapiens GN=ST13 PE=2 SV=1 - (Q0IJ56_HUMAN)	24.14	33.55	11	3	10	20	2.754E8	310	35.0	5.35
D3DTX7	Collagen, type I, alpha 1, isoform CRA_a OS=Homo sapiens GN=COL1A1 PE=4 SV=1 - (D3DTX7_HUMAN)	24.08	11.07	3	2	8	15	1.484E8	885	84.7	6.24
P06703	Protein S100-A6 OS=Homo sapiens GN=S100A6 PE=1 SV=1 - (S10A6_HUMAN)	23.93	35.56	2	5	6	14	8.770E7	90	10.2	5.48
Q96IV0-3	Isoform 3 of Peptide-N(4)-(N-acetyl-beta-glucosaminyl)asparagine amidase OS=Homo sapiens GN=NGLY1 - (NGLY1_HUMAN)	23.79	11.83	4	1	8	18	3.165E8	558	63.8	8.10
A8K8U1	cDNA FLJ77762, highly similar to Homo sapiens cullin-associated and neddylation-dissociated 1 (CAND1), mRNA OS=Homo sapiens PE=2 SV=1 - (A8K8U1_HUMAN)	23.62	6.67	8	5	7	12	2.858E9	1230	136.2	5.83
Q8N879	cDNA FLJ39859 fis, clone SPLEN2015094, moderately similar to Mus musculus histone deacetylase mHDA1 mRNA OS=Homo sapiens PE=2 SV=1 - (Q8N879_HUMAN)	23.55	7.81	24	1	7	20	7.589E7	768	84.8	6.90
Q9H2F9	Coiled-coil domain-containing protein 68 OS=Homo sapiens GN=CCDC68 PE=2 SV=1 - (CCD68_HUMAN)	23.54	11.34	1	1	4	19	5.110E8	335	38.8	8.65
B4DU58	Macrophage-capping protein OS=Homo sapiens GN=CAPG PE=2 SV=1 - (B4DU58_HUMAN)	23.50	11.93	7	3	4	12	3.836E7	327	36.2	6.25
P62277	40S ribosomal protein S13 OS=Homo sapiens GN=RPS13 PE=1 SV=2 - (RS13_HUMAN)	23.50	43.05	2	5	9	16	3.999E8	151	17.2	10.54
P62750	60S ribosomal protein L23a OS=Homo sapiens GN=RPL23A PE=1 SV=1 - (RL23A_HUMAN)	23.47	40.38	5	5	10	15	2.235E8	156	17.7	10.45
E9PK47	Phosphorylase OS=Homo sapiens GN=PYGL PE=3 SV=1 - (E9PK47_HUMAN)	23.05	13.31	16	3	11	18	2.631E8	819	94.0	7.31
Q5JWF2-2	Isoform XLas-2 of Guanine nucleotide-binding protein G(s) subunit alpha isoforms XLas OS=Homo sapiens GN=GNAS - (GNAS1_HUMAN)	23.00	5.28	38	2	5	14	7.333E8	1023	109.6	5.07
Q9BRZ2	E3 ubiquitin-protein ligase TRIM56 OS=Homo sapiens GN=TRIM56 PE=1 SV=3 - (TRI56_HUMAN)	22.92	12.85	1	1	6	21	3.405E8	755	81.4	7.74
P11137-3	Isoform 3 of Microtubule-associated protein 2 OS=Homo sapiens GN=MAP2 - (MAP2_HUMAN)	22.77	6.14	3	1	12	31	4.945E8	1823	199.0	4.91
B3KVR7	Serine-arginine repressor protein (35 kDa), isoform CRA_b OS=Homo sapiens GN=SRrp35 PE=2 SV=1 - (B3KVR7_HUMAN)	22.73	25.90	2	1	6	18	4.784E8	166	19.3	12.25
B0I1P5	Kidney ankyrin repeat-containing protein 3 OS=Homo sapiens GN=Kank3 PE=2 SV=1 - (B0I1P5_HUMAN)	22.73	16.32	3	1	13	35	2.497E9	821	85.9	5.07
B5MEH6	Zinc finger RNA-binding protein OS=Homo sapiens GN=ZFR PE=4 SV=1 - (B5MEH6_HUMAN)	22.70	5.68	4	1	8	16	6.419E7	1056	115.1	9.28
Q9NT22	EMILIN-3 OS=Homo sapiens GN=EMILIN3 PE=2 SV=2 - (EMIL3_HUMAN)	22.65	12.14	1	1	7	18	3.950E8	766	82.6	7.72
Q14517	Protocadherin Fat 1 OS=Homo sapiens GN=FAT1 PE=1 SV=2 - (FAT1_HUMAN)	22.56	1.96	1	1	10	30	6.501E8	4588	506.0	5.00
Q9H9Q2-2	Isoform 2 of COP9 signalosome complex subunit 7b OS=Homo sapiens GN=COPS7B - (CSN7B_HUMAN)	22.52	28.66	6	1	3	15	1.231E8	157	18.1	7.34
Q86SG7	Lysozyme g-like protein 2 OS=Homo sapiens GN=LYG2 PE=1 SV=2 - (LYG2_HUMAN)	22.44	39.62	4	7	7	8	5.389E7	212	23.5	8.87
P80188	Neutrophil gelatinase-associated lipocalin OS=Homo sapiens GN=LCN2 PE=1 SV=2 - (NGAL_HUMAN)	22.44	41.41	4	7	8	11	9.965E7	198	22.6	8.91
Q12955	Ankyrin-3 OS=Homo sapiens GN=ANK3 PE=1 SV=3 - (ANK3_HUMAN)	22.38	4.98	20	1	22	41	7.466E8	4377	480.1	6.49
Q9UI15	Transgelin-3 OS=Homo sapiens GN=TAGLN3 PE=1 SV=2 - (TAGL3_HUMAN)	22.35	31.16	4	1	5	14	1.809E8	199	22.5	7.33
Q6P1N0-2	Isoform 2 of Coiled-coil and C2 domain-containing protein 1A OS=Homo sapiens GN=CC2D1A - (C2D1A_HUMAN)	22.35	9.58	4	1	9	15	5.936E8	950	103.9	8.09
E7EWF1	60S ribosomal protein L4 OS=Homo sapiens GN=RPL4 PE=4 SV=1 - (E7EWF1_HUMAN)	22.33	26.60	11	4	10	23	2.305E8	406	45.5	11.03
Q53TK3	Putative uncharacterized protein PRO2015 OS=Homo sapiens GN=PRO2015 PE=4 SV=1 - (Q53TK3_HUMAN)	22.31	17.02	2	1	2	14	1.459E9	94	10.8	11.59
Q9ULZ3-3	Isoform 3 of Apoptosis-associated speck-like protein containing a CARD OS=Homo sapiens GN=PYCARD - (ASC_HUMAN)	22.28	45.93	5	3	5	9	3.204E7	135	15.0	7.44
P18621	60S ribosomal protein L17 OS=Homo sapiens GN=RPL17 PE=1 SV=3 - (RL17_HUMAN)	22.27	36.96	4	5	8	13	7.127E7	184	21.4	10.17
Q9NYJ1	Coiled-coil-helix-coiled-coil-helix domain-containing protein 8 OS=Homo sapiens GN=CHCHD8 PE=1 SV=2 - (CHCH8_HUMAN)	22.22	31.03	2	1	3	15	2.423E8	87	10.1	6.04
B4E2U0	6-phosphogluconate dehydrogenase, decarboxylating OS=Homo sapiens PE=2 SV=1 - (B4E2U0_HUMAN)	22.22	19.31	8	2	5	12	5.506E8	461	50.8	8.18
A2A3R5	40S ribosomal protein S6 OS=Homo sapiens GN=RPS6 PE=3 SV=1 - (A2A3R5_HUMAN)	22.15	30.73	4	4	8	17	1.564E8	218	25.0	11.14
P10599	Thioredoxin OS=Homo sapiens GN=TXN PE=1 SV=3 - (THIO_HUMAN)	22.09	51.43	3	4	5	13	1.349E8	105	11.7	4.92
B4DLJ7	cDNA FLJ59334, highly similar to Transmembrane glycoprotein NMB OS=Homo sapiens PE=2 SV=1 - (B4DLJ7_HUMAN)	22.00	19.65	11	2	8	15	4.972E8	514	57.2	6.77
Q6P0Q8	Microtubule-associated serine/threonine-protein kinase 2 OS=Homo sapiens GN=MAST2 PE=1 SV=2 - (MAST2_HUMAN)	21.84	7.23	5	2	10	29	2.763E8	1798	196.3	8.16
Q96MK8	cDNA FLJ32212 fis, clone PLACE6003399, weakly similar to SPIDROIN 1 OS=Homo sapiens PE=1 SV=1 - (Q96MK8_HUMAN)	21.81	14.06	1	1	3	11	2.340E8	256	26.3	11.81
C3UMV2	EWSR1/NFATC2 fusion protein OS=Homo sapiens PE=2 SV=1 - (C3UMV2_HUMAN)	21.55	8.15	11	1	7	31	2.554E8	859	93.2	8.19
F5H3P5	Actin-related protein 3 OS=Homo sapiens GN=ACTR3 PE=3 SV=1 - (F5H3P5_HUMAN)	21.35	14.33	15	2	5	11	1.038E8	356	40.7	5.96
Q8N264	Rho GTPase-activating protein 24 OS=Homo sapiens GN=ARHGAP24 PE=1 SV=2 - (RHG24_HUMAN)	21.31	4.01	6	1	5	17	4.124E8	748	84.2	6.67
Q92833	Protein Jumonji OS=Homo sapiens GN=JARID2 PE=1 SV=2 - (JARD2_HUMAN)	21.30	12.28	2	1	14	32	2.072E8	1246	138.6	9.38
P30041	Peroxiredoxin-6 OS=Homo sapiens GN=PRDX6 PE=1 SV=3 - (PRDX6_HUMAN)	21.29	31.25	3	4	6	10	1.395E8	224	25.0	6.38
E9PN51	NADH dehydrogenase (ubiquinone) iron-sulfur protein 8, mitochondrial (Fragment) OS=Homo sapiens GN=NDUFS8 PE=4 SV=1 - (E9PN51_HUMAN)	21.27	49.09	7	1	6	14	1.751E9	110	12.4	9.98
O75367-3	Isoform 3 of Core histone macro-H2A.1 OS=Homo sapiens GN=H2AFY - (H2AY_HUMAN)	21.18	15.90	9	3	5	13	1.231E8	371	39.5	9.77
Q5VUA4	Zinc finger protein 318 OS=Homo sapiens GN=ZNF318 PE=1 SV=2 - (ZN318_HUMAN)	21.17	9.43	1	1	21	30	4.092E8	2279	251.0	7.20
B4DL49	cDNA FLJ58073, moderately similar to Cathepsin B (EC 3.4.22.1) OS=Homo sapiens PE=2 SV=1 - (B4DL49_HUMAN)	21.12	26.74	20	4	6	10	2.479E7	273	30.7	6.62
B4E363	Phenylalanine--tRNA ligase alpha subunit OS=Homo sapiens GN=FARSA PE=2 SV=1 - (B4E363_HUMAN)	20.94	13.00	2	1	7	15	4.005E8	477	54.1	7.80
P51149	Ras-related protein Rab-7a OS=Homo sapiens GN=RAB7A PE=1 SV=1 - (RAB7A_HUMAN)	20.84	52.17	8	5	8	9	2.478E8	207	23.5	6.70
P49748	Very long-chain specific acyl-CoA dehydrogenase, mitochondrial OS=Homo sapiens GN=ACADVL PE=1 SV=1 - (ACADV_HUMAN)	20.79	18.17	10	6	9	13	9.754E7	655	70.3	8.75
E7EU78	NF-kappa-B essential modulator OS=Homo sapiens GN=IKBKG PE=4 SV=1 - (E7EU78_HUMAN)	20.77	15.05	11	1	6	21	9.424E8	412	47.4	5.53
Q9Y333	U6 snRNA-associated Sm-like protein LSm2 OS=Homo sapiens GN=LSM2 PE=1 SV=1 - (LSM2_HUMAN)	20.77	28.42	1	1	4	14	4.480E7	95	10.8	6.52
B4DGP8	cDNA FLJ55574, highly similar to Calnexin OS=Homo sapiens PE=2 SV=1 - (B4DGP8_HUMAN)	20.77	11.16	10	2	6	17	2.918E8	627	71.5	4.70
P29373	Cellular retinoic acid-binding protein 2 OS=Homo sapiens GN=CRABP2 PE=1 SV=2 - (RABP2_HUMAN)	20.52	55.80	2	6	7	15	1.640E8	138	15.7	5.40
Q92878-3	Isoform 3 of DNA repair protein RAD50 OS=Homo sapiens GN=RAD50 - (RAD50_HUMAN)	20.50	22.42	3	1	26	39	7.584E8	1173	138.3	6.55
P61254	60S ribosomal protein L26 OS=Homo sapiens GN=RPL26 PE=1 SV=1 - (RL26_HUMAN)	20.31	55.86	9	8	11	12	8.924E7	145	17.2	10.55
P45880-2	Isoform 2 of Voltage-dependent anion-selective channel protein 2 OS=Homo sapiens GN=VDAC2 - (VDAC2_HUMAN)	20.31	27.56	7	5	8	16	1.388E8	283	30.4	7.20
P13928	Annexin A8 OS=Homo sapiens GN=ANXA8 PE=1 SV=3 - (ANXA8_HUMAN)	20.26	25.38	15	5	8	12	2.754E8	327	36.9	5.78
Q5VTQ0-5	Isoform 5 of Tetratricopeptide repeat protein 39B OS=Homo sapiens GN=TTC39B - (TT39B_HUMAN)	20.25	8.12	7	1	4	12	1.468E8	517	59.4	7.58
H3BQH0	Calponin-2 (Fragment) OS=Homo sapiens GN=CNN2 PE=4 SV=1 - (H3BQH0_HUMAN)	20.18	29.25	8	1	3	7	1.176E8	212	23.1	8.59
H0YDT6	Eukaryotic translation initiation factor 3 subunit F (Fragment) OS=Homo sapiens GN=EIF3F PE=4 SV=1 - (H0YDT6_HUMAN)	20.15	19.00	5	1	1	5	3.661E6	100	10.8	5.81
Q13515	Phakinin OS=Homo sapiens GN=BFSP2 PE=1 SV=1 - (BFSP2_HUMAN)	20.14	25.30	1	1	10	20	1.974E8	415	45.9	5.55
Q92786	Prospero homeobox protein 1 OS=Homo sapiens GN=PROX1 PE=1 SV=2 - (PROX1_HUMAN)	20.10	7.19	2	1	5	12	8.639E7	737	83.2	7.18
P30084	Enoyl-CoA hydratase, mitochondrial OS=Homo sapiens GN=ECHS1 PE=1 SV=4 - (ECHM_HUMAN)	20.00	12.07	1	2	2	4	2.057E7	290	31.4	8.07
Q92788	Rad GTPase OS=Homo sapiens PE=2 SV=1 - (Q92788_HUMAN)	19.94	15.58	3	1	4	53	1.009E8	308	33.2	8.68
P12236	ADP/ATP translocase 3 OS=Homo sapiens GN=SLC25A6 PE=1 SV=4 - (ADT3_HUMAN)	19.91	27.85	9	4	9	20	2.234E8	298	32.8	9.74
Q13459	Unconventional myosin-IXb OS=Homo sapiens GN=MYO9B PE=1 SV=3 - (MYO9B_HUMAN)	19.91	5.61	4	1	11	22	1.073E8	2157	243.2	8.75
P07108-4	Isoform 4 of Acyl-CoA-binding protein OS=Homo sapiens GN=DBI - (ACBP_HUMAN)	19.90	52.71	8	5	7	14	1.408E8	129	14.4	9.42
D6R922	WD repeat-containing protein 36 OS=Homo sapiens GN=WDR36 PE=4 SV=1 - (D6R922_HUMAN)	19.89	11.02	3	1	5	34	1.147E9	581	64.3	9.29
B4DU23	cDNA FLJ53488, highly similar to Homo sapiens leucine-rich repeats and calponin homology (CH) domain containing 3 (LRCH3), mRNA OS=Homo sapiens PE=2 SV=1 - (B4DU23_HUMAN)	19.89	10.75	11	1	8	16	7.709E8	623	68.5	6.74
B3GQS7	Mitochondrial heat shock 60kD protein 1 variant 1 OS=Homo sapiens GN=HSPD1 PE=2 SV=1 - (B3GQS7_HUMAN)	19.89	16.70	16	4	7	8	7.565E7	569	60.6	6.04
Q4ZFX3	Putative uncharacterized protein VSNL1 (Fragment) OS=Homo sapiens GN=VSNL1 PE=2 SV=1 - (Q4ZFX3_HUMAN)	19.88	16.79	4	2	2	6	2.539E7	137	15.7	4.98
B7Z6M1	Plastin-3 OS=Homo sapiens GN=PLS3 PE=2 SV=1 - (B7Z6M1_HUMAN)	19.86	24.96	14	4	11	18	6.985E7	585	65.6	6.30
B3KTS5	cDNA FLJ38670 fis, clone HSYRA2000190, highly similar to Voltage-dependent anion-selective channel protein 1 OS=Homo sapiens PE=2 SV=1 - (B3KTS5_HUMAN)	19.73	32.51	7	3	9	17	1.048E8	283	30.7	7.90
B3KUT2	cDNA FLJ40562 fis, clone THYMU2003981, moderately similar to Rattus norvegicus macrophage expressed gene 1 (Mpeg1), mRNA OS=Homo sapiens PE=2 SV=1 - (B3KUT2_HUMAN)	19.72	5.45	2	1	4	15	7.564E8	716	78.5	7.65
P68402	Platelet-activating factor acetylhydrolase IB subunit beta OS=Homo sapiens GN=PAFAH1B2 PE=1 SV=1 - (PA1B2_HUMAN)	19.69	8.30	1	1	2	12	8.300E7	229	25.6	5.92
Q2TAC6	Kinesin-like protein KIF19 OS=Homo sapiens GN=KIF19 PE=2 SV=2 - (KIF19_HUMAN)	19.69	12.73	4	1	15	28	1.031E9	998	111.3	8.69
Q8NDH2	Coiled-coil domain-containing protein 168 OS=Homo sapiens GN=CCDC168 PE=2 SV=2 - (CC168_HUMAN)	19.66	8.69	1	1	20	37	2.885E8	2452	277.8	9.31
B3KVD6	cDNA FLJ16433 fis, clone BRACE3013936, highly similar to Cell death activator CIDE-B OS=Homo sapiens PE=2 SV=1 - (B3KVD6_HUMAN)	19.61	3.20	2	1	1	24	6.407E8	219	24.8	9.03
P05387	60S acidic ribosomal protein P2 OS=Homo sapiens GN=RPLP2 PE=1 SV=1 - (RLA2_HUMAN)	19.60	48.70	2	3	5	7	5.253E7	115	11.7	4.54
A5YM50	RAB11B protein OS=Homo sapiens GN=RAB11B PE=2 SV=1 - (A5YM50_HUMAN)	19.58	43.12	12	5	9	13	6.068E7	218	24.5	6.24
P62318	Small nuclear ribonucleoprotein Sm D3 OS=Homo sapiens GN=SNRPD3 PE=1 SV=1 - (SMD3_HUMAN)	19.49	30.95	2	1	4	12	1.075E8	126	13.9	10.32
D3DPC4	Putative uncharacterized protein FLJ21901 OS=Homo sapiens GN=FLJ21901 PE=4 SV=1 - (D3DPC4_HUMAN)	19.46	4.37	2	1	4	11	4.059E8	824	94.7	7.93
Q13228	Selenium-binding protein 1 OS=Homo sapiens GN=SELENBP1 PE=1 SV=2 - (SBP1_HUMAN)	19.37	12.71	10	3	6	14	8.790E7	472	52.4	6.37
Q9Y6R9	Coiled-coil domain-containing protein 61 OS=Homo sapiens GN=CCDC61 PE=1 SV=2 - (CCD61_HUMAN)	19.32	27.22	1	1	11	23	4.221E8	474	53.2	9.95
B7Z382	Cytosolic purine 5'-nucleotidase OS=Homo sapiens GN=NT5C2 PE=2 SV=1 - (B7Z382_HUMAN)	19.28	5.64	7	1	3	33	9.200E8	532	61.4	5.74
C9JX84	Inter-alpha-trypsin inhibitor heavy chain H3 OS=Homo sapiens GN=ITIH3 PE=4 SV=2 - (C9JX84_HUMAN)	19.26	7.46	7	1	6	14	6.513E8	885	99.1	5.91
P62263	40S ribosomal protein S14 OS=Homo sapiens GN=RPS14 PE=1 SV=3 - (RS14_HUMAN)	19.23	25.83	4	6	6	8	1.116E8	151	16.3	10.05
O94933	SLIT and NTRK-like protein 3 OS=Homo sapiens GN=SLITRK3 PE=2 SV=2 - (SLIK3_HUMAN)	18.96	6.24	1	1	4	21	5.901E7	977	108.9	7.37
Q9NXR1-2	Isoform 2 of Nuclear distribution protein nudE homolog 1 OS=Homo sapiens GN=NDE1 - (NDE1_HUMAN)	18.95	25.67	11	1	7	11	9.129E7	335	37.7	5.15
E9PGD1	Homocysteine-responsive endoplasmic reticulum-resident ubiquitin-like domain member 1 protein OS=Homo sapiens GN=HERPUD1 PE=4 SV=1 - (E9PGD1_HUMAN)	18.89	6.28	15	1	2	10	5.061E8	366	40.8	4.91
B4DTU7	cDNA FLJ52076, highly similar to Homo sapiens aldehyde dehydrogenase 1 family, member L2 (ALDH1L2), mRNA OS=Homo sapiens PE=2 SV=1 - (B4DTU7_HUMAN)	18.88	13.00	3	1	10	34	2.559E9	923	101.7	6.52
Q6FIG4	RAB1B protein OS=Homo sapiens GN=RAB1B PE=2 SV=1 - (Q6FIG4_HUMAN)	18.87	21.89	89	1	3	12	3.740E8	201	22.2	5.73
F5GY37	Prohibitin-2 OS=Homo sapiens GN=PHB2 PE=4 SV=1 - (F5GY37_HUMAN)	18.84	33.71	9	4	8	8	1.029E8	267	29.7	9.88
Q53H10	Postreplication repair protein hRAD18p variant (Fragment) OS=Homo sapiens PE=2 SV=1 - (Q53H10_HUMAN)	18.81	17.37	3	1	7	16	8.443E8	495	56.2	7.59
O14981	TATA-binding protein-associated factor 172 OS=Homo sapiens GN=BTAF1 PE=1 SV=2 - (BTAF1_HUMAN)	18.66	9.57	3	1	14	49	3.637E8	1849	206.8	6.52
P62266	40S ribosomal protein S23 OS=Homo sapiens GN=RPS23 PE=1 SV=3 - (RS23_HUMAN)	18.60	40.56	2	3	4	6	7.748E7	143	15.8	10.49
H0Y7A7	Calmodulin (Fragment) OS=Homo sapiens GN=CALM2 PE=4 SV=1 - (H0Y7A7_HUMAN)	18.54	28.34	8	2	3	9	7.057E7	187	20.7	4.36
P63167	Dynein light chain 1, cytoplasmic OS=Homo sapiens GN=DYNLL1 PE=1 SV=1 - (DYL1_HUMAN)	18.52	52.81	5	6	6	10	5.641E7	89	10.4	7.40
P27635	60S ribosomal protein L10 OS=Homo sapiens GN=RPL10 PE=1 SV=4 - (RL10_HUMAN)	18.45	26.17	8	5	6	15	7.267E7	214	24.6	10.08
B3KUD4	cDNA FLJ39594 fis, clone SKNSH2001875, highly similar to Ribonuclease H1 (EC 3.1.26.4) OS=Homo sapiens PE=2 SV=1 - (B3KUD4_HUMAN)	18.41	7.10	2	1	1	10	1.722E8	169	18.8	8.60
O60716-8	Isoform 1 of Catenin delta-1 OS=Homo sapiens GN=CTNND1 - (CTND1_HUMAN)	18.39	17.96	38	5	13	24	2.585E8	913	101.8	6.87
F8WD26	LIM domain only protein 7 OS=Homo sapiens GN=LMO7 PE=4 SV=1 - (F8WD26_HUMAN)	18.27	11.65	25	1	17	45	1.260E9	1614	185.0	7.97
P62854	40S ribosomal protein S26 OS=Homo sapiens GN=RPS26 PE=1 SV=3 - (RS26_HUMAN)	18.26	37.39	8	4	4	7	1.420E8	115	13.0	11.00
P11940-2	Isoform 2 of Polyadenylate-binding protein 1 OS=Homo sapiens GN=PABPC1 - (PABP1_HUMAN)	18.23	23.03	31	3	12	19	5.221E8	547	61.1	9.07
P28074	Proteasome subunit beta type-5 OS=Homo sapiens GN=PSMB5 PE=1 SV=3 - (PSB5_HUMAN)	18.16	21.29	4	5	6	7	2.193E7	263	28.5	6.92
Q92547	DNA topoisomerase 2-binding protein 1 OS=Homo sapiens GN=TOPBP1 PE=1 SV=3 - (TOPB1_HUMAN)	18.05	10.51	4	1	13	24	7.733E8	1522	170.6	6.96
Q04760-2	Isoform 2 of Lactoylglutathione lyase OS=Homo sapiens GN=GLO1 - (LGUL_HUMAN)	18.05	38.46	2	5	6	9	8.804E7	169	19.0	6.05
Q86US8	Telomerase-binding protein EST1A OS=Homo sapiens GN=SMG6 PE=1 SV=2 - (EST1A_HUMAN)	18.03	10.43	1	1	17	30	3.533E8	1419	160.4	7.05
B4DRH6	cDNA FLJ54509, highly similar to Trifunctional enzyme subunit alpha, mitochondrial OS=Homo sapiens PE=2 SV=1 - (B4DRH6_HUMAN)	18.00	11.02	6	3	7	9	6.656E7	717	78.3	8.92
Q14240-2	Isoform 2 of Eukaryotic initiation factor 4A-II OS=Homo sapiens GN=EIF4A2 - (IF4A2_HUMAN)	17.83	10.29	14	2	4	12	6.851E7	408	46.5	5.48
P54136	Arginine--tRNA ligase, cytoplasmic OS=Homo sapiens GN=RARS PE=1 SV=2 - (SYRC_HUMAN)	17.80	24.70	4	1	12	17	2.169E9	660	75.3	6.68
E7EQE3	Telomerase protein component 1 OS=Homo sapiens GN=TEP1 PE=4 SV=1 - (E7EQE3_HUMAN)	17.78	8.36	6	1	19	39	1.351E9	2619	289.3	8.10
C9JBW3	Protein sidekick-2 OS=Homo sapiens GN=SDK2 PE=4 SV=1 - (C9JBW3_HUMAN)	17.73	8.08	8	1	13	21	1.534E9	2216	244.2	7.12
C1PHA2	Tyrosine-protein kinase receptor OS=Homo sapiens GN=KIF5B-ALK PE=2 SV=1 - (C1PHA2_HUMAN)	17.66	8.29	5	1	14	24	2.742E8	1483	167.8	6.64
Q9NY30	Protein BTG4 OS=Homo sapiens GN=BTG4 PE=2 SV=1 - (BTG4_HUMAN)	17.65	12.56	1	1	3	16	3.330E8	223	26.0	8.57
Q96AG4	Leucine-rich repeat-containing protein 59 OS=Homo sapiens GN=LRRC59 PE=1 SV=1 - (LRC59_HUMAN)	17.62	27.04	2	4	11	19	5.783E7	307	34.9	9.57
E7EPK6	40S ribosomal protein S24 OS=Homo sapiens GN=RPS24 PE=3 SV=1 - (E7EPK6_HUMAN)	17.55	18.69	5	2	5	10	7.071E7	289	32.4	10.15
B7Z8H0	cDNA FLJ61742, highly similar to Origin recognition complex subunit 1 OS=Homo sapiens PE=2 SV=1 - (B7Z8H0_HUMAN)	17.52	9.81	4	1	8	14	4.857E8	856	96.7	9.13
O15173	Membrane-associated progesterone receptor component 2 OS=Homo sapiens GN=PGRMC2 PE=1 SV=1 - (PGRC2_HUMAN)	17.48	4.48	1	1	2	9	8.316E8	223	23.8	4.88
Q9UNM1	Chaperonin 10-related protein (Fragment) OS=Homo sapiens GN=EPFP1 PE=2 SV=1 - (Q9UNM1_HUMAN)	17.48	40.21	4	3	5	8	2.475E7	97	10.3	9.00
B3KTT6	cDNA FLJ38699 fis, clone KIDNE2002168, highly similar to Short chain 3-hydroxyacyl-CoA dehydrogenase, mitochondrial (EC 1.1.1.35) OS=Homo sapiens PE=2 SV=1 - (B3KTT6_HUMAN)	17.42	22.01	5	2	4	7	4.180E7	318	35.2	7.21
Q5TCK5	Progestin and adipoQ receptor family member 6 (Fragment) OS=Homo sapiens GN=PAQR6 PE=2 SV=1 - (Q5TCK5_HUMAN)	17.37	8.26	1	1	4	21	1.634E8	363	38.2	10.42
Q9HD67	Unconventionnal myosin-X OS=Homo sapiens GN=MYO10 PE=1 SV=3 - (MYO10_HUMAN)	17.31	7.48	4	1	17	25	5.029E8	2058	237.2	6.21
Q5VWI1	Transcription elongation regulator 1-like protein OS=Homo sapiens GN=TCERG1L PE=2 SV=2 - (TCRGL_HUMAN)	17.19	6.48	1	1	6	24	7.134E8	586	65.6	9.83
P14406	Cytochrome c oxidase subunit 7A2, mitochondrial OS=Homo sapiens GN=COX7A2 PE=1 SV=1 - (CX7A2_HUMAN)	17.18	43.37	6	2	3	6	1.369E7	83	9.4	9.76
B2R6V2	cDNA, FLJ93132, highly similar to Homo sapiens CDC7 cell division cycle 7 (S. cerevisiae) (CDC7), mRNA OS=Homo sapiens PE=2 SV=1 - (B2R6V2_HUMAN)	17.16	5.57	3	1	3	12	2.592E8	574	63.8	8.81
Q9BZJ0	Crooked neck-like protein 1 OS=Homo sapiens GN=CRNKL1 PE=1 SV=4 - (CRNL1_HUMAN)	17.12	2.95	1	1	3	23	4.311E9	848	100.4	8.00
Q9BQS8	FYVE and coiled-coil domain-containing protein 1 OS=Homo sapiens GN=FYCO1 PE=1 SV=3 - (FYCO1_HUMAN)	16.86	12.11	2	1	14	19	3.946E8	1478	166.9	4.92
Q5JS13-2	Isoform 2 of Ras-specific guanine nucleotide-releasing factor RalGPS1 OS=Homo sapiens GN=RALGPS1 - (RGPS1_HUMAN)	16.86	9.07	6	1	5	27	9.268E7	529	58.9	9.26
Q9P2E9-2	Isoform 1 of Ribosome-binding protein 1 OS=Homo sapiens GN=RRBP1 - (RRBP1_HUMAN)	16.85	11.66	8	1	17	26	1.628E8	1407	152.1	8.66
B3W5Y6	Squamous cell carcinoma antigen-1 isoform SCCA-PD OS=Homo sapiens GN=SERPINB3 PE=2 SV=1 - (B3W5Y6_HUMAN)	16.85	20.00	16	4	6	10	2.616E7	390	44.6	6.81
Q9UIS4	Small nuclear ribonucleoprotein-associated protein OS=Homo sapiens GN=SNRPB PE=2 SV=1 - (Q9UIS4_HUMAN)	16.84	25.10	12	2	6	18	2.661E8	243	27.2	9.41
P30044	Peroxiredoxin-5, mitochondrial OS=Homo sapiens GN=PRDX5 PE=1 SV=4 - (PRDX5_HUMAN)	16.83	48.60	6	6	7	8	2.828E8	214	22.1	8.70
Q04637-5	Isoform D of Eukaryotic translation initiation factor 4 gamma 1 OS=Homo sapiens GN=EIF4G1 - (IF4G1_HUMAN)	16.77	12.33	27	1	18	23	2.599E8	1435	158.4	5.21
P0CW22	40S ribosomal protein S17-like OS=Homo sapiens GN=RPS17L PE=3 SV=1 - (RS17L_HUMAN)	16.76	65.93	5	3	6	8	2.688E8	135	15.5	9.85
D6RAN4	60S ribosomal protein L9 (Fragment) OS=Homo sapiens GN=RPL9 PE=4 SV=1 - (D6RAN4_HUMAN)	16.75	28.57	8	4	6	8	7.712E7	182	20.9	10.20
Q96L93-6	Isoform 5 of Kinesin-like protein KIF16B OS=Homo sapiens GN=KIF16B - (KI16B_HUMAN)	16.65	13.17	6	2	16	22	1.848E9	1276	147.3	6.09
Q8TB01	Similar to cytoskeleton-associated protein 4 (Fragment) OS=Homo sapiens PE=2 SV=1 - (Q8TB01_HUMAN)	16.62	16.96	4	1	7	13	7.145E7	560	62.0	5.68
P62280	40S ribosomal protein S11 OS=Homo sapiens GN=RPS11 PE=1 SV=3 - (RS11_HUMAN)	16.60	41.14	1	6	8	13	1.398E8	158	18.4	10.30
Q9Y3D6	Mitochondrial fission 1 protein OS=Homo sapiens GN=FIS1 PE=1 SV=2 - (FIS1_HUMAN)	16.50	23.68	1	2	3	7	1.728E7	152	16.9	8.79
Q02543	60S ribosomal protein L18a OS=Homo sapiens GN=RPL18A PE=1 SV=2 - (RL18A_HUMAN)	16.30	34.09	8	6	8	10	6.964E7	176	20.7	10.71
B4E1G6	Galactokinase 1 OS=Homo sapiens GN=GALK1 PE=2 SV=1 - (B4E1G6_HUMAN)	16.30	15.88	6	1	7	15	2.672E8	422	45.3	6.68
A8K7B1	cDNA FLJ78384, highly similar to Homo sapiens RUN and FYVE domain containing 1 (RUFY1), mRNA OS=Homo sapiens PE=2 SV=1 - (A8K7B1_HUMAN)	16.18	19.67	6	1	11	25	4.296E8	600	69.0	5.91
D3DNE5	DAZ interacting protein 1-like, isoform CRA_c OS=Homo sapiens GN=DZIP1L PE=4 SV=1 - (D3DNE5_HUMAN)	16.12	12.13	3	1	9	21	1.737E8	767	86.8	7.05
Q12915	Ibd1 protein (Fragment) OS=Homo sapiens GN=Ibd1 PE=2 SV=1 - (Q12915_HUMAN)	16.11	9.31	1	1	2	8	3.319E7	204	22.1	8.46
P62424	60S ribosomal protein L7a OS=Homo sapiens GN=RPL7A PE=1 SV=2 - (RL7A_HUMAN)	16.04	31.58	4	3	8	9	1.170E8	266	30.0	10.61
Q99497	Protein DJ-1 OS=Homo sapiens GN=PARK7 PE=1 SV=2 - (PARK7_HUMAN)	16.01	55.56	1	5	8	12	6.219E7	189	19.9	6.79
E9PI98	Layilin (Fragment) OS=Homo sapiens GN=LAYN PE=4 SV=1 - (E9PI98_HUMAN)	15.96	14.06	1	1	1	9	2.294E8	64	7.4	8.00
P49770	Translation initiation factor eIF-2B subunit beta OS=Homo sapiens GN=EIF2B2 PE=1 SV=3 - (EI2BB_HUMAN)	15.94	10.26	3	1	4	13	1.245E8	351	39.0	6.16
E9PBV3	Suprabasin OS=Homo sapiens GN=SBSN PE=4 SV=1 - (E9PBV3_HUMAN)	15.94	19.49	1	2	4	9	4.290E7	590	60.5	7.01
P09429	High mobility group protein B1 OS=Homo sapiens GN=HMGB1 PE=1 SV=3 - (HMGB1_HUMAN)	15.91	22.79	13	4	6	13	7.325E7	215	24.9	5.74
B3KTC3	cDNA FLJ38059 fis, clone CTONG2014946, highly similar to Gigaxonin OS=Homo sapiens PE=2 SV=1 - (B3KTC3_HUMAN)	15.90	7.54	2	1	4	11	8.321E7	597	67.6	5.94
Q5T2S8	Armadillo repeat-containing protein 4 OS=Homo sapiens GN=ARMC4 PE=2 SV=1 - (ARMC4_HUMAN)	15.87	9.00	5	1	12	19	2.769E8	1044	115.6	7.77
B4DE02	Annexin OS=Homo sapiens PE=2 SV=1 - (B4DE02_HUMAN)	15.77	32.34	9	6	8	8	9.716E7	303	34.4	5.87
P62244	40S ribosomal protein S15a OS=Homo sapiens GN=RPS15A PE=1 SV=2 - (RS15A_HUMAN)	15.76	71.54	9	7	10	13	2.080E8	130	14.8	10.13
P37837	Transaldolase OS=Homo sapiens GN=TALDO1 PE=1 SV=2 - (TALDO_HUMAN)	15.67	28.19	3	4	8	13	1.085E8	337	37.5	6.81
B4DUI2	cDNA FLJ61415, highly similar to Protein kinase C and casein kinase substratein neurons protein 3 OS=Homo sapiens PE=2 SV=1 - (B4DUI2_HUMAN)	15.67	6.65	4	1	3	12	2.964E7	421	48.2	6.39
Q53T70	Putative uncharacterized protein RAB10 (Fragment) OS=Homo sapiens GN=RAB10 PE=2 SV=1 - (Q53T70_HUMAN)	15.65	27.39	82	1	4	10	1.168E8	157	17.8	8.25
Q8N3C0	Activating signal cointegrator 1 complex subunit 3 OS=Homo sapiens GN=ASCC3 PE=1 SV=3 - (ASCC3_HUMAN)	15.65	7.58	1	1	15	41	1.053E9	2202	251.3	7.09
P61088	Ubiquitin-conjugating enzyme E2 N OS=Homo sapiens GN=UBE2N PE=1 SV=1 - (UBE2N_HUMAN)	15.64	43.42	6	4	7	9	1.612E8	152	17.1	6.57
D6RAD4	Cyclin-dependent kinase 7 OS=Homo sapiens GN=CDK7 PE=4 SV=1 - (D6RAD4_HUMAN)	15.62	7.12	3	1	2	6	8.400E7	309	34.6	8.28
H0YJD6	Dynein light chain 1, axonemal (Fragment) OS=Homo sapiens GN=DNAL1 PE=4 SV=1 - (H0YJD6_HUMAN)	15.48	31.34	1	1	2	10	1.817E8	67	7.6	8.97
P49207	60S ribosomal protein L34 OS=Homo sapiens GN=RPL34 PE=1 SV=3 - (RL34_HUMAN)	15.47	45.30	2	3	7	10	7.012E7	117	13.3	11.47
Q00610-2	Isoform 2 of Clathrin heavy chain 1 OS=Homo sapiens GN=CLTC - (CLH1_HUMAN)	15.46	6.35	4	4	9	24	3.044E8	1639	187.8	5.69
Q8WUF5	RelA-associated inhibitor OS=Homo sapiens GN=PPP1R13L PE=1 SV=4 - (IASPP_HUMAN)	15.43	11.59	2	1	8	13	6.262E7	828	89.0	6.81
Q8IVG5	Sterile alpha motif domain-containing protein 9-like OS=Homo sapiens GN=SAMD9L PE=1 SV=2 - (SAM9L_HUMAN)	15.42	4.99	3	1	7	12	2.106E8	1584	184.4	8.02
A0PK11	Clarin-2 OS=Homo sapiens GN=CLRN2 PE=2 SV=1 - (CLRN2_HUMAN)	15.39	3.02	1	1	1	20	3.412E8	232	25.4	6.99
Q9BYR6	Keratin-associated protein 3-3 OS=Homo sapiens GN=KRTAP3-3 PE=1 SV=1 - (KRA33_HUMAN)	15.34	17.35	1	1	1	4	3.790E7	98	10.4	5.69
Q53SY7	Putative uncharacterized protein CAD (Fragment) OS=Homo sapiens GN=CAD PE=2 SV=1 - (Q53SY7_HUMAN)	15.32	4.51	3	1	7	17	2.495E8	2151	235.0	6.60
Q6NZ52	Ribosomal protein L27a OS=Homo sapiens GN=RPL27A PE=2 SV=1 - (Q6NZ52_HUMAN)	15.28	50.00	6	2	8	11	2.613E8	148	16.5	11.00
P16144-3	Isoform Beta-4B of Integrin beta-4 OS=Homo sapiens GN=ITGB4 - (ITB4_HUMAN)	15.28	9.36	8	1	17	21	8.809E8	1805	200.6	6.30
O60447	Ecotropic viral integration site 5 protein homolog OS=Homo sapiens GN=EVI5 PE=1 SV=3 - (EVI5_HUMAN)	15.22	15.56	4	2	10	23	5.503E8	810	92.9	6.10
Q2TU64	PIG48 OS=Homo sapiens PE=2 SV=1 - (Q2TU64_HUMAN)	15.15	18.17	13	4	12	23	1.572E8	545	60.5	6.35
Q14126	Desmoglein-2 OS=Homo sapiens GN=DSG2 PE=1 SV=2 - (DSG2_HUMAN)	15.09	6.80	1	4	6	6	1.547E8	1118	122.2	5.24
B7ZLX0	ITGAV protein OS=Homo sapiens GN=ITGAV PE=2 SV=1 - (B7ZLX0_HUMAN)	15.01	8.50	7	2	6	11	1.835E8	1012	112.2	5.94
D6R9P3	Heterogeneous nuclear ribonucleoprotein A/B OS=Homo sapiens GN=HNRNPAB PE=4 SV=1 - (D6R9P3_HUMAN)	15.00	24.64	9	1	6	14	3.461E8	280	30.3	7.91
F5H2P0	Tetraspanin-11 OS=Homo sapiens GN=TSPAN11 PE=4 SV=1 - (F5H2P0_HUMAN)	15.00	17.03	5	1	4	30	4.630E8	182	20.3	7.78
A7BKA4	ATP-binding cassette protein OS=Homo sapiens GN=ABCB5 PE=2 SV=1 - (A7BKA4_HUMAN)	14.88	5.97	5	1	8	13	1.430E8	1257	138.6	7.77
Q0VGD6	HNRPR protein (Fragment) OS=Homo sapiens GN=HNRPR PE=2 SV=1 - (Q0VGD6_HUMAN)	14.85	14.83	10	1	9	14	7.010E7	607	67.8	8.91
Q9P241-2	Isoform 2 of Probable phospholipid-transporting ATPase VD OS=Homo sapiens GN=ATP10D - (AT10D_HUMAN)	14.75	6.90	4	1	9	17	2.462E8	1231	138.8	7.15
B4DH02	cDNA FLJ50510, highly similar to Heat shock 70 kDa protein 4 OS=Homo sapiens PE=2 SV=1 - (B4DH02_HUMAN)	14.72	13.93	21	2	9	13	1.107E8	840	94.3	5.19
Q92887	Canalicular multispecific organic anion transporter 1 OS=Homo sapiens GN=ABCC2 PE=1 SV=3 - (MRP2_HUMAN)	14.67	5.05	1	1	9	14	2.202E8	1545	174.1	8.32
B2R5M8	Isocitrate dehydrogenase (NADP) OS=Homo sapiens PE=2 SV=1 - (B2R5M8_HUMAN)	14.66	18.36	10	3	6	7	5.730E7	414	46.6	7.01
Q9H7E2-3	Isoform 3 of Tudor domain-containing protein 3 OS=Homo sapiens GN=TDRD3 - (TDRD3_HUMAN)	14.65	10.48	3	1	9	51	4.035E9	744	83.1	9.00
F8W6N8	Tripartite motif-containing protein 5 OS=Homo sapiens GN=TRIM5 PE=4 SV=1 - (F8W6N8_HUMAN)	14.60	22.94	1	1	5	11	5.142E7	327	37.8	5.86
O95036	Similar to 60S ribosomal protein L7; similar to P18124 (PID:d133021) OS=Homo sapiens GN=WUGSC:H_RG054D04.1 PE=2 SV=1 - (O95036_HUMAN)	14.59	34.01	5	5	8	12	1.047E8	247	29.0	10.71
P52565	Rho GDP-dissociation inhibitor 1 OS=Homo sapiens GN=ARHGDIA PE=1 SV=3 - (GDIR1_HUMAN)	14.53	25.98	3	3	5	8	7.698E7	204	23.2	5.11
C9J9U2	Protein PRRC2A OS=Homo sapiens GN=PRRC2A PE=4 SV=1 - (C9J9U2_HUMAN)	14.50	7.90	18	1	17	22	1.949E9	2138	227.0	9.50
B3KM62	cDNA FLJ10371 fis, clone NT2RM2001903, highly similar to Homo sapiens ubiquitin protein ligase E3 component n-recognin 4 (UBR4), mRNA OS=Homo sapiens PE=2 SV=1 - (B3KM62_HUMAN)	14.48	5.81	1	1	3	8	8.610E7	654	73.5	6.46
P11216	Glycogen phosphorylase, brain form OS=Homo sapiens GN=PYGB PE=1 SV=5 - (PYGB_HUMAN)	14.45	7.59	13	1	8	20	5.256E7	843	96.6	6.86
B4DL79	Kinesin-like protein KIF20A OS=Homo sapiens GN=KIF20A PE=2 SV=1 - (B4DL79_HUMAN)	14.44	8.26	2	1	7	21	1.694E8	872	98.0	6.74
Q96AW5	QARS protein (Fragment) OS=Homo sapiens GN=QARS PE=2 SV=2 - (Q96AW5_HUMAN)	14.36	8.93	10	1	5	12	1.199E9	717	81.7	7.15
P02649	Apolipoprotein E OS=Homo sapiens GN=APOE PE=1 SV=1 - (APOE_HUMAN)	14.30	23.97	8	3	11	18	1.978E8	317	36.1	5.73
Q9NTK5	Obg-like ATPase 1 OS=Homo sapiens GN=OLA1 PE=1 SV=2 - (OLA1_HUMAN)	14.28	14.65	7	2	5	8	2.622E7	396	44.7	7.81
E9PKU4	60S ribosomal protein L8 (Fragment) OS=Homo sapiens GN=RPL8 PE=4 SV=1 - (E9PKU4_HUMAN)	14.23	17.87	7	3	5	8	1.071E8	235	25.6	11.00
P49720	Proteasome subunit beta type-3 OS=Homo sapiens GN=PSMB3 PE=1 SV=2 - (PSB3_HUMAN)	14.17	20.00	1	3	3	4	4.185E7	205	22.9	6.55
Q2TU89	Aging-associated protein 1 OS=Homo sapiens GN=AAG1 PE=2 SV=1 - (Q2TU89_HUMAN)	14.14	10.56	10	1	7	23	2.451E9	701	79.6	6.74
Q6EKJ0	General transcription factor II-I repeat domain-containing protein 2B OS=Homo sapiens GN=GTF2IRD2B PE=1 SV=1 - (GTD2B_HUMAN)	14.06	11.38	5	1	6	9	4.605E8	949	107.2	5.85
Q15126	Phosphomevalonate kinase OS=Homo sapiens GN=PMVK PE=1 SV=3 - (PMVK_HUMAN)	14.01	30.73	1	3	6	7	8.284E7	192	22.0	5.73
B4DJQ8	cDNA FLJ55694, highly similar to Dipeptidyl-peptidase 1 (EC 3.4.14.1) OS=Homo sapiens PE=2 SV=1 - (B4DJQ8_HUMAN)	14.01	12.33	6	5	5	8	7.836E7	446	50.1	6.99
P20618	Proteasome subunit beta type-1 OS=Homo sapiens GN=PSMB1 PE=1 SV=2 - (PSB1_HUMAN)	13.98	21.58	4	3	3	5	2.489E7	241	26.5	8.13
A8K9K1	cDNA FLJ77199, highly similar to Homo sapiens ATPase, Ca++ transporting, ubiquitous (ATP2A3), transcript variant 6, mRNA OS=Homo sapiens PE=2 SV=1 - (A8K9K1_HUMAN)	13.82	9.02	8	2	8	13	2.001E8	998	109.1	5.62
H0UI49	Laminin, alpha 4, isoform CRA_b OS=Homo sapiens GN=LAMA4 PE=4 SV=1 - (H0UI49_HUMAN)	13.73	7.49	4	1	14	23	3.116E8	1816	201.7	6.30
B9EGR7	Rho guanine nucleotide exchange factor (GEF) 5 OS=Homo sapiens GN=ARHGEF5 PE=2 SV=1 - (B9EGR7_HUMAN)	13.69	5.95	5	1	8	18	1.701E8	1597	176.5	5.55
Q2L696	Nucb2 splice variant OS=Homo sapiens GN=Nucb2 PE=2 SV=1 - (Q2L696_HUMAN)	13.69	10.77	5	1	4	9	7.779E8	390	46.6	5.22
Q15614	Olfactory receptor 8G2 OS=Homo sapiens GN=OR8G2 PE=2 SV=2 - (OR8G2_HUMAN)	13.63	8.39	1	1	1	7	9.716E7	310	34.5	7.64
Q9Y543-2	Isoform 2 of Transcription factor HES-2 OS=Homo sapiens GN=HES2 - (HES2_HUMAN)	13.57	11.84	2	1	2	7	5.073E5	76	8.6	10.49
P62333	26S protease regulatory subunit 10B OS=Homo sapiens GN=PSMC6 PE=1 SV=1 - (PRS10_HUMAN)	13.56	21.59	4	2	6	7	4.742E7	389	44.1	7.49
F5H7J5	Regulatory-associated protein of mTOR OS=Homo sapiens GN=RPTOR PE=4 SV=1 - (F5H7J5_HUMAN)	13.48	3.06	2	1	3	9	4.770E8	1177	131.4	7.03
B4DII5	Importin subunit alpha OS=Homo sapiens PE=2 SV=1 - (B4DII5_HUMAN)	13.47	5.63	5	1	4	9	9.007E7	533	59.6	5.01
Q96HX3	Similar to ribophorin I (Fragment) OS=Homo sapiens PE=2 SV=1 - (Q96HX3_HUMAN)	13.46	11.09	5	1	5	8	4.545E8	568	64.5	6.55
A8MXN1	Receptor expression-enhancing protein 6 OS=Homo sapiens GN=REEP6 PE=4 SV=1 - (A8MXN1_HUMAN)	13.43	11.67	1	1	6	34	1.737E8	480	49.7	11.24
E9PP21	Cysteine and glycine-rich protein 1 OS=Homo sapiens GN=CSRP1 PE=4 SV=1 - (E9PP21_HUMAN)	13.41	32.50	13	4	4	11	1.041E8	160	16.9	8.91
Q59EK6	TNF receptor-associated protein 1 variant (Fragment) OS=Homo sapiens PE=2 SV=1 - (Q59EK6_HUMAN)	13.40	8.96	2	1	5	8	2.362E8	703	79.9	8.21
Q7Z3U5	Putative uncharacterized protein DKFZp686L0869 OS=Homo sapiens GN=DKFZp686L0869 PE=2 SV=1 - (Q7Z3U5_HUMAN)	13.36	6.04	11	2	7	14	2.821E7	1242	142.2	6.04
P59998	Actin-related protein 2/3 complex subunit 4 OS=Homo sapiens GN=ARPC4 PE=1 SV=3 - (ARPC4_HUMAN)	13.35	19.05	6	1	4	7	2.164E7	168	19.7	8.43
B7Z2R9	Lysosome-associated membrane glycoprotein 2 OS=Homo sapiens GN=LAMP2 PE=2 SV=1 - (B7Z2R9_HUMAN)	13.34	5.67	5	1	2	8	4.257E7	300	33.0	6.87
Q5TA02	Glutathione S-transferase omega 1 (Fragment) OS=Homo sapiens GN=GSTO1 PE=2 SV=1 - (Q5TA02_HUMAN)	13.33	19.50	4	1	5	9	1.078E8	200	23.3	7.21
Q8IZI4	B-lymphocyte stimulator (Fragment) OS=Homo sapiens GN=TNFSF13B PE=2 SV=1 - (Q8IZI4_HUMAN)	13.32	6.33	5	1	1	8	5.500E5	158	17.8	5.59
E5RI99	60S ribosomal protein L30 (Fragment) OS=Homo sapiens GN=RPL30 PE=3 SV=1 - (E5RI99_HUMAN)	13.29	35.09	3	3	4	6	8.284E7	114	12.6	9.55
D6RA63	Protein GAPT (Fragment) OS=Homo sapiens GN=GAPT PE=4 SV=1 - (D6RA63_HUMAN)	13.24	14.71	3	1	1	7	9.483E8	68	7.6	11.80
O95461-2	Isoform 2 of Glycosyltransferase-like protein LARGE1 OS=Homo sapiens GN=LARGE - (LARGE_HUMAN)	13.20	7.24	7	1	6	14	2.241E8	704	81.8	7.74
Q6P4A8	Phospholipase B-like 1 OS=Homo sapiens GN=PLBD1 PE=1 SV=2 - (PLBL1_HUMAN)	13.17	6.87	2	1	4	19	8.559E8	553	63.2	9.06
Q53H42	Syndecan 1 variant (Fragment) OS=Homo sapiens PE=2 SV=1 - (Q53H42_HUMAN)	13.09	16.15	5	1	2	6	1.258E8	161	16.9	4.58
P28072	Proteasome subunit beta type-6 OS=Homo sapiens GN=PSMB6 PE=1 SV=4 - (PSB6_HUMAN)	13.03	26.36	2	3	6	9	5.988E8	239	25.3	4.92
Q2TU34	Fructose-1,6-bisphosphatase 1 OS=Homo sapiens GN=FBP1 PE=2 SV=1 - (Q2TU34_HUMAN)	12.92	16.57	2	1	4	7	4.219E7	338	36.8	6.99
O60506-2	Isoform 2 of Heterogeneous nuclear ribonucleoprotein Q OS=Homo sapiens GN=SYNCRIP - (HNRPQ_HUMAN)	12.91	11.90	10	1	7	20	6.055E7	588	65.6	8.60
B7Z233	Acetyl-CoA acetyltransferase, cytosolic OS=Homo sapiens GN=ACAT2 PE=2 SV=1 - (B7Z233_HUMAN)	12.90	18.08	4	3	6	8	5.955E7	426	44.6	8.46
F8VR50	Actin-related protein 2/3 complex subunit 3 (Fragment) OS=Homo sapiens GN=ARPC3 PE=4 SV=1 - (F8VR50_HUMAN)	12.89	42.86	6	3	4	5	6.162E7	84	9.7	7.93
B4E241	Serine/arginine-rich-splicing factor 3 OS=Homo sapiens GN=SRSF3 PE=2 SV=1 - (B4E241_HUMAN)	12.85	61.29	2	5	6	11	1.284E8	124	14.2	10.08
E9PC74	Translation initiation factor eIF-2B subunit epsilon OS=Homo sapiens GN=EIF2B5 PE=4 SV=1 - (E9PC74_HUMAN)	12.84	9.50	4	1	5	13	1.597E8	705	78.3	5.21
P25787	Proteasome subunit alpha type-2 OS=Homo sapiens GN=PSMA2 PE=1 SV=2 - (PSA2_HUMAN)	12.83	26.07	3	2	4	6	4.524E7	234	25.9	7.43
I3L0K2	Thioredoxin domain-containing protein 17 OS=Homo sapiens GN=TXNDC17 PE=4 SV=1 - (I3L0K2_HUMAN)	12.79	32.00	3	2	2	5	5.375E7	75	8.4	5.76
B4DNH0	cDNA FLJ60313, highly similar to Epithelial-cadherin OS=Homo sapiens PE=2 SV=1 - (B4DNH0_HUMAN)	12.62	7.58	11	1	3	7	7.307E8	475	52.6	5.07
B2RDN9	cDNA, FLJ96699, highly similar to Homo sapiens thyroid autoantigen 70kDa (Ku antigen) (G22P1), mRNA OS=Homo sapiens PE=2 SV=1 - (B2RDN9_HUMAN)	12.53	13.46	9	2	8	13	5.107E8	609	69.7	6.70
Q2VIN3	RBM1 (Fragment) OS=Homo sapiens PE=4 SV=1 - (Q2VIN3_HUMAN)	12.51	26.82	9	1	10	17	1.603E8	384	41.4	9.83
P07384	Calpain-1 catalytic subunit OS=Homo sapiens GN=CAPN1 PE=1 SV=1 - (CAN1_HUMAN)	12.51	15.55	6	5	11	18	9.236E7	714	81.8	5.67
B4DDL4	cDNA FLJ53966, moderately similar to Homo sapiens cytidylate kinase (CMPK), mRNA OS=Homo sapiens PE=2 SV=1 - (B4DDL4_HUMAN)	12.48	20.99	8	2	3	5	1.836E7	181	20.5	8.95
Q13283	Ras GTPase-activating protein-binding protein 1 OS=Homo sapiens GN=G3BP1 PE=1 SV=1 - (G3BP1_HUMAN)	12.48	15.24	8	1	5	27	5.487E8	466	52.1	5.52
Q8TEK3	Histone-lysine N-methyltransferase, H3 lysine-79 specific OS=Homo sapiens GN=DOT1L PE=1 SV=2 - (DOT1L_HUMAN)	12.29	8.34	3	1	12	16	3.279E8	1739	184.7	9.33
P54253	Ataxin-1 OS=Homo sapiens GN=ATXN1 PE=1 SV=2 - (ATX1_HUMAN)	12.22	2.70	1	1	2	5	9.358E8	815	86.9	8.35
H7C3X0	Protein O-GlcNAcase (Fragment) OS=Homo sapiens GN=MGEA5 PE=4 SV=1 - (H7C3X0_HUMAN)	12.20	13.67	5	1	3	8	8.156E7	256	29.7	7.01
E7EQT4	Apoptotic chromatin condensation inducer in the nucleus OS=Homo sapiens GN=ACIN1 PE=4 SV=2 - (E7EQT4_HUMAN)	12.18	7.38	2	1	11	14	6.710E7	1301	147.3	6.73
P62304	Small nuclear ribonucleoprotein E OS=Homo sapiens GN=SNRPE PE=1 SV=1 - (RUXE_HUMAN)	12.17	29.35	2	2	2	5	5.277E7	92	10.8	9.44
B7Z1H4	cDNA FLJ56052, highly similar to Puromycin-sensitive aminopeptidase (EC 3.4.11.-) OS=Homo sapiens PE=2 SV=1 - (B7Z1H4_HUMAN)	12.16	12.29	12	4	7	7	7.347E7	602	68.0	5.76
Q8TCJ6	Putative uncharacterized protein DKFZp667C0210 (Fragment) OS=Homo sapiens GN=DKFZp667C0210 PE=2 SV=1 - (Q8TCJ6_HUMAN)	12.15	9.11	3	1	3	6	1.458E8	461	52.1	7.33
H0YJA0	Putative E3 ubiquitin-protein ligase UBR7 (Fragment) OS=Homo sapiens GN=UBR7 PE=4 SV=1 - (H0YJA0_HUMAN)	12.12	17.68	1	1	2	7	4.722E6	164	18.0	4.64
Q5VVM3	Phosphopantothenate--cysteine ligase OS=Homo sapiens GN=PPCS PE=2 SV=1 - (Q5VVM3_HUMAN)	12.09	16.90	2	1	4	13	1.504E7	213	22.6	6.32
O95714	E3 ubiquitin-protein ligase HERC2 OS=Homo sapiens GN=HERC2 PE=1 SV=2 - (HERC2_HUMAN)	12.09	4.59	4	1	19	28	1.464E9	4834	526.9	6.28
Q5R207	Carbamoylphosphate synthetase I OS=Homo sapiens GN=CPS1 PE=2 SV=1 - (Q5R207_HUMAN)	12.01	8.69	11	1	6	9	1.005E8	932	102.1	7.31
Q6P084	MAP3K2 protein (Fragment) OS=Homo sapiens GN=MAP3K2 PE=2 SV=1 - (Q6P084_HUMAN)	11.99	14.53	1	1	3	9	4.866E8	234	26.8	9.23
P20742-2	Isoform 2 of Pregnancy zone protein OS=Homo sapiens GN=PZP - (PZP_HUMAN)	11.99	3.15	3	1	3	7	1.538E8	1268	140.3	6.29
B7Z7I4	cDNA FLJ54568, highly similar to T-complex protein 1 subunit eta OS=Homo sapiens PE=2 SV=1 - (B7Z7I4_HUMAN)	11.95	9.26	10	1	3	6	2.028E7	443	49.0	8.22
Q5HYW3	Retrotransposon gag domain-containing protein 4 OS=Homo sapiens GN=RGAG4 PE=2 SV=1 - (RGAG4_HUMAN)	11.93	7.56	1	1	4	14	1.173E8	569	64.7	4.75
B5MCX3	Septin-2 OS=Homo sapiens GN=SEPT2 PE=3 SV=1 - (B5MCX3_HUMAN)	11.86	19.94	15	4	5	5	4.851E7	321	36.9	6.18
H7BY74	Metal transporter CNNM1 OS=Homo sapiens GN=CNNM1 PE=4 SV=1 - (H7BY74_HUMAN)	11.78	3.25	8	1	3	15	1.657E8	830	91.7	6.55
Q8WVC2	40S ribosomal protein S21 OS=Homo sapiens GN=RPS21 PE=2 SV=1 - (Q8WVC2_HUMAN)	11.74	56.79	5	3	4	6	3.589E7	81	8.8	8.50
B4DKN9	cDNA FLJ57740, highly similar to Transforming protein RhoA OS=Homo sapiens PE=2 SV=1 - (B4DKN9_HUMAN)	11.73	39.88	15	3	6	9	5.032E7	173	19.6	8.22
O00231	26S proteasome non-ATPase regulatory subunit 11 OS=Homo sapiens GN=PSMD11 PE=1 SV=3 - (PSD11_HUMAN)	11.70	12.32	4	3	4	5	3.640E7	422	47.4	6.48
B3KND4	Coatomer subunit gamma OS=Homo sapiens PE=2 SV=1 - (B3KND4_HUMAN)	11.68	7.20	9	2	4	17	8.810E7	681	75.9	5.63
E9PPP7	Putative uncharacterized protein C8orf73 (Fragment) OS=Homo sapiens GN=C8orf73 PE=4 SV=1 - (E9PPP7_HUMAN)	11.65	11.48	1	1	6	48	1.603E8	549	56.2	9.79
O95425	Supervillin OS=Homo sapiens GN=SVIL PE=1 SV=2 - (SVIL_HUMAN)	11.60	9.53	2	1	24	48	3.534E8	2214	247.6	6.98
Q9Y5H1-2	Isoform 2 of Protocadherin gamma-A2 OS=Homo sapiens GN=PCDHGA2 - (PCDG2_HUMAN)	11.59	7.90	2	1	6	20	6.816E8	823	90.2	4.87
B4DMP5	cDNA FLJ60050, highly similar to Vacuolar protein sorting protein 72 homolog OS=Homo sapiens PE=2 SV=1 - (B4DMP5_HUMAN)	11.57	16.95	3	1	4	9	3.388E8	236	26.7	5.33
E5RFF9	DNA replication complex GINS protein SLD5 OS=Homo sapiens GN=GINS4 PE=4 SV=1 - (E5RFF9_HUMAN)	11.56	21.35	4	1	3	15	1.453E8	192	22.6	4.89
P43686-2	Isoform 2 of 26S protease regulatory subunit 6B OS=Homo sapiens GN=PSMC4 - (PRS6B_HUMAN)	11.52	17.57	2	2	4	4	1.633E7	387	43.5	5.26
P49662-2	Isoform 2 of Caspase-4 OS=Homo sapiens GN=CASP4 - (CASP4_HUMAN)	11.50	19.31	7	1	4	12	2.665E8	321	36.7	5.69
B4E2Z3	4F2 cell-surface antigen heavy chain OS=Homo sapiens GN=SLC3A2 PE=2 SV=1 - (B4E2Z3_HUMAN)	11.47	7.63	7	1	3	5	5.531E7	511	55.9	5.17
B3KQT2	cDNA PSEC0148 fis, clone PLACE1007202, highly similar to Protein disulfide-isomerase A3 (EC 5.3.4.1) OS=Homo sapiens PE=2 SV=1 - (B3KQT2_HUMAN)	11.45	17.11	7	3	7	10	2.326E8	485	54.9	6.86
E7ES33	Septin-7 OS=Homo sapiens GN=SEPT7 PE=3 SV=2 - (E7ES33_HUMAN)	11.41	9.86	48	2	3	7	6.628E7	436	50.6	8.63
A8K2Q6	Peptidyl-prolyl cis-trans isomerase OS=Homo sapiens PE=2 SV=1 - (A8K2Q6_HUMAN)	11.38	8.96	4	1	2	5	8.163E7	212	22.7	8.40
O75419-2	Isoform 2 of Cell division control protein 45 homolog OS=Homo sapiens GN=CDC45 - (CDC45_HUMAN)	11.36	6.73	4	1	3	10	1.273E8	520	60.2	5.81
F5H022	Histone H1.0 OS=Homo sapiens GN=H1F0 PE=3 SV=1 - (F5H022_HUMAN)	11.32	15.25	3	2	3	5	4.032E7	177	19.2	10.76
P00568	Adenylate kinase isoenzyme 1 OS=Homo sapiens GN=AK1 PE=1 SV=3 - (KAD1_HUMAN)	11.31	11.34	4	1	2	4	4.551E6	194	21.6	8.63
Q86V20-2	Isoform 2 of Protein FAM35A OS=Homo sapiens GN=FAM35A - (FA35A_HUMAN)	11.27	5.75	2	1	5	12	3.232E8	904	101.7	6.71
Q13867	Bleomycin hydrolase OS=Homo sapiens GN=BLMH PE=1 SV=1 - (BLMH_HUMAN)	11.26	7.69	3	1	4	9	5.713E8	455	52.5	6.27
P01024	Complement C3 OS=Homo sapiens GN=C3 PE=1 SV=2 - (CO3_HUMAN)	11.26	6.37	2	1	11	17	2.759E8	1663	187.0	6.40
A4D2P2	Ras-related C3 botulinum toxin substrate 1 (Rho family, small GTP binding protein Rac1) OS=Homo sapiens GN=RAC1 PE=3 SV=1 - (A4D2P2_HUMAN)	11.20	26.35	7	4	4	6	5.189E7	148	16.8	8.91
Q8ND71	GTPase IMAP family member 8 OS=Homo sapiens GN=GIMAP8 PE=1 SV=2 - (GIMA8_HUMAN)	11.19	7.82	1	1	6	9	2.434E8	665	74.8	8.34
Q58FF6	Putative heat shock protein HSP 90-beta 4 OS=Homo sapiens GN=HSP90AB4P PE=5 SV=1 - (H90B4_HUMAN)	11.19	5.94	1	1	3	6	2.595E7	505	58.2	4.73
H0YN26	Acidic leucine-rich nuclear phosphoprotein 32 family member A OS=Homo sapiens GN=ANP32A PE=4 SV=1 - (H0YN26_HUMAN)	11.10	19.21	13	3	3	5	4.108E7	177	20.0	4.58
Q92621	Nuclear pore complex protein Nup205 OS=Homo sapiens GN=NUP205 PE=1 SV=3 - (NU205_HUMAN)	11.10	3.38	2	1	7	15	3.162E8	2012	227.8	6.19
H7C5G6	Protein FLJ39061 (Fragment) OS=Homo sapiens GN=FLJ39061 PE=4 SV=1 - (H7C5G6_HUMAN)	11.07	7.94	2	1	5	15	2.372E8	642	73.9	7.93
Q53G71	Calreticulin variant (Fragment) OS=Homo sapiens PE=2 SV=1 - (Q53G71_HUMAN)	11.07	12.56	3	1	6	12	3.673E8	406	46.9	4.45
E7EWS7	Ubiquitin-conjugating enzyme E2 L3 OS=Homo sapiens GN=UBE2L3 PE=3 SV=1 - (E7EWS7_HUMAN)	11.05	42.45	5	1	6	6	6.526E7	212	24.0	8.75
H7BYH4	Superoxide dismutase (Cu-Zn) OS=Homo sapiens GN=SOD1 PE=3 SV=1 - (H7BYH4_HUMAN)	11.04	17.78	2	1	2	5	5.354E5	135	13.9	6.11
Q9BTA9	WW domain-containing adapter protein with coiled-coil OS=Homo sapiens GN=WAC PE=1 SV=3 - (WAC_HUMAN)	11.04	14.99	3	1	8	12	8.254E7	647	70.7	9.45
P84098	60S ribosomal protein L19 OS=Homo sapiens GN=RPL19 PE=1 SV=1 - (RL19_HUMAN)	10.99	42.35	5	3	9	9	5.739E7	196	23.5	11.47
F5GXF0	Nuclear receptor subfamily 4 group A member 1 OS=Homo sapiens GN=NR4A1 PE=3 SV=1 - (F5GXF0_HUMAN)	10.99	6.90	6	1	3	6	1.091E8	652	70.4	7.61
F8W118	Nucleosome assembly protein 1-like 1 (Fragment) OS=Homo sapiens GN=NAP1L1 PE=3 SV=1 - (F8W118_HUMAN)	10.94	20.10	20	2	3	5	6.823E7	209	24.7	4.68
H0YCV9	CD44 antigen (Fragment) OS=Homo sapiens GN=CD44 PE=4 SV=1 - (H0YCV9_HUMAN)	10.94	21.29	33	2	6	11	5.316E7	249	27.3	5.27
B3KRA9	cDNA FLJ33948 fis, clone CTONG2018379, highly similar to Hexokinase-1 (EC 2.7.1.1) OS=Homo sapiens PE=2 SV=1 - (B3KRA9_HUMAN)	10.89	17.14	25	2	10	22	2.142E8	566	63.3	7.91
Q17RC7	Exocyst complex component 3-like protein 4 OS=Homo sapiens GN=EXOC3L4 PE=2 SV=2 - (EX3L4_HUMAN)	10.89	9.97	1	1	9	21	3.476E8	722	79.8	6.32
Q0D2H7	PCF11 protein (Fragment) OS=Homo sapiens GN=PCF11 PE=2 SV=1 - (Q0D2H7_HUMAN)	10.82	19.62	1	1	2	9	7.242E8	158	17.0	8.02
H0YKD8	60S ribosomal protein L28 OS=Homo sapiens GN=RPL28 PE=4 SV=1 - (H0YKD8_HUMAN)	10.80	38.82	9	4	8	11	7.456E7	170	19.1	11.46
P30046	D-dopachrome decarboxylase OS=Homo sapiens GN=DDT PE=1 SV=3 - (DOPD_HUMAN)	10.80	42.37	7	3	4	7	6.684E7	118	12.7	7.30
Q9Y4G6	Talin-2 OS=Homo sapiens GN=TLN2 PE=1 SV=4 - (TLN2_HUMAN)	10.78	6.69	4	1	13	19	7.913E8	2542	271.4	5.57
E7EUY0	DNA-dependent protein kinase catalytic subunit OS=Homo sapiens GN=PRKDC PE=4 SV=1 - (E7EUY0_HUMAN)	10.78	5.37	4	1	21	44	1.866E9	4096	465.1	7.17
D3DNV8	Leprecan-like 1, isoform CRA_b OS=Homo sapiens GN=LEPREL1 PE=4 SV=1 - (D3DNV8_HUMAN)	10.77	5.31	3	1	3	15	1.575E8	527	60.3	5.07
Q53EM5	Transketolase variant (Fragment) OS=Homo sapiens PE=2 SV=1 - (Q53EM5_HUMAN)	10.75	17.50	9	4	10	13	2.452E9	623	67.9	7.81
Q53H34	Ribosomal protein L13a variant (Fragment) OS=Homo sapiens PE=2 SV=1 - (Q53H34_HUMAN)	10.73	35.47	8	2	8	10	1.673E7	203	23.5	10.86
Q8WVM7	Cohesin subunit SA-1 OS=Homo sapiens GN=STAG1 PE=1 SV=3 - (STAG1_HUMAN)	10.71	9.62	7	1	13	16	3.736E8	1258	144.3	5.59
Q0D2K2	Kelch-like protein 30 OS=Homo sapiens GN=KLHL30 PE=2 SV=3 - (KLH30_HUMAN)	10.71	5.71	1	1	4	8	9.323E7	578	63.9	5.94
Q6ISF6	KRTAP9-2 protein (Fragment) OS=Homo sapiens GN=KRTAP9-2 PE=2 SV=1 - (Q6ISF6_HUMAN)	10.70	8.09	1	1	1	3	7.582E6	173	18.1	7.69
B3KTJ9	cDNA FLJ38393 fis, clone FEBRA2007212 OS=Homo sapiens PE=2 SV=1 - (B3KTJ9_HUMAN)	10.68	8.56	7	2	8	11	2.173E8	923	102.9	5.22
Q5TIA1	Meiosis inhibitor protein 1 OS=Homo sapiens GN=MEI1 PE=2 SV=2 - (MEI1_HUMAN)	10.68	6.59	6	1	7	15	5.837E8	1274	141.1	6.70
P42345	Serine/threonine-protein kinase mTOR OS=Homo sapiens GN=MTOR PE=1 SV=1 - (MTOR_HUMAN)	10.67	3.69	4	2	9	10	1.275E8	2549	288.7	7.17
C0IMJ3	Periostin isoform thy6 OS=Homo sapiens PE=2 SV=1 - (C0IMJ3_HUMAN)	10.66	11.65	14	1	7	9	3.550E8	781	87.2	7.94
Q07954	Prolow-density lipoprotein receptor-related protein 1 OS=Homo sapiens GN=LRP1 PE=1 SV=2 - (LRP1_HUMAN)	10.64	4.40	1	1	19	35	6.469E8	4544	504.3	5.39
A8K646	cDNA FLJ75699, highly similar to Homo sapiens osteoclast stimulating factor 1 (OSTF1), mRNA OS=Homo sapiens PE=2 SV=1 - (A8K646_HUMAN)	10.64	13.08	2	2	3	6	5.320E7	214	23.7	5.68
B2R8K9	cDNA, FLJ93950, highly similar to Homo sapiens glutamyl aminopeptidase (aminopeptidase A) (ENPEP), mRNA OS=Homo sapiens PE=2 SV=1 - (B2R8K9_HUMAN)	10.61	11.39	13	1	11	23	4.443E8	957	109.2	5.47
Q14692	Ribosome biogenesis protein BMS1 homolog OS=Homo sapiens GN=BMS1 PE=1 SV=1 - (BMS1_HUMAN)	10.60	5.85	1	1	9	33	4.609E8	1282	145.7	6.44
P18077	60S ribosomal protein L35a OS=Homo sapiens GN=RPL35A PE=1 SV=2 - (RL35A_HUMAN)	10.60	38.18	4	4	5	6	4.463E7	110	12.5	11.06
B4E0B7	Condensin complex subunit 1 OS=Homo sapiens PE=2 SV=1 - (B4E0B7_HUMAN)	10.58	5.83	5	1	9	10	1.352E9	1356	152.1	6.74
G3V129	Putative uncharacterized protein LOC138046 OS=Homo sapiens GN=RALYL PE=4 SV=1 - (G3V129_HUMAN)	10.57	12.50	5	1	3	6	1.200E8	304	33.9	8.87
E9PQV5	Coiled-coil domain-containing protein 37 OS=Homo sapiens GN=CCDC37 PE=4 SV=1 - (E9PQV5_HUMAN)	10.53	8.55	1	1	2	6	9.444E7	117	13.4	7.02
B3KY56	Extended synaptotagmin-1 OS=Homo sapiens GN=ESYT1 PE=2 SV=1 - (B3KY56_HUMAN)	10.43	5.67	4	1	6	10	1.150E8	1058	117.3	5.90
P05090	Apolipoprotein D OS=Homo sapiens GN=APOD PE=1 SV=1 - (APOD_HUMAN)	10.42	15.34	4	2	3	5	3.240E7	189	21.3	5.15
Q96CA6	ACAA1 protein OS=Homo sapiens GN=ACAA1 PE=2 SV=1 - (Q96CA6_HUMAN)	10.41	11.78	8	1	3	5	2.627E7	331	34.6	8.38
C9JAB2	Serine/arginine-rich-splicing factor 7 OS=Homo sapiens GN=SRSF7 PE=4 SV=1 - (C9JAB2_HUMAN)	10.39	28.94	8	1	9	14	1.036E8	235	26.9	11.85
Q96LX7-5	Isoform 2 of Coiled-coil domain-containing protein 17 OS=Homo sapiens GN=CCDC17 - (CCD17_HUMAN)	10.37	14.85	4	1	6	10	4.242E7	613	66.6	6.95
E9PGK7	Transient receptor potential cation channel subfamily M member 2 OS=Homo sapiens GN=TRPM2 PE=4 SV=1 - (E9PGK7_HUMAN)	10.36	8.44	5	1	9	26	1.897E9	1553	176.4	7.74
B5ME19	Eukaryotic translation initiation factor 3 subunit C OS=Homo sapiens GN=EIF3CL PE=4 SV=1 - (B5ME19_HUMAN)	10.27	9.52	15	2	7	9	1.398E8	914	105.4	5.64
Q7Z4W1	L-xylulose reductase OS=Homo sapiens GN=DCXR PE=1 SV=2 - (DCXR_HUMAN)	10.26	20.49	1	1	3	5	1.490E7	244	25.9	8.10
O00533	Neural cell adhesion molecule L1-like protein OS=Homo sapiens GN=CHL1 PE=1 SV=4 - (CHL1_HUMAN)	10.25	7.04	3	1	8	12	1.288E8	1208	135.0	5.76
A8K0H3	cDNA FLJ78093, highly similar to Homo sapiens ribosomal protein L29 (RPL29), mRNA OS=Homo sapiens PE=2 SV=1 - (A8K0H3_HUMAN)	10.21	28.93	4	2	4	13	9.834E7	159	17.8	11.74
Q9ULW8	Protein-arginine deiminase type-3 OS=Homo sapiens GN=PADI3 PE=1 SV=2 - (PADI3_HUMAN)	10.21	6.33	1	1	4	5	3.208E6	664	74.7	5.54
H9E7F7	Cytochrome c oxidase subunit 2 (Fragment) OS=Homo sapiens GN=COX2 PE=3 SV=1 - (H9E7F7_HUMAN)	10.18	25.81	177	1	4	4	6.448E7	217	24.5	4.73
Q5VYY1	Ankyrin repeat domain-containing protein 22 OS=Homo sapiens GN=ANKRD22 PE=2 SV=1 - (ANR22_HUMAN)	10.13	12.57	1	1	3	7	2.959E7	191	21.8	8.84
O76090	Bestrophin-1 OS=Homo sapiens GN=BEST1 PE=1 SV=1 - (BEST1_HUMAN)	10.09	9.40	5	1	4	10	1.285E8	585	67.6	6.90
Q96CE4	Stathmin OS=Homo sapiens GN=STMN1 PE=2 SV=1 - (Q96CE4_HUMAN)	10.06	34.90	20	3	6	8	8.616E7	149	17.3	5.97
B7ZLS9	Synaptonemal complex protein 1 OS=Homo sapiens GN=SYCP1 PE=2 SV=1 - (B7ZLS9_HUMAN)	10.02	9.94	5	1	11	58	2.244E8	976	114.1	5.96
Q6NUJ1	Proactivator polypeptide-like 1 OS=Homo sapiens GN=PSAPL1 PE=2 SV=2 - (SAPL1_HUMAN)	10.01	10.56	1	1	3	3	6.452E7	521	56.6	7.27
Q9H5Y7	SLIT and NTRK-like protein 6 OS=Homo sapiens GN=SLITRK6 PE=1 SV=3 - (SLIK6_HUMAN)	10.00	4.64	1	1	3	31	1.348E8	841	95.0	6.52
Q59GS3	Proteasome 26S ATPase subunit 5 variant (Fragment) OS=Homo sapiens PE=2 SV=1 - (Q59GS3_HUMAN)	9.99	20.00	3	3	6	9	6.421E7	345	38.7	6.47
B7ZKX5	KIF6 protein OS=Homo sapiens GN=KIF6 PE=2 SV=1 - (B7ZKX5_HUMAN)	9.95	12.55	12	1	8	12	4.358E8	797	90.5	6.95
Q9P2Z0	THAP domain-containing protein 10 OS=Homo sapiens GN=THAP10 PE=2 SV=1 - (THA10_HUMAN)	9.94	11.28	2	1	4	13	2.974E8	257	28.3	8.60
P42766	60S ribosomal protein L35 OS=Homo sapiens GN=RPL35 PE=1 SV=2 - (RL35_HUMAN)	9.93	47.15	4	3	7	9	1.162E8	123	14.5	11.05
B7Z8B1	T-complex protein 1 subunit delta OS=Homo sapiens PE=2 SV=1 - (B7Z8B1_HUMAN)	9.93	14.15	8	2	8	9	4.012E7	509	54.7	7.83
Q8TE49	OTU domain-containing protein 7A OS=Homo sapiens GN=OTUD7A PE=1 SV=1 - (OTU7A_HUMAN)	9.92	12.96	2	1	10	14	6.128E8	926	100.6	8.40
P23396	40S ribosomal protein S3 OS=Homo sapiens GN=RPS3 PE=1 SV=2 - (RS3_HUMAN)	9.91	30.86	18	3	7	11	1.081E8	243	26.7	9.66
Q96N16-2	Isoform 2 of Janus kinase and microtubule-interacting protein 1 OS=Homo sapiens GN=JAKMIP1 - (JKIP1_HUMAN)	9.90	10.23	2	1	8	15	3.100E8	831	97.4	5.80
P35268	60S ribosomal protein L22 OS=Homo sapiens GN=RPL22 PE=1 SV=2 - (RL22_HUMAN)	9.89	30.47	2	2	4	9	1.974E8	128	14.8	9.19
B4DP66	cDNA FLJ60755, highly similar to Leucine zipper putative tumor suppressor 2 OS=Homo sapiens PE=2 SV=1 - (B4DP66_HUMAN)	9.89	12.84	4	1	7	10	1.075E9	631	67.3	7.69
P49458	Signal recognition particle 9 kDa protein OS=Homo sapiens GN=SRP9 PE=1 SV=2 - (SRP09_HUMAN)	9.87	39.53	6	3	4	7	4.062E7	86	10.1	7.97
Q2TNB3	Cell migration-inducing protein 22 OS=Homo sapiens PE=2 SV=1 - (Q2TNB3_HUMAN)	9.85	25.10	5	3	5	6	2.265E7	239	27.3	5.58
Q12789-3	Isoform 2 of General transcription factor 3C polypeptide 1 OS=Homo sapiens GN=GTF3C1 - (TF3C1_HUMAN)	9.85	5.42	5	1	11	29	2.989E8	2084	236.1	7.52
B3KTA3	cDNA FLJ37935 fis, clone CTONG2005290, highly similar to FASCIN OS=Homo sapiens PE=2 SV=1 - (B3KTA3_HUMAN)	9.85	17.37	7	2	9	14	1.339E8	472	52.2	7.65
F4ZW63	NF45 OS=Homo sapiens PE=2 SV=1 - (F4ZW63_HUMAN)	9.82	10.77	4	2	5	9	5.564E7	390	43.0	5.34
P13667	Protein disulfide-isomerase A4 OS=Homo sapiens GN=PDIA4 PE=1 SV=2 - (PDIA4_HUMAN)	9.81	16.43	1	1	11	13	1.241E8	645	72.9	5.07
Q5VZB9	Doublesex- and mab-3-related transcription factor A1 OS=Homo sapiens GN=DMRTA1 PE=2 SV=1 - (DMRTA_HUMAN)	9.81	12.10	11	1	5	12	5.768E7	504	53.1	8.90
P12532	Creatine kinase U-type, mitochondrial OS=Homo sapiens GN=CKMT1A PE=1 SV=1 - (KCRU_HUMAN)	9.81	16.79	15	2	8	11	1.494E8	417	47.0	8.34
A0MZ66-2	Isoform 2 of Shootin-1 OS=Homo sapiens GN=KIAA1598 - (SHOT1_HUMAN)	9.74	25.44	7	1	9	10	3.142E8	456	52.6	5.43
B3KMT7	cDNA FLJ12561 fis, clone NT2RM4000798, highly similar to Brefeldin A-inhibited guanine nucleotide-exchange protein 2 OS=Homo sapiens PE=2 SV=1 - (B3KMT7_HUMAN)	9.72	10.53	1	1	7	11	6.845E8	750	86.5	6.43
B3KMV8	cDNA FLJ12766 fis, clone NT2RP2001520, highly similar to Calcium-binding mitochondrial carrier protein Aralar1 OS=Homo sapiens PE=2 SV=1 - (B3KMV8_HUMAN)	9.71	11.21	3	1	6	9	4.552E7	678	74.7	8.38
Q9P2D3-3	Isoform 3 of HEAT repeat-containing protein 5B OS=Homo sapiens GN=HEATR5B - (HTR5B_HUMAN)	9.69	6.96	3	1	12	16	1.418E8	1982	214.9	7.42
F5H608	ATP synthase subunit d, mitochondrial OS=Homo sapiens GN=ATP5H PE=4 SV=1 - (F5H608_HUMAN)	9.68	35.29	4	2	3	4	4.988E7	102	11.4	9.09
B4DLZ5	N-acetylglucosamine kinase OS=Homo sapiens GN=NAGK PE=2 SV=1 - (B4DLZ5_HUMAN)	9.53	17.69	10	3	7	14	5.373E7	390	42.0	6.68
P25398	40S ribosomal protein S12 OS=Homo sapiens GN=RPS12 PE=1 SV=3 - (RS12_HUMAN)	9.52	25.76	1	3	3	6	1.488E8	132	14.5	7.21
E7EWW0	UPF0505 protein C16orf62 OS=Homo sapiens GN=C16orf62 PE=4 SV=2 - (E7EWW0_HUMAN)	9.52	6.56	8	1	5	8	8.021E7	1052	118.5	7.84
A2ABS5	Dimethylarginine dimethylaminohydrolase 2 (Fragment) OS=Homo sapiens GN=DDAH2 PE=4 SV=1 - (A2ABS5_HUMAN)	9.51	12.83	6	1	3	26	1.039E9	187	19.9	8.09
P62906	60S ribosomal protein L10a OS=Homo sapiens GN=RPL10A PE=1 SV=2 - (RL10A_HUMAN)	9.50	25.81	1	1	6	9	3.303E8	217	24.8	9.94
O75146	Huntingtin-interacting protein 1-related protein OS=Homo sapiens GN=HIP1R PE=1 SV=2 - (HIP1R_HUMAN)	9.47	10.39	4	1	10	20	2.089E8	1068	119.3	6.67
O75368	SH3 domain-binding glutamic acid-rich-like protein OS=Homo sapiens GN=SH3BGRL PE=1 SV=1 - (SH3L1_HUMAN)	9.47	27.19	3	1	2	3	1.727E7	114	12.8	5.25
B3KXC0	cDNA FLJ45113 fis, clone BRAWH3034743, weakly similar to Mus musculus microtubule-associated protein 7 (Mtap7), mRNA OS=Homo sapiens PE=2 SV=1 - (B3KXC0_HUMAN)	9.46	12.97	9	2	8	15	9.870E7	617	67.8	7.90
Q8NEY1-7	Isoform 7 of Neuron navigator 1 OS=Homo sapiens GN=NAV1 - (NAV1_HUMAN)	9.41	4.26	9	1	6	15	1.053E9	1809	194.9	8.28
Q59G46	Thioredoxin-like 1 variant (Fragment) OS=Homo sapiens PE=2 SV=1 - (Q59G46_HUMAN)	9.40	19.29	5	1	4	7	7.432E7	280	31.3	4.83
H7C5W9	Sarcoplasmic/endoplasmic reticulum calcium ATPase 2 (Fragment) OS=Homo sapiens GN=ATP2A2 PE=3 SV=1 - (H7C5W9_HUMAN)	9.38	5.89	12	1	6	7	1.027E8	933	102.5	5.59
Q6UW46	VGPW2523 OS=Homo sapiens GN=UNQ2523 PE=2 SV=1 - (Q6UW46_HUMAN)	9.38	14.29	1	1	5	11	1.146E8	315	35.9	5.35
B4DFL2	Isocitrate dehydrogenase (NADP) OS=Homo sapiens GN=IDH2 PE=2 SV=1 - (B4DFL2_HUMAN)	9.36	19.00	6	3	7	10	4.873E7	400	45.2	7.75
Q15572-4	Isoform 4 of TATA box-binding protein-associated factor RNA polymerase I subunit C OS=Homo sapiens GN=TAF1C - (TAF1C_HUMAN)	9.35	8.57	8	1	4	11	3.488E7	537	58.4	8.76
I3L518	Cytosolic Fe-S cluster assembly factor NUBP1 (Fragment) OS=Homo sapiens GN=NUBP1 PE=4 SV=1 - (I3L518_HUMAN)	9.26	22.22	1	1	1	3	2.183E8	63	7.1	9.74
Q9H1H9-3	Isoform 3 of Kinesin-like protein KIF13A OS=Homo sapiens GN=KIF13A - (KI13A_HUMAN)	9.24	8.58	9	1	14	31	9.914E8	1749	195.9	5.69
Q6A163	Keratin, type I cytoskeletal 39 OS=Homo sapiens GN=KRT39 PE=1 SV=2 - (K1C39_HUMAN)	9.21	15.89	11	2	8	25	9.653E8	491	55.6	5.26
Q6NUK1-2	Isoform 2 of Calcium-binding mitochondrial carrier protein SCaMC-1 OS=Homo sapiens GN=SLC25A24 - (SCMC1_HUMAN)	9.19	14.41	4	1	5	7	2.930E8	458	51.3	5.88
Q96BN8	Protein FAM105B OS=Homo sapiens GN=FAM105B PE=1 SV=3 - (F105B_HUMAN)	9.15	18.18	1	1	5	11	2.500E9	352	40.2	5.47
P41250	Glycine--tRNA ligase OS=Homo sapiens GN=GARS PE=1 SV=3 - (SYG_HUMAN)	9.13	4.74	1	1	4	9	1.624E8	739	83.1	7.03
Q9UBS4	DnaJ homolog subfamily B member 11 OS=Homo sapiens GN=DNAJB11 PE=1 SV=1 - (DJB11_HUMAN)	9.12	15.08	1	1	5	16	3.470E8	358	40.5	6.18
P62841	40S ribosomal protein S15 OS=Homo sapiens GN=RPS15 PE=1 SV=2 - (RS15_HUMAN)	9.03	24.83	1	1	3	6	5.264E7	145	17.0	10.39
E7EWJ1	Rab11 family-interacting protein 3 OS=Homo sapiens GN=RAB11FIP3 PE=4 SV=1 - (E7EWJ1_HUMAN)	9.03	12.61	4	1	8	11	2.870E8	460	52.4	4.74
P19086	Guanine nucleotide-binding protein G(z) subunit alpha OS=Homo sapiens GN=GNAZ PE=2 SV=3 - (GNAZ_HUMAN)	8.99	18.59	4	1	8	12	1.011E8	355	40.9	7.61
H0YCI4	Nucleosome assembly protein 1-like 4 (Fragment) OS=Homo sapiens GN=NAP1L4 PE=3 SV=1 - (H0YCI4_HUMAN)	8.97	16.67	13	2	3	4	2.323E7	204	23.4	4.92
F6RFD5	Destrin OS=Homo sapiens GN=DSTN PE=4 SV=1 - (F6RFD5_HUMAN)	8.93	33.33	4	1	4	6	1.248E8	135	15.4	8.59
Q99598	Translin-associated protein X OS=Homo sapiens GN=TSNAX PE=1 SV=1 - (TSNAX_HUMAN)	8.87	28.62	1	1	7	19	4.379E9	290	33.1	6.55
B4DKS8	Heterogeneous nuclear ribonucleoprotein F OS=Homo sapiens GN=HNRNPF PE=2 SV=1 - (B4DKS8_HUMAN)	8.86	17.16	7	1	5	8	1.459E8	338	37.2	6.05
P28066	Proteasome subunit alpha type-5 OS=Homo sapiens GN=PSMA5 PE=1 SV=3 - (PSA5_HUMAN)	8.85	15.77	3	3	3	4	4.958E7	241	26.4	4.79
Q9H4L5-5	Isoform 2a of Oxysterol-binding protein-related protein 3 OS=Homo sapiens GN=OSBPL3 - (OSBL3_HUMAN)	8.83	16.36	11	1	8	14	8.872E8	642	72.2	7.06
Q5T749	Keratinocyte proline-rich protein OS=Homo sapiens GN=KPRP PE=1 SV=1 - (KPRP_HUMAN)	8.81	6.04	1	1	4	7	4.935E7	579	64.1	8.27
Q0EFC6	HSR1 protein OS=Homo sapiens GN=HSR1 PE=4 SV=1 - (Q0EFC6_HUMAN)	8.77	12.48	13	1	5	6	1.630E8	545	62.1	9.10
Q9Y3B8	Oligoribonuclease, mitochondrial OS=Homo sapiens GN=REXO2 PE=1 SV=3 - (ORN_HUMAN)	8.76	37.55	6	1	7	7	6.415E7	237	26.8	6.87
P14735	Insulin-degrading enzyme OS=Homo sapiens GN=IDE PE=1 SV=4 - (IDE_HUMAN)	8.75	4.42	2	1	4	6	1.121E8	1019	117.9	6.61
Q9UHF5	Interleukin-17B OS=Homo sapiens GN=IL17B PE=2 SV=1 - (IL17B_HUMAN)	8.72	30.00	2	1	4	7	1.436E8	180	20.4	9.23
F5H018	GTP-binding nuclear protein Ran (Fragment) OS=Homo sapiens GN=RAN PE=4 SV=1 - (F5H018_HUMAN)	8.71	23.12	5	4	4	4	3.477E7	199	22.5	8.73
P61970	Nuclear transport factor 2 OS=Homo sapiens GN=NUTF2 PE=1 SV=1 - (NTF2_HUMAN)	8.71	50.39	2	3	4	7	2.124E7	127	14.5	5.38
B2R5V0	cDNA, FLJ92637, highly similar to Homo sapiens nuclear receptor coactivator 4 (NCOA4), mRNA OS=Homo sapiens PE=2 SV=1 - (B2R5V0_HUMAN)	8.70	8.63	7	1	4	5	1.069E8	614	69.7	6.11
Q9UBI6	Guanine nucleotide-binding protein G(I)/G(S)/G(O) subunit gamma-12 OS=Homo sapiens GN=GNG12 PE=1 SV=3 - (GBG12_HUMAN)	8.67	47.22	2	3	3	4	1.452E7	72	8.0	8.97
G3V2B8	Methylenetetrahydrofolate dehydrogenase OS=Homo sapiens GN=MTHFD1 PE=3 SV=1 - (G3V2B8_HUMAN)	8.65	8.98	4	1	5	7	3.126E7	935	101.5	7.30
Q9NS13	Placenta apolipoprotein B48 receptor type 2 OS=Homo sapiens GN=APOB48R PE=2 SV=1 - (Q9NS13_HUMAN)	8.59	11.06	5	1	11	18	3.142E8	1076	113.4	4.39
B1AN92	Eukaryotic translation initiation factor 4 gamma 3 (Fragment) OS=Homo sapiens GN=EIF4G3 PE=4 SV=1 - (B1AN92_HUMAN)	8.59	17.73	1	1	3	10	1.105E8	220	24.4	10.04
A6H8H3	C14orf49 protein OS=Homo sapiens GN=C14orf49 PE=2 SV=1 - (A6H8H3_HUMAN)	8.58	11.09	10	2	8	13	8.738E8	974	112.1	6.23
E7EPB3	60S ribosomal protein L14 OS=Homo sapiens GN=RPL14 PE=4 SV=1 - (E7EPB3_HUMAN)	8.57	32.26	7	3	5	11	2.637E8	124	14.5	10.21
B7Z1I2	Aspartate aminotransferase OS=Homo sapiens GN=GOT1 PE=2 SV=1 - (B7Z1I2_HUMAN)	8.53	13.39	4	1	4	4	3.792E8	366	41.0	7.18
B4DFR2	Dynein light chain roadblock-type 1 OS=Homo sapiens GN=DYNLRB1 PE=2 SV=1 - (B4DFR2_HUMAN)	8.51	19.83	5	1	2	3	4.310E7	121	13.4	10.08
Q96P63	Serpin B12 OS=Homo sapiens GN=SERPINB12 PE=1 SV=1 - (SPB12_HUMAN)	8.50	15.56	3	2	5	5	1.686E8	405	46.2	5.53
F5H0X6	Proactivator polypeptide OS=Homo sapiens GN=PSAP PE=4 SV=1 - (F5H0X6_HUMAN)	8.43	8.22	12	2	3	4	2.778E7	450	50.4	5.01
Q9Y3A5	Ribosome maturation protein SBDS OS=Homo sapiens GN=SBDS PE=1 SV=4 - (SBDS_HUMAN)	8.43	10.00	1	1	3	5	1.574E7	250	28.7	8.75
B4DWB4	cDNA FLJ58332, highly similar to MBD2-interacting zinc finger protein OS=Homo sapiens PE=2 SV=1 - (B4DWB4_HUMAN)	8.38	4.30	3	1	2	11	9.827E7	419	48.9	7.28
H0Y2S5	LIM and calponin homology domains-containing protein 1 OS=Homo sapiens GN=LIMCH1 PE=4 SV=1 - (H0Y2S5_HUMAN)	8.28	11.39	22	1	12	15	6.264E8	1466	164.2	6.35
Q9UJZ1	Stomatin-like protein 2 OS=Homo sapiens GN=STOML2 PE=1 SV=1 - (STML2_HUMAN)	8.27	16.29	2	1	3	4	1.073E8	356	38.5	7.39
P48637	Glutathione synthetase OS=Homo sapiens GN=GSS PE=1 SV=1 - (GSHB_HUMAN)	8.21	14.98	4	2	7	7	4.724E7	474	52.4	5.92
B4E0Z6	cDNA FLJ59809, highly similar to Bone marrow stromal antigen 2 OS=Homo sapiens PE=2 SV=1 - (B4E0Z6_HUMAN)	8.20	35.19	2	1	4	10	1.074E8	216	23.5	5.92
B7Z6D1	cDNA FLJ57430, highly similar to DNA-directed RNA polymerase II 33 kDa polypeptide (EC 2.7.7.6) OS=Homo sapiens PE=2 SV=1 - (B7Z6D1_HUMAN)	8.19	17.22	3	1	5	9	8.547E7	424	47.4	5.27
Q8NFA0	Ubiquitin carboxyl-terminal hydrolase 32 OS=Homo sapiens GN=USP32 PE=1 SV=1 - (UBP32_HUMAN)	8.15	6.98	2	1	11	22	7.268E7	1604	181.5	6.44
E7ERI3	Threonine--tRNA ligase, cytoplasmic OS=Homo sapiens GN=TARS PE=3 SV=1 - (E7ERI3_HUMAN)	8.13	20.60	9	1	10	16	1.564E8	602	70.3	8.56
Q9NQ38-3	Isoform long of Serine protease inhibitor Kazal-type 5 OS=Homo sapiens GN=SPINK5 - (ISK5_HUMAN)	8.13	8.50	2	1	9	18	1.079E9	1094	124.0	8.09
Q9BYR7	Keratin-associated protein 3-2 OS=Homo sapiens GN=KRTAP3-2 PE=1 SV=1 - (KRA32_HUMAN)	8.13	17.35	1	1	1	2	1.236E7	98	10.4	5.69
P17900	Ganglioside GM2 activator OS=Homo sapiens GN=GM2A PE=1 SV=4 - (SAP3_HUMAN)	8.08	13.47	1	1	2	3	5.774E6	193	20.8	5.31
F5H1F8	Chloride intracellular channel protein 4 OS=Homo sapiens GN=CLIC4 PE=4 SV=1 - (F5H1F8_HUMAN)	8.07	17.39	13	2	3	5	2.510E7	207	23.3	5.34
Q8N7R1	POM121-like protein 12 OS=Homo sapiens GN=POM121L12 PE=2 SV=3 - (P1L12_HUMAN)	8.06	11.82	1	1	2	5	2.663E8	296	31.8	9.03
B3KPC7	Actin-related protein 2/3 complex subunit 5 OS=Homo sapiens PE=2 SV=1 - (B3KPC7_HUMAN)	8.05	16.99	2	1	2	3	4.789E7	153	17.0	6.02
Q99714-2	Isoform 2 of 3-hydroxyacyl-CoA dehydrogenase type-2 OS=Homo sapiens GN=HSD17B10 - (HCD2_HUMAN)	8.04	15.87	3	2	2	2	1.762E7	252	26.0	7.21
D3DPB7	Sodium channel, voltage-gated, type IX, alpha, isoform CRA_a OS=Homo sapiens GN=SCN9A PE=3 SV=1 - (D3DPB7_HUMAN)	8.03	5.30	6	1	6	16	1.209E8	1339	152.8	6.21
Q6ZMU0	Delta-aminolevulinic acid dehydratase OS=Homo sapiens PE=2 SV=1 - (Q6ZMU0_HUMAN)	8.03	20.06	5	4	5	7	1.029E8	359	39.0	7.65
Q6P5X8	DNA-directed DNA/RNA polymerase mu OS=Homo sapiens GN=POLM PE=2 SV=1 - (Q6P5X8_HUMAN)	8.01	5.47	11	1	5	12	5.784E8	457	50.4	8.95
H0YGV0	DnaJ homolog subfamily C member 4 (Fragment) OS=Homo sapiens GN=DNAJC4 PE=4 SV=1 - (H0YGV0_HUMAN)	7.99	16.17	2	1	3	7	2.302E7	167	19.3	10.48
Q2YDC2	HCG1985580, isoform CRA_f OS=Homo sapiens GN=PDCD6 PE=2 SV=1 - (Q2YDC2_HUMAN)	7.97	21.16	5	3	4	4	4.186E7	189	21.7	5.40
H0YLM9	Proline-serine-threonine phosphatase-interacting protein 1 (Fragment) OS=Homo sapiens GN=PSTPIP1 PE=4 SV=1 - (H0YLM9_HUMAN)	7.96	30.86	2	1	6	14	2.569E7	175	19.9	8.41
Q9Y305	Acyl-coenzyme A thioesterase 9, mitochondrial OS=Homo sapiens GN=ACOT9 PE=1 SV=2 - (ACOT9_HUMAN)	7.96	11.62	2	1	6	9	1.771E8	439	49.9	8.60
P62273	40S ribosomal protein S29 OS=Homo sapiens GN=RPS29 PE=1 SV=2 - (RS29_HUMAN)	7.92	39.29	2	2	3	7	5.955E7	56	6.7	10.13
A6NDW3	Lipolysis-stimulated lipoprotein receptor OS=Homo sapiens GN=LSR PE=4 SV=1 - (A6NDW3_HUMAN)	7.87	6.37	5	1	4	6	5.484E7	581	64.0	8.78
Q8NC51-4	Isoform 4 of Plasminogen activator inhibitor 1 RNA-binding protein OS=Homo sapiens GN=SERBP1 - (PAIRB_HUMAN)	7.83	21.19	7	2	8	8	4.448E7	387	42.4	8.44
A7E2A5	ARAP2 protein (Fragment) OS=Homo sapiens GN=ARAP2 PE=2 SV=1 - (A7E2A5_HUMAN)	7.83	6.18	2	1	10	20	5.675E8	1634	185.4	7.37
Q9BVC6	Transmembrane protein 109 OS=Homo sapiens GN=TMEM109 PE=1 SV=1 - (TM109_HUMAN)	7.80	13.99	4	1	3	7	1.085E8	243	26.2	10.48
P42677	40S ribosomal protein S27 OS=Homo sapiens GN=RPS27 PE=1 SV=3 - (RS27_HUMAN)	7.80	40.48	7	3	3	3	1.394E7	84	9.5	9.45
G3V5Q1	DNA-(apurinic or apyrimidinic site) lyase (Fragment) OS=Homo sapiens GN=APEX1 PE=4 SV=1 - (G3V5Q1_HUMAN)	7.77	9.92	3	1	2	3	3.405E7	242	27.1	8.76
Q6ECI4	Zinc finger protein 470 OS=Homo sapiens GN=ZNF470 PE=1 SV=3 - (ZN470_HUMAN)	7.75	4.74	2	1	4	10	2.692E8	717	82.6	8.57
C9J8H1	V-type proton ATPase subunit E 1 (Fragment) OS=Homo sapiens GN=ATP6V1E1 PE=4 SV=1 - (C9J8H1_HUMAN)	7.73	10.84	3	1	2	4	5.144E7	203	23.5	9.04
Q9C0F0	Putative Polycomb group protein ASXL3 OS=Homo sapiens GN=ASXL3 PE=2 SV=3 - (ASXL3_HUMAN)	7.70	3.87	2	1	9	18	1.621E8	2248	241.8	6.14
Q6P179-3	Isoform 3 of Endoplasmic reticulum aminopeptidase 2 OS=Homo sapiens GN=ERAP2 - (ERAP2_HUMAN)	7.67	6.34	3	1	5	10	7.315E7	915	105.5	6.84
Q1JUQ5	Peptidyl-prolyl cis-trans isomerase OS=Homo sapiens GN=FKBP12-Exip2 PE=2 SV=1 - (Q1JUQ5_HUMAN)	7.67	15.22	4	1	1	2	1.783E7	92	10.1	5.90
P46778	60S ribosomal protein L21 OS=Homo sapiens GN=RPL21 PE=1 SV=2 - (RL21_HUMAN)	7.66	22.50	3	2	4	4	5.300E7	160	18.6	10.49
Q01664	Transcription factor AP-4 OS=Homo sapiens GN=TFAP4 PE=1 SV=2 - (TFAP4_HUMAN)	7.64	15.98	4	1	6	10	2.774E8	338	38.7	5.87
Q05DH1	Proteasome subunit alpha type (Fragment) OS=Homo sapiens GN=PSMA7 PE=2 SV=1 - (Q05DH1_HUMAN)	7.62	37.39	14	1	9	13	1.120E8	238	26.7	8.87
F6USW4	F-actin-capping protein subunit beta (Fragment) OS=Homo sapiens GN=CAPZB PE=4 SV=1 - (F6USW4_HUMAN)	7.61	12.14	8	1	1	2	5.509E6	206	23.1	5.94
Q6ZRK6	Coiled-coil domain-containing protein 73 OS=Homo sapiens GN=CCDC73 PE=1 SV=2 - (CCD73_HUMAN)	7.58	6.39	2	1	6	6	8.610E7	1079	124.1	5.58
C9IZG4	Protein CutA OS=Homo sapiens GN=CUTA PE=4 SV=1 - (C9IZG4_HUMAN)	7.58	23.70	5	2	2	2	7.555E7	135	14.4	5.45
B3KWD0	cDNA FLJ42779 fis, clone BRAWH3005300, highly similar to Exportin-1 OS=Homo sapiens PE=2 SV=1 - (B3KWD0_HUMAN)	7.58	9.47	4	2	5	5	3.215E7	718	82.4	5.71
P23297	Protein S100-A1 OS=Homo sapiens GN=S100A1 PE=1 SV=2 - (S10A1_HUMAN)	7.53	23.40	2	2	2	2	1.118E7	94	10.5	4.50
Q5W0H4	Tumor protein, translationally-controlled 1 OS=Homo sapiens GN=TPT1 PE=2 SV=1 - (Q5W0H4_HUMAN)	7.52	19.68	10	2	5	6	3.960E7	188	21.5	5.49
Q8IZF2-3	Isoform 3 of Probable G-protein coupled receptor 116 OS=Homo sapiens GN=GPR116 - (GP116_HUMAN)	7.52	4.24	6	1	5	6	2.278E8	1204	133.2	7.23
Q38G99	C-myc intron-binding protein 1 OS=Homo sapiens GN=MIBP1 PE=2 SV=1 - (Q38G99_HUMAN)	7.49	9.81	2	1	14	19	3.678E8	2446	268.6	6.92
Q96E29	mTERF domain-containing protein 1, mitochondrial OS=Homo sapiens GN=MTERFD1 PE=1 SV=2 - (MTER1_HUMAN)	7.49	6.71	3	1	3	5	3.427E7	417	47.9	8.53
H0YFA4	Cysteine-rich protein 2 (Fragment) OS=Homo sapiens GN=CRIP2 PE=4 SV=1 - (H0YFA4_HUMAN)	7.45	17.71	4	1	3	5	3.872E7	192	20.7	8.94
D6RF73	Aryl hydrocarbon receptor repressor OS=Homo sapiens GN=AHRR PE=4 SV=1 - (D6RF73_HUMAN)	7.44	8.96	5	1	5	7	2.260E8	547	59.4	8.57
Q5VU13	V-set and immunoglobulin domain-containing protein 8 OS=Homo sapiens GN=VSIG8 PE=1 SV=1 - (VSIG8_HUMAN)	7.42	9.66	1	3	4	10	2.712E7	414	43.9	7.20
Q7RTM1	Otopetrin-1 OS=Homo sapiens GN=OTOP1 PE=2 SV=1 - (OTOP1_HUMAN)	7.41	3.10	1	1	2	16	1.350E8	612	67.3	8.41
Q4J6C6-4	Isoform 4 of Prolyl endopeptidase-like OS=Homo sapiens GN=PREPL - (PPCEL_HUMAN)	7.40	9.40	3	1	5	14	6.822E8	638	73.3	5.44
Q96RP6	Ubiquitin-conjugating enzyme OS=Homo sapiens GN=PUBC1 PE=2 SV=1 - (Q96RP6_HUMAN)	7.39	42.37	15	2	3	5	4.358E7	118	13.6	8.29
C9JFR7	Cytochrome c (Fragment) OS=Homo sapiens GN=CYCS PE=3 SV=1 - (C9JFR7_HUMAN)	7.33	25.74	3	3	3	4	2.886E7	101	11.3	9.66
Q13011	Delta(3,5)-Delta(2,4)-dienoyl-CoA isomerase, mitochondrial OS=Homo sapiens GN=ECH1 PE=1 SV=2 - (ECH1_HUMAN)	7.30	17.99	1	1	4	5	2.228E7	328	35.8	8.00
Q02156	Protein kinase C epsilon type OS=Homo sapiens GN=PRKCE PE=1 SV=1 - (KPCE_HUMAN)	7.30	14.25	4	1	9	18	2.258E8	737	83.6	7.12
Q03252	Lamin-B2 OS=Homo sapiens GN=LMNB2 PE=1 SV=3 - (LMNB2_HUMAN)	7.27	20.33	1	1	11	14	3.500E8	600	67.6	5.35
B4DTD3	cDNA FLJ50209, highly similar to DNA-repair protein XRCC1 OS=Homo sapiens PE=2 SV=1 - (B4DTD3_HUMAN)	7.27	6.64	6	1	4	7	1.989E8	602	66.0	5.85
Q86UF4	Coiled-coil domain-containing protein C1orf110 OS=Homo sapiens GN=C1orf110 PE=2 SV=2 - (CA110_HUMAN)	7.26	27.15	1	1	6	9	6.566E8	302	34.1	9.57
Q9HAU6	Putative apoptosis inhibitor FKSG2 OS=Homo sapiens GN=FKSG2 PE=5 SV=1 - (FKSG2_HUMAN)	7.20	18.50	1	1	3	9	1.093E8	173	20.3	5.05
Q49MI3-4	Isoform 4 of Ceramide kinase-like protein OS=Homo sapiens GN=CERKL - (CERKL_HUMAN)	7.19	10.02	8	1	5	8	2.485E8	419	47.3	8.13
F8WDL0	AP-1 complex subunit beta-1 OS=Homo sapiens GN=AP1B1 PE=4 SV=1 - (F8WDL0_HUMAN)	7.17	7.94	6	1	6	6	2.991E7	919	101.3	5.11
G3V5R8	Alpha-1-antitrypsin (Fragment) OS=Homo sapiens GN=SERPINA1 PE=4 SV=1 - (G3V5R8_HUMAN)	7.14	15.22	6	1	1	2	2.126E7	92	9.9	5.58
A8K202	5-aminoimidazole-4-carboxamide ribonucleotide formyltransferase/IMP cyclohydrolase, isoform CRA_g OS=Homo sapiens GN=ATIC PE=2 SV=1 - (A8K202_HUMAN)	7.12	10.15	2	1	3	6	4.162E7	591	64.5	6.71
O75410-5	Isoform 5 of Transforming acidic coiled-coil-containing protein 1 OS=Homo sapiens GN=TACC1 - (TACC1_HUMAN)	7.11	10.35	19	1	5	8	5.474E7	367	40.9	5.08
Q9Y3U8	60S ribosomal protein L36 OS=Homo sapiens GN=RPL36 PE=1 SV=3 - (RL36_HUMAN)	7.10	21.90	1	3	3	3	6.568E7	105	12.2	11.59
A8K0T9	cDNA FLJ75422, highly similar to Homo sapiens capping protein (actin filament) muscle Z-line, alpha 1, mRNA OS=Homo sapiens PE=2 SV=1 - (A8K0T9_HUMAN)	7.10	11.19	5	2	3	4	1.263E8	286	32.9	5.69
Q9UPR0	Inactive phospholipase C-like protein 2 OS=Homo sapiens GN=PLCL2 PE=1 SV=2 - (PLCL2_HUMAN)	7.07	9.14	1	1	10	19	1.604E8	1127	125.8	6.90
B7ZBG9	Eukaryotic translation initiation factor 6 OS=Homo sapiens GN=EIF6 PE=3 SV=1 - (B7ZBG9_HUMAN)	6.99	22.57	2	2	3	6	6.936E7	226	23.7	5.17
Q14591-2	Isoform 2 of Zinc finger protein 271 OS=Homo sapiens GN=ZNF271 - (ZN271_HUMAN)	6.96	5.67	1	1	3	6	6.478E8	423	48.1	9.00
E9PAV3	Nascent polypeptide-associated complex subunit alpha OS=Homo sapiens GN=NACA PE=4 SV=1 - (E9PAV3_HUMAN)	6.95	5.49	9	1	5	5	1.947E8	2078	205.3	9.58
A8MVP6	T-cell surface glycoprotein CD3 delta chain OS=Homo sapiens GN=CD3D PE=4 SV=1 - (A8MVP6_HUMAN)	6.94	21.26	2	1	3	11	2.091E9	127	14.5	6.30
P60900	Proteasome subunit alpha type-6 OS=Homo sapiens GN=PSMA6 PE=1 SV=1 - (PSA6_HUMAN)	6.92	21.14	9	3	4	4	1.190E8	246	27.4	6.76
P14314-2	Isoform 2 of Glucosidase 2 subunit beta OS=Homo sapiens GN=PRKCSH - (GLU2B_HUMAN)	6.92	7.43	4	3	4	4	2.567E7	525	59.1	4.42
Q9H9L3	Interferon-stimulated 20 kDa exonuclease-like 2 OS=Homo sapiens GN=ISG20L2 PE=1 SV=1 - (I20L2_HUMAN)	6.92	22.38	1	1	7	12	1.365E8	353	39.1	9.94
E9PNX0	FYVE, RhoGEF and PH domain-containing protein 4 OS=Homo sapiens GN=FGD4 PE=4 SV=1 - (E9PNX0_HUMAN)	6.88	9.36	2	1	2	7	6.314E7	235	25.8	4.93
Q5VZ89-6	Isoform 6 of DENN domain-containing protein 4C OS=Homo sapiens GN=DENND4C - (DEN4C_HUMAN)	6.85	5.10	7	1	7	13	2.915E8	1607	179.3	7.05
O00425	Insulin-like growth factor 2 mRNA-binding protein 3 OS=Homo sapiens GN=IGF2BP3 PE=1 SV=2 - (IF2B3_HUMAN)	6.81	9.67	2	1	6	13	9.399E8	579	63.7	8.87
B2R806	Eukaryotic translation initiation factor 3 subunit E OS=Homo sapiens PE=2 SV=1 - (B2R806_HUMAN)	6.79	17.08	12	2	6	6	4.854E8	445	52.2	6.04
F8W950	Aminoacyl tRNA synthase complex-interacting multifunctional protein 2 OS=Homo sapiens GN=AIMP2 PE=4 SV=1 - (F8W950_HUMAN)	6.76	15.94	4	1	3	7	9.598E7	251	27.8	9.32
B2R8A2	cDNA, FLJ93804, highly similar to Homo sapiens gp25L2 protein (HSGP25L2G), mRNA OS=Homo sapiens PE=2 SV=1 - (B2R8A2_HUMAN)	6.76	14.02	2	2	3	6	1.621E7	214	25.1	7.18
Q9Y4F4	Protein FAM179B OS=Homo sapiens GN=FAM179B PE=1 SV=4 - (F179B_HUMAN)	6.75	6.16	1	1	10	13	2.179E8	1720	189.2	8.50
B4DWL3	Lysosome-associated membrane glycoprotein 1 OS=Homo sapiens GN=LAMP1 PE=2 SV=1 - (B4DWL3_HUMAN)	6.73	9.07	3	1	4	7	1.842E8	364	39.0	8.87
Q59GB4	Dihydropyrimidinase-like 2 variant (Fragment) OS=Homo sapiens PE=2 SV=1 - (Q59GB4_HUMAN)	6.71	11.15	13	1	6	7	9.128E7	628	68.1	6.24
Q9P278	Folliculin-interacting protein 2 OS=Homo sapiens GN=FNIP2 PE=1 SV=2 - (FNIP2_HUMAN)	6.70	4.04	2	1	6	22	1.517E9	1114	122.0	6.62
Q13185	Chromobox protein homolog 3 OS=Homo sapiens GN=CBX3 PE=1 SV=4 - (CBX3_HUMAN)	6.70	28.42	3	2	4	4	2.222E7	183	20.8	5.33
Q9H501	ESF1 homolog OS=Homo sapiens GN=ESF1 PE=1 SV=1 - (ESF1_HUMAN)	6.65	6.11	4	1	5	6	1.539E8	851	98.7	5.11
Q5H9U9	Probable ATP-dependent RNA helicase DDX60-like OS=Homo sapiens GN=DDX60L PE=2 SV=2 - (DDX6L_HUMAN)	6.64	6.86	3	1	10	12	8.144E7	1706	197.5	8.29
B2RTX8	WAPAL protein OS=Homo sapiens GN=WAPAL PE=2 SV=1 - (B2RTX8_HUMAN)	6.61	4.65	5	1	5	9	2.138E8	1184	132.1	5.48
Q70EL4-2	Isoform 2 of Ubiquitin carboxyl-terminal hydrolase 43 OS=Homo sapiens GN=USP43 - (UBP43_HUMAN)	6.60	18.58	1	1	7	9	1.179E8	635	69.3	9.98
Q96PU5-3	Isoform 3 of E3 ubiquitin-protein ligase NEDD4-like OS=Homo sapiens GN=NEDD4L - (NED4L_HUMAN)	6.60	5.05	13	1	4	5	9.483E7	871	100.7	5.58
P61129	Zinc finger CCCH domain-containing protein 6 OS=Homo sapiens GN=ZC3H6 PE=2 SV=2 - (ZC3H6_HUMAN)	6.55	8.33	3	1	6	14	2.802E8	1189	131.6	7.64
I1SRC5	UBE2L3/KRAS fusion protein OS=Homo sapiens PE=2 SV=1 - (I1SRC5_HUMAN)	6.53	17.23	4	1	5	6	5.945E7	296	34.0	8.10
Q9UFP1	Protein FAM198A OS=Homo sapiens GN=FAM198A PE=1 SV=3 - (F198A_HUMAN)	6.51	9.91	2	1	5	7	1.058E8	575	63.6	7.46
B4E086	cDNA FLJ51584, highly similar to Homo sapiens spindle pole body component 24 homolog (SPBC24), mRNA OS=Homo sapiens PE=2 SV=1 - (B4E086_HUMAN)	6.45	15.00	1	1	2	5	2.384E8	140	16.0	4.55
P36405	ADP-ribosylation factor-like protein 3 OS=Homo sapiens GN=ARL3 PE=1 SV=2 - (ARL3_HUMAN)	6.45	16.48	1	1	3	3	4.755E6	182	20.4	7.24
Q56VL3	OCIA domain-containing protein 2 OS=Homo sapiens GN=OCIAD2 PE=1 SV=1 - (OCAD2_HUMAN)	6.45	21.43	1	1	2	4	2.820E7	154	16.9	9.03
O00410	Importin-5 OS=Homo sapiens GN=IPO5 PE=1 SV=4 - (IPO5_HUMAN)	6.42	7.66	6	1	6	8	4.393E7	1097	123.5	4.94
Q9Y6W5	Wiskott-Aldrich syndrome protein family member 2 OS=Homo sapiens GN=WASF2 PE=1 SV=3 - (WASF2_HUMAN)	6.41	10.44	3	1	5	6	4.448E7	498	54.3	5.53
B4DVQ3	Calcium/calmodulin-dependent protein kinase type II subunit gamma OS=Homo sapiens GN=CAMK2G PE=2 SV=1 - (B4DVQ3_HUMAN)	6.41	9.44	1	1	1	5	1.457E8	180	20.5	8.75
A7E261	5-oxoprolinase (ATP-hydrolysing) OS=Homo sapiens GN=OPLAH PE=2 SV=1 - (A7E261_HUMAN)	6.41	4.74	2	1	6	11	2.490E8	1288	137.3	6.58
B4DF76	cDNA FLJ59191, highly similar to NADH dehydrogenase (ubiquinone) 1 alpha subcomplex subunit 13 (EC 1.6.5.3) OS=Homo sapiens PE=2 SV=1 - (B4DF76_HUMAN)	6.37	29.28	2	1	4	11	2.911E8	222	24.9	9.70
B3KPE5	cDNA FLJ31682 fis, clone NT2RI2005335, highly similar to MMS19-like protein OS=Homo sapiens PE=2 SV=1 - (B3KPE5_HUMAN)	6.37	8.72	6	1	5	10	1.139E8	665	72.9	7.18
B4DKB2	Endothelin-converting enzyme 1 OS=Homo sapiens GN=ECE1 PE=2 SV=1 - (B4DKB2_HUMAN)	6.37	8.27	5	1	5	10	2.008E8	738	83.5	5.73
G3XAD8	Stress-induced-phosphoprotein 1 OS=Homo sapiens GN=STIP1 PE=4 SV=1 - (G3XAD8_HUMAN)	6.36	11.69	6	1	7	12	1.012E9	590	68.0	7.74
Q96JD6-4	Isoform 4 of 1,5-anhydro-D-fructose reductase OS=Homo sapiens GN=AKR1E2 - (AKCL2_HUMAN)	6.34	19.22	4	1	4	11	4.901E7	307	34.9	7.46
P25788-2	Isoform 2 of Proteasome subunit alpha type-3 OS=Homo sapiens GN=PSMA3 - (PSA3_HUMAN)	6.33	31.85	4	1	5	8	5.912E7	248	27.6	5.33
Q8N0W0	Seven transmembrane helix receptor OS=Homo sapiens PE=2 SV=1 - (Q8N0W0_HUMAN)	6.30	4.64	1	1	1	6	5.161E6	194	22.0	8.81
E9PKV2	39S ribosomal protein L17, mitochondrial (Fragment) OS=Homo sapiens GN=MRPL17 PE=4 SV=1 - (E9PKV2_HUMAN)	6.25	20.42	2	1	3	14	2.490E7	142	16.4	10.48
Q5XUX1-3	Isoform 3 of F-box/WD repeat-containing protein 9 OS=Homo sapiens GN=FBXW9 - (FBXW9_HUMAN)	6.25	9.61	3	1	6	14	1.718E9	458	50.7	6.48
H7C0Q6	Tripartite motif-containing protein 4 (Fragment) OS=Homo sapiens GN=TRIM4 PE=4 SV=1 - (H7C0Q6_HUMAN)	6.24	10.89	6	1	2	3	1.022E8	202	23.5	7.44
P00491	Purine nucleoside phosphorylase OS=Homo sapiens GN=PNP PE=1 SV=2 - (PNPH_HUMAN)	6.24	14.19	5	2	4	8	8.425E6	289	32.1	6.95
Q8WX93	Palladin OS=Homo sapiens GN=PALLD PE=1 SV=3 - (PALLD_HUMAN)	6.23	3.69	13	2	5	5	1.532E8	1383	150.5	7.09
B1Q3B3	Ferritin (Fragment) OS=Homo sapiens GN=FTL PE=3 SV=1 - (B1Q3B3_HUMAN)	6.18	45.65	5	2	3	5	4.095E7	92	10.3	5.99
B4DXN6	cDNA FLJ53461, highly similar to Eukaryotic translation initiation factor 3 subunit 9 OS=Homo sapiens PE=2 SV=1 - (B4DXN6_HUMAN)	6.16	6.05	7	1	5	6	1.972E7	661	77.2	5.92
Q5U676	B3GAT3 protein (Fragment) OS=Homo sapiens GN=B3GAT3 PE=2 SV=1 - (Q5U676_HUMAN)	6.15	13.49	6	1	4	7	1.076E8	341	37.8	8.62
O75531	Barrier-to-autointegration factor OS=Homo sapiens GN=BANF1 PE=1 SV=1 - (BAF_HUMAN)	6.15	51.69	2	2	3	6	2.603E7	89	10.1	6.09
B4DL14	ATP synthase gamma chain OS=Homo sapiens GN=ATP5C1 PE=2 SV=1 - (B4DL14_HUMAN)	6.15	16.00	4	1	3	6	1.227E8	250	27.5	7.42
O95373	Importin-7 OS=Homo sapiens GN=IPO7 PE=1 SV=1 - (IPO7_HUMAN)	6.12	4.62	1	1	5	6	3.750E7	1038	119.4	4.82
Q6WCQ1-3	Isoform 3 of Myosin phosphatase Rho-interacting protein OS=Homo sapiens GN=MPRIP - (MPRIP_HUMAN)	6.12	7.90	4	1	7	20	2.736E9	1000	114.0	6.35
P12273	Prolactin-inducible protein OS=Homo sapiens GN=PIP PE=1 SV=1 - (PIP_HUMAN)	6.10	18.49	1	1	2	2	1.685E6	146	16.6	8.05
Q92945-2	Isoform 2 of Far upstream element-binding protein 2 OS=Homo sapiens GN=KHSRP - (FUBP2_HUMAN)	6.07	7.32	2	1	6	7	2.142E8	710	72.9	7.90
B7Z6L2	cDNA FLJ54156, highly similar to ADP-ribosylation factor GTPase-activating protein 3 OS=Homo sapiens PE=2 SV=1 - (B7Z6L2_HUMAN)	6.05	13.54	5	1	5	6	1.270E7	443	48.9	7.66
Q9Y2U5	Mitogen-activated protein kinase kinase kinase 2 OS=Homo sapiens GN=MAP3K2 PE=1 SV=2 - (M3K2_HUMAN)	5.99	8.08	2	1	5	30	4.678E8	619	69.7	8.00
Q9Y613	FH1/FH2 domain-containing protein 1 OS=Homo sapiens GN=FHOD1 PE=1 SV=3 - (FHOD1_HUMAN)	5.99	9.97	2	1	10	11	2.191E8	1164	126.5	6.39
P20290-2	Isoform 2 of Transcription factor BTF3 OS=Homo sapiens GN=BTF3 - (BTF3_HUMAN)	5.98	27.16	8	1	5	7	3.493E7	162	17.7	7.50
Q15751	Probable E3 ubiquitin-protein ligase HERC1 OS=Homo sapiens GN=HERC1 PE=1 SV=2 - (HERC1_HUMAN)	5.97	6.11	1	1	23	45	3.968E8	4861	531.9	6.04
A8KAP9	cDNA FLJ78448, highly similar to Homo sapiens argininosuccinate synthetase (ASS), transcript variant 1, mRNA OS=Homo sapiens PE=2 SV=1 - (A8KAP9_HUMAN)	5.96	6.80	4	1	3	5	3.264E8	412	46.5	8.03
P54920	Alpha-soluble NSF attachment protein OS=Homo sapiens GN=NAPA PE=1 SV=3 - (SNAA_HUMAN)	5.95	15.93	6	1	5	11	9.394E7	295	33.2	5.36
P62314	Small nuclear ribonucleoprotein Sm D1 OS=Homo sapiens GN=SNRPD1 PE=1 SV=1 - (SMD1_HUMAN)	5.92	27.73	2	2	2	2	2.959E7	119	13.3	11.56
Q14247-2	Isoform 2 of Src substrate cortactin OS=Homo sapiens GN=CTTN - (SRC8_HUMAN)	5.89	9.46	6	1	6	7	9.171E7	634	70.9	6.64
D6RA82	Annexin OS=Homo sapiens GN=ANXA3 PE=3 SV=1 - (D6RA82_HUMAN)	5.86	25.00	2	1	6	6	2.059E8	284	32.1	5.94
P04632	Calpain small subunit 1 OS=Homo sapiens GN=CAPNS1 PE=1 SV=1 - (CPNS1_HUMAN)	5.81	17.16	1	1	4	6	2.515E7	268	28.3	5.20
Q92508	Piezo-type mechanosensitive ion channel component 1 OS=Homo sapiens GN=PIEZO1 PE=1 SV=4 - (PIEZ1_HUMAN)	5.78	4.96	2	1	11	16	7.843E8	2521	286.6	7.47
Q15257-3	Isoform 3 of Serine/threonine-protein phosphatase 2A activator OS=Homo sapiens GN=PPP2R4 - (PTPA_HUMAN)	5.77	12.24	1	1	4	7	2.442E7	294	33.4	6.29
P84090	Enhancer of rudimentary homolog OS=Homo sapiens GN=ERH PE=1 SV=1 - (ERH_HUMAN)	5.76	23.08	1	1	3	5	3.088E7	104	12.3	5.92
B4DP21	Prostaglandin E synthase 3 OS=Homo sapiens GN=PTGES3 PE=2 SV=1 - (B4DP21_HUMAN)	5.75	28.46	5	2	3	3	6.407E7	130	14.9	4.77
P15374	Ubiquitin carboxyl-terminal hydrolase isozyme L3 OS=Homo sapiens GN=UCHL3 PE=1 SV=1 - (UCHL3_HUMAN)	5.73	13.91	1	1	2	2	1.052E8	230	26.2	4.92
A8MXP9	Matrin-3 OS=Homo sapiens GN=MATR3 PE=4 SV=1 - (A8MXP9_HUMAN)	5.71	10.84	16	1	12	26	9.416E8	895	99.9	6.04
Q15102	Platelet-activating factor acetylhydrolase IB subunit gamma OS=Homo sapiens GN=PAFAH1B3 PE=1 SV=1 - (PA1B3_HUMAN)	5.70	3.90	1	1	1	2	3.411E7	231	25.7	6.84
Q96M91	Coiled-coil domain-containing protein 11 OS=Homo sapiens GN=CCDC11 PE=2 SV=2 - (CCD11_HUMAN)	5.70	22.37	1	1	10	12	1.365E8	514	61.8	8.90
P62861	40S ribosomal protein S30 OS=Homo sapiens GN=FAU PE=1 SV=1 - (RS30_HUMAN)	5.68	50.85	2	1	4	7	2.674E8	59	6.6	12.15
B7Z9G1	cDNA, FLJ78825 OS=Homo sapiens PE=4 SV=1 - (B7Z9G1_HUMAN)	5.63	31.33	4	1	2	8	2.428E7	83	9.3	9.60
E9PN17	ATP synthase subunit g, mitochondrial OS=Homo sapiens GN=ATP5L PE=4 SV=1 - (E9PN17_HUMAN)	5.62	31.58	2	1	2	3	6.921E7	76	8.4	10.29
A0AVI2	Fer-1-like protein 5 OS=Homo sapiens GN=FER1L5 PE=2 SV=2 - (FR1L5_HUMAN)	5.60	5.02	3	1	10	17	4.572E8	2093	241.8	7.96
E9PKS9	Trafficking protein particle complex subunit 4 OS=Homo sapiens GN=TRAPPC4 PE=4 SV=1 - (E9PKS9_HUMAN)	5.58	13.49	5	1	2	7	2.831E7	126	14.1	6.11
Q5EFE6	Anti-RhD monoclonal T125 kappa light chain OS=Homo sapiens PE=2 SV=1 - (Q5EFE6_HUMAN)	5.55	19.66	10	2	3	4	2.950E7	234	25.7	8.43
B4DL80	Cell division cycle protein 27 homolog OS=Homo sapiens GN=CDC27 PE=2 SV=1 - (B4DL80_HUMAN)	5.55	6.55	7	1	5	13	1.210E9	763	84.8	7.31
B4DJ44	cDNA FLJ54716, highly similar to Target of Myb protein 1 OS=Homo sapiens PE=2 SV=1 - (B4DJ44_HUMAN)	5.54	10.35	12	1	4	5	8.756E7	454	49.5	5.08
Q13946-2	Isoform PDE7A2 of High affinity cAMP-specific 3',5'-cyclic phosphodiesterase 7A OS=Homo sapiens GN=PDE7A - (PDE7A_HUMAN)	5.54	5.48	2	1	4	5	1.420E8	456	52.7	7.50
Q7Z3Z4	Piwi-like protein 4 OS=Homo sapiens GN=PIWIL4 PE=2 SV=2 - (PIWL4_HUMAN)	5.51	1.64	1	1	2	9	2.271E8	852	96.5	8.88
C9JGI3	Thymidine phosphorylase (Fragment) OS=Homo sapiens GN=TYMP PE=4 SV=1 - (C9JGI3_HUMAN)	5.49	14.38	4	1	5	8	1.113E8	445	46.1	5.52
P58546	Myotrophin OS=Homo sapiens GN=MTPN PE=1 SV=2 - (MTPN_HUMAN)	5.48	22.03	2	1	2	2	2.537E7	118	12.9	5.52
P22531	Small proline-rich protein 2E OS=Homo sapiens GN=SPRR2E PE=2 SV=2 - (SPR2E_HUMAN)	5.48	43.06	8	2	2	3	3.847E7	72	7.8	8.31
B4DV28	cDNA FLJ54170, highly similar to Cytosolic nonspecific dipeptidase OS=Homo sapiens PE=2 SV=1 - (B4DV28_HUMAN)	5.47	11.88	3	1	5	5	4.252E7	463	51.5	6.43
B2R4D8	60S ribosomal protein L27 OS=Homo sapiens PE=2 SV=1 - (B2R4D8_HUMAN)	5.44	21.32	3	1	3	5	7.718E7	136	15.8	10.56
Q06323	Proteasome activator complex subunit 1 OS=Homo sapiens GN=PSME1 PE=1 SV=1 - (PSME1_HUMAN)	5.44	18.88	3	1	4	4	8.384E7	249	28.7	6.02
P49761	Dual specificity protein kinase CLK3 OS=Homo sapiens GN=CLK3 PE=1 SV=3 - (CLK3_HUMAN)	5.40	15.05	12	1	8	10	8.927E7	638	73.5	9.92
Q96JS4	Cerebral protein-3 OS=Homo sapiens GN=hucep-3 PE=2 SV=1 - (Q96JS4_HUMAN)	5.39	4.22	1	1	1	3	1.314E8	166	18.0	9.00
A6NMP4	Uncharacterized PRO1102-like protein LOC100132418 OS=Homo sapiens PE=4 SV=1 - (YJ010_HUMAN)	5.38	25.30	2	1	2	11	3.591E8	83	9.8	9.85
G3V0E5	Transferrin receptor (P90, CD71), isoform CRA_c OS=Homo sapiens GN=TFRC PE=4 SV=1 - (G3V0E5_HUMAN)	5.37	10.31	3	1	6	8	1.708E8	679	75.9	6.81
Q14730	La 4.1 protein (Fragment) OS=Homo sapiens PE=2 SV=1 - (Q14730_HUMAN)	5.36	11.82	4	1	5	13	5.303E7	296	33.7	7.88
B0QY59	GTP binding protein 1 (Fragment) OS=Homo sapiens GN=GTPBP1 PE=4 SV=1 - (B0QY59_HUMAN)	5.35	10.89	3	1	5	20	4.374E8	349	37.8	10.56
G5EA35	Caspase recruitment domain-containing protein 18 OS=Homo sapiens GN=CARD18 PE=4 SV=1 - (G5EA35_HUMAN)	5.32	17.65	2	1	1	2	6.443E7	51	5.7	7.88
Q15834	Coiled-coil domain-containing protein 85B OS=Homo sapiens GN=CCDC85B PE=1 SV=2 - (CC85B_HUMAN)	5.31	19.80	1	1	4	9	1.736E8	202	22.1	5.08
P49411	Elongation factor Tu, mitochondrial OS=Homo sapiens GN=TUFM PE=1 SV=2 - (EFTU_HUMAN)	5.31	9.51	1	2	3	4	3.354E7	452	49.5	7.61
H7C2W1	D-beta-hydroxybutyrate dehydrogenase, mitochondrial (Fragment) OS=Homo sapiens GN=BDH1 PE=4 SV=1 - (H7C2W1_HUMAN)	5.30	27.13	1	1	3	6	4.341E7	129	14.6	8.75
B2R7R2	cDNA, FLJ93566, highly similar to Homo sapiens RWD domain containing 2 (RWDD2), mRNA OS=Homo sapiens PE=2 SV=1 - (B2R7R2_HUMAN)	5.23	6.16	4	1	2	4	3.730E7	292	33.9	6.47
H0YCD4	T-lymphoma invasion and metastasis-inducing protein 2 OS=Homo sapiens GN=TIAM2 PE=4 SV=1 - (H0YCD4_HUMAN)	5.23	4.42	11	1	9	17	1.057E8	1947	218.5	7.09
E7EUJ8	Creatine kinase B-type OS=Homo sapiens GN=CKB PE=3 SV=2 - (E7EUJ8_HUMAN)	5.17	8.38	2	1	2	2	9.435E7	346	38.7	5.48
A8K4E4	cDNA FLJ75360, highly similar to Homo sapiens egf-like module containing, mucin-like, hormone receptor-like 3 (EMR3), transcript variant 1, mRNA OS=Homo sapiens PE=2 SV=1 - (A8K4E4_HUMAN)	5.13	4.60	7	1	3	6	7.479E7	652	72.6	8.05
E7EQV9	Ribosomal protein L15 (Fragment) OS=Homo sapiens GN=RPL15 PE=3 SV=1 - (E7EQV9_HUMAN)	5.13	37.36	8	2	6	6	8.163E7	174	20.5	11.28
Q96G46-2	Isoform 2 of tRNA-dihydrouridine(47) synthase (NAD(P)(+))-like OS=Homo sapiens GN=DUS3L - (DUS3L_HUMAN)	5.12	10.58	3	1	6	7	1.291E8	482	52.7	8.37
Q08554-2	Isoform 1B of Desmocollin-1 OS=Homo sapiens GN=DSC1 - (DSC1_HUMAN)	5.12	11.19	2	4	8	8	5.836E7	840	93.8	5.53
B7Z505	Protein NDRG1 OS=Homo sapiens GN=NDRG1 PE=2 SV=1 - (B7Z505_HUMAN)	5.08	24.32	15	2	4	5	1.155E7	222	23.9	7.56
H7C2M7	Armadillo repeat-containing protein 10 (Fragment) OS=Homo sapiens GN=ARMC10 PE=4 SV=1 - (H7C2M7_HUMAN)	5.07	9.19	9	1	1	2	2.012E7	185	20.6	7.01
O43598-2	Isoform 2 of Deoxyribonucleoside 5'-monophosphate N-glycosidase OS=Homo sapiens GN=RCL - (RCL_HUMAN)	5.06	22.97	3	1	3	5	2.679E7	148	16.2	6.79
P60866	40S ribosomal protein S20 OS=Homo sapiens GN=RPS20 PE=1 SV=1 - (RS20_HUMAN)	5.05	60.50	6	5	8	10	2.589E8	119	13.4	9.94
B7Z254	Protein disulfide-isomerase A6 OS=Homo sapiens GN=PDIA6 PE=2 SV=1 - (B7Z254_HUMAN)	5.03	8.70	7	2	4	6	5.954E7	437	47.8	5.08
A4D1I1	Similar to ribosomal protein S3a; 40S ribosomal protein S3a; v-fos transformation effector protein 1 OS=Homo sapiens GN=LOC402287 PE=4 SV=1 - (A4D1I1_HUMAN)	5.02	37.82	1	1	6	10	4.606E8	119	13.2	9.83
Q5D862	Filaggrin-2 OS=Homo sapiens GN=FLG2 PE=1 SV=1 - (FILA2_HUMAN)	4.98	2.05	1	1	5	8	3.330E7	2391	247.9	8.31
E9PR17	CD59 glycoprotein OS=Homo sapiens GN=CD59 PE=4 SV=1 - (E9PR17_HUMAN)	4.96	30.00	4	3	4	5	3.969E7	130	14.5	7.77
E9PRQ6	Acetyl-CoA acetyltransferase, mitochondrial OS=Homo sapiens GN=ACAT1 PE=4 SV=1 - (E9PRQ6_HUMAN)	4.96	12.06	4	1	1	1	6.960E6	141	15.2	9.47
Q49AN9	SNRPG protein OS=Homo sapiens GN=SNRPG PE=4 SV=1 - (Q49AN9_HUMAN)	4.93	31.25	6	2	2	2	2.314E7	64	7.1	7.18
Q8IX95	Putative cutaneous T-cell lymphoma-associated antigen 3 OS=Homo sapiens GN=CTAGE3P PE=5 SV=1 - (CTGE3_HUMAN)	4.90	27.22	1	1	3	12	5.752E7	158	18.0	4.92
H0UI63	Zinc finger, NFX1-type containing 1, isoform CRA_b OS=Homo sapiens GN=ZNFX1 PE=4 SV=1 - (H0UI63_HUMAN)	4.89	8.71	3	1	13	18	2.748E8	1918	220.1	7.30
P17022-2	Isoform 2 of Zinc finger protein 18 OS=Homo sapiens GN=ZNF18 - (ZNF18_HUMAN)	4.87	8.21	3	1	2	5	6.953E7	548	62.2	5.97
Q2VEU1	Sulfurtransferase (Fragment) OS=Homo sapiens GN=MPST PE=2 SV=1 - (Q2VEU1_HUMAN)	4.82	13.64	7	1	3	5	4.276E7	198	22.3	7.18
H0YIB4	Serine/arginine-rich-splicing factor 9 (Fragment) OS=Homo sapiens GN=SRSF9 PE=4 SV=1 - (H0YIB4_HUMAN)	4.80	23.64	4	1	2	3	8.449E7	110	12.8	5.38
Q6WWJ7	DELTA3PRO-IL-18 OS=Homo sapiens GN=IL18 PE=2 SV=1 - (Q6WWJ7_HUMAN)	4.79	10.58	4	1	3	4	4.680E7	189	21.9	4.78
O43399-4	Isoform 4 of Tumor protein D54 OS=Homo sapiens GN=TPD52L2 - (TPD54_HUMAN)	4.78	28.00	9	1	4	4	1.063E8	200	21.4	5.99
P20962	Parathymosin OS=Homo sapiens GN=PTMS PE=1 SV=2 - (PTMS_HUMAN)	4.74	22.55	5	2	2	2	4.902E6	102	11.5	4.16
B7Z478	Proteasome (Prosome, macropain) subunit, beta type, 2, isoform CRA_b OS=Homo sapiens GN=PSMB2 PE=2 SV=1 - (B7Z478_HUMAN)	4.71	10.80	2	1	2	2	5.907E7	176	20.2	7.44
Q13151	Heterogeneous nuclear ribonucleoprotein A0 OS=Homo sapiens GN=HNRNPA0 PE=1 SV=1 - (ROA0_HUMAN)	4.64	7.21	1	1	2	4	1.757E7	305	30.8	9.29
E9PM69	26S protease regulatory subunit 6A OS=Homo sapiens GN=PSMC3 PE=3 SV=1 - (E9PM69_HUMAN)	4.64	10.83	8	1	4	21	4.022E8	397	44.3	5.05
Q6IPM2-2	Isoform 2 of IQ domain-containing protein E OS=Homo sapiens GN=IQCE - (IQCE_HUMAN)	4.63	10.63	16	2	6	14	1.809E8	630	70.3	8.82
B0V2L2	HLA-B associated transcript 1 (Fragment) OS=Homo sapiens GN=BAT1 PE=4 SV=1 - (B0V2L2_HUMAN)	4.62	11.42	15	1	4	10	1.499E8	324	37.9	8.95
Q5BKY2	EIF3H protein OS=Homo sapiens GN=EIF3H PE=2 SV=1 - (Q5BKY2_HUMAN)	4.57	8.60	11	1	2	2	6.975E6	349	39.6	6.39
P51148	Ras-related protein Rab-5C OS=Homo sapiens GN=RAB5C PE=1 SV=2 - (RAB5C_HUMAN)	4.54	6.94	2	1	2	8	5.179E7	216	23.5	8.41
Q9BUP0	EF-hand domain-containing protein D1 OS=Homo sapiens GN=EFHD1 PE=1 SV=1 - (EFHD1_HUMAN)	4.52	20.92	4	1	6	6	2.054E7	239	26.9	5.39
B3KRM2	Serine/threonine-protein phosphatase OS=Homo sapiens PE=2 SV=1 - (B3KRM2_HUMAN)	4.51	19.74	8	1	6	10	7.199E8	309	35.5	5.43
B4E1W7	cDNA FLJ59114, highly similar to Amyotrophic lateral sclerosis 2 chromosomalregion candidate gene 8 protein (Calcium-responsefactor) (CaRF) (Protein NYD-SP24) OS=Homo sapiens PE=2 SV=1 - (B4E1W7_HUMAN)	4.51	17.41	7	1	7	10	2.092E8	649	72.4	6.15
B2R9G9	cDNA, FLJ94385, highly similar to Homo sapiens cofactor required for Sp1 transcriptional activation, subunit 7, 70kDa (CRSP7), mRNA OS=Homo sapiens PE=2 SV=1 - (B2R9G9_HUMAN)	4.51	9.67	4	1	6	9	5.835E7	600	65.3	9.23
Q5J7W2	Migration-inducing gene 9 protein OS=Homo sapiens PE=2 SV=1 - (Q5J7W2_HUMAN)	4.49	26.14	2	1	2	2	9.107E6	88	9.6	5.17
B5BU41	Calcium/calmodulin-dependent protein kinase I OS=Homo sapiens GN=CAMK1 PE=2 SV=1 - (B5BU41_HUMAN)	4.47	11.89	2	1	3	9	2.878E7	370	41.3	5.35
P42694	Probable helicase with zinc finger domain OS=Homo sapiens GN=HELZ PE=1 SV=2 - (HELZ_HUMAN)	4.47	4.84	4	1	7	10	2.164E8	1942	218.8	7.42
O00483	NADH dehydrogenase (ubiquinone) 1 alpha subcomplex subunit 4 OS=Homo sapiens GN=NDUFA4 PE=1 SV=1 - (NDUA4_HUMAN)	4.46	24.69	1	2	2	2	1.360E7	81	9.4	9.38
P53396-2	Isoform 2 of ATP-citrate synthase OS=Homo sapiens GN=ACLY - (ACLY_HUMAN)	4.43	4.22	6	1	5	5	3.279E7	1091	119.7	7.33
Q53HL1	Myosin regulatory light chain MRCL3 variant (Fragment) OS=Homo sapiens PE=2 SV=1 - (Q53HL1_HUMAN)	4.41	15.20	3	1	3	7	7.673E7	171	19.8	4.84
O15547	P2X purinoceptor 6 OS=Homo sapiens GN=P2RX6 PE=1 SV=2 - (P2RX6_HUMAN)	4.39	7.26	6	1	3	5	1.169E7	441	48.8	7.58
B4DLW8	cDNA FLJ59339, highly similar to Probable ATP-dependent RNA helicase DDX5 (EC 3.6.1.-) OS=Homo sapiens PE=2 SV=1 - (B4DLW8_HUMAN)	4.36	11.59	6	1	6	17	4.118E8	535	60.5	9.07
Q96RX5	NADH ubiquinone oxidoreductase PDSW subunit (RH 16p13.3) (Fragment) OS=Homo sapiens GN=NDUFB10 PE=2 SV=1 - (Q96RX5_HUMAN)	4.31	51.16	5	1	2	2	1.961E7	43	5.1	6.60
Q53RT3	Retroviral-like aspartic protease 1 OS=Homo sapiens GN=ASPRV1 PE=1 SV=1 - (APRV1_HUMAN)	4.30	7.00	1	1	2	12	8.547E7	343	37.0	5.44
Q59EW3	RAB18, member RAS oncogene family variant (Fragment) OS=Homo sapiens PE=2 SV=1 - (Q59EW3_HUMAN)	4.29	30.71	9	1	3	6	2.006E7	127	14.4	5.33
B7Z8R7	Nuclear receptor subfamily 1 group I member 3 OS=Homo sapiens GN=NR1I3 PE=2 SV=1 - (B7Z8R7_HUMAN)	4.28	11.34	30	1	2	8	2.992E8	238	26.8	8.37
Q9H6X5-2	Isoform 2 of Uncharacterized protein C19orf44 OS=Homo sapiens GN=C19orf44 - (CS044_HUMAN)	4.26	10.36	2	1	4	11	7.768E8	618	66.3	5.97
Q9NY47-4	Isoform 4 of Voltage-dependent calcium channel subunit alpha-2/delta-2 OS=Homo sapiens GN=CACNA2D2 - (CA2D2_HUMAN)	4.26	4.55	9	1	4	4	1.086E8	1076	122.0	5.38
Q2F838	Eukaryotic translation elongation factor 1 gamma (Fragment) OS=Homo sapiens PE=2 SV=1 - (Q2F838_HUMAN)	4.25	14.94	1	1	1	2	7.972E7	87	9.6	6.77
B4DM22	cDNA FLJ53357, highly similar to 26S proteasome non-ATPase regulatory subunit 2 OS=Homo sapiens PE=2 SV=1 - (B4DM22_HUMAN)	4.24	6.11	11	2	6	8	2.030E8	900	99.3	5.16
B3VRC5	5-hydroxytryptamine (Serotonin) receptor 2B (Fragment) OS=Homo sapiens GN=HTR2B PE=4 SV=1 - (B3VRC5_HUMAN)	4.24	7.09	2	1	3	9	8.370E7	296	33.6	9.52
H0YN18	Proteasome subunit alpha type-4 OS=Homo sapiens GN=PSMA4 PE=4 SV=1 - (H0YN18_HUMAN)	4.23	13.91	11	1	3	4	4.267E7	230	26.3	8.32
Q6T4R5	Nance-Horan syndrome protein OS=Homo sapiens GN=NHS PE=1 SV=1 - (NHS_HUMAN)	4.22	5.71	7	1	10	15	4.301E8	1630	176.6	6.79
O94929-2	Isoform 2 of Actin-binding LIM protein 3 OS=Homo sapiens GN=ABLIM3 - (ABLM3_HUMAN)	4.22	7.17	2	1	3	3	1.057E7	544	61.5	8.48
G3V4P8	Glia maturation factor beta (Fragment) OS=Homo sapiens GN=GMFB PE=4 SV=1 - (G3V4P8_HUMAN)	4.21	18.67	1	1	2	2	1.361E7	150	17.5	5.31
Q59H49	Polypyrimidine tract-binding protein 1 isoform c variant (Fragment) OS=Homo sapiens PE=2 SV=1 - (Q59H49_HUMAN)	4.21	13.98	4	1	4	4	1.465E7	329	35.5	8.98
D3DUP2	WNK lysine deficient protein kinase 1, isoform CRA_d OS=Homo sapiens GN=WNK1 PE=4 SV=1 - (D3DUP2_HUMAN)	4.21	3.99	11	1	9	10	8.474E8	2107	222.6	6.43
F5GX11	Proteasome subunit alpha type-1 OS=Homo sapiens GN=PSMA1 PE=4 SV=1 - (F5GX11_HUMAN)	4.20	12.18	6	2	3	3	4.237E7	238	26.5	6.73
H7BZW3	Voltage-dependent calcium channel subunit alpha-2-3 OS=Homo sapiens GN=CACNA2D3 PE=4 SV=1 - (H7BZW3_HUMAN)	4.18	5.21	1	1	4	26	6.124E8	518	59.4	5.86
B4E0J3	cDNA FLJ58965, highly similar to Nonspecific lipid-transfer protein (EC 2.3.1.176) OS=Homo sapiens PE=2 SV=1 - (B4E0J3_HUMAN)	4.16	6.61	10	1	1	2	2.544E7	121	13.0	5.57
B7Z6B3	cDNA FLJ53094, highly similar to Receptor expression-enhancing protein 5 OS=Homo sapiens PE=2 SV=1 - (B7Z6B3_HUMAN)	4.15	5.13	4	1	1	2	8.275E6	156	17.7	8.65
G3V117	Enhancer of yellow 2 homolog (Drosophila), isoform CRA_c OS=Homo sapiens GN=ENY2 PE=4 SV=1 - (G3V117_HUMAN)	4.14	45.83	3	1	4	4	8.829E7	96	11.0	9.13
O00151	PDZ and LIM domain protein 1 OS=Homo sapiens GN=PDLIM1 PE=1 SV=4 - (PDLI1_HUMAN)	4.13	10.03	1	1	2	2	1.761E7	329	36.0	7.02
Q5HYK3-2	Isoform 2 of 2-methoxy-6-polyprenyl-1,4-benzoquinol methylase, mitochondrial OS=Homo sapiens GN=COQ5 - (COQ5_HUMAN)	4.13	15.04	5	1	3	6	1.774E8	246	27.9	9.14
P19623	Spermidine synthase OS=Homo sapiens GN=SRM PE=1 SV=1 - (SPEE_HUMAN)	4.13	13.58	1	1	3	3	2.862E7	302	33.8	5.49
Q63HQ8	Putative uncharacterized protein DKFZp686M21196 OS=Homo sapiens GN=DKFZp686M21196 PE=2 SV=1 - (Q63HQ8_HUMAN)	4.12	17.36	5	2	7	8	5.992E7	530	58.5	9.82
Q8NHP1	Aflatoxin B1 aldehyde reductase member 4 OS=Homo sapiens GN=AKR7L PE=2 SV=6 - (ARK74_HUMAN)	4.10	8.46	3	1	2	2	2.009E8	331	36.9	6.76
Q8N2H2	cDNA FLJ90785 fis, clone THYRO1001457, moderately similar to H.sapiens protein kinase C mu OS=Homo sapiens PE=2 SV=1 - (Q8N2H2_HUMAN)	4.10	4.72	4	1	3	4	8.002E5	721	80.0	7.30
B4DMK9	cDNA FLJ56112, highly similar to Dihydrolipoyl dehydrogenase, mitochondrial (EC 1.8.1.4) OS=Homo sapiens PE=2 SV=1 - (B4DMK9_HUMAN)	4.07	8.85	7	1	5	6	2.167E7	486	51.8	7.96
B1ALC0	Actin-related protein 2/3 complex subunit 5 OS=Homo sapiens GN=ARPC5 PE=3 SV=1 - (B1ALC0_HUMAN)	4.06	26.67	4	1	5	6	1.405E8	135	14.8	5.06
Q8N7R7-3	Isoform 3 of Cyclin-Y-like protein 1 OS=Homo sapiens GN=CCNYL1 - (CCYL1_HUMAN)	4.06	26.30	3	1	6	9	5.777E7	289	33.4	6.93
E5RFM1	Uncharacterized protein OS=Homo sapiens PE=4 SV=1 - (E5RFM1_HUMAN)	4.04	12.50	2	1	1	16	1.031E8	64	7.2	9.85
D7PBN3	ESRP1/RAF1 fusion protein OS=Homo sapiens PE=2 SV=1 - (D7PBN3_HUMAN)	4.02	7.54	14	1	10	15	2.045E8	1061	118.5	8.22
F8VY04	Adenylate kinase 2, mitochondrial OS=Homo sapiens GN=AK2 PE=3 SV=1 - (F8VY04_HUMAN)	4.01	17.37	6	1	2	2	5.758E7	190	21.2	6.95
Q5QPM0	RNA binding protein, autoantigenic (HnRNP-associated with lethal yellow homolog (Mouse)) (Fragment) OS=Homo sapiens GN=RALY PE=2 SV=1 - (Q5QPM0_HUMAN)	3.98	31.40	6	1	6	7	3.544E8	172	18.7	10.02
B7Z1R5	V-type proton ATPase catalytic subunit A OS=Homo sapiens GN=ATP6V1A PE=2 SV=1 - (B7Z1R5_HUMAN)	3.95	6.85	4	2	4	4	5.189E7	584	64.7	5.66
Q96EY7	Pentatricopeptide repeat-containing protein 3, mitochondrial OS=Homo sapiens GN=PTCD3 PE=1 SV=3 - (PTCD3_HUMAN)	3.94	3.19	5	1	3	3	1.867E7	689	78.5	6.42
Q9Y349	KIAA0232 protein OS=Homo sapiens GN=KIAA0232 PE=2 SV=1 - (Q9Y349_HUMAN)	3.93	23.91	1	1	2	3	2.534E7	138	15.7	9.58
Q53F37	SAR1a gene homolog 2 variant (Fragment) OS=Homo sapiens PE=2 SV=1 - (Q53F37_HUMAN)	3.92	20.71	10	1	4	5	4.246E7	198	22.4	6.11
O95336	6-phosphogluconolactonase OS=Homo sapiens GN=PGLS PE=1 SV=2 - (6PGL_HUMAN)	3.92	11.24	1	1	2	2	1.924E7	258	27.5	6.05
H0Y3H1	Toll-interacting protein OS=Homo sapiens GN=TOLLIP PE=4 SV=1 - (H0Y3H1_HUMAN)	3.91	19.60	9	1	9	12	3.405E8	352	38.9	8.46
Q9P2F5-2	Isoform 2 of Storkhead-box protein 2 OS=Homo sapiens GN=STOX2 - (STOX2_HUMAN)	3.91	2.90	2	1	3	10	2.712E7	863	96.1	8.35
Q9NRS6-2	Isoform 2 of Sorting nexin-15 OS=Homo sapiens GN=SNX15 - (SNX15_HUMAN)	3.91	12.55	9	1	3	5	5.280E8	255	29.1	6.67
H0YLF3	Beta-2-microglobulin (Fragment) OS=Homo sapiens GN=B2M PE=3 SV=1 - (H0YLF3_HUMAN)	3.90	14.08	6	1	1	2	1.135E8	71	8.5	5.15
E5KNB2	DNA-apurinic or apyrimidinic site lyase 2 OS=Homo sapiens PE=4 SV=1 - (E5KNB2_HUMAN)	3.83	1.93	3	1	1	4	2.556E8	518	57.4	8.29
A8K6Y1	cDNA FLJ75526, highly similar to Homo sapiens proliferation-associated 2G4, 38kDa (PA2G4), mRNA (Fragment) OS=Homo sapiens PE=2 SV=1 - (A8K6Y1_HUMAN)	3.83	12.81	10	2	5	6	7.263E6	367	40.9	6.40
Q9NSA3	Beta-catenin-interacting protein 1 OS=Homo sapiens GN=CTNNBIP1 PE=1 SV=1 - (CNBP1_HUMAN)	3.83	38.27	1	1	3	3	9.824E7	81	9.2	5.41
C5IX07	Tumor-associated calcium signal transducer 2 OS=Homo sapiens GN=TACSTD2 PE=4 SV=1 - (C5IX07_HUMAN)	3.81	13.31	2	1	4	5	1.607E8	323	35.7	8.87
Q6ZUG2	cDNA FLJ43743 fis, clone TESTI2019042 OS=Homo sapiens PE=2 SV=1 - (Q6ZUG2_HUMAN)	3.79	11.26	1	1	3	5	6.458E7	293	33.1	10.56
B4DF39	cDNA FLJ59119, highly similar to Receptor expression-enhancing protein 6 OS=Homo sapiens PE=2 SV=1 - (B4DF39_HUMAN)	3.77	8.61	3	1	1	1	8.363E5	151	16.8	4.94
Q9H875	PRKR-interacting protein 1 OS=Homo sapiens GN=PRKRIP1 PE=1 SV=1 - (PKRI1_HUMAN)	3.74	10.87	1	1	2	3	3.322E8	184	21.0	9.79
P15121	Aldose reductase OS=Homo sapiens GN=AKR1B1 PE=1 SV=3 - (ALDR_HUMAN)	3.74	7.28	1	1	2	2	5.690E7	316	35.8	6.98
B1AH89	Tubulin tyrosine ligase-like family, member 12 OS=Homo sapiens GN=TTLL12 PE=4 SV=1 - (B1AH89_HUMAN)	3.71	5.50	2	1	5	9	2.121E7	636	73.5	5.54
Q69YN4-3	Isoform 3 of Protein virilizer homolog OS=Homo sapiens GN=KIAA1429 - (VIR_HUMAN)	3.70	2.62	4	1	4	16	4.061E7	1797	200.9	4.82
Q8IXI1	Mitochondrial Rho GTPase 2 OS=Homo sapiens GN=RHOT2 PE=1 SV=2 - (MIRO2_HUMAN)	3.69	3.40	1	1	3	6	4.285E7	618	68.1	5.86
F5H1H8	Activated RNA polymerase II transcriptional coactivator p15 OS=Homo sapiens GN=SUB1 PE=4 SV=1 - (F5H1H8_HUMAN)	3.69	16.26	3	1	2	3	3.306E7	123	14.0	9.73
P51178-2	Isoform 2 of 1-phosphatidylinositol 4,5-bisphosphate phosphodiesterase delta-1 OS=Homo sapiens GN=PLCD1 - (PLCD1_HUMAN)	3.69	11.58	6	1	9	11	4.999E7	777	88.1	7.23
E5KSX8	Mitochondrial transcription factor A OS=Homo sapiens PE=4 SV=1 - (E5KSX8_HUMAN)	3.69	18.29	4	1	4	6	5.179E7	246	29.1	9.72
Q9BU79	Transmembrane protein C7orf23 OS=Homo sapiens GN=C7orf23 PE=2 SV=1 - (CG023_HUMAN)	3.65	5.93	1	1	1	6	1.383E6	118	13.4	8.18
Q7LAX7	CKII beta binding protein 2 OS=Homo sapiens PE=2 SV=1 - (Q7LAX7_HUMAN)	3.63	15.32	2	1	3	5	2.070E7	124	14.5	9.61
H0Y8J7	Eukaryotic translation initiation factor 4E (Fragment) OS=Homo sapiens GN=EIF4E PE=3 SV=1 - (H0Y8J7_HUMAN)	3.62	11.61	7	2	2	2	2.475E7	155	18.5	6.55
Q8NGP8	Olfactory receptor 5M1 OS=Homo sapiens GN=OR5M1 PE=2 SV=1 - (OR5M1_HUMAN)	3.62	4.13	1	1	1	3	2.412E8	315	35.6	8.09
B7Z3W8	Transporter OS=Homo sapiens PE=2 SV=1 - (B7Z3W8_HUMAN)	3.56	3.30	5	1	2	4	5.727E8	637	70.7	7.55
Q9Y5Z4-2	Isoform 2 of Heme-binding protein 2 OS=Homo sapiens GN=HEBP2 - (HEBP2_HUMAN)	3.55	11.41	3	1	2	2	3.907E7	184	20.8	4.78
Q96L46	Calpain small subunit 2 OS=Homo sapiens GN=CAPNS2 PE=2 SV=2 - (CPNS2_HUMAN)	3.55	11.69	1	1	3	3	3.239E7	248	27.6	5.73
P35321	Cornifin-A OS=Homo sapiens GN=SPRR1A PE=1 SV=2 - (SPR1A_HUMAN)	3.55	17.98	3	1	1	2	1.864E7	89	9.9	8.48
Q9ULC4-2	Isoform 2 of Malignant T-cell-amplified sequence 1 OS=Homo sapiens GN=MCTS1 - (MCTS1_HUMAN)	3.53	22.49	3	1	4	4	1.141E8	169	19.2	8.25
C9JGI8	MAGUK p55 subfamily member 6 (Fragment) OS=Homo sapiens GN=MPP6 PE=4 SV=1 - (C9JGI8_HUMAN)	3.53	9.68	4	1	2	4	8.560E7	217	23.9	4.84
Q7Z739	YTH domain family protein 3 OS=Homo sapiens GN=YTHDF3 PE=1 SV=1 - (YTHD3_HUMAN)	3.51	6.32	10	1	5	7	6.989E7	585	63.8	9.04
B4E2I4	cDNA FLJ58227, highly similar to Glutamate--cysteine ligase catalytic subunit (EC 6.3.2.2) OS=Homo sapiens PE=2 SV=1 - (B4E2I4_HUMAN)	3.50	8.22	4	1	5	6	7.984E7	584	66.4	5.66
Q6NVY0	Calcyclin binding protein OS=Homo sapiens GN=CACYBP PE=2 SV=1 - (Q6NVY0_HUMAN)	3.50	16.23	4	1	5	7	1.118E8	228	26.2	7.87
P08621-2	Isoform 2 of U1 small nuclear ribonucleoprotein 70 kDa OS=Homo sapiens GN=SNRNP70 - (RU17_HUMAN)	3.48	6.54	4	1	4	5	5.651E7	428	50.6	9.89
A8K321	cDNA FLJ78524, highly similar to Homo sapiens SMILE protein (SMILE), mRNA OS=Homo sapiens PE=2 SV=1 - (A8K321_HUMAN)	3.45	11.01	4	1	10	13	1.225E8	899	102.0	8.84
B4DI45	cDNA FLJ56612, highly similar to Inner nuclear membrane protein Man1 OS=Homo sapiens PE=2 SV=1 - (B4DI45_HUMAN)	3.44	7.09	1	1	2	9	1.150E8	268	31.1	9.23
O95147	Dual specificity protein phosphatase 14 OS=Homo sapiens GN=DUSP14 PE=1 SV=1 - (DUS14_HUMAN)	3.43	10.61	1	1	1	1	3.437E6	198	22.2	9.57
Q8NAN7	Very low density lipoprotein receptor OS=Homo sapiens GN=VLDLR PE=2 SV=1 - (Q8NAN7_HUMAN)	3.42	8.78	5	1	6	18	2.784E8	752	82.8	4.91
F5H5Y2	Translocon-associated protein subunit alpha OS=Homo sapiens GN=SSR1 PE=4 SV=1 - (F5H5Y2_HUMAN)	3.40	7.72	8	1	3	4	2.789E7	259	29.4	4.72
Q99704-3	Isoform 3 of Docking protein 1 OS=Homo sapiens GN=DOK1 - (DOK1_HUMAN)	3.38	2.92	5	1	1	3	6.998E6	342	37.4	5.35
E9PKL8	NADH dehydrogenase (ubiquinone) iron-sulfur protein 3, mitochondrial (Fragment) OS=Homo sapiens GN=NDUFS3 PE=4 SV=1 - (E9PKL8_HUMAN)	3.33	34.62	6	1	2	4	2.049E8	78	8.6	8.03
P61960	Ubiquitin-fold modifier 1 OS=Homo sapiens GN=UFM1 PE=1 SV=1 - (UFM1_HUMAN)	3.32	25.88	3	1	2	2	3.969E6	85	9.1	9.31
O75691	Small subunit processome component 20 homolog OS=Homo sapiens GN=UTP20 PE=1 SV=3 - (UTP20_HUMAN)	3.29	3.84	1	1	9	10	4.258E8	2785	318.2	7.39
Q9Y3C4-2	Isoform 2 of TP53RK-binding protein OS=Homo sapiens GN=TPRKB - (TPRKB_HUMAN)	3.22	12.68	4	1	1	1		142	16.1	6.30
P60985-2	Isoform 2 of Keratinocyte differentiation-associated protein OS=Homo sapiens GN=KRTDAP - (KTDAP_HUMAN)	3.18	14.12	2	1	1	1	3.460E6	85	9.4	6.54
H7C125	Ras-related protein Rab-2A (Fragment) OS=Homo sapiens GN=RAB2A PE=4 SV=1 - (H7C125_HUMAN)	3.16	13.46	4	1	1	1	1.840E7	104	12.1	5.08
P24534	Elongation factor 1-beta OS=Homo sapiens GN=EEF1B2 PE=1 SV=3 - (EF1B_HUMAN)	3.14	17.33	2	1	4	6	3.735E7	225	24.7	4.67
B2R4N3	cDNA, FLJ92155, highly similar to Homo sapiens ubiquitin-like 5 (UBL5), mRNA OS=Homo sapiens PE=4 SV=1 - (B2R4N3_HUMAN)	3.09	24.66	2	1	3	3	5.694E6	73	8.5	7.28
B8ZZK4	60S ribosomal protein L31 OS=Homo sapiens GN=RPL31 PE=4 SV=1 - (B8ZZK4_HUMAN)	3.04	60.76	9	2	8	11	1.717E8	79	9.0	10.89
E5RJ48	Acyl-protein thioesterase 1 (Fragment) OS=Homo sapiens GN=LYPLA1 PE=4 SV=1 - (E5RJ48_HUMAN)	3.02	15.56	6	1	1	1	1.563E7	90	9.6	4.89
O00487	26S proteasome non-ATPase regulatory subunit 14 OS=Homo sapiens GN=PSMD14 PE=1 SV=1 - (PSDE_HUMAN)	3.01	15.81	2	1	4	4	9.003E7	310	34.6	6.52
Q9BTV4	Transmembrane protein 43 OS=Homo sapiens GN=TMEM43 PE=1 SV=1 - (TMM43_HUMAN)	2.95	6.75	2	1	3	3	1.273E7	400	44.8	8.13
G3V5F2	Peptidyl-prolyl cis-trans isomerase FKBP3 OS=Homo sapiens GN=FKBP3 PE=4 SV=1 - (G3V5F2_HUMAN)	2.93	23.08	1	1	1	1	4.174E7	65	7.2	8.57
H0YHG0	Uncharacterized protein (Fragment) OS=Homo sapiens PE=4 SV=1 - (H0YHG0_HUMAN)	2.93	8.22	5	1	5	12	5.673E7	523	59.1	8.59
Q9Y6D5	Brefeldin A-inhibited guanine nucleotide-exchange protein 2 OS=Homo sapiens GN=ARFGEF2 PE=1 SV=3 - (BIG2_HUMAN)	2.80	6.05	3	1	10	15	6.845E8	1785	201.9	6.33
A8K4W2	cDNA FLJ78635, highly similar to Homo sapiens ATP synthase, H+ transporting, mitochondrial F0 complex, subunit b, isoform 1 (ATP5F1), transcript variant 1, mRNA OS=Homo sapiens PE=2 SV=1 - (A8K4W2_HUMAN)	2.79	18.36	3	1	3	5	1.587E8	256	28.9	9.36
H7BYG3	Leucine-rich repeats and immunoglobulin-like domains protein 1 OS=Homo sapiens GN=LRIG1 PE=4 SV=1 - (H7BYG3_HUMAN)	2.72	2.71	2	1	3	3	3.607E7	996	109.0	6.80
Q96LZ7-4	Isoform 4 of Regulator of microtubule dynamics protein 2 OS=Homo sapiens GN=FAM82A1 - (RMD2_HUMAN)	2.65	13.96	5	1	3	24	4.832E8	265	30.9	7.43
Q3LI83	Keratin-associated protein 24-1 OS=Homo sapiens GN=KRTAP24-1 PE=1 SV=1 - (KR241_HUMAN)	2.62	8.66	1	1	2	2	4.196E7	254	27.7	8.29
B4DQZ2	cDNA FLJ50621, highly similar to DNA (cytosine-5)-methyltransferase-like protein 2 OS=Homo sapiens PE=2 SV=1 - (B4DQZ2_HUMAN)	2.54	8.39	7	1	2	4	1.732E8	310	35.6	7.40
Q9Y6K8-3	Isoform 3 of Adenylate kinase isoenzyme 5 OS=Homo sapiens GN=AK5 - (KAD5_HUMAN)	2.54	9.70	4	1	6	9	7.718E7	536	60.3	5.08
B4DPD5	cDNA FLJ56307, highly similar to Ubiquitin thioesterase protein OTUB1 (EC 3.4.-.-) OS=Homo sapiens PE=2 SV=1 - (B4DPD5_HUMAN)	2.52	8.12	5	1	2	3	5.476E7	308	35.2	5.59
B4DFL3	cDNA FLJ56661, highly similar to Proteasome subunit beta type 4 (EC 3.4.25.1) OS=Homo sapiens PE=2 SV=1 - (B4DFL3_HUMAN)	2.48	4.79	2	1	1	3	2.531E7	167	18.3	9.35
B4DEE5	cDNA FLJ60230, highly similar to Arsenical pump-driving ATPase (EC 3.6.3.16) OS=Homo sapiens PE=2 SV=1 - (B4DEE5_HUMAN)	2.48	5.94	2	1	2	2	1.597E7	320	35.8	4.84
A6NHJ4	Zinc finger protein 860 OS=Homo sapiens GN=ZNF860 PE=2 SV=3 - (ZN860_HUMAN)	2.41	3.48	1	1	2	3	1.699E7	632	73.7	9.33
H7C547	Sodium/potassium-transporting ATPase subunit beta-3 (Fragment) OS=Homo sapiens GN=ATP1B3 PE=3 SV=1 - (H7C547_HUMAN)	2.39	55.81	3	1	2	8	9.116E7	43	4.9	9.77
A5YM41	MTSS1 protein OS=Homo sapiens GN=MTSS1 PE=2 SV=1 - (A5YM41_HUMAN)	2.38	5.89	4	1	4	4	9.022E7	730	79.0	6.39
O00300	Tumor necrosis factor receptor superfamily member 11B OS=Homo sapiens GN=TNFRSF11B PE=1 SV=3 - (TR11B_HUMAN)	2.37	5.49	1	1	2	3	4.019E6	401	46.0	8.29
Q5VYB5	Nucleoporin 43kDa OS=Homo sapiens GN=NUP43 PE=2 SV=1 - (Q5VYB5_HUMAN)	2.34	20.97	1	1	3	4	2.007E8	248	27.0	6.30
P83881	60S ribosomal protein L36a OS=Homo sapiens GN=RPL36A PE=1 SV=2 - (RL36A_HUMAN)	2.33	23.58	6	1	3	3	1.422E8	106	12.4	10.58
H0YCR4	Post-GPI attachment to proteins factor 2 (Fragment) OS=Homo sapiens GN=PGAP2 PE=4 SV=1 - (H0YCR4_HUMAN)	2.27	47.62	1	1	2	5	6.929E7	42	4.6	10.04
G5E9R3	60S ribosomal protein L37a OS=Homo sapiens GN=RPL37A PE=4 SV=1 - (G5E9R3_HUMAN)	2.27	32.76	5	1	2	4	4.838E6	58	6.6	10.45
O60493-3	Isoform 3 of Sorting nexin-3 OS=Homo sapiens GN=SNX3 - (SNX3_HUMAN)	2.26	12.39	4	1	2	2	2.677E7	113	13.2	8.66
Q5SSJ5-2	Isoform 2 of Heterochromatin protein 1-binding protein 3 OS=Homo sapiens GN=HP1BP3 - (HP1B3_HUMAN)	2.26	17.86	5	1	9	9	1.259E8	515	57.2	9.76
E7EWI9	Heterogeneous nuclear ribonucleoprotein A3 OS=Homo sapiens GN=HNRNPA3 PE=4 SV=1 - (E7EWI9_HUMAN)	2.25	20.19	5	1	6	7	3.461E8	322	34.1	8.32
H0YLX4	WD repeat-containing protein 72 OS=Homo sapiens GN=WDR72 PE=4 SV=1 - (H0YLX4_HUMAN)	2.25	5.19	3	1	4	4	4.972E7	1099	123.0	6.76
D6RAG5	S-phase kinase-associated protein 2 OS=Homo sapiens GN=SKP2 PE=4 SV=1 - (D6RAG5_HUMAN)	2.25	14.29	1	1	3	5	1.489E8	217	24.7	7.44
H3BQZ7	HCG2044799 OS=Homo sapiens GN=hCG_2044799 PE=4 SV=1 - (H3BQZ7_HUMAN)	2.25	4.16	2	1	4	11	5.882E7	746	84.6	4.93
Q50KG7	Taste receptor type 2 (Fragment) OS=Homo sapiens GN=Hosa(Biaka)-T2R47 PE=3 SV=1 - (Q50KG7_HUMAN)	2.24	8.42	2	1	2	3	7.509E7	285	32.7	9.83
P53618	Coatomer subunit beta OS=Homo sapiens GN=COPB1 PE=1 SV=3 - (COPB_HUMAN)	2.24	4.51	2	1	4	4	9.605E7	953	107.1	6.05
Q96GI7	Protein FAM89A OS=Homo sapiens GN=FAM89A PE=2 SV=1 - (FA89A_HUMAN)	2.23	20.65	1	1	3	6	4.885E8	184	19.6	5.92
D6RGH1	Uncharacterized protein OS=Homo sapiens PE=4 SV=1 - (D6RGH1_HUMAN)	2.23	13.46	1	1	2	3	1.289E8	156	17.5	8.19
B3KNR6	cDNA FLJ30255 fis, clone BRACE2002444, highly similar to LAS1-like protein OS=Homo sapiens PE=2 SV=1 - (B3KNR6_HUMAN)	2.19	11.74	1	1	2	4	5.026E7	247	27.4	4.07
Q96AU7	Thymic stromal lymphopoietin OS=Homo sapiens GN=TSLP PE=2 SV=1 - (Q96AU7_HUMAN)	2.18	31.67	3	1	1	2	6.105E7	60	7.1	11.09
P05204	Non-histone chromosomal protein HMG-17 OS=Homo sapiens GN=HMGN2 PE=1 SV=3 - (HMGN2_HUMAN)	2.18	28.89	2	1	2	4	5.257E5	90	9.4	9.99
E5RIY5	Serine protease 45 OS=Homo sapiens GN=PRSS45 PE=3 SV=1 - (E5RIY5_HUMAN)	2.17	8.18	3	1	1	1	1.887E8	110	12.4	7.59
Q9NRX4-2	Isoform 2 of 14 kDa phosphohistidine phosphatase OS=Homo sapiens GN=PHPT1 - (PHP14_HUMAN)	2.16	9.68	3	1	1	1	2.180E6	124	13.7	7.47
A8K335	cDNA FLJ76254, highly similar to Homo sapiens gamma-glutamyl hydrolase (GGH), mRNA OS=Homo sapiens PE=2 SV=1 - (A8K335_HUMAN)	2.16	4.40	2	1	1	1	2.624E6	318	36.0	7.42
P07359	Platelet glycoprotein Ib alpha chain OS=Homo sapiens GN=GP1BA PE=1 SV=1 - (GP1BA_HUMAN)	2.15	4.31	4	1	3	3	5.093E7	626	68.9	6.68
Q9Y3R5-2	Isoform 2 of Protein dopey-2 OS=Homo sapiens GN=DOPEY2 - (DOP2_HUMAN)	2.15	4.89	2	2	11	21	4.190E8	2291	257.2	6.40
H7C1C6	Translocon-associated protein subunit delta (Fragment) OS=Homo sapiens GN=SSR4 PE=4 SV=1 - (H7C1C6_HUMAN)	2.12	25.00	3	2	2	2	2.027E7	120	13.1	7.50
Q53FH6	Mitogen-activated protein kinase kinase 1 interacting protein 1 variant (Fragment) OS=Homo sapiens PE=2 SV=1 - (Q53FH6_HUMAN)	2.11	8.06	2	1	1	1	1.051E7	124	13.6	7.34
Q6GMR7	Fatty-acid amide hydrolase 2 OS=Homo sapiens GN=FAAH2 PE=2 SV=1 - (FAAH2_HUMAN)	2.10	1.69	1	1	2	2	5.098E7	532	58.3	9.07
Q8N8A7	HCG38579, isoform CRA_c OS=Homo sapiens GN=TM6SF2 PE=2 SV=1 - (Q8N8A7_HUMAN)	2.10	11.27	1	1	1	2	1.079E8	213	22.5	6.52
C9JYN0	Synaptophysin-like protein 1 OS=Homo sapiens GN=SYPL1 PE=4 SV=1 - (C9JYN0_HUMAN)	2.09	4.91	4	1	1	1	2.455E7	224	24.8	7.14
Q9Y5S9	RNA-binding protein 8A OS=Homo sapiens GN=RBM8A PE=1 SV=1 - (RBM8A_HUMAN)	2.09	6.32	1	1	2	2	1.404E6	174	19.9	5.72
Q6LES1	TYRP1 protein (Fragment) OS=Homo sapiens GN=TYRP1 PE=2 SV=1 - (Q6LES1_HUMAN)	2.08	10.44	3	1	5	9	7.702E8	527	59.6	6.18
C9J0K6	Sorcin OS=Homo sapiens GN=SRI PE=4 SV=1 - (C9J0K6_HUMAN)	2.07	7.10	4	1	1	1	9.322E6	155	17.6	5.60
F5H3J5	Protein S100-A4 OS=Homo sapiens GN=S100A4 PE=4 SV=1 - (F5H3J5_HUMAN)	2.07	28.89	2	1	3	3	1.067E7	90	10.6	7.09
B2REB8	Protein SET OS=Homo sapiens GN=SET PE=3 SV=1 - (B2REB8_HUMAN)	2.06	3.77	6	1	1	1	4.428E7	265	31.0	4.23
P14854	Cytochrome c oxidase subunit 6B1 OS=Homo sapiens GN=COX6B1 PE=1 SV=2 - (CX6B1_HUMAN)	2.05	19.77	1	1	2	2	3.533E7	86	10.2	7.05
Q86UJ1	Putative uncharacterized protein CNTNAP2 (Fragment) OS=Homo sapiens GN=CNTNAP2 PE=2 SV=1 - (Q86UJ1_HUMAN)	2.05	21.43	1	1	3	12	2.626E9	154	17.4	7.12
P36969-2	Isoform Cytoplasmic of Phospholipid hydroperoxide glutathione peroxidase, mitochondrial OS=Homo sapiens GN=GPX4 - (GPX4_HUMAN)	2.04	18.82	1	1	3	5	6.950E6	170	19.5	7.94
P55010	Eukaryotic translation initiation factor 5 OS=Homo sapiens GN=EIF5 PE=1 SV=2 - (IF5_HUMAN)	2.04	2.09	2	1	1	1	4.294E6	431	49.2	5.58
B5BUG9	Bone morphogenetic protein receptor type IA (Fragment) OS=Homo sapiens GN=BMPR1A PE=2 SV=1 - (B5BUG9_HUMAN)	2.03	6.95	2	1	3	4	1.310E8	532	60.1	7.58
G3V4R8	Tubulin polyglutamylase TTLL5 OS=Homo sapiens GN=TTLL5 PE=4 SV=1 - (G3V4R8_HUMAN)	2.03	5.68	3	1	4	8	1.666E7	775	86.4	9.29
B4DFN5	cDNA FLJ56769, weakly similar to Nuclear pore complex-interacting protein OS=Homo sapiens PE=4 SV=1 - (B4DFN5_HUMAN)	2.02	21.37	9	1	3	6	4.136E7	117	13.2	10.40
F2Z317	Histone-lysine N-methyltransferase SETD2 OS=Homo sapiens GN=SETD2 PE=4 SV=2 - (F2Z317_HUMAN)	2.01	4.68	9	1	12	21	1.795E8	2564	287.4	6.16
P49773	Histidine triad nucleotide-binding protein 1 OS=Homo sapiens GN=HINT1 PE=1 SV=2 - (HINT1_HUMAN)	1.98	21.43	6	2	3	3	7.048E7	126	13.8	6.95
Q5TEU4	NADH dehydrogenase (ubiquinone) 1 alpha subcomplex assembly factor 5 OS=Homo sapiens GN=NDUFAF5 PE=1 SV=1 - (NDUF5_HUMAN)	1.95	10.72	3	1	4	5	1.331E8	345	38.9	6.57
B7Z8G2	Peptidyl-prolyl cis-trans isomerase FKBP5 OS=Homo sapiens GN=FKBP5 PE=2 SV=1 - (B7Z8G2_HUMAN)	1.93	12.62	6	1	5	6	8.437E7	420	47.3	5.87
F8W7F1	p53 apoptosis effector-related to PMP-22 OS=Homo sapiens GN=PERP PE=4 SV=1 - (F8W7F1_HUMAN)	1.92	4.00	4	1	1	1	2.149E7	175	19.5	7.03
E5RHK5	Glutathione peroxidase 3 OS=Homo sapiens GN=GPX3 PE=4 SV=1 - (E5RHK5_HUMAN)	1.91	23.00	1	1	4	4	4.917E7	100	11.4	10.56
Q53FX5	Lin-7 homolog C variant (Fragment) OS=Homo sapiens PE=2 SV=1 - (Q53FX5_HUMAN)	1.91	11.17	3	1	2	3	2.755E8	197	21.8	8.02
H7C0K5	Enhancer of polycomb homolog 2 (Fragment) OS=Homo sapiens GN=EPC2 PE=4 SV=1 - (H7C0K5_HUMAN)	1.91	6.35	1	1	1	1		126	14.9	9.64
Q9NZZ3-2	Isoform 2 of Charged multivesicular body protein 5 OS=Homo sapiens GN=CHMP5 - (CHMP5_HUMAN)	1.91	10.53	2	1	2	3	2.219E7	171	19.5	6.84
O15144	Actin-related protein 2/3 complex subunit 2 OS=Homo sapiens GN=ARPC2 PE=1 SV=1 - (ARPC2_HUMAN)	1.88	16.67	2	1	4	4	5.712E7	300	34.3	7.36
Q6UXN9	WD repeat-containing protein 82 OS=Homo sapiens GN=WDR82 PE=1 SV=1 - (WDR82_HUMAN)	1.87	7.35	1	1	3	6	2.206E8	313	35.1	7.69
Q15776	Zinc finger protein 192 OS=Homo sapiens GN=ZNF192 PE=1 SV=2 - (ZN192_HUMAN)	1.83	3.11	2	1	2	2	1.383E8	578	65.8	7.39
H7BY78	Hypoxanthine-guanine phosphoribosyltransferase OS=Homo sapiens GN=HPRT1 PE=4 SV=1 - (H7BY78_HUMAN)	1.82	4.59	3	1	1	1	2.415E7	218	24.6	7.02
B2RAH1	cDNA, FLJ94913, highly similar to Homo sapiens holocarboxylase synthetase(biotin-(proprionyl-Coenzyme A-carboxylase (ATP-hydrolysing))ligase) (HLCS), mRNA OS=Homo sapiens PE=2 SV=1 - (B2RAH1_HUMAN)	1.81	5.10	4	1	3	4	6.917E7	726	80.7	5.62
Q9NX07-2	Isoform 2 of tRNA selenocysteine 1-associated protein 1 OS=Homo sapiens GN=TRNAU1AP - (TSAP1_HUMAN)	1.80	5.08	2	1	1	1	5.624E7	177	20.4	4.55
H3BSP9	Metallothionein OS=Homo sapiens GN=MT2A PE=3 SV=1 - (H3BSP9_HUMAN)	1.79	12.33	1	1	1	1	2.363E6	73	7.7	8.37
Q96QR8	Transcriptional activator protein Pur-beta OS=Homo sapiens GN=PURB PE=1 SV=3 - (PURB_HUMAN)	1.78	2.88	1	1	2	17	1.501E7	312	33.2	5.43
P56385	ATP synthase subunit e, mitochondrial OS=Homo sapiens GN=ATP5I PE=1 SV=2 - (ATP5I_HUMAN)	1.78	10.14	1	1	1	2	9.735E6	69	7.9	9.35
Q6ZNE9	RUN and FYVE domain-containing protein 4 OS=Homo sapiens GN=RUFY4 PE=2 SV=2 - (RUFY4_HUMAN)	1.78	9.98	2	1	7	18	6.868E7	571	64.3	6.83
H0Y9T8	Protein PRR5-ARHGAP8 (Fragment) OS=Homo sapiens GN=PRR5-ARHGAP8 PE=4 SV=1 - (H0Y9T8_HUMAN)	1.78	18.45	16	1	6	6	8.120E7	504	57.6	9.45
B4DYI2	cDNA FLJ59639 OS=Homo sapiens PE=2 SV=1 - (B4DYI2_HUMAN)	1.77	8.73	1	1	10	18	2.294E8	1134	124.2	9.25
H7C4C8	T-complex protein 1 subunit theta (Fragment) OS=Homo sapiens GN=CCT8 PE=3 SV=1 - (H7C4C8_HUMAN)	1.76	13.62	4	1	4	7	3.499E8	323	35.5	5.96
Q8WUW1	Protein BRICK1 OS=Homo sapiens GN=BRK1 PE=1 SV=1 - (BRK1_HUMAN)	1.76	42.67	3	1	3	5	4.209E7	75	8.7	5.45
H0YCU2	Sharpin (Fragment) OS=Homo sapiens GN=SHARPIN PE=4 SV=1 - (H0YCU2_HUMAN)	1.75	7.37	2	1	1	2	2.436E7	95	10.0	5.17
Q8N2E1	cDNA PSEC0226 fis, clone HEMBA1005833, moderately similar to Mus musculus carboxypeptidase X2 mRNA OS=Homo sapiens PE=2 SV=1 - (Q8N2E1_HUMAN)	1.74	4.61	2	1	2	6	2.684E8	477	52.7	7.46
B2RDB6	cDNA, FLJ96540, highly similar to Homo sapiens zinc finger protein 3 (A8-51) (ZNF3), transcript variant 2, mRNA OS=Homo sapiens PE=2 SV=1 - (B2RDB6_HUMAN)	1.72	10.73	9	1	5	6	3.042E7	410	47.1	8.32
A6NLI5	Putative tripartite motif-containing protein 64C OS=Homo sapiens GN=TRIM64C PE=5 SV=3 - (TR64C_HUMAN)	1.72	8.28	8	2	4	5	4.814E7	447	51.3	5.52
Q9NTN9	Semaphorin-4G OS=Homo sapiens GN=SEMA4G PE=2 SV=1 - (SEM4G_HUMAN)	1.71	5.13	3	1	4	6	9.171E7	838	91.4	7.84
Q92574	Hamartin OS=Homo sapiens GN=TSC1 PE=1 SV=2 - (TSC1_HUMAN)	1.71	4.12	2	1	7	15	9.167E7	1164	129.7	6.47
A8K8F6	cDNA FLJ78417, highly similar to Homo sapiens low density lipoprotein receptor-related protein associated protein 1 (LRPAP1), mRNA OS=Homo sapiens PE=2 SV=1 - (A8K8F6_HUMAN)	1.70	12.89	3	1	5	8	4.526E8	357	41.4	9.06
A6NJH9	Eukaryotic translation initiation factor 1A, Y-chromosomal OS=Homo sapiens GN=EIF1AY PE=4 SV=1 - (A6NJH9_HUMAN)	1.70	27.56	3	1	4	8	8.266E7	127	14.4	5.34
Q9HAC7-2	Isoform 2 of CaiB/baiF CoA-transferase family protein C7orf10 OS=Homo sapiens GN=C7orf10 - (CG010_HUMAN)	1.70	8.76	8	1	4	6	9.352E7	434	47.2	9.13
B4DQI0	cDNA FLJ59385 OS=Homo sapiens PE=2 SV=1 - (B4DQI0_HUMAN)	1.70	6.02	1	1	2	2	1.424E7	299	33.3	6.02
O43781	Dual specificity tyrosine-phosphorylation-regulated kinase 3 OS=Homo sapiens GN=DYRK3 PE=1 SV=3 - (DYRK3_HUMAN)	1.69	5.78	7	1	4	8	3.579E7	588	65.7	9.32
Q9HBA9	Putative N-acetylated-alpha-linked acidic dipeptidase OS=Homo sapiens GN=FOLH1B PE=2 SV=1 - (FOH1B_HUMAN)	1.68	5.66	1	1	3	3	1.161E8	442	50.0	7.02
O95741	Copine-6 OS=Homo sapiens GN=CPNE6 PE=1 SV=3 - (CPNE6_HUMAN)	1.66	10.41	4	1	4	4	3.286E7	557	62.0	5.52
B4DI85	ADP-ribosylation factor-like 8B, isoform CRA_a OS=Homo sapiens GN=ARL8B PE=2 SV=1 - (B4DI85_HUMAN)	1.66	3.68	4	1	1	2	1.371E7	163	18.6	5.99
H3BMX4	Protein FAM188B2 OS=Homo sapiens GN=FAM188B2 PE=4 SV=1 - (H3BMX4_HUMAN)	1.65	7.87	3	1	3	9	1.335E8	356	40.6	7.69
H0YAR1	Lysyl oxidase homolog 2 (Fragment) OS=Homo sapiens GN=LOXL2 PE=4 SV=1 - (H0YAR1_HUMAN)	1.64	5.80	1	1	1	1		207	22.4	7.09
F1T0L1	Hypothetical LOC645967 OS=Homo sapiens GN=LOC645967 PE=2 SV=1 - (F1T0L1_HUMAN)	1.64	5.49	1	1	2	2	7.238E7	182	19.8	12.10
G3V1D7	Reticulon 4 receptor-like 2, isoform CRA_a OS=Homo sapiens GN=RTN4RL2 PE=4 SV=1 - (G3V1D7_HUMAN)	1.63	12.75	1	1	2	2	8.383E7	204	22.9	8.65
H0YG18	Mesothelin-like protein (Fragment) OS=Homo sapiens GN=MSLNL PE=4 SV=1 - (H0YG18_HUMAN)	1.63	6.91	2	1	5	7	2.225E6	752	79.7	7.37
D6RJD6	NADH dehydrogenase (ubiquinone) 1 alpha subcomplex subunit 2 OS=Homo sapiens GN=NDUFA2 PE=4 SV=1 - (D6RJD6_HUMAN)	1.63	14.47	2	1	2	4	1.388E7	76	8.5	10.11
C9J7D4	Uncharacterized protein OS=Homo sapiens GN=REPIN1 PE=4 SV=2 - (C9J7D4_HUMAN)	1.63	42.55	2	1	2	26	8.664E7	47	4.7	11.87
G3V1A8	Lymphocyte antigen 6 complex locus protein G6c OS=Homo sapiens GN=LY6G6C PE=4 SV=1 - (G3V1A8_HUMAN)	1.62	11.59	2	1	1	2	1.524E7	69	7.6	7.71
O43660-2	Isoform 2 of Pleiotropic regulator 1 OS=Homo sapiens GN=PLRG1 - (PLRG1_HUMAN)	1.62	3.37	4	1	2	3	6.714E7	505	56.3	9.17
B4DF97	cDNA FLJ59673, highly similar to Homo sapiens growth and transformation-dependent protein (E2IG5), mRNA OS=Homo sapiens PE=2 SV=1 - (B4DF97_HUMAN)	1.61	17.73	4	1	2	2	2.335E7	141	15.9	9.95
Q86UD0	Suppressor APC domain-containing protein 2 OS=Homo sapiens GN=SAPCD2 PE=2 SV=2 - (SAPC2_HUMAN)	1.61	9.90	1	1	4	6	8.281E7	394	42.6	8.82
Q9BV35	Calcium-binding mitochondrial carrier protein SCaMC-3 OS=Homo sapiens GN=SLC25A23 PE=1 SV=2 - (SCMC3_HUMAN)	1.61	7.05	4	1	3	4	6.931E7	468	52.3	7.33
P36639	7,8-dihydro-8-oxoguanine triphosphatase OS=Homo sapiens GN=NUDT1 PE=1 SV=3 - (8ODP_HUMAN)	0.00	5.08	1	1	1	2	2.065E6	197	22.5	5.27
Q9Y4X0-2	Isoform 2 of AMME syndrome candidate gene 1 protein OS=Homo sapiens GN=AMMECR1 - (AMMR1_HUMAN)	0.00	7.64	4	1	1	3	1.972E7	157	14.9	8.15
P35626	Beta-adrenergic receptor kinase 2 OS=Homo sapiens GN=ADRBK2 PE=2 SV=2 - (ARBK2_HUMAN)	0.00	6.69	4	1	5	10	6.601E7	688	79.7	7.62
P56381	ATP synthase subunit epsilon, mitochondrial OS=Homo sapiens GN=ATP5E PE=1 SV=2 - (ATP5E_HUMAN)	0.00	13.73	2	1	1	1	6.614E6	51	5.8	9.92
Q6ZTY9	Uncharacterized protein C7orf65 OS=Homo sapiens GN=C7orf65 PE=2 SV=1 - (CG065_HUMAN)	0.00	14.57	1	1	2	2	9.609E7	151	16.8	9.36
Q03013-2	Isoform 2 of Glutathione S-transferase Mu 4 OS=Homo sapiens GN=GSTM4 - (GSTM4_HUMAN)	0.00	12.31	8	1	2	4	7.960E7	195	22.8	5.10
Q6JEL2	Kelch-like protein 10 OS=Homo sapiens GN=KLHL10 PE=2 SV=1 - (KLH10_HUMAN)	0.00	9.21	2	1	4	8	3.019E8	608	68.9	5.68
Q8WVC0-2	Isoform 2 of RNA polymerase-associated protein LEO1 OS=Homo sapiens GN=LEO1 - (LEO1_HUMAN)	0.00	11.88	4	1	6	10	1.072E8	606	68.0	4.50
Q86VX9-3	Isoform 3 of Vacuolar fusion protein MON1 homolog A OS=Homo sapiens GN=MON1A - (MON1A_HUMAN)	0.00	6.04	4	1	1	1	1.135E8	298	33.7	9.42
Q5TC84	Opioid growth factor receptor-like protein 1 OS=Homo sapiens GN=OGFRL1 PE=2 SV=1 - (OGRL1_HUMAN)	0.00	9.09	1	1	4	10	2.536E8	451	51.2	6.13
Q8WVV4	Protein POF1B OS=Homo sapiens GN=POF1B PE=1 SV=3 - (POF1B_HUMAN)	0.00	2.89	2	1	1	1	2.601E7	589	68.0	6.32
P98179	Putative RNA-binding protein 3 OS=Homo sapiens GN=RBM3 PE=1 SV=1 - (RBM3_HUMAN)	0.00	32.48	1	1	4	4	2.246E7	157	17.2	8.91
Q9NR45	Sialic acid synthase OS=Homo sapiens GN=NANS PE=1 SV=2 - (SIAS_HUMAN)	0.00	5.85	1	1	1	3	3.969E8	359	40.3	6.74
Q9UIV8-2	Isoform 2 of Serpin B13 OS=Homo sapiens GN=SERPINB13 - (SPB13_HUMAN)	0.00	4.42	11	2	2	3	1.018E7	339	38.4	5.81
Q9Y3Q8	TSC22 domain family protein 4 OS=Homo sapiens GN=TSC22D4 PE=1 SV=2 - (T22D4_HUMAN)	0.00	4.81	2	1	2	3	5.807E6	395	41.0	7.21
Q8N7K0	Zinc finger protein 433 OS=Homo sapiens GN=ZNF433 PE=2 SV=1 - (ZN433_HUMAN)	0.00	15.01	2	1	7	29	5.917E9	673	77.2	9.20
Q8IW36	Zinc finger protein 695 OS=Homo sapiens GN=ZNF695 PE=2 SV=4 - (ZN695_HUMAN)	0.00	6.21	1	1	3	4	3.559E7	515	60.1	8.65
B3KUY1	Splicing factor, arginine/serine-rich 2, isoform CRA_d OS=Homo sapiens GN=SFRS2 PE=2 SV=1 - (B3KUY1_HUMAN)	0.00	17.22	5	1	4	5	1.564E8	209	24.2	11.94
D3DSM3	HCG1749250, isoform CRA_b OS=Homo sapiens GN=hCG_1749250 PE=4 SV=1 - (D3DSM3_HUMAN)	0.00	4.52	1	1	1	2	4.839E6	199	21.7	7.61
B4DY47	Prohibitin OS=Homo sapiens GN=PHB PE=2 SV=1 - (B4DY47_HUMAN)	0.00	14.84	10	1	2	2	8.697E7	155	17.0	8.79
Q7Z4B2	MSTP161 OS=Homo sapiens GN=MST161 PE=2 SV=1 - (Q7Z4B2_HUMAN)	0.00	10.18	3	1	2	2	1.634E8	226	26.0	9.22
A4UCS3	NADH:ubiquinone oxidoreductase SDAP subunit (Fragment) OS=Homo sapiens PE=2 SV=1 - (A4UCS3_HUMAN)	0.00	12.10	3	1	2	2	1.069E7	124	13.7	7.14
C9JUL6	Uncharacterized protein OS=Homo sapiens PE=4 SV=1 - (C9JUL6_HUMAN)	0.00	2.60	1	1	1	2		385	41.0	11.53
G1UI17	Glycogen debranching enzyme (Fragment) OS=Homo sapiens GN=AGL PE=2 SV=1 - (G1UI17_HUMAN)	0.00	3.88	4	1	4	4	3.896E7	1262	143.6	6.64
B4DQG8	cDNA FLJ54124, highly similar to DNA methyltransferase 1-associated protein 1 OS=Homo sapiens PE=2 SV=1 - (B4DQG8_HUMAN)	0.00	10.28	11	1	5	11	1.156E8	457	51.8	9.54
Q8TAK2	Similar to catalase (Fragment) OS=Homo sapiens PE=2 SV=1 - (Q8TAK2_HUMAN)	0.00	10.96	3	1	2	2	1.491E7	228	25.9	8.51
A8MXP7	Major facilitator superfamily domain-containing protein 12 OS=Homo sapiens GN=MFSD12 PE=4 SV=1 - (A8MXP7_HUMAN)	0.00	1.30	1	1	1	1	1.588E7	538	58.7	8.76
D0EZN6	Cytochrome p450 (Fragment) OS=Homo sapiens GN=CYP1B1 PE=4 SV=1 - (D0EZN6_HUMAN)	0.00	25.00	1	1	1	1	1.877E7	40	4.8	5.19
I3L1M9	Diphthamide biosynthesis protein 1 (Fragment) OS=Homo sapiens GN=DPH1 PE=4 SV=1 - (I3L1M9_HUMAN)	0.00	27.10	1	1	3	4	2.644E7	107	11.4	10.35
Q5RLJ0	CLE OS=Homo sapiens PE=2 SV=1 - (Q5RLJ0_HUMAN)	0.00	11.48	2	1	3	4	8.715E7	244	28.0	6.44
Q9UG64	Putative uncharacterized protein DKFZp586I1223 (Fragment) OS=Homo sapiens GN=DKFZp586I1223 PE=2 SV=1 - (Q9UG64_HUMAN)	0.00	16.77	6	1	4	13	3.278E8	316	35.2	6.67
F8WF17	Galectin-related protein OS=Homo sapiens GN=LGALSL PE=4 SV=1 - (F8WF17_HUMAN)	0.00	12.99	3	1	1	1	9.656E6	77	8.1	6.51
H0YCE4	Uncharacterized protein C1orf105 (Fragment) OS=Homo sapiens GN=C1orf105 PE=4 SV=1 - (H0YCE4_HUMAN)	0.00	15.20	3	1	2	4	1.428E5	125	14.4	9.25
F5GZZ9	Soluble CD163 OS=Homo sapiens GN=CD163 PE=4 SV=1 - (F5GZZ9_HUMAN)	0.00	4.78	8	1	5	10	1.522E8	1109	120.2	6.10
A0N5T0	V1 protein (Fragment) OS=Homo sapiens GN=V1 PE=4 SV=1 - (A0N5T0_HUMAN)	0.00	7.45	1	1	1	2	1.674E7	161	17.6	10.14
B7Z8Q1	cDNA FLJ53690, highly similar to SEC14-like protein 2 OS=Homo sapiens PE=2 SV=1 - (B7Z8Q1_HUMAN)	0.00	10.94	11	1	3	4	4.210E7	320	36.6	8.57
Q658V8	Putative uncharacterized protein DKFZp666C182 (Fragment) OS=Homo sapiens GN=DKFZp666C182 PE=2 SV=1 - (Q658V8_HUMAN)	0.00	8.24	3	1	3	3	5.221E7	449	50.0	6.89
E7BYD6	MHC class II antigen (Fragment) OS=Homo sapiens GN=HLA-DRB1 PE=4 SV=1 - (E7BYD6_HUMAN)	0.00	22.47	1	1	1	6	1.174E9	89	10.9	5.10

Appendix E 

**Table 7:** Proteins identified in Subject 3 (N3).

Accession	Description	Score	Coverage	# Proteins	# Unique Peptides	# Peptides	# PSMs	Area	# AAs	MW (kDa)	calc. pI
O43790	Keratin, type II cuticular Hb6 OS=Homo sapiens GN=KRT86 PE=1 SV=1 - (KRT86_HUMAN)	14929.14	94.65	13	6	85	6243	9.933E9	486	53.5	5.66
P78386	Keratin, type II cuticular Hb5 OS=Homo sapiens GN=KRT85 PE=1 SV=1 - (KRT85_HUMAN)	13339.35	93.49	7	31	85	5411	8.718E9	507	55.8	6.55
F8W6V8	Keratin, type II cuticular Hb1 OS=Homo sapiens GN=KRT81 PE=3 SV=1 - (F8W6V8_HUMAN)	13171.56	86.16	12	1	78	5723	9.933E9	484	53.1	5.47
P78385	Keratin, type II cuticular Hb3 OS=Homo sapiens GN=KRT83 PE=1 SV=2 - (KRT83_HUMAN)	10755.97	77.28	10	2	74	4888	9.933E9	493	54.2	5.64
A0JNT2	KRT83 protein OS=Homo sapiens GN=KRT83 PE=2 SV=1 - (A0JNT2_HUMAN)	10750.30	84.34	10	1	73	4889	9.933E9	447	49.6	5.39
Q15323	Keratin, type I cuticular Ha1 OS=Homo sapiens GN=KRT31 PE=2 SV=3 - (K1H1_HUMAN)	10153.70	74.28	2	17	62	4151	1.323E10	416	47.2	4.88
O76009	Keratin, type I cuticular Ha3-I OS=Homo sapiens GN=KRT33A PE=2 SV=2 - (KT33A_HUMAN)	8176.72	83.17	1	12	58	3273	1.323E10	404	45.9	4.82
Q14525	Keratin, type I cuticular Ha3-II OS=Homo sapiens GN=KRT33B PE=2 SV=3 - (KT33B_HUMAN)	7920.34	83.17	1	17	59	3109	1.320E10	404	46.2	4.84
A6NCN2	Keratin-81-like protein OS=Homo sapiens PE=2 SV=3 - (KT81L_HUMAN)	7438.10	62.76	5	3	54	3283	8.549E9	486	53.4	6.35
P13647	Keratin, type II cytoskeletal 5 OS=Homo sapiens GN=KRT5 PE=1 SV=3 - (K2C5_HUMAN)	5909.05	70.51	23	41	81	2484	4.157E9	590	62.3	7.74
B4DRR0	cDNA FLJ53910, highly similar to Keratin, type II cytoskeletal 6A OS=Homo sapiens PE=2 SV=1 - (B4DRR0_HUMAN)	5456.79	76.64	21	4	74	2315	4.157E9	535	57.8	8.00
P04259	Keratin, type II cytoskeletal 6B OS=Homo sapiens GN=KRT6B PE=1 SV=5 - (K2C6B_HUMAN)	5451.97	72.70	17	3	75	2304	4.157E9	564	60.0	8.00
I3L2N9	Uncharacterized protein OS=Homo sapiens PE=4 SV=1 - (I3L2N9_HUMAN)	5075.94	81.98	3	26	55	2067	1.323E10	394	44.7	4.78
P02533	Keratin, type I cytoskeletal 14 OS=Homo sapiens GN=KRT14 PE=1 SV=4 - (K1C14_HUMAN)	4842.22	84.53	13	31	68	1980	4.982E9	472	51.5	5.16
H6VRF8	Keratin 1 OS=Homo sapiens GN=KRT1 PE=3 SV=1 - (H6VRF8_HUMAN)	4837.96	75.47	17	56	71	1669	1.636E9	644	66.0	8.12
P35908	Keratin, type II cytoskeletal 2 epidermal OS=Homo sapiens GN=KRT2 PE=1 SV=2 - (K22E_HUMAN)	4567.97	81.53	5	41	65	1776	3.183E9	639	65.4	8.00
B4DKV4	cDNA FLJ60647, highly similar to Keratin, type II cytoskeletal 6B OS=Homo sapiens PE=2 SV=1 - (B4DKV4_HUMAN)	4512.81	68.63	13	2	64	1909	4.157E9	526	55.7	8.43
P13645	Keratin, type I cytoskeletal 10 OS=Homo sapiens GN=KRT10 PE=1 SV=6 - (K1C10_HUMAN)	3482.75	70.55	2	39	52	1337	2.644E9	584	58.8	5.21
O95678	Keratin, type II cytoskeletal 75 OS=Homo sapiens GN=KRT75 PE=1 SV=2 - (K2C75_HUMAN)	3343.77	61.52	13	20	51	1442	4.941E9	551	59.5	7.74
Q92764	Keratin, type I cuticular Ha5 OS=Homo sapiens GN=KRT35 PE=2 SV=5 - (KRT35_HUMAN)	3302.18	71.43	2	20	42	1323	9.142E9	455	50.3	4.91
Q04695	Keratin, type I cytoskeletal 17 OS=Homo sapiens GN=KRT17 PE=1 SV=2 - (K1C17_HUMAN)	3217.02	95.37	9	29	58	1378	4.982E9	432	48.1	5.02
P35527	Keratin, type I cytoskeletal 9 OS=Homo sapiens GN=KRT9 PE=1 SV=3 - (K1C9_HUMAN)	2856.13	68.22	1	40	44	948	7.612E8	623	62.0	5.24
P08779	Keratin, type I cytoskeletal 16 OS=Homo sapiens GN=KRT16 PE=1 SV=4 - (K1C16_HUMAN)	2762.13	83.51	12	21	50	1171	4.982E9	473	51.2	5.05
Q07283	Trichohyalin OS=Homo sapiens GN=TCHH PE=1 SV=2 - (TRHY_HUMAN)	2756.34	78.38	33	177	248	1756	6.643E8	1943	253.8	5.78
P15924	Desmoplakin OS=Homo sapiens GN=DSP PE=1 SV=3 - (DESP_HUMAN)	2710.04	68.79	19	179	239	1223	7.677E8	2871	331.6	6.81
Q5XKE5	Keratin, type II cytoskeletal 79 OS=Homo sapiens GN=KRT79 PE=1 SV=2 - (K2C79_HUMAN)	2486.18	25.98	5	1	24	1163	3.950E9	535	57.8	7.20
P12035	Keratin, type II cytoskeletal 3 OS=Homo sapiens GN=KRT3 PE=1 SV=3 - (K2C3_HUMAN)	1955.66	35.19	18	1	33	1121	3.950E9	628	64.4	6.48
Q9NSB4	Keratin, type II cuticular Hb2 OS=Homo sapiens GN=KRT82 PE=1 SV=3 - (KRT82_HUMAN)	1841.10	33.53	2	6	22	953	3.191E9	513	56.6	6.74
Q14532	Keratin, type I cuticular Ha2 OS=Homo sapiens GN=KRT32 PE=1 SV=3 - (K1H2_HUMAN)	1602.11	36.61	1	2	19	650	8.826E9	448	50.3	4.84
Q9NSB2	Keratin, type II cuticular Hb4 OS=Homo sapiens GN=KRT84 PE=1 SV=2 - (KRT84_HUMAN)	1468.49	13.67	3	1	14	838	3.950E9	600	64.8	7.56
P19012	Keratin, type I cytoskeletal 15 OS=Homo sapiens GN=KRT15 PE=1 SV=3 - (K1C15_HUMAN)	1460.08	62.50	8	1	34	632	4.982E9	456	49.2	4.77
B3KVF5	cDNA FLJ16494 fis, clone CTONG3004576, highly similar to Keratin, type I cytoskeletal 15 OS=Homo sapiens PE=2 SV=1 - (B3KVF5_HUMAN)	1327.93	62.06	6	2	31	528	4.982E9	456	49.1	4.79
Q3SY84	Keratin, type II cytoskeletal 71 OS=Homo sapiens GN=KRT71 PE=1 SV=3 - (K2C71_HUMAN)	1272.07	65.77	3	25	52	762	1.717E9	523	57.3	6.61
O76013-2	Isoform 2 of Keratin, type I cuticular Ha6 OS=Homo sapiens GN=KRT36 - (KRT36_HUMAN)	1210.08	25.42	2	1	16	570	5.287E9	417	47.5	4.81
F5GZD1	Keratin, type II cytoskeletal 7 OS=Homo sapiens GN=KRT7 PE=4 SV=1 - (F5GZD1_HUMAN)	1203.21	26.55	3	1	17	884	4.459E9	403	44.9	5.55
P08729	Keratin, type II cytoskeletal 7 OS=Homo sapiens GN=KRT7 PE=1 SV=5 - (K2C7_HUMAN)	1026.73	26.01	25	1	19	709	4.459E9	469	51.4	5.48
Q9NSB5	Type II hair keratin 1 (Fragment) OS=Homo sapiens GN=KRTHB1 PE=2 SV=1 - (Q9NSB5_HUMAN)	994.12	88.62	1	1	13	550	4.794E9	123	12.9	8.57
Q86Y46	Keratin, type II cytoskeletal 73 OS=Homo sapiens GN=KRT73 PE=1 SV=1 - (K2C73_HUMAN)	944.34	30.56	8	1	28	653	1.750E9	540	58.9	7.23
Q7RTS7	Keratin, type II cytoskeletal 74 OS=Homo sapiens GN=KRT74 PE=1 SV=2 - (K2C74_HUMAN)	885.31	46.69	7	7	35	626	1.750E9	529	57.8	7.71
Q8WVW5	Putative uncharacterized protein (Fragment) OS=Homo sapiens PE=2 SV=1 - (Q8WVW5_HUMAN)	846.81	75.76	82	8	32	335	1.651E9	363	40.5	6.14
Q7Z3Z0	Keratin, type I cytoskeletal 25 OS=Homo sapiens GN=KRT25 PE=1 SV=1 - (K1C25_HUMAN)	792.84	69.56	3	13	28	350	4.667E8	450	49.3	5.08
Q7Z3Y8	Keratin, type I cytoskeletal 27 OS=Homo sapiens GN=KRT27 PE=1 SV=2 - (K1C27_HUMAN)	763.74	50.11	1	7	23	331	4.651E8	459	49.8	5.05
P05787	Keratin, type II cytoskeletal 8 OS=Homo sapiens GN=KRT8 PE=1 SV=7 - (K2C8_HUMAN)	745.41	35.61	28	1	24	512	3.372E9	483	53.7	5.59
A1A4E9	Keratin 13 OS=Homo sapiens GN=KRT13 PE=2 SV=1 - (A1A4E9_HUMAN)	725.87	40.17	6	4	23	324	4.510E9	458	49.6	4.96
Q7Z3Y7	Keratin, type I cytoskeletal 28 OS=Homo sapiens GN=KRT28 PE=1 SV=2 - (K1C28_HUMAN)	708.59	28.88	1	3	16	410	3.418E9	464	50.5	5.47
Q0IIN1	Keratin 77 OS=Homo sapiens GN=KRT77 PE=2 SV=1 - (Q0IIN1_HUMAN)	656.56	15.74	4	1	9	416	2.428E9	578	61.8	5.85
B4DRW1	cDNA FLJ55805, highly similar to Keratin, type II cytoskeletal 4 OS=Homo sapiens PE=2 SV=1 - (B4DRW1_HUMAN)	626.30	41.14	10	4	25	330	1.007E9	474	51.7	6.81
Q5QNW6	Histone H2B type 2-F OS=Homo sapiens GN=HIST2H2BF PE=1 SV=3 - (H2B2F_HUMAN)	578.81	69.84	32	8	13	260	5.882E8	126	13.9	10.32
F5H5D3	Tubulin alpha-1C chain OS=Homo sapiens GN=TUBA1C PE=3 SV=1 - (F5H5D3_HUMAN)	483.94	44.70	24	1	20	149	3.544E8	519	57.7	5.07
B7Z565	cDNA FLJ54739, highly similar to Alpha-actinin-1 OS=Homo sapiens PE=2 SV=1 - (B7Z565_HUMAN)	451.65	51.58	23	12	37	153	1.870E8	822	94.7	5.69
O43707	Alpha-actinin-4 OS=Homo sapiens GN=ACTN4 PE=1 SV=2 - (ACTN4_HUMAN)	435.11	52.36	17	17	40	153	1.870E8	911	104.8	5.44
O76015	Keratin, type I cuticular Ha8 OS=Homo sapiens GN=KRT38 PE=2 SV=3 - (KRT38_HUMAN)	429.10	32.24	1	6	14	241	2.817E9	456	50.4	4.84
P68371	Tubulin beta-4B chain OS=Homo sapiens GN=TUBB4B PE=1 SV=1 - (TBB4B_HUMAN)	417.34	55.28	23	3	21	153	1.689E8	445	49.8	4.89
A8MUB1	Tubulin alpha-4A chain OS=Homo sapiens GN=TUBA4A PE=2 SV=1 - (A8MUB1_HUMAN)	403.13	45.03	14	4	18	129	3.242E8	433	48.3	5.01
P14923	Junction plakoglobin OS=Homo sapiens GN=JUP PE=1 SV=3 - (PLAK_HUMAN)	403.11	50.60	12	29	32	178	7.104E7	745	81.7	6.14
E7ENN3	Nesprin-1 OS=Homo sapiens GN=SYNE1 PE=4 SV=1 - (E7ENN3_HUMAN)	397.85	7.88	19	2	52	250	1.520E9	8409	966.0	5.55
G3V1U9	Tubulin alpha-1A chain OS=Homo sapiens GN=TUBA1A PE=3 SV=1 - (G3V1U9_HUMAN)	397.11	51.68	28	1	20	127	3.544E8	416	46.3	5.08
F8VW92	Tubulin beta chain OS=Homo sapiens GN=TUBB PE=3 SV=1 - (F8VW92_HUMAN)	396.77	53.79	42	3	20	146	1.689E8	435	48.5	5.20
P04792	Heat shock protein beta-1 OS=Homo sapiens GN=HSPB1 PE=1 SV=2 - (HSPB1_HUMAN)	393.81	94.15	4	19	19	158	8.326E8	205	22.8	6.40
Q96KK5	Histone H2A type 1-H OS=Homo sapiens GN=HIST1H2AH PE=1 SV=3 - (H2A1H_HUMAN)	392.83	42.19	16	2	9	134	3.541E8	128	13.9	10.89
P07355-2	Isoform 2 of Annexin A2 OS=Homo sapiens GN=ANXA2 - (ANXA2_HUMAN)	379.65	81.79	27	24	32	141	5.794E8	357	40.4	8.37
Q93077	Histone H2A type 1-C OS=Homo sapiens GN=HIST1H2AC PE=1 SV=3 - (H2A1C_HUMAN)	379.53	41.54	6	1	8	128	3.541E8	130	14.1	11.05
B2R6L0	cDNA, FLJ93005, highly similar to Homo sapiens tubulin, beta polypeptide (TUBB), mRNA OS=Homo sapiens PE=2 SV=1 - (B2R6L0_HUMAN)	371.43	52.81	24	1	20	146	1.545E8	445	49.9	4.89
B7Z6P1	cDNA FLJ53662, highly similar to Actin, alpha skeletal muscle OS=Homo sapiens PE=2 SV=1 - (B7Z6P1_HUMAN)	360.85	40.52	47	1	21	202	8.890E8	343	38.6	5.38
P31947	14-3-3 protein sigma OS=Homo sapiens GN=SFN PE=1 SV=1 - (1433S_HUMAN)	357.87	70.56	5	19	26	165	8.457E8	248	27.8	4.74
Q2M2I5	Keratin, type I cytoskeletal 24 OS=Homo sapiens GN=KRT24 PE=1 SV=1 - (K1C24_HUMAN)	352.71	9.33	1	1	6	199	2.292E9	525	55.1	4.96
Q5DT20	Hornerin OS=Homo sapiens GN=HRNR PE=2 SV=1 - (Q5DT20_HUMAN)	334.78	22.60	2	16	20	146	1.033E8	2850	282.2	10.02
F8VZY9	Keratin, type I cytoskeletal 18 OS=Homo sapiens GN=KRT18 PE=3 SV=1 - (F8VZY9_HUMAN)	332.29	23.53	3	1	9	200	2.302E9	391	43.7	5.35
Q9BYR8	Keratin-associated protein 3-1 OS=Homo sapiens GN=KRTAP3-1 PE=1 SV=1 - (KRA31_HUMAN)	331.81	27.55	1	3	3	100	2.319E8	98	10.5	6.40
P62805	Histone H4 OS=Homo sapiens GN=HIST1H4A PE=1 SV=2 - (H4_HUMAN)	326.84	57.28	3	10	11	124	5.094E8	103	11.4	11.36
Q15149-4	Isoform 4 of Plectin OS=Homo sapiens GN=PLEC - (PLEC_HUMAN)	325.87	24.98	12	40	113	260	7.230E8	4547	515.9	5.80
Q09666	Neuroblast differentiation-associated protein AHNAK OS=Homo sapiens GN=AHNAK PE=1 SV=2 - (AHNK_HUMAN)	283.60	37.56	5	45	90	188	2.564E8	5890	628.7	6.15
Q13835-2	Isoform 1 of Plakophilin-1 OS=Homo sapiens GN=PKP1 - (PKP1_HUMAN)	267.89	47.93	4	24	31	122	8.105E7	726	80.4	8.97
Q6A162	Keratin, type I cytoskeletal 40 OS=Homo sapiens GN=KRT40 PE=1 SV=2 - (K1C40_HUMAN)	264.09	24.59	3	1	11	164	3.914E9	431	48.1	4.41
P47929	Galectin-7 OS=Homo sapiens GN=LGALS7 PE=1 SV=2 - (LEG7_HUMAN)	262.52	93.38	1	13	16	91	5.213E8	136	15.1	7.62
Q562R1	Beta-actin-like protein 2 OS=Homo sapiens GN=ACTBL2 PE=1 SV=2 - (ACTBL_HUMAN)	253.61	26.60	13	1	15	139	1.053E9	376	42.0	5.59
Q8N1N4	Keratin, type II cytoskeletal 78 OS=Homo sapiens GN=KRT78 PE=2 SV=2 - (K2C78_HUMAN)	247.76	25.96	4	3	15	147	2.180E9	520	56.8	6.02
O75369-2	Isoform 2 of Filamin-B OS=Homo sapiens GN=FLNB - (FLNB_HUMAN)	237.39	19.01	15	24	39	97	3.784E8	2578	275.5	5.78
P35579	Myosin-9 OS=Homo sapiens GN=MYH9 PE=1 SV=4 - (MYH9_HUMAN)	236.01	32.14	20	24	62	129	4.062E8	1960	226.4	5.60
P63104	14-3-3 protein zeta/delta OS=Homo sapiens GN=YWHAZ PE=1 SV=1 - (1433Z_HUMAN)	233.11	67.35	17	11	20	98	5.952E8	245	27.7	4.79
P27348	14-3-3 protein theta OS=Homo sapiens GN=YWHAQ PE=1 SV=1 - (1433T_HUMAN)	226.79	59.59	9	9	20	91	6.235E8	245	27.7	4.78
Q8TC04	Keratin 23 (Histone deacetylase inducible) OS=Homo sapiens GN=KRT23 PE=2 SV=1 - (Q8TC04_HUMAN)	226.29	5.92	1	1	4	172	4.468E8	422	48.1	6.54
P04406	Glyceraldehyde-3-phosphate dehydrogenase OS=Homo sapiens GN=GAPDH PE=1 SV=3 - (G3P_HUMAN)	219.67	57.61	11	12	17	93	1.088E9	335	36.0	8.46
C9J1U3	Protein unc-80 homolog OS=Homo sapiens GN=UNC80 PE=4 SV=1 - (C9J1U3_HUMAN)	216.26	4.55	4	1	12	113	1.308E9	3234	360.6	6.92
Q7Z3Y9	Keratin, type I cytoskeletal 26 OS=Homo sapiens GN=KRT26 PE=1 SV=2 - (K1C26_HUMAN)	200.86	25.21	1	2	8	118	2.645E8	468	51.9	4.92
Q3LI55	Keratin associated protein OS=Homo sapiens GN=KRTAP11-1 PE=2 SV=1 - (Q3LI55_HUMAN)	196.99	26.99	2	4	5	129	7.964E7	163	17.0	7.78
Q9HD64-4	Isoform D of G antigen family D member 2 OS=Homo sapiens GN=XAGE1A - (GAGD2_HUMAN)	193.38	24.64	7	1	2	92	8.028E7	69	8.0	10.11
Q5HY54	Filamin-A OS=Homo sapiens GN=FLNA PE=2 SV=1 - (Q5HY54_HUMAN)	191.07	20.25	18	28	43	104	6.760E7	2607	276.4	6.05
O75116	Rho-associated protein kinase 2 OS=Homo sapiens GN=ROCK2 PE=1 SV=4 - (ROCK2_HUMAN)	176.42	8.72	4	1	14	109	2.142E9	1388	160.8	6.02
P61981	14-3-3 protein gamma OS=Homo sapiens GN=YWHAG PE=1 SV=2 - (1433G_HUMAN)	176.33	47.77	10	5	14	78	6.235E8	247	28.3	4.89
A0N4V7	HCG2039797 (Fragment) OS=Homo sapiens GN=Tcr-alpha PE=4 SV=1 - (A0N4V7_HUMAN)	172.95	38.10	1	1	1	298	1.036E10	21	2.2	9.74
P31946-2	Isoform Short of 14-3-3 protein beta/alpha OS=Homo sapiens GN=YWHAB - (1433B_HUMAN)	172.26	48.77	8	2	17	76	6.235E8	244	27.8	4.83
P68104	Elongation factor 1-alpha 1 OS=Homo sapiens GN=EEF1A1 PE=1 SV=1 - (EF1A1_HUMAN)	165.51	29.87	38	8	13	79	1.167E8	462	50.1	9.01
P11142	Heat shock cognate 71 kDa protein OS=Homo sapiens GN=HSPA8 PE=1 SV=1 - (HSP7C_HUMAN)	160.54	41.95	27	9	30	84	2.976E8	646	70.9	5.52
P54652	Heat shock-related 70 kDa protein 2 OS=Homo sapiens GN=HSPA2 PE=1 SV=1 - (HSP72_HUMAN)	157.55	45.23	3	9	29	80	2.976E8	639	70.0	5.74
H0YG33	Heat shock 70 kDa protein 1A/1B OS=Homo sapiens GN=HSPA1B PE=3 SV=1 - (H0YG33_HUMAN)	156.21	36.16	23	16	27	81	1.828E8	708	77.4	6.99
Q6DRA6	Putative histone H2B type 2-D OS=Homo sapiens GN=HIST2H2BD PE=5 SV=3 - (H2B2D_HUMAN)	154.99	32.32	20	2	6	71	9.315E7	164	18.0	10.58
P26038	Moesin OS=Homo sapiens GN=MSN PE=1 SV=3 - (MOES_HUMAN)	154.44	28.94	19	1	19	88	5.621E8	577	67.8	6.40
P08670	Vimentin OS=Homo sapiens GN=VIM PE=1 SV=4 - (VIME_HUMAN)	150.17	25.54	6	1	8	86	1.905E9	466	53.6	5.12
P15311	Ezrin OS=Homo sapiens GN=EZR PE=1 SV=4 - (EZRI_HUMAN)	149.37	40.10	25	5	28	82	5.213E8	586	69.4	6.27
F6KPG5	Albumin (Fragment) OS=Homo sapiens PE=2 SV=1 - (F6KPG5_HUMAN)	148.62	34.19	15	12	19	64	4.898E7	585	66.5	6.04
Q5I6Y6	Lamin A/C transcript variant 1 OS=Homo sapiens GN=LMNA PE=2 SV=1 - (Q5I6Y6_HUMAN)	141.79	55.57	17	18	33	70	1.648E8	664	74.0	7.18
Q01469	Fatty acid-binding protein, epidermal OS=Homo sapiens GN=FABP5 PE=1 SV=3 - (FABP5_HUMAN)	139.99	82.22	2	13	15	63	1.628E8	135	15.2	7.01
P62258	14-3-3 protein epsilon OS=Homo sapiens GN=YWHAE PE=1 SV=1 - (1433E_HUMAN)	137.30	49.02	12	7	12	54	5.393E8	255	29.2	4.74
P06733	Alpha-enolase OS=Homo sapiens GN=ENO1 PE=1 SV=2 - (ENOA_HUMAN)	133.87	44.01	24	6	14	52	7.651E7	434	47.1	7.39
Q9HCF6-8	Isoform 8 of Transient receptor potential cation channel subfamily M member 3 OS=Homo sapiens GN=TRPM3 - (TRPM3_HUMAN)	131.49	8.34	19	1	10	81	2.763E9	1679	191.3	7.15
Q86TY5	Full-length cDNA clone CS0DI041YE05 of Placenta of Homo sapiens (human) OS=Homo sapiens PE=2 SV=1 - (Q86TY5_HUMAN)	127.62	84.30	16	8	12	72	2.022E8	121	13.9	9.16
O94822	E3 ubiquitin-protein ligase listerin OS=Homo sapiens GN=LTN1 PE=1 SV=6 - (LTN1_HUMAN)	127.27	7.81	1	1	10	94	1.791E8	1766	200.4	6.25
Q96QV6	Histone H2A type 1-A OS=Homo sapiens GN=HIST1H2AA PE=1 SV=3 - (H2A1A_HUMAN)	123.66	40.46	6	2	7	55	2.535E8	131	14.2	10.86
P37802	Transgelin-2 OS=Homo sapiens GN=TAGLN2 PE=1 SV=3 - (TAGL2_HUMAN)	122.00	82.91	4	13	16	37	3.710E7	199	22.4	8.25
Q6A163	Keratin, type I cytoskeletal 39 OS=Homo sapiens GN=KRT39 PE=1 SV=2 - (K1C39_HUMAN)	119.73	29.12	16	9	16	80	2.527E8	491	55.6	5.26
Q7Z406	Myosin-14 OS=Homo sapiens GN=MYH14 PE=1 SV=2 - (MYH14_HUMAN)	117.65	19.85	22	7	39	93	3.385E8	1995	227.7	5.60
A8MTN3	Putative keratin-associated protein ENSP00000381495 OS=Homo sapiens PE=3 SV=2 - (KRA2X_HUMAN)	116.83	26.83	7	2	2	54	6.990E8	123	12.9	7.81
P55072	Transitional endoplasmic reticulum ATPase OS=Homo sapiens GN=VCP PE=1 SV=4 - (TERA_HUMAN)	116.40	29.90	23	17	25	48	1.402E8	806	89.3	5.26
P13639	Elongation factor 2 OS=Homo sapiens GN=EEF2 PE=1 SV=4 - (EF2_HUMAN)	113.95	33.10	8	14	22	46	3.308E8	858	95.3	6.83
A8K486	Peptidyl-prolyl cis-trans isomerase OS=Homo sapiens PE=2 SV=1 - (A8K486_HUMAN)	113.66	67.27	33	10	12	51	1.018E8	165	18.0	6.90
Q6KB66-2	Isoform 2 of Keratin, type II cytoskeletal 80 OS=Homo sapiens GN=KRT80 - (K2C80_HUMAN)	113.17	20.85	5	1	9	60	5.278E8	422	47.2	5.30
Q86UQ4	ATP-binding cassette sub-family A member 13 OS=Homo sapiens GN=ABCA13 PE=2 SV=3 - (ABCAD_HUMAN)	112.12	4.11	2	1	16	58	4.265E8	5058	575.8	6.46
B4E380	Histone H3 OS=Homo sapiens PE=3 SV=1 - (B4E380_HUMAN)	111.68	70.80	11	3	15	82	3.810E8	113	12.9	11.93
Q562V5	Actin-like protein (Fragment) OS=Homo sapiens GN=ACT PE=2 SV=1 - (Q562V5_HUMAN)	111.33	52.43	14	1	3	40	7.953E8	103	11.4	6.35
Q9BYR6	Keratin-associated protein 3-3 OS=Homo sapiens GN=KRTAP3-3 PE=1 SV=1 - (KRA33_HUMAN)	111.13	24.49	1	1	2	32	1.933E8	98	10.4	5.69
P60174-1	Isoform 2 of Triosephosphate isomerase OS=Homo sapiens GN=TPI1 - (TPIS_HUMAN)	110.90	81.12	6	13	15	39	6.034E7	249	26.7	6.90
Q14204	Cytoplasmic dynein 1 heavy chain 1 OS=Homo sapiens GN=DYNC1H1 PE=1 SV=5 - (DYHC1_HUMAN)	110.39	5.81	1	1	21	82	1.173E9	4646	532.1	6.40
P49327	Fatty acid synthase OS=Homo sapiens GN=FASN PE=1 SV=3 - (FAS_HUMAN)	108.49	5.50	1	2	14	84	3.374E9	2511	273.3	6.44
P62987	Ubiquitin-60S ribosomal protein L40 OS=Homo sapiens GN=UBA52 PE=1 SV=2 - (RL40_HUMAN)	108.46	58.59	30	6	8	58	7.260E7	128	14.7	9.83
Q02413	Desmoglein-1 OS=Homo sapiens GN=DSG1 PE=1 SV=2 - (DSG1_HUMAN)	108.01	20.88	2	11	18	44	1.843E7	1049	113.7	5.03
Q99996-5	Isoform 5 of A-kinase anchor protein 9 OS=Homo sapiens GN=AKAP9 - (AKAP9_HUMAN)	107.01	10.47	10	1	32	84	3.485E8	3857	447.5	4.98
O15078	Centrosomal protein of 290 kDa OS=Homo sapiens GN=CEP290 PE=1 SV=2 - (CE290_HUMAN)	104.03	11.05	2	2	29	106	3.377E8	2479	290.2	5.95
P05109	Protein S100-A8 OS=Homo sapiens GN=S100A8 PE=1 SV=1 - (S10A8_HUMAN)	100.57	41.94	1	5	6	45	2.679E8	93	10.8	7.03
Q92794	Histone acetyltransferase KAT6A OS=Homo sapiens GN=KAT6A PE=1 SV=2 - (KAT6A_HUMAN)	99.92	7.53	3	1	11	78	1.114E8	2004	224.9	5.68
P0C0S5	Histone H2A.Z OS=Homo sapiens GN=H2AFZ PE=1 SV=2 - (H2AZ_HUMAN)	98.99	31.25	9	2	5	45	2.349E8	128	13.5	10.58
B3KSC3	cDNA FLJ35987 fis, clone TESTI2014269, highly similar to D-3-phosphoglycerate dehydrogenase (EC 1.1.1.95) OS=Homo sapiens PE=2 SV=1 - (B3KSC3_HUMAN)	98.91	19.64	11	8	9	40	5.194E7	499	53.0	7.12
P06576	ATP synthase subunit beta, mitochondrial OS=Homo sapiens GN=ATP5B PE=1 SV=3 - (ATPB_HUMAN)	98.50	37.62	8	9	14	34	4.960E7	529	56.5	5.40
Q6ISF6	KRTAP9-2 protein (Fragment) OS=Homo sapiens GN=KRTAP9-2 PE=2 SV=1 - (Q6ISF6_HUMAN)	94.93	8.09	1	1	1	22	3.255E7	173	18.1	7.69
Q3KNW1	Zinc finger protein SNAI3 OS=Homo sapiens GN=SNAI3 PE=2 SV=1 - (SNAI3_HUMAN)	94.82	5.82	1	1	2	56	4.202E8	292	32.5	9.22
Q8NAM9	cDNA FLJ35099 fis, clone PLACE6006287 OS=Homo sapiens PE=2 SV=1 - (Q8NAM9_HUMAN)	94.82	2.68	1	1	1	78	8.352E8	224	24.0	11.75
B7Z2N5	cDNA FLJ51840, highly similar to Alpha-actinin-2 OS=Homo sapiens PE=2 SV=1 - (B7Z2N5_HUMAN)	94.71	26.40	10	1	17	44	1.051E8	803	92.9	5.81
P09211	Glutathione S-transferase P OS=Homo sapiens GN=GSTP1 PE=1 SV=2 - (GSTP1_HUMAN)	93.77	67.14	4	8	8	32	1.071E8	210	23.3	5.64
Q6PK75	PRSS2 protein (Fragment) OS=Homo sapiens GN=PRSS2 PE=2 SV=1 - (Q6PK75_HUMAN)	93.52	16.04	16	1	2	42	8.437E8	187	20.1	4.87
F8W8Y7	Armadillo repeat-containing X-linked protein 4 OS=Homo sapiens GN=ARMCX4 PE=4 SV=1 - (F8W8Y7_HUMAN)	92.66	8.94	3	1	16	65	9.660E7	2394	247.7	5.95
Q86SJ6	Desmoglein-4 OS=Homo sapiens GN=DSG4 PE=1 SV=1 - (DSG4_HUMAN)	92.00	19.81	2	13	16	41	7.571E7	1040	113.8	4.56
B4DF70	cDNA FLJ60461, highly similar to Peroxiredoxin-2 (EC 1.11.1.15) OS=Homo sapiens PE=2 SV=1 - (B4DF70_HUMAN)	90.89	40.98	4	7	9	35	1.469E8	183	20.1	8.78
Q5TEC6	Histone H3 OS=Homo sapiens GN=HIST2H3PS2 PE=3 SV=1 - (Q5TEC6_HUMAN)	90.73	61.03	1	1	13	72	3.810E8	136	15.4	11.27
Q9BYR7	Keratin-associated protein 3-2 OS=Homo sapiens GN=KRTAP3-2 PE=1 SV=1 - (KRA32_HUMAN)	90.32	24.49	1	1	3	26	2.275E8	98	10.4	5.69
D3DVC4	Nestin, isoform CRA_c OS=Homo sapiens GN=NES PE=3 SV=1 - (D3DVC4_HUMAN)	90.05	8.57	5	1	13	55	3.690E8	1621	177.3	4.36
Q14134-2	Isoform Beta of Tripartite motif-containing protein 29 OS=Homo sapiens GN=TRIM29 - (TRI29_HUMAN)	89.63	42.98	19	20	28	46	1.330E8	570	63.8	6.98
Q5SQ08	Complement component 8, gamma polypeptide (Fragment) OS=Homo sapiens GN=C8G PE=2 SV=1 - (Q5SQ08_HUMAN)	89.40	10.37	2	1	2	46	2.348E7	164	17.5	9.79
Q9P225-2	Isoform 2 of Dynein heavy chain 2, axonemal OS=Homo sapiens GN=DNAH2 - (DYH2_HUMAN)	88.95	6.17	3	2	21	69	2.156E8	4196	481.7	6.43
A6NC98	Coiled-coil domain-containing protein 88B OS=Homo sapiens GN=CCDC88B PE=1 SV=1 - (CC88B_HUMAN)	86.56	6.91	7	1	11	48	1.964E8	1476	164.7	5.11
F8WA45	Gelsolin OS=Homo sapiens GN=GSN PE=4 SV=1 - (F8WA45_HUMAN)	82.96	19.30	19	6	12	41	5.442E7	715	78.9	6.01
Q15075	Early endosome antigen 1 OS=Homo sapiens GN=EEA1 PE=1 SV=2 - (EEA1_HUMAN)	82.52	21.05	2	3	24	67	7.130E8	1411	162.4	5.68
Q9BYK8	Peroxisomal proliferator-activated receptor A-interacting complex 285 kDa protein OS=Homo sapiens GN=PRIC285 PE=1 SV=6 - (PR285_HUMAN)	80.94	7.63	4	1	16	63	5.007E8	2649	294.5	7.49
E9PK25	Cofilin-1 OS=Homo sapiens GN=CFL1 PE=4 SV=1 - (E9PK25_HUMAN)	80.15	61.27	14	8	11	27	1.841E8	204	22.7	8.34
P36952	Serpin B5 OS=Homo sapiens GN=SERPINB5 PE=1 SV=2 - (SPB5_HUMAN)	78.67	31.47	4	7	10	32	1.075E8	375	42.1	6.05
B4DMP5	cDNA FLJ60050, highly similar to Vacuolar protein sorting protein 72 homolog OS=Homo sapiens PE=2 SV=1 - (B4DMP5_HUMAN)	78.14	8.90	3	1	2	61	4.385E9	236	26.7	5.33
Q9H875	PRKR-interacting protein 1 OS=Homo sapiens GN=PRKRIP1 PE=1 SV=1 - (PKRI1_HUMAN)	77.43	10.87	1	1	2	52	7.507E7	184	21.0	9.79
F8VS61	Keratin, type II cytoskeletal 1b OS=Homo sapiens GN=KRT77 PE=4 SV=1 - (F8VS61_HUMAN)	76.90	15.32	1	1	4	58	4.737E8	248	25.4	8.69
P00338	L-lactate dehydrogenase A chain OS=Homo sapiens GN=LDHA PE=1 SV=2 - (LDHA_HUMAN)	75.20	44.58	25	8	16	41	5.517E7	332	36.7	8.27
F5H335	Eukaryotic translation initiation factor 3 subunit A OS=Homo sapiens GN=EIF3A PE=4 SV=1 - (F5H335_HUMAN)	75.10	16.99	8	1	20	64	3.757E8	1348	162.5	6.77
B2RAW1	cDNA, FLJ95155, highly similar to Homo sapiens Bruton agammaglobulinemia tyrosine kinase (BTK), mRNA OS=Homo sapiens PE=2 SV=1 - (B2RAW1_HUMAN)	75.03	8.04	14	1	5	44	2.157E8	659	76.2	7.77
H3BTN5	Pyruvate kinase (Fragment) OS=Homo sapiens GN=PKM PE=3 SV=1 - (H3BTN5_HUMAN)	74.35	33.61	27	9	15	29	3.095E7	485	53.0	6.84
Q8WXH0	Nesprin-2 OS=Homo sapiens GN=SYNE2 PE=1 SV=3 - (SYNE2_HUMAN)	74.13	8.28	6	1	48	89	3.698E9	6885	795.9	5.36
P09382	Galectin-1 OS=Homo sapiens GN=LGALS1 PE=1 SV=2 - (LEG1_HUMAN)	73.95	62.96	2	8	10	29	5.673E7	135	14.7	5.50
Q96AW5	QARS protein (Fragment) OS=Homo sapiens GN=QARS PE=2 SV=2 - (Q96AW5_HUMAN)	73.86	10.04	8	1	7	50	4.239E8	717	81.7	7.15
P58107	Epiplakin OS=Homo sapiens GN=EPPK1 PE=1 SV=2 - (EPIPL_HUMAN)	73.70	17.80	3	6	28	69	2.296E8	5090	555.3	5.60
Q86Y33-3	Isoform 3 of Cell division cycle protein 20 homolog B OS=Homo sapiens GN=CDC20B - (CD20B_HUMAN)	71.50	7.13	5	1	3	61	2.299E8	477	52.9	8.51
Q8WUT4	Leucine-rich repeat neuronal protein 4 OS=Homo sapiens GN=LRRN4 PE=1 SV=3 - (LRRN4_HUMAN)	71.03	5.41	2	1	3	48	4.658E8	740	78.8	7.20
P07737	Profilin-1 OS=Homo sapiens GN=PFN1 PE=1 SV=2 - (PROF1_HUMAN)	70.73	59.29	2	7	9	26	4.660E7	140	15.0	8.27
Q0QF37	Malate dehydrogenase (Fragment) OS=Homo sapiens GN=MDH2 PE=2 SV=1 - (Q0QF37_HUMAN)	69.85	40.66	8	6	9	21	3.677E7	305	31.9	7.88
Q9UPN3	Microtubule-actin cross-linking factor 1, isoforms 1/2/3/5 OS=Homo sapiens GN=MACF1 PE=1 SV=4 - (MACF1_HUMAN)	69.18	9.00	11	4	55	92	3.508E8	7388	837.8	5.39
Q8IUX7	Adipocyte enhancer-binding protein 1 OS=Homo sapiens GN=AEBP1 PE=1 SV=1 - (AEBP1_HUMAN)	67.77	4.32	3	1	4	71	1.926E8	1158	130.8	5.11
B7ZLH8	EVPL protein OS=Homo sapiens GN=EVPL PE=2 SV=1 - (B7ZLH8_HUMAN)	66.55	16.30	3	2	33	70	1.049E8	2055	233.7	7.25
Q8NGB0	Seven transmembrane helix receptor OS=Homo sapiens GN=GPR142 PE=2 SV=1 - (Q8NGB0_HUMAN)	66.25	5.46	1	1	7	32	8.273E8	1464	156.4	10.95
F8W6W4	Voltage-gated potassium channel subunit beta-1 OS=Homo sapiens GN=KCNAB1 PE=4 SV=1 - (F8W6W4_HUMAN)	65.44	8.33	7	1	3	35	5.269E7	372	41.4	9.41
P27482	Calmodulin-like protein 3 OS=Homo sapiens GN=CALML3 PE=1 SV=2 - (CALL3_HUMAN)	64.57	62.42	1	6	8	27	9.334E7	149	16.9	4.42
Q0Z7S8	Fatty acid-binding protein 9 OS=Homo sapiens GN=FABP9 PE=1 SV=1 - (FABP9_HUMAN)	64.21	87.88	1	12	16	30	4.138E7	132	15.1	8.09
B4DMA2	cDNA FLJ54023, highly similar to Heat shock protein HSP 90-beta OS=Homo sapiens PE=2 SV=1 - (B4DMA2_HUMAN)	63.49	27.11	33	6	19	34	1.716E8	686	79.1	5.02
Q2VIP3	PABP3 (Fragment) OS=Homo sapiens PE=2 SV=1 - (Q2VIP3_HUMAN)	62.56	23.02	23	2	15	51	2.887E8	630	69.9	9.67
H3BST3	Stomatin-like protein 1 OS=Homo sapiens GN=STOML1 PE=4 SV=1 - (H3BST3_HUMAN)	62.24	12.90	8	1	4	33	2.589E8	310	33.4	7.25
P33764	Protein S100-A3 OS=Homo sapiens GN=S100A3 PE=1 SV=1 - (S10A3_HUMAN)	61.32	48.51	1	5	5	26	4.110E7	101	11.7	4.78
Q6MZM0	Hephaestin-like protein 1 OS=Homo sapiens GN=HEPHL1 PE=2 SV=2 - (HPHL1_HUMAN)	60.25	13.98	1	7	10	22	3.090E7	1159	131.5	6.74
Q14574	Desmocollin-3 OS=Homo sapiens GN=DSC3 PE=1 SV=3 - (DSC3_HUMAN)	59.94	15.63	13	10	15	31	6.923E8	896	99.9	6.10
Q13228	Selenium-binding protein 1 OS=Homo sapiens GN=SELENBP1 PE=1 SV=2 - (SBP1_HUMAN)	58.86	32.63	14	10	13	35	3.535E7	472	52.4	6.37
B4DI38	Adenylyl cyclase-associated protein OS=Homo sapiens PE=2 SV=1 - (B4DI38_HUMAN)	58.80	28.54	18	4	11	23	1.371E7	452	49.0	8.21
Q2Q9B7	Glucose-6-phosphate 1-dehydrogenase (Fragment) OS=Homo sapiens GN=G6PD PE=2 SV=1 - (Q2Q9B7_HUMAN)	58.27	13.89	13	1	5	34	1.978E8	475	54.8	7.08
P81605	Dermcidin OS=Homo sapiens GN=DCD PE=1 SV=2 - (DCD_HUMAN)	58.21	46.36	2	3	6	21	2.109E7	110	11.3	6.54
B2R4M6	cDNA, FLJ92148, highly similar to Homo sapiens S100 calcium binding protein A9 (calgranulin B) (S100A9), mRNA OS=Homo sapiens PE=4 SV=1 - (B2R4M6_HUMAN)	58.05	44.74	2	5	8	23	7.149E7	114	13.2	6.13
B7Z7A9	Phosphoglycerate kinase OS=Homo sapiens GN=PGK1 PE=2 SV=1 - (B7Z7A9_HUMAN)	57.17	30.33	8	4	9	26	1.680E8	389	41.4	7.33
O14863	Zinc transporter 4 OS=Homo sapiens GN=SLC30A4 PE=2 SV=2 - (ZNT4_HUMAN)	56.70	4.66	1	1	2	50	5.465E8	429	47.5	6.58
Q93034	Cullin-5 OS=Homo sapiens GN=CUL5 PE=1 SV=4 - (CUL5_HUMAN)	56.63	7.82	1	1	6	36	2.582E8	780	90.9	7.94
H7C469	Uncharacterized protein (Fragment) OS=Homo sapiens PE=3 SV=1 - (H7C469_HUMAN)	55.58	24.27	6	5	7	24	5.998E8	379	40.4	5.76
F8WCP0	Nebulin OS=Homo sapiens GN=NEB PE=4 SV=1 - (F8WCP0_HUMAN)	55.53	9.44	6	1	50	98	5.680E8	8525	986.1	9.00
Q92556	Engulfment and cell motility protein 1 OS=Homo sapiens GN=ELMO1 PE=1 SV=2 - (ELMO1_HUMAN)	54.80	7.02	4	1	5	38	8.195E7	727	83.8	6.28
Q15059	Bromodomain-containing protein 3 OS=Homo sapiens GN=BRD3 PE=1 SV=1 - (BRD3_HUMAN)	53.65	10.06	3	1	7	20	2.961E8	726	79.5	9.36
P49454	Centromere protein F OS=Homo sapiens GN=CENPF PE=1 SV=2 - (CENPF_HUMAN)	53.33	8.69	1	1	25	70	3.533E8	3210	367.5	5.07
P07476	Involucrin OS=Homo sapiens GN=IVL PE=1 SV=2 - (INVO_HUMAN)	53.16	15.73	3	5	8	39	1.491E7	585	68.4	4.61
B4DHC2	cDNA FLJ56434, highly similar to p130Cas-associated protein OS=Homo sapiens PE=2 SV=1 - (B4DHC2_HUMAN)	52.92	11.50	1	1	11	56	1.470E9	1035	111.0	9.41
B9EK46	Cingulin OS=Homo sapiens GN=CGN PE=2 SV=1 - (B9EK46_HUMAN)	52.49	15.05	3	1	18	44	1.932E8	1203	137.0	5.52
Q5T7N8	Protein FAM27D1 OS=Homo sapiens GN=FAM27D1 PE=3 SV=2 - (F27D1_HUMAN)	52.39	21.86	5	1	5	31	1.493E8	215	24.9	11.93
Q2LD37-4	Isoform 4 of Uncharacterized protein KIAA1109 OS=Homo sapiens GN=KIAA1109 - (K1109_HUMAN)	51.99	5.94	6	1	24	50	6.542E8	5004	555.0	6.58
A6NCL7	Ankyrin repeat domain-containing protein 33B OS=Homo sapiens GN=ANKRD33B PE=4 SV=1 - (AN33B_HUMAN)	50.96	16.60	2	1	9	34	2.247E8	494	53.9	8.00
E7EQE3	Telomerase protein component 1 OS=Homo sapiens GN=TEP1 PE=4 SV=1 - (E7EQE3_HUMAN)	50.93	6.72	7	1	14	36	9.565E8	2619	289.3	8.10
Q562Q2	Actin-like protein (Fragment) OS=Homo sapiens GN=ACT PE=3 SV=1 - (Q562Q2_HUMAN)	50.48	33.98	10	1	3	27	2.306E8	103	11.5	6.68
P31949	Protein S100-A11 OS=Homo sapiens GN=S100A11 PE=1 SV=2 - (S10AB_HUMAN)	49.87	56.19	2	6	6	16	3.221E7	105	11.7	7.12
P18206-2	Isoform 1 of Vinculin OS=Homo sapiens GN=VCL - (VINC_HUMAN)	49.63	9.94	8	2	11	32	4.870E7	1066	116.6	6.09
Q9UMY1	Nucleolar protein 7 OS=Homo sapiens GN=NOL7 PE=1 SV=2 - (NOL7_HUMAN)	49.60	12.84	1	1	4	36	5.148E8	257	29.4	9.67
B2RUU3	Dedicator of cytokinesis 1 OS=Homo sapiens GN=DOCK1 PE=2 SV=1 - (B2RUU3_HUMAN)	49.01	8.58	2	1	15	41	1.962E9	1865	215.2	7.49
Q32MZ4-3	Isoform 3 of Leucine-rich repeat flightless-interacting protein 1 OS=Homo sapiens GN=LRRFIP1 - (LRRF1_HUMAN)	48.68	14.36	8	1	9	42	6.797E8	752	82.6	4.61
O95147	Dual specificity protein phosphatase 14 OS=Homo sapiens GN=DUSP14 PE=1 SV=1 - (DUS14_HUMAN)	47.83	36.36	1	4	6	13	1.705E7	198	22.2	9.57
Q9NS87-2	Isoform 2 of Kinesin-like protein KIF15 OS=Homo sapiens GN=KIF15 - (KIF15_HUMAN)	47.38	14.48	5	1	15	30	1.063E8	1291	149.7	6.05
Q9HCD5	Nuclear receptor coactivator 5 OS=Homo sapiens GN=NCOA5 PE=1 SV=2 - (NCOA5_HUMAN)	47.27	10.02	1	1	7	47	5.134E8	579	65.5	9.60
A0PK11	Clarin-2 OS=Homo sapiens GN=CLRN2 PE=2 SV=1 - (CLRN2_HUMAN)	46.79	3.02	1	1	1	37	6.198E8	232	25.4	6.99
Q13813	Spectrin alpha chain, brain OS=Homo sapiens GN=SPTAN1 PE=1 SV=3 - (SPTA2_HUMAN)	46.63	11.89	6	1	22	65	2.587E8	2472	284.4	5.35
Q6YHK3	CD109 antigen OS=Homo sapiens GN=CD109 PE=1 SV=2 - (CD109_HUMAN)	46.61	12.11	4	6	13	21	1.300E8	1445	161.6	5.85
Q15828	Cystatin-M OS=Homo sapiens GN=CST6 PE=1 SV=1 - (CYTM_HUMAN)	46.12	71.14	6	8	9	20	4.886E7	149	16.5	8.09
A8K092	ATP synthase subunit alpha OS=Homo sapiens GN=ATP5A1 PE=2 SV=1 - (A8K092_HUMAN)	46.06	23.66	4	5	9	18	2.980E7	503	54.5	8.24
P30050	60S ribosomal protein L12 OS=Homo sapiens GN=RPL12 PE=1 SV=1 - (RL12_HUMAN)	45.30	60.00	4	6	8	17	1.066E8	165	17.8	9.42
P46782	40S ribosomal protein S5 OS=Homo sapiens GN=RPS5 PE=1 SV=4 - (RS5_HUMAN)	44.96	21.08	2	2	4	14	5.168E7	204	22.9	9.72
Q06830	Peroxiredoxin-1 OS=Homo sapiens GN=PRDX1 PE=1 SV=1 - (PRDX1_HUMAN)	44.90	69.35	4	10	12	25	5.836E7	199	22.1	8.13
O94819	Kelch repeat and BTB domain-containing protein 11 OS=Homo sapiens GN=KBTBD11 PE=1 SV=1 - (KBTBB_HUMAN)	44.79	5.14	1	1	3	23	1.763E8	623	65.7	6.07
Q9Y446	Plakophilin-3 OS=Homo sapiens GN=PKP3 PE=1 SV=1 - (PKP3_HUMAN)	44.75	23.84	5	7	16	31	7.017E7	797	87.0	9.32
B2RBE5	cDNA, FLJ95468, highly similar to Homo sapiens transcriptional coactivator tubedown-100 (TBDN100),transcript variant 1, mRNA OS=Homo sapiens PE=2 SV=1 - (B2RBE5_HUMAN)	44.28	13.39	4	1	9	34	1.258E9	866	101.2	7.42
I0B0K8	Truncated profilaggrin OS=Homo sapiens GN=FLG PE=4 SV=1 - (I0B0K8_HUMAN)	44.12	18.88	16	8	34	70	7.260E8	4021	430.2	9.29
Q14240-2	Isoform 2 of Eukaryotic initiation factor 4A-II OS=Homo sapiens GN=EIF4A2 - (IF4A2_HUMAN)	43.58	26.47	12	2	7	17	1.966E8	408	46.5	5.48
P32926	Desmoglein-3 OS=Homo sapiens GN=DSG3 PE=1 SV=2 - (DSG3_HUMAN)	43.02	10.71	1	6	9	18	4.629E7	999	107.5	5.00
O15173	Membrane-associated progesterone receptor component 2 OS=Homo sapiens GN=PGRMC2 PE=1 SV=1 - (PGRC2_HUMAN)	42.09	6.73	1	1	3	29	4.083E8	223	23.8	4.88
B0AZQ4	cDNA, FLJ79494, highly similar to Structural maintenance of chromosome 3 OS=Homo sapiens PE=2 SV=1 - (B0AZQ4_HUMAN)	41.96	12.82	3	1	13	45	1.717E8	1217	141.4	7.18
D3DTX7	Collagen, type I, alpha 1, isoform CRA_a OS=Homo sapiens GN=COL1A1 PE=4 SV=1 - (D3DTX7_HUMAN)	41.64	9.94	5	3	7	21	1.402E7	885	84.7	6.24
Q5TZA2-2	Isoform 2 of Rootletin OS=Homo sapiens GN=CROCC - (CROCC_HUMAN)	41.19	20.03	6	1	26	50	4.778E8	1313	148.2	5.54
Q6ZQQ6	WD repeat-containing protein 87 OS=Homo sapiens GN=WDR87 PE=2 SV=3 - (WDR87_HUMAN)	41.17	7.83	4	2	24	41	5.115E8	2873	333.0	7.28
Q86SG7	Lysozyme g-like protein 2 OS=Homo sapiens GN=LYG2 PE=1 SV=2 - (LYG2_HUMAN)	40.21	34.43	4	6	6	12	1.920E7	212	23.5	8.87
Q5T6W2	Heterogeneous nuclear ribonucleoprotein K (Fragment) OS=Homo sapiens GN=HNRNPK PE=2 SV=1 - (Q5T6W2_HUMAN)	40.11	29.82	9	3	10	26	7.052E7	379	41.8	5.59
B3KWI4	cDNA FLJ43122 fis, clone CTONG3003737, highly similar to Leucine-rich repeat-containing protein 15 OS=Homo sapiens PE=2 SV=1 - (B3KWI4_HUMAN)	39.98	14.11	3	4	7	19	2.867E7	581	64.4	6.71
Q96AQ8	Coiled-coil domain-containing protein 90A, mitochondrial OS=Homo sapiens GN=CCDC90A PE=2 SV=1 - (CC90A_HUMAN)	39.90	13.37	2	1	4	32	1.035E8	359	39.7	9.63
P07900	Heat shock protein HSP 90-alpha OS=Homo sapiens GN=HSP90AA1 PE=1 SV=5 - (HS90A_HUMAN)	39.88	23.36	30	5	18	30	7.272E7	732	84.6	5.02
A1L390	Pleckstrin homology domain-containing family G member 3 OS=Homo sapiens GN=PLEKHG3 PE=1 SV=1 - (PKHG3_HUMAN)	39.57	3.53	3	1	4	20	5.611E7	1219	134.3	6.55
B2RA91	cDNA, FLJ94773, highly similar to Homo sapiens splicing factor, arginine/serine-rich 2, interacting protein (SFRS2IP), mRNA OS=Homo sapiens PE=2 SV=1 - (B2RA91_HUMAN)	39.21	12.11	4	1	12	42	4.555E7	1148	128.8	8.29
O15230	Laminin subunit alpha-5 OS=Homo sapiens GN=LAMA5 PE=1 SV=8 - (LAMA5_HUMAN)	38.82	4.30	2	1	14	30	8.424E7	3695	399.5	7.02
A4FUJ8	MKL1 protein OS=Homo sapiens GN=MKL1 PE=2 SV=1 - (A4FUJ8_HUMAN)	38.74	5.26	4	1	5	27	1.325E7	798	85.0	7.77
Q12955	Ankyrin-3 OS=Homo sapiens GN=ANK3 PE=1 SV=3 - (ANK3_HUMAN)	38.39	7.70	22	2	32	66	4.685E8	4377	480.1	6.49
Q9BYR4	Keratin-associated protein 4-3 OS=Homo sapiens GN=KRTAP4-3 PE=2 SV=2 - (KRA43_HUMAN)	38.34	5.64	1	1	1	12	2.788E7	195	20.5	7.94
P11021	78 kDa glucose-regulated protein OS=Homo sapiens GN=HSPA5 PE=1 SV=2 - (GRP78_HUMAN)	38.03	22.94	3	5	14	24	8.353E7	654	72.3	5.16
Q96JS0	Tubby homologue (Fragment) OS=Homo sapiens GN=TUB PE=2 SV=1 - (Q96JS0_HUMAN)	37.72	5.09	6	1	3	17	1.023E7	373	40.4	9.23
P28838-2	Isoform 2 of Cytosol aminopeptidase OS=Homo sapiens GN=LAP3 - (AMPL_HUMAN)	37.42	37.91	4	4	13	26	1.223E8	488	52.7	6.74
Q5THJ4	Vacuolar protein sorting-associated protein 13D OS=Homo sapiens GN=VPS13D PE=1 SV=1 - (VP13D_HUMAN)	37.07	5.81	4	1	17	77	2.611E8	4387	491.5	6.58
Q0VF96	Cingulin-like protein 1 OS=Homo sapiens GN=CGNL1 PE=1 SV=2 - (CGNL1_HUMAN)	36.98	13.52	2	1	15	31	4.441E8	1302	149.0	5.67
P16402	Histone H1.3 OS=Homo sapiens GN=HIST1H1D PE=1 SV=2 - (H13_HUMAN)	36.96	40.27	9	5	13	58	2.077E9	221	22.3	11.02
F8WAH5	Laminin subunit alpha-2 OS=Homo sapiens GN=LAMA2 PE=4 SV=1 - (F8WAH5_HUMAN)	36.51	7.72	5	1	21	89	2.585E8	3121	343.5	6.39
Q6ZU59	cDNA FLJ43980 fis, clone TESTI4018806 OS=Homo sapiens PE=2 SV=1 - (Q6ZU59_HUMAN)	36.50	13.29	1	1	2	22	1.278E8	143	16.6	6.80
P22314	Ubiquitin-like modifier-activating enzyme 1 OS=Homo sapiens GN=UBA1 PE=1 SV=3 - (UBA1_HUMAN)	36.21	12.00	7	5	8	14	1.238E7	1058	117.8	5.76
Q8N1C8	HSPA9 protein (Fragment) OS=Homo sapiens GN=HSPA9 PE=2 SV=1 - (Q8N1C8_HUMAN)	35.83	18.50	8	2	12	26	3.063E8	681	73.8	6.37
Q9H9Q2-2	Isoform 2 of COP9 signalosome complex subunit 7b OS=Homo sapiens GN=COPS7B - (CSN7B_HUMAN)	35.80	28.03	6	1	3	20	3.092E7	157	18.1	7.34
Q8WXX0	Dynein heavy chain 7, axonemal OS=Homo sapiens GN=DNAH7 PE=1 SV=2 - (DYH7_HUMAN)	35.34	3.70	1	1	16	30	3.976E8	4024	460.9	6.00
P62277	40S ribosomal protein S13 OS=Homo sapiens GN=RPS13 PE=1 SV=2 - (RS13_HUMAN)	35.25	49.67	2	8	9	14	4.479E7	151	17.2	10.54
P0CG32	Putative zinc finger CCHC domain-containing protein 18 OS=Homo sapiens GN=ZCCHC18 PE=5 SV=1 - (ZCC18_HUMAN)	35.17	9.68	1	1	3	21	2.360E8	403	45.1	7.39
P22392-2	Isoform 3 of Nucleoside diphosphate kinase B OS=Homo sapiens GN=NME2 - (NDKB_HUMAN)	34.54	59.55	10	7	11	20	7.576E7	267	30.1	8.92
G3V388	Coiled-coil domain-containing protein 18 OS=Homo sapiens GN=CCDC18 PE=4 SV=1 - (G3V388_HUMAN)	34.48	13.69	7	1	14	34	1.206E9	1417	163.8	6.06
Q9BU89	Deoxyhypusine hydroxylase OS=Homo sapiens GN=DOHH PE=1 SV=1 - (DOHH_HUMAN)	34.35	2.65	1	1	1	41	1.891E8	302	32.9	4.83
O14777	Kinetochore protein NDC80 homolog OS=Homo sapiens GN=NDC80 PE=1 SV=1 - (NDC80_HUMAN)	34.16	9.50	2	1	5	19	2.275E7	642	73.9	5.60
P04083	Annexin A1 OS=Homo sapiens GN=ANXA1 PE=1 SV=2 - (ANXA1_HUMAN)	34.16	38.73	5	8	11	17	8.558E7	346	38.7	7.02
P00918	Carbonic anhydrase 2 OS=Homo sapiens GN=CA2 PE=1 SV=2 - (CAH2_HUMAN)	34.08	17.31	3	2	3	8	1.003E7	260	29.2	7.40
P35580	Myosin-10 OS=Homo sapiens GN=MYH10 PE=1 SV=3 - (MYH10_HUMAN)	33.92	14.63	12	1	27	51	6.677E8	1976	228.9	5.54
A4QPB0	IQ motif containing GTPase activating protein 1 OS=Homo sapiens GN=IQGAP1 PE=2 SV=1 - (A4QPB0_HUMAN)	33.83	10.32	8	4	14	25	7.038E7	1657	189.2	6.48
F5H0C8	Enolase OS=Homo sapiens GN=ENO2 PE=3 SV=1 - (F5H0C8_HUMAN)	33.33	24.76	12	1	5	11	4.489E7	315	34.7	4.87
Q5HYW3	Retrotransposon gag domain-containing protein 4 OS=Homo sapiens GN=RGAG4 PE=2 SV=1 - (RGAG4_HUMAN)	33.30	11.60	1	1	7	23	1.705E8	569	64.7	4.75
F8VZV5	Myosin light polypeptide 6 OS=Homo sapiens GN=MYL6 PE=4 SV=1 - (F8VZV5_HUMAN)	33.13	50.37	20	5	6	13	3.279E7	135	15.0	5.00
Q8IW70	Transmembrane protein 151B OS=Homo sapiens GN=TMEM151B PE=2 SV=2 - (T151B_HUMAN)	32.95	8.48	1	1	4	18	4.077E7	566	61.5	7.14
P36543-2	Isoform 2 of V-type proton ATPase subunit E 1 OS=Homo sapiens GN=ATP6V1E1 - (VATE1_HUMAN)	32.93	26.96	3	1	6	27	2.081E8	204	23.6	8.98
E5RGV0	Heterogeneous nuclear ribonucleoprotein H, N-terminally processed (Fragment) OS=Homo sapiens GN=HNRNPH1 PE=4 SV=1 - (E5RGV0_HUMAN)	32.92	23.87	11	1	3	15	2.190E8	155	17.5	8.47
F8W7L8	Intersectin-1 OS=Homo sapiens GN=ITSN1 PE=4 SV=1 - (F8W7L8_HUMAN)	32.89	8.55	32	1	17	29	1.688E8	1650	187.6	7.93
Q86YN6-4	Isoform 4 of Peroxisome proliferator-activated receptor gamma coactivator 1-beta OS=Homo sapiens GN=PPARGC1B - (PRGC2_HUMAN)	32.69	8.84	4	1	6	23	1.976E7	996	110.3	5.25
A6H8W8	Intersectin 2 OS=Homo sapiens GN=ITSN2 PE=2 SV=1 - (A6H8W8_HUMAN)	32.61	7.19	10	1	14	36	3.679E7	1669	190.2	8.18
Q96JB1-2	Isoform 2 of Dynein heavy chain 8, axonemal OS=Homo sapiens GN=DNAH8 - (DYH8_HUMAN)	32.50	6.76	3	1	25	39	1.041E8	4454	510.1	6.29
P62269	40S ribosomal protein S18 OS=Homo sapiens GN=RPS18 PE=1 SV=3 - (RS18_HUMAN)	32.36	30.26	2	6	7	14	3.950E7	152	17.7	10.99
B4E1M1	cDNA FLJ60391, highly similar to Lactoperoxidase (EC 1.11.1.7) OS=Homo sapiens PE=2 SV=1 - (B4E1M1_HUMAN)	32.21	6.13	7	1	6	30	4.014E8	653	73.9	8.15
P20592	Interferon-induced GTP-binding protein Mx2 OS=Homo sapiens GN=MX2 PE=1 SV=1 - (MX2_HUMAN)	32.18	6.85	2	1	5	39	1.312E8	715	82.0	8.76
Q5CAQ5	Tumor rejection antigen (Gp96) 1 OS=Homo sapiens GN=TRA1 PE=2 SV=1 - (Q5CAQ5_HUMAN)	32.11	15.09	12	5	12	19	9.756E7	802	92.3	4.86
Q53F18	Homer 1 variant (Fragment) OS=Homo sapiens PE=2 SV=1 - (Q53F18_HUMAN)	32.08	9.04	5	1	5	31	1.558E7	354	40.2	5.44
B7ZKX5	KIF6 protein OS=Homo sapiens GN=KIF6 PE=2 SV=1 - (B7ZKX5_HUMAN)	31.95	9.16	12	2	6	20	7.115E7	797	90.5	6.95
P04075	Fructose-bisphosphate aldolase A OS=Homo sapiens GN=ALDOA PE=1 SV=2 - (ALDOA_HUMAN)	31.55	40.93	17	8	14	19	8.257E7	364	39.4	8.09
P62851	40S ribosomal protein S25 OS=Homo sapiens GN=RPS25 PE=1 SV=1 - (RS25_HUMAN)	31.54	32.80	1	5	6	14	6.894E7	125	13.7	10.11
A8MXQ7	Putative IQ motif and ankyrin repeat domain-containing protein LOC642574 OS=Homo sapiens PE=5 SV=2 - (YH010_HUMAN)	31.49	13.25	1	1	10	24	1.663E7	604	68.3	6.52
P42704	Leucine-rich PPR motif-containing protein, mitochondrial OS=Homo sapiens GN=LRPPRC PE=1 SV=3 - (LPPRC_HUMAN)	31.41	6.31	3	1	7	24	1.786E8	1394	157.8	6.13
P08758	Annexin A5 OS=Homo sapiens GN=ANXA5 PE=1 SV=2 - (ANXA5_HUMAN)	31.20	30.94	7	6	8	12	1.689E7	320	35.9	5.05
P46783	40S ribosomal protein S10 OS=Homo sapiens GN=RPS10 PE=1 SV=1 - (RS10_HUMAN)	31.09	55.76	5	5	11	24	5.326E7	165	18.9	10.15
A2RRR7	CCDC46 protein OS=Homo sapiens GN=CCDC46 PE=2 SV=1 - (A2RRR7_HUMAN)	30.47	14.46	6	1	12	31	2.074E8	913	107.6	6.34
Q6P9G8	Family with sequence similarity 184, member A OS=Homo sapiens GN=FAM184A PE=2 SV=2 - (Q6P9G8_HUMAN)	30.42	13.91	13	2	15	30	7.354E7	1107	129.4	5.72
Q96EA4	Protein Spindly OS=Homo sapiens GN=CCDC99 PE=1 SV=2 - (SPDLY_HUMAN)	30.21	9.42	4	1	6	21	8.241E7	605	70.1	5.47
B2RBR9	cDNA, FLJ95650, highly similar to Homo sapiens karyopherin (importin) beta 1 (KPNB1), mRNA OS=Homo sapiens PE=2 SV=1 - (B2RBR9_HUMAN)	30.02	15.18	6	4	10	15	6.249E7	876	97.1	4.78
O60437	Periplakin OS=Homo sapiens GN=PPL PE=1 SV=4 - (PEPL_HUMAN)	30.00	11.10	1	2	24	34	1.532E8	1756	204.6	5.60
Q9Y2F5	Uncharacterized protein KIAA0947 OS=Homo sapiens GN=KIAA0947 PE=1 SV=5 - (K0947_HUMAN)	29.98	9.05	1	1	16	35	2.812E8	2266	247.7	5.48
Q9H2F9	Coiled-coil domain-containing protein 68 OS=Homo sapiens GN=CCDC68 PE=2 SV=1 - (CCD68_HUMAN)	29.94	14.93	1	1	4	16	2.022E8	335	38.8	8.65
A8K884	cDNA FLJ77178 OS=Homo sapiens PE=2 SV=1 - (A8K884_HUMAN)	29.90	26.86	3	1	17	39	2.495E8	551	65.7	8.70
F5H3I4	Alpha-centractin OS=Homo sapiens GN=ACTR1A PE=3 SV=1 - (F5H3I4_HUMAN)	29.88	9.60	17	1	3	17	9.055E7	302	34.5	6.46
Q99714	3-hydroxyacyl-CoA dehydrogenase type-2 OS=Homo sapiens GN=HSD17B10 PE=1 SV=3 - (HCD2_HUMAN)	29.87	36.78	3	5	6	8	6.725E6	261	26.9	7.78
P31151	Protein S100-A7 OS=Homo sapiens GN=S100A7 PE=1 SV=4 - (S10A7_HUMAN)	29.86	48.51	2	6	6	11	9.200E6	101	11.5	6.77
P45880-2	Isoform 2 of Voltage-dependent anion-selective channel protein 2 OS=Homo sapiens GN=VDAC2 - (VDAC2_HUMAN)	29.74	25.44	7	6	7	19	4.773E7	283	30.4	7.20
P23284	Peptidyl-prolyl cis-trans isomerase B OS=Homo sapiens GN=PPIB PE=1 SV=2 - (PPIB_HUMAN)	29.52	41.67	5	6	9	16	3.531E7	216	23.7	9.41
Q9Y2D5-4	Isoform 2 of A-kinase anchor protein 2 OS=Homo sapiens GN=AKAP2 - (AKAP2_HUMAN)	29.48	4.26	8	1	7	32	3.497E8	1103	122.0	5.06
Q5D862	Filaggrin-2 OS=Homo sapiens GN=FLG2 PE=1 SV=1 - (FILA2_HUMAN)	29.17	10.00	1	4	10	20	3.299E7	2391	247.9	8.31
P04150-2	Isoform Beta of Glucocorticoid receptor OS=Homo sapiens GN=NR3C1 - (GCR_HUMAN)	28.79	9.30	12	1	5	23	4.952E8	742	81.5	6.37
Q5CZC0	Fibrous sheath-interacting protein 2 OS=Homo sapiens GN=FSIP2 PE=1 SV=4 - (FSIP2_HUMAN)	28.76	5.79	4	1	33	55	1.034E8	6907	780.1	6.71
P25787	Proteasome subunit alpha type-2 OS=Homo sapiens GN=PSMA2 PE=1 SV=2 - (PSA2_HUMAN)	28.48	31.62	5	4	5	9	1.674E7	234	25.9	7.43
B4DVQ3	Calcium/calmodulin-dependent protein kinase type II subunit gamma OS=Homo sapiens GN=CAMK2G PE=2 SV=1 - (B4DVQ3_HUMAN)	28.47	12.78	1	1	2	21	1.651E8	180	20.5	8.75
Q3LIE3	Putative uncharacterized protein Nbla04137 (Fragment) OS=Homo sapiens GN=Nbla04137 PE=2 SV=1 - (Q3LIE3_HUMAN)	28.44	10.73	1	1	4	15	1.520E8	289	33.3	5.21
Q5K634	SCCA2/SCCA1 fusion protein isoform 1 OS=Homo sapiens PE=2 SV=1 - (Q5K634_HUMAN)	28.38	25.38	7	1	12	18	3.430E7	390	44.6	6.39
E9PR44	Alpha-crystallin B chain (Fragment) OS=Homo sapiens GN=CRYAB PE=3 SV=1 - (E9PR44_HUMAN)	28.33	63.79	10	8	12	14	2.607E7	174	20.0	7.03
D3DX70	GRIP and coiled-coil domain containing 2, isoform CRA_a OS=Homo sapiens GN=GCC2 PE=4 SV=1 - (D3DX70_HUMAN)	28.09	10.80	2	1	13	26	9.048E7	1583	184.5	5.10
P62136-2	Isoform 2 of Serine/threonine-protein phosphatase PP1-alpha catalytic subunit OS=Homo sapiens GN=PPP1CA - (PP1A_HUMAN)	28.08	19.65	22	3	6	22	7.172E7	341	38.6	6.62
P18669	Phosphoglycerate mutase 1 OS=Homo sapiens GN=PGAM1 PE=1 SV=2 - (PGAM1_HUMAN)	28.07	37.80	14	8	9	12	1.877E7	254	28.8	7.18
Q5T6C5	Ataxin-7-like protein 2 OS=Homo sapiens GN=ATXN7L2 PE=2 SV=1 - (AT7L2_HUMAN)	27.94	12.47	2	1	8	25	4.926E7	722	77.1	9.23
F8VW21	60S acidic ribosomal protein P0 (Fragment) OS=Homo sapiens GN=RPLP0 PE=3 SV=1 - (F8VW21_HUMAN)	27.83	31.97	2	1	9	16	8.909E7	244	26.9	8.97
Q6ZPB4	cDNA FLJ26129 fis, clone TMS02971 OS=Homo sapiens PE=2 SV=1 - (Q6ZPB4_HUMAN)	27.60	17.14	1	1	2	17	2.584E8	140	16.1	6.77
E9PBV3	Suprabasin OS=Homo sapiens GN=SBSN PE=4 SV=1 - (E9PBV3_HUMAN)	27.54	30.34	1	2	8	14	7.508E7	590	60.5	7.01
B2R8Z8	cDNA, FLJ94136, highly similar to Homo sapiens synaptotagmin binding, cytoplasmic RNA interacting protein (SYNCRIP), mRNA OS=Homo sapiens PE=2 SV=1 - (B2R8Z8_HUMAN)	27.51	19.90	10	1	12	25	2.100E9	623	69.6	8.76
B4DMH5	cDNA FLJ55107, highly similar to Cell division control protein 42 homolog OS=Homo sapiens PE=2 SV=1 - (B4DMH5_HUMAN)	27.49	27.90	8	3	5	10	1.051E8	233	26.6	6.90
E5KNB2	DNA-apurinic or apyrimidinic site lyase 2 OS=Homo sapiens PE=4 SV=1 - (E5KNB2_HUMAN)	27.41	3.67	3	1	2	20	2.743E8	518	57.4	8.29
D6W5D1	KIAA1212, isoform CRA_a OS=Homo sapiens GN=KIAA1212 PE=4 SV=1 - (D6W5D1_HUMAN)	27.36	8.00	9	1	11	27	7.000E7	1788	205.9	6.00
B9EG55	GRB10 interacting GYF protein 2 OS=Homo sapiens GN=GIGYF2 PE=2 SV=1 - (B9EG55_HUMAN)	27.27	7.78	22	1	11	30	5.853E7	1298	149.9	5.54
B3KVD6	cDNA FLJ16433 fis, clone BRACE3013936, highly similar to Cell death activator CIDE-B OS=Homo sapiens PE=2 SV=1 - (B3KVD6_HUMAN)	27.26	5.94	2	1	2	32	8.906E8	219	24.8	9.03
P21796	Voltage-dependent anion-selective channel protein 1 OS=Homo sapiens GN=VDAC1 PE=1 SV=2 - (VDAC1_HUMAN)	27.05	27.21	7	5	7	15	2.796E7	283	30.8	8.54
Q96Q89-4	Isoform 4 of Kinesin-like protein KIF20B OS=Homo sapiens GN=KIF20B - (KI20B_HUMAN)	26.93	13.70	1	1	18	45	2.965E8	1722	199.7	5.24
Q15057	Arf-GAP with coiled-coil, ANK repeat and PH domain-containing protein 2 OS=Homo sapiens GN=ACAP2 PE=1 SV=3 - (ACAP2_HUMAN)	26.78	20.69	2	1	13	31	4.768E7	778	88.0	6.80
O96011-2	Isoform 2 of Peroxisomal membrane protein 11B OS=Homo sapiens GN=PEX11B - (PX11B_HUMAN)	26.50	17.96	4	1	4	15	1.229E7	245	26.7	9.91
Q9BYR3	Keratin-associated protein 4-4 OS=Homo sapiens GN=KRTAP4-4 PE=2 SV=1 - (KRA44_HUMAN)	26.47	29.52	1	3	3	15	1.672E7	166	18.0	7.91
Q7Z612	Acidic ribosomal phosphoprotein P1 OS=Homo sapiens PE=4 SV=1 - (Q7Z612_HUMAN)	26.34	14.16	4	1	1	7	8.603E7	113	11.4	4.36
Q3SAH0	G-protein-coupled receptor 34 splice variant OS=Homo sapiens GN=GPR34 PE=2 SV=1 - (Q3SAH0_HUMAN)	26.01	4.49	5	1	2	21	3.797E6	334	38.5	9.83
Q92833	Protein Jumonji OS=Homo sapiens GN=JARID2 PE=1 SV=2 - (JARD2_HUMAN)	25.96	6.34	2	1	7	20	1.134E8	1246	138.6	9.38
B8XPJ7	Soluble catechol-O-methyltransferase OS=Homo sapiens GN=COMT PE=2 SV=1 - (B8XPJ7_HUMAN)	25.86	23.98	10	2	5	7	9.913E6	221	24.5	5.45
B3KX96	cDNA FLJ45003 fis, clone BRAWH3011623, highly similar to Heterogeneous nuclear ribonucleoproteins C OS=Homo sapiens PE=2 SV=1 - (B3KX96_HUMAN)	25.71	29.69	29	3	10	22	3.352E7	293	32.3	5.14
C9JYJ6	Filamin A-interacting protein 1-like (Fragment) OS=Homo sapiens GN=FILIP1L PE=4 SV=1 - (C9JYJ6_HUMAN)	25.62	11.23	7	2	9	23	1.312E8	837	96.2	6.28
Q9BYF7	SCCA2b OS=Homo sapiens GN=SCCA2 PE=2 SV=1 - (Q9BYF7_HUMAN)	25.26	24.93	12	1	11	15	2.878E7	369	42.3	6.05
Q96IR1	RPS4X protein (Fragment) OS=Homo sapiens GN=RPS4X PE=2 SV=2 - (Q96IR1_HUMAN)	25.08	41.15	10	10	12	16	5.214E7	243	27.2	9.94
B3KR97	cDNA FLJ33900 fis, clone CTONG2008262, highly similar to Low density lipoprotein receptor adapter protein 1 OS=Homo sapiens PE=2 SV=1 - (B3KR97_HUMAN)	24.83	6.49	2	1	4	24	1.317E8	308	33.9	6.70
C9JXB8	60S ribosomal protein L24 OS=Homo sapiens GN=RPL24 PE=4 SV=1 - (C9JXB8_HUMAN)	24.71	45.45	3	5	6	14	3.040E7	121	14.4	11.31
Q9Y3P9	Rab GTPase-activating protein 1 OS=Homo sapiens GN=RABGAP1 PE=1 SV=3 - (RBGP1_HUMAN)	24.67	5.05	4	1	5	15	6.255E7	1069	121.7	5.25
O60744	Thioredoxin delta 3 (Fragment) OS=Homo sapiens GN=TXN delta 3 PE=2 SV=1 - (O60744_HUMAN)	24.66	69.05	3	6	7	9	1.342E7	84	9.3	6.04
H0YN65	Carbohydrate sulfotransferase 14 OS=Homo sapiens GN=CHST14 PE=4 SV=1 - (H0YN65_HUMAN)	24.44	10.83	2	1	4	20	3.743E7	351	39.9	9.31
Q92788	Rad GTPase OS=Homo sapiens PE=2 SV=1 - (Q92788_HUMAN)	24.28	19.48	3	1	5	39	2.855E7	308	33.2	8.68
Q15365	Poly(rC)-binding protein 1 OS=Homo sapiens GN=PCBP1 PE=1 SV=2 - (PCBP1_HUMAN)	24.28	15.45	25	3	5	16	9.681E7	356	37.5	7.09
B7Z3T9	cDNA FLJ53345, highly similar to Leucine-zipper-like transcriptional regulator1 OS=Homo sapiens PE=2 SV=1 - (B7Z3T9_HUMAN)	24.09	10.84	5	1	7	18	1.622E8	821	92.3	6.51
O15547	P2X purinoceptor 6 OS=Homo sapiens GN=P2RX6 PE=1 SV=2 - (P2RX6_HUMAN)	24.07	6.58	6	1	2	19	1.012E8	441	48.8	7.58
P16152	Carbonyl reductase (NADPH) 1 OS=Homo sapiens GN=CBR1 PE=1 SV=3 - (CBR1_HUMAN)	24.05	38.99	4	7	9	10	2.837E7	277	30.4	8.32
E0AF97	Cytochrome c oxidase subunit 2 OS=Homo sapiens GN=COX2 PE=3 SV=1 - (E0AF97_HUMAN)	23.99	30.84	177	1	6	11	2.855E7	227	25.5	4.82
Q5T749	Keratinocyte proline-rich protein OS=Homo sapiens GN=KPRP PE=1 SV=1 - (KPRP_HUMAN)	23.99	4.66	1	4	4	17	1.091E7	579	64.1	8.27
I3L0X1	Fatty aldehyde dehydrogenase OS=Homo sapiens GN=ALDH3A2 PE=4 SV=1 - (I3L0X1_HUMAN)	23.66	14.29	4	1	3	20	1.584E8	182	20.7	5.03
Q9NWH9	SAFB-like transcription modulator OS=Homo sapiens GN=SLTM PE=1 SV=2 - (SLTM_HUMAN)	23.59	13.15	3	2	15	31	5.509E7	1034	117.1	7.87
I3L397	Eukaryotic translation initiation factor 5A-1 (Fragment) OS=Homo sapiens GN=EIF5A PE=4 SV=1 - (I3L397_HUMAN)	23.47	40.14	9	4	6	12	3.163E7	147	16.1	5.00
Q8IWB6-3	Isoform 3 of Inactive serine/threonine-protein kinase TEX14 OS=Homo sapiens GN=TEX14 - (TEX14_HUMAN)	23.39	5.93	3	1	7	25	1.641E8	1451	162.6	5.25
Q96IU2	Zinc finger BED domain-containing protein 3 OS=Homo sapiens GN=ZBED3 PE=1 SV=1 - (ZBED3_HUMAN)	23.22	10.26	2	1	4	19	1.420E8	234	25.1	8.31
A8MXJ1	Protein STON1-GTF2A1L OS=Homo sapiens GN=STON1-GTF2A1L PE=4 SV=3 - (A8MXJ1_HUMAN)	23.18	2.64	8	1	3	13	4.661E7	1135	127.2	5.31
Q00610-2	Isoform 2 of Clathrin heavy chain 1 OS=Homo sapiens GN=CLTC - (CLH1_HUMAN)	23.17	10.92	4	2	15	25	1.675E8	1639	187.8	5.69
B4DLJ7	cDNA FLJ59334, highly similar to Transmembrane glycoprotein NMB OS=Homo sapiens PE=2 SV=1 - (B4DLJ7_HUMAN)	22.97	9.53	9	1	3	8	4.039E6	514	57.2	6.77
Q8IZC4-2	Isoform 2 of Rhotekin-2 OS=Homo sapiens GN=RTKN2 - (RTKN2_HUMAN)	22.78	15.37	3	1	5	13	1.269E8	527	60.0	6.61
A8MW50	L-lactate dehydrogenase (Fragment) OS=Homo sapiens GN=LDHB PE=3 SV=1 - (A8MW50_HUMAN)	22.71	15.09	14	2	4	13	2.163E7	232	25.2	5.81
O00571-2	Isoform 2 of ATP-dependent RNA helicase DDX3X OS=Homo sapiens GN=DDX3X - (DDX3X_HUMAN)	22.48	14.55	9	3	7	10	1.183E8	646	71.3	6.62
B4DFX6	cDNA FLJ58791, highly similar to Ankyrin repeat domain-containing protein 35 OS=Homo sapiens PE=2 SV=1 - (B4DFX6_HUMAN)	22.46	14.07	3	1	8	20	2.920E8	910	100.2	5.77
H3BQZ9	Adenine phosphoribosyltransferase OS=Homo sapiens GN=APRT PE=4 SV=1 - (H3BQZ9_HUMAN)	22.38	29.41	7	4	5	9	1.621E7	153	16.6	5.59
Q71F23-2	Isoform 2 of Centromere protein U OS=Homo sapiens GN=MLF1IP - (CENPU_HUMAN)	22.38	32.39	4	1	5	17	2.563E8	176	20.7	9.13
H3BMR2	Heparan sulfate glucosamine 3-O-sulfotransferase 2 OS=Homo sapiens GN=HS3ST2 PE=4 SV=1 - (H3BMR2_HUMAN)	22.29	7.41	5	1	1	9	8.979E7	162	17.5	10.36
Q9H3Q3	Galactose-3-O-sulfotransferase 2 OS=Homo sapiens GN=GAL3ST2 PE=1 SV=2 - (G3ST2_HUMAN)	22.22	14.32	1	1	5	21	1.055E8	398	46.1	10.01
P14314-2	Isoform 2 of Glucosidase 2 subunit beta OS=Homo sapiens GN=PRKCSH - (GLU2B_HUMAN)	22.17	11.24	4	2	5	14	2.389E7	525	59.1	4.42
Q96N67-4	Isoform 4 of Dedicator of cytokinesis protein 7 OS=Homo sapiens GN=DOCK7 - (DOCK7_HUMAN)	22.13	4.62	8	1	8	15	5.891E7	2098	238.1	6.83
A8MUX0	Putative keratin-associated protein 10-like ENSP00000375147 OS=Homo sapiens PE=3 SV=1 - (KR10D_HUMAN)	22.03	4.64	1	1	2	6	1.741E7	517	53.9	5.39
P30101	Protein disulfide-isomerase A3 OS=Homo sapiens GN=PDIA3 PE=1 SV=4 - (PDIA3_HUMAN)	22.00	14.85	5	1	4	15	1.506E8	505	56.7	6.35
Q5VYY1	Ankyrin repeat domain-containing protein 22 OS=Homo sapiens GN=ANKRD22 PE=2 SV=1 - (ANR22_HUMAN)	22.00	19.90	1	3	6	16	6.316E8	191	21.8	8.84
Q96LA8	Protein arginine N-methyltransferase 6 OS=Homo sapiens GN=PRMT6 PE=1 SV=1 - (ANM6_HUMAN)	21.96	6.40	2	1	2	10	3.342E7	375	41.9	5.44
Q86SQ0-2	Isoform 2 of Pleckstrin homology-like domain family B member 2 OS=Homo sapiens GN=PHLDB2 - (PHLB2_HUMAN)	21.84	9.26	11	1	11	21	2.832E8	1210	136.8	7.53
O00186	Syntaxin-binding protein 3 OS=Homo sapiens GN=STXBP3 PE=1 SV=2 - (STXB3_HUMAN)	21.84	7.26	1	1	6	18	1.257E8	592	67.7	7.80
Q0VF49	Uncharacterized protein KIAA2012 OS=Homo sapiens GN=KIAA2012 PE=2 SV=1 - (K2012_HUMAN)	21.79	17.54	2	1	8	21	2.238E7	553	65.1	6.42
Q8TAB0	CLOCK protein (Fragment) OS=Homo sapiens GN=clock PE=2 SV=1 - (Q8TAB0_HUMAN)	21.74	29.41	1	1	2	12	5.292E7	51	6.1	8.85
P30044	Peroxiredoxin-5, mitochondrial OS=Homo sapiens GN=PRDX5 PE=1 SV=4 - (PRDX5_HUMAN)	21.73	44.39	6	5	6	7	6.762E6	214	22.1	8.70
Q9Y6R9	Coiled-coil domain-containing protein 61 OS=Homo sapiens GN=CCDC61 PE=1 SV=2 - (CCD61_HUMAN)	21.62	13.08	1	1	6	18	3.617E7	474	53.2	9.95
B3KVR1	Small nuclear ribonucleoprotein-associated protein OS=Homo sapiens GN=SNRPN PE=2 SV=1 - (B3KVR1_HUMAN)	21.60	27.05	12	2	6	16	2.988E7	244	25.1	11.05
P15090	Fatty acid-binding protein, adipocyte OS=Homo sapiens GN=FABP4 PE=1 SV=3 - (FABP4_HUMAN)	21.56	43.18	1	5	5	8	1.745E7	132	14.7	7.14
E9PRY8	Elongation factor 1-delta OS=Homo sapiens GN=EEF1D PE=3 SV=1 - (E9PRY8_HUMAN)	21.52	14.63	27	2	12	16	9.524E7	697	76.5	7.05
B4DDF9	Annexin OS=Homo sapiens GN=ANXA4 PE=2 SV=1 - (B4DDF9_HUMAN)	21.43	28.69	8	3	7	10	7.683E7	237	27.0	6.06
A8MUD9	60S ribosomal protein L7 OS=Homo sapiens GN=RPL7 PE=3 SV=1 - (A8MUD9_HUMAN)	21.35	35.10	6	4	8	16	2.022E8	208	24.4	10.67
Q8TCG1	Protein CIP2A OS=Homo sapiens GN=KIAA1524 PE=1 SV=2 - (CIP2A_HUMAN)	21.16	10.61	2	2	8	19	2.421E7	905	102.1	6.23
Q86VG2	Splicing factor proline/glutamine-rich (Polypyrimidine tract binding protein associated) OS=Homo sapiens GN=SFPQ PE=2 SV=1 - (Q86VG2_HUMAN)	21.07	17.96	16	3	12	15	5.147E7	707	76.1	9.48
Q9NVR5	Protein kintoun OS=Homo sapiens GN=DNAAF2 PE=1 SV=2 - (KTU_HUMAN)	20.99	13.74	2	1	8	17	1.563E8	837	91.1	5.20
Q9H9L3	Interferon-stimulated 20 kDa exonuclease-like 2 OS=Homo sapiens GN=ISG20L2 PE=1 SV=1 - (I20L2_HUMAN)	20.95	5.10	1	1	2	27	8.568E7	353	39.1	9.94
P49720	Proteasome subunit beta type-3 OS=Homo sapiens GN=PSMB3 PE=1 SV=2 - (PSB3_HUMAN)	20.88	22.44	1	4	5	7	6.969E6	205	22.9	6.55
Q8NE70	Retinoblastoma-like 2 (P130) OS=Homo sapiens GN=RBL2 PE=2 SV=1 - (Q8NE70_HUMAN)	20.39	9.31	4	1	10	19	1.090E8	1139	128.3	7.36
C9K0X2	Uncharacterized protein OS=Homo sapiens PE=4 SV=1 - (C9K0X2_HUMAN)	20.30	5.92	4	2	2	25	1.572E8	152	15.7	6.33
B3KTP9	cDNA FLJ38578 fis, clone HCHON2007674, highly similar to NUCLEOLIN OS=Homo sapiens PE=2 SV=1 - (B3KTP9_HUMAN)	20.13	20.38	7	4	12	17	4.703E7	633	68.4	4.65
Q9BTI9	NPM1 protein (Fragment) OS=Homo sapiens GN=NPM1 PE=2 SV=2 - (Q9BTI9_HUMAN)	20.04	32.02	8	4	6	12	7.005E7	228	25.0	4.86
Q05DB9	PAPOLG protein (Fragment) OS=Homo sapiens GN=PAPOLG PE=2 SV=1 - (Q05DB9_HUMAN)	19.96	10.08	4	1	5	26	6.315E7	496	57.0	8.75
A0MZ66	Shootin-1 OS=Homo sapiens GN=KIAA1598 PE=1 SV=4 - (SHOT1_HUMAN)	19.93	20.29	4	1	11	16	1.035E8	631	71.6	5.33
Q5T2S8	Armadillo repeat-containing protein 4 OS=Homo sapiens GN=ARMC4 PE=2 SV=1 - (ARMC4_HUMAN)	19.89	8.24	5	1	7	22	9.416E7	1044	115.6	7.77
A6NJZ7	RIMS-binding protein 3C OS=Homo sapiens GN=RIMBP3C PE=1 SV=3 - (RIM3C_HUMAN)	19.75	6.53	2	1	12	22	1.957E7	1639	180.8	6.77
Q96L21	60S ribosomal protein L10-like OS=Homo sapiens GN=RPL10L PE=1 SV=3 - (RL10L_HUMAN)	19.37	27.57	8	4	6	14	1.700E7	214	24.5	10.01
Q8NGN2	Olfactory receptor 10S1 OS=Homo sapiens GN=OR10S1 PE=2 SV=2 - (O10S1_HUMAN)	19.22	2.72	1	1	1	14	3.430E8	331	36.5	8.12
B4DUB7	Phosphorylase OS=Homo sapiens PE=2 SV=1 - (B4DUB7_HUMAN)	19.05	15.87	18	4	12	15	2.178E8	813	92.9	7.17
G3XAD8	Stress-induced-phosphoprotein 1 OS=Homo sapiens GN=STIP1 PE=4 SV=1 - (G3XAD8_HUMAN)	18.91	9.66	6	1	5	11	5.852E8	590	68.0	7.74
P29034	Protein S100-A2 OS=Homo sapiens GN=S100A2 PE=1 SV=3 - (S10A2_HUMAN)	18.89	36.73	2	5	5	8	4.225E7	98	11.1	4.78
Q05682	Caldesmon OS=Homo sapiens GN=CALD1 PE=1 SV=3 - (CALD1_HUMAN)	18.83	21.82	20	5	16	20	5.644E8	793	93.2	5.66
F8VU65	60S acidic ribosomal protein P0 (Fragment) OS=Homo sapiens GN=RPLP0 PE=3 SV=1 - (F8VU65_HUMAN)	18.82	41.30	16	1	11	14	3.206E8	247	27.2	9.00
P80188	Neutrophil gelatinase-associated lipocalin OS=Homo sapiens GN=LCN2 PE=1 SV=2 - (NGAL_HUMAN)	18.82	31.82	4	5	6	8	2.262E7	198	22.6	8.91
Q9HCY8	Protein S100-A14 OS=Homo sapiens GN=S100A14 PE=1 SV=1 - (S10AE_HUMAN)	18.76	40.38	1	4	4	7	1.290E7	104	11.7	5.24
Q9HD67	Unconventionnal myosin-X OS=Homo sapiens GN=MYO10 PE=1 SV=3 - (MYO10_HUMAN)	18.61	5.98	6	1	12	18	8.433E7	2058	237.2	6.21
I3L2P8	Protein disulfide-isomerase OS=Homo sapiens GN=P4HB PE=4 SV=1 - (I3L2P8_HUMAN)	18.45	8.22	13	2	4	12	1.805E7	450	51.0	4.91
P63244	Guanine nucleotide-binding protein subunit beta-2-like 1 OS=Homo sapiens GN=GNB2L1 PE=1 SV=3 - (GBLP_HUMAN)	18.44	25.24	24	5	7	9	3.020E7	317	35.1	7.69
Q6NS36	Ferritin (Fragment) OS=Homo sapiens GN=FTH1 PE=2 SV=1 - (Q6NS36_HUMAN)	18.32	13.79	10	1	3	45	1.208E8	232	26.2	6.52
Q6NXR8	Ribosomal protein S3A OS=Homo sapiens GN=RPS3A PE=2 SV=1 - (Q6NXR8_HUMAN)	18.30	35.23	16	5	12	23	1.694E7	264	30.0	9.73
P30041	Peroxiredoxin-6 OS=Homo sapiens GN=PRDX6 PE=1 SV=3 - (PRDX6_HUMAN)	18.28	29.46	3	5	6	10	1.923E7	224	25.0	6.38
P60900	Proteasome subunit alpha type-6 OS=Homo sapiens GN=PSMA6 PE=1 SV=1 - (PSA6_HUMAN)	18.15	28.86	11	6	8	11	1.548E8	246	27.4	6.76
P50395-2	Isoform 2 of Rab GDP dissociation inhibitor beta OS=Homo sapiens GN=GDI2 - (GDIB_HUMAN)	18.05	15.50	11	2	5	7	1.314E7	400	45.6	6.24
B4DSG0	Janus kinase and microtubule-interacting protein 2 OS=Homo sapiens GN=JAKMIP2 PE=2 SV=1 - (B4DSG0_HUMAN)	17.95	16.97	3	1	13	76	3.136E8	778	91.5	5.78
P39019	40S ribosomal protein S19 OS=Homo sapiens GN=RPS19 PE=1 SV=2 - (RS19_HUMAN)	17.90	51.03	2	6	10	10	1.137E7	145	16.1	10.32
H0Y4M1	EF-hand calcium-binding domain-containing protein 5 OS=Homo sapiens GN=EFCAB5 PE=4 SV=1 - (H0Y4M1_HUMAN)	17.90	6.89	1	1	6	14	2.142E7	856	98.9	6.20
B2R6V2	cDNA, FLJ93132, highly similar to Homo sapiens CDC7 cell division cycle 7 (S. cerevisiae) (CDC7), mRNA OS=Homo sapiens PE=2 SV=1 - (B2R6V2_HUMAN)	17.80	8.71	4	1	6	14	7.815E7	574	63.8	8.81
P0CW22	40S ribosomal protein S17-like OS=Homo sapiens GN=RPS17L PE=3 SV=1 - (RS17L_HUMAN)	17.76	59.26	6	4	5	8	1.379E7	135	15.5	9.85
Q6ZV29-3	Isoform 3 of Patatin-like phospholipase domain-containing protein 7 OS=Homo sapiens GN=PNPLA7 - (PLPL7_HUMAN)	17.70	7.89	7	1	7	13	7.827E7	1254	138.8	7.75
Q8WUQ7	Uncharacterized protein C19orf29 OS=Homo sapiens GN=C19orf29 PE=1 SV=3 - (CS029_HUMAN)	17.67	20.32	4	1	14	34	1.675E8	758	88.6	9.14
F8VXI9	ARF GTPase-activating protein GIT2 OS=Homo sapiens GN=GIT2 PE=4 SV=1 - (F8VXI9_HUMAN)	17.58	6.78	1	1	4	23	3.030E8	708	78.8	7.46
B7ZLP0	DOCK8 protein OS=Homo sapiens GN=DOCK8 PE=2 SV=1 - (B7ZLP0_HUMAN)	17.43	5.35	14	2	12	28	6.374E7	1999	227.3	6.74
B4DXS5	cDNA FLJ57802, highly similar to Zinc finger protein 225 OS=Homo sapiens PE=2 SV=1 - (B4DXS5_HUMAN)	17.29	6.27	1	1	4	12	5.169E7	670	78.1	8.92
H0YG24	Coiled-coil domain-containing protein 151 OS=Homo sapiens GN=CCDC151 PE=4 SV=1 - (H0YG24_HUMAN)	17.13	10.80	6	1	6	12	6.429E6	574	66.9	8.91
O00299	Chloride intracellular channel protein 1 OS=Homo sapiens GN=CLIC1 PE=1 SV=4 - (CLIC1_HUMAN)	17.08	21.99	2	3	5	6	1.375E7	241	26.9	5.17
A7BI36	p180/ribosome receptor OS=Homo sapiens GN=RRBP1 PE=2 SV=2 - (A7BI36_HUMAN)	16.89	13.25	7	1	16	19	1.123E8	1540	165.6	8.97
Q7KZ85	Transcription elongation factor SPT6 OS=Homo sapiens GN=SUPT6H PE=1 SV=2 - (SPT6H_HUMAN)	16.75	5.74	2	1	10	17	5.037E7	1726	198.9	4.91
Q92830	Histone acetyltransferase KAT2A OS=Homo sapiens GN=KAT2A PE=1 SV=3 - (KAT2A_HUMAN)	16.75	10.16	2	1	6	18	1.593E8	837	93.9	9.04
Q5T0Z8	Uncharacterized protein C6orf132 OS=Homo sapiens GN=C6orf132 PE=1 SV=4 - (CF132_HUMAN)	16.63	5.13	1	1	5	12	1.707E7	1188	124.0	9.45
Q86VP6-2	Isoform 2 of Cullin-associated NEDD8-dissociated protein 1 OS=Homo sapiens GN=CAND1 - (CAND1_HUMAN)	16.60	10.17	4	3	6	10	1.805E8	1062	117.8	5.69
Q9NVE4	Coiled-coil domain-containing protein 87 OS=Homo sapiens GN=CCDC87 PE=2 SV=2 - (CCD87_HUMAN)	16.59	4.00	1	1	5	45	7.561E8	849	96.3	8.59
F8W8F5	Calpain-3 OS=Homo sapiens GN=CAPN3 PE=4 SV=1 - (F8W8F5_HUMAN)	16.52	6.99	9	1	7	14	8.479E7	815	93.5	5.91
O75629	Protein CREG1 OS=Homo sapiens GN=CREG1 PE=1 SV=1 - (CREG1_HUMAN)	16.48	14.09	1	1	2	8	1.843E7	220	24.1	7.59
Q13867	Bleomycin hydrolase OS=Homo sapiens GN=BLMH PE=1 SV=1 - (BLMH_HUMAN)	16.47	12.31	3	2	5	10	2.768E7	455	52.5	6.27
H7C0E8	Probable phospholipid-transporting ATPase IG (Fragment) OS=Homo sapiens GN=ATP11C PE=4 SV=1 - (H7C0E8_HUMAN)	16.44	24.75	5	1	5	23	2.311E9	202	22.9	5.10
Q5VUA4	Zinc finger protein 318 OS=Homo sapiens GN=ZNF318 PE=1 SV=2 - (ZN318_HUMAN)	16.43	10.18	2	1	21	26	6.169E7	2279	251.0	7.20
Q8N7B9	EF-hand calcium-binding domain-containing protein 3 OS=Homo sapiens GN=EFCAB3 PE=1 SV=1 - (EFCB3_HUMAN)	16.40	13.93	3	1	4	9	7.564E7	438	50.1	9.22
Q9UJL9	Zinc finger protein 643 OS=Homo sapiens GN=ZNF643 PE=2 SV=2 - (ZN643_HUMAN)	16.25	12.92	5	1	5	10	7.102E7	534	61.4	9.01
E5RIY5	Serine protease 45 OS=Homo sapiens GN=PRSS45 PE=3 SV=1 - (E5RIY5_HUMAN)	16.24	8.18	3	1	1	8	1.047E8	110	12.4	7.59
P52272-2	Isoform 2 of Heterogeneous nuclear ribonucleoprotein M OS=Homo sapiens GN=HNRNPM - (HNRPM_HUMAN)	16.11	21.13	7	4	14	26	5.793E8	691	73.6	8.82
P12236	ADP/ATP translocase 3 OS=Homo sapiens GN=SLC25A6 PE=1 SV=4 - (ADT3_HUMAN)	16.06	19.46	9	3	6	13	9.012E7	298	32.8	9.74
Q9UKK3	Poly (ADP-ribose) polymerase 4 OS=Homo sapiens GN=PARP4 PE=1 SV=3 - (PARP4_HUMAN)	16.02	4.99	1	1	6	20	6.465E7	1724	192.5	5.66
Q9UJX2	Cell division cycle protein 23 homolog OS=Homo sapiens GN=CDC23 PE=1 SV=3 - (CDC23_HUMAN)	15.96	7.54	5	2	6	32	1.481E8	597	68.8	7.02
D6RF73	Aryl hydrocarbon receptor repressor OS=Homo sapiens GN=AHRR PE=4 SV=1 - (D6RF73_HUMAN)	15.95	10.79	5	1	4	10	1.924E8	547	59.4	8.57
P05091	Aldehyde dehydrogenase, mitochondrial OS=Homo sapiens GN=ALDH2 PE=1 SV=2 - (ALDH2_HUMAN)	15.93	14.12	8	2	7	13	2.327E7	517	56.3	7.05
A8K2Q8	Serpin peptidase inhibitor, clade B (Ovalbumin), member 13, isoform CRA_d OS=Homo sapiens GN=SERPINB13 PE=2 SV=1 - (A8K2Q8_HUMAN)	15.87	17.39	11	4	9	23	1.868E7	391	44.3	5.71
E7ENJ7	Translin-associated factor X-interacting protein 1 OS=Homo sapiens GN=TSNAXIP1 PE=4 SV=1 - (E7ENJ7_HUMAN)	15.84	14.46	6	1	9	22	4.408E8	643	74.0	5.27
P11047	Laminin subunit gamma-1 OS=Homo sapiens GN=LAMC1 PE=1 SV=3 - (LAMC1_HUMAN)	15.79	9.32	1	1	13	20	5.127E7	1609	177.5	5.12
Q08188	Protein-glutamine gamma-glutamyltransferase E OS=Homo sapiens GN=TGM3 PE=1 SV=4 - (TGM3_HUMAN)	15.74	17.03	3	4	10	13	3.941E7	693	76.6	5.86
D3DTT5	TBK1 binding protein 1, isoform CRA_a OS=Homo sapiens GN=TBKBP1 PE=4 SV=1 - (D3DTT5_HUMAN)	15.72	11.23	4	1	6	24	8.813E7	463	50.3	5.26
Q96FL8	Multidrug and toxin extrusion protein 1 OS=Homo sapiens GN=SLC47A1 PE=1 SV=1 - (S47A1_HUMAN)	15.65	5.61	8	1	3	11	1.147E8	570	61.9	7.58
P12111-2	Isoform 2 of Collagen alpha-3(VI) chain OS=Homo sapiens GN=COL6A3 - (CO6A3_HUMAN)	15.59	7.30	13	2	15	20	9.333E7	2971	321.2	7.30
B4DE24	cDNA FLJ52155, moderately similar to Rattus norvegicus ring finger protein 34 (Rnf34), mRNA OS=Homo sapiens PE=2 SV=1 - (B4DE24_HUMAN)	15.56	3.21	2	1	1	10	5.314E8	280	31.4	8.38
H0YBX3	Ribonuclease UK114 (Fragment) OS=Homo sapiens GN=HRSP12 PE=4 SV=1 - (H0YBX3_HUMAN)	15.55	51.97	4	4	5	5	3.355E6	127	13.8	9.31
P68402	Platelet-activating factor acetylhydrolase IB subunit beta OS=Homo sapiens GN=PAFAH1B2 PE=1 SV=1 - (PA1B2_HUMAN)	15.55	8.30	1	1	2	8	1.923E7	229	25.6	5.92
B3KX72	Heterogeneous nuclear ribonucleoprotein U OS=Homo sapiens GN=HNRNPU PE=2 SV=1 - (B3KX72_HUMAN)	15.52	15.87	8	1	13	28	3.245E8	750	83.0	8.79
Q9HBI0-3	Isoform 3 of Gamma-parvin OS=Homo sapiens GN=PARVG - (PARVG_HUMAN)	15.44	7.49	3	1	1	7	7.831E7	187	21.4	5.47
Q52LG2	Keratin-associated protein 13-2 OS=Homo sapiens GN=KRTAP13-2 PE=1 SV=1 - (KR132_HUMAN)	15.39	52.57	1	5	5	5	1.599E7	175	18.7	8.29
A4GWZ0	MHC class I antigen (Fragment) OS=Homo sapiens GN=HLA-A PE=3 SV=1 - (A4GWZ0_HUMAN)	15.36	16.48	250	1	3	12	2.107E8	273	31.5	6.19
C9J1I8	Transcription factor TFIIIB component B'' homolog OS=Homo sapiens GN=BDP1 PE=4 SV=2 - (C9J1I8_HUMAN)	15.34	9.38	9	1	18	51	6.417E7	2132	238.2	5.00
B4DMK0	cDNA FLJ61134, highly similar to Ras-related protein Rab-11B OS=Homo sapiens PE=2 SV=1 - (B4DMK0_HUMAN)	15.31	29.61	12	4	5	6	1.290E7	179	20.0	5.96
Q4ZFX3	Putative uncharacterized protein VSNL1 (Fragment) OS=Homo sapiens GN=VSNL1 PE=2 SV=1 - (Q4ZFX3_HUMAN)	15.30	27.74	4	3	3	4	8.285E6	137	15.7	4.98
Q0D2K2	Kelch-like protein 30 OS=Homo sapiens GN=KLHL30 PE=2 SV=3 - (KLH30_HUMAN)	15.25	9.52	1	1	3	9	3.582E7	578	63.9	5.94
I3L3P7	40S ribosomal protein S15a OS=Homo sapiens GN=RPS15A PE=4 SV=1 - (I3L3P7_HUMAN)	15.23	45.00	4	4	6	9	1.157E7	100	11.5	10.15
O00221	NF-kappa-B inhibitor epsilon OS=Homo sapiens GN=NFKBIE PE=1 SV=3 - (IKBE_HUMAN)	15.22	4.40	6	1	2	9	3.928E6	500	52.8	6.68
A8MYX0	Protein diaphanous homolog 3 OS=Homo sapiens GN=DIAPH3 PE=4 SV=3 - (A8MYX0_HUMAN)	15.18	9.53	14	1	11	26	1.338E8	1123	128.9	7.11
H0YMZ1	Proteasome subunit alpha type (Fragment) OS=Homo sapiens GN=PSMA4 PE=3 SV=1 - (H0YMZ1_HUMAN)	15.14	18.10	11	1	4	14	7.382E6	221	24.6	6.77
Q9BTQ7	Similar to ribosomal protein L23 (Fragment) OS=Homo sapiens PE=1 SV=1 - (Q9BTQ7_HUMAN)	15.11	32.09	4	3	3	6	1.029E7	134	14.1	10.26
O95229	ZW10 interactor OS=Homo sapiens GN=ZWINT PE=1 SV=2 - (ZWINT_HUMAN)	15.07	19.86	2	1	7	24	5.315E8	277	31.3	5.15
I3L4A2	Leucine carboxyl methyltransferase 1 (Fragment) OS=Homo sapiens GN=LCMT1 PE=4 SV=1 - (I3L4A2_HUMAN)	15.06	6.96	3	1	1	6	1.837E8	158	18.2	5.60
Q96M42	Putative uncharacterized protein encoded by LINC00479 OS=Homo sapiens GN=LINC00479 PE=5 SV=2 - (CU129_HUMAN)	15.05	30.28	1	1	3	19	5.349E7	142	15.2	8.00
H0Y2N2	Ribonuclease 3 OS=Homo sapiens GN=DROSHA PE=4 SV=1 - (H0Y2N2_HUMAN)	15.04	3.62	7	1	6	15	5.421E8	1299	150.4	7.65
B4DXW2	cDNA FLJ60947, highly similar to Coiled-coil domain-containing protein 9 OS=Homo sapiens PE=2 SV=1 - (B4DXW2_HUMAN)	14.92	1.36	1	1	1	13	1.417E8	513	58.0	5.21
B2R717	cDNA, FLJ93235, highly similar to Homo sapiens cathepsin L2 (CTSL2), mRNA OS=Homo sapiens PE=2 SV=1 - (B2R717_HUMAN)	14.89	17.37	2	2	4	9	3.542E7	334	37.3	8.76
P62857	40S ribosomal protein S28 OS=Homo sapiens GN=RPS28 PE=1 SV=1 - (RS28_HUMAN)	14.89	46.38	1	3	3	5	8.555E6	69	7.8	10.70
D6REM6	Matrin-3 OS=Homo sapiens GN=MATR3 PE=4 SV=1 - (D6REM6_HUMAN)	14.85	14.36	15	1	10	30	2.369E8	794	88.3	5.76
P05090	Apolipoprotein D OS=Homo sapiens GN=APOD PE=1 SV=1 - (APOD_HUMAN)	14.83	15.34	4	2	3	6	1.175E7	189	21.3	5.15
Q96L12	Calreticulin-3 OS=Homo sapiens GN=CALR3 PE=1 SV=2 - (CALR3_HUMAN)	14.79	4.95	1	1	2	13	3.103E8	384	45.0	6.65
Q4W4Y1	Dopamine receptor interacting protein 4 OS=Homo sapiens GN=DRIP4 PE=2 SV=1 - (Q4W4Y1_HUMAN)	14.77	7.95	6	2	7	12	1.842E7	868	96.0	6.52
Q5SW79	Centrosomal protein of 170 kDa OS=Homo sapiens GN=CEP170 PE=1 SV=1 - (CE170_HUMAN)	14.75	10.10	8	1	11	19	4.478E8	1584	175.2	7.11
P62249	40S ribosomal protein S16 OS=Homo sapiens GN=RPS16 PE=1 SV=2 - (RS16_HUMAN)	14.74	23.97	2	3	4	8	9.844E6	146	16.4	10.21
Q6IPM2-4	Isoform 4 of IQ domain-containing protein E OS=Homo sapiens GN=IQCE - (IQCE_HUMAN)	14.70	13.70	14	1	12	21	1.981E8	679	75.6	9.32
A4D218	MAD1 mitotic arrest deficient-like 1 (Yeast) OS=Homo sapiens GN=MAD1L1 PE=4 SV=1 - (A4D218_HUMAN)	14.57	20.05	6	1	18	43	6.887E7	803	91.7	8.10
Q86UK0	ATP-binding cassette sub-family A member 12 OS=Homo sapiens GN=ABCA12 PE=1 SV=3 - (ABCAC_HUMAN)	14.57	7.63	2	1	15	18	4.262E7	2595	293.0	7.75
P06753	Tropomyosin alpha-3 chain OS=Homo sapiens GN=TPM3 PE=1 SV=1 - (TPM3_HUMAN)	14.52	27.46	42	1	7	10	1.818E7	284	32.8	4.72
B2RDE1	cDNA, FLJ96568, highly similar to Homo sapiens tropomyosin 3 (TPM3), mRNA OS=Homo sapiens PE=2 SV=1 - (B2RDE1_HUMAN)	14.52	36.29	59	2	9	12	1.938E7	248	29.0	4.75
P35321	Cornifin-A OS=Homo sapiens GN=SPRR1A PE=1 SV=2 - (SPR1A_HUMAN)	14.52	46.07	5	3	3	8	5.003E6	89	9.9	8.48
A7KAX9	Rho GTPase-activating protein 32 OS=Homo sapiens GN=ARHGAP32 PE=1 SV=1 - (RHG32_HUMAN)	14.49	3.93	8	1	7	18	1.468E8	2087	230.4	6.74
Q5JS13-2	Isoform 2 of Ras-specific guanine nucleotide-releasing factor RalGPS1 OS=Homo sapiens GN=RALGPS1 - (RGPS1_HUMAN)	14.48	10.02	3	1	5	29	1.315E8	529	58.9	9.26
Q68D86	Coiled-coil domain-containing protein 102B OS=Homo sapiens GN=CCDC102B PE=2 SV=4 - (C102B_HUMAN)	14.21	14.04	3	1	8	14	3.158E8	513	60.4	5.95
P98088	Mucin-5AC (Fragments) OS=Homo sapiens GN=MUC5AC PE=1 SV=3 - (MUC5A_HUMAN)	14.20	2.96	2	1	8	21	5.804E7	5030	526.3	6.90
D3DRN8	Putative uncharacterized protein LOC158381 (Fragment) OS=Homo sapiens GN=LOC158381 PE=4 SV=1 - (D3DRN8_HUMAN)	14.12	6.74	1	1	2	8	9.975E7	178	19.4	8.63
Q99797	Mitochondrial intermediate peptidase OS=Homo sapiens GN=MIPEP PE=1 SV=2 - (MIPEP_HUMAN)	14.09	13.60	1	1	7	8	5.908E7	713	80.6	7.05
Q9P1F3	Costars family protein ABRACL OS=Homo sapiens GN=ABRACL PE=1 SV=1 - (ABRAL_HUMAN)	14.05	27.16	1	3	3	4	7.375E5	81	9.1	6.29
P18621	60S ribosomal protein L17 OS=Homo sapiens GN=RPL17 PE=1 SV=3 - (RL17_HUMAN)	14.04	41.30	4	5	11	18	4.690E7	184	21.4	10.17
Q9ULZ3-2	Isoform 2 of Apoptosis-associated speck-like protein containing a CARD OS=Homo sapiens GN=PYCARD - (ASC_HUMAN)	13.99	26.14	5	3	4	5	2.145E6	176	20.0	5.95
P62913-2	Isoform 2 of 60S ribosomal protein L11 OS=Homo sapiens GN=RPL11 - (RL11_HUMAN)	13.99	32.20	5	4	6	7	4.905E7	177	20.1	9.60
Q5VWI1	Transcription elongation regulator 1-like protein OS=Homo sapiens GN=TCERG1L PE=2 SV=2 - (TCRGL_HUMAN)	13.95	7.68	1	1	4	11	3.387E7	586	65.6	9.83
H0YHU6	Uncharacterized protein OS=Homo sapiens PE=4 SV=1 - (H0YHU6_HUMAN)	13.94	14.84	2	1	2	27	5.012E8	128	14.3	9.92
E7EVA0	Microtubule-associated protein OS=Homo sapiens GN=MAP4 PE=4 SV=1 - (E7EVA0_HUMAN)	13.93	7.58	8	1	15	27	2.152E8	2297	245.3	6.23
B4E343	cDNA FLJ54249, highly similar to U3 small nucleolar RNA-associated protein 14 homolog A OS=Homo sapiens PE=2 SV=1 - (B4E343_HUMAN)	13.86	17.57	6	1	13	25	3.380E7	717	82.1	8.94
Q9Y3D6	Mitochondrial fission 1 protein OS=Homo sapiens GN=FIS1 PE=1 SV=2 - (FIS1_HUMAN)	13.81	19.08	1	1	4	10	2.036E6	152	16.9	8.79
Q6QEF7	Breast cancer anti-estrogen resistance 1 OS=Homo sapiens GN=BCAR1 PE=2 SV=1 - (Q6QEF7_HUMAN)	13.69	3.83	21	1	4	13	8.970E8	888	95.3	5.99
O60447	Ecotropic viral integration site 5 protein homolog OS=Homo sapiens GN=EVI5 PE=1 SV=3 - (EVI5_HUMAN)	13.63	13.46	4	2	8	19	1.602E8	810	92.9	6.10
Q8NEM0	Microcephalin OS=Homo sapiens GN=MCPH1 PE=1 SV=3 - (MCPH1_HUMAN)	13.60	9.94	5	1	7	10	2.850E7	835	92.8	8.25
Q9ULW8	Protein-arginine deiminase type-3 OS=Homo sapiens GN=PADI3 PE=1 SV=2 - (PADI3_HUMAN)	13.43	5.72	2	1	3	5	9.050E6	664	74.7	5.54
O15085-2	Isoform 2 of Rho guanine nucleotide exchange factor 11 OS=Homo sapiens GN=ARHGEF11 - (ARHGB_HUMAN)	13.34	6.85	2	1	11	17	9.351E7	1562	172.1	5.54
P62847-2	Isoform 2 of 40S ribosomal protein S24 OS=Homo sapiens GN=RPS24 - (RS24_HUMAN)	13.27	59.23	5	4	8	10	5.058E7	130	15.1	10.89
Q96L91-4	Isoform 4 of E1A-binding protein p400 OS=Homo sapiens GN=EP400 - (EP400_HUMAN)	13.22	5.29	5	1	17	22	6.824E7	3042	331.6	9.09
P62266	40S ribosomal protein S23 OS=Homo sapiens GN=RPS23 PE=1 SV=3 - (RS23_HUMAN)	13.22	20.98	1	1	3	5	5.480E6	143	15.8	10.49
P08195-2	Isoform 2 of 4F2 cell-surface antigen heavy chain OS=Homo sapiens GN=SLC3A2 - (4F2_HUMAN)	13.17	16.82	7	1	7	9	3.637E7	529	57.9	5.35
Q9Y4W2-3	Isoform 3 of Ribosomal biogenesis protein LAS1L OS=Homo sapiens GN=LAS1L - (LAS1L_HUMAN)	13.15	8.59	3	1	5	9	1.504E7	675	76.8	4.68
P62854	40S ribosomal protein S26 OS=Homo sapiens GN=RPS26 PE=1 SV=3 - (RS26_HUMAN)	13.08	27.83	6	2	3	6	1.059E7	115	13.0	11.00
Q9UMN6	Histone-lysine N-methyltransferase MLL4 OS=Homo sapiens GN=WBP7 PE=1 SV=1 - (MLL4_HUMAN)	12.90	7.00	1	1	19	39	2.224E8	2715	293.3	8.22
Q8NHS4-2	Isoform 2 of Uncharacterized protein C2orf63 OS=Homo sapiens GN=C2orf63 - (CB063_HUMAN)	12.83	7.33	2	1	4	18	5.252E7	464	52.9	5.69
Q96IV0-3	Isoform 3 of Peptide-N(4)-(N-acetyl-beta-glucosaminyl)asparagine amidase OS=Homo sapiens GN=NGLY1 - (NGLY1_HUMAN)	12.75	3.23	4	1	2	6	2.783E7	558	63.8	8.10
G3XAM7	Catenin (Cadherin-associated protein), alpha 1, 102kDa, isoform CRA_a OS=Homo sapiens GN=CTNNA1 PE=4 SV=1 - (G3XAM7_HUMAN)	12.74	9.99	18	2	9	12	1.474E8	841	92.7	5.52
Q14997	Proteasome activator complex subunit 4 OS=Homo sapiens GN=PSME4 PE=1 SV=2 - (PSME4_HUMAN)	12.72	6.35	5	1	12	22	9.436E7	1843	211.2	6.90
E7EUY0	DNA-dependent protein kinase catalytic subunit OS=Homo sapiens GN=PRKDC PE=4 SV=1 - (E7EUY0_HUMAN)	12.71	6.37	4	1	22	35	5.869E7	4096	465.1	7.17
Q14191	Werner syndrome ATP-dependent helicase OS=Homo sapiens GN=WRN PE=1 SV=2 - (WRN_HUMAN)	12.70	7.40	2	1	9	25	2.639E8	1432	162.4	6.34
Q63HQ8	Putative uncharacterized protein DKFZp686M21196 OS=Homo sapiens GN=DKFZp686M21196 PE=2 SV=1 - (Q63HQ8_HUMAN)	12.69	19.81	5	1	11	22	1.742E8	530	58.5	9.82
B7ZBG9	Eukaryotic translation initiation factor 6 OS=Homo sapiens GN=EIF6 PE=3 SV=1 - (B7ZBG9_HUMAN)	12.68	16.37	2	1	2	9	1.189E7	226	23.7	5.17
H7BY10	60S ribosomal protein L23a (Fragment) OS=Homo sapiens GN=RPL23A PE=3 SV=1 - (H7BY10_HUMAN)	12.68	25.95	5	4	5	6	2.356E7	158	17.8	10.45
P12273	Prolactin-inducible protein OS=Homo sapiens GN=PIP PE=1 SV=1 - (PIP_HUMAN)	12.55	17.81	1	2	2	3	3.075E6	146	16.6	8.05
H0YFA4	Cysteine-rich protein 2 (Fragment) OS=Homo sapiens GN=CRIP2 PE=4 SV=1 - (H0YFA4_HUMAN)	12.45	14.06	4	1	2	6	1.255E7	192	20.7	8.94
Q53RT3	Retroviral-like aspartic protease 1 OS=Homo sapiens GN=ASPRV1 PE=1 SV=1 - (APRV1_HUMAN)	12.36	3.79	1	1	1	4	1.343E7	343	37.0	5.44
C9J9K3	40S ribosomal protein SA (Fragment) OS=Homo sapiens GN=RPSA PE=3 SV=1 - (C9J9K3_HUMAN)	12.33	17.80	11	2	4	5	4.447E6	264	29.5	5.25
E7EPB3	60S ribosomal protein L14 OS=Homo sapiens GN=RPL14 PE=4 SV=1 - (E7EPB3_HUMAN)	12.31	46.77	7	4	6	10	1.376E8	124	14.5	10.21
Q6P084	MAP3K2 protein (Fragment) OS=Homo sapiens GN=MAP3K2 PE=2 SV=1 - (Q6P084_HUMAN)	12.30	13.68	1	1	3	9	2.649E7	234	26.8	9.23
G1UI17	Glycogen debranching enzyme (Fragment) OS=Homo sapiens GN=AGL PE=2 SV=1 - (G1UI17_HUMAN)	12.29	6.81	4	1	8	9	2.945E8	1262	143.6	6.64
P62081	40S ribosomal protein S7 OS=Homo sapiens GN=RPS7 PE=1 SV=1 - (RS7_HUMAN)	12.26	37.63	3	3	5	6	3.214E6	194	22.1	10.10
P26641	Elongation factor 1-gamma OS=Homo sapiens GN=EEF1G PE=1 SV=3 - (EF1G_HUMAN)	12.25	7.32	5	2	3	5	4.519E6	437	50.1	6.67
B7Z848	DNA-binding protein RFX5 OS=Homo sapiens GN=RFX5 PE=2 SV=1 - (B7Z848_HUMAN)	12.24	11.81	6	1	6	16	4.525E8	576	60.5	9.57
Q9BT92	Trichoplein keratin filament-binding protein OS=Homo sapiens GN=TCHP PE=1 SV=1 - (TCHP_HUMAN)	12.17	18.27	1	1	9	22	1.471E7	498	61.0	6.54
H0Y5J4	Drebrin-like protein (Fragment) OS=Homo sapiens GN=DBNL PE=4 SV=1 - (H0Y5J4_HUMAN)	12.15	20.89	5	1	6	24	4.928E7	359	40.2	5.10
B3KXY9	cDNA FLJ46359 fis, clone TESTI4049786, highly similar to Hexokinase-1 (EC 2.7.1.1) OS=Homo sapiens PE=2 SV=1 - (B3KXY9_HUMAN)	12.07	14.81	10	1	12	19	1.894E8	952	106.2	7.17
B4DNP0	cDNA FLJ59362, highly similar to Signal transducer and activator of transcription 3 OS=Homo sapiens PE=2 SV=1 - (B4DNP0_HUMAN)	12.01	7.29	7	1	6	25	1.018E8	672	76.1	6.30
Q53SF7-2	Isoform 2 of Cordon-bleu protein-like 1 OS=Homo sapiens GN=COBLL1 - (COBL1_HUMAN)	11.96	10.29	6	1	8	13	1.126E8	1166	127.6	6.95
G3V533	Nesprin-3 OS=Homo sapiens GN=C14orf49 PE=4 SV=1 - (G3V533_HUMAN)	11.95	9.29	10	1	4	10	1.097E8	732	83.9	6.65
Q15126	Phosphomevalonate kinase OS=Homo sapiens GN=PMVK PE=1 SV=3 - (PMVK_HUMAN)	11.94	22.92	1	3	5	11	2.836E7	192	22.0	5.73
Q8IUC6	TIR domain-containing adapter molecule 1 OS=Homo sapiens GN=TICAM1 PE=1 SV=1 - (TCAM1_HUMAN)	11.93	9.13	1	1	5	12	4.598E7	712	76.4	5.41
Q5T024	Centrosomal protein C10orf90 OS=Homo sapiens GN=RP11-422P15.2 PE=2 SV=1 - (Q5T024_HUMAN)	11.91	16.71	1	1	6	10	2.743E7	383	42.0	8.43
Q9P2Z0	THAP domain-containing protein 10 OS=Homo sapiens GN=THAP10 PE=2 SV=1 - (THA10_HUMAN)	11.82	14.79	2	1	4	12	9.689E6	257	28.3	8.60
G5EA45	Disheveled-associated activator of morphogenesis 2 OS=Homo sapiens GN=DAAM2 PE=4 SV=1 - (G5EA45_HUMAN)	11.65	8.81	2	1	7	14	6.917E7	1067	123.3	6.86
P61626	Lysozyme C OS=Homo sapiens GN=LYZ PE=1 SV=1 - (LYSC_HUMAN)	11.63	26.35	2	2	4	9	3.177E6	148	16.5	9.16
B2R9T9	cDNA, FLJ94551 OS=Homo sapiens PE=2 SV=1 - (B2R9T9_HUMAN)	11.61	15.64	4	1	4	5	8.993E6	243	26.2	10.67
C9JTA6	SWI/SNF-related matrix-associated actin-dependent regulator of chromatin subfamily B member 1 (Fragment) OS=Homo sapiens GN=SMARCB1 PE=4 SV=1 - (C9JTA6_HUMAN)	11.53	11.89	6	1	2	10	2.925E7	227	25.9	8.92
B3KRS1	cDNA FLJ34794 fis, clone NT2NE2005676, highly similar to Gamma-tubulin complex component 2 OS=Homo sapiens PE=2 SV=1 - (B3KRS1_HUMAN)	11.52	10.40	7	1	6	10	5.192E7	721	82.5	6.84
Q9Y2U5	Mitogen-activated protein kinase kinase kinase 2 OS=Homo sapiens GN=MAP3K2 PE=1 SV=2 - (M3K2_HUMAN)	11.48	9.21	2	1	5	32	2.579E7	619	69.7	8.00
A8MWV3	Osteoclast-associated immunoglobulin-like receptor OS=Homo sapiens GN=OSCAR PE=4 SV=1 - (A8MWV3_HUMAN)	11.38	34.78	1	1	4	11	3.152E7	161	16.4	12.04
P98161-2	Isoform 2 of Polycystin-1 OS=Homo sapiens GN=PKD1 - (PKD1_HUMAN)	11.34	3.75	10	2	15	25	8.934E7	4292	461.1	6.77
P61970	Nuclear transport factor 2 OS=Homo sapiens GN=NUTF2 PE=1 SV=1 - (NTF2_HUMAN)	11.33	40.16	2	3	4	6	4.409E6	127	14.5	5.38
P20618	Proteasome subunit beta type-1 OS=Homo sapiens GN=PSMB1 PE=1 SV=2 - (PSB1_HUMAN)	11.33	20.75	4	1	4	7	3.936E6	241	26.5	8.13
P30086	Phosphatidylethanolamine-binding protein 1 OS=Homo sapiens GN=PEBP1 PE=1 SV=3 - (PEBP1_HUMAN)	11.32	32.09	2	3	3	3	1.105E7	187	21.0	7.53
P60866	40S ribosomal protein S20 OS=Homo sapiens GN=RPS20 PE=1 SV=1 - (RS20_HUMAN)	11.32	36.13	5	4	5	5	2.133E7	119	13.4	9.94
P48047	ATP synthase subunit O, mitochondrial OS=Homo sapiens GN=ATP5O PE=1 SV=1 - (ATPO_HUMAN)	11.28	25.35	4	3	4	5	1.391E7	213	23.3	9.96
B4DEA8	cDNA FLJ56425, highly similar to Very-long-chain specific acyl-CoAdehydrogenase, mitochondrial (EC 1.3.99.-) OS=Homo sapiens PE=2 SV=1 - (B4DEA8_HUMAN)	11.27	8.27	9	2	5	8	2.667E7	701	75.2	8.88
Q59H49	Polypyrimidine tract-binding protein 1 isoform c variant (Fragment) OS=Homo sapiens PE=2 SV=1 - (Q59H49_HUMAN)	11.26	19.76	4	1	3	5	8.243E7	329	35.5	8.98
F5GX67	Keratin-associated protein 1-5 OS=Homo sapiens GN=KRTAP1-5 PE=4 SV=1 - (F5GX67_HUMAN)	11.20	5.49	2	1	2	8	8.530E7	164	17.0	7.06
H0YL91	Activating signal cointegrator 1 OS=Homo sapiens GN=TRIP4 PE=4 SV=1 - (H0YL91_HUMAN)	11.16	7.61	2	1	3	6	1.491E8	368	42.0	7.21
Q8NEY1-2	Isoform 2 of Neuron navigator 1 OS=Homo sapiens GN=NAV1 - (NAV1_HUMAN)	11.13	6.69	9	1	11	19	9.461E7	1869	201.4	8.22
F8VUA6	60S ribosomal protein L18 (Fragment) OS=Homo sapiens GN=RPL18 PE=4 SV=1 - (F8VUA6_HUMAN)	11.09	34.62	6	2	4	6	3.498E7	130	14.5	11.75
B2R608	cDNA, FLJ92718, highly similar to Homo sapiens tripeptidyl peptidase I (TPP1), mRNA OS=Homo sapiens PE=2 SV=1 - (B2R608_HUMAN)	11.09	10.48	9	1	4	8	1.835E7	563	61.2	6.52
P29373	Cellular retinoic acid-binding protein 2 OS=Homo sapiens GN=CRABP2 PE=1 SV=2 - (RABP2_HUMAN)	11.09	43.48	2	3	5	7	3.668E7	138	15.7	5.40
Q04760-2	Isoform 2 of Lactoylglutathione lyase OS=Homo sapiens GN=GLO1 - (LGUL_HUMAN)	11.09	18.34	2	2	5	9	7.893E7	169	19.0	6.05
H7BZW3	Voltage-dependent calcium channel subunit alpha-2-3 OS=Homo sapiens GN=CACNA2D3 PE=4 SV=1 - (H7BZW3_HUMAN)	11.09	2.51	1	1	1	14	1.293E8	518	59.4	5.86
F5H110	Zinc finger CCCH domain-containing protein 6 OS=Homo sapiens GN=ZC3H6 PE=4 SV=1 - (F5H110_HUMAN)	11.07	10.65	3	1	7	15	7.801E7	892	100.7	8.82
B2R983	cDNA, FLJ94267, highly similar to Homo sapiens glutathione S-transferase omega 1 (GSTO1), mRNA OS=Homo sapiens PE=2 SV=1 - (B2R983_HUMAN)	11.03	17.43	4	1	5	9	1.023E7	241	27.5	6.60
Q96AG4	Leucine-rich repeat-containing protein 59 OS=Homo sapiens GN=LRRC59 PE=1 SV=1 - (LRC59_HUMAN)	10.94	15.31	1	1	5	10	4.028E7	307	34.9	9.57
A4D2P2	Ras-related C3 botulinum toxin substrate 1 (Rho family, small GTP binding protein Rac1) OS=Homo sapiens GN=RAC1 PE=3 SV=1 - (A4D2P2_HUMAN)	10.94	37.16	8	5	5	5	1.245E7	148	16.8	8.91
Q5T0V4	Slit homolog 1 (Drosophila) (Fragment) OS=Homo sapiens GN=SLIT1 PE=2 SV=1 - (Q5T0V4_HUMAN)	10.94	16.24	6	1	9	19	1.129E9	782	87.1	8.56
P00568	Adenylate kinase isoenzyme 1 OS=Homo sapiens GN=AK1 PE=1 SV=3 - (KAD1_HUMAN)	10.83	15.46	4	1	3	4	9.928E7	194	21.6	8.63
B3KUD4	cDNA FLJ39594 fis, clone SKNSH2001875, highly similar to Ribonuclease H1 (EC 3.1.26.4) OS=Homo sapiens PE=2 SV=1 - (B3KUD4_HUMAN)	10.77	22.49	2	1	2	11	6.526E7	169	18.8	8.60
A8K8P3-10	Isoform 10 of Protein SFI1 homolog OS=Homo sapiens GN=SFI1 - (SFI1_HUMAN)	10.74	13.33	11	1	18	21	1.688E8	1148	136.6	10.71
P01011	Alpha-1-antichymotrypsin OS=Homo sapiens GN=SERPINA3 PE=1 SV=2 - (AACT_HUMAN)	10.73	18.68	4	2	5	8	1.614E8	423	47.6	5.52
Q9P153	PRO2729 OS=Homo sapiens PE=2 SV=1 - (Q9P153_HUMAN)	10.69	9.03	1	1	2	11	6.700E7	144	15.6	8.65
Q59G27	Stathmin (Fragment) OS=Homo sapiens PE=2 SV=1 - (Q59G27_HUMAN)	10.67	29.85	20	2	3	6	9.718E6	134	15.1	8.27
H0YDT6	Eukaryotic translation initiation factor 3 subunit F (Fragment) OS=Homo sapiens GN=EIF3F PE=4 SV=1 - (H0YDT6_HUMAN)	10.61	19.00	5	1	1	2	1.814E6	100	10.8	5.81
F8VR50	Actin-related protein 2/3 complex subunit 3 (Fragment) OS=Homo sapiens GN=ARPC3 PE=4 SV=1 - (F8VR50_HUMAN)	10.61	32.14	6	3	3	3	1.730E7	84	9.7	7.93
B3KXQ9	cDNA FLJ45894 fis, clone OCBBF3023913, highly similar to R3H domain-containing protein 1 OS=Homo sapiens PE=2 SV=1 - (B3KXQ9_HUMAN)	10.60	7.47	11	1	8	24	2.550E8	1044	114.8	8.78
F5H018	GTP-binding nuclear protein Ran (Fragment) OS=Homo sapiens GN=RAN PE=4 SV=1 - (F5H018_HUMAN)	10.60	16.08	4	2	3	5	2.875E7	199	22.5	8.73
P20290	Transcription factor BTF3 OS=Homo sapiens GN=BTF3 PE=1 SV=1 - (BTF3_HUMAN)	10.57	21.84	8	3	5	5	7.152E7	206	22.2	9.38
A2A3R5	40S ribosomal protein S6 OS=Homo sapiens GN=RPS6 PE=3 SV=1 - (A2A3R5_HUMAN)	10.54	16.51	3	1	4	8	1.102E7	218	25.0	11.14
B3KUZ8	Aspartate aminotransferase OS=Homo sapiens PE=2 SV=1 - (B3KUZ8_HUMAN)	10.48	22.91	5	2	7	11	2.203E8	371	41.3	8.84
P10266	HERV-K_5q33.3 provirus ancestral Pol protein OS=Homo sapiens PE=3 SV=2 - (POK10_HUMAN)	10.46	9.57	1	1	6	23	1.358E8	1014	114.8	8.95
Q9NY97	UDP-GlcNAc:betaGal beta-1,3-N-acetylglucosaminyltransferase 2 OS=Homo sapiens GN=B3GNT2 PE=1 SV=2 - (B3GN2_HUMAN)	10.44	11.34	2	1	3	6	7.511E7	397	46.0	8.54
B4DJQ8	cDNA FLJ55694, highly similar to Dipeptidyl-peptidase 1 (EC 3.4.14.1) OS=Homo sapiens PE=2 SV=1 - (B4DJQ8_HUMAN)	10.37	8.97	4	3	4	7	1.581E7	446	50.1	6.99
Q9Y6N8	Cadherin-10 OS=Homo sapiens GN=CDH10 PE=1 SV=2 - (CAD10_HUMAN)	10.37	6.47	1	1	3	14	7.243E7	788	88.4	4.98
Q5VU13	V-set and immunoglobulin domain-containing protein 8 OS=Homo sapiens GN=VSIG8 PE=1 SV=1 - (VSIG8_HUMAN)	10.34	8.21	1	2	3	7	6.442E6	414	43.9	7.20
Q07DS4	Envelope glycoprotein OS=Homo sapiens GN=env PE=4 SV=1 - (Q07DS4_HUMAN)	10.32	9.59	1	1	4	13	1.946E7	438	49.6	8.50
Q8TAS0	ATP synthase gamma chain (Fragment) OS=Homo sapiens PE=2 SV=1 - (Q8TAS0_HUMAN)	10.28	16.15	5	3	5	6	3.378E7	291	32.2	9.11
H0YH34	ADP-ribosylation factor-like protein 13A (Fragment) OS=Homo sapiens GN=ARL13A PE=4 SV=1 - (H0YH34_HUMAN)	10.27	26.61	3	1	5	8	1.516E8	124	14.2	9.64
H7BXN2	Heterogeneous nuclear ribonucleoprotein D0 OS=Homo sapiens GN=HNRNPD PE=4 SV=1 - (H7BXN2_HUMAN)	10.26	15.30	18	2	4	6	1.728E7	281	30.8	8.76
P22626-2	Isoform A2 of Heterogeneous nuclear ribonucleoproteins A2/B1 OS=Homo sapiens GN=HNRNPA2B1 - (ROA2_HUMAN)	10.16	20.82	2	4	5	6	7.373E6	341	36.0	8.65
O75146	Huntingtin-interacting protein 1-related protein OS=Homo sapiens GN=HIP1R PE=1 SV=2 - (HIP1R_HUMAN)	10.00	15.36	1	1	14	18	1.328E8	1068	119.3	6.67
Q7Z5A3	Small nuclear ribonucleoprotein Sm D1 OS=Homo sapiens GN=SNRPD1 PE=2 SV=1 - (Q7Z5A3_HUMAN)	9.98	23.53	2	1	2	3	4.705E7	119	13.3	11.56
Q15008	26S proteasome non-ATPase regulatory subunit 6 OS=Homo sapiens GN=PSMD6 PE=1 SV=1 - (PSMD6_HUMAN)	9.96	15.68	7	1	4	7	1.620E7	389	45.5	5.62
P19105	Myosin regulatory light chain 12A OS=Homo sapiens GN=MYL12A PE=1 SV=2 - (ML12A_HUMAN)	9.95	27.49	8	2	4	7	1.853E7	171	19.8	4.81
B4DUI2	cDNA FLJ61415, highly similar to Protein kinase C and casein kinase substratein neurons protein 3 OS=Homo sapiens PE=2 SV=1 - (B4DUI2_HUMAN)	9.87	9.26	4	1	5	12	2.972E7	421	48.2	6.39
Q8N1G2	Cap-specific mRNA (nucleoside-2'-O-)-methyltransferase 1 OS=Homo sapiens GN=FTSJD2 PE=1 SV=1 - (MTR1_HUMAN)	9.86	3.95	1	1	3	8	8.769E7	835	95.3	7.05
Q96E11-7	Isoform 7 of Ribosome-recycling factor, mitochondrial OS=Homo sapiens GN=MRRF - (RRFM_HUMAN)	9.78	32.26	7	1	3	6	1.154E8	124	13.6	9.57
P28702	Retinoic acid receptor RXR-beta OS=Homo sapiens GN=RXRB PE=1 SV=2 - (RXRB_HUMAN)	9.75	3.94	5	1	2	7	4.649E7	533	56.9	8.18
P40939	Trifunctional enzyme subunit alpha, mitochondrial OS=Homo sapiens GN=HADHA PE=1 SV=2 - (ECHA_HUMAN)	9.69	12.45	5	4	10	10	1.385E7	763	82.9	9.04
Q6AI18	Putative uncharacterized protein DKFZp686M148 (Fragment) OS=Homo sapiens GN=DKFZp686M148 PE=2 SV=1 - (Q6AI18_HUMAN)	9.69	33.55	13	3	4	8	1.691E8	155	16.3	8.56
B4DPW9	cDNA FLJ51929, highly similar to Plastin-3 OS=Homo sapiens PE=2 SV=1 - (B4DPW9_HUMAN)	9.55	13.43	10	1	5	7	1.564E7	603	67.6	5.66
C9J592	Ras-related protein Rab-7a (Fragment) OS=Homo sapiens GN=RAB7A PE=3 SV=1 - (C9J592_HUMAN)	9.53	36.00	8	2	4	7	5.098E7	150	17.0	8.85
P07108-4	Isoform 4 of Acyl-CoA-binding protein OS=Homo sapiens GN=DBI - (ACBP_HUMAN)	9.47	32.56	8	2	4	16	4.463E7	129	14.4	9.42
B1AHC7	X-ray repair complementing defective repair in Chinese hamster cells 6 (Ku autoantigen, 70kDa) OS=Homo sapiens GN=XRCC6 PE=4 SV=1 - (B1AHC7_HUMAN)	9.46	7.54	8	1	3	5	2.799E7	557	64.0	6.83
B2RBF9	cDNA, FLJ95487, highly similar to Homo sapiens peptidyl arginine deiminase, type I (PADI1), mRNA OS=Homo sapiens PE=2 SV=1 - (B2RBF9_HUMAN)	9.45	12.37	2	1	9	14	9.047E7	663	74.6	6.49
Q92928	Putative Ras-related protein Rab-1C OS=Homo sapiens GN=RAB1C PE=5 SV=2 - (RAB1C_HUMAN)	9.45	24.88	9	1	3	7	3.859E7	201	22.0	5.43
Q3LI72	Keratin-associated protein 19-5 OS=Homo sapiens GN=KRTAP19-5 PE=1 SV=1 - (KR195_HUMAN)	9.45	22.22	1	1	1	2	2.510E7	72	7.6	8.13
P31944	Caspase-14 OS=Homo sapiens GN=CASP14 PE=1 SV=2 - (CASPE_HUMAN)	9.38	20.25	2	1	5	9	2.587E7	242	27.7	5.58
Q9UGU0-2	Isoform 2 of Transcription factor 20 OS=Homo sapiens GN=TCF20 - (TCF20_HUMAN)	9.38	6.66	3	1	10	23	1.078E8	1938	209.5	9.09
Q9P0L2	Serine/threonine-protein kinase MARK1 OS=Homo sapiens GN=MARK1 PE=1 SV=2 - (MARK1_HUMAN)	9.30	13.21	5	1	8	21	1.049E8	795	88.9	9.36
P15822	Zinc finger protein 40 OS=Homo sapiens GN=HIVEP1 PE=1 SV=3 - (ZEP1_HUMAN)	9.18	4.97	6	1	12	25	2.091E9	2718	296.7	7.84
Q86YL3	INM04 OS=Homo sapiens PE=2 SV=1 - (Q86YL3_HUMAN)	9.16	5.44	1	1	1	5	5.075E7	147	16.1	9.44
B2RBL3	cDNA, FLJ95575, highly similar to Homo sapiens endothelial cell growth factor 1 (platelet-derived) (ECGF1), mRNA OS=Homo sapiens PE=2 SV=1 - (B2RBL3_HUMAN)	9.14	29.25	4	2	8	10	7.268E7	482	49.9	5.53
Q9Y5H0	Protocadherin gamma-A3 OS=Homo sapiens GN=PCDHGA3 PE=2 SV=2 - (PCDG3_HUMAN)	9.14	6.65	6	1	4	9	1.964E7	932	100.9	5.00
B2R8A2	cDNA, FLJ93804, highly similar to Homo sapiens gp25L2 protein (HSGP25L2G), mRNA OS=Homo sapiens PE=2 SV=1 - (B2R8A2_HUMAN)	9.14	18.22	2	2	5	6	6.584E6	214	25.1	7.18
Q9BYR2	Keratin-associated protein 4-5 OS=Homo sapiens GN=KRTAP4-5 PE=2 SV=4 - (KRA45_HUMAN)	9.12	15.47	4	1	1	3	7.134E7	181	19.3	7.78
B4DY09	cDNA FLJ51660, highly similar to Interleukin enhancer-binding factor 2 OS=Homo sapiens PE=2 SV=1 - (B4DY09_HUMAN)	9.05	17.05	4	1	3	6	7.172E7	352	38.9	4.94
F8VSZ4	Plexin-A1 OS=Homo sapiens GN=PLXNA1 PE=4 SV=1 - (F8VSZ4_HUMAN)	9.04	3.84	2	1	7	12	7.198E7	1873	208.4	6.87
Q0D2H7	PCF11 protein (Fragment) OS=Homo sapiens GN=PCF11 PE=2 SV=1 - (Q0D2H7_HUMAN)	9.02	8.86	1	1	1	7	1.293E8	158	17.0	8.02
A8K8P1	cDNA FLJ78752, highly similar to Homo sapiens centromere protein J (CENPJ), mRNA OS=Homo sapiens PE=2 SV=1 - (A8K8P1_HUMAN)	9.00	8.15	5	1	10	42	3.497E8	1338	152.9	6.61
Q13535-2	Isoform 2 of Serine/threonine-protein kinase ATR OS=Homo sapiens GN=ATR - (ATR_HUMAN)	8.94	7.64	3	1	16	32	1.303E8	2580	294.0	7.43
P05387	60S acidic ribosomal protein P2 OS=Homo sapiens GN=RPLP2 PE=1 SV=1 - (RLA2_HUMAN)	8.88	33.91	1	2	2	3	5.223E6	115	11.7	4.54
Q7Z353	Highly divergent homeobox OS=Homo sapiens GN=HDX PE=1 SV=1 - (HDX_HUMAN)	8.82	4.64	2	1	3	12	6.031E7	690	77.2	5.87
P26373	60S ribosomal protein L13 OS=Homo sapiens GN=RPL13 PE=1 SV=4 - (RL13_HUMAN)	8.75	26.54	4	4	7	13	8.637E7	211	24.2	11.65
B4DPU7	cDNA FLJ53623, moderately similar to Homo sapiens cytidylate kinase (CMPK), mRNA OS=Homo sapiens PE=2 SV=1 - (B4DPU7_HUMAN)	8.75	31.84	8	2	4	4	1.564E6	179	20.3	8.32
D6RD18	Heterogeneous nuclear ribonucleoprotein A/B OS=Homo sapiens GN=HNRNPAB PE=4 SV=1 - (D6RD18_HUMAN)	8.73	30.39	9	1	6	8	1.928E7	283	30.5	7.91
P14174	Macrophage migration inhibitory factor OS=Homo sapiens GN=MIF PE=1 SV=4 - (MIF_HUMAN)	8.70	13.91	2	2	2	3	2.695E7	115	12.5	7.88
B4DTD3	cDNA FLJ50209, highly similar to DNA-repair protein XRCC1 OS=Homo sapiens PE=2 SV=1 - (B4DTD3_HUMAN)	8.66	3.65	6	1	2	10	6.622E7	602	66.0	5.85
B4DLZ5	N-acetylglucosamine kinase OS=Homo sapiens GN=NAGK PE=2 SV=1 - (B4DLZ5_HUMAN)	8.65	13.33	7	1	5	10	4.312E6	390	42.0	6.68
A8K321	cDNA FLJ78524, highly similar to Homo sapiens SMILE protein (SMILE), mRNA OS=Homo sapiens PE=2 SV=1 - (A8K321_HUMAN)	8.58	8.34	4	1	7	20	2.648E8	899	102.0	8.84
P62318	Small nuclear ribonucleoprotein Sm D3 OS=Homo sapiens GN=SNRPD3 PE=1 SV=1 - (SMD3_HUMAN)	8.42	15.08	2	1	2	5	1.093E7	126	13.9	10.32
Q86UP2-3	Isoform 3 of Kinectin OS=Homo sapiens GN=KTN1 - (KTN1_HUMAN)	8.40	9.19	4	1	12	21	1.900E8	1306	150.3	5.74
E7ETU9	Procollagen-lysine,2-oxoglutarate 5-dioxygenase 2 OS=Homo sapiens GN=PLOD2 PE=4 SV=1 - (E7ETU9_HUMAN)	8.40	10.38	5	1	6	13	4.073E7	703	81.1	6.65
P13489	Ribonuclease inhibitor OS=Homo sapiens GN=RNH1 PE=1 SV=2 - (RINI_HUMAN)	8.40	17.57	2	1	6	7	2.470E7	461	49.9	4.82
B7Z948	cDNA FLJ53589, highly similar to Tyrosine-protein phosphatase non-receptor type3 (EC 3.1.3.48) OS=Homo sapiens PE=2 SV=1 - (B7Z948_HUMAN)	8.32	8.06	13	1	5	12	1.102E8	868	98.8	6.84
P17900	Ganglioside GM2 activator OS=Homo sapiens GN=GM2A PE=1 SV=4 - (SAP3_HUMAN)	8.29	19.17	1	2	3	3	5.556E6	193	20.8	5.31
A8K781	cDNA FLJ75299, highly similar to Xenopus laevis proteasome (prosome, macropain) 26S subunit, ATPase 3, mRNA OS=Homo sapiens PE=2 SV=1 - (A8K781_HUMAN)	8.19	14.89	8	1	6	12	4.872E7	423	47.3	5.22
Q96FQ6	Protein S100-A16 OS=Homo sapiens GN=S100A16 PE=1 SV=1 - (S10AG_HUMAN)	8.13	36.89	1	3	3	3	2.005E6	103	11.8	6.79
Q16630	Cleavage and polyadenylation specificity factor subunit 6 OS=Homo sapiens GN=CPSF6 PE=1 SV=2 - (CPSF6_HUMAN)	8.12	11.25	5	1	4	6	3.051E7	551	59.2	7.15
B4DJV9	cDNA FLJ60607, highly similar to Acyl-protein thioesterase 1 (EC 3.1.2.-) OS=Homo sapiens PE=2 SV=1 - (B4DJV9_HUMAN)	8.10	14.45	6	1	2	3	2.991E6	263	28.3	7.64
Q9P265	Disco-interacting protein 2 homolog B OS=Homo sapiens GN=DIP2B PE=1 SV=3 - (DIP2B_HUMAN)	8.01	4.31	2	1	6	7	6.024E7	1576	171.4	8.09
P46779-2	Isoform 2 of 60S ribosomal protein L28 OS=Homo sapiens GN=RPL28 - (RL28_HUMAN)	7.96	33.74	9	4	5	7	1.972E7	163	18.4	10.78
P62280	40S ribosomal protein S11 OS=Homo sapiens GN=RPS11 PE=1 SV=3 - (RS11_HUMAN)	7.91	20.89	1	3	4	6	5.558E6	158	18.4	10.30
H0Y9N1	Programmed cell death protein 6 (Fragment) OS=Homo sapiens GN=PDCD6 PE=4 SV=1 - (H0Y9N1_HUMAN)	7.91	43.70	4	2	5	7	5.464E6	135	15.6	8.19
A4QN25	NBPF6 protein (Fragment) OS=Homo sapiens GN=NBPF6 PE=2 SV=1 - (A4QN25_HUMAN)	7.87	21.29	7	1	8	16	6.551E7	573	64.8	4.82
B4E363	Phenylalanine--tRNA ligase alpha subunit OS=Homo sapiens GN=FARSA PE=2 SV=1 - (B4E363_HUMAN)	7.87	11.11	2	1	6	9	8.905E7	477	54.1	7.80
Q7L5L3-1	Isoform 2 of Glycerophosphodiester phosphodiesterase domain-containing protein 3 OS=Homo sapiens GN=GDPD3 - (GDPD3_HUMAN)	7.81	12.50	2	1	3	4	9.891E6	256	29.6	7.39
I3L4X2	Multidrug resistance-associated protein 1 (Fragment) OS=Homo sapiens GN=ABCC1 PE=4 SV=1 - (I3L4X2_HUMAN)	7.77	5.63	15	1	7	12	3.707E7	1440	160.4	6.74
B4DW36	cDNA FLJ58862, highly similar to THUMP domain-containing protein 2 OS=Homo sapiens PE=2 SV=1 - (B4DW36_HUMAN)	7.74	10.69	5	1	3	9	3.492E7	290	34.0	8.81
G3V1B3	60S ribosomal protein L21 OS=Homo sapiens GN=RPL21 PE=4 SV=1 - (G3V1B3_HUMAN)	7.71	49.43	3	1	6	7	1.275E7	87	9.9	10.29
Q96QA5	Gasdermin-A OS=Homo sapiens GN=GSDMA PE=2 SV=4 - (GSDMA_HUMAN)	7.65	8.54	1	2	3	5	3.138E6	445	49.3	5.29
A8MXN1	Receptor expression-enhancing protein 6 OS=Homo sapiens GN=REEP6 PE=4 SV=1 - (A8MXN1_HUMAN)	7.64	7.08	1	1	3	34	6.852E7	480	49.7	11.24
A2RRP1	Neuroblastoma-amplified sequence OS=Homo sapiens GN=NBAS PE=1 SV=2 - (NBAS_HUMAN)	7.64	7.04	3	1	15	28	1.875E8	2371	268.4	5.96
Q9ULC4-2	Isoform 2 of Malignant T-cell-amplified sequence 1 OS=Homo sapiens GN=MCTS1 - (MCTS1_HUMAN)	7.63	14.79	3	1	2	2	9.493E5	169	19.2	8.25
Q5T8Y7	COMM domain containing 3 (Fragment) OS=Homo sapiens GN=COMMD3 PE=2 SV=1 - (Q5T8Y7_HUMAN)	7.62	16.89	5	1	2	5	8.360E7	148	16.6	7.88
H7C2W9	60S ribosomal protein L31 (Fragment) OS=Homo sapiens GN=RPL31 PE=4 SV=1 - (H7C2W9_HUMAN)	7.61	59.26	10	3	8	14	2.856E7	108	12.8	11.00
Q8WXC6-1	Isoform 2 of Myeloma-overexpressed gene 2 protein OS=Homo sapiens GN=MYEOV2 - (MYOV2_HUMAN)	7.60	18.25	1	1	5	10	4.874E7	252	27.6	6.33
F8W1S7	Heterogeneous nuclear ribonucleoprotein A1 OS=Homo sapiens GN=HNRNPA1 PE=4 SV=1 - (F8W1S7_HUMAN)	7.56	28.18	19	2	9	11	2.064E7	330	35.5	9.11
B4E2V4	Proteasome (Prosome, macropain) subunit, alpha type, 5, isoform CRA_c OS=Homo sapiens GN=PSMA5 PE=2 SV=1 - (B4E2V4_HUMAN)	7.53	14.21	3	2	2	3	8.505E6	183	20.0	4.73
F8W651	Coatomer subunit zeta-1 OS=Homo sapiens GN=COPZ1 PE=4 SV=1 - (F8W651_HUMAN)	7.52	32.77	9	1	3	3	5.464E6	119	13.4	4.82
Q6GX22	Midline 2 OS=Homo sapiens GN=MID2 PE=2 SV=1 - (Q6GX22_HUMAN)	7.52	7.30	3	1	6	8	5.280E7	685	77.9	7.05
B3KUI2	cDNA FLJ39965 fis, clone SPLEN2027157, highly similar to Homo sapiens tumor necrosis factor, alpha-induced protein 8 (TNFAIP8), mRNA OS=Homo sapiens PE=2 SV=1 - (B3KUI2_HUMAN)	7.50	27.78	7	1	3	6	2.327E7	198	23.0	7.91
O43594	Ku86 autoantigen related protein 1 (Fragment) OS=Homo sapiens GN=KARP-1 PE=1 SV=1 - (O43594_HUMAN)	7.45	33.97	1	1	7	18	1.263E8	156	17.9	12.37
A8K4W8	cDNA FLJ77917, highly similar to Homo sapiens ubiquitin-conjugating enzyme E2L 3 (UBE2L3), transcript variant 1, mRNA OS=Homo sapiens PE=2 SV=1 - (A8K4W8_HUMAN)	7.44	22.08	6	1	3	4	4.980E7	154	17.8	8.75
P18510-2	Isoform 2 of Interleukin-1 receptor antagonist protein OS=Homo sapiens GN=IL1RN - (IL1RA_HUMAN)	7.38	24.53	4	1	3	4	4.483E7	159	17.9	5.35
B4DKX4	cDNA FLJ58014, highly similar to Homo sapiens programmed cell death 4, transcript variant 1, mRNA OS=Homo sapiens PE=2 SV=1 - (B4DKX4_HUMAN)	7.37	7.69	5	1	3	6	2.358E7	455	50.2	5.47
Q5T7W0-2	Isoform 2 of Zinc finger protein 618 OS=Homo sapiens GN=ZNF618 - (ZN618_HUMAN)	7.35	7.90	3	1	7	16	1.078E7	861	95.6	7.12
Q9BQQ5	Ribosomal protein L27a OS=Homo sapiens GN=L27a PE=2 SV=1 - (Q9BQQ5_HUMAN)	7.34	24.53	6	2	3	4	3.398E7	106	12.0	11.46
A8K1B1	cDNA FLJ75211, highly similar to Homo sapiens ubiquitin specific peptidase like 1, mRNA OS=Homo sapiens PE=2 SV=1 - (A8K1B1_HUMAN)	7.33	4.58	4	1	4	8	1.535E8	1092	120.3	6.19
H0YB22	40S ribosomal protein S14 (Fragment) OS=Homo sapiens GN=RPS14 PE=4 SV=1 - (H0YB22_HUMAN)	7.30	25.83	3	1	2	3	1.632E7	120	12.9	9.85
P43686-2	Isoform 2 of 26S protease regulatory subunit 6B OS=Homo sapiens GN=PSMC4 - (PRS6B_HUMAN)	7.29	11.37	2	1	2	2	1.933E6	387	43.5	5.26
P09467	Fructose-1,6-bisphosphatase 1 OS=Homo sapiens GN=FBP1 PE=1 SV=5 - (F16P1_HUMAN)	7.29	12.72	2	1	4	7	1.566E8	338	36.8	6.99
B2RDB6	cDNA, FLJ96540, highly similar to Homo sapiens zinc finger protein 3 (A8-51) (ZNF3), transcript variant 2, mRNA OS=Homo sapiens PE=2 SV=1 - (B2RDB6_HUMAN)	7.28	11.46	9	1	4	10	5.608E7	410	47.1	8.32
P0C7V6	Putative protein FAM48B2 OS=Homo sapiens GN=FAM48B2 PE=5 SV=1 - (F48B2_HUMAN)	7.23	9.06	1	1	6	19	4.129E7	817	87.5	8.79
Q56VL3	OCIA domain-containing protein 2 OS=Homo sapiens GN=OCIAD2 PE=1 SV=1 - (OCAD2_HUMAN)	7.23	22.73	1	1	2	3	2.391E6	154	16.9	9.03
Q9NQ38-3	Isoform long of Serine protease inhibitor Kazal-type 5 OS=Homo sapiens GN=SPINK5 - (ISK5_HUMAN)	7.20	10.33	2	1	14	20	1.447E8	1094	124.0	8.09
Q5JR95	40S ribosomal protein S8 OS=Homo sapiens GN=RPS8 PE=4 SV=1 - (Q5JR95_HUMAN)	7.16	29.26	3	2	5	7	3.158E7	188	21.9	10.36
Q96RP6	Ubiquitin-conjugating enzyme OS=Homo sapiens GN=PUBC1 PE=2 SV=1 - (Q96RP6_HUMAN)	7.15	36.44	9	2	3	3	3.982E6	118	13.6	8.29
E9PN17	ATP synthase subunit g, mitochondrial OS=Homo sapiens GN=ATP5L PE=4 SV=1 - (E9PN17_HUMAN)	7.14	36.84	2	2	2	3	7.104E6	76	8.4	10.29
Q96PI1	Small proline-rich protein 4 OS=Homo sapiens GN=SPRR4 PE=2 SV=1 - (SPRR4_HUMAN)	7.09	11.39	1	1	1	3	4.923E5	79	8.8	9.66
Q6NWZ1	CKAP4 protein (Fragment) OS=Homo sapiens GN=CKAP4 PE=2 SV=1 - (Q6NWZ1_HUMAN)	7.08	17.83	4	2	8	11	3.696E7	600	67.8	9.31
B4DN59	cDNA FLJ52702, highly similar to Homo sapiens CD44 antigen (homing function and Indian blood group system) (CD44), transcript variant 4, mRNA OS=Homo sapiens PE=2 SV=1 - (B4DN59_HUMAN)	7.07	12.77	32	1	4	5	5.301E6	329	36.0	5.90
P49411	Elongation factor Tu, mitochondrial OS=Homo sapiens GN=TUFM PE=1 SV=2 - (EFTU_HUMAN)	6.96	15.49	1	1	5	5	7.810E6	452	49.5	7.61
E9PDE0	Uncharacterized protein KIAA1683 OS=Homo sapiens GN=KIAA1683 PE=4 SV=1 - (E9PDE0_HUMAN)	6.86	8.12	8	1	9	22	1.073E8	1367	147.2	10.17
O75368	SH3 domain-binding glutamic acid-rich-like protein OS=Homo sapiens GN=SH3BGRL PE=1 SV=1 - (SH3L1_HUMAN)	6.86	31.58	1	1	3	4	3.696E6	114	12.8	5.25
Q8WX93-2	Isoform 2 of Palladin OS=Homo sapiens GN=PALLD - (PALLD_HUMAN)	6.81	9.49	9	2	8	12	2.926E7	1106	122.0	6.92
Q5T123	SH3 domain binding glutamic acid-rich protein like 3 OS=Homo sapiens GN=SH3BGRL3 PE=2 SV=1 - (Q5T123_HUMAN)	6.78	31.82	4	2	3	3	1.922E6	88	9.4	9.36
Q13515	Phakinin OS=Homo sapiens GN=BFSP2 PE=1 SV=1 - (BFSP2_HUMAN)	6.77	13.98	1	1	3	4	1.451E7	415	45.9	5.55
E9PE88	cGMP-dependent 3',5'-cyclic phosphodiesterase OS=Homo sapiens GN=PDE2A PE=4 SV=2 - (E9PE88_HUMAN)	6.75	6.74	9	1	6	12	3.810E8	950	106.1	5.64
Q5H9J9	Novel protein similar to t-complex 11 homolog (Mouse) TCP11 OS=Homo sapiens GN=LL0XNC01-19D8.1 PE=4 SV=1 - (Q5H9J9_HUMAN)	6.74	8.35	2	1	3	4	4.804E6	407	46.3	5.19
D3DV26	S100 calcium binding protein A10 (Annexin II ligand, calpactin I, light polypeptide (P11)), isoform CRA_b (Fragment) OS=Homo sapiens GN=S100A10 PE=4 SV=1 - (D3DV26_HUMAN)	6.71	17.07	3	2	3	6	1.882E7	205	22.3	10.33
E9PI98	Layilin (Fragment) OS=Homo sapiens GN=LAYN PE=4 SV=1 - (E9PI98_HUMAN)	6.65	14.06	1	1	1	3	2.733E7	64	7.4	8.00
H7BZ60	Protein kinase C gamma type (Fragment) OS=Homo sapiens GN=PRKCG PE=4 SV=1 - (H7BZ60_HUMAN)	6.64	12.90	1	1	3	4	6.721E6	186	21.0	9.57
H0Y3H1	Toll-interacting protein OS=Homo sapiens GN=TOLLIP PE=4 SV=1 - (H0Y3H1_HUMAN)	6.62	24.72	5	1	9	13	6.586E7	352	38.9	8.46
Q9NT22	EMILIN-3 OS=Homo sapiens GN=EMILIN3 PE=2 SV=2 - (EMIL3_HUMAN)	6.61	6.79	1	1	5	10	4.971E8	766	82.6	7.72
B2R761	cDNA, FLJ93299, highly similar to Homo sapiens sterol carrier protein 2 (SCP2), mRNA OS=Homo sapiens PE=2 SV=1 - (B2R761_HUMAN)	6.59	8.04	10	1	4	6	6.111E6	547	59.0	7.05
P27824	Calnexin OS=Homo sapiens GN=CANX PE=1 SV=2 - (CALX_HUMAN)	6.54	8.61	12	2	4	4	6.080E6	592	67.5	4.60
Q12906	Interleukin enhancer-binding factor 3 OS=Homo sapiens GN=ILF3 PE=1 SV=3 - (ILF3_HUMAN)	6.52	7.05	14	1	5	6	4.709E7	894	95.3	8.76
B4E0B1	cDNA FLJ52100 OS=Homo sapiens PE=2 SV=1 - (B4E0B1_HUMAN)	6.51	7.52	4	1	8	14	1.366E8	891	100.9	8.27
P10909-4	Isoform 4 of Clusterin OS=Homo sapiens GN=CLU - (CLUS_HUMAN)	6.51	11.06	8	1	4	4	1.838E7	416	48.8	6.71
Q3LI83	Keratin-associated protein 24-1 OS=Homo sapiens GN=KRTAP24-1 PE=1 SV=1 - (KR241_HUMAN)	6.50	7.87	1	1	2	3	7.119E6	254	27.7	8.29
Q9BPX5	Actin-related protein 2/3 complex subunit 5-like protein OS=Homo sapiens GN=ARPC5L PE=1 SV=1 - (ARP5L_HUMAN)	6.48	29.41	2	1	5	8	4.685E7	153	16.9	6.60
P16401	Histone H1.5 OS=Homo sapiens GN=HIST1H1B PE=1 SV=3 - (H15_HUMAN)	6.41	14.16	1	1	4	8	3.117E7	226	22.6	10.92
Q9UJ55	MAGE-like protein 2 OS=Homo sapiens GN=MAGEL2 PE=1 SV=1 - (MAGL2_HUMAN)	6.40	3.21	5	1	1	5	1.509E7	529	58.6	7.99
Q5HYC5	Putative uncharacterized protein DKFZp686L08115 (Fragment) OS=Homo sapiens GN=DKFZp686L08115 PE=2 SV=1 - (Q5HYC5_HUMAN)	6.40	20.97	1	1	4	5	1.778E7	248	27.6	9.54
B4DYI3	cDNA FLJ57061, highly similar to NADH-ubiquinone oxidoreductase 23 kDa subunit, mitochondrial (EC 1.6.5.3) OS=Homo sapiens PE=2 SV=1 - (B4DYI3_HUMAN)	6.38	29.82	7	1	4	5	6.558E6	171	18.6	9.01
B4E1M5	60S ribosomal protein L9 OS=Homo sapiens GN=RPL9 PE=2 SV=1 - (B4E1M5_HUMAN)	6.37	21.09	8	1	5	5	8.250E7	147	16.9	10.70
G5E9Y3	Arf-GAP with Rho-GAP domain, ANK repeat and PH domain-containing protein 3 OS=Homo sapiens GN=ARAP3 PE=4 SV=1 - (G5E9Y3_HUMAN)	6.36	9.02	1	1	7	16	6.239E7	1375	151.0	6.86
B2R862	cDNA, FLJ93754, highly similar to Homo sapiens tripartite motif-containing 38 (TRIM38), mRNA OS=Homo sapiens PE=2 SV=1 - (B2R862_HUMAN)	6.36	11.83	4	1	6	7	1.025E8	465	53.4	7.02
F8WCF6	Actin-related protein 2/3 complex subunit 4 OS=Homo sapiens GN=ARPC4 PE=4 SV=1 - (F8WCF6_HUMAN)	6.36	33.70	6	1	5	6	7.554E7	181	21.0	8.76
H0YJA0	Putative E3 ubiquitin-protein ligase UBR7 (Fragment) OS=Homo sapiens GN=UBR7 PE=4 SV=1 - (H0YJA0_HUMAN)	6.31	11.59	1	1	2	4	4.599E6	164	18.0	4.64
Q8NHY3	GAS2-like protein 2 OS=Homo sapiens GN=GAS2L2 PE=2 SV=1 - (GA2L2_HUMAN)	6.29	9.32	3	1	7	8	4.723E7	880	96.5	9.13
A6NCD2	T-complex protein 1 subunit zeta OS=Homo sapiens GN=CCT6A PE=3 SV=1 - (A6NCD2_HUMAN)	6.29	8.85	7	1	4	4	2.017E7	486	53.3	7.27
B3KPG6	cDNA FLJ31769 fis, clone NT2RI2007956, highly similar to Chondroitin sulfate glucuronyltransferase (EC 2.4.1.226) OS=Homo sapiens PE=2 SV=1 - (B3KPG6_HUMAN)	6.26	5.76	5	1	2	5	3.546E7	764	85.1	7.85
Q9Y3R5-2	Isoform 2 of Protein dopey-2 OS=Homo sapiens GN=DOPEY2 - (DOP2_HUMAN)	6.25	6.11	2	1	12	49	5.616E7	2291	257.2	6.40
Q9Y4F4	Protein FAM179B OS=Homo sapiens GN=FAM179B PE=1 SV=4 - (F179B_HUMAN)	6.24	8.55	1	1	13	17	1.467E8	1720	189.2	8.50
B4E190	cDNA FLJ57770, moderately similar to ADP-ribosylation factor 3 OS=Homo sapiens PE=2 SV=1 - (B4E190_HUMAN)	6.22	30.56	16	1	3	3	7.542E6	144	16.1	7.34
H0YCD4	T-lymphoma invasion and metastasis-inducing protein 2 OS=Homo sapiens GN=TIAM2 PE=4 SV=1 - (H0YCD4_HUMAN)	6.21	6.99	8	1	15	18	1.785E8	1947	218.5	7.09
E9PF10	Nuclear pore complex protein Nup155 OS=Homo sapiens GN=NUP155 PE=4 SV=1 - (E9PF10_HUMAN)	6.20	5.28	5	1	6	11	1.022E8	1327	148.0	6.30
Q53TD0	Putative uncharacterized protein SP100 (Fragment) OS=Homo sapiens GN=SP100 PE=2 SV=1 - (Q53TD0_HUMAN)	6.15	7.35	11	1	7	11	2.534E7	843	96.5	8.31
Q53GD1	Guanine nucleotide-binding protein subunit gamma (Fragment) OS=Homo sapiens PE=2 SV=1 - (Q53GD1_HUMAN)	6.15	31.94	2	1	2	2	1.012E6	72	8.0	8.97
E9PI54	DNA repair protein RAD51 homolog 1 OS=Homo sapiens GN=RAD51 PE=4 SV=1 - (E9PI54_HUMAN)	6.07	29.00	1	1	3	6	1.105E7	100	11.1	5.07
O60658-2	Isoform 2 of High affinity cAMP-specific and IBMX-insensitive 3',5'-cyclic phosphodiesterase 8A OS=Homo sapiens GN=PDE8A - (PDE8A_HUMAN)	6.07	5.75	3	1	3	5	1.400E7	783	88.2	6.23
P62273	40S ribosomal protein S29 OS=Homo sapiens GN=RPS29 PE=1 SV=2 - (RS29_HUMAN)	6.06	26.79	2	2	3	4	2.029E7	56	6.7	10.13
O95601	Mih1/Tx protein OS=Homo sapiens GN=mih1/Tx PE=2 SV=1 - (O95601_HUMAN)	6.03	26.72	7	1	3	11	3.488E7	116	13.3	8.75
F4NBY2	MHC class I antigen (Fragment) OS=Homo sapiens GN=HLA-B PE=3 SV=1 - (F4NBY2_HUMAN)	6.02	7.12	1	1	3	5	3.535E7	337	38.2	6.80
P62304	Small nuclear ribonucleoprotein E OS=Homo sapiens GN=SNRPE PE=1 SV=1 - (RUXE_HUMAN)	6.02	11.96	1	1	1	3	3.991E6	92	10.8	9.44
Q96C19	EF-hand domain-containing protein D2 OS=Homo sapiens GN=EFHD2 PE=1 SV=1 - (EFHD2_HUMAN)	5.99	14.58	10	1	3	7	2.331E7	240	26.7	5.20
Q14126	Desmoglein-2 OS=Homo sapiens GN=DSG2 PE=1 SV=2 - (DSG2_HUMAN)	5.94	5.37	1	1	3	3	1.451E7	1118	122.2	5.24
P61081	NEDD8-conjugating enzyme Ubc12 OS=Homo sapiens GN=UBE2M PE=1 SV=1 - (UBC12_HUMAN)	5.93	14.75	1	1	2	2	2.614E6	183	20.9	7.69
Q6UXL0	Interleukin-20 receptor subunit beta OS=Homo sapiens GN=IL20RB PE=1 SV=1 - (I20RB_HUMAN)	5.90	6.75	1	1	2	4	3.425E7	311	35.1	5.03
B7Z3I7	Malate dehydrogenase OS=Homo sapiens PE=2 SV=1 - (B7Z3I7_HUMAN)	5.89	16.48	8	2	5	5	2.915E6	352	38.6	8.00
B7Z1I2	Aspartate aminotransferase OS=Homo sapiens GN=GOT1 PE=2 SV=1 - (B7Z1I2_HUMAN)	5.88	7.38	4	1	3	4	1.106E8	366	41.0	7.18
Q08554-2	Isoform 1B of Desmocollin-1 OS=Homo sapiens GN=DSC1 - (DSC1_HUMAN)	5.86	10.24	2	2	7	10	1.065E7	840	93.8	5.53
B4DKN9	cDNA FLJ57740, highly similar to Transforming protein RhoA OS=Homo sapiens PE=2 SV=1 - (B4DKN9_HUMAN)	5.85	20.23	15	2	3	5	6.445E6	173	19.6	8.22
Q8WXF0	Serine/arginine-rich splicing factor 12 OS=Homo sapiens GN=SRSF12 PE=1 SV=1 - (SRS12_HUMAN)	5.84	14.56	2	1	4	6	6.001E7	261	30.5	11.69
G3V193	Activating molecule in BECN1-regulated autophagy protein 1 OS=Homo sapiens GN=AMBRA1 PE=4 SV=1 - (G3V193_HUMAN)	5.84	3.31	8	1	4	17	1.601E8	1179	129.5	6.98
Q02156	Protein kinase C epsilon type OS=Homo sapiens GN=PRKCE PE=1 SV=1 - (KPCE_HUMAN)	5.83	9.63	8	1	7	10	2.784E8	737	83.6	7.12
C9J1E7	AP-1 complex subunit beta-1 (Fragment) OS=Homo sapiens GN=AP1B1 PE=4 SV=1 - (C9J1E7_HUMAN)	5.83	6.40	6	1	3	3	2.408E6	578	65.3	5.19
E7EPR8	Breast carcinoma-amplified sequence 1 OS=Homo sapiens GN=BCAS1 PE=4 SV=1 - (E7EPR8_HUMAN)	5.79	10.68	2	1	5	9	6.048E7	384	41.5	8.70
Q9UNN8	Endothelial protein C receptor OS=Homo sapiens GN=PROCR PE=1 SV=1 - (EPCR_HUMAN)	5.78	3.78	1	1	1	3	4.683E6	238	26.7	7.18
Q9NQ94-2	Isoform 2 of APOBEC1 complementation factor OS=Homo sapiens GN=A1CF - (A1CF_HUMAN)	5.77	13.14	8	1	6	13	6.085E7	586	64.2	8.59
O14497-2	Isoform 2 of AT-rich interactive domain-containing protein 1A OS=Homo sapiens GN=ARID1A - (ARI1A_HUMAN)	5.76	4.84	2	1	8	10	1.057E8	2068	218.2	6.52
Q05CV8	PRPF39 protein (Fragment) OS=Homo sapiens GN=PRPF39 PE=2 SV=1 - (Q05CV8_HUMAN)	5.76	4.59	2	1	1	4	1.847E7	218	25.5	9.39
Q5T8U3	60S ribosomal protein L7a (Fragment) OS=Homo sapiens GN=RPL7A PE=4 SV=1 - (Q5T8U3_HUMAN)	5.74	11.52	2	1	2	3	5.751E6	191	21.5	11.02
Q5UBV6	NIN/PDGFRB fusion protein (Fragment) OS=Homo sapiens GN=NIN/PDGFRB fusion PE=2 SV=1 - (Q5UBV6_HUMAN)	5.66	8.08	2	1	3	5	6.059E7	334	38.5	7.37
Q15102	Platelet-activating factor acetylhydrolase IB subunit gamma OS=Homo sapiens GN=PAFAH1B3 PE=1 SV=1 - (PA1B3_HUMAN)	5.62	3.90	1	1	1	2	4.186E6	231	25.7	6.84
Q5MCW4	Zinc finger protein 569 OS=Homo sapiens GN=ZNF569 PE=2 SV=1 - (ZN569_HUMAN)	5.55	8.60	1	1	6	9	2.039E7	686	79.5	8.66
B7Z993	Zinc finger protein 418 OS=Homo sapiens GN=ZNF418 PE=2 SV=1 - (B7Z993_HUMAN)	5.50	9.19	3	1	5	7	1.583E8	642	74.2	8.79
Q53FL7	Vascular cell adhesion molecule 1 isoform a variant (Fragment) OS=Homo sapiens PE=2 SV=1 - (Q53FL7_HUMAN)	5.48	15.70	7	1	6	13	1.907E8	739	81.2	5.22
B4DJ44	cDNA FLJ54716, highly similar to Target of Myb protein 1 OS=Homo sapiens PE=2 SV=1 - (B4DJ44_HUMAN)	5.46	9.03	12	1	4	4	2.814E6	454	49.5	5.08
Q9P1U1-3	Isoform 3 of Actin-related protein 3B OS=Homo sapiens GN=ACTR3B - (ARP3B_HUMAN)	5.46	18.68	13	1	6	6	4.364E6	348	39.7	5.11
C9J1Z8	ADP-ribosylation factor 5 (Fragment) OS=Homo sapiens GN=ARF5 PE=3 SV=1 - (C9J1Z8_HUMAN)	5.45	14.00	14	1	2	2	9.902E6	150	17.1	7.34
Q9P1W3	Transmembrane protein 63C OS=Homo sapiens GN=TMEM63C PE=2 SV=1 - (TM63C_HUMAN)	5.43	3.60	1	1	3	4	6.016E6	806	93.3	7.03
E7EQY4	Metastasis-associated protein MTA3 OS=Homo sapiens GN=MTA3 PE=4 SV=1 - (E7EQY4_HUMAN)	5.40	13.42	8	2	7	7	6.773E7	514	58.7	8.21
Q5JW98	Protein FAM26D OS=Homo sapiens GN=FAM26D PE=1 SV=1 - (FA26D_HUMAN)	5.40	9.87	5	1	2	4	1.034E7	314	35.0	6.87
E5RGF0	Gamma-glutamyl hydrolase (Fragment) OS=Homo sapiens GN=GGH PE=4 SV=1 - (E5RGF0_HUMAN)	5.35	14.11	4	2	2	2	2.590E6	163	18.4	7.24
F5GYN4	Ubiquitin thioesterase OTUB1 OS=Homo sapiens GN=OTUB1 PE=4 SV=1 - (F5GYN4_HUMAN)	5.35	10.79	5	1	2	3	6.297E6	241	28.0	5.29
A1L3A9	TBC1 domain family, member 9B (With GRAM domain) OS=Homo sapiens GN=TBC1D9B PE=2 SV=1 - (A1L3A9_HUMAN)	5.32	2.84	4	1	4	8	9.796E6	1232	138.5	5.33
Q8TDY8	Immunoglobulin superfamily DCC subclass member 4 OS=Homo sapiens GN=IGDCC4 PE=2 SV=1 - (IGDC4_HUMAN)	5.29	1.76	1	1	3	7	1.457E8	1250	134.1	6.20
O15054	Lysine-specific demethylase 6B OS=Homo sapiens GN=KDM6B PE=1 SV=4 - (KDM6B_HUMAN)	5.28	1.58	3	1	3	9	2.089E7	1643	176.5	8.54
Q8IU60	m7GpppN-mRNA hydrolase OS=Homo sapiens GN=DCP2 PE=1 SV=2 - (DCP2_HUMAN)	5.28	15.71	2	1	8	16	4.435E7	420	48.4	8.00
Q2NLA0	Coatomer subunit gamma OS=Homo sapiens GN=COPG2 PE=2 SV=1 - (Q2NLA0_HUMAN)	5.22	12.53	8	1	6	93	2.009E7	726	81.4	6.58
F5GY37	Prohibitin-2 OS=Homo sapiens GN=PHB2 PE=4 SV=1 - (F5GY37_HUMAN)	5.15	11.61	2	1	3	3	6.315E6	267	29.7	9.88
P42766	60S ribosomal protein L35 OS=Homo sapiens GN=RPL35 PE=1 SV=2 - (RL35_HUMAN)	5.13	45.53	4	4	7	9	1.926E7	123	14.5	11.05
Q5JXB2	Putative ubiquitin-conjugating enzyme E2 N-like OS=Homo sapiens GN=UBE2NL PE=5 SV=1 - (UE2NL_HUMAN)	5.10	20.92	3	1	3	9	6.811E7	153	17.4	5.92
O75154	Rab11 family-interacting protein 3 OS=Homo sapiens GN=RAB11FIP3 PE=1 SV=1 - (RFIP3_HUMAN)	5.09	6.08	4	1	4	7	4.114E7	756	82.4	4.51
Q8N7Y3	cDNA FLJ40219 fis, clone TESTI2021637, moderately similar to LIVER CARBOXYLESTERASE (EC 3.1.1.1) OS=Homo sapiens PE=2 SV=1 - (Q8N7Y3_HUMAN)	5.07	5.31	1	1	1	4	8.129E6	207	21.8	6.90
C9IZG4	Protein CutA OS=Homo sapiens GN=CUTA PE=4 SV=1 - (C9IZG4_HUMAN)	5.07	14.81	4	1	2	3	4.470E6	135	14.4	5.45
A8K5A5	cDNA FLJ75393, highly similar to Homo sapiens PHD finger protein 16 (PHF16), mRNA OS=Homo sapiens PE=2 SV=1 - (A8K5A5_HUMAN)	5.06	10.09	2	1	9	14	1.866E8	823	93.8	7.05
Q8IXI1	Mitochondrial Rho GTPase 2 OS=Homo sapiens GN=RHOT2 PE=1 SV=2 - (MIRO2_HUMAN)	5.01	2.75	1	1	2	9	1.984E8	618	68.1	5.86
D9MWM3	Soluble Fc mu receptor OS=Homo sapiens GN=FCMR PE=2 SV=1 - (D9MWM3_HUMAN)	5.01	15.03	4	1	3	4	9.457E6	306	33.8	10.07
Q96PT7	Integrin beta 2 (Fragment) OS=Homo sapiens GN=ITGB2 PE=2 SV=1 - (Q96PT7_HUMAN)	4.99	37.50	6	1	2	6	1.237E8	56	6.4	8.53
Q8NFQ5	BPI fold-containing family B member 6 OS=Homo sapiens GN=BPIFB6 PE=2 SV=1 - (BPIB6_HUMAN)	4.97	5.08	1	1	1	5	7.437E6	453	49.7	8.84
B7Z5E7	cDNA FLJ51046, highly similar to 60 kDa heat shock protein, mitochondrial OS=Homo sapiens PE=2 SV=1 - (B7Z5E7_HUMAN)	4.95	5.80	12	1	2	2	6.676E6	517	55.0	5.60
C9JFR7	Cytochrome c (Fragment) OS=Homo sapiens GN=CYCS PE=3 SV=1 - (C9JFR7_HUMAN)	4.94	18.81	3	1	2	2		101	11.3	9.66
Q8NFU7	Methylcytosine dioxygenase TET1 OS=Homo sapiens GN=TET1 PE=1 SV=2 - (TET1_HUMAN)	4.92	5.06	1	1	11	18	1.317E8	2136	235.2	8.24
Q5JPJ9	Putative uncharacterized protein DKFZp686D0114 OS=Homo sapiens GN=DKFZp686D0114 PE=2 SV=1 - (Q5JPJ9_HUMAN)	4.91	16.67	4	1	5	8	1.372E7	294	30.5	8.27
Q9UJZ1	Stomatin-like protein 2 OS=Homo sapiens GN=STOML2 PE=1 SV=1 - (STML2_HUMAN)	4.90	9.83	2	1	3	4	9.911E7	356	38.5	7.39
Q9NTK5	Obg-like ATPase 1 OS=Homo sapiens GN=OLA1 PE=1 SV=2 - (OLA1_HUMAN)	4.90	14.65	5	1	5	5	1.619E7	396	44.7	7.81
Q5HYE3	Putative uncharacterized protein DKFZp686H1812 OS=Homo sapiens GN=DKFZp686H1812 PE=2 SV=1 - (Q5HYE3_HUMAN)	4.90	14.93	3	1	4	6	3.823E7	402	45.9	7.91
P30046	D-dopachrome decarboxylase OS=Homo sapiens GN=DDT PE=1 SV=3 - (DOPD_HUMAN)	4.80	19.49	2	1	2	4	1.556E8	118	12.7	7.30
B2R679	cDNA, FLJ92825, highly similar to Homo sapiens SAR1a gene homolog 1 (S. cerevisiae) (SARA1), mRNA OS=Homo sapiens PE=2 SV=1 - (B2R679_HUMAN)	4.75	9.60	10	1	2	4	1.714E7	198	22.3	7.03
E9PCW0	Prohibitin OS=Homo sapiens GN=PHB PE=4 SV=1 - (E9PCW0_HUMAN)	4.71	13.55	10	1	3	4	2.033E7	214	23.6	6.81
A8K865	cDNA FLJ75789, highly similar to Homo sapiens synaptophysin-like 1 (SYPL1), transcript variant 1, mRNA OS=Homo sapiens PE=2 SV=1 - (A8K865_HUMAN)	4.70	8.49	4	1	2	2	4.606E7	259	28.5	7.99
D6RA82	Annexin OS=Homo sapiens GN=ANXA3 PE=3 SV=1 - (D6RA82_HUMAN)	4.68	10.56	2	2	3	4	4.907E7	284	32.1	5.94
Q99497	Protein DJ-1 OS=Homo sapiens GN=PARK7 PE=1 SV=2 - (PARK7_HUMAN)	4.67	20.63	1	2	3	4	6.160E6	189	19.9	6.79
B3KTA3	cDNA FLJ37935 fis, clone CTONG2005290, highly similar to FASCIN OS=Homo sapiens PE=2 SV=1 - (B3KTA3_HUMAN)	4.66	14.41	7	2	4	7	1.335E7	472	52.2	7.65
E1V2K1	MHC class II antigen (Fragment) OS=Homo sapiens GN=HLA-DRB1 PE=4 SV=1 - (E1V2K1_HUMAN)	4.63	32.58	34	1	2	8	9.254E6	89	10.6	5.85
Q70V32	ABO glycosyltransferase (Fragment) OS=Homo sapiens GN=ABO PE=4 SV=1 - (Q70V32_HUMAN)	4.62	11.01	5	1	3	5	1.695E7	345	38.8	8.94
B4DJV2	Citrate synthase OS=Homo sapiens GN=CS PE=2 SV=1 - (B4DJV2_HUMAN)	4.62	10.38	5	2	3	5	1.903E7	453	50.4	7.90
Q9UKB3	DnaJ homolog subfamily C member 12 OS=Homo sapiens GN=DNAJC12 PE=1 SV=1 - (DJC12_HUMAN)	4.61	13.64	1	1	3	3	1.089E8	198	23.4	5.71
P00491	Purine nucleoside phosphorylase OS=Homo sapiens GN=PNP PE=1 SV=2 - (PNPH_HUMAN)	4.60	13.84	5	2	4	6	5.719E6	289	32.1	6.95
P37837	Transaldolase OS=Homo sapiens GN=TALDO1 PE=1 SV=2 - (TALDO_HUMAN)	4.59	22.85	2	2	8	8	7.932E7	337	37.5	6.81
Q86XR8-4	Isoform 4 of Centrosomal protein of 57 kDa OS=Homo sapiens GN=CEP57 - (CEP57_HUMAN)	4.54	5.74	8	1	3	7	3.024E7	488	56.0	9.35
E9PRA1	Putative deoxyribonuclease TATDN3 (Fragment) OS=Homo sapiens GN=TATDN3 PE=4 SV=1 - (E9PRA1_HUMAN)	4.49	27.16	11	1	4	11	9.767E7	162	18.3	6.80
Q58FF6	Putative heat shock protein HSP 90-beta 4 OS=Homo sapiens GN=HSP90AB4P PE=5 SV=1 - (H90B4_HUMAN)	4.48	5.15	1	1	2	3	5.936E6	505	58.2	4.73
P02792	Ferritin light chain OS=Homo sapiens GN=FTL PE=1 SV=2 - (FRIL_HUMAN)	4.47	24.57	2	1	4	4	9.221E8	175	20.0	5.78
B8ZZL8	10 kDa heat shock protein, mitochondrial OS=Homo sapiens GN=HSPE1 PE=3 SV=1 - (B8ZZL8_HUMAN)	4.46	14.85	3	1	2	3	4.659E6	101	10.7	9.58
Q9Y5Z4-2	Isoform 2 of Heme-binding protein 2 OS=Homo sapiens GN=HEBP2 - (HEBP2_HUMAN)	4.46	7.07	3	1	1	1	2.257E6	184	20.8	4.78
Q3HKR1	BTB/POZ domain-containing protein KCTD7 OS=Homo sapiens GN=KCTD7 PE=2 SV=1 - (Q3HKR1_HUMAN)	4.46	20.64	10	1	7	7	9.404E7	407	47.0	7.14
Q8TEU7	Rap guanine nucleotide exchange factor 6 OS=Homo sapiens GN=RAPGEF6 PE=1 SV=2 - (RPGF6_HUMAN)	4.46	6.56	15	1	9	12	3.348E7	1601	179.3	6.40
P41252	Isoleucine--tRNA ligase, cytoplasmic OS=Homo sapiens GN=IARS PE=1 SV=2 - (SYIC_HUMAN)	4.44	8.16	6	1	7	10	7.156E7	1262	144.4	6.15
P04632	Calpain small subunit 1 OS=Homo sapiens GN=CAPNS1 PE=1 SV=1 - (CPNS1_HUMAN)	4.43	3.36	1	1	1	2	4.672E5	268	28.3	5.20
P63167	Dynein light chain 1, cytoplasmic OS=Homo sapiens GN=DYNLL1 PE=1 SV=1 - (DYL1_HUMAN)	4.42	20.22	4	2	2	3	2.124E7	89	10.4	7.40
Q8N8D1	Programmed cell death protein 7 OS=Homo sapiens GN=PDCD7 PE=1 SV=1 - (PDCD7_HUMAN)	4.40	9.28	1	1	5	7	3.125E7	485	54.7	9.95
B4DPT5	cDNA FLJ50023, highly similar to Alanine aminotransferase 1 (EC 2.6.1.2) OS=Homo sapiens PE=2 SV=1 - (B4DPT5_HUMAN)	4.39	20.52	1	1	4	5	3.229E8	385	41.4	8.65
B0YJ28	Interleukin-18 OS=Homo sapiens GN=IL18 PE=3 SV=1 - (B0YJ28_HUMAN)	4.38	13.47	4	1	3	3	3.116E7	193	22.3	4.84
B1AJY7	26S proteasome non-ATPase regulatory subunit 10 OS=Homo sapiens GN=PSMD10 PE=4 SV=1 - (B1AJY7_HUMAN)	4.38	16.06	4	1	3	9	6.142E7	193	20.8	6.64
Q14CB2	DOK1 protein OS=Homo sapiens GN=DOK1 PE=2 SV=1 - (Q14CB2_HUMAN)	4.33	6.37	7	1	1	3	1.023E7	267	28.7	5.08
Q86TR6	Full-length cDNA 5-PRIME end of clone CS0DF014YL24 of Fetal brain of Homo sapiens (human) (Fragment) OS=Homo sapiens PE=2 SV=1 - (Q86TR6_HUMAN)	4.33	15.25	2	1	2	3	5.103E7	177	20.4	6.00
Q96KP4-2	Isoform 2 of Cytosolic non-specific dipeptidase OS=Homo sapiens GN=CNDP2 - (CNDP2_HUMAN)	4.32	16.37	6	1	4	4	1.733E7	391	43.8	6.48
O96013	Serine/threonine-protein kinase PAK 4 OS=Homo sapiens GN=PAK4 PE=1 SV=1 - (PAK4_HUMAN)	4.32	11.34	9	1	5	8	4.879E7	591	64.0	9.73
E5RGX1	Transcription factor IIIB 50 kDa subunit OS=Homo sapiens GN=BRF2 PE=4 SV=1 - (E5RGX1_HUMAN)	4.31	3.96	4	1	1	11	3.096E7	202	22.3	5.85
P28072	Proteasome subunit beta type-6 OS=Homo sapiens GN=PSMB6 PE=1 SV=4 - (PSB6_HUMAN)	4.31	13.39	2	1	2	4	5.447E7	239	25.3	4.92
F8W543	Nucleosome assembly protein 1-like 1 OS=Homo sapiens GN=NAP1L1 PE=3 SV=1 - (F8W543_HUMAN)	4.30	7.32	17	1	2	2	2.621E8	328	38.2	4.56
E9PF49	NADH dehydrogenase (ubiquinone) 1 beta subcomplex subunit 9 OS=Homo sapiens GN=NDUFB9 PE=4 SV=1 - (E9PF49_HUMAN)	4.30	12.67	1	1	2	3	3.901E7	221	26.6	8.87
F8WF32	Dolichyl-diphosphooligosaccharide--protein glycosyltransferase subunit 1 OS=Homo sapiens GN=RPN1 PE=4 SV=1 - (F8WF32_HUMAN)	4.29	12.40	5	1	1	1	2.427E6	121	12.7	5.21
B2RDY0	cDNA, FLJ96821, highly similar to Homo sapiens solute carrier family 35, member F5 (SLC35F5), mRNA OS=Homo sapiens PE=2 SV=1 - (B2RDY0_HUMAN)	4.29	6.31	5	1	3	5	3.697E7	523	58.8	9.26
B4DVR5	cDNA FLJ56251, highly similar to Cyclin-L2 OS=Homo sapiens PE=2 SV=1 - (B4DVR5_HUMAN)	4.29	11.20	1	1	2	3	4.984E7	241	26.2	6.68
Q92841-1	Isoform 2 of Probable ATP-dependent RNA helicase DDX17 OS=Homo sapiens GN=DDX17 - (DDX17_HUMAN)	4.27	14.92	17	1	8	9	1.757E7	650	72.3	8.59
B3KX75	cDNA FLJ44930 fis, clone BRAMY3015549, highly similar to Neural cell adhesion molecule L1-like protein (Fragment) OS=Homo sapiens PE=2 SV=1 - (B3KX75_HUMAN)	4.25	2.96	3	1	3	5	1.231E7	1113	124.7	6.16
A8K0H3	cDNA FLJ78093, highly similar to Homo sapiens ribosomal protein L29 (RPL29), mRNA OS=Homo sapiens PE=2 SV=1 - (A8K0H3_HUMAN)	4.23	14.47	4	2	2	2	4.689E6	159	17.8	11.74
B4E086	cDNA FLJ51584, highly similar to Homo sapiens spindle pole body component 24 homolog (SPBC24), mRNA OS=Homo sapiens PE=2 SV=1 - (B4E086_HUMAN)	4.23	5.71	1	1	1	2	2.941E7	140	16.0	4.55
Q96QR8	Transcriptional activator protein Pur-beta OS=Homo sapiens GN=PURB PE=1 SV=3 - (PURB_HUMAN)	4.22	9.94	1	1	2	22	2.744E7	312	33.2	5.43
E9PI41	Exosome complex component RRP41 (Fragment) OS=Homo sapiens GN=EXOSC4 PE=4 SV=1 - (E9PI41_HUMAN)	4.21	23.28	1	1	5	11	1.940E7	262	28.4	6.44
E9PJ67	Cathepsin B (Fragment) OS=Homo sapiens GN=CTSB PE=4 SV=1 - (E9PJ67_HUMAN)	4.20	20.79	16	1	2	3		101	11.8	8.50
P49207	60S ribosomal protein L34 OS=Homo sapiens GN=RPL34 PE=1 SV=3 - (RL34_HUMAN)	4.20	33.33	1	1	4	4	5.103E6	117	13.3	11.47
Q2TU64	PIG48 OS=Homo sapiens PE=2 SV=1 - (Q2TU64_HUMAN)	4.17	12.84	7	1	6	14	1.857E7	545	60.5	6.35
D6RA63	Protein GAPT (Fragment) OS=Homo sapiens GN=GAPT PE=4 SV=1 - (D6RA63_HUMAN)	4.10	14.71	3	1	2	4	2.548E7	68	7.6	11.80
B3KX08	cDNA FLJ44420 fis, clone UTERU2002198, highly similar to RNA polymerase II elongation factor ELL3 OS=Homo sapiens PE=2 SV=1 - (B3KX08_HUMAN)	4.10	9.07	2	1	3	4	2.227E7	397	45.3	6.06
B3KMV5	cDNA FLJ12728 fis, clone NT2RP2000040, highly similar to Protein FAM62A OS=Homo sapiens PE=2 SV=1 - (B3KMV5_HUMAN)	4.10	5.98	4	1	6	6	1.187E7	1104	122.8	5.85
Q4J6C6-4	Isoform 4 of Prolyl endopeptidase-like OS=Homo sapiens GN=PREPL - (PPCEL_HUMAN)	4.09	10.19	3	1	4	10	2.731E7	638	73.3	5.44
A8K670	cDNA FLJ75703, highly similar to Homo sapiens nitric oxide synthase interacting protein (NOSIP), mRNA OS=Homo sapiens PE=2 SV=1 - (A8K670_HUMAN)	4.09	8.97	3	1	4	6	1.497E6	301	33.2	8.82
P12532	Creatine kinase U-type, mitochondrial OS=Homo sapiens GN=CKMT1A PE=1 SV=1 - (KCRU_HUMAN)	4.08	11.27	24	3	5	8	5.723E8	417	47.0	8.34
B0I1R4	KIF26A variant protein (Fragment) OS=Homo sapiens PE=2 SV=1 - (B0I1R4_HUMAN)	4.08	7.76	3	1	10	14	1.087E8	1869	193.2	8.79
E4W6B6	RPL27/NME2 fusion protein (Fragment) OS=Homo sapiens GN=RPL27 PE=2 SV=1 - (E4W6B6_HUMAN)	4.07	23.02	3	1	3	4	2.056E8	126	14.2	10.46
Q2TNB3	Cell migration-inducing protein 22 OS=Homo sapiens PE=2 SV=1 - (Q2TNB3_HUMAN)	4.07	11.30	3	1	2	2	1.878E6	239	27.3	5.58
B7Z7I4	cDNA FLJ54568, highly similar to T-complex protein 1 subunit eta OS=Homo sapiens PE=2 SV=1 - (B7Z7I4_HUMAN)	4.04	14.45	10	1	4	7	1.428E8	443	49.0	8.22
E9PCA6	Protocadherin-10 OS=Homo sapiens GN=PCDH10 PE=3 SV=1 - (E9PCA6_HUMAN)	4.04	4.52	4	1	4	6	2.438E7	1040	112.9	4.88
Q59G39	G protein-coupled receptor 22 variant (Fragment) OS=Homo sapiens PE=2 SV=1 - (Q59G39_HUMAN)	4.03	6.01	1	1	2	18	2.924E8	449	51.2	9.45
Q9P241-2	Isoform 2 of Probable phospholipid-transporting ATPase VD OS=Homo sapiens GN=ATP10D - (AT10D_HUMAN)	4.02	3.49	4	1	5	5	1.723E7	1231	138.8	7.15
P35268	60S ribosomal protein L22 OS=Homo sapiens GN=RPL22 PE=1 SV=2 - (RL22_HUMAN)	4.01	31.25	2	1	4	4	8.723E6	128	14.8	9.19
A2ABS5	Dimethylarginine dimethylaminohydrolase 2 (Fragment) OS=Homo sapiens GN=DDAH2 PE=4 SV=1 - (A2ABS5_HUMAN)	4.00	11.23	6	1	2	6	9.190E7	187	19.9	8.09
Q53EM5	Transketolase variant (Fragment) OS=Homo sapiens PE=2 SV=1 - (Q53EM5_HUMAN)	3.95	10.27	8	1	5	5	4.662E8	623	67.9	7.81
Q13098-5	Isoform 4 of COP9 signalosome complex subunit 1 OS=Homo sapiens GN=GPS1 - (CSN1_HUMAN)	3.94	13.96	8	1	6	9	6.419E7	487	55.1	6.61
P49721	Proteasome subunit beta type-2 OS=Homo sapiens GN=PSMB2 PE=1 SV=1 - (PSB2_HUMAN)	3.93	8.96	2	1	2	2	1.439E7	201	22.8	7.02
D3DNI3	Profilin (Fragment) OS=Homo sapiens GN=PFN2 PE=3 SV=1 - (D3DNI3_HUMAN)	3.93	26.18	10	1	4	4	6.426E5	340	35.5	10.20
Q3SYB5	SERPINB12 protein OS=Homo sapiens GN=SERPINB12 PE=2 SV=1 - (Q3SYB5_HUMAN)	3.92	4.37	3	1	1	2	3.844E6	183	20.9	5.87
O14957	Cytochrome b-c1 complex subunit 10 OS=Homo sapiens GN=UQCR11 PE=2 SV=1 - (QCR10_HUMAN)	3.92	21.43	1	1	1	1	4.820E5	56	6.6	9.88
Q86UY0	TXNDC5 protein OS=Homo sapiens GN=TXNDC5 PE=2 SV=1 - (Q86UY0_HUMAN)	3.90	11.39	5	1	6	7	1.341E7	360	40.3	5.83
Q59FJ2	Ubiquilin 1 isoform 1 variant (Fragment) OS=Homo sapiens PE=2 SV=1 - (Q59FJ2_HUMAN)	3.89	4.62	9	1	1	2	1.161E7	325	34.7	5.30
A8K7S0	cDNA FLJ75478, highly similar to Homo sapiens splicing factor 4 (SF4), transcript variant a, mRNA OS=Homo sapiens PE=2 SV=1 - (A8K7S0_HUMAN)	3.88	8.37	7	1	5	5	2.491E7	645	72.4	7.61
Q8NHH1	Tubulin polyglutamylase TTLL11 OS=Homo sapiens GN=TTLL11 PE=2 SV=1 - (TTL11_HUMAN)	3.87	2.79	3	1	2	7	1.838E7	538	58.0	9.20
Q9HB15	Potassium channel subfamily K member 12 OS=Homo sapiens GN=KCNK12 PE=1 SV=1 - (KCNKC_HUMAN)	3.85	10.23	1	1	3	4	1.091E7	430	46.9	9.77
B4DF76	cDNA FLJ59191, highly similar to NADH dehydrogenase (ubiquinone) 1 alpha subcomplex subunit 13 (EC 1.6.5.3) OS=Homo sapiens PE=2 SV=1 - (B4DF76_HUMAN)	3.85	27.48	2	1	4	4	3.145E6	222	24.9	9.70
Q14692	Ribosome biogenesis protein BMS1 homolog OS=Homo sapiens GN=BMS1 PE=1 SV=1 - (BMS1_HUMAN)	3.82	3.98	1	1	5	20	8.768E7	1282	145.7	6.44
P22531	Small proline-rich protein 2E OS=Homo sapiens GN=SPRR2E PE=2 SV=2 - (SPR2E_HUMAN)	3.80	61.11	8	1	3	3	1.918E6	72	7.8	8.31
C9JLK5	Eukaryotic translation elongation factor 1 epsilon-1 OS=Homo sapiens GN=EEF1E1 PE=4 SV=1 - (C9JLK5_HUMAN)	3.79	30.22	5	1	3	6	1.985E6	139	15.5	8.34
Q5JYB1	Zinc finger and BTB domain containing 17 (Fragment) OS=Homo sapiens GN=ZBTB17 PE=2 SV=1 - (Q5JYB1_HUMAN)	3.78	7.92	2	1	2	31	5.458E6	240	26.6	9.03
B1ALC0	Actin-related protein 2/3 complex subunit 5 OS=Homo sapiens GN=ARPC5 PE=3 SV=1 - (B1ALC0_HUMAN)	3.77	20.74	4	1	3	4	4.269E7	135	14.8	5.06
Q9Y2S1	Galanin-related peptide OS=Homo sapiens PE=4 SV=1 - (Q9Y2S1_HUMAN)	3.76	9.43	1	1	1	14	1.944E8	106	11.8	5.97
Q9H6F5	Coiled-coil domain-containing protein 86 OS=Homo sapiens GN=CCDC86 PE=1 SV=1 - (CCD86_HUMAN)	3.72	9.44	1	1	4	6	4.897E9	360	40.2	10.33
O00470-2	Isoform 2 of Homeobox protein Meis1 OS=Homo sapiens GN=MEIS1 - (MEIS1_HUMAN)	3.71	9.72	2	1	3	7	3.804E6	463	50.5	6.55
B1AK87	Capping protein (Actin filament) muscle Z-line, beta OS=Homo sapiens GN=CAPZB PE=4 SV=1 - (B1AK87_HUMAN)	3.71	9.62	8	2	3	3	1.075E6	260	29.3	6.92
P62699	Protein yippee-like 5 OS=Homo sapiens GN=YPEL5 PE=2 SV=1 - (YPEL5_HUMAN)	3.70	11.57	1	1	1	1	6.172E5	121	13.8	7.31
P46781	40S ribosomal protein S9 OS=Homo sapiens GN=RPS9 PE=1 SV=3 - (RS9_HUMAN)	3.70	21.13	6	1	6	7	2.026E7	194	22.6	10.65
O75445	Usherin OS=Homo sapiens GN=USH2A PE=1 SV=3 - (USH2A_HUMAN)	3.70	3.11	3	1	11	17	1.751E8	5202	575.2	6.83
B4DU58	Macrophage-capping protein OS=Homo sapiens GN=CAPG PE=2 SV=1 - (B4DU58_HUMAN)	3.69	7.34	5	1	2	2	1.510E6	327	36.2	6.25
P62633-2	Isoform 2 of Cellular nucleic acid-binding protein OS=Homo sapiens GN=CNBP - (CNBP_HUMAN)	3.68	20.59	7	1	3	4	6.626E6	170	18.7	7.71
F5H4D0	Protein CLEC16A OS=Homo sapiens GN=CLEC16A PE=4 SV=1 - (F5H4D0_HUMAN)	3.66	4.18	2	1	4	8	2.808E7	1053	117.6	5.86
Q6NWR3	G protein-coupled receptor 83 OS=Homo sapiens GN=GPR83 PE=2 SV=1 - (Q6NWR3_HUMAN)	3.63	13.95	1	1	4	7	2.200E7	423	48.3	9.23
D6RJD6	NADH dehydrogenase (ubiquinone) 1 alpha subcomplex subunit 2 OS=Homo sapiens GN=NDUFA2 PE=4 SV=1 - (D6RJD6_HUMAN)	3.63	36.84	2	1	3	4	5.666E6	76	8.5	10.11
Q5T3W0	Absent in melanoma 2 (Fragment) OS=Homo sapiens GN=AIM2 PE=2 SV=1 - (Q5T3W0_HUMAN)	3.57	10.78	1	1	2	4	4.374E6	167	19.1	9.51
P00441	Superoxide dismutase (Cu-Zn) OS=Homo sapiens GN=SOD1 PE=1 SV=2 - (SODC_HUMAN)	3.56	19.48	3	1	2	2	7.710E6	154	15.9	6.13
O15399	Glutamate (NMDA) receptor subunit epsilon-4 OS=Homo sapiens GN=GRIN2D PE=2 SV=2 - (NMDE4_HUMAN)	3.53	2.10	1	1	3	11	1.866E7	1336	143.7	8.35
F8WAS3	NADH dehydrogenase (ubiquinone) 1 alpha subcomplex subunit 5 OS=Homo sapiens GN=NDUFA5 PE=4 SV=1 - (F8WAS3_HUMAN)	3.52	14.29	5	1	1	1	9.831E5	70	7.8	9.44
H7C2V0	Fatty acid desaturase 1 (Fragment) OS=Homo sapiens GN=FADS1 PE=3 SV=1 - (H7C2V0_HUMAN)	3.50	12.28	1	1	2	13	1.235E8	228	25.5	7.96
Q06323	Proteasome activator complex subunit 1 OS=Homo sapiens GN=PSME1 PE=1 SV=1 - (PSME1_HUMAN)	3.46	12.45	3	1	2	2	2.686E6	249	28.7	6.02
B4DGV0	cDNA FLJ60251, highly similar to Contactin-6 OS=Homo sapiens PE=2 SV=1 - (B4DGV0_HUMAN)	3.46	5.44	2	1	4	5	4.410E7	956	105.8	5.86
B4DMT0	Transmembrane protein 143 OS=Homo sapiens GN=TMEM143 PE=2 SV=1 - (B4DMT0_HUMAN)	3.45	8.02	3	1	4	11	8.150E6	424	47.7	9.92
Q5TA44	Cleavage and polyadenylation specific factor 3-like (Fragment) OS=Homo sapiens GN=CPSF3L PE=2 SV=1 - (Q5TA44_HUMAN)	3.43	10.72	11	1	3	7	1.318E8	401	45.1	7.56
Q96HE7	ERO1-like protein alpha OS=Homo sapiens GN=ERO1L PE=1 SV=2 - (ERO1A_HUMAN)	3.39	5.34	1	1	3	4	1.050E8	468	54.4	5.68
P04080	Cystatin-B OS=Homo sapiens GN=CSTB PE=1 SV=2 - (CYTB_HUMAN)	3.38	18.37	1	1	2	3	8.257E6	98	11.1	7.56
Q92688-2	Isoform 2 of Acidic leucine-rich nuclear phosphoprotein 32 family member B OS=Homo sapiens GN=ANP32B - (AN32B_HUMAN)	3.38	13.33	10	2	3	4	7.782E6	195	22.3	4.30
Q8NEK5	Zinc finger protein 548 OS=Homo sapiens GN=ZNF548 PE=2 SV=2 - (ZN548_HUMAN)	3.38	4.13	3	1	2	3	3.979E6	533	62.7	8.03
Q9HBA9	Putative N-acetylated-alpha-linked acidic dipeptidase OS=Homo sapiens GN=FOLH1B PE=2 SV=1 - (FOH1B_HUMAN)	3.38	15.16	1	1	4	7	1.393E8	442	50.0	7.02
A6NM62	Leucine-rich repeat-containing protein 53 OS=Homo sapiens GN=LRRC53 PE=4 SV=2 - (LRC53_HUMAN)	3.36	3.69	2	1	5	6	2.050E7	1247	140.7	7.91
B7ZBU0	Novel protein (Fragment) OS=Homo sapiens GN=RP11-60H7.2-001 PE=4 SV=1 - (B7ZBU0_HUMAN)	3.36	12.66	2	1	2	2	5.546E6	403	46.2	8.35
H0YJG0	Creatine kinase B-type (Fragment) OS=Homo sapiens GN=CKB PE=3 SV=1 - (H0YJG0_HUMAN)	3.35	9.50	4	1	1	1	1.063E6	179	20.1	6.70
Q14697-2	Isoform 2 of Neutral alpha-glucosidase AB OS=Homo sapiens GN=GANAB - (GANAB_HUMAN)	3.33	6.42	3	1	6	7	5.491E6	966	109.4	6.24
Q5VXS2	Ubiquitin-fold modifier 1 (Fragment) OS=Homo sapiens GN=UFM1 PE=2 SV=1 - (Q5VXS2_HUMAN)	3.30	18.75	3	1	1	1	4.774E6	80	8.5	9.47
B2RD96	cDNA, FLJ96514 OS=Homo sapiens PE=2 SV=1 - (B2RD96_HUMAN)	3.23	8.06	2	1	1	1		211	23.4	8.91
Q49AN9	SNRPG protein OS=Homo sapiens GN=SNRPG PE=4 SV=1 - (Q49AN9_HUMAN)	3.20	20.31	4	1	1	1	7.325E5	64	7.1	7.18
Q9NP97	Dynein light chain roadblock-type 1 OS=Homo sapiens GN=DYNLRB1 PE=1 SV=3 - (DLRB1_HUMAN)	3.14	21.88	5	1	2	6	3.374E6	96	10.9	7.25
Q6IPX5	Ribosomal protein S2 OS=Homo sapiens GN=RPS2 PE=2 SV=1 - (Q6IPX5_HUMAN)	3.11	31.06	11	1	8	9	8.440E7	293	31.3	10.24
B4E3K9	Superoxide dismutase OS=Homo sapiens GN=SOD2 PE=2 SV=1 - (B4E3K9_HUMAN)	3.11	8.64	5	1	1	1	1.624E6	162	18.3	8.41
E5RJY1	Protein NDRG1 OS=Homo sapiens GN=NDRG1 PE=4 SV=1 - (E5RJY1_HUMAN)	3.06	13.59	15	1	1	1	1.433E6	103	11.0	8.98
E9PNW4	CD59 glycoprotein OS=Homo sapiens GN=CD59 PE=4 SV=1 - (E9PNW4_HUMAN)	3.05	10.19	3	1	2	2	3.449E7	108	12.0	5.74
P62888	60S ribosomal protein L30 OS=Homo sapiens GN=RPL30 PE=1 SV=2 - (RL30_HUMAN)	3.04	21.74	2	1	2	8	1.125E8	115	12.8	9.63
H0YLR1	Protein FAM63B (Fragment) OS=Homo sapiens GN=FAM63B PE=4 SV=1 - (H0YLR1_HUMAN)	2.98	6.45	4	1	1	1	5.904E6	155	17.8	4.58
F5H022	Histone H1.0 OS=Homo sapiens GN=H1F0 PE=3 SV=1 - (F5H022_HUMAN)	2.90	5.65	3	1	1	1	3.394E6	177	19.2	10.76
Q6L8H4	Keratin-associated protein 5-1 OS=Homo sapiens GN=KRTAP5-1 PE=2 SV=1 - (KRA51_HUMAN)	2.88	3.96	1	1	1	1	5.911E5	278	24.2	7.91
B4DFV7	cDNA FLJ60481 OS=Homo sapiens PE=2 SV=1 - (B4DFV7_HUMAN)	2.88	7.35	4	1	3	4	5.002E7	612	67.2	5.49
B7Z4B2	cDNA FLJ56108, highly similar to Puromycin-sensitive aminopeptidase (EC 3.4.11.-) OS=Homo sapiens PE=2 SV=1 - (B7Z4B2_HUMAN)	2.83	4.61	9	1	3	4	3.038E7	825	92.8	5.40
P61254	60S ribosomal protein L26 OS=Homo sapiens GN=RPL26 PE=1 SV=1 - (RL26_HUMAN)	2.80	15.17	8	2	3	3	6.548E6	145	17.2	10.55
P06703	Protein S100-A6 OS=Homo sapiens GN=S100A6 PE=1 SV=1 - (S10A6_HUMAN)	2.76	8.89	1	1	1	2	3.994E7	90	10.2	5.48
P04626-4	Isoform 4 of Receptor tyrosine-protein kinase erbB-2 OS=Homo sapiens GN=ERBB2 - (ERBB2_HUMAN)	2.75	3.95	5	1	4	12	1.516E8	1240	136.4	6.00
E9PNA4	Transcriptional activator Myb OS=Homo sapiens GN=MYB PE=4 SV=1 - (E9PNA4_HUMAN)	2.74	8.05	5	1	5	6	3.018E7	745	83.6	7.24
Q53FH6	Mitogen-activated protein kinase kinase 1 interacting protein 1 variant (Fragment) OS=Homo sapiens PE=2 SV=1 - (Q53FH6_HUMAN)	2.73	8.06	2	1	1	1	2.162E6	124	13.6	7.34
Q32XH3	Ribosomal protein L18a-like protein OS=Homo sapiens PE=2 SV=1 - (Q32XH3_HUMAN)	2.72	27.94	4	1	3	3	1.515E7	136	15.8	10.27
B4DEL6	Phospholipase D3 OS=Homo sapiens GN=PLD3 PE=2 SV=1 - (B4DEL6_HUMAN)	2.72	3.98	2	1	2	2	3.348E5	402	44.8	6.46
Q96AZ6	Interferon-stimulated gene 20 kDa protein OS=Homo sapiens GN=ISG20 PE=1 SV=2 - (ISG20_HUMAN)	2.70	11.60	1	1	2	2	8.131E6	181	20.4	8.92
Q00688	Peptidyl-prolyl cis-trans isomerase FKBP3 OS=Homo sapiens GN=FKBP3 PE=1 SV=1 - (FKBP3_HUMAN)	2.70	10.27	2	1	2	8	9.913E8	224	25.2	9.28
Q96BK5	PIN2/TERF1-interacting telomerase inhibitor 1 OS=Homo sapiens GN=PINX1 PE=1 SV=2 - (PINX1_HUMAN)	2.70	8.54	1	1	2	7	1.140E7	328	37.0	9.60
A8K0T9	cDNA FLJ75422, highly similar to Homo sapiens capping protein (actin filament) muscle Z-line, alpha 1, mRNA OS=Homo sapiens PE=2 SV=1 - (A8K0T9_HUMAN)	2.69	5.24	2	1	1	1	4.163E6	286	32.9	5.69
H7C1C6	Translocon-associated protein subunit delta (Fragment) OS=Homo sapiens GN=SSR4 PE=4 SV=1 - (H7C1C6_HUMAN)	2.68	15.83	3	1	1	1	1.499E6	120	13.1	7.50
D3YTB1	60S ribosomal protein L32 (Fragment) OS=Homo sapiens GN=RPL32 PE=4 SV=1 - (D3YTB1_HUMAN)	2.67	12.78	3	1	2	2	1.110E7	133	15.6	11.44
Q53ER2	Cyclin D2 variant (Fragment) OS=Homo sapiens PE=2 SV=1 - (Q53ER2_HUMAN)	2.61	9.34	1	1	1	1	3.980E6	289	33.1	5.19
B5MD17	Chromobox protein homolog 1 (Fragment) OS=Homo sapiens GN=CBX1 PE=4 SV=1 - (B5MD17_HUMAN)	2.57	17.86	3	1	2	2	1.241E6	168	19.3	5.02
O95336	6-phosphogluconolactonase OS=Homo sapiens GN=PGLS PE=1 SV=2 - (6PGL_HUMAN)	2.57	8.14	1	1	2	2	8.189E6	258	27.5	6.05
F8VQ14	T-complex protein 1 subunit beta OS=Homo sapiens GN=CCT2 PE=3 SV=1 - (F8VQ14_HUMAN)	2.55	13.94	4	1	4	6	1.043E8	416	44.8	6.54
A8K3Y8	cDNA FLJ76141 OS=Homo sapiens PE=2 SV=1 - (A8K3Y8_HUMAN)	2.53	12.46	5	1	2	2	6.898E6	297	33.7	7.31
Q5HYM2	Putative uncharacterized protein DKFZp686O2462 (Fragment) OS=Homo sapiens GN=DKFZp686O2462 PE=2 SV=1 - (Q5HYM2_HUMAN)	2.52	5.43	4	1	3	3	3.240E7	626	72.0	5.36
Q14161-11	Isoform 11 of ARF GTPase-activating protein GIT2 OS=Homo sapiens GN=GIT2 - (GIT2_HUMAN)	2.52	8.87	16	1	5	9	2.698E7	631	70.1	6.57
Q9Y5P6-2	Isoform 2 of Mannose-1-phosphate guanyltransferase beta OS=Homo sapiens GN=GMPPB - (GMPPB_HUMAN)	2.51	10.34	2	1	3	6	2.178E7	387	42.6	6.29
I3L0K2	Thioredoxin domain-containing protein 17 OS=Homo sapiens GN=TXNDC17 PE=4 SV=1 - (I3L0K2_HUMAN)	2.51	13.33	3	1	1	1	1.937E6	75	8.4	5.76
Q5JQR1	Testis expressed 11 (Fragment) OS=Homo sapiens GN=TEX11 PE=2 SV=1 - (Q5JQR1_HUMAN)	2.49	7.72	5	1	6	8	2.171E7	920	105.8	5.14
B4E241	Serine/arginine-rich-splicing factor 3 OS=Homo sapiens GN=SRSF3 PE=2 SV=1 - (B4E241_HUMAN)	2.47	16.13	2	1	2	3	2.248E7	124	14.2	10.08
Q9H3A6	PRO2179 OS=Homo sapiens PE=2 SV=1 - (Q9H3A6_HUMAN)	2.47	21.43	2	1	2	3	1.468E7	140	15.6	9.16
B4DHP2	Prostaglandin E synthase 3 OS=Homo sapiens GN=PTGES3 PE=2 SV=1 - (B4DHP2_HUMAN)	2.47	10.24	5	1	1	1	5.345E6	127	14.8	4.11
Q9Y3U8	60S ribosomal protein L36 OS=Homo sapiens GN=RPL36 PE=1 SV=3 - (RL36_HUMAN)	2.47	26.67	6	2	3	3	5.430E7	105	12.2	11.59
B3KRM2	Serine/threonine-protein phosphatase OS=Homo sapiens PE=2 SV=1 - (B3KRM2_HUMAN)	2.46	11.00	10	2	4	6	6.261E6	309	35.5	5.43
I3L1R8	Trafficking protein particle complex subunit 1 OS=Homo sapiens GN=TRAPPC1 PE=4 SV=1 - (I3L1R8_HUMAN)	2.41	42.86	1	1	1	6	3.929E8	42	5.0	7.44
B4DKB2	Endothelin-converting enzyme 1 OS=Homo sapiens GN=ECE1 PE=2 SV=1 - (B4DKB2_HUMAN)	2.36	5.69	5	1	4	5	7.714E6	738	83.5	5.73
P25788-2	Isoform 2 of Proteasome subunit alpha type-3 OS=Homo sapiens GN=PSMA3 - (PSA3_HUMAN)	2.34	20.56	3	2	4	4	4.921E6	248	27.6	5.33
A4VCH6	LONP1 protein (Fragment) OS=Homo sapiens GN=LONP1 PE=2 SV=1 - (A4VCH6_HUMAN)	2.28	4.38	1	1	1	1	1.027E7	297	34.2	5.39
H7BZJ8	Zinc-alpha-2-glycoprotein (Fragment) OS=Homo sapiens GN=AZGP1 PE=4 SV=1 - (H7BZJ8_HUMAN)	2.28	35.29	4	1	2	5	1.339E7	85	9.7	9.70
O95833	Chloride intracellular channel protein 3 OS=Homo sapiens GN=CLIC3 PE=1 SV=2 - (CLIC3_HUMAN)	2.28	5.08	1	1	2	2	7.681E6	236	26.6	6.43
P56556	NADH dehydrogenase (ubiquinone) 1 alpha subcomplex subunit 6 OS=Homo sapiens GN=NDUFA6 PE=1 SV=3 - (NDUA6_HUMAN)	2.25	10.39	1	1	2	2	2.111E6	154	17.9	10.14
H7BY78	Hypoxanthine-guanine phosphoribosyltransferase OS=Homo sapiens GN=HPRT1 PE=4 SV=1 - (H7BY78_HUMAN)	2.24	4.59	3	1	1	1	1.068E6	218	24.6	7.02
C6K4L3	MHC class I antigen (Fragment) OS=Homo sapiens GN=HLA-C PE=3 SV=1 - (C6K4L3_HUMAN)	2.22	11.60	1	1	2	4	1.085E7	181	20.8	6.09
H0YEM8	Uncharacterized protein KIAA0408 (Fragment) OS=Homo sapiens GN=KIAA0408 PE=4 SV=1 - (H0YEM8_HUMAN)	2.18	13.64	1	1	1	1	1.326E7	88	10.0	8.40
H0YDB2	Stromal interaction molecule 1 (Fragment) OS=Homo sapiens GN=STIM1 PE=4 SV=1 - (H0YDB2_HUMAN)	2.18	7.90	1	1	4	8	5.203E7	405	45.3	6.84
Q8TE77-3	Isoform 3 of Protein phosphatase Slingshot homolog 3 OS=Homo sapiens GN=SSH3 - (SSH3_HUMAN)	2.18	4.29	4	1	2	2	2.975E7	513	56.9	5.45
Q13018-2	Isoform 2 of Secretory phospholipase A2 receptor OS=Homo sapiens GN=PLA2R1 - (PLA2R_HUMAN)	2.17	1.36	3	1	2	2	3.229E6	1324	152.7	6.09
A8K6K4	cDNA FLJ77565, highly similar to Homo sapiens interleukin 1 receptor accessory protein (IL1RAP), transcript variant 1, mRNA OS=Homo sapiens PE=2 SV=1 - (A8K6K4_HUMAN)	2.15	6.84	6	1	3	5	1.952E7	570	65.4	8.22
B7Z1K1	cDNA FLJ60431, highly similar to Regulator of G-protein signaling 11 OS=Homo sapiens PE=2 SV=1 - (B7Z1K1_HUMAN)	2.14	10.23	6	1	3	3	3.140E6	352	39.1	9.36
A8MUL3	Putative uncharacterized protein ADARB2-AS1 OS=Homo sapiens GN=ADARB2-AS1 PE=5 SV=2 - (ADAS1_HUMAN)	2.13	14.29	1	1	2	18	2.317E8	147	15.8	7.81
F2Z2D5	Protein POF1B OS=Homo sapiens GN=POF1B PE=4 SV=1 - (F2Z2D5_HUMAN)	2.13	3.68	4	1	1	1		299	34.2	6.73
H0YJM8	Proteasome subunit beta type-5 (Fragment) OS=Homo sapiens GN=PSMB5 PE=3 SV=1 - (H0YJM8_HUMAN)	2.13	22.88	3	1	2	3	2.897E6	118	12.8	9.57
Q9BY89	Uncharacterized protein KIAA1671 OS=Homo sapiens GN=KIAA1671 PE=1 SV=2 - (K1671_HUMAN)	2.12	7.03	1	1	8	11	6.432E7	1806	196.6	8.47
Q9H0R4-2	Isoform 2 of Haloacid dehalogenase-like hydrolase domain-containing protein 2 OS=Homo sapiens GN=HDHD2 - (HDHD2_HUMAN)	2.08	4.73	2	1	1	1	2.667E6	169	18.5	5.54
G3V2B8	Methylenetetrahydrofolate dehydrogenase OS=Homo sapiens GN=MTHFD1 PE=3 SV=1 - (G3V2B8_HUMAN)	2.05	3.74	4	1	3	3	3.048E6	935	101.5	7.30
O15144	Actin-related protein 2/3 complex subunit 2 OS=Homo sapiens GN=ARPC2 PE=1 SV=1 - (ARPC2_HUMAN)	2.04	10.00	2	1	3	3	6.972E6	300	34.3	7.36
B7Z6B3	cDNA FLJ53094, highly similar to Receptor expression-enhancing protein 5 OS=Homo sapiens PE=2 SV=1 - (B7Z6B3_HUMAN)	2.03	5.13	4	1	1	1		156	17.7	8.65
Q9Y6D5	Brefeldin A-inhibited guanine nucleotide-exchange protein 2 OS=Homo sapiens GN=ARFGEF2 PE=1 SV=3 - (BIG2_HUMAN)	2.02	5.60	3	1	10	18	1.330E8	1785	201.9	6.33
Q59EJ5	Glutathione S-transferase M3 variant (Fragment) OS=Homo sapiens PE=2 SV=1 - (Q59EJ5_HUMAN)	2.02	21.65	17	1	4	5	1.462E7	231	27.2	6.02
E0YMJ4	HNF1 alpha A splice variant 8 OS=Homo sapiens GN=HNF1A PE=2 SV=1 - (E0YMJ4_HUMAN)	2.01	7.87	18	1	2	5	2.773E7	394	42.6	6.39
B7ZLL0	KIAA0528 protein OS=Homo sapiens GN=KIAA0528 PE=2 SV=1 - (B7ZLL0_HUMAN)	2.01	5.13	8	1	4	7	2.401E7	1053	116.3	6.39
A6NMS2	Ribose-phosphate pyrophosphokinase 2 (Fragment) OS=Homo sapiens GN=PRPS2 PE=4 SV=2 - (A6NMS2_HUMAN)	2.00	21.77	12	1	2	2	2.447E6	147	16.1	6.99
O95803	Bifunctional heparan sulfate N-deacetylase/N-sulfotransferase 3 OS=Homo sapiens GN=NDST3 PE=2 SV=1 - (NDST3_HUMAN)	1.98	2.75	1	1	3	4	1.935E8	873	100.8	8.06
B2RAN0	cDNA, FLJ95010, highly similar to Homo sapiens Bloom syndrome (BLM), mRNA OS=Homo sapiens PE=2 SV=1 - (B2RAN0_HUMAN)	1.96	4.87	8	1	5	7	4.275E7	1417	158.9	7.56
C9K0U8	Single-stranded DNA-binding protein, mitochondrial (Fragment) OS=Homo sapiens GN=SSBP1 PE=4 SV=1 - (C9K0U8_HUMAN)	1.93	4.96	5	1	1	1		121	14.1	9.57
Q9NSK3	Putative uncharacterized protein DKFZp762I166 (Fragment) OS=Homo sapiens GN=DKFZp762I166 PE=2 SV=1 - (Q9NSK3_HUMAN)	1.93	12.01	2	1	4	4	4.172E7	283	31.4	5.15
A8K9U7	cDNA FLJ75829, highly similar to Homo sapiens potassium inwardly-rectifying channel, subfamily J, member 15 (KCNJ15), transcript variant 2, mRNA OS=Homo sapiens PE=2 SV=1 - (A8K9U7_HUMAN)	1.92	9.60	2	1	4	5	2.652E7	375	42.5	7.69
B2REA7	Ribosomal protein L36a OS=Homo sapiens GN=RPL36A PE=3 SV=1 - (B2REA7_HUMAN)	1.92	26.79	6	1	3	3	3.751E6	112	13.2	10.48
B7Z2T1	cDNA FLJ58061, highly similar to Eukaryotic translation initiation factor 4E OS=Homo sapiens PE=2 SV=1 - (B7Z2T1_HUMAN)	1.92	5.15	7	1	1	1		136	16.3	5.91
H3BLV8	Vacuolar protein sorting-associated protein 29 (Fragment) OS=Homo sapiens GN=VPS29 PE=4 SV=1 - (H3BLV8_HUMAN)	1.89	8.04	5	1	2	2	2.311E6	199	22.3	7.62
D3VVI3	Ataxin 3 variant ref (Fragment) OS=Homo sapiens GN=ATXN3 PE=2 SV=1 - (D3VVI3_HUMAN)	1.87	11.55	1	1	3	12	1.055E8	329	37.8	4.89
B4DI85	ADP-ribosylation factor-like 8B, isoform CRA_a OS=Homo sapiens GN=ARL8B PE=2 SV=1 - (B4DI85_HUMAN)	1.87	3.68	4	1	1	1	1.753E6	163	18.6	5.99
E3VWE2	Regulator of calcineurin 3 isoform 1,3,4,5 OS=Homo sapiens GN=RCAN3 PE=4 SV=1 - (E3VWE2_HUMAN)	1.85	6.90	1	1	1	1		116	12.7	4.82
F5GZ04	Zinc finger and SCAN domain-containing protein 2 OS=Homo sapiens GN=ZSCAN2 PE=4 SV=1 - (F5GZ04_HUMAN)	1.83	9.05	11	1	2	4	4.748E6	210	23.7	4.36
Q8NEJ1	Putative uncharacterized protein OS=Homo sapiens PE=2 SV=1 - (Q8NEJ1_HUMAN)	1.82	8.90	21	1	2	6	2.631E8	236	25.0	7.69
Q56VW4	Transformation-related gene 6 OS=Homo sapiens PE=4 SV=1 - (Q56VW4_HUMAN)	1.82	8.54	1	1	1	1	4.355E5	82	9.2	9.86
E9PN70	Trafficking protein particle complex subunit 4 OS=Homo sapiens GN=TRAPPC4 PE=4 SV=1 - (E9PN70_HUMAN)	1.81	11.28	5	1	3	10	3.660E6	257	28.8	8.51
B4DNT4	Cardiotrophin-like cytokine factor 1 OS=Homo sapiens GN=CLCF1 PE=2 SV=1 - (B4DNT4_HUMAN)	1.79	3.72	2	1	1	3	1.834E7	215	24.1	8.70
B4DWH7	V-type proton ATPase subunit B, kidney isoform OS=Homo sapiens GN=ATP6V1B1 PE=2 SV=1 - (B4DWH7_HUMAN)	1.79	10.45	4	1	3	3	4.602E7	488	54.3	5.66
E9PJ29	Out at first protein homolog OS=Homo sapiens GN=OAF PE=4 SV=1 - (E9PJ29_HUMAN)	1.79	5.73	2	1	1	1	2.887E6	157	18.1	7.64
F8W7F1	p53 apoptosis effector-related to PMP-22 OS=Homo sapiens GN=PERP PE=4 SV=1 - (F8W7F1_HUMAN)	1.79	4.00	4	1	1	1	3.217E6	175	19.5	7.03
P25786-2	Isoform Long of Proteasome subunit alpha type-1 OS=Homo sapiens GN=PSMA1 - (PSA1_HUMAN)	1.76	12.64	6	1	3	3	4.872E6	269	30.2	6.99
A8MWY0-2	Isoform 2 of UPF0577 protein KIAA1324-like OS=Homo sapiens GN=KIAA1324L - (K132L_HUMAN)	1.76	5.20	5	1	3	4	9.900E6	789	87.0	6.43
Q53H37	Calmodulin-like skin protein variant (Fragment) OS=Homo sapiens PE=2 SV=1 - (Q53H37_HUMAN)	1.75	28.77	2	1	3	3	1.042E7	146	15.9	4.44
G3V1A8	Lymphocyte antigen 6 complex locus protein G6c OS=Homo sapiens GN=LY6G6C PE=4 SV=1 - (G3V1A8_HUMAN)	1.75	11.59	2	1	1	1	1.402E6	69	7.6	7.71
P05089-3	Isoform 3 of Arginase-1 OS=Homo sapiens GN=ARG1 - (ARGI1_HUMAN)	1.74	4.66	3	1	1	1	1.477E6	236	25.3	8.51
O95835	Serine/threonine-protein kinase LATS1 OS=Homo sapiens GN=LATS1 PE=1 SV=1 - (LATS1_HUMAN)	1.73	6.81	2	1	7	8	1.260E8	1130	126.8	8.70
D6RB53	Ufm1-specific protease 2 OS=Homo sapiens GN=UFSP2 PE=4 SV=1 - (D6RB53_HUMAN)	1.73	21.43	4	1	2	2	2.500E6	112	12.8	8.84
H3BQF3	S phase cyclin A-associated protein in the endoplasmic reticulum (Fragment) OS=Homo sapiens GN=SCAPER PE=4 SV=1 - (H3BQF3_HUMAN)	1.72	23.38	1	1	3	4	3.069E7	154	17.2	10.11
Q9H246	Uncharacterized protein C1orf21 OS=Homo sapiens GN=C1orf21 PE=1 SV=1 - (CA021_HUMAN)	1.71	23.14	1	1	3	12	7.814E7	121	13.9	5.22
F5H5Y2	Translocon-associated protein subunit alpha OS=Homo sapiens GN=SSR1 PE=4 SV=1 - (F5H5Y2_HUMAN)	1.70	8.11	8	1	3	4	4.633E6	259	29.4	4.72
B4E0H6	cDNA FLJ52584, highly similar to Heat shock 70 kDa protein 4 OS=Homo sapiens PE=2 SV=1 - (B4E0H6_HUMAN)	1.69	4.61	1	1	2	3	7.289E6	564	63.8	4.84
O75844	CAAX prenyl protease 1 homolog OS=Homo sapiens GN=ZMPSTE24 PE=1 SV=2 - (FACE1_HUMAN)	1.67	1.26	1	1	1	2	5.615E6	475	54.8	7.49
Q3SWV3	LOC389833 protein (Fragment) OS=Homo sapiens GN=LOC389833 PE=2 SV=1 - (Q3SWV3_HUMAN)	1.67	13.73	1	1	3	7	4.211E6	233	26.5	8.47
A8K4K5	cDNA FLJ77843, highly similar to Homo sapiens TATA box binding protein (TBP)-associated factor, RNA polymerase I, A, 48kDa (TAF1A), transcript variant 1, mRNA OS=Homo sapiens PE=2 SV=1 - (A8K4K5_HUMAN)	1.67	15.56	5	1	5	5	1.719E7	450	52.7	9.20
F5H1H8	Activated RNA polymerase II transcriptional coactivator p15 OS=Homo sapiens GN=SUB1 PE=4 SV=1 - (F5H1H8_HUMAN)	1.64	28.46	3	1	4	9	2.610E7	123	14.0	9.73
I3NI19	Neuropeptide B-29 OS=Homo sapiens GN=NPB PE=4 SV=1 - (I3NI19_HUMAN)	1.64	5.13	1	1	1	1	1.230E7	156	16.3	10.33
Q96QS1-3	Isoform 3 of Tetraspanin-32 OS=Homo sapiens GN=TSPAN32 - (TSN32_HUMAN)	1.62	14.62	1	1	3	3	1.003E8	253	27.8	8.76
E9PNP2	Translocon-associated protein subunit beta OS=Homo sapiens GN=SSR2 PE=4 SV=1 - (E9PNP2_HUMAN)	1.60	9.68	10	1	1	1	2.669E6	62	7.0	9.41
O14523	C2 domain-containing protein 2-like OS=Homo sapiens GN=C2CD2L PE=1 SV=3 - (C2C2L_HUMAN)	0.00	8.22	4	1	7	19	1.535E8	706	76.1	7.69
Q03692	Collagen alpha-1(X) chain OS=Homo sapiens GN=COL10A1 PE=1 SV=2 - (COAA1_HUMAN)	0.00	5.59	1	1	2	2	7.895E7	680	66.1	9.67
Q8IUH2	Protein CREG2 OS=Homo sapiens GN=CREG2 PE=1 SV=1 - (CREG2_HUMAN)	0.00	12.07	2	1	3	11	7.007E7	290	32.1	8.98
O60716-8	Isoform 1 of Catenin delta-1 OS=Homo sapiens GN=CTNND1 - (CTND1_HUMAN)	0.00	9.20	38	2	8	14	2.466E7	913	101.8	6.87
P21802-11	Isoform 11 of Fibroblast growth factor receptor 2 OS=Homo sapiens GN=FGFR2 - (FGFR2_HUMAN)	0.00	4.94	29	1	3	3	4.302E7	830	92.7	6.89
Q12967-2	Isoform 2 of Ral guanine nucleotide dissociation stimulator OS=Homo sapiens GN=RALGDS - (GNDS_HUMAN)	0.00	1.58	1	1	2	2	8.535E6	951	104.5	6.54
O75525	KH domain-containing, RNA-binding, signal transduction-associated protein 3 OS=Homo sapiens GN=KHDRBS3 PE=1 SV=1 - (KHDR3_HUMAN)	0.00	15.03	6	1	5	18	1.955E7	346	38.8	7.61
P01704	Ig lambda chain V-II region TOG OS=Homo sapiens PE=1 SV=1 - (LV201_HUMAN)	0.00	18.92	1	1	1	3	2.765E8	111	11.7	8.48
P29966	Myristoylated alanine-rich C-kinase substrate OS=Homo sapiens GN=MARCKS PE=1 SV=4 - (MARCS_HUMAN)	0.00	10.54	1	1	3	4	9.330E6	332	31.5	4.45
O75448-2	Isoform 2 of Mediator of RNA polymerase II transcription subunit 24 OS=Homo sapiens GN=MED24 - (MED24_HUMAN)	0.00	4.30	20	1	5	14	1.190E8	976	108.9	6.87
Q6W4X9	Mucin-6 OS=Homo sapiens GN=MUC6 PE=1 SV=3 - (MUC6_HUMAN)	0.00	1.23	1	1	2	5	1.107E8	2439	256.9	7.39
Q6ZVX7	Non-specific cytotoxic cell receptor protein 1 homolog OS=Homo sapiens GN=NCCRP1 PE=1 SV=1 - (NCRP1_HUMAN)	0.00	7.27	1	1	2	2	1.160E7	275	30.8	6.62
O14788	Tumor necrosis factor ligand superfamily member 11 OS=Homo sapiens GN=TNFSF11 PE=1 SV=1 - (TNF11_HUMAN)	0.00	5.36	3	1	2	2		317	35.5	7.65
Q9UGI0	Ubiquitin thioesterase ZRANB1 OS=Homo sapiens GN=ZRANB1 PE=1 SV=2 - (ZRAN1_HUMAN)	0.00	3.39	1	1	2	2	2.328E7	708	80.9	5.69
B4DQB5	Leukocyte surface antigen CD53 OS=Homo sapiens GN=CD53 PE=2 SV=1 - (B4DQB5_HUMAN)	0.00	4.38	2	1	1	2	1.041E5	160	17.9	8.06
B7Z4K4	Putative ribosomal RNA methyltransferase 1 OS=Homo sapiens GN=FTSJ1 PE=2 SV=1 - (B7Z4K4_HUMAN)	0.00	9.90	1	1	1	2	8.954E7	192	21.2	5.20
Q5T446	Uroporphyrinogen decarboxylase (Fragment) OS=Homo sapiens GN=UROD PE=2 SV=1 - (Q5T446_HUMAN)	0.00	21.76	5	1	3	7	3.745E5	193	21.4	5.20
I3NI52	Dehydrogenase/reductase SDR family member 7C (Fragment) OS=Homo sapiens GN=DHRS7C PE=4 SV=1 - (I3NI52_HUMAN)	0.00	9.85	3	1	2	2	2.341E8	132	15.0	9.99
Q96RX5	NADH ubiquinone oxidoreductase PDSW subunit (RH 16p13.3) (Fragment) OS=Homo sapiens GN=NDUFB10 PE=2 SV=1 - (Q96RX5_HUMAN)	0.00	32.56	5	1	1	1	3.387E6	43	5.1	6.60
Q9BS84	STK4 protein OS=Homo sapiens PE=2 SV=1 - (Q9BS84_HUMAN)	0.00	58.97	1	1	1	1	8.008E7	39	4.6	4.96
B2R5M8	Isocitrate dehydrogenase (NADP) OS=Homo sapiens PE=2 SV=1 - (B2R5M8_HUMAN)	0.00	6.76	7	1	2	2	3.664E6	414	46.6	7.01
B4DEE1	cDNA FLJ56055, highly similar to ER degradation-enhancingalpha-mannosidase-like 2 OS=Homo sapiens PE=2 SV=1 - (B4DEE1_HUMAN)	0.00	5.32	6	1	2	2	1.059E7	357	40.9	5.73
B3KWY2	cDNA FLJ44205 fis, clone THYMU3001379, highly similar to 116 kDa U5 small nuclear ribonucleoprotein component OS=Homo sapiens PE=2 SV=1 - (B3KWY2_HUMAN)	0.00	6.55	9	1	3	5	1.139E7	550	61.0	5.60
C9J0K6	Sorcin OS=Homo sapiens GN=SRI PE=4 SV=1 - (C9J0K6_HUMAN)	0.00	7.10	4	1	1	2	3.049E6	155	17.6	5.60
Q8NF00	FLJ00404 protein (Fragment) OS=Homo sapiens GN=FLJ00404 PE=2 SV=1 - (Q8NF00_HUMAN)	0.00	13.41	1	1	4	9	5.535E7	261	27.9	11.62
B7ZLZ5	TMEM44 protein OS=Homo sapiens GN=TMEM44 PE=2 SV=1 - (B7ZLZ5_HUMAN)	0.00	6.62	1	1	2	2	3.222E6	438	48.3	7.30
Q5HYI2	Putative uncharacterized protein DKFZp313N0733 OS=Homo sapiens GN=DKFZp313N0733 PE=2 SV=1 - (Q5HYI2_HUMAN)	0.00	8.92	5	1	3	5	1.162E7	482	53.0	7.53
Q3KNR5	PAX4 protein OS=Homo sapiens GN=PAX4 PE=2 SV=1 - (Q3KNR5_HUMAN)	0.00	6.23	7	1	1	1	4.238E6	257	28.0	10.10
Q6UWG2	ALLW1950 OS=Homo sapiens GN=UNQ1950 PE=4 SV=1 - (Q6UWG2_HUMAN)	0.00	10.71	1	1	1	1	1.169E7	84	9.3	9.42
B2RB24	cDNA, FLJ95266 OS=Homo sapiens PE=2 SV=1 - (B2RB24_HUMAN)	0.00	4.79	2	1	1	1	2.588E7	292	32.5	5.72
Q65ZQ3	FBRNP OS=Homo sapiens GN=D10S102 PE=2 SV=1 - (Q65ZQ3_HUMAN)	0.00	14.50	1	1	4	6	5.936E7	269	29.3	8.29
B7Z4R9	cDNA FLJ59037 OS=Homo sapiens PE=2 SV=1 - (B7Z4R9_HUMAN)	0.00	14.29	1	1	2	2	6.929E6	133	15.2	10.18
B4DZ94	cDNA FLJ57095 OS=Homo sapiens PE=2 SV=1 - (B4DZ94_HUMAN)	0.00	7.56	2	1	1	1	1.245E7	225	24.8	9.67
F5GWE1	Glycine receptor subunit beta OS=Homo sapiens GN=GLRB PE=3 SV=1 - (F5GWE1_HUMAN)	0.00	4.95	2	1	1	2	7.377E6	303	34.9	8.37
B3Y1W9	Wings apart-like homolog variant 8 (Fragment) OS=Homo sapiens GN=WAPL PE=2 SV=1 - (B3Y1W9_HUMAN)	0.00	22.62	1	1	2	4	2.528E5	84	9.5	12.07
Q9BRL5	CALM3 protein OS=Homo sapiens PE=2 SV=1 - (Q9BRL5_HUMAN)	0.00	15.65	8	1	2	2	1.629E7	147	16.5	4.46
E9PP00	Probable ribonuclease ZC3H12C OS=Homo sapiens GN=ZC3H12C PE=4 SV=1 - (E9PP00_HUMAN)	0.00	4.93	4	1	4	4	5.725E6	852	95.8	6.87
B1AHM3	Transmembrane protein 8 (Five membrane-spanning domains) (Fragment) OS=Homo sapiens GN=TMEM8 PE=4 SV=1 - (B1AHM3_HUMAN)	0.00	5.81	2	1	2	4	2.634E7	740	82.0	8.05

Appendix F 

**Table 8:** Proteins identified in Subject 4 (N4).

Accession	Description	Score	Coverage	# Proteins	# Unique Peptides	# Peptides	# PSMs	Area	# AAs	MW (kDa)	calc. pI
O43790	Keratin, type II cuticular Hb6 OS=Homo sapiens GN=KRT86 PE=1 SV=1 - (KRT86_HUMAN)	12768.64	94.65	13	7	80	5388	2.582E10	486	53.5	5.66
F8W6V8	Keratin, type II cuticular Hb1 OS=Homo sapiens GN=KRT81 PE=3 SV=1 - (F8W6V8_HUMAN)	11086.29	79.75	12	1	72	4903	2.046E10	484	53.1	5.47
P78386	Keratin, type II cuticular Hb5 OS=Homo sapiens GN=KRT85 PE=1 SV=1 - (KRT85_HUMAN)	10623.80	93.49	7	28	81	4479	2.568E10	507	55.8	6.55
P78385	Keratin, type II cuticular Hb3 OS=Homo sapiens GN=KRT83 PE=1 SV=2 - (KRT83_HUMAN)	9864.38	79.72	10	4	73	4438	2.568E10	493	54.2	5.64
A0JNT2	KRT83 protein OS=Homo sapiens GN=KRT83 PE=2 SV=1 - (A0JNT2_HUMAN)	9600.22	84.34	10	1	70	4359	2.568E10	447	49.6	5.39
Q15323	Keratin, type I cuticular Ha1 OS=Homo sapiens GN=KRT31 PE=2 SV=3 - (K1H1_HUMAN)	8625.84	74.28	4	15	54	3613	3.255E10	416	47.2	4.88
H6VRF8	Keratin 1 OS=Homo sapiens GN=KRT1 PE=3 SV=1 - (H6VRF8_HUMAN)	6998.27	72.67	15	54	65	2527	3.063E9	644	66.0	8.12
O76009	Keratin, type I cuticular Ha3-I OS=Homo sapiens GN=KRT33A PE=2 SV=2 - (KT33A_HUMAN)	6906.18	83.17	3	10	50	2835	3.255E10	404	45.9	4.82
Q14525	Keratin, type I cuticular Ha3-II OS=Homo sapiens GN=KRT33B PE=2 SV=3 - (KT33B_HUMAN)	6692.14	83.17	3	15	52	2677	3.080E10	404	46.2	4.84
P13647	Keratin, type II cytoskeletal 5 OS=Homo sapiens GN=KRT5 PE=1 SV=3 - (K2C5_HUMAN)	6171.61	65.76	23	18	80	2753	1.059E10	590	62.3	7.74
P04259	Keratin, type II cytoskeletal 6B OS=Homo sapiens GN=KRT6B PE=1 SV=5 - (K2C6B_HUMAN)	6112.38	72.34	18	2	80	2739	1.059E10	564	60.0	8.00
B4DRR0	cDNA FLJ53910, highly similar to Keratin, type II cytoskeletal 6A OS=Homo sapiens PE=2 SV=1 - (B4DRR0_HUMAN)	6026.37	76.26	22	5	81	2685	1.059E10	535	57.8	8.00
P35908	Keratin, type II cytoskeletal 2 epidermal OS=Homo sapiens GN=KRT2 PE=1 SV=2 - (K22E_HUMAN)	5144.20	78.25	9	41	65	2075	5.465E9	639	65.4	8.00
P02533	Keratin, type I cytoskeletal 14 OS=Homo sapiens GN=KRT14 PE=1 SV=4 - (K1C14_HUMAN)	4934.69	85.38	12	24	64	2194	1.370E10	472	51.5	5.16
B4DKV4	cDNA FLJ60647, highly similar to Keratin, type II cytoskeletal 6B OS=Homo sapiens PE=2 SV=1 - (B4DKV4_HUMAN)	4739.50	68.63	16	2	68	2120	1.059E10	526	55.7	8.43
I3L2N9	Uncharacterized protein OS=Homo sapiens PE=4 SV=1 - (I3L2N9_HUMAN)	4413.44	80.71	5	21	46	1872	3.255E10	394	44.7	4.78
P13645	Keratin, type I cytoskeletal 10 OS=Homo sapiens GN=KRT10 PE=1 SV=6 - (K1C10_HUMAN)	4127.53	69.35	20	37	48	1720	1.076E10	584	58.8	5.21
P35527	Keratin, type I cytoskeletal 9 OS=Homo sapiens GN=KRT9 PE=1 SV=3 - (K1C9_HUMAN)	3775.12	69.02	9	37	40	1402	2.427E9	623	62.0	5.24
Q04695	Keratin, type I cytoskeletal 17 OS=Homo sapiens GN=KRT17 PE=1 SV=2 - (K1C17_HUMAN)	3772.02	86.34	8	18	57	1707	1.370E10	432	48.1	5.02
P08779	Keratin, type I cytoskeletal 16 OS=Homo sapiens GN=KRT16 PE=1 SV=4 - (K1C16_HUMAN)	3643.88	84.99	11	28	57	1594	1.370E10	473	51.2	5.05
O95678	Keratin, type II cytoskeletal 75 OS=Homo sapiens GN=KRT75 PE=1 SV=2 - (K2C75_HUMAN)	3234.01	59.53	17	18	55	1477	1.439E10	551	59.5	7.74
Q92764	Keratin, type I cuticular Ha5 OS=Homo sapiens GN=KRT35 PE=2 SV=5 - (KRT35_HUMAN)	2643.94	59.34	2	16	34	1198	2.381E10	455	50.3	4.91
H0YI76	Keratin, type II cytoskeletal 5 (Fragment) OS=Homo sapiens GN=KRT5 PE=3 SV=1 - (H0YI76_HUMAN)	2590.24	75.62	11	1	33	961	5.578E9	201	23.1	4.97
Q5XKE5	Keratin, type II cytoskeletal 79 OS=Homo sapiens GN=KRT79 PE=1 SV=2 - (K2C79_HUMAN)	2126.15	32.52	8	1	28	1099	9.763E9	535	57.8	7.20
Q701L7	Type II hair keratin 2 OS=Homo sapiens GN=KRTHB2 PE=2 SV=1 - (Q701L7_HUMAN)	1454.04	37.23	2	5	23	793	5.876E9	513	56.6	6.74
Q14532	Keratin, type I cuticular Ha2 OS=Homo sapiens GN=KRT32 PE=1 SV=3 - (K1H2_HUMAN)	1435.32	39.73	2	6	18	655	2.468E10	448	50.3	4.84
B3KVF5	cDNA FLJ16494 fis, clone CTONG3004576, highly similar to Keratin, type I cytoskeletal 15 OS=Homo sapiens PE=2 SV=1 - (B3KVF5_HUMAN)	1422.42	53.51	6	9	29	564	1.370E10	456	49.1	4.79
P15924	Desmoplakin OS=Homo sapiens GN=DSP PE=1 SV=3 - (DESP_HUMAN)	1420.94	48.62	15	105	164	751	1.409E9	2871	331.6	6.81
H9KV34	Uncharacterized protein OS=Homo sapiens PE=3 SV=1 - (H9KV34_HUMAN)	1404.46	43.25	8	1	26	688	5.941E9	437	48.2	4.97
Q07283	Trichohyalin OS=Homo sapiens GN=TCHH PE=1 SV=2 - (TRHY_HUMAN)	1401.04	64.95	28	103	157	963	5.504E8	1943	253.8	5.78
P08727	Keratin, type I cytoskeletal 19 OS=Homo sapiens GN=KRT19 PE=1 SV=4 - (K1C19_HUMAN)	1350.17	30.50	7	2	20	731	1.307E10	400	44.1	5.14
F5GZD1	Keratin, type II cytoskeletal 7 OS=Homo sapiens GN=KRT7 PE=4 SV=1 - (F5GZD1_HUMAN)	1263.86	19.11	4	1	14	891	1.041E10	403	44.9	5.55
Q9NSB2	Keratin, type II cuticular Hb4 OS=Homo sapiens GN=KRT84 PE=1 SV=2 - (KRT84_HUMAN)	1139.53	20.17	6	5	17	695	9.278E9	600	64.8	7.56
Q3SY84	Keratin, type II cytoskeletal 71 OS=Homo sapiens GN=KRT71 PE=1 SV=3 - (K2C71_HUMAN)	1118.35	63.10	4	26	51	779	2.881E9	523	57.3	6.61
B3KY79	cDNA FLJ46620 fis, clone TLUNG2000654, highly similar to Keratin, type II cytoskeletal 7 OS=Homo sapiens PE=2 SV=1 - (B3KY79_HUMAN)	994.01	33.93	25	1	22	670	9.875E9	445	49.0	5.48
Q86Y46	Keratin, type II cytoskeletal 73 OS=Homo sapiens GN=KRT73 PE=1 SV=1 - (K2C73_HUMAN)	955.40	32.04	8	1	28	717	3.548E9	540	58.9	7.23
Q7Z3Y7	Keratin, type I cytoskeletal 28 OS=Homo sapiens GN=KRT28 PE=1 SV=2 - (K1C28_HUMAN)	939.69	28.23	1	3	17	543	9.634E9	464	50.5	5.47
Q7Z794	Keratin, type II cytoskeletal 1b OS=Homo sapiens GN=KRT77 PE=1 SV=3 - (K2C1B_HUMAN)	932.26	20.07	5	1	12	518	3.795E9	578	61.9	5.99
Q7Z3Y8	Keratin, type I cytoskeletal 27 OS=Homo sapiens GN=KRT27 PE=1 SV=2 - (K1C27_HUMAN)	909.66	60.35	1	7	24	427	1.083E9	459	49.8	5.05
Q7RTS7	Keratin, type II cytoskeletal 74 OS=Homo sapiens GN=KRT74 PE=1 SV=2 - (K2C74_HUMAN)	892.93	44.80	7	8	34	677	3.548E9	529	57.8	7.71
Q7Z3Z0	Keratin, type I cytoskeletal 25 OS=Homo sapiens GN=KRT25 PE=1 SV=1 - (K1C25_HUMAN)	873.19	59.11	3	10	26	417	1.083E9	450	49.3	5.08
P05787	Keratin, type II cytoskeletal 8 OS=Homo sapiens GN=KRT8 PE=1 SV=7 - (K2C8_HUMAN)	832.12	36.44	31	1	26	552	8.082E9	483	53.7	5.59
A1A4E9	Keratin 13 OS=Homo sapiens GN=KRT13 PE=2 SV=1 - (A1A4E9_HUMAN)	817.43	43.01	5	3	22	362	1.188E10	458	49.6	4.96
Q9NSB5	Type II hair keratin 1 (Fragment) OS=Homo sapiens GN=KRTHB1 PE=2 SV=1 - (Q9NSB5_HUMAN)	770.89	80.49	1	1	11	456	1.486E10	123	12.9	8.57
B4DRW1	cDNA FLJ55805, highly similar to Keratin, type II cytoskeletal 4 OS=Homo sapiens PE=2 SV=1 - (B4DRW1_HUMAN)	647.86	36.08	12	3	22	370	3.036E9	474	51.7	6.81
Q8WVW5	Putative uncharacterized protein (Fragment) OS=Homo sapiens PE=2 SV=1 - (Q8WVW5_HUMAN)	646.58	75.21	87	9	31	276	2.119E9	363	40.5	6.14
Q99456	Keratin, type I cytoskeletal 12 OS=Homo sapiens GN=KRT12 PE=1 SV=1 - (K1C12_HUMAN)	493.78	23.89	1	1	14	267	2.372E9	494	53.5	4.78
O76015	Keratin, type I cuticular Ha8 OS=Homo sapiens GN=KRT38 PE=2 SV=3 - (KRT38_HUMAN)	442.67	18.86	2	3	11	278	1.129E10	456	50.4	4.84
Q2M2I5	Keratin, type I cytoskeletal 24 OS=Homo sapiens GN=KRT24 PE=1 SV=1 - (K1C24_HUMAN)	417.30	19.43	1	1	12	249	9.358E9	525	55.1	4.96
B7ZBC3	Spectrin repeat containing, nuclear envelope 1 OS=Homo sapiens GN=SYNE1 PE=4 SV=1 - (B7ZBC3_HUMAN)	386.30	5.85	17	2	43	226	3.545E9	8797	1010.4	5.53
P04792	Heat shock protein beta-1 OS=Homo sapiens GN=HSPB1 PE=1 SV=2 - (HSPB1_HUMAN)	384.24	75.61	4	18	18	145	1.990E9	205	22.8	6.40
Q86YZ3	Hornerin OS=Homo sapiens GN=HRNR PE=1 SV=2 - (HORN_HUMAN)	357.27	26.67	2	14	21	193	8.051E7	2850	282.2	10.04
Q7Z3Y9	Keratin, type I cytoskeletal 26 OS=Homo sapiens GN=KRT26 PE=1 SV=2 - (K1C26_HUMAN)	342.94	14.96	1	1	6	198	8.034E8	468	51.9	4.92
P31947	14-3-3 protein sigma OS=Homo sapiens GN=SFN PE=1 SV=1 - (1433S_HUMAN)	341.00	73.79	11	18	27	159	1.370E9	248	27.8	4.74
Q8N1N4	Keratin, type II cytoskeletal 78 OS=Homo sapiens GN=KRT78 PE=2 SV=2 - (K2C78_HUMAN)	331.77	30.00	4	3	18	195	6.900E9	520	56.8	6.02
B7Z6P1	cDNA FLJ53662, highly similar to Actin, alpha skeletal muscle OS=Homo sapiens PE=2 SV=1 - (B7Z6P1_HUMAN)	324.09	40.23	51	1	21	172	1.266E9	343	38.6	5.38
Q8TC04	Keratin 23 (Histone deacetylase inducible) OS=Homo sapiens GN=KRT23 PE=2 SV=1 - (Q8TC04_HUMAN)	319.59	9.95	1	1	5	245	2.100E9	422	48.1	6.54
F8VW92	Tubulin beta chain OS=Homo sapiens GN=TUBB PE=3 SV=1 - (F8VW92_HUMAN)	317.52	55.63	43	2	19	134	1.554E8	435	48.5	5.20
P07355	Annexin A2 OS=Homo sapiens GN=ANXA2 PE=1 SV=2 - (ANXA2_HUMAN)	310.95	73.75	27	25	30	119	6.617E8	339	38.6	7.75
Q53GA7	Tubulin alpha 6 variant (Fragment) OS=Homo sapiens PE=2 SV=1 - (Q53GA7_HUMAN)	303.98	51.00	24	3	22	106	2.613E8	449	49.8	5.14
O60814	Histone H2B type 1-K OS=Homo sapiens GN=HIST1H2BK PE=1 SV=3 - (H2B1K_HUMAN)	291.72	55.56	32	9	11	144	1.354E9	126	13.9	10.32
P68371	Tubulin beta-4B chain OS=Homo sapiens GN=TUBB4B PE=1 SV=1 - (TBB4B_HUMAN)	279.18	51.24	22	1	19	126	1.554E8	445	49.8	4.89
P14923	Junction plakoglobin OS=Homo sapiens GN=JUP PE=1 SV=3 - (PLAK_HUMAN)	265.75	41.61	13	21	26	138	1.264E8	745	81.7	6.14
B2R6L0	cDNA, FLJ93005, highly similar to Homo sapiens tubulin, beta polypeptide (TUBB), mRNA OS=Homo sapiens PE=2 SV=1 - (B2R6L0_HUMAN)	259.56	51.24	25	1	19	121	1.685E8	445	49.9	4.89
P47929	Galectin-7 OS=Homo sapiens GN=LGALS7 PE=1 SV=2 - (LEG7_HUMAN)	252.11	93.38	1	12	15	98	6.741E8	136	15.1	7.62
B7TY16	Actinin alpha 1 isoform 3 OS=Homo sapiens GN=ACTN1 PE=2 SV=1 - (B7TY16_HUMAN)	248.03	29.03	26	6	24	91	2.040E8	930	107.1	5.58
A8MUB1	Tubulin alpha-4A chain OS=Homo sapiens GN=TUBA4A PE=2 SV=1 - (A8MUB1_HUMAN)	245.87	46.88	14	3	20	89	2.453E8	433	48.3	5.01
O15446-2	Isoform 2 of DNA-directed RNA polymerase I subunit RPA34 OS=Homo sapiens GN=CD3EAP - (RPA34_HUMAN)	239.17	3.71	7	1	2	114	2.264E8	512	55.1	8.37
Q562R1	Beta-actin-like protein 2 OS=Homo sapiens GN=ACTBL2 PE=1 SV=2 - (ACTBL_HUMAN)	232.91	22.87	17	1	14	126	1.881E9	376	42.0	5.59
A0N4V7	HCG2039797 (Fragment) OS=Homo sapiens GN=Tcr-alpha PE=4 SV=1 - (A0N4V7_HUMAN)	228.08	38.10	1	1	1	339	1.337E10	21	2.2	9.74
P04406	Glyceraldehyde-3-phosphate dehydrogenase OS=Homo sapiens GN=GAPDH PE=1 SV=3 - (G3P_HUMAN)	221.74	59.40	11	15	20	112	5.097E9	335	36.0	8.46
Q9BYR8	Keratin-associated protein 3-1 OS=Homo sapiens GN=KRTAP3-1 PE=1 SV=1 - (KRA31_HUMAN)	213.44	27.55	1	2	3	68	6.919E8	98	10.5	6.40
Q96KK5	Histone H2A type 1-H OS=Homo sapiens GN=HIST1H2AH PE=1 SV=3 - (H2A1H_HUMAN)	212.84	51.56	22	1	8	77	1.036E9	128	13.9	10.89
Q8WZ42-2	Isoform 2 of Titin OS=Homo sapiens GN=TTN - (TITIN_HUMAN)	208.01	4.00	14	1	123	352	9.530E8	34258	3803.3	6.34
O43707	Alpha-actinin-4 OS=Homo sapiens GN=ACTN4 PE=1 SV=2 - (ACTN4_HUMAN)	206.38	21.51	19	5	18	71	1.185E8	911	104.8	5.44
Q93077	Histone H2A type 1-C OS=Homo sapiens GN=HIST1H2AC PE=1 SV=3 - (H2A1C_HUMAN)	202.19	46.92	12	1	7	73	1.036E9	130	14.1	11.05
H0YA55	Serum albumin (Fragment) OS=Homo sapiens GN=ALB PE=4 SV=1 - (H0YA55_HUMAN)	195.45	18.50	15	6	9	75	1.150E9	454	51.5	6.95
P63104	14-3-3 protein zeta/delta OS=Homo sapiens GN=YWHAZ PE=1 SV=1 - (1433Z_HUMAN)	191.75	70.20	20	9	18	95	7.783E8	245	27.7	4.79
O94868-3	Isoform 3 of FCH and double SH3 domains protein 2 OS=Homo sapiens GN=FCHSD2 - (FCSD2_HUMAN)	189.51	9.13	7	1	6	123	9.801E9	515	58.8	5.91
Q13835-2	Isoform 1 of Plakophilin-1 OS=Homo sapiens GN=PKP1 - (PKP1_HUMAN)	188.76	33.47	4	19	24	98	6.926E8	726	80.4	8.97
P27348	14-3-3 protein theta OS=Homo sapiens GN=YWHAQ PE=1 SV=1 - (1433T_HUMAN)	182.21	59.18	12	8	20	82	9.814E8	245	27.7	4.78
Q86UQ4	ATP-binding cassette sub-family A member 13 OS=Homo sapiens GN=ABCA13 PE=2 SV=3 - (ABCAD_HUMAN)	181.30	4.11	2	1	20	97	2.579E9	5058	575.8	6.46
O15078	Centrosomal protein of 290 kDa OS=Homo sapiens GN=CEP290 PE=1 SV=2 - (CE290_HUMAN)	180.03	11.94	2	1	29	173	6.987E8	2479	290.2	5.95
Q15149-4	Isoform 4 of Plectin OS=Homo sapiens GN=PLEC - (PLEC_HUMAN)	177.86	20.45	12	21	93	198	1.075E9	4547	515.9	5.80
Q13748	Tubulin alpha-3C/D chain OS=Homo sapiens GN=TUBA3C PE=1 SV=3 - (TBA3C_HUMAN)	175.03	47.56	14	2	21	71	2.453E8	450	49.9	5.10
P68104	Elongation factor 1-alpha 1 OS=Homo sapiens GN=EEF1A1 PE=1 SV=1 - (EF1A1_HUMAN)	174.61	29.00	38	10	15	78	2.164E8	462	50.1	9.01
C9J1U3	Protein unc-80 homolog OS=Homo sapiens GN=UNC80 PE=4 SV=1 - (C9J1U3_HUMAN)	174.41	6.34	4	1	18	95	2.208E9	3234	360.6	6.92
F5H288	Vimentin OS=Homo sapiens GN=VIM PE=3 SV=1 - (F5H288_HUMAN)	171.91	12.78	6	1	6	104	6.493E9	407	47.0	5.00
P62805	Histone H4 OS=Homo sapiens GN=HIST1H4A PE=1 SV=2 - (H4_HUMAN)	168.41	66.02	3	9	13	69	7.272E8	103	11.4	11.36
Q3LI55	Keratin associated protein OS=Homo sapiens GN=KRTAP11-1 PE=2 SV=1 - (Q3LI55_HUMAN)	165.57	26.99	2	4	5	124	1.615E8	163	17.0	7.78
D3DVC4	Nestin, isoform CRA_c OS=Homo sapiens GN=NES PE=3 SV=1 - (D3DVC4_HUMAN)	161.72	9.32	5	1	14	95	1.155E9	1621	177.3	4.36
P31946-2	Isoform Short of 14-3-3 protein beta/alpha OS=Homo sapiens GN=YWHAB - (1433B_HUMAN)	152.60	44.67	11	2	15	77	1.053E9	244	27.8	4.83
A8MTN3	Putative keratin-associated protein ENSP00000381495 OS=Homo sapiens PE=3 SV=2 - (KRA2X_HUMAN)	150.09	26.83	7	2	2	72	1.129E9	123	12.9	7.81
P61981	14-3-3 protein gamma OS=Homo sapiens GN=YWHAG PE=1 SV=2 - (1433G_HUMAN)	142.90	42.91	13	2	11	72	9.814E8	247	28.3	4.89
Q9P225-2	Isoform 2 of Dynein heavy chain 2, axonemal OS=Homo sapiens GN=DNAH2 - (DYH2_HUMAN)	142.54	6.53	3	1	27	99	3.794E8	4196	481.7	6.43
O75369-6	Isoform 6 of Filamin-B OS=Homo sapiens GN=FLNB - (FLNB_HUMAN)	139.57	11.35	15	17	26	62	2.562E8	2537	271.2	5.72
P81605	Dermcidin OS=Homo sapiens GN=DCD PE=1 SV=2 - (DCD_HUMAN)	138.50	47.27	2	6	7	55	1.341E8	110	11.3	6.54
Q8WVJ2	NudC domain-containing protein 2 OS=Homo sapiens GN=NUDCD2 PE=1 SV=1 - (NUDC2_HUMAN)	134.27	17.83	1	1	2	110	3.693E8	157	17.7	5.07
Q8NAM9	cDNA FLJ35099 fis, clone PLACE6006287 OS=Homo sapiens PE=2 SV=1 - (Q8NAM9_HUMAN)	130.32	2.68	1	1	1	105	6.852E8	224	24.0	11.75
P25054-2	Isoform Short of Adenomatous polyposis coli protein OS=Homo sapiens GN=APC - (APC_HUMAN)	127.34	8.21	5	1	17	92	3.519E9	2742	300.3	7.77
A2A3F7	Transient receptor potential cation channel, subfamily M, member 3 OS=Homo sapiens GN=TRPM3 PE=4 SV=1 - (A2A3F7_HUMAN)	125.21	3.74	19	1	5	78	6.670E9	1711	195.2	7.56
P35579	Myosin-9 OS=Homo sapiens GN=MYH9 PE=1 SV=4 - (MYH9_HUMAN)	124.28	20.61	9	7	39	78	1.867E9	1960	226.4	5.60
Q6PK75	PRSS2 protein (Fragment) OS=Homo sapiens GN=PRSS2 PE=2 SV=1 - (Q6PK75_HUMAN)	123.31	16.04	16	1	2	52	1.020E9	187	20.1	4.87
O75182-2	Isoform 2 of Paired amphipathic helix protein Sin3b OS=Homo sapiens GN=SIN3B - (SIN3B_HUMAN)	122.33	9.12	3	1	8	127	1.880E8	1130	129.3	6.98
Q01469	Fatty acid-binding protein, epidermal OS=Homo sapiens GN=FABP5 PE=1 SV=3 - (FABP5_HUMAN)	117.63	82.22	2	14	17	65	3.151E8	135	15.2	7.01
Q09666	Neuroblast differentiation-associated protein AHNAK OS=Homo sapiens GN=AHNAK PE=1 SV=2 - (AHNK_HUMAN)	117.40	23.55	5	18	41	92	5.429E8	5890	628.7	6.15
Q14980-2	Isoform 2 of Nuclear mitotic apparatus protein 1 OS=Homo sapiens GN=NUMA1 - (NUMA1_HUMAN)	114.79	9.09	9	1	19	73	5.782E8	2101	236.4	5.80
B4E380	Histone H3 OS=Homo sapiens PE=3 SV=1 - (B4E380_HUMAN)	111.30	63.72	11	2	15	80	1.194E9	113	12.9	11.93
Q2VIP3	PABP3 (Fragment) OS=Homo sapiens PE=2 SV=1 - (Q2VIP3_HUMAN)	109.20	18.41	5	1	9	92	2.982E9	630	69.9	9.67
Q13136-2	Isoform 2 of Liprin-alpha-1 OS=Homo sapiens GN=PPFIA1 - (LIPA1_HUMAN)	106.68	9.54	3	1	11	65	1.021E10	1185	133.9	6.25
Q6KB66-2	Isoform 2 of Keratin, type II cytoskeletal 80 OS=Homo sapiens GN=KRT80 - (K2C80_HUMAN)	101.85	10.43	5	1	5	55	2.183E9	422	47.2	5.30
P46939	Utrophin OS=Homo sapiens GN=UTRN PE=1 SV=2 - (UTRO_HUMAN)	101.66	6.41	3	1	20	101	5.603E8	3433	394.2	5.33
F8VZ45	60S ribosomal protein L6 (Fragment) OS=Homo sapiens GN=RPL6 PE=4 SV=1 - (F8VZ45_HUMAN)	101.22	22.01	13	2	4	79	3.336E8	159	17.8	10.77
B3KXE7	cDNA FLJ45304 fis, clone BRHIP3003984, highly similar to IkappaB kinase complex-associated protein OS=Homo sapiens PE=2 SV=1 - (B3KXE7_HUMAN)	100.70	3.36	1	1	3	40	1.484E8	983	111.4	6.27
Q14204	Cytoplasmic dynein 1 heavy chain 1 OS=Homo sapiens GN=DYNC1H1 PE=1 SV=5 - (DYHC1_HUMAN)	99.58	5.47	1	1	20	84	2.767E9	4646	532.1	6.40
Q6ISF6	KRTAP9-2 protein (Fragment) OS=Homo sapiens GN=KRTAP9-2 PE=2 SV=1 - (Q6ISF6_HUMAN)	98.06	8.09	1	1	1	22	4.489E7	173	18.1	7.69
Q6A163	Keratin, type I cytoskeletal 39 OS=Homo sapiens GN=KRT39 PE=1 SV=2 - (K1C39_HUMAN)	97.17	25.87	16	9	16	61	1.536E8	491	55.6	5.26
P62258	14-3-3 protein epsilon OS=Homo sapiens GN=YWHAE PE=1 SV=1 - (1433E_HUMAN)	96.19	40.78	11	3	11	49	7.084E8	255	29.2	4.74
P02545-3	Isoform ADelta10 of Prelamin-A/C OS=Homo sapiens GN=LMNA - (LMNA_HUMAN)	95.52	35.49	17	11	19	50	5.681E8	634	70.6	8.40
Q02413	Desmoglein-1 OS=Homo sapiens GN=DSG1 PE=1 SV=2 - (DSG1_HUMAN)	94.79	14.87	2	8	13	41	1.639E8	1049	113.7	5.03
Q15906	Vacuolar protein sorting-associated protein 72 homolog OS=Homo sapiens GN=VPS72 PE=1 SV=1 - (VPS72_HUMAN)	91.35	7.69	3	1	3	62	1.027E9	364	40.6	6.48
Q5TEC6	Histone H3 OS=Homo sapiens GN=HIST2H3PS2 PE=3 SV=1 - (Q5TEC6_HUMAN)	90.44	38.97	3	1	10	66	1.194E9	136	15.4	11.27
B3KTV0	cDNA FLJ38781 fis, clone LIVER2000216, highly similar to HEAT SHOCK COGNATE 71 kDa PROTEIN OS=Homo sapiens PE=2 SV=1 - (B3KTV0_HUMAN)	88.50	33.17	28	7	21	55	2.515E8	621	67.9	5.45
P60174	Triosephosphate isomerase OS=Homo sapiens GN=TPI1 PE=1 SV=3 - (TPIS_HUMAN)	88.11	64.34	6	12	15	39	8.489E7	286	30.8	5.92
P15311	Ezrin OS=Homo sapiens GN=EZR PE=1 SV=4 - (EZRI_HUMAN)	87.97	23.38	27	8	16	55	2.811E9	586	69.4	6.27
P54652	Heat shock-related 70 kDa protein 2 OS=Homo sapiens GN=HSPA2 PE=1 SV=1 - (HSP72_HUMAN)	86.86	35.99	4	5	21	52	3.105E8	639	70.0	5.74
Q6AWC2-4	Isoform 4 of Protein WWC2 OS=Homo sapiens GN=WWC2 - (WWC2_HUMAN)	86.21	6.39	6	1	8	56	1.537E9	1143	128.6	5.90
O95613-2	Isoform 2 of Pericentrin OS=Homo sapiens GN=PCNT - (PCNT_HUMAN)	85.13	8.98	3	1	26	82	7.902E8	3139	355.7	5.47
Q6P1J9	Parafibromin OS=Homo sapiens GN=CDC73 PE=1 SV=1 - (CDC73_HUMAN)	83.66	14.69	2	1	7	72	2.937E9	531	60.5	9.61
H0YG33	Heat shock 70 kDa protein 1A/1B OS=Homo sapiens GN=HSPA1B PE=3 SV=1 - (H0YG33_HUMAN)	82.67	27.40	23	9	21	52	2.698E8	708	77.4	6.99
Q5UGI3	Ubiquitin C splice variant OS=Homo sapiens GN=UBC PE=2 SV=1 - (Q5UGI3_HUMAN)	81.46	77.12	30	6	8	48	1.208E8	153	17.1	8.34
Q5HY54	Filamin-A OS=Homo sapiens GN=FLNA PE=2 SV=1 - (Q5HY54_HUMAN)	81.29	9.70	19	11	22	48	2.765E8	2607	276.4	6.05
Q86TY5	Full-length cDNA clone CS0DI041YE05 of Placenta of Homo sapiens (human) OS=Homo sapiens PE=2 SV=1 - (Q86TY5_HUMAN)	81.26	71.07	16	10	10	49	1.970E8	121	13.9	9.16
B4DF70	cDNA FLJ60461, highly similar to Peroxiredoxin-2 (EC 1.11.1.15) OS=Homo sapiens PE=2 SV=1 - (B4DF70_HUMAN)	80.26	41.53	4	6	8	35	1.640E8	183	20.1	8.78
H0YM23	Ankyrin repeat domain-containing protein 17 (Fragment) OS=Homo sapiens GN=ANKRD17 PE=4 SV=1 - (H0YM23_HUMAN)	80.18	5.83	14	2	15	62	4.903E9	2487	263.2	6.38
Q2Q9B7	Glucose-6-phosphate 1-dehydrogenase (Fragment) OS=Homo sapiens GN=G6PD PE=2 SV=1 - (Q2Q9B7_HUMAN)	79.12	3.37	12	1	2	55	1.497E9	475	54.8	7.08
F8W8Y7	Armadillo repeat-containing X-linked protein 4 OS=Homo sapiens GN=ARMCX4 PE=4 SV=1 - (F8W8Y7_HUMAN)	79.03	8.44	3	1	15	80	2.707E8	2394	247.7	5.95
P06733	Alpha-enolase OS=Homo sapiens GN=ENO1 PE=1 SV=2 - (ENOA_HUMAN)	78.41	47.93	25	10	15	30	1.698E8	434	47.1	7.39
Q8NEY8-5	Isoform 5 of Periphilin-1 OS=Homo sapiens GN=PPHLN1 - (PPHLN_HUMAN)	77.65	15.12	2	1	4	105	7.487E8	258	29.8	9.20
Q9BYR7	Keratin-associated protein 3-2 OS=Homo sapiens GN=KRTAP3-2 PE=1 SV=1 - (KRA32_HUMAN)	75.63	24.49	1	1	2	20	4.911E8	98	10.4	5.69
Q8WUT4	Leucine-rich repeat neuronal protein 4 OS=Homo sapiens GN=LRRN4 PE=1 SV=3 - (LRRN4_HUMAN)	73.47	6.62	2	1	4	43	3.762E8	740	78.8	7.20
B4E3S1	cDNA FLJ57040, highly similar to Myosin-9 OS=Homo sapiens PE=2 SV=1 - (B4E3S1_HUMAN)	73.30	18.57	5	1	16	40	1.827E9	964	110.8	8.25
P37802	Transgelin-2 OS=Homo sapiens GN=TAGLN2 PE=1 SV=3 - (TAGL2_HUMAN)	72.33	63.32	4	10	12	27	4.557E7	199	22.4	8.25
Q8NGB0	Seven transmembrane helix receptor OS=Homo sapiens GN=GPR142 PE=2 SV=1 - (Q8NGB0_HUMAN)	69.30	7.65	1	1	12	49	7.075E8	1464	156.4	10.95
P09382	Galectin-1 OS=Homo sapiens GN=LGALS1 PE=1 SV=2 - (LEG1_HUMAN)	69.12	58.52	2	7	9	31	1.956E8	135	14.7	5.50
A8K486	Peptidyl-prolyl cis-trans isomerase OS=Homo sapiens PE=2 SV=1 - (A8K486_HUMAN)	69.07	67.27	34	12	13	34	2.598E8	165	18.0	6.90
I0B0K8	Truncated profilaggrin OS=Homo sapiens GN=FLG PE=4 SV=1 - (I0B0K8_HUMAN)	68.82	17.01	15	5	33	89	1.977E9	4021	430.2	9.29
Q6DRA6	Putative histone H2B type 2-D OS=Homo sapiens GN=HIST2H2BD PE=5 SV=3 - (H2B2D_HUMAN)	68.67	29.88	20	3	7	39	1.669E8	164	18.0	10.58
Q3V6T2-4	Isoform 4 of Girdin OS=Homo sapiens GN=CCDC88A - (GRDN_HUMAN)	67.77	10.21	9	1	19	43	2.899E8	1842	212.4	5.97
H3BTN5	Pyruvate kinase (Fragment) OS=Homo sapiens GN=PKM PE=3 SV=1 - (H3BTN5_HUMAN)	67.19	25.36	18	9	10	21	1.693E8	485	53.0	6.84
Q15075	Early endosome antigen 1 OS=Homo sapiens GN=EEA1 PE=1 SV=2 - (EEA1_HUMAN)	65.51	16.23	2	1	21	56	6.595E8	1411	162.4	5.68
B4DHC2	cDNA FLJ56434, highly similar to p130Cas-associated protein OS=Homo sapiens PE=2 SV=1 - (B4DHC2_HUMAN)	64.85	9.86	1	1	9	56	6.197E8	1035	111.0	9.41
O94819	Kelch repeat and BTB domain-containing protein 11 OS=Homo sapiens GN=KBTBD11 PE=1 SV=1 - (KBTBB_HUMAN)	64.85	5.30	1	1	3	34	2.112E8	623	65.7	6.07
Q06830	Peroxiredoxin-1 OS=Homo sapiens GN=PRDX1 PE=1 SV=1 - (PRDX1_HUMAN)	62.28	46.73	4	10	11	30	1.054E8	199	22.1	8.13
B3KTL8	cDNA FLJ38476 fis, clone FEBRA2022504, highly similar to YTH domain protein 1 OS=Homo sapiens PE=2 SV=1 - (B3KTL8_HUMAN)	61.79	9.30	4	1	4	34	6.670E8	559	60.8	8.90
B2RAW1	cDNA, FLJ95155, highly similar to Homo sapiens Bruton agammaglobulinemia tyrosine kinase (BTK), mRNA OS=Homo sapiens PE=2 SV=1 - (B2RAW1_HUMAN)	60.79	2.88	14	1	2	46	4.455E8	659	76.2	7.77
Q96AQ8	Coiled-coil domain-containing protein 90A, mitochondrial OS=Homo sapiens GN=CCDC90A PE=2 SV=1 - (CC90A_HUMAN)	60.42	13.09	2	1	4	50	3.198E8	359	39.7	9.63
A1L390	Pleckstrin homology domain-containing family G member 3 OS=Homo sapiens GN=PLEKHG3 PE=1 SV=1 - (PKHG3_HUMAN)	60.39	4.92	3	1	6	36	4.751E8	1219	134.3	6.55
B0AZQ4	cDNA, FLJ79494, highly similar to Structural maintenance of chromosome 3 OS=Homo sapiens PE=2 SV=1 - (B0AZQ4_HUMAN)	60.29	10.85	3	1	12	51	6.407E8	1217	141.4	7.18
Q6P0Q8	Microtubule-associated serine/threonine-protein kinase 2 OS=Homo sapiens GN=MAST2 PE=1 SV=2 - (MAST2_HUMAN)	60.11	4.95	2	1	9	58	7.186E8	1798	196.3	8.16
Q562Q2	Actin-like protein (Fragment) OS=Homo sapiens GN=ACT PE=3 SV=1 - (Q562Q2_HUMAN)	59.62	33.98	10	1	3	36	6.726E8	103	11.5	6.68
P13639	Elongation factor 2 OS=Homo sapiens GN=EEF2 PE=1 SV=4 - (EF2_HUMAN)	59.45	13.52	15	8	11	27	1.943E8	858	95.3	6.83
Q7Z406	Myosin-14 OS=Homo sapiens GN=MYH14 PE=1 SV=2 - (MYH14_HUMAN)	58.38	11.63	80	1	23	61	2.318E8	1995	227.7	5.60
Q9HCD5	Nuclear receptor coactivator 5 OS=Homo sapiens GN=NCOA5 PE=1 SV=2 - (NCOA5_HUMAN)	57.38	18.13	1	1	11	65	5.010E8	579	65.5	9.60
P27482	Calmodulin-like protein 3 OS=Homo sapiens GN=CALML3 PE=1 SV=2 - (CALL3_HUMAN)	56.98	53.69	1	5	7	24	1.757E8	149	16.9	4.42
B3KSC3	cDNA FLJ35987 fis, clone TESTI2014269, highly similar to D-3-phosphoglycerate dehydrogenase (EC 1.1.1.95) OS=Homo sapiens PE=2 SV=1 - (B3KSC3_HUMAN)	56.92	14.23	11	4	6	24	5.581E7	499	53.0	7.12
B4DJI1	L-lactate dehydrogenase OS=Homo sapiens PE=2 SV=1 - (B4DJI1_HUMAN)	56.62	38.03	22	5	12	39	9.291E7	305	33.6	8.46
Q9BYR6	Keratin-associated protein 3-3 OS=Homo sapiens GN=KRTAP3-3 PE=1 SV=1 - (KRA33_HUMAN)	55.54	24.49	1	1	2	15	2.864E8	98	10.4	5.69
H0Y4W4	NF-kappa-B inhibitor epsilon (Fragment) OS=Homo sapiens GN=NFKBIE PE=4 SV=1 - (H0Y4W4_HUMAN)	55.47	14.55	6	1	1	29	1.759E7	55	5.9	4.42
F8WA45	Gelsolin OS=Homo sapiens GN=GSN PE=4 SV=1 - (F8WA45_HUMAN)	54.30	16.22	19	6	12	33	5.445E7	715	78.9	6.01
Q8IUE6	Histone H2A type 2-B OS=Homo sapiens GN=HIST2H2AB PE=1 SV=3 - (H2A2B_HUMAN)	53.38	41.54	3	2	5	22	5.443E8	130	14.0	10.89
B7ZLH8	EVPL protein OS=Homo sapiens GN=EVPL PE=2 SV=1 - (B7ZLH8_HUMAN)	52.59	12.12	2	1	28	54	1.295E9	2055	233.7	7.25
Q9NRC6	Spectrin beta chain, brain 4 OS=Homo sapiens GN=SPTBN5 PE=1 SV=1 - (SPTN5_HUMAN)	52.51	9.31	1	1	30	52	3.758E8	3674	416.6	6.67
Q0Z7S8	Fatty acid-binding protein 9 OS=Homo sapiens GN=FABP9 PE=1 SV=1 - (FABP9_HUMAN)	52.20	71.21	1	9	9	23	9.558E7	132	15.1	8.09
Q12955	Ankyrin-3 OS=Homo sapiens GN=ANK3 PE=1 SV=3 - (ANK3_HUMAN)	52.06	4.82	1	1	23	59	5.684E8	4377	480.1	6.49
Q5T655	Coiled-coil domain-containing protein 147 OS=Homo sapiens GN=CCDC147 PE=2 SV=1 - (CC147_HUMAN)	51.47	5.96	2	2	6	36	5.636E8	872	103.4	8.41
P05109	Protein S100-A8 OS=Homo sapiens GN=S100A8 PE=1 SV=1 - (S10A8_HUMAN)	50.98	60.22	1	9	10	32	2.920E8	93	10.8	7.03
A2RRR7	CCDC46 protein OS=Homo sapiens GN=CCDC46 PE=2 SV=1 - (A2RRR7_HUMAN)	50.68	8.87	6	2	9	45	2.749E8	913	107.6	6.34
Q5SQ08	Complement component 8, gamma polypeptide (Fragment) OS=Homo sapiens GN=C8G PE=2 SV=1 - (Q5SQ08_HUMAN)	50.16	4.88	2	1	1	25	1.343E7	164	17.5	9.79
Q9NS87	Kinesin-like protein KIF15 OS=Homo sapiens GN=KIF15 PE=1 SV=1 - (KIF15_HUMAN)	50.07	8.00	8	2	10	30	6.111E8	1388	160.1	6.00
O94822	E3 ubiquitin-protein ligase listerin OS=Homo sapiens GN=LTN1 PE=1 SV=6 - (LTN1_HUMAN)	49.94	3.34	1	1	6	46	1.899E8	1766	200.4	6.25
A0PK11	Clarin-2 OS=Homo sapiens GN=CLRN2 PE=2 SV=1 - (CLRN2_HUMAN)	49.70	3.02	1	1	1	41	1.319E9	232	25.4	6.99
Q96M86	Dynein heavy chain domain-containing protein 1 OS=Homo sapiens GN=DNHD1 PE=1 SV=2 - (DNHD1_HUMAN)	49.10	3.66	3	1	16	39	1.588E8	4753	533.3	6.71
P04083	Annexin A1 OS=Homo sapiens GN=ANXA1 PE=1 SV=2 - (ANXA1_HUMAN)	48.40	34.10	5	10	13	26	2.587E8	346	38.7	7.02
F8WAQ9	Microtubule-associated serine/threonine-protein kinase 4 OS=Homo sapiens GN=MAST4 PE=4 SV=1 - (F8WAQ9_HUMAN)	48.27	7.50	10	1	17	43	4.478E8	2626	284.2	8.62
P31151	Protein S100-A7 OS=Homo sapiens GN=S100A7 PE=1 SV=4 - (S10A7_HUMAN)	48.01	48.51	2	7	7	17	3.310E7	101	11.5	6.77
P09211	Glutathione S-transferase P OS=Homo sapiens GN=GSTP1 PE=1 SV=2 - (GSTP1_HUMAN)	47.95	52.86	4	6	8	26	1.204E8	210	23.3	5.64
B3KX72	Heterogeneous nuclear ribonucleoprotein U OS=Homo sapiens GN=HNRNPU PE=2 SV=1 - (B3KX72_HUMAN)	47.91	22.00	8	3	17	45	5.770E8	750	83.0	8.79
Q5TZA2	Rootletin OS=Homo sapiens GN=CROCC PE=1 SV=1 - (CROCC_HUMAN)	47.83	16.71	5	1	38	61	3.603E8	2017	228.4	5.50
Q96LA8-2	Isoform 2 of Protein arginine N-methyltransferase 6 OS=Homo sapiens GN=PRMT6 - (ANM6_HUMAN)	47.67	4.79	2	1	1	20	7.479E8	292	32.5	5.00
P50607-2	Isoform 2 of Tubby protein homolog OS=Homo sapiens GN=TUB - (TUB_HUMAN)	46.69	7.13	6	1	6	24	3.135E7	561	62.1	9.54
O15173	Membrane-associated progesterone receptor component 2 OS=Homo sapiens GN=PGRMC2 PE=1 SV=1 - (PGRC2_HUMAN)	46.43	4.04	1	1	2	32	1.510E9	223	23.8	4.88
Q7RTZ2	Putative ubiquitin carboxyl-terminal hydrolase 17-like protein 1 OS=Homo sapiens GN=USP17L1P PE=5 SV=1 - (U17L1_HUMAN)	46.40	5.85	16	1	4	48	7.296E8	530	59.6	7.72
Q9Y6R9	Coiled-coil domain-containing protein 61 OS=Homo sapiens GN=CCDC61 PE=1 SV=2 - (CCD61_HUMAN)	46.15	14.98	1	1	7	30	2.761E8	474	53.2	9.95
Q0QEN7	ATP synthase subunit beta (Fragment) OS=Homo sapiens GN=ATP5B PE=2 SV=1 - (Q0QEN7_HUMAN)	43.24	29.89	8	9	10	14	5.386E7	445	48.1	5.07
Q86Y33-5	Isoform 5 of Cell division cycle protein 20 homolog B OS=Homo sapiens GN=CDC20B - (CD20B_HUMAN)	42.42	9.38	5	1	1	46	1.115E9	192	21.7	8.15
P07737	Profilin-1 OS=Homo sapiens GN=PFN1 PE=1 SV=2 - (PROF1_HUMAN)	42.28	63.57	2	8	9	19	4.607E7	140	15.0	8.27
Q14134-2	Isoform Beta of Tripartite motif-containing protein 29 OS=Homo sapiens GN=TRIM29 - (TRI29_HUMAN)	42.15	26.67	18	13	15	26	7.126E7	570	63.8	6.98
P36952	Serpin B5 OS=Homo sapiens GN=SERPINB5 PE=1 SV=2 - (SPB5_HUMAN)	41.98	28.27	3	2	9	18	7.188E7	375	42.1	6.05
B9EK46	Cingulin OS=Homo sapiens GN=CGN PE=2 SV=1 - (B9EK46_HUMAN)	41.98	11.64	3	1	16	44	5.376E8	1203	137.0	5.52
Q5HYW3	Retrotransposon gag domain-containing protein 4 OS=Homo sapiens GN=RGAG4 PE=2 SV=1 - (RGAG4_HUMAN)	41.95	8.61	1	1	5	24	2.398E8	569	64.7	4.75
Q6ZQQ6	WD repeat-containing protein 87 OS=Homo sapiens GN=WDR87 PE=2 SV=3 - (WDR87_HUMAN)	41.65	7.14	3	1	22	45	7.208E8	2873	333.0	7.28
P98164	Low-density lipoprotein receptor-related protein 2 OS=Homo sapiens GN=LRP2 PE=1 SV=3 - (LRP2_HUMAN)	41.30	3.67	4	1	14	40	1.023E9	4655	521.6	5.08
O14863	Zinc transporter 4 OS=Homo sapiens GN=SLC30A4 PE=2 SV=2 - (ZNT4_HUMAN)	41.08	4.43	1	1	2	40	1.411E9	429	47.5	6.58
Q15853-2	Isoform USF2A-delta-H of Upstream stimulatory factor 2 OS=Homo sapiens GN=USF2 - (USF2_HUMAN)	40.66	5.33	1	1	2	38	4.340E8	338	36.2	4.97
B2R4M6	cDNA, FLJ92148, highly similar to Homo sapiens S100 calcium binding protein A9 (calgranulin B) (S100A9), mRNA OS=Homo sapiens PE=4 SV=1 - (B2R4M6_HUMAN)	39.84	48.25	2	7	8	17	1.198E8	114	13.2	6.13
E9PC74	Translation initiation factor eIF-2B subunit epsilon OS=Homo sapiens GN=EIF2B5 PE=4 SV=1 - (E9PC74_HUMAN)	39.68	6.10	4	1	3	25	1.272E8	705	78.3	5.21
Q53G25	Ribosomal protein S5 variant (Fragment) OS=Homo sapiens PE=2 SV=1 - (Q53G25_HUMAN)	39.61	25.49	2	1	6	14	3.478E7	204	23.0	9.74
B4E3Q9	cDNA FLJ59659, highly similar to Vinculin OS=Homo sapiens PE=2 SV=1 - (B4E3Q9_HUMAN)	38.93	9.49	7	1	10	29	1.226E8	927	100.9	5.48
B7Z7A9	Phosphoglycerate kinase OS=Homo sapiens GN=PGK1 PE=2 SV=1 - (B7Z7A9_HUMAN)	37.83	32.13	8	5	9	18	1.492E8	389	41.4	7.33
Q32MZ4-2	Isoform 2 of Leucine-rich repeat flightless-interacting protein 1 OS=Homo sapiens GN=LRRFIP1 - (LRRF1_HUMAN)	37.82	12.63	8	1	9	39	9.063E8	784	86.4	4.65
Q7L7X3	Serine/threonine-protein kinase TAO1 OS=Homo sapiens GN=TAOK1 PE=1 SV=1 - (TAOK1_HUMAN)	37.81	13.59	2	1	11	40	6.673E7	1001	116.0	7.65
P31949	Protein S100-A11 OS=Homo sapiens GN=S100A11 PE=1 SV=2 - (S10AB_HUMAN)	37.47	56.19	2	7	7	13	4.837E7	105	11.7	7.12
G3V1A4	Cofilin 1 (Non-muscle), isoform CRA_a OS=Homo sapiens GN=CFL1 PE=4 SV=1 - (G3V1A4_HUMAN)	37.31	48.32	10	5	5	12	1.656E8	149	16.8	8.35
B3KVD6	cDNA FLJ16433 fis, clone BRACE3013936, highly similar to Cell death activator CIDE-B OS=Homo sapiens PE=2 SV=1 - (B3KVD6_HUMAN)	37.04	3.20	2	1	1	53	1.571E9	219	24.8	9.03
B2R6V2	cDNA, FLJ93132, highly similar to Homo sapiens CDC7 cell division cycle 7 (S. cerevisiae) (CDC7), mRNA OS=Homo sapiens PE=2 SV=1 - (B2R6V2_HUMAN)	36.64	8.01	4	1	6	34	2.690E8	574	63.8	8.81
Q6P9G8	Family with sequence similarity 184, member A OS=Homo sapiens GN=FAM184A PE=2 SV=2 - (Q6P9G8_HUMAN)	36.50	15.00	12	1	18	53	5.054E8	1107	129.4	5.72
P33764	Protein S100-A3 OS=Homo sapiens GN=S100A3 PE=1 SV=1 - (S10A3_HUMAN)	35.87	62.38	1	7	7	14	1.179E8	101	11.7	4.78
B2RUU3	Dedicator of cytokinesis 1 OS=Homo sapiens GN=DOCK1 PE=2 SV=1 - (B2RUU3_HUMAN)	35.36	5.68	2	1	12	28	4.342E9	1865	215.2	7.49
Q5T321	Neurobeachin OS=Homo sapiens GN=NBEA PE=2 SV=1 - (Q5T321_HUMAN)	35.23	3.23	10	1	9	36	1.523E8	2943	327.3	6.16
Q99996-6	Isoform 6 of A-kinase anchor protein 9 OS=Homo sapiens GN=AKAP9 - (AKAP9_HUMAN)	34.92	5.88	10	1	21	34	2.529E8	3929	455.1	4.98
Q4L180-2	Isoform 2 of Filamin A-interacting protein 1-like OS=Homo sapiens GN=FILIP1L - (FIL1L_HUMAN)	34.36	14.12	7	1	13	28	7.393E8	1133	130.1	6.61
P55072	Transitional endoplasmic reticulum ATPase OS=Homo sapiens GN=VCP PE=1 SV=4 - (TERA_HUMAN)	34.31	19.48	9	9	13	17	1.715E7	806	89.3	5.26
Q96IR1	RPS4X protein (Fragment) OS=Homo sapiens GN=RPS4X PE=2 SV=2 - (Q96IR1_HUMAN)	34.08	39.51	12	9	11	16	6.056E7	243	27.2	9.94
Q5T011-5	Isoform 3 of Protein SZT2 OS=Homo sapiens GN=SZT2 - (SZT2_HUMAN)	34.01	3.70	2	1	16	53	1.438E8	3375	371.6	6.33
Q5CZC0	Fibrous sheath-interacting protein 2 OS=Homo sapiens GN=FSIP2 PE=1 SV=4 - (FSIP2_HUMAN)	33.66	5.70	4	1	34	60	2.556E9	6907	780.1	6.71
Q9BYR4	Keratin-associated protein 4-3 OS=Homo sapiens GN=KRTAP4-3 PE=2 SV=2 - (KRA43_HUMAN)	33.00	12.82	1	2	2	11	3.156E7	195	20.5	7.94
Q96CS3	FAS-associated factor 2 OS=Homo sapiens GN=FAF2 PE=1 SV=2 - (FAF2_HUMAN)	32.87	2.25	1	1	1	14	1.331E9	445	52.6	5.62
Q02388	Collagen alpha-1(VII) chain OS=Homo sapiens GN=COL7A1 PE=1 SV=2 - (CO7A1_HUMAN)	32.56	7.34	2	1	18	53	3.392E9	2944	295.0	6.27
B4DVQ3	Calcium/calmodulin-dependent protein kinase type II subunit gamma OS=Homo sapiens GN=CAMK2G PE=2 SV=1 - (B4DVQ3_HUMAN)	32.49	9.44	1	1	1	22	1.588E8	180	20.5	8.75
D3YTG3	Target of Nesh-SH3 OS=Homo sapiens GN=ABI3BP PE=4 SV=1 - (D3YTG3_HUMAN)	32.27	3.38	8	1	5	22	6.338E8	1777	195.2	9.73
Q86SJ6	Desmoglein-4 OS=Homo sapiens GN=DSG4 PE=1 SV=1 - (DSG4_HUMAN)	32.21	9.90	2	4	9	25	1.972E8	1040	113.8	4.56
B4DWS9	cDNA FLJ57640, highly similar to Serpin B5 OS=Homo sapiens PE=2 SV=1 - (B4DWS9_HUMAN)	31.86	26.48	1	1	7	15	7.188E7	287	32.4	6.54
H0YJZ4	Striatin-3 (Fragment) OS=Homo sapiens GN=STRN3 PE=4 SV=1 - (H0YJZ4_HUMAN)	31.72	14.39	1	1	2	60	7.827E8	139	14.6	4.07
Q92788	Rad GTPase OS=Homo sapiens PE=2 SV=1 - (Q92788_HUMAN)	31.10	14.94	3	1	5	66	5.752E7	308	33.2	8.68
B4DE44	cDNA FLJ52880, highly similar to Malate dehydrogenase, mitochondrial (EC 1.1.1.37) OS=Homo sapiens PE=2 SV=1 - (B4DE44_HUMAN)	30.96	32.77	8	6	7	13	5.010E7	296	30.9	8.43
Q8IWY4	Signal peptide, CUB and EGF-like domain-containing protein 1 OS=Homo sapiens GN=SCUBE1 PE=1 SV=3 - (SCUB1_HUMAN)	30.70	3.64	3	1	3	19	1.628E8	988	107.8	6.83
P11021	78 kDa glucose-regulated protein OS=Homo sapiens GN=HSPA5 PE=1 SV=2 - (GRP78_HUMAN)	30.63	10.40	3	2	7	14	1.060E8	654	72.3	5.16
F8W7K3	Spectrin alpha chain, brain OS=Homo sapiens GN=SPTAN1 PE=4 SV=1 - (F8W7K3_HUMAN)	29.75	7.83	6	1	15	47	1.469E8	2452	282.0	5.34
P30050	60S ribosomal protein L12 OS=Homo sapiens GN=RPL12 PE=1 SV=1 - (RL12_HUMAN)	29.74	60.61	8	5	9	13	6.050E8	165	17.8	9.42
Q5CAQ5	Tumor rejection antigen (Gp96) 1 OS=Homo sapiens GN=TRA1 PE=2 SV=1 - (Q5CAQ5_HUMAN)	29.58	13.72	13	3	10	16	5.660E9	802	92.3	4.86
A6H8W8	Intersectin 2 OS=Homo sapiens GN=ITSN2 PE=2 SV=1 - (A6H8W8_HUMAN)	29.48	8.93	10	2	14	27	1.459E8	1669	190.2	8.18
P11055	Myosin-3 OS=Homo sapiens GN=MYH3 PE=1 SV=3 - (MYH3_HUMAN)	29.36	6.34	2	1	12	38	4.411E8	1940	223.8	5.81
H0YIV1	Nuclear receptor subfamily 2 group C member 1 (Fragment) OS=Homo sapiens GN=NR2C1 PE=4 SV=1 - (H0YIV1_HUMAN)	28.98	10.96	1	1	1	38	2.504E7	73	8.2	9.72
Q5SQQ9-2	Isoform 2 of Ventral anterior homeobox 1 OS=Homo sapiens GN=VAX1 - (VAX1_HUMAN)	28.79	15.05	3	1	3	37	2.278E8	186	21.0	9.60
Q86Y02	FAM167A protein OS=Homo sapiens GN=FAM167A PE=2 SV=1 - (Q86Y02_HUMAN)	28.79	11.76	1	1	1	15	5.456E8	85	10.0	8.73
I3L2X7	5-azacytidine-induced protein 1 (Fragment) OS=Homo sapiens GN=AZI1 PE=4 SV=1 - (I3L2X7_HUMAN)	28.72	14.69	4	1	8	44	2.752E8	524	62.0	7.15
P04075	Fructose-bisphosphate aldolase A OS=Homo sapiens GN=ALDOA PE=1 SV=2 - (ALDOA_HUMAN)	28.51	25.82	16	6	11	21	1.112E8	364	39.4	8.09
Q9H9L3	Interferon-stimulated 20 kDa exonuclease-like 2 OS=Homo sapiens GN=ISG20L2 PE=1 SV=1 - (I20L2_HUMAN)	28.35	11.05	1	1	4	42	2.790E8	353	39.1	9.94
Q6DT37	Serine/threonine-protein kinase MRCK gamma OS=Homo sapiens GN=CDC42BPG PE=1 SV=2 - (MRCKG_HUMAN)	28.23	8.12	1	1	11	40	3.677E8	1551	172.4	6.28
Q96HA7	Tonsoku-like protein OS=Homo sapiens GN=TONSL PE=1 SV=2 - (TONSL_HUMAN)	28.22	8.35	2	1	12	25	1.579E9	1378	150.8	6.42
F8VVB8	Meiosis arrest female protein 1 OS=Homo sapiens GN=KIAA0430 PE=4 SV=1 - (F8VVB8_HUMAN)	27.81	8.39	9	1	7	25	1.221E8	1037	112.9	8.38
A8TX70-2	Isoform 2 of Collagen alpha-5(VI) chain OS=Homo sapiens GN=COL6A5 - (CO6A5_HUMAN)	27.72	8.04	4	1	16	36	5.590E8	2526	279.8	7.36
H0Y5Q9	Phosphatidylinositol 3,4,5-trisphosphate 5-phosphatase 1 (Fragment) OS=Homo sapiens GN=INPP5D PE=4 SV=1 - (H0Y5Q9_HUMAN)	27.60	6.79	8	1	5	44	1.529E8	810	90.5	8.19
Q5T6W2	Heterogeneous nuclear ribonucleoprotein K (Fragment) OS=Homo sapiens GN=HNRNPK PE=2 SV=1 - (Q5T6W2_HUMAN)	27.35	36.68	10	5	10	22	5.565E7	379	41.8	5.59
B4DMA2	cDNA FLJ54023, highly similar to Heat shock protein HSP 90-beta OS=Homo sapiens PE=2 SV=1 - (B4DMA2_HUMAN)	27.26	24.49	39	3	20	26	8.871E7	686	79.1	5.02
Q9Y446	Plakophilin-3 OS=Homo sapiens GN=PKP3 PE=1 SV=1 - (PKP3_HUMAN)	27.24	19.57	8	7	12	18	7.117E7	797	87.0	9.32
P62269	40S ribosomal protein S18 OS=Homo sapiens GN=RPS18 PE=1 SV=3 - (RS18_HUMAN)	27.21	51.32	2	7	10	16	6.456E7	152	17.7	10.99
H7C469	Uncharacterized protein (Fragment) OS=Homo sapiens PE=3 SV=1 - (H7C469_HUMAN)	27.06	19.53	6	4	6	16	1.571E8	379	40.4	5.76
P62277	40S ribosomal protein S13 OS=Homo sapiens GN=RPS13 PE=1 SV=2 - (RS13_HUMAN)	26.95	42.38	2	6	8	13	3.312E7	151	17.2	10.54
Q9Y6V0	Protein piccolo OS=Homo sapiens GN=PCLO PE=1 SV=4 - (PCLO_HUMAN)	26.70	3.00	9	1	14	30	6.150E8	5065	552.9	6.51
P07476	Involucrin OS=Homo sapiens GN=IVL PE=1 SV=2 - (INVO_HUMAN)	26.61	22.56	3	4	11	29	4.097E7	585	68.4	4.61
O75185	Calcium-transporting ATPase type 2C member 2 OS=Homo sapiens GN=ATP2C2 PE=1 SV=2 - (AT2C2_HUMAN)	26.39	6.87	7	1	6	21	2.302E8	946	103.1	5.66
Q8NI35	InaD-like protein OS=Homo sapiens GN=INADL PE=1 SV=3 - (INADL_HUMAN)	26.37	4.39	8	1	7	23	1.309E8	1801	196.2	4.94
E5RGT7	Uncharacterized protein OS=Homo sapiens PE=4 SV=1 - (E5RGT7_HUMAN)	26.36	39.13	2	1	3	19	2.912E7	69	7.5	9.86
P39019	40S ribosomal protein S19 OS=Homo sapiens GN=RPS19 PE=1 SV=2 - (RS19_HUMAN)	26.32	52.41	2	8	11	24	4.422E7	145	16.1	10.32
P58107	Epiplakin OS=Homo sapiens GN=EPPK1 PE=1 SV=2 - (EPIPL_HUMAN)	26.31	12.06	3	1	16	30	2.546E8	5090	555.3	5.60
O60437	Periplakin OS=Homo sapiens GN=PPL PE=1 SV=4 - (PEPL_HUMAN)	26.26	10.88	1	4	19	24	2.010E8	1756	204.6	5.60
Q5K634	SCCA2/SCCA1 fusion protein isoform 1 OS=Homo sapiens PE=2 SV=1 - (Q5K634_HUMAN)	26.25	19.23	16	5	6	19	4.119E7	390	44.6	6.39
Q15126	Phosphomevalonate kinase OS=Homo sapiens GN=PMVK PE=1 SV=3 - (PMVK_HUMAN)	26.17	21.88	1	2	4	21	1.866E8	192	22.0	5.73
A4UGR9-2	Isoform 2 of Xin actin-binding repeat-containing protein 2 OS=Homo sapiens GN=XIRP2 - (XIRP2_HUMAN)	26.14	7.42	3	1	20	33	1.203E8	3327	377.1	6.42
I3L0X1	Fatty aldehyde dehydrogenase OS=Homo sapiens GN=ALDH3A2 PE=4 SV=1 - (I3L0X1_HUMAN)	26.14	10.44	4	1	2	33	1.708E9	182	20.7	5.03
E5KNB2	DNA-apurinic or apyrimidinic site lyase 2 OS=Homo sapiens PE=4 SV=1 - (E5KNB2_HUMAN)	26.12	3.28	3	1	2	16	5.892E8	518	57.4	8.29
H3BLZ6	Sterol regulatory element-binding protein 1 (Fragment) OS=Homo sapiens GN=SREBF1 PE=4 SV=1 - (H3BLZ6_HUMAN)	26.08	4.85	13	1	8	50	4.505E9	1155	122.3	8.88
P25705	ATP synthase subunit alpha, mitochondrial OS=Homo sapiens GN=ATP5A1 PE=1 SV=1 - (ATPA_HUMAN)	26.03	23.15	4	7	11	16	1.047E8	553	59.7	9.13
P00918	Carbonic anhydrase 2 OS=Homo sapiens GN=CA2 PE=1 SV=2 - (CAH2_HUMAN)	25.73	20.77	3	4	4	7	3.155E7	260	29.2	7.40
Q68D86	Coiled-coil domain-containing protein 102B OS=Homo sapiens GN=CCDC102B PE=2 SV=4 - (C102B_HUMAN)	25.63	10.72	3	1	7	31	5.290E8	513	60.4	5.95
A8K2G5	Rap guanine nucleotide exchange factor (GEF) 3, isoform CRA_a OS=Homo sapiens GN=RAPGEF3 PE=2 SV=1 - (A8K2G5_HUMAN)	25.17	5.22	8	1	5	32	2.567E8	881	99.3	7.74
E7EVA0	Microtubule-associated protein OS=Homo sapiens GN=MAP4 PE=4 SV=1 - (E7EVA0_HUMAN)	25.17	9.75	13	1	17	28	1.078E8	2297	245.3	6.23
E9PBV3	Suprabasin OS=Homo sapiens GN=SBSN PE=4 SV=1 - (E9PBV3_HUMAN)	25.12	23.73	1	1	5	12	1.373E7	590	60.5	7.01
Q15828	Cystatin-M OS=Homo sapiens GN=CST6 PE=1 SV=1 - (CYTM_HUMAN)	25.06	30.87	6	4	5	18	7.889E7	149	16.5	8.09
E9PR44	Alpha-crystallin B chain (Fragment) OS=Homo sapiens GN=CRYAB PE=3 SV=1 - (E9PR44_HUMAN)	25.01	55.75	10	8	8	14	3.141E7	174	20.0	7.03
B7Z6Z4	Myosin light polypeptide 6 OS=Homo sapiens GN=MYL6 PE=2 SV=1 - (B7Z6Z4_HUMAN)	24.84	24.37	20	3	5	11	5.325E7	238	26.7	5.08
B7ZKX5	KIF6 protein OS=Homo sapiens GN=KIF6 PE=2 SV=1 - (B7ZKX5_HUMAN)	24.83	12.92	8	1	8	24	1.559E8	797	90.5	6.95
P28838-2	Isoform 2 of Cytosol aminopeptidase OS=Homo sapiens GN=LAP3 - (AMPL_HUMAN)	24.71	19.47	4	3	8	15	2.811E7	488	52.7	6.74
Q8WUF5	RelA-associated inhibitor OS=Homo sapiens GN=PPP1R13L PE=1 SV=4 - (IASPP_HUMAN)	24.62	7.25	4	1	8	19	2.876E8	828	89.0	6.81
A8K4Z4	cDNA FLJ75549, highly similar to Homo sapiens ribosomal protein, large, P0 (RPLP0), transcript variant 1, mRNA OS=Homo sapiens PE=2 SV=1 - (A8K4Z4_HUMAN)	24.51	29.65	20	7	9	10	5.019E7	317	34.2	5.97
B4DK00	cDNA FLJ58506 OS=Homo sapiens PE=2 SV=1 - (B4DK00_HUMAN)	24.34	3.72	4	1	4	14	6.505E7	1236	137.1	8.73
Q9NVU3	cDNA FLJ10506 fis, clone NT2RP2000510 OS=Homo sapiens PE=2 SV=1 - (Q9NVU3_HUMAN)	24.28	9.14	5	1	9	27	8.360E7	864	96.3	6.65
Q1MSJ5-1	Isoform 1 of Centrosome and spindle pole-associated protein 1 OS=Homo sapiens GN=CSPP1 - (CSPP1_HUMAN)	24.27	11.47	3	1	12	20	2.270E8	1221	141.7	6.61
Q6UXS9-3	Isoform 3 of Inactive caspase-12 OS=Homo sapiens GN=CASP12 - (CASPC_HUMAN)	23.98	17.90	3	1	3	13	6.685E8	257	29.4	6.21
O00186	Syntaxin-binding protein 3 OS=Homo sapiens GN=STXBP3 PE=1 SV=2 - (STXB3_HUMAN)	23.91	3.38	1	1	2	24	9.539E7	592	67.7	7.80
Q08188	Protein-glutamine gamma-glutamyltransferase E OS=Homo sapiens GN=TGM3 PE=1 SV=4 - (TGM3_HUMAN)	23.83	15.73	3	4	11	27	4.254E7	693	76.6	5.86
B3KWI4	cDNA FLJ43122 fis, clone CTONG3003737, highly similar to Leucine-rich repeat-containing protein 15 OS=Homo sapiens PE=2 SV=1 - (B3KWI4_HUMAN)	23.73	8.43	3	2	4	13	6.031E7	581	64.4	6.71
Q86SG7	Lysozyme g-like protein 2 OS=Homo sapiens GN=LYG2 PE=1 SV=2 - (LYG2_HUMAN)	22.59	31.13	4	5	5	9	1.521E7	212	23.5	8.87
P14314-2	Isoform 2 of Glucosidase 2 subunit beta OS=Homo sapiens GN=PRKCSH - (GLU2B_HUMAN)	22.55	5.52	3	2	4	15	9.447E7	525	59.1	4.42
Q0VF96	Cingulin-like protein 1 OS=Homo sapiens GN=CGNL1 PE=1 SV=2 - (CGNL1_HUMAN)	22.43	12.37	2	1	14	28	7.771E8	1302	149.0	5.67
Q8NF50-2	Isoform 2 of Dedicator of cytokinesis protein 8 OS=Homo sapiens GN=DOCK8 - (DOCK8_HUMAN)	22.32	7.16	9	1	16	50	3.172E8	2067	235.0	6.79
Q9Y4C2-2	Isoform 2 of Protein FAM115A OS=Homo sapiens GN=FAM115A - (F115A_HUMAN)	22.30	5.33	5	1	4	14	4.014E8	919	101.8	6.51
P18669	Phosphoglycerate mutase 1 OS=Homo sapiens GN=PGAM1 PE=1 SV=2 - (PGAM1_HUMAN)	22.21	27.95	14	6	7	12	9.628E7	254	28.8	7.18
O14777	Kinetochore protein NDC80 homolog OS=Homo sapiens GN=NDC80 PE=1 SV=1 - (NDC80_HUMAN)	22.12	9.19	2	1	8	18	2.446E7	642	73.9	5.60
P22392-2	Isoform 3 of Nucleoside diphosphate kinase B OS=Homo sapiens GN=NME2 - (NDKB_HUMAN)	21.99	48.31	10	6	8	12	1.051E8	267	30.1	8.92
Q5D862	Filaggrin-2 OS=Homo sapiens GN=FLG2 PE=1 SV=1 - (FILA2_HUMAN)	21.94	3.76	1	3	5	11	1.454E7	2391	247.9	8.31
O95147	Dual specificity protein phosphatase 14 OS=Homo sapiens GN=DUSP14 PE=1 SV=1 - (DUS14_HUMAN)	21.74	37.37	1	4	6	10	2.798E7	198	22.2	9.57
Q9BTQ7	Similar to ribosomal protein L23 (Fragment) OS=Homo sapiens PE=1 SV=1 - (Q9BTQ7_HUMAN)	21.64	50.75	4	4	7	9	2.744E7	134	14.1	10.26
Q8IW70	Transmembrane protein 151B OS=Homo sapiens GN=TMEM151B PE=2 SV=2 - (T151B_HUMAN)	21.63	5.12	1	1	3	11	5.418E7	566	61.5	7.14
P06734-2	Isoform B of Low affinity immunoglobulin epsilon Fc receptor OS=Homo sapiens GN=FCER2 - (FCER2_HUMAN)	20.71	10.00	2	1	3	15	3.336E7	320	36.3	5.83
P62136-2	Isoform 2 of Serine/threonine-protein phosphatase PP1-alpha catalytic subunit OS=Homo sapiens GN=PPP1CA - (PP1A_HUMAN)	20.55	12.90	22	4	4	22	8.147E7	341	38.6	6.62
Q6ZS99	cDNA FLJ45706 fis, clone FEBRA2028457, highly similar to Nucleolin OS=Homo sapiens PE=2 SV=1 - (Q6ZS99_HUMAN)	20.46	17.58	7	2	11	28	1.794E8	603	65.9	4.55
Q86VG2	Splicing factor proline/glutamine-rich (Polypyrimidine tract binding protein associated) OS=Homo sapiens GN=SFPQ PE=2 SV=1 - (Q86VG2_HUMAN)	20.28	17.54	27	1	13	22	1.592E8	707	76.1	9.48
O00362	Putative p150 OS=Homo sapiens PE=4 SV=1 - (O00362_HUMAN)	20.26	6.20	20	1	9	20	1.293E8	1275	149.1	9.69
Q66LE6	Serine/threonine-protein phosphatase 2A 55 kDa regulatory subunit B delta isoform OS=Homo sapiens GN=PPP2R2D PE=1 SV=1 - (2ABD_HUMAN)	20.17	9.49	17	2	4	11	3.676E8	453	52.0	6.39
P52272-2	Isoform 2 of Heterogeneous nuclear ribonucleoprotein M OS=Homo sapiens GN=HNRNPM - (HNRPM_HUMAN)	20.14	17.22	3	1	11	53	6.816E8	691	73.6	8.82
E9PI98	Layilin (Fragment) OS=Homo sapiens GN=LAYN PE=4 SV=1 - (E9PI98_HUMAN)	20.01	14.06	1	1	1	9	1.375E8	64	7.4	8.00
Q92878-3	Isoform 3 of DNA repair protein RAD50 OS=Homo sapiens GN=RAD50 - (RAD50_HUMAN)	19.97	11.94	3	1	17	24	2.528E8	1173	138.3	6.55
B3KR97	cDNA FLJ33900 fis, clone CTONG2008262, highly similar to Low density lipoprotein receptor adapter protein 1 OS=Homo sapiens PE=2 SV=1 - (B3KR97_HUMAN)	19.95	8.44	2	1	3	14	9.928E7	308	33.9	6.70
Q5T749	Keratinocyte proline-rich protein OS=Homo sapiens GN=KPRP PE=1 SV=1 - (KPRP_HUMAN)	19.93	4.49	1	3	3	21	1.839E7	579	64.1	8.27
Q9H8X9	Probable palmitoyltransferase ZDHHC11 OS=Homo sapiens GN=ZDHHC11 PE=2 SV=1 - (ZDH11_HUMAN)	19.92	9.47	1	1	3	16	1.186E8	412	45.9	8.31
Q8IWB6-3	Isoform 3 of Inactive serine/threonine-protein kinase TEX14 OS=Homo sapiens GN=TEX14 - (TEX14_HUMAN)	19.89	8.82	3	1	10	19	3.216E8	1451	162.6	5.25
C9JQD1	Glucokinase OS=Homo sapiens GN=GCK PE=3 SV=1 - (C9JQD1_HUMAN)	19.82	8.93	5	1	5	17	1.931E8	448	50.1	5.27
Q8WXX0	Dynein heavy chain 7, axonemal OS=Homo sapiens GN=DNAH7 PE=1 SV=2 - (DYH7_HUMAN)	19.60	3.33	1	1	11	15	2.216E8	4024	460.9	6.00
E9PRY8	Elongation factor 1-delta OS=Homo sapiens GN=EEF1D PE=3 SV=1 - (E9PRY8_HUMAN)	19.51	9.04	24	2	7	14	4.146E8	697	76.5	7.05
P11137-3	Isoform 3 of Microtubule-associated protein 2 OS=Homo sapiens GN=MAP2 - (MAP2_HUMAN)	19.48	5.54	6	1	9	17	1.778E8	1823	199.0	4.91
Q9H2L5	Ras association domain-containing protein 4 OS=Homo sapiens GN=RASSF4 PE=1 SV=2 - (RASF4_HUMAN)	19.38	17.45	7	1	3	9	1.107E8	321	36.7	7.75
G3V193	Activating molecule in BECN1-regulated autophagy protein 1 OS=Homo sapiens GN=AMBRA1 PE=4 SV=1 - (G3V193_HUMAN)	19.38	7.21	7	1	8	16	1.028E8	1179	129.5	6.98
P07195	L-lactate dehydrogenase B chain OS=Homo sapiens GN=LDHB PE=1 SV=2 - (LDHB_HUMAN)	19.36	13.77	14	3	5	12	7.714E7	334	36.6	6.05
A8K8P3-10	Isoform 10 of Protein SFI1 homolog OS=Homo sapiens GN=SFI1 - (SFI1_HUMAN)	19.31	9.93	11	1	13	29	2.284E9	1148	136.6	10.71
C9K0X2	Uncharacterized protein OS=Homo sapiens PE=4 SV=1 - (C9K0X2_HUMAN)	19.25	5.92	4	2	2	15	2.935E8	152	15.7	6.33
B2RAP5	cDNA, FLJ95039, highly similar to Homo sapiens peroxisomal biogenesis factor 5-like (PEX5L), mRNA OS=Homo sapiens PE=2 SV=1 - (B2RAP5_HUMAN)	19.09	6.23	15	1	3	11	5.617E7	626	69.6	5.29
P62851	40S ribosomal protein S25 OS=Homo sapiens GN=RPS25 PE=1 SV=1 - (RS25_HUMAN)	18.96	32.80	1	4	5	10	7.623E7	125	13.7	10.11
D6RAD4	Cyclin-dependent kinase 7 OS=Homo sapiens GN=CDK7 PE=4 SV=1 - (D6RAD4_HUMAN)	18.84	7.12	3	1	2	8	6.895E7	309	34.6	8.28
B2RDE1	cDNA, FLJ96568, highly similar to Homo sapiens tropomyosin 3 (TPM3), mRNA OS=Homo sapiens PE=2 SV=1 - (B2RDE1_HUMAN)	18.80	31.05	63	5	9	16	8.446E7	248	29.0	4.75
Q53F18	Homer 1 variant (Fragment) OS=Homo sapiens PE=2 SV=1 - (Q53F18_HUMAN)	18.78	13.84	5	1	6	18	1.067E7	354	40.2	5.44
Q14997	Proteasome activator complex subunit 4 OS=Homo sapiens GN=PSME4 PE=1 SV=2 - (PSME4_HUMAN)	18.68	2.39	4	1	6	20	4.915E7	1843	211.2	6.90
Q9UGL1-2	Isoform 2 of Lysine-specific demethylase 5B OS=Homo sapiens GN=KDM5B - (KDM5B_HUMAN)	18.65	6.08	13	2	9	24	2.364E8	1580	179.3	6.76
P68402	Platelet-activating factor acetylhydrolase IB subunit beta OS=Homo sapiens GN=PAFAH1B2 PE=1 SV=1 - (PA1B2_HUMAN)	18.64	11.79	1	1	3	20	9.984E7	229	25.6	5.92
I3L518	Cytosolic Fe-S cluster assembly factor NUBP1 (Fragment) OS=Homo sapiens GN=NUBP1 PE=4 SV=1 - (I3L518_HUMAN)	18.55	22.22	1	1	1	8	1.847E8	63	7.1	9.74
B4DXS5	cDNA FLJ57802, highly similar to Zinc finger protein 225 OS=Homo sapiens PE=2 SV=1 - (B4DXS5_HUMAN)	18.54	7.61	1	1	6	16	4.518E7	670	78.1	8.92
Q15365	Poly(rC)-binding protein 1 OS=Homo sapiens GN=PCBP1 PE=1 SV=2 - (PCBP1_HUMAN)	18.45	9.27	1	1	3	16	3.313E8	356	37.5	7.09
Q9BV44	THUMP domain-containing protein 3 OS=Homo sapiens GN=THUMPD3 PE=1 SV=1 - (THUM3_HUMAN)	18.44	11.24	2	1	6	15	9.358E7	507	57.0	6.37
F8WD27	Ryanodine receptor 3 OS=Homo sapiens GN=RYR3 PE=4 SV=1 - (F8WD27_HUMAN)	18.35	3.21	5	1	15	48	6.575E8	4866	551.4	5.71
B4DWN9	cDNA FLJ55258, highly similar to Homo sapiens pleckstrin homology domain containing, family F member 1, mRNA OS=Homo sapiens PE=2 SV=1 - (B4DWN9_HUMAN)	18.31	11.54	1	1	5	14	2.390E8	364	40.7	8.63
H0YGD3	Nesprin-1 (Fragment) OS=Homo sapiens GN=SYNE1 PE=4 SV=1 - (H0YGD3_HUMAN)	18.09	3.86	1	1	1	24	4.058E7	207	23.9	8.12
P07900	Heat shock protein HSP 90-alpha OS=Homo sapiens GN=HSP90AA1 PE=1 SV=5 - (HS90A_HUMAN)	18.08	19.54	35	3	17	30	6.911E7	732	84.6	5.02
B3KUD4	cDNA FLJ39594 fis, clone SKNSH2001875, highly similar to Ribonuclease H1 (EC 3.1.26.4) OS=Homo sapiens PE=2 SV=1 - (B3KUD4_HUMAN)	18.05	22.49	2	1	2	14	9.747E7	169	18.8	8.60
P18124	60S ribosomal protein L7 OS=Homo sapiens GN=RPL7 PE=1 SV=1 - (RL7_HUMAN)	18.03	38.71	5	4	10	17	1.558E8	248	29.2	10.65
Q13228	Selenium-binding protein 1 OS=Homo sapiens GN=SELENBP1 PE=1 SV=2 - (SBP1_HUMAN)	17.97	15.68	12	4	7	18	5.111E7	472	52.4	6.37
Q8N264	Rho GTPase-activating protein 24 OS=Homo sapiens GN=ARHGAP24 PE=1 SV=2 - (RHG24_HUMAN)	17.95	7.22	6	1	5	13	2.512E8	748	84.2	6.67
E5RFJ1	E3 SUMO-protein ligase NSE2 OS=Homo sapiens GN=NSMCE2 PE=4 SV=1 - (E5RFJ1_HUMAN)	17.89	21.93	2	1	5	28	3.629E8	187	21.8	8.66
G8JLF2	Coiled-coil and C2 domain-containing protein 1B OS=Homo sapiens GN=CC2D1B PE=4 SV=1 - (G8JLF2_HUMAN)	17.86	15.23	7	1	11	21	1.534E9	755	83.2	5.63
Q6YHK3-4	Isoform 4 of CD109 antigen OS=Homo sapiens GN=CD109 - (CD109_HUMAN)	17.86	6.79	4	2	11	12	2.335E8	1428	159.6	5.80
P16402	Histone H1.3 OS=Homo sapiens GN=HIST1H1D PE=1 SV=2 - (H13_HUMAN)	17.48	30.32	6	3	10	56	4.401E7	221	22.3	11.02
P08758	Annexin A5 OS=Homo sapiens GN=ANXA5 PE=1 SV=2 - (ANXA5_HUMAN)	17.29	18.44	4	1	6	9	1.104E8	320	35.9	5.05
Q5VYY1	Ankyrin repeat domain-containing protein 22 OS=Homo sapiens GN=ANKRD22 PE=2 SV=1 - (ANR22_HUMAN)	17.04	12.57	1	2	3	11	8.549E7	191	21.8	8.84
C9JNW5	60S ribosomal protein L24 OS=Homo sapiens GN=RPL24 PE=4 SV=1 - (C9JNW5_HUMAN)	16.98	25.33	3	3	4	9	4.073E7	150	17.5	10.78
E1NZA1	Peroxisome proliferator activated receptor interacting complex protein OS=Homo sapiens GN=PRIC295 PE=2 SV=1 - (E1NZA1_HUMAN)	16.87	6.36	3	1	17	29	1.332E9	2671	292.6	7.43
B4DEB9	cDNA FLJ61099, highly similar to ADP-ribosylation factor 1 OS=Homo sapiens PE=2 SV=1 - (B4DEB9_HUMAN)	16.73	24.86	19	2	4	10	6.501E7	173	19.6	8.94
H9C5F6	MHC class I antigen (Fragment) OS=Homo sapiens GN=HLA-A PE=3 SV=1 - (H9C5F6_HUMAN)	16.57	13.26	1	1	2	9	6.372E8	181	20.9	5.48
F5GZX2	Tektin-1 OS=Homo sapiens GN=TEKT1 PE=4 SV=1 - (F5GZX2_HUMAN)	16.12	12.13	4	1	4	14	3.207E7	272	31.0	6.34
P11940-2	Isoform 2 of Polyadenylate-binding protein 1 OS=Homo sapiens GN=PABPC1 - (PABP1_HUMAN)	16.03	14.99	21	2	7	26	8.178E7	547	61.1	9.07
A8K1U9	cDNA FLJ76762, highly similar to Homo sapiens suppressor of zeste 12 homolog (Drosophila) (SUZ12), mRNA OS=Homo sapiens PE=2 SV=1 - (A8K1U9_HUMAN)	16.02	2.71	2	1	2	9	5.476E7	739	83.0	8.81
P62913-2	Isoform 2 of 60S ribosomal protein L11 OS=Homo sapiens GN=RPL11 - (RL11_HUMAN)	15.99	28.25	5	4	5	9	3.499E7	177	20.1	9.60
P30613-2	Isoform L-type of Pyruvate kinase isozymes R/L OS=Homo sapiens GN=PKLR - (KPYR_HUMAN)	15.85	19.52	1	1	8	16	1.606E8	543	58.5	7.05
B4DXF3	cDNA FLJ57650, highly similar to Bleomycin hydrolase (EC 3.4.22.40) OS=Homo sapiens PE=2 SV=1 - (B4DXF3_HUMAN)	15.78	6.52	3	2	3	9	2.364E7	368	42.4	6.25
E7EVW9	Oxygen-regulated protein 1 OS=Homo sapiens GN=RP1 PE=4 SV=1 - (E7EVW9_HUMAN)	15.76	10.26	3	1	7	13	3.567E8	916	102.5	7.71
B4DXW2	cDNA FLJ60947, highly similar to Coiled-coil domain-containing protein 9 OS=Homo sapiens PE=2 SV=1 - (B4DXW2_HUMAN)	15.69	12.28	1	1	8	28	2.633E8	513	58.0	5.21
Q96EA4	Protein Spindly OS=Homo sapiens GN=CCDC99 PE=1 SV=2 - (SPDLY_HUMAN)	15.56	12.89	6	1	8	14	6.183E7	605	70.1	5.47
Q8N6N2	Tetratricopeptide repeat protein 9B OS=Homo sapiens GN=TTC9B PE=2 SV=1 - (TTC9B_HUMAN)	15.35	10.46	2	1	3	9	8.381E6	239	25.9	9.48
Q8IZX4	Transcription initiation factor TFIID subunit 1-like OS=Homo sapiens GN=TAF1L PE=1 SV=1 - (TAF1L_HUMAN)	15.32	3.94	7	1	7	41	5.097E8	1826	207.2	5.40
Q8NEZ4	Histone-lysine N-methyltransferase MLL3 OS=Homo sapiens GN=MLL3 PE=1 SV=3 - (MLL3_HUMAN)	15.32	2.89	25	1	13	26	7.111E7	4911	541.0	6.49
Q5JR95	40S ribosomal protein S8 OS=Homo sapiens GN=RPS8 PE=4 SV=1 - (Q5JR95_HUMAN)	15.18	35.64	3	5	6	7	3.278E7	188	21.9	10.36
Q7Z353	Highly divergent homeobox OS=Homo sapiens GN=HDX PE=1 SV=1 - (HDX_HUMAN)	15.10	8.41	1	1	5	15	1.150E8	690	77.2	5.87
Q9BYR3	Keratin-associated protein 4-4 OS=Homo sapiens GN=KRTAP4-4 PE=2 SV=1 - (KRA44_HUMAN)	15.08	7.23	1	1	1	9	2.845E7	166	18.0	7.91
B2R717	cDNA, FLJ93235, highly similar to Homo sapiens cathepsin L2 (CTSL2), mRNA OS=Homo sapiens PE=2 SV=1 - (B2R717_HUMAN)	15.04	22.75	2	1	5	8	4.577E7	334	37.3	8.76
I3L3E2	Multidrug and toxin extrusion protein 1 (Fragment) OS=Homo sapiens GN=SLC47A1 PE=4 SV=1 - (I3L3E2_HUMAN)	15.00	5.59	8	1	1	8	5.057E8	161	17.1	5.82
P13010	X-ray repair cross-complementing protein 5 OS=Homo sapiens GN=XRCC5 PE=1 SV=3 - (XRCC5_HUMAN)	14.97	8.20	2	1	5	11	1.659E8	732	82.7	5.81
Q14966-3	Isoform 3 of Zinc finger protein 638 OS=Homo sapiens GN=ZNF638 - (ZN638_HUMAN)	14.96	6.54	8	1	11	26	5.301E8	1957	218.1	6.42
P10599	Thioredoxin OS=Homo sapiens GN=TXN PE=1 SV=3 - (THIO_HUMAN)	14.94	51.43	3	5	6	7	4.444E7	105	11.7	4.92
P29034	Protein S100-A2 OS=Homo sapiens GN=S100A2 PE=1 SV=3 - (S10A2_HUMAN)	14.92	36.73	2	6	6	8	7.731E7	98	11.1	4.78
H0YFW4	Low-density lipoprotein receptor-related protein 1B OS=Homo sapiens GN=LRP1B PE=4 SV=1 - (H0YFW4_HUMAN)	14.90	1.90	5	1	8	46	2.748E8	4537	508.5	5.36
D3DPI2	HCG1641229, isoform CRA_a OS=Homo sapiens GN=hCG_1641229 PE=4 SV=1 - (D3DPI2_HUMAN)	14.89	33.79	33	3	9	28	8.748E8	293	32.3	4.97
P50395	Rab GDP dissociation inhibitor beta OS=Homo sapiens GN=GDI2 PE=1 SV=2 - (GDIB_HUMAN)	14.78	13.48	11	2	5	10	2.211E7	445	50.6	6.47
Q6P084	MAP3K2 protein (Fragment) OS=Homo sapiens GN=MAP3K2 PE=2 SV=1 - (Q6P084_HUMAN)	14.77	10.68	1	1	2	14	9.394E7	234	26.8	9.23
B4E2M0	cDNA FLJ55443, highly similar to Ras GTPase-activating-like protein IQGAP1 OS=Homo sapiens PE=2 SV=1 - (B4E2M0_HUMAN)	14.71	8.39	1	1	9	21	7.632E7	1085	124.5	8.47
H0YH34	ADP-ribosylation factor-like protein 13A (Fragment) OS=Homo sapiens GN=ARL13A PE=4 SV=1 - (H0YH34_HUMAN)	14.70	8.06	3	1	1	7	1.535E9	124	14.2	9.64
Q9BYX2-4	Isoform 4 of TBC1 domain family member 2A OS=Homo sapiens GN=TBC1D2 - (TBD2A_HUMAN)	14.69	13.52	7	1	9	21	1.299E8	710	81.6	7.21
P46783	40S ribosomal protein S10 OS=Homo sapiens GN=RPS10 PE=1 SV=1 - (RS10_HUMAN)	14.65	43.64	3	2	7	13	1.141E8	165	18.9	10.15
B4E1M1	cDNA FLJ60391, highly similar to Lactoperoxidase (EC 1.11.1.7) OS=Homo sapiens PE=2 SV=1 - (B4E1M1_HUMAN)	14.64	7.20	3	1	5	27	5.575E8	653	73.9	8.15
B4DNL5	cDNA FLJ59361, highly similar to Protein disulfide-isomerase (EC 5.3.4.1) OS=Homo sapiens PE=2 SV=1 - (B4DNL5_HUMAN)	14.62	11.18	17	3	6	9	3.005E7	492	55.3	4.79
P60900	Proteasome subunit alpha type-6 OS=Homo sapiens GN=PSMA6 PE=1 SV=1 - (PSA6_HUMAN)	14.62	22.36	9	3	6	7	1.931E8	246	27.4	6.76
P05089	Arginase-1 OS=Homo sapiens GN=ARG1 PE=1 SV=2 - (ARGI1_HUMAN)	14.53	12.42	3	1	3	7	7.030E6	322	34.7	7.21
Q6ZPB4	cDNA FLJ26129 fis, clone TMS02971 OS=Homo sapiens PE=2 SV=1 - (Q6ZPB4_HUMAN)	14.49	25.71	1	1	3	10	1.516E8	140	16.1	6.77
B2R9T9	cDNA, FLJ94551 OS=Homo sapiens PE=2 SV=1 - (B2R9T9_HUMAN)	14.41	16.05	4	1	4	7	9.265E6	243	26.2	10.67
A5YM50	RAB11B protein OS=Homo sapiens GN=RAB11B PE=2 SV=1 - (A5YM50_HUMAN)	14.37	22.02	12	4	5	6	5.850E8	218	24.5	6.24
A4D1E1	Zinc finger protein 804B OS=Homo sapiens GN=ZNF804B PE=1 SV=2 - (Z804B_HUMAN)	14.32	7.93	1	1	10	16	1.063E8	1349	152.5	8.54
B4DDM6	Mitotic checkpoint protein BUB3 OS=Homo sapiens GN=BUB3 PE=2 SV=1 - (B4DDM6_HUMAN)	14.28	4.44	3	1	1	20	7.017E8	248	28.2	7.11
P80188	Neutrophil gelatinase-associated lipocalin OS=Homo sapiens GN=LCN2 PE=1 SV=2 - (NGAL_HUMAN)	14.23	30.81	4	3	6	12	2.043E7	198	22.6	8.91
P32926	Desmoglein-3 OS=Homo sapiens GN=DSG3 PE=1 SV=2 - (DSG3_HUMAN)	14.16	7.61	1	2	6	8	6.535E7	999	107.5	5.00
F8WCF6	Actin-related protein 2/3 complex subunit 4 OS=Homo sapiens GN=ARPC4 PE=4 SV=1 - (F8WCF6_HUMAN)	14.02	27.07	6	1	5	9	2.158E8	181	21.0	8.76
G3V492	Uncharacterized protein OS=Homo sapiens PE=4 SV=1 - (G3V492_HUMAN)	14.01	44.66	1	1	4	19	6.419E7	103	11.0	10.98
Q9HCY8	Protein S100-A14 OS=Homo sapiens GN=S100A14 PE=1 SV=1 - (S10AE_HUMAN)	13.95	42.31	1	3	4	6	1.527E7	104	11.7	5.24
O60303	Uncharacterized protein KIAA0556 OS=Homo sapiens GN=KIAA0556 PE=1 SV=4 - (K0556_HUMAN)	13.94	5.25	2	1	9	28	9.219E7	1618	180.8	5.87
B4DYN3	cDNA FLJ55634, highly similar to Autophagy-related protein 9A OS=Homo sapiens PE=2 SV=1 - (B4DYN3_HUMAN)	13.90	8.10	7	1	6	22	1.184E8	778	87.2	6.92
F8WCJ1	Eukaryotic translation initiation factor 5A-2 OS=Homo sapiens GN=EIF5A2 PE=4 SV=1 - (F8WCJ1_HUMAN)	13.87	32.38	9	2	5	8	9.919E7	105	11.7	9.14
E9PP21	Cysteine and glycine-rich protein 1 OS=Homo sapiens GN=CSRP1 PE=4 SV=1 - (E9PP21_HUMAN)	13.85	32.50	13	3	4	12	3.156E7	160	16.9	8.91
P62857	40S ribosomal protein S28 OS=Homo sapiens GN=RPS28 PE=1 SV=1 - (RS28_HUMAN)	13.84	46.38	1	3	3	5	7.739E6	69	7.8	10.70
A6H8Y1-7	Isoform 7 of Transcription factor TFIIIB component B'' homolog OS=Homo sapiens GN=BDP1 - (BDP1_HUMAN)	13.80	3.80	9	1	11	46	7.839E8	2552	286.0	5.17
Q86VG7	Uncharacterized protein OS=Homo sapiens PE=2 SV=1 - (Q86VG7_HUMAN)	13.75	17.47	1	1	3	8	6.982E7	229	24.4	9.94
Q9NY97-2	Isoform 2 of UDP-GlcNAc:betaGal beta-1,3-N-acetylglucosaminyltransferase 2 OS=Homo sapiens GN=B3GNT2 - (B3GN2_HUMAN)	13.74	10.94	2	1	3	14	5.291E7	393	45.5	8.13
Q9Y3P9	Rab GTPase-activating protein 1 OS=Homo sapiens GN=RABGAP1 PE=1 SV=3 - (RBGP1_HUMAN)	13.70	5.99	4	1	6	10	1.489E8	1069	121.7	5.25
Q9UPY3	Endoribonuclease Dicer OS=Homo sapiens GN=DICER1 PE=1 SV=3 - (DICER_HUMAN)	13.68	3.85	2	1	7	20	1.147E8	1922	218.5	5.68
Q9UG77	Putative uncharacterized protein DKFZp586K091 (Fragment) OS=Homo sapiens GN=DKFZp586K091 PE=2 SV=1 - (Q9UG77_HUMAN)	13.60	17.74	6	1	7	15	1.757E9	434	45.0	9.55
P18084	Integrin beta-5 OS=Homo sapiens GN=ITGB5 PE=1 SV=1 - (ITB5_HUMAN)	13.58	6.13	3	1	5	12	1.187E8	799	88.0	6.06
Q5JYB1	Zinc finger and BTB domain containing 17 (Fragment) OS=Homo sapiens GN=ZBTB17 PE=2 SV=1 - (Q5JYB1_HUMAN)	13.56	10.00	2	1	2	28	7.944E6	240	26.6	9.03
Q6ECI4	Zinc finger protein 470 OS=Homo sapiens GN=ZNF470 PE=1 SV=3 - (ZN470_HUMAN)	13.45	5.58	2	1	4	15	6.058E7	717	82.6	8.57
C9J9K3	40S ribosomal protein SA (Fragment) OS=Homo sapiens GN=RPSA PE=3 SV=1 - (C9J9K3_HUMAN)	13.43	17.80	4	3	4	5	8.880E7	264	29.5	5.25
Q7L5Y1-2	Isoform 2 of Mitochondrial enolase superfamily member 1 OS=Homo sapiens GN=ENOSF1 - (ENOF1_HUMAN)	13.41	4.16	14	1	2	15	5.115E8	361	41.0	6.46
P07741	Adenine phosphoribosyltransferase OS=Homo sapiens GN=APRT PE=1 SV=2 - (APT_HUMAN)	13.35	24.44	7	4	4	5	7.626E6	180	19.6	6.02
P13928	Annexin A8 OS=Homo sapiens GN=ANXA8 PE=1 SV=3 - (ANXA8_HUMAN)	13.31	12.54	14	3	4	7	2.756E7	327	36.9	5.78
P45880-2	Isoform 2 of Voltage-dependent anion-selective channel protein 2 OS=Homo sapiens GN=VDAC2 - (VDAC2_HUMAN)	13.30	19.43	7	4	6	12	3.156E7	283	30.4	7.20
E9PGT1	Translin OS=Homo sapiens GN=TSN PE=4 SV=1 - (E9PGT1_HUMAN)	12.91	17.49	5	1	5	14	4.235E8	223	25.6	6.89
Q8NFC6	Biorientation of chromosomes in cell division protein 1-like 1 OS=Homo sapiens GN=BOD1L1 PE=1 SV=2 - (BD1L1_HUMAN)	12.86	6.06	1	1	17	25	1.011E8	3051	330.3	5.08
F8VXI9	ARF GTPase-activating protein GIT2 OS=Homo sapiens GN=GIT2 PE=4 SV=1 - (F8VXI9_HUMAN)	12.78	6.07	1	1	3	20	7.391E8	708	78.8	7.46
Q86YL3	INM04 OS=Homo sapiens PE=2 SV=1 - (Q86YL3_HUMAN)	12.75	12.93	1	1	2	9	2.092E8	147	16.1	9.44
P16401	Histone H1.5 OS=Homo sapiens GN=HIST1H1B PE=1 SV=3 - (H15_HUMAN)	12.74	18.58	1	2	7	9	3.864E7	226	22.6	10.92
P15090	Fatty acid-binding protein, adipocyte OS=Homo sapiens GN=FABP4 PE=1 SV=3 - (FABP4_HUMAN)	12.70	50.76	1	4	5	8	6.871E7	132	14.7	7.14
B3KWQ6	cDNA FLJ43599 fis, clone SMINT2017781, highly similar to PERIPHERIN OS=Homo sapiens PE=2 SV=1 - (B3KWQ6_HUMAN)	12.65	8.51	4	1	5	41	1.348E9	470	53.6	5.47
Q8NHS4-2	Isoform 2 of Uncharacterized protein C2orf63 OS=Homo sapiens GN=C2orf63 - (CB063_HUMAN)	12.62	9.48	2	1	4	14	1.875E8	464	52.9	5.69
P62249	40S ribosomal protein S16 OS=Homo sapiens GN=RPS16 PE=1 SV=2 - (RS16_HUMAN)	12.56	26.03	3	3	4	5	4.098E7	146	16.4	10.21
B4DRA1	cDNA FLJ58609, highly similar to SH3-containing GRB2-like protein 1 OS=Homo sapiens PE=2 SV=1 - (B4DRA1_HUMAN)	12.48	15.63	4	1	6	13	1.872E8	320	36.2	5.19
B3KXG3	cDNA FLJ45362 fis, clone BRHIP3014675, highly similar to Homo sapiens BTB (POZ) domain containing 11 (BTBD11), transcript variant 2, mRNA OS=Homo sapiens PE=2 SV=1 - (B3KXG3_HUMAN)	12.39	7.80	2	1	5	24	1.575E8	641	71.9	6.87
Q9Y4G6	Talin-2 OS=Homo sapiens GN=TLN2 PE=1 SV=4 - (TLN2_HUMAN)	12.23	5.90	4	1	12	28	1.006E8	2542	271.4	5.57
P25787	Proteasome subunit alpha type-2 OS=Homo sapiens GN=PSMA2 PE=1 SV=2 - (PSA2_HUMAN)	12.07	26.50	4	3	5	7	5.679E7	234	25.9	7.43
Q5BJF6-2	Isoform 2 of Outer dense fiber protein 2 OS=Homo sapiens GN=ODF2 - (ODFP2_HUMAN)	12.05	11.93	6	1	8	26	2.590E8	763	88.5	6.49
Q96PN6	Adenylate cyclase type 10 OS=Homo sapiens GN=ADCY10 PE=1 SV=3 - (ADCYA_HUMAN)	12.02	4.97	4	1	9	19	1.252E8	1610	187.0	7.31
Q9UIS4	Small nuclear ribonucleoprotein-associated protein OS=Homo sapiens GN=SNRPB PE=2 SV=1 - (Q9UIS4_HUMAN)	11.99	18.11	12	2	5	16	5.361E7	243	27.2	9.41
P22626-2	Isoform A2 of Heterogeneous nuclear ribonucleoproteins A2/B1 OS=Homo sapiens GN=HNRNPA2B1 - (ROA2_HUMAN)	11.85	15.25	2	3	5	8	1.634E7	341	36.0	8.65
P0CW22	40S ribosomal protein S17-like OS=Homo sapiens GN=RPS17L PE=3 SV=1 - (RS17L_HUMAN)	11.79	39.26	5	3	4	6	2.020E7	135	15.5	9.85
B4DE36	Glucose-6-phosphate isomerase OS=Homo sapiens PE=2 SV=1 - (B4DE36_HUMAN)	11.79	12.08	4	2	5	6	1.369E8	530	60.1	8.15
Q7Z612	Acidic ribosomal phosphoprotein P1 OS=Homo sapiens PE=4 SV=1 - (Q7Z612_HUMAN)	11.69	14.16	4	1	1	3	5.260E7	113	11.4	4.36
Q9NVE4	Coiled-coil domain-containing protein 87 OS=Homo sapiens GN=CCDC87 PE=2 SV=2 - (CCD87_HUMAN)	11.57	2.83	1	1	4	26	9.317E6	849	96.3	8.59
P16157-7	Isoform Er6 of Ankyrin-1 OS=Homo sapiens GN=ANK1 - (ANK1_HUMAN)	11.57	8.27	17	1	11	27	1.722E9	1718	188.6	6.89
B4DLQ3	cDNA FLJ52326, highly similar to Oral-facial-digital syndrome 1 protein OS=Homo sapiens PE=2 SV=1 - (B4DLQ3_HUMAN)	11.52	8.82	7	1	7	15	9.719E7	680	77.7	5.63
A8MXJ1	Protein STON1-GTF2A1L OS=Homo sapiens GN=STON1-GTF2A1L PE=4 SV=3 - (A8MXJ1_HUMAN)	11.45	1.59	8	1	2	7	9.269E7	1135	127.2	5.31
Q5M9N0	Coiled-coil domain-containing protein 158 OS=Homo sapiens GN=CCDC158 PE=2 SV=2 - (CD158_HUMAN)	11.32	11.77	3	1	15	31	3.230E9	1113	127.1	6.46
Q9UK61	Protein FAM208A OS=Homo sapiens GN=FAM208A PE=1 SV=3 - (F208A_HUMAN)	11.29	6.29	4	1	10	20	3.538E7	1670	188.9	5.80
Q4W4Y1	Dopamine receptor interacting protein 4 OS=Homo sapiens GN=DRIP4 PE=2 SV=1 - (Q4W4Y1_HUMAN)	11.22	5.65	6	1	5	8	5.372E7	868	96.0	6.52
E5RGC2	Zinc finger protein 354A OS=Homo sapiens GN=ZNF354A PE=4 SV=1 - (E5RGC2_HUMAN)	11.19	28.79	1	1	1	9	2.292E8	66	7.8	8.66
Q8N7B9	EF-hand calcium-binding domain-containing protein 3 OS=Homo sapiens GN=EFCAB3 PE=1 SV=1 - (EFCB3_HUMAN)	11.07	15.30	3	1	7	13	1.670E8	438	50.1	9.22
B4DTD3	cDNA FLJ50209, highly similar to DNA-repair protein XRCC1 OS=Homo sapiens PE=2 SV=1 - (B4DTD3_HUMAN)	11.07	2.66	6	1	2	12	2.630E7	602	66.0	5.85
Q6NXR8	Ribosomal protein S3A OS=Homo sapiens GN=RPS3A PE=2 SV=1 - (Q6NXR8_HUMAN)	11.07	26.52	14	4	9	14	3.064E7	264	30.0	9.73
Q6ZUS5	Coiled-coil domain-containing protein 121 OS=Homo sapiens GN=CCDC121 PE=2 SV=1 - (CC121_HUMAN)	11.04	10.43	1	1	3	8	2.754E8	278	33.0	9.83
Q6MZM0	Hephaestin-like protein 1 OS=Homo sapiens GN=HEPHL1 PE=2 SV=2 - (HPHL1_HUMAN)	11.00	7.51	1	3	7	11	1.045E8	1159	131.5	6.74
Q5VVM6	Coiled-coil domain-containing protein 30 OS=Homo sapiens GN=CCDC30 PE=2 SV=1 - (CCD30_HUMAN)	10.99	11.24	2	1	9	11	1.971E8	783	91.3	5.77
Q53SV2	Putative uncharacterized protein FLJ10035 OS=Homo sapiens GN=FLJ10035 PE=2 SV=1 - (Q53SV2_HUMAN)	10.98	7.27	9	1	4	8	3.109E8	523	58.2	6.25
A7KAX9-2	Isoform 2 of Rho GTPase-activating protein 32 OS=Homo sapiens GN=ARHGAP32 - (RHG32_HUMAN)	10.94	1.15	2	1	2	20	2.133E8	1738	190.9	6.86
H0YA96	Heterogeneous nuclear ribonucleoprotein D0 (Fragment) OS=Homo sapiens GN=HNRNPD PE=4 SV=1 - (H0YA96_HUMAN)	10.84	24.29	16	1	5	10	1.475E8	210	23.9	9.58
B3KTS5	cDNA FLJ38670 fis, clone HSYRA2000190, highly similar to Voltage-dependent anion-selective channel protein 1 OS=Homo sapiens PE=2 SV=1 - (B3KTS5_HUMAN)	10.81	11.66	6	1	4	8	1.988E7	283	30.7	7.90
E7EQG2	Eukaryotic initiation factor 4A-II OS=Homo sapiens GN=EIF4A2 PE=3 SV=1 - (E7EQG2_HUMAN)	10.80	17.68	8	1	7	11	3.470E7	362	41.3	5.64
Q6AWC6	Putative uncharacterized protein DKFZp686N14223 OS=Homo sapiens GN=DKFZp686N14223 PE=2 SV=1 - (Q6AWC6_HUMAN)	10.77	10.09	9	1	5	18	4.148E7	446	50.8	7.52
Q9BTI9	NPM1 protein (Fragment) OS=Homo sapiens GN=NPM1 PE=2 SV=2 - (Q9BTI9_HUMAN)	10.73	24.12	17	4	5	9	2.834E7	228	25.0	4.86
H0YB22	40S ribosomal protein S14 (Fragment) OS=Homo sapiens GN=RPS14 PE=4 SV=1 - (H0YB22_HUMAN)	10.70	46.67	3	2	5	9	3.646E7	120	12.9	9.85
Q2TU64	PIG48 OS=Homo sapiens PE=2 SV=1 - (Q2TU64_HUMAN)	10.68	16.51	7	1	6	23	4.907E7	545	60.5	6.35
P23284	Peptidyl-prolyl cis-trans isomerase B OS=Homo sapiens GN=PPIB PE=1 SV=2 - (PPIB_HUMAN)	10.54	10.19	5	2	3	8	7.174E7	216	23.7	9.41
Q5H9J9	Novel protein similar to t-complex 11 homolog (Mouse) TCP11 OS=Homo sapiens GN=LL0XNC01-19D8.1 PE=4 SV=1 - (Q5H9J9_HUMAN)	10.51	5.90	2	1	2	6	1.397E8	407	46.3	5.19
Q4J6C6-4	Isoform 4 of Prolyl endopeptidase-like OS=Homo sapiens GN=PREPL - (PPCEL_HUMAN)	10.47	1.72	3	1	1	13	4.019E7	638	73.3	5.44
Q96BY7	Autophagy-related protein 2 homolog B OS=Homo sapiens GN=ATG2B PE=1 SV=5 - (ATG2B_HUMAN)	10.44	5.77	1	1	10	19	1.514E8	2078	232.6	5.76
P35442	Thrombospondin-2 OS=Homo sapiens GN=THBS2 PE=1 SV=2 - (TSP2_HUMAN)	10.37	9.98	6	1	8	20	8.395E7	1172	129.9	4.83
P18621	60S ribosomal protein L17 OS=Homo sapiens GN=RPL17 PE=1 SV=3 - (RL17_HUMAN)	10.33	23.37	2	2	6	8	1.925E7	184	21.4	10.17
E9PP36	60S ribosomal protein L8 OS=Homo sapiens GN=RPL8 PE=4 SV=1 - (E9PP36_HUMAN)	10.32	22.97	7	3	4	6	6.638E7	148	16.2	11.90
Q7Z3D4	LysM and putative peptidoglycan-binding domain-containing protein 3 OS=Homo sapiens GN=LYSMD3 PE=2 SV=2 - (LYSM3_HUMAN)	10.29	11.44	1	1	2	6	1.457E8	306	34.5	5.97
Q13435	Splicing factor 3B subunit 2 OS=Homo sapiens GN=SF3B2 PE=1 SV=2 - (SF3B2_HUMAN)	10.27	15.98	5	1	14	23	1.862E8	895	100.2	5.67
B9EK47	HEAT repeat containing 5B OS=Homo sapiens GN=HEATR5B PE=2 SV=1 - (B9EK47_HUMAN)	10.24	5.17	3	1	10	14	8.346E7	2071	224.1	7.18
B4DH02	cDNA FLJ50510, highly similar to Heat shock 70 kDa protein 4 OS=Homo sapiens PE=2 SV=1 - (B4DH02_HUMAN)	10.24	8.57	10	1	6	10	9.029E7	840	94.3	5.19
Q96FQ6	Protein S100-A16 OS=Homo sapiens GN=S100A16 PE=1 SV=1 - (S10AG_HUMAN)	10.20	21.36	1	2	2	5	6.278E6	103	11.8	6.79
Q12915	Ibd1 protein (Fragment) OS=Homo sapiens GN=Ibd1 PE=2 SV=1 - (Q12915_HUMAN)	10.19	13.24	1	1	2	4	1.274E8	204	22.1	8.46
Q96L21	60S ribosomal protein L10-like OS=Homo sapiens GN=RPL10L PE=1 SV=3 - (RL10L_HUMAN)	10.09	29.44	8	4	6	7	1.569E7	214	24.5	10.01
Q9H0C2	ADP/ATP translocase 4 OS=Homo sapiens GN=SLC25A31 PE=1 SV=1 - (ADT4_HUMAN)	10.08	22.22	9	2	9	16	1.419E8	315	35.0	9.89
F5GX67	Keratin-associated protein 1-5 OS=Homo sapiens GN=KRTAP1-5 PE=4 SV=1 - (F5GX67_HUMAN)	10.07	4.27	2	1	1	12	4.252E8	164	17.0	7.06
B7Z688	cDNA FLJ57192, highly similar to Tuftelin OS=Homo sapiens PE=2 SV=1 - (B7Z688_HUMAN)	10.03	16.57	1	1	5	11	2.681E7	338	38.6	6.15
P02649	Apolipoprotein E OS=Homo sapiens GN=APOE PE=1 SV=1 - (APOE_HUMAN)	10.01	18.61	5	1	8	22	8.569E7	317	36.1	5.73
Q9Y4D8-4	Isoform 4 of Probable E3 ubiquitin-protein ligase C12orf51 OS=Homo sapiens GN=C12orf51 - (K0614_HUMAN)	9.98	4.62	7	1	13	20	1.058E9	3984	437.8	6.16
P46781	40S ribosomal protein S9 OS=Homo sapiens GN=RPS9 PE=1 SV=3 - (RS9_HUMAN)	9.97	30.41	5	3	6	9	4.568E7	194	22.6	10.65
Q8WXF0	Serine/arginine-rich splicing factor 12 OS=Homo sapiens GN=SRSF12 PE=1 SV=1 - (SRS12_HUMAN)	9.96	6.90	2	1	3	7	1.473E8	261	30.5	11.69
Q9HD67	Unconventionnal myosin-X OS=Homo sapiens GN=MYO10 PE=1 SV=3 - (MYO10_HUMAN)	9.95	4.42	6	1	9	18	3.446E8	2058	237.2	6.21
B9EGR0	C4orf8 protein OS=Homo sapiens GN=C4orf8 PE=2 SV=1 - (B9EGR0_HUMAN)	9.95	2.89	10	1	4	11	1.986E8	1211	134.2	6.40
B4E3K4	cDNA FLJ57020, highly similar to Rho-guanine nucleotide exchange factor OS=Homo sapiens PE=2 SV=1 - (B4E3K4_HUMAN)	9.87	6.54	8	1	8	14	2.948E8	1392	157.1	6.40
Q8IXI1	Mitochondrial Rho GTPase 2 OS=Homo sapiens GN=RHOT2 PE=1 SV=2 - (MIRO2_HUMAN)	9.83	2.59	1	1	2	17	9.524E7	618	68.1	5.86
Q08999	Retinoblastoma-like protein 2 OS=Homo sapiens GN=RBL2 PE=1 SV=3 - (RBL2_HUMAN)	9.79	2.81	4	1	4	7	1.720E7	1139	128.3	7.44
P12273	Prolactin-inducible protein OS=Homo sapiens GN=PIP PE=1 SV=1 - (PIP_HUMAN)	9.79	10.96	1	1	1	2	5.054E6	146	16.6	8.05
Q13451	Peptidyl-prolyl cis-trans isomerase FKBP5 OS=Homo sapiens GN=FKBP5 PE=1 SV=2 - (FKBP5_HUMAN)	9.69	10.07	9	2	5	10	3.168E7	457	51.2	5.90
B7Z5R8	cDNA FLJ61653, highly similar to Potassium/sodium hyperpolarization-activated cyclic nucleotide-gated channel 3 OS=Homo sapiens PE=2 SV=1 - (B7Z5R8_HUMAN)	9.68	10.23	3	1	5	10	4.895E7	469	50.9	10.51
Q96P63	Serpin B12 OS=Homo sapiens GN=SERPINB12 PE=1 SV=1 - (SPB12_HUMAN)	9.59	8.40	3	2	3	5	4.042E7	405	46.2	5.53
Q9UK96	F-box only protein 10 OS=Homo sapiens GN=FBXO10 PE=2 SV=3 - (FBX10_HUMAN)	9.53	11.09	3	1	9	14	6.005E7	956	105.1	8.13
A7E2C5	BZRAP1 protein OS=Homo sapiens GN=BZRAP1 PE=2 SV=1 - (A7E2C5_HUMAN)	9.53	5.45	6	1	9	18	1.809E8	1797	193.4	5.05
Q68CX2	Putative uncharacterized protein DKFZp761A1822 OS=Homo sapiens GN=DKFZp761A1822 PE=2 SV=2 - (Q68CX2_HUMAN)	9.50	4.74	1	1	1	6	3.099E7	211	22.7	7.46
Q5VWI1	Transcription elongation regulator 1-like protein OS=Homo sapiens GN=TCERG1L PE=2 SV=2 - (TCRGL_HUMAN)	9.48	5.46	1	1	4	12	6.089E7	586	65.6	9.83
Q5T7W0-2	Isoform 2 of Zinc finger protein 618 OS=Homo sapiens GN=ZNF618 - (ZN618_HUMAN)	9.47	4.30	3	1	5	8	1.090E8	861	95.6	7.12
Q9P2Z0	THAP domain-containing protein 10 OS=Homo sapiens GN=THAP10 PE=2 SV=1 - (THA10_HUMAN)	9.47	10.89	2	1	4	14	7.944E7	257	28.3	8.60
B8XPJ7	Soluble catechol-O-methyltransferase OS=Homo sapiens GN=COMT PE=2 SV=1 - (B8XPJ7_HUMAN)	9.46	13.57	6	1	3	3	1.076E8	221	24.5	5.45
Q93084-7	Isoform SERCA3F of Sarcoplasmic/endoplasmic reticulum calcium ATPase 3 OS=Homo sapiens GN=ATP2A3 - (AT2A3_HUMAN)	9.39	9.68	8	1	8	10	7.285E8	1033	112.7	5.81
Q8N4Y2	EF-hand calcium-binding domain-containing protein 4A OS=Homo sapiens GN=EFCAB4A PE=2 SV=3 - (EFC4A_HUMAN)	9.39	5.51	2	1	3	14	3.059E8	399	44.9	5.34
P49454	Centromere protein F OS=Homo sapiens GN=CENPF PE=1 SV=2 - (CENPF_HUMAN)	9.22	4.89	1	1	16	28	9.818E8	3210	367.5	5.07
Q5VU13	V-set and immunoglobulin domain-containing protein 8 OS=Homo sapiens GN=VSIG8 PE=1 SV=1 - (VSIG8_HUMAN)	9.20	8.45	1	2	3	4	1.053E7	414	43.9	7.20
E5RIY5	Serine protease 45 OS=Homo sapiens GN=PRSS45 PE=3 SV=1 - (E5RIY5_HUMAN)	9.15	8.18	3	1	1	4	2.229E8	110	12.4	7.59
P35268	60S ribosomal protein L22 OS=Homo sapiens GN=RPL22 PE=1 SV=2 - (RL22_HUMAN)	9.14	28.91	2	1	5	7	1.783E7	128	14.8	9.19
H0YN18	Proteasome subunit alpha type-4 OS=Homo sapiens GN=PSMA4 PE=4 SV=1 - (H0YN18_HUMAN)	9.13	12.17	11	1	3	15	6.520E6	230	26.3	8.32
E7EPB3	60S ribosomal protein L14 OS=Homo sapiens GN=RPL14 PE=4 SV=1 - (E7EPB3_HUMAN)	9.03	21.77	7	2	4	21	1.997E9	124	14.5	10.21
H0YN94	Protein FAM81A (Fragment) OS=Homo sapiens GN=FAM81A PE=4 SV=1 - (H0YN94_HUMAN)	8.98	37.96	1	1	4	6	1.825E8	108	12.6	8.88
P60866	40S ribosomal protein S20 OS=Homo sapiens GN=RPS20 PE=1 SV=1 - (RS20_HUMAN)	8.97	42.86	5	2	5	6	4.999E7	119	13.4	9.94
P48047	ATP synthase subunit O, mitochondrial OS=Homo sapiens GN=ATP5O PE=1 SV=1 - (ATPO_HUMAN)	8.90	22.54	4	2	4	7	2.115E7	213	23.3	9.96
B7ZLJ4	Peroxiredoxin 5 OS=Homo sapiens GN=PRDX5 PE=2 SV=1 - (B7ZLJ4_HUMAN)	8.87	32.35	4	1	4	9	1.672E7	170	17.4	8.76
F8W6N3	Ubiquitin carboxyl-terminal hydrolase BAP1 OS=Homo sapiens GN=BAP1 PE=4 SV=1 - (F8W6N3_HUMAN)	8.85	2.95	3	1	3	11	2.873E7	711	78.1	6.81
P30041	Peroxiredoxin-6 OS=Homo sapiens GN=PRDX6 PE=1 SV=3 - (PRDX6_HUMAN)	8.84	13.39	2	3	3	5	2.462E7	224	25.0	6.38
Q69YN2	CWF19-like protein 1 OS=Homo sapiens GN=CWF19L1 PE=1 SV=2 - (C19L1_HUMAN)	8.79	2.42	1	1	2	23	1.160E8	538	60.6	7.24
P04080	Cystatin-B OS=Homo sapiens GN=CSTB PE=1 SV=2 - (CYTB_HUMAN)	8.59	30.61	1	2	3	5	2.462E7	98	11.1	7.56
A6NJ78	Probable methyltransferase-like protein 15 OS=Homo sapiens GN=METTL15 PE=2 SV=1 - (MET15_HUMAN)	8.59	13.76	4	1	3	5	2.467E7	407	46.1	8.62
Q8IY92	Structure-specific endonuclease subunit SLX4 OS=Homo sapiens GN=SLX4 PE=1 SV=3 - (SLX4_HUMAN)	8.56	5.94	2	1	8	20	4.009E7	1834	199.9	6.06
B4DHC1	cDNA FLJ53868 OS=Homo sapiens PE=2 SV=1 - (B4DHC1_HUMAN)	8.51	24.63	5	1	7	14	2.537E8	268	31.0	8.07
Q9NQ90-3	Isoform 3 of Anoctamin-2 OS=Homo sapiens GN=ANO2 - (ANO2_HUMAN)	8.34	9.72	5	1	6	16	1.148E8	998	113.4	6.48
P14174	Macrophage migration inhibitory factor OS=Homo sapiens GN=MIF PE=1 SV=4 - (MIF_HUMAN)	8.32	13.91	2	2	2	4	3.835E7	115	12.5	7.88
Q14574-2	Isoform 3B of Desmocollin-3 OS=Homo sapiens GN=DSC3 - (DSC3_HUMAN)	8.28	7.75	3	1	6	10	2.538E7	839	93.4	6.32
Q96AU7	Thymic stromal lymphopoietin OS=Homo sapiens GN=TSLP PE=2 SV=1 - (Q96AU7_HUMAN)	8.25	31.67	3	1	1	9	5.211E8	60	7.1	11.09
P26373	60S ribosomal protein L13 OS=Homo sapiens GN=RPL13 PE=1 SV=4 - (RL13_HUMAN)	8.24	20.38	4	3	6	14	2.183E8	211	24.2	11.65
B9EJE3	Putative uncharacterized protein OS=Homo sapiens PE=2 SV=1 - (B9EJE3_HUMAN)	8.22	15.38	5	1	2	4	1.325E8	156	17.5	10.20
B2R646	cDNA, FLJ92780, highly similar to Homo sapiens phosphodiesterase 2A, cGMP-stimulated (PDE2A), mRNA OS=Homo sapiens PE=2 SV=1 - (B2R646_HUMAN)	8.19	6.80	9	1	5	14	2.124E8	941	105.7	5.41
F5H018	GTP-binding nuclear protein Ran (Fragment) OS=Homo sapiens GN=RAN PE=4 SV=1 - (F5H018_HUMAN)	8.19	19.10	5	4	5	5	1.094E7	199	22.5	8.73
P62847-2	Isoform 2 of 40S ribosomal protein S24 OS=Homo sapiens GN=RPS24 - (RS24_HUMAN)	8.02	37.69	5	2	5	6	3.999E7	130	15.1	10.89
F5H110	Zinc finger CCCH domain-containing protein 6 OS=Homo sapiens GN=ZC3H6 PE=4 SV=1 - (F5H110_HUMAN)	8.00	6.73	3	1	5	15	3.002E8	892	100.7	8.82
P19105	Myosin regulatory light chain 12A OS=Homo sapiens GN=MYL12A PE=1 SV=2 - (ML12A_HUMAN)	7.99	24.56	7	3	4	7	4.336E7	171	19.8	4.81
Q96JP0	Protein fem-1 homolog C OS=Homo sapiens GN=FEM1C PE=2 SV=1 - (FEM1C_HUMAN)	7.99	11.18	1	1	5	13	9.710E7	617	68.6	7.68
Q9NYQ6	Cadherin EGF LAG seven-pass G-type receptor 1 OS=Homo sapiens GN=CELSR1 PE=1 SV=1 - (CELR1_HUMAN)	7.97	5.21	3	1	13	20	2.253E8	3014	329.3	5.92
B4DMB1	cDNA FLJ53358, highly similar to Heterogeneous nuclear ribonucleoprotein R OS=Homo sapiens PE=2 SV=1 - (B4DMB1_HUMAN)	7.93	12.61	19	2	7	7	8.245E7	595	67.0	8.29
B4DPU9	cDNA FLJ55129, highly similar to HMG box-containing protein 1 OS=Homo sapiens PE=2 SV=1 - (B4DPU9_HUMAN)	7.92	6.70	5	1	3	6	2.045E8	433	48.4	7.50
D3DPK5	SH3 domain binding glutamic acid-rich protein like 3, isoform CRA_a (Fragment) OS=Homo sapiens GN=SH3BGRL3 PE=4 SV=1 - (D3DPK5_HUMAN)	7.92	14.79	4	2	5	9	6.862E8	257	26.8	8.38
E7EWK6	Uncharacterized protein OS=Homo sapiens PE=4 SV=1 - (E7EWK6_HUMAN)	7.90	21.23	2	1	5	9	1.570E8	405	44.1	8.60
B4DS76	Mitogen-activated protein kinase kinase kinase 11 OS=Homo sapiens GN=MAP3K11 PE=2 SV=1 - (B4DS76_HUMAN)	7.85	11.02	3	1	6	9	2.626E8	590	65.3	9.22
Q96AG4	Leucine-rich repeat-containing protein 59 OS=Homo sapiens GN=LRRC59 PE=1 SV=1 - (LRC59_HUMAN)	7.83	6.19	1	1	2	4	9.485E6	307	34.9	9.57
B2RBR9	cDNA, FLJ95650, highly similar to Homo sapiens karyopherin (importin) beta 1 (KPNB1), mRNA OS=Homo sapiens PE=2 SV=1 - (B2RBR9_HUMAN)	7.82	6.96	2	1	5	6	2.838E7	876	97.1	4.78
B2RDM2	HCG1811539, isoform CRA_b OS=Homo sapiens GN=TXNDC5 PE=2 SV=1 - (B2RDM2_HUMAN)	7.81	8.33	5	1	4	7	3.848E7	324	36.2	5.47
C9J8H1	V-type proton ATPase subunit E 1 (Fragment) OS=Homo sapiens GN=ATP6V1E1 PE=4 SV=1 - (C9J8H1_HUMAN)	7.66	13.30	3	1	3	8	3.887E8	203	23.5	9.04
Q9BPX5	Actin-related protein 2/3 complex subunit 5-like protein OS=Homo sapiens GN=ARPC5L PE=1 SV=1 - (ARP5L_HUMAN)	7.64	24.18	2	1	4	7	2.067E7	153	16.9	6.60
P0C7V6	Putative protein FAM48B2 OS=Homo sapiens GN=FAM48B2 PE=5 SV=1 - (F48B2_HUMAN)	7.52	9.06	1	1	6	15	5.391E7	817	87.5	8.79
P35712-4	Isoform 4 of Transcription factor SOX-6 OS=Homo sapiens GN=SOX6 - (SOX6_HUMAN)	7.46	5.99	4	1	5	34	3.990E7	801	88.9	7.21
Q59FN4	LATS homolog 1 variant (Fragment) OS=Homo sapiens PE=2 SV=1 - (Q59FN4_HUMAN)	7.46	8.19	2	1	6	11	1.040E8	830	92.3	9.45
O94804	Serine/threonine-protein kinase 10 OS=Homo sapiens GN=STK10 PE=1 SV=1 - (STK10_HUMAN)	7.45	12.09	1	1	12	17	2.402E8	968	112.1	6.95
B7Z382	Cytosolic purine 5'-nucleotidase OS=Homo sapiens GN=NT5C2 PE=2 SV=1 - (B7Z382_HUMAN)	7.45	4.70	7	1	3	18	7.091E7	532	61.4	5.74
Q8NGI6	Olfactory receptor 4D10 OS=Homo sapiens GN=OR4D10 PE=2 SV=1 - (OR4DA_HUMAN)	7.43	9.00	2	1	3	12	5.031E7	311	35.3	8.81
Q02040	A-kinase anchor protein 17A OS=Homo sapiens GN=AKAP17A PE=1 SV=2 - (AK17A_HUMAN)	7.42	9.06	4	1	9	13	1.600E7	695	80.7	9.73
B4DLS2	cDNA FLJ50803, highly similar to Zinc finger protein 23 OS=Homo sapiens PE=2 SV=1 - (B4DLS2_HUMAN)	7.42	4.96	6	1	3	6	1.992E7	585	67.1	8.31
A8MXN1	Receptor expression-enhancing protein 6 OS=Homo sapiens GN=REEP6 PE=4 SV=1 - (A8MXN1_HUMAN)	7.37	5.63	1	1	4	44	1.001E8	480	49.7	11.24
B7Z399	cDNA FLJ50162, highly similar to Glycosyltransferase-like protein LARGE1 (EC 2.4.-.-) OS=Homo sapiens PE=2 SV=1 - (B7Z399_HUMAN)	7.36	7.27	7	1	4	5	3.548E7	688	80.5	7.36
P22528	Cornifin-B OS=Homo sapiens GN=SPRR1B PE=1 SV=2 - (SPR1B_HUMAN)	7.34	26.97	3	1	2	6	2.192E7	89	9.9	8.48
Q8N3C0	Activating signal cointegrator 1 complex subunit 3 OS=Homo sapiens GN=ASCC3 PE=1 SV=3 - (ASCC3_HUMAN)	7.28	7.54	1	1	13	33	1.005E9	2202	251.3	7.09
Q70J99	Protein unc-13 homolog D OS=Homo sapiens GN=UNC13D PE=1 SV=1 - (UN13D_HUMAN)	7.23	2.66	3	1	3	7	1.293E9	1090	123.2	6.65
D6RER6	Coproporphyrinogen-III oxidase, mitochondrial OS=Homo sapiens GN=CPOX PE=4 SV=1 - (D6RER6_HUMAN)	7.23	10.29	3	1	2	7	2.836E6	175	18.3	9.88
B7Z5E7	cDNA FLJ51046, highly similar to 60 kDa heat shock protein, mitochondrial OS=Homo sapiens PE=2 SV=1 - (B7Z5E7_HUMAN)	7.22	9.67	8	2	4	4	2.480E7	517	55.0	5.60
P16152	Carbonyl reductase (NADPH) 1 OS=Homo sapiens GN=CBR1 PE=1 SV=3 - (CBR1_HUMAN)	7.20	15.52	4	3	3	3	1.568E7	277	30.4	8.32
Q92623	Tetratricopeptide repeat protein 9A OS=Homo sapiens GN=TTC9 PE=2 SV=3 - (TTC9A_HUMAN)	7.20	22.97	1	1	5	9	8.935E8	222	24.4	8.98
Q17RC7	Exocyst complex component 3-like protein 4 OS=Homo sapiens GN=EXOC3L4 PE=2 SV=2 - (EX3L4_HUMAN)	7.16	10.39	1	2	7	10	7.704E7	722	79.8	6.32
Q9ULV0	Unconventional myosin-Vb OS=Homo sapiens GN=MYO5B PE=1 SV=3 - (MYO5B_HUMAN)	7.15	5.19	6	1	12	20	2.857E8	1848	213.5	7.20
Q96PI1	Small proline-rich protein 4 OS=Homo sapiens GN=SPRR4 PE=2 SV=1 - (SPRR4_HUMAN)	7.12	11.39	1	1	1	3	6.272E5	79	8.8	9.66
F5GY37	Prohibitin-2 OS=Homo sapiens GN=PHB2 PE=4 SV=1 - (F5GY37_HUMAN)	7.12	11.99	6	3	3	4	7.156E6	267	29.7	9.88
Q16658	Fascin OS=Homo sapiens GN=FSCN1 PE=1 SV=3 - (FSCN1_HUMAN)	7.07	11.76	7	1	4	6	1.633E7	493	54.5	7.24
F8VUA6	60S ribosomal protein L18 (Fragment) OS=Homo sapiens GN=RPL18 PE=4 SV=1 - (F8VUA6_HUMAN)	7.02	12.31	9	1	2	5	7.052E6	130	14.5	11.75
B7Z9M9	cDNA, FLJ78893, highly similar to Destrin OS=Homo sapiens PE=2 SV=1 - (B7Z9M9_HUMAN)	7.00	20.27	4	1	3	4	4.309E7	148	16.5	8.50
Q5Y190	Anchor protein OS=Homo sapiens GN=RESDA1 PE=2 SV=1 - (Q5Y190_HUMAN)	6.97	2.82	3	1	13	23	9.891E7	4186	453.0	6.83
Q14692	Ribosome biogenesis protein BMS1 homolog OS=Homo sapiens GN=BMS1 PE=1 SV=1 - (BMS1_HUMAN)	6.95	2.81	1	1	5	26	8.387E7	1282	145.7	6.44
B4E0S6	cDNA FLJ55635, highly similar to pre-mRNA-splicing factorATP-dependent RNA helicase DHX15 (EC 3.6.1.-) OS=Homo sapiens PE=2 SV=1 - (B4E0S6_HUMAN)	6.93	10.08	3	1	8	14	2.755E7	784	89.5	7.46
B7Z3I7	Malate dehydrogenase OS=Homo sapiens PE=2 SV=1 - (B7Z3I7_HUMAN)	6.89	17.05	6	1	5	7	7.513E7	352	38.6	8.00
Q9H0E3-2	Isoform 2 of Histone deacetylase complex subunit SAP130 OS=Homo sapiens GN=SAP130 - (SP130_HUMAN)	6.88	5.87	5	1	4	8	2.319E7	613	65.6	9.63
E9PJD9	60S ribosomal protein L27a OS=Homo sapiens GN=RPL27A PE=3 SV=1 - (E9PJD9_HUMAN)	6.84	37.36	6	2	4	6	3.843E7	91	10.1	10.21
P61626	Lysozyme C OS=Homo sapiens GN=LYZ PE=1 SV=1 - (LYSC_HUMAN)	6.83	25.68	2	1	4	8	7.977E6	148	16.5	9.16
P26641	Elongation factor 1-gamma OS=Homo sapiens GN=EEF1G PE=1 SV=3 - (EF1G_HUMAN)	6.83	5.95	5	1	4	6	1.728E7	437	50.1	6.67
Q5T0R7	Adenylyl cyclase-associated protein (Fragment) OS=Homo sapiens GN=CAP1 PE=2 SV=1 - (Q5T0R7_HUMAN)	6.82	25.29	12	1	2	2	1.741E7	174	19.2	7.05
B7Z1C9	Chaperonin containing TCP1, subunit 7 (Eta), isoform CRA_a OS=Homo sapiens GN=CCT7 PE=2 SV=1 - (B7Z1C9_HUMAN)	6.79	11.08	10	1	2	2	5.728E7	415	46.1	7.65
A8K321	cDNA FLJ78524, highly similar to Homo sapiens SMILE protein (SMILE), mRNA OS=Homo sapiens PE=2 SV=1 - (A8K321_HUMAN)	6.78	4.89	4	1	4	29	9.607E7	899	102.0	8.84
A9UFC0	Caspase 14 OS=Homo sapiens GN=CASP14 PE=2 SV=1 - (A9UFC0_HUMAN)	6.74	9.92	2	1	2	7	4.022E7	242	27.6	5.34
A8K865	cDNA FLJ75789, highly similar to Homo sapiens synaptophysin-like 1 (SYPL1), transcript variant 1, mRNA OS=Homo sapiens PE=2 SV=1 - (A8K865_HUMAN)	6.71	8.49	4	1	2	3	2.114E7	259	28.5	7.99
D3WYY8	Cytochrome c oxidase subunit 2 (Fragment) OS=Homo sapiens PE=4 SV=1 - (D3WYY8_HUMAN)	6.66	26.32	149	1	1	3	1.855E7	38	4.3	4.44
F2Z393	Transaldolase OS=Homo sapiens GN=TALDO1 PE=3 SV=1 - (F2Z393_HUMAN)	6.63	19.50	2	2	7	9	3.054E7	318	35.3	8.97
Q8N300	Coiled-coil domain-containing protein 23 OS=Homo sapiens GN=CCDC23 PE=2 SV=1 - (CCD23_HUMAN)	6.59	40.91	1	1	3	13	4.458E7	66	7.8	9.16
B4DLZ5	N-acetylglucosamine kinase OS=Homo sapiens GN=NAGK PE=2 SV=1 - (B4DLZ5_HUMAN)	6.59	7.95	9	2	4	7	1.682E7	390	42.0	6.68
F8W1S7	Heterogeneous nuclear ribonucleoprotein A1 OS=Homo sapiens GN=HNRNPA1 PE=4 SV=1 - (F8W1S7_HUMAN)	6.53	24.24	16	1	6	9	3.576E7	330	35.5	9.11
B2R8A3	cDNA, FLJ93805, highly similar to Homo sapiens creatine kinase, mitochondrial 2 (sarcomeric) (CKMT2), nuclear gene encoding mitochondrial protein, mRNA OS=Homo sapiens PE=2 SV=1 - (B2R8A3_HUMAN)	6.47	10.74	15	1	5	9	6.115E9	419	47.5	8.19
Q12981	Vesicle transport protein SEC20 OS=Homo sapiens GN=BNIP1 PE=1 SV=3 - (SEC20_HUMAN)	6.45	22.81	2	1	6	14	1.441E8	228	26.1	8.95
Q8IYE0	Coiled-coil domain-containing protein 146 OS=Homo sapiens GN=CCDC146 PE=2 SV=2 - (CC146_HUMAN)	6.42	13.40	2	1	9	13	3.757E7	955	112.7	8.48
B3KRS1	cDNA FLJ34794 fis, clone NT2NE2005676, highly similar to Gamma-tubulin complex component 2 OS=Homo sapiens PE=2 SV=1 - (B3KRS1_HUMAN)	6.41	5.83	7	1	4	6	6.538E7	721	82.5	6.84
P63167	Dynein light chain 1, cytoplasmic OS=Homo sapiens GN=DYNLL1 PE=1 SV=1 - (DYL1_HUMAN)	6.39	21.35	4	3	3	3	7.311E6	89	10.4	7.40
Q00610-2	Isoform 2 of Clathrin heavy chain 1 OS=Homo sapiens GN=CLTC - (CLH1_HUMAN)	6.36	4.45	2	2	7	11	6.909E7	1639	187.8	5.69
D3DV26	S100 calcium binding protein A10 (Annexin II ligand, calpactin I, light polypeptide (P11)), isoform CRA_b (Fragment) OS=Homo sapiens GN=S100A10 PE=4 SV=1 - (D3DV26_HUMAN)	6.33	15.12	3	1	3	5	3.508E7	205	22.3	10.33
Q68DE3	Basic helix-loop-helix domain-containing protein KIAA2018 OS=Homo sapiens GN=KIAA2018 PE=1 SV=3 - (K2018_HUMAN)	6.32	4.54	1	1	11	17	4.265E8	2245	241.5	7.61
Q8NE71-2	Isoform 2 of ATP-binding cassette sub-family F member 1 OS=Homo sapiens GN=ABCF1 - (ABCF1_HUMAN)	6.28	6.57	5	1	5	11	1.264E8	807	91.6	7.68
A5YM43	PHC3 protein OS=Homo sapiens GN=PHC3 PE=2 SV=1 - (A5YM43_HUMAN)	6.26	4.07	7	1	5	15	6.030E7	983	106.1	6.62
Q52LG2	Keratin-associated protein 13-2 OS=Homo sapiens GN=KRTAP13-2 PE=1 SV=1 - (KR132_HUMAN)	6.25	11.43	2	2	2	3	2.695E7	175	18.7	8.29
G3V2K4	Brain-enriched guanylate kinase-associated protein (Fragment) OS=Homo sapiens GN=BEGAIN PE=4 SV=1 - (G3V2K4_HUMAN)	6.18	10.71	5	1	2	6	2.570E8	168	19.9	6.73
Q9Y4K1	Absent in melanoma 1 protein OS=Homo sapiens GN=AIM1 PE=1 SV=3 - (AIM1_HUMAN)	6.16	6.21	1	1	11	15	8.329E7	1723	188.6	5.86
A8MWV3	Osteoclast-associated immunoglobulin-like receptor OS=Homo sapiens GN=OSCAR PE=4 SV=1 - (A8MWV3_HUMAN)	6.14	34.78	1	1	4	10	2.458E7	161	16.4	12.04
G3V3A0	Alpha-1-antichymotrypsin OS=Homo sapiens GN=SERPINA3 PE=3 SV=1 - (G3V3A0_HUMAN)	6.12	9.76	4	2	2	2	2.912E7	205	23.4	5.64
Q53T70	Putative uncharacterized protein RAB10 (Fragment) OS=Homo sapiens GN=RAB10 PE=2 SV=1 - (Q53T70_HUMAN)	6.08	11.46	2	1	2	4	5.521E6	157	17.8	8.25
P09467	Fructose-1,6-bisphosphatase 1 OS=Homo sapiens GN=FBP1 PE=1 SV=5 - (F16P1_HUMAN)	6.01	7.69	2	1	2	2	9.362E6	338	36.8	6.99
A8K2E6	Hermansky-Pudlak syndrome 4, isoform CRA_b OS=Homo sapiens GN=HPS4 PE=2 SV=1 - (A8K2E6_HUMAN)	6.00	4.24	11	1	2	3	5.267E7	708	76.9	5.50
P62266	40S ribosomal protein S23 OS=Homo sapiens GN=RPS23 PE=1 SV=3 - (RS23_HUMAN)	5.98	16.08	2	2	2	2	2.119E7	143	15.8	10.49
E9PG49	Sodium channel protein type 1 subunit alpha OS=Homo sapiens GN=SCN1A PE=3 SV=1 - (E9PG49_HUMAN)	5.95	2.32	16	1	5	21	1.980E8	1981	226.0	5.92
P84090	Enhancer of rudimentary homolog OS=Homo sapiens GN=ERH PE=1 SV=1 - (ERH_HUMAN)	5.94	22.12	1	1	2	3	2.194E7	104	12.3	5.92
Q9H694	Protein bicaudal C homolog 1 OS=Homo sapiens GN=BICC1 PE=1 SV=2 - (BICC1_HUMAN)	5.91	8.32	1	1	7	11	4.101E7	974	104.8	8.54
Q9UKF2-2	Isoform Beta of Disintegrin and metalloproteinase domain-containing protein 30 OS=Homo sapiens GN=ADAM30 - (ADA30_HUMAN)	5.89	9.99	3	1	7	25	2.072E8	781	87.9	7.61
O15013-4	Isoform 4 of Rho guanine nucleotide exchange factor 10 OS=Homo sapiens GN=ARHGEF10 - (ARHGA_HUMAN)	5.87	3.43	4	1	5	6	3.994E7	1340	148.2	5.78
Q16176	Lsk protein OS=Homo sapiens GN=lsk PE=2 SV=1 - (Q16176_HUMAN)	5.85	2.37	1	1	1	3	4.421E7	465	51.9	8.51
P60953	Cell division control protein 42 homolog OS=Homo sapiens GN=CDC42 PE=1 SV=2 - (CDC42_HUMAN)	5.79	18.32	5	2	3	3	3.313E6	191	21.2	6.55
Q59H50	Integrin beta (Fragment) OS=Homo sapiens PE=1 SV=1 - (Q59H50_HUMAN)	5.79	9.68	1	1	5	9	2.364E7	475	52.4	6.54
Q08211	ATP-dependent RNA helicase A OS=Homo sapiens GN=DHX9 PE=1 SV=4 - (DHX9_HUMAN)	5.78	4.65	3	1	4	5	6.641E7	1270	140.9	6.84
Q9HA11	Histone H2A OS=Homo sapiens PE=2 SV=1 - (Q9HA11_HUMAN)	5.76	10.36	8	1	3	4	8.560E6	280	29.9	10.07
Q3LI83	Keratin-associated protein 24-1 OS=Homo sapiens GN=KRTAP24-1 PE=1 SV=1 - (KR241_HUMAN)	5.76	8.66	1	1	2	4	1.251E7	254	27.7	8.29
P11217-2	Isoform 2 of Glycogen phosphorylase, muscle form OS=Homo sapiens GN=PYGM - (PYGM_HUMAN)	5.74	6.37	19	2	6	8	3.574E7	754	87.3	8.41
A8K0T9	cDNA FLJ75422, highly similar to Homo sapiens capping protein (actin filament) muscle Z-line, alpha 1, mRNA OS=Homo sapiens PE=2 SV=1 - (A8K0T9_HUMAN)	5.72	10.84	7	2	3	3	2.254E7	286	32.9	5.69
Q9NRZ7-2	Isoform 2 of 1-acyl-sn-glycerol-3-phosphate acyltransferase gamma OS=Homo sapiens GN=AGPAT3 - (PLCC_HUMAN)	5.72	16.88	5	1	4	42	5.919E7	314	36.2	8.25
H0YKD8	60S ribosomal protein L28 OS=Homo sapiens GN=RPL28 PE=4 SV=1 - (H0YKD8_HUMAN)	5.70	27.06	4	1	4	5	4.558E7	170	19.1	11.46
Q13422-7	Isoform Ik7 of DNA-binding protein Ikaros OS=Homo sapiens GN=IKZF1 - (IKZF1_HUMAN)	5.66	18.45	16	1	8	8	3.734E8	477	52.7	6.29
Q5TG40	DNA methyltransferase 1 associated protein 1 (Fragment) OS=Homo sapiens GN=DMAP1 PE=2 SV=1 - (Q5TG40_HUMAN)	5.64	6.62	3	1	3	8	8.889E7	302	35.4	9.60
B3KN82	cDNA FLJ13913 fis, clone Y79AA1000231, highly similar to Nucleolar protein NOP5 OS=Homo sapiens PE=2 SV=1 - (B3KN82_HUMAN)	5.62	4.72	2	1	3	4	2.480E7	466	52.3	9.01
P49720	Proteasome subunit beta type-3 OS=Homo sapiens GN=PSMB3 PE=1 SV=2 - (PSB3_HUMAN)	5.61	16.10	1	2	3	3	7.628E6	205	22.9	6.55
P28062-2	Isoform 2 of Proteasome subunit beta type-8 OS=Homo sapiens GN=PSMB8 - (PSB8_HUMAN)	5.59	17.65	3	1	3	6	1.326E7	272	29.8	5.82
B2RBA0	cDNA, FLJ95388, highly similar to Homo sapiens step II splicing factor SLU7 (SLU7), mRNA OS=Homo sapiens PE=2 SV=1 - (B2RBA0_HUMAN)	5.51	10.24	4	1	6	12	2.796E9	586	68.3	7.14
F4MH93	Ubiquitously transcribed tetratricopeptide repeat protein Y-linked transcript variant 246 OS=Homo sapiens GN=UTY PE=2 SV=1 - (F4MH93_HUMAN)	5.46	3.94	3	1	2	7	8.178E7	482	54.9	6.99
P62318	Small nuclear ribonucleoprotein Sm D3 OS=Homo sapiens GN=SNRPD3 PE=1 SV=1 - (SMD3_HUMAN)	5.46	24.60	2	1	3	5	9.969E6	126	13.9	10.32
Q76N57	Ribosomal protein S26 (Fragment) OS=Homo sapiens PE=2 SV=1 - (Q76N57_HUMAN)	5.45	39.13	6	1	1	2	4.860E7	23	2.5	4.91
E9PQM0	Protein-tyrosine phosphatase mitochondrial 1 OS=Homo sapiens GN=PTPMT1 PE=4 SV=1 - (E9PQM0_HUMAN)	5.39	10.71	2	1	2	5	2.375E7	168	18.2	10.62
Q9P241	Probable phospholipid-transporting ATPase VD OS=Homo sapiens GN=ATP10D PE=2 SV=3 - (AT10D_HUMAN)	5.32	2.52	4	1	4	5	2.451E7	1426	160.2	7.15
Q4G134	NUDT13 protein OS=Homo sapiens GN=NUDT13 PE=2 SV=1 - (Q4G134_HUMAN)	5.32	8.30	4	1	3	8	1.006E8	241	27.1	8.65
O00299	Chloride intracellular channel protein 1 OS=Homo sapiens GN=CLIC1 PE=1 SV=4 - (CLIC1_HUMAN)	5.31	17.43	15	2	3	4	1.824E7	241	26.9	5.17
O15054	Lysine-specific demethylase 6B OS=Homo sapiens GN=KDM6B PE=1 SV=4 - (KDM6B_HUMAN)	5.28	3.16	3	1	6	23	4.023E7	1643	176.5	8.54
B4DLJ7	cDNA FLJ59334, highly similar to Transmembrane glycoprotein NMB OS=Homo sapiens PE=2 SV=1 - (B4DLJ7_HUMAN)	5.28	7.98	11	1	5	6	2.812E8	514	57.2	6.77
B2R5M1	cDNA, FLJ92527, highly similar to Homo sapiens cytochrome P450, family 17, subfamily A, polypeptide 1 (CYP17A1), mRNA OS=Homo sapiens PE=2 SV=1 - (B2R5M1_HUMAN)	5.28	8.46	1	1	3	7	1.515E7	508	57.3	8.70
Q8IYY4-2	Isoform 2 of Zinc finger protein DZIP1L OS=Homo sapiens GN=DZIP1L - (DZI1L_HUMAN)	5.26	15.21	3	1	6	10	4.561E7	539	62.4	7.37
P61254	60S ribosomal protein L26 OS=Homo sapiens GN=RPL26 PE=1 SV=1 - (RL26_HUMAN)	5.19	22.76	8	2	3	8	5.839E7	145	17.2	10.55
Q5SYZ4	Cellular retinoic acid binding protein 2 (Fragment) OS=Homo sapiens GN=CRABP2 PE=2 SV=1 - (Q5SYZ4_HUMAN)	5.14	29.27	2	2	3	3	1.888E7	82	9.2	5.45
B3KTF6	cDNA FLJ38174 fis, clone FCBBF1000061, highly similar to Antizyme inhibitor 1 OS=Homo sapiens PE=2 SV=1 - (B3KTF6_HUMAN)	5.12	4.13	5	1	2	16	2.320E8	363	39.8	5.27
Q9UPP2	IQ motif and SEC7 domain-containing protein 3 OS=Homo sapiens GN=IQSEC3 PE=2 SV=3 - (IQEC3_HUMAN)	5.09	3.47	11	1	5	7	1.531E7	1182	127.5	6.51
I3NI21	Promethin (Fragment) OS=Homo sapiens GN=TMEM159 PE=4 SV=1 - (I3NI21_HUMAN)	5.08	17.39	5	1	1	7	1.500E9	46	5.2	5.24
B3KQ30	Iron-sulfur cluster assembly enzyme ISCU, mitochondrial OS=Homo sapiens GN=ISCU PE=2 SV=1 - (B3KQ30_HUMAN)	5.07	19.23	5	1	3	55	4.129E8	156	16.9	9.64
B4DND6	cDNA FLJ55804, highly similar to COP9 signalosome complex subunit 1 OS=Homo sapiens PE=2 SV=1 - (B4DND6_HUMAN)	5.05	10.14	8	2	5	8	1.799E8	483	54.6	6.90
B7Z3I9	Delta-aminolevulinic acid dehydratase OS=Homo sapiens PE=2 SV=1 - (B7Z3I9_HUMAN)	4.94	12.78	5	2	3	3	2.174E7	313	34.5	7.20
Q6WWJ7	DELTA3PRO-IL-18 OS=Homo sapiens GN=IL18 PE=2 SV=1 - (Q6WWJ7_HUMAN)	4.86	17.99	4	1	3	4	1.228E7	189	21.9	4.78
Q9Y5Z0-4	Isoform 4 of Beta-secretase 2 OS=Homo sapiens GN=BACE2 - (BACE2_HUMAN)	4.83	2.51	1	1	1	12	1.027E8	439	48.2	4.97
P61077-3	Isoform 3 of Ubiquitin-conjugating enzyme E2 D3 OS=Homo sapiens GN=UBE2D3 - (UB2D3_HUMAN)	4.69	15.44	9	1	2	3	9.789E6	149	16.9	7.74
Q9P1F3	Costars family protein ABRACL OS=Homo sapiens GN=ABRACL PE=1 SV=1 - (ABRAL_HUMAN)	4.67	16.05	1	1	1	1	1.549E6	81	9.1	6.29
Q15102	Platelet-activating factor acetylhydrolase IB subunit gamma OS=Homo sapiens GN=PAFAH1B3 PE=1 SV=1 - (PA1B3_HUMAN)	4.65	7.36	1	2	2	2	4.593E6	231	25.7	6.84
B7Z1I2	Aspartate aminotransferase OS=Homo sapiens GN=GOT1 PE=2 SV=1 - (B7Z1I2_HUMAN)	4.65	12.02	4	1	5	6	5.971E7	366	41.0	7.18
H0YCY8	Dipeptidyl peptidase 1 light chain (Fragment) OS=Homo sapiens GN=CTSC PE=4 SV=1 - (H0YCY8_HUMAN)	4.62	7.35	4	1	2	2	2.008E7	245	27.9	9.16
Q96QA5	Gasdermin-A OS=Homo sapiens GN=GSDMA PE=2 SV=4 - (GSDMA_HUMAN)	4.61	9.44	1	1	4	9	9.503E7	445	49.3	5.29
B8X2Z3	CARMIL2b OS=Homo sapiens GN=RLTPR PE=2 SV=1 - (B8X2Z3_HUMAN)	4.56	1.60	4	1	2	5	9.686E7	1372	148.1	6.55
Q9NS37	CREB/ATF bZIP transcription factor OS=Homo sapiens GN=CREBZF PE=1 SV=2 - (ZHANG_HUMAN)	4.56	13.84	3	1	3	6	1.909E8	354	37.1	5.24
Q9BYR2	Keratin-associated protein 4-5 OS=Homo sapiens GN=KRTAP4-5 PE=2 SV=4 - (KRA45_HUMAN)	4.52	15.47	4	1	1	1	1.339E7	181	19.3	7.78
H7C4J1	Actin-related protein 3C (Fragment) OS=Homo sapiens GN=ACTR3C PE=4 SV=1 - (H7C4J1_HUMAN)	4.49	19.71	13	1	4	6	1.930E7	208	23.8	5.86
A2A3R5	40S ribosomal protein S6 OS=Homo sapiens GN=RPS6 PE=3 SV=1 - (A2A3R5_HUMAN)	4.49	4.59	3	1	1	2	4.456E6	218	25.0	11.14
P48739-2	Isoform 2 of Phosphatidylinositol transfer protein beta isoform OS=Homo sapiens GN=PITPNB - (PIPNB_HUMAN)	4.49	11.76	4	1	3	4	4.504E7	272	31.6	6.25
Q5HYM2	Putative uncharacterized protein DKFZp686O2462 (Fragment) OS=Homo sapiens GN=DKFZp686O2462 PE=2 SV=1 - (Q5HYM2_HUMAN)	4.44	9.27	4	1	5	9	3.480E7	626	72.0	5.36
A8K132	cDNA FLJ75476, highly similar to Homo sapiens glutaminase (GLS), mRNA OS=Homo sapiens PE=2 SV=1 - (A8K132_HUMAN)	4.42	3.74	7	1	3	4	2.855E7	669	73.5	7.77
H7C2W9	60S ribosomal protein L31 (Fragment) OS=Homo sapiens GN=RPL31 PE=4 SV=1 - (H7C2W9_HUMAN)	4.40	36.11	9	1	5	6	2.383E8	108	12.8	11.00
E9PLD0	Ras-related protein Rab-1B OS=Homo sapiens GN=RAB1B PE=3 SV=1 - (E9PLD0_HUMAN)	4.36	20.12	9	1	3	5	2.615E7	169	18.5	5.72
P49411	Elongation factor Tu, mitochondrial OS=Homo sapiens GN=TUFM PE=1 SV=2 - (EFTU_HUMAN)	4.32	4.87	1	1	2	3	1.277E7	452	49.5	7.61
B1ALC0	Actin-related protein 2/3 complex subunit 5 OS=Homo sapiens GN=ARPC5 PE=3 SV=1 - (B1ALC0_HUMAN)	4.30	14.07	4	1	2	5	6.970E7	135	14.8	5.06
B4E0W3	cDNA FLJ60386, moderately similar to Signal-induced proliferation-associated protein 1 OS=Homo sapiens PE=2 SV=1 - (B4E0W3_HUMAN)	4.30	6.70	4	1	5	5	5.377E7	940	101.7	6.48
Q0VD83-3	Isoform 3 of Apolipoprotein B receptor OS=Homo sapiens GN=APOBR - (APOBR_HUMAN)	4.27	9.64	5	1	10	14	7.845E7	1069	112.9	4.37
D6W5P0	Cyclin D binding myb-like transcription factor 1, isoform CRA_a OS=Homo sapiens GN=DMTF1 PE=4 SV=1 - (D6W5P0_HUMAN)	4.23	1.82	4	1	1	3	7.423E7	494	54.2	4.56
C9JZ20	Prohibitin (Fragment) OS=Homo sapiens GN=PHB PE=4 SV=1 - (C9JZ20_HUMAN)	4.22	10.45	10	1	2	2	3.178E7	201	22.3	5.96
H0YBX3	Ribonuclease UK114 (Fragment) OS=Homo sapiens GN=HRSP12 PE=4 SV=1 - (H0YBX3_HUMAN)	4.20	16.54	4	1	2	2	8.045E6	127	13.8	9.31
P42695	Condensin-2 complex subunit D3 OS=Homo sapiens GN=NCAPD3 PE=1 SV=2 - (CNDD3_HUMAN)	4.20	4.81	3	1	7	15	5.967E8	1498	168.8	7.50
P42766	60S ribosomal protein L35 OS=Homo sapiens GN=RPL35 PE=1 SV=2 - (RL35_HUMAN)	4.18	34.15	4	1	5	7	2.209E7	123	14.5	11.05
B3VRC5	5-hydroxytryptamine (Serotonin) receptor 2B (Fragment) OS=Homo sapiens GN=HTR2B PE=4 SV=1 - (B3VRC5_HUMAN)	4.18	17.23	2	1	5	12	1.157E8	296	33.6	9.52
Q5QNZ2	ATP synthase subunit b, mitochondrial OS=Homo sapiens GN=ATP5F1 PE=2 SV=1 - (Q5QNZ2_HUMAN)	4.14	8.21	4	2	2	2	4.276E6	195	22.3	9.26
H7BY24	Centrosomal protein of 55 kDa OS=Homo sapiens GN=CEP55 PE=4 SV=1 - (H7BY24_HUMAN)	4.13	5.01	4	1	3	5	3.444E7	399	46.7	7.88
O00151	PDZ and LIM domain protein 1 OS=Homo sapiens GN=PDLIM1 PE=1 SV=4 - (PDLI1_HUMAN)	4.11	6.38	1	2	2	2	3.941E6	329	36.0	7.02
Q9BT92	Trichoplein keratin filament-binding protein OS=Homo sapiens GN=TCHP PE=1 SV=1 - (TCHP_HUMAN)	4.06	12.65	1	1	6	11	6.318E7	498	61.0	6.54
Q9Y3D6	Mitochondrial fission 1 protein OS=Homo sapiens GN=FIS1 PE=1 SV=2 - (FIS1_HUMAN)	4.04	12.50	1	1	2	3	9.378E6	152	16.9	8.79
P49207	60S ribosomal protein L34 OS=Homo sapiens GN=RPL34 PE=1 SV=3 - (RL34_HUMAN)	4.04	16.24	2	2	3	3	7.474E6	117	13.3	11.47
Q8TCA0-3	Isoform 3 of Leucine-rich repeat-containing protein 20 OS=Homo sapiens GN=LRRC20 - (LRC20_HUMAN)	4.04	8.59	3	1	1	2	2.152E8	128	14.1	9.64
Q7Z5N2	Kangai 1 OS=Homo sapiens GN=KAI1 PE=2 SV=1 - (Q7Z5N2_HUMAN)	4.04	4.96	1	1	1	11	4.892E7	242	26.8	5.11
Q96KP4-2	Isoform 2 of Cytosolic non-specific dipeptidase OS=Homo sapiens GN=CNDP2 - (CNDP2_HUMAN)	4.03	13.04	6	2	4	5	1.087E7	391	43.8	6.48
E5RIW3	Tubulin-specific chaperone A OS=Homo sapiens GN=TBCA PE=4 SV=1 - (E5RIW3_HUMAN)	4.02	20.24	4	1	2	7	1.355E8	84	10.1	4.63
P63173	60S ribosomal protein L38 OS=Homo sapiens GN=RPL38 PE=1 SV=2 - (RL38_HUMAN)	4.02	18.57	1	1	1	1	1.893E7	70	8.2	10.10
Q59FS3	Ribosomal protein S7 variant (Fragment) OS=Homo sapiens PE=2 SV=1 - (Q59FS3_HUMAN)	4.00	28.21	3	1	1	1	9.008E6	78	8.9	8.57
H0YCI4	Nucleosome assembly protein 1-like 4 (Fragment) OS=Homo sapiens GN=NAP1L4 PE=3 SV=1 - (H0YCI4_HUMAN)	3.99	21.57	13	2	3	3	1.921E7	204	23.4	4.92
A2VDJ1	GOLGA6 protein (Fragment) OS=Homo sapiens GN=GOLGA6 PE=2 SV=1 - (A2VDJ1_HUMAN)	3.96	7.59	5	1	3	5	9.305E7	632	73.6	5.78
B7Z7G8	cDNA FLJ53530 OS=Homo sapiens PE=2 SV=1 - (B7Z7G8_HUMAN)	3.96	5.70	1	1	1	3	1.564E8	158	17.0	11.63
O95833	Chloride intracellular channel protein 3 OS=Homo sapiens GN=CLIC3 PE=1 SV=2 - (CLIC3_HUMAN)	3.92	11.86	1	2	3	3	7.182E6	236	26.6	6.43
A7E2S3	RPS3 protein (Fragment) OS=Homo sapiens GN=RPS3 PE=2 SV=1 - (A7E2S3_HUMAN)	3.91	12.03	9	1	2	2	1.172E8	133	14.7	10.26
C9JGI3	Thymidine phosphorylase (Fragment) OS=Homo sapiens GN=TYMP PE=4 SV=1 - (C9JGI3_HUMAN)	3.91	8.99	4	1	3	8	1.091E8	445	46.1	5.52
P62273	40S ribosomal protein S29 OS=Homo sapiens GN=RPS29 PE=1 SV=2 - (RS29_HUMAN)	3.88	39.29	2	2	3	4	9.240E6	56	6.7	10.13
P22314	Ubiquitin-like modifier-activating enzyme 1 OS=Homo sapiens GN=UBA1 PE=1 SV=3 - (UBA1_HUMAN)	3.88	3.21	5	1	3	4	5.266E7	1058	117.8	5.76
E5RJ48	Acyl-protein thioesterase 1 (Fragment) OS=Homo sapiens GN=LYPLA1 PE=4 SV=1 - (E5RJ48_HUMAN)	3.88	15.56	6	1	1	1	5.530E6	90	9.6	4.89
B4DUZ6	cDNA FLJ56608, highly similar to Homo sapiens chloride channel, calcium activated, family member 1 (CLCA1), mRNA OS=Homo sapiens PE=2 SV=1 - (B4DUZ6_HUMAN)	3.87	8.42	2	1	7	8	5.351E7	677	73.6	6.15
B4DFL3	cDNA FLJ56661, highly similar to Proteasome subunit beta type 4 (EC 3.4.25.1) OS=Homo sapiens PE=2 SV=1 - (B4DFL3_HUMAN)	3.85	21.56	2	1	3	4	1.177E8	167	18.3	9.35
B2R761	cDNA, FLJ93299, highly similar to Homo sapiens sterol carrier protein 2 (SCP2), mRNA OS=Homo sapiens PE=2 SV=1 - (B2R761_HUMAN)	3.81	5.67	10	1	4	5	1.599E8	547	59.0	7.05
H3BPQ4	Hydroxyacylglutathione hydrolase, mitochondrial OS=Homo sapiens GN=HAGH PE=3 SV=1 - (H3BPQ4_HUMAN)	3.80	12.02	5	1	3	3	1.152E7	258	27.9	7.28
A8MUX0	Putative keratin-associated protein 10-like ENSP00000375147 OS=Homo sapiens PE=3 SV=1 - (KR10D_HUMAN)	3.80	2.90	1	1	1	1	4.709E6	517	53.9	5.39
B7Z2R9	Lysosome-associated membrane glycoprotein 2 OS=Homo sapiens GN=LAMP2 PE=2 SV=1 - (B7Z2R9_HUMAN)	3.80	2.67	5	1	1	2	4.436E6	300	33.0	6.87
Q6ZUA9	cDNA FLJ43860 fis, clone TESTI4007404 OS=Homo sapiens PE=2 SV=1 - (Q6ZUA9_HUMAN)	3.79	7.66	2	1	6	12	1.803E8	1318	149.0	7.58
Q5T8U3	60S ribosomal protein L7a (Fragment) OS=Homo sapiens GN=RPL7A PE=4 SV=1 - (Q5T8U3_HUMAN)	3.74	15.71	2	1	3	3	9.454E6	191	21.5	11.02
P84103	Serine/arginine-rich splicing factor 3 OS=Homo sapiens GN=SRSF3 PE=1 SV=1 - (SRSF3_HUMAN)	3.73	37.20	2	1	5	9	2.807E7	164	19.3	11.65
Q9NQR4	Omega-amidase NIT2 OS=Homo sapiens GN=NIT2 PE=1 SV=1 - (NIT2_HUMAN)	3.71	6.16	2	1	2	2	3.339E6	276	30.6	7.21
P28072	Proteasome subunit beta type-6 OS=Homo sapiens GN=PSMB6 PE=1 SV=4 - (PSB6_HUMAN)	3.70	13.39	2	1	2	4	7.093E7	239	25.3	4.92
E9PJP5	Junctional adhesion molecule B OS=Homo sapiens GN=JAM2 PE=4 SV=1 - (E9PJP5_HUMAN)	3.69	15.28	7	1	6	9	2.242E7	301	33.7	9.28
P52565	Rho GDP-dissociation inhibitor 1 OS=Homo sapiens GN=ARHGDIA PE=1 SV=3 - (GDIR1_HUMAN)	3.69	15.69	1	1	3	5	2.218E7	204	23.2	5.11
F8W9N0	Tubulin polyglutamylase TTLL6 OS=Homo sapiens GN=TTLL6 PE=4 SV=1 - (F8W9N0_HUMAN)	3.65	19.71	5	1	3	8	4.771E7	208	24.3	6.23
B4DII5	Importin subunit alpha OS=Homo sapiens PE=2 SV=1 - (B4DII5_HUMAN)	3.65	4.69	5	1	3	5	4.792E6	533	59.6	5.01
B2RBF9	cDNA, FLJ95487, highly similar to Homo sapiens peptidyl arginine deiminase, type I (PADI1), mRNA OS=Homo sapiens PE=2 SV=1 - (B2RBF9_HUMAN)	3.64	3.92	3	1	3	8	1.457E8	663	74.6	6.49
O00515	Ladinin-1 OS=Homo sapiens GN=LAD1 PE=1 SV=2 - (LAD1_HUMAN)	3.64	13.73	5	1	6	6	1.370E8	517	57.1	9.67
Q8NCA9	Zinc finger protein 784 OS=Homo sapiens GN=ZNF784 PE=2 SV=1 - (ZN784_HUMAN)	3.62	10.84	1	1	3	6	2.171E8	323	34.2	7.80
Q53H42	Syndecan 1 variant (Fragment) OS=Homo sapiens PE=2 SV=1 - (Q53H42_HUMAN)	3.61	16.15	5	1	2	4	1.762E7	161	16.9	4.58
E9PBH2	Leucine-rich repeat-containing protein 34 OS=Homo sapiens GN=LRRC34 PE=4 SV=1 - (E9PBH2_HUMAN)	3.57	2.31	3	1	1	4	4.314E5	432	47.7	6.65
B7Z478	Proteasome (Prosome, macropain) subunit, beta type, 2, isoform CRA_b OS=Homo sapiens GN=PSMB2 PE=2 SV=1 - (B7Z478_HUMAN)	3.54	11.93	2	1	2	2	6.404E7	176	20.2	7.44
Q8IWV2-3	Isoform 3 of Contactin-4 OS=Homo sapiens GN=CNTN4 - (CNTN4_HUMAN)	3.51	3.80	1	1	4	7	2.038E7	1025	113.3	7.47
A8K1I4	cDNA FLJ78426 OS=Homo sapiens PE=2 SV=1 - (A8K1I4_HUMAN)	3.47	4.52	5	1	3	9	1.475E8	619	67.8	8.82
Q96L46	Calpain small subunit 2 OS=Homo sapiens GN=CAPNS2 PE=2 SV=2 - (CPNS2_HUMAN)	3.46	6.45	1	1	1	1	4.116E6	248	27.6	5.73
B4E2Q6	Regulation of nuclear pre-mRNA domain-containing protein 2 OS=Homo sapiens GN=RPRD2 PE=2 SV=1 - (B4E2Q6_HUMAN)	3.44	2.14	2	1	2	3	2.450E7	934	100.2	6.48
Q9UJW2-2	Isoform 2 of Tubulointerstitial nephritis antigen OS=Homo sapiens GN=TINAG - (TINAG_HUMAN)	3.38	9.31	4	1	3	14	8.072E7	333	38.7	7.69
D0EZN6	Cytochrome p450 (Fragment) OS=Homo sapiens GN=CYP1B1 PE=4 SV=1 - (D0EZN6_HUMAN)	3.38	25.00	1	1	1	2	2.072E7	40	4.8	5.19
Q9Y520-6	Isoform 6 of Protein PRRC2C OS=Homo sapiens GN=PRRC2C - (PRC2C_HUMAN)	3.38	4.03	10	1	11	46	1.309E8	2752	301.4	9.10
Q8TE68-2	Isoform 2 of Epidermal growth factor receptor kinase substrate 8-like protein 1 OS=Homo sapiens GN=EPS8L1 - (ES8L1_HUMAN)	3.33	7.21	7	1	4	5	2.500E7	596	66.4	6.24
P62888	60S ribosomal protein L30 OS=Homo sapiens GN=RPL30 PE=1 SV=2 - (RL30_HUMAN)	3.33	26.96	2	1	3	5	9.409E7	115	12.8	9.63
F8VQQ8	Ubiquitin-conjugating enzyme E2 N OS=Homo sapiens GN=UBE2N PE=3 SV=1 - (F8VQQ8_HUMAN)	3.30	20.22	5	2	2	2	1.051E7	89	10.1	8.79
O60518	Ran-binding protein 6 OS=Homo sapiens GN=RANBP6 PE=1 SV=2 - (RNBP6_HUMAN)	3.30	4.62	3	1	5	11	2.009E8	1105	124.6	5.01
B7Z2Z8	T-complex protein 1 subunit delta OS=Homo sapiens PE=2 SV=1 - (B7Z2Z8_HUMAN)	3.28	14.70	7	1	4	4	7.012E7	483	52.3	6.57
H0YFX4	4F2 cell-surface antigen heavy chain (Fragment) OS=Homo sapiens GN=SLC3A2 PE=4 SV=1 - (H0YFX4_HUMAN)	3.28	9.84	7	1	1	1	2.442E6	122	13.3	5.26
C9J6I7	UDP-GalNAc:beta-1,3-N-acetylgalactosaminyltransferase 1 (Fragment) OS=Homo sapiens GN=B3GALNT1 PE=4 SV=1 - (C9J6I7_HUMAN)	3.23	4.67	1	1	1	8	4.970E8	150	17.1	10.36
Q53RT3	Retroviral-like aspartic protease 1 OS=Homo sapiens GN=ASPRV1 PE=1 SV=1 - (APRV1_HUMAN)	3.22	3.79	1	1	1	1	1.531E7	343	37.0	5.44
Q99714	3-hydroxyacyl-CoA dehydrogenase type-2 OS=Homo sapiens GN=HSD17B10 PE=1 SV=3 - (HCD2_HUMAN)	3.15	14.94	2	2	3	5	2.373E7	261	26.9	7.78
Q86VP6-2	Isoform 2 of Cullin-associated NEDD8-dissociated protein 1 OS=Homo sapiens GN=CAND1 - (CAND1_HUMAN)	3.10	3.20	4	1	3	6	1.167E8	1062	117.8	5.69
Q59GW1	High-mobility group box 1 variant (Fragment) OS=Homo sapiens PE=2 SV=1 - (Q59GW1_HUMAN)	3.09	18.75	8	1	3	4	2.100E7	176	20.2	9.74
P68036	Ubiquitin-conjugating enzyme E2 L3 OS=Homo sapiens GN=UBE2L3 PE=1 SV=1 - (UB2L3_HUMAN)	3.01	39.61	6	1	4	7	2.817E7	154	17.9	8.51
B4DLC0	Poly(rC)-binding protein 2 OS=Homo sapiens GN=PCBP2 PE=2 SV=1 - (B4DLC0_HUMAN)	2.94	11.30	15	1	3	3	2.208E8	301	32.1	7.90
F8VVM2	Phosphate carrier protein, mitochondrial OS=Homo sapiens GN=SLC25A3 PE=3 SV=1 - (F8VVM2_HUMAN)	2.88	8.64	6	1	3	4	3.103E7	324	36.1	9.26
E9PH29	Thioredoxin-dependent peroxide reductase, mitochondrial OS=Homo sapiens GN=PRDX3 PE=4 SV=1 - (E9PH29_HUMAN)	2.86	5.04	3	1	1	1	1.297E6	238	25.8	7.46
Q5JW98	Protein FAM26D OS=Homo sapiens GN=FAM26D PE=1 SV=1 - (FA26D_HUMAN)	2.84	13.38	2	1	3	5	6.028E7	314	35.0	6.87
P25398	40S ribosomal protein S12 OS=Homo sapiens GN=RPS12 PE=1 SV=3 - (RS12_HUMAN)	2.82	18.94	1	1	2	3	1.661E7	132	14.5	7.21
P62304	Small nuclear ribonucleoprotein E OS=Homo sapiens GN=SNRPE PE=1 SV=1 - (RUXE_HUMAN)	2.78	11.96	1	1	1	1	5.285E6	92	10.8	9.44
G3V5M2	Purine nucleoside phosphorylase (Fragment) OS=Homo sapiens GN=PNP PE=4 SV=1 - (G3V5M2_HUMAN)	2.67	6.79	4	1	2	18	1.550E7	221	24.5	9.51
Q99497	Protein DJ-1 OS=Homo sapiens GN=PARK7 PE=1 SV=2 - (PARK7_HUMAN)	2.67	20.11	1	2	3	3	1.309E7	189	19.9	6.79
O95336	6-phosphogluconolactonase OS=Homo sapiens GN=PGLS PE=1 SV=2 - (6PGL_HUMAN)	2.65	8.14	1	1	2	2	7.282E6	258	27.5	6.05
A8K0D2	cDNA FLJ77740, highly similar to Homo sapiens 7-dehydrocholesterol reductase, mRNA OS=Homo sapiens PE=2 SV=1 - (A8K0D2_HUMAN)	2.65	3.58	3	1	2	2	7.288E6	475	54.4	8.78
B7ZKY6	Membrane metallo-endopeptidase OS=Homo sapiens GN=MME PE=2 SV=1 - (B7ZKY6_HUMAN)	2.63	2.40	2	1	1	1	1.064E6	750	85.5	5.91
B4DNP0	cDNA FLJ59362, highly similar to Signal transducer and activator of transcription 3 OS=Homo sapiens PE=2 SV=1 - (B4DNP0_HUMAN)	2.63	9.38	7	1	4	27	6.064E7	672	76.1	6.30
C9JFR7	Cytochrome c (Fragment) OS=Homo sapiens GN=CYCS PE=3 SV=1 - (C9JFR7_HUMAN)	2.59	18.81	3	2	2	2	7.880E6	101	11.3	9.66
P06703	Protein S100-A6 OS=Homo sapiens GN=S100A6 PE=1 SV=1 - (S10A6_HUMAN)	2.57	16.67	2	2	2	2	5.821E7	90	10.2	5.48
F8WAS3	NADH dehydrogenase (ubiquinone) 1 alpha subcomplex subunit 5 OS=Homo sapiens GN=NDUFA5 PE=4 SV=1 - (F8WAS3_HUMAN)	2.53	14.29	5	1	1	1	1.115E6	70	7.8	9.44
Q8IX95	Putative cutaneous T-cell lymphoma-associated antigen 3 OS=Homo sapiens GN=CTAGE3P PE=5 SV=1 - (CTGE3_HUMAN)	2.51	15.82	1	1	1	5	4.472E7	158	18.0	4.92
H7C1C6	Translocon-associated protein subunit delta (Fragment) OS=Homo sapiens GN=SSR4 PE=4 SV=1 - (H7C1C6_HUMAN)	2.48	24.17	3	1	2	2	3.018E6	120	13.1	7.50
B2R983	cDNA, FLJ94267, highly similar to Homo sapiens glutathione S-transferase omega 1 (GSTO1), mRNA OS=Homo sapiens PE=2 SV=1 - (B2R983_HUMAN)	2.48	7.05	4	1	2	3	1.205E7	241	27.5	6.60
B7Z254	Protein disulfide-isomerase A6 OS=Homo sapiens GN=PDIA6 PE=2 SV=1 - (B7Z254_HUMAN)	2.48	6.18	7	1	3	4	1.853E8	437	47.8	5.08
Q04941	Proteolipid protein 2 OS=Homo sapiens GN=PLP2 PE=1 SV=1 - (PLP2_HUMAN)	2.45	8.55	1	1	1	1	2.820E7	152	16.7	7.24
Q8NFI4	Putative protein FAM10A5 OS=Homo sapiens GN=ST13P5 PE=5 SV=1 - (F10A5_HUMAN)	2.41	8.67	11	1	4	5	1.354E7	369	41.4	5.05
P51801	Chloride channel protein ClC-Kb OS=Homo sapiens GN=CLCNKB PE=1 SV=3 - (CLCKB_HUMAN)	2.41	8.44	14	1	5	7	5.843E8	687	75.4	8.00
C9IZG4	Protein CutA OS=Homo sapiens GN=CUTA PE=4 SV=1 - (C9IZG4_HUMAN)	2.41	14.81	4	1	2	3	6.188E6	135	14.4	5.45
E9PJU8	Ester hydrolase C11orf54 (Fragment) OS=Homo sapiens GN=C11orf54 PE=4 SV=1 - (E9PJU8_HUMAN)	2.37	6.42	7	1	1	1	5.279E6	187	21.1	6.73
Q9NTX5-6	Isoform 6 of Ethylmalonyl-CoA decarboxylase OS=Homo sapiens GN=ECHDC1 - (ECHD1_HUMAN)	2.34	13.03	7	1	4	12	3.060E7	284	31.1	8.85
H0YDQ0	FYVE, RhoGEF and PH domain-containing protein 4 (Fragment) OS=Homo sapiens GN=FGD4 PE=4 SV=1 - (H0YDQ0_HUMAN)	2.34	18.46	2	1	1	1	4.200E7	65	7.8	4.88
B3KXK4	cDNA FLJ45605 fis, clone BRTHA3021971, highly similar to Nuclear receptor coactivator 7 OS=Homo sapiens PE=2 SV=1 - (B3KXK4_HUMAN)	2.32	5.59	12	1	5	5	6.787E7	931	104.8	5.55
H0YHG0	Uncharacterized protein (Fragment) OS=Homo sapiens PE=4 SV=1 - (H0YHG0_HUMAN)	2.31	9.37	5	1	5	31	1.642E8	523	59.1	8.59
Q8N5Z0-2	Isoform 2 of Kynurenine/alpha-aminoadipate aminotransferase, mitochondrial OS=Homo sapiens GN=AADAT - (AADAT_HUMAN)	2.30	5.59	2	1	2	3	9.616E6	429	47.8	7.21
B4E2V4	Proteasome (Prosome, macropain) subunit, alpha type, 5, isoform CRA_c OS=Homo sapiens GN=PSMA5 PE=2 SV=1 - (B4E2V4_HUMAN)	2.30	3.83	3	1	1	1	1.174E7	183	20.0	4.73
G5EA35	Caspase recruitment domain-containing protein 18 OS=Homo sapiens GN=CARD18 PE=4 SV=1 - (G5EA35_HUMAN)	2.29	17.65	2	1	1	1	4.331E6	51	5.7	7.88
Q53SX6	Putative uncharacterized protein KYNU (Fragment) OS=Homo sapiens GN=KYNU PE=2 SV=1 - (Q53SX6_HUMAN)	2.22	9.30	6	1	2	3	2.040E7	301	33.8	5.85
P30086	Phosphatidylethanolamine-binding protein 1 OS=Homo sapiens GN=PEBP1 PE=1 SV=3 - (PEBP1_HUMAN)	2.20	12.30	2	1	2	3	9.957E7	187	21.0	7.53
C9J592	Ras-related protein Rab-7a (Fragment) OS=Homo sapiens GN=RAB7A PE=3 SV=1 - (C9J592_HUMAN)	2.13	8.67	2	1	1	1		150	17.0	8.85
C9JR72	Kelch repeat and BTB domain-containing protein 13 OS=Homo sapiens GN=KBTBD13 PE=1 SV=1 - (KBTBD_HUMAN)	2.13	4.80	1	1	2	5	8.561E7	458	49.5	5.71
H0YE60	UPF0722 protein C11orf88 (Fragment) OS=Homo sapiens GN=C11orf88 PE=4 SV=1 - (H0YE60_HUMAN)	2.13	79.31	1	1	2	75	7.288E8	29	3.4	6.55
C9J0K6	Sorcin OS=Homo sapiens GN=SRI PE=4 SV=1 - (C9J0K6_HUMAN)	2.11	7.10	4	1	1	1	2.762E6	155	17.6	5.60
Q6UXN8	C-type lectin domain family 9 member A OS=Homo sapiens GN=CLEC9A PE=1 SV=1 - (CLC9A_HUMAN)	2.10	6.64	1	1	2	3	7.281E8	241	27.3	6.83
Q96IV0-5	Isoform 5 of Peptide-N(4)-(N-acetyl-beta-glucosaminyl)asparagine amidase OS=Homo sapiens GN=NGLY1 - (NGLY1_HUMAN)	2.09	6.05	4	1	5	6	5.237E7	612	70.2	7.27
Q8WU49	Uncharacterized protein C7orf33 OS=Homo sapiens GN=C7orf33 PE=2 SV=1 - (CG033_HUMAN)	2.08	10.73	1	1	2	6	3.633E6	177	19.5	10.24
Q9Y3U8	60S ribosomal protein L36 OS=Homo sapiens GN=RPL36 PE=1 SV=3 - (RL36_HUMAN)	2.07	23.81	1	1	3	3	8.057E6	105	12.2	11.59
Q9BRA2	Thioredoxin domain-containing protein 17 OS=Homo sapiens GN=TXNDC17 PE=1 SV=1 - (TXD17_HUMAN)	2.07	13.82	3	1	2	4	2.967E6	123	13.9	5.52
Q2YDC2	HCG1985580, isoform CRA_f OS=Homo sapiens GN=PDCD6 PE=2 SV=1 - (Q2YDC2_HUMAN)	2.06	9.52	4	1	2	2	8.481E6	189	21.7	5.40
Q6WQI6	Putative cancer susceptibility gene HEPN1 protein OS=Homo sapiens GN=HEPN1 PE=5 SV=2 - (HEPN1_HUMAN)	2.04	13.64	1	1	2	4	8.296E7	88	10.3	8.28
H0YCU9	Transgelin (Fragment) OS=Homo sapiens GN=TAGLN PE=4 SV=1 - (H0YCU9_HUMAN)	2.04	15.23	5	1	2	2	6.642E6	151	16.8	9.36
B2R8A2	cDNA, FLJ93804, highly similar to Homo sapiens gp25L2 protein (HSGP25L2G), mRNA OS=Homo sapiens PE=2 SV=1 - (B2R8A2_HUMAN)	2.03	4.21	2	1	1	1	4.450E6	214	25.1	7.18
A8K0H3	cDNA FLJ78093, highly similar to Homo sapiens ribosomal protein L29 (RPL29), mRNA OS=Homo sapiens PE=2 SV=1 - (A8K0H3_HUMAN)	2.03	13.21	3	1	2	3	5.311E6	159	17.8	11.74
I3L1R8	Trafficking protein particle complex subunit 1 OS=Homo sapiens GN=TRAPPC1 PE=4 SV=1 - (I3L1R8_HUMAN)	2.03	42.86	1	1	2	7	3.317E8	42	5.0	7.44
P51572	B-cell receptor-associated protein 31 OS=Homo sapiens GN=BCAP31 PE=1 SV=3 - (BAP31_HUMAN)	2.01	14.63	9	1	4	4	8.380E6	246	28.0	8.44
Q53FH6	Mitogen-activated protein kinase kinase 1 interacting protein 1 variant (Fragment) OS=Homo sapiens PE=2 SV=1 - (Q53FH6_HUMAN)	2.01	8.06	2	1	1	1	1.396E6	124	13.6	7.34
O75051	Plexin-A2 OS=Homo sapiens GN=PLXNA2 PE=1 SV=4 - (PLXA2_HUMAN)	1.99	1.37	2	1	3	5	1.118E8	1894	211.0	6.48
P51814-4	Isoform 4 of Zinc finger protein 41 OS=Homo sapiens GN=ZNF41 - (ZNF41_HUMAN)	1.99	7.94	9	1	4	4	9.940E6	693	79.5	8.94
O00522-3	Isoform 3 of Krev interaction trapped protein 1 OS=Homo sapiens GN=KRIT1 - (KRIT1_HUMAN)	1.98	4.94	3	1	4	6	2.220E7	688	78.6	8.84
H0YJM8	Proteasome subunit beta type-5 (Fragment) OS=Homo sapiens GN=PSMB5 PE=3 SV=1 - (H0YJM8_HUMAN)	1.97	8.47	3	1	1	1	8.827E5	118	12.8	9.57
P62280	40S ribosomal protein S11 OS=Homo sapiens GN=RPS11 PE=1 SV=3 - (RS11_HUMAN)	1.95	15.19	1	1	3	4	3.585E7	158	18.4	10.30
F8W7F1	p53 apoptosis effector-related to PMP-22 OS=Homo sapiens GN=PERP PE=4 SV=1 - (F8W7F1_HUMAN)	1.95	4.00	4	1	1	1	1.178E7	175	19.5	7.03
P61106	Ras-related protein Rab-14 OS=Homo sapiens GN=RAB14 PE=1 SV=4 - (RAB14_HUMAN)	1.94	4.19	1	1	1	2	1.720E7	215	23.9	6.21
Q9UJA3-2	Isoform 2 of DNA replication licensing factor MCM8 OS=Homo sapiens GN=MCM8 - (MCM8_HUMAN)	1.91	7.19	6	1	7	10	1.343E8	793	88.7	7.49
Q8IV16	Glycosylphosphatidylinositol-anchored high density lipoprotein-binding protein 1 OS=Homo sapiens GN=GPIHBP1 PE=1 SV=2 - (HDBP1_HUMAN)	1.90	5.98	1	1	2	2	3.895E7	184	19.8	4.79
H0YJA0	Putative E3 ubiquitin-protein ligase UBR7 (Fragment) OS=Homo sapiens GN=UBR7 PE=4 SV=1 - (H0YJA0_HUMAN)	1.89	6.10	1	1	1	1		164	18.0	4.64
F8W7I0	2',3'-cyclic-nucleotide 3'-phosphodiesterase OS=Homo sapiens GN=CNP PE=4 SV=1 - (F8W7I0_HUMAN)	1.85	13.47	4	1	4	6	6.612E7	297	32.7	9.44
Q14427	GM2-activator protein (Fragment) OS=Homo sapiens PE=2 SV=1 - (Q14427_HUMAN)	1.83	7.77	5	1	1	1	2.637E6	103	11.1	4.75
E7EX53	Ribosomal protein L15 (Fragment) OS=Homo sapiens GN=RPL15 PE=3 SV=1 - (E7EX53_HUMAN)	1.82	6.77	5	1	1	1	3.834E6	133	15.7	11.00
H0Y9B1	Interleukin-6 receptor subunit alpha (Fragment) OS=Homo sapiens GN=IL6R PE=4 SV=1 - (H0Y9B1_HUMAN)	1.82	2.38	3	1	1	1	5.605E6	294	33.0	6.86
H0Y9T5	m7GpppN-mRNA hydrolase (Fragment) OS=Homo sapiens GN=DCP2 PE=3 SV=1 - (H0Y9T5_HUMAN)	1.81	6.47	3	1	3	7	4.809E7	402	44.9	9.47
O15482	Testis-specific protein TEX28 OS=Homo sapiens GN=TEX28 PE=2 SV=1 - (TEX28_HUMAN)	1.81	5.37	1	1	2	2	4.638E7	410	46.1	6.24
D0QYQ8	MYB/NFIB fusion protein variant 1 (Fragment) OS=Homo sapiens GN=MYB/NFIB fusion PE=2 SV=1 - (D0QYQ8_HUMAN)	1.80	3.15	1	1	2	2	2.006E7	603	67.7	6.84
Q9Y3R5-2	Isoform 2 of Protein dopey-2 OS=Homo sapiens GN=DOPEY2 - (DOP2_HUMAN)	1.79	2.84	2	1	6	43	5.024E7	2291	257.2	6.40
A9XAC0	Alpha-helix coiled-coil rod homologue (Fragment) OS=Homo sapiens GN=HCR PE=4 SV=1 - (A9XAC0_HUMAN)	1.78	27.36	68	1	3	3	6.798E7	106	12.0	5.15
F5H4D0	Protein CLEC16A OS=Homo sapiens GN=CLEC16A PE=4 SV=1 - (F5H4D0_HUMAN)	1.78	5.22	2	1	4	13	8.039E7	1053	117.6	5.86
H3BNI0	Zinc finger protein 19 OS=Homo sapiens GN=ZNF19 PE=4 SV=1 - (H3BNI0_HUMAN)	1.76	2.40	3	1	1	2	2.102E7	416	47.6	7.80
Q5H9S0	Putative uncharacterized protein DKFZp781N1974 OS=Homo sapiens GN=DKFZp781N1974 PE=2 SV=1 - (Q5H9S0_HUMAN)	1.74	4.46	2	1	3	4	2.611E7	763	84.5	9.13
I3L3P7	40S ribosomal protein S15a OS=Homo sapiens GN=RPS15A PE=4 SV=1 - (I3L3P7_HUMAN)	1.74	39.00	4	1	3	4	5.652E7	100	11.5	10.15
Q86VY2	Copine-8 OS=Homo sapiens GN=CPNE8 PE=2 SV=1 - (Q86VY2_HUMAN)	1.72	3.43	3	1	1	2	1.551E7	233	25.6	5.62
Q7L8L6	FAST kinase domain-containing protein 5 OS=Homo sapiens GN=FASTKD5 PE=1 SV=1 - (FAKD5_HUMAN)	1.71	4.58	1	1	4	14	6.238E7	764	86.5	8.13
P22460	Potassium voltage-gated channel subfamily A member 5 OS=Homo sapiens GN=KCNA5 PE=1 SV=4 - (KCNA5_HUMAN)	1.70	4.40	2	1	2	3	1.360E7	613	67.2	5.95
Q6PJI0	Potassium intermediate/small conductance calcium-activated channel, subfamily N, member 2 OS=Homo sapiens GN=KCNN2 PE=2 SV=1 - (Q6PJI0_HUMAN)	1.69	14.72	3	1	3	4	1.524E6	231	26.3	9.76
Q9UNN8	Endothelial protein C receptor OS=Homo sapiens GN=PROCR PE=1 SV=1 - (EPCR_HUMAN)	1.69	3.78	1	1	1	1	2.723E6	238	26.7	7.18
Q6IPX5	Ribosomal protein S2 OS=Homo sapiens GN=RPS2 PE=2 SV=1 - (Q6IPX5_HUMAN)	1.69	21.50	10	1	6	8	1.418E8	293	31.3	10.24
Q07DS4	Envelope glycoprotein OS=Homo sapiens GN=env PE=4 SV=1 - (Q07DS4_HUMAN)	1.69	3.65	1	1	3	8	4.766E7	438	49.6	8.50
Q9Y2S1	Galanin-related peptide OS=Homo sapiens PE=4 SV=1 - (Q9Y2S1_HUMAN)	1.68	9.43	1	1	1	27	7.074E8	106	11.8	5.97
Q53HD3	60S ribosomal protein L18a (Fragment) OS=Homo sapiens PE=2 SV=1 - (Q53HD3_HUMAN)	1.67	21.59	7	1	3	3	4.089E7	176	20.7	10.71
Q9H869	YY1-associated protein 1 OS=Homo sapiens GN=YY1AP1 PE=1 SV=2 - (YYAP1_HUMAN)	1.66	7.04	2	1	5	11	2.179E7	796	87.9	7.84
Q04760-2	Isoform 2 of Lactoylglutathione lyase OS=Homo sapiens GN=GLO1 - (LGUL_HUMAN)	1.66	10.06	2	1	2	3	1.360E7	169	19.0	6.05
H7C2D4	Nuclear receptor corepressor 2 (Fragment) OS=Homo sapiens GN=NCOR2 PE=4 SV=1 - (H7C2D4_HUMAN)	1.65	11.58	1	1	3	10	8.126E7	311	33.0	7.56
P04632	Calpain small subunit 1 OS=Homo sapiens GN=CAPNS1 PE=1 SV=1 - (CPNS1_HUMAN)	1.65	3.36	1	1	1	1	6.673E5	268	28.3	5.20
D7PBN3	ESRP1/RAF1 fusion protein OS=Homo sapiens PE=2 SV=1 - (D7PBN3_HUMAN)	1.65	4.90	14	1	6	6	2.192E8	1061	118.5	8.22
Q3LTJ6	MHC class II antigen (Fragment) OS=Homo sapiens GN=HLA-DRB1 PE=4 SV=1 - (Q3LTJ6_HUMAN)	1.63	35.62	34	1	2	6	3.353E7	73	8.6	5.80
P62316	Small nuclear ribonucleoprotein Sm D2 OS=Homo sapiens GN=SNRPD2 PE=1 SV=1 - (SMD2_HUMAN)	1.63	11.86	1	1	2	3	3.299E6	118	13.5	9.91
P25786-2	Isoform Long of Proteasome subunit alpha type-1 OS=Homo sapiens GN=PSMA1 - (PSA1_HUMAN)	1.63	13.38	5	2	3	3	3.220E7	269	30.2	6.99
A6NHJ4	Zinc finger protein 860 OS=Homo sapiens GN=ZNF860 PE=2 SV=3 - (ZN860_HUMAN)	1.62	9.02	1	1	5	6	2.750E7	632	73.7	9.33
Q5T3W0	Absent in melanoma 2 (Fragment) OS=Homo sapiens GN=AIM2 PE=2 SV=1 - (Q5T3W0_HUMAN)	1.61	17.37	1	1	2	5	2.976E7	167	19.1	9.51
E5RGF0	Gamma-glutamyl hydrolase (Fragment) OS=Homo sapiens GN=GGH PE=4 SV=1 - (E5RGF0_HUMAN)	1.60	4.91	4	1	1	1	3.477E6	163	18.4	7.24
P24588	A-kinase anchor protein 5 OS=Homo sapiens GN=AKAP5 PE=1 SV=3 - (AKAP5_HUMAN)	0.00	9.37	2	1	3	11	7.458E7	427	47.1	4.93
Q8N8A2	Serine/threonine-protein phosphatase 6 regulatory ankyrin repeat subunit B OS=Homo sapiens GN=ANKRD44 PE=1 SV=3 - (ANR44_HUMAN)	0.00	3.22	10	1	2	2	2.941E7	993	107.5	6.30
Q9H246	Uncharacterized protein C1orf21 OS=Homo sapiens GN=C1orf21 PE=1 SV=1 - (CA021_HUMAN)	0.00	23.14	1	1	3	7	3.718E8	121	13.9	5.22
O00548	Delta-like protein 1 OS=Homo sapiens GN=DLL1 PE=2 SV=2 - (DLL1_HUMAN)	0.00	4.15	1	1	2	9	1.944E7	723	78.0	6.24
P54851	Epithelial membrane protein 2 OS=Homo sapiens GN=EMP2 PE=2 SV=1 - (EMP2_HUMAN)	0.00	3.59	1	1	1	4	9.524E6	167	19.2	7.62
Q9HAU6	Putative apoptosis inhibitor FKSG2 OS=Homo sapiens GN=FKSG2 PE=5 SV=1 - (FKSG2_HUMAN)	0.00	12.72	1	1	2	2	6.907E7	173	20.3	5.05
Q9NQE9	Histidine triad nucleotide-binding protein 3 OS=Homo sapiens GN=HINT3 PE=1 SV=1 - (HINT3_HUMAN)	0.00	4.95	1	1	1	6	1.769E7	182	20.3	6.60
Q4G112	Heat shock factor protein 5 OS=Homo sapiens GN=HSF5 PE=2 SV=2 - (HSF5_HUMAN)	0.00	3.86	1	1	2	2	4.970E7	596	65.2	7.23
Q5JT82	Krueppel-like factor 17 OS=Homo sapiens GN=KLF17 PE=1 SV=1 - (KLF17_HUMAN)	0.00	4.63	1	1	1	1	5.719E6	389	42.6	6.74
Q8TE12	LIM homeobox transcription factor 1-alpha OS=Homo sapiens GN=LMX1A PE=2 SV=1 - (LMX1A_HUMAN)	0.00	4.71	1	1	2	5	1.148E7	382	42.7	7.27
Q9BV57-2	Isoform 2 of 1,2-dihydroxy-3-keto-5-methylthiopentene dioxygenase OS=Homo sapiens GN=ADI1 - (MTND_HUMAN)	0.00	3.47	2	1	1	1	2.197E7	173	20.3	6.29
P56556	NADH dehydrogenase (ubiquinone) 1 alpha subcomplex subunit 6 OS=Homo sapiens GN=NDUFA6 PE=1 SV=3 - (NDUA6_HUMAN)	0.00	5.19	1	1	1	1		154	17.9	10.14
Q9BT67	NEDD4 family-interacting protein 1 OS=Homo sapiens GN=NDFIP1 PE=1 SV=1 - (NFIP1_HUMAN)	0.00	4.52	1	1	1	1	2.120E6	221	24.9	4.68
Q0D2K0-2	Isoform 2 of Magnesium transporter NIPA4 OS=Homo sapiens GN=NIPAL4 - (NIPA4_HUMAN)	0.00	1.57	2	1	1	1	2.383E7	447	47.9	7.66
P20618	Proteasome subunit beta type-1 OS=Homo sapiens GN=PSMB1 PE=1 SV=2 - (PSB1_HUMAN)	0.00	9.54	4	1	2	3	8.175E6	241	26.5	8.13
Q92932-2	Isoform 2 of Receptor-type tyrosine-protein phosphatase N2 OS=Homo sapiens GN=PTPRN2 - (PTPR2_HUMAN)	0.00	4.06	6	1	5	6	4.361E7	986	108.0	5.92
Q96QR8	Transcriptional activator protein Pur-beta OS=Homo sapiens GN=PURB PE=1 SV=3 - (PURB_HUMAN)	0.00	2.24	1	1	1	8	3.335E7	312	33.2	5.43
Q9HAU5	Regulator of nonsense transcripts 2 OS=Homo sapiens GN=UPF2 PE=1 SV=1 - (RENT2_HUMAN)	0.00	6.13	3	1	9	31	1.951E8	1272	147.7	5.69
Q96SB8	Structural maintenance of chromosomes protein 6 OS=Homo sapiens GN=SMC6 PE=1 SV=2 - (SMC6_HUMAN)	0.00	4.67	3	1	5	5	2.064E7	1091	126.2	6.99
Q13573	SNW domain-containing protein 1 OS=Homo sapiens GN=SNW1 PE=1 SV=1 - (SNW1_HUMAN)	0.00	7.46	6	1	5	10	8.826E7	536	61.5	9.52
Q9UIV8-2	Isoform 2 of Serpin B13 OS=Homo sapiens GN=SERPINB13 - (SPB13_HUMAN)	0.00	10.62	8	1	5	5	8.024E6	339	38.4	5.81
Q86UX6	Serine/threonine-protein kinase 32C OS=Homo sapiens GN=STK32C PE=1 SV=1 - (ST32C_HUMAN)	0.00	4.32	1	1	2	7	5.575E7	486	55.0	6.61
Q9UJW7	Zinc finger protein 229 OS=Homo sapiens GN=ZNF229 PE=2 SV=3 - (ZN229_HUMAN)	0.00	0.73	1	1	1	37	2.053E7	825	93.6	8.53
Q8N184-2	Isoform 2 of Zinc finger protein 567 OS=Homo sapiens GN=ZNF567 - (ZN567_HUMAN)	0.00	2.37	9	1	2	3	1.980E7	591	68.6	9.07
Q6PDB4	Zinc finger protein 880 OS=Homo sapiens GN=ZNF880 PE=2 SV=2 - (ZN880_HUMAN)	0.00	4.16	3	1	3	31	2.120E7	577	66.7	9.29
B4DL80	Cell division cycle protein 27 homolog OS=Homo sapiens GN=CDC27 PE=2 SV=1 - (B4DL80_HUMAN)	0.00	5.90	5	1	3	6	6.455E7	763	84.8	7.31
B4DP65	EMG1 nucleolar protein homolog (S. cerevisiae), isoform CRA_a OS=Homo sapiens GN=EMG1 PE=2 SV=1 - (B4DP65_HUMAN)	0.00	9.93	4	1	1	1	8.819E6	141	15.6	9.80
Q7L3Y3	HCG1785781 OS=Homo sapiens GN=PSD3 PE=4 SV=1 - (Q7L3Y3_HUMAN)	0.00	6.73	1	1	1	7	2.053E7	104	10.9	9.11
C9JD75	Biotin--(acetyl-CoA-carboxylase) ligase (Fragment) OS=Homo sapiens GN=HLCS PE=4 SV=1 - (C9JD75_HUMAN)	0.00	25.29	4	1	2	14	8.570E6	87	9.6	5.24
A8K8F6	cDNA FLJ78417, highly similar to Homo sapiens low density lipoprotein receptor-related protein associated protein 1 (LRPAP1), mRNA OS=Homo sapiens PE=2 SV=1 - (A8K8F6_HUMAN)	0.00	8.12	3	1	4	5	2.365E8	357	41.4	9.06
B4DQK8	cDNA FLJ54750, moderately similar to Pyrroline-5-carboxylate reductase 2 (EC 1.5.1.2) OS=Homo sapiens PE=2 SV=1 - (B4DQK8_HUMAN)	0.00	14.21	6	1	4	4	2.841E8	359	36.7	11.37
E7EWB4	Dihydropyrimidinase-related protein 5 (Fragment) OS=Homo sapiens GN=DPYSL5 PE=4 SV=1 - (E7EWB4_HUMAN)	0.00	3.68	3	1	1	5	5.036E8	190	20.9	6.42
Q6WG76	Antigen MLAA-20 (Fragment) OS=Homo sapiens PE=2 SV=1 - (Q6WG76_HUMAN)	0.00	9.43	1	1	2	3	1.677E7	244	28.1	4.96
A2BF29	Major histocompatibility complex class I B (Fragment) OS=Homo sapiens GN=HLA-B PE=3 SV=1 - (A2BF29_HUMAN)	0.00	15.04	2	1	3	7	6.105E7	246	27.8	6.32
A4D142	Similar to ribosomal protein L23 OS=Homo sapiens GN=LOC222901 PE=3 SV=1 - (A4D142_HUMAN)	0.00	28.97	1	1	2	22	4.862E8	107	11.0	10.40
A8K6K4	cDNA FLJ77565, highly similar to Homo sapiens interleukin 1 receptor accessory protein (IL1RAP), transcript variant 1, mRNA OS=Homo sapiens PE=2 SV=1 - (A8K6K4_HUMAN)	0.00	6.84	6	1	4	5	8.117E7	570	65.4	8.22
B4DDX6	cDNA FLJ57296 OS=Homo sapiens PE=2 SV=1 - (B4DDX6_HUMAN)	0.00	9.42	1	1	2	3	2.599E7	138	15.1	11.03
C9JZZ1	Rabenosyn-5 OS=Homo sapiens GN=ZFYVE20 PE=4 SV=1 - (C9JZZ1_HUMAN)	0.00	6.82	1	1	1	1	2.615E6	88	10.1	6.65
B3KT45	cDNA FLJ37607 fis, clone BRCOC2010980, highly similar to Thioredoxin-like protein 1 OS=Homo sapiens PE=2 SV=1 - (B3KT45_HUMAN)	0.00	4.22	5	1	1	1	1.566E7	166	18.9	4.78
B3KMV8	cDNA FLJ12766 fis, clone NT2RP2001520, highly similar to Calcium-binding mitochondrial carrier protein Aralar1 OS=Homo sapiens PE=2 SV=1 - (B3KMV8_HUMAN)	0.00	4.72	3	1	3	5	3.204E7	678	74.7	8.38
B7Z284	DNA-directed RNA polymerase OS=Homo sapiens PE=2 SV=1 - (B7Z284_HUMAN)	0.00	3.81	4	1	5	6	7.902E6	1207	136.9	6.86
B4E2C8	cDNA FLJ61064, highly similar to Homo sapiens DEAD/H (Asp-Glu-Ala-Asp/His) box polypeptide 26 (DDX26), mRNA OS=Homo sapiens PE=2 SV=1 - (B4E2C8_HUMAN)	0.00	11.43	1	1	5	7	4.911E7	455	51.9	9.26
B4E0E0	cDNA FLJ54898, highly similar to Homo sapiens transmembrane protein 41B (TMEM41B), mRNA OS=Homo sapiens PE=2 SV=1 - (B4E0E0_HUMAN)	0.00	7.56	2	1	2	3	8.133E8	291	32.5	9.51
B4E053	cDNA FLJ61195, highly similar to Ubiquitin thioesterase protein OTUB1 (EC 3.4.-.-) OS=Homo sapiens PE=2 SV=1 - (B4E053_HUMAN)	0.00	7.50	10	1	2	3	6.295E6	280	31.0	7.52
B4E2F2	cDNA FLJ61068, highly similar to Amyloid beta A4 protein-bindingfamily B member 2 (Fe65-like protein) OS=Homo sapiens PE=2 SV=1 - (B4E2F2_HUMAN)	0.00	10.69	7	1	7	12	1.643E7	758	83.3	6.18
H0YDB6	PIH1 domain-containing protein 2 (Fragment) OS=Homo sapiens GN=PIH1D2 PE=4 SV=1 - (H0YDB6_HUMAN)	0.00	12.35	5	1	2	2		162	18.6	9.33
Q59GT9	Gap junction protein (Fragment) OS=Homo sapiens PE=2 SV=1 - (Q59GT9_HUMAN)	0.00	7.50	6	1	4	5	1.672E8	600	66.9	6.46
A8K5N5	cDNA FLJ78142, highly similar to Homo sapiens transglutaminase 1 (K polypeptide epidermal type I, protein-glutamine-gamma-glutamyltransferase), mRNA OS=Homo sapiens PE=2 SV=1 - (A8K5N5_HUMAN)	0.00	6.00	7	1	5	7	4.309E8	817	89.7	6.04
H3BNA1	Ubiquitin carboxyl-terminal hydrolase 10 (Fragment) OS=Homo sapiens GN=USP10 PE=4 SV=1 - (H3BNA1_HUMAN)	0.00	4.00	1	1	1	1		150	17.0	4.58
Q8NAG5	cDNA FLJ35389 fis, clone SKNMC2000635, moderately similar to 65 kDa FK506-BINDING PROTEIN (EC 5.2.1.8) OS=Homo sapiens PE=2 SV=1 - (Q8NAG5_HUMAN)	0.00	6.17	8	1	3	12	1.139E8	470	52.0	5.44
E7EXA5	Exocyst complex component 3-like protein OS=Homo sapiens GN=EXOC3L1 PE=4 SV=1 - (E7EXA5_HUMAN)	0.00	5.02	8	1	2	3	2.060E7	677	73.3	7.44
B4DW36	cDNA FLJ58862, highly similar to THUMP domain-containing protein 2 OS=Homo sapiens PE=2 SV=1 - (B4DW36_HUMAN)	0.00	12.76	5	1	3	7	5.274E7	290	34.0	8.81
Q16234	HuD protein OS=Homo sapiens GN=HuD PE=4 SV=1 - (Q16234_HUMAN)	0.00	54.55	1	1	1	1		11	1.3	11.00
Q6PI41	AURKAIP1 protein (Fragment) OS=Homo sapiens GN=AURKAIP1 PE=2 SV=1 - (Q6PI41_HUMAN)	0.00	9.26	1	1	2	2	1.554E8	162	18.6	10.51

Appendix G  

**Table 9:** Proteins identified in Subject 5 (N5).

Accession	Description	Score	Coverage	# Proteins	# Unique Peptides	# Peptides	# PSMs	Area	# AAs	MW (kDa)	calc. pI
O43790	Keratin, type II cuticular Hb6 OS=Homo sapiens GN=KRT86 PE=1 SV=1 - (KRT86_HUMAN)	16337.46	96.91	13	6	87	6710	1.189E10	486	53.5	5.66
F8W6V8	Keratin, type II cuticular Hb1 OS=Homo sapiens GN=KRT81 PE=3 SV=1 - (F8W6V8_HUMAN)	14375.55	84.09	12	1	79	6119	1.050E10	484	53.1	5.47
P78386	Keratin, type II cuticular Hb5 OS=Homo sapiens GN=KRT85 PE=1 SV=1 - (KRT85_HUMAN)	13590.88	95.66	7	32	89	5514	1.189E10	507	55.8	6.55
P78385	Keratin, type II cuticular Hb3 OS=Homo sapiens GN=KRT83 PE=1 SV=2 - (KRT83_HUMAN)	12072.09	81.95	10	2	77	5318	1.189E10	493	54.2	5.64
A0JNT2	KRT83 protein OS=Homo sapiens GN=KRT83 PE=2 SV=1 - (A0JNT2_HUMAN)	11987.73	86.80	10	1	76	5283	1.189E10	447	49.6	5.39
Q15323	Keratin, type I cuticular Ha1 OS=Homo sapiens GN=KRT31 PE=2 SV=3 - (K1H1_HUMAN)	11028.58	74.28	2	16	57	4464	2.090E10	416	47.2	4.88
O76009	Keratin, type I cuticular Ha3-I OS=Homo sapiens GN=KRT33A PE=2 SV=2 - (KT33A_HUMAN)	9276.07	85.40	1	12	54	3663	2.090E10	404	45.9	4.82
Q14525	Keratin, type I cuticular Ha3-II OS=Homo sapiens GN=KRT33B PE=2 SV=3 - (KT33B_HUMAN)	8795.31	85.40	1	14	55	3450	1.959E10	404	46.2	4.84
A6NCN2	Keratin-81-like protein OS=Homo sapiens PE=2 SV=3 - (KT81L_HUMAN)	8249.14	57.41	5	3	53	3538	1.186E10	486	53.4	6.35
H6VRF8	Keratin 1 OS=Homo sapiens GN=KRT1 PE=3 SV=1 - (H6VRF8_HUMAN)	6125.55	73.76	14	59	72	2080	1.910E9	644	66.0	8.12
C4AMA3	Keratin, type I cuticular Ha4 OS=Homo sapiens GN=KRT34 PE=3 SV=1 - (C4AMA3_HUMAN)	5570.49	84.52	2	25	50	2263	1.958E10	394	44.7	4.78
P13647	Keratin, type II cytoskeletal 5 OS=Homo sapiens GN=KRT5 PE=1 SV=3 - (K2C5_HUMAN)	5276.83	66.27	24	17	78	2243	4.066E9	590	62.3	7.74
P35908	Keratin, type II cytoskeletal 2 epidermal OS=Homo sapiens GN=KRT2 PE=1 SV=2 - (K22E_HUMAN)	5230.12	93.74	5	45	71	1967	2.448E9	639	65.4	8.00
P04259	Keratin, type II cytoskeletal 6B OS=Homo sapiens GN=KRT6B PE=1 SV=5 - (K2C6B_HUMAN)	5094.93	69.86	19	1	72	2175	3.862E9	564	60.0	8.00
B4DRR0	cDNA FLJ53910, highly similar to Keratin, type II cytoskeletal 6A OS=Homo sapiens PE=2 SV=1 - (B4DRR0_HUMAN)	5040.77	75.51	23	4	74	2152	3.862E9	535	57.8	8.00
P13645	Keratin, type I cytoskeletal 10 OS=Homo sapiens GN=KRT10 PE=1 SV=6 - (K1C10_HUMAN)	4313.11	71.06	21	42	53	1595	6.396E9	584	58.8	5.21
B4DKV4	cDNA FLJ60647, highly similar to Keratin, type II cytoskeletal 6B OS=Homo sapiens PE=2 SV=1 - (B4DKV4_HUMAN)	4139.47	65.59	15	2	63	1764	3.862E9	526	55.7	8.43
P02533	Keratin, type I cytoskeletal 14 OS=Homo sapiens GN=KRT14 PE=1 SV=4 - (K1C14_HUMAN)	4031.81	83.90	13	23	61	1707	7.682E9	472	51.5	5.16
Q92764	Keratin, type I cuticular Ha5 OS=Homo sapiens GN=KRT35 PE=2 SV=5 - (KRT35_HUMAN)	3537.59	74.73	2	23	40	1407	1.613E10	455	50.3	4.91
P35527	Keratin, type I cytoskeletal 9 OS=Homo sapiens GN=KRT9 PE=1 SV=3 - (K1C9_HUMAN)	3231.93	72.23	1	43	47	1095	5.258E8	623	62.0	5.24
O95678	Keratin, type II cytoskeletal 75 OS=Homo sapiens GN=KRT75 PE=1 SV=2 - (K2C75_HUMAN)	3096.82	53.90	15	16	48	1345	5.772E9	551	59.5	7.74
P08779	Keratin, type I cytoskeletal 16 OS=Homo sapiens GN=KRT16 PE=1 SV=4 - (K1C16_HUMAN)	2634.03	81.40	12	25	52	1138	7.682E9	473	51.2	5.05
Q04695	Keratin, type I cytoskeletal 17 OS=Homo sapiens GN=KRT17 PE=1 SV=2 - (K1C17_HUMAN)	2524.76	83.80	9	22	50	1106	7.682E9	432	48.1	5.02
H0YI76	Keratin, type II cytoskeletal 5 (Fragment) OS=Homo sapiens GN=KRT5 PE=3 SV=1 - (H0YI76_HUMAN)	2437.46	75.62	10	1	32	861	1.966E9	201	23.1	4.97
P12035	Keratin, type II cytoskeletal 3 OS=Homo sapiens GN=KRT3 PE=1 SV=3 - (K2C3_HUMAN)	2096.91	30.10	21	1	30	1130	3.709E9	628	64.4	6.48
Q701L7	Type II hair keratin 2 OS=Homo sapiens GN=KRTHB2 PE=2 SV=1 - (Q701L7_HUMAN)	2005.37	37.23	2	4	26	994	2.574E9	513	56.6	6.74
Q14532	Keratin, type I cuticular Ha2 OS=Homo sapiens GN=KRT32 PE=1 SV=3 - (K1H2_HUMAN)	1729.38	33.48	1	4	18	720	1.666E10	448	50.3	4.84
Q9NSB2	Keratin, type II cuticular Hb4 OS=Homo sapiens GN=KRT84 PE=1 SV=2 - (KRT84_HUMAN)	1722.15	31.67	6	8	23	904	3.840E9	600	64.8	7.56
P15924	Desmoplakin OS=Homo sapiens GN=DSP PE=1 SV=3 - (DESP_HUMAN)	1372.16	56.01	15	112	171	632	3.608E8	2871	331.6	6.81
F5GZD1	Keratin, type II cytoskeletal 7 OS=Homo sapiens GN=KRT7 PE=4 SV=1 - (F5GZD1_HUMAN)	1360.50	26.80	3	2	15	929	4.684E9	403	44.9	5.55
O76013	Keratin, type I cuticular Ha6 OS=Homo sapiens GN=KRT36 PE=1 SV=1 - (KRT36_HUMAN)	1286.44	20.77	3	1	14	573	1.076E10	467	52.2	4.94
Q9NSB5	Type II hair keratin 1 (Fragment) OS=Homo sapiens GN=KRTHB1 PE=2 SV=1 - (Q9NSB5_HUMAN)	1077.97	97.56	1	1	14	595	8.272E9	123	12.9	8.57
B3KVF5	cDNA FLJ16494 fis, clone CTONG3004576, highly similar to Keratin, type I cytoskeletal 15 OS=Homo sapiens PE=2 SV=1 - (B3KVF5_HUMAN)	1029.64	38.38	6	9	23	387	7.682E9	456	49.1	4.79
P08727	Keratin, type I cytoskeletal 19 OS=Homo sapiens GN=KRT19 PE=1 SV=4 - (K1C19_HUMAN)	947.27	42.00	8	1	25	517	7.410E9	400	44.1	5.14
Q3SY84	Keratin, type II cytoskeletal 71 OS=Homo sapiens GN=KRT71 PE=1 SV=3 - (K2C71_HUMAN)	891.49	62.14	4	19	42	645	9.562E8	523	57.3	6.61
Q86Y46	Keratin, type II cytoskeletal 73 OS=Homo sapiens GN=KRT73 PE=1 SV=1 - (K2C73_HUMAN)	857.30	31.30	6	1	24	634	1.080E9	540	58.9	7.23
Q7RTS7	Keratin, type II cytoskeletal 74 OS=Homo sapiens GN=KRT74 PE=1 SV=2 - (K2C74_HUMAN)	803.49	37.43	5	3	25	606	1.080E9	529	57.8	7.71
Q07283	Trichohyalin OS=Homo sapiens GN=TCHH PE=1 SV=2 - (TRHY_HUMAN)	790.54	60.78	21	92	145	535	1.724E8	1943	253.8	5.78
Q0IIN1	Keratin 77 OS=Homo sapiens GN=KRT77 PE=2 SV=1 - (Q0IIN1_HUMAN)	749.94	18.17	5	3	10	415	1.773E9	578	61.8	5.85
Q7Z3Y7	Keratin, type I cytoskeletal 28 OS=Homo sapiens GN=KRT28 PE=1 SV=2 - (K1C28_HUMAN)	726.40	26.29	1	1	15	385	5.904E9	464	50.5	5.47
B4DRW1	cDNA FLJ55805, highly similar to Keratin, type II cytoskeletal 4 OS=Homo sapiens PE=2 SV=1 - (B4DRW1_HUMAN)	663.26	33.97	10	2	21	342	1.329E9	474	51.7	6.81
Q7Z3Y8	Keratin, type I cytoskeletal 27 OS=Homo sapiens GN=KRT27 PE=1 SV=2 - (K1C27_HUMAN)	628.83	48.80	2	5	19	284	7.848E8	459	49.8	5.05
Q7Z3Z0	Keratin, type I cytoskeletal 25 OS=Homo sapiens GN=KRT25 PE=1 SV=1 - (K1C25_HUMAN)	624.01	46.22	4	5	17	279	7.000E8	450	49.3	5.08
O60814	Histone H2B type 1-K OS=Homo sapiens GN=HIST1H2BK PE=1 SV=3 - (H2B1K_HUMAN)	506.71	67.46	32	10	13	223	1.110E9	126	13.9	10.32
Q8WVW5	Putative uncharacterized protein (Fragment) OS=Homo sapiens PE=2 SV=1 - (Q8WVW5_HUMAN)	468.64	76.03	87	9	30	214	1.140E9	363	40.5	6.14
O76015	Keratin, type I cuticular Ha8 OS=Homo sapiens GN=KRT38 PE=2 SV=3 - (KRT38_HUMAN)	457.57	32.89	2	8	16	245	6.078E9	456	50.4	4.84
P14923	Junction plakoglobin OS=Homo sapiens GN=JUP PE=1 SV=3 - (PLAK_HUMAN)	364.23	42.15	8	21	27	179	6.072E7	745	81.7	6.14
P62805	Histone H4 OS=Homo sapiens GN=HIST1H4A PE=1 SV=2 - (H4_HUMAN)	359.85	73.79	3	11	15	147	1.841E9	103	11.4	11.36
Q2M2I5	Keratin, type I cytoskeletal 24 OS=Homo sapiens GN=KRT24 PE=1 SV=1 - (K1C24_HUMAN)	343.06	12.57	1	1	9	177	5.780E9	525	55.1	4.96
Q8N1N4	Keratin, type II cytoskeletal 78 OS=Homo sapiens GN=KRT78 PE=2 SV=2 - (K2C78_HUMAN)	342.69	25.96	5	3	15	196	2.624E9	520	56.8	6.02
B7ZBC3	Spectrin repeat containing, nuclear envelope 1 OS=Homo sapiens GN=SYNE1 PE=4 SV=1 - (B7ZBC3_HUMAN)	325.48	7.38	21	2	55	212	1.144E9	8797	1010.4	5.53
Q9BYR8	Keratin-associated protein 3-1 OS=Homo sapiens GN=KRTAP3-1 PE=1 SV=1 - (KRA31_HUMAN)	316.61	27.55	1	3	3	101	2.310E9	98	10.5	6.40
Q86YZ3	Hornerin OS=Homo sapiens GN=HRNR PE=1 SV=2 - (HORN_HUMAN)	303.70	26.04	2	15	24	157	3.141E7	2850	282.2	10.04
P07355-2	Isoform 2 of Annexin A2 OS=Homo sapiens GN=ANXA2 - (ANXA2_HUMAN)	296.14	78.99	27	23	30	103	3.804E8	357	40.4	8.37
Q9BTM1	Histone H2A.J OS=Homo sapiens GN=H2AFJ PE=1 SV=1 - (H2AJ_HUMAN)	291.28	48.06	23	3	12	102	8.386E8	129	14.0	10.90
Q93077	Histone H2A type 1-C OS=Homo sapiens GN=HIST1H2AC PE=1 SV=3 - (H2A1C_HUMAN)	277.59	41.54	13	1	9	92	8.386E8	130	14.1	11.05
Q3LI55	Keratin associated protein OS=Homo sapiens GN=KRTAP11-1 PE=2 SV=1 - (Q3LI55_HUMAN)	267.24	26.99	2	4	5	169	1.542E8	163	17.0	7.78
G3V1U9	Tubulin alpha-1A chain OS=Homo sapiens GN=TUBA1A PE=3 SV=1 - (G3V1U9_HUMAN)	256.34	55.77	30	6	21	83	1.508E8	416	46.3	5.08
Q8TC04	Keratin 23 (Histone deacetylase inducible) OS=Homo sapiens GN=KRT23 PE=2 SV=1 - (Q8TC04_HUMAN)	253.08	8.29	1	1	5	190	7.424E8	422	48.1	6.54
P04792	Heat shock protein beta-1 OS=Homo sapiens GN=HSPB1 PE=1 SV=2 - (HSPB1_HUMAN)	248.96	60.49	4	13	16	109	5.786E8	205	22.8	6.40
P31947	14-3-3 protein sigma OS=Homo sapiens GN=SFN PE=1 SV=1 - (1433S_HUMAN)	236.09	69.76	11	15	21	99	3.967E8	248	27.8	4.74
B7TY16	Actinin alpha 1 isoform 3 OS=Homo sapiens GN=ACTN1 PE=2 SV=1 - (B7TY16_HUMAN)	230.50	25.91	26	4	22	85	3.833E7	930	107.1	5.58
A8MUB1	Tubulin alpha-4A chain OS=Homo sapiens GN=TUBA4A PE=2 SV=1 - (A8MUB1_HUMAN)	223.75	44.57	14	3	18	74	1.380E8	433	48.3	5.01
B0YJC4	Vimentin OS=Homo sapiens GN=VIM PE=3 SV=1 - (B0YJC4_HUMAN)	215.46	23.90	6	1	8	113	2.468E9	431	49.6	5.25
B7Z6P1	cDNA FLJ53662, highly similar to Actin, alpha skeletal muscle OS=Homo sapiens PE=2 SV=1 - (B7Z6P1_HUMAN)	214.21	40.82	51	1	20	133	7.336E8	343	38.6	5.38
P47929	Galectin-7 OS=Homo sapiens GN=LGALS7 PE=1 SV=2 - (LEG7_HUMAN)	205.69	93.38	1	13	16	90	1.031E9	136	15.1	7.62
O43707	Alpha-actinin-4 OS=Homo sapiens GN=ACTN4 PE=1 SV=2 - (ACTN4_HUMAN)	193.39	22.39	19	5	18	63	3.874E7	911	104.8	5.44
Q05BI1	CD3EAP protein (Fragment) OS=Homo sapiens GN=CD3EAP PE=2 SV=1 - (Q05BI1_HUMAN)	192.27	7.14	7	1	3	94	1.481E7	392	42.0	9.11
F8VW92	Tubulin beta chain OS=Homo sapiens GN=TUBB PE=3 SV=1 - (F8VW92_HUMAN)	188.42	46.44	41	1	16	89	1.030E8	435	48.5	5.20
P68371	Tubulin beta-4B chain OS=Homo sapiens GN=TUBB4B PE=1 SV=1 - (TBB4B_HUMAN)	183.54	48.76	23	2	18	88	1.030E8	445	49.8	4.89
B2R6L0	cDNA, FLJ93005, highly similar to Homo sapiens tubulin, beta polypeptide (TUBB), mRNA OS=Homo sapiens PE=2 SV=1 - (B2R6L0_HUMAN)	182.14	46.07	26	1	17	92	9.403E7	445	49.9	4.89
Q8WVJ2	NudC domain-containing protein 2 OS=Homo sapiens GN=NUDCD2 PE=1 SV=1 - (NUDC2_HUMAN)	178.56	17.83	1	1	2	98	6.389E7	157	17.7	5.07
O15078	Centrosomal protein of 290 kDa OS=Homo sapiens GN=CEP290 PE=1 SV=2 - (CE290_HUMAN)	174.88	17.39	9	3	38	177	3.578E8	2479	290.2	5.95
A0N4V7	HCG2039797 (Fragment) OS=Homo sapiens GN=Tcr-alpha PE=4 SV=1 - (A0N4V7_HUMAN)	173.86	38.10	1	1	1	254	5.761E9	21	2.2	9.74
O43149	Zinc finger ZZ-type and EF-hand domain-containing protein 1 OS=Homo sapiens GN=ZZEF1 PE=1 SV=6 - (ZZEF1_HUMAN)	163.87	3.68	2	1	11	97	1.064E9	2961	330.9	5.95
O94822	E3 ubiquitin-protein ligase listerin OS=Homo sapiens GN=LTN1 PE=1 SV=6 - (LTN1_HUMAN)	162.54	2.77	1	1	4	116	1.660E8	1766	200.4	6.25
Q6KB66-2	Isoform 2 of Keratin, type II cytoskeletal 80 OS=Homo sapiens GN=KRT80 - (K2C80_HUMAN)	160.34	27.96	5	3	10	65	6.431E8	422	47.2	5.30
Q562R1	Beta-actin-like protein 2 OS=Homo sapiens GN=ACTBL2 PE=1 SV=2 - (ACTBL_HUMAN)	159.41	26.86	17	1	14	100	9.035E8	376	42.0	5.59
Q6A163	Keratin, type I cytoskeletal 39 OS=Homo sapiens GN=KRT39 PE=1 SV=2 - (K1C39_HUMAN)	155.77	30.55	16	11	17	77	6.882E7	491	55.6	5.26
Q8IZT6	Abnormal spindle-like microcephaly-associated protein OS=Homo sapiens GN=ASPM PE=1 SV=2 - (ASPM_HUMAN)	155.69	11.13	6	1	35	141	2.476E9	3477	409.5	10.45
B4E380	Histone H3 OS=Homo sapiens PE=3 SV=1 - (B4E380_HUMAN)	154.51	66.37	12	8	17	109	8.819E8	113	12.9	11.93
O75116	Rho-associated protein kinase 2 OS=Homo sapiens GN=ROCK2 PE=1 SV=4 - (ROCK2_HUMAN)	153.13	6.56	4	1	10	89	2.346E9	1388	160.8	6.02
P12036-2	Isoform 2 of Neurofilament heavy polypeptide OS=Homo sapiens GN=NEFH - (NFH_HUMAN)	150.24	8.10	3	1	8	64	3.087E8	963	105.6	5.91
A8MTN3	Putative keratin-associated protein ENSP00000381495 OS=Homo sapiens PE=3 SV=2 - (KRA2X_HUMAN)	144.82	26.83	7	2	2	73	1.098E9	123	12.9	7.81
Q13835-2	Isoform 1 of Plakophilin-1 OS=Homo sapiens GN=PKP1 - (PKP1_HUMAN)	142.51	35.26	4	21	24	72	6.006E7	726	80.4	8.97
Q86UQ4	ATP-binding cassette sub-family A member 13 OS=Homo sapiens GN=ABCA13 PE=2 SV=3 - (ABCAD_HUMAN)	138.09	4.53	2	1	19	76	7.720E8	5058	575.8	6.46
Q15149-4	Isoform 4 of Plectin OS=Homo sapiens GN=PLEC - (PLEC_HUMAN)	136.34	16.71	11	13	65	151	5.080E8	4547	515.9	5.80
Q2TSD0	Glyceraldehyde-3-phosphate dehydrogenase OS=Homo sapiens PE=2 SV=1 - (Q2TSD0_HUMAN)	134.44	51.64	11	11	13	74	5.389E9	335	36.0	8.46
C9J1U3	Protein unc-80 homolog OS=Homo sapiens GN=UNC80 PE=4 SV=1 - (C9J1U3_HUMAN)	129.28	5.19	4	1	15	71	1.014E9	3234	360.6	6.92
P35579	Myosin-9 OS=Homo sapiens GN=MYH9 PE=1 SV=4 - (MYH9_HUMAN)	129.04	19.54	7	11	38	83	1.159E9	1960	226.4	5.60
Q9P225-2	Isoform 2 of Dynein heavy chain 2, axonemal OS=Homo sapiens GN=DNAH2 - (DYH2_HUMAN)	128.36	7.53	3	1	26	82	5.578E8	4196	481.7	6.43
P46939	Utrophin OS=Homo sapiens GN=UTRN PE=1 SV=2 - (UTRO_HUMAN)	123.77	7.22	3	1	21	92	4.489E8	3433	394.2	5.33
Q6DRA6	Putative histone H2B type 2-D OS=Homo sapiens GN=HIST2H2BD PE=5 SV=3 - (H2B2D_HUMAN)	122.64	27.44	20	2	6	55	1.330E8	164	18.0	10.58
Q6PK75	PRSS2 protein (Fragment) OS=Homo sapiens GN=PRSS2 PE=2 SV=1 - (Q6PK75_HUMAN)	121.94	16.04	16	1	2	58	4.960E8	187	20.1	4.87
Q8NAM9	cDNA FLJ35099 fis, clone PLACE6006287 OS=Homo sapiens PE=2 SV=1 - (Q8NAM9_HUMAN)	120.29	7.59	1	1	2	109	2.050E8	224	24.0	11.75
P26038	Moesin OS=Homo sapiens GN=MSN PE=1 SV=3 - (MOES_HUMAN)	115.39	24.78	30	6	16	77	1.497E9	577	67.8	6.40
Q9BYR6	Keratin-associated protein 3-3 OS=Homo sapiens GN=KRTAP3-3 PE=1 SV=1 - (KRA33_HUMAN)	112.67	24.49	1	1	2	31	1.397E9	98	10.4	5.69
C9JH62	Zinc finger protein 277 (Fragment) OS=Homo sapiens GN=ZNF277 PE=4 SV=1 - (C9JH62_HUMAN)	110.64	13.57	4	1	2	46	2.911E9	199	22.7	6.16
F6KPG5	Albumin (Fragment) OS=Homo sapiens PE=2 SV=1 - (F6KPG5_HUMAN)	107.38	25.81	15	7	11	51	6.638E7	585	66.5	6.04
Q01469	Fatty acid-binding protein, epidermal OS=Homo sapiens GN=FABP5 PE=1 SV=3 - (FABP5_HUMAN)	105.92	91.11	2	15	19	54	2.496E8	135	15.2	7.01
B4DMP5	cDNA FLJ60050, highly similar to Vacuolar protein sorting protein 72 homolog OS=Homo sapiens PE=2 SV=1 - (B4DMP5_HUMAN)	105.73	7.63	3	1	2	67	6.518E9	236	26.7	5.33
D3DVC4	Nestin, isoform CRA_c OS=Homo sapiens GN=NES PE=3 SV=1 - (D3DVC4_HUMAN)	105.64	8.45	5	1	12	61	9.173E8	1621	177.3	4.36
Q02413	Desmoglein-1 OS=Homo sapiens GN=DSG1 PE=1 SV=2 - (DSG1_HUMAN)	104.29	18.59	2	9	15	42	3.367E7	1049	113.7	5.03
P68104	Elongation factor 1-alpha 1 OS=Homo sapiens GN=EEF1A1 PE=1 SV=1 - (EF1A1_HUMAN)	100.13	23.16	40	10	11	47	2.016E8	462	50.1	9.01
Q2VIP3	PABP3 (Fragment) OS=Homo sapiens PE=2 SV=1 - (Q2VIP3_HUMAN)	98.85	16.98	19	2	8	82	6.873E8	630	69.9	9.67
P27348	14-3-3 protein theta OS=Homo sapiens GN=YWHAQ PE=1 SV=1 - (1433T_HUMAN)	96.74	44.90	12	7	13	38	3.111E8	245	27.7	4.78
Q9BYR7	Keratin-associated protein 3-2 OS=Homo sapiens GN=KRTAP3-2 PE=1 SV=1 - (KRA32_HUMAN)	96.43	27.55	1	2	3	25	1.007E9	98	10.4	5.69
B2RAW1	cDNA, FLJ95155, highly similar to Homo sapiens Bruton agammaglobulinemia tyrosine kinase (BTK), mRNA OS=Homo sapiens PE=2 SV=1 - (B2RAW1_HUMAN)	96.22	5.77	14	1	3	56	3.249E8	659	76.2	7.77
O75369-8	Isoform 8 of Filamin-B OS=Homo sapiens GN=FLNB - (FLNB_HUMAN)	94.41	7.94	21	6	17	41	1.775E8	2633	281.5	5.71
F8W8Y7	Armadillo repeat-containing X-linked protein 4 OS=Homo sapiens GN=ARMCX4 PE=4 SV=1 - (F8W8Y7_HUMAN)	92.33	8.73	3	1	16	68	4.282E8	2394	247.7	5.95
P63104	14-3-3 protein zeta/delta OS=Homo sapiens GN=YWHAZ PE=1 SV=1 - (1433Z_HUMAN)	91.48	47.35	20	4	12	43	2.617E8	245	27.7	4.79
Q6AWC2-4	Isoform 4 of Protein WWC2 OS=Homo sapiens GN=WWC2 - (WWC2_HUMAN)	91.39	9.19	6	1	10	57	5.893E8	1143	128.6	5.90
P81605	Dermcidin OS=Homo sapiens GN=DCD PE=1 SV=2 - (DCD_HUMAN)	89.87	37.27	2	3	4	32	3.105E7	110	11.3	6.54
O95613	Pericentrin OS=Homo sapiens GN=PCNT PE=1 SV=4 - (PCNT_HUMAN)	88.34	12.29	3	1	34	102	1.011E9	3336	377.8	5.55
P61981	14-3-3 protein gamma OS=Homo sapiens GN=YWHAG PE=1 SV=2 - (1433G_HUMAN)	87.34	27.53	13	1	9	43	3.111E8	247	28.3	4.89
Q86TY5	Full-length cDNA clone CS0DI041YE05 of Placenta of Homo sapiens (human) OS=Homo sapiens PE=2 SV=1 - (Q86TY5_HUMAN)	85.11	76.03	16	9	10	51	1.066E8	121	13.9	9.16
Q5SQ08	Complement component 8, gamma polypeptide (Fragment) OS=Homo sapiens GN=C8G PE=2 SV=1 - (Q5SQ08_HUMAN)	85.10	18.29	2	1	2	47	6.820E7	164	17.5	9.79
P09382	Galectin-1 OS=Homo sapiens GN=LGALS1 PE=1 SV=2 - (LEG1_HUMAN)	84.26	50.37	2	6	9	38	1.396E8	135	14.7	5.50
B4DYS1	cDNA FLJ50921, highly similar to Eukaryotic translation initiation factor 3 subunit 10 OS=Homo sapiens PE=2 SV=1 - (B4DYS1_HUMAN)	84.17	21.88	8	2	27	69	5.319E8	1348	162.3	6.99
Q8NCY6	Myb/SANT-like DNA-binding domain-containing protein 4 OS=Homo sapiens GN=MSANTD4 PE=1 SV=1 - (MSD4_HUMAN)	83.63	13.91	1	1	6	45	1.007E9	345	41.1	5.44
Q15075	Early endosome antigen 1 OS=Homo sapiens GN=EEA1 PE=1 SV=2 - (EEA1_HUMAN)	83.62	22.75	2	1	27	74	6.043E8	1411	162.4	5.68
Q96QV6	Histone H2A type 1-A OS=Homo sapiens GN=HIST1H2AA PE=1 SV=3 - (H2A1A_HUMAN)	81.22	40.46	13	1	7	37	7.256E8	131	14.2	10.86
P62987	Ubiquitin-60S ribosomal protein L40 OS=Homo sapiens GN=UBA52 PE=1 SV=2 - (RL40_HUMAN)	79.65	53.91	30	5	8	53	2.316E8	128	14.7	9.83
Q5I6Y6	Lamin A/C transcript variant 1 OS=Homo sapiens GN=LMNA PE=2 SV=1 - (Q5I6Y6_HUMAN)	77.24	29.22	20	8	18	43	5.182E8	664	74.0	7.18
Q8N5Z7	60S ribosomal protein L6 OS=Homo sapiens GN=RPL6 PE=2 SV=1 - (Q8N5Z7_HUMAN)	77.06	12.85	11	1	5	61	9.454E7	288	32.7	10.58
Q09666	Neuroblast differentiation-associated protein AHNAK OS=Homo sapiens GN=AHNAK PE=1 SV=2 - (AHNK_HUMAN)	76.61	19.54	5	15	36	60	1.855E8	5890	628.7	6.15
Q8NGB0	Seven transmembrane helix receptor OS=Homo sapiens GN=GPR142 PE=2 SV=1 - (Q8NGB0_HUMAN)	75.20	6.90	1	1	9	41	5.650E8	1464	156.4	10.95
O14863	Zinc transporter 4 OS=Homo sapiens GN=SLC30A4 PE=2 SV=2 - (ZNT4_HUMAN)	73.66	2.10	1	1	1	58	9.362E8	429	47.5	6.58
P62937	Peptidyl-prolyl cis-trans isomerase A OS=Homo sapiens GN=PPIA PE=1 SV=2 - (PPIA_HUMAN)	73.25	71.52	19	11	14	34	1.559E8	165	18.0	7.81
C0JYZ1	Dynein, axonemal, heavy chain 11 OS=Homo sapiens GN=DNAH11 PE=4 SV=1 - (C0JYZ1_HUMAN)	72.46	5.31	3	1	24	80	6.370E8	4522	521.0	6.47
B5BU24	14-3-3 protein beta/alpha OS=Homo sapiens GN=YWHAB PE=2 SV=1 - (B5BU24_HUMAN)	70.53	39.43	11	1	12	35	3.111E8	246	28.1	4.83
Q13439-3	Isoform 3 of Golgin subfamily A member 4 OS=Homo sapiens GN=GOLGA4 - (GOGA4_HUMAN)	70.44	13.09	7	1	28	71	6.299E8	2223	260.2	5.40
P54652	Heat shock-related 70 kDa protein 2 OS=Homo sapiens GN=HSPA2 PE=1 SV=1 - (HSP72_HUMAN)	69.80	30.36	3	4	19	41	1.002E8	639	70.0	5.74
B4DHC2	cDNA FLJ56434, highly similar to p130Cas-associated protein OS=Homo sapiens PE=2 SV=1 - (B4DHC2_HUMAN)	69.73	10.92	1	1	10	64	3.971E8	1035	111.0	9.41
A0PK11	Clarin-2 OS=Homo sapiens GN=CLRN2 PE=2 SV=1 - (CLRN2_HUMAN)	69.63	3.02	1	1	1	49	4.539E8	232	25.4	6.99
Q0Z7S8	Fatty acid-binding protein 9 OS=Homo sapiens GN=FABP9 PE=1 SV=1 - (FABP9_HUMAN)	68.02	68.18	1	8	11	33	7.978E7	132	15.1	8.09
Q9H875	PRKR-interacting protein 1 OS=Homo sapiens GN=PRKRIP1 PE=1 SV=1 - (PKRI1_HUMAN)	67.48	16.85	1	1	3	40	2.007E8	184	21.0	9.79
P33764	Protein S100-A3 OS=Homo sapiens GN=S100A3 PE=1 SV=1 - (S10A3_HUMAN)	66.92	48.51	1	6	6	27	2.237E8	101	11.7	4.78
Q7Z406	Myosin-14 OS=Homo sapiens GN=MYH14 PE=1 SV=2 - (MYH14_HUMAN)	65.53	15.24	13	3	32	60	1.106E8	1995	227.7	5.60
B3KTV0	cDNA FLJ38781 fis, clone LIVER2000216, highly similar to HEAT SHOCK COGNATE 71 kDa PROTEIN OS=Homo sapiens PE=2 SV=1 - (B3KTV0_HUMAN)	64.58	30.92	27	3	19	39	2.182E8	621	67.9	5.45
Q99996-5	Isoform 5 of A-kinase anchor protein 9 OS=Homo sapiens GN=AKAP9 - (AKAP9_HUMAN)	64.48	8.56	10	1	28	65	1.595E8	3857	447.5	4.98
O95147	Dual specificity protein phosphatase 14 OS=Homo sapiens GN=DUSP14 PE=1 SV=1 - (DUS14_HUMAN)	64.08	65.66	1	7	11	23	4.745E7	198	22.2	9.57
A2RUB1	Uncharacterized protein C17orf104 OS=Homo sapiens GN=C17orf104 PE=2 SV=3 - (CQ104_HUMAN)	64.00	9.14	5	1	11	47	1.610E8	952	107.5	7.12
Q86Y33-5	Isoform 5 of Cell division cycle protein 20 homolog B OS=Homo sapiens GN=CDC20B - (CD20B_HUMAN)	62.56	13.02	5	1	2	60	4.435E8	192	21.7	8.15
P00338	L-lactate dehydrogenase A chain OS=Homo sapiens GN=LDHA PE=1 SV=2 - (LDHA_HUMAN)	62.34	31.93	22	7	12	33	4.454E7	332	36.7	8.27
A1L390	Pleckstrin homology domain-containing family G member 3 OS=Homo sapiens GN=PLEKHG3 PE=1 SV=1 - (PKHG3_HUMAN)	60.91	8.45	3	1	11	40	3.089E8	1219	134.3	6.55
Q8TDJ6-2	Isoform 2 of DmX-like protein 2 OS=Homo sapiens GN=DMXL2 - (DMXL2_HUMAN)	60.60	2.58	5	1	7	59	6.432E8	2400	270.0	6.60
Q3V6T2-2	Isoform 2 of Girdin OS=Homo sapiens GN=CCDC88A - (GRDN_HUMAN)	60.39	10.96	10	2	17	42	1.315E8	1843	212.5	5.97
Q6ISF6	KRTAP9-2 protein (Fragment) OS=Homo sapiens GN=KRTAP9-2 PE=2 SV=1 - (Q6ISF6_HUMAN)	60.17	8.09	1	1	1	15	2.143E7	173	18.1	7.69
P07737	Profilin-1 OS=Homo sapiens GN=PFN1 PE=1 SV=2 - (PROF1_HUMAN)	59.87	55.71	2	7	8	26	5.123E7	140	15.0	8.27
O15066	Kinesin-like protein KIF3B OS=Homo sapiens GN=KIF3B PE=1 SV=1 - (KIF3B_HUMAN)	59.54	10.31	2	1	9	54	9.402E7	747	85.1	7.69
Q96AQ8	Coiled-coil domain-containing protein 90A, mitochondrial OS=Homo sapiens GN=CCDC90A PE=2 SV=1 - (CC90A_HUMAN)	58.43	16.43	2	1	6	57	2.341E8	359	39.7	9.63
P20592	Interferon-induced GTP-binding protein Mx2 OS=Homo sapiens GN=MX2 PE=1 SV=1 - (MX2_HUMAN)	56.72	16.08	2	1	12	60	3.057E8	715	82.0	8.76
H0YGD3	Nesprin-1 (Fragment) OS=Homo sapiens GN=SYNE1 PE=4 SV=1 - (H0YGD3_HUMAN)	55.83	4.35	1	1	2	75	7.367E7	207	23.9	8.12
P27482	Calmodulin-like protein 3 OS=Homo sapiens GN=CALML3 PE=1 SV=2 - (CALL3_HUMAN)	55.53	67.79	13	6	9	30	3.171E8	149	16.9	4.42
B7Z4V2	Stress-70 protein, mitochondrial OS=Homo sapiens GN=HSPA9 PE=2 SV=1 - (B7Z4V2_HUMAN)	55.19	11.73	8	1	7	33	4.833E8	665	72.4	5.94
Q32MZ4-2	Isoform 2 of Leucine-rich repeat flightless-interacting protein 1 OS=Homo sapiens GN=LRRFIP1 - (LRRF1_HUMAN)	54.88	14.41	8	1	9	52	3.961E8	784	86.4	4.65
Q9UMY1	Nucleolar protein 7 OS=Homo sapiens GN=NOL7 PE=1 SV=2 - (NOL7_HUMAN)	54.41	5.84	1	1	2	31	6.905E8	257	29.4	9.67
E7ETI5	G-protein-coupled receptor 98 OS=Homo sapiens GN=GPR98 PE=4 SV=1 - (E7ETI5_HUMAN)	53.81	2.22	3	1	10	32	2.813E8	6306	692.7	4.64
Q14134-2	Isoform Beta of Tripartite motif-containing protein 29 OS=Homo sapiens GN=TRIM29 - (TRI29_HUMAN)	53.29	30.70	21	10	17	31	6.406E7	570	63.8	6.98
B4DF70	cDNA FLJ60461, highly similar to Peroxiredoxin-2 (EC 1.11.1.15) OS=Homo sapiens PE=2 SV=1 - (B4DF70_HUMAN)	52.85	30.05	3	4	7	20	3.957E7	183	20.1	8.78
P08107	Heat shock 70 kDa protein 1A/1B OS=Homo sapiens GN=HSPA1A PE=1 SV=5 - (HSP71_HUMAN)	52.73	28.86	24	10	17	33	5.599E7	641	70.0	5.66
Q86SJ6	Desmoglein-4 OS=Homo sapiens GN=DSG4 PE=1 SV=1 - (DSG4_HUMAN)	52.01	10.29	2	5	10	30	2.837E8	1040	113.8	4.56
B3KVD6	cDNA FLJ16433 fis, clone BRACE3013936, highly similar to Cell death activator CIDE-B OS=Homo sapiens PE=2 SV=1 - (B3KVD6_HUMAN)	51.45	12.79	2	1	2	46	1.252E8	219	24.8	9.03
Q13228	Selenium-binding protein 1 OS=Homo sapiens GN=SELENBP1 PE=1 SV=2 - (SBP1_HUMAN)	51.36	26.06	14	7	12	35	4.600E7	472	52.4	6.37
I0B0K8	Truncated profilaggrin OS=Homo sapiens GN=FLG PE=4 SV=1 - (I0B0K8_HUMAN)	50.53	17.36	13	2	36	93	1.794E9	4021	430.2	9.29
Q5MJQ3	Potassium voltage-gated channel beta subunit OS=Homo sapiens GN=KCNAB1 PE=2 SV=1 - (Q5MJQ3_HUMAN)	50.45	9.68	7	1	4	32	6.044E7	372	41.3	9.47
Q8NEY8-5	Isoform 5 of Periphilin-1 OS=Homo sapiens GN=PPHLN1 - (PPHLN_HUMAN)	50.37	6.20	2	1	2	67	8.640E8	258	29.8	9.20
P62258	14-3-3 protein epsilon OS=Homo sapiens GN=YWHAE PE=1 SV=1 - (1433E_HUMAN)	50.35	30.59	11	2	8	26	2.586E8	255	29.2	4.74
Q96JS0	Tubby homologue (Fragment) OS=Homo sapiens GN=TUB PE=2 SV=1 - (Q96JS0_HUMAN)	50.28	12.33	6	1	4	27	2.644E7	373	40.4	9.23
B3KR97	cDNA FLJ33900 fis, clone CTONG2008262, highly similar to Low density lipoprotein receptor adapter protein 1 OS=Homo sapiens PE=2 SV=1 - (B3KR97_HUMAN)	50.25	10.06	2	2	4	41	5.968E7	308	33.9	6.70
O94819	Kelch repeat and BTB domain-containing protein 11 OS=Homo sapiens GN=KBTBD11 PE=1 SV=1 - (KBTBB_HUMAN)	49.50	5.46	1	1	3	23	6.692E7	623	65.7	6.07
Q5HY54	Filamin-A OS=Homo sapiens GN=FLNA PE=2 SV=1 - (Q5HY54_HUMAN)	48.91	9.55	17	4	21	38	2.813E8	2607	276.4	6.05
O95447	Lebercilin-like protein OS=Homo sapiens GN=LCA5L PE=2 SV=1 - (LCA5L_HUMAN)	48.73	12.24	2	1	6	24	1.125E8	670	76.5	9.48
B0AZQ4	cDNA, FLJ79494, highly similar to Structural maintenance of chromosome 3 OS=Homo sapiens PE=2 SV=1 - (B0AZQ4_HUMAN)	48.73	18.08	3	1	22	51	2.575E8	1217	141.4	7.18
Q15828	Cystatin-M OS=Homo sapiens GN=CST6 PE=1 SV=1 - (CYTM_HUMAN)	48.67	56.38	6	7	8	25	1.877E8	149	16.5	8.09
B4DVQ3	Calcium/calmodulin-dependent protein kinase type II subunit gamma OS=Homo sapiens GN=CAMK2G PE=2 SV=1 - (B4DVQ3_HUMAN)	48.60	12.78	1	1	2	37	2.117E8	180	20.5	8.75
A2RRR7	CCDC46 protein OS=Homo sapiens GN=CCDC46 PE=2 SV=1 - (A2RRR7_HUMAN)	47.71	11.72	6	1	13	41	3.327E8	913	107.6	6.34
P60174	Triosephosphate isomerase OS=Homo sapiens GN=TPI1 PE=1 SV=3 - (TPIS_HUMAN)	46.67	39.16	6	6	11	20	3.808E7	286	30.8	5.92
Q6MZM0	Hephaestin-like protein 1 OS=Homo sapiens GN=HEPHL1 PE=2 SV=2 - (HPHL1_HUMAN)	46.25	13.37	1	9	12	20	2.732E7	1159	131.5	6.74
O15173	Membrane-associated progesterone receptor component 2 OS=Homo sapiens GN=PGRMC2 PE=1 SV=1 - (PGRC2_HUMAN)	46.12	14.35	1	1	3	28	7.894E8	223	23.8	4.88
Q8WZ74	Cortactin-binding protein 2 OS=Homo sapiens GN=CTTNBP2 PE=1 SV=1 - (CTTB2_HUMAN)	45.98	13.95	8	1	20	52	1.507E8	1663	180.9	7.93
I3L0X1	Fatty aldehyde dehydrogenase OS=Homo sapiens GN=ALDH3A2 PE=4 SV=1 - (I3L0X1_HUMAN)	45.68	14.29	4	1	3	33	4.869E8	182	20.7	5.03
Q6ZNS7	CDNA FLJ27229 fis, clone SYN05701 OS=Homo sapiens PE=2 SV=1 - (Q6ZNS7_HUMAN)	45.03	9.28	6	1	2	27	4.861E6	194	20.8	6.60
P58107	Epiplakin OS=Homo sapiens GN=EPPK1 PE=1 SV=2 - (EPIPL_HUMAN)	44.93	15.74	3	3	28	70	1.506E8	5090	555.3	5.60
P13639	Elongation factor 2 OS=Homo sapiens GN=EEF2 PE=1 SV=4 - (EF2_HUMAN)	44.89	18.07	15	9	15	23	4.607E7	858	95.3	6.83
O94832	Unconventional myosin-Id OS=Homo sapiens GN=MYO1D PE=1 SV=2 - (MYO1D_HUMAN)	44.77	8.15	2	1	7	30	3.936E8	1006	116.1	9.39
P37802	Transgelin-2 OS=Homo sapiens GN=TAGLN2 PE=1 SV=3 - (TAGL2_HUMAN)	44.42	37.69	3	5	6	14	2.248E7	199	22.4	8.25
Q12955	Ankyrin-3 OS=Homo sapiens GN=ANK3 PE=1 SV=3 - (ANK3_HUMAN)	43.76	4.77	12	2	16	49	5.849E8	4377	480.1	6.49
Q6IQ47	PARD3 protein OS=Homo sapiens GN=PARD3 PE=2 SV=1 - (Q6IQ47_HUMAN)	41.91	9.85	11	1	10	29	3.421E8	1340	149.6	8.02
Q2M1J6	Oxidase (Cytochrome c) assembly 1-like OS=Homo sapiens GN=OXA1L PE=2 SV=1 - (Q2M1J6_HUMAN)	41.87	10.08	6	1	6	34	1.769E8	496	55.3	9.69
P31949	Protein S100-A11 OS=Homo sapiens GN=S100A11 PE=1 SV=2 - (S10AB_HUMAN)	41.57	56.19	2	6	6	16	6.032E7	105	11.7	7.12
Q6ZMD1	cDNA FLJ23994 fis, clone HRC11286 OS=Homo sapiens PE=2 SV=1 - (Q6ZMD1_HUMAN)	40.86	16.72	2	1	4	34	1.014E8	287	30.9	7.75
B7ZLH8	EVPL protein OS=Homo sapiens GN=EVPL PE=2 SV=1 - (B7ZLH8_HUMAN)	40.26	15.82	2	2	31	54	5.397E8	2055	233.7	7.25
F8W7K3	Spectrin alpha chain, brain OS=Homo sapiens GN=SPTAN1 PE=4 SV=1 - (F8W7K3_HUMAN)	39.93	9.58	6	1	19	51	6.045E7	2452	282.0	5.34
Q9NS87-2	Isoform 2 of Kinesin-like protein KIF15 OS=Homo sapiens GN=KIF15 - (KIF15_HUMAN)	39.69	14.41	5	1	18	33	2.430E8	1291	149.7	6.05
P55072	Transitional endoplasmic reticulum ATPase OS=Homo sapiens GN=VCP PE=1 SV=4 - (TERA_HUMAN)	39.68	17.49	8	9	11	19	7.088E6	806	89.3	5.26
E7EP17	Dynein heavy chain 9, axonemal OS=Homo sapiens GN=DNAH9 PE=4 SV=1 - (E7EP17_HUMAN)	39.68	7.19	3	1	26	73	2.658E8	4410	503.0	5.90
B7Z7A9	Phosphoglycerate kinase OS=Homo sapiens GN=PGK1 PE=2 SV=1 - (B7Z7A9_HUMAN)	39.46	25.45	5	2	7	23	1.170E8	389	41.4	7.33
Q9Y6R9	Coiled-coil domain-containing protein 61 OS=Homo sapiens GN=CCDC61 PE=1 SV=2 - (CCD61_HUMAN)	39.45	6.33	1	1	5	28	2.017E8	474	53.2	9.95
Q13332-6	Isoform 2 of Receptor-type tyrosine-protein phosphatase S OS=Homo sapiens GN=PTPRS - (PTPRS_HUMAN)	39.33	3.30	10	1	6	23	1.567E8	1910	212.4	6.74
O43175	D-3-phosphoglycerate dehydrogenase OS=Homo sapiens GN=PHGDH PE=1 SV=4 - (SERA_HUMAN)	37.81	18.57	11	4	8	20	3.166E7	533	56.6	6.71
O00186	Syntaxin-binding protein 3 OS=Homo sapiens GN=STXBP3 PE=1 SV=2 - (STXB3_HUMAN)	37.56	7.94	1	1	4	31	6.641E7	592	67.7	7.80
Q8TE73	Dynein heavy chain 5, axonemal OS=Homo sapiens GN=DNAH5 PE=1 SV=3 - (DYH5_HUMAN)	37.50	6.14	2	1	23	46	1.146E8	4624	528.7	6.10
P06733	Alpha-enolase OS=Homo sapiens GN=ENO1 PE=1 SV=2 - (ENOA_HUMAN)	37.21	34.33	25	7	13	22	6.297E7	434	47.1	7.39
P42858	Huntingtin OS=Homo sapiens GN=HTT PE=1 SV=2 - (HD_HUMAN)	37.19	8.69	2	1	16	32	1.742E8	3142	347.4	6.20
E9PDB1	Non-erythrocytic beta-spectrin 4 OS=Homo sapiens GN=SPTBN4 PE=2 SV=2 - (E9PDB1_HUMAN)	37.14	9.52	8	2	22	72	3.183E8	2564	288.9	6.06
B4DRT3	Pyruvate kinase OS=Homo sapiens PE=2 SV=1 - (B4DRT3_HUMAN)	36.98	18.40	18	4	8	15	3.638E7	511	55.9	7.50
P36952	Serpin B5 OS=Homo sapiens GN=SERPINB5 PE=1 SV=2 - (SPB5_HUMAN)	36.84	26.67	4	7	9	19	9.764E7	375	42.1	6.05
F8WCW5	Hyaluronan mediated motility receptor OS=Homo sapiens GN=HMMR PE=4 SV=1 - (F8WCW5_HUMAN)	36.79	14.41	13	1	10	26	2.786E8	701	81.6	5.82
Q96LA8-2	Isoform 2 of Protein arginine N-methyltransferase 6 OS=Homo sapiens GN=PRMT6 - (ANM6_HUMAN)	36.78	4.79	2	1	1	15	1.374E8	292	32.5	5.00
E5KNB2	DNA-apurinic or apyrimidinic site lyase 2 OS=Homo sapiens PE=4 SV=1 - (E5KNB2_HUMAN)	36.65	4.05	3	1	2	22	3.514E8	518	57.4	8.29
A8MXQ7	Putative IQ motif and ankyrin repeat domain-containing protein LOC642574 OS=Homo sapiens PE=5 SV=2 - (YH010_HUMAN)	36.18	16.56	1	1	11	22	8.473E7	604	68.3	6.52
Q03252	Lamin-B2 OS=Homo sapiens GN=LMNB2 PE=1 SV=3 - (LMNB2_HUMAN)	36.07	15.67	4	1	9	25	9.794E7	600	67.6	5.35
Q6ZQQ6	WD repeat-containing protein 87 OS=Homo sapiens GN=WDR87 PE=2 SV=3 - (WDR87_HUMAN)	35.37	8.14	4	2	24	47	4.325E8	2873	333.0	7.28
Q9BYR4	Keratin-associated protein 4-3 OS=Homo sapiens GN=KRTAP4-3 PE=2 SV=2 - (KRA43_HUMAN)	35.29	12.82	1	2	2	11	7.516E7	195	20.5	7.94
Q5SQQ9	Ventral anterior homeobox 1 OS=Homo sapiens GN=VAX1 PE=1 SV=1 - (VAX1_HUMAN)	35.08	12.28	3	1	5	38	1.341E8	334	34.7	9.38
P18206-2	Isoform 1 of Vinculin OS=Homo sapiens GN=VCL - (VINC_HUMAN)	34.86	14.73	3	1	16	29	3.779E7	1066	116.6	6.09
Q59FI9	Ribosomal protein L12 variant (Fragment) OS=Homo sapiens PE=2 SV=1 - (Q59FI9_HUMAN)	34.66	49.24	4	4	9	18	1.408E8	197	21.5	10.10
Q08378-2	Isoform 2 of Golgin subfamily A member 3 OS=Homo sapiens GN=GOLGA3 - (GOGA3_HUMAN)	34.58	11.29	7	1	14	34	1.638E8	1390	155.6	5.40
Q59FC6	Tumor rejection antigen (Gp96) 1 variant (Fragment) OS=Homo sapiens PE=2 SV=1 - (Q59FC6_HUMAN)	34.51	23.44	10	5	12	17	3.857E8	576	65.9	5.19
P15090	Fatty acid-binding protein, adipocyte OS=Homo sapiens GN=FABP4 PE=1 SV=3 - (FABP4_HUMAN)	33.48	55.30	1	4	7	13	7.091E7	132	14.7	7.14
B3KWI4	cDNA FLJ43122 fis, clone CTONG3003737, highly similar to Leucine-rich repeat-containing protein 15 OS=Homo sapiens PE=2 SV=1 - (B3KWI4_HUMAN)	33.38	10.67	3	3	6	17	1.196E7	581	64.4	6.71
Q9H9L3	Interferon-stimulated 20 kDa exonuclease-like 2 OS=Homo sapiens GN=ISG20L2 PE=1 SV=1 - (I20L2_HUMAN)	32.69	5.10	1	1	2	38	6.381E7	353	39.1	9.94
Q52LG2	Keratin-associated protein 13-2 OS=Homo sapiens GN=KRTAP13-2 PE=1 SV=1 - (KR132_HUMAN)	32.60	29.14	2	5	5	13	4.336E7	175	18.7	8.29
C9J8H1	V-type proton ATPase subunit E 1 (Fragment) OS=Homo sapiens GN=ATP6V1E1 PE=4 SV=1 - (C9J8H1_HUMAN)	32.57	20.20	3	1	4	33	2.036E8	203	23.5	9.04
Q9NZE6	BM-010 OS=Homo sapiens GN=EIF4A2 PE=2 SV=1 - (Q9NZE6_HUMAN)	32.41	23.72	8	1	6	15	9.383E7	312	36.1	7.44
C9JQD1	Glucokinase OS=Homo sapiens GN=GCK PE=3 SV=1 - (C9JQD1_HUMAN)	32.23	6.92	5	1	2	15	3.621E8	448	50.1	5.27
C9K0F9	Histone acetyltransferase KAT6A (Fragment) OS=Homo sapiens GN=KAT6A PE=4 SV=1 - (C9K0F9_HUMAN)	32.03	4.81	1	1	4	23	2.735E7	728	84.1	6.55
Q5T749	Keratinocyte proline-rich protein OS=Homo sapiens GN=KPRP PE=1 SV=1 - (KPRP_HUMAN)	31.91	4.66	1	3	4	22	1.792E7	579	64.1	8.27
Q562Q2	Actin-like protein (Fragment) OS=Homo sapiens GN=ACT PE=3 SV=1 - (Q562Q2_HUMAN)	31.68	33.98	10	1	3	20	1.924E8	103	11.5	6.68
P62913-2	Isoform 2 of 60S ribosomal protein L11 OS=Homo sapiens GN=RPL11 - (RL11_HUMAN)	30.87	36.16	5	5	7	20	3.606E7	177	20.1	9.60
Q9H2F9	Coiled-coil domain-containing protein 68 OS=Homo sapiens GN=CCDC68 PE=2 SV=1 - (CCD68_HUMAN)	30.78	6.87	1	1	2	17	7.909E8	335	38.8	8.65
H0YL80	Tropomyosin alpha-1 chain (Fragment) OS=Homo sapiens GN=TPM1 PE=4 SV=1 - (H0YL80_HUMAN)	30.71	64.71	37	1	5	17	9.842E7	68	8.0	4.82
Q6ZU59	cDNA FLJ43980 fis, clone TESTI4018806 OS=Homo sapiens PE=2 SV=1 - (Q6ZU59_HUMAN)	30.71	13.29	1	1	2	19	2.071E8	143	16.6	6.80
Q15058	Kinesin-like protein KIF14 OS=Homo sapiens GN=KIF14 PE=1 SV=1 - (KIF14_HUMAN)	30.65	7.52	1	1	16	49	2.478E8	1648	186.4	7.91
B2R4P2	cDNA, FLJ92164, highly similar to Homo sapiens peroxiredoxin 1 (PRDX1), mRNA OS=Homo sapiens PE=2 SV=1 - (B2R4P2_HUMAN)	30.57	28.64	4	5	7	14	4.361E7	199	22.2	8.38
Q8TBY8	Polyamine-modulated factor 1-binding protein 1 OS=Homo sapiens GN=PMFBP1 PE=2 SV=1 - (PMFBP_HUMAN)	30.45	18.49	10	1	14	29	1.253E9	1022	119.0	6.29
B4DTD3	cDNA FLJ50209, highly similar to DNA-repair protein XRCC1 OS=Homo sapiens PE=2 SV=1 - (B4DTD3_HUMAN)	30.39	1.50	6	1	1	16	6.995E7	602	66.0	5.85
E9PJL5	Putative uncharacterized protein C12orf63 OS=Homo sapiens GN=C12orf63 PE=4 SV=1 - (E9PJL5_HUMAN)	30.12	5.75	2	1	13	21	1.704E8	3096	351.7	8.10
E9PK25	Cofilin-1 OS=Homo sapiens GN=CFL1 PE=4 SV=1 - (E9PK25_HUMAN)	30.08	36.76	10	4	7	16	2.170E8	204	22.7	8.34
I1E4Y4	Meiosis arrest female protein 1 OS=Homo sapiens GN=KIAA0430 PE=4 SV=1 - (I1E4Y4_HUMAN)	30.05	6.48	9	1	9	27	5.033E8	1682	186.2	7.99
Q5D862	Filaggrin-2 OS=Homo sapiens GN=FLG2 PE=1 SV=1 - (FILA2_HUMAN)	29.95	2.47	1	3	4	12	1.110E7	2391	247.9	8.31
E9PR44	Alpha-crystallin B chain (Fragment) OS=Homo sapiens GN=CRYAB PE=3 SV=1 - (E9PR44_HUMAN)	29.92	55.75	10	8	8	16	2.850E7	174	20.0	7.03
F5GZT0	Meiosis inhibitor protein 1 OS=Homo sapiens GN=MEI1 PE=4 SV=1 - (F5GZT0_HUMAN)	29.51	11.07	5	1	5	19	3.090E8	506	56.2	6.39
P62249	40S ribosomal protein S16 OS=Homo sapiens GN=RPS16 PE=1 SV=2 - (RS16_HUMAN)	29.40	52.05	3	6	9	20	3.020E9	146	16.4	10.21
Q8NB66	Protein unc-13 homolog C OS=Homo sapiens GN=UNC13C PE=1 SV=3 - (UN13C_HUMAN)	29.19	6.32	14	1	12	28	2.646E9	2214	250.8	5.92
P15822	Zinc finger protein 40 OS=Homo sapiens GN=HIVEP1 PE=1 SV=3 - (ZEP1_HUMAN)	29.05	6.99	1	1	14	44	3.930E9	2718	296.7	7.84
B4E1M1	cDNA FLJ60391, highly similar to Lactoperoxidase (EC 1.11.1.7) OS=Homo sapiens PE=2 SV=1 - (B4E1M1_HUMAN)	28.92	4.59	7	1	3	28	3.723E8	653	73.9	8.15
P12236	ADP/ATP translocase 3 OS=Homo sapiens GN=SLC25A6 PE=1 SV=4 - (ADT3_HUMAN)	28.71	25.84	9	5	8	21	1.100E8	298	32.8	9.74
Q96NX9-2	Isoform 2 of Dachshund homolog 2 OS=Homo sapiens GN=DACH2 - (DACH2_HUMAN)	28.67	13.13	5	1	3	27	1.654E8	571	61.9	8.65
Q01082	Spectrin beta chain, brain 1 OS=Homo sapiens GN=SPTBN1 PE=1 SV=2 - (SPTB2_HUMAN)	28.63	5.75	4	1	13	29	6.750E8	2364	274.4	5.57
P09211	Glutathione S-transferase P OS=Homo sapiens GN=GSTP1 PE=1 SV=2 - (GSTP1_HUMAN)	28.38	42.38	4	5	7	15	6.725E7	210	23.3	5.64
C9K0X2	Uncharacterized protein OS=Homo sapiens PE=4 SV=1 - (C9K0X2_HUMAN)	28.29	5.92	4	2	2	33	2.961E8	152	15.7	6.33
D6R9P1	Ectonucleotide pyrophosphatase/phosphodiesterase family member 6 soluble form (Fragment) OS=Homo sapiens GN=ENPP6 PE=4 SV=1 - (D6R9P1_HUMAN)	28.06	14.95	2	1	3	21	4.154E8	194	22.3	6.93
H0YF27	Tripartite motif-containing protein 29 (Fragment) OS=Homo sapiens GN=TRIM29 PE=4 SV=1 - (H0YF27_HUMAN)	28.00	28.99	7	1	4	17	2.188E7	169	18.7	9.92
P22392-2	Isoform 3 of Nucleoside diphosphate kinase B OS=Homo sapiens GN=NME2 - (NDKB_HUMAN)	27.98	46.44	10	3	8	16	3.467E7	267	30.1	8.92
B4DSW9	cDNA FLJ59415, highly similar to Beta-catenin OS=Homo sapiens PE=2 SV=1 - (B4DSW9_HUMAN)	27.98	14.25	6	1	8	27	2.860E7	709	77.5	6.38
Q9P2K1	Coiled-coil and C2 domain-containing protein 2A OS=Homo sapiens GN=CC2D2A PE=1 SV=3 - (C2D2A_HUMAN)	27.86	8.77	2	1	13	25	1.172E8	1620	186.1	6.74
O60437	Periplakin OS=Homo sapiens GN=PPL PE=1 SV=4 - (PEPL_HUMAN)	27.71	13.21	1	1	23	35	2.351E8	1756	204.6	5.60
A8MXJ1	Protein STON1-GTF2A1L OS=Homo sapiens GN=STON1-GTF2A1L PE=4 SV=3 - (A8MXJ1_HUMAN)	27.30	2.29	8	1	3	15	5.981E7	1135	127.2	5.31
A6NCL7	Ankyrin repeat domain-containing protein 33B OS=Homo sapiens GN=ANKRD33B PE=4 SV=1 - (AN33B_HUMAN)	27.28	11.54	2	1	6	21	3.270E8	494	53.9	8.00
Q8IW70	Transmembrane protein 151B OS=Homo sapiens GN=TMEM151B PE=2 SV=2 - (T151B_HUMAN)	27.24	4.95	1	1	3	13	2.075E7	566	61.5	7.14
E7EVW9	Oxygen-regulated protein 1 OS=Homo sapiens GN=RP1 PE=4 SV=1 - (E7EVW9_HUMAN)	27.23	8.30	3	1	7	19	4.358E8	916	102.5	7.71
Q01518-2	Isoform 2 of Adenylyl cyclase-associated protein 1 OS=Homo sapiens GN=CAP1 - (CAP1_HUMAN)	26.84	23.42	18	2	6	9	2.225E7	474	51.8	8.06
P05109	Protein S100-A8 OS=Homo sapiens GN=S100A8 PE=1 SV=1 - (S10A8_HUMAN)	26.73	44.09	1	4	5	16	1.190E8	93	10.8	7.03
Q00610-2	Isoform 2 of Clathrin heavy chain 1 OS=Homo sapiens GN=CLTC - (CLH1_HUMAN)	26.72	5.49	4	2	8	18	1.168E8	1639	187.8	5.69
P39019	40S ribosomal protein S19 OS=Homo sapiens GN=RPS19 PE=1 SV=2 - (RS19_HUMAN)	26.50	37.93	2	5	8	17	3.478E7	145	16.1	10.32
Q3LI72	Keratin-associated protein 19-5 OS=Homo sapiens GN=KRTAP19-5 PE=1 SV=1 - (KR195_HUMAN)	26.46	52.78	1	2	2	5	1.927E8	72	7.6	8.13
Q6P2D8-3	Isoform 3 of X-ray radiation resistance-associated protein 1 OS=Homo sapiens GN=XRRA1 - (XRRA1_HUMAN)	26.32	18.51	2	1	11	31	6.634E7	778	88.3	9.03
B4DLJ7	cDNA FLJ59334, highly similar to Transmembrane glycoprotein NMB OS=Homo sapiens PE=2 SV=1 - (B4DLJ7_HUMAN)	26.31	11.09	11	2	5	16	1.813E8	514	57.2	6.77
Q9Y2F5	Uncharacterized protein KIAA0947 OS=Homo sapiens GN=KIAA0947 PE=1 SV=5 - (K0947_HUMAN)	26.17	5.25	1	1	13	28	6.153E8	2266	247.7	5.48
A7MCY6	TANK-binding kinase 1-binding protein 1 OS=Homo sapiens GN=TBKBP1 PE=1 SV=1 - (TBKB1_HUMAN)	26.02	4.39	4	2	4	18	2.203E7	615	67.7	5.86
P45880-2	Isoform 2 of Voltage-dependent anion-selective channel protein 2 OS=Homo sapiens GN=VDAC2 - (VDAC2_HUMAN)	26.02	25.44	7	6	7	16	2.864E7	283	30.4	7.20
B7ZKX5	KIF6 protein OS=Homo sapiens GN=KIF6 PE=2 SV=1 - (B7ZKX5_HUMAN)	25.91	9.54	12	2	7	15	7.201E7	797	90.5	6.95
Q08188	Protein-glutamine gamma-glutamyltransferase E OS=Homo sapiens GN=TGM3 PE=1 SV=4 - (TGM3_HUMAN)	25.71	11.69	3	3	6	17	2.564E7	693	76.6	5.86
B7ZVZ7	BZRAP1 protein OS=Homo sapiens GN=BZRAP1 PE=2 SV=1 - (B7ZVZ7_HUMAN)	25.61	8.77	7	1	15	24	8.759E7	1848	199.0	5.11
Q96M42	Putative uncharacterized protein encoded by LINC00479 OS=Homo sapiens GN=LINC00479 PE=5 SV=2 - (CU129_HUMAN)	25.61	4.93	1	1	1	20	8.705E7	142	15.2	8.00
B7Z2X4	cDNA FLJ53327, highly similar to Gelsolin OS=Homo sapiens PE=2 SV=1 - (B7Z2X4_HUMAN)	25.18	13.33	18	3	10	17	1.357E8	705	77.7	5.69
Q9Y3P9-3	Isoform 3 of Rab GTPase-activating protein 1 OS=Homo sapiens GN=RABGAP1 - (RBGP1_HUMAN)	25.18	5.43	4	1	3	12	9.316E7	479	53.2	5.16
E5RIY5	Serine protease 45 OS=Homo sapiens GN=PRSS45 PE=3 SV=1 - (E5RIY5_HUMAN)	24.80	8.18	3	1	1	14	5.165E8	110	12.4	7.59
Q96L12	Calreticulin-3 OS=Homo sapiens GN=CALR3 PE=1 SV=2 - (CALR3_HUMAN)	24.51	3.91	1	1	2	20	2.736E8	384	45.0	6.65
P62269	40S ribosomal protein S18 OS=Homo sapiens GN=RPS18 PE=1 SV=3 - (RS18_HUMAN)	24.17	35.53	2	6	6	12	1.231E7	152	17.7	10.99
Q8N4C6-10	Isoform 3 of Ninein OS=Homo sapiens GN=NIN - (NIN_HUMAN)	23.93	8.84	3	1	17	33	2.942E8	2116	246.0	5.03
A4D1E1	Zinc finger protein 804B OS=Homo sapiens GN=ZNF804B PE=1 SV=2 - (Z804B_HUMAN)	23.90	7.12	1	1	8	15	4.792E7	1349	152.5	8.54
Q8IWB6-3	Isoform 3 of Inactive serine/threonine-protein kinase TEX14 OS=Homo sapiens GN=TEX14 - (TEX14_HUMAN)	23.87	5.79	3	2	8	17	1.390E8	1451	162.6	5.25
Q96IU2	Zinc finger BED domain-containing protein 3 OS=Homo sapiens GN=ZBED3 PE=1 SV=1 - (ZBED3_HUMAN)	23.67	22.22	2	2	5	21	2.055E9	234	25.1	8.31
Q9Y446	Plakophilin-3 OS=Homo sapiens GN=PKP3 PE=1 SV=1 - (PKP3_HUMAN)	23.64	21.58	8	7	15	19	6.050E7	797	87.0	9.32
B2R7G4	cDNA, FLJ93435, highly similar to Homo sapiens general transcription factor IIH, polypeptide 1 (62kD subunit) (GTF2H1), mRNA OS=Homo sapiens PE=2 SV=1 - (B2R7G4_HUMAN)	23.48	6.39	4	1	3	14	7.506E7	548	62.0	8.84
Q59GY2	Ribosomal protein L4 variant (Fragment) OS=Homo sapiens PE=2 SV=1 - (Q59GY2_HUMAN)	23.47	39.00	6	1	14	24	2.741E8	441	49.0	11.02
B3KX91	cDNA FLJ44992 fis, clone BRAWH3009961, highly similar to Regulating synaptic membrane exocytosis protein 2 OS=Homo sapiens PE=2 SV=1 - (B3KX91_HUMAN)	23.45	12.01	9	1	12	28	7.013E8	1349	153.2	9.20
Q04656-5	Isoform 5 of Copper-transporting ATPase 1 OS=Homo sapiens GN=ATP7A - (ATP7A_HUMAN)	23.39	5.20	6	1	5	29	6.077E8	1422	154.3	6.05
P04075	Fructose-bisphosphate aldolase A OS=Homo sapiens GN=ALDOA PE=1 SV=2 - (ALDOA_HUMAN)	23.34	30.22	21	9	11	15	1.807E7	364	39.4	8.09
B3KXG3	cDNA FLJ45362 fis, clone BRHIP3014675, highly similar to Homo sapiens BTB (POZ) domain containing 11 (BTBD11), transcript variant 2, mRNA OS=Homo sapiens PE=2 SV=1 - (B3KXG3_HUMAN)	23.24	6.40	2	1	4	21	3.979E7	641	71.9	6.87
B7ZW38	LOC649330 protein OS=Homo sapiens GN=LOC649330 PE=2 SV=1 - (B7ZW38_HUMAN)	23.23	20.14	29	1	6	18	2.361E9	293	32.0	5.68
P11021	78 kDa glucose-regulated protein OS=Homo sapiens GN=HSPA5 PE=1 SV=2 - (GRP78_HUMAN)	23.15	18.96	3	3	12	14	4.868E7	654	72.3	5.16
Q5VT06	Centrosome-associated protein 350 OS=Homo sapiens GN=CEP350 PE=1 SV=1 - (CE350_HUMAN)	22.78	7.73	5	1	22	33	3.457E8	3117	350.7	6.33
P32456	Interferon-induced guanylate-binding protein 2 OS=Homo sapiens GN=GBP2 PE=2 SV=3 - (GBP2_HUMAN)	22.77	5.25	1	1	4	15	1.614E8	591	67.2	5.71
F8W805	Metabotropic glutamate receptor 1 OS=Homo sapiens GN=GRM1 PE=3 SV=1 - (F8W805_HUMAN)	22.56	8.37	4	1	7	19	2.275E9	908	101.4	8.13
Q9UG77	Putative uncharacterized protein DKFZp586K091 (Fragment) OS=Homo sapiens GN=DKFZp586K091 PE=2 SV=1 - (Q9UG77_HUMAN)	22.48	5.76	6	1	2	15	2.079E9	434	45.0	9.55
B2RUU3	Dedicator of cytokinesis 1 OS=Homo sapiens GN=DOCK1 PE=2 SV=1 - (B2RUU3_HUMAN)	22.38	6.33	2	1	12	22	1.804E9	1865	215.2	7.49
B3KSS3	cDNA FLJ36854 fis, clone ASTRO2014561, highly similar to Peroxisomal membrane protein 11B OS=Homo sapiens PE=2 SV=1 - (B3KSS3_HUMAN)	22.35	20.47	4	1	2	10	1.204E7	127	13.4	9.48
P62851	40S ribosomal protein S25 OS=Homo sapiens GN=RPS25 PE=1 SV=1 - (RS25_HUMAN)	21.88	32.80	1	3	4	12	2.845E7	125	13.7	10.11
Q6IBG5	MYL6 protein OS=Homo sapiens GN=MYL6 PE=2 SV=1 - (Q6IBG5_HUMAN)	21.85	50.00	20	4	6	9	3.389E7	116	13.0	4.70
P62136-2	Isoform 2 of Serine/threonine-protein phosphatase PP1-alpha catalytic subunit OS=Homo sapiens GN=PPP1CA - (PP1A_HUMAN)	21.77	7.92	19	1	3	19	1.620E7	341	38.6	6.62
Q8N1G2	Cap-specific mRNA (nucleoside-2'-O-)-methyltransferase 1 OS=Homo sapiens GN=FTSJD2 PE=1 SV=1 - (MTR1_HUMAN)	21.73	5.03	1	1	4	17	7.702E7	835	95.3	7.05
Q6YHK3-4	Isoform 4 of CD109 antigen OS=Homo sapiens GN=CD109 - (CD109_HUMAN)	21.40	8.33	4	2	10	14	1.350E8	1428	159.6	5.80
B4DE24	cDNA FLJ52155, moderately similar to Rattus norvegicus ring finger protein 34 (Rnf34), mRNA OS=Homo sapiens PE=2 SV=1 - (B4DE24_HUMAN)	21.36	3.21	2	1	1	16	8.020E8	280	31.4	8.38
O14578	Citron Rho-interacting kinase OS=Homo sapiens GN=CIT PE=1 SV=2 - (CTRO_HUMAN)	21.28	9.08	4	1	16	26	7.326E7	2027	231.3	6.57
A5D8W1-2	Isoform 2 of Uncharacterized protein C7orf63 OS=Homo sapiens GN=C7orf63 - (CG063_HUMAN)	21.28	7.04	7	1	5	21	3.221E9	895	100.7	7.05
I3L3E2	Multidrug and toxin extrusion protein 1 (Fragment) OS=Homo sapiens GN=SLC47A1 PE=4 SV=1 - (I3L3E2_HUMAN)	21.28	11.18	8	1	2	13	5.649E8	161	17.1	5.82
Q9Y493-7	Isoform 7 of Zonadhesin OS=Homo sapiens GN=ZAN - (ZAN_HUMAN)	21.25	4.85	10	1	9	18	9.541E7	2866	310.9	5.90
B3KXD3	cDNA FLJ45230 fis, clone BRCAN2021325, highly similar to Carboxypeptidase E (EC 3.4.17.10) OS=Homo sapiens PE=2 SV=1 - (B3KXD3_HUMAN)	21.25	15.91	6	1	5	18	2.095E8	421	47.3	4.97
Q8TCY9-3	Isoform 3 of Up-regulator of cell proliferation OS=Homo sapiens GN=URGCP - (URGCP_HUMAN)	21.17	5.37	6	1	6	24	4.856E7	856	96.6	7.30
F8WCF6	Actin-related protein 2/3 complex subunit 4 OS=Homo sapiens GN=ARPC4 PE=4 SV=1 - (F8WCF6_HUMAN)	21.08	26.52	8	3	6	11	3.927E8	181	21.0	8.76
A8K7H3	cDNA FLJ77670, highly similar to Homo sapiens ribosomal protein S15a (RPS15A), mRNA OS=Homo sapiens PE=2 SV=1 - (A8K7H3_HUMAN)	21.06	50.77	4	3	8	23	2.134E8	130	14.8	10.13
Q6P084	MAP3K2 protein (Fragment) OS=Homo sapiens GN=MAP3K2 PE=2 SV=1 - (Q6P084_HUMAN)	20.98	16.24	1	1	3	15	7.954E7	234	26.8	9.23
Q5T7N8	Protein FAM27D1 OS=Homo sapiens GN=FAM27D1 PE=3 SV=2 - (F27D1_HUMAN)	20.88	20.93	5	1	5	14	6.277E7	215	24.9	11.93
Q9ULT8	E3 ubiquitin-protein ligase HECTD1 OS=Homo sapiens GN=HECTD1 PE=1 SV=3 - (HECD1_HUMAN)	20.88	5.21	5	1	9	21	5.826E8	2610	289.2	5.35
P62277	40S ribosomal protein S13 OS=Homo sapiens GN=RPS13 PE=1 SV=2 - (RS13_HUMAN)	20.88	41.06	2	3	6	11	6.411E8	151	17.2	10.54
F8VWT9	Probable E3 ubiquitin-protein ligase C12orf51 OS=Homo sapiens GN=C12orf51 PE=4 SV=1 - (F8VWT9_HUMAN)	20.82	4.61	7	1	14	17	9.174E8	4272	468.4	6.14
Q9UGU0-2	Isoform 2 of Transcription factor 20 OS=Homo sapiens GN=TCF20 - (TCF20_HUMAN)	20.78	10.22	3	1	17	32	2.369E8	1938	209.5	9.09
O15230	Laminin subunit alpha-5 OS=Homo sapiens GN=LAMA5 PE=1 SV=8 - (LAMA5_HUMAN)	20.54	4.93	2	1	13	21	1.137E8	3695	399.5	7.02
B7ZLW1	CAMSAP1 protein OS=Homo sapiens GN=CAMSAP1 PE=2 SV=1 - (B7ZLW1_HUMAN)	20.46	9.34	4	1	11	29	6.517E8	1467	163.2	6.95
P67936-2	Isoform 2 of Tropomyosin alpha-4 chain OS=Homo sapiens GN=TPM4 - (TPM4_HUMAN)	20.35	22.54	58	2	6	15	8.255E7	284	32.7	4.73
A8MYJ7	Tetratricopeptide repeat protein 34 OS=Homo sapiens GN=TTC34 PE=2 SV=2 - (TTC34_HUMAN)	20.14	13.60	1	1	7	14	5.101E7	566	60.9	7.33
H7C292	Phosphatidylinositol 3,4,5-trisphosphate 5-phosphatase 1 OS=Homo sapiens GN=INPP5D PE=4 SV=1 - (H7C292_HUMAN)	19.92	14.56	8	1	12	54	3.982E8	941	105.2	8.56
E7ENF9	Isochorismatase domain-containing protein 1 (Fragment) OS=Homo sapiens GN=ISOC1 PE=4 SV=1 - (E7ENF9_HUMAN)	19.82	24.27	5	1	5	52	3.937E8	239	26.4	7.77
O95466-2	Isoform 2 of Formin-like protein 1 OS=Homo sapiens GN=FMNL1 - (FMNL_HUMAN)	19.76	10.78	5	1	11	18	5.489E7	1104	122.3	5.83
P0CG32	Putative zinc finger CCHC domain-containing protein 18 OS=Homo sapiens GN=ZCCHC18 PE=5 SV=1 - (ZCC18_HUMAN)	19.56	14.39	1	1	4	22	2.751E8	403	45.1	7.39
Q14574-2	Isoform 3B of Desmocollin-3 OS=Homo sapiens GN=DSC3 - (DSC3_HUMAN)	19.55	10.13	3	2	8	19	1.644E8	839	93.4	6.32
Q9NT68	Teneurin-2 OS=Homo sapiens GN=ODZ2 PE=1 SV=3 - (TEN2_HUMAN)	19.50	5.48	7	2	14	28	7.438E7	2774	307.6	6.68
Q9C0C7-2	Isoform 2 of Activating molecule in BECN1-regulated autophagy protein 1 OS=Homo sapiens GN=AMBRA1 - (AMRA1_HUMAN)	19.44	8.40	8	1	9	25	1.509E8	1238	135.4	7.18
Q5H9J9	Novel protein similar to t-complex 11 homolog (Mouse) TCP11 OS=Homo sapiens GN=LL0XNC01-19D8.1 PE=4 SV=1 - (Q5H9J9_HUMAN)	19.35	4.91	2	1	2	10	1.737E8	407	46.3	5.19
E9PBV3	Suprabasin OS=Homo sapiens GN=SBSN PE=4 SV=1 - (E9PBV3_HUMAN)	19.18	20.68	1	2	5	9	1.390E7	590	60.5	7.01
P46782	40S ribosomal protein S5 OS=Homo sapiens GN=RPS5 PE=1 SV=4 - (RS5_HUMAN)	19.07	16.18	2	2	3	7	3.269E7	204	22.9	9.72
P68402	Platelet-activating factor acetylhydrolase IB subunit beta OS=Homo sapiens GN=PAFAH1B2 PE=1 SV=1 - (PA1B2_HUMAN)	19.03	8.30	1	1	2	12	4.630E7	229	25.6	5.92
Q53GR6	SH3 domain and tetratricopeptide repeats 1 variant (Fragment) OS=Homo sapiens PE=2 SV=1 - (Q53GR6_HUMAN)	18.89	7.78	6	1	8	16	8.443E7	1195	131.8	6.09
P28838-2	Isoform 2 of Cytosol aminopeptidase OS=Homo sapiens GN=LAP3 - (AMPL_HUMAN)	18.88	9.84	4	2	5	11	2.540E8	488	52.7	6.74
B4DMA2	cDNA FLJ54023, highly similar to Heat shock protein HSP 90-beta OS=Homo sapiens PE=2 SV=1 - (B4DMA2_HUMAN)	18.73	13.99	29	3	9	13	1.482E7	686	79.1	5.02
Q6PIB3	KN motif and ankyrin repeat domains 1 OS=Homo sapiens GN=KANK1 PE=2 SV=1 - (Q6PIB3_HUMAN)	18.57	11.56	7	1	11	18	1.191E8	1194	129.6	5.14
Q96EA4	Protein Spindly OS=Homo sapiens GN=CCDC99 PE=1 SV=2 - (SPDLY_HUMAN)	18.50	12.73	6	1	8	22	9.748E7	605	70.1	5.47
Q9BYR3	Keratin-associated protein 4-4 OS=Homo sapiens GN=KRTAP4-4 PE=2 SV=1 - (KRA44_HUMAN)	18.47	14.46	1	2	2	16	1.004E8	166	18.0	7.91
Q12766	HMG domain-containing protein 3 OS=Homo sapiens GN=HMGXB3 PE=2 SV=2 - (HMGX3_HUMAN)	18.33	4.62	4	1	8	18	1.322E9	1538	168.2	8.05
O75976	Carboxypeptidase D OS=Homo sapiens GN=CPD PE=1 SV=2 - (CBPD_HUMAN)	18.16	4.49	5	1	7	21	3.757E8	1380	152.8	6.05
Q5VU13	V-set and immunoglobulin domain-containing protein 8 OS=Homo sapiens GN=VSIG8 PE=1 SV=1 - (VSIG8_HUMAN)	18.01	14.01	1	3	4	13	1.779E7	414	43.9	7.20
I3NI21	Promethin (Fragment) OS=Homo sapiens GN=TMEM159 PE=4 SV=1 - (I3NI21_HUMAN)	17.93	17.39	5	1	1	13	8.020E8	46	5.2	5.24
Q8N3C0	Activating signal cointegrator 1 complex subunit 3 OS=Homo sapiens GN=ASCC3 PE=1 SV=3 - (ASCC3_HUMAN)	17.83	8.27	1	1	16	32	3.274E8	2202	251.3	7.09
Q96HZ4-2	Isoform 2 of Transcription cofactor HES-6 OS=Homo sapiens GN=HES6 - (HES6_HUMAN)	17.73	23.36	2	1	4	10	8.772E7	214	23.5	11.94
P07476	Involucrin OS=Homo sapiens GN=IVL PE=1 SV=2 - (INVO_HUMAN)	17.70	8.55	3	2	4	12	3.193E6	585	68.4	4.61
Q0QEN7	ATP synthase subunit beta (Fragment) OS=Homo sapiens GN=ATP5B PE=2 SV=1 - (Q0QEN7_HUMAN)	17.64	20.45	7	4	6	6	2.008E7	445	48.1	5.07
Q53EQ3	Tubulin, gamma complex associated protein 2 variant (Fragment) OS=Homo sapiens PE=2 SV=1 - (Q53EQ3_HUMAN)	17.56	9.54	7	1	7	15	2.910E8	901	102.4	6.84
A8K884	cDNA FLJ77178 OS=Homo sapiens PE=2 SV=1 - (A8K884_HUMAN)	17.54	20.15	3	1	11	25	3.910E8	551	65.7	8.70
P25705	ATP synthase subunit alpha, mitochondrial OS=Homo sapiens GN=ATP5A1 PE=1 SV=1 - (ATPA_HUMAN)	17.53	11.39	4	4	5	6	1.745E7	553	59.7	9.13
B4DXF3	cDNA FLJ57650, highly similar to Bleomycin hydrolase (EC 3.4.22.40) OS=Homo sapiens PE=2 SV=1 - (B4DXF3_HUMAN)	17.41	14.40	3	1	3	10	4.359E7	368	42.4	6.25
Q70J99	Protein unc-13 homolog D OS=Homo sapiens GN=UNC13D PE=1 SV=1 - (UN13D_HUMAN)	17.35	6.42	3	1	5	12	3.720E8	1090	123.2	6.65
B4DE44	cDNA FLJ52880, highly similar to Malate dehydrogenase, mitochondrial (EC 1.1.1.37) OS=Homo sapiens PE=2 SV=1 - (B4DE44_HUMAN)	17.34	20.95	6	1	4	7	3.142E7	296	30.9	8.43
Q659C4-9	Isoform 9 of La-related protein 1B OS=Homo sapiens GN=LARP1B - (LAR1B_HUMAN)	17.34	6.73	10	1	6	18	6.376E7	787	90.3	7.44
Q15365	Poly(rC)-binding protein 1 OS=Homo sapiens GN=PCBP1 PE=1 SV=2 - (PCBP1_HUMAN)	17.26	10.96	10	1	4	13	1.746E8	356	37.5	7.09
Q8N1A4	Similar to phospholipase C, beta 3 (Phosphatidylinositol-specific) (Fragment) OS=Homo sapiens PE=2 SV=1 - (Q8N1A4_HUMAN)	16.92	4.53	3	1	6	12	1.408E8	1191	133.4	6.11
Q6FG99	RPLP1 protein OS=Homo sapiens GN=RPLP1 PE=4 SV=1 - (Q6FG99_HUMAN)	16.89	21.93	4	1	2	5	1.372E7	114	11.6	4.37
Q92805	Golgin subfamily A member 1 OS=Homo sapiens GN=GOLGA1 PE=1 SV=3 - (GOGA1_HUMAN)	16.86	13.43	2	1	7	17	8.890E7	767	88.1	5.27
P18124	60S ribosomal protein L7 OS=Homo sapiens GN=RPL7 PE=1 SV=1 - (RL7_HUMAN)	16.86	23.79	5	2	7	11	3.404E8	248	29.2	10.65
Q9NVU3	cDNA FLJ10506 fis, clone NT2RP2000510 OS=Homo sapiens PE=2 SV=1 - (Q9NVU3_HUMAN)	16.82	6.48	5	1	5	17	7.655E7	864	96.3	6.65
E9PI98	Layilin (Fragment) OS=Homo sapiens GN=LAYN PE=4 SV=1 - (E9PI98_HUMAN)	16.48	29.69	1	1	2	9	3.501E7	64	7.4	8.00
Q5T653	39S ribosomal protein L2, mitochondrial OS=Homo sapiens GN=MRPL2 PE=1 SV=2 - (RM02_HUMAN)	16.46	9.51	1	1	3	10	8.311E7	305	33.3	11.30
P23284	Peptidyl-prolyl cis-trans isomerase B OS=Homo sapiens GN=PPIB PE=1 SV=2 - (PPIB_HUMAN)	16.38	27.78	8	4	6	9	3.879E7	216	23.7	9.41
P12273	Prolactin-inducible protein OS=Homo sapiens GN=PIP PE=1 SV=1 - (PIP_HUMAN)	16.30	26.03	1	3	4	6	7.798E5	146	16.6	8.05
F8W6N8	Tripartite motif-containing protein 5 OS=Homo sapiens GN=TRIM5 PE=4 SV=1 - (F8W6N8_HUMAN)	16.23	12.54	1	1	3	10	2.565E7	327	37.8	5.86
Q9ULL5-2	Isoform 2 of Proline-rich protein 12 OS=Homo sapiens GN=PRR12 - (PRR12_HUMAN)	16.23	5.07	4	1	4	12	5.244E7	1143	121.8	7.88
Q8N4Y2	EF-hand calcium-binding domain-containing protein 4A OS=Homo sapiens GN=EFCAB4A PE=2 SV=3 - (EFC4A_HUMAN)	16.12	18.55	2	2	8	20	7.220E7	399	44.9	5.34
Q6PCE3	Glucose 1,6-bisphosphate synthase OS=Homo sapiens GN=PGM2L1 PE=1 SV=3 - (PGM2L_HUMAN)	16.10	3.86	1	1	2	8	3.602E8	622	70.4	7.15
Q4L180-2	Isoform 2 of Filamin A-interacting protein 1-like OS=Homo sapiens GN=FILIP1L - (FIL1L_HUMAN)	16.03	9.00	7	1	10	18	2.135E8	1133	130.1	6.61
P55157	Microsomal triglyceride transfer protein large subunit OS=Homo sapiens GN=MTTP PE=1 SV=1 - (MTP_HUMAN)	15.93	4.81	4	1	4	13	5.321E7	894	99.3	8.41
G3V5X3	Cochlin (Fragment) OS=Homo sapiens GN=COCH PE=4 SV=1 - (G3V5X3_HUMAN)	15.76	17.82	7	1	3	12	5.266E8	202	21.7	9.11
H0YF99	Heparan sulfate glucosamine 3-O-sulfotransferase 2 OS=Homo sapiens GN=HS3ST2 PE=4 SV=1 - (H0YF99_HUMAN)	15.74	15.99	5	1	4	9	3.042E7	344	38.3	10.05
B2RBR9	cDNA, FLJ95650, highly similar to Homo sapiens karyopherin (importin) beta 1 (KPNB1), mRNA OS=Homo sapiens PE=2 SV=1 - (B2RBR9_HUMAN)	15.72	9.02	6	2	5	10	4.515E6	876	97.1	4.78
F8WD26	LIM domain only protein 7 OS=Homo sapiens GN=LMO7 PE=4 SV=1 - (F8WD26_HUMAN)	15.66	11.96	28	2	19	33	4.551E8	1614	185.0	7.97
Q9BTQ7	Similar to ribosomal protein L23 (Fragment) OS=Homo sapiens PE=1 SV=1 - (Q9BTQ7_HUMAN)	15.65	47.01	4	5	7	7	1.467E7	134	14.1	10.26
Q96NL6-3	Isoform 3 of Sodium channel and clathrin linker 1 OS=Homo sapiens GN=SCLT1 - (SCLT1_HUMAN)	15.49	29.14	4	1	4	16	9.913E7	175	20.5	5.69
P08195-2	Isoform 2 of 4F2 cell-surface antigen heavy chain OS=Homo sapiens GN=SLC3A2 - (4F2_HUMAN)	15.46	13.23	7	1	4	7	1.446E7	529	57.9	5.35
Q6ZMW3-2	Isoform 2 of Echinoderm microtubule-associated protein-like 6 OS=Homo sapiens GN=EML6 - (EMAL6_HUMAN)	15.30	3.15	2	1	6	14	7.311E8	1874	208.7	7.85
P62263	40S ribosomal protein S14 OS=Homo sapiens GN=RPS14 PE=1 SV=3 - (RS14_HUMAN)	15.25	25.17	3	1	5	6	2.331E7	151	16.3	10.05
P63162	Small nuclear ribonucleoprotein-associated protein N OS=Homo sapiens GN=SNRPN PE=1 SV=1 - (RSMN_HUMAN)	14.97	36.67	12	2	7	14	3.440E7	240	24.6	11.19
H0YH34	ADP-ribosylation factor-like protein 13A (Fragment) OS=Homo sapiens GN=ARL13A PE=4 SV=1 - (H0YH34_HUMAN)	14.69	8.06	3	1	1	7	9.563E8	124	14.2	9.64
Q96IR1	RPS4X protein (Fragment) OS=Homo sapiens GN=RPS4X PE=2 SV=2 - (Q96IR1_HUMAN)	14.64	18.11	9	5	5	7	1.697E7	243	27.2	9.94
D5FHQ7	MHC class I antigen (Fragment) OS=Homo sapiens GN=HLA-A PE=3 SV=1 - (D5FHQ7_HUMAN)	14.56	7.73	1	1	1	7	2.901E8	181	21.1	6.93
Q8IY92	Structure-specific endonuclease subunit SLX4 OS=Homo sapiens GN=SLX4 PE=1 SV=3 - (SLX4_HUMAN)	14.56	4.80	2	1	9	28	8.799E6	1834	199.9	6.06
B3KWN0	cDNA FLJ43389 fis, clone OCBBF2007068, highly similar to Ankyrin repeat domain-containing protein 52 OS=Homo sapiens PE=2 SV=1 - (B3KWN0_HUMAN)	14.47	3.62	2	1	3	13	6.598E7	1076	115.0	6.51
Q86VG2	Splicing factor proline/glutamine-rich (Polypyrimidine tract binding protein associated) OS=Homo sapiens GN=SFPQ PE=2 SV=1 - (Q86VG2_HUMAN)	14.46	14.57	27	2	10	20	1.555E7	707	76.1	9.48
F1T0K4	DmX-like protein 1 OS=Homo sapiens GN=DMXL1 PE=2 SV=1 - (F1T0K4_HUMAN)	14.45	3.50	4	1	8	17	2.526E9	2854	318.4	6.34
A8K1B1	cDNA FLJ75211, highly similar to Homo sapiens ubiquitin specific peptidase like 1, mRNA OS=Homo sapiens PE=2 SV=1 - (A8K1B1_HUMAN)	14.40	3.75	4	1	3	11	6.908E7	1092	120.3	6.19
P11047	Laminin subunit gamma-1 OS=Homo sapiens GN=LAMC1 PE=1 SV=3 - (LAMC1_HUMAN)	14.39	7.58	1	1	10	16	3.952E8	1609	177.5	5.12
B2R4M6	cDNA, FLJ92148, highly similar to Homo sapiens S100 calcium binding protein A9 (calgranulin B) (S100A9), mRNA OS=Homo sapiens PE=4 SV=1 - (B2R4M6_HUMAN)	14.36	42.11	2	1	4	8	1.319E7	114	13.2	6.13
P04083	Annexin A1 OS=Homo sapiens GN=ANXA1 PE=1 SV=2 - (ANXA1_HUMAN)	14.27	23.70	5	3	8	10	1.275E8	346	38.7	7.02
A8MW50	L-lactate dehydrogenase (Fragment) OS=Homo sapiens GN=LDHB PE=3 SV=1 - (A8MW50_HUMAN)	14.24	11.21	11	1	3	8	3.729E7	232	25.2	5.81
Q5VYY1	Ankyrin repeat domain-containing protein 22 OS=Homo sapiens GN=ANKRD22 PE=2 SV=1 - (ANR22_HUMAN)	14.16	18.32	1	1	4	11	7.051E7	191	21.8	8.84
O60744	Thioredoxin delta 3 (Fragment) OS=Homo sapiens GN=TXN delta 3 PE=2 SV=1 - (O60744_HUMAN)	14.00	50.00	3	4	5	6	4.216E7	84	9.3	6.04
E5RFM1	Uncharacterized protein OS=Homo sapiens PE=4 SV=1 - (E5RFM1_HUMAN)	13.99	12.50	2	1	1	8	3.384E6	64	7.2	9.85
F5GXU8	Leucine-zipper-like transcriptional regulator 1 OS=Homo sapiens GN=LZTR1 PE=4 SV=1 - (F5GXU8_HUMAN)	13.97	9.39	6	1	7	17	7.206E7	799	90.6	6.64
Q9Y2U5	Mitogen-activated protein kinase kinase kinase 2 OS=Homo sapiens GN=MAP3K2 PE=1 SV=2 - (M3K2_HUMAN)	13.96	11.95	2	1	8	26	1.002E8	619	69.7	8.00
B1AMD2	Ring finger and WD repeat domain 2 OS=Homo sapiens GN=RFWD2 PE=4 SV=1 - (B1AMD2_HUMAN)	13.96	6.11	4	1	4	9	1.692E8	491	55.6	6.35
Q86Y13	E3 ubiquitin-protein ligase DZIP3 OS=Homo sapiens GN=DZIP3 PE=1 SV=2 - (DZIP3_HUMAN)	13.85	8.03	5	1	9	15	1.385E7	1208	138.5	6.87
B4DUQ1	cDNA FLJ54552, highly similar to Heterogeneous nuclear ribonucleoprotein K OS=Homo sapiens PE=2 SV=1 - (B4DUQ1_HUMAN)	13.76	35.76	9	2	11	22	3.334E8	439	48.5	5.92
P62857	40S ribosomal protein S28 OS=Homo sapiens GN=RPS28 PE=1 SV=1 - (RS28_HUMAN)	13.59	62.32	1	3	4	8	1.913E7	69	7.8	10.70
A8MXN1	Receptor expression-enhancing protein 6 OS=Homo sapiens GN=REEP6 PE=4 SV=1 - (A8MXN1_HUMAN)	13.57	11.25	1	1	5	30	7.816E7	480	49.7	11.24
B4DEI3	cDNA FLJ57715, highly similar to Voltage-dependent anion-selective channel protein 1 OS=Homo sapiens PE=2 SV=1 - (B4DEI3_HUMAN)	13.56	28.85	6	1	4	9	1.925E7	156	16.7	9.39
F8VU65	60S acidic ribosomal protein P0 (Fragment) OS=Homo sapiens GN=RPLP0 PE=3 SV=1 - (F8VU65_HUMAN)	13.34	25.10	17	2	6	12	9.131E7	247	27.2	9.00
Q96QA5	Gasdermin-A OS=Homo sapiens GN=GSDMA PE=2 SV=4 - (GSDMA_HUMAN)	13.33	25.84	1	1	8	14	1.435E8	445	49.3	5.29
E7ETU9	Procollagen-lysine,2-oxoglutarate 5-dioxygenase 2 OS=Homo sapiens GN=PLOD2 PE=4 SV=1 - (E7ETU9_HUMAN)	13.33	10.24	5	1	9	17	4.049E8	703	81.1	6.65
Q86SG7	Lysozyme g-like protein 2 OS=Homo sapiens GN=LYG2 PE=1 SV=2 - (LYG2_HUMAN)	13.31	11.79	4	3	3	6	1.536E7	212	23.5	8.87
Q9P241	Probable phospholipid-transporting ATPase VD OS=Homo sapiens GN=ATP10D PE=2 SV=3 - (AT10D_HUMAN)	13.30	4.70	4	1	7	10	2.784E7	1426	160.2	7.15
F8VQ14	T-complex protein 1 subunit beta OS=Homo sapiens GN=CCT2 PE=3 SV=1 - (F8VQ14_HUMAN)	13.29	13.46	4	1	3	5	1.177E7	416	44.8	6.54
Q96BA7	HNRPU protein OS=Homo sapiens PE=2 SV=1 - (Q96BA7_HUMAN)	13.24	8.86	8	1	7	25	5.852E8	722	79.7	7.87
O00362	Putative p150 OS=Homo sapiens PE=4 SV=1 - (O00362_HUMAN)	13.21	8.39	20	1	11	24	6.520E7	1275	149.1	9.69
P07900	Heat shock protein HSP 90-alpha OS=Homo sapiens GN=HSP90AA1 PE=1 SV=5 - (HS90A_HUMAN)	13.20	16.12	34	4	12	16	1.130E7	732	84.6	5.02
C9JNV4	Tripartite motif-containing protein 15 OS=Homo sapiens GN=TRIM15 PE=4 SV=1 - (C9JNV4_HUMAN)	13.17	6.06	7	1	2	6	5.861E6	396	44.8	5.73
A4QPB0	IQ motif containing GTPase activating protein 1 OS=Homo sapiens GN=IQGAP1 PE=2 SV=1 - (A4QPB0_HUMAN)	13.16	8.51	5	2	12	18	1.464E8	1657	189.2	6.48
Q5JW98	Protein FAM26D OS=Homo sapiens GN=FAM26D PE=1 SV=1 - (FA26D_HUMAN)	13.12	14.97	5	3	4	7	1.368E7	314	35.0	6.87
B4DEB9	cDNA FLJ61099, highly similar to ADP-ribosylation factor 1 OS=Homo sapiens PE=2 SV=1 - (B4DEB9_HUMAN)	13.10	28.90	19	1	4	9	4.024E7	173	19.6	8.94
H0YB22	40S ribosomal protein S14 (Fragment) OS=Homo sapiens GN=RPS14 PE=4 SV=1 - (H0YB22_HUMAN)	13.06	35.00	3	1	5	6	2.003E7	120	12.9	9.85
B4DDC4	cDNA FLJ50435, highly similar to Homo sapiens nuclear domain 10 protein (NDP52), mRNA OS=Homo sapiens PE=2 SV=1 - (B4DDC4_HUMAN)	12.97	14.44	9	1	4	9	4.158E7	374	43.5	4.81
P60866	40S ribosomal protein S20 OS=Homo sapiens GN=RPS20 PE=1 SV=1 - (RS20_HUMAN)	12.96	22.69	5	3	3	5	3.872E7	119	13.4	9.94
Q7Z6R9	Transcription factor AP-2-delta OS=Homo sapiens GN=TFAP2D PE=2 SV=1 - (AP2D_HUMAN)	12.86	19.47	1	1	8	16	1.979E8	452	49.5	8.16
C9JXB8	60S ribosomal protein L24 OS=Homo sapiens GN=RPL24 PE=4 SV=1 - (C9JXB8_HUMAN)	12.81	38.84	3	4	5	6	6.909E6	121	14.4	11.31
P02649	Apolipoprotein E OS=Homo sapiens GN=APOE PE=1 SV=1 - (APOE_HUMAN)	12.77	22.08	1	1	9	19	1.303E8	317	36.1	5.73
B9A6J0	Ecotropic viral integration site 5 OS=Homo sapiens GN=EVI5 PE=2 SV=1 - (B9A6J0_HUMAN)	12.69	11.45	3	1	7	10	4.256E7	821	94.0	6.04
P35268	60S ribosomal protein L22 OS=Homo sapiens GN=RPL22 PE=1 SV=2 - (RL22_HUMAN)	12.61	25.78	2	2	4	7	3.619E7	128	14.8	9.19
H3BRU6	Poly(rC)-binding protein 2 (Fragment) OS=Homo sapiens GN=PCBP2 PE=4 SV=1 - (H3BRU6_HUMAN)	12.60	27.24	28	2	5	6	2.470E7	301	31.7	8.44
Q9BZW7	Testis-specific gene 10 protein OS=Homo sapiens GN=TSGA10 PE=1 SV=1 - (TSG10_HUMAN)	12.57	16.19	4	1	11	23	2.318E8	698	81.4	5.97
H7C469	Uncharacterized protein (Fragment) OS=Homo sapiens PE=3 SV=1 - (H7C469_HUMAN)	12.55	15.30	5	2	4	9	3.871E8	379	40.4	5.76
P14314-2	Isoform 2 of Glucosidase 2 subunit beta OS=Homo sapiens GN=PRKCSH - (GLU2B_HUMAN)	12.50	3.81	3	1	2	11	4.405E7	525	59.1	4.42
P62081	40S ribosomal protein S7 OS=Homo sapiens GN=RPS7 PE=1 SV=1 - (RS7_HUMAN)	12.45	29.38	3	4	4	4	1.862E7	194	22.1	10.10
B4DEA8	cDNA FLJ56425, highly similar to Very-long-chain specific acyl-CoAdehydrogenase, mitochondrial (EC 1.3.99.-) OS=Homo sapiens PE=2 SV=1 - (B4DEA8_HUMAN)	12.45	13.98	8	1	6	10	4.552E7	701	75.2	8.88
Q6AI18	Putative uncharacterized protein DKFZp686M148 (Fragment) OS=Homo sapiens GN=DKFZp686M148 PE=2 SV=1 - (Q6AI18_HUMAN)	12.40	22.58	13	2	3	9	4.304E7	155	16.3	8.56
O14770-5	Isoform 5 of Homeobox protein Meis2 OS=Homo sapiens GN=MEIS2 - (MEIS2_HUMAN)	12.33	10.26	19	1	2	10	4.984E7	302	32.8	6.04
B5BTY4	ATP-dependent RNA helicase DDX3X OS=Homo sapiens GN=DDX3X PE=2 SV=1 - (B5BTY4_HUMAN)	12.33	13.29	5	1	9	19	3.031E8	662	73.1	7.36
F8WEV3	PR domain zinc finger protein 16 OS=Homo sapiens GN=PRDM16 PE=4 SV=1 - (F8WEV3_HUMAN)	12.17	7.29	10	1	8	17	7.089E7	1275	140.1	6.21
O14523	C2 domain-containing protein 2-like OS=Homo sapiens GN=C2CD2L PE=1 SV=3 - (C2C2L_HUMAN)	12.15	6.66	2	1	6	24	2.292E8	706	76.1	7.69
P04080	Cystatin-B OS=Homo sapiens GN=CSTB PE=1 SV=2 - (CYTB_HUMAN)	12.07	39.80	1	2	4	7	2.160E7	98	11.1	7.56
Q4LDG9-3	Isoform 3 of Dynein light chain 1, axonemal OS=Homo sapiens GN=DNAL1 - (DNAL1_HUMAN)	12.02	25.17	3	1	2	6	3.732E8	151	17.1	5.41
Q05DB9	PAPOLG protein (Fragment) OS=Homo sapiens GN=PAPOLG PE=2 SV=1 - (Q05DB9_HUMAN)	12.01	17.74	4	1	6	17	1.092E8	496	57.0	8.75
B2RWP4	TACC2 protein OS=Homo sapiens GN=TACC2 PE=2 SV=1 - (B2RWP4_HUMAN)	11.86	4.42	8	1	8	18	1.165E8	2826	296.6	4.79
P28074	Proteasome subunit beta type-5 OS=Homo sapiens GN=PSMB5 PE=1 SV=3 - (PSB5_HUMAN)	11.86	19.77	3	2	4	7	1.997E7	263	28.5	6.92
Q9HCY8	Protein S100-A14 OS=Homo sapiens GN=S100A14 PE=1 SV=1 - (S10AE_HUMAN)	11.86	40.38	1	3	4	6	2.771E7	104	11.7	5.24
Q12981	Vesicle transport protein SEC20 OS=Homo sapiens GN=BNIP1 PE=1 SV=3 - (SEC20_HUMAN)	11.84	9.65	2	1	2	7	5.139E7	228	26.1	8.95
B4E2K4	cDNA FLJ54576, highly similar to Aspartyl/asparaginyl beta-hydroxylase (EC 1.14.11.16) OS=Homo sapiens PE=2 SV=1 - (B4E2K4_HUMAN)	11.81	6.17	3	1	3	6	5.807E7	729	83.2	4.94
H7C346	Protein FAM9C (Fragment) OS=Homo sapiens GN=FAM9C PE=4 SV=1 - (H7C346_HUMAN)	11.73	12.70	4	1	3	8	3.664E8	126	14.8	5.50
H0YLN8	Transient receptor potential cation channel subfamily M member 7 OS=Homo sapiens GN=TRPM7 PE=4 SV=1 - (H0YLN8_HUMAN)	11.72	5.58	16	1	11	19	9.629E7	1864	212.5	7.88
C9JDG1	Protein diaphanous homolog 3 OS=Homo sapiens GN=DIAPH3 PE=4 SV=1 - (C9JDG1_HUMAN)	11.66	7.53	14	1	9	36	1.428E8	1182	135.5	7.02
Q8TAF3-3	Isoform 3 of WD repeat-containing protein 48 OS=Homo sapiens GN=WDR48 - (WDR48_HUMAN)	11.66	9.43	6	1	5	9	2.303E8	668	75.3	7.15
F5H816	Phosphorylase OS=Homo sapiens GN=PYGL PE=3 SV=1 - (F5H816_HUMAN)	11.64	11.69	18	4	10	15	4.237E7	813	93.1	7.30
B3KX35	cDNA FLJ44610 fis, clone BRACE2012317, highly similar to Sodium/potassium-transporting ATPase alpha-2 chain (EC 3.6.3.9) OS=Homo sapiens PE=2 SV=1 - (B3KX35_HUMAN)	11.63	18.47	28	1	9	13	7.808E7	655	72.6	5.88
Q8IZQ8	Myocardin OS=Homo sapiens GN=MYOCD PE=1 SV=1 - (MYCD_HUMAN)	11.55	6.18	5	1	6	14	8.166E7	938	101.9	6.62
B7Z6H4	DNA-directed RNA polymerase OS=Homo sapiens PE=2 SV=1 - (B7Z6H4_HUMAN)	11.52	5.62	5	1	8	15	2.691E8	1369	153.4	8.48
P22531	Small proline-rich protein 2E OS=Homo sapiens GN=SPRR2E PE=2 SV=2 - (SPR2E_HUMAN)	11.52	43.06	8	2	2	5	6.129E6	72	7.8	8.31
P06744-2	Isoform 2 of Glucose-6-phosphate isomerase OS=Homo sapiens GN=GPI - (G6PI_HUMAN)	11.46	11.78	4	1	5	8	8.418E8	569	64.3	8.91
Q2NLA0	Coatomer subunit gamma OS=Homo sapiens GN=COPG2 PE=2 SV=1 - (Q2NLA0_HUMAN)	11.40	7.99	8	1	5	74	3.365E7	726	81.4	6.58
Q9NZM3-3	Isoform 3 of Intersectin-2 OS=Homo sapiens GN=ITSN2 - (ITSN2_HUMAN)	11.36	8.57	10	1	8	15	5.384E7	1249	141.7	7.75
B3KTF6	cDNA FLJ38174 fis, clone FCBBF1000061, highly similar to Antizyme inhibitor 1 OS=Homo sapiens PE=2 SV=1 - (B3KTF6_HUMAN)	11.34	9.37	5	1	3	17	7.429E7	363	39.8	5.27
H7BZW3	Voltage-dependent calcium channel subunit alpha-2-3 OS=Homo sapiens GN=CACNA2D3 PE=4 SV=1 - (H7BZW3_HUMAN)	11.32	6.37	1	1	3	17	4.874E7	518	59.4	5.86
Q7Z2Y8	Interferon-induced very large GTPase 1 OS=Homo sapiens GN=GVINP1 PE=2 SV=2 - (GVIN1_HUMAN)	11.29	7.23	1	1	17	44	3.207E8	2422	278.9	6.55
Q9Y5J6	Mitochondrial import inner membrane translocase subunit Tim9 B OS=Homo sapiens GN=FXC1 PE=1 SV=1 - (TIM9B_HUMAN)	11.27	24.27	2	1	2	9	9.875E6	103	11.6	7.43
A8K335	cDNA FLJ76254, highly similar to Homo sapiens gamma-glutamyl hydrolase (GGH), mRNA OS=Homo sapiens PE=2 SV=1 - (A8K335_HUMAN)	11.25	11.95	4	2	3	6	9.952E6	318	36.0	7.42
Q5T5N4	Uncharacterized protein C6orf118 OS=Homo sapiens GN=C6orf118 PE=2 SV=1 - (CF118_HUMAN)	11.23	19.19	3	1	7	9	6.569E7	469	53.7	8.37
Q96L21	60S ribosomal protein L10-like OS=Homo sapiens GN=RPL10L PE=1 SV=3 - (RL10L_HUMAN)	11.20	22.90	8	4	5	7	1.130E7	214	24.5	10.01
Q6ZWJ8	Kielin/chordin-like protein OS=Homo sapiens GN=KCP PE=2 SV=2 - (KCP_HUMAN)	11.19	2.33	1	1	4	10	2.677E8	1503	159.8	5.74
B8XPJ7	Soluble catechol-O-methyltransferase OS=Homo sapiens GN=COMT PE=2 SV=1 - (B8XPJ7_HUMAN)	11.18	18.10	5	1	2	4	1.594E8	221	24.5	5.45
B4DV51	GTP-binding nuclear protein Ran OS=Homo sapiens GN=RAN PE=2 SV=1 - (B4DV51_HUMAN)	11.13	18.75	4	2	2	4	1.066E7	128	14.7	6.32
Q8NEY1-7	Isoform 7 of Neuron navigator 1 OS=Homo sapiens GN=NAV1 - (NAV1_HUMAN)	11.07	4.98	9	1	9	19	1.348E8	1809	194.9	8.28
P22314	Ubiquitin-like modifier-activating enzyme 1 OS=Homo sapiens GN=UBA1 PE=1 SV=3 - (UBA1_HUMAN)	10.97	5.67	1	1	4	5	1.509E7	1058	117.8	5.76
H0YBX3	Ribonuclease UK114 (Fragment) OS=Homo sapiens GN=HRSP12 PE=4 SV=1 - (H0YBX3_HUMAN)	10.92	29.92	4	2	3	4	1.713E7	127	13.8	9.31
G3V3A5	Tetratricopeptide repeat protein 6 OS=Homo sapiens GN=TTC6 PE=4 SV=1 - (G3V3A5_HUMAN)	10.90	7.48	1	1	13	25	6.369E8	1886	216.3	8.81
P02452	Collagen alpha-1(I) chain OS=Homo sapiens GN=COL1A1 PE=1 SV=5 - (CO1A1_HUMAN)	10.88	4.03	3	1	5	7	1.883E7	1464	138.9	5.80
B3KXK4	cDNA FLJ45605 fis, clone BRTHA3021971, highly similar to Nuclear receptor coactivator 7 OS=Homo sapiens PE=2 SV=1 - (B3KXK4_HUMAN)	10.74	5.69	12	1	5	9	2.486E8	931	104.8	5.55
O94844	Rho-related BTB domain-containing protein 1 OS=Homo sapiens GN=RHOBTB1 PE=1 SV=2 - (RHBT1_HUMAN)	10.68	6.90	1	1	6	23	1.542E7	696	79.4	6.87
Q96FQ6	Protein S100-A16 OS=Homo sapiens GN=S100A16 PE=1 SV=1 - (S10AG_HUMAN)	10.68	21.36	1	2	2	5	1.272E7	103	11.8	6.79
Q59G39	G protein-coupled receptor 22 variant (Fragment) OS=Homo sapiens PE=2 SV=1 - (Q59G39_HUMAN)	10.66	6.01	1	1	2	32	3.014E8	449	51.2	9.45
P26599	Polypyrimidine tract-binding protein 1 OS=Homo sapiens GN=PTBP1 PE=1 SV=1 - (PTBP1_HUMAN)	10.65	14.69	4	1	4	5	2.455E8	531	57.2	9.17
O14979-3	Isoform 3 of Heterogeneous nuclear ribonucleoprotein D-like OS=Homo sapiens GN=HNRPDL - (HNRDL_HUMAN)	10.60	23.36	16	1	6	14	9.968E7	244	27.2	8.65
Q13620-3	Isoform 3 of Cullin-4B OS=Homo sapiens GN=CUL4B - (CUL4B_HUMAN)	10.55	8.51	4	1	5	9	1.796E8	717	84.0	6.79
P25398	40S ribosomal protein S12 OS=Homo sapiens GN=RPS12 PE=1 SV=3 - (RS12_HUMAN)	10.47	31.82	1	2	4	6	1.438E7	132	14.5	7.21
E9PIU1	N-acetylated-alpha-linked acidic dipeptidase-like protein (Fragment) OS=Homo sapiens GN=NAALADL1 PE=4 SV=1 - (E9PIU1_HUMAN)	10.44	100.00	14	1	2	6	1.341E8	27	3.4	5.78
Q8WU76-2	Isoform 2 of Sec1 family domain-containing protein 2 OS=Homo sapiens GN=SCFD2 - (SCFD2_HUMAN)	10.37	3.13	2	1	2	8	5.663E7	639	69.9	6.77
O75031	Heat shock factor 2-binding protein OS=Homo sapiens GN=HSF2BP PE=1 SV=1 - (HSF2B_HUMAN)	10.32	10.48	2	1	4	10	2.116E8	334	37.6	5.53
Q69YN2	CWF19-like protein 1 OS=Homo sapiens GN=CWF19L1 PE=1 SV=2 - (C19L1_HUMAN)	10.27	1.30	1	1	1	20	9.752E7	538	60.6	7.24
P06703	Protein S100-A6 OS=Homo sapiens GN=S100A6 PE=1 SV=1 - (S10A6_HUMAN)	10.25	8.89	1	1	1	4	1.484E8	90	10.2	5.48
Q01668-2	Isoform Beta-cell-type of Voltage-dependent L-type calcium channel subunit alpha-1D OS=Homo sapiens GN=CACNA1D - (CAC1D_HUMAN)	10.25	7.47	4	1	13	16	4.464E7	2181	247.4	6.98
B7ZMB1	ZNF214 protein OS=Homo sapiens GN=ZNF214 PE=2 SV=1 - (B7ZMB1_HUMAN)	10.25	14.36	2	1	8	20	7.025E7	606	71.0	8.56
Q0VF49	Uncharacterized protein KIAA2012 OS=Homo sapiens GN=KIAA2012 PE=2 SV=1 - (K2012_HUMAN)	10.22	10.67	2	1	6	16	2.240E7	553	65.1	6.42
Q6UWV6	Ectonucleotide pyrophosphatase/phosphodiesterase family member 7 OS=Homo sapiens GN=ENPP7 PE=1 SV=3 - (ENPP7_HUMAN)	10.21	5.24	1	1	2	8	2.617E8	458	51.5	6.89
Q9BZ22	Solute carrier family 15 (Oligopeptide transporter), member 1 OS=Homo sapiens GN=SLC15A1 PE=2 SV=1 - (Q9BZ22_HUMAN)	10.16	4.61	1	1	1	6	2.943E7	282	30.9	8.60
Q53G17	NADH dehydrogenase (Ubiquinone) Fe-S protein 8, 23kDa (NADH-coenzyme Q reductase) variant (Fragment) OS=Homo sapiens PE=2 SV=1 - (Q53G17_HUMAN)	10.02	17.14	7	1	4	7	4.708E8	210	23.7	5.69
Q9BPX5	Actin-related protein 2/3 complex subunit 5-like protein OS=Homo sapiens GN=ARPC5L PE=1 SV=1 - (ARP5L_HUMAN)	9.93	29.41	2	1	5	9	1.692E7	153	16.9	6.60
D3TTZ3	Tumor necrosis factor alpha-induced protein 3 OS=Homo sapiens GN=TNFAIP3 PE=2 SV=1 - (D3TTZ3_HUMAN)	9.92	9.37	8	1	5	18	5.741E7	790	89.6	8.22
Q96L93-6	Isoform 5 of Kinesin-like protein KIF16B OS=Homo sapiens GN=KIF16B - (KI16B_HUMAN)	9.73	11.52	5	1	13	16	3.570E7	1276	147.3	6.09
F8WCJ1	Eukaryotic translation initiation factor 5A-2 OS=Homo sapiens GN=EIF5A2 PE=4 SV=1 - (F8WCJ1_HUMAN)	9.63	34.29	9	2	4	6	4.414E6	105	11.7	9.14
P62888	60S ribosomal protein L30 OS=Homo sapiens GN=RPL30 PE=1 SV=2 - (RL30_HUMAN)	9.59	33.04	9	2	4	7	3.284E7	115	12.8	9.63
H3BQZ9	Adenine phosphoribosyltransferase OS=Homo sapiens GN=APRT PE=4 SV=1 - (H3BQZ9_HUMAN)	9.58	22.88	7	2	3	8	5.598E6	153	16.6	5.59
B4DRA1	cDNA FLJ58609, highly similar to SH3-containing GRB2-like protein 1 OS=Homo sapiens PE=2 SV=1 - (B4DRA1_HUMAN)	9.55	9.38	4	1	5	11	6.755E7	320	36.2	5.19
P31151	Protein S100-A7 OS=Homo sapiens GN=S100A7 PE=1 SV=4 - (S10A7_HUMAN)	9.49	24.75	2	3	3	3	1.016E7	101	11.5	6.77
Q149N8-4	Isoform 3 of E3 ubiquitin-protein ligase SHPRH OS=Homo sapiens GN=SHPRH - (SHPRH_HUMAN)	9.48	8.32	3	1	12	15	3.613E8	1659	190.4	7.62
A8K8P1	cDNA FLJ78752, highly similar to Homo sapiens centromere protein J (CENPJ), mRNA OS=Homo sapiens PE=2 SV=1 - (A8K8P1_HUMAN)	9.47	9.34	5	1	12	40	1.606E8	1338	152.9	6.61
E0Z3F3	DUX4 (Fragment) OS=Homo sapiens GN=DUX4 PE=3 SV=1 - (E0Z3F3_HUMAN)	9.39	9.27	2	1	4	15	5.413E7	356	37.2	6.89
P25787	Proteasome subunit alpha type-2 OS=Homo sapiens GN=PSMA2 PE=1 SV=2 - (PSA2_HUMAN)	9.35	16.67	3	2	3	5	7.387E7	234	25.9	7.43
C9JZ61	Ankyrin repeat domain-containing protein 53 OS=Homo sapiens GN=ANKRD53 PE=4 SV=1 - (C9JZ61_HUMAN)	9.32	8.47	4	1	3	14	1.267E8	496	56.0	9.48
O95235	Kinesin-like protein KIF20A OS=Homo sapiens GN=KIF20A PE=1 SV=1 - (KI20A_HUMAN)	9.21	12.25	2	1	7	9	2.587E7	890	100.2	6.92
A1A5D9	Bicaudal D-related protein 2 OS=Homo sapiens GN=CCDC64B PE=2 SV=2 - (BICR2_HUMAN)	9.20	10.63	2	1	5	9	7.571E7	508	56.8	5.02
F8VR50	Actin-related protein 2/3 complex subunit 3 (Fragment) OS=Homo sapiens GN=ARPC3 PE=4 SV=1 - (F8VR50_HUMAN)	9.20	30.95	6	3	3	4	6.110E6	84	9.7	7.93
Q53SY7	Putative uncharacterized protein CAD (Fragment) OS=Homo sapiens GN=CAD PE=2 SV=1 - (Q53SY7_HUMAN)	9.14	2.14	5	1	4	10	2.792E7	2151	235.0	6.60
Q04760-2	Isoform 2 of Lactoylglutathione lyase OS=Homo sapiens GN=GLO1 - (LGUL_HUMAN)	9.07	11.83	2	2	3	7	7.441E7	169	19.0	6.05
P19105	Myosin regulatory light chain 12A OS=Homo sapiens GN=MYL12A PE=1 SV=2 - (ML12A_HUMAN)	9.01	24.56	7	3	4	8	2.222E7	171	19.8	4.81
P61254	60S ribosomal protein L26 OS=Homo sapiens GN=RPL26 PE=1 SV=1 - (RL26_HUMAN)	8.97	28.28	8	5	6	7	1.445E7	145	17.2	10.55
Q0D2H7	PCF11 protein (Fragment) OS=Homo sapiens GN=PCF11 PE=2 SV=1 - (Q0D2H7_HUMAN)	8.90	8.86	1	1	1	5	9.793E6	158	17.0	8.02
Q9UKJ3	G patch domain-containing protein 8 OS=Homo sapiens GN=GPATCH8 PE=1 SV=2 - (GPTC8_HUMAN)	8.88	7.32	2	1	12	24	4.305E7	1502	164.1	8.66
B5MC21	Interleukin-6 OS=Homo sapiens GN=IL6 PE=4 SV=1 - (B5MC21_HUMAN)	8.87	13.24	4	1	1	6	7.034E6	136	15.6	5.57
B4DX78	cDNA FLJ55484, highly similar to ATP-dependent RNA helicase DDX39 (EC 3.6.1.-) OS=Homo sapiens PE=2 SV=1 - (B4DX78_HUMAN)	8.87	9.79	15	1	4	8	1.856E7	470	53.7	5.90
P05091	Aldehyde dehydrogenase, mitochondrial OS=Homo sapiens GN=ALDH2 PE=1 SV=2 - (ALDH2_HUMAN)	8.77	7.16	7	1	4	13	6.105E7	517	56.3	7.05
P35030	Trypsin-3 OS=Homo sapiens GN=PRSS3 PE=1 SV=2 - (TRY3_HUMAN)	8.76	15.13	12	1	4	15	3.416E7	304	32.5	7.49
B7ZAH9	cDNA, FLJ79193, highly similar to Beta-1,4-galactosyltransferase 1 (EC 2.4.1.-) OS=Homo sapiens PE=2 SV=1 - (B7ZAH9_HUMAN)	8.73	22.48	5	1	5	10	1.746E8	258	30.0	8.60
Q86YV0-2	Isoform 2 of RAS protein activator like-3 OS=Homo sapiens GN=RASAL3 - (RASL3_HUMAN)	8.72	13.89	2	1	11	15	2.084E7	943	104.2	8.95
H7C2W9	60S ribosomal protein L31 (Fragment) OS=Homo sapiens GN=RPL31 PE=4 SV=1 - (H7C2W9_HUMAN)	8.67	36.11	9	2	7	17	1.845E8	108	12.8	11.00
Q6ZV71	cDNA FLJ42918 fis, clone BRHIP3027137, highly similar to 10-formyltetrahydrofolate dehydrogenase OS=Homo sapiens PE=2 SV=1 - (Q6ZV71_HUMAN)	8.67	10.43	1	1	5	8	2.960E7	470	50.8	8.16
B7ZL41	ABCG8 protein OS=Homo sapiens GN=ABCG8 PE=2 SV=1 - (B7ZL41_HUMAN)	8.54	8.93	4	1	6	24	1.521E8	672	75.5	8.31
I1YDD2	2'-5'-oligoadenylate synthetase-like transcript variant 4 OS=Homo sapiens GN=OASLd PE=2 SV=1 - (I1YDD2_HUMAN)	8.48	13.80	4	1	3	6	1.158E6	384	43.9	9.19
B6ZGS4	Retinoid-related orphan receptor alpha OS=Homo sapiens GN=NR1F1 PE=2 SV=1 - (B6ZGS4_HUMAN)	8.46	16.83	5	1	8	25	1.968E8	523	59.0	6.86
P29992	Guanine nucleotide-binding protein subunit alpha-11 OS=Homo sapiens GN=GNA11 PE=1 SV=2 - (GNA11_HUMAN)	8.41	20.06	2	1	4	9	3.262E7	359	42.1	5.69
H0YKD8	60S ribosomal protein L28 OS=Homo sapiens GN=RPL28 PE=4 SV=1 - (H0YKD8_HUMAN)	8.38	31.76	4	1	5	7	5.239E7	170	19.1	11.46
Q9Y2I9	TBC1 domain family member 30 OS=Homo sapiens GN=TBC1D30 PE=1 SV=2 - (TBC30_HUMAN)	8.38	7.03	6	1	7	28	1.014E8	924	102.7	8.29
P49720	Proteasome subunit beta type-3 OS=Homo sapiens GN=PSMB3 PE=1 SV=2 - (PSB3_HUMAN)	8.33	13.66	1	2	3	3	2.665E7	205	22.9	6.55
P46783	40S ribosomal protein S10 OS=Homo sapiens GN=RPS10 PE=1 SV=1 - (RS10_HUMAN)	8.32	33.33	3	2	5	10	1.122E7	165	18.9	10.15
P62699	Protein yippee-like 5 OS=Homo sapiens GN=YPEL5 PE=2 SV=1 - (YPEL5_HUMAN)	8.30	58.68	1	2	6	6	1.022E7	121	13.8	7.31
Q8N300	Coiled-coil domain-containing protein 23 OS=Homo sapiens GN=CCDC23 PE=2 SV=1 - (CCD23_HUMAN)	8.21	25.76	1	1	2	12	6.565E6	66	7.8	9.16
P29373	Cellular retinoic acid-binding protein 2 OS=Homo sapiens GN=CRABP2 PE=1 SV=2 - (RABP2_HUMAN)	8.21	25.36	2	1	4	6	2.261E7	138	15.7	5.40
Q8NHS4-2	Isoform 2 of Uncharacterized protein C2orf63 OS=Homo sapiens GN=C2orf63 - (CB063_HUMAN)	8.21	7.33	2	1	3	8	1.008E8	464	52.9	5.69
P28066	Proteasome subunit alpha type-5 OS=Homo sapiens GN=PSMA5 PE=1 SV=3 - (PSA5_HUMAN)	8.19	7.88	3	2	2	3	6.859E6	241	26.4	4.79
Q8NHY3	GAS2-like protein 2 OS=Homo sapiens GN=GAS2L2 PE=2 SV=1 - (GA2L2_HUMAN)	8.06	8.98	1	1	7	10	6.285E7	880	96.5	9.13
P32926	Desmoglein-3 OS=Homo sapiens GN=DSG3 PE=1 SV=2 - (DSG3_HUMAN)	8.01	4.90	1	1	4	5	3.457E7	999	107.5	5.00
B2RBF9	cDNA, FLJ95487, highly similar to Homo sapiens peptidyl arginine deiminase, type I (PADI1), mRNA OS=Homo sapiens PE=2 SV=1 - (B2RBF9_HUMAN)	7.99	12.37	3	1	8	16	9.194E7	663	74.6	6.49
B4DS76	Mitogen-activated protein kinase kinase kinase 11 OS=Homo sapiens GN=MAP3K11 PE=2 SV=1 - (B4DS76_HUMAN)	7.93	5.42	3	1	3	11	4.328E7	590	65.3	9.22
B4DX24	NADPH:adrenodoxin oxidoreductase, mitochondrial OS=Homo sapiens PE=2 SV=1 - (B4DX24_HUMAN)	7.90	8.70	11	1	4	15	1.456E9	483	52.9	8.44
Q9H091-2	Isoform 2 of Zinc finger MYND domain-containing protein 15 OS=Homo sapiens GN=ZMYND15 - (ZMY15_HUMAN)	7.90	5.55	3	1	4	7	1.903E7	703	77.3	6.89
Q9UIV8-2	Isoform 2 of Serpin B13 OS=Homo sapiens GN=SERPINB13 - (SPB13_HUMAN)	7.81	20.65	10	2	8	16	1.137E7	339	38.4	5.81
Q59FN4	LATS homolog 1 variant (Fragment) OS=Homo sapiens PE=2 SV=1 - (Q59FN4_HUMAN)	7.77	7.71	2	1	7	12	5.234E7	830	92.3	9.45
B2R717	cDNA, FLJ93235, highly similar to Homo sapiens cathepsin L2 (CTSL2), mRNA OS=Homo sapiens PE=2 SV=1 - (B2R717_HUMAN)	7.76	9.58	2	1	2	5	1.949E7	334	37.3	8.76
Q16176	Lsk protein OS=Homo sapiens GN=lsk PE=2 SV=1 - (Q16176_HUMAN)	7.69	9.03	1	1	4	5	4.574E7	465	51.9	8.51
Q5T8Y7	COMM domain containing 3 (Fragment) OS=Homo sapiens GN=COMMD3 PE=2 SV=1 - (Q5T8Y7_HUMAN)	7.66	28.38	5	1	3	6	4.428E7	148	16.6	7.88
P50395-2	Isoform 2 of Rab GDP dissociation inhibitor beta OS=Homo sapiens GN=GDI2 - (GDIB_HUMAN)	7.51	12.25	6	1	3	4	1.156E7	400	45.6	6.24
Q9H2K8	Serine/threonine-protein kinase TAO3 OS=Homo sapiens GN=TAOK3 PE=1 SV=2 - (TAOK3_HUMAN)	7.48	12.14	5	1	11	16	1.440E8	898	105.3	7.30
G3V3A0	Alpha-1-antichymotrypsin OS=Homo sapiens GN=SERPINA3 PE=3 SV=1 - (G3V3A0_HUMAN)	7.43	9.27	4	1	2	3	8.710E6	205	23.4	5.64
Q6ECI4	Zinc finger protein 470 OS=Homo sapiens GN=ZNF470 PE=1 SV=3 - (ZN470_HUMAN)	7.41	4.18	2	1	4	15	3.711E7	717	82.6	8.57
Q6MZV7	Putative uncharacterized protein DKFZp686C11235 OS=Homo sapiens GN=DKFZp686C11235 PE=1 SV=1 - (Q6MZV7_HUMAN)	7.40	7.82	13	1	3	4	6.543E7	473	52.1	7.58
P14174	Macrophage migration inhibitory factor OS=Homo sapiens GN=MIF PE=1 SV=4 - (MIF_HUMAN)	7.38	18.26	1	1	2	4	2.840E7	115	12.5	7.88
Q16832	Discoidin domain-containing receptor 2 OS=Homo sapiens GN=DDR2 PE=1 SV=2 - (DDR2_HUMAN)	7.36	9.01	2	1	8	24	9.829E7	855	96.7	5.36
P62266	40S ribosomal protein S23 OS=Homo sapiens GN=RPS23 PE=1 SV=3 - (RS23_HUMAN)	7.30	16.08	1	1	3	4	6.728E6	143	15.8	10.49
B4DT13	Ras-related protein Rab-11A OS=Homo sapiens GN=RAB11A PE=2 SV=1 - (B4DT13_HUMAN)	7.28	19.35	12	3	3	3	6.734E6	155	17.6	8.85
B4DFX6	cDNA FLJ58791, highly similar to Ankyrin repeat domain-containing protein 35 OS=Homo sapiens PE=2 SV=1 - (B4DFX6_HUMAN)	7.26	14.40	3	1	9	12	4.556E7	910	100.2	5.77
Q4J6C6	Prolyl endopeptidase-like OS=Homo sapiens GN=PREPL PE=1 SV=1 - (PPCEL_HUMAN)	7.25	6.74	3	1	4	13	1.873E7	727	83.9	6.38
A6QKW0	SHINC3 OS=Homo sapiens GN=SHINC3 PE=2 SV=1 - (A6QKW0_HUMAN)	7.20	23.89	3	1	5	7	7.359E6	247	27.9	9.70
Q2TAC6-2	Isoform 2 of Kinesin-like protein KIF19 OS=Homo sapiens GN=KIF19 - (KIF19_HUMAN)	7.03	9.85	2	1	6	10	7.861E7	548	61.9	7.59
Q53RT3	Retroviral-like aspartic protease 1 OS=Homo sapiens GN=ASPRV1 PE=1 SV=1 - (APRV1_HUMAN)	6.97	10.50	1	1	3	4	4.971E8	343	37.0	5.44
F5GX67	Keratin-associated protein 1-5 OS=Homo sapiens GN=KRTAP1-5 PE=4 SV=1 - (F5GX67_HUMAN)	6.97	4.27	2	1	1	6	2.255E8	164	17.0	7.06
E7EPB3	60S ribosomal protein L14 OS=Homo sapiens GN=RPL14 PE=4 SV=1 - (E7EPB3_HUMAN)	6.97	21.77	7	1	4	18	5.910E8	124	14.5	10.21
Q5SXQ8	Novel protein (Fragment) OS=Homo sapiens GN=RP11-792P23.2 PE=2 SV=1 - (Q5SXQ8_HUMAN)	6.96	14.19	1	1	4	7	8.737E7	296	34.8	5.26
B4DN33	Transmembrane protein 35 OS=Homo sapiens GN=TMEM35 PE=2 SV=1 - (B4DN33_HUMAN)	6.95	16.67	2	1	1	6	1.583E7	126	14.0	10.10
P62750	60S ribosomal protein L23a OS=Homo sapiens GN=RPL23A PE=1 SV=1 - (RL23A_HUMAN)	6.92	29.49	5	2	4	5	1.407E7	156	17.7	10.45
A6NJH9	Eukaryotic translation initiation factor 1A, Y-chromosomal OS=Homo sapiens GN=EIF1AY PE=4 SV=1 - (A6NJH9_HUMAN)	6.90	18.11	3	1	3	9	2.940E6	127	14.4	5.34
D3DV26	S100 calcium binding protein A10 (Annexin II ligand, calpactin I, light polypeptide (P11)), isoform CRA_b (Fragment) OS=Homo sapiens GN=S100A10 PE=4 SV=1 - (D3DV26_HUMAN)	6.90	24.88	3	1	5	8	7.273E7	205	22.3	10.33
Q86XP0	Cytosolic phospholipase A2 delta OS=Homo sapiens GN=PLA2G4D PE=2 SV=2 - (PA24D_HUMAN)	6.84	5.01	1	1	3	10	4.449E7	818	91.9	5.50
P29034	Protein S100-A2 OS=Homo sapiens GN=S100A2 PE=1 SV=3 - (S10A2_HUMAN)	6.81	36.73	2	3	5	9	1.007E8	98	11.1	4.78
P49368-2	Isoform 2 of T-complex protein 1 subunit gamma OS=Homo sapiens GN=CCT3 - (TCPG_HUMAN)	6.76	17.16	7	1	7	13	2.805E6	507	56.4	6.49
E5RJ48	Acyl-protein thioesterase 1 (Fragment) OS=Homo sapiens GN=LYPLA1 PE=4 SV=1 - (E5RJ48_HUMAN)	6.76	40.00	6	1	2	2	6.798E6	90	9.6	4.89
Q96FJ2	Dynein light chain 2, cytoplasmic OS=Homo sapiens GN=DYNLL2 PE=1 SV=1 - (DYL2_HUMAN)	6.71	40.45	2	2	4	6	4.657E7	89	10.3	7.37
O15054	Lysine-specific demethylase 6B OS=Homo sapiens GN=KDM6B PE=1 SV=4 - (KDM6B_HUMAN)	6.68	2.56	3	1	4	11	6.683E6	1643	176.5	8.54
Q5VSX7	Tripartite motif-containing 62 OS=Homo sapiens GN=TRIM62 PE=2 SV=1 - (Q5VSX7_HUMAN)	6.63	10.66	3	1	3	10	2.977E8	394	44.8	6.32
P26641	Elongation factor 1-gamma OS=Homo sapiens GN=EEF1G PE=1 SV=3 - (EF1G_HUMAN)	6.63	5.95	5	1	3	5	8.238E6	437	50.1	6.67
B2YKU2	Cytochrome c oxidase subunit 2 (Fragment) OS=Homo sapiens GN=COX2 PE=3 SV=1 - (B2YKU2_HUMAN)	6.59	34.09	177	2	2	2	1.615E7	88	9.7	7.28
P30046	D-dopachrome decarboxylase OS=Homo sapiens GN=DDT PE=1 SV=3 - (DOPD_HUMAN)	6.53	19.49	2	1	3	4	2.187E7	118	12.7	7.30
B5BTZ6	Signal transducer and activator of transcription 3 isoform 2 OS=Homo sapiens GN=STAT3 PE=2 SV=1 - (B5BTZ6_HUMAN)	6.50	11.83	7	1	7	36	7.997E7	769	87.9	6.20
G5E9Y3	Arf-GAP with Rho-GAP domain, ANK repeat and PH domain-containing protein 3 OS=Homo sapiens GN=ARAP3 PE=4 SV=1 - (G5E9Y3_HUMAN)	6.49	7.71	1	1	9	14	4.161E7	1375	151.0	6.86
Q9H694	Protein bicaudal C homolog 1 OS=Homo sapiens GN=BICC1 PE=1 SV=2 - (BICC1_HUMAN)	6.48	9.03	1	1	7	10	4.297E7	974	104.8	8.54
E2GH19	T-cell factor-4 variant I OS=Homo sapiens GN=TCF7L2 PE=2 SV=1 - (E2GH19_HUMAN)	6.42	1.87	1	1	1	6	1.081E8	482	53.7	8.81
P78509-2	Isoform 2 of Reelin OS=Homo sapiens GN=RELN - (RELN_HUMAN)	6.41	2.63	6	1	9	17	1.675E7	3458	388.0	5.88
Q5JYB1	Zinc finger and BTB domain containing 17 (Fragment) OS=Homo sapiens GN=ZBTB17 PE=2 SV=1 - (Q5JYB1_HUMAN)	6.37	14.17	2	1	2	47	1.305E7	240	26.6	9.03
P49207	60S ribosomal protein L34 OS=Homo sapiens GN=RPL34 PE=1 SV=3 - (RL34_HUMAN)	6.36	31.62	2	2	5	5	5.066E6	117	13.3	11.47
F8VUA6	60S ribosomal protein L18 (Fragment) OS=Homo sapiens GN=RPL18 PE=4 SV=1 - (F8VUA6_HUMAN)	6.35	26.15	6	1	3	3	3.064E7	130	14.5	11.75
F8W1S7	Heterogeneous nuclear ribonucleoprotein A1 OS=Homo sapiens GN=HNRNPA1 PE=4 SV=1 - (F8W1S7_HUMAN)	6.32	15.15	16	1	6	10	2.609E7	330	35.5	9.11
Q53T70	Putative uncharacterized protein RAB10 (Fragment) OS=Homo sapiens GN=RAB10 PE=2 SV=1 - (Q53T70_HUMAN)	6.32	24.84	2	1	3	6	2.024E7	157	17.8	8.25
Q59H50	Integrin beta (Fragment) OS=Homo sapiens PE=1 SV=1 - (Q59H50_HUMAN)	6.32	8.00	6	2	3	7	2.771E7	475	52.4	6.54
Q5SSD1	Synaptotagmin-like 1 OS=Homo sapiens GN=SYTL1 PE=4 SV=1 - (Q5SSD1_HUMAN)	6.25	15.92	6	1	3	6	1.769E7	201	22.0	9.69
Q15181	Inorganic pyrophosphatase OS=Homo sapiens GN=PPA1 PE=1 SV=2 - (IPYR_HUMAN)	6.20	7.27	1	1	2	3	7.544E8	289	32.6	5.86
P62847-2	Isoform 2 of 40S ribosomal protein S24 OS=Homo sapiens GN=RPS24 - (RS24_HUMAN)	6.19	20.00	5	1	2	2	3.432E7	130	15.1	10.89
Q9Y4F4	Protein FAM179B OS=Homo sapiens GN=FAM179B PE=1 SV=4 - (F179B_HUMAN)	6.16	8.37	1	1	14	15	2.231E8	1720	189.2	8.50
P17706-2	Isoform PTPA of Tyrosine-protein phosphatase non-receptor type 2 OS=Homo sapiens GN=PTPN2 - (PTN2_HUMAN)	6.14	15.25	3	1	4	10	9.705E7	387	45.1	8.13
Q66LE6	Serine/threonine-protein phosphatase 2A 55 kDa regulatory subunit B delta isoform OS=Homo sapiens GN=PPP2R2D PE=1 SV=1 - (2ABD_HUMAN)	6.14	1.99	1	1	1	3	1.397E8	453	52.0	6.39
C9JJR0	Neurexin-3-beta, soluble form OS=Homo sapiens GN=NRXN3 PE=4 SV=2 - (C9JJR0_HUMAN)	6.12	8.69	5	1	9	10	6.609E7	1392	153.9	6.02
Q96BD5	PHD finger protein 21A OS=Homo sapiens GN=PHF21A PE=1 SV=1 - (PF21A_HUMAN)	6.09	7.79	6	1	5	9	1.507E7	680	74.8	9.45
P98161-2	Isoform 2 of Polycystin-1 OS=Homo sapiens GN=PKD1 - (PKD1_HUMAN)	6.08	5.29	10	1	18	34	8.284E7	4292	461.1	6.77
B2R761	cDNA, FLJ93299, highly similar to Homo sapiens sterol carrier protein 2 (SCP2), mRNA OS=Homo sapiens PE=2 SV=1 - (B2R761_HUMAN)	6.05	13.16	10	1	6	8	2.028E7	547	59.0	7.05
A8MWD9	Small nuclear ribonucleoprotein G-like protein OS=Homo sapiens PE=3 SV=2 - (RUXGL_HUMAN)	6.05	73.68	6	2	4	4	2.550E8	76	8.5	8.84
Q15650	Activating signal cointegrator 1 OS=Homo sapiens GN=TRIP4 PE=1 SV=4 - (TRIP4_HUMAN)	5.98	6.88	2	1	3	5	4.914E7	581	66.1	7.85
D6RF23	Guanine nucleotide-binding protein subunit beta-2-like 1 OS=Homo sapiens GN=GNB2L1 PE=4 SV=1 - (D6RF23_HUMAN)	5.97	20.79	11	2	2	2	1.530E7	101	11.2	4.88
P60900	Proteasome subunit alpha type-6 OS=Homo sapiens GN=PSMA6 PE=1 SV=1 - (PSA6_HUMAN)	5.96	29.67	7	1	6	7	6.921E7	246	27.4	6.76
P62273	40S ribosomal protein S29 OS=Homo sapiens GN=RPS29 PE=1 SV=2 - (RS29_HUMAN)	5.84	26.79	2	2	2	3	1.769E7	56	6.7	10.13
Q53GF9	Full-length cDNA 5-PRIME end of clone CS0DF013YM24 of Fetal brain of Homo sapiens (Human) variant (Fragment) OS=Homo sapiens PE=2 SV=1 - (Q53GF9_HUMAN)	5.74	11.11	4	1	2	3	1.146E6	225	25.6	6.70
Q14330	N-arachidonyl glycine receptor OS=Homo sapiens GN=GPR18 PE=2 SV=2 - (GPR18_HUMAN)	5.71	8.46	1	1	4	11	1.383E8	331	38.1	9.26
A8K3R3	cDNA FLJ75284, highly similar to Homo sapiens RAB guanine nucleotide exchange factor (GEF) 1 (RABGEF1), mRNA OS=Homo sapiens PE=2 SV=1 - (A8K3R3_HUMAN)	5.71	9.78	10	1	5	7	1.648E8	491	56.8	7.27
A6NG06	Peroxiredoxin-5, mitochondrial OS=Homo sapiens GN=PRDX5 PE=4 SV=1 - (A6NG06_HUMAN)	5.68	28.24	4	1	2	2	5.987E6	170	17.4	8.94
Q9BYR2	Keratin-associated protein 4-5 OS=Homo sapiens GN=KRTAP4-5 PE=2 SV=4 - (KRA45_HUMAN)	5.68	15.47	4	1	1	2	1.293E8	181	19.3	7.78
Q86V48-2	Isoform 2 of Leucine zipper protein 1 OS=Homo sapiens GN=LUZP1 - (LUZP1_HUMAN)	5.65	9.45	6	1	11	19	1.038E8	1026	114.5	8.88
P62314	Small nuclear ribonucleoprotein Sm D1 OS=Homo sapiens GN=SNRPD1 PE=1 SV=1 - (SMD1_HUMAN)	5.61	16.81	2	1	1	1	1.745E6	119	13.3	11.56
Q9UNN8	Endothelial protein C receptor OS=Homo sapiens GN=PROCR PE=1 SV=1 - (EPCR_HUMAN)	5.60	3.78	1	1	1	3	1.714E6	238	26.7	7.18
B4DE53	cDNA FLJ61213, highly similar to Tubulin-specific chaperone D OS=Homo sapiens PE=2 SV=1 - (B4DE53_HUMAN)	5.56	9.19	7	1	7	18	8.498E7	849	94.4	6.13
Q12915	Ibd1 protein (Fragment) OS=Homo sapiens GN=Ibd1 PE=2 SV=1 - (Q12915_HUMAN)	5.52	4.41	1	1	1	2	4.742E7	204	22.1	8.46
Q99935	Proline-rich protein 1 OS=Homo sapiens GN=PROL1 PE=1 SV=2 - (PROL1_HUMAN)	5.51	8.06	1	1	1	3	3.161E7	248	27.2	10.42
Q9Y3D6	Mitochondrial fission 1 protein OS=Homo sapiens GN=FIS1 PE=1 SV=2 - (FIS1_HUMAN)	5.49	13.16	1	1	2	3	4.270E6	152	16.9	8.79
P05387	60S acidic ribosomal protein P2 OS=Homo sapiens GN=RPLP2 PE=1 SV=1 - (RLA2_HUMAN)	5.46	20.00	1	1	2	3	1.859E7	115	11.7	4.54
F5GY37	Prohibitin-2 OS=Homo sapiens GN=PHB2 PE=4 SV=1 - (F5GY37_HUMAN)	5.43	21.35	2	1	6	6	1.336E7	267	29.7	9.88
Q5JNZ5	Putative 40S ribosomal protein S26-like 1 OS=Homo sapiens GN=RPS26P11 PE=5 SV=1 - (RS26L_HUMAN)	5.37	14.78	6	1	2	8	1.945E8	115	13.0	10.54
P62304	Small nuclear ribonucleoprotein E OS=Homo sapiens GN=SNRPE PE=1 SV=1 - (RUXE_HUMAN)	5.32	60.87	1	1	3	3	1.331E7	92	10.8	9.44
C9JFR7	Cytochrome c (Fragment) OS=Homo sapiens GN=CYCS PE=3 SV=1 - (C9JFR7_HUMAN)	5.22	18.81	3	2	2	3	5.365E6	101	11.3	9.66
Q14997	Proteasome activator complex subunit 4 OS=Homo sapiens GN=PSME4 PE=1 SV=2 - (PSME4_HUMAN)	5.21	6.08	3	1	11	14	2.147E8	1843	211.2	6.90
B4DMH5	cDNA FLJ55107, highly similar to Cell division control protein 42 homolog OS=Homo sapiens PE=2 SV=1 - (B4DMH5_HUMAN)	5.20	12.45	5	1	2	3	1.345E6	233	26.6	6.90
Q8IYY4-2	Isoform 2 of Zinc finger protein DZIP1L OS=Homo sapiens GN=DZIP1L - (DZI1L_HUMAN)	5.14	11.69	3	1	7	10	6.528E7	539	62.4	7.37
Q9H3E7	MSTP038 OS=Homo sapiens PE=4 SV=1 - (Q9H3E7_HUMAN)	5.11	17.92	1	1	1	7	1.111E8	106	12.2	8.50
B7Z815	Ubiquitin carboxyl-terminal hydrolase OS=Homo sapiens GN=USP7 PE=2 SV=1 - (B7Z815_HUMAN)	5.03	9.67	6	1	8	14	1.842E8	1086	126.2	5.71
B4DE15	cDNA FLJ53300, highly similar to UDP-galactose translocator OS=Homo sapiens PE=2 SV=1 - (B4DE15_HUMAN)	5.03	9.61	7	1	2	8	1.675E7	406	42.2	9.86
I3L518	Cytosolic Fe-S cluster assembly factor NUBP1 (Fragment) OS=Homo sapiens GN=NUBP1 PE=4 SV=1 - (I3L518_HUMAN)	5.02	22.22	1	1	1	2	3.413E7	63	7.1	9.74
F6RFD5	Destrin OS=Homo sapiens GN=DSTN PE=4 SV=1 - (F6RFD5_HUMAN)	5.01	21.48	4	1	3	3	2.002E7	135	15.4	8.59
B2R8A2	cDNA, FLJ93804, highly similar to Homo sapiens gp25L2 protein (HSGP25L2G), mRNA OS=Homo sapiens PE=2 SV=1 - (B2R8A2_HUMAN)	5.01	9.81	2	1	2	3	4.075E7	214	25.1	7.18
Q9BYQ3	Keratin-associated protein 9-3 OS=Homo sapiens GN=KRTAP9-3 PE=1 SV=1 - (KRA93_HUMAN)	4.99	11.32	1	1	1	1		159	16.8	7.53
D7PBN3	ESRP1/RAF1 fusion protein OS=Homo sapiens PE=2 SV=1 - (D7PBN3_HUMAN)	4.95	6.13	11	1	7	8	1.127E8	1061	118.5	8.22
D3DSL5	Chromosome 21 open reading frame 2, isoform CRA_a OS=Homo sapiens GN=C21orf2 PE=4 SV=1 - (D3DSL5_HUMAN)	4.95	22.48	1	1	3	3	1.227E7	218	23.9	5.68
Q96PI1	Small proline-rich protein 4 OS=Homo sapiens GN=SPRR4 PE=2 SV=1 - (SPRR4_HUMAN)	4.88	27.85	1	1	2	3	4.892E6	79	8.8	9.66
Q05BX4	PSMD1 protein (Fragment) OS=Homo sapiens GN=PSMD1 PE=2 SV=1 - (Q05BX4_HUMAN)	4.88	7.52	6	1	5	10	3.894E7	824	91.0	6.04
P00918	Carbonic anhydrase 2 OS=Homo sapiens GN=CA2 PE=1 SV=2 - (CAH2_HUMAN)	4.83	10.00	1	1	2	2	1.360E6	260	29.2	7.40
Q59GK9	Ribosomal protein L21 variant (Fragment) OS=Homo sapiens PE=2 SV=1 - (Q59GK9_HUMAN)	4.81	33.13	3	2	5	5	6.536E6	163	18.9	10.54
D6RIG4	Protocadherin-18 OS=Homo sapiens GN=PCDH18 PE=4 SV=1 - (D6RIG4_HUMAN)	4.80	3.06	6	1	2	5	3.515E7	914	101.1	5.19
P42766	60S ribosomal protein L35 OS=Homo sapiens GN=RPL35 PE=1 SV=2 - (RL35_HUMAN)	4.78	29.27	3	1	4	5	6.430E6	123	14.5	11.05
P62318	Small nuclear ribonucleoprotein Sm D3 OS=Homo sapiens GN=SNRPD3 PE=1 SV=1 - (SMD3_HUMAN)	4.72	39.68	2	1	4	5	1.035E7	126	13.9	10.32
B3KY56	Extended synaptotagmin-1 OS=Homo sapiens GN=ESYT1 PE=2 SV=1 - (B3KY56_HUMAN)	4.69	2.65	4	1	3	4	1.191E6	1058	117.3	5.90
Q96AZ6	Interferon-stimulated gene 20 kDa protein OS=Homo sapiens GN=ISG20 PE=1 SV=2 - (ISG20_HUMAN)	4.66	23.20	1	1	3	3	4.018E6	181	20.4	8.92
P48729	Casein kinase I isoform alpha OS=Homo sapiens GN=CSNK1A1 PE=1 SV=2 - (KC1A_HUMAN)	4.66	13.95	6	1	3	4	1.108E8	337	38.9	9.57
Q96AG4	Leucine-rich repeat-containing protein 59 OS=Homo sapiens GN=LRRC59 PE=1 SV=1 - (LRC59_HUMAN)	4.65	9.77	1	1	3	3	3.615E6	307	34.9	9.57
P48047	ATP synthase subunit O, mitochondrial OS=Homo sapiens GN=ATP5O PE=1 SV=1 - (ATPO_HUMAN)	4.62	20.19	4	2	4	4	1.612E7	213	23.3	9.96
C0IMJ4	Periostin isoform thy8 OS=Homo sapiens PE=2 SV=1 - (C0IMJ4_HUMAN)	4.62	3.88	5	1	3	6	2.908E7	721	80.3	8.19
H3BQV3	Conserved oligomeric Golgi complex subunit 8 OS=Homo sapiens GN=COG8 PE=4 SV=1 - (H3BQV3_HUMAN)	4.60	5.99	4	1	3	7	6.435E7	534	60.4	5.81
Q5H9L4	Transcription initiation factor TFIID subunit 7-like OS=Homo sapiens GN=TAF7L PE=2 SV=1 - (TAF7L_HUMAN)	4.58	10.39	2	1	3	9	3.192E7	462	52.6	4.65
Q9HA11	Histone H2A OS=Homo sapiens PE=2 SV=1 - (Q9HA11_HUMAN)	4.53	17.14	8	1	4	5	5.778E6	280	29.9	10.07
H8WV78	MHC class II antigen (Fragment) OS=Homo sapiens GN=HLA-DRB1 PE=4 SV=1 - (H8WV78_HUMAN)	4.53	15.73	1	1	1	6	2.011E7	89	10.6	6.30
Q9NS37	CREB/ATF bZIP transcription factor OS=Homo sapiens GN=CREBZF PE=1 SV=2 - (ZHANG_HUMAN)	4.44	17.23	3	1	5	8	1.102E7	354	37.1	5.24
Q5VWK0	Neuroblastoma breakpoint family member 6 OS=Homo sapiens GN=NBPF6 PE=2 SV=2 - (NBPF6_HUMAN)	4.42	9.25	7	1	5	8	6.300E6	638	72.2	4.93
F5H7J5	Regulatory-associated protein of mTOR OS=Homo sapiens GN=RPTOR PE=4 SV=1 - (F5H7J5_HUMAN)	4.40	3.14	2	1	4	6	9.783E7	1177	131.4	7.03
I3L398	Protein disulfide-isomerase (Fragment) OS=Homo sapiens GN=P4HB PE=4 SV=1 - (I3L398_HUMAN)	4.39	7.50	12	1	2	3	5.043E6	200	23.0	5.02
Q5JU85	IQ motif and SEC7 domain-containing protein 2 OS=Homo sapiens GN=IQSEC2 PE=1 SV=1 - (IQEC2_HUMAN)	4.36	6.36	11	1	10	15	1.892E8	1478	161.6	8.56
P41250	Glycine--tRNA ligase OS=Homo sapiens GN=GARS PE=1 SV=3 - (SYG_HUMAN)	4.34	5.01	1	1	3	3	4.309E6	739	83.1	7.03
B4DK14	cDNA FLJ58890, highly similar to GTP-binding protein PTD004 OS=Homo sapiens PE=2 SV=1 - (B4DK14_HUMAN)	4.34	12.45	5	1	3	3	5.614E6	273	30.7	7.27
Q49A13	ZNF800 protein OS=Homo sapiens GN=ZNF800 PE=2 SV=1 - (Q49A13_HUMAN)	4.28	13.95	1	1	4	5	5.864E7	258	29.4	7.01
B4DFE6	cDNA FLJ59861, highly similar to ATP synthase gamma chain, mitochondrial (EC 3.6.3.14) OS=Homo sapiens PE=2 SV=1 - (B4DFE6_HUMAN)	4.22	15.38	5	1	2	2	7.560E6	143	15.7	10.29
Q9Y2G7	Zinc finger protein 30 homolog OS=Homo sapiens GN=ZFP30 PE=2 SV=1 - (ZFP30_HUMAN)	4.21	10.21	2	1	3	5	3.032E7	519	61.5	8.91
P80188	Neutrophil gelatinase-associated lipocalin OS=Homo sapiens GN=LCN2 PE=1 SV=2 - (NGAL_HUMAN)	4.13	33.84	4	1	6	8	3.059E7	198	22.6	8.91
Q96DX7	Tripartite motif-containing protein 44 OS=Homo sapiens GN=TRIM44 PE=1 SV=1 - (TRI44_HUMAN)	4.13	2.62	1	1	1	2	1.539E8	344	38.4	4.21
C9J9K3	40S ribosomal protein SA (Fragment) OS=Homo sapiens GN=RPSA PE=3 SV=1 - (C9J9K3_HUMAN)	4.11	14.39	10	2	4	4	2.377E7	264	29.5	5.25
A8K0D4	cDNA FLJ78693, highly similar to Homo sapiens TAF6-like RNA polymerase II, p300/CBP-associated factor (PCAF)-associated factor, 65kDa (TAF6L), mRNA OS=Homo sapiens PE=2 SV=1 - (A8K0D4_HUMAN)	4.10	6.11	4	1	3	4	4.359E7	622	67.7	9.06
B4E0S6	cDNA FLJ55635, highly similar to pre-mRNA-splicing factorATP-dependent RNA helicase DHX15 (EC 3.6.1.-) OS=Homo sapiens PE=2 SV=1 - (B4E0S6_HUMAN)	4.07	3.95	3	1	4	8	1.251E7	784	89.5	7.46
Q9UM13	Anaphase-promoting complex subunit 10 OS=Homo sapiens GN=ANAPC10 PE=1 SV=1 - (APC10_HUMAN)	4.04	18.38	2	1	3	4	5.630E7	185	21.2	9.06
E4W6B6	RPL27/NME2 fusion protein (Fragment) OS=Homo sapiens GN=RPL27 PE=2 SV=1 - (E4W6B6_HUMAN)	4.02	7.14	3	1	1	2	9.180E6	126	14.2	10.46
Q9P1F3	Costars family protein ABRACL OS=Homo sapiens GN=ABRACL PE=1 SV=1 - (ABRAL_HUMAN)	4.02	16.05	1	1	1	1	1.149E6	81	9.1	6.29
O94761	ATP-dependent DNA helicase Q4 OS=Homo sapiens GN=RECQL4 PE=1 SV=1 - (RECQ4_HUMAN)	4.00	5.13	1	1	6	11	2.190E7	1208	133.0	8.09
P62280	40S ribosomal protein S11 OS=Homo sapiens GN=RPS11 PE=1 SV=3 - (RS11_HUMAN)	3.99	29.11	1	2	5	6	1.457E7	158	18.4	10.30
D6RA63	Protein GAPT (Fragment) OS=Homo sapiens GN=GAPT PE=4 SV=1 - (D6RA63_HUMAN)	3.99	14.71	3	1	1	2	9.823E7	68	7.6	11.80
P0C7V6	Putative protein FAM48B2 OS=Homo sapiens GN=FAM48B2 PE=5 SV=1 - (F48B2_HUMAN)	3.94	7.83	1	1	5	12	2.729E7	817	87.5	8.79
P12532	Creatine kinase U-type, mitochondrial OS=Homo sapiens GN=CKMT1A PE=1 SV=1 - (KCRU_HUMAN)	3.93	10.31	12	1	6	6	3.030E6	417	47.0	8.34
Q5VWQ0	Round spermatid basic protein 1 OS=Homo sapiens GN=RSBN1 PE=2 SV=2 - (RSBN1_HUMAN)	3.86	7.73	3	1	6	12	9.057E8	802	90.0	8.60
B4DLJ5	cDNA FLJ55716, highly similar to Desmocollin-2 OS=Homo sapiens PE=2 SV=1 - (B4DLJ5_HUMAN)	3.83	7.57	9	1	5	6	8.093E7	912	101.0	5.27
Q6PHZ4	GON4L protein (Fragment) OS=Homo sapiens GN=GON4L PE=2 SV=1 - (Q6PHZ4_HUMAN)	3.82	9.11	1	1	4	7	8.637E6	571	65.3	4.77
P61970	Nuclear transport factor 2 OS=Homo sapiens GN=NUTF2 PE=1 SV=1 - (NTF2_HUMAN)	3.79	35.43	2	1	4	5	3.254E7	127	14.5	5.38
Q8N5P7	MGC50722 protein (Fragment) OS=Homo sapiens GN=MGC50722 PE=2 SV=1 - (Q8N5P7_HUMAN)	3.78	4.40	2	1	4	4	1.741E7	1045	113.1	9.86
Q8IXI1	Mitochondrial Rho GTPase 2 OS=Homo sapiens GN=RHOT2 PE=1 SV=2 - (MIRO2_HUMAN)	3.77	5.18	1	1	3	17	2.288E7	618	68.1	5.86
Q8NAV1	Pre-mRNA-splicing factor 38A OS=Homo sapiens GN=PRPF38A PE=1 SV=1 - (PR38A_HUMAN)	3.76	2.56	1	1	1	2	7.119E5	312	37.5	9.96
F8W6N3	Ubiquitin carboxyl-terminal hydrolase BAP1 OS=Homo sapiens GN=BAP1 PE=4 SV=1 - (F8W6N3_HUMAN)	3.76	2.25	3	1	2	11	2.759E7	711	78.1	6.81
Q9Y2S1	Galanin-related peptide OS=Homo sapiens PE=4 SV=1 - (Q9Y2S1_HUMAN)	3.75	12.26	1	1	2	29	1.262E8	106	11.8	5.97
H0YJA4	Chromodomain-helicase-DNA-binding protein 8 (Fragment) OS=Homo sapiens GN=CHD8 PE=4 SV=1 - (H0YJA4_HUMAN)	3.74	16.37	1	1	3	6	2.999E7	226	25.0	9.86
Q7RTP6	Protein-methionine sulfoxide oxidase MICAL3 OS=Homo sapiens GN=MICAL3 PE=1 SV=2 - (MICA3_HUMAN)	3.74	3.45	2	1	8	18	1.464E7	2002	224.2	5.55
H7C146	Microtubule-associated serine/threonine-protein kinase 4 (Fragment) OS=Homo sapiens GN=MAST4 PE=4 SV=1 - (H7C146_HUMAN)	3.69	22.73	1	1	2	17	1.880E9	66	7.5	9.85
Q16658	Fascin OS=Homo sapiens GN=FSCN1 PE=1 SV=3 - (FSCN1_HUMAN)	3.68	9.94	7	1	4	5	5.466E6	493	54.5	7.24
P30041	Peroxiredoxin-6 OS=Homo sapiens GN=PRDX6 PE=1 SV=3 - (PRDX6_HUMAN)	3.64	12.95	2	1	3	4	7.698E7	224	25.0	6.38
Q6IAG3	IL17B protein OS=Homo sapiens GN=IL17B PE=2 SV=1 - (Q6IAG3_HUMAN)	3.63	22.22	2	1	3	5	5.539E6	180	20.5	9.23
Q05BU7	WASF2 protein (Fragment) OS=Homo sapiens GN=WASF2 PE=2 SV=1 - (Q05BU7_HUMAN)	3.62	4.35	3	1	1	2	3.264E7	184	21.4	9.25
Q6N069	N-alpha-acetyltransferase 16, NatA auxiliary subunit OS=Homo sapiens GN=NAA16 PE=1 SV=2 - (NAA16_HUMAN)	3.62	6.25	3	1	5	11	3.730E7	864	101.4	7.87
Q6ZVX7	Non-specific cytotoxic cell receptor protein 1 homolog OS=Homo sapiens GN=NCCRP1 PE=1 SV=1 - (NCRP1_HUMAN)	3.61	13.45	1	1	5	7	8.906E7	275	30.8	6.62
Q9BY12	S phase cyclin A-associated protein in the endoplasmic reticulum OS=Homo sapiens GN=SCAPER PE=1 SV=1 - (SCAPE_HUMAN)	3.60	6.15	5	1	9	13	3.175E7	1399	158.1	7.44
Q86Z22	Putative uncharacterized protein OS=Homo sapiens PE=2 SV=1 - (Q86Z22_HUMAN)	3.60	12.39	4	1	3	3	1.624E6	226	23.8	9.06
B7Z478	Proteasome (Prosome, macropain) subunit, beta type, 2, isoform CRA_b OS=Homo sapiens GN=PSMB2 PE=2 SV=1 - (B7Z478_HUMAN)	3.57	6.25	2	1	1	1	1.013E7	176	20.2	7.44
A8K5A5	cDNA FLJ75393, highly similar to Homo sapiens PHD finger protein 16 (PHF16), mRNA OS=Homo sapiens PE=2 SV=1 - (A8K5A5_HUMAN)	3.56	7.90	2	1	7	18	2.888E7	823	93.8	7.05
P18077	60S ribosomal protein L35a OS=Homo sapiens GN=RPL35A PE=1 SV=2 - (RL35A_HUMAN)	3.55	36.36	4	1	5	6	1.917E7	110	12.5	11.06
B4DP80	cDNA FLJ56357, highly similar to Homo sapiens apolipoprotein A-I binding protein (APOA1BP), mRNA OS=Homo sapiens PE=2 SV=1 - (B4DP80_HUMAN)	3.53	5.86	3	1	2	4	4.435E7	307	33.6	8.73
B4E1B2	cDNA FLJ53691, highly similar to Serotransferrin OS=Homo sapiens PE=2 SV=1 - (B4E1B2_HUMAN)	3.51	9.29	6	1	7	8	5.904E7	678	74.8	7.12
B4DZC0	cDNA FLJ51771, highly similar to SWI/SNF-related matrix-associatedactin-dependent regulator of chromatin subfamily A member5 (EC 3.6.1.-) OS=Homo sapiens PE=2 SV=1 - (B4DZC0_HUMAN)	3.51	7.94	4	1	7	14	7.711E7	995	116.7	8.34
F2Z2S8	40S ribosomal protein S3 OS=Homo sapiens GN=RPS3 PE=4 SV=1 - (F2Z2S8_HUMAN)	3.50	28.21	9	1	3	4	1.570E7	117	13.1	9.91
B9EK40	SPATA21 protein OS=Homo sapiens GN=SPATA21 PE=2 SV=1 - (B9EK40_HUMAN)	3.49	9.64	4	1	4	6	8.158E7	446	49.4	6.79
Q53FR4	Vacuolar protein sorting 35 variant (Fragment) OS=Homo sapiens PE=2 SV=1 - (Q53FR4_HUMAN)	3.42	13.32	4	1	7	8	6.421E7	796	91.6	5.49
O60809	PRAME family member 10 OS=Homo sapiens GN=PRAMEF10 PE=1 SV=3 - (PRA10_HUMAN)	3.36	5.70	3	1	3	7	5.353E7	474	55.0	8.10
B5MD17	Chromobox protein homolog 1 (Fragment) OS=Homo sapiens GN=CBX1 PE=4 SV=1 - (B5MD17_HUMAN)	3.36	9.52	3	1	1	1		168	19.3	5.02
D6RFM0	Ubiquitin-conjugating enzyme E2 D2 (Fragment) OS=Homo sapiens GN=UBE2D2 PE=3 SV=1 - (D6RFM0_HUMAN)	3.31	24.49	8	1	1	1	1.314E6	98	11.1	6.76
Q9NZD1-2	Isoform 2 of G-protein coupled receptor family C group 5 member D OS=Homo sapiens GN=GPRC5D - (GPC5D_HUMAN)	3.23	9.33	3	1	1	1		300	34.1	5.86
A8K3Z3	26S protease regulatory subunit 8 OS=Homo sapiens GN=PSMC5 PE=2 SV=1 - (A8K3Z3_HUMAN)	3.13	12.31	3	1	5	8	5.351E7	398	44.8	8.18
Q9BVC6	Transmembrane protein 109 OS=Homo sapiens GN=TMEM109 PE=1 SV=1 - (TM109_HUMAN)	3.12	12.76	4	1	3	3	8.654E7	243	26.2	10.48
O75340	Programmed cell death protein 6 OS=Homo sapiens GN=PDCD6 PE=1 SV=1 - (PDCD6_HUMAN)	3.07	31.94	5	2	5	5	3.737E6	191	21.9	5.40
Q7RTQ0	Kinesin light chain 1N OS=Homo sapiens GN=KNS2 PE=2 SV=1 - (Q7RTQ0_HUMAN)	2.99	12.18	30	1	6	10	1.747E8	624	70.5	6.77
A8K865	cDNA FLJ75789, highly similar to Homo sapiens synaptophysin-like 1 (SYPL1), transcript variant 1, mRNA OS=Homo sapiens PE=2 SV=1 - (A8K865_HUMAN)	2.97	8.49	4	1	2	3	3.301E7	259	28.5	7.99
B4DPP0	CD9 antigen OS=Homo sapiens GN=CD9 PE=2 SV=1 - (B4DPP0_HUMAN)	2.95	11.35	4	1	2	2	2.370E6	141	15.9	6.74
H0Y8I5	Transmembrane protein 184C (Fragment) OS=Homo sapiens GN=TMEM184C PE=4 SV=1 - (H0Y8I5_HUMAN)	2.95	56.94	1	1	1	1	1.202E7	72	8.2	8.29
Q68DN7	Putative uncharacterized protein DKFZp781P1017 OS=Homo sapiens GN=DKFZp781P1017 PE=2 SV=1 - (Q68DN7_HUMAN)	2.94	5.56	3	1	1	2	1.457E9	252	26.4	6.60
C9J326	Probable guanine nucleotide exchange factor MCF2L2 (Fragment) OS=Homo sapiens GN=MCF2L2 PE=4 SV=1 - (C9J326_HUMAN)	2.93	15.32	1	1	1	2	3.266E6	124	14.0	8.59
Q3LI83	Keratin-associated protein 24-1 OS=Homo sapiens GN=KRTAP24-1 PE=1 SV=1 - (KR241_HUMAN)	2.90	5.12	1	1	1	1	2.997E7	254	27.7	8.29
E9PN17	ATP synthase subunit g, mitochondrial OS=Homo sapiens GN=ATP5L PE=4 SV=1 - (E9PN17_HUMAN)	2.88	17.11	2	1	1	1	3.288E7	76	8.4	10.29
P20933	N(4)-(beta-N-acetylglucosaminyl)-L-asparaginase OS=Homo sapiens GN=AGA PE=1 SV=2 - (ASPG_HUMAN)	2.88	5.49	2	1	2	2	2.017E6	346	37.2	6.28
P0CW22	40S ribosomal protein S17-like OS=Homo sapiens GN=RPS17L PE=3 SV=1 - (RS17L_HUMAN)	2.86	14.81	6	2	2	3	7.106E6	135	15.5	9.85
P61960	Ubiquitin-fold modifier 1 OS=Homo sapiens GN=UFM1 PE=1 SV=1 - (UFM1_HUMAN)	2.77	24.71	3	1	2	4	1.430E7	85	9.1	9.31
Q15102	Platelet-activating factor acetylhydrolase IB subunit gamma OS=Homo sapiens GN=PAFAH1B3 PE=1 SV=1 - (PA1B3_HUMAN)	2.72	7.36	1	1	2	2	3.296E6	231	25.7	6.84
P18621	60S ribosomal protein L17 OS=Homo sapiens GN=RPL17 PE=1 SV=3 - (RL17_HUMAN)	2.72	16.85	2	1	3	3	1.364E7	184	21.4	10.17
Q9BTA9-2	Isoform 2 of WW domain-containing adapter protein with coiled-coil OS=Homo sapiens GN=WAC - (WAC_HUMAN)	2.63	6.31	3	1	2	2	6.673E6	602	65.4	9.36
P15880	40S ribosomal protein S2 OS=Homo sapiens GN=RPS2 PE=1 SV=2 - (RS2_HUMAN)	2.63	22.18	11	1	7	8	5.896E7	293	31.3	10.24
Q86WV2	COX4I1 protein OS=Homo sapiens GN=COX4I1 PE=2 SV=1 - (Q86WV2_HUMAN)	2.57	13.25	5	1	1	1	4.438E6	83	9.3	8.48
Q5U5X8	Protein FAM222A OS=Homo sapiens GN=FAM222A PE=2 SV=1 - (F222A_HUMAN)	2.57	13.05	1	1	4	4	2.238E7	452	46.8	9.03
Q04941	Proteolipid protein 2 OS=Homo sapiens GN=PLP2 PE=1 SV=1 - (PLP2_HUMAN)	2.52	8.55	1	1	1	1	6.866E6	152	16.7	7.24
Q96P20-2	Isoform 1 of NACHT, LRR and PYD domains-containing protein 3 OS=Homo sapiens GN=NLRP3 - (NALP3_HUMAN)	2.52	5.21	6	1	4	4	1.770E8	922	105.9	6.64
C9JZ20	Prohibitin (Fragment) OS=Homo sapiens GN=PHB PE=4 SV=1 - (C9JZ20_HUMAN)	2.46	10.45	10	1	2	2	2.156E7	201	22.3	5.96
P09669	Cytochrome c oxidase subunit 6C OS=Homo sapiens GN=COX6C PE=1 SV=2 - (COX6C_HUMAN)	2.46	26.67	1	1	2	2	2.638E7	75	8.8	10.39
Q04671-3	Isoform 3 of P protein OS=Homo sapiens GN=OCA2 - (P_HUMAN)	2.43	2.69	3	1	2	2	3.852E7	668	74.3	8.50
C9J592	Ras-related protein Rab-7a (Fragment) OS=Homo sapiens GN=RAB7A PE=3 SV=1 - (C9J592_HUMAN)	2.42	8.67	2	1	1	1		150	17.0	8.85
Q9P0J0	NADH dehydrogenase (ubiquinone) 1 alpha subcomplex subunit 13 OS=Homo sapiens GN=NDUFA13 PE=1 SV=3 - (NDUAD_HUMAN)	2.40	18.75	2	1	2	2	6.168E6	144	16.7	8.43
Q4VAN1	BTN1A1 protein OS=Homo sapiens GN=BTN1A1 PE=2 SV=1 - (Q4VAN1_HUMAN)	2.39	5.47	3	1	3	3	4.472E6	494	55.4	5.38
I3L1H9	Zymogen granule protein 16 homolog B (Fragment) OS=Homo sapiens GN=ZG16B PE=4 SV=1 - (I3L1H9_HUMAN)	2.37	13.04	4	1	1	1		69	7.5	9.32
P62424	60S ribosomal protein L7a OS=Homo sapiens GN=RPL7A PE=1 SV=2 - (RL7A_HUMAN)	2.36	12.78	7	2	5	5	3.246E6	266	30.0	10.61
Q5TA02	Glutathione S-transferase omega 1 (Fragment) OS=Homo sapiens GN=GSTO1 PE=2 SV=1 - (Q5TA02_HUMAN)	2.35	10.50	4	1	2	2	1.040E7	200	23.3	7.21
Q9Y3U8	60S ribosomal protein L36 OS=Homo sapiens GN=RPL36 PE=1 SV=3 - (RL36_HUMAN)	2.35	9.52	1	1	1	1	1.181E7	105	12.2	11.59
H7C2D7	Claudin-15 (Fragment) OS=Homo sapiens GN=CLDN15 PE=4 SV=1 - (H7C2D7_HUMAN)	2.33	10.38	3	1	1	1	8.347E5	106	11.1	7.03
Q53FH6	Mitogen-activated protein kinase kinase 1 interacting protein 1 variant (Fragment) OS=Homo sapiens PE=2 SV=1 - (Q53FH6_HUMAN)	2.31	8.06	2	1	1	1	5.924E6	124	13.6	7.34
O15277	Matrix metalloproteinase RASI-1 OS=Homo sapiens PE=4 SV=1 - (O15277_HUMAN)	2.29	14.12	1	1	1	1	4.953E6	85	9.0	11.06
C9JUG7	F-actin-capping protein subunit alpha-2 OS=Homo sapiens GN=CAPZA2 PE=4 SV=1 - (C9JUG7_HUMAN)	2.29	15.75	7	1	2	2	1.082E7	146	16.7	5.11
Q9NQC3	Reticulon-4 OS=Homo sapiens GN=RTN4 PE=1 SV=2 - (RTN4_HUMAN)	2.26	1.85	6	1	2	3	5.693E7	1192	129.9	4.50
C9JUL6	Uncharacterized protein OS=Homo sapiens PE=4 SV=1 - (C9JUL6_HUMAN)	2.22	2.60	1	1	1	2	6.842E6	385	41.0	11.53
Q9ULE0	Protein WWC3 OS=Homo sapiens GN=WWC3 PE=2 SV=3 - (WWC3_HUMAN)	2.21	5.22	3	1	6	8	6.892E7	1092	122.6	6.37
Q6P4A8	Phospholipase B-like 1 OS=Homo sapiens GN=PLBD1 PE=1 SV=2 - (PLBL1_HUMAN)	2.20	10.31	2	1	5	10	1.200E8	553	63.2	9.06
P39023	60S ribosomal protein L3 OS=Homo sapiens GN=RPL3 PE=1 SV=2 - (RL3_HUMAN)	2.17	16.13	7	1	6	7	2.485E7	403	46.1	10.18
Q9NQ66-2	Isoform B of 1-phosphatidylinositol 4,5-bisphosphate phosphodiesterase beta-1 OS=Homo sapiens GN=PLCB1 - (PLCB1_HUMAN)	2.16	5.54	6	1	6	7	8.310E7	1173	133.6	6.42
Q9NZM4-2	Isoform 2 of Glioma tumor suppressor candidate region gene 1 protein OS=Homo sapiens GN=GLTSCR1 - (GSCR1_HUMAN)	2.16	3.11	2	1	3	3	6.228E7	1318	134.1	9.23
P28070	Proteasome subunit beta type-4 OS=Homo sapiens GN=PSMB4 PE=1 SV=4 - (PSB4_HUMAN)	2.15	8.71	2	1	2	2	1.308E7	264	29.2	5.97
Q71RD3	PP11517 OS=Homo sapiens PE=2 SV=1 - (Q71RD3_HUMAN)	2.14	4.98	1	1	1	1	4.440E6	201	22.2	11.77
Q6ZNE9	RUN and FYVE domain-containing protein 4 OS=Homo sapiens GN=RUFY4 PE=2 SV=2 - (RUFY4_HUMAN)	2.13	14.36	2	1	7	27	9.718E7	571	64.3	6.83
Q96EG3	Zinc finger protein 837 OS=Homo sapiens GN=ZNF837 PE=2 SV=2 - (ZN837_HUMAN)	2.12	5.08	1	1	3	6	7.961E7	531	58.0	9.25
H7C5G3	F-box only protein 9 (Fragment) OS=Homo sapiens GN=FBXO9 PE=4 SV=1 - (H7C5G3_HUMAN)	2.10	5.84	1	1	1	3	7.384E7	154	18.2	9.38
A8K6K4	cDNA FLJ77565, highly similar to Homo sapiens interleukin 1 receptor accessory protein (IL1RAP), transcript variant 1, mRNA OS=Homo sapiens PE=2 SV=1 - (A8K6K4_HUMAN)	2.09	9.12	6	1	3	6	4.558E7	570	65.4	8.22
Q13535-2	Isoform 2 of Serine/threonine-protein kinase ATR OS=Homo sapiens GN=ATR - (ATR_HUMAN)	2.02	4.69	3	1	13	29	8.900E7	2580	294.0	7.43
E9PJD9	60S ribosomal protein L27a OS=Homo sapiens GN=RPL27A PE=3 SV=1 - (E9PJD9_HUMAN)	2.02	18.68	6	1	2	2	1.985E7	91	10.1	10.21
Q3SYB5	SERPINB12 protein OS=Homo sapiens GN=SERPINB12 PE=2 SV=1 - (Q3SYB5_HUMAN)	2.02	10.93	3	1	2	3	1.229E8	183	20.9	5.87
O43313	ATM interactor OS=Homo sapiens GN=ATMIN PE=1 SV=2 - (ATMIN_HUMAN)	2.01	4.37	3	1	3	7	5.476E7	823	88.3	5.16
E5G0Z0	MHC class I antigen (Fragment) OS=Homo sapiens GN=HLA-Cw PE=3 SV=1 - (E5G0Z0_HUMAN)	2.00	4.26	1	1	1	1	7.613E6	188	21.7	6.60
P16402	Histone H1.3 OS=Homo sapiens GN=HIST1H1D PE=1 SV=2 - (H13_HUMAN)	2.00	24.43	6	2	6	27	1.156E9	221	22.3	11.02
Q9UNM1	Chaperonin 10-related protein (Fragment) OS=Homo sapiens GN=EPFP1 PE=2 SV=1 - (Q9UNM1_HUMAN)	1.97	7.22	3	1	1	1	7.543E6	97	10.3	9.00
Q9NXW9	Probable alpha-ketoglutarate-dependent dioxygenase ABH4 OS=Homo sapiens GN=ALKBH4 PE=1 SV=1 - (ALKB4_HUMAN)	1.97	10.60	1	1	3	3	2.121E7	302	33.8	6.67
Q6NXR8	Ribosomal protein S3A OS=Homo sapiens GN=RPS3A PE=2 SV=1 - (Q6NXR8_HUMAN)	1.95	18.18	10	1	7	11	1.716E7	264	30.0	9.73
Q9BTF0	THUMP domain-containing protein 2 OS=Homo sapiens GN=THUMPD2 PE=2 SV=2 - (THUM2_HUMAN)	1.93	8.95	5	1	3	6	1.021E8	503	56.4	7.81
Q6ZRK0	Uncharacterized protein OS=Homo sapiens PE=2 SV=1 - (Q6ZRK0_HUMAN)	1.85	9.49	1	1	2	4	3.556E6	158	16.4	9.00
B4DVX8	cDNA FLJ53566 OS=Homo sapiens PE=2 SV=1 - (B4DVX8_HUMAN)	1.84	6.19	1	1	1	1	1.401E7	210	23.0	10.08
P56556	NADH dehydrogenase (ubiquinone) 1 alpha subcomplex subunit 6 OS=Homo sapiens GN=NDUFA6 PE=1 SV=3 - (NDUA6_HUMAN)	1.83	5.19	1	1	1	1	3.589E6	154	17.9	10.14
G3V3E8	Epididymal secretory protein E1 OS=Homo sapiens GN=NPC2 PE=4 SV=1 - (G3V3E8_HUMAN)	1.83	8.62	10	1	2	2	1.076E6	174	19.2	8.44
E9PNX2	Neuronal acetylcholine receptor subunit alpha-10 OS=Homo sapiens GN=CHRNA10 PE=4 SV=1 - (E9PNX2_HUMAN)	1.81	6.50	1	1	1	1	4.270E6	123	13.2	9.01
Q9BZJ0	Crooked neck-like protein 1 OS=Homo sapiens GN=CRNKL1 PE=1 SV=4 - (CRNL1_HUMAN)	1.81	5.78	1	1	5	12	2.709E7	848	100.4	8.00
P62306	Small nuclear ribonucleoprotein F OS=Homo sapiens GN=SNRPF PE=1 SV=1 - (RUXF_HUMAN)	1.80	9.30	1	1	1	2	6.495E6	86	9.7	4.67
Q5TA51	Cleavage and polyadenylation specific factor 3-like (Fragment) OS=Homo sapiens GN=CPSF3L PE=2 SV=1 - (Q5TA51_HUMAN)	1.80	10.71	1	1	3	3	3.856E7	308	32.9	8.43
P16152	Carbonyl reductase (NADPH) 1 OS=Homo sapiens GN=CBR1 PE=1 SV=3 - (CBR1_HUMAN)	1.78	9.39	1	1	2	2	1.284E7	277	30.4	8.32
Q99714	3-hydroxyacyl-CoA dehydrogenase type-2 OS=Homo sapiens GN=HSD17B10 PE=1 SV=3 - (HCD2_HUMAN)	1.77	3.07	1	1	1	1	9.205E6	261	26.9	7.78
I3NI19	Neuropeptide B-29 OS=Homo sapiens GN=NPB PE=4 SV=1 - (I3NI19_HUMAN)	1.77	5.13	1	1	1	1	3.504E6	156	16.3	10.33
Q8IUC2	Keratin-associated protein 8-1 OS=Homo sapiens GN=KRTAP8-1 PE=2 SV=1 - (KRA81_HUMAN)	1.75	12.70	1	1	2	6	5.043E7	63	6.8	7.74
P22528	Cornifin-B OS=Homo sapiens GN=SPRR1B PE=1 SV=2 - (SPR1B_HUMAN)	1.74	26.97	5	2	2	5	4.152E6	89	9.9	8.48
P54851	Epithelial membrane protein 2 OS=Homo sapiens GN=EMP2 PE=2 SV=1 - (EMP2_HUMAN)	1.74	3.59	1	1	1	2	1.363E6	167	19.2	7.62
Q6UW63	KDEL motif-containing protein 1 OS=Homo sapiens GN=KDELC1 PE=1 SV=1 - (KDEL1_HUMAN)	1.72	2.99	2	1	2	3	3.589E6	502	58.0	7.71
Q99497	Protein DJ-1 OS=Homo sapiens GN=PARK7 PE=1 SV=2 - (PARK7_HUMAN)	1.71	3.70	1	1	1	1	6.404E6	189	19.9	6.79
F8VQQ8	Ubiquitin-conjugating enzyme E2 N OS=Homo sapiens GN=UBE2N PE=3 SV=1 - (F8VQQ8_HUMAN)	1.70	21.35	4	1	2	2	2.033E8	89	10.1	8.79
P04632	Calpain small subunit 1 OS=Homo sapiens GN=CAPNS1 PE=1 SV=1 - (CPNS1_HUMAN)	1.69	3.36	1	1	1	1	2.694E5	268	28.3	5.20
O75844	CAAX prenyl protease 1 homolog OS=Homo sapiens GN=ZMPSTE24 PE=1 SV=2 - (FACE1_HUMAN)	1.68	2.95	1	1	2	7	1.882E8	475	54.8	7.49
Q1JQ76	Ribosomal protein (Fragment) OS=Homo sapiens GN=RPL10A PE=2 SV=1 - (Q1JQ76_HUMAN)	1.67	9.71	4	1	4	4	2.800E8	206	23.5	10.07
Q6ZP98	Putative uncharacterized protein C16orf47 OS=Homo sapiens GN=C16orf47 PE=2 SV=1 - (CP047_HUMAN)	1.67	19.55	1	1	2	4	2.993E6	133	14.6	10.15
B4DI85	ADP-ribosylation factor-like 8B, isoform CRA_a OS=Homo sapiens GN=ARL8B PE=2 SV=1 - (B4DI85_HUMAN)	1.67	7.98	4	1	2	3	2.251E6	163	18.6	5.99
B4DT92	Acetylcholine receptor subunit delta OS=Homo sapiens GN=CHRND PE=2 SV=1 - (B4DT92_HUMAN)	1.66	5.98	3	1	3	7	3.009E7	502	57.0	6.99
F6KCZ3	Phosphatase and tensin-like protein OS=Homo sapiens PE=4 SV=1 - (F6KCZ3_HUMAN)	1.66	1.99	5	1	1	3	4.167E7	403	47.1	6.27
B4DUR3	cDNA FLJ55386, highly similar to Homo sapiens methyl-CpG binding domain protein 1 (MBD1), transcript variant 4, mRNA OS=Homo sapiens PE=2 SV=1 - (B4DUR3_HUMAN)	1.66	7.48	1	1	3	4	3.583E7	535	58.8	8.92
A8K0S5	cDNA FLJ77003, highly similar to Homo sapiens cerebellin 4 (CBLN4), mRNA OS=Homo sapiens PE=2 SV=1 - (A8K0S5_HUMAN)	1.65	12.44	2	1	2	2	3.558E7	201	21.8	8.73
P07108-4	Isoform 4 of Acyl-CoA-binding protein OS=Homo sapiens GN=DBI - (ACBP_HUMAN)	1.65	49.61	8	1	7	15	3.397E7	129	14.4	9.42
A8MT70	Zinc finger B-box domain-containing protein 1 OS=Homo sapiens GN=ZBBX PE=2 SV=3 - (ZBBX_HUMAN)	1.64	1.88	4	1	2	7	5.004E6	800	91.4	5.59
Q8TB52	F-box only protein 30 OS=Homo sapiens GN=FBXO30 PE=1 SV=3 - (FBX30_HUMAN)	1.64	5.77	1	1	4	9	3.409E7	745	82.3	5.40
Q8NEP3-3	Isoform 3 of Dynein assembly factor 1, axonemal OS=Homo sapiens GN=DNAAF1 - (DAAF1_HUMAN)	1.64	4.31	3	1	2	3	1.106E6	418	47.2	5.24
O95833	Chloride intracellular channel protein 3 OS=Homo sapiens GN=CLIC3 PE=1 SV=2 - (CLIC3_HUMAN)	1.62	2.54	1	1	1	1	6.121E6	236	26.6	6.43
Q6ZW33	MICAL C-terminal-like protein OS=Homo sapiens GN=MICALCL PE=2 SV=3 - (MICLK_HUMAN)	1.62	8.63	1	1	6	13	1.204E8	695	77.2	8.50
Q9XRX5	HERV-H LTR-associating protein 3 OS=Homo sapiens GN=HHLA3 PE=1 SV=3 - (HHLA3_HUMAN)	1.62	23.68	1	1	3	3	8.753E6	114	12.9	8.82
O95429-2	Isoform 2 of BAG family molecular chaperone regulator 4 OS=Homo sapiens GN=BAG4 - (BAG4_HUMAN)	0.00	2.85	3	1	1	2	4.121E7	421	45.4	5.05
P20290	Transcription factor BTF3 OS=Homo sapiens GN=BTF3 PE=1 SV=1 - (BTF3_HUMAN)	0.00	20.39	4	1	3	5	1.684E7	206	22.2	9.38
Q9BRK5-4	Isoform 4 of 45 kDa calcium-binding protein OS=Homo sapiens GN=SDF4 - (CAB45_HUMAN)	0.00	4.46	8	1	1	3	6.631E5	202	23.0	6.29
Q8IX95	Putative cutaneous T-cell lymphoma-associated antigen 3 OS=Homo sapiens GN=CTAGE3P PE=5 SV=1 - (CTGE3_HUMAN)	0.00	21.52	1	1	2	3	3.665E6	158	18.0	4.92
Q6SJ93	Protein FAM111B OS=Homo sapiens GN=FAM111B PE=2 SV=1 - (F111B_HUMAN)	0.00	7.90	3	1	7	9	1.611E8	734	84.6	8.59
Q9Y5L2	Hypoxia-inducible lipid droplet-associated protein OS=Homo sapiens GN=HILPDA PE=1 SV=1 - (HLPDA_HUMAN)	0.00	15.87	1	1	1	1	6.411E5	63	6.9	7.43
P48735	Isocitrate dehydrogenase (NADP), mitochondrial OS=Homo sapiens GN=IDH2 PE=1 SV=2 - (IDHP_HUMAN)	0.00	5.75	5	1	3	6	1.308E7	452	50.9	8.69
P78414	Iroquois-class homeodomain protein IRX-1 OS=Homo sapiens GN=IRX1 PE=1 SV=3 - (IRX1_HUMAN)	0.00	7.29	1	1	2	3	1.119E8	480	49.6	6.20
P01597	Ig kappa chain V-I region DEE OS=Homo sapiens PE=1 SV=1 - (KV105_HUMAN)	0.00	10.19	1	1	1	3	1.044E7	108	11.7	9.36
Q96G30	Melanocortin-2 receptor accessory protein 2 OS=Homo sapiens GN=MRAP2 PE=2 SV=2 - (MRAP2_HUMAN)	0.00	16.59	1	1	1	1	2.762E8	205	23.5	5.01
O76036-5	Isoform 5 of Natural cytotoxicity triggering receptor 1 OS=Homo sapiens GN=NCR1 - (NCTR1_HUMAN)	0.00	7.11	6	1	1	1	3.805E7	197	22.2	7.50
Q96QR8	Transcriptional activator protein Pur-beta OS=Homo sapiens GN=PURB PE=1 SV=3 - (PURB_HUMAN)	0.00	7.05	1	1	3	12	6.876E6	312	33.2	5.43
Q15858-3	Isoform 3 of Sodium channel protein type 9 subunit alpha OS=Homo sapiens GN=SCN9A - (SCN9A_HUMAN)	0.00	3.39	6	1	6	9	1.546E7	1977	225.1	7.06
Q9H5Y7	SLIT and NTRK-like protein 6 OS=Homo sapiens GN=SLITRK6 PE=1 SV=3 - (SLIK6_HUMAN)	0.00	4.64	1	1	2	7	8.469E7	841	95.0	6.52
Q9GZM7-3	Isoform 3 of Tubulointerstitial nephritis antigen-like OS=Homo sapiens GN=TINAGL1 - (TINAL_HUMAN)	0.00	6.65	1	1	2	7	1.654E8	436	48.8	7.33
Q8NC26	Zinc finger protein 114 OS=Homo sapiens GN=ZNF114 PE=2 SV=1 - (ZN114_HUMAN)	0.00	9.83	1	1	2	3	1.269E7	417	47.7	8.59
P17022-2	Isoform 2 of Zinc finger protein 18 OS=Homo sapiens GN=ZNF18 - (ZNF18_HUMAN)	0.00	12.59	3	1	4	4	1.627E7	548	62.2	5.97
F8W1E4	Protein G8 OS=Homo sapiens GN=C6orf48 PE=4 SV=1 - (F8W1E4_HUMAN)	0.00	40.00	1	1	2	3	9.186E6	125	13.1	8.40
Q8WVC2	40S ribosomal protein S21 OS=Homo sapiens GN=RPS21 PE=2 SV=1 - (Q8WVC2_HUMAN)	0.00	12.35	2	1	1	1	1.605E7	81	8.8	8.50
B7Z7G5	Probable tRNA pseudouridine synthase 2 OS=Homo sapiens GN=TRUB2 PE=2 SV=1 - (B7Z7G5_HUMAN)	0.00	5.82	2	1	1	1	8.413E5	275	30.4	6.99
B7Z7N4	Poly(U)-specific endoribonuclease OS=Homo sapiens GN=ENDOU PE=2 SV=1 - (B7Z7N4_HUMAN)	0.00	13.91	5	1	2	7	1.630E8	151	17.6	6.30
A1BQX2	MARVEL domain-containing protein 2 OS=Homo sapiens GN=MARVELD2 PE=2 SV=1 - (A1BQX2_HUMAN)	0.00	8.14	6	1	5	8	3.164E7	442	51.1	7.58
E9PAT1	Protein phosphatase 1 regulatory subunit 14D OS=Homo sapiens GN=PPP1R14D PE=4 SV=1 - (E9PAT1_HUMAN)	0.00	11.00	1	1	1	2	2.007E8	200	22.4	10.36
Q5GRG0	ESC42-RELB OS=Homo sapiens PE=2 SV=1 - (Q5GRG0_HUMAN)	0.00	8.05	2	1	1	3	1.938E8	87	10.0	8.73
B5MDV0	Protein monoglycylase TTLL8 OS=Homo sapiens GN=TTLL8 PE=4 SV=2 - (B5MDV0_HUMAN)	0.00	9.95	2	1	6	8	5.845E7	834	92.8	8.16
I3L3D7	Serine protease 33 (Fragment) OS=Homo sapiens GN=PRSS33 PE=4 SV=1 - (I3L3D7_HUMAN)	0.00	9.75	1	1	1	1	5.597E5	236	25.4	9.23
I3L3X7	Proteasome subunit beta type-6 (Fragment) OS=Homo sapiens GN=PSMB6 PE=4 SV=1 - (I3L3X7_HUMAN)	0.00	9.80	2	1	1	1		102	10.7	5.31
E9PQT9	Disks large homolog 2 (Fragment) OS=Homo sapiens GN=DLG2 PE=4 SV=1 - (E9PQT9_HUMAN)	0.00	20.21	7	1	2	9	4.684E7	94	10.8	9.63
Q8N730	Peripheral-type Benzodiazepine Receptor (Fragment) OS=Homo sapiens GN=PBR PE=2 SV=1 - (Q8N730_HUMAN)	0.00	13.11	4	1	1	1	8.724E6	61	6.7	9.06
A8K0H3	cDNA FLJ78093, highly similar to Homo sapiens ribosomal protein L29 (RPL29), mRNA OS=Homo sapiens PE=2 SV=1 - (A8K0H3_HUMAN)	0.00	5.03	3	1	1	2	2.415E6	159	17.8	11.74
B2RD27	cDNA, FLJ96428, highly similar to Homo sapiens proteasome (prosome, macropain) 26S subunit, non-ATPase, 7 (Mov34 homolog) (PSMD7), mRNA OS=Homo sapiens PE=2 SV=1 - (B2RD27_HUMAN)	0.00	8.95	5	1	2	3	1.390E7	324	37.0	6.77
D9MXF4	Fanconi anemia associated nuclease 1 OS=Homo sapiens GN=FAN1 PE=2 SV=1 - (D9MXF4_HUMAN)	0.00	2.36	2	1	2	2	5.861E7	1017	114.3	7.25
E9PL38	26S proteasome non-ATPase regulatory subunit 13 (Fragment) OS=Homo sapiens GN=PSMD13 PE=4 SV=1 - (E9PL38_HUMAN)	0.00	10.27	8	1	2	2	5.912E7	263	29.7	6.30
Q8NF08	FLJ00396 protein (Fragment) OS=Homo sapiens GN=FLJ00396 PE=2 SV=1 - (Q8NF08_HUMAN)	0.00	18.02	1	1	1	2	1.491E7	172	19.3	11.21
F5H402	Aryl hydrocarbon receptor nuclear translocator-like protein 2 OS=Homo sapiens GN=ARNTL2 PE=4 SV=1 - (F5H402_HUMAN)	0.00	11.67	11	1	4	8	1.486E7	540	60.4	8.66
H0Y6B5	Abl interactor 2 (Fragment) OS=Homo sapiens GN=ABI2 PE=4 SV=1 - (H0Y6B5_HUMAN)	0.00	4.78	1	1	1	4	2.470E8	293	31.9	5.33
A8K8K6	cDNA FLJ76430, highly similar to Homo sapiens zinc finger protein 165 (ZNF165), mRNA OS=Homo sapiens PE=2 SV=1 - (A8K8K6_HUMAN)	0.00	8.04	3	1	3	6	7.224E7	485	55.7	7.15
B7Z210	cDNA FLJ50128, highly similar to Homo sapiens acyl-CoA synthetase bubblegum family member 1 (ACSBG1), mRNA OS=Homo sapiens PE=2 SV=1 - (B7Z210_HUMAN)	0.00	6.64	5	1	3	3	4.660E6	482	53.9	5.15
E9PEN5	Intermediate filament family orphan 2 OS=Homo sapiens GN=IFFO2 PE=4 SV=1 - (E9PEN5_HUMAN)	0.00	6.29	2	1	3	7	4.790E7	461	50.9	4.96
E9PIE0	Connector enhancer of kinase suppressor of ras 1 OS=Homo sapiens GN=CNKSR1 PE=4 SV=1 - (E9PIE0_HUMAN)	0.00	19.05	1	1	1	1	2.180E5	63	6.9	9.44
C9JQH3	Tripartite motif-containing protein 73 OS=Homo sapiens GN=TRIM73 PE=4 SV=1 - (C9JQH3_HUMAN)	0.00	5.88	5	1	1	1	6.317E6	119	13.9	6.10
Q86W19	Protease serine 1 (Fragment) OS=Homo sapiens GN=PRSS1 PE=2 SV=1 - (Q86W19_HUMAN)	0.00	23.81	1	1	1	4	8.047E7	84	9.2	9.47
F6XQ08	Lymphocyte antigen 6 complex locus protein G6d (Fragment) OS=Homo sapiens GN=LY6G6D PE=4 SV=1 - (F6XQ08_HUMAN)	0.00	16.09	2	1	2	2	7.862E5	174	18.8	10.62

Appendix H  

**Table 10:** Gene ontology of common proteins across five samples.

S. no.	Uniprot ID	ID EMSEBL	Protein name	GO molecular function	GO biological process	GO cellular component	Protein class	Species
1	P35908	HUMAN|Ensembl=ENSG00000172867|UniProtKB=P35908	Keratin, type II cytoskeletal 2 epidermal	structural constituent of cytoskeleton	cellular process cellular component morphogenesis cellular component organization	intermediate filament cytoskeleton intracellular	structural protein intermediate filament	Homo sapiens
2	P15924	HUMAN|Ensembl=ENSG00000096696|UniProtKB=P15924	Desmoplakin	structural constituent of cytoskeleton actin binding	cellular component movement cellular component morphogenesis cellular component organization	actin cytoskeleton intracellular	non-motor actin binding protein	Homo sapiens
3	P62805	HUMAN|Ensembl=ENSG00000198558|UniProtKB=P62805	Histone H4	-	-	-	-	Homo sapiens
4	P62805	HUMAN|Ensembl=ENSG00000198518|UniProtKB=P62805	Histone H4	-	-	-	-	Homo sapiens
5	P04792	HUMAN|Ensembl=ENSG00000106211|UniProtKB=P04792	Heat shock protein beta-1	structural molecule activity	immune system process protein folding muscle contraction visual perception sensory perception response to stress	-	structural protein chaperone	Homo sapiens
6	P31947	HUMAN|Ensembl=ENSG00000175793|UniProtKB=P31947	14-3-3 protein sigma	-	cell cycle cell communication	-	chaperone	Homo sapiens
7	O94819	HUMAN|Ensembl=ENSG00000176595|UniProtKB=O94819	Kelch repeat and BTB domain-containing protein 11	-	-	-	-	Homo sapiens
8	Q15828	HUMAN|Ensembl=ENSG00000175315|UniProtKB=Q15828	Cystatin-M	cysteine-type peptidase activity protein binding cysteine-type endopeptidase inhibitor activity	proteolysis regulation of catalytic activity	-	cysteine protease inhibitor	Homo sapiens
9	Q6ZQQ6	HUMAN|Ensembl=ENSG00000171804|UniProtKB=Q6ZQQ6	WD repeat-containing protein 87	catalytic activity mRNA binding	RNA splicing, via transesterification reactions mRNA splicing, via spliceosome RNA splicing, via transesterification reactions cellular process	-	mRNA splicing factor	Homo sapiens
10	P04083	HUMAN|Ensembl=ENSG00000135046|UniProtKB=P04083	Annexin A1	-	fatty acid metabolic process cell communication	-	-	Homo sapiens
11	P06703	HUMAN|Ensembl=ENSG00000197956|UniProtKB=P06703	Protein S100-A6	calcium ion binding growth factor activity calmodulin binding	macrophage activation DNA replication cell cycle cell communication	-	growth factor calmodulin	Homo sapiens
12	P31151	HUMAN|Ensembl=ENSG00000143556|UniProtKB=P31151	Protein S100-A7	calcium ion binding receptor binding calmodulin binding	macrophage activation DNA replication cell cycle cell communication	-	signaling molecule calmodulin	Homo sapiens
13	Q8IXI1	HUMAN|Ensembl=ENSG00000140983|UniProtKB=Q8IXI1	Mitochondrial Rho GTPase 2	GTPase activity protein binding	metabolic process cell communication intracellular protein transport receptor-mediated endocytosis	-	small GTPase	Homo sapiens
14	Q9Y3U8	HUMAN|Ensembl=ENSG00000130255|UniProtKB=Q9Y3U8	60S ribosomal protein L36	structural constituent of ribosome nucleic acid binding	translation	-	ribosomal protein	Homo sapiens
15	Q99497	HUMAN|Ensembl=ENSG00000116288|UniProtKB=Q99497	Protein DJ-1	sequence-specific DNA binding transcription factor activity cysteine-type peptidase activity sequence-specific DNA binding transcription factor activity RNA binding	transcription from RNA polymerase II promoter proteolysis response to stress regulation of transcription from RNA polymerase II promoter	-	transcription factor cysteine protease RNA binding protein cysteine protease	Homo sapiens
16	Q14525	HUMAN|Ensembl=ENSG00000131738|UniProtKB=Q14525	Keratin, type I cuticular Ha3-II, KRT33B	structural constituent of cytoskeleton	cellular process cellular component morphogenesis cellular component organization	intermediate filament cytoskeleton intracellular	structural protein intermediate filament	Homo sapiens
17	Q92764	HUMAN|Ensembl=ENSG00000197079|UniProtKB=Q92764	Keratin, type I cuticular Ha5 KRT35	structural constituent of cytoskeleton	cellular process cellular component morphogenesis cellular component organization	intermediate filament cytoskeleton intracellular	structural protein intermediate filament	Homo sapiens
18	Q04695	HUMAN|Ensembl=ENSG00000128422|UniProtKB=Q04695	Keratin, type I cytoskeletal 17	structural constituent of cytoskeleton	cellular process cellular component morphogenesis cellular component organization	intermediate filament cytoskeleton intracellular	structural protein intermediate filament	Homo sapiens
19	Q3SY84	HUMAN|Ensembl=ENSG00000139648|UniProtKB=Q3SY84	Keratin, type II cytoskeletal 71	structural constituent of cytoskeleton	cellular process, cellular component morphogenesis, cellular component organization	intermediate filament cytoskeleton, intracellular	structural protein, intermediate filament	Homo sapiens
20	Q7RTS7	HUMAN|Ensembl=ENSG00000170484|UniProtKB=Q7RTS7	Keratin, type II cytoskeletal 74, KRT74	structural constituent of cytoskeleton	cellular process, cellular component morphogenesis, cellular component organization	intermediate filament cytoskeleton, intracellular	structural protein, intermediate filament	Homo sapiens
21	P62805	HUMAN|Ensembl=ENSG00000197061|UniProtKB=P62805	Histone H4	-	-	-	-	Homo sapiens
22	P62805	HUMAN|Ensembl=ENSG00000196176|UniProtKB=P62805	Histone H4	-	-	-	-	Homo sapiens
23	P35579	HUMAN|Ensembl=ENSG00000100345|UniProtKB=P35579	Myosin-9 MYH9	motor activity structural constituent of cytoskeleton protein binding enzyme regulator activity	metabolic process, cytokinesis, cellular component movement, mitosis, cell communication, muscle contraction, sensory perception of sound, sensory perception, mesoderm development, cellular component morphogenesis, muscle organ development, intracellular protein transport, vesicle-mediated transport, regulation of catalytic activity, cellular component organization	plasma membrane cell junction actin cytoskeleton intracellular	G-protein modulator actin binding motor protein cell junction protein	Homo sapiens
24	Q7Z406	HUMAN|Ensembl=ENSG00000105357|UniProtKB=Q7Z406	Myosin-14	motor activity structural constituent of cytoskeleton protein binding enzyme regulator activity	metabolic process, cytokinesis, cellular component movement, mitosis, cell communication, muscle contraction, sensory perception of sound, sensory perception, mesoderm development, cellular component morphogenesis, muscle organ development, intracellular protein transport, vesicle-mediated transport, regulation of catalytic activity, cellular component organization	plasma membrane, cell junction actin cytoskeleton intracellular	G-protein modulator, actin binding motor protein cell junction protein	Homo sapiens
25	P55072	HUMAN|Ensembl=ENSG00000165280|UniProtKB=P55072	Transitional endoplasmic reticulum ATPase, VCP	-	-	-	-	Homo sapiens
26	P05109	HUMAN|Ensembl=ENSG00000143546|UniProtKB=P05109	Protein S100-A8	calcium ion binding, growth factor activity, calmodulin binding	macrophage activation, DNA replication, cell cycle, cell communication	-	growth factor, calmodulin	Homo sapiens
27	P04075	HUMAN|Ensembl=ENSG00000149925|UniProtKB=P04075	Fructose-bisphosphate aldolase A	-	-	-	-	Homo sapiens
28	P62851	HUMAN|Ensembl=ENSG00000118181|UniProtKB=P62851	40S ribosomal protein S25	-	-	-	-	Homo sapiens
29	P68402	HUMAN|Ensembl=ENSG00000168092|UniProtKB=P68402	Platelet-activating factor acetylhydrolase IB subunit beta	-	-	-	-	Homo sapiens
30	P23284	HUMAN|Ensembl=ENSG00000166794|UniProtKB=P23284	Peptidyl-prolyl cis-trans isomerase B	isomerase activity	immune system process, protein folding, intracellular protein transport, nuclear transport	-	isomerase	Homo sapiens
31	Q5VYY1	HUMAN|Ensembl=ENSG00000152766|UniProtKB=Q5VYY1	Ankyrin repeat domain-containing protein 22	phospholipase activity, protein binding	immune system process, apoptotic process, phospholipid metabolic process, transcription from RNA polymerase II promoter, phospholipid metabolic process, cell communication, apoptotic process, response to stress, intracellular protein transport, regulation of transcription from RNA polymerase II promoter	-	phospholipase, enzyme modulator	Homo sapiens
32	Q9HCY8	HUMAN|Ensembl=ENSG00000189334|UniProtKB=Q9HCY8	Protein S100-A14	calcium ion binding	macrophage activation, DNA replication, cell cycle, cell communication	-	signaling molecule, calmodulin	Homo sapiens
33	P22314	HUMAN|Ensembl=ENSG00000130985|UniProtKB=P22314	Ubiquitin-like modifier-activating enzyme 1	ligase activity	coenzyme metabolic process, cellular protein modification process, proteolysis, cell communication, intracellular protein transport, nuclear transport	-	transfer/carrier protein, ligase	Homo sapiens
34	P61254	HUMAN|Ensembl=ENSG00000161970|UniProtKB=P61254	60S ribosomal protein L26	structural constituent of ribosome, nucleic acid binding	translation	-	ribosomal protein	Homo sapiens
35	P62273	HUMAN|Ensembl=ENSG00000213741|UniProtKB=P62273	40S ribosomal protein S29	-	-	-	-	Homo sapiens
36	P48047	HUMAN|Ensembl=ENSG00000241837|UniProtKB=P48047	ATP synthase subunit O, mitochondrial	hydrolase activity, cation transmembrane transporter activity, hydrogen ion transmembrane transporter activity	coenzyme metabolic process, nucleobase-containing compound metabolic process, cation transport	-	ATP synthase, hydrolase	Homo sapiens
37	P80188	HUMAN|Ensembl=ENSG00000148346|UniProtKB=P80188	Neutrophil gelatinase-associated lipocalin	isomerase activity binding,	fatty acid biosynthetic process, cell communication, response to pheromone transport,	-	transfer/carrier protein, isomerase	Homo sapiens
38	P16402	HUMAN|Ensembl=ENSG00000124575|UniProtKB=P16402	Histone H1.3	DNA binding	nucleobase-containing compound metabolic process, cellular process, chromatin organization	-	histone	Homo sapiens
39	O43790	HUMAN|Ensembl=ENSG00000258832|UniProtKB=O43790	Keratin, type II cuticular Hb6	structural constituent of cytoskeleton	cellular process, cellular component morphogenesis, cellular component organization	intermediate filament cytoskeleton, intracellular	structural protein, intermediate filament	Homo sapiens
40	P13647	HUMAN|Ensembl=ENSG00000186081|UniProtKB=P13647	Keratin, type II cytoskeletal 5	structural constituent of cytoskeleton	cellular process, cellular component morphogenesis, cellular component organization	intermediate filament cytoskeleton, intracellular	structural protein, intermediate filament	Homo sapiens
41	P02533	HUMAN|Ensembl=ENSG00000186847|UniProtKB=P02533	Keratin, type I cytoskeletal 14	structural constituent of cytoskeleton	cellular process, cellular component morphogenesis, cellular component organization, cellular process	intermediate filament cytoskeleton, intracellular	structural protein, intermediate filament	Homo sapiens
42	O95678	HUMAN|Ensembl=ENSG00000170454|UniProtKB=O95678	Keratin, type II cytoskeletal 75	structural constituent of cytoskeleton	cellular process, cellular component morphogenesis, cellular component organization, cellular process	intermediate filament cytoskeleton, intracellular	structural protein, intermediate filament	Homo sapiens
43	Q7Z3Y7	HUMAN|Ensembl=ENSG00000173908|UniProtKB=Q7Z3Y7	Keratin, type I cytoskeletal 28	structural constituent of cytoskeleton	cellular process, cellular component morphogenesis, cellular component organization, cellular process	intermediate filament cytoskeleton, intracellular	structural protein, intermediate filament	Homo sapiens
44	Q7Z3Y8	HUMAN|Ensembl=ENSG00000171446|UniProtKB=Q7Z3Y8	Keratin, type I cytoskeletal 27	structural constituent of cytoskeleton	cellular process, cellular component morphogenesis, cellular component organization, cellular process	intermediate filament cytoskeleton, intracellular	structural protein, intermediate filament	Homo sapiens
45	P14923	HUMAN|Ensembl=ENSG00000261577|UniProtKB=P14923	Junction plakoglobin	-	-	-	-	Homo sapiens
46	Q93077	HUMAN|Ensembl=ENSG00000180573|UniProtKB=Q93077	Histone H2A type 1-C	DNA binding	cellular process, cellular component morphogenesis, cellular component organization, cellular process	-	histone	Homo sapiens
47	Q6A163	HUMAN|Ensembl=ENSG00000262164|UniProtKB=Q6A163	Keratin, type I cytoskeletal 39	structural constituent of cytoskeleton	cellular process	intermediate filament cytoskeleton	structural protein	Homo sapiens
			KRT39		cellular component morphogenesis	intracellular	intermediate filament	
			ortholog		cellular component organization			
48	Q86UQ4	HUMAN|Ensembl=ENSG00000179869|UniProtKB=Q86UQ4	ATP-binding cassette sub-family A member 13	ATPase activity, coupled to transmembrane movement of substances, transmembrane transporter activity	phospholipid metabolic process, phospholipid metabolic process, cholesterol metabolic process, lipid transport	-	ATP-binding cassette (ABC) transporter	Homo sapiens
49	Q02413	HUMAN|Ensembl=ENSG00000134760|UniProtKB=Q02413	Desmoglein-1	calcium ion binding	cellular process	plasma membrane, cell part	cell junction protein, cadherin	Homo sapiens
50	P27348	HUMAN|Ensembl=ENSG00000134308|UniProtKB=P27348	14-3-3 protein theta	-	cell cycle, cell communication	-	chaperone	Homo sapiens
51	P63104	HUMAN|Ensembl=ENSG00000164924|UniProtKB=P63104	14-3-3 protein zeta/delta	-	cell cycle, cell communication	-	chaperone	Homo sapiens
52	Q09666	HUMAN|Ensembl=ENSG00000124942|UniProtKB=Q09666	Neuroblast differentiation-associated protein AHNAK	-	-	-	-	Homo sapiens
53	P54652	HUMAN|Ensembl=ENSG00000126803|UniProtKB=P54652	Heat shock-related 70 kDa protein 2	-	protein folding, protein complex assembly, response to stress, protein complex biogenesis	-	Hsp70 family chaperone	Homo sapiens
54	O95147	HUMAN|Ensembl=ENSG00000161326|UniProtKB=O95147	Dual specificity protein phosphatase 14	phosphoprotein phosphatase activity	phosphate-containing compound metabolic process, cellular protein modification process, cellular process	-	protein phosphatase, kinase inhibitor	Homo sapiens
55	Q5T749	HUMAN|Ensembl=ENSG00000203786|UniProtKB=Q5T749	Keratinocyte proline-rich protein	ligase activity, structural constituent of cytoskeleton	protein metabolic process	microtubule, cytoskeleton, intracellular	ligase, microtubule family cytoskeletal protein	Homo sapiens
56	P62269	HUMAN|Ensembl=ENSG00000223367|UniProtKB=P62269	40S ribosomal protein S18	-	-	-	-	Homo sapiens
57	P62277	HUMAN|Ensembl=ENSG00000110700|UniProtKB=P62277	40S ribosomal protein S13	structural constituent of ribosome, nucleic acid binding	protein metabolic process	-	ribosomal protein	Homo sapiens
58	Q96QA5	HUMAN|Ensembl=ENSG00000167914|UniProtKB=Q96QA5	Gasdermin-A	-	-	-	-	Homo sapiens
59	P04080	HUMAN|Ensembl=ENSG00000160213|UniProtKB=P04080	Cystatin-B	cysteine-type peptidase activity, protein binding, cysteine-type endopeptidase inhibitor activity	proteolysis, regulation of catalytic activity	-	cysteine protease inhibitor	Homo sapiens
60	P25787	HUMAN|Ensembl=ENSG00000106588|UniProtKB=P25787	Proteasome subunit alpha type-2	peptidase activity	proteolysis	-	protease	Homo sapiens
61	P46783	HUMAN|Ensembl=ENSG00000124614|UniProtKB=P46783	40S ribosomal protein S10	structural constituent of ribosome, nucleic acid binding	translation	-	ribosomal protein	Homo sapiens
62	P62266	HUMAN|Ensembl=ENSG00000186468|UniProtKB=P62266	40S ribosomal protein S23	structural constituent of ribosome, nucleic acid binding	translation	-	ribosomal protein	Homo sapiens
63	P60900	HUMAN|Ensembl=ENSG00000100902|UniProtKB=P60900	Proteasome subunit alpha type-6	peptidase activity	proteolysis	-	protease	Homo sapiens
64	P62304	HUMAN|Ensembl=ENSG00000182004|UniProtKB=P62304	Small nuclear ribonucleoprotein E	catalytic activity, mRNA binding, protein binding	RNA splicing, via transesterification reactions, mRNA splicing, via spliceosome, RNA splicing, via transesterification reactions	-	mRNA splicing factor	Homo sapiens
65	P04632	HUMAN|Ensembl=ENSG00000126247|UniProtKB=P04632	Calpain small subunit 1	cysteine-type peptidase activity, calcium ion binding, calmodulin binding, calcium-dependent phospholipid binding	immune response, induction of apoptosis, proteolysis, cell communication, muscle contraction, induction of apoptosis, response to stimulus	-	transmembrane receptor regulatory/adaptor protein, cysteine protease, cysteine protease, annexin, calmodulin	Homo sapiens
66	P78386	HUMAN|Ensembl=ENSG00000135443|UniProtKB=P78386	Keratin, type II cuticular Hb5	structural constituent of cytoskeleton	cellular process, cellular component morphogenesis, cellular component organization, cellular process	intermediate filament cytoskeleton, intracellular, intermediate filament cytoskeleton	structural protein, intermediate filament,	Homo sapiens
67	Q7Z3Z0	HUMAN|Ensembl=ENSG00000204897|UniProtKB=Q7Z3Z0	Keratin, type I cytoskeletal 25	structural constituent of cytoskeleton	cellular process, cellular component morphogenesis, cellular component organization	intracellular	structural protein, intermediate filament	Homo sapiens
68	P14923	HUMAN|Ensembl=ENSG00000173801|UniProtKB=P14923	Junction plakoglobin	transcription cofactor activity, protein binding	female gamete generation, nitrogen compound metabolic process, biosynthetic process, transcription from RNA polymerase II promoter, cell-cell signaling, cell adhesion, pattern specification process, pattern specification process, cellular component morphogenesis, embryo development, cell differentiation, heart development, response to stimulus, protein localization, regulation of transcription from RNA polymerase II promoter, cytoskeleton organization	plasma membrane, cell junction, protein complex, nucleus, cytoskeleton, cytoplasm	storage protein, signaling molecule, cytoskeletal protein, cell adhesion molecule	Homo sapiens
69	P62805	HUMAN|Ensembl=ENSG00000188987|UniProtKB=P62805	Histone H4	-	-	-	-	Homo sapiens
70	P62805	HUMAN|Ensembl=ENSG00000124529|UniProtKB=P62805	Histone H4	-	-	-	-	Homo sapiens
71	Q2M2I5	HUMAN|Ensembl=ENSG00000167916|UniProtKB=Q2M2I5	Keratin, type I cytoskeletal 24	structural constituent of cytoskeleton	cellular process, cellular component morphogenesis	intermediate filament cytoskeleton, intracellular	structural protein, intermediate filament	Homo sapiens
72	Q562R1	HUMAN|Ensembl=ENSG00000169067|UniProtKB=Q562R1	Beta-actin-like protein 2	structural constituent of cytoskeleton	cytokinesis, mitosis, cellular component morphogenesis, intracellular protein transport, exocytosis, endocytosis, cellular component organization	actin cytoskeleton, intracellular	actin and actin related protein	Homo sapiens
73	Q6A163	HUMAN|Ensembl=ENSG00000196859|UniProtKB=Q6A163	Keratin, type I cytoskeletal 39	-	-	-	-	Homo sapiens
74	Q9BYR6	HUMAN|Ensembl=ENSG00000212899|UniProtKB=Q9BYR6	Keratin-associated protein 3-3	-	-	-	-	Homo sapiens
75	Q0Z7S8	HUMAN|Ensembl=ENSG00000205186|UniProtKB=Q0Z7S8	Fatty acid-binding protein 9	lipid binding	lipid metabolic process, cell communication, ectoderm development, lipid transport, vitamin transport	-	transfer/carrier protein	Homo sapiens
76	Q9Y6R9	HUMAN|Ensembl=ENSG00000104983|UniProtKB=Q9Y6R9	Coiled-coil domain-containing protein 61	-	-	-	-	Homo sapiens
77	P36952	HUMAN|Ensembl=ENSG00000206075|UniProtKB=P36952	Serpin B5	serine-type peptidase activity, peptidase inhibitor activity	proteolysis, regulation of biological process, regulation of catalytic activity	intracellular	serine protease inhibitor	Homo sapiens
78	P15090	HUMAN|Ensembl=ENSG00000170323|UniProtKB=P15090	Fatty acid-binding protein, adipocyte	lipid binding	lipid metabolic process, cell communication, ectoderm development, lipid transport, vitamin transport	-	transfer/carrier protein	Homo sapiens
79	P62851	HUMAN|Ensembl=ENSG00000262088|UniProtKB=P62851	40S ribosomal protein S25	-	-	-	-	Homo sapiens
80	P07476	HUMAN|Ensembl=ENSG00000163207|UniProtKB=P07476	Involucrin	structural constituent of cytoskeleton	cellular process, cellular component morphogenesis, cellular component organization	extracellular region, extracellular matrix, cytoskeleton, intracellular	extracellular matrix protein, cytoskeletal protein	Homo sapiens
81	P14174	HUMAN|Ensembl=ENSG00000240972|UniProtKB=P14174	Macrophage migration inhibitory factor	-	-	-	-	Homo sapiens
82	Q53RT3	HUMAN|Ensembl=ENSG00000244617|UniProtKB=Q53RT3	Retroviral-like aspartic protease 1	-	-	-	-	Homo sapiens
83	Q9Y3D6	HUMAN|Ensembl=ENSG00000214253|UniProtKB=Q9Y3D6	Mitochondrial fission 1 protein	-	-	-	-	Homo sapiens
84	P42766	HUMAN|Ensembl=ENSG00000136942|UniProtKB=P42766	60S ribosomal protein L35	transferase activity, transferring glycosyl groups	translation	-	glycosyltransferase	Homo sapiens
			RPL35	structural constituent of ribosome	protein glycosylation		ribosomal protein	
			ortholog	nucleic acid binding				
85	P62280	HUMAN|Ensembl=ENSG00000142534|UniProtKB=P62280	40S ribosomal protein S11	structural constituent of ribosome, nucleic acid binding	translation	-	ribosomal protein	Homo sapiens
86	P0CW22	HUMAN|Ensembl=ENSG00000182774|UniProtKB=P0CW22	40S ribosomal protein S17-like	-	biosynthetic process, protein metabolic process, cellular process, cellular component biogenesis	ribosome, organelle, cytosol	ribosomal protein	Homo sapiens
87	Q15102	HUMAN|Ensembl=ENSG00000079462|UniProtKB=Q15102	Platelet-activating factor acetylhydrolase IB subunit gamma	-	-	-	-	Homo sapiens
88	P78385	HUMAN|Ensembl=ENSG00000170523|UniProtKB=P78385	Keratin, type II cuticular Hb3	structural constituent of cytoskeleton	cellular process, cellular component morphogenesis, cellular component organization	intermediate filament cytoskeleton, intracellular	structural protein, intermediate filament	Homo sapiens
89	Q15323	HUMAN|Ensembl=ENSG00000094796|UniProtKB=Q15323	Keratin, type I cuticular Ha1	structural constituent of cytoskeleton	cellular process, cellular component morphogenesis, cellular component organization	intermediate filament cytoskeleton, intracellular	structural protein, intermediate filament	Homo sapiens
90	P35527	HUMAN|Ensembl=ENSG00000171403|UniProtKB=P35527	Keratin, type I cytoskeletal 9	structural constituent of cytoskeleton	cellular process, cellular component morphogenesis, cellular component organization	intermediate filament cytoskeleton, intracellular	structural protein, intermediate filament	Homo sapiens
91	Q9NSB2	HUMAN|Ensembl=ENSG00000161849|UniProtKB=Q9NSB2	Keratin, type II cuticular Hb4	structural constituent of cytoskeleton	cellular process, cellular component morphogenesis, cellular component organization	intermediate filament cytoskeleton, intracellular	structural protein, intermediate filament	Homo sapiens
92	P62805	HUMAN|Ensembl=ENSG00000197238|UniProtKB=P62805	Histone H4	-	-	-	-	Homo sapiens
93	Q9BYR8	HUMAN|Ensembl=ENSG00000212901|UniProtKB=Q9BYR8	Keratin-associated protein 3-1	-	-	-	-	Homo sapiens
94	P61981	HUMAN|Ensembl=ENSG00000170027|UniProtKB=P61981	14-3-3 protein gamma	-	cell cycle, cell communication	-	chaperone	Homo sapiens
95	P62258	HUMAN|Ensembl=ENSG00000108953|UniProtKB=P62258	14-3-3 protein epsilon	-	cell cycle	-	chaperone	Homo sapiens
96	O60437	HUMAN|Ensembl=ENSG00000118898|UniProtKB=O60437	Periplakin	structural constituent of cytoskeleton, intermediate filament binding	cellular process	intracellular	intermediate filament binding protein	Homo sapiens
97	Q5VU13	HUMAN|Ensembl=ENSG00000243284|UniProtKB=Q5VU13	V-set and immunoglobulin domain-containing protein 8	-	-	-	-	Homo sapiens
98	P29034	HUMAN|Ensembl=ENSG00000196754|UniProtKB=P29034	Protein S100-A2	calcium ion binding, growth factor activity, calmodulin binding	macrophage activation, DNA replication, cell cycle, cell communication	-	growth factor, calmodulin	Homo sapiens
99	P26641	HUMAN|Ensembl=ENSG00000254772|UniProtKB=P26641	Elongation factor 1-gamma	oxidoreductase activity, transferase activity, racemase and epimerase activity, structural constituent of cytoskeleton, translation elongation factor activity, translation elongation factor activity, receptor binding, translation elongation factor activity	immune system process, tralslation, cell communication, response to toxic substance, regulation of translation	cytoskeleton, intracellular	transferase, signaling molecule, reductase, translation elongation factor, epimerase/racemase, cytoskeletal protein	Homo sapiens
100	Q96AG4	HUMAN|Ensembl=ENSG00000108829|UniProtKB=Q96AG4	Leucine-rich repeat-containing protein 59	-	-	-	-	Homo sapiens
101	Q3LI83	HUMAN|Ensembl=ENSG00000188694|UniProtKB=Q3LI83	Keratin-associated protein 24-1	-	-	-	-	Homo sapiens
102	Q14532	HUMAN|Ensembl=ENSG00000108759|UniProtKB=Q14532	Keratin, type I cuticular Ha2	structural constituent of cytoskeleton	cellular process, cellular component morphogenesis, cellular component organization	intermediate filament cytoskeleton, intracellular	structural protein, intermediate filament	Homo sapiens
103	Q86Y46	HUMAN|Ensembl=ENSG00000186049|UniProtKB=Q86Y46	Keratin, type II cytoskeletal 73	structural constituent of cytoskeleton	cellular process, cellular component morphogenesis, cellular component organization	intermediate filament cytoskeleton, intracellular	structural protein, intermediate filament	Homo sapiens
104	P62805	HUMAN|Ensembl=ENSG00000183941|UniProtKB=P62805	Histone H4	-	-	-	-	Homo sapiens
105	P62805	HUMAN|Ensembl=ENSG00000197914|UniProtKB=P62805	Histone H4	-	-	-	-	Homo sapiens
106	P62805	HUMAN|Ensembl=ENSG00000198339|UniProtKB=P62805	Histone H4	DNA binding	nucleobase-containing compound metabolic process, cellular process, chromatin organization	-	histone	Homo sapiens
107	P62805	HUMAN|Ensembl=ENSG00000158406|UniProtKB=P62805	Histone H4	-	-	-	-	Homo sapiens
108	O43707	HUMAN|Ensembl=ENSG00000130402|UniProtKB=O43707	Alpha-actinin-4	structural constituent of cytoskeleton.actin binding	cellular component movement, cell cycle, cell communication	actin cytoskeleton	non-motor actin binding protein	Homo sapiens
			ACTN4		cellular component morphogenesis	intracellular		
			ortholog		cellular component organization			
109	A0PK11	HUMAN|Ensembl=ENSG00000249581|UniProtKB=A0PK11	Clarin-2	-	-	-	-	Homo sapiens
110	P33764	HUMAN|Ensembl=ENSG00000188015|UniProtKB=P33764	Protein S100-A3	calcium ion binding, growth factor activity, calmodulin binding	macrophage activation, DNA replication, cell cycle, cell communication	-	growth factor, calmodulin	Homo sapiens
111	P07737	HUMAN|Ensembl=ENSG00000108518|UniProtKB=P07737	Profilin-1	-	-	-	-	Homo sapiens
112	O15173	HUMAN|Ensembl=ENSG00000164040|UniProtKB=O15173	Membrane-associated progesterone receptor component 2	receptor activity, receptor binding	cell communication	-	signaling molecule, receptor	Homo sapiens
113	P13639	HUMAN|Ensembl=ENSG00000167658|UniProtKB=P13639	Elongation factor 2	GTPase activity, translation initiation factor activity, translation elongation factor activity, translation initiation factor activity, translation elongation factor activity, protein binding, translation initiation factor activity, translation elongation factor activity	translation	-	translation elongation factor	Homo sapiens
114	P31949	HUMAN|Ensembl=ENSG00000163191|UniProtKB=P31949	Protein S100-A11	calcium ion binding, receptor binding, calmodulin binding	macrophage activation, DNA replication, cell cycle, cell communication	-	signaling molecule, calmodulin	Homo sapiens
115	P06733	HUMAN|Ensembl=ENSG00000074800|UniProtKB=P06733	Alpha-enolase	lyase activity	glycolysis	-	lyase	Homo sapiens
116	Q5D862	HUMAN|Ensembl=ENSG00000143520|UniProtKB=Q5D862	Filaggrin-2	-	-	-	-	Homo sapiens
117	P62249	HUMAN|Ensembl=ENSG00000105193|UniProtKB=P62249	40S ribosomal protein S16	structural constituent of ribosome, RNA binding	translation	ribonucleoprotein complex	ribonucleoprotein, ribosomal protein,	Homo sapiens
118	P11021	HUMAN|Ensembl=ENSG00000044574|UniProtKB=P11021	78 kDa glucose-regulated protein	-	protein folding, protein complex assembly, protein complex biogenesis	-	Hsp70 family chaperone	Homo sapiens
119	P62857	HUMAN|Ensembl=ENSG00000233927|UniProtKB=P62857	40S ribosomal protein S28	structural molecule activity	cellular process, RNA localization, nucleobase-containing compound transport, nuclear transport, cellular component biogenesis	ribosome, organelle, cytosol	ribosomal protein	Homo sapiens
120	Q86SG7	HUMAN|Ensembl=ENSG00000185674|UniProtKB=Q86SG7	Lysozyme g-like protein 2	-	-	-	-	Homo sapiens
121	Q9P1F3	HUMAN|Ensembl=ENSG00000146386|UniProtKB=Q9P1F3	Costars family protein ABRACL	-	-	-	-	Homo sapiens
122	P30041	HUMAN|Ensembl=ENSG00000117592|UniProtKB=P30041	Peroxiredoxin-6	oxidoreductase activity, peroxidase activity	metabolic process	-	peroxidase	Homo sapiens
123	P16152	HUMAN|Ensembl=ENSG00000159228|UniProtKB=P16152	Carbonyl reductase (NADPH) 1	oxidoreductase activity	steroid metabolic process, visual perception, sensory perception	-	dehydrogenase, reductase	Homo sapiens
124	Q96QR8	HUMAN|Ensembl=ENSG00000146676|UniProtKB=Q96QR8	Transcriptional activator protein Pur-beta	sequence-specific DNA binding transcription factor activity, single-stranded DNA binding, sequence-specific DNA binding transcription factor activity	transcription from RNA polymerase II promoter	-	transcription factor, DNA binding protein	Homo sapiens
125	O76009	HUMAN|Ensembl=ENSG00000006059|UniProtKB=O76009	Keratin, type I cuticular Ha3-I	structural constituent of cytoskeleton	cellular process, cellular component morphogenesis, cellular component organization	intermediate filament cytoskeleton, intracellular	structural protein, intermediate filament	Homo sapiens
126	P04259	HUMAN|Ensembl=ENSG00000185479|UniProtKB=P04259	Keratin, type II cytoskeletal 6B	structural constituent of cytoskeleton	cellular process, cellular component morphogenesis, cellular component organization	intermediate filament cytoskeleton, intracellular	structural protein, intermediate filament	Homo sapiens
127	P13645	HUMAN|Ensembl=ENSG00000186395|UniProtKB=P13645	Keratin, type I cytoskeletal 10	structural constituent of cytoskeleton	cellular process, cellular component morphogenesis, cellular component organization	intermediate filament cytoskeleton, intracellular	structural protein, intermediate filament	Homo sapiens
128	P08779	HUMAN|Ensembl=ENSG00000186832|UniProtKB=P08779	Keratin, type I cytoskeletal 16	structural constituent of cytoskeleton	cellular process, cellular component morphogenesis, cellular component organization	intermediate filament cytoskeleton, intracellular	structural protein, intermediate filament	Homo sapiens
129	P62805	HUMAN|Ensembl=ENSG00000197837|UniProtKB=P62805	Histone H4	-	-	-	-	Homo sapiens
130	Q9BYR8	HUMAN|Ensembl=ENSG00000262068|UniProtKB=Q9BYR8	Keratin-associated protein 3-1	-	-	-	-	Homo sapiens
131	P47929	HUMAN|Ensembl=ENSG00000178934|UniProtKB=P47929	Galectin-7	receptor binding	cellular process	-	signaling molecule, cell adhesion molecule	Homo sapiens
132	Q9BYR7	HUMAN|Ensembl=ENSG00000263296|UniProtKB=Q9BYR7	Keratin-associated protein 3-2	-	-	-	-	Homo sapiens
133	P27482	HUMAN|Ensembl=ENSG00000178363|UniProtKB=P27482	Calmodulin-like protein 3	calcium ion binding, calmodulin binding	cellular component movement, cell cycle, cell communication	-	calmodulin	Homo sapiens
134	Q13228	HUMAN|Ensembl=ENSG00000143416|UniProtKB=Q13228	Selenium-binding protein 1	-	-	-	defense/immunity protein	Homo sapiens
135	P09211	HUMAN|Ensembl=ENSG00000084207|UniProtKB=P09211	Glutathione S-transferase P	-	-	-	-	Homo sapiens
136	P62269	HUMAN|Ensembl=ENSG00000231500|UniProtKB=P62269	40S ribosomal protein S18	-	-	-	-	Homo sapiens
137	P62269	HUMAN|Ensembl=ENSG00000096150|UniProtKB=P62269	40S ribosomal protein S18	structural molecule activity	biosynthetic process, protein metabolic process, cellular process, cellular component biogenesis	ribosome, organelle, cytoplasm	-	Homo sapiens
138	Q9Y446	HUMAN|Ensembl=ENSG00000184363|UniProtKB=Q9Y446	Plakophilin-3	structural constituent of cytoskeleton, actin binding	cell communication, cell adhesion	plasma membrane, cell junction, intracellular	intermediate filament binding protein, cell junction protein, cell adhesion molecule	Homo sapiens
139	Q5VU13	HUMAN|Ensembl=ENSG00000188004|UniProtKB=Q5VU13	V-set and immunoglobulin domain-containing protein 8	receptor activity	immune system process, cellular process, cell adhesion	-	receptor, cell adhesion molecule	Homo sapiens
140	P60866	HUMAN|Ensembl=ENSG00000008988|UniProtKB=P60866	40S ribosomal protein S20	structural constituent of ribosome, nucleic acid binding	translation	-	ribosomal protein	Homo sapiens
141	Q96FQ6	HUMAN|Ensembl=ENSG00000188643|UniProtKB=Q96FQ6	Protein S100-A16	calcium ion binding, receptor binding, calmodulin binding	macrophage activation, DNA replication, cell cycle, cell communication	-	signaling molecule, calmodulin	Homo sapiens
142	P49207	HUMAN|Ensembl=ENSG00000109475|UniProtKB=P49207	60S ribosomal protein L34	structural constituent of ribosome, nucleic acid binding	translation	-	ribosomal protein	Homo sapiens
143	P00918	HUMAN|Ensembl=ENSG00000104267|UniProtKB=P00918	Carbonic anhydrase 2	hydro-lyase activity	metabolic process	-	dehydratase	Homo sapiens
144	P62805	HUMAN|Ensembl=ENSG00000182217|UniProtKB=P62805	Histone H4	-	-	-	-	Homo sapiens
145	P62805	HUMAN|Ensembl=ENSG00000198327|UniProtKB=P62805	Histone H4	-	-	-	-	Homo sapiens
146	Q8N1N4	HUMAN|Ensembl=ENSG00000170423|UniProtKB=Q8N1N4	Keratin, type II cytoskeletal 78	structural constituent of cytoskeleton	cellular process, cellular component morphogenesis, cellular component organization	intermediate filament cytoskeleton, intracellular	structural protein, intermediate filament	Homo sapiens
147	P47929	HUMAN|Ensembl=ENSG00000205076|UniProtKB=P47929	Galectin-7	-	-	-	-	Homo sapiens
148	Q9BYR6	HUMAN|Ensembl=ENSG00000263101|UniProtKB=Q9BYR6	Keratin-associated protein 3-3	-	-	-	-	Homo sapiens
149	Q01469	HUMAN|Ensembl=ENSG00000164687|UniProtKB=Q01469	Fatty acid-binding protein, epidermal	lipid binding	lipid metabolic process, cell communication, ectoderm development, lipid transport, vitamin transport	-	transfer/carrier protein	Homo sapiens
150	Q9BYR7	HUMAN|Ensembl=ENSG00000212900|UniProtKB=Q9BYR7	Keratin-associated protein 3-2	-	-	-	-	Homo sapiens
151	P09382	HUMAN|Ensembl=ENSG00000100097|UniProtKB=P09382	Galectin-1	receptor binding	cellular process	-	signaling molecule, cell adhesion molecule	Homo sapiens
152	Q86SJ6	HUMAN|Ensembl=ENSG00000175065|UniProtKB=Q86SJ6	Desmoglein-4	calcium ion binding	cellular process	plasma membrane, cell part	cell junction protein, cadherin	Homo sapiens
153	P37802	HUMAN|Ensembl=ENSG00000158710|UniProtKB=P37802	Transgelin-2	structural constituent of cytoskeleton, actin binding	muscle contraction	actin cytoskeleton, intracellular	non-motor actin binding protein	Homo sapiens
154	Q9H9L3	HUMAN|Ensembl=ENSG00000143319|UniProtKB=Q9H9L3	Interferon-stimulated 20 kDa exonuclease-like 2	exoribonuclease activity, nucleic acid binding	RNA catabolic process	-	exoribonuclease, nuclease	Homo sapiens
155	P62269	HUMAN|Ensembl=ENSG00000226225|UniProtKB=P62269	40S ribosomal protein S18	-	-	-	-	Homo sapiens
156	P62269	HUMAN|Ensembl=ENSG00000235650|UniProtKB=P62269	40S ribosomal protein S18	-	-	-	-	Homo sapiens
157	Q15365	HUMAN|Ensembl=ENSG00000169564|UniProtKB=Q15365	Poly(rC)-binding protein 1	catalytic activity, mRNA binding, protein binding	induction of apoptosis, RNA splicing, via transesterification reactions, transcription from RNA polymerase II promoter, mRNA splicing, via spliceosome, RNA splicing, via transesterification reactions, protein metabolic process, cell cycle, cell communication, neurological system process, induction of apoptosis, cell communication, neurological system process, intracellular protein transport, nuclear transport	ribonucleoprotein complex	mRNA splicing factor, ribonucleoprotein, enzyme modulator	Homo sapiens
158	P32926	HUMAN|Ensembl=ENSG00000134757|UniProtKB=P32926	Desmoglein-3	calcium ion binding	cellular process	plasma membrane, cell part	cell junction protein, cadherin	Homo sapiens
159	P18621	HUMAN|Ensembl=ENSG00000265681|UniProtKB=P18621	60S ribosomal protein L17	-	protein metabolic process	-	-	Homo sapiens

 Appendix I

**Table 11:** Gene network analysis of proteins common in all samples, ranked based on number of connections on nodes.

Gene name in node	Number of connections
KRT5	88
KRT14	78
DSP	71
KRT6B	70
DSG1	67
KRT17	67
KRT10	64
PPL	64
S100A3	64
KRT16	63
RPS18	63
GSTP1	62
JUP	62
S100A16	62
ANXA1	61
RPS13	61
IVL	60
S100A14	60
CSTB	59
HSPB1	59
KRT24	59
KRT33A	59
KRT2	58
S100A11	58
SFN	57
KRT31	56
KRT75	56
KRT83	56
KRT85	55
KRT9	55
YWHAZ	55
SERPINB5	54
FABP5	53
ACTN4	52
DUSP14	51
RPS20	50
CST6	49
KRT84	49
PKP3	49
KRT32	48
KRT33B	48
PFN1	47
TCHH	46
PPIB	45
KRT35	44
KRT27	43
KRT78	43
TAGLN2	43
ALDOA	42
CALML3	42
EEF2	42
MYH9	42
RPS25	42
AHNAK	41
KRTAP3-1	41
S100A8	41
YWHAE	41
KRT71	40
LGALS1	40
KRT25	38
PCBP1	38
YWHAG	38
HSPA5	37
WDR87	37
FABP4	36
FLG2	36
HSPA2	35
KRTAP3-3	35
RPS16	35
KRT73	34
KRTAP3-2	34
DSG4	33
KRTAP2-2	33
LYG2	33
PAFAH1B2	33
ENO1	32
KRT74	32
UBA1	31
VCP	31
PGRMC2	30
DCD	29
HIST1H2AC	29
YWHAQ	28
RPS28	26
KBTBD11	25
SELENBP1	24
ABCA13	23
ANKRD22	23
MYH14	21
ISG20L2	16
CCDC61	8
ACTBL2	4
GSDMA	4
KRT28	4
